# Global dynamics of stage-specific transcription factor binding during thymocyte development

**DOI:** 10.1038/s41598-018-23774-9

**Published:** 2018-04-04

**Authors:** Tomonori Hosoya, Ricardo D’Oliveira Albanus, John Hensley, Greggory Myers, Yasuhiro Kyono, Jacob Kitzman, Stephen C. J. Parker, James Douglas Engel

**Affiliations:** 1 Department of Cell and Developmental Biology, Ann Arbor, USA; 2 Department of Computational Medicine and Bioinformatics, Ann Arbor, USA; 3 Department of Human Genetics, University of Michigan, 3035 BSRB, 109 Zina Pitcher Place, Ann Arbor, Michigan 48109-2200 USA

## Abstract

In vertebrates, multiple transcription factors (TFs) bind to gene regulatory elements (promoters, enhancers, and silencers) to execute developmental expression changes. ChIP experiments are often used to identify where TFs bind to regulatory elements in the genome, but the requirement of TF-specific antibodies hampers analyses of tens of TFs at multiple loci. Here we tested whether TF binding predictions using ATAC-seq can be used to infer the identity of TFs that bind to functionally validated enhancers of the *Cd4*, *Cd8*, and *Gata3* genes in thymocytes. We performed ATAC-seq at four distinct stages of development in mouse thymus, probing the chromatin accessibility landscape in double negative (DN), double positive (DP), CD4 single positive (SP4) and CD8 SP (SP8) thymocytes. Integration of chromatin accessibility with TF motifs genome-wide allowed us to infer stage-specific occupied TF binding sites within known and potentially novel regulatory elements. Our results provide genome-wide stage-specific T cell open chromatin profiles, and allow the identification of candidate TFs that drive thymocyte differentiation at each developmental stage.

## Introduction

T cells develop in the thymus, where biologically distinct events driven by the interplay of multiple transcription factors (TFs) acting in coordination take place at each thymocyte stage. After migration of thymic seeding progenitors from the bone marrow and their occupation of supportive niches in the thymic medulla, early thymic progenitors (ETP) develop through immature double negative (DN; CD4^−^CD8^−^) cells to the double positive (DP; CD4^+^CD8^+^) stage, and then mature into either CD4 single-positive (SP4) helper or CD8 SP (SP8) killer T cells. While ETP retain multi-lineage differentiation capacity, they gradually lose the potential to become non-T lineage cells and become increasingly restricted to a T lineage fate^1–4^. During the DN stages, committed, developing T cells undergo immune system-specific DNA recombination, and must successfully recombine a *Trb* gene allele (encoding the T cell β receptor, TCRβ) to then pass the β selection checkpoint (when the formation of a pre-TCR complex is assessed)^5,6^ in order to survive. At the next DP stage (where both CD4 and CD8 are expressed on the cell surface), the TCRα receptor rearranges, and only cells expressing a functional cell surface TCR complex (TCRα plus TCRβ) that is able to bind with appropriate affinity to the major histocompatibility complex (MHC) survive positive selection^7^. DP cells recognizing MHC class I can then mature into SP8 T cells, while DP cells recognizing MHC class II mature into SP4 T cells. Finally, negative selection eliminates by apoptosis cells that bind to self-peptides presented by the MHC, and only cells that do not exhibit high affinity to self-peptides survive^7^.

Although T cell developmental stage-specific gene expression profiling has been previously described^8,9^, the mechanisms that regulate those spatial and temporal expression patterns are far less well understood for all but a handful of genes. DNA-binding TFs play a central role governing gene expression in each cell, often eliciting transcriptional responses through specialized regulatory elements, including promoters, enhancers, and silencers. A widely accepted model for gene expression is that multiple transcription factors bind to an enhancer, assemble an enhanceosome, and then recruit co-activators and chromatin-remodeling proteins to the promoter^10,11^. Given the limitations of ChIP-seq to detect a single TF per assay, an alternative approach for detecting TF binding is using open chromatin assays, such ATAC-seq^12,13^. The genome is highly compact except within transcribed genes and regulatory elements, where chromatin is open and sensitive to cleavage by DNaseI^14–16^ or transposition by Tn5 transposase^17^. The binding of TFs to DNA affects DNase/transposase cleavage in the vicinity of the bound site, allowing for TF occupancy to be predicted from the chromatin accessibility data^12,13,18^. Thus DNase/ATAC footprinting can be used to identify TF binding motif sequences within regulatory elements.

To generate genome-wide profiles of stage-specific chromatin accessibility and TF binding during thymocyte development, we performed ATAC-seq at four different stages of adult thymocyte development: DN, DP, SP4 and SP8 stages. The open chromatin regions identified by ATAC-seq highlighted both known, biologically validated regulatory elements, as well as many novel potential regulatory elements. Furthermore, footprinting analysis^12,13^ of those open chromatin regions revealed the high-resolution landscape of predicted TF-bound motifs within those sequences. Our ATAC-seq data enabled the discovery of both stage-independent and stage-specific domains of open chromatin, and the TF footprinting data revealed 10–20 novel protein bound sequences within the previously validated enhancers of the *Cd4*, *Cd8, Trb* and *Gata3* genes. Furthermore, enrichment analyses of TF binding in stage-specific open chromatin allowed the identification of TF motifs potentially driving each stage of thymocyte development. These data demonstrate that stage-specific changes in open chromatin are highly dynamic as thymocytes develop and provide deep insight into how the stage-specific binding of multiple TFs orchestrate transcriptional regulatory networks.

## Results

### T cell developmental stage-specific genome-wide mapping of accessible chromatin

To gain insight into developing T cell stage-specific chromatin opening, DN, DP, SP4 and SP8 cells were isolated from adult thymi by flow cytometry (Supplementary Fig. S1). 50,000–100,000 cells were processed for ATAC-library preparation as described^17^. The ATAC-seq reads (Supplementary Table S1) were then mapped to mouse reference genome mm10 using BWA^19^ and peaks were called using MACS2^20^. ATAC-seq signals depicted in the IGB browser^21^ were reproducible in thymocytes recovered from 4 individual animals (Supplementary Fig. S2), and all peaks were highly correlated across biological and technical replicates (median Spearman correlations: DN = 0.89, DP = 0.87, SP4 = 0.88, SP8 = 0.90; Supplementary Fig. S3). ATAC-seq signals at the DP stage (which comprises approximately 85% of total thymocytes, Supplementary Fig. S1) reflected profiles that were similar to DNase-seq peaks of total adult thymocytes^22^ (Supplementary Fig. S2), as anticipated. On a global scale, DP ATAC-seq peak signals were highly correlated with DNase-seq peak signals of total thymocytes (median Spearman correlation = 0.70 to 0.79 Supplementary Fig. S3). Based on these results, we concluded that ATAC-seq provides a biologically reliable strategy to attain deeper insights into T cell stage-specific chromatin accessibility and transcription factor binding.

We identified 150,139 (DN), 107,110 (DP), 115,074 (SP4) and 104,411 (SP8) genomic open chromatin peaks at 5% FDR (Supplementary Fig. S4). These open chromatin domains correspond to 1.63% (DN), 1.22% (DP), 1.32% (DP) and 1.26% (SP4) of the mouse genome. 73,177 peaks were present at all four stages of thymocyte development, while the others were stage-specific. 20% (DN), 27% (DP), 24% (SP4) and 26% (SP8) of the ATAC peaks overlapped with promoter regions (defined as 200 bp upstream of a gene transcriptional start site). 10% (DN), 9% (DP), 8% (SP4) and 9% (SP8) of the ATAC peaks overlapped with an exon, but not with a promoter. 73% (DN), 63% (DP), 68% (SP4) and 65% (SP8) of the ATAC peaks overlapped with neither an exon nor a promoter (Supplementary Fig. S4).

**Figure 1:**
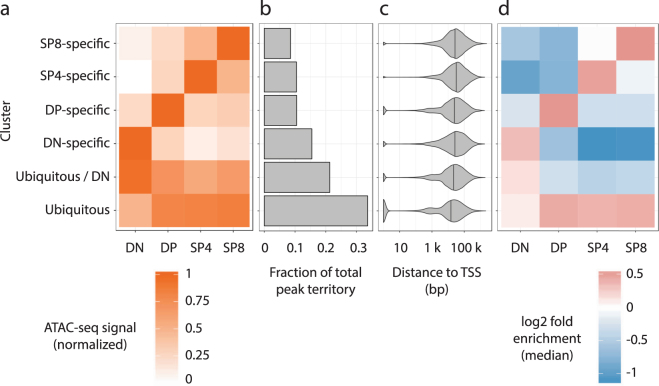
Analysis of stage-specific and ubiquitous ATAC-seq clusters. (**a**) *k*-means clustering results for the ATAC-seq signal in the four samples. Colors indicate the mean ATAC-seq signal for each sample in the respective cluster (*i.e*. the cluster centers). (**b**) Fraction of total peaks territory covered by each of the clusters. (**c**) TSS distance distribution (in log_10_ scale) for each of the clusters. Vertical bars in the violin plots correspond to the median of the dataset. (**d**) Median GAT footprint enrichment of all motifs for each dataset in the *k*-means clusters. GAT footprint enrichment heatmaps for each motif are shown in Supplementary Fig. S7.

We next sought to quantify the full spectrum (from specific to ubiquitous) of patterns of chromatin accessibility across the analyzed thymocyte developmental stages in an unbiased manner. We performed *k*-means clustering using the ATAC-seq signal. This analysis yielded 6 clusters of accessible regions: four that were specific for each stage (DN, DP, SP4, SP8), one that was ubiquitous, and one that was a combination of DN and ubiquitous (Fig. 1a, Supplementary Fig. S5). The ubiquitous cluster covered more genomic territory than any of the stage-specific clusters, while the DN-specific cluster covered more territory than the other stage-specific clusters (Fig. 1b), which is consistent with the previous conclusion that in general differentiated cells maintain a more compact chromatin architecture than their immature counterparts^23^.

We next measured the distance of each peak in the four clusters to the nearest TSS and found that the ubiquitous cluster was significantly closer to TSS than the other clusters (*p* < 10^−3^ ^24^, pairwise Kolmogorov-Smirnov tests with Bonferroni correction), suggestive of it being more associated with promoters and housekeeping genes than cell-identity features (Fig. 1c). Supporting this hypothesis, we found that SP4- and SP8-specific clusters were the most enriched for T cell related GO terms using ChIP-Enrich^24^ (Supplementary Fig. S6). The DP-specific cluster also had high enrichment for terms related to T cell differentiation, but to a lesser extent. Conversely, the DN-specific and ubiquitous clusters were strongly enriched for non-specific developmental terms, suggesting that these might regulate more general functions. These results form a comprehensive map of developmental dynamics in the open chromatin landscape across thymocyte maturation.

### TF binding identification by ATAC-seq footprinting

In order to achieve greater insights into genomic DNA sequences that are bound by TFs, we performed TF footprinting predictions using CENTIPEDE^25^. To validate the performance of CENTIPEDE footprint calls in our thymocyte data, we first compared our results with GATA3 ChIP-seq data in DN and DP thymocytes (GSE20898)^26^ and CTCF ChIP-seq in total thymocytes (ENCODE, ENCSR000CDZ)^22^. We used the Genomic Annotation Tester (GAT) tool^27^ to statistically evaluate the overlap between footprint calls and ChIP-seq bound motifs, while controlling for genome and feature sizes, as well as mapability issues (see Methods for details). Tests on both datasets showed significant overlap between ChIP-seq and footprint data (*p* < 10^−3^), indicating that the footprint predictions recapitulate actual protein binding events detected by ChIP-seq for the corresponding TF (Supplementary Table S2). These data demonstrate the effectiveness of the footprint calls from these deeply sequenced ATAC-seq data in order to generate a high confidence catalogue of putative TF-bound sequences in a thymocyte stage-specific manner.

We next focused on TF binding motifs for T cell activators and repressors that were predicted by Jojic *et al*.^28^ from stage-specific gene expression profiling. We predicted the binding for the Jojic factors and their families. As expected, we found that the footprints called in each sample were enriched both in its own specific cluster and the ubiquitous cluster, but depleted in the other sample-specific clusters using GAT, indicating that CENTIPEDE did not detect bound TF binding sites in regions that were not active in the sample being analyzed (Fig. 1d and Supplementary Fig. S7).

**Figure 2:**
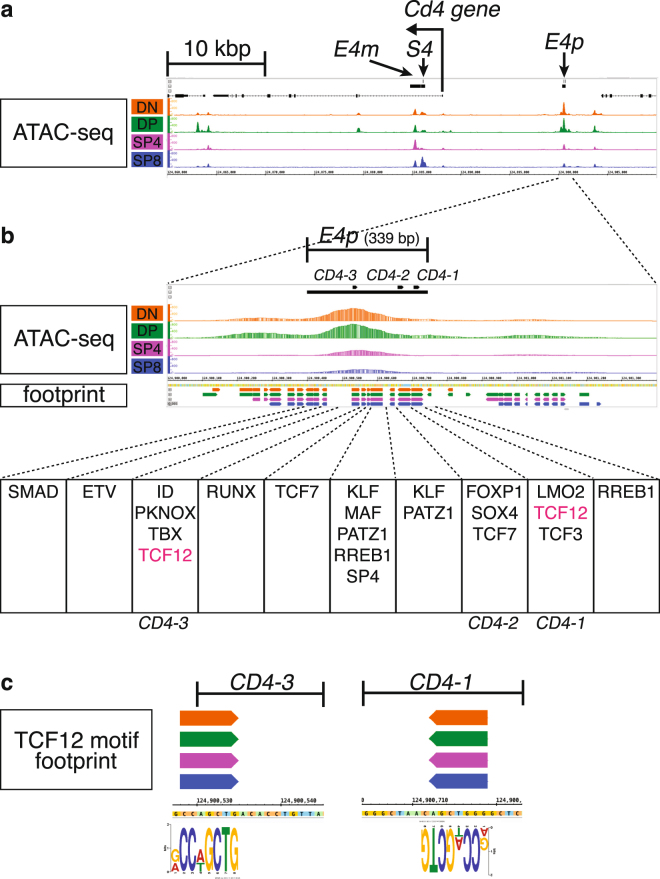
ATAC-seq signal and CENTIPEDE footprint calls around the functionally validated *E4p Cd4* gene enhancer. (**a**) ATAC-seq signals are shown on the IGB browser within around 50 kbp of the *Cd4* locus; mm10, chr6:124,860,001–124,910,000. The positions of the *E4p*, *E4m* enhancers and *S4* silencer^40–42^ are shown at the top. (**b**) ATAC signal and footprint calls around *E4p* are depicted. *CD4-1, CD4-2* and *CD4-3* sequences were first identified by DNaseI footprinting in the SL3B T cell line^40^. (**c**) TCF12 (aka HEB) motif footprints in the *CD4-1* and *CD4-3* sequences. Footprint calls within *S4* and *E4m* are shown in Supplementary Fig. S8.

**Figure 3:**
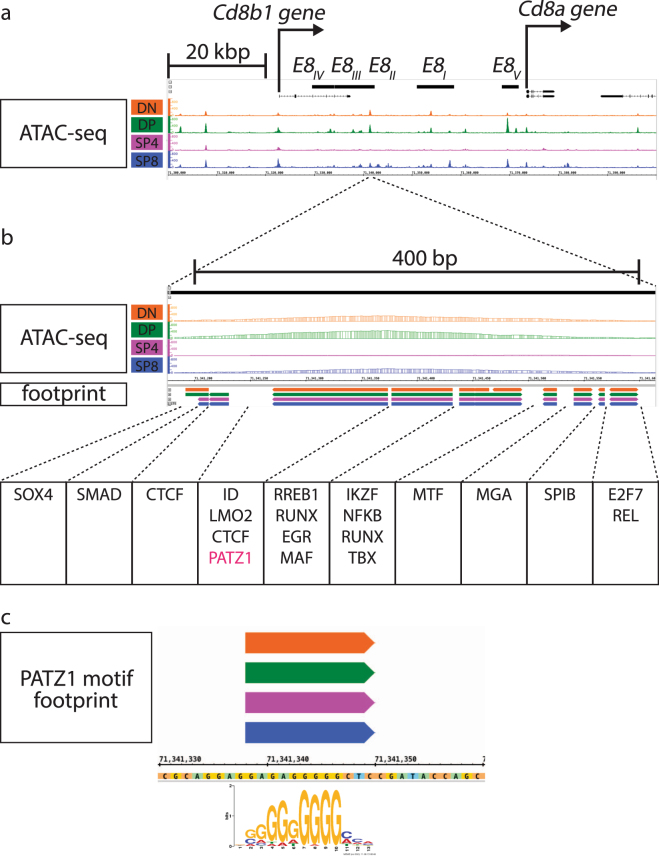
ATAC-seq signal and footprint calls within functionally validated enhancers for the *Cd8* gene. (**a**) ATAC-seq signals are shown on the IGB browser within around 100 kbp of the *Cd8* locus; mm10, chr6:71,300,001-71,400,000. The positions of the *E8*_*I*_ - *E8*_*V*_ enhancers^43–45^ are depicted at the top (**b**) ATAC signals and footprint calls at an ATAC peak identified in *E8*_*II*_ are shown. (**c**) A PATZ1 motif footprint within *E8*_*II*_ is shown. Footprint calls within *E8*_*I*_ and *E8*_*V*_ are shown in Supplementary Fig. S9.

**Figure 4:**
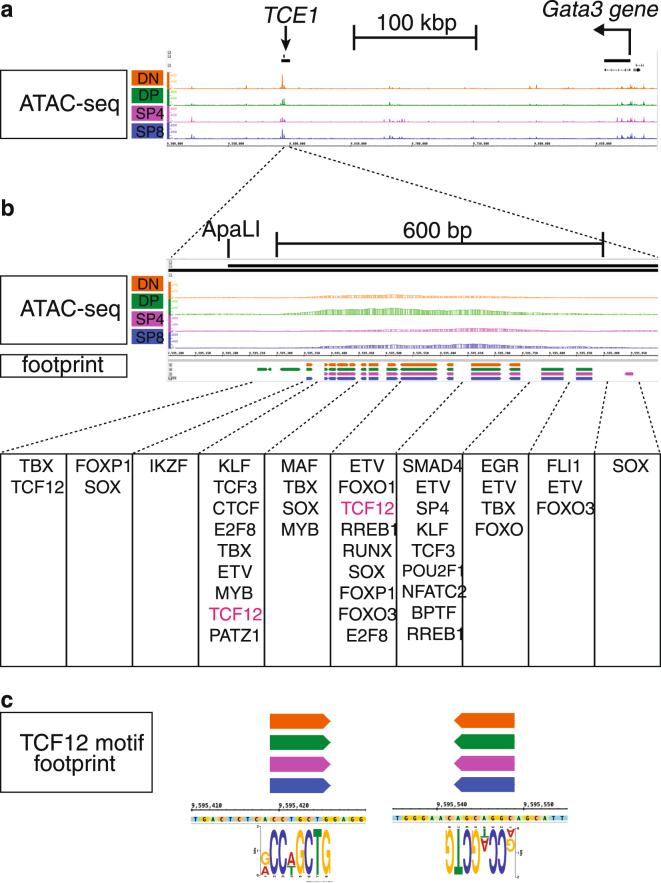
ATAC-seq signal and footprint calls within the functionally validated *TCE1 Gata3* enhancer. (**a**) ATAC-seq signals are shown on the IGB browser around 400 kbp of the *Gata3* gene; mm10, chr2: 9,500,001–9,900,000. (**b**) ATAC peak and TF footprint calls at an ATAC peak found in *TCE1* core^33,34^. (**c**) TCF12 (aka HEB) motif footprints.

We next focused on footprint calls within functionally validated enhancers. The classic definition of enhancer requires that it must be functionally validated by tests for both sufficiency and necessity in regulating its specific target gene expression, but to date only few T cell enhancers have been tested for both *in vivo*. The ATAC-seq data identified open chromatin regions within functionally validated regulatory elements for the *Cd4* (Fig. 2 and Supplementary Fig. S8), *Cd8* (Fig. 3 and Supplementary Fig. S9), *Trb* (Supplementary Fig. S10) and *Gata3* (Fig. 4) genes. The fact that the ATAC footprints recapitulated TF binding to the previously characterized motifs within these regulatory elements underscore the robust nature of the footprint approach employed in this study. Furthermore, our footprint data unveiled 8–20 novel sequences that were predicted to be bound within each of these regulatory elements (Figs 2–4 and Supplementary Figs S8–10). Based on these data, we propose that TFs bind to these sequences to assemble an active structural element that initiates and/or maintains the activity of each of these regulatory modules.

### Changes in global TF binding during thymocyte development

**Figure 5:**
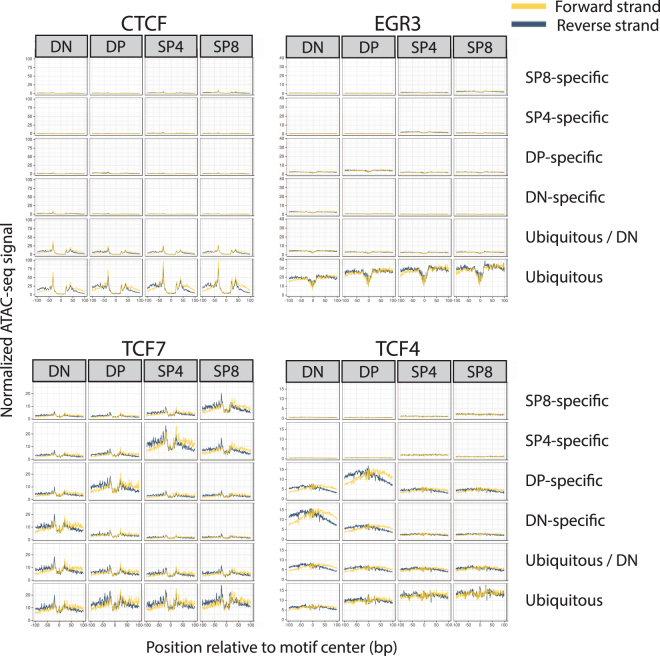
Footprint occupancies across samples and clusters. Normalized occupancy signals (see Methods) at ±100 bp of motif center for CTCF, EGR3, TCF7 and TCF4. Horizontal facets correspond to the ATAC-seq samples, and vertical facets correspond to the *k*-means clusters.

We next sought to identify higher-resolution differences in predicted TF binding across samples by measuring the pattern of chromatin accessibility anchored on footprint motifs. We found striking differences for the footprint motifs across the samples and clusters in which they were active (Fig. 5 and Supplementary Fig. S11). CTCF had strong detectable binding patterns only in the ubiquitous cluster, and a similar pattern was observed for EGR3. TCF7 (aka TCF-1) had significant binding in all clusters. TCF4, on the other hand, was detected more strongly in the DP and DN clusters, was mostly absent in the common clusters, and almost undetectable in SP4 and SP8 thymocytes, even though it was one of the most significantly enriched motifs in these two stages (*p* = 0.001). RUNX patterns were visible in the common and DN clusters, but not in the more differentiated stages. Although GATA footprints were enriched in all clusters, we could not detect strong binding patterns, which is suggestive that it may have weaker interactions with DNA^29^. Interestingly, we did not find any SP4- or SP8-specific occupancy patterns, even though some motifs, such as TCF3 and ID4, had higher enrichment values in the SP4- and SP8-specific clusters than in the ubiquitous cluster (see next and Supplementary Table. S3 for the enrichment values). These different patterns between stages for TCF3 and ID4 suggest that the availability (expression or protein levels) of these TFs changes or that different TFs recognize these motifs at each stage.

**Figure 6:**
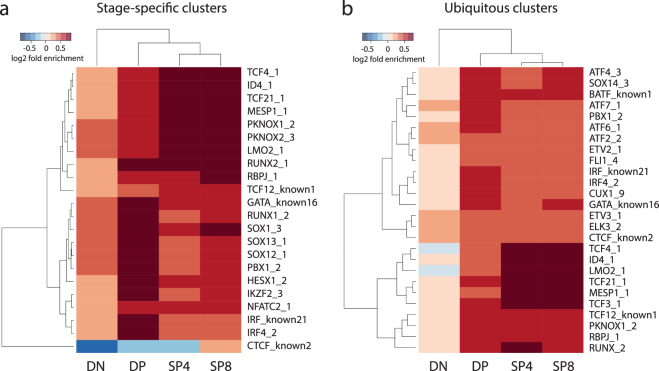
Individual footprint enrichments in each of the samples. (**a**) GAT enrichment scores for the top factors that maximize the variance between the stage-specific clusters of each of the ATAC-seq samples. (**b**) Similar to (**a**), but comparing the enrichments for each ATAC-seq sample in the ubiquitous cluster. Darker red colors correspond to stronger enrichments, and blue colors correspond to depletions.

We finally asked which footprints were enriched in each of the stage-specific open chromatin clusters defined in Fig. 1a. Each cluster showed enrichment of different TF motif footprints (Fig. 6 and Supplementary Table S3). Of note, we independently performed motif enrichment in the ATAC-seq clusters using HOMER (Supplementary Fig. S12), but this approach did not capture the nuanced enrichments we detected with the footprinting approach, as HOMER only takes the motif occurrences and not the ATAC-seq signal into account. These data support the concept that many TFs bind to specific transcriptional regulatory elements at each developmental stage to achieve stage-specific gene expression patterns, and that the binding of these individual factors is reflected in the dynamic changes in transcriptional networks that must accompany thymocyte developmental progression from one stage to the next.

These footprinting data identified potential stage-specific regulators. Out of the 34 SOX family TF motifs tested, 10 and 9, respectively, were within the top 20 enrichment scores for DN and DP, but not in in SP4 and SP8 (Supplementary Table S3), suggesting that a SOX family TF(s) is important for DN- and DP-specific gene expression. Of 3 PBX family TF motifs tested, 2 were within the top 20 fold-enrichment scores for the DP-specific cluster (Supplementary Table S3). These data suggest a role for PBX family TFs in DP-specific gene expression. Out of 4 PKNOX family TF motifs tested, 3 were within the top 20 enrichment scores for the SP4 and SP8 clusters (Supplementary Table S3), indicating that PKNOX family TFs contribute to SP4- and SP8-specific gene transcription. Finally, out of 7 MAF family TF motifs tested, 1 and 3 were within the top 20 fold-enrichment scores for the SP4 and SP8 clusters, respectively (Supplementary Table S3), supporting the hypothesis that MAF family TFs are important for SP8-specific gene expression.

## Discussion

We performed ATAC-seq experiments and footprint analyses at four major stages of thymocyte development in order to compile a catalogue of stage-specific accessible chromatin sequences as well as to identify specific sequences bound by TFs. We identified ubiquitous and stage-specific open chromatin regions, recapitulating the identity of functionally validated regulatory elements, as well as revealing novel regulatory loci. The ATAC-seq footprinting data for predicted αβT cell activators and repressors highlighted TF-bound motifs within those regulatory regions, as well as bound motifs that were enriched in each of the thymocyte stage-specific accessible chromatin clusters, providing an in-depth view into the inner regulatory workings of thymocyte development. We identified between 8 and 20 novel sequences that were predicted to be bound by proteins within previously identified regulatory elements for the *Cd4*, *Cd8*, *Trb* and *Gata3* genes, which supports the idea that an approximately 8–20 TFs bind to an enhancer in order to form a TF complex/enhanceosome that is capable of supporting the initiation and/or activation of enhancer activity. Thus one future goal is to investigate the ability of individually bound sequences to contribute to enhancer activity, which can be tested by *in vivo* ablation or mutation of specific TF motifs. The genome-wide footprinting approach detailed here is an alternative to ChIP experiments, but the two are complementary. It is well known and has been documented that several different proteins can bind to a given sequence motif (*e.g*. all six vertebrate GATA factors bind with reasonably high affinity to the AGATAA sequence motif, so identification of a given *cis* element in the absence of data regarding the tissue specificity of a given family of factors may only be marginally informative). ChIP experiments, in contrast, can capture indirect binding by virtue of protein-protein interactions that occur in larger complexes formed with a specific DNA binding protein^30,31^, potentially complicating assignment of which factor is genuinely bound to DNA at any given site.

The thymocyte stage-specific open chromatin regions identified here by ATAC-seq followed by *k*-means clustering approach highlighted the positions for thousands of potentially novel developmental stage-specific regulatory elements. The ATAC peaks provided evidence for the previously predicted closed- or open-chromatin status in both the *Cd4* and *Cd8* loci during thymocyte development^32^. Furthermore, the identification of two major ATAC peaks within the 7.1 kbp that originally defined the *Gata3* enhancer, *TCE1*^33,34^, suggests that one or both of these two open chromatin domains (of approximately 600 bp and 500 bp) play a major role in the enhancer activity of *TCE1*. In agreement with this hypothesis, one of these ATAC-seq peaks aligns perfectly with a 1.2 kbp “core” sequence that exerts similar reporter gene activation in thymocytes of transgenic mice that is roughly equivalent to the whole 7.1 kbp *TCE1* sequence^34^.

Enrichment of footprints in the stage-specific open chromatin clusters highlighted TF families binding to the motif as potential stage-specific regulators. These data provide an additional layer of information to the αβ T cell factors^28^ predicted from lineage specific gene expression profiles. The most immediate future plans following these identifications are to investigate whether or not each bound sequence is necessary for any specific enhancer/silencer activity, which can be tested by *in vivo* genomic DNA mutation of the TF motif. In summary, the genome-wide view of open chromatin presented here as well as the identification of the sequence motifs bound by TFs at four different stages of thymocyte development is a useful point from which to begin to assemble precise models for transcriptional regulation of T cell stage-specific gene expression.

## Methods

### ATAC-seq

ATAC libraries were prepared as described previously^17^. In brief, 50,000 to 100,000 DN, DP, CD4 SP and CD8 SP thymocytes were isolated by flow cytometry (Supplementary Fig. S1). Cells were processed for ATAC reaction, and then the ATAC libraries were PCR amplified with barcoded primers. The ATAC-seq libraries were paired end 75 bp sequenced on a HiSeq 4000 at the UM Sequencing Core. Raw reads were trimmed for barcodes and aligned to the mm10 reference genome using BWA^19^; duplicates were removed with Picard and then filtered for high quality (mapq ≥ 30), properly paired alignments and uniquely mapped as described in our previous study^12^.

### Peak calling

In order to account for sequencing depth differences between each library, we down-sampled reads (keeping read pairs intact) to the median depth of all libraries after the pruning steps described above. This ensures that sequencing depth would not confound the analysis. After this step, we combined all replicates from each stage into a single BAM file to increase sequencing depth (ranging from 120 to 134 million reads per stage) and called peaks using MACS2^20^ with options *–nomodel –shift -50 –extsize 100 -B –keep-dup all*. For testing the reproducibility between samples, we generated a set of regions that were called (narrow) peaks in at least one of the merged samples, retrieved the number of fragments mapping to these regions in each replicate and calculated the pairwise Pearson correlations between all replicates of the same stage.

### *k*-means clustering and functional enrichments

To perform k-means clustering, we generated a set of genomic regions that were called peaks in at least one of the samples (master peaks list) by using bedtools merge in the combined MACS2 output for all samples. For each sample, we calculated the FPKM in each of the master peaks regions, and normalized the signal by dividing the values by the TSS enrichment of the sample, which accounts for the signal-to-noise ratio, and then applied robust IQR scaling $({X}_{scaled}=\frac{{x}_{i}-median(X)}{IQR(X)})$, where IQR is the distance between the 1^st^ and 3^rd^ quartiles, to make the values comparable across samples. This signal was then row-wise normalized by the maximum of every sample $({Y}_{normalized}=\frac{{y}_{i}}{{\rm{\max }}(Y)})$. Using this matrix of genomic coordinates per samples, we ran the k-means implementation available in R 3.3.1 for *k* = 1, 2, …, 15 *k* values and determined that *k* = 6 was suitable for our analyses. Increasing *k* to higher values only marginally decreased variance and yielded repetitive clusters patterns, with 1,000 random starts for robustness. We analyzed the within cluster variances for all (Supplementary Fig. S5). In order to perform functional annotation of the clusters, we used the ChIP-Enrich R package^24^, which allow us to directly compare the enrichment scores and *p* values for the same GO terms across samples.

### PWM scans and ATAC-seq footprints

In the current study we focused on TF binding motifs for T cell activators and repressors that were predicted by Jojic *et al*.^28^ from stage-specific gene expression profiling (171 αβT cell factors, Supplementary Table 10 in ref.^28^). Position weight matrix (PWMs) for each motif was obtained from ENCODE^35^, JASPAR^36^ and TF pairs identified by Jolma *et al*.^37^. Total 417 binding motifs for 67 out of 171 Jojic αβ T cell factors were derived from these databases.

We scanned the mm10 genome for the PWMs for the 417 motifs using FIMO^38^ with the G-C content background frequency for mm10 (41.7%), and used the default 10^−4^ P value threshold, also filtering for motif occurrences intersecting regions with known mapability issues (blacklisted regions). CENTIPEDE^25^ was used to call footprints from the ATAC-seq data as we have done previously^12,13^. Briefly, for each PWM scan result we generated a strand-specific (relative to the motif orientation) single base pair resolution matrix encoding the number of Tn5 transposase integration events in a region ±100 bp from each motif occurrence. A motif occurrence was considered bound if the CENTIPEDE posterior probability was higher than 0.99 and its coordinates were entirely contained by an ATAC-seq peak. To generate the motif occupancy plots for each factor, we aggregated the signal used as input for CENTIPEDE for all the predicted bound motifs, as well as an equal number of motifs with posteriors less than or equal to 0.5 and not intersecting ATAC-seq peaks in that sample. The normalized signal plotted was obtained by dividing the bound signal by the unbound.

### Overlap of ATAC-seq footprint and ChIP-seq

In order to test the correspondence between footprint calls and ChIP-seq data for GATA and CTCF, we used GAT^27^ with the workspace set as all the GATA or CTCF motif matches in the mm10 genome, the respective ChIP-seq peaks as the segments, and the respective CENTIPEDE footprint calls as the annotation. By limiting the workspace only to the specific motifs, the data stringently delimit the space for genomic interval overlap testing. The footprint enrichments in the ATAC-seq clusters were performed separately for each sample and for each motif. We used as workspace all the motif occurrences within the master peaks regions (see *k*-means clustering above) for the individual motif being analyzed. As annotations, we used the cluster designations from the *k*-means analysis. The segments were all the footprints for that motif in that sample. Additionally, we used the option -n to 1,000 in order to increase statistical robustness. This resulted in a table with the GAT results for every motif in each cluster and in each sample.

### Data Availability

ATAC-seq and footprint data have been deposited in GEO database^39^ and accessible through accession number GSE107076.

## Electronic supplementary material

Supplementary Information
