# Supplementary material for: Global dynamics of stage-specific transcription factor binding during thymocyte development
Source: Sci Rep. 2018 Apr 4;8:5605. doi: 10.1038/s41598-018-23774-9 (PMC5884796; doi:10.1038/s41598-018-23774-9)
Supplement: Supplementary file 1 — Supplementary Information [file 41598_2018_23774_MOESM1_ESM.pdf]

Supplementary Information

Global dynamics of stage-specific transcription factor binding during thymocyte development

Tomonori Hosoya[1,*], Ricardo D'Oliveira Albanus[2,*], John Hensley[2], Greggory Myers[1], Yasuhiro Kyono[2,3], Jacob Kitzman[2,3], Stephen C. J. Parker[2,3] and James Douglas Engel[1]

[1]Department of Cell and Developmental Biology

[2]Department of Computational Medicine and Bioinformatics

[3]Department of Human Genetics

University of Michigan

3035 BSRB

109 Zina Pitcher Place

Ann Arbor, Michigan 48109-2200

* Co-first authors

Supplementary Figure S1. Isolation of staged thymocytes. (a) Individual stages of thymocytes were isolated using a FACSAria III (BD). Representative dot plots from 1 mouse (out of 4 animals examined in two different experiments) are shown. Area Scaling was set with total thymocytes and doublets were gated out using FSC-A vs. FSC-H. PI was used to discriminate dead from live cells. The gates (blue) are shown for DN ($Lin^-CD4^-CD8^-CD3^-$), DP ($Lin^-CD4^+CD8^+$), SP4 ($Lin^-CD4^+CD8^-CD3^+$) and SP8 ($Lin^-CD4^-CD8^+CD3^+$) cells. The numbers near the boxed areas indicate the mean percentage of cells in each gate. The lineage cocktail used was a mixture of e450-conjugated antibodies recognizing TER119 (TER119), B220 (RA2-6B2), CD19 (1D3), Mac1 (M1/70), Gr1 (RB6-8C5), CD11c (N418), NK1.1 (PK136) and gdTCR (GL3). Cells were also stained with PECy-CD4 (RM4-5), APC-CD8 (53-6.7) and PE-CD3e (145-2C11) purchased from eBiosciences or BioLegend. (b) A small fraction of the sorted cells were reanalyzed to check for purity.

Supplementary Figure S1

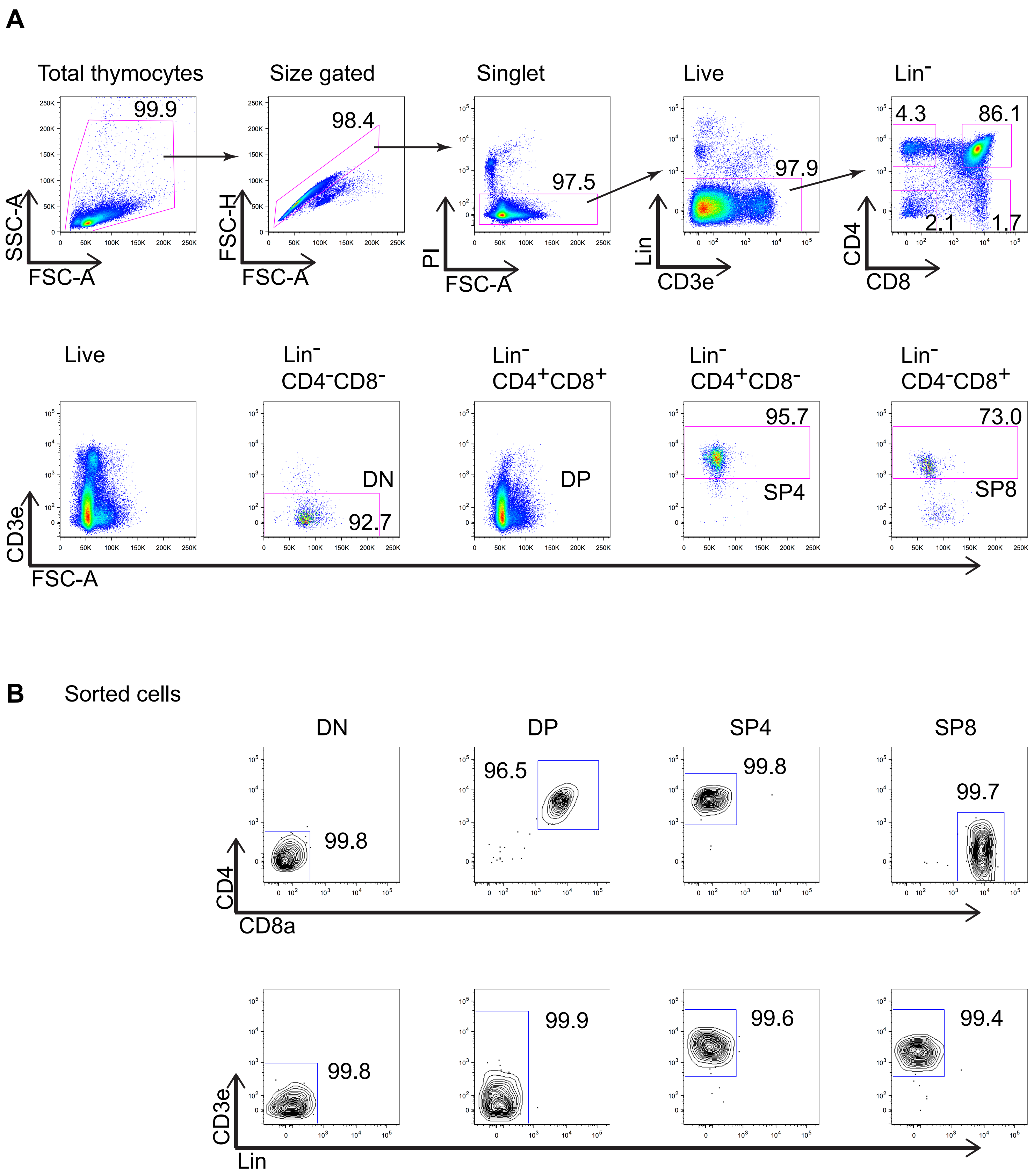

Supplementary Figure S2. ATAC-seq profiles of thymocytes. ATAC-seq signals (MACS2 bedgraph converted to bigwig format) and peaks (MACS2 broad peak calling) surrounding the *Cd4* (a), *Cd8* (b) and *Trb* (c, encoding TCRβ), *Gata3* (d) loci and *TCE1* enhancer for *Gata3* gene (e). Data are on the IGB browser around 50 kbp of the *Cd4* locus (a, mm10, chr6:124,860,001-124,910,000), around 100 kbp of the *Cd8* locus (b, chr6:71,300,001-71,400,000), around 50 kbp of *Trb* gene beta enhancer (c, $E\beta$, chr6:41,520,001-41,570,000), within +/- 1.2 Mbp of the *Gata3* gene (d, chr2: 8,600,001-11,000,000) and around *TCE1* enhancer for *Gata3* gene (e). (Top) ATAC-seq peaks were generated in quadruplicate in order to analyze chromatin accessibility in DN (orange), DP (green), SP4 (pink) and SP8 (blue) stage thymocytes. (Bottom) ATAC-seq peaks in DP stage (green), which compose approximately 85% of total thymocytes, were compared with DNase-seq (DHS, middle, ENCODE, ENCSR000COB, isogenic replicate 1 and 2) and H3K27ac ChIP-seq (bottom, ENCODE, ENCSR000CCH, isogenic replicate 1 and 2) in thymocytes.

Supplementary Figure S2

a

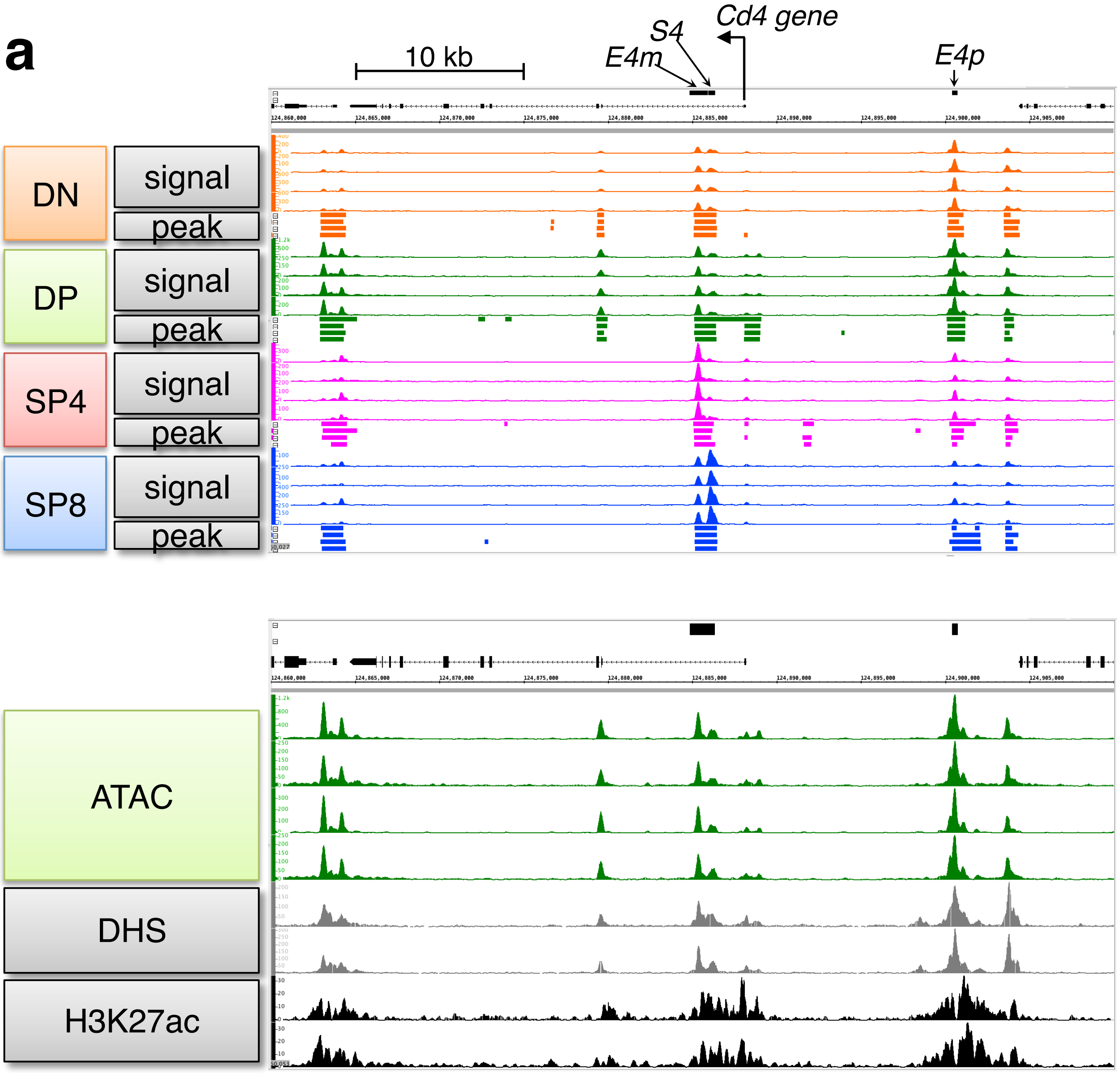

Supplementary Figure S2 (cont'd)

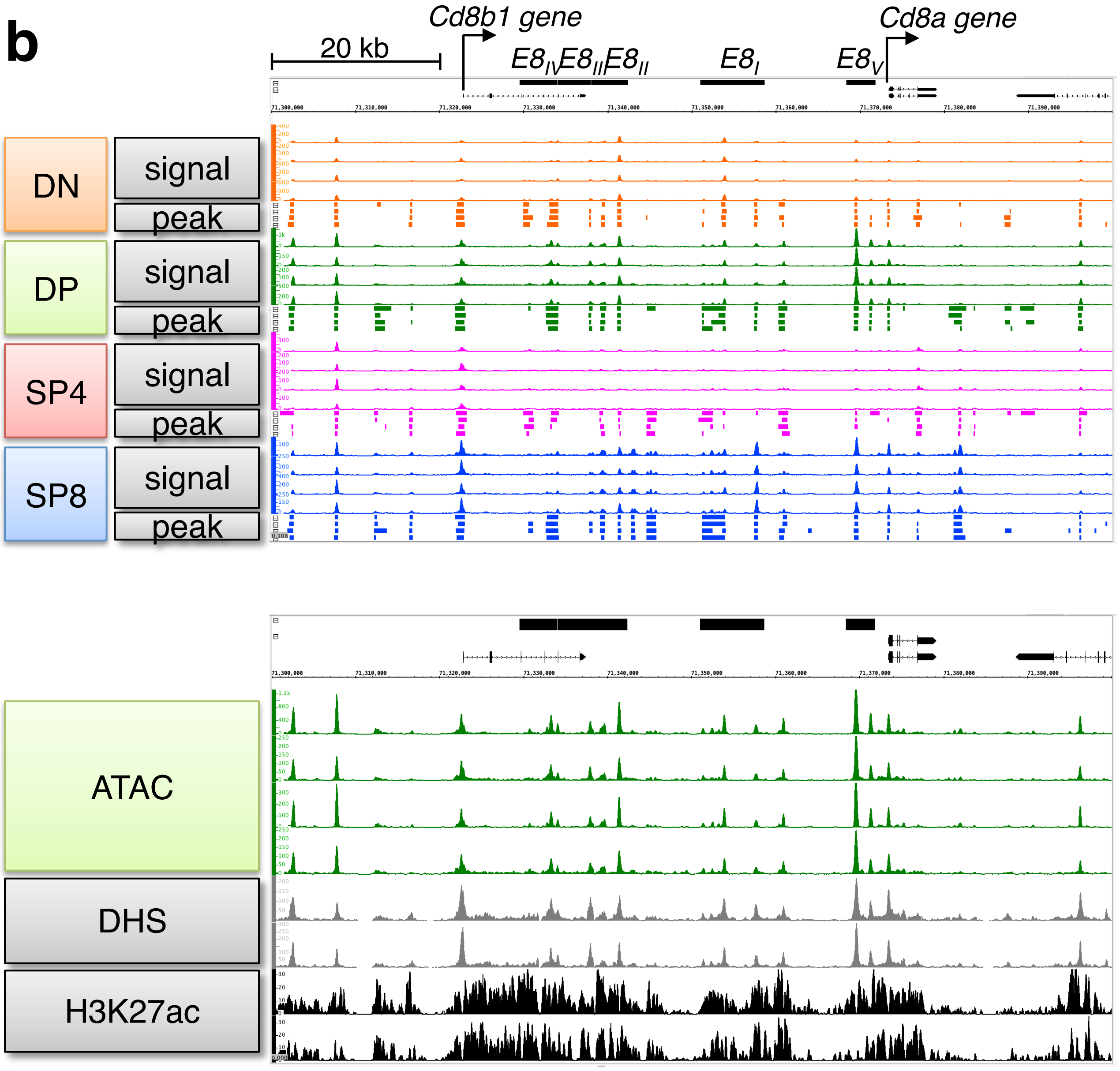

Supplementary Figure S2 (cont'd)

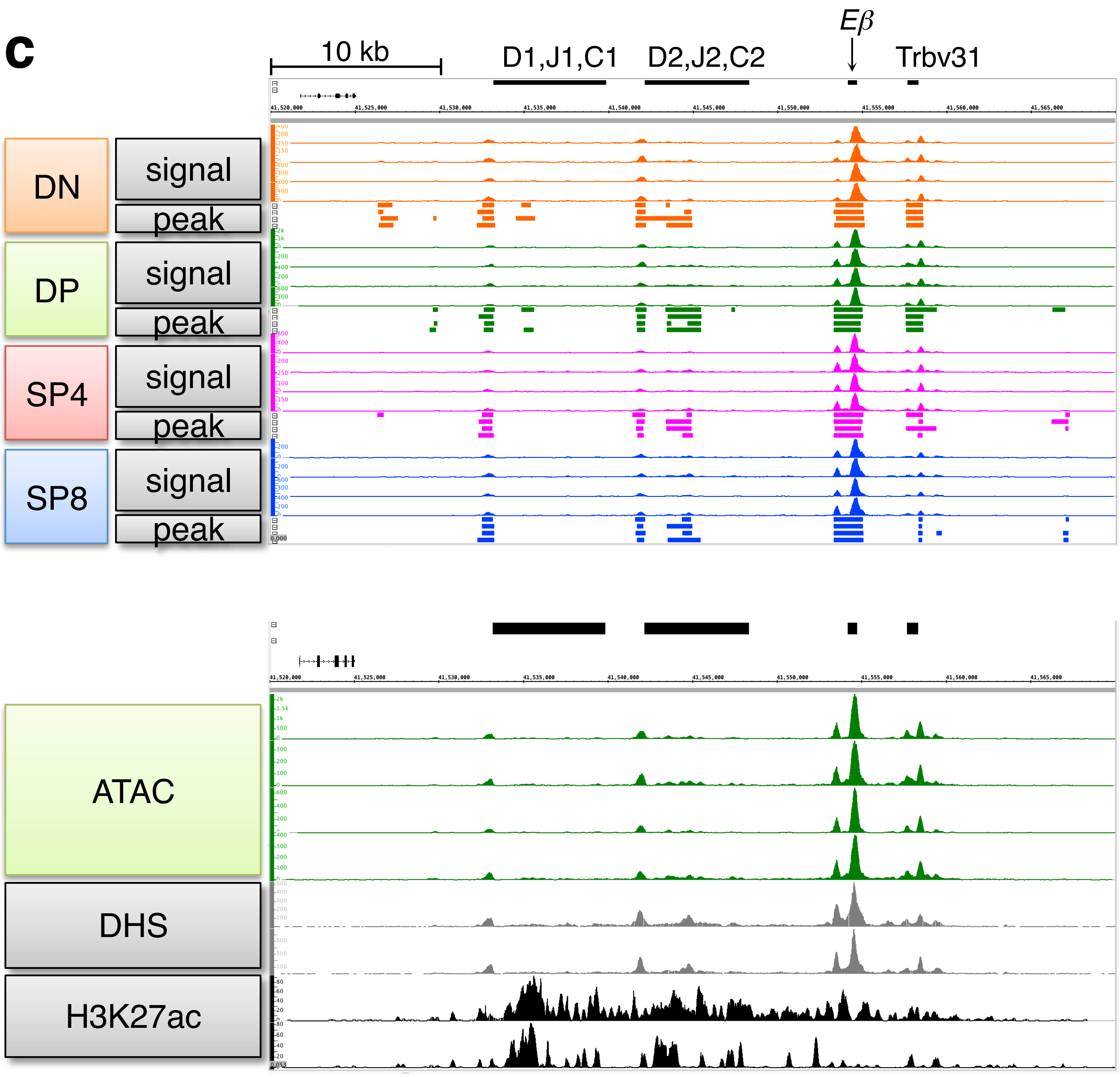

Supplementary Figure S2 (cont'd)

d

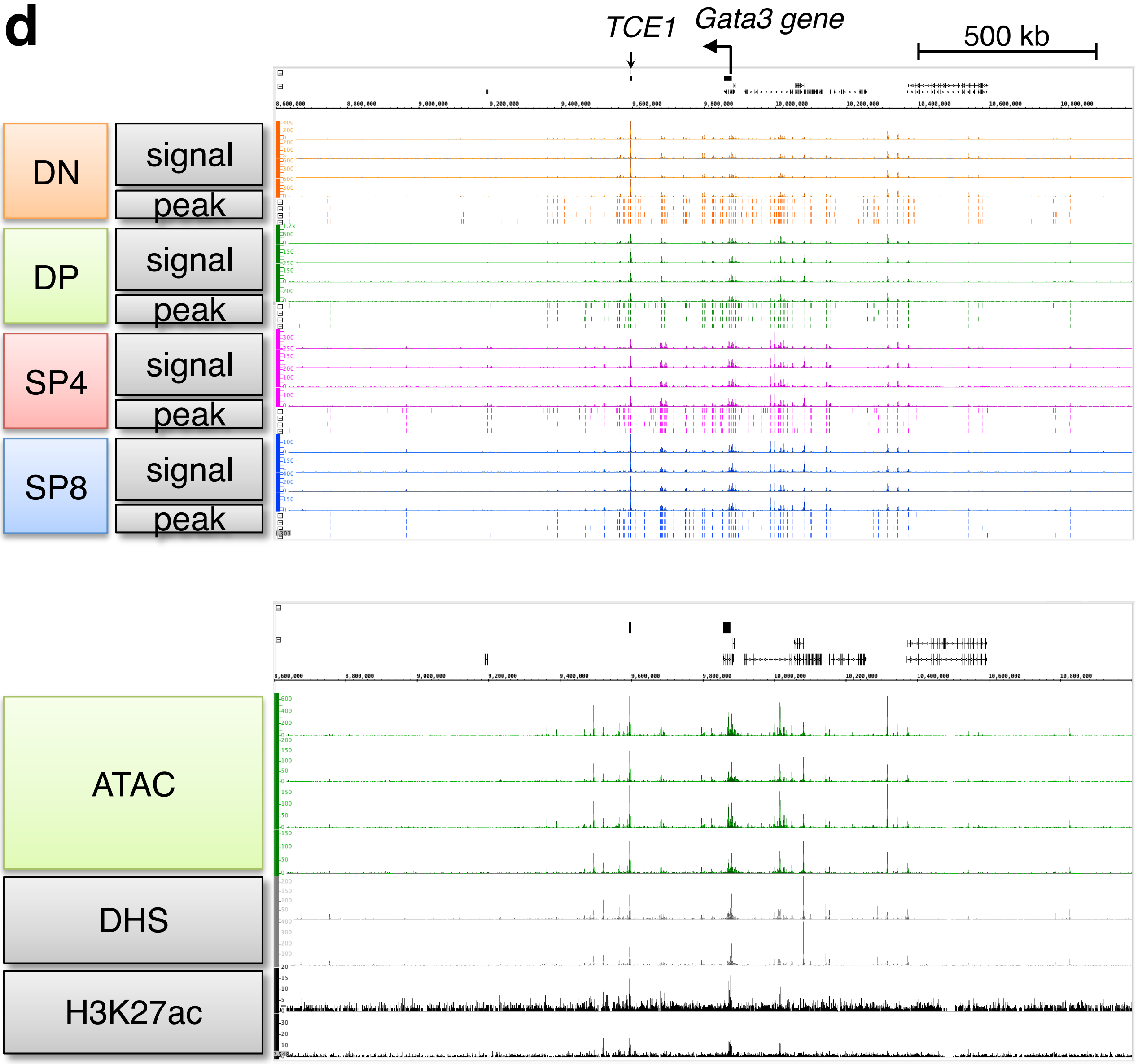

Supplementary Figure S2 (cont'd)

e

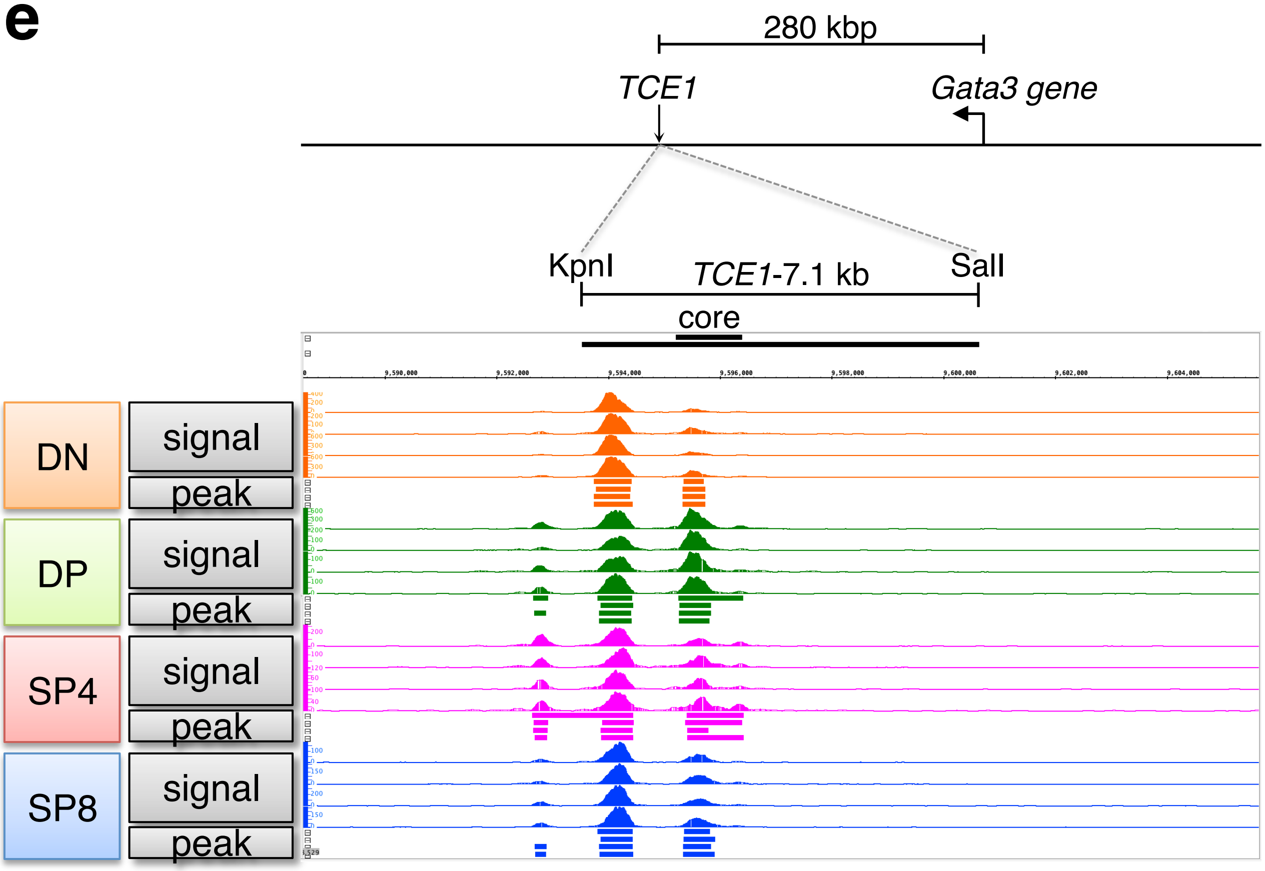

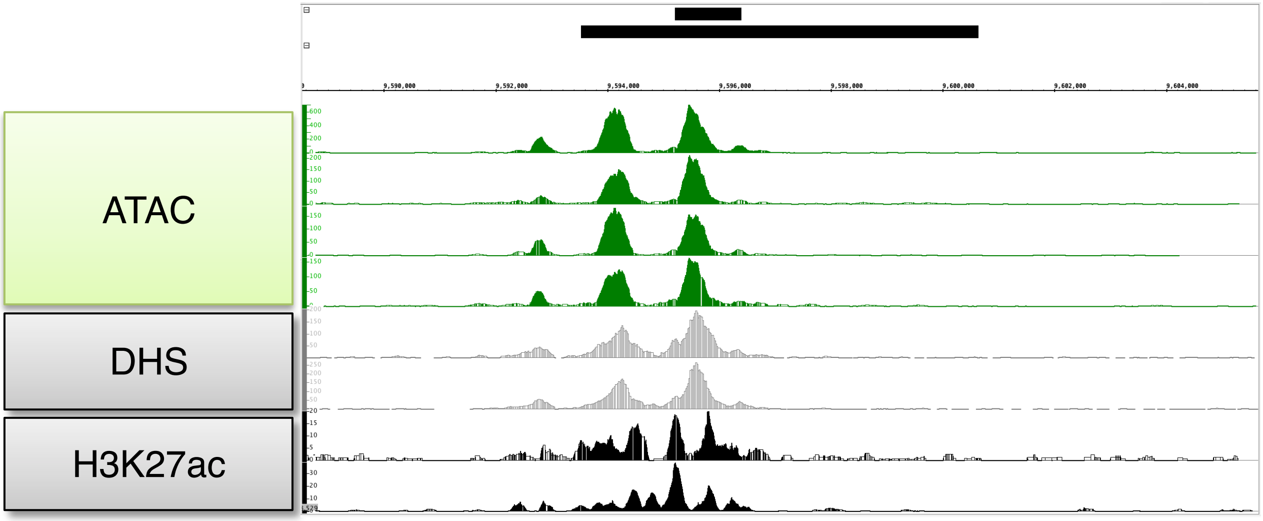

Supplementary Figure S3. **Correlation of the ATAC-seq signal between replicates**. (a) Correlation between ATAC-seq libraries. (b) Correlation between DP ATAC-seq libraries and adult thymocytes DNase-seq data from total adult thymocytes (ENCODE, ENCSR000COB) [1]. Bottom facets: each data point corresponds to an ATAC-seq peak that was called in at least one sample (see Methods). The values plotted are the number of fragments in each peak in the corresponding samples (labelled on the diagonal), and the red and black lines correspond, respectively to the linear model fit from the two datasets and the identity (*i.e.* $x = y$). Upper facets: Spearman correlation values for the comparisons.

Supplementary Figure S3

a

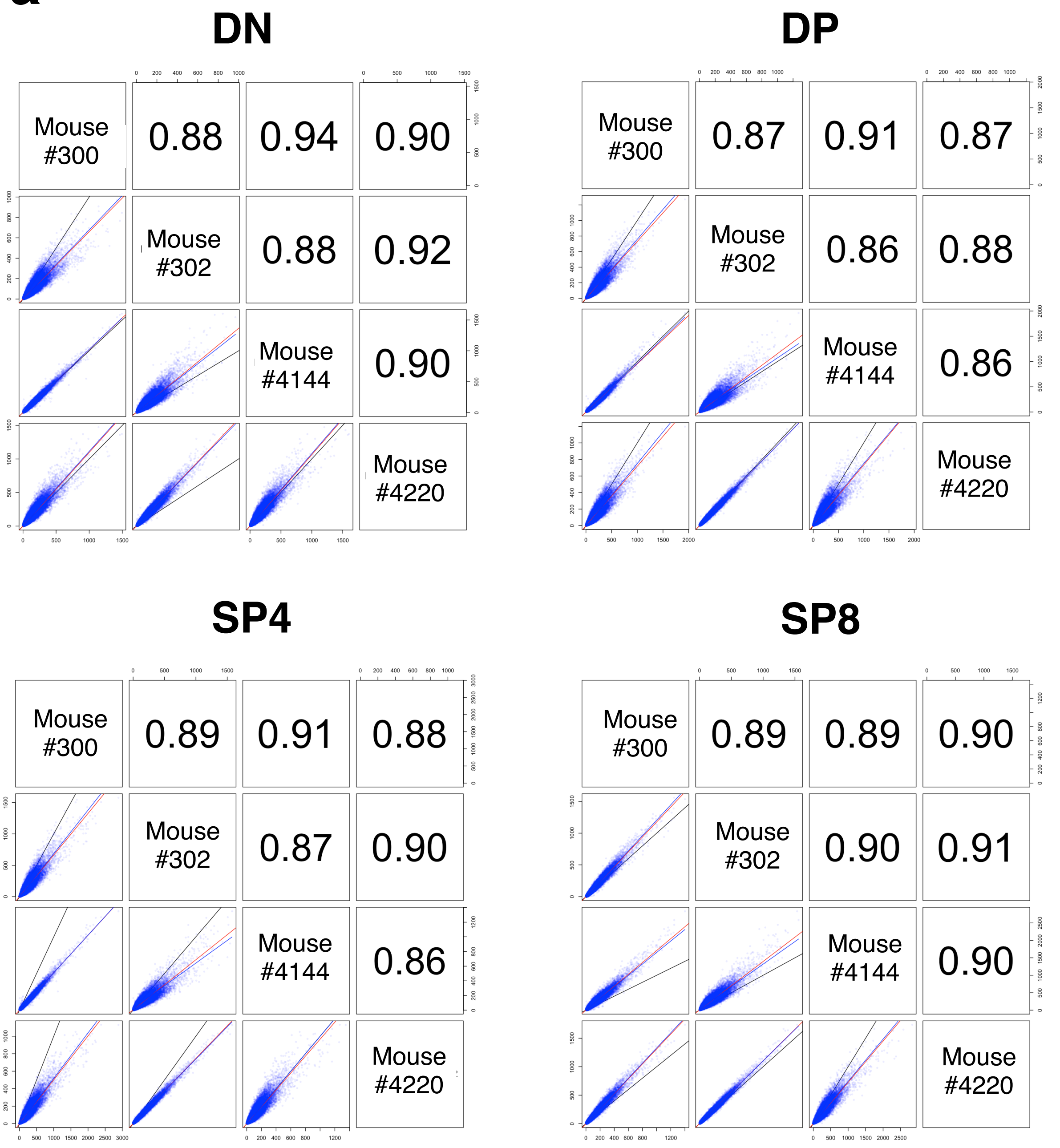

Supplementary Figure S3 (cont'd)

b

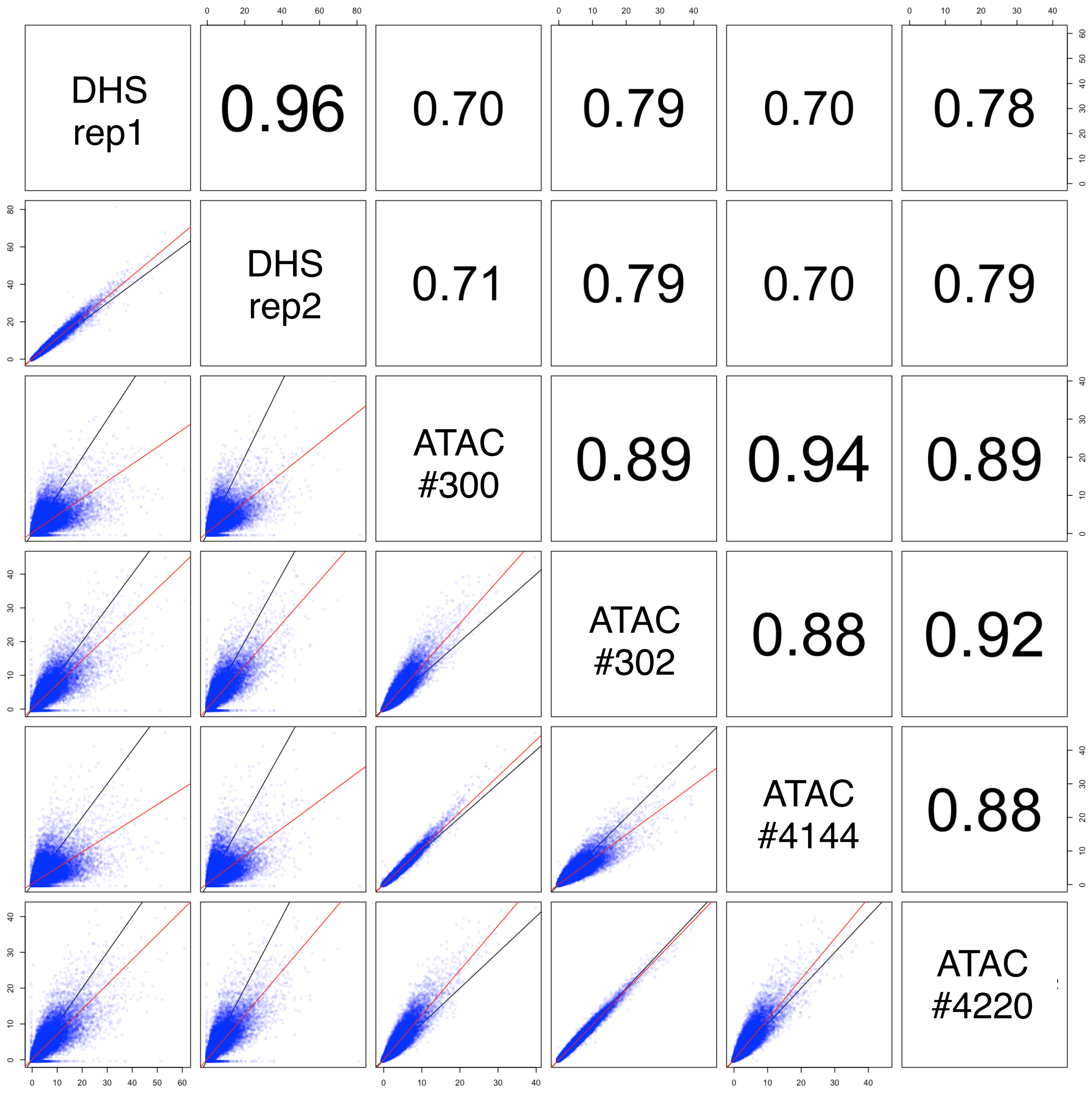

Supplementary Figure S4. Open chromatin regions defined by ATAC-seq peaks. (a) Number of ATAC-seq peaks in DN (orange), DP (green), SP4 (pink) and SP8 (blue) stage thymocytes. (b) Total length of ATAC-seq peaks assigned by MACS2 (narrow peak calling) is shown as a fraction of the whole mouse genome length. (c) The overlap among ATAC-seq peak calls at the four stages examined in this study is shown as a Venn diagram. (d) ATAC-seq peaks that overlap within 200 bp 5' to a gene were characterized as promoters (left). The peaks that overlap with exons, but are not with the promoters, are shown as exons (middle). The peaks that overlap with neither promoters nor exons are characterized as distal (right). The gene annotations were downloaded from the UCSC Table Browser. Annotations for RefSeq genes and UCSC genes are combined.

Supplementary Figure S4

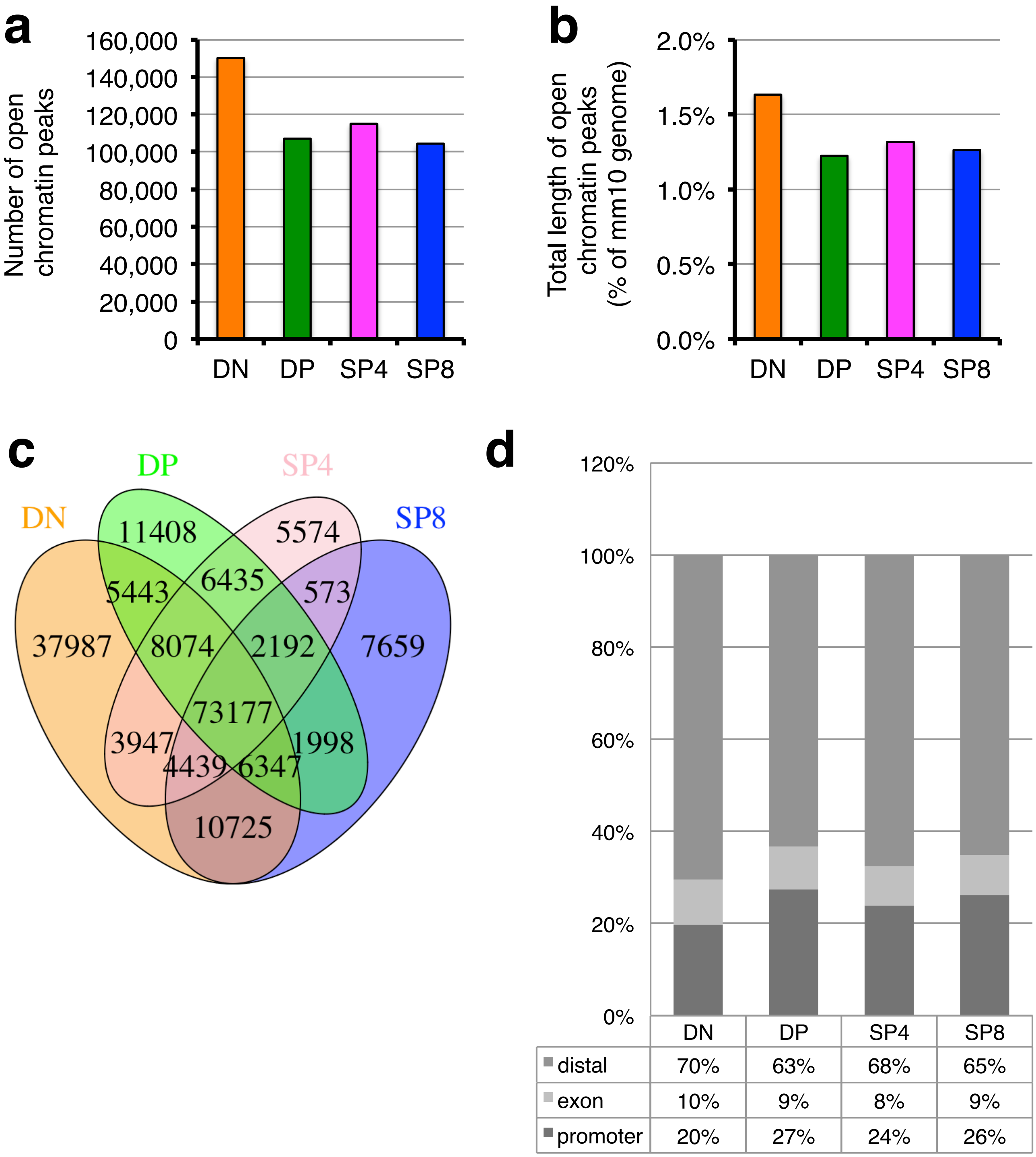

Supplementary Figure S5. Additional information on *k*-means clustering. Distribution of the ATAC-seq signal in the master peaks before (a) and after (b) normalization. (c) Elbow plot showing variances within clusters for each value of *k*.

Supplementary Figure S5

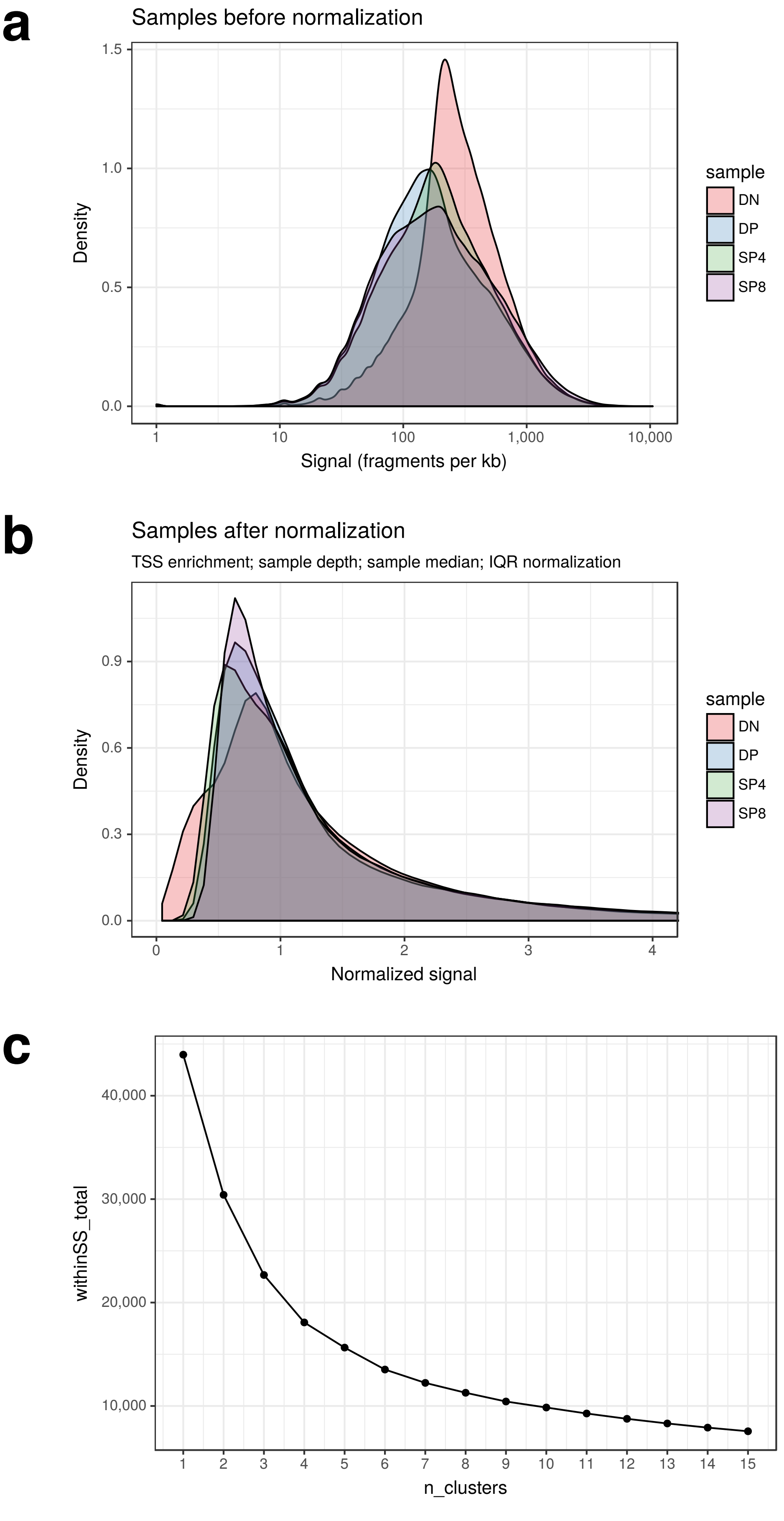

Supplementary Figure S6. ChIP-Enrich results for the ATAC-seq clusters. GO term enrichment values for the top 15 terms called in each of the clusters and their corresponding enrichments for all clusters.

Supplementary Figure S6

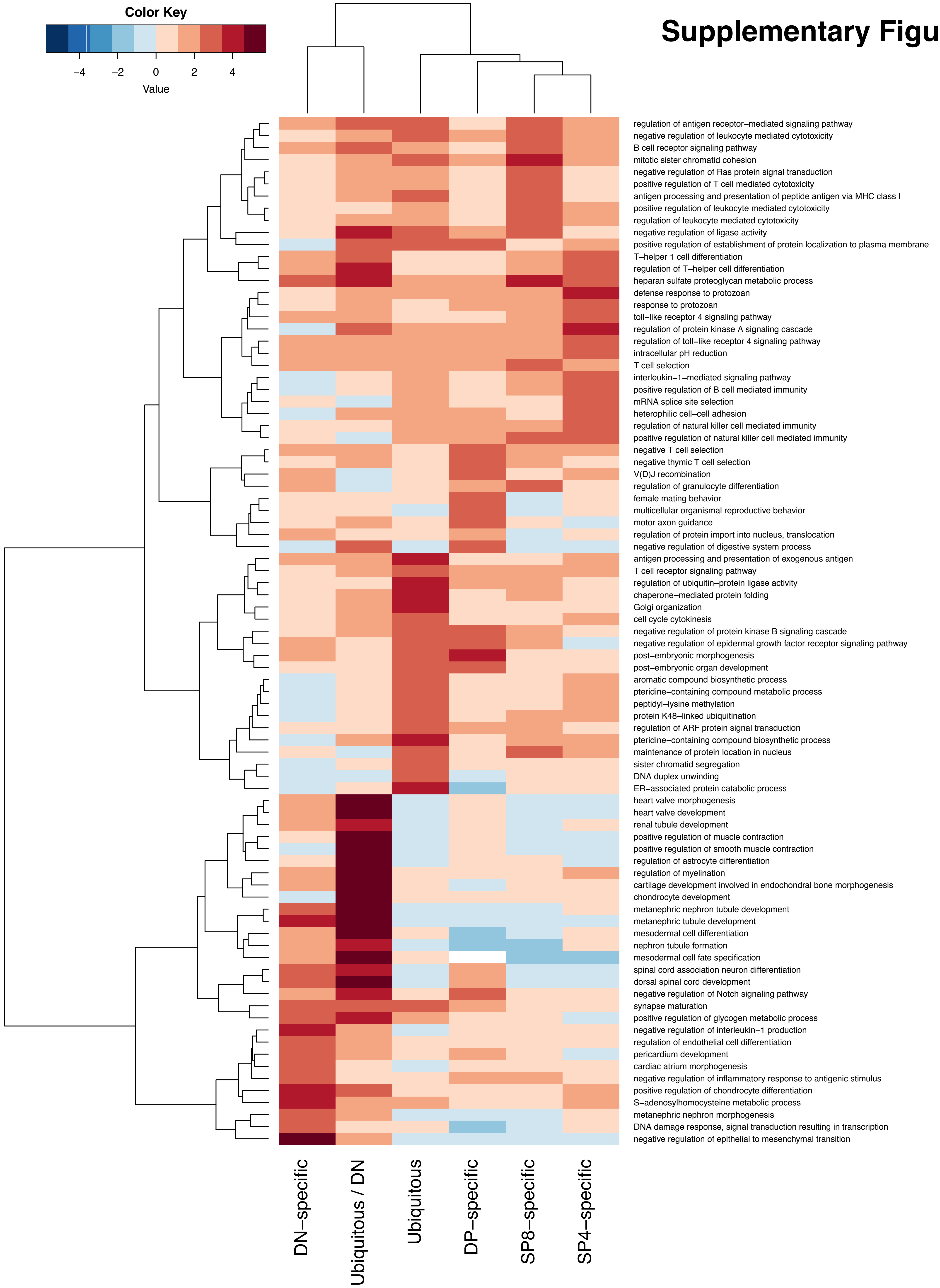

Supplementary Figure S7. Footprint enrichment results from GAT. Heatmaps showing GAT enrichments of each of the motifs for the individual *k*-means clusters in (a) DN, (b) DP, (c) SP4, and (d) SP8 samples.

Figure S7a DN

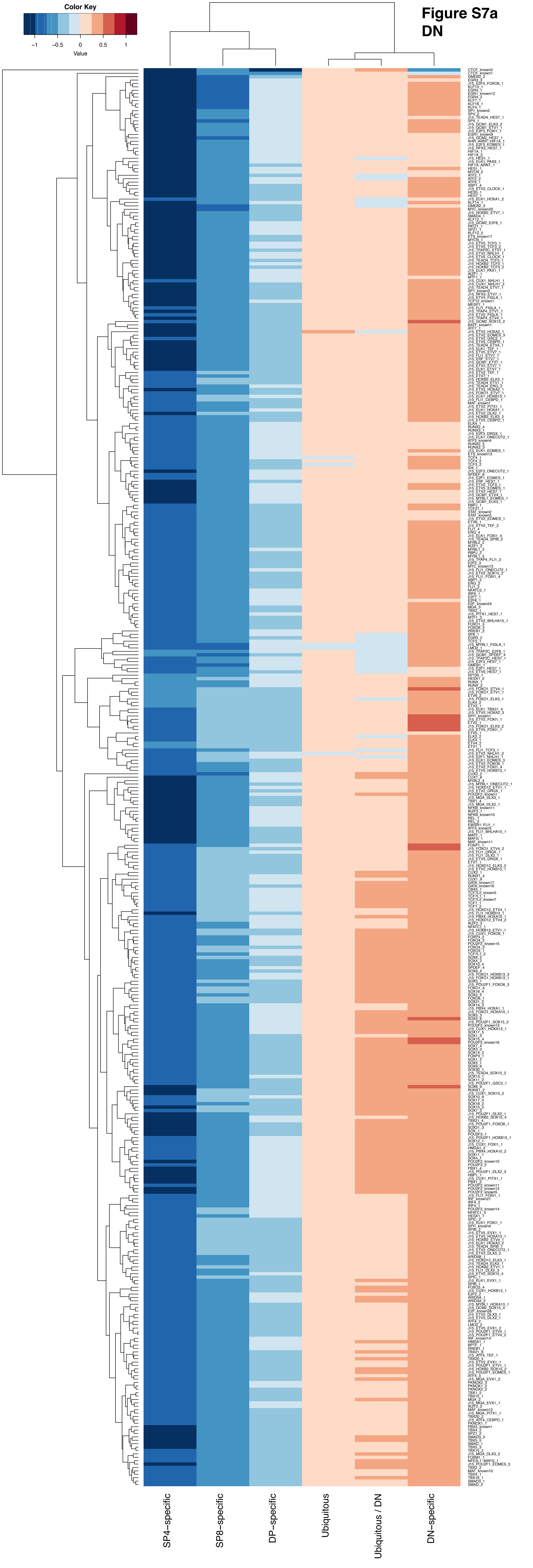

Figure S7b
DP

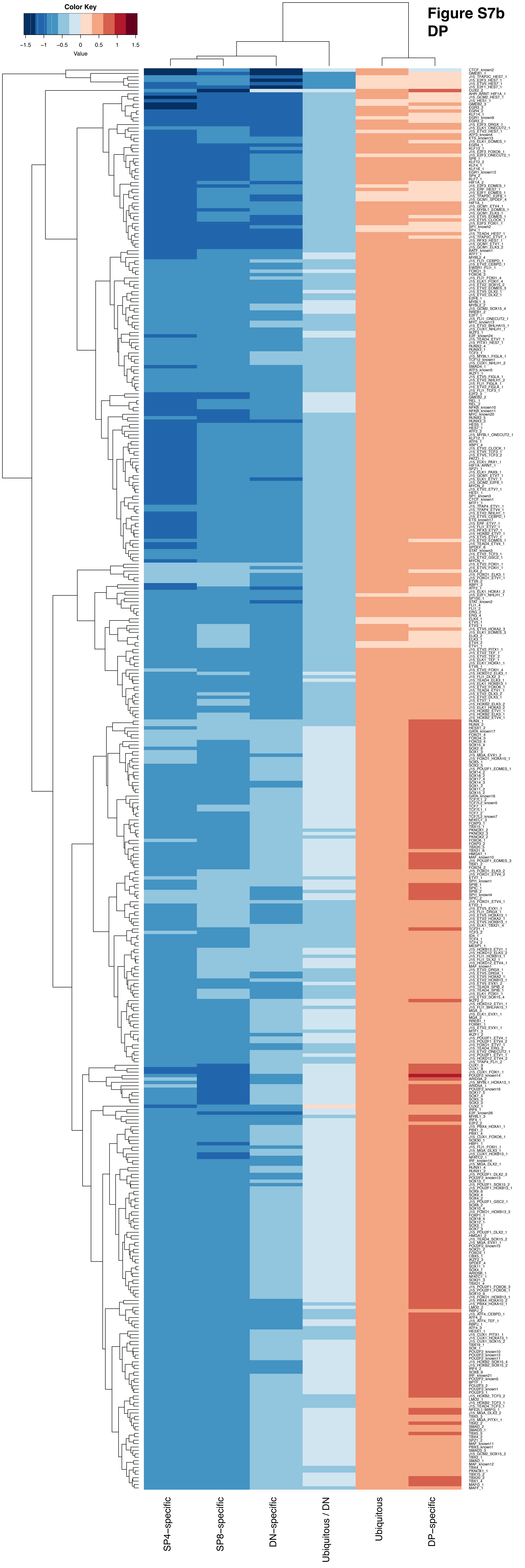

Figure S7c
SP4

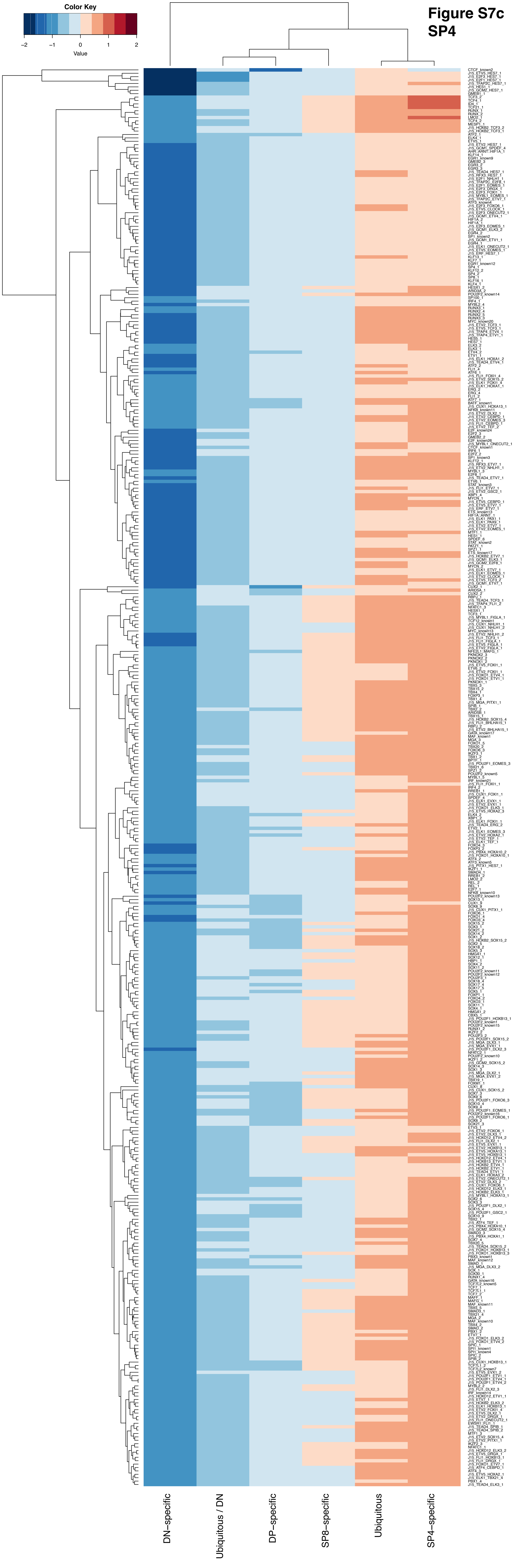

Figure S7d
SP8

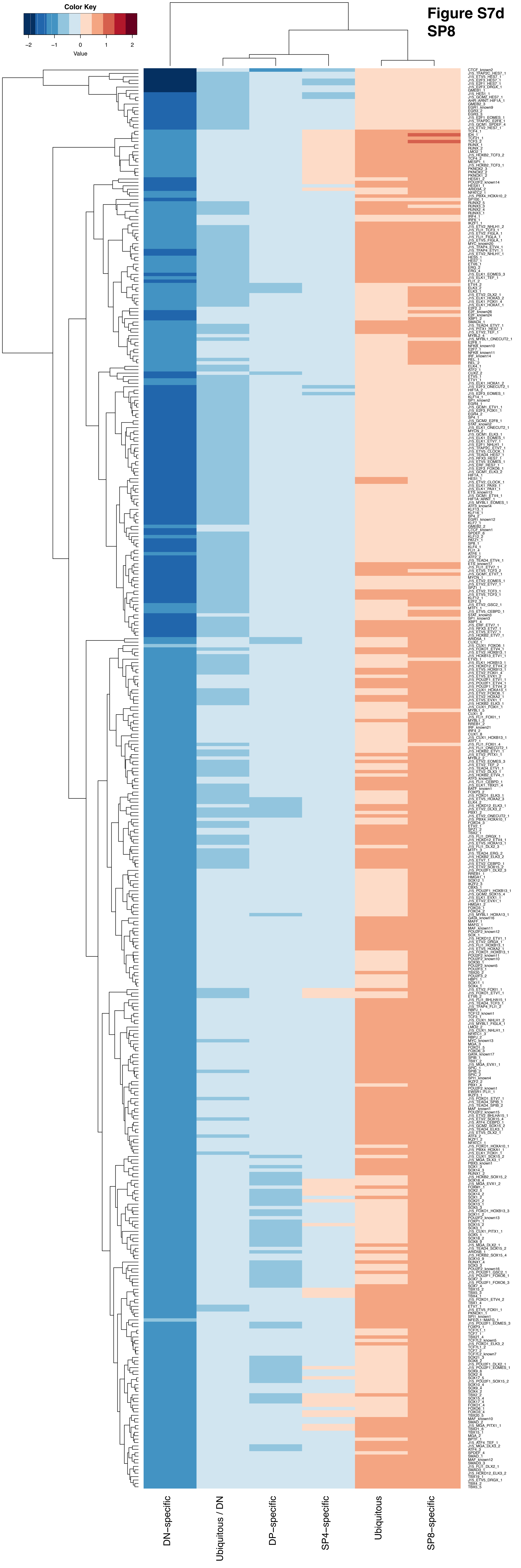

Supplementary Figure S8. CENTIPEDE footprint calls within functionally validated regulatory elements for the *Cd4* gene. Related to Fig. 2. ATAC signals and footprint calls around *S4* silencer (a) and *E4m* enhancer (b). Transgenic reporter analyses have shown that *Cd4* transcription is regulated by at least two proximal enhancers (*E4p* and *E4m*) and a silencer (*S4*) [2-4]. In mice ablated for *E4p* only, CD4 expression was reduced in pre-selection DP thymocytes [5] but was completely abrogated when both *E4p* and *E4m* were deleted [6]. Removal of *S4* from the mouse genome resulted in ectopic *Cd4* expression in DN and SP8 cells, which are both CD4-negative in wild type mice [7-9]. We identified ATAC-seq open chromatin regions in the *Cd4* locus *E4p*, *E4m* and *S4* (Fig. 2). The ATAC-peak at *E4p* belongs to DP-specific cluster shown in Fig. 1a, while the ATAC-peak found in *E4m* belongs to SP4-specific cluster, in keeping with its developmental function [5,6]. The open chromatin at *S4* belongs to SP8-specific cluster, as would be expected from its *Cd4* gene silencing function. Minor open chromatin regions were also found approximately 3 kbp 5' to *E4p*, as well proximal to the *Cd4* 1st and 2nd exons. Our digital footprint data in mouse primary thymocytes identified approximately 10 sequences bound by protein within *E4p, S4* and *E4m*, respectively. Based on known and novel protein binding found here, we propose that approximately 10 proteins shown in bind to/around *E4p, S4* and *E4m* in order to contribute to activity of the enhancers/silencer.

Supplementary Figure S8

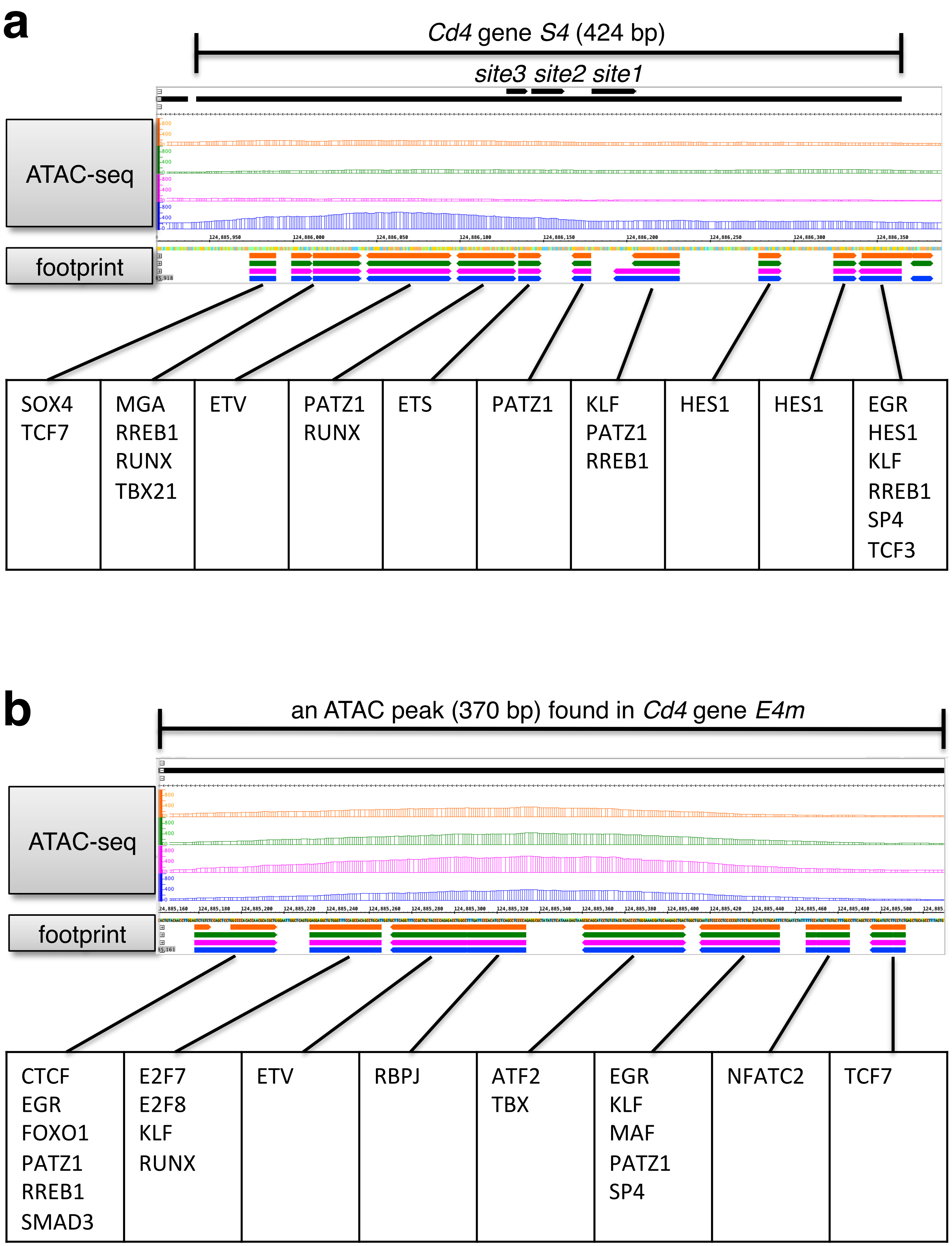

Supplementary Figure S9. CENTIPEDE footprint calls within functionally validated enhancers for the *Cd8* gene. Related to Fig. 3. ATAC signals and footprint calls within peaks found in $E8_I$ silencer (a,b) and $E8_V$ enhancer (c,d). Transgenic reporter analysis showed that expression of the *Cd8* genes is regulated by multiple enhancers ($E8_I$, $E8_{II}$, $E8_{III}$, $E8_{IV}$ and $E8_V$) [10-12]. Removal of either $E8_I$/$E8_{II}$ or $E8_V$ from the mouse genome results in reduced CD8 expression [13,14]. In the present analysis, open chromatin regions were identified in the *E8* enhancers and exons, but also revealed the presence of possibly novel and previously undetected regulatory elements (Fig. 3). The footprint data recapitulated TF binding to IKAROS motifs [15], RUNT motifs [16] and PATZ1 [17] motifs within the known *Cd8* enhancers. We conclude that experimental information obtained at the *Cd4* and *Cd8* loci by ATAC demonstrate agreement between protein binding to previously well characterized enhancers (and one silencer) and our footprint predictions, supporting the hypothesis that TF footprints revealed by ATAC-seq provide a reliable complement to experimental ChIP-qPCR and ChIP-seq data. This highlights the use of ATAC-seq to generate highly informative predictions of TF binding to regulatory elements prior to experimental validation.

Supplementary Figure S9

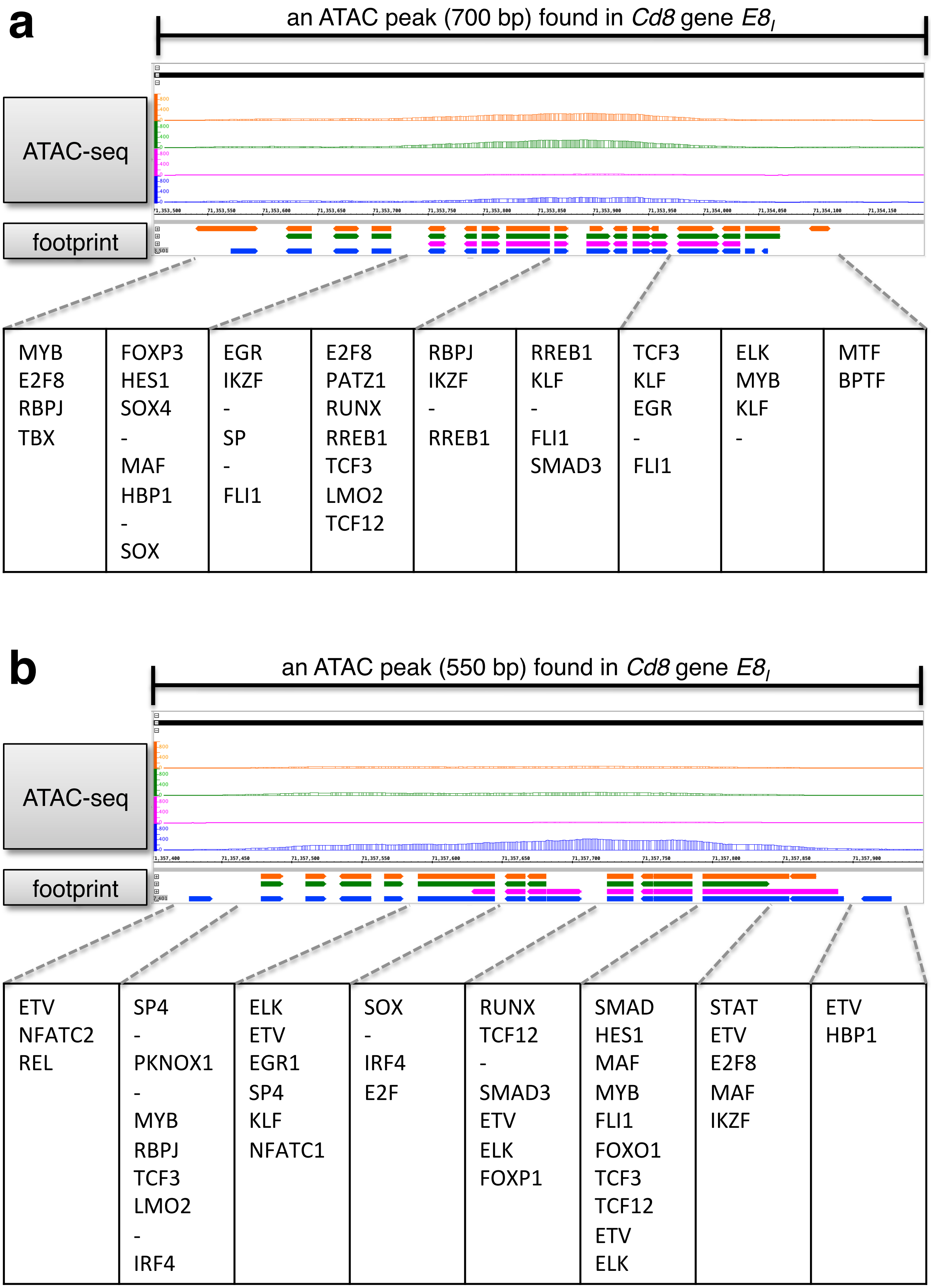

Supplementary Figure S9 (cont'd)

c

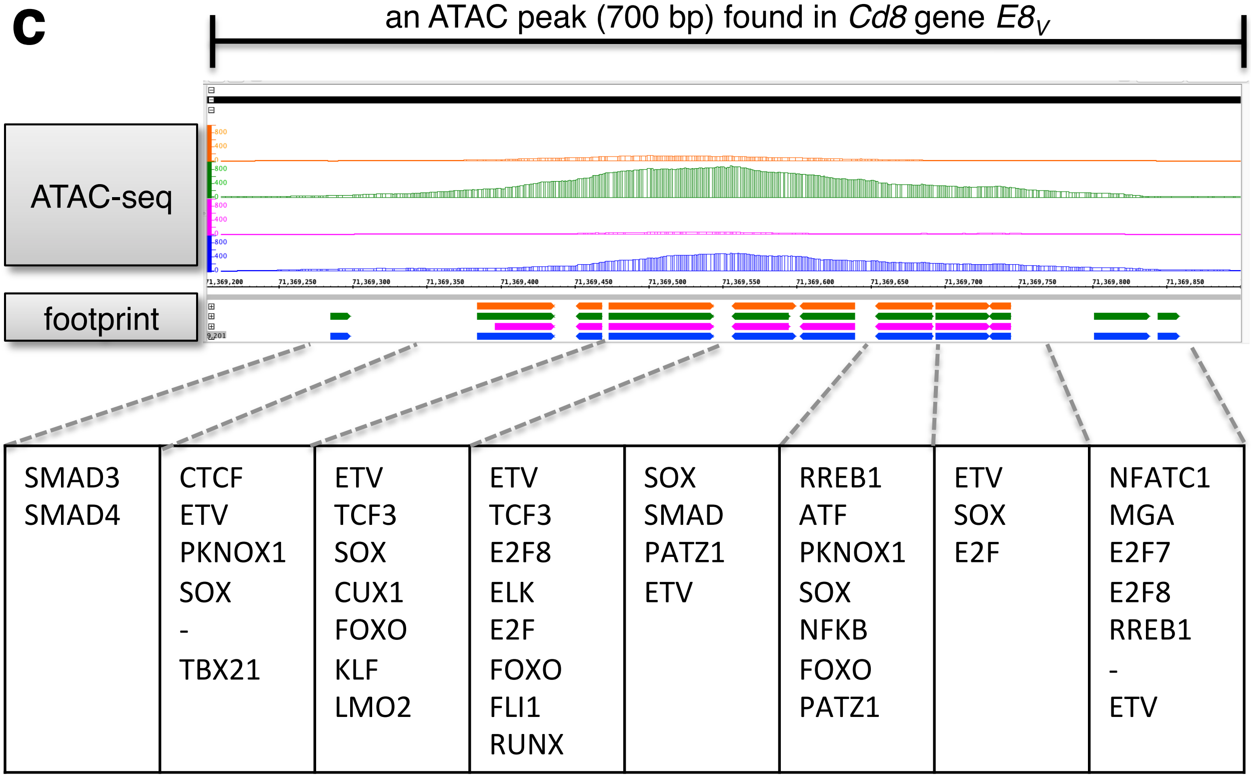

d

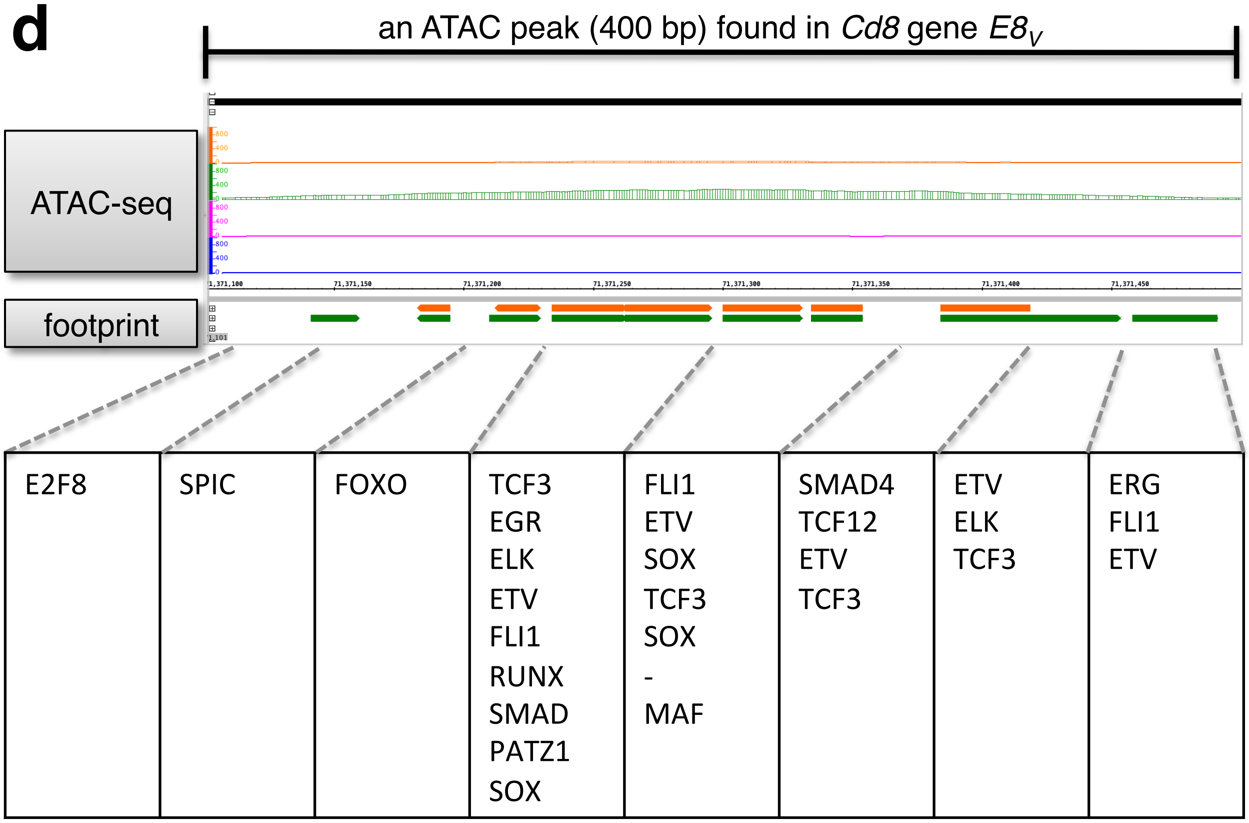

Supplementary Figure S10. ATAC-seq signal and CENTIPEDE footprint calls around functionally validated βE enhancer for the *Trb* gene. (a) ATAC-seq signals are shown on the IGB browser within around 50 kbp of the *Trb* locus (encoding TCRβ); mm10, chr6: 41,520,001-41,570,000. $E\beta$ enhancer, DJC and Trbv31 regions are shown at the top. (b) ATAC signals and footprint calls around $E\beta$ are shown. Deletion of $E\beta$ enhancer from mouse genome blocks αβT cell development [18,19].

Supplementary Figure S10

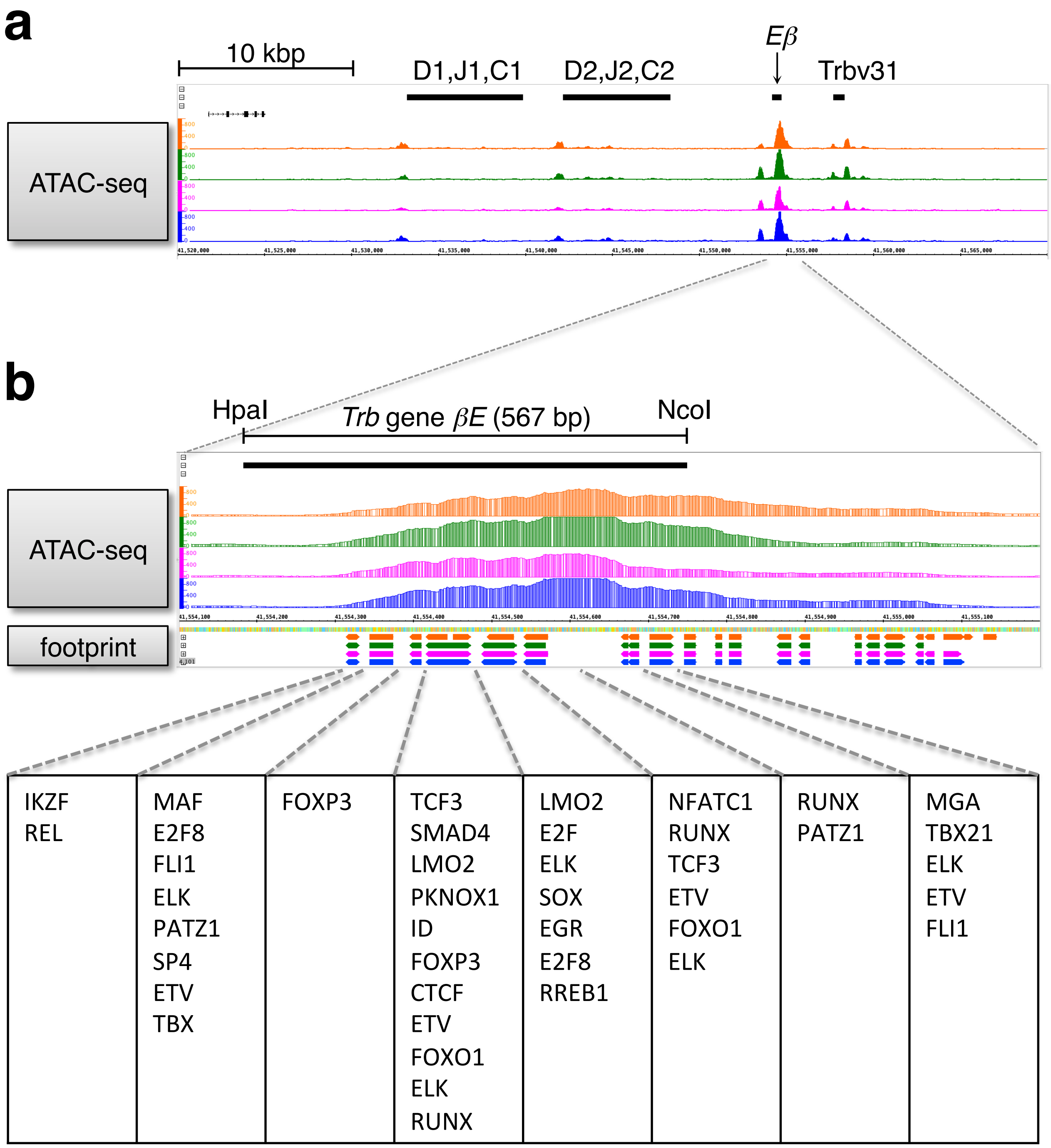

Supplementary Figure S11. Footprint occupancies across samples and clusters. Normalized occupancy signals (see Methods) at ±100 bp of motif center for RUNX1, GATA, TCF3, and ID4. Horizontal facets correspond to the ATAC-seq samples, and vertical facets correspond to the *k*-means clusters.

Supplementary Figure S11

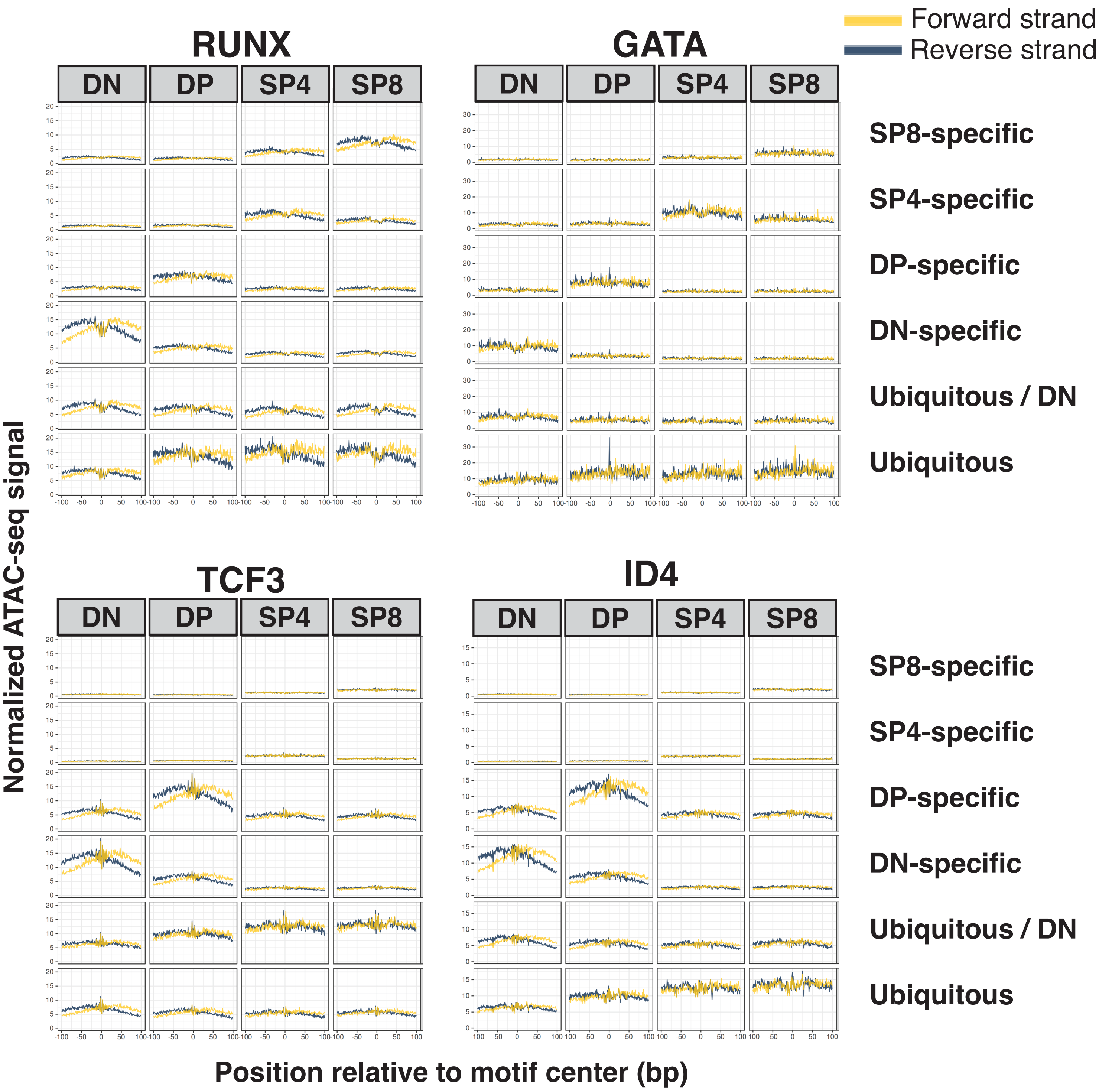

Supplementary Figure S12. HOMER motif enrichment analysis. HOMER known motifs that were called as significant in each of the clusters are plotted with their enrichment values in the color scale.

Supplementary Figure S12

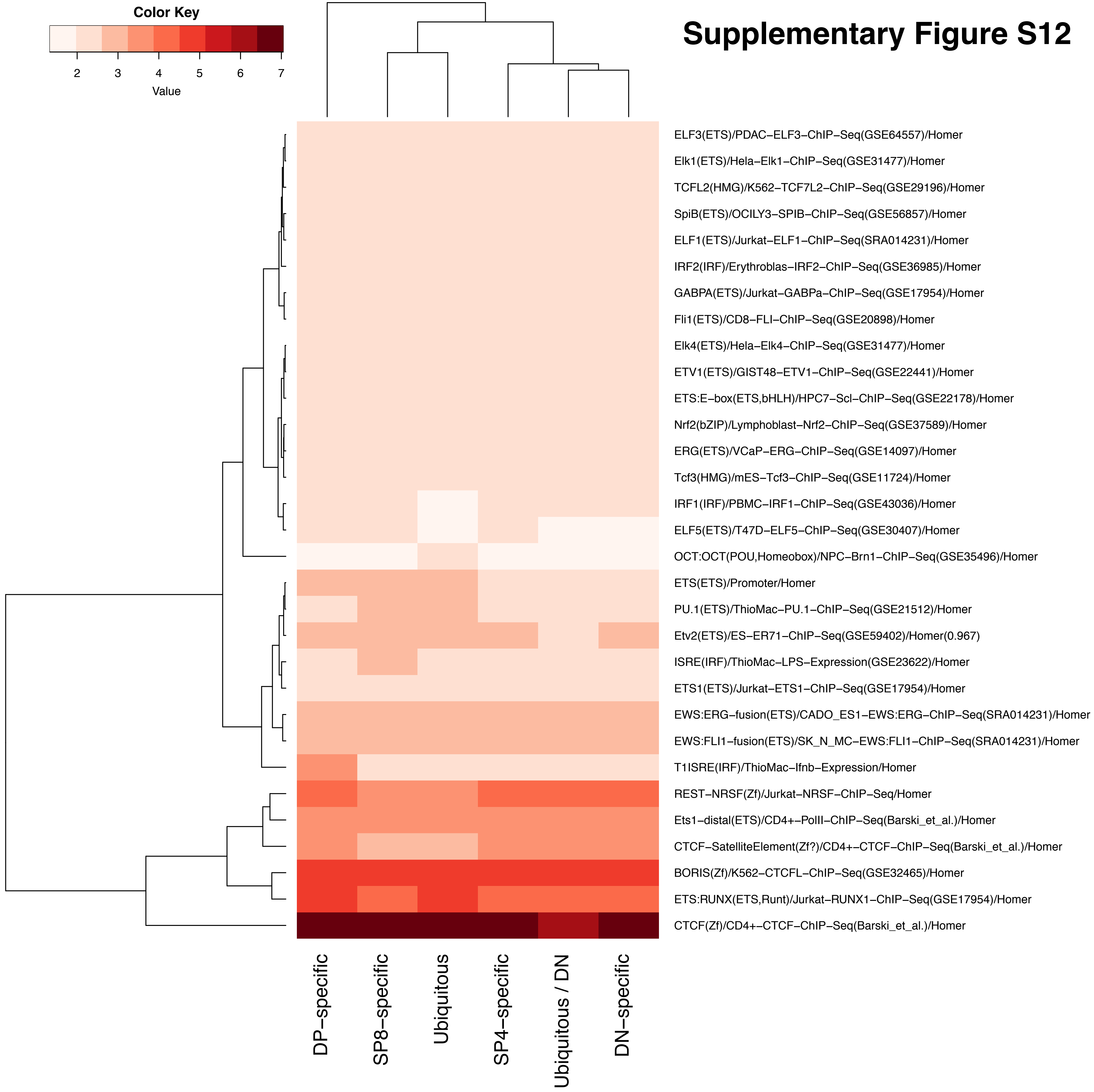

Supplementary References

1. Yue, F. *et al.* A comparative encyclopedia of DNA elements in the mouse genome. *Nature* **515,** 355-364 (2014).
2. Sawada, S. & Littman, D. R. Identification and characterization of a T-cell-specific enhancer adjacent to the murine CD4 gene. *Mol Cell Biol* **11,** 5506-5515 (1991).
3. Siu, G., Wurster, A. L., Duncan, D. D., Soliman, T. M. & Hedrick, S. M. A transcriptional silencer controls the developmental expression of the CD4 gene. *EMBO J* **13,** 3570-3579 (1994).
4. Wurster, A. L., Siu, G., Leiden, J. M. & Hedrick, S. M. Elf-1 binds to a critical element in a second CD4 enhancer. *Mol Cell Biol* **14,** 6452-6463 (1994).
5. Chong, M. M. W. *et al.* Epigenetic propagation of CD4 expression is established by the Cd4 proximal enhancer in helper T cells. *Genes Dev* **24,** 659-669 (2010).
6. Kakugawa, K. *et al.* Essential Roles of SATB1 in Specifying T Lymphocyte Subsets. *Cell Rep* **19,** 1176-1188 (2017).
7. Zou, Y. R. *et al.* Epigenetic silencing of CD4 in T cells committed to the cytotoxic lineage. *Nat Genet* **29,** 332-336 (2001).
8. Leung, R. K. M. *et al.* Deletion of the CD4 silencer element supports a stochastic mechanism of thymocyte lineage commitment. *Nature Immunology* **2,** 1167 (2001).
9. Taniuchi, I., Sunshine, M. J., Festenstein, R. & Littman, D. R. Evidence for distinct CD4 silencer functions at different stages of thymocyte differentiation. *Mol Cell* **10,** 1083-1096 (2002).
10. Hostert, A. *et al.* A region in the CD8 gene locus that directs expression to the mature CD8 T cell subset in transgenic mice. *Immunity* **7,** 525-536 (1997).

11. Hostert, A. *et al.* A CD8 genomic fragment that directs subset-specific expression of CD8 in transgenic mice. *J Immunol* **158,** 4270-4281 (1997).

12. Ellmeier, W., Sunshine, M. J., Losos, K., Hatam, F. & Littman, D. R. An enhancer that directs lineage-specific expression of CD8 in positively selected thymocytes and mature T cells. *Immunity* **7,** 537-547 (1997).

13. Ellmeier, W., Sunshine, M. J., Maschek, R. & Littman, D. R. Combined deletion of CD8 locus cis-regulatory elements affects initiation but not maintenance of CD8 expression. *Immunity* **16,** 623-634 (2002).

14. Garefalaki, A. *et al.* Variegated expression of CD8 alpha resulting from in situ deletion of regulatory sequences. *Immunity* **16,** 635-647 (2002).

15. Harker, N. *et al.* The CD8alpha gene locus is regulated by the Ikaros family of proteins. *Mol Cell* **10,** 1403-1415 (2002).

16. Sato, T. *et al.* Dual functions of Runx proteins for reactivating CD8 and silencing CD4 at the commitment process into CD8 thymocytes. *Immunity* **22,** 317-328 (2005).

17. Bilic, I. *et al.* Negative regulation of CD8 expression via Cd8 enhancer-mediated recruitment of the zinc finger protein MAZR. *Nat Immunol* **7,** 392-400 (2006).

18. Bouvier, G. *et al.* Deletion of the mouse T-cell receptor beta gene enhancer blocks alphabeta T-cell development. *Proc Natl Acad Sci U S A* **93,** 7877-7881 (1996).

19. Bories, J. C., Demengeot, J., Davidson, L. & Alt, F. W. Gene-targeted deletion and replacement mutations of the T-cell receptor beta-chain enhancer: the role of enhancer elements in controlling V(D)J recombination accessibility. *Proc Natl Acad Sci U S A* **93,** 7871-7876 (1996).

Supplementary Table 1:- ATAC-seq reads

Technical replicates	cell number used	Gender	Age	Mouse ID	The number of ATAC-seq pruned reads			
					DN	DP	SP4	SP8
exp1	50,000	Male	8w 5d old	Mouse300	34,746,770	133,561,133	46,752,311	34,153,821
exp2	100,000	Male	9w 1d old	Mouse302	31,185,833	37,269,073	30,944,280	31,185,833
exp1	50,000	Female	9w 2d old	Mouse4144	54,244,850	26,266,581	27,351,306	36,033,798
exp2	100,000	Female	9w 1d old	Mouse4220	53,352,406	30,633,272	24,178,547	35,557,147

Supplementary Table 2: GAT Chip-seq

motif	sample	annotation	segments	track	observed	expected	CI95low	CI95high	stddev	fold	l2fold	pvalue	qvalue	track_nsegm	track_size	track_density	annotation_r	annotation_s	annotation_c	overlap_nseg	overlap_size	overlap_dens	percent_over	percent_over	percent_over	percent_over
GATA_known16	DN	centipede_predicted_bound	gata_chip-seq_peaks	GSM523221_DN-GATA3	11851	338.5490	206.0000	483.0000	85.0196	34.9051	5.1254	1.0000e-03	1.00E-03	932	539099	4.94E+00	6786	142995	1.31E+00	569	11851	1.09E-01	61.0515	2.1983	8.3849	8.2877
GATA_known16	DP	centipede_predicted_bound	gata_chip-seq_peaks	GSM523222_DP-GATA3	6425	107.3960	42.0000	189.0000	48.2010	59.2826	5.8895	1.0000e-03	1.00E-03	474	281259	2.58E+00	4229	89087	8.16E-01	308	6425	5.88E-02	64.9789	2.2844	7.283	7.2121
CTCF_known2	DP	centipede_predicted_bound	ctcf_chip-seq_peaks	ENCFF714WDP_CTCF	79	3.48	0	13	4.9244	17.8571	4.1584	1.00E-03	1.00E-03	10535	3832475	3.51E+01	30	166	1.52E-03	17	79	7.23E-04	0.1614	0.0021	56.6667	47.5904

percent_overlap_size_annotation

Supplementary Table 3: GAT footprints

sample	motif	annotation	cluster	observed	expected	CI95low	CI95high	stddev	fold	l2fold	pvalue	qvalue	track_nsegments	track_size	track_density	annotation_nsegments	annotation_size	annotation_density	overlap_nsegments	overlap_size	overlap_density	percent_overlap_nsegments_track	percent_overlap_size_track	percent_overlap_nsegments_annotation	percent_overlap_size_annotation
DP	POU2F2_known14	cluster_2	DP	1361	712.298	624	803	58.4265	1.9094	0.9331	0.001	0.0012632	685	10526	46.78	114	1601	7.1152	94	1361	6.0486	13.7226	12.9299	82.4561	85.0094
DP	CUX1_8	cluster_2	DP	729	391.412	322	465	44.8352	1.8603	0.8955	0.001	0.0011429	409	6571	49.473	61	863	6.4975	51	729	5.4886	12.4694	11.0942	83.6066	84.4728
SP4	TCF3_2	cluster_4	SP4	30745	16550.183	16073	16985	290.6251	1.8576	0.8935	0.001	0.0010435	23468	354325	46.199	2555	36581	4.7697	2113	30745	4.0087	9.0037	8.6771	82.7006	84.0464
DP	RUNX1_4	cluster_2	DP	3682	1987.891	1883	2123	74.0636	1.8518	0.8889	0.001	0.0010909	2351	23810	47.054	442	4280	8.4583	375	3682	7.2765	15.9507	15.4641	84.8416	86.028
SP8	TCF3_2	cluster_1	SP8	26106	14160.259	13709	14557	247.5237	1.8436	0.8825	0.001	0.0010435	23089	348566	45.448	2188	31655	4.1274	1776	26106	3.4039	7.692	7.4895	81.17	82.4704
SP8	ID4_1	cluster_1	SP8	21230	11584.589	11312	11892	180.8516	1.8325	0.8738	0.001	0.0010435	29653	268120	45.899	2880	25444	4.3557	2381	21230	3.6343	8.0295	7.9181	82.6736	83.4381
SP4	TCF4_1	cluster_4	SP4	22445	12261.171	11948	12574	187.8852	1.8305	0.8722	0.001	0.0010909	33174	299877	45.732	3083	27113	4.1348	2531	22445	3.4229	7.6295	7.4847	82.0954	82.7832
DP	TCF7_1	cluster_2	DP	27184	15780.248	15352	16234	261.8387	1.7226	0.7846	0.001	0.0010435	9326	149704	48.954	2102	32593	10.658	1726	27184	8.8892	18.5074	18.1585	82.1123	83.4044
DP	HESX1_2	cluster_2	DP	1193	652.43	566	740	50.001	1.8273	0.8697	0.001	0.0014118	733	10262	51.563	105	1325	6.6576	94	1193	5.9944	12.824	11.6254	89.5238	90.0377
SP8	TCF21_1	cluster_1	SP8	13235	7295.51	7029	7590	174.4038	1.814	0.8592	0.001	0.001	10166	132387	47.014	1237	15681	5.5687	1032	13235	4.7001	10.1515	9.9972	83.4276	84.4015
DP	SOX17_5	cluster_2	DP	4155	2299.102	2130	2454	96.9825	1.8069	0.8535	0.001	0.0011429	1842	25857	46.902	381	5012	9.0912	306	4155	7.5367	16.6124	16.0691	80.315	82.901
SP4	ID4_1	cluster_4	SP4	23702	13148.075	12827	13465	186.6787	1.8026	0.8501	0.001	0.0010435	30226	273311	46.788	3214	28330	4.8498	2665	23702	4.0575	8.8169	8.6722	82.9185	83.664
SP4	TCF21_1	cluster_4	SP4	15466	8586.04	8291	8857	174.8941	1.8012	0.849	0.001	0.001	10517	136988	48.648	1441	17927	6.3663	1219	15466	5.4924	11.5908	11.29	84.594	86.2721
DP	SOX15_4	cluster_2	DP	3936	2192.197	2022	2350	96.6444	1.7951	0.8441	0.001	0.0011429	1805	25291	45.839	374	4898	8.8775	292	3936	7.1339	16.1773	15.5628	78.0749	80.3593
DP	GATA_known16	cluster_2	DP	12722	7132.596	6782	7558	221.1013	1.7835	0.8347	0.001	0.0010435	4004	84333	48.865	772	15025	8.7059	635	12722	7.3715	15.8591	15.0854	82.2539	84.6722
DP	SOX3_3	cluster_2	DP	6015	3374.904	3184	3576	120.8475	1.782	0.8335	0.001	0.0010909	2264	36279	46.72	486	7333	9.4434	390	6015	7.7461	17.2261	16.5798	80.2469	82.0265
DP	SOX1_3	cluster_2	DP	5798	3256.924	3060	3460	121.7581	1.78	0.8319	0.001	0.0010909	2535	35523	47.207	533	7047	9.3648	424	5798	7.705	16.7258	16.3218	79.5497	82.2761
DP	SOX13_1	cluster_2	DP	3688	2072.051	1917	2230	95.8978	1.7795	0.8315	0.001	0.0010909	1690	25424	51.067	309	4228	8.4923	264	3688	7.4077	15.6213	14.506	85.4369	87.228
DP	POU2F2_known16	cluster_2	DP	4777	2687.552	2516	2853	105.7564	1.7772	0.8296	0.001	0.0010909	2099	27573	46.898	479	5889	10.016	381	4777	8.1251	18.1515	17.3249	79.5407	81.1173
DP	SOX18_4	cluster_2	DP	4208	2368.15	2210	2543	102.195	1.7766	0.8291	0.001	0.0012	1791	25128	47.709	385	5045	9.5787	312	4208	7.9895	17.4204	16.7463	81.039	83.4093
DP	ARID3A_2	cluster_2	DP	1961	1104.156	995	1213	63.6366	1.7753	0.8281	0.001	0.0013333	987	15963	50.765	155	2265	7.2031	131	1961	6.2363	13.2725	12.2847	84.5161	86.5784
DP	PBX1_2	cluster_2	DP	4833	2725.042	2566	2874	98.6319	1.7733	0.8264	0.001	0.0010435	2395	33982	48.403	430	5750	8.1901	356	4833	6.8839	14.8643	14.2222	82.7907	84.0522
DP	PBX1_4	cluster_2	DP	3664	2073.483	1936	2219	83.3417	1.7667	0.8211	0.001	0.0010909	2319	25948	48.177	409	4372	8.1173	340	3664	6.8028	14.6615	14.1205	83.1296	83.806
SP8	PKNOX1_2	cluster_1	SP8	14489	8213.49	7943	8482	154.2804	1.764	0.8188	0.001	0.0010435	9433	104620	49.524	1569	16823	7.9634	1333	14489	6.8586	14.1312	13.8492	84.9586	86.1261
SP4	LMO2_1	cluster_4	SP4	30837	17482.683	17082	17852	227.5727	1.7638	0.8187	0.001	0.001	35624	395160	47.238	3507	37544	4.4881	2844	30837	3.6863	7.9834	7.8037	81.095	82.1356
DP	SOX8_9	cluster_2	DP	5605	3187.09	2996	3383	121.335	1.7584	0.8143	0.001	0.0010909	2184	34944	45.366	485	7176	9.3163	369	5605	7.2768	16.8956	16.0399	76.0825	78.1076
SP8	PKNOX2_2	cluster_1	SP8	15930	9061.401	8762	9331	174.4952	1.7579	0.8139	0.001	0.0010435	10799	119886	49.272	1726	18579	7.6357	1460	15930	6.547	13.5198	13.2876	84.5886	85.742
SP4	TCF4_2	cluster_4	SP4	24110	13800.406	13426	14110	207.0676	1.747	0.8049	0.001	0.001	28828	260376	48.438	3276	28814	5.3602	2720	24110	4.4852	9.4353	9.2597	83.0281	83.6746
SP8	TCF4_2	cluster_1	SP8	22012	12613.17	12286	12898	185.0352	1.7451	0.8033	0.001	0.001	28489	257334	47.872	3017	26562	4.9413	2471	22012	4.0949	8.6735	8.5539	81.9026	82.8703
DP	SOX2_6	cluster_2	DP	4160	2387.764	2218	2574	107.8018	1.7419	0.8007	0.001	0.0010435	1589	25439	47.17	347	5211	9.6623	271	4160	7.7136	17.0548	16.3528	78.098	79.8311
DP	SOX17_4	cluster_2	DP	5114	2939.69	2764	3133	109.6463	1.7394	0.7986	0.001	0.0010909	2192	30688	48.463	464	6163	9.7328	379	5114	8.0762	17.2901	16.6645	81.681	82.9791
DP	IRF_known21	cluster_2	DP	9726	5591.459	5349	5843	154.5961	1.7393	0.7985	0.001	0.001	6273	90082	51.133	824	11147	6.3273	709	9726	5.5207	11.3024	10.7968	86.0437	87.2522
DP	TCF7L1_1	cluster_2	DP	25273	14545.435	14124	14987	252.6679	1.7375	0.797	0.001	0.0010435	8494	136288	48.648	1956	30243	10.795	1604	25273	9.0212	18.8839	18.5438	82.0041	83.5664
DP	SOX12_1	cluster_2	DP	5335	3074.314	2893	3249	110.0846	1.7351	0.795	0.001	0.0010435	2452	31970	50.662	501	6174	9.7837	427	5335	8.4542	17.4144	16.6875	85.2295	86.4108
SP4	MESP1_1	cluster_4	SP4	20625	11890.5	11577	12180	184.0304	1.7345	0.7945	0.001	0.001	28293	255419	48.619	2809	24683	4.6984	2327	20625	3.9259	8.2246	8.075	82.8409	83.5595
DP	RUNX1_2	cluster_2	DP	8353	4816.204	4590	5070	153.8404	1.7342	0.7943	0.001	0.0010435	3573	55648	49.329	694	10049	8.9078	558	8353	7.4044	15.6171	15.0104	80.4035	83.1227
SP8	PKNOX2_3	cluster_1	SP8	15112	8716.99	8441	8979	170.9946	1.7335	0.7937	0.001	0.0010435	9661	107482	50.165	1632	17576	8.2032	1385	15112	7.0532	14.336	14.06	84.8652	85.9809
DP	SOX14_3	cluster_2	DP	6354	3666.783	3452	3844	116.8767	1.7327	0.793	0.001	0.0010909	2759	38663	47.284	592	7830	9.5759	466	6354	7.7708	16.8902	16.4343	78.7162	81.1494
DP	SOX7_4	cluster_2	DP	5740	3313.523	3097	3535	130.1173	1.7321	0.7925	0.001	0.0010909	2231	35768	47.798	471	7079	9.46	375	5740	7.7606	16.8086	16.0479	79.6178	81.0849
DP	FOXO3_4	cluster_2	DP	3425	1977.58	1859	2121	80.0602	1.7315	0.7921	0.001	0.0010909	1629	21240	50.833	319	3951	9.4558	272	3425	8.1969	16.6974	16.1252	85.2665	86.6869
SP8	MESP1_1	cluster_1	SP8	20386	11775.49	11477	12072	182.9194	1.7312	0.7917	0.001	0.001	28229	254858	48.512	2782	24456	4.6552	2290	20386	3.8804	8.1122	7.999	82.3149	83.3579
DP	GATA_known17	cluster_2	DP	6858	3965.514	3715	4224	152.5548	1.7292	0.7901	0.001	0.0010909	3215	51515	48.405	554	8351	7.8469	443	6858	6.444	13.7792	13.3126	79.9639	82.1219
DP	SOX7_3	cluster_2	DP	7296	4219.146	3990	4422	134.6917	1.7291	0.79	0.001	0.0010909	2800	44824	49.319	583	8749	9.6263	472	7296	8.0276	16.8571	16.277	80.9605	83.3924
DP	FOXO4_3	cluster_2	DP	3851	2227.845	2069	2391	94.3172	1.7282	0.7893	0.001	0.0010909	1828	23799	50.665	359	4443	9.4586	304	3851	8.1983	16.6302	16.1814	84.6797	86.6757
DP	RUNX_1	cluster_2	DP	39938	23118.881	22582	23669	317.7723	1.7275	0.7887	0.001	0.0010435	21137	297645	46.5	3658	49981	7.8084	2878	39938	6.2394	13.6159	13.418	78.6769	79.9064
DP	SOX18_2	cluster_2	DP	5349	3102.283	2929	3276	103.7075	1.724	0.7857	0.001	0.0010909	2288	32032	48.779	483	6451	9.8237	393	5349	8.1455	17.1766	16.6989	81.3665	82.9174
DP	TCF4_1	cluster_2	DP	90679	60438.383	59779	60996	363.6716	1.5003	0.5853	0.001	0.0010909	39369	356288	54.334	12410	111339	16.979	10035	90679	13.829	25.4896	25.451	80.8622	81.4441
DP	SOX10_9	cluster_2	DP	6316	3675.857	3487	3879	123.2519	1.718	0.7808	0.001	0.0010435	2814	39418	46.89	597	7939	9.4439	468	6316	7.5132	16.6311	16.0231	78.392	79.5566
SP8	LMO2_1	cluster_1	SP8	30642	17843.237	17400	18261	255.1138	1.7172	0.7801	0.001	0.001	35542	394212	47.125	3559	38297	4.5781	2803	30642	3.663	7.8864	7.773	78.7581	80.0115
DP	SOX3_1	cluster_2	DP	4652	2710.758	2528	2888	107.6454	1.7159	0.7789	0.001	0.0010909	1698	27182	49.21	376	5627	10.187	303	4652	8.4219	17.8445	17.1143	80.5851	82.6728
DP	TCF7L2_known7	cluster_2	DP	25714	15002.683	14600	15399	230.752	1.7139	0.7773	0.001	0.0010435	10010	140599	49.374	2272	30780	10.809	1870	25714	9.0299	18.6813	18.2889	82.3063	83.5413
DP	RUNX_2	cluster_2	DP	32783	19140.326	18679	19646	296.6987	1.7127	0.7763	0.001	0.0010435	17458	245690	47.274	2986	40745	7.8399	2367	32783	6.3079	13.5583	13.3432	79.2699	80.459
DP	POU2F2_known15	cluster_2	DP	4809	2810.025	2661	2961	93.3964	1.7111	0.7749	0.001	0.0010435	2667	29451	51.273	523	5548	9.6588	447	4809	8.3722	16.7604	16.3288	85.4685	86.6799
DP	SOX5_3	cluster_2	DP	4433	2590.315	2393	2766	109.5854	1.7111	0.7749	0.001	0.0011429	2038	30641	52.026	366	5129	8.7086	310	4433	7.5268	15.211	14.4675	84.6995	86.4301
DP	SOX11_2	cluster_2	DP	6583	3858.07	3653	4087	123.2048	1.7061	0.7707	0.001	0.0010909	3067	42939	50.606	589	7774	9.1621	487	6583	7.7584	15.8787	15.3311	82.6825	84.6797
SP4	RUNX_1	cluster_4	SP4	40385	23682.57	23160	24196	311.4276	1.7052	0.77	0.001	0.0010435	22871	322027	50.309	3468	47492	7.4195	2918	40385	6.3092	12.7585	12.5409	84.1407	85.0354
DP	SOX1_2	cluster_2	DP	6871	4031.924	3812	4205	121.2481	1.704	0.7689	0.001	0.0010909	2774	38842	48.471	621	8368	10.443	501	6871	8.5744	18.0606	17.6896	80.6763	82.1104
DP	POU2F2_known1	cluster_2	DP	7924	4670.891	4424	4927	153.3097	1.6963	0.7624	0.001	0.0010435	2722	49255	50.993	558	9322	9.651	462	7924	8.2037	16.9728	16.0877	82.7957	85.0032
DP	POU2F2_known13	cluster_2	DP	7050	4157.719	3941	4406	144.4392	1.6955	0.7617	0.001	0.0010909	2228	46947	52.881	436	8267	9.312	361	7050	7.9412	16.2029	15.0169	82.7982	85.2788
DP	POU2F3_2	cluster_2	DP	3748	2210.562	2107	2312	63.9579	1.6952	0.7614	0.001	0.0010909	2892	23182	50.653	567	4389	9.5901	480	3748	8.1895	16.5975	16.1677	84.6561	85.3953
SP4	PKNOX1_2	cluster_4	SP4	19580	11550.501	11219	11890	209.4397	1.6951	0.7614	0.001	0.0010435	9911	109901	52.023	2075	22398	10.602	1792	19580	9.2685	18.0809	17.816	86.3614	87.4185
DP	CBX5_1	cluster_2	DP	5611	3312.321	3144	3465	106.0661	1.6938	0.7602	0.001	0.0010435	3561	39214	51.585	629	6602	8.6848	530	5611	7.3811	14.8835	14.3087	84.2607	84.9894
DP	SOX15_2	cluster_2	DP	5647	3336.109	3159	3519	111.9578	1.6925	0.7591	0.001	0.0010909	2513	35262	49.216	510	6849	9.5594	416	5647	7.8817	16.5539	16.0144	81.5686	82.45
DP	FOXP1_1	cluster_2	DP	27739	16391.541	15873	16892	321.6271	1.6922	0.7589	0.001	0.0010435	8153	191364	49.307	1880	37085	9.5554	1362	27739	7.1473	16.7055	14.4954	72.4468	74.7984
DP	FOXO1_4	cluster_2	DP	4199	2481.437	2324	2635	94.7669	1.6919	0.7586	0.001	0.0010909	1950	25417	51.042	405	4964	9.9687	336	4199	8.4324	17.2308	16.5204	82.963	84.589
DP	POU2F2_known10	cluster_2	DP	6350	3755.002	3609	3908	90.0414	1.6909	0.7578	0.001	0.0010435	4216	38032	52.25	838	7279	10	725	6350	8.724	17.1964	16.6965	86.5155	87.2373
DP	TCF7_2	cluster_2	DP	21182	12535.662	12199	12836	205.2192	1.6897	0.7568	0.001	0.0010435	10707	118115	49.917	2353	25359	10.717	1948	21182	8.9517	18.1937	17.9334	82.7879	83.5285
DP	IRF4_2	cluster_2	DP	8414	4979.878	4780	5204	130.8322	1.6895	0.7566	0.001	0.001	5446	77752	53.165	723	9646	6.5957	620	8414	5.7533	11.3845	10.8216	85.7538	87.2279
DP	HBP1_1	cluster_2	DP	8400	4972.537	4750	5182	131.6143	1.6891	0.7563	0.001	0.0010435	3549	54705	50.814	703	10052	9.3371	571	8400	7.8026	16.089	15.3551	81.2233	83.5655
DP	SOX9_6	cluster_2	DP	8853	5241.013	5007	5479	137.8147	1.689	0.7562	0.001	0.0010909	3625	54432	49.299	759	10845	9.8222	614	8853	8.0181	16.9379	16.2643	80.8959	81.6321
DP	POU2F3_1	cluster_2	DP	7491	4436.596	4211	4664	130.4895	1.6883	0.7556	0.001	0.0010909	3087	46662	50.134	635	9011	9.6815	519	7491	8.0484	16.8124	16.0537	81.7323	83.1317
SP4	PKNOX2_2	cluster_4	SP4	21674	12837.192	12508	13164	192.7536	1.6883	0.7556	0.001	0.0010435	11364	126178	51.858	2309	24959	10.258	1980	21674	8.9078	17.4234	17.1773	85.7514	86.8384
SP8	TCF4_1	cluster_6	Common_2	113748	67411.509	66817	68096	386.6488	1.6874	0.7548	0.001	0.0010909	32686	295493	45.063	16835	150077	22.887	12658	113748	17.347	38.7261	38.4943	75.1886	75.7931
SP4	RUNX_2	cluster_4	SP4	31788	18842.688	18325	19279	270.1852	1.687	0.7544	0.001	0.0010435	18786	264509	50.895	2748	37394	7.1951	2309	31788	6.1165	12.2911	12.0177	84.0247	85.0083
DP	SOX5_1	cluster_2	DP	2756	1637.334	1543	1742	63.9816	1.6828	0.7509	0.001	0.0010909	1887	16983	50.471	373	3266	9.7061	312	2756	8.1904	16.5342	16.228	83.6461	84.3846
DP	POU2F2_known11	cluster_2	DP	6209	3689.778	3530	3854	95.2532	1.6826	0.7507	0.001	0.0010909	3816	38242	51.233	762	7325	9.8134	638	6209	8.3183	16.7191	16.2361	83.727	84.7645
DP	POU2F2_known12	cluster_2	DP	5688	3381.331	3242	3528	88.1983	1.682	0.7502	0.001	0.0010435	3757	37632	51.558	697	6675	9.1451	587	5688	7.7928	15.6242	15.1148	84.2181	85.2135
DP	FOXO6_1	cluster_2	DP	5232	3113.128	2951	3284	97.5286	1.6804	0.7488	0.001	0.0010435	2247	29326	51.149	496	6186	10.789	410	5232	9.1255	18.2466	17.8408	82.6613	84.5781
DP	SOX14_2	cluster_2	DP	5317	3164.006	3006	3336	102.2211	1.6802	0.7487	0.001	0.0010909	2895	31972	49.947	603	6367	9.9466	496	5317	8.3063	17.133	16.6302	82.2554	83.5087
SP8	RUNX_1	cluster_1	SP8	57342	34207.231	33609	34716	337.0007	1.6763	0.7453	0.001	0.0010435	23767	334786	52.302	4759	65825	10.284	4113	57342	8.9583	17.3055	17.128	86.4257	87.1128
SP8	TCF3_2	cluster_6	Common_2	128703	76822.184	76013	77692	499.2123	1.6753	0.7444	0.001	0.0010435	23089	348566	45.448	11592	170739	22.262	8622	128703	16.781	37.3425	36.9236	74.3789	75.38
DP	SOX30_1	cluster_2	DP	7587	4530.861	4319	4742	132.4068	1.6744	0.7436	0.001	0.0010435	3080	46247	52.079	626	8864	9.9818	527	7587	8.5437	17.1104	16.4054	84.1853	85.5934
DP	TCF7L2_known5	cluster_2	DP	28645	17154.443	16704	17602	277.3772	1.6698	0.7397	0.001	0.0010435	9748	156401	49.931	2240	34675	11.07	1819	28645	9.1449	18.6602	18.3151	81.2054	82.6099
DP	POU2F2_known5	cluster_2	DP	6390	3826.563	3653	4008	108.4739	1.6697	0.7396	0.001	0.0010909	3181	41440	50.684	612	7622	9.3222	503	6390	7.8154	15.8126	15.4199	82.1895	83.8363
DP	TBX19_1	cluster_2	DP	11519	6899.341	6579	7221	189.1995	1.6695	0.7394	0.001	0.0010909	3308	63063	49.328	770	14099	11.028	616	11519	9.0101	18.6215	18.2659	80	81.7008
SP4	PKNOX2_3	cluster_4	SP4	20888	12522.02	12198	12835	188.1507	1.668	0.7382	0.001	0.0010435	10156	113004	52.742	2218	23981	11.193	1910	20888	9.749	18.8066	18.4843	86.1136	87.1023
DP	SOX21_2	cluster_2	DP	7253	4349	4130	4536	123.6117	1.6676	0.7378	0.001	0.0010909	3145	44041	49.589	668	8902	10.023	534	7253	8.1667	16.9793	16.4687	79.9401	81.4761
DP	SOX4_2	cluster_2	DP	9366	5617.712	5358	5858	159.0695	1.6671	0.7373	0.001	0.0010909	3926	59025	51.393	791	11178	9.7326	646	9366	8.1549	16.4544	15.8679	81.6688	83.7896
SP8	RBPJ_1	cluster_1	SP8	9708	5824.112	5671	5995	99.4728	1.6667	0.737	0.001	0.0010435	10911	76393	53.125	1618	11061	7.692	1412	9708	6.7511	12.9411	12.708	87.2682	87.7678
SP4	TCF4_1	cluster_6	Common_2	113678	68325.361	67699	68969	392.9496	1.6638	0.7345	0.001	0.0010909	33174	299877	45.732	16835	150077	22.887	12664	113678	17.336	38.1745	37.9082	75.2242	75.7465
DP	HESX1_1	cluster_2	DP	1150	692.528	623	763	42.1094	1.6596	0.7309	0.001	0.0012632	1124	10127	51.054	158	1360	6.8562	131	1150	5.7975	11.6548	11.3558	82.9114	84.5588
SP8	ETV7_1	cluster_1	SP8	29780	17945.789	17578	18268	209.5885	1.6594	0.7307	0.001	0.001	25471	229674	51.731	3931	34860	7.8518	3332	29780	6.7076	13.0815	12.9662	84.7621	85.4274
SP4	TCF3_2	cluster_6	Common_2	129388	78043.92	77199	78910	527.8227	1.6579	0.7293	0.001	0.0010435	23468	354325	46.199	11592	170739	22.262	8666	129388	16.87	36.9269	36.5168	74.7585	75.7812
SP8	SPIB_1	cluster_1	SP8	14192	8579.79	8404	8768	112.5582	1.654	0.726	0.001	0.0010435	16443	98658	52.897	2742	16291	8.7346	2387	14192	7.6092	14.5168	14.385	87.0532	87.1156
SP8	ID4_1	cluster_6	Common_2	103849	62786.932	62151	63445	391.3925	1.654	0.7259	0.001	0.0010435	29653	268120	45.899	15416	137254	23.496	11565	103849	17.778	39.0011	38.7323	75.0195	75.6619
DP	BPTF_1	cluster_2	DP	22248	13459.004	13088	13806	213.9869	1.653	0.7251	0.001	0.0010909	10963	146228	52.127	2078	26531	9.4577	1719	22248	7.9309	15.68	15.2146	82.7238	83.8566
DP	SOX9_4	cluster_2	DP	10899	6599.314	6288	6876	175.3838	1.6514	0.7237	0.001	0.0010435	4428	66520	51.448	910	13004	10.058	752	10899	8.4295	16.9828	16.3845	82.6374	83.8127
SP8	RUNX1_4	cluster_1	SP8	4441	2692.688	2543	2842	87.548	1.649	0.7216	0.001	0.0010909	2766	28019	55.372	512	4957	9.7962	457	4441	8.7765	16.5221	15.85	89.2578	89.5905
SP8	TBX5_3	cluster_1	SP8	18351	11134.932	10860	11394	157.3921	1.648	0.7207	0.001	0.001	14898	134209	54.179	2358	20762	8.3814	2076	18351	7.4081	13.9348	13.6734	88.0407	88.3874
DP	SOX10_4	cluster_2	DP	11588	7045.726	6778	7320	162.8889	1.6446	0.7177	0.001	0.0010909	4770	71762	51.378	973	13974	10.005	794	11588	8.2965	16.6457	16.1478	81.6033	82.9254
DP	HMGA1_2	cluster_2	DP	8629	5250.682	5056	5489	136.6983	1.6433	0.7166	0.001	0.0010435	4747	66572	54.16	772	10035	8.164	650	8629	7.0201	13.6929	12.9619	84.1969	85.989
DP	TCF7L1_2	cluster_2	DP	20275	12343.586	12041	12689	194.2647	1.6425	0.7159	0.001	0.0010435	10535	116448	51.095	2272	24364	10.69	1872	20275	8.8963	17.7693	17.4112	82.3944	83.217
SP8	MAFG_1	cluster_1	SP8	15807	9624.678	9331	9927	195.5397	1.6423	0.7157	0.001	0.0010435	6025	120737	52.864	971	18472	8.0878	810	15807	6.921	13.444	13.0921	83.4192	85.5728
SP8	RUNX_2	cluster_1	SP8	46883	28556.288	27990	29038	306.5118	1.6418	0.7152	0.001	0.0010435	19635	276608	53.223	3931	54123	10.414	3367	46883	9.021	17.148	16.9493	85.6525	86.6231
DP	SOX_1	cluster_2	DP	11190	6822.598	6572	7059	141.7602	1.64	0.7137	0.001	0.0010435	5830	70005	50.837	1171	13573	9.8566	951	11190	8.1261	16.3122	15.9846	81.2126	82.4431
DP	IKZF2_3	cluster_2	DP	6073	3703.184	3558	3851	90.5549	1.6398	0.7135	0.001	0.0010909	4868	48814	55.261	728	6899	7.8102	636	6073	6.8751	13.0649	12.4411	87.3626	88.0273
SP8	NFE2L1::MAFG_1	cluster_1	SP8	25102	15308.482	14780	15825	298.9174	1.6397	0.7134	0.001	0.0010435	8430	177761	54.694	1453	28629	8.8086	1244	25102	7.7234	14.7568	14.1212	85.616	87.6803
DP	SOX2_5	cluster_2	DP	6808	4154.871	3927	4385	138.6546	1.6384	0.7123	0.001	0.0010909	2721	43557	49.369	561	8492	9.6251	441	6808	7.7164	16.2073	15.6301	78.6096	80.1696
DP	SOX8_2	cluster_2	DP	10393	6347.037	6086	6643	169.5038	1.6374	0.7114	0.001	0.0010909	4342	65204	50.995	886	12634	9.8809	720	10393	8.1282	16.5822	15.9392	81.2641	82.2621
DP	CUX1_9	cluster_2	DP	838	511.691	427	588	47.438	1.6365	0.7106	0.001	0.0012632	440	7588	51.249	63	1004	6.781	51	838	5.6599	11.5909	11.0438	80.9524	83.4661
DP	NFATC1_3	cluster_2	DP	4736	2895.808	2734	3047	98.6233	1.6352	0.7095	0.001	0.0010909	2771	36045	53.14	457	5575	8.2191	382	4736	6.9822	13.7856	13.1391	83.5886	84.9507
SP8	MAFF_1	cluster_1	SP8	17034	10418.667	10118	10723	188.7017	1.6349	0.7092	0.001	0.001	9420	132144	52.834	1463	19885	7.9504	1235	17034	6.8105	13.1104	12.8905	84.4156	85.6626
SP8	PKNOX1_1	cluster_1	SP8	20853	12754.59	12402	13105	221.4364	1.6349	0.7092	0.001	0.0010435	10991	165550	53.351	1682	24195	7.7972	1421	20853	6.7202	12.9288	12.5962	84.4828	86.1872
SP8	POU2F2_known16	cluster_1	SP8	5723	3503.508	3335	3679	99.4676	1.6333	0.7078	0.001	0.0010909	2431	32048	54.51	529	6610	11.243	454	5723	9.7342	18.6754	17.8576	85.8223	86.5809
DP	FOXO3_1	cluster_2	DP	10512	6438.431	6231	6675	138.3726	1.6326	0.7072	0.001	0.0010435	4685	61564	56.333	965	11860	10.852	843	10512	9.6188	17.9936	17.0749	87.3575	88.6341
DP	SPDEF_4	cluster_2	DP	15023	9201.766	8906	9544	192.5805	1.6326	0.7071	0.001	0.0010435	6274	94414	52.37	1256	17937	9.9495	1037	15023	8.3331	16.5285	15.9118	82.5637	83.7543
SP4	ID4_1	cluster_6	Common_2	104286	63878.205	63184	64511	379.8538	1.6326	0.7071	0.001	0.0010435	30226	273311	46.788	15416	137254	23.496	11625	104286	17.853	38.4603	38.1565	75.4087	75.9803
SP8	LMO2_1	cluster_6	Common_2	160835	98519.709	97672	99403	525.5193	1.6325	0.7071	0.001	0.001	35542	394212	47.125	19245	210148	25.122	14597	160835	19.227	41.0697	40.7991	75.8483	76.5342
DP	TBX1_2	cluster_2	DP	13463	8256.658	7928	8585	202.5755	1.6305	0.7053	0.001	0.0010909	4390	79089	50.708	979	16664	10.684	773	13463	8.6319	17.6082	17.0226	78.9581	80.7909
DP	IRF_known14	cluster_2	DP	28839	17694.324	17235	18177	279.0563	1.6298	0.7047	0.001	0.001	11310	235509	53.453	1777	34195	7.7612	1466	28839	6.5456	12.962	12.2454	82.4986	84.3369
SP8	SOX1_3	cluster_1	SP8	7509	4614.703	4412	4818	120.7026	1.6271	0.7023	0.001	0.0010909	2972	41649	55.348	648	8538	11.346	562	7509	9.9787	18.9098	18.0292	86.7284	87.948
SP8	SPI1_known1	cluster_1	SP8	32365	19916.957	19618	20174	181.8553	1.625	0.7004	0.001	0.001	35122	247084	52.91	5460	37816	8.0978	4643	32365	6.9306	13.2196	13.0988	85.0366	85.5855
SP8	SOX3_3	cluster_1	SP8	7590	4674.304	4467	4860	126.9604	1.6236	0.6992	0.001	0.0010909	2699	43266	55.718	591	8743	11.259	500	7590	9.7744	18.5254	17.5426	84.6024	86.8123

SP8	SOX14_3	cluster_1	SP8	8131	5008.076	4806	5218	125.9374	1.6235	0.6991	0.001	0.0010909	3228	45250	55.339	698	9269	11.336	606	8131	9.944	18.7732	17.9691	86.8195	87.7225
DP	ARID5A_1	cluster_2	DP	1014	624.794	553	696	44.5599	1.6219	0.6977	0.001	0.0012632	647	8899	52.495	100	1257	7.4151	80	1014	5.9816	12.3648	11.3945	80	80.6683
DP	PKNOX2_3	cluster_2	DP	22094	13633.374	13300	13961	197.2065	1.6205	0.6965	0.001	0.0010435	9600	106895	49.891	2518	27407	12.792	2002	22094	10.312	20.8542	20.6689	79.5075	80.6144
SP8	RUNX1_2	cluster_1	SP8	11093	6846.538	6588	7123	164.4675	1.6201	0.6961	0.001	0.0010435	4117	64095	56.816	859	12459	11.044	748	11093	9.8333	18.1686	17.3071	87.078	89.036
DP	NFATC2_1	cluster_2	DP	4089	2524.695	2425	2620	58.5734	1.6194	0.6954	0.001	0.0010909	5104	30624	54.868	798	4659	8.3474	696	4089	7.3261	13.6364	13.3523	87.218	87.7656
SP4	LMO2_1	cluster_6	Common_2	159783	98675.417	97887	99532	491.8278	1.6193	0.6953	0.001	0.001	35624	395160	47.238	19245	210148	25.122	14516	159783	19.101	40.7478	40.435	75.4274	76.0336
DP	PKNOX1_2	cluster_2	DP	22715	14034.729	13649	14345	200.9202	1.6184	0.6946	0.001	0.0010435	9569	106189	50.266	2578	28014	13.261	2065	22715	10.753	21.5801	21.3911	80.1009	81.0845
DP	ATF4_3	cluster_2	DP	6986	4318.962	4133	4517	120.8847	1.6174	0.6937	0.001	0.0010909	3413	44407	51.027	685	8615	9.8993	550	6986	8.0275	16.1149	15.7318	80.292	81.0911
SP8	TCF7L2_known5	cluster_1	SP8	31954	19757.294	19274	20190	278.2958	1.6173	0.6936	0.001	0.0010435	10823	173781	55.479	2312	36014	11.497	2036	31954	10.201	18.8118	18.3875	88.0623	88.7266
SP8	SOX1_2	cluster_1	SP8	8134	5031.454	4823	5238	121.8	1.6165	0.6929	0.001	0.0010909	3221	45113	56.297	686	9166	11.438	597	8134	10.15	18.5346	18.0303	87.0262	88.741
DP	SOX4_1	cluster_2	DP	29352	18164.453	17713	18685	279.4644	1.6159	0.6923	0.001	0.0010435	10641	172327	54.726	2224	34044	10.811	1887	29352	9.3214	17.7333	17.0327	84.8471	86.2178
SP8	MESP1_1	cluster_6	Common_2	108065	66890.102	66373	67480	336.8547	1.6156	0.692	0.001	0.001	28229	254858	48.512	15538	138355	26.336	12044	108065	20.57	42.6653	42.402	77.5132	78.107
SP8	SOX15_4	cluster_1	SP8	6070	3759.743	3581	3934	109.1546	1.6143	0.6909	0.001	0.0011429	2193	30727	55.692	529	6964	12.622	456	6070	11.002	20.7934	19.7546	86.2004	87.1626
SP8	GATA_known17	cluster_1	SP8	9237	5723.364	5476	5974	154.5981	1.6138	0.6905	0.001	0.0010909	3680	58991	55.43	690	10533	9.8972	600	9237	8.6794	16.3043	15.6583	86.9565	87.6958
SP8	SOX2_5	cluster_1	SP8	8957	5549.971	5323	5756	129.8334	1.6138	0.6904	0.001	0.0010909	3178	50901	57.693	655	9906	11.228	582	8957	10.152	18.3134	17.5969	88.855	90.4199
DP	SOX11_1	cluster_2	DP	27040	16766.86	16328	17152	266.0451	1.6127	0.6894	0.001	0.0010435	9836	159338	54.566	2057	31531	10.798	1736	27040	9.2599	17.6495	16.9702	84.3947	85.7569
DP	PKNOX2_2	cluster_2	DP	26308	16319.073	15944	16664	223.3982	1.6121	0.6889	0.001	0.0010435	11043	122680	50.42	2996	32542	13.374	2393	26308	10.812	21.6698	21.4444	79.8732	80.8432
SP8	TCF21_1	cluster_6	Common_2	48028	29792.873	29252	30317	314.5381	1.612	0.6889	0.001	0.001	10166	132387	47.014	5010	63749	22.639	3722	48028	17.056	36.6122	36.2785	74.2914	75.3392
SP8	TCF7L2_known7	cluster_1	SP8	29459	18290.672	17894	18703	250.0749	1.6106	0.6876	0.001	0.0010435	11335	159258	55.926	2430	33136	11.636	2147	29459	10.345	18.9413	18.4977	88.3539	88.9033
DP	HMGA1_1	cluster_2	DP	2088	1296.365	1221	1370	43.5862	1.6102	0.6872	0.001	0.0010435	3004	18138	53.492	417	2455	7.2402	353	2088	6.1578	11.751	11.5117	84.6523	85.0509
SP8	TBX1_4	cluster_1	SP8	25075	15573.363	15151	16054	269.909	1.6101	0.6871	0.001	0.0010909	8594	163899	55.977	1543	28290	9.6621	1343	25075	8.564	15.6272	15.2991	87.0382	88.6356
SP8	GATA_known16	cluster_1	SP8	13969	8676.546	8305	9000	217.7867	1.6099	0.687	0.001	0.0010435	4509	95037	55.067	822	16180	9.3751	696	13969	8.094	15.4358	14.6985	84.6715	86.335
DP	FOXP3_1	cluster_2	DP	19384	12040.852	11668	12406	225.2389	1.6098	0.6869	0.001	0.0010909	7025	112814	50.937	1558	23954	10.816	1230	19384	8.7522	17.5089	17.1823	78.9474	80.9218
DP	SOX21_3	cluster_2	DP	6897	4285.452	4078	4500	127.92	1.6093	0.6864	0.001	0.0010909	3002	45066	51.174	612	8556	9.7157	486	6897	7.8318	16.1892	15.3042	79.4118	80.6101
SP8	TCF7_2	cluster_1	SP8	24496	15232.079	14923	15584	199.5628	1.6081	0.6854	0.001	0.0010435	11953	131925	55.753	2583	27660	11.689	2271	24496	10.352	18.9994	18.5681	87.921	88.5611
SP8	TBX4_1	cluster_1	SP8	12291	7645.227	7478	7827	114.7605	1.6076	0.6849	0.001	0.0010435	11799	82621	55.065	2026	13954	9.2999	1778	12291	8.1916	15.0691	14.8764	87.7591	88.0823
SP8	TCF4_2	cluster_6	Common_2	105760	65816.672	65121	66513	384.5768	1.6069	0.6843	0.001	0.001	28489	257334	47.872	15507	138006	25.673	11786	105760	19.674	41.3704	41.0983	76.0044	76.6343
SP8	TCF7L1_1	cluster_1	SP8	30722	19135.746	18669	19553	266.3018	1.6054	0.683	0.001	0.0010435	9778	157054	56.06	2221	34617	12.357	1956	30722	10.966	20.0041	19.5614	88.0684	88.7483
SP8	TCF4_1	cluster_2	DP	45356	50015.9	49390	50593	357.3047	0.9068	-0.1411	0.001	0.0010909	32686	295493	45.063	12410	111339	16.979	5015	45356	6.9168	15.343	15.3493	40.411	40.7368
SP8	SPIB_2	cluster_1	SP8	31661	19766.635	19362	20170	248.0332	1.6017	0.6796	0.001	0.0010435	17242	225293	55.874	2806	35711	8.8566	2463	31661	7.8522	14.2849	14.0533	87.7762	88.659
SP8	TBX19_1	cluster_1	SP8	10613	6626.723	6348	6912	165.0087	1.6015	0.6794	0.001	0.0010435	3619	68942	53.926	692	12512	9.7869	577	10613	8.3015	15.9436	15.3941	83.3815	84.8226
SP8	TBX15_2	cluster_1	SP8	15309	9559.561	9350	9790	126.5889	1.6014	0.6793	0.001	0.0010909	14966	104782	54.532	2561	17651	9.1862	2214	15309	7.9674	14.7935	14.6103	86.4506	86.7316
SP8	SPIC_1	cluster_1	SP8	27344	17082.858	16693	17520	246.5816	1.6006	0.6786	0.001	0.001	14831	193520	54.959	2474	31470	8.9373	2130	27344	7.7655	14.3618	14.1298	86.0954	86.8891
SP8	ETV6_2	cluster_1	SP8	9119	5697.976	5408	5966	164.9092	1.6003	0.6783	0.001	0.0011429	9151	82658	22.329	2882	25678	6.9365	1020	9119	2.4633	11.1463	11.0322	35.3921	35.5129
SP8	POU2F3_1	cluster_1	SP8	8907	5566.829	5351	5799	135.7463	1.5999	0.678	0.001	0.0010435	3586	54118	58.145	681	9801	10.53	613	8907	9.5698	17.0943	16.4585	90.0147	90.8785
SP8	SPIC_2	cluster_1	SP8	29149	18220.975	17834	18603	230.3994	1.5997	0.6778	0.001	0.0010435	15787	205822	55.49	2615	33138	8.934	2276	29149	7.8586	14.4169	14.1622	87.0363	87.9625
SP4	TCF21_1	cluster_6	Common_2	49215	30773.589	30219	31310	322.4384	1.5992	0.6774	0.001	0.001	10517	136988	48.648	5010	63749	22.639	3813	49215	17.477	36.2556	35.9265	76.1078	77.2012
SP4	MESP1_1	cluster_6	Common_2	107021	66929.325	66320	67491	368.8683	1.599	0.6772	0.001	0.001	28293	255419	48.619	15538	138355	26.336	11930	107021	20.371	42.1659	41.9002	76.7795	77.3525
SP4	TBX15_2	cluster_4	SP4	15699	9819.6	9597	10022	127.1267	1.5987	0.6769	0.001	0.0010909	14978	104871	54.579	2637	18153	9.4475	2267	15699	8.1703	15.1355	14.9698	85.9689	86.4816
DP	NFATC1_1	cluster_2	DP	11326	7084.897	6802	7375	177.0972	1.5985	0.6767	0.001	0.0010909	4535	87510	54.48	757	13426	8.3585	621	11326	7.0511	13.6935	12.9425	82.0343	84.3587
SP4	TCF3_1	cluster_4	SP4	45881	28744.08	28218	29269	319.9052	1.5962	0.6746	0.001	0.0011429	45052	639694	51.735	4178	56573	4.5754	3323	45881	3.7106	7.3759	7.1723	79.5357	81.1005
SP8	TBX21_4	cluster_1	SP8	18037	11301.189	10960	11619	201.9465	1.596	0.6744	0.001	0.0010909	7628	114668	56.477	1404	20314	10.005	1231	18037	8.8836	16.1379	15.7298	87.6781	88.791
SP8	SOX7_4	cluster_1	SP8	7164	4494.822	4292	4710	131.228	1.5937	0.6724	0.001	0.0010909	2647	42472	56.757	551	8220	10.985	473	7164	9.5736	17.8693	16.8676	85.8439	87.1533
DP	ARID5B_1	cluster_2	DP	8009	5025.582	4798	5253	134.8371	1.5935	0.6722	0.001	0.0010909	4087	53311	52.552	781	9708	9.5698	635	8009	7.895	15.5371	15.0232	81.306	82.499
SP8	SOX21_2	cluster_1	SP8	9338	5866.054	5651	6080	130.3922	1.5918	0.6706	0.001	0.0010909	3688	51655	58.162	767	10297	11.594	686	9338	10.514	18.6009	18.0776	89.4394	90.6866
SP8	SOX9_6	cluster_1	SP8	11187	7029.052	6764	7315	166.8262	1.5915	0.6703	0.001	0.0010909	4230	63611	57.612	871	12456	11.281	773	11187	10.132	18.2742	17.5866	88.7486	89.8121
SP4	TCF4_2	cluster_6	Common_2	105583	66484.003	65909	67066	371.9945	1.5881	0.6673	0.001	0.001	28828	260376	48.438	15507	138006	25.673	11770	105583	19.642	40.8284	40.5502	75.9012	76.5061
SP8	TBX5_5	cluster_1	SP8	15951	10046.622	9705	10373	205.9089	1.5876	0.6669	0.001	0.0010435	5748	109382	56.634	980	17964	9.3011	863	15951	8.2588	15.0139	14.5828	88.0612	88.7943
DP	FOXP3_2	cluster_2	DP	4641	2923.452	2799	3036	69.703	1.5873	0.6666	0.001	0.0010909	4108	24648	54.212	909	5388	11.851	781	4641	10.208	19.0117	18.8291	85.9186	86.1359
DP	TBX15_1	cluster_2	DP	16890	10640.208	10280	10970	218.3678	1.5873	0.6666	0.001	0.0010909	5512	99328	52.536	1217	20690	10.943	969	16890	8.9333	17.5798	17.0043	79.622	81.6336
SP8	SOX14_2	cluster_1	SP8	6535	4119.359	3959	4304	103.1663	1.5863	0.6656	0.001	0.0010909	3330	36787	57.469	690	7315	11.428	610	6535	10.209	18.3183	17.7644	88.4058	89.337
DP	ATF4_2	cluster_2	DP	6100	3845.372	3653	4050	116.6248	1.5862	0.6655	0.001	0.0010909	3457	41526	51.125	650	7608	9.3666	518	6100	7.51	14.9841	14.6896	79.6923	80.1788
SP8	FOXP3_1	cluster_1	SP8	18854	11890.002	11524	12255	221.8334	1.5857	0.6651	0.001	0.0010909	7647	122767	55.431	1425	21824	9.8538	1215	18854	8.5128	15.8886	15.3575	85.2632	86.3911
DP	FOXO4_2	cluster_2	DP	12383	7814.611	7541	8049	151.6241	1.5845	0.664	0.001	0.0010435	5614	73565	57.489	1117	13917	10.876	985	12383	9.677	17.5454	16.8327	88.1826	88.9775
SP8	SPI1_known4	cluster_1	SP8	26183	16531.436	16152	16912	230.7467	1.5838	0.6634	0.001	0.0010435	14322	186719	57.192	2317	29252	8.9598	2054	26183	8.0198	14.3416	14.0227	88.6491	89.5084
SP8	TBX2_2	cluster_1	SP8	14242	8994.619	8730	9239	146.6303	1.5833	0.663	0.001	0.0010909	9067	90775	56.983	1622	15897	9.9792	1443	14242	8.9403	15.9149	15.6893	88.9642	89.5892
DP	TBX21_4	cluster_2	DP	19183	12121.201	11807	12475	217.9585	1.5826	0.6623	0.001	0.0010909	7045	105923	52.17	1649	23664	11.655	1314	19183	9.4481	18.6515	18.1103	79.6847	81.0641
SP4	TBX4_1	cluster_4	SP4	13131	8296.726	8110	8475	111.5754	1.5826	0.6623	0.001	0.0010435	11905	83358	55.556	2178	15032	10.018	1892	13131	8.7514	15.8925	15.7525	86.8687	87.3536
DP	RBPJ_1	cluster_2	DP	10044	6350.375	6179	6522	104.7448	1.5815	0.6613	0.001	0.0010435	10434	73042	50.795	1836	12593	8.7574	1452	10044	6.9848	13.916	13.751	79.085	79.7586
SP8	SOX18_2	cluster_1	SP8	7135	4512.698	4334	4710	114.2764	1.581	0.6608	0.001	0.0010909	2756	38602	58.784	591	7867	11.98	528	7135	10.865	19.1582	18.4835	89.3401	90.6953
SP8	TCF7L1_2	cluster_1	SP8	22871	14465.831	14199	14750	178.9926	1.581	0.6608	0.001	0.0010435	11685	129185	56.684	2407	25849	11.342	2112	22871	10.035	18.0745	17.7041	87.7441	88.4792
SP8	MAF_known1	cluster_1	SP8	51471	32559.28	31919	33073	342.1431	1.5808	0.6607	0.001	0.001	25525	385561	56.181	4027	58739	8.559	3485	51471	7.5	13.6533	13.3496	86.5408	87.6266
SP8	POU2F2_known1	cluster_1	SP8	9148	5786.968	5536	6032	157.4067	1.5807	0.6606	0.001	0.0010435	3160	57168	59.186	595	10054	10.409	531	9148	9.4709	16.8038	16.002	89.2437	90.9887
SP8	SOX10_9	cluster_1	SP8	8366	5303.634	5086	5542	137.2006	1.5773	0.6575	0.001	0.0010435	3412	47797	56.857	721	9597	11.416	619	8366	9.9518	18.1419	17.5032	85.853	87.1731
SP8	TCF12_known1	cluster_1	SP8	21383	13582.339	13314	13847	163.0608	1.5743	0.6547	0.001	0.0010909	34302	246920	53.035	3620	25716	5.5234	3013	21383	4.5928	8.7837	8.6599	83.232	83.1506
SP8	FOXO6_1	cluster_1	SP8	5986	3802.27	3629	3984	105.7391	1.5742	0.6546	0.001	0.0010909	2530	32988	57.537	544	6763	11.796	476	5986	10.441	18.8142	18.146	87.5	88.511
SP4	RBPJ_1	cluster_4	SP4	10464	6650.302	6455	6810	110.1747	1.5734	0.6539	0.001	0.0010435	11294	79074	54.99	1817	12285	8.5432	1530	10464	7.2769	13.547	13.2332	84.2047	85.177
SP8	RBPJ_2	cluster_1	SP8	12523	7958.484	7708	8213	147.9384	1.5735	0.6539	0.001	0.0010909	10072	100834	56.034	1475	14370	7.9855	1270	12523	6.9591	12.6092	12.4194	86.1017	87.1468
SP4	TBX5_3	cluster_4	SP4	22306	14177.934	13938	14484	167.1402	1.5732	0.6537	0.001	0.001	15391	138670	55.98	2921	25603	10.336	2527	22306	9.0047	16.4187	16.0857	86.5115	87.1226
SP8	MAF_known11	cluster_1	SP8	18881	12005.26	11663	12336	215.8891	1.5727	0.6532	0.001	0.0010435	10315	166406	54.325	1445	22364	7.3009	1197	18881	6.1639	11.6045	11.3463	82.8374	84.4259
SP8	SOX17_5	cluster_1	SP8	5968	3794.717	3612	3999	117.8344	1.5726	0.6531	0.001	0.0011429	2277	31990	58.026	514	6773	12.286	447	5968	10.825	19.6311	18.6558	86.965	88.1146
SP4	ETV6_2	cluster_4	SP4	15595	9917.657	9608	10269	201.8989	1.5724	0.653	0.001	0.0011429	10041	90698	24.501	4569	40741	11.006	1738	15595	4.2127	17.309	17.1944	38.039	38.2784
SP8	SMAD_2	cluster_1	SP8	30523	19414.129	19076	19764	212.0625	1.5722	0.6528	0.001	0.0010435	21409	221606	55.683	3462	35177	8.8389	2974	30523	7.6695	13.8914	13.7735	85.9041	86.7698
SP8	ARID5B_1	cluster_1	SP8	9689	6164.461	5952	6381	129.7573	1.5717	0.6523	0.001	0.0010909	4547	59233	58.39	872	10810	10.656	771	9689	9.5511	16.9562	16.3574	88.4174	89.63
SP8	SOX4_2	cluster_1	SP8	11514	7327.62	7070	7592	150.3446	1.5712	0.6519	0.001	0.0010909	4506	67750	58.989	888	12727	11.081	795	11514	10.025	17.6431	16.9948	89.527	90.4691
SP8	TBX4_2	cluster_1	SP8	17331	11030.235	10663	11413	224.1917	1.5712	0.6518	0.001	0.0010909	6718	127933	57.269	1068	19559	8.7555	935	17331	7.7581	13.9178	13.5469	87.5468	88.6088
DP	SPIB_1	cluster_2	DP	14866	9462.74	9266	9681	121.3964	1.5709	0.6516	0.001	0.0010435	15545	93270	50.008	3185	18963	10.167	2499	14866	7.9706	16.0759	15.9387	78.4615	78.3948
SP8	PBX1_2	cluster_1	SP8	8012	5100.505	4888	5304	119.8396	1.5707	0.6514	0.001	0.0010435	2880	41061	58.486	666	8974	12.782	585	8012	11.412	20.3125	19.5124	87.8378	89.2801
SP8	SOX18_4	cluster_1	SP8	5245	3339.534	3154	3498	108.0988	1.5704	0.6511	0.001	0.0012	2159	30301	57.531	451	5993	11.379	390	5245	9.9584	18.0639	17.3097	86.4745	87.5188
SP8	TBX1_2	cluster_1	SP8	13817	8798.027	8522	9104	180.7483	1.5704	0.6511	0.001	0.0010909	4843	87270	55.954	933	16006	10.262	793	13817	8.8589	16.3741	15.8325	84.9946	86.3239
SP8	TCF3_1	cluster_1	SP8	46912	29875.309	29289	30436	343.6356	1.5702	0.651	0.001	0.0011429	45409	645019	52.166	4255	58143	4.7023	3385	46912	3.794	7.4545	7.273	79.5535	80.6838
DP	SPI1_known4	cluster_2	DP	26340	16796.991	16416	17166	235.3278	1.5681	0.649	0.001	0.0010435	13368	174316	53.393	2515	31724	9.717	2057	26340	8.0679	15.3875	15.1105	81.7893	83.0286
DP	MAF_known10	cluster_2	DP	15772	10063.166	9794	10350	171.511	1.5672	0.6482	0.001	0.0010909	7909	87167	53.303	1769	18966	11.598	1452	15772	9.6447	18.3588	18.094	82.0803	83.1593
SP8	TBX15_1	cluster_1	SP8	15714	10038.475	9672	10356	209.0125	1.5653	0.6465	0.001	0.0010909	5892	106171	56.155	1054	18196	9.6241	899	15714	8.3113	15.258	14.8007	85.2941	86.3596
DP	CUX2_2	cluster_2	DP	248	158.11	127	189	19.1399	1.565	0.6461	0.001	0.0013333	279	2511	51.763	36	310	6.3904	28	248	5.1123	10.0358	9.8765	77.7778	80
SP8	POU2F2_known11	cluster_1	SP8	7414	4738.722	4583	4904	95.9438	1.5644	0.6456	0.001	0.0010435	4428	44372	59.446	843	8123	10.882	764	7414	9.9326	17.2538	16.7087	90.6287	91.2717
SP8	FOXO1_4	cluster_1	SP8	5403	3453.671	3298	3628	102.918	1.5643	0.6455	0.001	0.0010909	2229	29043	58.324	492	6089	12.228	431	5403	10.85	19.336	18.6035	87.6016	88.7338
SP8	SOX7_3	cluster_1	SP8	10267	6563.102	6297	6826	160.688	1.5643	0.6455	0.001	0.0010909	3353	53689	59.073	758	11442	12.589	670	10267	11.297	19.9821	19.1231	88.3905	89.7308
SP8	SOX8_9	cluster_1	SP8	8028	5134.664	4904	5352	131.0443	1.5634	0.6447	0.001	0.0010909	2766	44281	57.488	615	9226	11.978	526	8028	10.422	19.0166	18.1297	85.5285	87.015
SP8	SOX10_4	cluster_1	SP8	13606	8705.215	8449	8955	154.961	1.5629	0.6442	0.001	0.0010909	5401	81262	58.18	1057	15221	10.898	933	13606	9.7413	17.2746	16.7434	88.2687	89.3897
SP8	POU2F2_known15	cluster_1	SP8	5725	3665.098	3496	3830	96.737	1.5619	0.6433	0.001	0.0010435	3075	33949	59.103	593	6304	10.975	533	5725	9.9669	17.3333	16.8635	89.882	90.8154
SP8	POU2F2_known12	cluster_1	SP8	7325	4690.768	4511	4854	103.7765	1.5615	0.6429	0.001	0.0010435	4381	43877	60.114	834	7995	10.954	760	7325	10.036	17.3476	16.6944	91.1271	91.6198
SP8	POU2F2_known14	cluster_1	SP8	2339	1497.901	1389	1627	70.2012	1.5611	0.6426	0.001	0.0012632	928	14244	63.304	183	2502	11.12	168	2339	10.395	18.1034	16.4209	91.8033	93.4852
SP8	TCF3_1	cluster_6	Common_2	310350	198833.528	197717	200019	703.9226	1.5609	0.6423	0.001	0.0011429	45409	645019	52.166	27526	383634	31.026	22038	310350	25.1	48.5322	48.1149	80.0625	80.8974
DP	SPIC_1	cluster_2	DP	29240	18740.926	18294	19169	282.2241	1.5602	0.6417	0.001	0.001	13902	181322	51.494	2896	36649	10.408	2277	29240	8.304	16.3789	16.126	78.6257	79.7839
SP4	NFE2L1::MAFG_1	cluster_4	SP4	31631	20273.683	19721	20760	308.6798	1.5602	0.6417	0.001	0.0010435	8781	185151	56.968	1843	36408	11.202	1570	31631	9.7323	17.8795	17.0839	85.1872	86.8793
SP8	SOX30_1	cluster_1	SP8	8617	5523.299	5320	5743	137.7665	1.56	0.6416	0.001	0.0010435	3553	53360	60.089	664	9477	10.672	594	8617	9.7036	16.7183	16.1488	89.4578	90.9254
DP	MYBL1_3	cluster_2	DP	2263	1450.694	1349	1549	60.9619	1.5596	0.6411	0.001	0.0010909	1344	14803	55.839	247	2626	9.9057	212	2263	8.5364	15.7738	15.2874	85.83	86.1767
SP8	SOX8_2	cluster_1	SP8	12488	8007.04	7749	8256	156.4057	1.5596	0.6411	0.001	0.0010909	4985	74903	58.581	975	13966	10.923	861	12488	9.7667	17.2718	16.6722	88.3077	89.4172
SP8	MAF_known10	cluster_1	SP8	13034	8362.189	8140	8596	139.8129	1.5586	0.6403	0.001	0.0010909	8275	91208	55.774	1423	15177	9.2809	1207	13034	7.9704	14.5861	14.2904	84.8208	85.8799
SP8	TBX20_2	cluster_1	SP8	14228	9130.275	8803	9458	191.285	1.5583	0.6399	0.001	0.0010435	5480	98825	56.841	942	16294	9.3719	813	14228	8.1835	14.8358	14.3972	86.3057	87.3205
SP8	SOX17_4	cluster_1	SP8	6774	4348.542	4156	4524	114.3488	1.5576	0.6394	0.001	0.0010909	2660	37246	58.82	569	7564	11.945	502	6774	10.698	18.8722	18.1872	88.225	89.5558
SP8	NFATC1_3	cluster_1	SP8	5772	3706.812	3523	3884	106.7361	1.557	0.6388	0.001	0.0010909	3018	39257	57.876	541	6612	9.7479	466	5772	8.5095	15.4407	14.7031	86.1368	87.2958
SP4	TCF12_known1	cluster_4	SP4	23490	15087.087	14751	15358	190.9818	1.5569	0.6387	0.001	0.0010909	34730	249719	53.636	4034	28399	6.0997	3327	23490	5.0453	9.5796	9.4066	82.474	82.7142
SP8	IKZF2_2	cluster_1	SP8	4074	2616.446	2490	2743	77.8753	1.5569	0.6386	0.001	0.0010435	3469	34690	57.903	483	4645	7.7532	421	4074	6.8001	12.1361	11.744	87.1636	87.7072
SP8	PBX1_4	cluster_1	SP8	5609	3603.663	3419	3766	101.5623	1.5563	0.6381	0.001	0.0010909	2771	31106	57.753	591	6347	11.784	516	5609	10.414	18.6214	18.0319	87.3096	88.3725
SP8	POU2F3_2	cluster_1	SP8	4718	3031.498	2917	3147	71.7402	1.5561	0.638	0.001	0.0010909	3384	27131	59.282	656	5138	11.227	600	4718	10.309	17.7305	17.3897	91.4634	91.8256
SP8	SOX9_4	cluster_1	SP8	12107	7780.679	7546	8025	147.3133	1.556	0.6378	0.001	0.0010909	5012	75280	58.223	957	13666	10.57	835	12107	9.3638	16.66	16.0826	87.2518	88.5921
SP8	POU2F2_known5	cluster_1	SP8	7967	5123.274	4915	5344	133.7651	1.555	0.6369	0.001	0.0010909	3679	47888	58.57	730	8982	10.986	641	7967	9.7441	17.4232	16.6367	87.8082	88.6996
SP8	MGA_2	cluster_1	SP8	11670	7505.538	7227	7774	167.342	1.5548	0.6367	0.001	0.0010909	5058	86090	56.587	839	13515	8.8834	710	11670	7.6707	14.0372	13.5556	84.6246	86.3485
DP	IKZF2_2	cluster_2	DP	4753	3057.087	2908	3201	89.1103	1.5546	0.6365	0.001	0.0010435	3311	33116	55.275	584	5590	9.3305	488	4753	7.9334	14.7387	14.3526	83.5616	85.0268
DP	TBX20_5	cluster_2	DP	31666	20372.906	19907	20852	274.3426	1.5543	0.6363	0.001	0.0010909	11935	182788	54.157	2625	38253	11.334	2138	31666	9.3822	17.9137	17.3239	81.4476	82.7804
DP	TBX1_4	cluster_2	DP	27172	17490.32	17057	17989	290.0894	1.5535	0.6355	0.001	0.0010909	7984	152301	52.016	1881	34050	11.629	1465	27172	9.2802	18.3492	17.841	77.8841	79.8003
DP	TCF21_1	cluster_2	DP	34970	22514.284	22012	22968	266.2693	1.5532	0.6353	0.001	0.001	11430	148923	52.886	3322	42596	15.127	2706	34970	12.419	23.6745	23.4819	81.457	82.0969
SP8	HBP1_1	cluster_1	SP8	10133	6525.05	6292	6791	155.2543	1.5529	0.6349	0.001	0.0010435	4130	63662	59.134	791	11380	10.571	697	10133	9.4123	16.8765	15.9169	88.1163	89.0422
SP8	SMAD3_1	cluster_1	SP8	22987	14819.734	14546	15106	175.4441	1.5511	0.6333	0.001	0.0010435	19958	165967	56.427	3179	26335	8.9536	2760	22987	7.8153	13.829	13.8503	86.8198	87.2869
SP8	SOX5_1	cluster_1	SP8	3528	2275.22	2169	2380	66.089	1.5504	0.6326	0.001	0.0010909	2249	20248	60.174	438	3829	11.379	400	3528	10.485	17.7857	17.4239	91.3242	92.1389
DP	LMO2_2	cluster_2	DP	3867	2494.471	2385	2619	71.2366	1.55	0.6323	0.001	0.0010435	3407	27268	53.534	598	4669	9.1664	490	3867	7.5919	14.3822	14.1815	81.9398	82.8229
DP	MAFG_1	cluster_2	DP	22399	14459.394	14031	14920	266.8647	1.5491	0.6314	0.001	0.0010435	5947	119184	52.184	1475	28081	12.295	1144	22399	9.8072	19.2366	18.7936	77.5593	79.7657
SP8	HESX1_2	cluster_1	SP8	2164	1396.626	1291	1500	62.5335	1.5491	0.6314	0.001	0.0014118	899	12600	63.31	177	2307	11.592	164	2164	10.873	18.2425	17.1746	92.6554	93.8015
DP	TBX2_2	cluster_2	DP	15317	9887.879	9620	10137	155.2073	1.549	0.6313	0.001	0.0010909	8541	85538	53.696	1906	18653	11.709	1561	15317	9.6151	18.2765	17.9067	81.8993	82.1155
SP8	HESX1_1	cluster_1	SP8	1532	988.803	913	1054	43.1312	1.5488	0.6311	0.001	0.0012632	1301	11720	59.084	201	1706	8.6005	179	1532	7.7233	13.7586	13.0717	89.0547	89.8007
DP	TBX20_2	cluster_2	DP	15689	10134.289	9815	10434	204.4601	1.5481	0.6305	0.001	0.0010435	5116	92297	53.087	1123	19395	11.155	890	15689	9.0239	17.3964	16.9984	79.252	80.892
SP4	TCF3_1	cluster_6	Common_2	305158	197145.533	196004	198227	684.3222	1.5479	0.6303	0.001	0.0011429	45052	639694	51.735	27526	383634	31.026	21664	305158	24.68	48.0867	47.7037	78.7038	79.544
SP8	SOX11_2	cluster_1	SP8	8860	5724.123	5523	5928	127.6552	1.5477	0.6302	0.001	0.0010909	3666	51361	60.532	731	9670	11.397	662	8860	10.442	18.0578	17.2504	90.5609	91.6236

SP4	TBX21_4	cluster_4	SP4	22012	14226.855	13898	14572	203.0973	1.5472	0.6296	0.001	0.0010909	7892	118588	58.407	1733	24847	12.238	1516	22012	10.841	19.2093	18.5617	87.4784	88.5902
SP4	ARID5B_1	cluster_4	SP4	10468	6773.224	6559	6974	131.5839	1.5454	0.628	0.001	0.0010909	4639	60416	59.556	937	11641	11.475	835	10468	10.319	17.9996	17.3265	89.1142	89.9235
SP8	SOX2_6	cluster_1	SP8	5465	3538.957	3376	3738	110.7973	1.5441	0.6268	0.001	0.0010435	1941	31108	57.681	424	6325	11.728	358	5465	10.133	18.4441	17.5678	84.434	86.4032
DP	SPIC_2	cluster_2	DP	31317	20285.533	19841	20751	265.4518	1.5438	0.6265	0.001	0.0010435	14989	195470	52.699	3056	38712	10.437	2433	31317	8.4431	16.2319	16.0214	79.6139	80.8974
DP	IRF4_1	cluster_2	DP	3970	2573.225	2417	2736	95.415	1.5426	0.6254	0.001	0.0010435	2787	39153	57.73	341	4540	6.6941	291	3970	5.8536	10.4413	10.1397	85.3372	87.4449
DP	NFE2L1::MAFG_1	cluster_2	DP	32626	21153.028	20619	21675	324.4012	1.5424	0.6251	0.001	0.0010435	8197	173116	53.265	2037	40418	12.436	1597	32626	10.038	19.4827	18.8463	78.3996	80.7215
SP4	POU2F2_known16	cluster_4	SP4	7988	5180.081	4992	5387	122.3174	1.542	0.6248	0.001	0.0010909	2634	34687	58.999	737	9083	15.449	639	7988	13.587	24.2597	23.0288	86.7028	87.9445
SP8	TBX21_6	cluster_1	SP8	22267	14439.898	14045	14829	254.9359	1.542	0.6248	0.001	0.0010909	8068	145632	57.226	1472	25611	10.064	1266	22267	8.7498	15.6916	15.2899	86.0054	86.9431
SP4	RUNX_1	cluster_6	Common_2	113142	73437.035	72631	74217	502.403	1.5407	0.6235	0.001	0.0010435	22871	322027	50.309	10721	147133	22.986	8133	113142	17.676	35.5603	35.1343	75.8605	76.8978
SP8	SPDEF_4	cluster_1	SP8	15376	9979.81	9653	10252	177.3894	1.5407	0.6235	0.001	0.0010435	7014	105694	58.627	1228	17521	9.7187	1062	15376	8.5289	15.1411	14.5477	86.4821	87.7575
SP8	SOX11_1	cluster_1	SP8	27991	18170.626	17710	18586	263.1465	1.5404	0.6233	0.001	0.0010435	10921	177105	60.65	2020	30899	10.581	1807	27991	9.5856	16.5461	15.8047	89.4554	90.5887
SP8	FOXO4_3	cluster_1	SP8	5108	3316.053	3145	3482	102.8522	1.5402	0.6231	0.001	0.0010909	2096	27285	58.087	459	5801	12.35	400	5108	10.874	19.084	18.7209	87.146	88.0538
SP8	SOX12_1	cluster_1	SP8	7001	4547.749	4356	4726	114.8906	1.5393	0.6223	0.001	0.0010435	2969	38740	61.39	625	7670	12.154	561	7001	11.094	18.8953	18.0718	89.76	91.2777
DP	TBX5_5	cluster_2	DP	18072	11742.894	11374	12142	232.1174	1.5389	0.6219	0.001	0.0010435	5461	103978	53.836	1223	22231	11.51	976	18072	9.357	17.8722	17.3806	79.8038	81.2919
SP8	SOX_1	cluster_1	SP8	12635	8213.047	7989	8457	149.1029	1.5383	0.6214	0.001	0.0010435	6625	79556	57.773	1257	14434	10.482	1085	12635	9.1755	16.3774	15.8819	86.3166	87.5364
SP4	TBX1_2	cluster_4	SP4	15868	10315.775	9970	10651	204.2889	1.5382	0.6212	0.001	0.0010909	4990	89894	57.636	1076	18201	11.67	917	15868	10.174	18.3768	17.6519	85.223	87.182
SP8	FOXO3_4	cluster_1	SP8	4674	3039.514	2892	3200	87.4217	1.5376	0.6207	0.001	0.0010909	1903	24809	59.374	419	5251	12.567	369	4674	11.186	19.3904	18.8399	88.0668	89.0116
SP4	PKNOX1_1	cluster_4	SP4	28865	18779.166	18408	19199	229.7936	1.537	0.6202	0.001	0.0010435	11588	174615	56.273	2333	33838	10.905	1967	28865	9.3022	16.9745	16.5307	84.312	85.3035
SP4	RUNX_2	cluster_6	Common_2	97406	63384.468	62580	64204	463.8938	1.5367	0.6199	0.001	0.0010435	18786	264509	50.895	9187	125839	24.213	7006	97406	18.742	37.2937	36.8252	76.2599	77.4053
DP	SPIB_2	cluster_2	DP	32685	21269.278	20814	21712	260.5546	1.5367	0.6198	0.001	0.0010435	16381	214053	53.087	3187	40326	10.001	2545	32685	8.1061	15.5363	15.2696	79.8557	81.0519
SP8	EWSR1::FLI1_1	cluster_1	SP8	96942	63111.267	62245	64035	562.396	1.536	0.6192	0.001	0.0010435	45443	904295	55.466	6241	116963	7.1741	5066	96942	5.9461	11.148	10.7202	81.1729	82.8826
SP8	SPZ1_2	cluster_1	SP8	16815	10947.751	10707	11201	153.1568	1.5359	0.6191	0.001	0.0010435	13891	139218	56.526	1996	19554	7.9394	1698	16815	6.8273	12.2237	12.0782	85.0701	85.9926
SP8	PBX3_known1	cluster_1	SP8	10359	6748.223	6511	6967	140.4514	1.535	0.6182	0.001	0.0010435	6890	76602	56.96	1114	11983	8.9104	956	10359	7.7028	13.8752	13.5231	85.8169	86.4475
SP8	TBX20_5	cluster_1	SP8	29499	19226.16	18822	19580	230.5445	1.5343	0.6176	0.001	0.0010909	12908	197715	58.58	2275	33402	9.8965	1987	29499	8.7401	15.3936	14.92	87.3407	88.3151
SP8	SOX4_1	cluster_1	SP8	29454	19198.406	18778	19681	269.7736	1.5342	0.6175	0.001	0.0010435	11786	191082	60.682	2132	32642	10.366	1903	29454	9.3537	16.1463	15.4143	89.2589	90.2334
SP4	TBX15_1	cluster_4	SP4	18769	12236.169	11869	12595	219.5425	1.5339	0.6172	0.001	0.0010909	6066	109292	57.806	1270	21552	11.399	1082	18769	9.9271	17.8371	17.1733	85.1969	87.087
SP8	SMAD_1	cluster_1	SP8	15958	10410.784	10194	10642	135.8495	1.5328	0.6162	0.001	0.0010435	15801	126448	57.942	2290	18078	8.2838	2009	15958	7.3124	12.7144	12.6202	87.7293	88.273
DP	E2F2_2	cluster_2	DP	6614	4316.138	4108	4525	124.3947	1.5323	0.6157	0.001	0.0010909	2804	47831	58.459	465	7560	9.2398	403	6614	8.0836	14.3723	13.8279	86.6667	87.4868
SP4	FOXO1_4	cluster_4	SP4	7156	4674.952	4473	4855	113.4586	1.5306	0.6141	0.001	0.0010909	2354	30679	61.609	628	7807	15.678	569	7156	14.371	24.1716	23.3254	90.6051	91.6613
DP	TBX5_3	cluster_2	DP	26457	17292.33	16981	17615	197.0247	1.53	0.6135	0.001	0.001	14895	134213	54.181	3634	32120	12.967	2968	26457	10.68	19.9261	19.7127	81.6731	82.3692
DP	RUNX_1	cluster_6	Common_2	103861	67889.215	67215	68681	464.5389	1.5299	0.6134	0.001	0.0010435	21137	297645	46.5	10721	147133	22.986	7445	103861	16.226	35.2226	34.8943	69.4431	70.5899
SP8	POU2F2_known10	cluster_1	SP8	6781	4433.929	4271	4576	91.0108	1.5292	0.6128	0.001	0.0010435	4821	43499	59.761	854	7508	10.315	765	6781	9.3161	15.8681	15.5889	89.5785	90.317
SP8	SOX15_2	cluster_1	SP8	8170	5344.86	5143	5546	125.7125	1.5285	0.6121	0.001	0.0010909	3083	43248	60.363	679	9108	12.712	604	8170	11.403	19.5913	18.891	88.9543	89.7014
DP	RBPJ_2	cluster_2	DP	13920	9107.707	8867	9351	150.4297	1.5283	0.6119	0.001	0.0010909	9585	95962	53.327	1775	17292	9.6093	1407	13920	7.7354	14.6792	14.5057	79.2676	80.4997
SP4	POU2F2_known14	cluster_4	SP4	2377	1554.999	1440	1663	67.8977	1.5283	0.6119	0.001	0.0012632	922	14165	62.953	195	2647	11.764	171	2377	10.564	18.5466	16.7808	87.6923	89.7998
SP4	SOX15_4	cluster_4	SP4	7865	5146.118	4964	5345	114.506	1.5282	0.6119	0.001	0.0011429	2335	32728	59.319	676	8954	16.229	588	7865	14.255	25.182	24.0314	86.9822	87.8378
SP8	MAF_known12	cluster_1	SP8	15969	10450.885	10115	10780	203.7762	1.528	0.6116	0.001	0.0010435	7662	130608	59.214	1102	17974	8.1489	963	15969	7.2399	12.5685	12.2267	87.3866	88.845
DP	MAFF_1	cluster_2	DP	24202	15862.081	15454	16258	239.2437	1.5257	0.6095	0.001	0.001	9294	130389	52.132	2253	30581	12.227	1752	24202	9.6764	18.8509	18.5614	77.763	79.1406
DP	PBX3_known1	cluster_2	DP	10364	6794.847	6569	7025	139.5685	1.5252	0.609	0.001	0.0010435	6370	70693	52.566	1236	13124	9.7589	957	10364	7.7066	15.0235	14.6606	77.4272	78.9698
SP8	SOX3_1	cluster_1	SP8	6466	4240.584	4038	4425	114.8632	1.5247	0.6085	0.001	0.0010909	2117	33889	61.352	481	7166	12.973	427	6466	11.706	20.1701	19.0799	88.7734	90.2316
DP	SMAD_2	cluster_2	DP	36435	23900.827	23517	24308	244.5903	1.5244	0.6082	0.001	0.0010435	20655	212873	53.488	4479	45031	11.315	3593	36435	9.155	17.3953	17.1158	80.2188	80.9109
SP4	MGA_2	cluster_4	SP4	14625	9601.906	9297	9929	185.9318	1.5231	0.607	0.001	0.0010909	5162	87892	57.771	1040	16927	11.126	879	14625	9.613	17.0283	16.6397	84.5192	86.4004
SP4	SOX3_3	cluster_4	SP4	10488	6893.683	6619	7142	157.3643	1.5213	0.6053	0.001	0.0010909	2900	46500	59.883	791	11919	15.349	687	10488	13.506	23.6897	22.5548	86.8521	87.994
SP8	FOXM1_1	cluster_1	SP8	5572	3664.006	3540	3795	75.9481	1.5206	0.6046	0.001	0.0010909	4986	39915	56.233	835	6541	9.2151	707	5572	7.85	14.1797	13.9597	84.6707	85.1858
SP8	SOX21_3	cluster_1	SP8	8573	5640.738	5416	5854	137.0837	1.5197	0.6038	0.001	0.0010909	3510	52680	59.82	686	9659	10.968	600	8573	9.735	17.094	16.2737	87.4636	88.7566
SP4	HESX1_1	cluster_4	SP4	1785	1174.839	1102	1252	48.04	1.5189	0.603	0.001	0.0012632	1328	11959	60.289	235	1983	9.997	206	1785	8.9988	15.512	14.926	87.6596	90.0151
DP	RUNX_2	cluster_6	Common_2	89668	59039.48	58332	59804	453.9051	1.5188	0.6029	0.001	0.0010435	17458	245690	47.274	9187	125839	24.213	6438	89668	17.253	36.8771	36.4964	70.0773	71.2561
DP	TBX21_6	cluster_2	DP	22063	14532.103	14123	14959	254.2793	1.5182	0.6024	0.001	0.0010909	7546	136200	53.52	1600	27516	10.812	1258	22063	8.6696	16.6711	16.199	78.625	80.1824
SP4	FOXO6_1	cluster_4	SP4	7946	5235.211	5037	5407	114.3661	1.5177	0.6019	0.001	0.0010909	2688	35069	61.166	700	8761	15.281	632	7946	13.859	23.5119	22.6582	90.2857	90.6974
SP4	TBX2_2	cluster_4	SP4	16283	10733.821	10512	10982	142.9637	1.5169	0.6012	0.001	0.0010909	9302	93136	58.465	1911	18576	11.661	1658	16283	10.222	17.8241	17.483	86.7609	87.6561
SP4	FOXM1_1	cluster_4	SP4	6735	4439.759	4291	4589	92.2064	1.5169	0.6011	0.001	0.0010909	5181	41487	58.448	992	7674	10.811	862	6735	9.4885	16.6377	16.234	86.8952	87.7639
DP	PKNOX1_1	cluster_2	DP	29262	19297.415	18849	19732	277.2825	1.5163	0.6006	0.001	0.0010435	10906	164350	52.965	2530	36762	11.847	1974	29262	9.4302	18.1001	17.8047	78.0237	79.5985
DP	TCF3_2	cluster_2	DP	107560	70951.992	70156	71790	515.1292	1.5159	0.6002	0.001	0.0010435	27591	417427	54.427	8790	130696	17.041	7148	107560	14.024	25.907	25.7674	81.3197	82.2979
DP	FOXM1_1	cluster_2	DP	5965	3934.968	3793	4061	81.9751	1.5158	0.6	0.001	0.0010909	4728	37858	53.335	953	7417	10.449	760	5965	8.4037	16.0745	15.7562	79.7482	80.4234
SP8	TCF12_known1	cluster_6	Common_2	113955	75185.214	74619	75747	349.9099	1.5157	0.5999	0.001	0.0010909	34302	246920	53.035	19920	142178	30.538	15889	113955	24.476	46.3209	46.1506	79.7641	80.1495
DP	TBX4_1	cluster_2	DP	14993	9895.48	9689	10115	127.2135	1.5151	0.5994	0.001	0.0010435	11484	80419	53.597	2687	18553	12.365	2160	14993	9.9924	18.8088	18.6436	80.387	80.8117
SP8	ETV2_1	cluster_1	SP8	19905	13150.442	12888	13413	162.2739	1.5136	0.598	0.001	0.0010435	16594	166680	56.55	2363	23363	7.9265	1994	19905	6.7533	12.0164	11.942	84.3843	85.1988
SP4	TBX21_6	cluster_4	SP4	26333	17397.949	16984	17843	265.9833	1.5135	0.5979	0.001	0.0010909	8330	150313	59.065	1741	29870	11.737	1502	26333	10.348	18.0312	17.5188	86.2723	88.1587
DP	CUX1_8	cluster_6	Common_2	2740	1811.332	1685	1948	82.3372	1.5124	0.5969	0.001	0.0011429	409	6571	49.473	266	3873	29.16	182	2740	20.629	44.4988	41.6984	68.4211	70.7462
DP	MAF_known1	cluster_2	DP	56911	37626.581	37051	38267	355.8647	1.5125	0.5969	0.001	0.001	24308	367004	53.477	4920	71240	10.381	3850	56911	8.2927	15.8384	15.5069	78.252	79.8863
SP8	NFATC1_1	cluster_1	SP8	13017	8605.966	8261	8886	189.7791	1.5125	0.5969	0.001	0.0010909	5066	97783	60.876	818	14633	9.1099	713	13017	8.1039	14.0742	13.3121	87.1638	88.9565
DP	IKZF3_1	cluster_2	DP	10299	6809.322	6573	7045	144.261	1.5124	0.5968	0.001	0.0010435	6241	75044	55.627	1082	12441	9.222	883	10299	7.6342	14.1484	13.7239	81.6081	82.7827
SP8	SOX5_3	cluster_1	SP8	6540	4324.684	4152	4499	108.1735	1.5121	0.5966	0.001	0.0011429	2486	37371	63.453	516	7140	12.123	465	6540	11.104	18.7047	17.5002	90.1163	91.5966
SP4	TBX19_1	cluster_4	SP4	13495	8928.378	8630	9222	182.1875	1.5114	0.5959	0.001	0.0010435	3815	72708	56.872	888	16009	12.522	732	13495	10.556	19.1874	18.5605	82.4324	84.2963
SP8	MGA_3	cluster_1	SP8	11027	7296.672	7033	7574	155.4764	1.5112	0.5957	0.001	0.0010909	4956	84386	59.559	760	12400	8.7519	668	11027	7.7828	13.4786	13.0673	87.8947	88.9274
SP8	SOX13_1	cluster_1	SP8	5596	3703.11	3528	3879	107.5364	1.511	0.5955	0.001	0.0010909	2089	31443	63.156	443	6166	12.385	395	5596	11.24	18.9086	17.7973	89.1648	90.7558
SP8	BPTF_1	cluster_1	SP8	27878	18450.45	18095	18852	235.3734	1.5109	0.5954	0.001	0.0010909	12575	168522	60.074	2436	31404	11.195	2143	27878	9.9379	17.0417	16.5426	87.9721	88.7721
SP4	MYC_known13	cluster_4	SP4	10901	7215.925	6981	7421	128.683	1.5106	0.5951	0.001	0.0010909	9038	99518	55.876	1221	13066	7.3361	1010	10901	6.1205	11.175	10.9538	82.7191	83.4303
DP	SMAD3_3	cluster_2	DP	4860	3218.015	3104	3350	74.6072	1.5101	0.5946	0.001	0.0010435	3577	32203	55.927	650	5760	10.003	545	4860	8.4404	15.2362	15.0918	83.8462	84.375
DP	TBX4_2	cluster_2	DP	21234	14060.945	13626	14452	263.6167	1.5101	0.5946	0.001	0.0010909	6487	123601	55.329	1423	25905	11.596	1147	21234	9.5053	17.6815	17.1795	80.6044	81.9687
DP	SMAD3_1	cluster_2	DP	26493	17545.637	17239	17851	189.6144	1.5099	0.5945	0.001	0.0010435	19225	159039	54.071	4021	32716	11.123	3241	26493	9.0073	16.8583	16.6582	80.6018	80.9787
SP4	SOX14_3	cluster_4	SP4	10436	6911.245	6677	7138	134.9392	1.5099	0.5945	0.001	0.0010909	3418	47906	58.588	902	12080	14.774	768	10436	12.763	22.4693	21.7843	85.1441	86.3907
SP8	FOXO1_5	cluster_1	SP8	38683	25637.424	25188	26042	259.377	1.5088	0.5934	0.001	0.0010435	25271	279379	58.574	4092	44229	9.2729	3545	38683	8.1102	14.0279	13.8461	86.6325	87.4607
SP8	ATF4_2	cluster_1	SP8	6385	4233.943	4057	4402	108.4549	1.5079	0.5926	0.001	0.0010909	3830	46012	56.648	650	7549	9.2939	545	6385	7.8609	14.2298	13.8768	83.8462	84.5807
SP4	FOXP3_1	cluster_4	SP4	24525	16265.756	15877	16648	234.6868	1.5077	0.5924	0.001	0.0010909	7981	128134	57.854	1889	28677	12.948	1596	24525	11.073	19.9975	19.1401	84.4891	85.5215
SP4	RBPJ_2	cluster_4	SP4	13939	9262.288	9036	9498	152.2501	1.5049	0.5896	0.001	0.0010909	10316	103270	57.388	1692	16343	9.0819	1424	13939	7.746	13.8038	13.4976	84.1608	85.2903
SP4	SMAD_2	cluster_4	SP4	36392	24185.22	23798	24632	253.3975	1.5047	0.5895	0.001	0.0010435	22080	227820	57.244	4228	42666	10.721	3589	36392	9.1442	16.2545	15.974	84.8865	85.2951
SP4	SOX14_2	cluster_4	SP4	8180	5436.694	5238	5613	113.1853	1.5045	0.5893	0.001	0.0010909	3491	38572	60.257	866	9223	14.408	762	8180	12.779	21.8276	21.2071	87.9908	88.6913
SP4	TBX5_5	cluster_4	SP4	19076	12683.698	12289	13069	238.8627	1.5039	0.5887	0.001	0.0010435	5992	114030	59.04	1206	21904	11.341	1039	19076	9.8768	17.3398	16.7289	86.1526	87.0891
SP4	SOX18_4	cluster_4	SP4	7502	4989.936	4789	5175	113.963	1.5033	0.5882	0.001	0.0012	2335	32760	62.2	642	8375	15.901	567	7502	14.244	24.2827	22.8999	88.3178	89.5761
SP4	MAF_known10	cluster_4	SP4	17082	11363.46	11134	11637	159.1501	1.5032	0.588	0.001	0.0010909	8761	96521	59.023	1819	19475	11.909	1580	17082	10.446	18.0345	17.6977	86.8609	87.7125
DP	ID4_1	cluster_2	DP	78738	52386.648	51770	52962	363.3849	1.503	0.5879	0.001	0.0010435	35081	317583	54.366	10793	96618	16.54	8729	78738	13.479	24.8824	24.7929	80.8765	81.4941
SP4	SOX7_4	cluster_4	SP4	10734	7145.036	6901	7418	161.0727	1.5022	0.5871	0.001	0.0010909	2839	45561	60.885	802	12131	16.211	699	10734	14.344	24.6213	23.5596	87.1571	88.484
SP8	IKZF3_1	cluster_1	SP8	7880	5246.17	5053	5437	122.4502	1.502	0.5868	0.001	0.0010435	6473	77854	57.71	817	9334	6.9189	683	7880	5.8411	10.5515	10.1215	83.5985	84.4225
SP4	SOX2_5	cluster_4	SP4	11607	7728.348	7484	8010	161.5905	1.5018	0.5867	0.001	0.0010909	3385	54207	61.44	874	13039	14.779	765	11607	13.156	22.5997	21.4124	87.5286	89.0176
DP	TBX15_2	cluster_2	DP	18775	12504.923	12253	12734	144.3418	1.5014	0.5863	0.001	0.0010909	14654	102598	53.396	3419	23567	12.265	2709	18775	9.7712	18.4864	18.2996	79.2337	79.6665
DP	POU2F2_known14	cluster_6	Common_2	4416	2942.076	2771	3109	100.4405	1.5008	0.5857	0.001	0.0012632	685	10526	46.78	474	6572	29.208	301	4416	19.626	43.9416	41.9533	63.5021	67.1942
SP8	TCF7_1	cluster_2	DP	14373	18004.538	17638	18408	226.1932	0.7983	-0.325	0.001	0.0010435	10694	171757	56.165	2102	32593	10.658	903	14373	4.7	8.444	8.3682	42.9591	44.0984
SP8	FOXP1_1	cluster_1	SP8	37925	25282.68	24749	25879	349.3987	1.5	0.585	0.001	0.0010435	10498	244896	63.1	2385	45631	11.757	1944	37925	9.7718	18.5178	15.4862	81.5094	83.1124
SP4	FOXO1_5	cluster_4	SP4	35571	23721.172	23338	24172	251.6056	1.4995	0.5845	0.001	0.0010435	25037	276688	58.01	3867	41421	8.6842	3284	35571	7.4577	13.1166	12.856	84.9237	85.8767
DP	IRF6_1	cluster_2	DP	4112	2743.849	2569	2918	104.6782	1.4984	0.5835	0.001	0.0010909	2388	38278	58.421	317	4808	7.3381	266	4112	6.2759	11.139	10.7425	83.9117	85.5241
SP8	IKZF1_2	cluster_1	SP8	19270	12859.722	12617	13083	142.002	1.4984	0.5835	0.001	0.0010435	20377	163023	59.485	2799	21823	7.9629	2454	19270	7.0313	12.043	11.8204	87.6742	88.3013
SP4	TBX20_2	cluster_4	SP4	16418	10956.728	10585	11292	212.1463	1.4984	0.5834	0.001	0.0010435	5606	101105	58.153	1131	19160	11.02	945	16418	9.4432	16.8569	16.2386	83.5544	85.6889
SP8	TBX2_1	cluster_1	SP8	19079	12739.59	12392	13085	212.794	1.4976	0.5826	0.001	0.0010435	8536	145462	59.643	1317	21623	8.866	1148	19079	7.8229	13.4489	13.1161	87.1678	88.2348
SP4	SOX17_4	cluster_4	SP4	8561	5716.746	5532	5929	116.4234	1.4974	0.5825	0.001	0.0010909	2822	39514	62.402	725	9491	14.988	645	8561	13.52	22.8561	21.6657	88.9655	90.2012
DP	LMO2_1	cluster_2	DP	105911	70774.65	70025	71388	404.7397	1.4964	0.5815	0.001	0.001	40696	452099	54.045	11969	131187	15.682	9576	105911	12.661	23.5306	23.4265	80.0067	80.7328
SP4	FOXO4_3	cluster_4	SP4	6639	4436.371	4257	4606	105.8663	1.4964	0.5815	0.001	0.0010909	2238	29139	62.034	581	7296	15.532	526	6639	14.134	23.5031	22.7839	90.5336	90.9951
SP8	HMGA1_2	cluster_1	SP8	12806	8557.447	8298	8818	154.2701	1.4964	0.5815	0.001	0.0010435	5565	78050	63.498	1076	14034	11.417	971	12806	10.418	17.4483	16.4074	90.2416	91.2498
SP4	TBX4_2	cluster_4	SP4	19848	13273.433	12865	13635	229.072	1.4953	0.5804	0.001	0.0010909	6921	131760	58.982	1263	22909	10.255	1079	19848	8.8849	15.5902	15.0638	85.4315	86.6384
SP4	SOX1_3	cluster_4	SP4	9737	6514.698	6294	6746	142.0505	1.4945	0.5797	0.001	0.0010909	3151	44155	58.678	855	11384	15.128	722	9737	12.94	22.9134	22.0519	84.4444	85.5323
DP	MGA_3	cluster_2	DP	13851	9271.661	8943	9566	187.7051	1.4939	0.579	0.001	0.0010909	4692	79902	56.395	1019	16639	11.744	840	13851	9.776	17.9028	17.335	82.4338	83.2442
SP8	FOXP3_2	cluster_1	SP8	4670	3126.416	2999	3233	70.5054	1.4936	0.5788	0.001	0.0010909	4518	27108	59.623	895	5284	11.622	787	4670	10.271	17.4192	17.2274	87.933	88.38
SP4	FOXO3_4	cluster_4	SP4	6246	4182.451	4007	4339	101.5435	1.4933	0.5785	0.001	0.0010909	2012	26245	62.811	546	6810	16.298	496	6246	14.948	24.6521	23.7988	90.8425	91.7181
SP8	POU2F2_known13	cluster_1	SP8	8625	5776.594	5545	6036	149.6906	1.493	0.5782	0.001	0.0010909	2653	55941	63.012	494	9555	10.763	437	8625	9.7152	16.4719	15.418	88.4615	90.2669
SP8	FOXO6_3	cluster_1	SP8	38074	25504.335	25108	25973	260.3868	1.4928	0.578	0.001	0.0010435	22108	289208	59.065	3425	43712	8.9273	2955	38074	7.7759	13.3662	13.1649	86.2774	87.1019
DP	MGA_2	cluster_2	DP	14313	9588.257	9278	9891	186.878	1.4927	0.5779	0.001	0.0010909	4878	83018	54.568	1087	17784	11.689	858	14313	9.4079	17.5892	17.2408	78.9328	80.4825
SP4	TBX20_5	cluster_4	SP4	35306	23671.15	23234	24110	277.5658	1.4915	0.5768	0.001	0.0010909	13251	203127	60.183	2754	40150	11.896	2389	35306	10.461	18.0288	17.3812	86.7466	87.9352
SP4	SOX21_2	cluster_4	SP4	11168	7488.492	7241	7712	150.7035	1.4913	0.5766	0.001	0.0010909	3895	54547	61.419	940	12505	14.08	831	11168	12.575	21.335	20.4741	88.4043	89.3083
SP4	SOX1_2	cluster_4	SP4	9804	6578.6	6328	6801	144.2075	1.4902	0.5755	0.001	0.0010909	3418	47871	59.739	855	11323	14.13	730	9804	12.235	21.3575	20.48	85.3801	86.5848
SP4	TCF12_known1	cluster_6	Common_2	113272	76027.452	75496	76543	303.2291	1.4899	0.5752	0.001	0.0010909	34730	249719	53.636	19920	142178	30.538	15806	113272	24.329	45.5111	45.3598	79.3474	79.6691
SP8	ATF4_3	cluster_1	SP8	7209	4838.48	4633	5036	117.8738	1.4898	0.5751	0.001	0.0010909	3790	49324	56.677	688	8653	9.943	567	7209	8.2837	14.9604	14.6156	82.4128	83.3121
SP4	SPZ1_2	cluster_4	SP4	19489	13082.862	12810	13358	176.5485	1.4896	0.5749	0.001	0.0010435	14241	142725	57.95	2339	22816	9.2638	1986	19489	7.913	13.9456	13.6549	84.9081	85.4181
SP4	NFATC1_3	cluster_4	SP4	6309	4240.043	4082	4428	102.4989	1.4878	0.5732	0.001	0.0010909	3149	40963	60.391	597	7250	10.688	510	6309	9.3012	16.1956	15.4017	85.4271	87.0207
SP4	FOXO6_3	cluster_4	SP4	34987	23521.536	23064	23936	262.3749	1.4874	0.5728	0.001	0.0010435	21804	285200	58.247	3237	40909	8.3549	2740	34987	7.1454	12.5665	12.2675	84.6463	85.524
SP4	MAFF_1	cluster_4	SP4	21463	14430.934	14119	14786	211.8636	1.4873	0.5727	0.001	0.001	9927	139260	55.679	1924	26154	10.457	1551	21463	8.5813	15.6241	15.4122	80.6133	82.0639
DP	TCF4_2	cluster_2	DP	69590	46819.03	46295	47358	312.0457	1.4864	0.5718	0.001	0.001	32705	295539	54.979	9571	85376	15.882	7731	69590	12.946	23.6386	23.5468	80.7753	81.51
SP4	MAFG_1	cluster_4	SP4	20922	14078.57	13696	14538	243.2861	1.4861	0.5715	0.001	0.0010435	6447	129183	56.562	1341	25357	11.102	1073	20922	9.1605	16.6434	16.1956	80.0149	82.5098
SP8	LMO2_2	cluster_1	SP8	3582	2410.243	2295	2529	71.2053	1.486	0.5714	0.001	0.0010435	3634	29090	57.111	541	4238	8.3202	453	3582	7.0324	12.4656	12.3135	83.7338	84.521
SP4	POU2F2_known5	cluster_4	SP4	10318	6949.827	6732	7171	129.0703	1.4846	0.57	0.001	0.0010909	3860	50256	61.466	935	11592	14.178	822	10318	12.62	21.2953	20.5309	87.9144	89.0097
SP8	PKNOX2_2	cluster_6	Common_2	42838	28867.359	28472	29348	266.625	1.4839	0.5694	0.001	0.0010435	10799	119886	49.272	5428	59074	24.279	3906	42838	17.606	36.17	35.7323	71.9602	72.5158
SP8	MYC_known13	cluster_1	SP8	9966	6718.494	6468	6936	140.8605	1.4833	0.5688	0.001	0.0010909	9016	99281	55.743	1134	12181	6.8392	915	9966	5.5955	10.1486	10.0382	80.6878	81.8159
SP4	ATF4_2	cluster_4	SP4	8702	5869.365	5645	6076	121.0477	1.4825	0.5681	0.001	0.0010909	3978	47794	58.841	871	10071	12.399	739	8702	10.713	18.5772	18.2073	84.845	86.4065
DP	E2F_known26	cluster_2	DP	3194	2154.117	2002	2290	88.4254	1.4825	0.568	0.001	0.0010435	1515	25773	59.594	237	3703	8.5622	200	3194	7.3853	13.2013	12.3928	84.3882	86.2544
SP8	IKZF2_3	cluster_1	SP8	7815	5271.737	5099	5430	104.8719	1.4823	0.5679	0.001	0.0010909	5533	55534	62.869	904	8622	9.7608	811	7815	8.8472	14.6575	14.0725	89.7124	90.6402
SP4	IKZF1_2	cluster_4	SP4	20248	13660.304	13385	13916	155.4524	1.4822	0.5678	0.001	0.0010435	20507	164063	59.864	2975	23108	8.4318	2584	20248	7.3882	12.6006	12.3416	86.8571	87.6233

DP	RREB1_1	cluster_2	DP	115516	77948.729	77097	78866	548.4424	1.4819	0.5675	0.001	0.001	45950	762192	60.431	8626	133890	10.616	7311	115516	9.1587	15.9108	15.1558	84.7554	86.2768
SP8	PKNOX1_2	cluster_6	Common_2	37083	25037.124	24637	25433	238.4274	1.4811	0.5667	0.001	0.0010435	9433	104620	49.524	4710	51072	24.176	3389	37083	17.554	35.9271	35.4454	71.9533	72.6093
SP8	CBX5_1	cluster_1	SP8	7797	5264.833	5084	5434	107.2777	1.4809	0.5664	0.001	0.0010435	4252	46829	61.603	838	8765	11.53	742	7797	10.257	17.4506	16.6499	88.5442	88.9561
SP8	SMAD3_3	cluster_1	SP8	4313	2912.364	2788	3058	81.1989	1.4808	0.5663	0.001	0.0010435	3767	33918	58.906	570	5013	8.7061	486	4313	7.4904	12.9015	12.716	85.2632	86.0363
SP4	SOX18_2	cluster_4	SP4	8960	6051.054	5845	6257	129.0443	1.4807	0.5662	0.001	0.0010909	2930	41032	62.484	766	10021	15.26	675	8960	13.644	23.0375	21.8366	88.1201	89.4122
DP	SPI1_known1	cluster_2	DP	42696	28845.529	28452	29277	240.5959	1.4801	0.5657	0.001	0.001	34618	243610	52.166	7958	55408	11.865	6092	42696	9.1428	17.5978	17.5264	76.5519	77.0575
SP4	TBX1_4	cluster_4	SP4	27534	18619.452	18127	19103	298.1796	1.4788	0.5644	0.001	0.0010909	8929	170354	58.182	1814	32620	11.141	1490	27534	9.4038	16.6872	16.1628	82.1389	84.4083
SP4	SOX8_9	cluster_4	SP4	11725	7933.531	7661	8188	150.7505	1.4778	0.5635	0.001	0.0010909	2995	47951	62.253	884	13207	17.146	769	11725	15.222	25.6761	24.452	86.991	88.7787
SP4	SOX17_5	cluster_4	SP4	8058	5455.556	5269	5651	121.2706	1.4769	0.5626	0.001	0.0011429	2416	33940	61.564	691	9164	16.623	599	8058	14.616	24.793	23.7419	86.686	87.931
SP4	SMAD3_1	cluster_4	SP4	27766	18800.569	18514	19086	178.4716	1.4768	0.5625	0.001	0.0010435	20754	171916	58.449	3964	32413	11.02	3407	27766	9.4401	16.4161	16.1509	85.9485	85.6632
DP	MAF_known11	cluster_2	DP	29653	20083.98	19615	20553	273.8877	1.4764	0.5621	0.001	0.0010435	10294	166060	54.212	2417	37391	12.207	1876	29653	9.6805	18.2242	17.8568	77.6169	79.3052
SP4	SOX2_6	cluster_4	SP4	8292	5619.004	5381	5838	136.6532	1.4756	0.5613	0.001	0.0010435	2099	33627	62.352	627	9364	17.363	541	8292	15.375	25.7742	24.6588	86.2839	88.5519
SP8	RUNX_1	cluster_6	Common_2	112564	76288.716	75588	77024	440.8892	1.4755	0.5612	0.001	0.0010435	23767	334786	52.302	10721	147133	22.986	8089	112564	17.585	34.0346	33.6227	75.4501	76.5049
DP	RREB1_2	cluster_2	DP	176324	119547.153	118276	120825	773.8301	1.4749	0.5606	0.001	0.001	48689	1172396	58.91	9551	211427	10.624	7747	176324	8.8599	15.9112	15.0396	81.1119	83.3971
SP4	SOX7_3	cluster_4	SP4	12094	8203.789	7929	8435	155.1684	1.4741	0.5599	0.001	0.0010909	3572	57193	62.928	905	13558	14.918	798	12094	13.307	22.3404	21.1459	88.1768	89.2019
DP	HESX1_1	cluster_6	Common_2	4542	3081.152	2948	3201	77.8505	1.474	0.5597	0.001	0.0012632	1124	10127	51.054	700	6114	30.823	511	4542	22.898	45.4626	44.8504	73	74.2885
SP4	SOX10_9	cluster_4	SP4	12227	8297.398	8050	8533	151.5094	1.4735	0.5593	0.001	0.0010435	3684	51623	61.408	1036	13825	16.446	901	12227	14.545	24.4571	23.6852	86.9691	88.4412
SP4	BPTF_1	cluster_4	SP4	30063	20409.647	20038	20775	231.9129	1.473	0.5587	0.001	0.0010909	12832	172114	61.355	2650	34033	12.132	2320	30063	10.717	18.0798	17.4669	87.5472	88.3349
SP4	SOX9_4	cluster_4	SP4	17199	11678.045	11407	11964	175.0122	1.4727	0.5585	0.001	0.0010909	5343	80252	62.068	1338	19227	14.871	1182	17199	13.302	22.1224	21.4312	88.3408	89.4523
SP4	SOX15_2	cluster_4	SP4	9476	6434.362	6225	6670	131.4494	1.4726	0.5584	0.001	0.0010909	3199	44866	62.621	809	10650	14.865	710	9476	13.226	22.1944	21.1207	87.7627	88.9765
SP4	POU2F3_1	cluster_4	SP4	12069	8199.494	7952	8481	153.9894	1.4719	0.5576	0.001	0.0010435	3852	58181	62.51	940	13469	14.471	833	12069	12.967	21.6251	20.7439	88.617	89.6058
SP4	GATA_known17	cluster_4	SP4	16086	10929.949	10630	11203	173.0211	1.4717	0.5575	0.001	0.0010909	4062	65192	61.257	1185	18187	17.089	1033	16086	15.115	25.4308	24.6748	87.173	88.4478
SP4	PBX3_known1	cluster_4	SP4	11620	7895.897	7686	8124	137.5127	1.4716	0.5574	0.001	0.0010435	7076	78591	58.439	1282	13739	10.216	1075	11620	8.6405	15.1922	14.7854	83.8534	84.5768
SP8	ELK4_2	cluster_1	SP8	6035	4103.429	3869	4328	132.3506	1.4706	0.5564	0.001	0.0011429	7080	64048	23.72	1952	17453	6.4637	672	6035	2.2351	9.4915	9.4226	34.4262	34.5786
SP4	TBX2_1	cluster_4	SP4	19592	13335.246	12958	13721	224.5846	1.4692	0.555	0.001	0.0010435	8640	147190	60.352	1365	22454	9.2067	1179	19592	8.0332	13.6458	13.3107	86.3736	87.2539
SP4	MAF_known11	cluster_4	SP4	24464	16652.603	16275	17034	232.9263	1.4691	0.5549	0.001	0.0010435	10831	174705	57.034	1919	29572	9.654	1556	24464	7.9865	14.3662	14.003	81.0839	82.7269
SP4	IKZF2_2	cluster_4	SP4	5849	3981.331	3833	4147	92.3667	1.469	0.5548	0.001	0.0010435	3698	36980	61.725	694	6615	11.041	612	5849	9.7628	16.5495	15.8167	88.1844	88.4203
SP4	SOX9_6	cluster_4	SP4	15275	10397.874	10121	10678	162.997	1.469	0.5548	0.001	0.0010909	4528	68048	61.63	1220	17382	15.743	1053	15275	13.834	23.2553	22.4474	86.3115	87.8783
DP	BATF_known1	cluster_2	DP	5808	3954.058	3762	4158	122.444	1.4688	0.5546	0.001	0.0010435	3507	45701	52.784	592	7502	8.6647	448	5808	6.7082	12.7745	12.7087	75.6757	77.4194
DP	CUX2_1	cluster_2	DP	726	493.968	426	570	44.4914	1.4688	0.5546	0.001	0.0012632	328	6022	56.539	51	888	8.3372	41	726	6.8163	12.5	12.0558	80.3922	81.7568
SP4	SOX10_4	cluster_4	SP4	17980	12243.632	11963	12566	181.6627	1.4685	0.5543	0.001	0.0010909	5739	86325	61.805	1406	20226	14.481	1234	17980	12.873	21.502	20.8283	87.7667	88.8955
DP	EWSR1::FLI1_1	cluster_2	DP	139645	95099.769	93985	96262	709.2274	1.4684	0.5542	0.001	0.0010435	43660	868688	53.282	9825	183050	11.228	7312	139645	8.5653	16.7476	16.0754	74.4224	76.2879
DP	SPZ1_2	cluster_2	DP	23601	16074.578	15758	16383	187.7462	1.4682	0.554	0.001	0.0010435	13790	138253	56.134	2953	28884	11.728	2388	23601	9.5825	17.3169	17.0709	80.8669	81.7096
SP4	ATF4_3	cluster_4	SP4	10069	6858.19	6625	7098	134.6458	1.4681	0.554	0.001	0.0010909	3989	51908	59.647	930	11616	13.348	790	10069	11.57	19.8045	19.3978	84.9462	86.6822
SP4	ETV7_1	cluster_4	SP4	50807	34606.678	34175	34987	253.1151	1.4681	0.554	0.001	0.001	27857	251236	56.588	6880	61259	13.798	5664	50807	11.444	20.3324	20.2228	82.3256	82.938
SP4	ARID3A_2	cluster_4	SP4	3267	2225.117	2092	2365	83.1331	1.468	0.5539	0.001	0.0013333	1263	20439	64.999	247	3631	11.547	220	3267	10.39	17.4188	15.9841	89.0688	89.9752
SP8	XBP1_2	cluster_1	SP8	6605	4502.972	4277	4752	139.3509	1.4667	0.5526	0.001	0.0010435	2655	61164	63.418	332	7249	7.5161	298	6605	6.8484	11.2241	10.7988	89.759	91.116
SP8	RUNX_2	cluster_6	Common_2	97372	66393.775	65647	67055	429.8951	1.4666	0.5525	0.001	0.0010435	19635	276608	53.223	9187	125839	24.213	7008	97372	18.736	35.6914	35.2022	76.2817	77.3782
SP4	SOX8_2	cluster_4	SP4	17211	11735.676	11472	12003	169.9098	1.4665	0.5524	0.001	0.0010909	5294	79524	62.195	1344	19312	15.104	1182	17211	13.461	22.3272	21.6425	87.9464	89.1208
SP4	POU2F2_known1	cluster_4	SP4	12378	8442.537	8146	8729	164.9077	1.4661	0.552	0.001	0.0010435	3339	60392	62.523	825	13977	14.47	723	12378	12.815	21.6532	20.4961	87.6364	88.5598
DP	IRF_known21	cluster_6	Common_2	41289	28167.842	27685	28669	295.1776	1.4658	0.5517	0.001	0.001	6273	90082	51.133	3962	55647	31.587	2908	41289	23.437	46.3574	45.8349	73.3973	74.1981
DP	FOXO1_5	cluster_2	DP	42646	29119.943	28673	29567	277.0136	1.4645	0.5504	0.001	0.0010435	23967	264888	55.536	4914	52812	11.072	3926	42646	8.941	16.3809	16.0996	79.8942	80.7506
DP	REL_1	cluster_2	DP	13277	9068.206	8856	9283	134.9318	1.4641	0.55	0.001	0.001	11260	101830	56.863	1831	16059	8.9675	1494	13277	7.414	13.2682	13.0384	81.5948	82.6764
SP4	MAF_known1	cluster_4	SP4	59786	40836.337	40223	41529	380.9218	1.464	0.5499	0.001	0.001	26418	399043	58.146	4884	71057	10.354	4039	59786	8.7116	15.2888	14.9823	82.6986	84.1381
SP4	SOX3_1	cluster_4	SP4	7955	5436.453	5241	5675	124.5113	1.4632	0.5491	0.001	0.0010909	2214	35441	64.162	600	8890	16.094	528	7955	14.402	23.8482	22.4458	88	89.4826
SP8	IKZF1_1	cluster_1	SP8	10129	6925.792	6684	7142	134.515	1.4624	0.5484	0.001	0.0010435	8739	105142	60.209	1030	11778	6.7446	875	10129	5.8003	10.0126	9.6336	84.9515	85.9993
DP	MESP1_1	cluster_2	DP	69601	47594.236	47109	48093	300.5535	1.4624	0.5483	0.001	0.001	32605	294479	56.054	9529	85097	16.198	7736	69601	13.248	23.7264	23.6353	81.1838	81.7902
SP4	SPIB_1	cluster_4	SP4	22519	15400.339	15144	15659	150.9714	1.4622	0.5482	0.001	0.0010435	18137	108822	58.346	4466	26536	14.228	3782	22519	12.074	20.8524	20.6934	84.6843	84.8621
SP4	GATA_known16	cluster_4	SP4	27525	18828.003	18341	19256	274.0044	1.4619	0.5478	0.001	0.0010435	5122	107965	62.558	1561	30959	17.939	1364	27525	15.949	26.6302	25.4944	87.3799	88.9079
SP4	SMAD_1	cluster_4	SP4	18303	12521.77	12274	12776	149.0304	1.4617	0.5476	0.001	0.0010435	16152	129249	59.226	2718	21297	9.7589	2322	18303	8.3869	14.3759	14.161	85.4305	85.9417
DP	TBX2_1	cluster_2	DP	22978	15728.213	15294	16115	245.2612	1.4609	0.5469	0.001	0.0010435	8226	140221	57.494	1691	27780	11.391	1379	22978	9.4216	16.7639	16.387	81.5494	82.7142
SP4	POU2F3_2	cluster_4	SP4	5952	4074.161	3936	4193	79.999	1.4608	0.5468	0.001	0.0010909	3575	28645	62.59	848	6599	14.419	760	5952	13.005	21.2587	20.7785	89.6226	90.1955
DP	ETV7_1	cluster_2	DP	42601	29163.574	28704	29607	281.8941	1.4607	0.5467	0.001	0.001	25386	228951	51.568	6344	56465	12.718	4739	42601	9.5953	18.6678	18.607	74.7005	75.4467
SP8	RBPJ_1	cluster_6	Common_2	32021	21922.576	21611	22204	179.2718	1.4606	0.5466	0.001	0.0010435	10911	76393	53.125	6050	41625	28.947	4615	32021	22.268	42.2968	41.9161	76.281	76.9273
DP	GATA_known17	cluster_6	Common_2	18767	12851.478	12488	13159	202.282	1.4603	0.5462	0.001	0.0010909	3215	51515	48.405	1780	27104	25.468	1200	18767	17.634	37.325	36.4302	67.4157	69.2407
DP	ARID3A_2	cluster_6	Common_2	6764	4632.952	4428	4834	125.3978	1.4599	0.5458	0.001	0.0013333	987	15963	50.765	641	9493	30.189	436	6764	21.511	44.1743	42.373	68.0187	71.2525
SP4	TCF7L2_known5	cluster_4	SP4	49759	34100.74	33552	34655	335.6052	1.4592	0.5451	0.001	0.0010435	11860	190547	60.832	3628	56623	18.077	3154	49759	15.886	26.5936	26.1138	86.935	87.8777
SP4	POU2F2_known15	cluster_4	SP4	7580	5195.155	5017	5359	104.567	1.459	0.5449	0.001	0.0010435	3260	36005	62.683	800	8497	14.793	710	7580	13.196	21.7791	21.0526	88.75	89.208
SP8	ARID3A_2	cluster_1	SP8	3401	2331.879	2187	2481	86.2103	1.4583	0.5443	0.001	0.0013333	1242	20101	63.924	256	3819	12.145	224	3401	10.816	18.0354	16.9196	87.5	89.0547
SP4	ATF3_known5	cluster_4	SP4	22476	15413.041	14989	15811	247.5492	1.4582	0.5442	0.001	0.001	11915	167390	58.274	1972	26814	9.3348	1627	22476	7.8246	13.6551	13.4273	82.5051	83.8219
SP4	SPI1_known1	cluster_4	SP4	50535	34658.506	34297	35020	224.404	1.4581	0.5441	0.001	0.001	38052	267768	57.339	8708	60564	12.969	7222	50535	10.821	18.9793	18.8727	82.9352	83.4407
SP4	MGA_3	cluster_4	SP4	13317	9134.665	8847	9411	179.1244	1.4578	0.5438	0.001	0.0010909	5104	86900	61.334	917	15111	10.665	802	13317	9.3991	15.7132	15.3245	87.4591	88.1279
SP4	IKZF3_1	cluster_4	SP4	9710	6661.521	6459	6844	118.3582	1.4576	0.5436	0.001	0.0010435	6633	79768	59.129	1001	11533	8.5489	835	9710	7.1976	12.5886	12.1728	83.4166	84.1932
SP4	SMAD3_3	cluster_4	SP4	5784	3967.99	3825	4098	83.2013	1.4575	0.5435	0.001	0.0010435	3934	35416	61.507	744	6513	11.311	659	5784	10.045	16.7514	16.3316	88.5753	88.807
SP4	SOX4_2	cluster_4	SP4	14913	10233.108	9971	10489	162.1951	1.4573	0.5433	0.001	0.0010909	4816	72432	63.066	1180	16678	14.521	1040	14913	12.985	21.5947	20.589	88.1356	89.4172
DP	E2F2_3	cluster_2	DP	4141	2842.027	2678	3023	98.921	1.4569	0.5429	0.001	0.001	1923	32730	61.531	304	4770	8.9673	259	4141	7.7849	13.4685	12.652	85.1974	86.8134
SP8	NFATC2_1	cluster_1	SP8	4603	3159.039	3054	3253	62.5045	1.4569	0.5429	0.001	0.0010909	5758	34548	61.898	877	5163	9.2504	776	4603	8.247	13.4769	13.3235	88.4835	89.1536
DN	FOXP1_1	cluster_3	DN	46225	31730.099	31101	32378	372.7272	1.4568	0.5428	0.001	0.0010435	10678	252594	65.084	3115	57864	14.909	2464	46225	11.91	23.0755	18.3001	79.1011	79.8856
DP	RBPJ_1	cluster_6	Common_2	30515	20970.331	20680	21276	184.3908	1.4551	0.5411	0.001	0.0010435	10434	73042	50.795	6050	41625	28.947	4401	30515	21.221	42.1794	41.7773	72.7438	73.3093
SP4	SOX21_3	cluster_4	SP4	11516	7919.127	7656	8172	161.4443	1.4541	0.5402	0.001	0.0010909	3663	54981	62.433	903	13016	14.78	793	11516	13.077	21.6489	20.9454	87.8184	88.4757
SP8	FOXO3_1	cluster_1	SP8	12189	8381.857	8152	8596	140.8508	1.4542	0.5402	0.001	0.0010435	5381	70700	64.693	1083	13447	12.304	969	12189	11.153	18.0078	17.2405	89.4737	90.6448
SP8	ATF3_known5	cluster_1	SP8	20910	14380.516	14030	14730	214.5196	1.454	0.54	0.001	0.001	11933	167611	58.351	1826	24906	8.6706	1516	20910	7.2794	12.7043	12.4753	83.023	83.9557
DN	SOX8_9	cluster_3	DN	11013	7574.514	7342	7793	137.2531	1.4539	0.5399	0.001	0.0010909	3303	52872	68.642	784	11527	14.965	737	11013	14.298	22.313	20.8296	94.0051	95.5409
SP8	PKNOX1_1	cluster_6	Common_2	68205	46928.435	46215	47462	361.3557	1.4534	0.5394	0.001	0.0010435	10991	165550	53.351	6051	88894	28.648	4583	68205	21.98	41.6978	41.199	75.7395	76.7262
DP	BATF_known1	cluster_6	Common_2	22672	15602.096	15238	15977	210.0461	1.4531	0.5391	0.001	0.0010435	3507	45701	52.784	2327	29701	34.304	1751	22672	26.186	49.9287	49.6094	75.2471	76.3341
SP4	HESX1_2	cluster_4	SP4	2239	1540.653	1442	1638	58.5433	1.453	0.539	0.001	0.0014118	906	12691	63.767	192	2490	12.511	171	2239	11.25	18.8742	17.6424	89.0625	89.9197
SP4	MYC_known13	cluster_6	Common_2	46858	32259.658	31807	32650	253.6355	1.4525	0.5386	0.001	0.0010909	9038	99518	55.876	5376	58122	32.633	4287	46858	26.309	47.4331	47.0849	79.7433	80.6201
SP4	PKNOX1_2	cluster_6	Common_2	38217	26317.306	25890	26746	254.8242	1.4521	0.5382	0.001	0.0010435	9911	109901	52.023	4710	51072	24.176	3495	38217	18.091	35.2638	34.774	74.2038	74.8297
SP8	BATF_known1	cluster_1	SP8	6718	4628.009	4439	4850	118.2314	1.4515	0.5375	0.001	0.0010435	3843	50078	57.839	638	8084	9.3369	524	6718	7.7592	13.6352	13.4151	82.1317	83.1024
DP	MYC_known13	cluster_2	DP	17574	12113.051	11855	12400	170.7109	1.4508	0.5368	0.001	0.0010909	9190	101216	56.829	1982	21373	12	1610	17574	9.8672	17.519	17.3629	81.2311	82.2252
SP8	MYC_known13	cluster_6	Common_2	46742	32219.088	31773	32635	253.7702	1.4507	0.5368	0.001	0.0010909	9016	99281	55.743	5376	58122	32.633	4278	46742	26.244	47.449	47.0805	79.5759	80.4205
SP4	PKNOX2_2	cluster_6	Common_2	44130	30420.402	30015	30858	253.2149	1.4507	0.5367	0.001	0.0010435	11364	126178	51.858	5428	59074	24.279	4021	44130	18.137	35.3837	34.9744	74.0789	74.7029
DP	IRF4_2	cluster_6	Common_2	36161	24931.997	24560	25379	242.4011	1.4504	0.5364	0.001	0.001	5446	77752	53.165	3385	47396	32.408	2565	36161	24.726	47.0988	46.5081	75.7755	76.2955
DP	REL_2	cluster_2	DP	13493	9303.647	9077	9525	138.2729	1.4502	0.5363	0.001	0.0010435	11409	103099	57.237	1867	16362	9.0836	1521	13493	7.4908	13.3316	13.0874	81.4676	82.4655
SP8	PKNOX2_3	cluster_6	Common_2	38235	26366.903	26001	26739	238.9179	1.4501	0.5361	0.001	0.0010435	9661	107482	50.165	4884	53142	24.803	3481	38235	17.845	36.0315	35.5734	71.2735	71.9487
DP	FOXO6_3	cluster_2	DP	43212	29802.218	29248	30318	306.8279	1.4499	0.536	0.001	0.0010435	21070	275513	56.268	4217	53359	10.898	3364	43212	8.8252	15.9658	15.6842	79.7724	80.9835
DP	PBX1_2	cluster_6	Common_2	14029	9684.566	9407	9964	168.8093	1.4485	0.5346	0.001	0.0010435	2395	33982	48.403	1495	20344	28.977	1005	14029	19.982	41.9624	41.2836	67.2241	68.9589
SP8	HMGA1_1	cluster_1	SP8	3297	2276.722	2194	2359	51.1784	1.4479	0.534	0.001	0.0010435	3543	21381	63.056	611	3646	10.753	551	3297	9.7234	15.5518	15.4202	90.18	90.4279
SP8	MTF1_3	cluster_1	SP8	16471	11388.531	11077	11726	191.0173	1.4462	0.5323	0.001	0.001	12742	167257	61.78	1462	18557	6.8544	1282	16471	6.0839	10.0612	9.8477	87.6881	88.759
SP8	SMAD4_1	cluster_1	SP8	37282	25815.19	25312	26292	295.3684	1.4442	0.5302	0.001	0.001	25393	360702	58.545	3229	44540	7.2292	2670	37282	6.0512	10.5147	10.336	82.6881	83.7045
SP4	MYC_known20	cluster_4	SP4	14966	10363.509	10023	10695	204.4091	1.4441	0.5301	0.001	0.001	8841	144867	57.464	1173	18285	7.253	938	14966	5.9365	10.6097	10.3309	79.9659	81.8485
SP4	IKZF1_1	cluster_4	SP4	11286	7820.131	7571	8047	143.2686	1.4431	0.5292	0.001	0.0010435	8843	106411	60.935	1153	13125	7.5159	984	11286	6.4628	11.1274	10.606	85.3426	85.9886
SP4	SPIC_2	cluster_4	SP4	44484	30825.85	30385	31327	284.1499	1.4431	0.5291	0.001	0.0010435	17268	225108	60.689	4012	51184	13.799	3458	44484	11.993	20.0255	19.7612	86.1914	86.91
SP4	POU2F2_known11	cluster_4	SP4	10404	7214.606	7026	7419	121.4305	1.442	0.5281	0.001	0.0010435	4718	47310	63.382	1193	11573	15.504	1066	10404	13.938	22.5943	21.9911	89.3546	89.8989
SP4	ETV2_1	cluster_4	SP4	15921	11041.442	10729	11400	202.1056	1.4419	0.528	0.001	0.0010435	7748	77780	26.389	4238	42029	14.259	1595	15921	5.4016	20.586	20.4693	37.6357	37.881
SP4	RUNX3_1	cluster_4	SP4	21527	14934.173	14596	15285	200.0189	1.4414	0.5275	0.001	0.0010435	18902	289786	59.972	1715	25185	5.2121	1447	21527	4.4551	7.6553	7.4286	84.3732	85.4755
DP	E2F_known24	cluster_2	DP	6073	4214.462	4024	4409	115.5048	1.4409	0.527	0.001	0.001	2757	47016	60.976	441	7068	9.1666	376	6073	7.8762	13.638	12.9169	85.2608	85.9225
SP4	RBPJ_1	cluster_6	Common_2	32615	22637.275	22348	22921	175.1491	1.4407	0.5268	0.001	0.0010435	11294	79074	54.99	6050	41625	28.947	4710	32615	22.681	41.7036	41.2462	77.8512	78.3544
DP	MAF_known12	cluster_2	DP	20210	14041.305	13654	14401	231.3677	1.4393	0.5254	0.001	0.0010435	7384	125901	57.08	1524	24862	11.272	1218	20210	9.1627	16.4951	16.0523	79.9213	81.2887
SP8	IRF_known14	cluster_1	SP8	40620	28224.409	27636	28801	358.3343	1.4392	0.5252	0.001	0.001	13279	278624	63.239	2296	45729	10.379	2001	40620	9.2195	15.0689	14.5788	87.1516	88.8277
SP4	LMO2_2	cluster_4	SP4	5211	3621.311	3485	3752	81.7765	1.4389	0.5249	0.001	0.0010435	3781	30272	59.431	783	6146	12.066	661	5211	10.23	17.4821	17.2139	84.4189	84.7869
SP8	FOXO4_2	cluster_1	SP8	12781	8884.703	8675	9116	134.0765	1.4385	0.5246	0.001	0.0010435	6205	81377	63.594	1152	14358	11.22	1014	12781	9.988	16.3417	15.7059	88.0208	89.0166
SP4	NFATC2_1	cluster_4	SP4	5708	3968.396	3853	4080	70.6682	1.4383	0.5243	0.001	0.0010909	5908	35448	63.511	1082	6319	11.322	973	5708	10.227	16.4692	16.1025	89.9261	90.3307
DP	RUNX3_1	cluster_2	DP	38476	26772.34	26293	27309	308.9973	1.4371	0.5232	0.001	0.0010435	18870	289545	59.922	3043	45082	9.3299	2564	38476	7.9627	13.5877	13.2884	84.259	85.3467
SP4	RREB1_2	cluster_4	SP4	155526	108222.014	107002	109473	734.2292	1.4371	0.5232	0.001	0.001	51020	1245776	62.597	7941	179955	9.0423	6659	155526	7.8148	13.0517	12.4843	83.8559	86.4249
SP4	RUNX3_1	cluster_6	Common_2	150579	104774.529	103969	105548	453.9058	1.4372	0.5232	0.001	0.0010435	18902	289786	59.972	11621	175566	36.334	9888	150579	31.163	52.3119	51.9621	85.0873	85.7677
SP4	TCF7L2_known7	cluster_4	SP4	48470	33731.536	33261	34212	284.3199	1.4369	0.523	0.001	0.0010435	12562	176542	61.995	4019	55081	19.343	3504	48470	17.021	27.8936	27.4552	87.1859	87.9977
DP	TCF12_known1	cluster_2	DP	49590	34517.23	34154	34911	241.6954	1.4367	0.5227	0.001	0.0010909	36358	261819	56.235	8606	61446	13.198	6926	49590	10.651	19.0495	18.9406	80.4787	80.705
SP4	RREB1_1	cluster_4	SP4	107601	74931.163	74113	75768	500.4802	1.436	0.522	0.001	0.001	47895	812464	64.416	7266	119347	9.4625	6422	107601	8.5312	13.4085	13.2438	88.3843	90.1581
DN	SPI1_known1	cluster_3	DN	24918	17354.303	16997	17704	223.4117	1.4358	0.5219	0.001	0.001	15535	109231	23.39	10728	74696	15.995	3586	24918	5.3359	23.0834	22.8122	33.4265	33.3592
DP	SOX14_3	cluster_6	Common_2	14680	10224.035	9940	10547	185.0462	1.4358	0.5218	0.001	0.0010909	2759	38663	47.284	1626	21909	26.794	1066	14680	17.953	38.6372	37.9691	65.5597	67.0044
SP4	TCF7_1	cluster_4	SP4	52204	36363.329	35739	36915	343.5544	1.4356	0.5217	0.001	0.0010435	11748	188705	61.707	3834	59721	19.529	3320	52204	17.071	28.2601	27.6643	86.5936	87.4131
SP4	TCF7L1_1	cluster_4	SP4	48133	33526.485	33017	34021	302.3374	1.4357	0.5217	0.001	0.0010435	10758	172842	61.696	3526	55052	19.651	3055	48133	17.181	28.3975	27.848	86.6421	87.4319
SP4	TCF7L1_2	cluster_4	SP4	34492	24038.471	23682	24421	238.9655	1.4348	0.5209	0.001	0.0010435	12702	140496	61.647	3643	39382	17.28	3160	34492	15.134	24.878	24.5502	86.7417	87.5832
SP8	RREB1_1	cluster_1	SP8	113911	79398.085	78629	80127	488.1145	1.4347	0.5207	0.001	0.001	48842	818149	64.867	7972	126523	10.031	7078	113911	9.0315	14.4916	13.923	88.7858	90.0319
SP4	SPIB_2	cluster_4	SP4	45685	31845.355	31313	32318	291.3341	1.4346	0.5206	0.001	0.0010435	18724	244697	60.687	4131	52835	13.103	3548	45685	11.33	18.9489	18.67	85.8872	86.4673
DP	MYBL2_2	cluster_2	DP	4519	3151.228	2995	3311	98.5769	1.4339	0.52	0.001	0.0010435	2149	32599	60.53	362	5258	9.7631	305	4519	8.3909	14.1926	13.8624	84.2541	85.9452
DP	CUX1_9	cluster_6	Common_2	3438	2398.166	2274	2549	86.7685	1.4334	0.5195	0.001	0.0012632	440	7588	51.249	296	4797	32.399	204	3438	23.22	46.3636	45.3084	68.9189	71.6698
DP	ATF3_known5	cluster_2	DP	25954	18109.726	17708	18579	263.4569	1.4331	0.5192	0.001	0.001	11264	158203	55.075	2441	33101	11.523	1885	25954	9.0354	16.7347	16.4055	77.2224	78.4085
SP4	NFATC1_1	cluster_4	SP4	17972	12540.621	12187	12898	217.1548	1.4331	0.5191	0.001	0.0010909	5359	103439	64.397	1121	20131	12.533	987	17972	11.189	18.4176	17.3745	88.0464	89.2752
DP	SOX8_9	cluster_6	Common_2	13950	9735.031	9418	10040	177.889	1.4329	0.519	0.001	0.0010909	2184	34944	45.366	1457	22004	28.567	897	13950	18.111	41.0714	39.921	61.5649	63.3976
SP8	MYC_known20	cluster_6	Common_2	76104	53144.479	52561	53750	354.0786	1.432	0.518	0.001	0.001	8781	143953	57.101	5818	93509	37.092	4663	76104	30.188	53.1033	52.8673	80.1478	81.3868
DP	E2F8_1	cluster_2	DP	10112	7065.247	6853	7288	126.7355	1.4312	0.5172	0.001	0.001	7146	78795	61.175	1101	11689	9.0751	937	10112	7.8507	13.1122	12.8333	85.1045	86.5087
SP4	SOX11_2	cluster_4	SP4	11669	8153.55	7911	8377	140.9489	1.4311	0.5171	0.001	0.0010909	3896	54587	64.334	979	13014	15.338	868	11669	13.753	22.2793	21.3769	88.6619	89.665
DN	POU2F2_known16	cluster_3	DN	8294	5796.549	5630	5956	103.7587	1.4308	0.5168	0.001	0.0010909	3024	39849	67.778	719	8853	15.058	666	8294	14.107	22.0238	20.8136	92.6287	93.6858

DP	E2F7_1	cluster_2	DP	6190	4326.398	4140	4509	110.6998	1.4307	0.5167	0.001	0.0010435	4150	54006	59.789	594	7377	8.1669	492	6190	6.8528	11.8554	11.4617	82.8283	83.9094
SP4	FOXP3_2	cluster_4	SP4	5648	3947.875	3840	4067	70.0441	1.4305	0.5166	0.001	0.0010909	4729	28374	62.407	1078	6365	13.999	952	5648	12.422	20.1311	19.9055	88.3117	88.7353
DN	ETV2_1	cluster_3	DN	11974	8376.215	8089	8669	180.6517	1.4295	0.5155	0.001	0.0010435	7018	70432	23.896	3573	35299	11.976	1209	11974	4.0625	17.2271	17.0008	33.8371	33.9216
DP	RUNX1_4	cluster_6	Common_2	8144	5698.475	5502	5891	119.4552	1.4291	0.5151	0.001	0.0010909	2351	23810	47.054	1246	12216	24.142	815	8144	16.095	34.6661	34.2041	65.4093	66.6667
SP4	RUNX2_4	cluster_6	Common_2	131962	92355.248	91612	93041	437.4683	1.4288	0.5149	0.001	0.0010435	16422	250904	60.883	10116	152450	36.993	8693	131962	32.021	52.9351	52.5946	85.9332	86.5608
SP4	PBX1_2	cluster_4	SP4	8663	6064.526	5868	6277	126.2047	1.4284	0.5144	0.001	0.0010435	3037	43176	61.498	746	10083	14.362	633	8663	12.339	20.8429	20.0644	84.8525	85.9169
SP4	RUNX1_2	cluster_4	SP4	16769	11739.822	11423	12009	171.2206	1.4283	0.5143	0.001	0.0010435	4574	71425	63.314	1297	19205	17.024	1119	16769	14.865	24.4644	23.4778	86.276	87.3158
DP	IKZF1_2	cluster_2	DP	24039	16831.009	16536	17105	176.3937	1.4282	0.5142	0.001	0.0010435	20144	161159	58.804	3711	28886	10.54	3064	24039	8.7715	15.2105	14.9163	82.5653	83.2202
SP4	POU2F2_known13	cluster_4	SP4	12041	8431.851	8151	8722	181.6593	1.428	0.514	0.001	0.0010909	2728	57442	64.703	704	13662	15.389	613	12041	13.563	22.4707	20.962	87.0739	88.135
SP4	TCF7_2	cluster_4	SP4	39142	27410.158	27039	27789	231.1589	1.428	0.514	0.001	0.0010435	13256	146350	61.849	4118	44691	18.887	3579	39142	16.542	26.9991	26.7455	86.9111	87.5836
DP	GATA_known16	cluster_6	Common_2	30078	21066.227	20582	21591	316.704	1.4278	0.5138	0.001	0.0010435	4004	84333	48.865	2227	44036	25.516	1474	30078	17.428	36.8132	35.6658	66.1877	68.3032
DP	MYBL2_4	cluster_2	DP	3100	2170.8	2049	2295	75.4527	1.4278	0.5138	0.001	0.0010435	1563	21943	61.958	263	3526	9.956	228	3100	8.7531	14.5873	14.1275	86.692	87.9183
SP8	HESX1_1	cluster_6	Common_2	5046	3534.026	3409	3659	75.5274	1.4277	0.5137	0.001	0.0012632	1301	11720	59.084	700	6114	30.823	568	5046	25.439	43.6587	43.0546	81.1429	82.5319
SP4	POU2F2_known12	cluster_4	SP4	10151	7110.34	6904	7303	116.3793	1.4276	0.5136	0.001	0.0010435	4643	46514	63.727	1183	11402	15.621	1046	10151	13.907	22.5285	21.8235	88.4193	89.0282
DP	ATF4_3	cluster_6	Common_2	18782	13157.339	12854	13481	180.9267	1.4275	0.5135	0.001	0.0010909	3413	44407	51.027	2058	25990	29.865	1460	18782	21.582	42.7776	42.2951	70.9427	72.2663
DP	PBX1_4	cluster_6	Common_2	10936	7662.448	7435	7906	140.6701	1.4272	0.5132	0.001	0.0010909	2319	25948	48.177	1474	16039	29.779	988	10936	20.304	42.6046	42.1458	67.0285	68.1838
SP4	MAF_known12	cluster_4	SP4	20072	14064.001	13700	14430	223.502	1.4272	0.5131	0.001	0.0010435	7957	135675	61.511	1414	23154	10.497	1208	20072	9.1001	15.1816	14.7942	85.4314	86.6891
SP4	PKNOX2_3	cluster_6	Common_2	39616	27765.878	27355	28197	255.0377	1.4268	0.5128	0.001	0.0010435	10156	113004	52.742	4884	53142	24.803	3605	39616	18.49	35.4963	35.0572	73.8124	74.5474
DP	CUX2_2	cluster_6	Common_2	1259	882.242	821	949	39.835	1.4266	0.5125	0.001	0.0013333	279	2511	51.763	207	1769	36.467	145	1259	25.953	51.9713	50.1394	70.0483	71.1702
DP	SMAD_1	cluster_2	DP	18875	13232.46	13003	13493	149.698	1.4264	0.5124	0.001	0.0010435	15462	123736	56.699	3011	23523	10.779	2398	18875	8.6491	15.509	15.2543	79.6413	80.2406
SP8	HES5_1	cluster_6	Common_2	55683	39042.967	38653	39478	258.6345	1.4262	0.5122	0.001	0.0010435	9348	103156	59.163	6066	66167	37.949	5069	55683	31.936	54.2255	53.9794	83.5641	84.1552
SP8	HES5_1	cluster_1	SP8	7795	5465.469	5284	5658	116.3053	1.4261	0.5121	0.001	0.0010435	9348	103156	59.163	867	9291	5.3287	716	7795	4.4707	7.6594	7.5565	82.5836	83.8984
SP4	SPIC_1	cluster_4	SP4	42144	29552.229	29086	29980	273.692	1.4261	0.512	0.001	0.001	16203	211420	60.042	3926	49711	14.118	3287	42144	11.969	20.2864	19.9338	83.7239	84.778
DP	NFKB_known10	cluster_2	DP	17989	12615.16	12331	12897	175.2677	1.4259	0.5119	0.001	0.001	10029	121099	60.099	1818	21243	10.542	1523	17989	8.9275	15.186	14.8548	83.7734	84.682
SP4	IKZF2_3	cluster_4	SP4	10895	7640.541	7452	7817	117.9046	1.4259	0.5119	0.001	0.0010909	5825	58444	66.163	1246	11893	13.464	1132	10895	12.334	19.4335	18.6418	90.8507	91.6085
SP4	SPI1_known4	cluster_4	SP4	37823	26551.383	26137	26991	253.7881	1.4245	0.5105	0.001	0.0010435	15528	202516	62.03	3409	43240	13.244	2961	37823	11.585	19.0688	18.6765	86.8583	87.4722
SP8	CUX1_8	cluster_1	SP8	1653	1160.227	1076	1240	52.2509	1.4244	0.5103	0.001	0.0011429	544	8813	66.353	122	1836	13.823	109	1653	12.445	20.0368	18.7564	89.3443	90.0327
SP4	SOX30_1	cluster_4	SP4	10834	7606.336	7356	7844	150.3873	1.4243	0.5102	0.001	0.0010435	3742	56229	63.32	872	12336	13.892	759	10834	12.2	20.2833	19.2676	87.0413	87.8243
DP	RBPJ_2	cluster_6	Common_2	42497	29840.524	29405	30246	242.9614	1.4241	0.5101	0.001	0.0010909	9585	95962	53.327	5759	56386	31.334	4291	42497	23.616	44.7679	44.2852	74.5095	75.368
DP	POU2F3_2	cluster_6	Common_2	9128	6409.823	6247	6574	102.6038	1.424	0.5099	0.001	0.0010909	2892	23182	50.653	1627	12784	27.933	1151	9128	19.945	39.7994	39.3754	70.7437	71.4018
DP	ATF4_2	cluster_6	Common_2	18194	12777.849	12485	13084	172.6725	1.4238	0.5098	0.001	0.0010909	3457	41526	51.125	2144	25111	30.915	1528	18194	22.4	44.2002	43.8135	71.2687	72.4543
DP	IRF_known14	cluster_6	Common_2	106597	74892.899	73969	75830	577.1623	1.4233	0.5093	0.001	0.001	11310	235509	53.453	7220	143449	32.558	5241	106597	24.194	46.3395	45.2624	72.59	74.31
DP	RUNX2_4	cluster_2	DP	32419	22780.929	22373	23232	261.861	1.4231	0.509	0.001	0.0010435	16438	251335	60.987	2540	37614	9.1272	2164	32419	7.8666	13.1646	12.8987	85.1969	86.1887
DP	NFATC1_3	cluster_6	Common_2	14868	10448.564	10208	10729	160.3812	1.4229	0.5089	0.001	0.0010909	2771	36045	53.14	1619	20119	29.661	1173	14868	21.92	42.3313	41.2484	72.4521	73.9003
SP4	FOXO4_2	cluster_4	SP4	16668	11714.961	11453	11961	152.92	1.4228	0.5087	0.001	0.0010435	6478	84929	66.37	1470	18241	14.255	1333	16668	13.026	20.5773	19.6258	90.6803	91.3766
SP4	SOX13_1	cluster_4	SP4	6776	4762.955	4583	4968	111.956	1.4226	0.5085	0.001	0.0010909	2170	32640	65.561	555	7626	15.318	485	6776	13.61	22.3502	20.7598	87.3874	88.8539
SP4	PKNOX1_1	cluster_6	Common_2	70305	49425.349	48762	50086	394.8118	1.4224	0.5084	0.001	0.0010435	11588	174615	56.273	6051	88894	28.648	4725	70305	22.657	40.7749	40.2629	78.0863	79.0886
SP8	TBX5_3	cluster_6	Common_2	52955	37229.759	36835	37654	258.1109	1.4224	0.5083	0.001	0.001	14898	134209	54.179	7844	69272	27.965	5935	52955	21.377	39.8376	39.4571	75.6629	76.445
SP4	RUNX2_5	cluster_6	Common_2	172608	121362.636	120410	122330	548.4555	1.4222	0.5082	0.001	0.001	17450	305925	60.5	11640	201282	39.805	9862	172608	34.135	56.5158	56.4217	84.7251	85.7543
DP	POU2F2_known12	cluster_6	Common_2	14718	10350.212	10085	10592	148.9518	1.422	0.5079	0.001	0.0010435	3757	37632	51.558	2101	20388	27.933	1494	14718	20.164	39.7658	39.1103	71.109	72.1895
SP4	NFKB_known10	cluster_4	SP4	16023	11271.154	10994	11551	177.5827	1.4216	0.5075	0.001	0.001	10445	126213	62.637	1529	18124	8.9945	1340	16023	7.9519	12.8291	12.6952	87.639	88.4076
SP8	RREB1_2	cluster_1	SP8	171008	120289.94	118927	121549	777.6795	1.4216	0.5075	0.001	0.001	51915	1257959	63.21	8721	197558	9.9268	7406	171008	8.5928	14.2656	13.5941	84.9215	86.5609
SP4	POU2F2_known10	cluster_4	SP4	9700	6823.748	6663	6967	101.1489	1.4214	0.5074	0.001	0.0010435	5130	46258	63.552	1251	10899	14.974	1103	9700	13.326	21.501	20.9693	88.1695	88.999
DP	POU2F2_known11	cluster_6	Common_2	14803	10417.853	10195	10663	139.3115	1.4209	0.5068	0.001	0.0010435	3816	38242	51.233	2129	20693	27.723	1498	14803	19.832	39.2558	38.7087	70.3617	71.5363
SP4	CBX5_1	cluster_4	SP4	10117	7120.588	6945	7311	116.5184	1.4208	0.5067	0.001	0.0010435	4490	49467	65.073	1068	11228	14.77	957	10117	13.309	21.314	20.452	89.6067	90.1051
SP8	MYC_known20	cluster_1	SP8	13244	9321.763	8989	9644	198.6452	1.4207	0.5066	0.001	0.001	8781	143953	57.101	1049	16496	6.5434	825	13244	5.2535	9.3953	9.2002	78.6463	80.2861
SP4	CUX1_8	cluster_4	SP4	1922	1352.697	1249	1457	64.19	1.4206	0.5065	0.001	0.0011429	545	8762	65.969	140	2170	16.338	124	1922	14.471	22.7523	21.9356	88.5714	88.5714
DP	IKZF1_1	cluster_2	DP	12795	9008.257	8741	9283	159.1185	1.4203	0.5062	0.001	0.0010435	8537	102703	58.812	1347	15540	8.8989	1088	12795	7.327	12.7445	12.4583	80.7721	82.3359
DP	TCF3_1	cluster_2	DP	143612	101151.298	100195	102013	554.6242	1.4198	0.5057	0.001	0.0011429	49855	709136	57.352	12668	177031	14.317	10173	143612	11.615	20.4052	20.2517	80.3047	81.1225
SP4	RUNX1_4	cluster_4	SP4	8608	6063.643	5902	6220	97.6588	1.4195	0.5054	0.001	0.0010909	3165	32119	63.475	998	9723	19.215	875	8608	17.012	27.6461	26.8003	87.6754	88.5323
DP	ARID5A_1	cluster_6	Common_2	3588	2527.738	2400	2651	79.1604	1.4193	0.5052	0.001	0.0012632	647	8899	52.495	410	5064	29.873	278	3588	21.166	42.9675	40.3191	67.8049	70.8531
SP4	TBX15_2	cluster_6	Common_2	42185	29721.426	29384	30013	200.0291	1.4193	0.5052	0.001	0.0010909	14978	104871	54.579	7932	54774	28.506	6065	42185	21.955	40.4927	40.2256	76.4624	77.0165
SP4	HBP1_1	cluster_4	SP4	13195	9304.265	9045	9585	160.7828	1.4181	0.504	0.001	0.0010435	4429	68253	63.399	1056	15222	14.139	901	13195	12.257	20.3432	19.3325	85.322	86.6837
SP8	HES7_1	cluster_6	Common_2	59345	41846.085	41456	42301	249.5961	1.4182	0.504	0.001	0.0010435	9675	106849	60.312	6360	69516	39.239	5392	59345	33.498	55.7313	55.541	84.7799	85.3688
SP4	SOX_1	cluster_4	SP4	17864	12601.588	12351	12857	151.0195	1.4176	0.5034	0.001	0.0010435	7156	85938	62.408	1772	20522	14.903	1524	17864	12.973	21.2968	20.7871	86.0045	87.048
SP8	REL_2	cluster_1	SP8	14595	10300.212	10079	10528	135.5993	1.4169	0.5028	0.001	0.0010435	12473	112795	62.62	1862	16571	9.1996	1629	14595	8.1026	13.0602	12.9394	87.4866	88.0756
SP8	RBPJ_2	cluster_6	Common_2	44378	31324.04	30902	31787	252.542	1.4167	0.5026	0.001	0.0010909	10072	100834	56.034	5759	56386	31.334	4480	44378	24.661	44.4797	44.0109	77.7913	78.7039
DP	RUNX3_1	cluster_6	Common_2	148276	104668.457	104022	105463	448.5987	1.4166	0.5025	0.001	0.0010435	18870	289545	59.922	11621	175566	36.334	9725	148276	30.686	51.5368	51.21	83.6847	84.456
DP	SOX2_5	cluster_6	Common_2	16669	11766.176	11427	12078	200.8105	1.4167	0.5025	0.001	0.0010909	2721	43557	49.369	1609	24458	27.721	1070	16669	18.893	39.3238	38.2694	66.5009	68.1536
SP4	RUNX2_4	cluster_4	SP4	18566	13108.424	12737	13468	231.6015	1.4163	0.5021	0.001	0.0010435	16422	250904	60.883	1481	21763	5.2809	1250	18566	4.5051	7.6117	7.3996	84.4024	85.3099
DN	SOX15_4	cluster_3	DN	8915	6295.751	6086	6485	120.2181	1.416	0.5018	0.001	0.0011429	2731	38267	69.358	715	9431	17.094	668	8915	16.158	24.4599	23.2968	93.4266	94.5287
DP	SOX15_2	cluster_6	Common_2	13492	9528.022	9224	9810	176.6861	1.416	0.5018	0.001	0.0010909	2513	35262	49.216	1502	19922	27.806	993	13492	18.831	39.5145	38.2622	66.1119	67.7241
SP8	MAFG_1	cluster_6	Common_2	48467	34236.988	33602	34882	396.4434	1.4156	0.5014	0.001	0.0010435	6025	120737	52.864	3395	65678	28.757	2453	48467	21.221	40.7137	40.1426	72.2533	73.7949
SP4	MYC_known20	cluster_6	Common_2	75648	53440.992	52906	54076	372.7625	1.4155	0.5013	0.001	0.001	8841	144867	57.464	5818	93509	37.092	4632	75648	30.007	52.3923	52.2189	79.615	80.8992
SP8	MYBL2_2	cluster_1	SP8	4075	2879.022	2737	3048	90.2399	1.4153	0.5011	0.001	0.0010435	2285	34653	64.344	315	4541	8.4317	279	4075	7.5665	12.2101	11.7594	88.5714	89.7379
DP	SPIB_1	cluster_6	Common_2	35833	25328.641	25012	25645	192.9984	1.4147	0.5005	0.001	0.0010435	15545	93270	50.008	8514	50762	27.217	5999	35833	19.212	38.5912	38.4186	70.4604	70.5902
SP4	RBPJ_2	cluster_6	Common_2	45331	32042.861	31654	32440	229.6287	1.4147	0.5005	0.001	0.0010909	10316	103270	57.388	5759	56386	31.334	4578	45331	25.191	44.3777	43.8956	79.493	80.3941
DN	SOX2_6	cluster_3	DN	7950	5619.616	5394	5808	123.3598	1.4146	0.5004	0.001	0.0010435	2362	37807	70.103	562	8388	15.553	528	7950	14.741	22.3539	21.0279	93.9502	94.7783
SP4	SOX5_1	cluster_4	SP4	4804	3397.162	3275	3508	71.4251	1.414	0.4998	0.001	0.0010909	2386	21481	63.838	618	5390	16.018	547	4804	14.277	22.9254	22.3639	88.5113	89.128
SP4	HES5_1	cluster_6	Common_2	55345	39143.3	38708	39548	262.9722	1.4139	0.4997	0.001	0.0010435	9376	103478	59.348	6066	66167	37.949	5041	55345	31.742	53.7649	53.4848	83.1025	83.6444
SP8	MAFF_1	cluster_6	Common_2	54874	38811.658	38279	39328	314.8314	1.4138	0.4996	0.001	0.001	9420	132144	52.834	5394	73858	29.53	3940	54874	21.94	41.8259	41.5259	73.0441	74.2966
SP8	TBX15_2	cluster_6	Common_2	42011	29721.757	29383	30036	191.2975	1.4135	0.4992	0.001	0.0010909	14966	104782	54.532	7932	54774	28.506	6041	42011	21.864	40.3648	40.0937	76.1599	76.6988
SP8	EWSR1::FLI1_1	cluster_6	Common_2	409771	289928.65	288262	291660	1042.814	1.4133	0.4991	0.001	0.0010435	45443	904295	55.466	28139	535783	32.863	21165	409771	25.134	46.5748	45.3139	75.2159	76.4808
SP8	CUX1_9	cluster_1	SP8	1466	1037.049	947	1130	55.1278	1.4132	0.499	0.001	0.0012632	577	9969	67.331	102	1619	10.935	93	1466	9.9014	16.1179	14.7056	91.1765	90.5497
SP8	SPIB_1	cluster_6	Common_2	37866	26793.765	26469	27122	192.0451	1.4132	0.499	0.001	0.0010435	16443	98658	52.897	8514	50762	27.217	6338	37866	20.302	38.5453	38.3811	74.4421	74.5952
DP	SOX1_3	cluster_6	Common_2	12975	9183.048	8883	9485	187.7117	1.4129	0.4986	0.001	0.0010909	2535	35523	47.207	1464	19722	26.209	942	12975	17.243	37.1598	36.5256	64.3443	65.7895
DP	LMO2_2	cluster_6	Common_2	12186	8628.857	8422	8821	117.1652	1.4122	0.4979	0.001	0.0010435	3407	27268	53.534	2056	16178	31.761	1533	12186	23.924	44.9956	44.6897	74.5623	75.3245
DP	NFKB_known11	cluster_2	DP	19686	13941.084	13603	14261	211.0973	1.4121	0.4978	0.001	0.001	11257	136572	59.553	1997	23563	10.275	1651	19686	8.5842	14.6664	14.4144	82.674	83.5462
DP	POU2F2_known15	cluster_6	Common_2	11644	8245.619	8037	8478	139.3864	1.4121	0.4978	0.001	0.0010435	2667	29451	51.273	1524	16326	28.423	1072	11644	20.272	40.195	39.5369	70.3412	71.3218
DP	IRF6_1	cluster_6	Common_2	19929	14113.867	13829	14416	182.0202	1.412	0.4977	0.001	0.0010909	2388	38278	58.421	1569	24456	37.325	1262	19929	30.416	52.8476	52.0638	80.4334	81.4892
SP4	EWSR1::FLI1_1	cluster_4	SP4	141406	100156.248	99250	101202	600.8617	1.4118	0.4976	0.001	0.0010435	47664	952228	58.406	9280	176015	10.796	7318	141406	8.6734	15.3533	14.85	78.8578	80.3375
SP4	SOX5_3	cluster_4	SP4	8568	6070.102	5870	6279	126.439	1.4114	0.4972	0.001	0.0011429	2611	39250	66.643	690	9567	16.244	608	8568	14.548	23.2861	21.8293	88.1159	89.5579
SP8	REL_1	cluster_1	SP8	14833	10508.716	10284	10727	136.8576	1.4115	0.4972	0.001	0.001	12386	112079	62.586	1898	16902	9.4382	1654	14833	8.2828	13.3538	13.2344	87.1444	87.7588
SP4	SOX11_1	cluster_4	SP4	40964	29024.403	28498	29529	309.8266	1.4113	0.4971	0.001	0.0010435	11736	190304	65.17	3012	45946	15.734	2648	40964	14.028	22.5631	21.5256	87.915	89.1568
DP	MYC_known20	cluster_2	DP	22907	16230.659	15790	16669	270.7552	1.4113	0.497	0.001	0.001	8735	143000	56.723	1812	28806	11.426	1414	22907	9.0864	16.1878	16.0189	78.0353	79.5216
SP4	PBX1_4	cluster_4	SP4	6883	4877.236	4689	5051	103.9525	1.4112	0.4969	0.001	0.0010909	2946	33026	61.318	750	8086	15.013	628	6883	12.779	21.317	20.8412	83.7333	85.1224
DP	ATF7_1	cluster_6	Common_2	20886	14802.884	14527	15106	185.5983	1.4109	0.4966	0.001	0.001	3062	39860	54.566	2132	27214	37.254	1613	20886	28.592	52.678	52.3984	75.6567	76.7473
DP	SPIB_2	cluster_6	Common_2	87174	61788.227	61222	62398	371.5399	1.4108	0.4966	0.001	0.0010435	16381	214053	53.087	9156	117165	29.058	6736	87174	21.62	41.1208	40.7254	73.5692	74.4028
SP4	TBX5_3	cluster_6	Common_2	54195	38412.769	38031	38825	251.4047	1.4108	0.4966	0.001	0.001	15391	138670	55.98	7844	69272	27.965	6079	54195	21.878	39.4971	39.082	77.4987	78.2351
SP8	ETV7_1	cluster_6	Common_2	90381	64061.353	63465	64660	355.7779	1.4108	0.4966	0.001	0.001	25471	229674	51.731	13931	124176	27.969	10067	90381	20.357	39.5234	39.3519	72.2633	72.7846
SP4	MTF1_3	cluster_4	SP4	20456	14502.289	14124	14778	197.0982	1.4105	0.4962	0.001	0.001	12953	170029	62.803	1830	23295	8.6045	1587	20456	7.5558	12.252	12.0309	86.7213	87.8128
DP	SOX7_4	cluster_6	Common_2	13261	9403.44	9075	9723	187.1388	1.4102	0.4959	0.001	0.0010909	2231	35768	47.798	1326	20183	26.971	850	13261	17.721	38.0995	37.075	64.1026	65.7038
SP8	SPI1_known1	cluster_6	Common_2	98444	69808.332	69305	70325	311.5685	1.4102	0.4959	0.001	0.001	35122	247084	52.91	18984	132280	28.326	14059	98444	21.081	40.029	39.8423	74.0571	74.4209
DP	SOX18_2	cluster_6	Common_2	12148	8615.013	8329	8890	165.1113	1.41	0.4957	0.001	0.0010909	2288	32032	48.779	1357	18124	27.599	892	12148	18.499	38.986	37.9246	65.7332	67.0271
SP4	SOX4_1	cluster_4	SP4	43659	30965.881	30508	31456	294.3349	1.4099	0.4956	0.001	0.0010435	12657	205161	65.153	3207	48978	15.554	2824	43659	13.865	22.3118	21.2804	88.0574	89.14
SP8	NFE2L1::MAFG_1	cluster_6	Common_2	66904	47450.972	46676	48172	424.908	1.41	0.4956	0.001	0.0010435	8430	177761	54.694	4408	88615	27.265	3240	66904	20.585	38.4342	37.6371	73.5027	75.4996
DP	RUNX1_2	cluster_6	Common_2	20415	14481.615	14102	14838	229.2604	1.4097	0.4954	0.001	0.0010435	3573	55648	49.329	2063	30260	26.824	1362	20415	18.097	38.1192	36.686	66.0204	67.4653
SP4	SOX12_1	cluster_4	SP4	9439	6695.867	6481	6874	122.1055	1.4096	0.4953	0.001	0.0010435	3166	41308	65.459	869	10644	16.867	761	9439	14.958	24.0366	22.8503	87.5719	88.6791
SP4	TBX4_1	cluster_6	Common_2	33111	23490.951	23191	23778	176.3192	1.4095	0.4952	0.001	0.0010435	11905	83358	55.556	6158	42515	28.335	4762	33111	22.068	40	39.7214	77.3303	77.8807
SP8	TBX4_2	cluster_6	Common_2	52617	37337.311	36764	37911	353.9464	1.4092	0.4949	0.001	0.0010909	6718	127933	57.269	3636	66282	29.671	2818	52617	23.554	41.947	41.1286	77.5028	79.3835
DP	SOX_1	cluster_6	Common_2	27867	19776.44	19409	20130	216.4259	1.4091	0.4948	0.001	0.0010435	5830	70005	50.837	3385	39308	28.545	2355	27867	20.237	40.3945	39.8072	69.5716	70.894
SP4	FOXP1_1	cluster_4	SP4	47989	34054.901	33394	34702	386.2062	1.4092	0.4948	0.001	0.0010435	11002	256998	66.218	3085	58974	15.195	2450	47989	12.365	22.2687	18.6729	79.4165	81.3731
DP	POU2F2_known5	cluster_6	Common_2	15962	11334.037	11053	11612	170.3241	1.4083	0.4939	0.001	0.0010909	3181	41440	50.684	1820	22745	27.819	1249	15962	19.523	39.2644	38.5183	68.6264	70.1781
SP4	SMAD4_1	cluster_4	SP4	36691	26061.711	25608	26506	273.1397	1.4078	0.4935	0.001	0.001	25162	357474	58.021	3269	45282	7.3496	2610	36691	5.9553	10.3728	10.264	79.8409	81.0278
DP	ARID5B_1	cluster_6	Common_2	21826	15507.485	15196	15856	206.8104	1.4074	0.4931	0.001	0.0010909	4087	53311	52.552	2368	29918	29.492	1696	21826	21.515	41.4974	40.9409	71.6216	72.9527
SP8	ETV6_1	cluster_1	SP8	30872	21934.411	21484	22388	264.0936	1.4075	0.4931	0.001	0.001	26153	376727	62.445	2565	35529	5.8892	2201	30872	5.1173	8.4159	8.1948	85.809	86.8924
DP	POU2F2_known16	cluster_6	Common_2	10606	7535.81	7251	7801	160.5425	1.4074	0.493	0.001	0.0010909	2099	27573	46.898	1305	16413	27.917	825	10606	18.04	39.3044	38.4652	63.2184	64.6195
DN	SOX3_3	cluster_3	DN	13318	9464.475	9217	9700	144.7774	1.4071	0.4927	0.001	0.0010909	3414	54700	70.442	920	13934	17.944	869	13318	17.151	25.454	24.3473	94.4565	95.5792
DP	MYBL1_3	cluster_6	Common_2	7171	5096.325	4927	5244	94.4084	1.407	0.4926	0.001	0.0010909	1344	14803	55.839	844	9130	34.44	660	7171	27.05	49.1071	48.4429	78.1991	78.5433
SP8	ATF4_2	cluster_6	Common_2	19792	14068.309	13787	14329	168.8734	1.4068	0.4924	0.001	0.0010909	3830	46012	56.648	2144	25111	30.915	1661	19792	24.367	43.3681	43.0149	77.472	78.818
SP8	CUX2_1	cluster_1	SP8	1394	990.755	921	1061	45.7509	1.4066	0.4922	0.001	0.0012632	434	7992	75.035	91	1512	14.196	84	1394	13.088	19.3548	17.4424	92.3077	92.1958
DP	POU2F2_known1	cluster_6	Common_2	19448	13826.664	13455	14213	230.2343	1.4065	0.4921	0.001	0.0010435	2722	49255	50.993	1610	27604	28.578	1095	19448	20.134	40.2278	39.4843	68.0124	70.4536
SP4	ELK4_2	cluster_4	SP4	10559	7516.987	7236	7812	167.6422	1.4046	0.4902	0.001	0.0011429	7834	70855	26.241	3236	28879	10.695	1179	10559	3.9105	15.0498	14.9023	36.4339	36.5629
SP4	ARID5A_1	cluster_4	SP4	1968	1401.1	1309	1492	55.0954	1.4043	0.4899	0.001	0.0012632	810	11102	65.491	181	2239	13.208	157	1968	11.609	19.3827	17.7265	86.7403	87.8964
SP8	TBX4_1	cluster_6	Common_2	32674	23269.264	23007	23558	168.4848	1.4042	0.4897	0.001	0.0010435	11799	82621	55.065	6158	42515	28.335	4700	32674	21.776	39.8339	39.5468	76.3235	76.8529
DP	RUNX2_4	cluster_6	Common_2	130037	92617.333	91916	93336	456.3193	1.404	0.4896	0.001	0.0010435	16438	251335	60.987	10116	152450	36.993	8559	130037	31.554	52.0684	51.7385	84.6085	85.2981
SP8	HES7_1	cluster_1	SP8	7634	5437.113	5246	5611	111.1658	1.404	0.4895	0.001	0.0010435	9675	106849	60.312	845	9092	5.1321	703	7634	4.3091	7.2661	7.1447	83.1953	83.9639
DP	BPTF_1	cluster_6	Common_2	57535	40995.262	40479	41563	339.861	1.4034	0.489	0.001	0.0010909	10963	146228	52.127	6263	80341	28.64	4421	57535	20.51	40.3266	39.3461	70.5892	71.6135
DP	REL_1	cluster_6	Common_2	51351	36595.884	36228	36970	229.2942	1.4032	0.4887	0.001	0.001	11260	101830	56.863	7263	64662	36.108	5718	51351	28.675	50.7815	50.4282	78.7278	79.4145
DP	SOX10_9	cluster_6	Common_2	15205	10835.892	10543	11163	181.9022	1.4032	0.4887	0.001	0.0010435	2814	39418	46.89	1761	23524	27.983	1111	15205	18.087	39.4812	38.5737	63.0892	64.6361
DP	POU2F3_1	cluster_6	Common_2	18544	13216.099	12895	13566	207.8554	1.4031	0.4886	0.001	0.0010435	3087	46662	50.134	1853	26777	28.77	1248	18544	19.924	40.4276	39.7411	67.3502	69.2535
SP8	IKZF1_1	cluster_6	Common_2	52478	37407	36919	37823	260.2276	1.4029	0.4884	0.001	0.0010435	8739	105142	60.209	5426	63254	36.222	4446	52478	30.051	50.8754	49.9115	81.9388	82.9639
DP	SOX17_5	cluster_6	Common_2	9126	6507.95	6281	6740	148.4719	1.4022	0.4877	0.001	0.0011429	1842	25857	46.902	1054	14122	25.616	664	9126	16.554	36.0478	35.2941	62.9981	64.6226
SP8	GATA_known17	cluster_6	Common_2	20605	14696.402	14346	15036	213.2598	1.402	0.4875	0.001	0.0010909	3680	58991	55.43	1780	27104	25.468	1317	20605	19.361	35.788	34.9291	73.9888	76.022
SP8	IKZF3_1	cluster_6	Common_2	37613	26834.303	26444	27206	231.996	1.4017	0.4871	0.001	0.0010435	6473	77854	57.71	4061	47307	35.067	3191	37613	27.881	49.2971	48.3122	78.5767	79.5083

DP	RUNX2_5	cluster_2	DP	40162	28655.087	28122	29189	326.9685	1.4016	0.487	0.001	0.001	17640	310092	61.324	2750	46946	9.284	2329	40162	7.9424	13.2029	12.9516	84.6909	85.5494
DP	REL_2	cluster_6	Common_2	52020	37117.672	36746	37548	249.1903	1.4015	0.4869	0.001	0.0010435	11409	103099	57.237	7335	65260	36.23	5799	52020	28.88	50.8283	50.4564	79.0593	79.7119
SP4	HES7_1	cluster_6	Common_2	58942	42060.258	41611	42479	258.1073	1.4014	0.4868	0.001	0.0010435	9737	107540	60.702	6360	69516	39.239	5360	58942	33.27	55.0478	54.8094	84.2767	84.7891
SP8	SPIB_2	cluster_6	Common_2	91041	64985.644	64392	65634	391.4083	1.4009	0.4864	0.001	0.0010435	17242	225293	55.874	9156	117165	29.058	7042	91041	22.579	40.8421	40.41	76.9113	77.7032
SP8	E2F8_1	cluster_1	SP8	9810	7003.26	6816	7206	119.4503	1.4007	0.4862	0.001	0.001	7551	83269	64.648	1040	11009	8.5472	916	9810	7.6163	12.1308	11.7811	88.0769	89.1089
SP4	E2F7_1	cluster_4	SP4	7432	5305.822	5141	5473	101.9845	1.4006	0.4861	0.001	0.0010435	4567	59426	65.789	669	8269	9.1544	597	7432	8.2278	13.072	12.5063	89.2377	89.8779
DN	ETV7_1	cluster_3	DN	58675	41896.871	41525	42273	237.9017	1.4005	0.4859	0.001	0.001	30877	278376	62.701	7508	66847	15.056	6536	58675	13.216	21.1679	21.0776	87.0538	87.7751
DP	ATF7_1	cluster_2	DP	4884	3487.203	3297	3668	111.0315	1.4004	0.4859	0.001	0.001	3062	39860	54.566	503	6389	8.7462	380	4884	6.6859	12.4102	12.2529	75.5467	76.4439
SP8	TBX5_5	cluster_6	Common_2	43442	31025.594	30427	31578	355.8734	1.4002	0.4856	0.001	0.0010435	5748	109382	56.634	3074	55701	28.84	2329	43442	22.493	40.5184	39.7159	75.7645	77.9914
DP	TCF7_1	cluster_6	Common_2	45870	32763.937	32181	33388	363.7821	1.4	0.4854	0.001	0.0010435	9326	149704	48.954	4400	67875	22.195	2917	45870	15	31.2781	30.6405	66.2955	67.5801
SP4	E2F8_1	cluster_4	SP4	9192	6565.615	6366	6766	118.5623	1.4	0.4854	0.001	0.001	7471	82374	63.953	989	10443	8.1077	864	9192	7.1365	11.5647	11.1589	87.361	88.0207
DN	SOX3_1	cluster_3	DN	8899	6357.052	6140	6549	121.739	1.3998	0.4852	0.001	0.0010909	2538	40630	73.556	615	9153	16.57	591	8899	16.111	23.2861	21.9025	96.0976	97.225
DP	TCF7L1_1	cluster_6	Common_2	41565	29695.879	29154	30344	354.1024	1.3997	0.4851	0.001	0.0010435	8494	136288	48.648	4005	61781	22.053	2638	41565	14.837	31.0572	30.4979	65.8677	67.278
SP8	MAF_known11	cluster_6	Common_2	72909	52090.388	51534	52714	375.2024	1.3997	0.4851	0.001	0.0010435	10315	166406	54.325	6133	96460	31.49	4568	72909	23.802	44.285	43.8139	74.4823	75.5847
DP	RUNX2_5	cluster_6	Common_2	172226	123063.928	122104	123962	534.7543	1.3995	0.4849	0.001	0.001	17640	310092	61.324	11640	201282	39.805	9829	172226	34.059	55.72	55.5403	84.4416	85.5645
SP4	SMAD4_1	cluster_6	Common_2	179360	128168.577	127229	129084	532.9501	1.3994	0.4848	0.001	0.001	25162	357474	58.021	15994	222382	36.094	12740	179360	29.112	50.6319	50.1743	79.6549	80.654
SP4	FOXO6_3	cluster_6	Common_2	130041	92938.76	92309	93687	432.1484	1.3992	0.4846	0.001	0.0010435	21804	285200	58.247	12666	161340	32.951	10067	130041	26.558	46.1704	45.5964	79.4805	80.6006
SP8	SMAD4_1	cluster_6	Common_2	180956	129325.355	128406	130143	553.5273	1.3992	0.4846	0.001	0.001	25393	360702	58.545	15994	222382	36.094	12851	180956	29.371	50.6084	50.1677	80.3489	81.3717
DP	TCF7L2_known5	cluster_6	Common_2	50817	36331.101	35782	36966	371.7261	1.3987	0.4841	0.001	0.0010435	9748	156401	49.931	4751	73608	23.499	3221	50817	16.223	33.0427	32.4915	67.7963	69.0373
DP	SPIC_2	cluster_6	Common_2	76882	54976.304	54256	55689	424.495	1.3984	0.4838	0.001	0.0010435	14989	195470	52.699	8230	104958	28.297	5951	76882	20.727	39.7024	39.3319	72.3086	73.2503
SP8	SPZ1_2	cluster_6	Common_2	62043	44372.948	43955	44805	264.3626	1.3982	0.4836	0.001	0.0010435	13891	139218	56.526	8044	79031	32.088	6248	62043	25.191	44.9788	44.5654	77.6728	78.5046
SP4	SPDEF_4	cluster_4	SP4	25660	18356.705	17961	18709	242.401	1.3978	0.4832	0.001	0.0010435	7657	115318	63.966	2070	29469	16.346	1778	25660	14.233	23.2206	22.2515	85.8937	87.0746
DP	SOX11_2	cluster_6	Common_2	16776	12005.559	11714	12318	186.9291	1.3973	0.4827	0.001	0.0010909	3067	42939	50.606	1822	24297	28.635	1231	16776	19.771	40.1369	39.0694	67.5631	69.0456
DP	HMGA1_2	cluster_6	Common_2	27198	19465.457	19138	19862	218.7657	1.3972	0.4826	0.001	0.0010435	4747	66572	54.16	2777	36941	30.053	2002	27198	22.127	42.174	40.855	72.0922	73.6255
DP	SOX3_3	cluster_6	Common_2	13080	9362.159	9045	9745	199.0128	1.3971	0.4824	0.001	0.0010909	2264	36279	46.72	1342	20452	26.338	837	13080	16.844	36.97	36.0539	62.3696	63.9546
SP8	ETS_known17	cluster_6	Common_2	118430	84783.667	84019	85543	459.6676	1.3968	0.4822	0.001	0.001	12384	211645	59.804	8506	142533	40.275	6978	118430	33.464	56.3469	55.9569	82.0362	83.0895
DN	CUX2_1	cluster_3	DN	1251	895.344	813	970	47.1941	1.3968	0.4821	0.001	0.0012632	444	7998	75.092	86	1315	12.346	80	1251	11.745	18.018	15.6414	93.0233	95.1331
SP4	KLF12_1	cluster_4	SP4	9921	7103.627	6956	7248	93.9575	1.3966	0.4819	0.001	0.001	19302	115812	58.735	2058	12154	6.164	1666	9921	5.0315	8.6312	8.5665	80.9524	81.6274
DP	HMGA1_1	cluster_6	Common_2	7835	5611.092	5484	5751	81.4836	1.3963	0.4816	0.001	0.0010435	3004	18138	53.492	1786	10593	31.24	1312	7835	23.107	43.6751	43.1966	73.4602	73.9639
DP	SPI1_known4	cluster_6	Common_2	69835	50014.699	49352	50570	365.9237	1.3963	0.4816	0.001	0.0010435	13368	174316	53.393	7415	94443	28.928	5411	69835	21.39	40.4773	40.0623	72.9737	73.9441
SP8	NFATC1_3	cluster_6	Common_2	15866	11364.923	11093	11600	156.987	1.396	0.4813	0.001	0.0010909	3018	39257	57.876	1619	20119	29.661	1259	15866	23.391	41.7164	40.4157	77.7641	78.8608
DP	SOX17_4	cluster_6	Common_2	11619	8324.632	8057	8602	162.546	1.3957	0.481	0.001	0.0010909	2192	30688	48.463	1317	17642	27.861	852	11619	18.349	38.8686	37.8617	64.6925	65.8599
DP	SPIC_1	cluster_6	Common_2	70774	50730.571	50059	51329	376.3763	1.3951	0.4804	0.001	0.001	13902	181322	51.494	7805	99240	28.184	5485	70774	20.099	39.4548	39.0322	70.2755	71.316
DP	SPDEF_4	cluster_6	Common_2	37136	26619.188	26131	27049	279.9778	1.3951	0.4803	0.001	0.0010435	6274	94414	52.37	3617	51863	28.768	2522	37136	20.599	40.1976	39.3331	69.7263	71.604
SP8	BATF_known1	cluster_6	Common_2	23792	17054.598	16725	17386	202.3841	1.395	0.4803	0.001	0.0010435	3843	50078	57.839	2327	29701	34.304	1842	23792	27.479	47.9313	47.5099	79.1577	80.105
DP	SOX7_3	cluster_6	Common_2	16845	12075.2	11697	12430	216.2845	1.395	0.4802	0.001	0.0010909	2800	44824	49.319	1672	25254	27.786	1084	16845	18.534	38.7143	37.5803	64.8325	66.7023
DP	IKZF1_1	cluster_6	Common_2	51102	36637.296	36263	37100	267.3331	1.3948	0.4801	0.001	0.0010435	8537	102703	58.812	5426	63254	36.222	4320	51102	29.263	50.6033	49.7571	79.6167	80.7886
SP8	GATA_known16	cluster_6	Common_2	32884	23575.559	23031	24103	327.1402	1.3948	0.4801	0.001	0.0010435	4509	95037	55.067	2227	44036	25.516	1608	32884	19.054	35.662	34.6013	72.2048	74.6753
SP8	MAF_known1	cluster_6	Common_2	162486	116523.11	115614	117467	561.6737	1.3944	0.4797	0.001	0.001	25525	385561	56.181	14285	209608	30.543	10918	162486	23.676	42.7738	42.1427	76.4298	77.519
DN	SOX17_5	cluster_3	DN	8515	6106.296	5889	6277	111.427	1.3944	0.4796	0.001	0.0011429	2792	39191	71.088	680	8911	16.164	641	8515	15.445	22.9585	21.7269	94.2647	95.5561
DP	SOX21_3	cluster_6	Common_2	17246	12367.871	12074	12726	197.9699	1.3944	0.4796	0.001	0.0010909	3002	45066	51.174	1735	24817	28.181	1180	17246	19.583	39.3071	38.2683	68.0115	69.4927
DP	SOX5_3	cluster_6	Common_2	11532	8270.434	7992	8575	172.3893	1.3943	0.4796	0.001	0.0011429	2038	30641	52.026	1177	16437	27.909	799	11532	19.58	39.2051	37.6358	67.8845	70.1588
SP4	FOXO1_5	cluster_6	Common_2	121383	87075.949	86403	87755	398.2127	1.394	0.4792	0.001	0.0010435	25037	276688	58.01	13987	151415	31.745	11107	121383	25.449	44.3623	43.87	79.4095	80.1658
SP4	HES5_1	cluster_4	SP4	9020	6471.79	6273	6700	128.0806	1.3937	0.4789	0.001	0.0010435	9376	103478	59.348	1020	10976	6.2951	828	9020	5.1732	8.8311	8.7168	81.1765	82.1793
DP	SOX3_1	cluster_6	Common_2	9996	7172.96	6905	7476	168.4853	1.3935	0.4787	0.001	0.0010909	1698	27182	49.21	1001	15050	27.246	644	9996	18.097	37.927	36.7743	64.3357	66.4186
SP4	MYBL1_3	cluster_4	SP4	2333	1674.5	1575	1773	60.0867	1.393	0.4782	0.001	0.0010909	1511	16654	62.822	257	2708	10.215	218	2333	8.8005	14.4275	14.0086	84.8249	86.1521
SP8	XBP1_4	cluster_1	SP8	6689	4802.352	4610	4990	117.5791	1.3928	0.478	0.001	0.0010435	5176	67397	61.616	616	7855	7.1813	519	6689	6.1153	10.027	9.9248	84.2532	85.156
SP8	MAF_known10	cluster_6	Common_2	34920	25074.959	24661	25441	239.8905	1.3926	0.4778	0.001	0.0010909	8275	91208	55.774	4229	45435	27.784	3212	34920	21.354	38.8157	38.2861	75.9518	76.857
DP	SOX15_4	cluster_6	Common_2	8786	6309.204	6046	6518	143.102	1.3925	0.4777	0.001	0.0011429	1805	25291	45.839	1053	13999	25.373	642	8786	15.924	35.5679	34.7396	60.9687	62.7616
SP8	SPIC_1	cluster_6	Common_2	75145	53971.484	53351	54613	374.3363	1.3923	0.4775	0.001	0.001	14831	193520	54.959	7805	99240	28.184	5836	75145	21.341	39.35	38.8306	74.7726	75.7205
DP	SOX12_1	cluster_6	Common_2	11498	8258.397	8001	8535	157.252	1.3922	0.4774	0.001	0.0010435	2452	31970	50.662	1367	16802	26.625	913	11498	18.22	37.2349	35.965	66.7886	68.4323
DP	IRF4_1	cluster_6	Common_2	19528	14034.073	13715	14346	194.6413	1.3914	0.4766	0.001	0.0010435	2787	39153	57.73	1798	24589	36.256	1405	19528	28.793	50.4126	49.8761	78.1424	79.4176
SP4	IKZF1_1	cluster_6	Common_2	52692	37868.48	37398	38325	268.7883	1.3914	0.4766	0.001	0.0010435	8843	106411	60.935	5426	63254	36.222	4455	52692	30.174	50.3788	49.5174	82.1047	83.3022
SP8	RUNX1_4	cluster_6	Common_2	9282	6671.075	6493	6856	115.4958	1.3913	0.4765	0.001	0.0010909	2766	28019	55.372	1246	12216	24.142	928	9282	18.344	33.5503	33.1275	74.4783	75.9823
DP	POU2F2_known10	cluster_6	Common_2	14836	10664.26	10406	10918	152.3809	1.3912	0.4763	0.001	0.0010435	4216	38032	52.25	2357	20734	28.485	1662	14836	20.382	39.4213	39.0093	70.5134	71.554
SP8	RUNX3_1	cluster_6	Common_2	152427	109565.27	108742	110358	501.2	1.3912	0.4763	0.001	0.0010435	19753	302988	62.704	11621	175566	36.334	10003	152427	31.545	50.6404	50.3079	86.0769	86.8203
DP	EWSR1::FLI1_1	cluster_6	Common_2	388526	279298.373	277670	281091	1014.2216	1.3911	0.4762	0.001	0.0010435	43660	868688	53.282	28139	535783	32.863	20000	388526	23.831	45.8085	44.7256	71.0757	72.5156
DP	FOXO3_4	cluster_6	Common_2	7831	5629.481	5429	5821	119.684	1.391	0.4761	0.001	0.0010909	1629	21240	50.833	913	11306	27.058	620	7831	18.742	38.0602	36.8691	67.908	69.2641
SP4	RUNX3_3	cluster_6	Common_2	241023	173279.416	172291	174205	600.9239	1.3909	0.4761	0.001	0.001	22811	405311	62.285	15867	278975	42.871	13568	241023	37.039	59.4801	59.4662	85.5108	86.3959
DN	SOX18_2	cluster_3	DN	10874	7820.02	7613	8028	124.9754	1.3905	0.4756	0.001	0.0010909	3405	47699	72.637	847	11219	17.084	814	10874	16.559	23.906	22.7971	96.1039	96.9249
DP	PKNOX1_1	cluster_6	Common_2	65026	46771.604	46103	47327	382.7809	1.3903	0.4754	0.001	0.0010435	10906	164350	52.965	6051	88894	28.648	4358	65026	20.956	39.9597	39.5656	72.0212	73.15
SP4	NFKB_known11	cluster_4	SP4	19225	13827.826	13560	14132	168.1869	1.3903	0.4754	0.001	0.001	11939	145036	63.244	1852	22046	9.6133	1596	19225	8.3832	13.368	13.2553	86.1771	87.204
DP	TBX21_6	cluster_6	Common_2	53650	38597.199	38002	39161	349.0202	1.39	0.4751	0.001	0.0010909	7546	136200	53.52	4211	73113	28.73	3025	53650	21.082	40.0875	39.3906	71.8357	73.3796
DN	SOX7_4	cluster_3	DN	12344	8880.776	8648	9106	137.6778	1.3899	0.475	0.001	0.0010909	3341	53567	71.584	863	12935	17.286	814	12344	16.496	24.364	23.044	94.3221	95.431
DP	TCF7L1_2	cluster_6	Common_2	40516	29150.635	28739	29630	272.5701	1.3899	0.475	0.001	0.0010435	10535	116448	51.095	5380	57741	25.336	3721	40516	17.778	35.3204	34.7932	69.1636	70.1685
SP4	HESX1_1	cluster_6	Common_2	5046	3630.481	3501	3749	70.3802	1.3898	0.4749	0.001	0.0012632	1328	11959	60.289	700	6114	30.823	569	5046	25.439	42.8464	42.1942	81.2857	82.5319
SP4	RUNX2_5	cluster_4	SP4	19875	14305.213	13901	14679	233.5161	1.3893	0.4744	0.001	0.001	17450	305925	60.5	1441	24027	4.7516	1177	19875	3.9305	6.745	6.4967	81.6794	82.7194
SP8	MYBL1_3	cluster_1	SP8	1795	1291.782	1201	1387	58.6514	1.3893	0.4743	0.001	0.0010909	1462	16095	60.713	205	2158	8.1403	166	1795	6.771	11.3543	11.1525	80.9756	83.1789
DN	SOX10_9	cluster_3	DN	12846	9247.068	9013	9491	142.6474	1.3892	0.4742	0.001	0.0010435	4222	59134	70.343	1017	13538	16.104	958	12846	15.281	22.6907	21.7235	94.1986	94.8885
SP8	SPIC_2	cluster_6	Common_2	80236	57786.982	57109	58415	389.6507	1.3885	0.4735	0.001	0.0010435	15787	205822	55.49	8230	104958	28.297	6222	80236	21.632	39.4122	38.9832	75.6015	76.4458
SP4	MYBL1_5	cluster_4	SP4	5120	3687.378	3502	3848	106.5606	1.3884	0.4734	0.001	0.0010435	2888	46605	66.715	371	5637	8.0693	335	5120	7.3293	11.5997	10.9859	90.2965	90.8285
SP8	E2F_known26	cluster_1	SP8	3421	2464.297	2316	2611	90.1619	1.3881	0.4731	0.001	0.0010435	1668	28373	65.605	241	3860	8.9253	206	3421	7.9102	12.3501	12.0572	85.4772	88.6269
DN	TCF7L1_1	cluster_3	DN	40986	29529.844	29097	29976	260.0355	1.3879	0.4729	0.001	0.0010435	12339	198117	70.718	2782	42859	15.298	2643	40986	14.63	21.4199	20.6878	95.0036	95.6299
DP	SOX1_2	cluster_6	Common_2	14612	10529.228	10178	10882	196.7726	1.3877	0.4727	0.001	0.0010909	2774	38842	48.471	1650	22104	27.584	1068	14612	18.234	38.5004	37.6191	64.7273	66.1057
DP	TCF7_2	cluster_6	Common_2	37997	27381.613	26967	27832	262.0755	1.3877	0.4727	0.001	0.0010435	10707	118115	49.917	5168	55411	23.417	3499	37997	16.058	32.6796	32.1695	67.7051	68.573
SP8	STAT_known3	cluster_1	SP8	12720	9167.187	8825	9485	196.5776	1.3875	0.4725	0.001	0.0010435	7066	141634	65.658	756	14273	6.6166	662	12720	5.8966	9.3688	8.9809	87.5661	89.1193
SP8	RUNX3_1	cluster_1	SP8	33145	23888.711	23433	24367	295.3223	1.3875	0.4724	0.001	0.0010435	19753	302988	62.704	2579	38479	7.9634	2199	33145	6.8595	11.1325	10.9394	85.2656	86.1379
DP	NFATC2_1	cluster_6	Common_2	13793	9942.293	9771	10099	99.8152	1.3873	0.4722	0.001	0.0010909	5104	30624	54.868	3099	18285	32.761	2322	13793	24.712	45.4937	45.0398	74.9274	75.4334
SP4	NFATC1_3	cluster_6	Common_2	16396	11818.664	11525	12092	176.2405	1.3873	0.4722	0.001	0.0010909	3149	40963	60.391	1619	20119	29.661	1300	16396	24.172	41.2829	40.0264	80.2965	81.4951
SP8	FOXO6_3	cluster_6	Common_2	130720	94231.084	93522	95004	443.2321	1.3872	0.4722	0.001	0.0010435	22108	289208	59.065	12666	161340	32.951	10128	130720	26.697	45.8115	45.1993	79.9621	81.0214
SP8	NFKB_known10	cluster_1	SP8	17001	12257.312	11995	12530	169.6398	1.387	0.4719	0.001	0.001	10708	129395	64.216	1617	19222	9.5395	1417	17001	8.4372	13.2331	13.1388	87.6314	88.4455
SP4	KLF12_1	cluster_6	Common_2	62041	44734.765	44417	45080	188.1587	1.3869	0.4718	0.001	0.001	19302	115812	58.735	12826	76314	38.703	10376	62041	31.465	53.7561	53.5704	80.8982	81.297
SP8	ATF7_1	cluster_1	SP8	5969	4304.53	4138	4484	106.052	1.3866	0.4715	0.001	0.001	3427	44593	61.045	561	7117	9.7428	465	5969	8.1712	13.5687	13.3855	82.8877	83.8696
SP8	ETS_known17	cluster_1	SP8	15773	11377.367	11036	11715	206.1417	1.3863	0.4713	0.001	0.001	12384	211645	59.804	1185	19310	5.4563	948	15773	4.4569	7.655	7.4526	80	81.6831
DP	MYC_known20	cluster_6	Common_2	73283	52870.66	52290	53514	377.6072	1.3861	0.471	0.001	0.001	8735	143000	56.723	5818	93509	37.092	4492	73283	29.069	51.4253	51.2469	77.2087	78.37
DP	XBP1_2	cluster_6	Common_2	28725	20724.744	20292	21178	268.1061	1.386	0.4709	0.001	0.0010435	2416	55665	57.716	1647	36240	37.575	1265	28725	29.784	52.3593	51.6033	76.8063	79.2632
SP8	RUNX2_4	cluster_6	Common_2	133204	96109.978	95437	96817	460.3948	1.3859	0.4709	0.001	0.0010435	17076	261045	63.344	10116	152450	36.993	8771	133204	32.322	51.3645	51.0272	86.7042	87.3755
DP	FOXO1_5	cluster_6	Common_2	115656	83458.519	82674	84188	446.3305	1.3858	0.4707	0.001	0.0010435	23967	264888	55.536	13987	151415	31.745	10559	115656	24.248	44.0564	43.6622	75.4915	76.3834
DP	MAF_known1	cluster_6	Common_2	153989	111122.101	110224	112259	572.5663	1.3858	0.4707	0.001	0.001	24308	367004	53.477	14285	209608	30.543	10321	153989	22.438	42.4593	41.9584	72.2506	73.4652
DP	POU2F2_known13	cluster_6	Common_2	19252	13892.285	13525	14292	247.4851	1.3858	0.4707	0.001	0.0010909	2228	46947	52.881	1401	27261	30.707	948	19252	21.686	42.5494	41.0079	67.666	70.621
DP	XBP1_4	cluster_6	Common_2	35546	25657.137	25266	26027	233.8036	1.3854	0.4703	0.001	0.0010435	4834	62945	57.546	3479	44601	40.775	2741	35546	32.497	56.7025	56.4715	78.787	79.6978
SP8	ATF4_3	cluster_6	Common_2	20148	14542.98	14205	14863	197.8873	1.3854	0.4703	0.001	0.0010909	3790	49324	56.677	2058	25990	29.865	1569	20148	23.152	41.3984	40.8483	76.2391	77.5221
SP8	SPI1_known4	cluster_6	Common_2	74052	53451.681	52894	54025	358.3075	1.3854	0.4703	0.001	0.0010435	14322	186719	57.192	7415	94443	28.928	5749	74052	22.682	40.141	39.6596	77.532	78.4092
DP	SOX2_6	cluster_6	Common_2	9483	6845.34	6608	7145	160.7457	1.3853	0.4702	0.001	0.0010435	1589	25439	47.17	987	14899	27.626	610	9483	17.584	38.3889	37.2774	61.8034	63.6486
SP8	FOXO1_5	cluster_6	Common_2	121806	87925.645	87258	88588	394.7408	1.3853	0.4702	0.001	0.0010435	25271	279379	58.574	13987	151415	31.745	11148	121806	25.537	44.1138	43.5988	79.7026	80.4451
SP4	BATF_known1	cluster_6	Common_2	24446	17649.384	17314	17973	196.9937	1.3851	0.47	0.001	0.0010435	3974	51803	59.832	2327	29701	34.304	1887	24446	28.235	47.4836	47.1903	81.0915	82.307
SP4	BATF_known1	cluster_4	SP4	8007	5781.015	5581	5959	118.9502	1.385	0.4699	0.001	0.0010435	3974	51803	59.832	763	9682	11.183	626	8007	9.248	15.7524	15.4566	82.0446	82.6999
DP	TBX1_4	cluster_6	Common_2	61735	44579.866	43897	45293	432.1903	1.3848	0.4697	0.001	0.0010909	7984	152301	52.016	4722	86734	29.623	3276	61735	21.085	41.0321	40.5349	69.3774	71.1774
SP8	IKZF2_2	cluster_6	Common_2	15542	11224.47	10996	11432	130.3278	1.3846	0.4695	0.001	0.0010435	3469	34690	57.903	2039	19720	32.915	1584	15542	25.942	45.6616	44.8025	77.6851	78.8134
DP	XBP1_2	cluster_2	DP	6820	4925.5	4657	5159	153.1792	1.3846	0.4694	0.001	0.0010435	2416	55665	57.716	396	8574	8.8899	307	6820	7.0713	12.707	12.2519	77.5253	79.5428
DP	MYBL1_5	cluster_2	DP	5937	4288.234	4110	4444	108.7939	1.3844	0.4693	0.001	0.0010435	2741	44252	63.347	445	6853	9.81	378	5937	8.4988	13.7906	13.4163	84.9438	86.6336
SP8	POU2F2_known14	cluster_6	Common_2	5359	3870.86	3736	4030	96.1027	1.3843	0.4692	0.001	0.0012632	928	14244	63.304	474	6572	29.208	376	5359	23.817	40.5172	37.6229	79.3249	81.5429
DP	FOXO6_3	cluster_6	Common_2	124286	89785.895	89090	90556	456.3307	1.3842	0.4691	0.001	0.0010435	21070	275513	56.268	12666	161340	32.951	9614	124286	25.383	45.6289	45.1108	75.904	77.0336
SP4	SPZ1_2	cluster_6	Common_2	62855	45407.035	44935	45844	277.2475	1.3842	0.4691	0.001	0.0010435	14241	142725	57.95	8044	79031	32.088	6330	62855	25.521	44.4491	44.0392	78.6922	79.5321
DP	TBX5_5	cluster_6	Common_2	40824	29494.283	28981	30069	331.3114	1.3841	0.469	0.001	0.0010435	5461	103978	53.836	3074	55701	28.84	2186	40824	21.137	40.0293	39.2622	71.1126	73.2913
SP8	ERG_2	cluster_1	SP8	7758	5606.46	5393	5813	126.1581	1.3837	0.4685	0.001	0.001	6800	88560	61.21	729	9239	6.3857	604	7758	5.3621	8.8824	8.7602	82.8532	83.9701
DN	FOXO6_1	cluster_3	DN	8838	6388.077	6190	6571	115.8588	1.3835	0.4683	0.001	0.0010909	3202	41765	72.845	738	9094	15.861	714	8838	15.415	22.2986	21.1613	96.748	97.185
DN	TCF7L2_known7	cluster_3	DN	40708	29423.736	28994	29771	226.4135	1.3835	0.4683	0.001	0.0010435	14358	201641	70.809	3148	42486	14.92	3000	40708	14.295	20.8943	20.1884	95.2986	95.8151
SP8	MAF_known12	cluster_6	Common_2	57408	41494.558	40881	42098	366.5586	1.3835	0.4683	0.001	0.0010435	7662	130608	59.214	4314	71119	32.243	3422	57408	26.027	44.662	43.9544	79.3231	80.721
SP8	IKZF1_2	cluster_6	Common_2	74665	53976.39	53524	54413	270.3978	1.3833	0.4681	0.001	0.0010435	20377	163023	59.485	11645	91533	33.399	9428	74665	27.244	46.2679	45.8003	80.9618	81.5717
DN	SOX18_4	cluster_3	DN	8563	6191.826	6029	6367	105.1503	1.3829	0.4677	0.001	0.0012	2707	37982	72.115	673	8902	16.902	639	8563	16.258	23.6055	22.5449	94.948	96.1919
DP	HBP1_1	cluster_6	Common_2	22387	16188.899	15836	16567	224.616	1.3828	0.4676	0.001	0.0010435	3549	54705	50.814	2217	32501	30.189	1484	22387	20.795	41.8146	40.9231	66.9373	68.881
SP4	TBX4_2	cluster_6	Common_2	53087	38392.197	37829	38964	352.6542	1.3827	0.4675	0.001	0.0010909	6921	131760	58.982	3636	66282	29.671	2846	53087	23.764	41.1212	40.2907	78.2728	80.0926
SP8	TBX19_1	cluster_6	Common_2	27200	19677.191	19183	20142	272.0875	1.3823	0.4671	0.001	0.0010435	3619	68942	53.926	2041	37143	29.053	1458	27200	21.276	40.2874	39.4535	71.4356	73.2305
SP4	FOXO3_1	cluster_4	SP4	14649	10598.457	10339	10853	156.6229	1.3821	0.4669	0.001	0.0010435	5633	74003	67.715	1311	16241	14.861	1177	14649	13.404	20.8947	19.7951	89.7788	90.1976
DN	ETV6_2	cluster_3	DN	16863	12201.27	11878	12531	206.556	1.382	0.4668	0.001	0.0011429	10518	94973	25.655	5363	47824	12.919	1886	16863	4.5553	17.9312	17.7556	35.1669	35.2605
DP	TCF7L2_known7	cluster_6	Common_2	44992	32556.512	31993	33116	359.9909	1.382	0.4667	0.001	0.0010435	10010	140599	49.374	4944	66905	23.495	3268	44992	15.8	32.6474	32.0002	66.1003	67.2476
SP8	TBX21_6	cluster_6	Common_2	56921	41202.487	40590	41853	364.3321	1.3815	0.4662	0.001	0.0010909	8068	145632	57.226	4211	73113	28.73	3211	56921	22.367	39.7992	39.0855	76.2527	77.8535
SP4	NFE2L1::MAFG_1	cluster_6	Common_2	68202	49371.066	48674	50024	418.935	1.3814	0.4661	0.001	0.0010435	8781	185151	56.968	4408	88615	27.265	3299	68202	20.985	37.5698	36.8359	74.8412	76.9644
SP8	E2F7_1	cluster_1	SP8	6526	4723.893	4537	4913	104.6399	1.3814	0.4661	0.001	0.0010435	4510	58679	64.962	603	7441	8.2378	520	6526	7.2248	11.5299	11.1215	86.2355	87.7033
SP8	PBX3_known1	cluster_6	Common_2	33838	24495.931	24118	24827	216.9613	1.3814	0.4661	0.001	0.0010435	6890	76602	56.96	4029	43510	32.354	3088	33838	25.162	44.8186	44.1738	76.6443	77.7706
SP4	IKZF3_1	cluster_6	Common_2	37876	27423.306	27008	27836	241.7277	1.3811	0.4659	0.001	0.0010435	6633	79768	59.129	4061	47307	35.067	3213	37876	28.076	48.4396	47.4827	79.1184	80.0643
SP8	MYBL2_4	cluster_1	SP8	2210	1600.025	1487	1697	62.1171	1.381	0.4657	0.001	0.0010435	1616	22677	64.03	186	2524	7.1267	159	2210	6.2401	9.8391	9.7456	85.4839	87.5594
SP4	MAFF_1	cluster_6	Common_2	56514	40926.746	40429	41496	329.1726	1.3808	0.4656	0.001	0.001	9927	139260	55.679	5394	73858	29.53	4058	56514	22.595	40.8784	40.5816	75.2317	76.5171
SP8	MGA_2	cluster_6	Common_2	34811	25208.482	24815	25708	258.5899	1.3809	0.4656	0.001	0.0010909	5058	86090	56.587	2767	45310	29.782	2078	34811	22.881	41.0834	40.4356	75.0994	76.8285

SP8	ELK3_2	cluster_1	SP8	4142	2999.705	2830	3167	107.1288	1.3807	0.4654	0.001	0.0011429	5710	51570	24.526	1388	12358	5.8774	462	4142	1.9699	8.0911	8.0318	33.2853	33.5168
DP	CBX5_1	cluster_6	Common_2	15540	11255.789	10999	11519	156.8191	1.3806	0.4653	0.001	0.0010435	3561	39214	51.585	2111	22302	29.338	1449	15540	20.443	40.6908	39.6287	68.6405	69.6798
DP	TBX4_2	cluster_6	Common_2	49981	36203.42	35598	36812	362.7898	1.3805	0.4652	0.001	0.0010909	6487	123601	55.329	3636	66282	29.671	2666	49981	22.374	41.0976	40.4374	73.3223	75.4066
DP	SOX9_6	cluster_6	Common_2	20141	14590.079	14239	14993	217.5308	1.3804	0.4651	0.001	0.0010909	3625	54432	49.299	2124	30252	27.399	1379	20141	18.242	38.0414	37.0021	64.9247	66.5774
SP8	IRF_known21	cluster_1	SP8	21125	15304.43	14990	15596	199.5315	1.3803	0.465	0.001	0.001	7912	114984	65.268	1643	23596	13.394	1461	21125	11.991	18.4656	18.3721	88.9227	89.5279
DN	TCF7_1	cluster_3	DN	45170	32728.496	32229	33193	295.3274	1.3801	0.4648	0.001	0.0010435	13527	217263	71.046	3079	47321	15.474	2920	45170	14.771	21.5865	20.7905	94.836	95.4544
DP	IKZF2_3	cluster_6	Common_2	20552	14890.749	14641	15164	163.7216	1.3802	0.4648	0.001	0.0010909	4868	48814	55.261	2881	27614	31.261	2103	20552	23.267	43.2005	42.1027	72.9955	74.426
SP8	FOXP3_1	cluster_6	Common_2	46227	33493.662	32818	33990	338.5913	1.3802	0.4648	0.001	0.0010909	7647	122767	55.431	3984	61409	27.727	2932	46227	20.872	38.3418	37.6543	73.5944	75.2772
SP4	HMGA1_1	cluster_4	SP4	4449	3223.574	3133	3310	56.4689	1.38	0.4647	0.001	0.0010435	3745	22602	66.657	821	4883	14.401	748	4449	13.121	19.9733	19.6841	91.1084	91.112
SP8	SMAD3_1	cluster_6	Common_2	67967	49257.833	48837	49720	272.809	1.3798	0.4645	0.001	0.0010435	19958	165967	56.427	10800	88038	29.932	8305	67967	23.108	41.6124	40.9521	76.8981	77.2019
DN	SOX17_4	cluster_3	DN	10023	7264.01	7073	7462	124.464	1.3798	0.4644	0.001	0.0010909	3286	46015	72.668	786	10403	16.429	751	10023	15.829	22.8545	21.782	95.5471	96.3472
DP	ATF3_known5	cluster_6	Common_2	74119	53720.023	53105	54361	347.8014	1.3797	0.4644	0.001	0.001	11264	158203	55.075	7138	98043	34.132	5319	74119	25.803	47.2212	46.8506	74.5167	75.5985
DP	NFATC1_1	cluster_6	Common_2	38101	27616.97	27099	28159	306.7848	1.3796	0.4643	0.001	0.0010909	4535	87510	54.48	2869	52065	32.414	2027	38101	23.72	44.6968	43.539	70.6518	73.1797
DP	SOX30_1	cluster_6	Common_2	18311	13273.407	12955	13626	210.7349	1.3795	0.4641	0.001	0.0010435	3080	46247	52.079	1811	26029	29.311	1251	18311	20.62	40.6169	39.5939	69.0779	70.3485
SP4	E2F2_2	cluster_4	SP4	8002	5800.46	5593	6013	124.0891	1.3795	0.4641	0.001	0.0010909	3236	55175	67.435	551	8817	10.776	493	8002	9.78	15.2349	14.5029	89.4737	90.7565
SP4	FOXP3_1	cluster_6	Common_2	48089	34861.134	34357	35382	309.1214	1.3794	0.4641	0.001	0.0010909	7981	128134	57.854	3984	61409	27.727	3051	48089	21.713	38.2283	37.5302	76.5813	78.3094
SP8	RUNX2_5	cluster_1	SP8	32424	23505.833	23010	23983	291.8596	1.3794	0.464	0.001	0.001	18306	320965	63.474	2260	37691	7.4537	1914	32424	6.4122	10.4556	10.102	84.6903	86.0258
SP4	HES7_1	cluster_4	SP4	8950	6490.22	6299	6684	122.22	1.3789	0.4636	0.001	0.0010435	9737	107540	60.702	1003	10767	6.0776	826	8950	5.0519	8.4831	8.3225	82.3529	83.1244
DN	SOX1_3	cluster_3	DN	12365	8969.508	8758	9179	137.5523	1.3785	0.4631	0.001	0.0010909	3794	53161	70.646	979	13064	17.361	919	12365	16.432	24.2225	23.2595	93.8713	94.6494
SP4	REL_1	cluster_4	SP4	16573	12022.225	11802	12253	147.1003	1.3785	0.4631	0.001	0.001	12552	113599	63.434	2162	19122	10.678	1862	16573	9.2545	14.8343	14.589	86.124	86.6698
DP	SMAD4_1	cluster_2	DP	55894	40548.136	39977	41141	364.8544	1.3785	0.463	0.001	0.001	25085	356038	57.788	5161	70716	11.478	4003	55894	9.0721	15.9577	15.6989	77.5625	79.0401
SP4	IKZF1_2	cluster_6	Common_2	74874	54319.49	53913	54740	259.0357	1.3784	0.463	0.001	0.0010435	20507	164063	59.864	11645	91533	33.399	9455	74874	27.32	46.1062	45.6373	81.1936	81.8
DP	PBX3_known1	cluster_6	Common_2	31259	22682.337	22299	23028	222.1158	1.3781	0.4627	0.001	0.0010435	6370	70693	52.566	4029	43510	32.354	2850	31259	23.244	44.741	44.218	70.7372	71.8433
DP	SOX13_1	cluster_6	Common_2	9815	7121.631	6862	7363	156.0293	1.3781	0.4627	0.001	0.0010909	1690	25424	51.067	1044	14517	29.159	683	9815	19.714	40.4142	38.6053	65.4215	67.6104
SP8	RUNX2_5	cluster_6	Common_2	175478	127354.132	126506	128226	507.0269	1.3779	0.4624	0.001	0.001	18306	320965	63.474	11640	201282	39.805	10037	175478	34.702	54.829	54.672	86.2285	87.1802
SP8	KLF12_1	cluster_1	SP8	12322	8947.205	8775	9100	100.3967	1.3771	0.4617	0.001	0.001	19874	119244	60.476	2507	14856	7.5344	2069	12322	6.2492	10.4106	10.3334	82.5289	82.9429
SP8	LMO2_2	cluster_6	Common_2	12679	9206.165	9001	9373	107.4913	1.3772	0.4617	0.001	0.0010435	3634	29090	57.111	2056	16178	31.761	1598	12679	24.892	43.9736	43.5854	77.7237	78.3719
SP8	SMAD_2	cluster_6	Common_2	88636	64361.865	63755	64956	364.5142	1.3771	0.4617	0.001	0.0010435	21409	221606	55.683	11532	116795	29.347	8701	88636	22.271	40.6418	39.9971	75.4509	75.8902
DP	TBX15_2	cluster_6	Common_2	40084	29108.851	28755	29422	205.6761	1.377	0.4616	0.001	0.0010909	14654	102598	53.396	7932	54774	28.506	5769	40084	20.861	39.3681	39.069	72.7307	73.1807
SP8	RUNX1_2	cluster_6	Common_2	22927	16650.56	16290	17003	221.3604	1.3769	0.4615	0.001	0.0010435	4117	64095	56.816	2063	30260	26.824	1526	22927	20.323	37.0658	35.7703	73.9699	75.7667
DN	FOXO3_4	cluster_3	DN	6185	4492.991	4344	4631	86.3608	1.3765	0.461	0.001	0.0010909	2345	30568	73.157	521	6382	15.274	501	6185	14.802	21.3646	20.2336	96.1612	96.9132
SP4	ATF4_2	cluster_6	Common_2	20102	14603.247	14310	14871	164.6342	1.3765	0.461	0.001	0.0010909	3978	47794	58.841	2144	25111	30.915	1692	20102	24.749	42.5339	42.0597	78.9179	80.0526
SP8	ERG_4	cluster_1	SP8	7740	5624.086	5422	5824	121.7298	1.3762	0.4606	0.001	0.001	6801	88600	61.768	724	9199	6.4131	603	7740	5.3959	8.8663	8.7359	83.2873	84.1396
SP8	TBX2_1	cluster_6	Common_2	63711	46302.034	45756	46868	327.8658	1.376	0.4605	0.001	0.0010435	8536	145462	59.643	4773	78671	32.257	3787	63711	26.123	44.365	43.7991	79.3421	80.9841
SP8	TBX15_1	cluster_6	Common_2	41203	29945.844	29449	30458	313.1611	1.3759	0.4604	0.001	0.0010909	5892	106171	56.155	3141	54275	28.707	2327	41203	21.793	39.4942	38.8081	74.0847	75.9152
DP	SPI1_known1	cluster_6	Common_2	94823	68919.228	68297	69440	312.1732	1.3759	0.4603	0.001	0.001	34618	243610	52.166	18984	132280	28.326	13527	94823	20.305	39.075	38.9241	71.2547	71.6836
SP8	SMAD_1	cluster_6	Common_2	55780	40549.972	40176	40936	244.7289	1.3756	0.46	0.001	0.0010435	15801	126448	57.942	8902	70228	32.18	7016	55780	25.56	44.4023	44.113	78.8137	79.427
DP	IKZF3_1	cluster_6	Common_2	35702	25957.588	25570	26360	230.564	1.3754	0.4598	0.001	0.0010435	6241	75044	55.627	4061	47307	35.067	3027	35702	26.464	48.5018	47.5748	74.5383	75.4687
SP4	HMGA1_2	cluster_4	SP4	16153	11744.702	11457	12028	178.0075	1.3753	0.4598	0.001	0.0010435	5885	82554	67.162	1373	18136	14.755	1209	16153	13.141	20.5438	19.5666	88.0554	89.0659
SP4	REL_2	cluster_4	SP4	16321	11866.668	11628	12107	143.3591	1.3753	0.4598	0.001	0.0010435	12661	114508	63.571	2133	18854	10.467	1836	16321	9.0608	14.5012	14.2532	86.0759	86.5652
DP	SOX14_2	cluster_6	Common_2	12389	9008.532	8807	9224	133.9113	1.3752	0.4597	0.001	0.0010909	2895	31972	49.947	1716	18299	28.587	1141	12389	19.354	39.4128	38.7495	66.4918	67.7032
DN	SOX14_3	cluster_3	DN	13411	9751.979	9533	9990	147.7202	1.3752	0.4596	0.001	0.0010909	4092	57347	70.134	1064	14226	17.398	994	13411	16.401	24.2913	23.3857	93.4211	94.2711
DP	ATF6_1	cluster_6	Common_2	15014	10917.578	10730	11091	110.5909	1.3752	0.4596	0.001	0.0010435	3671	25707	59.612	2634	18311	42.461	2151	15014	34.816	58.5944	58.4043	81.6629	81.9944
SP4	TBX1_4	cluster_6	Common_2	68233	49630.159	48949	50286	397.2707	1.3748	0.4592	0.001	0.0010909	8929	170354	58.182	4722	86734	29.623	3638	68233	23.304	40.7436	40.0537	77.0436	78.6693
SP8	KLF12_1	cluster_6	Common_2	63295	46043.849	45700	46355	197.9748	1.3747	0.4591	0.001	0.001	19874	119244	60.476	12826	76314	38.703	10592	63295	32.101	53.2958	53.0802	82.5823	82.9402
SP8	ARID5A_1	cluster_1	SP8	1932	1405.288	1314	1492	56.7067	1.3745	0.4589	0.001	0.0012632	834	11458	67.591	182	2224	13.119	155	1932	11.397	18.5851	16.8616	85.1648	86.8705
DP	TCF21_1	cluster_6	Common_2	46117	33553.984	33001	34075	325.7845	1.3744	0.4588	0.001	0.001	11430	148923	52.886	5010	63749	22.639	3569	46117	16.377	31.2248	30.967	71.2375	72.3415
SP8	RUNX2_4	cluster_1	SP8	27133	19745.065	19335	20188	242.9088	1.3741	0.4585	0.001	0.0010435	17076	261045	63.344	2118	31471	7.6366	1809	27133	6.5839	10.5938	10.394	85.4108	86.2159
DN	SOX9_6	cluster_3	DN	16416	11947.01	11669	12200	160.9106	1.374	0.4584	0.001	0.0010909	5272	79251	71.777	1215	17231	15.606	1151	16416	14.868	21.8323	20.7139	94.7325	95.2702
DN	TCF7_2	cluster_3	DN	33921	24703.45	24347	25021	203.5085	1.3731	0.4575	0.001	0.0010435	15144	167157	70.642	3302	35484	14.996	3147	33921	14.335	20.7805	20.2929	95.3059	95.5952
DP	SOX21_2	cluster_6	Common_2	16897	12305.705	12009	12628	186.6933	1.3731	0.4574	0.001	0.0010909	3145	44041	49.589	1889	25323	28.513	1240	16897	19.026	39.4277	38.3665	65.6432	66.7259
SP4	TBX21_6	cluster_6	Common_2	58167	42367.209	41827	42999	349.5189	1.3729	0.4572	0.001	0.0010909	8330	150313	59.065	4211	73113	28.73	3293	58167	22.857	39.5318	38.6973	78.2	79.5577
SP8	SOX1_3	cluster_6	Common_2	14621	10651.323	10417	10918	156.1744	1.3727	0.457	0.001	0.0010909	2972	41649	55.348	1464	19722	26.209	1066	14621	19.43	35.8681	35.1053	72.8142	74.1355
DN	SOX1_2	cluster_3	DN	13079	9528.534	9314	9745	132.5312	1.3726	0.4569	0.001	0.0010909	4151	58133	72.545	1031	13594	16.964	979	13079	16.321	23.5847	22.4984	94.9564	96.2116
SP4	TBX5_5	cluster_6	Common_2	44218	32216.692	31602	32744	337.6032	1.3725	0.4568	0.001	0.0010435	5992	114030	59.04	3074	55701	28.84	2379	44218	22.894	39.7029	38.7775	77.391	79.3846
DP	TCF12_known1	cluster_6	Common_2	109453	79751.567	79194	80231	313.7854	1.3724	0.4567	0.001	0.0010909	36358	261819	56.235	19920	142178	30.538	15271	109453	23.509	42.0018	41.8048	76.6616	76.9831
SP4	E2F2_3	cluster_4	SP4	3979	2899.207	2760	3053	94.0307	1.3723	0.4566	0.001	0.001	2073	35280	66.325	283	4492	8.4447	248	3979	7.4803	11.9633	11.2783	87.6325	88.5797
SP8	MGA_3	cluster_6	Common_2	35546	25905.205	25442	26339	268.1641	1.3721	0.4564	0.001	0.0010909	4956	84386	59.559	2681	44055	31.094	2115	35546	25.088	42.6755	42.1231	78.8885	80.6855
DP	SOX11_1	cluster_6	Common_2	60979	44464.225	43897	45035	368.6395	1.3714	0.4557	0.001	0.0010435	9836	159338	54.566	5407	83403	28.561	3867	60979	20.882	39.3148	38.2702	71.5184	73.1137
DP	SOX4_1	cluster_6	Common_2	66615	48572.787	47923	49212	399.8234	1.3714	0.4557	0.001	0.0010435	10641	172327	54.726	5901	90966	28.888	4223	66615	21.155	39.6861	38.6562	71.5641	73.2307
DP	TCF3_1	cluster_6	Common_2	300037	218819.651	217600	220112	724.4183	1.3712	0.4554	0.001	0.0011429	49855	709136	57.352	27526	383634	31.026	21272	300037	24.266	42.6677	42.3102	77.2797	78.2092
DP	TBX20_2	cluster_6	Common_2	37360	27251.372	26688	27800	332.7206	1.3709	0.4552	0.001	0.0010435	5116	92297	53.087	2975	51847	29.821	2101	37360	21.488	41.0672	40.478	70.6218	72.0582
SP8	POU2F2_known1	cluster_6	Common_2	21817	15918.903	15545	16274	230.5425	1.3705	0.4547	0.001	0.0010435	3160	57168	59.186	1610	27604	28.578	1236	21817	22.587	39.1139	38.163	76.7702	79.0356
DP	FOXO4_3	cluster_6	Common_2	8886	6483.978	6250	6708	136.2381	1.3704	0.4546	0.001	0.0010909	1828	23799	50.665	1056	13081	27.848	701	8886	18.917	38.3479	37.3377	66.3826	67.9306
DP	SOX9_4	cluster_6	Common_2	24733	18048.28	17669	18411	229.4851	1.3704	0.4546	0.001	0.0010909	4428	66520	51.448	2495	35804	27.691	1678	24733	19.129	37.8952	37.1813	67.2545	69.0789
DP	CUX2_1	cluster_6	Common_2	2585	1886.197	1755	2010	84.5474	1.3703	0.4545	0.001	0.0012632	328	6022	56.539	241	3664	34.401	156	2585	24.27	47.561	42.9259	64.7303	70.5513
SP8	ATF3_known5	cluster_6	Common_2	77879	56835.124	56234	57391	367.978	1.3703	0.4544	0.001	0.001	11933	167611	58.351	7138	98043	34.132	5600	77879	27.112	46.9287	46.4641	78.4533	79.4335
DN	FOXO1_4	cluster_3	DN	7680	5605.592	5447	5759	94.8769	1.37	0.4542	0.001	0.0010909	2797	36450	73.199	647	7957	15.979	620	7680	15.423	22.1666	21.07	95.8269	96.5188
DP	PKNOX1_2	cluster_6	Common_2	34860	25459.868	25053	25842	247.3856	1.3692	0.4533	0.001	0.0010435	9569	106189	50.266	4710	51072	24.176	3179	34860	16.502	33.2219	32.8283	67.4947	68.2566
SP8	SOX14_3	cluster_6	Common_2	16271	11883.391	11593	12157	181.1884	1.3692	0.4533	0.001	0.0010909	3228	45250	55.339	1626	21909	26.794	1187	16271	19.899	36.772	35.958	73.0012	74.2663
SP8	TBX20_2	cluster_6	Common_2	39804	29072.684	28563	29613	328.5223	1.3691	0.4532	0.001	0.0010435	5480	98825	56.841	2975	51847	29.821	2238	39804	22.894	40.8394	40.2773	75.2269	76.772
SP8	MTF1_3	cluster_6	Common_2	82000	59910.889	59369	60478	330.9368	1.3687	0.4528	0.001	0.001	12742	167257	61.78	7551	97546	36.03	6291	82000	30.288	49.3722	49.0263	83.3135	84.0629
DP	ETV7_1	cluster_6	Common_2	87598	64018.188	63421	64623	354.7375	1.3683	0.4524	0.001	0.001	25386	228951	51.568	13931	124176	27.969	9744	87598	19.73	38.3834	38.2606	69.9447	70.5434
SP4	MAF_known1	cluster_6	Common_2	165085	120668.61	119865	121465	510.3014	1.3681	0.4522	0.001	0.001	26418	399043	58.146	14285	209608	30.543	11084	165085	24.055	41.9562	41.3702	77.5919	78.7589
SP4	MAFG_1	cluster_6	Common_2	50218	36712.341	36053	37295	370.0002	1.3679	0.4519	0.001	0.0010435	6447	129183	56.562	3395	65678	28.757	2540	50218	21.988	39.3982	38.8735	74.8159	76.4609
DN	SOX2_5	cluster_3	DN	14522	10617.5	10355	10879	155.974	1.3677	0.4518	0.001	0.0010909	4046	64802	73.448	1000	15053	17.061	956	14522	16.46	23.6283	22.4098	95.6	96.4725
DP	SOX8_2	cluster_6	Common_2	24256	17734.84	17353	18132	237.41	1.3677	0.4517	0.001	0.0010909	4342	65204	50.995	2477	35522	27.781	1650	24256	18.97	38.0009	37.2002	66.6128	68.2844
DP	HES5_1	cluster_2	DP	17383	12712.78	12414	13013	174.1117	1.3673	0.4514	0.001	0.0010435	9609	106096	60.849	1941	21036	12.065	1588	17383	9.9697	16.5262	16.3842	81.8135	82.6345
DP	MYC_known13	cluster_6	Common_2	44884	32825.227	32457	33239	232.6855	1.3674	0.4514	0.001	0.0010909	9190	101216	56.829	5376	58122	32.633	4106	44884	25.201	44.679	44.3448	76.3765	77.2238
DN	SOX15_2	cluster_3	DN	11115	8128.81	7923	8318	119.6273	1.3673	0.4513	0.001	0.0010909	3740	52487	73.258	869	11549	16.119	831	11115	15.514	22.2193	21.1767	95.6272	96.2421
DP	MGA_3	cluster_6	Common_2	33597	24573.565	24163	25037	261.3113	1.3672	0.4512	0.001	0.0010909	4692	79902	56.395	2681	44055	31.094	2000	33597	23.713	42.6257	42.0478	74.599	76.2615
DN	SOX7_3	cluster_3	DN	14115	10324.309	10084	10573	150.8028	1.3671	0.4511	0.001	0.0010909	4157	66552	73.226	976	14704	16.179	926	14115	15.53	22.2757	21.209	94.877	95.9943
SP8	TBX1_2	cluster_6	Common_2	32929	24087.941	23615	24511	271.7044	1.367	0.451	0.001	0.0010909	4843	87270	55.954	2536	43862	28.122	1856	32929	21.113	38.3234	37.7323	73.1861	75.0741
SP4	SMAD_1	cluster_6	Common_2	56507	41340.506	40938	41750	246.7661	1.3669	0.4509	0.001	0.0010435	16152	129249	59.226	8902	70228	32.18	7112	56507	25.893	44.0317	43.7195	79.8922	80.4622
SP4	TBX2_1	cluster_6	Common_2	64116	46906.758	46374	47439	343.1757	1.3669	0.4509	0.001	0.0010435	8640	147190	60.352	4773	78671	32.257	3821	64116	26.289	44.2245	43.56	80.0545	81.4989
DP	MAFF_1	cluster_6	Common_2	52412	38352.767	37802	38856	317.8023	1.3666	0.4506	0.001	0.001	9294	130389	52.132	5394	73858	29.53	3761	52412	20.955	40.467	40.1966	69.7256	70.9632
SP4	MAF_known11	cluster_6	Common_2	74695	54655.31	54107	55293	381.1639	1.3666	0.4506	0.001	0.0010435	10831	174705	57.034	6133	96460	31.49	4681	74695	24.385	43.2185	42.7549	76.3248	77.4362
SP8	TBX21_4	cluster_6	Common_2	41801	30589.157	30100	31099	292.4694	1.3665	0.4505	0.001	0.0010909	7628	114668	56.477	3809	55074	27.125	2827	41801	20.588	37.0608	36.4539	74.219	75.8997
DP	SOX5_1	cluster_6	Common_2	6683	4890.857	4728	5024	87.8435	1.3664	0.4503	0.001	0.0010909	1887	16983	50.471	1115	9815	29.169	754	6683	19.861	39.9576	39.3511	67.6233	68.0897
DP	ETV2_1	cluster_2	DP	27237	19937.706	19567	20282	220.2672	1.3661	0.45	0.001	0.0010435	15356	154289	52.346	3829	37988	12.888	2721	27237	9.2408	17.7195	17.6532	71.0629	71.699
DP	NFKB_known11	cluster_6	Common_2	68487	50134.405	49636	50657	313.6801	1.3661	0.45	0.001	0.001	11257	136572	59.553	7103	84596	36.889	5689	68487	29.864	50.5374	50.1472	80.0929	80.9577
DP	TBX21_4	cluster_6	Common_2	38720	28344.834	27846	28795	289.16	1.366	0.45	0.001	0.0010909	7045	105923	52.17	3809	55074	27.125	2617	38720	19.071	37.1469	36.5549	68.7057	70.3054
SP4	ATF3_known5	cluster_6	Common_2	77478	56722.357	56074	57304	360.7212	1.3659	0.4499	0.001	0.001	11915	167390	58.274	7138	98043	34.132	5574	77478	26.973	46.7814	46.2859	78.0891	79.0245
DP	TBX4_1	cluster_6	Common_2	30969	22672.812	22393	22960	178.4968	1.3659	0.4498	0.001	0.0010435	11484	80419	53.597	6158	42515	28.335	4454	30969	20.64	38.7844	38.5096	72.3287	72.8425
DN	TCF7L2_known5	cluster_3	DN	45943	33642.892	33220	34025	252.783	1.3656	0.4495	0.001	0.0010435	13945	223921	71.487	3117	48148	15.371	2956	45943	14.667	21.1976	20.5175	94.8348	95.4204
SP4	SMAD_2	cluster_6	Common_2	90430	66222.942	65690	66730	325.2271	1.3655	0.4495	0.001	0.0010435	22080	227820	57.244	11532	116795	29.347	8897	90430	22.722	40.2944	39.6936	77.1505	77.4263
DN	PBX1_2	cluster_3	DN	10657	7804.517	7603	8002	117.3223	1.3654	0.4494	0.001	0.0010435	3613	51272	73.03	821	11039	15.724	790	10657	15.179	21.8655	20.7852	96.2241	96.5395
DP	MTF1_3	cluster_2	DP	27147	19881.54	19531	20270	226.2392	1.3654	0.4493	0.001	0.001	12915	169509	62.611	2484	31937	11.797	2088	27147	10.027	16.1672	16.0151	84.058	85.0017
SP4	MGA_2	cluster_6	Common_2	35107	25712.453	25213	26165	285.3156	1.3654	0.4493	0.001	0.0010909	5162	87892	57.771	2767	45310	29.782	2103	35107	23.076	40.74	39.9433	76.0029	77.4818
DP	FOXP3_1	cluster_6	Common_2	42120	30853.631	30317	31332	311.386	1.3651	0.4491	0.001	0.0010909	7025	112814	50.937	3984	61409	27.727	2664	42120	19.018	37.9217	37.3358	66.8675	68.5893
SP8	NFKB_known11	cluster_1	SP8	20863	15284.378	14996	15594	185.022	1.365	0.4489	0.001	0.001	12335	149870	65.352	1970	23567	10.277	1733	20863	9.0975	14.0495	13.9207	87.9695	88.5263
SP4	TBX15_1	cluster_6	Common_2	41933	30722.603	30237	31277	318.8523	1.3649	0.4488	0.001	0.0010909	6066	109292	57.806	3141	54275	28.707	2375	41933	22.179	39.1527	38.3679	75.6129	77.2602
SP4	SMAD3_1	cluster_6	Common_2	69588	50986.045	50563	51443	270.6709	1.3648	0.4487	0.001	0.0010435	20754	171916	58.449	10800	88038	29.932	8513	69588	23.659	41.0186	40.4779	78.8241	79.0431
DN	LMO2_1	cluster_3	DN	83461	61169.922	60418	61958	440.8962	1.3644	0.4483	0.001	0.001	22843	252436	30.177	18571	204084	24.397	7633	83461	9.9771	33.4151	33.0622	41.1017	40.8954
DN	FOXO4_3	cluster_3	DN	7044	5162.523	5010	5307	90.6066	1.3644	0.4482	0.001	0.0010909	2636	34308	73.038	592	7294	15.528	568	7044	14.996	21.5478	20.5317	95.9459	96.5725
SP4	TBX20_2	cluster_6	Common_2	40556	29727.134	29197	30225	313.3983	1.3643	0.4481	0.001	0.0010435	5606	101105	58.153	2975	51847	29.821	2285	40556	23.327	40.7599	40.1128	76.8067	78.2225
SP4	MYBL1_3	cluster_6	Common_2	7796	5715.32	5540	5856	96.1928	1.364	0.4478	0.001	0.0010909	1511	16654	62.822	844	9130	34.44	718	7796	29.408	47.5182	46.8116	85.0711	85.3888
DP	E2F8_1	cluster_6	Common_2	40169	29451.792	29082	29794	220.581	1.3639	0.4477	0.001	0.001	7146	78795	61.175	4519	48700	37.81	3684	40169	31.186	51.5533	50.9791	81.5225	82.4825
SP4	FOXM1_1	cluster_6	Common_2	17289	12678.882	12460	12906	127.932	1.3636	0.4474	0.001	0.0010909	5181	41487	58.448	2799	21955	30.931	2187	17289	24.357	42.2119	41.6733	78.135	78.7474
DP	SMAD4_1	cluster_6	Common_2	174054	127661.76	126789	128521	519.2931	1.3634	0.4472	0.001	0.001	25085	356038	57.788	15994	222382	36.094	12357	174054	28.25	49.2605	48.8864	77.2602	78.268
SP8	TBX1_4	cluster_6	Common_2	65183	47810.644	47055	48550	440.0182	1.3633	0.4472	0.001	0.0010909	8594	163899	55.977	4722	86734	29.623	3477	65183	22.262	40.4585	39.7702	73.6341	75.1528
DN	TCF7L1_2	cluster_3	DN	32736	24014.648	23699	24317	189.6197	1.3632	0.4469	0.001	0.0010435	14657	161969	71.069	3198	34366	15.079	3036	32736	14.364	20.7137	20.2113	94.9343	95.2569
SP4	EWSR1::FLI1_1	cluster_6	Common_2	415988	305183.316	303748	306746	929.4355	1.3631	0.4469	0.001	0.0010435	47664	952228	58.406	28139	535783	32.863	21437	415988	25.515	44.9752	43.6858	76.1825	77.6411
DP	TBX19_1	cluster_6	Common_2	24660	18092.781	17678	18535	264.2015	1.363	0.4467	0.001	0.0010435	3308	63063	49.328	2041	37143	29.053	1316	24660	19.289	39.7823	39.1038	64.4782	66.3921
SP8	ELK3_1	cluster_1	SP8	4197	3079.05	2889	3273	113.0686	1.363	0.4467	0.001	0.0011429	5938	53644	24.833	1397	12448	5.7624	469	4197	1.9429	7.8983	7.8238	33.5719	33.7163
SP4	ELK3_1	cluster_4	SP4	6944	5095.343	4875	5315	133.7001	1.3627	0.4465	0.001	0.0011429	6270	56640	26.22	2201	19599	9.0727	776	6944	3.2145	12.3764	12.2599	35.2567	35.4304
DP	E2F7_1	cluster_6	Common_2	27771	20383.192	20077	20651	185.0141	1.3624	0.4462	0.001	0.0010435	4150	54006	59.789	2740	34586	38.289	2170	27771	30.745	52.2892	51.4221	79.1971	80.2955
SP8	NFATC1_1	cluster_6	Common_2	41779	30673.36	30077	31118	304.1647	1.362	0.4458	0.001	0.0010909	5066	97783	60.876	2869	52065	32.414	2241	41779	26.01	44.2361	42.7262	78.1108	80.2439
SP4	MAF_known12	cluster_6	Common_2	58679	43090.085	42500	43643	343.1497	1.3618	0.4455	0.001	0.0010435	7957	135675	61.511	4314	71119	32.243	3497	58679	26.603	43.9487	43.2497	81.0617	82.5082
DN	RREB1_2	cluster_3	DN	89269	65561.542	64159	66931	835.2603	1.3616	0.4453	0.001	0.001	18681	474233	23.829	14003	307557	15.454	4025	89269	4.4856	21.546	18.8239	28.7438	29.0252
SP4	ELK3_2	cluster_4	SP4	6958	5110.06	4871	5297	136.0738	1.3616	0.4453	0.001	0.0011429	6107	55145	26.227	2198	19577	9.3107	777	6958	3.3092	12.7231	12.6176	35.3503	35.5417
DP	SMAD_1	cluster_6	Common_2	53906	39603.949	39215	39975	221.8464	1.3611	0.4448	0.001	0.0010435	15462	123736	56.699	8902	70228	32.18	6777	53906	24.701	43.83	43.5653	76.129	76.7586
DP	SMAD3_1	cluster_6	Common_2	64307	47249.49	46835	47635	247.3172	1.361	0.4447	0.001	0.0010435	19225	159039	54.071	10800	88038	29.932	7875	64307	21.864	40.9623	40.4347	72.9167	73.0446
DP	MGA_2	cluster_6	Common_2	33253	24432.884	23938	24872	293.7539	1.361	0.4446	0.001	0.0010909	4878	83018	54.568	2767	45310	29.782	1989	33253	21.857	40.7749	40.0552	71.8829	73.39
SP4	IRF_known14	cluster_4	SP4	54980	40397.535	39781	40984	364.4912	1.361	0.4446	0.001	0.001	13966	293131	66.532	3125	62308	14.142	2724	54980	12.479	19.5045	18.7561	87.168	88.2391

SP8	FLI1_2	cluster_1	SP8	7120	5231.358	5007	5459	130.3186	1.361	0.4446	0.001	0.001	6676	86936	61.84	671	8532	6.069	553	7120	5.0646	8.2834	8.1899	82.4143	83.4505
DP	TBX1_2	cluster_6	Common_2	29834	21926.644	21501	22433	271.6127	1.3606	0.4443	0.001	0.0010909	4390	79089	50.708	2536	43862	28.122	1682	29834	19.128	38.3144	37.7221	66.3249	68.0179
DP	MAF_known12	cluster_6	Common_2	54734	40235.384	39627	40795	361.5486	1.3603	0.444	0.001	0.0010435	7384	125901	57.08	4314	71119	32.243	3248	54734	24.815	43.987	43.4738	75.2898	76.9611
DN	TCF3_1	cluster_3	DN	81813	60145.636	59239	61179	605.1461	1.3602	0.4439	0.001	0.0011429	20600	290144	23.465	18450	258597	20.914	5895	81813	6.6166	28.6165	28.1974	31.9512	31.6373
DP	TBX2_1	cluster_6	Common_2	60920	44795.096	44253	45357	334.3362	1.36	0.4436	0.001	0.0010435	8226	140221	57.494	4773	78671	32.257	3619	60920	24.979	43.9947	43.4457	75.8223	77.4364
SP8	ARID5B_1	cluster_6	Common_2	23377	17190.101	16876	17506	203.0266	1.3599	0.4435	0.001	0.0010909	4547	59233	58.39	2368	29918	29.492	1828	23377	23.044	40.2023	39.4662	77.1959	78.1369
SP8	SOX_1	cluster_6	Common_2	30425	22373.109	22019	22722	216.5986	1.3599	0.4435	0.001	0.0010435	6625	79556	57.773	3385	39308	28.545	2580	30425	22.094	38.9434	38.2435	76.2186	77.4015
DP	RUNX3_3	cluster_6	Common_2	241862	177864.39	176830	178901	622.7781	1.3598	0.4434	0.001	0.001	23341	416133	63.949	15867	278975	42.871	13598	241862	37.168	58.258	58.1213	85.6999	86.6967
SP8	POU2F2_known12	cluster_6	Common_2	16370	12039.272	11815	12280	151.5882	1.3597	0.4433	0.001	0.0010435	4381	43877	60.114	2101	20388	27.933	1664	16370	22.428	37.9822	37.3088	79.2004	80.2923
DP	FOXO6_1	cluster_6	Common_2	10469	7699.827	7460	7973	150.8866	1.3596	0.4432	0.001	0.0010909	2247	29326	51.149	1244	15377	26.82	826	10469	18.26	36.7601	35.6987	66.3987	68.0822
SP8	ETV6_1	cluster_6	Common_2	207149	152359.491	151487	153314	537.9064	1.3596	0.4432	0.001	0.001	26153	376727	62.445	17137	244277	40.491	14385	207149	34.337	55.0033	54.9865	83.9412	84.8009
SP8	SMAD3_3	cluster_6	Common_2	14898	10956.961	10744	11174	125.6293	1.3597	0.4432	0.001	0.0010435	3767	33918	58.906	2115	18720	32.511	1672	14898	25.874	44.3855	43.9236	79.0544	79.5833
SP4	GMEB2_2	cluster_4	SP4	4009	2948.796	2827	3077	78.2675	1.3594	0.443	0.001	0.001	2773	36777	66.35	360	4500	8.1185	314	4009	7.2327	11.3235	10.9008	87.2222	89.0889
DP	KLF12_1	cluster_6	Common_2	61145	44986.551	44658	45288	200.1392	1.3592	0.4427	0.001	0.001	19413	116478	59.073	12826	76314	38.703	10229	61145	31.01	52.6915	52.4949	79.7521	80.1229
SP4	MYBL2_2	cluster_4	SP4	3984	2931.203	2796	3060	85.3379	1.359	0.4426	0.001	0.0010435	2320	35130	65.23	325	4617	8.5729	274	3984	7.3975	11.8103	11.3407	84.3077	86.2898
SP4	HES1_1	cluster_6	Common_2	200337	147450.108	146596	148237	512.3519	1.3587	0.4422	0.001	0.001	24677	349612	62.645	16910	236209	42.325	14216	200337	35.897	57.6083	57.3027	84.0686	84.8134
DP	NFKB_known10	cluster_6	Common_2	59702	43947.507	43536	44387	267.1617	1.3585	0.442	0.001	0.001	10029	121099	60.099	6222	73667	36.559	4995	59702	29.629	49.8056	49.3002	80.2797	81.0431
SP4	ARID5B_1	cluster_6	Common_2	23806	17523.834	17219	17868	198.0901	1.3585	0.442	0.001	0.0010909	4639	60416	59.556	2368	29918	29.492	1860	23806	23.467	40.0948	39.4035	78.5473	79.5708
SP4	SOX1_3	cluster_6	Common_2	15320	11277.289	11001	11549	163.436	1.3585	0.442	0.001	0.0010909	3151	44155	58.678	1464	19722	26.209	1116	15320	20.359	35.4173	34.696	76.2295	77.6797
SP8	IRF4_2	cluster_1	SP8	17195	12657.586	12398	12929	153.4931	1.3584	0.442	0.001	0.001	6695	96589	66.045	1355	19304	13.2	1197	17195	11.758	17.879	17.8022	88.3395	89.0748
SP8	XBP1_4	cluster_6	Common_2	37193	27381.099	27056	27706	200.2948	1.3583	0.4418	0.001	0.0010435	5176	67397	61.616	3479	44601	40.775	2875	37193	34.003	55.5448	55.1849	82.6387	83.3905
SP8	TBX2_2	cluster_6	Common_2	35149	25877.993	25498	26236	214.7456	1.3582	0.4417	0.001	0.0010909	9067	90775	56.983	4675	45779	28.737	3549	35149	22.065	39.1419	38.721	75.9144	76.7797
SP8	E2F2_3	cluster_1	SP8	3471	2555.493	2417	2692	90.023	1.3581	0.4416	0.001	0.001	2069	35210	66.193	247	3972	7.4671	211	3471	6.5253	10.1982	9.858	85.4251	87.3867
SP8	POU2F2_known15	cluster_6	Common_2	12879	9483.235	9265	9704	127.5992	1.358	0.4415	0.001	0.0010435	3075	33949	59.103	1524	16326	28.423	1192	12879	22.422	38.7642	37.9363	78.2152	78.8864
DP	SMAD3_3	cluster_6	Common_2	14136	10409.567	10221	10608	125.1651	1.3579	0.4414	0.001	0.0010435	3577	32203	55.927	2115	18720	32.511	1587	14136	24.55	44.3668	43.8965	75.0355	75.5128
DN	PBX1_4	cluster_3	DN	7603	5599.426	5417	5748	94.5348	1.3578	0.4412	0.001	0.0010909	3459	38700	71.853	745	7979	14.814	706	7603	14.116	20.4105	19.646	94.7651	95.2876
SP4	TBX2_2	cluster_6	Common_2	35977	26497.398	26146	26872	214.3646	1.3577	0.4412	0.001	0.0010909	9302	93136	58.465	4675	45779	28.737	3639	35977	22.584	39.1206	38.6285	77.8396	78.5884
SP8	ERG_4	cluster_6	Common_2	48941	36047.07	35626	36459	244.6944	1.3577	0.4412	0.001	0.001	6801	88600	61.768	4544	58432	40.736	3776	48941	34.119	55.5212	55.2381	83.0986	83.7572
DP	TBX15_1	cluster_6	Common_2	38104	28065.733	27612	28592	308.6823	1.3577	0.4411	0.001	0.0010909	5512	99328	52.536	3141	54275	28.707	2151	38104	20.154	39.0239	38.3618	68.4814	70.2054
DP	NFE2L1::MAFG_1	cluster_6	Common_2	63016	46419.622	45703	47196	471.2149	1.3575	0.441	0.001	0.0010435	8197	173116	53.265	4408	88615	27.265	3046	63016	19.389	37.1599	36.401	69.1016	71.1121
SP8	MYBL1_3	cluster_6	Common_2	7488	5515.869	5352	5677	89.8264	1.3575	0.4409	0.001	0.0010909	1462	16095	60.713	844	9130	34.44	688	7488	28.246	47.0588	46.5238	81.5166	82.0153
SP8	FOXM1_1	cluster_6	Common_2	16612	12238.317	12044	12448	131.8035	1.3573	0.4408	0.001	0.0010909	4986	39915	56.233	2799	21955	30.931	2102	16612	23.403	42.158	41.6184	75.0982	75.6639
SP8	SOX1_2	cluster_6	Common_2	16545	12189.835	11884	12474	178.7429	1.3572	0.4407	0.001	0.0010909	3221	45113	56.297	1650	22104	27.584	1212	16545	20.647	37.6281	36.6746	73.4545	74.8507
SP8	ERG_2	cluster_6	Common_2	48464	35711.181	35298	36113	245.898	1.3571	0.4405	0.001	0.001	6800	88560	61.21	4547	58454	40.401	3742	48464	33.497	55.0294	54.7245	82.296	82.9096
SP8	FLI1_2	cluster_6	Common_2	49166	36236.199	35802	36643	246.9959	1.3568	0.4402	0.001	0.001	6676	86936	61.84	4558	58681	41.741	3792	49166	34.973	56.8005	56.5542	83.1944	83.7852
DP	FOXM1_1	cluster_6	Common_2	15824	11664.88	11445	11917	136.9707	1.3565	0.4399	0.001	0.0010909	4728	37858	53.335	2799	21955	30.931	1992	15824	22.293	42.132	41.7983	71.1683	72.0747
SP4	ATF4_3	cluster_6	Common_2	20728	15286.076	14960	15584	187.1786	1.356	0.4393	0.001	0.0010909	3989	51908	59.647	2058	25990	29.865	1615	20728	23.818	40.4863	39.9322	78.4742	79.7538
SP4	MAF_known10	cluster_6	Common_2	35997	26547.177	26202	26938	224.4489	1.3559	0.4393	0.001	0.0010909	8761	96521	59.023	4229	45435	27.784	3308	35997	22.012	37.7582	37.2945	78.2218	79.2275
DP	MAFG_1	cluster_6	Common_2	46085	33990.215	33395	34595	366.3535	1.3558	0.4392	0.001	0.0010435	5947	119184	52.184	3395	65678	28.757	2322	46085	20.178	39.0449	38.6671	68.3947	70.1681
DP	PKNOX2_2	cluster_6	Common_2	40089	29573.958	29105	30013	280.2756	1.3555	0.4389	0.001	0.0010435	11043	122680	50.42	5428	59074	24.279	3650	40089	16.476	33.0526	32.6777	67.2439	67.8623
DP	SOX10_4	cluster_6	Common_2	26764	19743.984	19328	20141	239.6361	1.3555	0.4389	0.001	0.0010909	4770	71762	51.378	2719	39147	28.027	1814	26764	19.162	38.0294	37.2955	66.7157	68.3679
SP4	TBX21_4	cluster_6	Common_2	42820	31594.264	31071	32093	299.0732	1.3553	0.4386	0.001	0.0010909	7892	118588	58.407	3809	55074	27.125	2900	42820	21.09	36.7461	36.1082	76.1355	77.7499
DP	ETV2_1	cluster_6	Common_2	68278	50382.458	49879	50862	305.0099	1.3552	0.4385	0.001	0.0010435	15356	154289	52.346	9620	95999	32.57	6785	68278	23.165	44.1847	44.2533	70.5301	71.1237
DP	MESP1_1	cluster_6	Common_2	104751	77318.494	76728	77948	378.2292	1.3548	0.4381	0.001	0.001	32605	294479	56.054	15538	138355	26.336	11664	104751	19.939	35.7737	35.5716	75.0676	75.7118
DP	PKNOX2_3	cluster_6	Common_2	35680	26335.37	25998	26775	240.4744	1.3548	0.4381	0.001	0.0010435	9600	106895	49.891	4884	53142	24.803	3244	35680	16.653	33.7917	33.3785	66.421	67.1409
SP4	POU2F2_known14	cluster_6	Common_2	5195	3834.811	3658	3990	96.9425	1.3546	0.4379	0.001	0.0012632	922	14165	62.953	474	6572	29.208	363	5195	23.088	39.3709	36.6749	76.5823	79.0475
SP4	SPIB_2	cluster_6	Common_2	95428	70464.386	69779	71112	401.8419	1.3543	0.4375	0.001	0.0010435	18724	244697	60.687	9156	117165	29.058	7381	95428	23.667	39.42	38.9984	80.6138	81.4475
DP	SPZ1_2	cluster_6	Common_2	59746	44124.545	43699	44555	272.0794	1.354	0.4373	0.001	0.0010435	13790	138253	56.134	8044	79031	32.088	6007	59746	24.258	43.5606	43.215	74.6768	75.5982
SP8	ETV2_1	cluster_6	Common_2	73580	54338.1	53822	54856	323.7273	1.3541	0.4373	0.001	0.0010435	16594	166680	56.55	9620	95999	32.57	7324	73580	24.964	44.1364	44.1445	76.1331	76.6466
DP	RUNX3_3	cluster_2	DP	53913	39822.09	39164	40443	386.8997	1.3538	0.4371	0.001	0.001	23341	416133	63.949	3611	62662	9.6295	3076	53913	8.285	13.1785	12.9557	85.1842	86.0378
SP8	SOX7_4	cluster_6	Common_2	14986	11068.972	10756	11355	168.132	1.3538	0.4371	0.001	0.0010909	2647	42472	56.757	1326	20183	26.971	968	14986	20.026	36.5697	35.2844	73.0015	74.2506
DN	FOXO3_1	cluster_3	DN	14188	10481.051	10282	10702	132.4222	1.3536	0.4369	0.001	0.0010435	6232	81801	74.85	1195	14663	13.417	1150	14188	12.982	18.4531	17.3445	96.2343	96.7606
SP4	MTF1_3	cluster_6	Common_2	82476	60925.66	60349	61524	342.008	1.3537	0.4369	0.001	0.001	12953	170029	62.803	7551	97546	36.03	6333	82476	30.464	48.8921	48.507	83.8697	84.5509
SP8	HES1_1	cluster_6	Common_2	203467	150308.572	149451	151173	532.8168	1.3537	0.4369	0.001	0.001	25160	356406	63.862	16910	236209	42.325	14448	203467	36.458	57.4245	57.0885	85.4406	86.1385
DN	SOX11_2	cluster_3	DN	13626	10066.249	9853	10306	138.2549	1.3536	0.4368	0.001	0.0010909	4488	62863	74.087	1065	14154	16.681	1022	13626	16.059	22.7718	21.6757	95.9624	96.2696
SP8	RUNX3_3	cluster_6	Common_2	245492	181364.8	180444	182336	571.0834	1.3536	0.4368	0.001	0.001	23891	424439	65.225	15867	278975	42.871	13835	245492	37.726	57.9088	57.8392	87.1935	87.9978
DN	SOX14_2	cluster_3	DN	9710	7175.197	7002	7335	106.9665	1.3532	0.4364	0.001	0.0010909	4224	46656	72.886	952	10109	15.792	910	9710	15.169	21.5436	20.8119	95.5882	96.053
SP4	CTCF_known1	cluster_6	Common_2	578138	427216.158	425489	429047	1065.5265	1.3533	0.4364	0.001	0.0010435	55221	1020372	58.86	39885	727160	41.946	31407	578138	33.35	56.8751	56.6595	78.7439	79.5063
DP	LMO2_1	cluster_6	Common_2	153062	113114.18	112284	113946	508.6219	1.3532	0.4363	0.001	0.001	40696	452099	54.045	19245	210148	25.122	13876	153062	18.297	34.0967	33.8559	72.1018	72.8353
SP4	E2F8_1	cluster_6	Common_2	41540	30705.088	30377	31033	209.3196	1.3529	0.436	0.001	0.001	7471	82374	63.953	4519	48700	37.81	3819	41540	32.251	51.1177	50.4285	84.5098	85.2977
SP4	SPI1_known1	cluster_6	Common_2	102339	75658.44	75104	76164	311.8592	1.3526	0.4358	0.001	0.001	38052	267768	57.339	18984	132280	28.326	14610	102339	21.915	38.3948	38.2193	76.9595	77.3654
SP8	E2F2_2	cluster_1	SP8	6865	5075.388	4857	5280	129.814	1.3525	0.4357	0.001	0.0010909	3179	54200	66.243	488	7854	9.5991	421	6865	8.3904	13.2432	12.6661	86.2705	87.4077
SP8	SPDEF_4	cluster_6	Common_2	40043	29613.134	29193	30054	266	1.3522	0.4353	0.001	0.0010435	7014	105694	58.627	3617	51863	28.768	2730	40043	22.211	38.9222	37.8858	75.4769	77.2092
SP4	ETS_known17	cluster_6	Common_2	119611	88464.604	87751	89207	429.6415	1.3521	0.4352	0.001	0.001	12925	220877	62.412	8506	142533	40.275	7049	119611	33.798	54.5377	54.1528	82.8709	83.9181
SP8	POU2F3_2	cluster_6	Common_2	10137	7496.779	7342	7670	102.6949	1.3521	0.4352	0.001	0.0010909	3384	27131	59.282	1627	12784	27.933	1277	10137	22.15	37.7364	37.3632	78.488	79.2944
SP8	E2F8_1	cluster_6	Common_2	41984	31052.34	30704	31358	205.3185	1.352	0.4351	0.001	0.001	7551	83269	64.648	4519	48700	37.81	3862	41984	32.596	51.1455	50.4197	85.4614	86.2094
SP8	BPTF_1	cluster_6	Common_2	63756	47159.087	46629	47715	320.0842	1.3519	0.435	0.001	0.0010909	12575	168522	60.074	6263	80341	28.64	4909	63756	22.728	39.0378	37.8324	78.381	79.3567
SP8	MTF1_1	cluster_1	SP8	34629	25617.123	25202	25985	242.1773	1.3518	0.4349	0.001	0.001	38926	519131	65.795	3054	39477	5.0033	2656	34629	4.3889	6.8232	6.6706	86.9679	87.7194
DP	TBX20_5	cluster_6	Common_2	70526	52185.87	51580	52814	382.7418	1.3514	0.4345	0.001	0.0010909	11935	182788	54.157	6610	97846	28.99	4681	70526	20.896	39.2208	38.5835	70.8169	72.0786
DP	MAF_known10	cluster_6	Common_2	32426	23995.092	23638	24351	225.4656	1.3513	0.4344	0.001	0.0010909	7909	87167	53.303	4229	45435	27.784	2976	32426	19.829	37.628	37.1999	70.3712	71.3679
SP4	PBX3_known1	cluster_6	Common_2	33905	25092.904	24715	25456	230.593	1.3512	0.4342	0.001	0.0010435	7076	78591	58.439	4029	43510	32.354	3096	33905	25.211	43.7535	43.1411	76.8429	77.9246
SP4	SPIB_1	cluster_6	Common_2	39895	29526.829	29198	29838	200.2483	1.3511	0.4342	0.001	0.0010435	18137	108822	58.346	8514	50762	27.217	6682	39895	21.39	36.8418	36.6608	78.4825	78.5923
SP4	SP1_known3	cluster_4	SP4	43689	32344.212	31905	32827	277.6934	1.3507	0.4338	0.001	0.001	39133	513297	64.396	4045	50952	6.3922	3424	43689	5.481	8.7496	8.5114	84.6477	85.7454
DP	ATF2_2	cluster_2	DP	6332	4687.947	4500	4846	103.4778	1.3506	0.4336	0.001	0.0010435	4893	53941	62.848	694	7476	8.7104	582	6332	7.3775	11.8945	11.7388	83.8617	84.6977
DN	CUX1_9	cluster_3	DN	2232	1652.507	1554	1748	61.0276	1.3505	0.4335	0.001	0.0012632	631	10867	73.396	151	2358	15.926	143	2232	15.075	22.6624	20.5392	94.702	94.6565
DP	E2F_known26	cluster_6	Common_2	13354	9888.625	9633	10143	154.4333	1.3504	0.4334	0.001	0.0010435	1515	25773	59.594	1040	16856	38.975	801	13354	30.878	52.8713	51.8139	77.0192	79.224
DP	TCF4_2	cluster_6	Common_2	102136	75640.107	75023	76220	373.5945	1.3503	0.4333	0.001	0.001	32705	295539	54.979	15507	138006	25.673	11366	102136	19	34.7531	34.5592	73.2959	74.0084
DN	SOX13_1	cluster_3	DN	7793	5771.559	5610	5956	99.4498	1.3502	0.4332	0.001	0.0010909	2495	37512	75.346	581	8084	16.237	558	7793	15.653	22.3647	20.7747	96.0413	96.4003
SP8	PBX1_2	cluster_6	Common_2	15673	11607.759	11344	11886	158.5369	1.3502	0.4332	0.001	0.0010435	2880	41061	58.486	1495	20344	28.977	1129	15673	22.324	39.2014	38.17	75.5184	77.0399
DP	HESX1_2	cluster_6	Common_2	4057	3004.694	2859	3168	91.2732	1.3501	0.4331	0.001	0.0014118	733	10262	51.563	451	5980	30.047	298	4057	20.385	40.6548	39.5342	66.0754	67.8428
SP8	SOX2_5	cluster_6	Common_2	18411	13636.206	13312	14021	202.4014	1.3501	0.4331	0.001	0.0010909	3178	50901	57.693	1609	24458	27.721	1190	18411	20.868	37.4449	36.1702	73.959	75.276
DP	TBX2_2	cluster_6	Common_2	32869	24347.217	24031	24713	211.0738	1.35	0.433	0.001	0.0010909	8541	85538	53.696	4675	45779	28.737	3321	32869	20.633	38.883	38.4262	71.0374	71.7993
DP	ERG_4	cluster_6	Common_2	48185	35700.463	35325	36062	236.7317	1.3497	0.4326	0.001	0.001	6744	87870	61.259	4544	58432	40.736	3717	48185	33.592	55.1157	54.8367	81.8002	82.4634
DP	MAF_known11	cluster_6	Common_2	70222	52035.392	51377	52691	385.868	1.3495	0.4324	0.001	0.0010435	10294	166060	54.212	6133	96460	31.49	4393	70222	22.925	42.6753	42.2871	71.6289	72.7991
SP8	MYCN_1	cluster_6	Common_2	65566	48586.625	48178	49023	260.2211	1.3495	0.4324	0.001	0.001	10432	115067	63.978	6966	76024	42.27	5969	65566	36.455	57.2182	56.9807	85.6876	86.2438
DP	XBP1_4	cluster_2	DP	7889	5846.748	5616	6097	138.9751	1.3492	0.4321	0.001	0.0010435	4834	62945	57.546	808	10192	9.3178	616	7889	7.2123	12.7431	12.5332	76.2376	77.4038
SP8	XBP1_2	cluster_6	Common_2	30524	22623.816	22151	23110	286.4217	1.3492	0.4321	0.001	0.0010435	2655	61164	63.418	1647	36240	37.575	1353	30524	31.649	50.9605	49.9052	82.1494	84.2274
DP	SOX18_4	cluster_6	Common_2	9006	6675.194	6411	6914	150.4038	1.3491	0.432	0.001	0.0012	1791	25128	47.709	1070	14264	27.082	659	9006	17.099	36.7951	35.8405	61.5888	63.138
SP4	E2F_known24	cluster_4	SP4	6064	4496.495	4290	4700	118.8042	1.3485	0.4314	0.001	0.001	3037	51804	67.185	431	6870	8.9098	375	6064	7.8645	12.3477	11.7057	87.007	88.2678
DN	SOX21_3	cluster_3	DN	13953	10347.808	10151	10553	128.9063	1.3484	0.4312	0.001	0.0010909	4326	64953	73.757	1020	14522	16.49	977	13953	15.844	22.5844	21.4817	95.7843	96.0818
SP8	POU2F2_known5	cluster_6	Common_2	17548	13013.813	12732	13281	172.7148	1.3484	0.4312	0.001	0.0010909	3679	47888	58.57	1820	22745	27.819	1383	17548	21.462	37.5917	36.6438	75.989	77.151
DP	STAT_known3	cluster_2	DP	15898	11795.595	11454	12135	217.6967	1.3478	0.4306	0.001	0.0010435	6852	137400	63.695	997	18856	8.7411	822	15898	7.3699	11.9965	11.5706	82.4473	84.3127
SP4	RUNX3_3	cluster_4	SP4	22570	16746.18	16338	17170	255.4552	1.3477	0.4306	0.001	0.001	22811	405311	62.285	1632	27443	4.2173	1325	22570	3.4684	5.8086	5.5686	81.1887	82.2432
DP	TCF4_1	cluster_6	Common_2	109631	81347.872	80628	81949	384.2848	1.3477	0.4305	0.001	0.0010909	39369	356288	54.334	16835	150077	22.887	12196	109631	16.719	30.9787	30.7703	72.4443	73.0498
SP4	TBX19_1	cluster_6	Common_2	27948	20737.504	20338	21175	257.309	1.3477	0.4305	0.001	0.0010435	3815	72708	56.872	2041	37143	29.053	1497	27948	21.861	39.2398	38.4387	73.3464	75.2443
DP	ETS_known17	cluster_6	Common_2	115455	85671.531	84997	86378	441.5608	1.3476	0.4304	0.001	0.001	12494	213553	60.343	8506	142533	40.275	6794	115455	32.624	54.3781	54.0639	79.873	81.0023
DP	ERG_2	cluster_6	Common_2	47477	35237.682	34801	35703	261.1997	1.3473	0.4301	0.001	0.001	6712	87427	60.427	4547	58454	40.401	3664	47477	32.814	54.5888	54.3047	80.5806	81.2211
DP	TCF3_2	cluster_6	Common_2	124171	92169.571	91275	93134	544.2956	1.3472	0.43	0.001	0.0010435	27591	417427	54.427	11592	170739	22.262	8300	124171	16.19	30.0823	29.7468	71.6011	72.7256
SP4	BPTF_1	cluster_6	Common_2	64732	48048.781	47488	48531	321.9223	1.3472	0.43	0.001	0.0010909	12832	172114	61.355	6263	80341	28.64	4985	64732	23.076	38.8482	37.61	79.5944	80.5716
DP	HES5_1	cluster_6	Common_2	54003	40088.111	39626	40521	269.158	1.3471	0.4299	0.001	0.0010435	9609	106096	60.849	6066	66167	37.949	4916	54003	30.972	51.1604	50.9001	81.0419	81.6162
DP	IKZF1_2	cluster_6	Common_2	71941	53403.201	52908	53841	277.4915	1.3471	0.4299	0.001	0.0010435	20144	161159	58.804	11645	91533	33.399	9073	71941	26.25	45.0407	44.6398	77.9133	78.5957
DN	SOX_1	cluster_3	DN	20642	15325.064	15059	15571	149.864	1.3469	0.4297	0.001	0.0010435	8206	98530	71.552	1911	21909	15.91	1789	20642	14.99	21.8011	20.95	93.6159	94.217
SP8	FLI1_4	cluster_6	Common_2	52913	39282.704	38874	39717	240.4424	1.347	0.4297	0.001	0.001	7101	92567	63.122	4842	62328	42.502	4080	52913	36.082	57.4567	57.1618	84.2627	84.8944
DN	SOX5_1	cluster_3	DN	4881	3624.301	3526	3726	63.9299	1.3466	0.4294	0.001	0.0010909	2705	24345	72.35	590	5116	15.204	559	4881	14.506	20.6654	20.0493	94.7458	95.4066
DP	TBX5_3	cluster_6	Common_2	50184	37266.902	36835	37718	265.6905	1.3466	0.4293	0.001	0.001	14895	134213	54.181	7844	69272	27.965	5622	50184	20.259	37.7442	37.3913	71.6726	72.4449
SP4	TBX1_2	cluster_6	Common_2	33402	24806.118	24313	25293	293.4864	1.3465	0.4292	0.001	0.0010909	4990	89894	57.636	2536	43862	28.122	1885	33402	21.416	37.7756	37.1571	74.3297	76.1525
DP	ATF2_2	cluster_6	Common_2	31906	23698.407	23411	23984	175.0525	1.3463	0.429	0.001	0.0010435	4893	53941	62.848	3437	37664	43.883	2897	31906	37.174	59.207	59.1498	84.2886	84.7122
SP4	SOX14_3	cluster_6	Common_2	16876	12535.8	12263	12822	173.9729	1.3462	0.4289	0.001	0.0010909	3418	47906	58.588	1626	21909	26.794	1230	16876	20.639	35.986	35.2273	75.6458	77.0277
SP4	XBP1_2	cluster_4	SP4	8931	6635.234	6375	6902	162.9563	1.3459	0.4286	0.001	0.0010435	2736	63041	65.364	478	10402	10.785	400	8931	9.2601	14.6199	14.167	83.682	85.8585
SP8	POU2F2_known11	cluster_6	Common_2	16284	12100.339	11885	12325	131.38	1.3457	0.4284	0.001	0.0010435	4428	44372	59.446	2129	20693	27.723	1652	16284	21.816	37.308	36.6988	77.5951	78.6933
DP	SOX4_2	cluster_6	Common_2	21652	16091.154	15757	16440	203.6792	1.3456	0.4282	0.001	0.0010909	3926	59025	51.393	2240	32027	27.886	1482	21652	18.852	37.7483	36.6828	66.1607	67.6055
SP4	E2F_known26	cluster_4	SP4	3669	2727.464	2586	2874	85.797	1.3451	0.4277	0.001	0.0010435	1689	28742	66.459	267	4221	9.76	233	3669	8.4836	13.7951	12.7653	87.2659	86.9225
SP4	SPI1_known4	cluster_6	Common_2	77851	57884.998	57322	58466	332.1288	1.3449	0.4275	0.001	0.0010435	15528	202516	62.03	7415	94443	28.928	6045	77851	23.846	38.9297	38.4419	81.5239	82.4317
DN	EGR3_2	cluster_3	DN	24525	18236.445	17614	18813	345.5204	1.3448	0.4274	0.001	0.001	13370	205199	20.858	6402	91970	9.3486	1734	24525	2.4929	12.9693	11.9518	27.0853	26.6663
DP	GMEB2_2	cluster_2	DP	4150	3085.773	2944	3239	89.6104	1.3448	0.4274	0.001	0.001	2591	34443	62.139	402	5050	9.1108	323	4150	7.4871	12.4662	12.0489	80.3483	82.1782
DP	RREB1_2	cluster_6	Common_2	516971	384459.399	382376	386539	1240.3047	1.3447	0.4273	0.001	0.001	48689	1172396	58.91	30076	678272	34.082	22512	516971	25.977	46.2363	44.0953	74.8504	76.2188
DN	SOX12_1	cluster_3	DN	9639	7169.896	6986	7353	108.0757	1.3443	0.4269	0.001	0.0010435	3600	46915	74.344	813	10059	15.94	776	9639	15.275	21.5556	20.5457	95.449	95.8246
SP4	ETV7_1	cluster_6	Common_2	94209	70102.188	69547	70619	335.7193	1.3439	0.4264	0.001	0.001	27857	251236	56.588	13931	124176	27.969	10481	94209	21.219	37.6243	37.4982	75.2351	75.8673
DN	RUNX1_2	cluster_3	DN	17836	13273.283	12997	13532	160.5957	1.3437	0.4262	0.001	0.0010435	5376	83982	74.445	1268	18623	16.508	1204	17836	15.811	22.3958	21.2379	94.9527	95.774
DP	ID4_1	cluster_6	Common_2	99966	74395.606	73817	75062	374.0728	1.3437	0.4262	0.001	0.0010435	35081	317583	54.366	15416	137254	23.496	11130	99966	17.113	31.7266	31.4771	72.1977	72.8329
SP8	SP1_known3	cluster_1	SP8	44475	33098.048	32619	33614	296.8053	1.3437	0.4262	0.001	0.001	39576	519194	65.135	4032	51385	6.4465	3455	44475	5.5796	8.73	8.5662	85.6895	86.5525
SP8	MYCN_2	cluster_6	Common_2	94598	70413.686	70023	70893	274.1138	1.3435	0.4259	0.001	0.001	17431	157501	65.162	12049	108167	44.751	10492	94598	39.137	60.1916	60.0618	87.0778	87.4555
SP4	MYCN_1	cluster_6	Common_2	64642	48126.901	47734	48488	238.8129	1.3432	0.4256	0.001	0.001	10339	114073	63.425	6966	76024	42.27	5887	64642	35.941	56.9397	56.6672	84.5105	85.0284
SP8	ATF7_1	cluster_6	Common_2	22197	16529.88	16183	16832	184.1823	1.3428	0.4253	0.001	0.001	3427	44593	61.045	2132	27214	37.254	1716	22197	30.386	50.073	49.7769	80.4878	81.5646
SP4	MYCN_2	cluster_6	Common_2	93549	69670.619	69230	70088	263.6873	1.3427	0.4252	0.001	0.001	17249	155885	64.493	12049	108167	44.751	10372	93549	38.703	60.131	60.0115	86.0818	86.4857

SP8	MTF1_1	cluster_6	Common_2	293926	218926.625	217994	219858	559.2614	1.3426	0.425	0.001	0.001	38926	519131	65.795	25336	334415	42.384	22161	293926	37.252	56.9311	56.6188	87.4684	87.8926
SP8	MYBL1_5	cluster_1	SP8	4246	3162.444	2980	3330	102.0524	1.3425	0.4249	0.001	0.0010435	2849	45954	65.783	322	4890	7	277	4246	6.0781	9.7227	9.2397	86.0248	86.8303
SP4	MGA_3	cluster_6	Common_2	35793	26663.155	26195	27069	269.7684	1.3424	0.4248	0.001	0.0010909	5104	86900	61.334	2681	44055	31.094	2133	35793	25.263	41.7908	41.1887	79.5599	81.2462
SP4	ETS_known17	cluster_4	SP4	24031	17907.535	17448	18317	260.5331	1.3419	0.4243	0.001	0.001	12925	220877	62.412	1774	29054	8.2097	1436	24031	6.7903	11.1103	10.8798	80.947	82.7115
SP8	TCF7L1_2	cluster_6	Common_2	43381	32327.848	31942	32742	236.7547	1.3419	0.4243	0.001	0.0010435	11685	129185	56.684	5380	57741	25.336	3987	43381	19.035	34.1207	33.5805	74.1078	75.1303
DN	HBP1_1	cluster_3	DN	15951	11886.978	11628	12131	156.7738	1.3419	0.4242	0.001	0.0010435	5153	79302	73.662	1145	16681	15.495	1089	15951	14.817	21.1333	20.1142	95.1092	95.6238
DP	SMAD_2	cluster_6	Common_2	83156	61976.03	61400	62543	353.8837	1.3417	0.4241	0.001	0.0010435	20655	212873	53.488	11532	116795	29.347	8185	83156	20.895	39.6272	39.0637	70.9764	71.1983
SP4	XBP1_4	cluster_4	SP4	7610	5671.652	5448	5878	125.7227	1.3417	0.4241	0.001	0.0010435	5207	67800	61.985	739	9250	8.4566	594	7610	6.9573	11.4077	11.2242	80.3789	82.2703
SP8	FLI1_4	cluster_1	SP8	7374	5495.806	5275	5695	121.6404	1.3417	0.424	0.001	0.001	7101	92567	63.122	690	8773	5.9824	574	7374	5.0284	8.0834	7.9661	83.1884	84.0533
SP8	TCF7L2_known5	cluster_6	Common_2	53990	40254.512	39714	40865	340.7187	1.3412	0.4235	0.001	0.0010435	10823	173781	55.479	4751	73608	23.499	3420	53990	17.236	31.5994	31.0678	71.9848	73.348
DP	HES7_1	cluster_6	Common_2	57920	43198.722	42790	43596	245.4848	1.3408	0.4231	0.001	0.0010435	9992	110416	62.326	6360	69516	39.239	5264	57920	32.694	52.6821	52.4562	82.7673	83.3189
DN	SOX21_2	cluster_3	DN	13719	10234.323	9964	10462	144.4991	1.3405	0.4227	0.001	0.0010909	4666	65357	73.59	1089	14396	16.21	1030	13719	15.447	22.0746	20.9909	94.5822	95.2973
SP8	E2F7_1	cluster_6	Common_2	29483	21995.584	21668	22329	195.2004	1.3404	0.4227	0.001	0.0010435	4510	58679	64.962	2740	34586	38.289	2307	29483	32.64	51.153	50.2446	84.1971	85.2455
SP4	LMO2_2	cluster_6	Common_2	12770	9530.657	9341	9701	109.3193	1.3399	0.4221	0.001	0.0010435	3781	30272	59.431	2056	16178	31.761	1612	12770	25.071	42.6342	42.1842	78.4047	78.9344
SP8	NFATC2_1	cluster_6	Common_2	15008	11201.161	11028	11372	101.6576	1.3398	0.4221	0.001	0.0010909	5758	34548	61.898	3099	18285	32.761	2533	15008	26.889	43.991	43.441	81.736	82.0782
SP8	ATF6_1	cluster_6	Common_2	15631	11667.376	11499	11851	104.4461	1.3397	0.4219	0.001	0.0010435	3930	27520	63.816	2634	18311	42.461	2241	15631	36.247	57.0229	56.7987	85.0797	85.364
SP4	SPIC_2	cluster_6	Common_2	84561	63122.588	62525	63676	353.9408	1.3396	0.4218	0.001	0.0010435	17268	225108	60.689	8230	104958	28.297	6556	84561	22.798	37.9662	37.5646	79.6598	80.5665
DN	SOX5_3	cluster_3	DN	9275	6923.973	6724	7115	121.4873	1.3395	0.4217	0.001	0.0011429	2922	43910	74.555	681	9692	16.456	651	9275	15.748	22.2793	21.1228	95.5947	95.6975
DP	IKZF2_2	cluster_6	Common_2	14423	10768.258	10525	10995	146.9339	1.3394	0.4216	0.001	0.0010435	3311	33116	55.275	2039	19720	32.915	1470	14423	24.074	44.3975	43.553	72.0942	73.1389
SP4	XBP1_4	cluster_6	Common_2	36879	27539.758	27166	27906	223.3931	1.3391	0.4213	0.001	0.0010435	5207	67800	61.985	3479	44601	40.775	2850	36879	33.716	54.734	54.3938	81.9201	82.6865
DN	EWSR1::FLI1_1	cluster_3	DN	228286	170500.809	169360	171730	734.9998	1.3389	0.4211	0.001	0.0010435	56590	1127364	69.149	13531	254490	15.61	11933	228286	14.002	21.0868	20.2495	88.1901	89.7033
SP4	SPIC_1	cluster_6	Common_2	78852	58890.487	58329	59506	364.8081	1.339	0.4211	0.001	0.001	16203	211420	60.042	7805	99240	28.184	6126	78852	22.394	37.8078	37.2964	78.4881	79.4559
SP8	POU2F2_known10	cluster_6	Common_2	16367	12224.707	11998	12465	135.5028	1.3388	0.421	0.001	0.0010435	4821	43499	59.761	2357	20734	28.485	1836	16367	22.486	38.0834	37.6262	77.8956	78.938
SP8	POU2F2_known16	cluster_6	Common_2	11663	8713.244	8470	8970	162.1423	1.3385	0.4206	0.001	0.0010909	2431	32048	54.51	1305	16413	27.917	913	11663	19.837	37.5566	36.3923	69.9617	71.0595
SP8	CTCF_known1	cluster_6	Common_2	594270	444067.201	442289	445974	1136.5557	1.3382	0.4203	0.001	0.0010435	57414	1061210	61.215	39885	727160	41.946	32289	594270	34.28	56.2389	55.9993	80.9552	81.7248
SP8	TBX20_5	cluster_6	Common_2	75523	56434.726	55730	57126	387.8004	1.3382	0.4203	0.001	0.0010909	12908	197715	58.58	6610	97846	28.99	5025	75523	22.376	38.9293	38.1979	76.0212	77.1856
DN	CUX1_8	cluster_3	DN	1952	1459.25	1373	1549	56.467	1.3374	0.4195	0.001	0.0011429	619	9964	75.019	143	2072	15.6	135	1952	14.697	21.8094	19.5905	94.4056	94.2085
SP4	RREB1_2	cluster_6	Common_2	542998	406007.631	404057	408112	1284.9029	1.3374	0.4194	0.001	0.001	51020	1245776	62.597	30076	678272	34.082	23582	542998	27.284	46.2211	43.5871	78.408	80.0561
SP8	SOX3_3	cluster_6	Common_2	14802	11067.892	10711	11376	199.4816	1.3374	0.4194	0.001	0.0010909	2699	43266	55.718	1342	20452	26.338	954	14802	19.062	35.3464	34.2116	71.0879	72.3743
DP	FOXO4_2	cluster_6	Common_2	30219	22598.127	22210	22928	220.5508	1.3372	0.4192	0.001	0.0010435	5614	73565	57.489	3236	40377	31.554	2367	30219	23.615	42.1625	41.078	73.1459	74.8421
DN	FOXP3_1	cluster_3	DN	35362	26448.818	26015	26854	253.5824	1.337	0.419	0.001	0.0010909	9966	159936	72.213	2429	37397	16.885	2276	35362	15.966	22.8376	22.1101	93.7011	94.5584
SP8	RUNX3_3	cluster_1	SP8	37729	28219.206	27772	28631	275.8988	1.337	0.419	0.001	0.001	23891	424439	65.225	2618	44080	6.7739	2203	37729	5.7979	9.221	8.8891	84.1482	85.5921
SP4	MTF1_1	cluster_6	Common_2	290638	217441.052	216552	218485	593.2425	1.3366	0.4186	0.001	0.001	38636	515202	65.297	25336	334415	42.384	21901	290638	36.836	56.6855	56.4124	86.4422	86.9094
SP8	SOX4_1	cluster_6	Common_2	71853	53762.753	53038	54485	424.7022	1.3365	0.4184	0.001	0.0010435	11786	191082	60.682	5901	90966	28.888	4569	71853	22.818	38.7663	37.6032	77.4276	78.9889
DP	FOXO1_4	cluster_6	Common_2	8957	6702.347	6465	6948	139.8135	1.3363	0.4183	0.001	0.0010909	1950	25417	51.042	1097	13493	27.097	711	8957	17.987	36.4615	35.2402	64.8131	66.3826
SP4	KLF13_1	cluster_6	Common_2	208818	156263.548	155275	157179	550.7815	1.3363	0.4183	0.001	0.001	20223	347988	63.027	14628	248271	44.967	12189	208818	37.821	60.273	60.0072	83.3265	84.1089
SP8	IRF_known14	cluster_6	Common_2	117737	88110.497	87223	89052	557.2737	1.3362	0.4182	0.001	0.001	13279	278624	63.239	7220	143449	32.558	5795	117737	26.723	43.6403	42.2566	80.2632	82.0759
SP8	SOX11_1	cluster_6	Common_2	65733	49202.932	48579	49832	374.8577	1.336	0.4179	0.001	0.0010435	10921	177105	60.65	5407	83403	28.561	4178	65733	22.51	38.2566	37.1153	77.2702	78.8137
SP8	TCF7L2_known7	cluster_6	Common_2	49159	36798.988	36289	37277	303.5923	1.3359	0.4178	0.001	0.0010435	11335	159258	55.926	4944	66905	23.495	3564	49159	17.263	31.4424	30.8675	72.0874	73.4758
DN	GATA_known16	cluster_3	DN	29177	21842.92	21462	22227	233.7555	1.3357	0.4176	0.001	0.0010435	6076	128052	74.197	1533	30496	17.67	1453	29177	16.906	23.9138	22.7853	94.7815	95.6748
DP	HES7_1	cluster_2	DP	17788	13316.81	13044	13596	173.1971	1.3357	0.4176	0.001	0.0010435	9992	110416	62.326	1979	21462	12.114	1625	17788	10.041	16.263	16.11	82.1122	82.8814
SP8	SOX10_9	cluster_6	Common_2	17506	13107.16	12793	13439	197.6616	1.3356	0.4175	0.001	0.0010435	3412	47797	56.857	1761	23524	27.983	1286	17506	20.824	37.6905	36.6257	73.0267	74.4176
SP8	ETS_known13	cluster_6	Common_2	98563	73809.082	73175	74409	365.1854	1.3354	0.4172	0.001	0.001	10079	161754	66.163	7079	111951	45.792	6177	98563	40.316	61.2858	60.9339	87.2581	88.0412
SP4	IRF4_2	cluster_4	SP4	18343	13737.466	13462	14016	168.0346	1.3352	0.4171	0.001	0.001	6862	99125	67.779	1455	20483	14.006	1294	18343	12.542	18.8575	18.5049	88.9347	89.5523
DN	POU2F2_known13	cluster_3	DN	12977	9719.527	9443	9977	161.193	1.3351	0.417	0.001	0.0010909	3197	67338	75.85	702	13503	15.21	666	12977	14.617	20.832	19.2714	94.8718	96.1046
DN	MAFF_1	cluster_3	DN	39909	29894.867	29483	30321	246.2069	1.335	0.4168	0.001	0.001	12562	176219	70.456	3131	42825	17.122	2894	39909	15.956	23.0377	22.6474	92.4305	93.1909
DP	KLF12_1	cluster_2	DP	17074	12789.995	12592	12985	127.0671	1.3349	0.4168	0.001	0.001	19413	116478	59.073	3661	21678	10.994	2869	17074	8.6593	14.7788	14.6586	78.3666	78.7619
SP4	FOXO6_1	cluster_6	Common_2	12180	9124.19	8899	9363	135.0298	1.3349	0.4167	0.001	0.0010909	2688	35069	61.166	1244	15377	26.82	969	12180	21.244	36.0491	34.7315	77.8939	79.2092
DP	HES1_1	cluster_6	Common_2	198128	148436.357	147594	149189	489.9317	1.3348	0.4166	0.001	0.001	24843	352037	63.079	16910	236209	42.325	14070	198128	35.501	56.6357	56.2804	83.2052	83.8783
DP	FLI1_2	cluster_6	Common_2	48338	36221.091	35841	36614	254.4577	1.3345	0.4163	0.001	0.001	6679	86994	61.881	4558	58681	41.741	3726	48338	34.384	55.7868	55.5648	81.7464	82.3742
SP4	ATF2_2	cluster_6	Common_2	33160	24848.838	24587	25121	164.7197	1.3345	0.4163	0.001	0.0010435	5130	56548	65.885	3437	37664	43.883	3014	33160	38.635	58.7524	58.6404	87.6928	88.0416
SP8	POU2F3_1	cluster_6	Common_2	20357	15253.787	14931	15598	203.5796	1.3345	0.4163	0.001	0.0010435	3586	54118	58.145	1853	26777	28.77	1379	20357	21.872	38.4551	37.616	74.4199	76.0242
DN	SP8_1	cluster_3	DN	38813	29086.677	28450	29735	400.5555	1.3344	0.4162	0.001	0.001	21179	262744	21.027	12378	144666	11.577	3317	38813	3.1062	15.6617	14.7722	26.7975	26.8294
SP4	TBX20_5	cluster_6	Common_2	77211	57862.654	57224	58492	384.1742	1.3344	0.4162	0.001	0.0010909	13251	203127	60.183	6610	97846	28.99	5136	77211	22.876	38.7593	38.0112	77.7005	78.9107
SP4	SOX1_2	cluster_6	Common_2	17235	12916.733	12633	13233	184.7683	1.3343	0.4161	0.001	0.0010909	3418	47871	59.739	1650	22104	27.584	1263	17235	21.508	36.9514	36.003	76.5455	77.9723
SP8	ETV6_2	cluster_6	Common_2	39866	29878.566	29406	30306	288.3562	1.3343	0.416	0.001	0.0011429	9151	82658	22.329	14847	133594	36.088	4418	39866	10.769	48.2789	48.2301	29.7569	29.8412
DN	SPDEF_4	cluster_3	DN	25763	19310.726	18954	19683	215.598	1.3341	0.4159	0.001	0.0010435	8815	132735	73.627	1919	27215	15.096	1807	25763	14.29	20.4991	19.4093	94.1636	94.6647
DP	FOXO3_1	cluster_6	Common_2	24575	18421.368	18065	18783	214.42	1.334	0.4158	0.001	0.0010435	4685	61564	56.333	2720	33781	30.911	1941	24575	22.487	41.4301	39.9178	71.3603	72.748
DP	GMEB2_2	cluster_6	Common_2	19449	14578.756	14321	14861	163.439	1.334	0.4158	0.001	0.001	2591	34443	62.139	1808	23595	42.568	1478	19449	35.088	57.0436	56.4672	81.7478	82.4285
DN	SOX8_2	cluster_3	DN	19904	14920.936	14643	15172	165.8424	1.3339	0.4157	0.001	0.0010909	6301	94659	74.032	1449	20773	16.246	1379	19904	15.567	21.8854	21.0271	95.1691	95.8167
DN	POU2F2_known5	cluster_3	DN	12864	9645.162	9457	9853	120.4259	1.3337	0.4154	0.001	0.0010909	4660	60686	74.223	1066	13379	16.363	1019	12864	15.733	21.867	21.1976	95.591	96.1507
DP	FOXP3_2	cluster_6	Common_2	9803	7350.085	7207	7503	89.3631	1.3337	0.4154	0.001	0.0010909	4108	24648	54.212	2303	13622	29.961	1647	9803	21.561	40.0925	39.772	71.5154	71.9645
SP8	REL_2	cluster_6	Common_2	54169	40615.851	40311	40979	198.0112	1.3337	0.4154	0.001	0.0010435	12473	112795	62.62	7335	65260	36.23	6041	54169	30.073	48.4326	48.0243	82.3586	83.0049
SP8	ATF6_1	cluster_1	SP8	2764	2073.233	1977	2157	52.6611	1.333	0.4147	0.001	0.0010435	3930	27520	63.816	470	3261	7.5619	396	2764	6.4094	10.0763	10.0436	84.2553	84.7593
SP8	ETV4_2	cluster_1	SP8	3091	2318.818	2159	2494	102.0689	1.3329	0.4145	0.001	0.0011429	4775	43196	25.078	1051	9352	5.4294	344	3091	1.7945	7.2042	7.1558	32.7307	33.0518
SP8	ETV3_1	cluster_1	SP8	10058	7546.58	7361	7731	113.77	1.3327	0.4144	0.001	0.0010435	13610	123083	65.171	1310	11627	6.1563	1122	10058	5.3256	8.2439	8.1717	85.6489	86.5055
SP8	E2F_known24	cluster_1	SP8	5937	4454.869	4251	4644	116.8324	1.3326	0.4143	0.001	0.001	2992	51042	66.197	422	6854	8.8891	362	5937	7.6998	12.0989	11.6316	85.782	86.621
SP4	ATF6_1	cluster_6	Common_2	15496	11629.417	11432	11802	112.1513	1.3325	0.4141	0.001	0.0010435	3915	27415	63.572	2634	18311	42.461	2219	15496	35.934	56.6794	56.5238	84.2445	84.6267
DP	FLI1_4	cluster_6	Common_2	52194	39174.55	38738	39590	265.0745	1.3323	0.414	0.001	0.001	7089	92416	63.019	4842	62328	42.502	4024	52194	35.592	56.764	56.4772	83.1062	83.7409
SP8	ATF2_2	cluster_6	Common_2	33024	24786.858	24477	25093	182.6654	1.3323	0.4139	0.001	0.0010435	5120	56438	65.757	3437	37664	43.883	2999	33024	38.477	58.5742	58.5138	87.2563	87.6805
SP4	CUX1_8	cluster_6	Common_2	3227	2422.267	2285	2543	74.3829	1.3321	0.4137	0.001	0.0011429	545	8762	65.969	266	3873	29.16	217	3227	24.296	39.8165	36.8295	81.5789	83.3204
DN	MAFG_1	cluster_3	DN	36632	27501.458	27062	27882	256.4882	1.332	0.4136	0.001	0.0010435	8246	165248	72.352	2020	38737	16.961	1888	36632	16.039	22.8959	22.1679	93.4653	94.5659
SP8	REL_1	cluster_6	Common_2	53551	40208.65	39839	40547	206.2506	1.3318	0.4134	0.001	0.001	12386	112079	62.586	7263	64662	36.108	5967	53551	29.903	48.1754	47.7797	82.1561	82.8168
SP8	HES1_1	cluster_1	SP8	29390	22070.931	21669	22488	256.322	1.3316	0.4132	0.001	0.001	25160	356406	63.862	2546	34903	6.2541	2116	29390	5.2662	8.4102	8.2462	83.1108	84.2048
SP8	TCF7_2	cluster_6	Common_2	40655	30537.352	30099	30938	253.5971	1.3313	0.4128	0.001	0.0010435	11953	131925	55.753	5168	55411	23.417	3740	40655	17.181	31.2892	30.8168	72.3684	73.3699
DN	POU2F2_known11	cluster_3	DN	11683	8776.357	8582	8938	107.916	1.3312	0.4127	0.001	0.0010435	5502	55147	73.881	1256	12148	16.275	1203	11683	15.652	21.8648	21.1852	95.7803	96.1722
DP	ETV6_1	cluster_6	Common_2	203917	153200.302	152273	154099	543.6089	1.331	0.4126	0.001	0.001	26268	378682	62.769	17137	244277	40.491	14158	203917	33.801	53.8983	53.8491	82.6166	83.4778
SP4	SMAD3_3	cluster_6	Common_2	15194	11418.062	11215	11630	123.7704	1.3307	0.4122	0.001	0.0010435	3934	35416	61.507	2115	18720	32.511	1707	15194	26.388	43.391	42.9015	80.7092	81.1645
SP8	FOXP3_2	cluster_6	Common_2	10720	8055.494	7909	8190	85.7389	1.3307	0.4122	0.001	0.0010909	4518	27108	59.623	2303	13622	29.961	1803	10720	23.578	39.907	39.5455	78.2892	78.6962
SP4	E2F7_1	cluster_6	Common_2	29617	22264.407	21962	22567	190.8767	1.3302	0.4117	0.001	0.0010435	4567	59426	65.789	2740	34586	38.289	2320	29617	32.788	50.7992	49.8385	84.6715	85.6329
SP8	IRF4_1	cluster_1	SP8	6261	4707.191	4529	4895	109.9992	1.33	0.4115	0.001	0.0010435	3249	45692	67.371	513	7035	10.373	451	6261	9.2317	13.8812	13.7026	87.9142	88.9979
SP4	ATF7_1	cluster_6	Common_2	22405	16845.783	16572	17115	174.0239	1.33	0.4114	0.001	0.001	3484	45358	62.093	2132	27214	37.254	1729	22405	30.671	49.6269	49.3959	81.0976	82.3289
SP4	MTF1_1	cluster_4	SP4	37568	28248.849	27894	28702	263.0068	1.3299	0.4113	0.001	0.001	38636	515202	65.297	3421	43884	5.5619	2889	37568	4.7614	7.4775	7.2919	84.449	85.6075
SP4	POU2F3_2	cluster_6	Common_2	10504	7900.523	7723	8064	95.8382	1.3295	0.4109	0.001	0.0010909	3575	28645	62.59	1627	12784	27.933	1326	10504	22.952	37.0909	36.6696	81.4997	82.1652
DN	POU2F3_1	cluster_3	DN	14879	11191.705	10944	11425	146.4922	1.3294	0.4108	0.001	0.0010435	4595	69456	74.624	1089	15505	16.659	1036	14879	15.986	22.5462	21.4222	95.1331	95.9626
DN	CBX5_1	cluster_3	DN	11617	8739.552	8573	8903	99.6451	1.3292	0.4106	0.001	0.0010435	5190	57165	75.199	1143	12008	15.796	1100	11617	15.282	21.1946	20.3219	96.238	96.7438
SP8	SOX21_2	cluster_6	Common_2	19088	14360.943	14004	14692	193.2491	1.3291	0.4105	0.001	0.0010909	3688	51655	58.162	1889	25323	28.513	1401	19088	21.493	37.9881	36.9529	74.1662	75.3781
DN	ELK4_2	cluster_3	DN	9805	7377.067	7108	7617	155.9005	1.3291	0.4104	0.001	0.0011429	7819	70678	26.176	3169	28339	10.495	1094	9805	3.6313	13.9916	13.8728	34.5219	34.599
DN	TBX19_1	cluster_3	DN	19468	14648.841	14311	14966	194.7231	1.329	0.4103	0.001	0.0010435	4779	91001	71.181	1156	21010	16.434	1058	19468	15.228	22.1385	21.3932	91.5225	92.6606
SP8	MYBL2_2	cluster_6	Common_2	17333	13042.329	12801	13286	155.6184	1.329	0.4103	0.001	0.0010435	2285	34653	64.344	1404	20538	38.135	1163	17333	32.184	50.8972	50.0188	82.8348	84.3948
SP8	SP1_known3	cluster_6	Common_2	289589	217938.247	216901	218952	601.3028	1.3288	0.4101	0.001	0.001	39576	519194	65.135	26174	337202	42.304	22256	289589	36.33	56.2361	55.7766	85.0309	85.88
SP8	CUX1_8	cluster_6	Common_2	3211	2416.775	2298	2528	69.2757	1.3285	0.4098	0.001	0.0011429	544	8813	66.353	266	3873	29.16	217	3211	24.176	39.8897	36.4348	81.5789	82.9073
SP4	ATF7_1	cluster_4	SP4	6736	5070.382	4898	5251	106.9174	1.3284	0.4097	0.001	0.001	3484	45358	62.093	650	8215	11.246	526	6736	9.2212	15.0976	14.8507	80.9231	81.9963
SP8	MYBL2_4	cluster_6	Common_2	11573	8712.439	8542	8919	117.0857	1.3283	0.4096	0.001	0.0010435	1616	22677	64.03	990	13637	38.505	832	11573	32.677	51.4851	51.0341	84.0404	84.8647
DN	SOX9_4	cluster_3	DN	20331	15307.529	15029	15590	176.9496	1.3281	0.4094	0.001	0.0010909	6382	95858	74.138	1492	21287	16.464	1415	20331	15.724	22.1717	21.2095	94.8391	95.509
DN	SOX11_1	cluster_3	DN	41271	31075.528	30626	31492	256.2202	1.3281	0.4093	0.001	0.0010435	13495	218540	74.839	2854	43312	14.832	2708	41271	14.133	20.0667	18.8849	94.8844	95.2877
SP8	IKZF2_3	cluster_6	Common_2	22421	16882.663	16589	17124	163.2455	1.328	0.4093	0.001	0.0010909	5533	55534	62.869	2881	27614	31.261	2304	22421	25.382	41.6411	40.3735	79.9722	81.1943
SP8	SOX14_2	cluster_6	Common_2	13738	10348.118	10116	10596	147.1598	1.3276	0.4088	0.001	0.0010909	3330	36787	57.469	1716	18299	28.587	1268	13738	21.462	38.0781	37.3447	73.8928	75.0751
SP8	FOXO4_3	cluster_6	Common_2	9854	7425.779	7239	7617	120.5443	1.327	0.4081	0.001	0.0010909	2096	27285	58.087	1056	13081	27.848	784	9854	20.978	37.4046	36.1151	74.2424	75.3306
SP4	POU2F2_known5	cluster_6	Common_2	18065	13615.117	13377	13838	156.4508	1.3268	0.408	0.001	0.0010909	3860	50256	61.466	1820	22745	27.819	1425	18065	22.095	36.9171	35.946	78.2967	79.424
DN	SOX4_1	cluster_3	DN	44232	33338.282	32897	33775	276.7498	1.3268	0.4079	0.001	0.0010435	14546	235575	74.812	3071	46460	14.754	2910	44232	14.047	20.0055	18.7762	94.7574	95.2045
DP	RREB1_1	cluster_6	Common_2	318476	240143.523	238904	241321	760.2178	1.3262	0.4073	0.001	0.001	45950	762192	60.431	26028	411023	32.588	20098	318476	25.25	43.7388	41.7842	77.2168	77.4837
DN	RUNX1_4	cluster_3	DN	8685	6548.948	6400	6710	91.3801	1.3261	0.4072	0.001	0.0010909	3683	37261	73.637	931	9119	18.021	881	8685	17.164	23.9207	23.3086	94.6294	95.2407
DN	SOX30_1	cluster_3	DN	13367	10079.379	9843	10308	144.456	1.3261	0.4072	0.001	0.0010435	4394	65950	74.266	987	14037	15.807	935	13367	15.053	21.279	20.2684	94.7315	95.2269
SP4	NFATC2_1	cluster_6	Common_2	15215	11473.668	11319	11646	98.0704	1.3261	0.4071	0.001	0.0010909	5908	35448	63.511	3099	18285	32.761	2568	15215	27.26	43.4665	42.922	82.8654	83.2103
SP8	SPZ1_1	cluster_6	Common_2	422032	318323.515	317200	319538	703.9839	1.3258	0.4069	0.001	0.001	49464	724618	65.702	33952	488441	44.288	29066	422032	38.266	58.7619	58.242	85.6091	86.4039
SP8	SOX18_2	cluster_6	Common_2	13662	10306.591	10054	10541	149.4445	1.3255	0.4066	0.001	0.0010909	2756	38602	58.784	1357	18124	27.599	1010	13662	20.805	36.6473	35.3919	74.4289	75.3807
DP	E2F2_3	cluster_6	Common_2	17670	13330.741	13063	13606	170.9997	1.3255	0.4065	0.001	0.001	1923	32730	61.531	1330	21927	41.222	1051	17670	33.219	54.6542	53.9872	79.0226	80.5856
DN	ATF4_3	cluster_3	DN	13472	10165.785	9954	10400	138.5439	1.3252	0.4062	0.001	0.0010909	4863	63288	72.723	1124	14204	16.322	1062	13472	15.48	21.8384	21.2868	94.484	94.8465
SP4	SOX21_2	cluster_6	Common_2	20088	15159.568	14874	15468	196.403	1.3251	0.4061	0.001	0.0010909	3895	54547	61.419	1889	25323	28.513	1470	20088	22.619	37.7407	36.827	77.819	79.3271
SP8	TCF7_1	cluster_6	Common_2	49723	37530.219	36963	38085	340.4226	1.3249	0.4059	0.001	0.0010435	10694	171757	56.165	4400	67875	22.195	3169	49723	16.26	29.6334	28.9496	72.0227	73.2567
SP4	SPZ1_1	cluster_6	Common_2	409907	309414.532	308295	310664	738.1747	1.3248	0.4058	0.001	0.001	48085	704127	63.844	33952	488441	44.288	28238	409907	37.167	58.7252	58.2149	83.1704	83.9215
SP8	NFKB_known10	cluster_6	Common_2	62321	47044.046	46566	47546	296.4089	1.3247	0.4057	0.001	0.001	10708	129395	64.216	6222	73667	36.559	5210	62321	30.929	48.6552	48.1634	83.7351	84.5983
DN	ATF7_1	cluster_3	DN	9258	6989.331	6812	7173	111.7337	1.3245	0.4055	0.001	0.001	3904	50828	69.581	801	10168	13.919	724	9258	12.674	18.5451	18.2144	90.387	91.0504
SP8	FOXO6_1	cluster_6	Common_2	11433	8631.494	8375	8886	150.916	1.3245	0.4055	0.001	0.0010909	2530	32988	57.537	1244	15377	26.82	908	11433	19.941	35.8893	34.6581	72.9904	74.3513
SP4	RREB1_1	cluster_6	Common_2	336827	254341.052	253068	255749	797.9166	1.3243	0.4052	0.001	0.001	47895	812464	64.416	26028	411023	32.588	21036	336827	26.705	43.9211	41.4575	80.8207	81.9485
SP4	NFKB_known10	cluster_6	Common_2	60688	45832.569	45391	46236	260.0366	1.3241	0.405	0.001	0.001	10445	126213	62.637	6222	73667	36.559	5070	60688	30.118	48.54	48.0838	81.4851	82.3815
SP8	GMEB2_2	cluster_6	Common_2	20238	15285.408	15052	15538	152.1319	1.324	0.4049	0.001	0.001	2724	36104	65.136	1808	23595	42.568	1544	20238	36.512	56.6814	56.0547	85.3982	85.7724
DP	KLF13_1	cluster_6	Common_2	203525	153734.862	152756	154670	559.5876	1.3239	0.4048	0.001	0.001	19895	342302	61.998	14628	248271	44.967	11879	203525	36.862	59.7085	59.4577	81.2073	81.977
SP4	SP1_known3	cluster_6	Common_2	285281	215478.287	214426	216396	606.738	1.3239	0.4048	0.001	0.001	39133	513297	64.396	26174	337202	42.304	21935	285281	35.79	56.0524	55.5782	83.8045	84.6024
SP8	RREB1_2	cluster_6	Common_2	545192	411794.184	410103	413652	1121.8342	1.3239	0.4048	0.001	0.001	51915	1257959	63.21	30076	678272	34.082	23730	545192	27.395	45.7093	43.3394	78.9001	80.3796
SP4	IRF_known21	cluster_4	SP4	23368	17655.529	17321	17941	190.0029	1.3235	0.4044	0.001	0.001	8196	119181	67.65	1864	26418	14.995	1639	23368	13.264	19.9976	19.6072	87.9292	88.4548
DN	SOX4_2	cluster_3	DN	17951	13566.961	13295	13826	158.3913	1.3231	0.4039	0.001	0.0010909	5733	86204	75.057	1328	18761	16.335	1258	17951	15.63	21.9431	20.8239	94.7289	95.6825
DN	SPIB_1	cluster_3	DN	29685	22435.326	22212	22667	133.9911	1.3231	0.4039	0.001	0.0010435	22113	132678	71.137	5324	31674	16.982	4984	29685	15.916	22.5388	22.3737	93.6138	93.7204
SP8	HIF1A::ARNT_1	cluster_6	Common_2	45033	34038.233	33767	34285	161.9533	1.323	0.4038	0.001	0.001	10355	74439	66.239	7202	51412	45.748	6288	45033	40.072	60.7243	60.4965	87.3091	87.5924

SP8	PBX1_4	cluster_6	Common_2	12117	9159.166	8897	9382	145.1015	1.3229	0.4037	0.001	0.0010909	2771	31106	57.753	1474	16039	29.779	1094	12117	22.497	39.4803	38.9539	74.2198	75.5471
SP8	ELK4_2	cluster_6	Common_2	33803	25553.77	25153	26012	258.5243	1.3228	0.4036	0.001	0.0011429	7080	64048	23.72	11869	107416	39.782	3733	33803	12.519	52.726	52.7776	31.4517	31.4692
SP8	SOX21_3	cluster_6	Common_2	19118	14452.246	14144	14743	180.4497	1.3228	0.4036	0.001	0.0010909	3510	52680	59.82	1735	24817	28.181	1310	19118	21.709	37.3219	36.2908	75.5043	77.0359
DN	SPIC_1	cluster_3	DN	54106	40906.011	40444	41340	286.0607	1.3227	0.4035	0.001	0.001	19310	251988	71.563	4562	57978	16.465	4224	54106	15.366	21.8747	21.4717	92.591	93.3216
SP8	SOX9_4	cluster_6	Common_2	26932	20360.787	19951	20762	246.8063	1.3227	0.4035	0.001	0.0010909	5012	75280	58.223	2495	35804	27.691	1839	26932	20.83	36.6919	35.7758	73.7074	75.2206
SP8	TCF7L1_1	cluster_6	Common_2	45063	34073.43	33581	34599	320.6744	1.3225	0.4033	0.001	0.0010435	9778	157054	56.06	4005	61781	22.053	2871	45063	16.085	29.3618	28.6927	71.6854	72.9399
DN	POU2F2_known12	cluster_3	DN	11494	8692.459	8513	8894	106.553	1.3223	0.403	0.001	0.0010435	5446	54541	74.724	1240	11923	16.335	1191	11494	15.747	21.8693	21.0741	96.0484	96.4019
SP4	SOX2_5	cluster_6	Common_2	19198	14518.485	14137	14868	217.1932	1.3223	0.403	0.001	0.0010909	3385	54207	61.44	1609	24458	27.721	1240	19198	21.76	36.6322	35.4161	77.0665	78.4937
DP	FOXP1_1	cluster_6	Common_2	70573	53383.319	52487	54172	506.3206	1.322	0.4027	0.001	0.0010435	8153	191364	49.307	6055	120673	31.093	3335	70573	18.184	40.9052	36.8789	55.0784	58.4828
DP	MYCN_1	cluster_6	Common_2	63379	47954.825	47503	48428	263.6264	1.3216	0.4023	0.001	0.001	10303	113712	63.225	6966	76024	42.27	5777	63379	35.239	56.071	55.7364	82.9314	83.3671
SP4	POU2F2_known15	cluster_6	Common_2	13223	10004.833	9752	10238	140.9419	1.3216	0.4023	0.001	0.0010435	3260	36005	62.683	1524	16326	28.423	1228	13223	23.021	37.6687	36.7255	80.5774	80.9935
SP8	HBP1_1	cluster_6	Common_2	24666	18664.414	18271	19035	229.2286	1.3215	0.4022	0.001	0.0010435	4130	63662	59.134	2217	32501	30.189	1640	24666	22.912	39.7094	38.7452	73.9738	75.893
DN	FOXP3_2	cluster_3	DN	6087	4606.449	4511	4702	59.713	1.3213	0.402	0.001	0.0010909	5499	32994	72.569	1087	6401	14.079	1031	6087	13.388	18.7489	18.4488	94.8482	95.0945
DP	ETV6_2	cluster_2	DP	13839	10474.935	10147	10802	204.4806	1.3211	0.4018	0.001	0.0011429	9680	87477	23.63	4960	44381	11.989	1540	13839	3.7384	15.9091	15.8202	31.0484	31.1823
DN	FOXO4_2	cluster_3	DN	17183	13006.982	12744	13228	152.1261	1.321	0.4017	0.001	0.0010435	7348	96298	75.255	1450	17939	14.019	1381	17183	13.428	18.7942	17.8436	95.2414	95.7857
SP4	CUX1_9	cluster_4	SP4	2403	1819.007	1705	1937	71.8195	1.3209	0.4015	0.001	0.0012632	597	10309	69.627	174	2765	18.675	150	2403	16.23	25.1256	23.3097	86.2069	86.9078
SP4	IRF4_1	cluster_4	SP4	7187	5440.663	5253	5648	114.8544	1.3209	0.4015	0.001	0.0010435	3307	46509	68.576	591	8045	11.862	521	7187	10.597	15.7545	15.4529	88.1557	89.335
SP8	SOX9_6	cluster_6	Common_2	22399	16961.519	16538	17369	252.2872	1.3206	0.4011	0.001	0.0010909	4230	63611	57.612	2124	30252	27.399	1538	22399	20.287	36.3593	35.2125	72.4105	74.0414
SP8	STAT_known2	cluster_1	SP8	12855	9735.913	9436	10051	191.2425	1.3203	0.4009	0.001	0.001	8911	178852	67.814	782	14719	5.5809	672	12855	4.8742	7.5412	7.1875	85.9335	87.3361
DN	SOX10_4	cluster_3	DN	21799	16511.966	16225	16847	190.079	1.3202	0.4007	0.001	0.0010909	6860	103161	73.858	1603	23013	16.476	1511	21799	15.607	22.0262	21.131	94.2608	94.7247
SP4	ERG_2	cluster_6	Common_2	49707	37652.956	37307	38027	224.2382	1.3201	0.4007	0.001	0.001	7181	93517	64.636	4547	58454	40.401	3843	49707	34.356	53.5162	53.1529	84.5173	85.0361
SP8	SOX3_1	cluster_6	Common_2	11662	8833.957	8579	9095	157.5298	1.3201	0.4006	0.001	0.0010909	2117	33889	61.352	1001	15050	27.246	762	11662	21.113	35.9943	34.4123	76.1239	77.4884
SP4	NFATC1_1	cluster_6	Common_2	42830	32450.754	31954	32987	316.3002	1.3198	0.4004	0.001	0.0010909	5359	103439	64.397	2869	52065	32.414	2301	42830	26.664	42.9371	41.406	80.2022	82.2626
SP4	STAT_known3	cluster_4	SP4	14424	10928.263	10630	11235	185.2873	1.3199	0.4004	0.001	0.0010435	7269	145730	67.556	869	16490	7.6443	749	14424	6.6866	10.304	9.8978	86.191	87.4712
DP	STAT_known3	cluster_6	Common_2	77338	58598.839	58037	59305	358.811	1.3198	0.4003	0.001	0.0010435	6852	137400	63.695	4799	93340	43.27	3911	77338	35.852	57.0782	56.2868	81.4961	82.8562
SP4	SP8_1	cluster_6	Common_2	457055	346409.594	345331	347590	684.7041	1.3194	0.3999	0.001	0.001	64724	792047	63.386	45886	551019	44.097	37900	457055	36.577	58.5563	57.7055	82.596	82.9472
SP8	E2F_known26	cluster_6	Common_2	14252	10805.164	10569	11066	155.3278	1.319	0.3994	0.001	0.0010435	1668	28373	65.605	1040	16856	38.975	863	14252	32.954	51.7386	50.2309	82.9808	84.5515
DP	SP100_1	cluster_2	DP	1100	833.808	759	906	44.5813	1.3189	0.3993	0.001	0.0013333	864	11233	66.811	101	1262	7.5061	86	1100	6.5426	9.9537	9.7926	85.1485	87.1632
SP8	ARID3A_2	cluster_6	Common_2	7631	5785.732	5570	5955	116.8648	1.3189	0.3993	0.001	0.0013333	1242	20101	63.924	641	9493	30.189	497	7631	24.268	40.0161	37.9633	77.5351	80.3855
SP4	FOXP3_2	cluster_6	Common_2	11111	8424.783	8276	8565	88.3833	1.3188	0.3992	0.001	0.0010909	4729	28374	62.407	2303	13622	29.961	1872	11111	24.438	39.5855	39.1591	81.2853	81.5666
DN	PKNOX2_3	cluster_3	DN	40837	30967.462	30635	31354	224.0355	1.3187	0.3991	0.001	0.0010435	13787	153514	71.649	3958	43496	20.301	3701	40837	19.06	26.8441	26.6015	93.5068	93.8868
DP	KLF12_2	cluster_6	Common_2	323937	245646.561	244672	246552	584.2894	1.3187	0.3991	0.001	0.001	38298	549311	63.594	27197	386464	44.741	22636	323937	37.502	59.1049	58.9715	83.2298	83.8207
DN	TBX1_2	cluster_3	DN	25450	19304.934	18956	19678	216.8638	1.3183	0.3987	0.001	0.0010909	6383	115007	73.738	1562	26827	17.2	1469	25450	16.317	23.0143	22.1291	94.0461	94.8671
DP	MTF1_3	cluster_6	Common_2	80117	60775.606	60215	61370	348.6862	1.3182	0.3986	0.001	0.001	12915	169509	62.611	7551	97546	36.03	6146	80117	29.593	47.5881	47.2642	81.3932	82.1325
SP4	SOX3_3	cluster_6	Common_2	15663	11881.836	11563	12168	190.6655	1.3182	0.3986	0.001	0.0010909	2900	46500	59.883	1342	20452	26.338	1007	15663	20.171	34.7241	33.6839	75.0373	76.5842
SP8	STAT_known3	cluster_6	Common_2	79567	60364.98	59742	60975	359.8691	1.3181	0.3985	0.001	0.0010435	7066	141634	65.658	4799	93340	43.27	4023	79567	36.885	56.9346	56.1779	83.83	85.2443
SP8	KLF13_1	cluster_6	Common_2	213353	161895.567	161038	162792	537.3097	1.3178	0.3982	0.001	0.001	20972	360883	65.363	14628	248271	44.967	12471	213353	38.642	59.465	59.1197	85.2543	85.9355
SP8	SOX8_2	cluster_6	Common_2	26780	20321.001	19960	20687	230.8376	1.3178	0.3982	0.001	0.0010909	4985	74903	58.581	2477	35522	27.781	1828	26780	20.944	36.67	35.7529	73.799	75.3899
SP4	ERG_4	cluster_6	Common_2	49897	37865.627	37496	38277	245.3149	1.3177	0.3981	0.001	0.001	7152	93169	64.953	4544	58432	40.736	3855	49897	34.786	53.901	53.5554	84.8371	85.3933
SP4	XBP1_2	cluster_6	Common_2	30694	23299.094	22865	23708	256.1353	1.3174	0.3977	0.001	0.0010435	2736	63041	65.364	1647	36240	37.575	1360	30694	31.825	49.7076	48.6889	82.5744	84.6965
SP4	FLI1_2	cluster_6	Common_2	50094	38026.492	37615	38418	248.9309	1.3173	0.3976	0.001	0.001	7024	91445	65.047	4558	58681	41.741	3871	50094	35.633	55.111	54.7805	84.9276	85.3666
SP8	CBX5_1	cluster_6	Common_2	17642	13393.007	13129	13685	155.2898	1.3172	0.3975	0.001	0.0010435	4252	46829	61.603	2111	22302	29.338	1641	17642	23.208	38.5936	37.6732	77.7357	79.105
SP8	SOX10_4	cluster_6	Common_2	29401	22319.754	21932	22709	238.5867	1.3172	0.3975	0.001	0.0010909	5401	81262	58.18	2719	39147	28.027	1997	29401	21.05	36.9746	36.1805	73.4461	75.1041
SP8	IKZF2_2	cluster_6	Common_2	15732	11943.828	11702	12190	139.2684	1.3171	0.3974	0.001	0.0010435	3698	36980	61.725	2039	19720	32.915	1601	15732	26.259	43.2937	42.5419	78.5189	79.7769
SP4	REL_2	cluster_6	Common_2	54241	41182.458	40815	41588	236.0612	1.3171	0.3973	0.001	0.0010435	7335	114508	63.571	4390	65260	36.23	6048	54241	30.113	47.7687	47.3687	82.454	83.1152
DN	POU2F2_known10	cluster_3	DN	11195	8500.257	8327	8677	102.1651	1.317	0.3972	0.001	0.0010435	6013	54229	74.503	1333	11620	15.964	1279	11195	15.38	21.2706	20.6439	95.949	96.3425
DP	CTCF_known1	cluster_2	DP	134009	101759.151	100748	102828	614.4306	1.3169	0.3972	0.001	0.0010435	55720	1030489	59.443	9691	172310	9.9396	7383	134009	7.7302	13.2502	13.0044	76.1841	77.772
SP4	SOX14_2	cluster_6	Common_2	14266	10833.59	10615	11070	141.7438	1.3168	0.397	0.001	0.0010909	3491	38572	60.257	1716	18299	28.587	1316	14266	22.286	37.6969	36.9854	76.69	77.9605
SP8	SOX5_1	cluster_6	Common_2	7690	5840.984	5691	5973	87.4978	1.3165	0.3967	0.001	0.0010909	2249	20248	60.174	1115	9815	29.169	867	7690	22.854	38.5505	37.9791	77.7578	78.3495
DN	TBX1_4	cluster_3	DN	44587	33873.148	33379	34364	304.5347	1.3163	0.3965	0.001	0.0010909	11107	211898	72.371	2605	47743	16.306	2410	44587	15.228	21.698	21.0417	92.5144	93.3896
SP4	POU2F2_known10	cluster_6	Common_2	17074	12971.126	12771	13209	133.0836	1.3163	0.3965	0.001	0.0010435	5130	46258	63.552	2357	20734	28.485	1923	17074	23.457	37.4854	36.9104	81.5868	82.3478
SP4	REL_1	cluster_6	Common_2	53617	40732.105	40372	41072	217.7249	1.3163	0.3965	0.001	0.001	12552	113599	63.434	7263	64662	36.108	5975	53617	29.94	47.602	47.1985	82.2663	82.9189
DP	SP8_1	cluster_6	Common_2	448486	340729.092	339483	341900	733.0494	1.3163	0.3964	0.001	0.001	63765	778558	62.307	45886	551019	44.097	37223	448486	35.892	58.3753	57.6047	81.1206	81.3921
SP4	ATF3_known4	cluster_6	Common_2	72794	55308.056	54825	55773	284.1186	1.3161	0.3963	0.001	0.001	8312	117305	66.718	5968	83194	47.317	5173	72794	41.402	62.2353	62.0553	86.679	87.4991
DN	ATF3_known5	cluster_3	DN	39050	29674.528	29262	30048	229.0525	1.3159	0.3961	0.001	0.001	14705	206515	71.894	3073	41829	14.562	2844	39050	13.595	19.3404	18.909	92.548	93.3563
SP4	KLF12_2	cluster_6	Common_2	330899	251451.909	250242	252558	668.8761	1.316	0.3961	0.001	0.001	39236	562601	65.133	27197	386464	44.741	23137	330899	38.308	58.9688	58.8159	85.0719	85.6222
SP4	ETV6_1	cluster_6	Common_2	209429	159162.184	158167	160078	545.6168	1.3158	0.396	0.001	0.001	27340	393690	65.257	17137	244277	40.491	14559	209429	34.714	53.2516	53.1964	84.9565	85.7342
DN	POU2F2_known1	cluster_3	DN	15066	11450.888	11184	11717	165.054	1.3157	0.3958	0.001	0.0010435	4025	72871	75.443	935	15805	16.363	884	15066	15.598	21.9627	20.6749	94.5455	95.3243
SP8	IRF6_1	cluster_6	Common_2	21423	16282.97	16016	16584	169.7529	1.3156	0.3958	0.001	0.0010909	2749	44071	67.262	1569	24456	37.325	1357	21423	32.696	49.3634	48.6102	86.4882	87.5981
SP8	GATA_known17	cluster_6	Common_2	21323	16207.424	15894	16557	211.0114	1.3156	0.3957	0.001	0.0010909	4062	65192	61.257	1780	27104	25.468	1371	21323	20.036	33.7518	32.708	77.0225	78.671
SP4	E2F_known26	cluster_6	Common_2	14332	10894.415	10658	11151	154.1561	1.3155	0.3956	0.001	0.0010435	1689	28742	66.459	1040	16856	38.975	866	14332	33.139	51.2729	49.8643	83.2692	85.0261
DN	SMAD3_1	cluster_3	DN	46098	35045.381	34744	35334	183.7545	1.3154	0.3955	0.001	0.0010435	26078	216611	73.645	5906	48192	16.385	5651	46098	15.673	21.6696	21.2815	95.6824	95.6549
SP4	FOXO4_3	cluster_6	Common_2	10394	7902.202	7688	8113	127.5975	1.3153	0.3954	0.001	0.0010909	2238	29139	62.034	1056	13081	27.848	826	10394	22.128	36.908	35.6704	78.2197	79.4588
SP4	POU2F2_known1	cluster_6	Common_2	22008	16731.94	16349	17122	231.4305	1.3153	0.3954	0.001	0.0010435	3339	60392	62.523	1610	27604	28.578	1252	22008	22.785	37.4963	36.4419	77.764	79.7276
SP8	MYCN_1	cluster_1	SP8	8986	6833.752	6627	7046	121.2992	1.3149	0.395	0.001	0.001	10432	115067	63.978	997	10742	5.9726	822	8986	4.9963	7.8796	7.8094	82.4473	83.653
SP8	SOX17_5	cluster_6	Common_2	10463	7958.688	7712	8189	146.2054	1.3146	0.3947	0.001	0.0011429	2277	31990	58.026	1054	14122	25.616	768	10463	18.979	33.7286	32.7071	72.8653	74.0901
SP4	GMEB2_2	cluster_6	Common_2	20401	15520.529	15256	15801	158.7111	1.3144	0.3944	0.001	0.001	2773	36777	66.35	1808	23595	42.568	1552	20401	36.806	55.9683	55.4722	85.8407	86.4632
SP4	HIF1A::ARNT_1	cluster_6	Common_2	45181	34380.319	34135	34603	150.1764	1.3141	0.3941	0.001	0.001	10457	75241	66.952	7202	51412	45.748	6309	45181	40.204	60.3328	60.0484	87.6007	87.8803
DN	SMAD_2	cluster_3	DN	63400	48253.254	47862	48636	236.3366	1.3139	0.3938	0.001	0.0010435	28215	291809	73.323	6601	66737	16.769	6252	63400	15.93	22.1584	21.7265	94.7129	94.9998
SP4	NFKB_known11	cluster_6	Common_2	69848	53165.555	52678	53622	295.8865	1.3138	0.3937	0.001	0.001	11939	145036	63.244	7103	84596	36.889	5807	69848	30.458	48.6389	48.1591	81.7542	82.5666
DN	MAF_known11	cluster_3	DN	46672	35542.436	35090	35977	270.2143	1.3131	0.393	0.001	0.0010435	13743	221582	72.337	3193	49735	16.236	2965	46672	15.236	21.5746	21.0631	92.8594	93.8414
SP4	MYCN_1	cluster_4	SP4	10013	7625.436	7406	7834	130.5546	1.3131	0.3929	0.001	0.001	10339	114073	63.425	1121	12081	6.7171	922	10013	5.5673	8.9177	8.7777	82.248	82.8822
DN	TBX21_4	cluster_3	DN	32991	25126.563	24757	25501	229.9111	1.313	0.3928	0.001	0.0010909	9894	148765	73.27	2406	34959	17.218	2253	32991	16.249	22.7714	22.1766	93.6409	94.3705
SP4	E2F2_3	cluster_6	Common_2	18774	14300.589	14023	14593	171.6269	1.3128	0.3926	0.001	0.001	2073	35280	66.325	1330	21927	41.222	1120	18774	35.294	54.028	53.2143	84.2105	85.6205
DP	HES1_1	cluster_2	DP	47722	36353.709	35851	36880	309.5721	1.3127	0.3925	0.001	0.001	24843	352037	63.079	4230	58105	10.411	3415	47722	8.551	13.7463	13.556	80.7329	82.1306
DP	E2F2_2	cluster_6	Common_2	23257	17719.528	17344	18093	216.8028	1.3125	0.3923	0.001	0.0010909	2804	47831	58.459	1874	30764	37.6	1387	23257	28.425	49.465	48.6233	74.0128	75.5981
SP4	SPZ1_1	cluster_4	SP4	51336	39133.486	38591	39712	337.8854	1.3118	0.3916	0.001	0.001	48085	704127	63.844	4489	62429	5.6605	3634	51336	4.6547	7.5575	7.2907	80.9534	82.231
SP4	PBX1_2	cluster_6	Common_2	16049	12234.29	11929	12544	176.7875	1.3118	0.3915	0.001	0.0010435	3037	43176	61.498	1495	20344	28.977	1153	16049	22.86	37.9651	37.1711	77.1237	78.8881
DN	TBX20_5	cluster_3	DN	53068	40458.998	40034	40902	273.0664	1.3116	0.3914	0.001	0.0010909	16570	253871	75.218	3760	55218	16.36	3597	53068	15.723	21.7079	20.9035	95.6649	96.1063
SP4	ERG_4	cluster_4	SP4	11518	8781.581	8562	9027	140.3203	1.3116	0.3913	0.001	0.001	7152	93169	64.953	1082	13662	9.5245	899	11518	8.0298	12.5699	12.3625	83.0869	84.3068
DP	MYCN_1	cluster_2	DP	16964	12936.248	12634	13205	172.9067	1.3113	0.391	0.001	0.001	10303	113712	63.225	1894	20515	11.406	1549	16964	9.4321	15.0345	14.9184	81.7846	82.6907
SP4	RREB1_1	cluster_6	Common_2	337181	257137.012	255819	258456	729.3472	1.3113	0.391	0.001	0.001	48842	818149	64.867	26028	411023	32.588	21192	337181	26.733	43.3889	41.2127	81.42	82.0346
SP8	SOX18_4	cluster_6	Common_2	10413	7941.885	7657	8176	144.5411	1.3111	0.3908	0.001	0.0012	2159	30301	57.531	1070	14264	27.082	769	10413	19.771	35.6183	34.3652	71.8692	73.002
SP4	ETS_known13	cluster_6	Common_2	98263	74954.929	74369	75526	348.3608	1.311	0.3906	0.001	0.001	10234	164235	67.178	7079	111951	45.792	6155	98263	40.193	60.1427	59.8307	86.9473	87.7732
SP4	ETV2_1	cluster_6	Common_2	33286	25389.906	24974	25836	267.5663	1.311	0.3906	0.001	0.0010435	7748	77780	26.389	9620	95999	32.57	3316	33286	11.293	42.7981	42.7951	34.4699	34.6733
SP8	MYBL1_5	cluster_6	Common_2	23295	17771.733	17427	18071	192.0614	1.3108	0.3904	0.001	0.0010435	2849	45954	65.783	1754	27428	39.263	1468	23295	33.347	51.5269	50.692	83.6944	84.9315
DN	TBX20_2	cluster_3	DN	27799	21209.14	20854	21572	220.6698	1.3107	0.3903	0.001	0.0010435	7160	129186	74.304	1683	29129	16.754	1598	27799	15.989	22.3184	21.5186	94.9495	95.4341
SP4	FLI1_4	cluster_6	Common_2	53905	41144.682	40693	41510	243.5866	1.3101	0.3897	0.001	0.001	7443	97009	66.151	4842	62328	42.502	4159	53905	36.758	55.878	55.567	85.8943	86.486
SP4	PATZ1_1	cluster_6	Common_2	537639	410371.844	409075	411805	840.4732	1.3101	0.3897	0.001	0.001	71621	944599	64.129	49543	644744	43.772	41193	537639	36.501	57.5153	56.9172	83.146	83.388
SP4	SOX18_2	cluster_6	Common_2	14339	10944.776	10685	11226	160.1803	1.3101	0.3897	0.001	0.0010909	2930	41032	62.484	1357	18124	27.599	1060	14339	21.836	36.1775	34.9459	78.1135	79.1161
DN	PBX3_known1	cluster_3	DN	21170	16159.303	15905	16395	139.8916	1.3101	0.3896	0.001	0.0010435	8906	98882	73.528	2087	22368	16.633	1964	21170	15.742	22.0525	21.4094	94.1064	94.6441
SP8	FOXO4_2	cluster_6	Common_2	32697	24962.283	24630	25303	210.0811	1.3098	0.3894	0.001	0.0010435	6205	81377	63.594	3236	40377	31.554	2568	32697	25.552	41.386	40.2757	79.3572	80.9793
SP4	ETV6_1	cluster_4	SP4	46840	35761.532	35251	36260	306.1737	1.3098	0.3893	0.001	0.001	27340	393690	65.257	3975	55382	9.18	3320	46840	7.7641	12.1434	11.8977	83.522	84.5762
SP4	POU2F2_known11	cluster_6	Common_2	16857	12871.852	12622	13123	159.8499	1.3096	0.3891	0.001	0.0010435	4718	47310	63.382	2129	20693	27.723	1717	16857	22.583	36.3925	35.6309	80.6482	81.4623
SP4	SOX7_4	cluster_6	Common_2	15591	11905.334	11591	12229	194.5296	1.3096	0.3891	0.001	0.0010909	2839	45561	60.885	1326	20183	26.971	1009	15591	20.835	35.5407	34.2201	76.0935	77.2482
SP4	IRF6_1	cluster_4	SP4	6226	4754.174	4576	4957	115.3669	1.3095	0.389	0.001	0.0010909	2762	44279	67.58	466	7185	10.966	398	6226	9.5023	14.4098	14.0608	85.4077	86.6527
SP8	E2F2_3	cluster_6	Common_2	18720	14296.013	14012	14563	166.7663	1.3094	0.3889	0.001	0.001	2069	35210	66.193	1330	21927	41.222	1118	18720	35.193	54.0358	53.1667	84.0602	85.3742
DP	ETS_known17	cluster_2	DP	31279	23889.876	23438	24354	266.4517	1.3093	0.3888	0.001	0.001	12494	213553	60.343	2413	39722	11.224	1854	31279	8.8384	14.8391	14.6469	76.8338	78.7448
SP8	ETV3_1	cluster_6	Common_2	68598	52397.799	52010	52819	240.0758	1.3092	0.3887	0.001	0.0010435	13610	123083	65.171	8919	80297	42.516	7583	68598	36.322	55.7164	55.7331	85.0207	85.4303
SP4	IKZF2_3	cluster_6	Common_2	23201	17722.416	17429	18023	169.4905	1.3091	0.3886	0.001	0.0010909	5825	58444	66.163	2881	27614	31.261	2390	23201	26.265	41.03	39.6978	82.9573	84.019
SP4	CTCF_known2	cluster_6	Common_2	148250	113270.865	112284	114177	622.326	1.3088	0.3883	0.001	0.0010435	15039	241923	28.953	24385	390108	46.688	9218	148250	17.743	61.294	61.2798	37.8019	38.0023
SP8	FOXO3_4	cluster_6	Common_2	8621	6586.381	6400	6804	121.7666	1.3089	0.3883	0.001	0.0010909	1903	24809	59.374	913	11306	27.058	684	8621	20.632	35.9432	34.7495	74.9179	76.2515
SP8	SOX8_9	cluster_6	Common_2	16064	12272.881	11941	12583	193.0711	1.3089	0.3883	0.001	0.0010909	2766	44281	57.488	1457	22004	28.567	1042	16064	20.855	37.6717	36.2774	71.5168	73.0049
DN	GATA_known17	cluster_3	DN	18509	14144.234	13885	14396	160.9491	1.3086	0.388	0.001	0.0010909	5009	80296	75.449	1265	19323	18.157	1201	18509	17.392	23.9768	23.051	94.9407	95.7874
SP4	IRF6_1	cluster_6	Common_2	21356	16319.527	16043	16599	166.7708	1.3086	0.388	0.001	0.0010909	2762	44279	67.58	1569	24456	37.325	1352	21356	32.594	48.95	48.2305	86.1695	87.3242
SP4	ERG_2	cluster_4	SP4	11677	8923.582	8672	9167	145.5098	1.3085	0.3879	0.001	0.001	7181	93517	64.636	1107	13976	9.6597	910	11677	8.0707	12.6723	12.4865	82.2042	83.5504
DP	ETV6_1	cluster_2	DP	52742	40315.911	39771	40802	326.1778	1.3082	0.3876	0.001	0.001	26268	378682	62.769	4600	64454	10.684	3709	52742	8.7424	14.1198	13.9278	80.6304	81.8289
SP4	HES1_1	cluster_4	SP4	23506	17968.82	17616	18353	221.8449	1.3081	0.3875	0.001	0.001	24677	349612	62.645	2115	28979	5.1926	1691	23506	4.2119	6.8525	6.7235	79.9527	81.1139
SP8	SOX15_2	cluster_6	Common_2	15188	11609.933	11360	11859	158.8598	1.3082	0.3875	0.001	0.0010909	3083	43248	60.363	1502	19922	27.806	1121	15188	21.198	36.3607	35.1184	74.6338	76.2373
SP8	HMGA1_2	cluster_6	Common_2	29643	22663.364	22249	23052	234.5586	1.308	0.3873	0.001	0.0010435	5565	78050	63.498	2777	36941	30.053	2195	29643	24.116	39.4429	37.9795	79.0421	80.2442
DP	STAT_known2	cluster_6	Common_2	103696	79294.764	78676	79994	394.9681	1.3077	0.3871	0.001	0.001	8789	176424	66.894	6134	120009	45.503	5220	103696	39.318	59.3924	58.7766	85.0994	86.4069
SP8	STAT_known2	cluster_6	Common_2	105049	80327.353	79688	80906	361.9482	1.3078	0.3871	0.001	0.001	8911	178852	67.814	6134	120009	45.503	5300	105049	39.831	59.4771	58.7352	86.4037	87.5343
DN	POU2F3_2	cluster_3	DN	7411	5667.46	5541	5791	76.4896	1.3076	0.3869	0.001	0.0010909	4271	34232	74.798	988	7694	16.812	947	7411	16.193	22.1728	21.6493	95.8502	96.3218
SP4	KLF4_1	cluster_6	Common_2	444748	340120.914	339079	341096	605.1579	1.3076	0.3869	0.001	0.001	78036	752571	64.791	54917	525754	45.263	46267	444748	38.289	59.2893	59.0971	84.249	84.5924
SP8	ARID5A_1	cluster_6	Common_2	4191	3204.825	3078	3335	81.3144	1.3076	0.3869	0.001	0.0012632	834	11458	67.591	410	5064	29.873	330	4191	24.723	39.5683	36.5771	80.4878	82.7607
SP8	ETV1_1	cluster_1	SP8	3088	2361.336	2189	2521	98.575	1.3076	0.3869	0.001	0.0010909	4842	43840	25.062	1065	9493	5.4269	343	3088	1.7653	7.0838	7.0438	32.2066	32.5292
SP4	RUNX1_2	cluster_6	Common_2	24196	18511.45	18116	18863	223.6358	1.3071	0.3863	0.001	0.0010435	4574	71425	63.314	2063	30260	26.824	1616	24196	21.448	35.3301	33.8761	78.3325	79.9603
DP	MYBL2_4	cluster_6	Common_2	11042	8448.65	8237	8642	122.822	1.3069	0.3862	0.001	0.0010435	1563	21943	61.958	990	13637	38.505	791	11042	31.178	50.6078	50.3213	79.899	80.9709
DP	MYBL1_5	cluster_6	Common_2	22409	17147.034	16837	17467	185.6684	1.3069	0.3861	0.001	0.0010435	2741	44252	63.347	1754	27428	39.263	1409	22409	32.078	51.4046	50.6395	80.3307	81.7012
SP8	SOX30_1	cluster_6	Common_2	19970	15280.787	14952	15603	195.7878	1.3068	0.3861	0.001	0.0010435	3553	53360	60.089	1811	26029	29.311	1369	19970	22.488	38.5308	37.425	75.5936	76.7221
DN	TBX21_6	cluster_3	DN	40873	31276.874	30818	31734	273.4207	1.3068	0.386	0.001	0.0010909	10503	189588	74.498	2481	42917	16.864	2342	40873	16.061	22.2984	21.5589	94.3974	95.2373
DN	PKNOX1_2	cluster_3	DN	41312	31617.186	31249	31996	235.0891	1.3066	0.3858	0.001	0.0010435	13772	152898	72.377	4017	43989	20.823	3757	41312	19.556	27.28	27.0193	93.5275	93.9144
SP4	SP100_1	cluster_4	SP4	1346	1029.912	965	1094	38.2985	1.3066	0.3858	0.001	0.0013333	962	12518	74.454	115	1406	8.3626	107	1346	8.0057	11.1227	10.7525	93.0435	95.7326
SP8	HIF1A_1	cluster_6	Common_2	81940	62716.756	62284	63130	249.8877	1.3065	0.3857	0.001	0.0010435	11568	130194	67.952	8327	92629	48.346	7331	81940	42.767	63.3731	62.9368	88.0389	88.4604
DN	BATF_known1	cluster_3	DN	12753	9763.211	9543	9988	134.1496	1.3062	0.3854	0.001	0.0010435	4780	62340	72.002	1070	13651	15.767	994	12753	14.73	20.795	20.4572	92.8972	93.4217

DN	TBX15_1	cluster_3	DN	31536	24146.762	23762	24552	241.499	1.306	0.3852	0.001	0.0010909	7884	142057	75.135	1916	33027	17.468	1817	31536	16.68	23.0467	22.1995	94.833	95.4855
DP	MYBL2_2	cluster_6	Common_2	16044	12286.201	12002	12582	170.1605	1.3058	0.385	0.001	0.0010435	2149	32599	60.53	1404	20538	38.135	1072	16044	29.791	49.8837	49.2162	76.3533	78.1186
SP8	SPZ1_1	cluster_1	SP8	57971	44392.187	43778	45020	371.5847	1.3059	0.385	0.001	0.001	49464	724618	65.702	4890	68733	6.2321	4085	57971	5.2563	8.2585	8.0002	83.5378	84.3423
SP8	PATZ1_1	cluster_6	Common_2	553631	424089.36	422865	425451	810.5316	1.3055	0.3846	0.001	0.001	74077	975342	66.217	49543	644744	43.772	42447	553631	37.586	57.3012	56.7628	85.6771	85.8683
SP4	SOX_1	cluster_6	Common_2	31490	24123.877	23775	24454	210.3382	1.3053	0.3844	0.001	0.0010435	7156	85938	62.408	3385	39308	28.545	2670	31490	22.868	37.3113	36.6427	78.8774	80.1109
SP8	NFKB_known11	cluster_6	Common_2	71740	54958.813	54454	55426	291.9121	1.3053	0.3844	0.001	0.001	12335	149870	65.352	7103	84596	36.889	5964	71740	31.283	48.3502	47.8682	83.9645	84.8031
SP8	SOX12_1	cluster_6	Common_2	12977	9941.797	9718	10193	144.1424	1.3053	0.3843	0.001	0.0010435	2969	38740	61.39	1367	16802	26.625	1032	12977	20.564	34.7592	33.4977	75.4938	77.2349
SP8	SOX7_3	cluster_6	Common_2	18791	14396.104	14022	14727	212.6178	1.3053	0.3843	0.001	0.0010909	3353	53689	59.073	1672	25254	27.786	1216	18791	20.675	36.266	34.9997	72.7273	74.408
DP	ETV6_2	cluster_6	Common_2	41321	31662.407	31192	32201	300.7472	1.305	0.3841	0.001	0.0011429	9680	87477	23.63	14847	133594	36.088	4571	41321	11.162	47.2211	47.2364	30.7874	30.9303
SP4	SOX17_5	cluster_6	Common_2	11011	8437.059	8192	8671	148.786	1.305	0.3841	0.001	0.0011429	2416	33940	61.564	1054	14122	25.616	809	11011	19.973	33.4851	32.4425	76.7552	77.9705
DN	TBX2_2	cluster_3	DN	24820	19019.138	18757	19280	160.9629	1.305	0.384	0.001	0.0010909	11786	118013	74.082	2659	26010	16.328	2535	24820	15.581	21.5086	21.0316	95.3366	95.4248
DN	ATF4_2	cluster_3	DN	12967	9937.055	9721	10104	115.7476	1.3049	0.3839	0.001	0.0010909	4963	59634	73.418	1173	13733	16.907	1105	12967	15.964	22.2648	21.7443	94.2029	94.4222
DP	E2F_known24	cluster_6	Common_2	24622	18871.872	18509	19281	223.2602	1.3047	0.3837	0.001	0.001	2757	47016	60.976	1910	31448	40.785	1471	24622	31.933	53.3551	52.3694	77.0157	78.2943
SP8	SOX2_6	cluster_6	Common_2	10823	8296.108	8046	8536	148.5926	1.3046	0.3836	0.001	0.0010435	1941	31108	57.681	987	14899	27.626	705	10823	20.068	36.3215	34.7917	71.4286	72.6425
SP8	SP8_1	cluster_6	Common_2	469525	359946.33	358797	361113	682.3972	1.3044	0.3834	0.001	0.001	67362	823083	65.87	45886	551019	44.097	38968	469525	37.575	57.8486	57.0447	84.9235	85.2103
SP4	POU2F3_1	cluster_6	Common_2	21285	16318.878	16013	16639	188.9843	1.3043	0.3833	0.001	0.0010435	3852	58181	62.51	1853	26777	28.77	1447	21285	22.869	37.5649	36.5841	78.0896	79.4899
SP4	FOXO3_4	cluster_6	Common_2	9029	6923.146	6733	7094	116.5393	1.3041	0.3831	0.001	0.0010909	2012	26245	62.811	913	11306	27.058	718	9029	21.609	35.6859	34.4027	78.6418	79.8603
SP4	POU2F2_known12	cluster_6	Common_2	16636	12756.272	12546	13001	140.7093	1.3041	0.3831	0.001	0.0010435	4643	46514	63.727	2101	20388	27.933	1695	16636	22.792	36.5066	35.7656	80.6759	81.597
SP4	RUNX1_4	cluster_6	Common_2	9923	7609.726	7421	7783	113.5094	1.3039	0.3829	0.001	0.0010909	3165	32119	63.475	1246	12216	24.142	991	9923	19.61	31.3112	30.8945	79.5345	81.2295
DN	CUX2_2	cluster_3	DN	522	400.1	359	432	22.4112	1.3039	0.3828	0.001	0.0013333	368	3312	68.275	69	591	12.183	60	522	10.761	16.3043	15.7609	86.9565	88.3249
SP4	SOX21_3	cluster_6	Common_2	19644	15068.052	14766	15401	184.3279	1.3037	0.3826	0.001	0.0010909	3663	54981	62.433	1735	24817	28.181	1348	19644	22.307	36.8004	35.7287	77.6945	79.1554
SP8	SOX11_2	cluster_6	Common_2	18630	14292.234	13953	14594	197.8198	1.3035	0.3824	0.001	0.0010909	3666	51361	60.532	1822	24297	28.635	1368	18630	21.956	37.3159	36.2727	75.0823	76.6761
DP	KLF4_1	cluster_6	Common_2	436201	334672.179	333718	335694	597.2428	1.3034	0.3822	0.001	0.001	76804	740702	63.769	54917	525754	45.263	45395	436201	37.554	59.105	58.8902	82.6611	82.9667
SP8	ELK3_2	cluster_6	Common_2	29153	22370.887	21999	22762	237.14	1.3032	0.382	0.001	0.0011429	5710	51570	24.526	10075	90986	43.272	3230	29153	13.865	56.5674	56.5309	32.0596	32.0412
DP	SP8_1	cluster_2	DP	105497	80960.833	80297	81584	377.9166	1.3031	0.3819	0.001	0.001	63765	778558	62.307	11105	131511	10.525	8787	105497	8.4428	13.7803	13.5503	79.1265	80.2191
DP	MYCN_2	cluster_6	Common_2	92260	70823.821	70377	71299	278.7686	1.3027	0.3815	0.001	0.001	17531	158428	65.545	12049	108167	44.751	10231	92260	38.17	58.3595	58.2347	84.9116	85.294
DN	NFE2L1::MAFG_1	cluster_3	DN	54464	41825.392	41255	42296	321.607	1.3022	0.3809	0.001	0.0010435	11526	243206	74.83	2840	57401	17.661	2664	54464	16.758	23.113	22.3942	93.8028	94.8834
SP8	SOX17_4	cluster_6	Common_2	13139	10093.103	9854	10369	150.3155	1.3018	0.3805	0.001	0.0010909	2660	37246	58.82	1317	17642	27.861	965	13139	20.75	36.2782	35.2763	73.2726	74.4757
SP4	PBX1_4	cluster_6	Common_2	12702	9759.862	9506	9966	137.0041	1.3014	0.3801	0.001	0.0010909	2946	33026	61.318	1474	16039	29.779	1152	12702	23.583	39.1039	38.4606	78.1547	79.1945
SP4	SOX9_6	cluster_6	Common_2	23583	18120.071	17737	18453	208.5034	1.3015	0.3801	0.001	0.0010909	4528	68048	61.63	2124	30252	27.399	1628	23583	21.359	35.9541	34.6564	76.6478	77.9552
DP	EGR4_1	cluster_6	Common_2	204241	156943.144	156309	157625	424.6768	1.3014	0.38	0.001	0.001	29511	334771	66.296	21237	238147	47.161	18118	204241	40.447	61.3941	61.0092	85.3134	85.7626
DP	CTCF_known1	cluster_6	Common_2	560878	431031.163	429237	432960	1128.9635	1.3012	0.3799	0.001	0.0010435	55720	1030489	59.443	39885	727160	41.946	30472	560878	32.354	54.6877	54.4283	76.3996	77.1327
SP4	KLF16_1	cluster_6	Common_2	503404	386880.144	385767	388030	722.0958	1.3012	0.3798	0.001	0.001	76586	858580	65.244	54190	597132	45.377	45524	503404	38.254	59.4417	58.6322	84.0081	84.3036
SP4	MYBL2_2	cluster_6	Common_2	17181	13208.507	12947	13477	159.307	1.3007	0.3793	0.001	0.0010435	2320	35130	65.23	1404	20538	38.135	1157	17181	31.902	49.8707	48.9069	82.4074	83.6547
SP4	TCF7L1_2	cluster_6	Common_2	45714	35146.796	34756	35550	251.3368	1.3007	0.3792	0.001	0.0010435	12702	140496	61.647	5380	57741	25.336	4203	45714	20.058	33.0893	32.5376	78.1227	79.1708
SP8	IRF_known21	cluster_6	Common_2	46600	35827.963	35421	36260	269.9242	1.3007	0.3792	0.001	0.001	7912	114984	65.268	3962	55647	31.587	3274	46600	26.451	41.3802	40.5274	82.635	83.7422
SP8	SOX15_4	cluster_6	Common_2	9827	7556.27	7342	7804	139.7745	1.3005	0.379	0.001	0.0011429	2193	30727	55.692	1053	13999	25.373	724	9827	17.811	33.0141	31.9816	68.7559	70.1979
SP8	HMGA1_1	cluster_6	Common_2	8604	6616.437	6498	6748	75.6987	1.3004	0.3789	0.001	0.0010435	3543	21381	63.056	1786	10593	31.24	1441	8604	25.375	40.6717	40.2413	80.6831	81.2234
DN	POU2F2_known15	cluster_3	DN	9260	7121.184	6962	7259	90.6428	1.3003	0.3788	0.001	0.0010435	3911	43212	75.23	905	9687	16.865	865	9260	16.121	22.1171	21.4292	95.5801	95.592
SP4	CUX2_1	cluster_4	SP4	1452	1116.585	1029	1194	51.4678	1.3001	0.3787	0.001	0.0012632	439	7949	74.631	110	1680	15.773	95	1452	13.633	21.6401	18.2664	86.3636	86.4286
DN	PKNOX2_2	cluster_3	DN	47664	36661.838	36289	37010	220.1157	1.3001	0.3786	0.001	0.0010435	15871	176400	72.498	4632	50861	20.903	4322	47664	19.589	27.2321	27.0204	93.3074	93.7142
SP4	PATZ1_1	cluster_4	SP4	74054	56966.955	56295	57561	370.021	1.2999	0.3784	0.001	0.001	71621	944599	64.129	7074	90275	6.1288	5748	74054	5.0276	8.0256	7.8397	81.2553	82.0316
SP4	TCF7L2_known5	cluster_6	Common_2	57409	44178.081	43512	44750	351.5307	1.2995	0.3779	0.001	0.0010435	11860	190547	60.832	4751	73608	23.499	3634	57409	18.328	30.6408	30.1285	76.4892	77.9929
SP4	CUX2_2	cluster_4	SP4	640	492.378	445	532	25.911	1.2992	0.3776	0.001	0.0013333	387	3483	71.8	84	716	14.76	75	640	13.193	19.3798	18.375	89.2857	89.3855
SP4	ATF6_1	cluster_4	SP4	2968	2285.403	2197	2374	55.24	1.2985	0.3769	0.001	0.0010435	3915	27415	63.572	521	3605	8.3596	427	2968	6.8825	10.9068	10.8262	81.9578	82.3301
SP8	SOX4_2	cluster_6	Common_2	23872	18387.471	18040	18786	220.8103	1.2983	0.3766	0.001	0.0010909	4506	67750	58.989	2240	32027	27.886	1633	23872	20.785	36.2406	35.2354	72.9018	74.5371
SP8	SPDEF_6	cluster_6	Common_2	71630	55178.74	54698	55631	289.5748	1.2981	0.3764	0.001	0.001	8460	118816	68.647	5862	80942	46.765	5150	71630	41.385	60.8747	60.2865	87.854	88.4955
SP4	IRF_known14	cluster_6	Common_2	120319	92692.594	91888	93354	462.8207	1.298	0.3763	0.001	0.001	13966	293131	66.532	7220	143449	32.558	5917	120319	27.309	42.3672	41.0462	81.9529	83.8758
SP8	ELK3_1	cluster_6	Common_2	30585	23566.437	23124	23993	259.0361	1.2978	0.3761	0.001	0.0011429	5938	53644	24.833	10482	94751	43.862	3387	30585	14.158	57.0394	57.0148	32.3125	32.2793
DN	CUX1_8	cluster_5	Common_1_DN	2262	1742.946	1649	1838	58.8402	1.2976	0.3759	0.001	0.0011429	619	9964	75.019	164	2468	18.582	148	2262	17.031	23.9095	22.7017	90.2439	91.6532
DN	BPTF_1	cluster_3	DN	45756	35261.724	34908	35650	237.2137	1.2976	0.3758	0.001	0.0010909	15844	211822	75.51	3764	48126	17.156	3566	45756	16.311	22.5069	21.6012	94.7396	95.0754
SP4	ARID5A_1	cluster_6	Common_2	3999	3081.708	2946	3204	78.4709	1.2976	0.3758	0.001	0.0012632	810	11102	65.491	410	5064	29.873	314	3999	23.59	38.7654	36.0205	76.5854	78.9692
SP4	SOX2_6	cluster_6	Common_2	11618	8953.56	8705	9210	153.2879	1.2976	0.3758	0.001	0.0010435	2099	33627	62.352	987	14899	27.626	757	11618	21.542	36.0648	34.5496	76.6971	77.9784
SP8	ATF3_known4	cluster_6	Common_2	74335	57287.279	56821	57734	285.1509	1.2976	0.3758	0.001	0.001	8603	121435	69.067	5968	83194	47.317	5280	74335	42.279	61.3739	61.2138	88.4718	89.3514
SP4	CTCF_known1	cluster_4	SP4	66036	50899.422	50063	51660	490.0622	1.2974	0.3756	0.001	0.0010435	55221	1020372	58.86	4997	87610	5.0537	3677	66036	3.8093	6.6587	6.4718	73.5842	75.375
SP4	SOX9_4	cluster_6	Common_2	28141	21693.464	21327	22070	230.4819	1.2972	0.3754	0.001	0.0010909	5343	80252	62.068	2495	35804	27.691	1922	28141	21.765	35.9723	35.0658	77.0341	78.5974
SP8	SOX5_3	cluster_6	Common_2	12931	9968.392	9707	10235	154.9176	1.2972	0.3754	0.001	0.0011429	2486	37371	63.453	1177	16437	27.909	904	12931	21.956	36.3636	34.6017	76.8054	78.6701
DP	MYCN_2	cluster_2	DP	21903	16885.751	16578	17148	170.5283	1.2971	0.3753	0.001	0.001	17531	158428	65.545	2916	25887	10.71	2445	21903	9.0618	13.9467	13.8252	83.8477	84.61
SP4	SOX15_2	cluster_6	Common_2	15639	12059.388	11786	12380	174.6651	1.2968	0.375	0.001	0.0010909	3199	44866	62.621	1502	19922	27.806	1156	15639	21.828	36.1363	34.8571	76.964	78.5012
DN	FOXM1_1	cluster_3	DN	11081	8545.716	8391	8703	91.8168	1.2966	0.3748	0.001	0.0010909	6570	52629	74.145	1490	11672	16.444	1411	11081	15.611	21.4764	21.0549	94.698	94.9366
SP4	POU2F2_known13	cluster_6	Common_2	21806	16817.21	16428	17240	237.3245	1.2966	0.3748	0.001	0.0010909	2728	57442	64.703	1401	27261	30.707	1085	21806	24.562	39.7727	37.9618	77.4447	79.9897
SP4	HIF1A_1	cluster_6	Common_2	81191	62623.161	62181	62992	252.986	1.2965	0.3746	0.001	0.0010435	11555	130065	67.885	8327	92629	48.346	7265	81191	42.376	62.8732	62.4234	87.2463	87.6518
SP4	SOX10_4	cluster_6	Common_2	30616	23614.9	23178	24037	245.7253	1.2965	0.3746	0.001	0.0010909	5739	86325	61.805	2719	39147	28.027	2086	30616	21.92	36.3478	35.466	76.7194	78.2078
SP4	SPDEF_6	cluster_6	Common_2	70623	54471.056	53957	54952	285.1827	1.2965	0.3746	0.001	0.001	8360	117397	67.827	5862	80942	46.765	5077	70623	40.803	60.7297	60.1574	86.6087	87.2514
SP4	STAT_known3	cluster_6	Common_2	80460	62060.958	61397	62624	369.2819	1.2965	0.3746	0.001	0.0010435	7269	145730	67.556	4799	93340	43.27	4077	80460	37.299	56.0875	55.2117	84.9552	86.201
DP	KLF16_1	cluster_6	Common_2	497023	383471.435	382211	384715	728.7017	1.2961	0.3742	0.001	0.001	76014	850789	64.652	54190	597132	45.377	44987	497023	37.769	59.1825	58.4191	83.0172	83.235
SP4	SPDEF_6	cluster_4	SP4	7289	5625.727	5416	5839	127.5387	1.2956	0.3736	0.001	0.001	8360	117397	67.827	625	8420	4.8647	535	7289	4.2113	6.3995	6.2088	85.6	86.5677
DP	SP1_known3	cluster_6	Common_2	282089	217746.54	216859	218645	564.9607	1.2955	0.3735	0.001	0.001	39534	518521	65.051	26174	337202	42.304	21672	282089	35.389	54.8186	54.4026	82.7997	83.6558
SP4	STAT_known2	cluster_6	Common_2	105148	81165.981	80446	81820	418.9792	1.2955	0.3735	0.001	0.001	9013	180903	68.592	6134	120009	45.503	5302	105148	39.868	58.8261	58.124	86.4363	87.6168
SP4	ARID3A_2	cluster_6	Common_2	7611	5875.567	5692	6047	106.2243	1.2953	0.3733	0.001	0.0013333	1263	20439	64.999	641	9493	30.189	501	7611	24.204	39.6675	37.2376	78.1591	80.1749
SP8	PATZ1_1	cluster_1	SP8	82950	64041.578	63436	64663	377.8598	1.2952	0.3732	0.001	0.001	74077	975342	66.217	7774	98544	6.6902	6495	82950	5.6315	8.7679	8.5047	83.5477	84.1756
SP8	IRF6_1	cluster_1	SP8	5215	4026.36	3869	4179	94.4634	1.2951	0.3731	0.001	0.0010909	2749	44071	67.262	391	6065	9.2566	331	5215	7.9593	12.0407	11.8332	84.6547	85.9852
DP	SP1_known3	cluster_2	DP	76323	58942.62	58335	59475	353.5546	1.2949	0.3728	0.001	0.001	39534	518521	65.051	7198	91416	11.469	5927	76323	9.5751	14.9922	14.7194	82.3423	83.4898
DN	HMGA1_2	cluster_3	DN	18240	14092.426	13859	14333	147.9425	1.2943	0.3722	0.001	0.0010435	6789	95209	77.457	1457	19052	15.5	1384	18240	14.839	20.3859	19.1579	94.9897	95.738
DP	ERG_2	cluster_2	DP	12145	9384.626	9120	9656	159.7322	1.2941	0.372	0.001	0.001	6712	87427	60.427	1235	15627	10.801	949	12145	8.3942	14.1389	13.8916	76.8421	77.7181
SP8	E2F_known24	cluster_6	Common_2	26410	20407.604	20095	20743	202.032	1.2941	0.372	0.001	0.001	2992	51042	66.197	1910	31448	40.785	1584	26410	34.252	52.9412	51.7417	82.9319	83.9799
SP8	IRF4_2	cluster_6	Common_2	40034	30936.464	30536	31295	233.9054	1.2941	0.3719	0.001	0.001	6695	96589	66.045	3385	47396	32.408	2828	40034	27.374	42.2405	41.4478	83.5451	84.467
SP8	POU2F2_known13	cluster_6	Common_2	21223	16400.322	16010	16760	236.2745	1.294	0.3719	0.001	0.0010909	2653	55941	63.012	1401	27261	30.707	1059	21223	23.906	39.9171	37.9382	75.5889	77.8511
SP4	MYBL1_5	cluster_6	Common_2	23332	18031.754	17709	18324	194.3441	1.2939	0.3718	0.001	0.0010435	2888	46605	66.715	1754	27428	39.263	1470	23332	33.4	50.9003	50.0633	83.8084	85.0664
DP	SPZ1_1	cluster_6	Common_2	409627	316675.281	315364	317884	767.026	1.2935	0.3713	0.001	0.001	49189	720565	65.335	33952	488441	44.288	28202	409627	37.142	57.334	56.848	83.0643	83.8642
SP4	FLI1_2	cluster_4	SP4	10576	8176.605	7940	8420	144.99	1.2934	0.3712	0.001	0.001	7024	91445	65.047	1001	12687	9.0246	822	10576	7.523	11.7027	11.5654	82.1179	83.3609
SP8	KLF4_1	cluster_6	Common_2	453784	350922.638	349984	351958	599.948	1.2931	0.3709	0.001	0.001	80637	776764	66.873	54917	525754	45.263	47240	453784	39.067	58.5835	58.4198	86.0207	86.3111
SP8	KLF12_2	cluster_6	Common_2	336846	260503.851	259658	261537	614.9787	1.2931	0.3708	0.001	0.001	40686	583267	67.525	27197	386464	44.741	23577	336846	38.997	57.9487	57.7516	86.6897	87.161
SP4	SOX7_3	cluster_6	Common_2	19755	15279.213	14915	15608	205.8283	1.2929	0.3706	0.001	0.0010909	3572	57193	62.928	1672	25254	27.786	1281	19755	21.736	35.8623	34.5409	76.6148	78.2252
DN	MAF_known1	cluster_3	DN	108170	83668.194	83060	84244	367.4441	1.2928	0.3705	0.001	0.001	33530	506311	73.776	7841	115038	16.763	7317	108170	15.762	21.8222	21.3643	93.3172	94.0298
SP8	HIF1A_2	cluster_6	Common_2	116841	90410.762	89895	90935	318.6036	1.2923	0.37	0.001	0.001	13715	183722	69.394	9872	130767	49.392	8770	116841	44.132	63.9446	63.5966	88.8371	89.3505
SP4	SOX15_4	cluster_6	Common_2	10363	8019.622	7797	8281	141.9871	1.2922	0.3698	0.001	0.0011429	2335	32728	59.319	1053	13999	25.373	763	10363	18.783	32.6767	31.664	72.4596	74.0267
DP	ATF3_known4	cluster_6	Common_2	72359	56006.604	55571	56401	273.412	1.292	0.3696	0.001	0.001	8408	118694	67.508	5968	83194	47.317	5142	72359	41.155	61.156	60.9626	86.1595	86.9762
SP4	MYBL2_4	cluster_6	Common_2	11630	9001.536	8792	9210	125.8029	1.292	0.3696	0.001	0.0010435	1678	23553	66.504	990	13637	38.505	837	11630	32.838	49.8808	49.378	84.5455	85.2827
DP	ETS_known13	cluster_6	Common_2	97650	75587.171	75057	76071	309.7273	1.2919	0.3695	0.001	0.001	10314	165505	67.697	7079	111951	45.792	6120	97650	39.942	59.3368	59.0012	86.4529	87.2257
SP8	SP100_1	cluster_1	SP8	1115	862.846	797	925	38.5869	1.2919	0.3695	0.001	0.0013333	956	12440	73.99	97	1202	7.1492	90	1115	6.6318	9.4142	8.963	92.7835	92.7621
SP8	ATF2_2	cluster_1	SP8	5091	3941.357	3776	4088	95.8398	1.2916	0.3692	0.001	0.0010435	5120	56438	65.757	567	6053	7.0525	469	5091	5.9316	9.1602	9.0205	82.716	84.1071
DN	ELK3_2	cluster_3	DN	6825	5284.724	5045	5501	141.8342	1.2914	0.3689	0.001	0.0011429	6130	55327	26.313	2266	20219	9.6161	765	6825	3.2459	12.4796	12.3357	33.7599	33.7554
DP	STAT_known2	cluster_2	DP	20117	15579.004	15230	15926	219.3639	1.2913	0.3688	0.001	0.001	8789	176424	66.894	1258	23721	8.9942	1041	20117	7.6276	11.8444	11.4026	82.7504	84.8067
SP8	KLF16_1	cluster_6	Common_2	516412	399934.43	398874	401029	679.2176	1.2912	0.3688	0.001	0.001	79277	887559	67.446	54190	597132	45.377	46727	516412	39.243	58.9414	58.1834	86.2281	86.4821
SP4	SOX18_4	cluster_6	Common_2	11023	8536.645	8276	8791	146.6782	1.2912	0.3687	0.001	0.0012	2335	32760	62.2	1070	14264	27.082	815	11023	20.929	34.9036	33.6477	76.1682	77.2785
SP8	IRF4_1	cluster_6	Common_2	21189	16409.896	16155	16670	156.3761	1.2912	0.3687	0.001	0.0010435	3249	45692	67.371	1798	24589	36.256	1527	21189	31.243	46.9991	46.3735	84.9277	86.1727
DN	PKNOX1_1	cluster_3	DN	53681	41583.11	41143	42038	269.8026	1.2909	0.3684	0.001	0.0010435	15318	230880	74.405	3874	56704	18.274	3636	53681	17.3	23.7368	23.2506	93.8565	94.6688
DP	ATF2_1	cluster_6	Common_2	5937	4601.634	4512	4679	50.625	1.2901	0.3675	0.001	0.0010435	1296	9072	66.75	984	6855	50.438	850	5937	43.683	65.5864	65.4431	86.3821	86.6083
SP4	STAT_known2	cluster_4	SP4	14886	11538.128	11216	11852	187.4738	1.2901	0.3675	0.001	0.001	9013	180903	68.592	910	17211	6.5258	776	14886	5.6442	8.6098	8.2287	85.2747	86.4912
SP4	FOXO1_4	cluster_6	Common_2	10382	8049.388	7843	8273	128.1673	1.2898	0.3671	0.001	0.0010909	2354	30679	61.609	1097	13493	27.097	830	10382	20.849	35.2591	33.8407	75.6609	76.9436
SP4	SOX11_2	cluster_6	Common_2	19551	15158.021	14891	15452	170.3856	1.2898	0.3671	0.001	0.0010909	3896	54587	64.334	1822	24297	28.635	1439	19551	23.042	36.9353	35.8162	78.9791	80.4667
SP4	SOX8_2	cluster_6	Common_2	27805	21559.449	21171	21954	223.9831	1.2897	0.367	0.001	0.0010909	5294	79524	62.195	2477	35522	27.781	1903	27805	21.746	35.9464	34.9643	76.8268	78.2754
SP4	HBP1_1	cluster_6	Common_2	25756	19979.479	19610	20306	210.3309	1.2891	0.3664	0.001	0.0010435	4429	68253	63.399	2217	32501	30.189	1724	25756	23.924	38.9253	37.7361	77.7627	79.2468
SP4	SPDEF_4	cluster_6	Common_2	41531	32217.107	31766	32709	280.2313	1.2891	0.3664	0.001	0.0010435	7657	115318	63.966	3617	51863	28.768	2842	41531	23.037	37.1164	36.0143	78.5734	80.0783
SP8	MYCN_2	cluster_1	SP8	10238	7943.062	7746	8150	124.0764	1.2889	0.3661	0.001	0.001	17431	157501	65.162	1383	12270	5.0764	1146	10238	4.2357	6.5745	6.5003	82.8633	83.4393
SP8	E2F2_2	cluster_6	Common_2	25718	19955.703	19634	20319	205.4133	1.2887	0.366	0.001	0.0010909	3179	54200	66.243	1874	30764	37.6	1544	25718	31.432	48.5687	47.4502	82.3906	83.5977
SP4	TCF7_1	cluster_6	Common_2	53187	41272.746	40662	41905	363.539	1.2887	0.3659	0.001	0.0010435	11748	188705	61.707	4400	67875	22.195	3384	53187	17.392	28.8049	28.1853	76.9091	78.3602
DN	MAF_known10	cluster_3	DN	26681	20704.601	20445	20953	157.1408	1.2886	0.3658	0.001	0.0010909	11107	122400	74.849	2609	28034	17.143	2470	26681	16.316	22.2382	21.7982	94.6723	95.1737
SP4	KLF7_1	cluster_6	Common_2	716199	555811.842	554146	557361	952.9071	1.2886	0.3658	0.001	0.001	72331	1170811	66.136	52413	841335	47.525	44405	716199	40.456	61.3914	61.1712	84.7213	85.1265
SP4	SOX17_4	cluster_6	Common_2	13787	10698.71	10415	11000	167.3319	1.2886	0.3658	0.001	0.0010909	2822	39514	62.402	1317	17642	27.861	1014	13787	21.773	35.932	34.8914	76.9932	78.1487
SP4	EGR4_1	cluster_6	Common_2	203690	158105.633	157365	158800	436.9063	1.2883	0.3655	0.001	0.001	29753	337110	66.76	21237	238147	47.161	18081	203690	40.338	60.7703	60.4224	85.1391	85.5312
DN	TBX5_5	cluster_3	DN	30915	23997.989	23615	24406	238.6062	1.2882	0.3654	0.001	0.0010435	7574	144355	74.742	1794	32801	16.983	1673	30915	16.007	22.0887	21.416	93.2553	94.2502
DP	KLF12_2	cluster_2	DP	66469	51611.909	50984	52310	376.0193	1.2879	0.365	0.001	0.001	38298	549311	63.594	5930	81999	9.4931	4735	66469	7.6952	12.3636	12.1004	79.8482	81.0607
SP4	HMGA1_1	cluster_6	Common_2	9003	6991.508	6875	7127	78.6435	1.2877	0.3648	0.001	0.0010435	3745	22602	66.657	1786	10593	31.24	1507	9003	26.551	40.2403	39.8328	84.3785	84.9901
SP8	SOX13_1	cluster_6	Common_2	11236	8727.464	8495	8951	141.0804	1.2874	0.3645	0.001	0.0010909	2089	31443	63.156	1044	14517	29.159	786	11236	22.569	37.6257	35.7345	75.2874	77.3989
DP	HIF1A::ARNT_1	cluster_6	Common_2	44294	34411.003	34189	34634	148.9935	1.2872	0.3642	0.001	0.001	10494	75476	67.161	7202	51412	45.748	6185	44294	39.414	58.9384	58.6862	85.8789	86.155
SP4	SOX3_1	cluster_6	Common_2	11929	9267.938	8989	9532	158.7639	1.2871	0.3641	0.001	0.0010909	2214	35441	64.162	1001	15050	27.246	778	11929	21.596	35.14	33.6588	77.7223	79.2625
SP4	SP4_2	cluster_6	Common_2	590575	458840.039	457390	460231	848.8593	1.2871	0.3641	0.001	0.001	57022	957821	67.232	40984	683173	47.954	35269	590575	41.454	61.8516	61.6582	86.0555	86.4459
DN	LMO2_2	cluster_3	DN	7778	6046.112	5923	6176	77.2811	1.2864	0.3633	0.001	0.0010435	4777	38246	75.086	1039	8150	16	988	7778	15.27	20.6824	20.3368	95.0914	95.4356
SP4	MYCN_2	cluster_4	SP4	10213	7940.486	7745	8125	114.9397	1.2862	0.3631	0.001	0.001	17249	155885	64.493	1415	12426	5.1409	1151	10213	4.2254	6.6729	6.5516	81.3428	82.1906
SP4	IRF4_2	cluster_6	Common_2	40692	31644.534	31250	32045	247.9278	1.2859	0.3628	0.001	0.001	6862	99125	67.779	3385	47396	32.408	2874	40692	27.824	41.8828	41.0512	84.904	85.8553
SP8	FOXP1_1	cluster_6	Common_2	86854	67545.536	66678	68366	506.46	1.2859	0.3627	0.001	0.0010435	10498	244896	63.1	6055	120673	31.093	4181	86854	22.379	39.8266	35.4657	69.0504	71.9747
SP4	KLF14_1	cluster_6	Common_2	421548	327882.04	326872	328877	611.3423	1.2857	0.3625	0.001	0.001	49055	664031	67.164	36453	489216	49.482	31309	421548	42.638	63.8243	63.4832	85.8887	86.1681
SP4	TCF7L1_1	cluster_6	Common_2	48301	37572.056	36986	38097	329.1101	1.2855	0.3624	0.001	0.0010435	10758	172842	61.696	4005	61781	22.053	3072	48301	17.241	28.5555	27.9452	76.7041	78.181

SP4	IRF_known21	cluster_6	Common_2	47637	37062.857	36608	37572	287.5859	1.2853	0.3621	0.001	0.001	8196	119181	67.65	3962	55647	31.587	3349	47637	27.04	40.8614	39.9703	84.528	85.6057
DN	SOX3_1	cluster_5	Common_1_DN	8301	6459.148	6276	6661	118.5471	1.2851	0.3619	0.001	0.0010909	2538	40630	73.556	651	9351	16.929	573	8301	15.028	22.5768	20.4307	88.0184	88.7713
DN	ARID3A_2	cluster_3	DN	5312	4134.269	4001	4279	87.0814	1.2848	0.3615	0.001	0.0013333	1580	25566	81.304	379	5462	17.37	363	5312	16.893	22.9747	20.7776	95.7784	97.2538
DN	ARID5A_1	cluster_3	DN	2883	2243.923	2148	2335	59.1581	1.2847	0.3614	0.001	0.0012632	992	13600	80.227	232	2962	17.473	224	2883	17.007	22.5806	21.1985	96.5517	97.3329
DP	CTCF_known2	cluster_6	Common_2	141477	110125.342	109035	111261	687.5111	1.2847	0.3614	0.001	0.0010435	14645	235553	28.191	24385	390108	46.688	8801	141477	16.932	60.0956	60.0616	36.0919	36.2661
SP8	CUX1_9	cluster_6	Common_2	3957	3079.836	2946	3209	81.5097	1.2847	0.3614	0.001	0.0012632	577	9969	67.331	296	4797	32.399	239	3957	26.726	41.4211	39.693	80.7432	82.4891
SP4	TCF7_2	cluster_6	Common_2	43546	33902.142	33458	34307	256.5829	1.2845	0.3612	0.001	0.0010435	13256	146350	61.849	5168	55411	23.417	4009	43546	18.403	30.2429	29.7547	77.5735	78.5873
SP4	GATA_known16	cluster_6	Common_2	34363	26755.622	26224	27189	288.9967	1.2843	0.361	0.001	0.0010435	5122	107965	62.558	2227	44036	25.516	1687	34363	19.911	32.9364	31.8279	75.7521	78.0339
SP4	SOX10_9	cluster_6	Common_2	18120	14111.847	13813	14417	180.0865	1.284	0.3607	0.001	0.0010435	3684	51623	61.408	1761	23524	27.983	1327	18120	21.555	36.0206	35.1006	75.3549	77.0277
SP8	EGR4_1	cluster_6	Common_2	208309	162285.446	161589	162989	423.4617	1.2836	0.3602	0.001	0.001	30552	346098	68.54	21237	238147	47.161	18500	208309	41.252	60.5525	60.1879	87.1121	87.4708
DP	KLF13_1	cluster_2	DP	40121	31259.353	30692	31781	341.9629	1.2835	0.3601	0.001	0.001	19895	342302	61.998	3116	51120	9.2588	2397	40121	7.2667	12.0483	11.7209	76.9255	78.484
SP4	SOX4_2	cluster_6	Common_2	25159	19604.657	19291	19898	199.0658	1.2833	0.3599	0.001	0.0010909	4816	72432	63.066	2240	32027	27.886	1721	25159	21.906	35.735	34.7346	76.8304	78.5556
DP	SP100_1	cluster_6	Common_2	6644	5177.201	5043	5307	78.9188	1.2833	0.3598	0.001	0.0013333	864	11233	66.811	620	7821	46.518	519	6644	39.517	60.0694	59.1472	83.7097	84.9508
SP8	HIF1A::ARNT_1	cluster_1	SP8	4682	3648.808	3530	3759	66.9818	1.2831	0.3596	0.001	0.001	10355	74439	66.239	775	5516	4.9083	655	4682	4.1662	6.3254	6.2897	84.5161	84.8803
SP4	SOX13_1	cluster_6	Common_2	11584	9028.795	8803	9276	140.561	1.283	0.3595	0.001	0.0010909	2170	32640	65.561	1044	14517	29.159	812	11584	23.268	37.4194	35.4902	77.7778	79.7961
SP8	FOXO1_4	cluster_6	Common_2	9830	7661.536	7413	7905	140.5652	1.283	0.3595	0.001	0.0010909	2229	29043	58.324	1097	13493	27.097	785	9830	19.741	35.2176	33.8464	71.5588	72.8526
DN	SPIC_2	cluster_3	DN	57034	44457.547	44037	44910	263.7214	1.2829	0.3594	0.001	0.0010435	21125	275317	74.226	4773	60618	16.343	4469	57034	15.376	21.155	20.7158	93.6308	94.0876
DP	PATZ1_1	cluster_6	Common_2	534827	416894.224	415621	418218	791.6634	1.2829	0.3594	0.001	0.001	72910	959184	65.12	49543	644744	43.772	40996	534827	36.31	56.2282	55.7585	82.7483	82.9518
DP	PATZ1_1	cluster_2	DP	144248	112472.406	111631	113342	530.9175	1.2825	0.359	0.001	0.001	72910	959184	65.12	13788	175131	11.89	11244	144248	9.7931	15.4218	15.0386	81.5492	82.3658
SP4	POU2F2_known16	cluster_6	Common_2	12047	9393.82	9179	9647	145.9564	1.2824	0.3589	0.001	0.0010909	2634	34687	58.999	1305	16413	27.917	946	12047	20.491	35.915	34.7306	72.4904	73.3991
SP4	IRF4_1	cluster_6	Common_2	21326	16630.123	16334	16921	159.6368	1.2824	0.3588	0.001	0.0010435	3307	46509	68.576	1798	24589	36.256	1539	21326	31.445	46.5376	45.8535	85.5951	86.7298
DP	KLF16_1	cluster_2	DP	116050	90506.666	89834	91169	414.475	1.2822	0.3586	0.001	0.001	76014	850789	64.652	12962	141421	10.747	10488	116050	8.8187	13.7975	13.6403	80.9134	82.0599
SP4	ETV6_2	cluster_6	Common_2	41981	32740.814	32179	33235	303.638	1.2822	0.3586	0.001	0.0011429	10041	90698	24.501	14847	133594	36.088	4652	41981	11.34	46.33	46.2866	31.3329	31.4243
SP4	EGR1_known12	cluster_6	Common_2	494114	385447.461	384093	386651	775.8039	1.2819	0.3583	0.001	0.001	50263	834726	66.661	35995	584207	46.655	30219	494114	39.46	60.1218	59.1948	83.9533	84.5786
SP4	SOX4_1	cluster_6	Common_2	73616	57434.649	56881	58057	358.8183	1.2817	0.3581	0.001	0.0010435	12657	205161	65.153	5901	90966	28.888	4688	73616	23.378	37.0388	35.8821	79.4442	80.9269
SP4	HMGA1_2	cluster_6	Common_2	30693	23957.395	23593	24295	211.6693	1.2811	0.3574	0.001	0.0010435	5885	82554	67.162	2777	36941	30.053	2268	30693	24.97	38.5387	37.1793	81.6709	83.0865
DN	IRF_known14	cluster_3	DN	62676	48929.68	48419	49497	322.7346	1.2809	0.3572	0.001	0.001	16394	342215	77.672	3403	65717	14.916	3206	62676	14.226	19.5559	18.3148	94.211	95.3726
SP8	AHR::ARNT::HIF1A_1	cluster_6	Common_2	69755	54467.865	54147	54788	193.938	1.2807	0.3569	0.001	0.0010435	12611	103685	71.095	9395	76813	52.669	8532	69755	47.83	67.6552	67.2759	90.8143	90.8115
DP	EGR1_known12	cluster_6	Common_2	492299	384501.155	383349	385835	790.1386	1.2804	0.3565	0.001	0.001	50191	833460	66.56	35995	584207	46.655	30122	492299	39.315	60.0147	59.0669	83.6838	84.2679
SP4	HIF1A_2	cluster_6	Common_2	115457	90184.153	89628	90732	339.1053	1.2802	0.3564	0.001	0.001	13692	183403	69.274	9872	130767	49.392	8667	115457	43.609	63.2997	62.9526	87.7938	88.2922
DN	CUX2_2	cluster_5	Common_1_DN	657	512.991	467	552	26.2294	1.2802	0.3563	0.001	0.0013333	368	3312	68.275	97	790	16.285	79	657	13.544	21.4674	19.837	81.4433	83.1646
DP	KLF7_1	cluster_6	Common_2	708755	553706.692	552074	555364	972.879	1.28	0.3562	0.001	0.001	72055	1166887	65.914	52413	841335	47.525	43941	708755	40.036	60.9826	60.739	83.8361	84.2417
SP4	ATF3_known4	cluster_4	SP4	8116	6340.585	6127	6523	122.5746	1.28	0.3561	0.001	0.001	8312	117305	66.718	717	9606	5.4635	596	8116	4.616	7.1704	6.9187	83.1241	84.4889
DP	KLF4_1	cluster_2	DP	98364	76853.471	76233	77462	362.3423	1.2799	0.356	0.001	0.001	76804	740702	63.769	12881	121293	10.442	10326	98364	8.4684	13.4446	13.2798	80.1646	81.0962
SP4	ETV3_1	cluster_6	Common_2	69278	54136.548	53764	54494	223.3326	1.2797	0.3558	0.001	0.0010435	14073	127252	67.378	8919	80297	42.516	7658	69278	36.682	54.4163	54.4416	85.8616	86.2772
SP4	SOX30_1	cluster_6	Common_2	20510	16028.905	15669	16377	204.0577	1.2795	0.3556	0.001	0.0010435	3742	56229	63.32	1811	26029	29.311	1409	20510	23.096	37.6537	36.4758	77.8023	78.7967
SP4	MYBL2_4	cluster_4	SP4	2883	2253.27	2126	2378	75.1055	1.2793	0.3554	0.001	0.0010435	1678	23553	66.504	254	3426	9.6736	213	2883	8.1404	12.6937	12.2405	83.8583	84.1506
DP	HIF1A_1	cluster_6	Common_2	80324	62803.412	62384	63258	260.8558	1.279	0.355	0.001	0.0010435	11588	130466	68.094	8327	92629	48.346	7183	80324	41.923	61.9865	61.567	86.2616	86.7158
DP	EGR4_1	cluster_2	DP	43017	33659.232	33309	34034	232.6638	1.278	0.3539	0.001	0.001	29511	334771	66.296	4596	51128	10.125	3850	43017	8.5189	13.046	12.8497	83.7685	84.1359
DP	GMEB1_1	cluster_6	Common_2	65602	51336.004	50910	51742	257.7451	1.2779	0.3538	0.001	0.0010435	5680	92646	67.496	4719	76123	55.459	4041	65602	47.794	71.1444	70.8093	85.6325	86.1789
DN	TBX4_1	cluster_3	DN	24731	19354.627	19138	19562	121.3518	1.2778	0.3536	0.001	0.0010435	16022	112186	74.769	3769	26059	17.368	3577	24731	16.482	22.3256	22.0446	94.9058	94.9039
DN	ELK3_1	cluster_3	DN	6976	5459.647	5248	5708	148.103	1.2777	0.3535	0.001	0.0011429	6350	57329	26.539	2319	20674	9.5703	781	6976	3.2293	12.2992	12.1684	33.6783	33.7429
SP4	SOX12_1	cluster_6	Common_2	13563	10616.204	10405	10833	136.2699	1.2775	0.3534	0.001	0.0010435	3166	41308	65.459	1367	16802	26.625	1083	13563	21.493	34.2072	32.8338	79.2246	80.7225
SP4	SOX11_1	cluster_6	Common_2	67286	52674.473	52122	53324	361.3112	1.2774	0.3532	0.001	0.0010435	11736	190304	65.17	5407	83403	28.561	4292	67286	23.042	36.5712	35.3571	79.3786	80.6758
DN	TCF7L2_known7	cluster_5	Common_1_DN	49743	38944.844	38407	39343	273.2359	1.2773	0.3531	0.001	0.0010435	14358	201641	70.809	4202	56378	19.798	3668	49743	17.468	25.5467	24.6691	87.2918	88.2312
DN	TCF7L1_1	cluster_5	Common_1_DN	48919	38303.806	37877	38750	279.0152	1.2771	0.3529	0.001	0.0010435	12339	198117	70.718	3640	55600	19.846	3172	48919	17.462	25.7071	24.692	87.1429	87.9838
SP8	CTCF_known1	cluster_1	SP8	91402	71570.201	70619	72403	559.469	1.2771	0.3529	0.001	0.0010435	57414	1061210	61.215	6625	118066	6.8106	5044	91402	5.2725	8.7853	8.613	76.1358	77.416
SP4	TCF7L2_known7	cluster_6	Common_2	52128	40824.364	40294	41362	319.851	1.2769	0.3526	0.001	0.0010435	12562	176542	61.995	4944	66905	23.495	3784	52128	18.306	30.1226	29.5273	76.5372	77.9135
DN	MGA_2	cluster_3	DN	23920	18736.61	18449	19032	173.5955	1.2766	0.3523	0.001	0.0010909	6706	114146	75.028	1556	25429	16.714	1450	23920	15.723	21.6224	20.9556	93.1877	94.0658
SP4	HESX1_2	cluster_6	Common_2	4712	3691.695	3557	3815	82.2658	1.2763	0.352	0.001	0.0014118	906	12691	63.767	451	5980	30.047	346	4712	23.676	38.1898	37.1287	76.7184	78.796
SP4	FLI1_4	cluster_4	SP4	11188	8767.588	8534	8988	137.2901	1.276	0.3517	0.001	0.001	7443	97009	66.151	1063	13399	9.1369	874	11188	7.6292	11.7426	11.533	82.2201	83.4988
SP8	ETS_known13	cluster_1	SP8	9542	7477.288	7218	7722	154.2088	1.2761	0.3517	0.001	0.001	10079	161754	66.163	743	11441	4.6798	609	9542	3.903	6.0423	5.8991	81.965	83.4018
SP4	KLF12_2	cluster_4	SP4	45497	35658.839	35205	36234	294.6449	1.2759	0.3515	0.001	0.001	39236	562601	65.133	4070	55774	6.457	3277	45497	5.2672	8.352	8.0869	80.516	81.5739
DP	MTF1_1	cluster_6	Common_2	291418	228438.588	227412	229326	569.9615	1.2757	0.3513	0.001	0.001	40601	541813	68.67	25336	334415	42.384	21971	291418	36.934	54.1144	53.7857	86.7185	87.1426
SP8	FOXO3_1	cluster_6	Common_2	26971	21142.17	20801	21453	193.4582	1.2757	0.3513	0.001	0.0010435	5381	70700	64.693	2720	33781	30.911	2133	26971	24.679	39.6395	38.1485	78.4191	79.8407
DP	ATF6_1	cluster_2	DP	2821	2211.632	2115	2297	55.8865	1.2754	0.351	0.001	0.0010435	3671	25707	59.612	534	3697	8.573	404	2821	6.5416	11.0052	10.9737	75.6554	76.3051
DP	ERG_4	cluster_2	DP	11741	9205.133	8951	9446	143.5955	1.2755	0.351	0.001	0.001	6744	87870	61.259	1199	15137	10.553	917	11741	8.1852	13.5973	13.3618	76.4804	77.5649
SP8	GMEB2_2	cluster_1	SP8	3101	2431.145	2312	2542	72.2858	1.2754	0.351	0.001	0.001	2724	36104	65.136	297	3755	6.7744	238	3101	5.5945	8.7372	8.5891	80.1347	82.5832
DN	TBX5_3	cluster_3	DN	42486	33312.242	32956	33607	196.0018	1.2754	0.3509	0.001	0.001	20740	186864	75.435	5059	44697	18.044	4799	42486	17.151	23.1389	22.7363	94.8606	95.0534
DN	KLF14_1	cluster_3	DN	27735	21751.528	21138	22342	369.7218	1.2751	0.3506	0.001	0.001	16591	224164	22.673	7718	99743	10.089	2165	27735	2.8053	13.0492	12.3726	28.0513	27.8065
SP4	CBX5_1	cluster_6	Common_2	18005	14120.122	13852	14372	162.2185	1.2751	0.3506	0.001	0.0010435	4490	49467	65.073	2111	22302	29.338	1678	18005	23.685	37.3719	36.398	79.4884	80.7327
DN	ARID5B_1	cluster_3	DN	16757	13142.585	12922	13383	139.2446	1.275	0.3505	0.001	0.0010909	5946	77508	76.405	1409	17650	17.399	1333	16757	16.518	22.4184	21.6197	94.6061	94.9405
DN	HMGA1_1	cluster_3	DN	5059	3967.713	3886	4054	53.1686	1.275	0.3505	0.001	0.0010435	4316	26040	76.796	893	5260	15.513	858	5059	14.92	19.8795	19.4278	96.0806	96.1787
DN	TBX15_2	cluster_3	DN	32032	25123.299	24900	25369	141.3981	1.275	0.3505	0.001	0.0010909	20505	143570	74.719	4913	33885	17.635	4643	32032	16.671	22.6433	22.3111	94.5044	94.5315
DN	ETV3_1	cluster_3	DN	6333	4969.46	4736	5209	142.8099	1.2743	0.3497	0.001	0.0010435	5350	48305	25.577	2201	19610	10.383	712	6333	3.3532	13.3084	13.1104	32.3489	32.2947
DP	EGR1_known12	cluster_2	DP	113816	89343.094	88506	90122	493.1498	1.2739	0.3493	0.001	0.001	50191	833460	66.56	8547	136439	10.896	7022	113816	9.0893	13.9906	13.6558	82.1575	83.419
DP	SP4_2	cluster_6	Common_2	584624	458922.069	457661	460337	822.1449	1.2739	0.3493	0.001	0.001	56997	957954	67.242	40984	683173	47.954	34878	584624	41.036	61.1927	61.0284	85.1015	85.5748
SP4	ETV3_1	cluster_4	SP4	15137	11883.677	11643	12092	140.733	1.2737	0.3491	0.001	0.0010435	14073	127252	67.378	1986	17704	9.374	1685	15137	8.0148	11.9733	11.8953	84.8439	85.5005
SP8	EGR1_known12	cluster_6	Common_2	507981	398842.419	397671	400171	776.1339	1.2736	0.349	0.001	0.001	52057	864606	69.047	35995	584207	46.655	31084	507981	40.567	59.7115	58.7529	86.3564	86.9522
DN	IRF_known21	cluster_3	DN	24124	18944.742	18664	19242	173.2616	1.2734	0.3487	0.001	0.001	9344	135063	76.665	1832	25304	14.363	1738	24124	13.693	18.6002	17.8613	94.869	95.3367
DN	TCF7_1	cluster_5	Common_1_DN	53702	42172.082	41659	42689	315.8669	1.2734	0.3487	0.001	0.0010435	13527	217263	71.046	4000	61044	19.962	3483	53702	17.561	25.7485	24.7175	87.075	87.9726
SP4	SP8_1	cluster_4	SP4	67136	52742.864	52213	53384	355.903	1.2729	0.3481	0.001	0.001	64724	792047	63.386	7107	84496	6.7621	5555	67136	5.3728	8.5826	8.4763	78.1624	79.4546
SP8	KLF7_1	cluster_6	Common_2	734504	577083.045	575621	578627	936.1246	1.2728	0.348	0.001	0.001	75221	1217400	68.768	52413	841335	47.525	45577	734504	41.49	60.5908	60.3338	86.9574	87.3022
SP8	SP4_2	cluster_6	Common_2	603531	474279.365	472826	475706	882.2978	1.2725	0.3477	0.001	0.001	59003	991063	69.566	40984	683173	47.954	36064	603531	42.364	61.1223	60.8973	87.9953	88.3423
SP4	SOX5_1	cluster_6	Common_2	7879	6192.377	6044	6349	92.5988	1.2723	0.3475	0.001	0.0010909	2386	21481	63.838	1115	9815	29.169	887	7879	23.415	37.1752	36.6789	79.5516	80.2751
DP	KLF14_1	cluster_6	Common_2	417457	328192.704	327105	329304	655.0714	1.272	0.3471	0.001	0.001	49087	664659	67.227	36453	489216	49.482	31000	417457	42.224	63.1532	62.8077	85.041	85.3318
SP4	SOX5_3	cluster_6	Common_2	13246	10417.798	10173	10687	155.1787	1.2715	0.3465	0.001	0.0011429	2611	39250	66.643	1177	16437	27.909	929	13246	22.49	35.5802	33.7478	78.9295	80.5865
SP4	AHR::ARNT::HIF1A_1	cluster_6	Common_2	69018	54286.703	53965	54601	194.3814	1.2714	0.3464	0.001	0.0010435	12561	103445	70.93	9395	76813	52.669	8436	69018	47.324	67.1603	66.7195	89.7924	89.852
SP4	E2F_known24	cluster_6	Common_2	26350	20732.264	20418	21112	221.7705	1.271	0.3459	0.001	0.001	3037	51804	67.185	1910	31448	40.785	1578	26350	34.174	51.9592	50.8648	82.6178	83.7891
SP8	KLF12_2	cluster_1	SP8	58338	45905.999	45392	46514	342.1842	1.2708	0.3457	0.001	0.001	40686	583267	67.525	5003	69033	7.992	4175	58338	6.7538	10.2615	10.0019	83.4499	84.5074
SP4	FOXO4_2	cluster_6	Common_2	32985	25961.835	25601	26347	221.1042	1.2705	0.3454	0.001	0.0010435	6478	84929	66.37	3236	40377	31.554	2602	32985	25.777	40.1667	38.8383	80.4079	81.6925
SP4	HIF1A::ARNT_1	cluster_4	SP4	5429	4273.017	4153	4407	78.193	1.2705	0.3454	0.001	0.001	10457	75241	66.952	915	6438	5.7288	766	5429	4.8309	7.3252	7.2155	83.7158	84.3274
DP	SPZ1_1	cluster_2	DP	104143	81978.361	81073	82711	479.5683	1.2704	0.3452	0.001	0.001	49189	720565	65.335	9016	127108	11.525	7280	104143	9.4428	14.8001	14.453	80.7453	81.9327
DN	IRF4_2	cluster_3	DN	20557	16183.303	15913	16439	160.8335	1.2702	0.3451	0.001	0.001	7884	113080	77.321	1556	21390	14.626	1485	20557	14.056	18.8356	18.1792	95.437	96.1057
DN	SOX8_9	cluster_5	Common_1_DN	11589	9126.437	8917	9360	138.1552	1.2698	0.3446	0.001	0.0010909	3303	52872	68.642	940	13886	18.028	766	11589	15.046	23.191	21.919	81.4894	83.4582
DN	FOXO1_5	cluster_3	DN	78035	61456.885	61053	61933	261.5843	1.2697	0.3445	0.001	0.0010435	32721	361528	75.797	7636	82243	17.243	7216	78035	16.361	22.0531	21.5848	94.4997	94.8835
SP4	ELK4_2	cluster_6	Common_2	35845	28252.195	27854	28650	248.3148	1.2687	0.3434	0.001	0.0011429	7834	70855	26.241	11869	107416	39.782	3955	35845	13.275	50.4851	50.5892	33.3221	33.3703
DN	NFKB_known11	cluster_3	DN	27395	21593.461	21325	21911	180.4068	1.2687	0.3433	0.001	0.001	14339	173964	75.858	2446	28875	12.591	2313	27395	11.946	16.1308	15.7475	94.5626	94.8745
SP4	ETV4_2	cluster_4	SP4	4291	3382.055	3193	3567	113.9973	1.2687	0.3433	0.001	0.0011429	4906	44382	25.767	1473	13170	7.646	477	4291	2.4912	9.7228	9.6683	32.3829	32.5816
DP	FLI1_2	cluster_2	DP	12336	9724.2	9482	9961	141.3485	1.2686	0.3432	0.001	0.001	6679	86994	61.881	1240	15749	11.203	961	12336	8.7749	14.3884	14.1803	77.5	78.3288
DN	SOX5_3	cluster_5	Common_1_DN	9823	7744.27	7542	7934	118.2479	1.2684	0.343	0.001	0.0011429	2922	43910	74.555	789	10931	18.56	697	9823	16.679	23.8535	22.3708	88.3397	89.8637
SP8	SPDEF_6	cluster_1	SP8	6626	5223.837	5033	5406	113.1706	1.2684	0.343	0.001	0.001	8460	118816	68.647	572	7714	4.4568	485	6626	3.8282	5.7329	5.5767	84.7902	85.8958
DN	TBX4_2	cluster_3	DN	36162	28513.916	28131	28915	241.1828	1.2682	0.3428	0.001	0.0010909	8920	170122	76.154	2070	38122	17.065	1947	36162	16.188	21.8274	21.2565	94.058	94.8586
DP	ETV3_1	cluster_6	Common_2	68066	53673.768	53313	54082	230.4909	1.2681	0.3427	0.001	0.0010435	13947	126127	66.782	8919	80297	42.516	7518	68066	36.04	53.9041	53.9662	84.292	84.7678
SP8	CTCF_known2	cluster_6	Common_2	151008	119082.01	117800	120220	722.6372	1.2681	0.3427	0.001	0.0010435	15803	254255	30.429	24385	390108	46.688	9384	151008	18.073	59.3811	59.3923	38.4827	38.7093
SP8	KLF14_1	cluster_6	Common_2	432138	340839.29	339791	341975	655.8664	1.2679	0.3424	0.001	0.001	51058	690823	69.873	36453	489216	49.482	32119	432138	43.709	62.9069	62.5541	88.1107	88.3328
DN	SOX5_1	cluster_5	Common_1_DN	5603	4420.405	4314	4522	64.7623	1.2675	0.342	0.001	0.0010909	2705	24345	72.35	715	6233	18.524	639	5603	16.651	23.6229	23.015	89.3706	89.8925
SP4	ELK3_2	cluster_6	Common_2	30298	23903.909	23509	24305	245.5176	1.2675	0.342	0.001	0.0011429	6107	55145	26.227	10075	90986	43.272	3355	30298	14.41	54.937	54.9424	33.3002	33.2996
DP	HIF1A_2	cluster_6	Common_2	115387	91089.824	90508	91625	336.6064	1.2667	0.3411	0.001	0.001	13829	185302	69.991	9872	130767	49.392	8661	115387	43.583	62.6293	62.2697	87.733	88.2386
SP4	SOX8_9	cluster_6	Common_2	16740	13220.652	12898	13541	185.1084	1.2662	0.3405	0.001	0.0010909	2995	47951	62.253	1457	22004	28.567	1080	16740	21.733	36.0601	34.9106	74.1249	76.0771
SP4	ELK3_1	cluster_6	Common_2	31498	24881.846	24465	25278	246.1588	1.2659	0.3402	0.001	0.0011429	6270	56640	26.22	10482	94751	43.862	3485	31498	14.581	55.5821	55.6109	33.2475	33.2429
SP4	EGR4_2	cluster_6	Common_2	496470	392398.302	391151	393584	730.1105	1.2652	0.3394	0.001	0.001	48413	794683	69.102	35489	572963	49.822	30606	496470	43.171	63.2186	62.474	86.2408	86.6496
DN	SPIB_2	cluster_3	DN	63176	49939.074	49518	50367	241.4707	1.2651	0.3392	0.001	0.0010435	23403	305650	75.803	5238	66763	16.558	4936	63176	15.668	21.0913	20.6694	94.2344	94.6273
DN	XBP1_4	cluster_3	DN	13447	10629.12	10390	10840	144.6221	1.2651	0.3392	0.001	0.0010435	6101	79442	72.628	1167	14760	13.494	1051	13447	12.294	17.2267	16.9268	90.06	91.1043
SP8	CUX2_2	cluster_1	SP8	585	462.242	418	505	27.2289	1.265	0.3391	0.001	0.0013333	381	3429	70.686	79	675	13.915	69	585	12.059	18.1102	17.0604	87.3418	86.6667
SP4	KLF4_1	cluster_4	SP4	60662	47958.014	47452	48425	290.4281	1.2649	0.339	0.001	0.001	78036	752571	64.791	8010	74996	6.4566	6422	60662	5.2225	8.2295	8.0606	80.1748	80.887
SP8	ETV4_2	cluster_6	Common_2	26292	20786.343	20451	21166	215.738	1.2649	0.339	0.001	0.0011429	4775	43196	25.078	9121	82630	47.972	2906	26292	15.264	60.8586	60.8667	31.8605	31.819
SP8	CUX2_2	cluster_6	Common_2	1508	1192.051	1142	1248	34.629	1.2648	0.3389	0.001	0.0013333	381	3429	70.686	207	1769	36.467	176	1508	31.086	46.1942	43.9778	85.0242	85.2459
DN	MYBL1_3	cluster_3	DN	4129	3265.05	3165	3368	65.8309	1.2645	0.3386	0.001	0.0010909	1868	20588	77.661	400	4270	16.107	385	4129	15.575	20.6103	20.0554	96.25	96.6979
SP8	KLF4_1	cluster_1	SP8	72132	57050.607	56491	57619	335.509	1.2643	0.3384	0.001	0.001	80637	776764	66.873	9288	86550	7.4513	7695	72132	6.21	9.5428	9.2862	82.8488	83.3414
DN	SOX2_6	cluster_5	Common_1_DN	8261	6535.352	6330	6760	125.744	1.264	0.338	0.001	0.0010435	2362	37807	70.103	667	9744	18.068	558	8261	15.318	23.624	21.8505	83.6582	84.7804
DN	SMAD3_3	cluster_3	DN	8369	6623.123	6471	6769	88.3195	1.2636	0.3375	0.001	0.0010435	4812	43323	75.24	1019	8929	15.507	952	8369	14.535	19.7839	19.3177	93.4249	93.7283
SP4	ETV1_1	cluster_4	SP4	4336	3431.353	3254	3633	110.9988	1.2636	0.3375	0.001	0.0010909	5001	45274	25.882	1491	13333	7.6221	482	4336	2.4788	9.6381	9.5772	32.3273	32.5208
SP8	ATF2_1	cluster_6	Common_2	6119	4842.43	4761	4931	52.6253	1.2636	0.3375	0.001	0.0010435	1370	9590	70.561	984	6855	50.438	876	6119	45.022	63.9416	63.806	89.0244	89.2633
SP4	GMEB1_1	cluster_6	Common_2	67833	53697.4	53259	54188	269.399	1.2632	0.3371	0.001	0.0010435	5959	97141	70.771	4719	76123	55.459	4180	67833	49.419	70.146	69.8294	88.5781	89.1097
DN	RUNX1_2	cluster_5	Common_1_DN	19906	15758.972	15452	16046	178.9777	1.2631	0.337	0.001	0.0010435	5376	83982	74.445	1533	22215	19.692	1361	19906	17.645	25.3162	23.7027	88.7802	89.6061
DP	MTF1_1	cluster_2	DP	84315	66752.888	66059	67329	376.7648	1.2631	0.337	0.001	0.001	40601	541813	68.67	7485	97795	12.395	6403	84315	10.686	15.7705	15.5616	85.5444	86.2161
SP4	SP1_known2	cluster_6	Common_2	1001224	792765.972	791302	794356	978.8625	1.2629	0.3368	0.001	0.001	112736	1565680	69.085	82234	1146633	50.595	72080	1001224	44.179	63.937	63.9482	87.6523	87.3186
SP8	SP8_1	cluster_1	SP8	77842	61637.224	61035	62274	391.4907	1.2629	0.3367	0.001	0.001	67362	823083	65.87	8014	95072	7.6085	6482	77842	6.2296	9.6226	9.4574	80.8835	81.8769
DN	SPZ1_2	cluster_3	DN	37957	30057.7	29768	30361	180.5689	1.2628	0.3366	0.001	0.0010435	18565	186152	75.582	4088	40215	16.328	3843	37957	15.411	20.7002	20.3903	94.0068	94.3852
SP4	EGR3_3	cluster_6	Common_2	295635	234164.443	233319	235086	543.4047	1.2625	0.3363	0.001	0.001	31018	457668	70.037	23105	336738	51.531	20185	295635	45.241	65.0751	64.596	87.362	87.7938
DN	TCF7_2	cluster_5	Common_1_DN	42127	33370.041	32998	33741	237.9856	1.2624	0.3362	0.001	0.0010435	15144	167157	70.642	4487	48020	20.294	3910	42127	17.803	25.8188	25.2021	87.1406	87.728
DN	XBP1_2	cluster_3	DN	12343	9780.46	9512	10034	165.0492	1.262	0.3357	0.001	0.0010435	3231	74429	77.172	600	12989	13.468	565	12343	12.798	17.4868	16.5836	94.1667	95.0266
DN	SMAD4_1	cluster_3	DN	88673	70268.456	69666	70841	344.7029	1.2619	0.3356	0.001	0.001	32547	462220	75.022	6894	94795	15.386	6385	88673	14.392	19.6178	19.1842	92.6168	93.5419
DN	SOX9_6	cluster_5	Common_1_DN	19429	15397.152	15073	15734	199.0303	1.2618	0.3355	0.001	0.0010909	5272	79251	71.777	1562	22247	20.149	1345	19429	17.597	25.5121	24.5158	86.1076	87.3331

DN	SMAD_1	cluster_3	DN	33266	26366.364	26080	26623	154.565	1.2617	0.3353	0.001	0.0010435	20794	166413	76.255	4442	34936	16.009	4217	33266	15.243	20.2799	19.99	94.9347	95.2198
DN	RREB1_1	cluster_3	DN	192765	152804.276	152067	153679	497.5189	1.2615	0.3352	0.001	0.001	60217	1008607	79.968	13009	201534	15.979	12365	192765	15.283	20.5341	19.112	95.0496	95.6489
DN	POU2F2_known16	cluster_5	Common_1_DN	9890	7839.729	7636	8029	121.6861	1.2615	0.3351	0.001	0.0010909	3024	39849	67.778	965	11945	20.317	781	9890	16.822	25.8267	24.8187	80.9326	82.7961
DN	CUX2_1	cluster_5	Common_1_DN	1409	1117.111	1027	1193	50.9774	1.2611	0.3346	0.001	0.0012632	444	7998	75.092	100	1592	14.947	86	1409	13.229	19.3694	17.6169	86	88.505
DN	MYC_known20	cluster_3	DN	35849	28430.526	27984	28826	253.6507	1.2609	0.3345	0.001	0.001	11316	185626	73.632	2458	39039	15.485	2237	35849	14.22	19.7685	19.3125	91.009	91.8287
DN	SOX17_4	cluster_5	Common_1_DN	10615	8420.808	8221	8604	120.847	1.2605	0.334	0.001	0.0010909	3286	46015	72.668	919	12059	19.044	799	10615	16.764	24.3153	23.0686	86.9423	88.0255
SP8	ETV1_1	cluster_6	Common_2	26735	21209.405	20861	21572	220.5282	1.2605	0.334	0.001	0.0010909	4842	43840	25.062	9308	84323	48.205	2952	26735	15.284	60.9665	60.9831	31.7147	31.7055
SP4	E2F2_2	cluster_6	Common_2	25686	20378.648	20036	20685	196.0616	1.2604	0.3339	0.001	0.0010909	3236	55175	67.435	1874	30764	37.6	1541	25686	31.393	47.6205	46.5537	82.2305	83.4937
DN	ETV4_2	cluster_3	DN	4937	3917.292	3720	4128	124.893	1.2602	0.3337	0.001	0.0011429	5010	45275	26.285	1685	15032	8.7271	553	4937	2.8662	11.0379	10.9045	32.819	32.8433
DN	TCF7L1_2	cluster_5	Common_1_DN	40622	32236.46	31866	32597	222.3259	1.2601	0.3336	0.001	0.0010435	14657	161969	71.069	4326	46203	20.273	3773	40622	17.824	25.742	25.0801	87.2168	87.9207
DN	IKZF2_2	cluster_3	DN	9310	7388.361	7238	7536	88.2177	1.2601	0.3335	0.001	0.0010435	4704	47046	78.526	1013	9683	16.162	972	9310	15.54	20.6633	19.7891	95.9526	96.1479
SP4	ATF2_1	cluster_6	Common_2	6138	4872.521	4788	4957	50.823	1.2597	0.333	0.001	0.0010435	1377	9639	70.922	984	6855	50.438	880	6138	45.162	63.907	63.6788	89.4309	89.5405
SP4	EGR3_2	cluster_6	Common_2	442904	351623.662	350521	352800	669.8026	1.2596	0.333	0.001	0.001	45033	687127	69.845	33684	506918	51.527	29275	442904	45.02	65.0079	64.4574	86.9107	87.3719
DP	ELK4_2	cluster_6	Common_2	34956	27755.765	27320	28238	277.5698	1.2594	0.3327	0.001	0.0011429	7680	69469	25.728	11869	107416	39.782	3855	34956	12.946	50.1953	50.3188	32.4796	32.5426
DP	ETS_known13	cluster_2	DP	22297	17704.682	17319	18055	209.6934	1.2594	0.3327	0.001	0.001	10314	165505	67.697	1676	26269	10.745	1402	22297	9.1202	13.5932	13.4721	83.6516	84.8795
DN	SOX15_2	cluster_5	Common_1_DN	11907	9456.721	9257	9676	122.7458	1.2591	0.3324	0.001	0.0010909	3740	52487	73.258	1049	13569	18.939	908	11907	16.619	24.2781	22.6856	86.5586	87.7515
DP	HIF1A::ARNT_1	cluster_2	DP	10171	8083.171	7939	8239	92.8702	1.2583	0.3314	0.001	0.001	10494	75476	67.161	1713	12082	10.751	1433	10171	9.0505	13.6554	13.4758	83.6544	84.1831
SP8	GMEB2_3	cluster_6	Common_2	20006	15902.409	15682	16120	131.2292	1.258	0.3312	0.001	0.0010435	2094	29627	71.145	1593	22378	53.738	1417	20006	48.042	67.6695	67.5262	88.9517	89.4003
DN	MAF_known12	cluster_3	DN	34731	27608.707	27226	27969	224.7349	1.258	0.3311	0.001	0.0010435	9989	170271	77.196	2214	36416	16.51	2098	34731	15.746	21.0031	20.3975	94.7606	95.3729
SP4	ATF2_2	cluster_4	SP4	5441	4325.467	4186	4473	87.676	1.2578	0.3309	0.001	0.0010435	5130	56548	65.885	616	6617	7.7096	503	5441	6.3394	9.8051	9.6219	81.6558	82.2276
SP8	HESX1_2	cluster_6	Common_2	4631	3682.212	3527	3812	87.3437	1.2576	0.3307	0.001	0.0014118	899	12600	63.31	451	5980	30.047	341	4631	23.269	37.931	36.754	75.6098	77.4415
SP4	FOXO3_1	cluster_6	Common_2	27700	22029.251	21698	22353	189.8443	1.2574	0.3305	0.001	0.0010435	5633	74003	67.715	2720	33781	30.911	2198	27700	25.346	39.0201	37.4309	80.8088	81.9988
DN	FOXO6_3	cluster_3	DN	78625	62541.918	62037	63048	296.6044	1.2572	0.3302	0.001	0.0010435	28591	373838	76.349	6537	83159	16.984	6154	78625	16.058	21.5243	21.0318	94.141	94.5478
DN	E2F_known24	cluster_3	DN	8613	6851.334	6678	7030	107.0124	1.2571	0.3301	0.001	0.001	3603	61420	79.657	582	9023	11.702	553	8613	11.17	15.3483	14.0231	95.0172	95.4561
SP4	KLF16_1	cluster_4	SP4	68958	54877.684	54314	55403	336.2174	1.2566	0.3295	0.001	0.001	76586	858580	65.244	7768	85275	6.4801	6187	68958	5.2402	8.0785	8.0316	79.6473	80.8654
DP	EGR4_2	cluster_6	Common_2	498601	396824.927	395724	398009	740.3644	1.2565	0.3294	0.001	0.001	49002	804691	69.973	35489	572963	49.822	30754	498601	43.356	62.7607	61.9618	86.6578	87.0215
DN	MGA_3	cluster_3	DN	21340	16988.391	16699	17280	173.0042	1.2561	0.329	0.001	0.0010909	6415	109267	77.12	1383	22542	15.91	1297	21340	15.062	20.2182	19.5301	93.7816	94.6677
DN	NFATC1_3	cluster_3	DN	12656	10076.273	9905	10258	105.663	1.256	0.3288	0.001	0.0010909	4137	53815	79.338	1065	13171	19.418	1017	12656	18.658	24.583	23.5176	95.493	96.0899
SP4	ETV4_2	cluster_6	Common_2	26827	21358.68	21036	21696	209.7096	1.256	0.3288	0.001	0.0011429	4906	44382	25.767	9121	82630	47.972	2963	26827	15.575	60.3954	60.4457	32.4855	32.4664
SP4	SP4_1	cluster_6	Common_2	912023	726342.599	724769	728044	1029.2358	1.2556	0.3284	0.001	0.001	80215	1441564	69.533	58871	1048812	50.589	51033	912023	43.991	63.6203	63.2662	86.6861	86.9577
DP	KLF7_1	cluster_2	DP	149619	119178.064	118194	120199	601.343	1.2554	0.3282	0.001	0.001	72055	1166887	65.914	11820	183259	10.352	9500	149619	8.4516	13.1844	12.8221	80.3723	81.6435
SP8	GMEB1_1	cluster_6	Common_2	69131	55062.835	54668	55487	254.3142	1.2555	0.3282	0.001	0.0010435	6106	99511	72.498	4719	76123	55.459	4262	69131	50.365	69.8002	69.4707	90.3157	90.8149
DP	SPDEF_6	cluster_6	Common_2	70814	56411.179	55991	56823	271.9233	1.2553	0.328	0.001	0.001	8667	121736	70.334	5862	80942	46.765	5090	70814	40.913	58.7285	58.1701	86.8304	87.4873
DN	SOX15_4	cluster_5	Common_1_DN	9119	7265.946	7067	7474	127.111	1.255	0.3277	0.001	0.0011429	2731	38267	69.358	840	10927	19.805	690	9119	16.528	25.2655	23.8299	82.1429	83.4538
SP4	FOXP1_1	cluster_6	Common_2	88404	70463.831	69531	71329	540.8395	1.2546	0.3272	0.001	0.0010435	11002	256998	66.218	6055	120673	31.093	4266	88404	22.778	38.7748	34.3987	70.4542	73.2591
SP8	EGR4_2	cluster_6	Common_2	509243	406051.112	405002	407287	710.1569	1.2541	0.3267	0.001	0.001	50126	822981	71.563	35489	572963	49.822	31409	509243	44.282	62.6601	61.8779	88.5035	88.8789
DN	REL_1	cluster_3	DN	24964	19905.804	19675	20126	138.2706	1.2541	0.3266	0.001	0.001	15073	136291	76.106	3006	26515	14.806	2816	24964	13.94	18.6824	18.3167	93.6793	94.1505
DN	SPI1_known4	cluster_3	DN	52477	41844.417	41445	42276	241.4681	1.2541	0.3266	0.001	0.0010435	19284	251359	76.991	4360	55279	16.932	4123	52477	16.074	21.3804	20.8773	94.5642	94.9312
DN	NFKB_known10	cluster_3	DN	25391	20249.655	19998	20511	164.0453	1.2539	0.3264	0.001	0.001	12896	155666	77.254	2275	26672	13.237	2159	25391	12.601	16.7416	16.3112	94.9011	95.1972
DN	RUNX1_4	cluster_5	Common_1_DN	9265	7388.69	7223	7535	92.184	1.2539	0.3264	0.001	0.0010909	3683	37261	73.637	1062	10306	20.367	947	9265	18.31	25.7127	24.8651	89.1714	89.8991
DP	FLI1_4	cluster_2	DP	12214	9740.801	9484	10046	163.8519	1.2539	0.3264	0.001	0.001	7089	92416	63.019	1231	15560	10.611	955	12214	8.3288	13.4716	13.2163	77.5792	78.4961
SP8	SP1_known2	cluster_6	Common_2	1026245	818469.827	816788	820073	987.0843	1.2539	0.3264	0.001	0.001	116483	1617881	71.388	82234	1146633	50.595	73877	1026245	45.283	63.423	63.4314	89.8375	89.5007
SP4	ETV1_1	cluster_6	Common_2	27437	21884.438	21524	22246	225.5662	1.2537	0.3262	0.001	0.0010909	5001	45274	25.882	9308	84323	48.205	3028	27437	15.685	60.5479	60.6021	32.5312	32.538
DP	EGR3_3	cluster_6	Common_2	297236	237153.458	236316	238119	532.9858	1.2533	0.3258	0.001	0.001	31417	463984	71.003	23105	336738	51.531	20306	297236	45.486	64.6338	64.0617	87.8857	88.2692
SP8	HIF1A_1	cluster_1	SP8	8129	6485.447	6316	6663	107.6755	1.2534	0.3258	0.001	0.0010435	11568	130194	67.952	879	9649	5.0361	734	8129	4.2428	6.3451	6.2438	83.504	84.2471
SP8	ATF3_known4	cluster_1	SP8	9697	7737.551	7512	7946	130.7117	1.2532	0.3256	0.001	0.001	8603	121435	69.067	825	11313	6.4343	700	9697	5.5152	8.1367	7.9853	84.8485	85.7155
DN	NFATC1_1	cluster_3	DN	26225	20929.645	20608	21297	207.5416	1.253	0.3254	0.001	0.0010909	6650	128410	79.943	1524	27428	17.076	1444	26225	16.327	21.7143	20.4229	94.7507	95.614
DN	TCF7L2_known5	cluster_5	Common_1_DN	56116	44807.67	44380	45278	282.452	1.2524	0.3247	0.001	0.0010435	13945	223921	71.487	4181	64167	20.485	3616	56116	17.915	25.9304	25.0606	86.4865	87.4531
DN	SOX14_2	cluster_5	Common_1_DN	11251	8987.953	8797	9181	116.3407	1.2518	0.324	0.001	0.0010909	4224	46656	72.886	1208	12699	19.838	1062	11251	17.576	25.142	24.1148	87.9139	88.5975
DP	AHR::ARNT::HIF1A_1	cluster_6	Common_2	68603	54804.955	54493	55106	190.4887	1.2518	0.324	0.001	0.0010435	12707	104472	71.635	9395	76813	52.669	8390	68603	47.04	66.0266	65.6664	89.3028	89.3117
SP8	EGR3_3	cluster_6	Common_2	302795	241971.164	241107	242850	505.7372	1.2514	0.3235	0.001	0.001	32104	473266	72.424	23105	336738	51.531	20702	302795	46.336	64.4842	63.9799	89.5997	89.9201
DN	TBX2_1	cluster_3	DN	37809	30219.226	29864	30607	223.4641	1.2511	0.3233	0.001	0.0010435	11067	188839	77.429	2405	39675	16.268	2277	37809	15.503	20.5747	20.0218	94.6778	95.2968
SP4	GMEB2_3	cluster_6	Common_2	20324	16245.855	16042	16443	124.7371	1.251	0.3231	0.001	0.0010435	2133	30178	72.468	1593	22378	53.738	1439	20324	48.805	67.4637	67.3471	90.3327	90.8213
DN	SOX30_1	cluster_5	Common_1_DN	16155	12922.769	12630	13183	165.9111	1.2501	0.322	0.001	0.0010435	4394	65950	74.266	1285	18059	20.336	1129	16155	18.192	25.6941	24.4958	87.8599	89.4568
DN	REL_2	cluster_3	DN	25066	20061.213	19844	20299	135.8017	1.2495	0.3213	0.001	0.0010435	15212	137460	76.313	3018	26629	14.783	2829	25066	13.916	18.5972	18.2351	93.7376	94.1305
DN	KLF12_1	cluster_3	DN	26466	21182.572	20967	21380	124.2082	1.2494	0.3212	0.001	0.001	24570	147420	74.766	4821	28504	14.456	4462	26466	13.423	18.1604	17.9528	92.5534	92.8501
SP8	EGR3_2	cluster_6	Common_2	454074	363529.586	362464	364505	639.9969	1.2491	0.3209	0.001	0.001	46634	711154	72.287	33684	506918	51.527	30061	454074	46.156	64.4616	63.8503	89.2442	89.5754
SP8	KLF13_1	cluster_1	SP8	30713	24587.753	24126	25002	271.9318	1.2491	0.3209	0.001	0.001	20972	360883	65.363	2316	38167	6.9128	1832	30713	5.5627	8.7355	8.5105	79.1019	80.47
DP	HIF1A_1	cluster_2	DP	16747	13410.828	13145	13672	157.3325	1.2487	0.3205	0.001	0.0010435	11588	130466	68.094	1804	19792	10.33	1506	16747	8.7407	12.9962	12.8363	83.4812	84.615
DN	SOX7_3	cluster_5	Common_1_DN	15013	12025.842	11780	12300	157.9071	1.2484	0.3201	0.001	0.0010909	4157	66552	73.226	1166	17179	18.902	1006	15013	16.518	24.2001	22.5583	86.2779	87.3916
SP4	EGR1_known9	cluster_6	Common_2	250642	200768.772	200041	201492	445.7516	1.2484	0.3201	0.001	0.001	28464	386336	71.535	21069	282472	52.303	18606	250642	46.41	65.3668	64.8767	88.3098	88.7316
SP8	SP4_1	cluster_6	Common_2	936420	750162.896	748356	751966	1079.5049	1.2483	0.32	0.001	0.001	82983	1490454	71.892	58871	1048812	50.589	52432	936420	45.168	63.184	62.8278	89.0625	89.2839
DN	SOX11_2	cluster_5	Common_1_DN	14140	11329.496	11081	11563	146.7527	1.248	0.3197	0.001	0.0010909	4488	62863	74.087	1219	15941	18.787	1068	14140	16.665	23.7968	22.4934	87.6128	88.7021
SP8	KLF16_1	cluster_1	SP8	78364	62824.587	62251	63450	371.254	1.2473	0.3189	0.001	0.001	79277	887559	67.446	8719	94633	7.1912	7127	78364	5.955	8.99	8.8292	81.741	82.8083
DN	FOXO3_1	cluster_5	Common_1_DN	17196	13788.607	13545	14035	155.5488	1.2471	0.3186	0.001	0.0010435	6232	81801	74.85	1584	19294	17.655	1398	17196	15.735	22.4326	21.0217	88.2576	89.1262
DP	ELK3_2	cluster_6	Common_2	30205	24220	23810	24567	235.6407	1.2471	0.3186	0.001	0.0011429	6179	55790	26.533	10075	90986	43.272	3344	30205	14.365	54.1188	54.1405	33.1911	33.1974
DN	IKZF2_3	cluster_3	DN	14609	11715.323	11531	11908	109.9932	1.247	0.3184	0.001	0.0010909	7087	71094	80.484	1594	15124	17.122	1537	14609	16.539	21.6876	20.5489	96.4241	96.5948
DP	EGR3_2	cluster_6	Common_2	445637	357393.809	356313	358361	647.9455	1.2469	0.3184	0.001	0.001	45781	699461	71.099	33684	506918	51.527	29481	445637	45.298	64.3957	63.7115	87.5223	87.9111
DN	E2F2_2	cluster_3	DN	9737	7808.988	7606	8006	119.5963	1.2469	0.3183	0.001	0.0010909	3788	64579	78.928	657	10343	12.641	617	9737	11.901	16.2883	15.0777	93.9117	94.141
SP4	CUX1_9	cluster_6	Common_2	3980	3191.836	3058	3333	85.0638	1.2469	0.3183	0.001	0.0012632	597	10309	69.627	296	4797	32.399	239	3980	26.881	40.0335	38.607	80.7432	82.9685
SP4	EGR1_known12	cluster_4	SP4	66900	53656.033	53081	54239	356.9859	1.2468	0.3183	0.001	0.001	50263	834726	66.661	5218	82372	6.5782	4160	66900	5.3426	8.2765	8.0146	79.724	81.2169
DN	SOX13_1	cluster_5	Common_1_DN	8155	6540.787	6338	6730	113.4185	1.2468	0.3182	0.001	0.0010909	2495	37512	75.346	667	9165	18.409	582	8155	16.38	23.3267	21.7397	87.2564	88.9798
DN	RBPJ_2	cluster_3	DN	31917	25605.371	25336	25852	155.5057	1.2465	0.3179	0.001	0.0010909	13859	138744	77.101	3446	33705	18.73	3252	31917	17.736	23.4649	23.0042	94.3703	94.6951
DP	ATF3_known4	cluster_2	DP	14941	11985.834	11697	12277	174.0296	1.2465	0.3179	0.001	0.001	8408	118694	67.508	1302	17867	10.162	1075	14941	8.4978	12.7854	12.5878	82.5653	83.6234
DN	SOX1_2	cluster_5	Common_1_DN	13585	10900.666	10672	11138	140.5774	1.2462	0.3176	0.001	0.0010909	4151	58133	72.545	1185	15579	19.441	1021	13585	16.953	24.5965	23.3688	86.1603	87.2007
DN	SOX2_5	cluster_5	Common_1_DN	15166	12179.171	11932	12429	159.0802	1.2452	0.3164	0.001	0.0010909	4046	64802	73.448	1164	17280	19.586	1006	15166	17.19	24.8641	23.4036	86.4261	87.7662
DN	SOX3_3	cluster_5	Common_1_DN	12876	10344.095	10080	10606	157.1862	1.2447	0.3158	0.001	0.0010909	3414	54700	70.442	1032	15271	19.666	852	12876	16.582	24.9561	23.5393	82.5581	84.3167
DN	SOX7_4	cluster_5	Common_1_DN	12168	9776.305	9536	10025	149.3952	1.2446	0.3157	0.001	0.0010909	3341	53567	71.584	969	14283	19.087	811	12168	16.261	24.2742	22.7155	83.6945	85.1922
SP4	KLF7_1	cluster_4	SP4	84902	68229.71	67516	68915	438.1614	1.2444	0.3154	0.001	0.001	72331	1170811	66.136	6936	105944	5.9845	5470	84902	4.7959	7.5625	7.2516	78.8639	80.1386
DN	MYC_known13	cluster_3	DN	31411	25247.772	24952	25552	176.0886	1.2441	0.3151	0.001	0.0010909	12241	134783	75.676	3113	33619	18.876	2891	31411	17.636	23.6174	23.3049	92.8686	93.4323
SP4	KLF13_1	cluster_4	SP4	26192	21062.206	20612	21470	271.9031	1.2435	0.3145	0.001	0.001	20223	347988	63.027	2092	34137	6.1829	1572	26192	4.7439	7.7733	7.5267	75.1434	76.7261
DN	SOX11_1	cluster_5	Common_1_DN	50746	40813.194	40332	41269	285.3168	1.2434	0.3143	0.001	0.0010435	13495	218540	74.839	3781	56921	19.493	3320	50746	17.378	24.6017	23.2205	87.8075	89.1516
SP8	GMEB2_3	cluster_1	SP8	1532	1232.081	1144	1323	52.0069	1.2432	0.3141	0.001	0.0010435	2094	29627	71.145	130	1755	4.2144	112	1532	3.6789	5.3486	5.171	86.1538	87.2934
DN	FOXO6_1	cluster_5	Common_1_DN	9756	7847.956	7674	8032	113.3345	1.2431	0.3139	0.001	0.0010909	3202	41765	72.845	901	11153	19.453	780	9756	17.016	24.3598	23.3593	86.5705	87.4742
DN	ETS_known17	cluster_3	DN	43563	35057.455	34619	35485	282.1659	1.2426	0.3134	0.001	0.001	15240	260380	73.574	2924	48134	13.601	2608	43563	12.309	17.1129	16.7305	89.1929	90.5036
DN	CTCF_known2	cluster_5	Common_1_DN	87670	70554.45	69611	71525	560.9598	1.2426	0.3133	0.001	0.0010435	17849	287162	34.367	13031	205911	24.643	5477	87670	10.492	30.6852	30.5298	42.0305	42.5766
DN	HESX1_2	cluster_3	DN	3404	2739.648	2644	2837	57.2035	1.2424	0.3131	0.001	0.0014118	1143	16019	80.489	281	3567	17.923	265	3404	17.104	23.1846	21.2498	94.306	95.4303
DP	SPDEF_6	cluster_2	DP	16137	12989.277	12712	13273	172.7124	1.2423	0.313	0.001	0.001	8667	121736	70.334	1390	18717	10.814	1179	16137	9.3233	13.6033	13.2557	84.8201	86.2157
DN	SOX18_2	cluster_5	Common_1_DN	10392	8368.779	8178	8566	122.7639	1.2417	0.3124	0.001	0.0010909	3405	47699	72.637	919	11986	18.252	787	10392	15.825	23.1131	21.7866	85.6366	86.7012
DP	EGR4_2	cluster_2	DP	106429	85709.125	84964	86461	446.8849	1.2417	0.3124	0.001	0.001	49002	804691	69.973	7829	124130	10.794	6623	106429	9.2546	13.5158	13.2261	84.5957	85.74
DP	GMEB2_3	cluster_2	DP	3183	2564.502	2459	2693	71.5621	1.2411	0.3116	0.001	0.0010435	2081	29462	70.749	267	3659	8.7866	231	3183	7.6435	11.1004	10.8037	86.5169	86.991
DN	E2F_known26	cluster_3	DN	5663	4563.657	4418	4696	87.7691	1.2408	0.3113	0.001	0.0010435	2046	34813	80.496	379	5910	13.665	361	5663	13.094	17.6442	16.2669	95.2507	95.8206
SP4	SP4_2	cluster_4	SP4	66874	53903.873	53292	54582	386.3775	1.2406	0.3111	0.001	0.001	57022	957821	67.232	5244	82431	5.7861	4184	66874	4.6941	7.3375	6.9819	79.7864	81.1272
DP	ELK3_1	cluster_6	Common_2	31655	25516.268	25102	25929	243.3542	1.2406	0.311	0.001	0.0011429	6415	57946	26.824	10482	94751	43.862	3502	31655	14.654	54.5908	54.6284	33.4097	33.4086
SP8	EGR4_1	cluster_1	SP8	29871	24094.046	23747	24461	206.7026	1.2398	0.3101	0.001	0.001	30552	346098	68.54	3232	35618	7.0536	2682	29871	5.9155	8.7785	8.6308	82.9827	83.8649
DN	SOX4_1	cluster_5	Common_1_DN	54984	44354.091	43830	44885	309.1979	1.2397	0.3099	0.001	0.0010435	14546	235575	74.812	4095	61800	19.626	3587	54984	17.461	24.6597	23.3403	87.5946	88.9709
DN	ERG_2	cluster_3	DN	16662	13441.862	13194	13683	148.9583	1.2395	0.3098	0.001	0.001	8341	108611	75.068	1430	18064	12.485	1308	16662	11.516	15.6816	15.341	91.4685	92.2387
DN	SOX12_1	cluster_5	Common_1_DN	10375	8373.356	8160	8580	121.5407	1.239	0.3092	0.001	0.0010435	3600	46915	74.344	975	11756	18.629	852	10375	16.441	23.6667	22.1145	87.3846	88.2528
DP	SP4_2	cluster_2	DP	114959	92793.339	91982	93630	492.988	1.2389	0.309	0.001	0.001	56997	957954	67.242	8726	140136	9.8366	7061	114959	8.0693	12.3884	12.0005	80.9191	82.0339
DP	SP1_known2	cluster_6	Common_2	1004749	811287.483	809894	812775	914.7823	1.2385	0.3085	0.001	0.001	115319	1604370	70.792	82234	1146633	50.595	72256	1004749	44.334	62.6575	62.6258	87.8663	87.626
DN	SOX18_4	cluster_5	Common_1_DN	8680	7011.809	6832	7213	112.6583	1.2379	0.3079	0.001	0.0012	2707	37982	72.115	773	10090	19.157	656	8680	16.48	24.2335	22.8529	84.8642	86.0258
DP	GMEB2_3	cluster_6	Common_2	19645	15868.89	15644	16095	132.8588	1.2379	0.3079	0.001	0.0010435	2081	29462	70.749	1593	22378	53.738	1391	19645	47.175	66.8429	66.6791	87.3195	87.7871
DP	SP4_1	cluster_6	Common_2	915030	739431.213	737621	741077	1043.8692	1.2375	0.3074	0.001	0.001	81673	1469721	70.891	58871	1048812	50.589	51173	915030	44.136	62.656	62.2588	86.924	87.2444
SP8	HIF1A_2	cluster_1	SP8	10759	8694.978	8469	8905	133.3095	1.2374	0.3073	0.001	0.001	13715	183722	69.394	964	12652	4.7788	815	10759	4.0638	5.9424	5.8561	84.5436	85.0379
DP	KLF14_1	cluster_2	DP	80803	65308.226	64636	65992	416.4551	1.2373	0.3071	0.001	0.001	49087	664659	67.227	7473	98037	9.916	6083	80803	8.1728	12.3923	12.1571	81.3997	82.4209
DN	SOX21_2	cluster_5	Common_1_DN	15228	12309.9	12076	12523	138.1226	1.237	0.3069	0.001	0.0010909	4666	65357	73.59	1332	17389	19.58	1149	15228	17.146	24.6249	23.2997	86.2613	87.5726
DN	KLF16_1	cluster_3	DN	131728	106507.106	105905	107147	391.9703	1.2368	0.3066	0.001	0.001	87857	980098	74.479	13662	146104	11.103	12254	131728	10.01	13.9477	13.4403	89.694	90.1604
DN	FLI1_2	cluster_3	DN	15143	12244.932	11990	12455	143.8306	1.2367	0.3064	0.001	0.001	8020	104443	74.293	1315	16623	11.824	1185	15143	10.772	14.7756	14.4988	90.1141	91.0967
DN	SOX4_2	cluster_5	Common_1_DN	20974	16960.88	16685	17229	170.7523	1.2366	0.3064	0.001	0.0010909	5733	86204	75.057	1660	23480	20.444	1467	20974	18.262	25.5887	24.3307	88.3735	89.3271
SP8	SP4_2	cluster_1	SP8	77515	62680.729	62013	63330	389.1527	1.2367	0.3064	0.001	0.001	59003	991063	69.566	5789	92205	6.4721	4802	77515	5.441	8.1386	7.8214	82.9504	84.0681
DN	FOXO1_4	cluster_5	Common_1_DN	8283	6700.3	6507	6883	109.0183	1.2362	0.3059	0.001	0.0010909	2797	36450	73.199	766	9486	19.05	663	8283	16.634	23.704	22.7243	86.5535	87.3182
DN	PATZ1_1	cluster_3	DN	162025	131091.519	130318	131877	457.0958	1.236	0.3056	0.001	0.001	85759	1123464	76.273	14139	176126	11.957	12968	162025	11	15.1214	14.4219	91.7179	91.9938
DN	FOXO3_4	cluster_5	Common_1_DN	7049	5706.205	5550	5860	97.1645	1.2353	0.3048	0.001	0.0010909	2345	30568	73.157	658	8084	19.347	568	7049	16.87	24.2217	23.0601	86.3222	87.1969
SP8	EGR1_known9	cluster_6	Common_2	256553	207808.856	207060	208581	460.8857	1.2346	0.304	0.001	0.001	29509	400130	74.089	21069	282472	52.303	19074	256553	47.504	64.6379	64.1174	90.5311	90.8242
DN	SPDEF_4	cluster_5	Common_1_DN	31890	25840.212	25495	26165	207.9582	1.2341	0.3035	0.001	0.0010435	8815	132735	73.627	2551	36276	20.122	2219	31890	17.689	25.173	24.0253	86.9855	87.9094
DN	TCF12_known1	cluster_3	DN	86974	70487.489	70071	70894	236.0838	1.2339	0.3032	0.001	0.0010909	48943	352576	75.728	13030	93370	20.055	12157	86974	18.681	24.8391	24.6682	93.3001	93.1498
SP8	EGR1_known12	cluster_1	SP8	74448	60350.889	59685	60983	409.4104	1.2336	0.3029	0.001	0.001	52057	864606	69.047	5616	89364	7.1366	4601	74448	5.9454	8.8384	8.6106	81.9266	83.3087
DN	TCF4_1	cluster_3	DN	156283	126711.405	126132	127298	337.9468	1.2334	0.3026	0.001	0.0010909	54166	490170	74.751	18932	169953	25.918	17343	156283	23.833	32.0182	31.8834	91.6068	91.9566
DN	EGR1_known12	cluster_3	DN	120825	98007.315	97281	98748	429.668	1.2328	0.302	0.001	0.001	57885	958377	76.536	8501	131962	10.538	7718	120825	9.6491	13.3333	12.6073	90.7893	91.5604
DN	KLF4_1	cluster_3	DN	118899	96475.234	95983	97023	321.0561	1.2324	0.3015	0.001	0.001	90604	870001	74.9	14112	130789	11.26	12790	118899	10.236	14.1164	13.6665	90.6321	90.909
DP	EGR1_known9	cluster_6	Common_2	253400	205630.934	204872	206416	466.7838	1.2323	0.3014	0.001	0.001	29172	396004	73.325	21069	282472	52.303	18834	253400	46.92	64.5619	63.9893	89.392	89.708
DN	FOXO4_2	cluster_5	Common_1_DN	20593	16713.056	16438	16993	167.3403	1.2321	0.3012	0.001	0.0010435	7348	96298	75.255	1895	23131	18.076	1664	20593	16.093	22.6456	21.3847	87.81	89.0277
DN	ETV1_1	cluster_3	DN	4898	3976.646	3762	4162	127.3147	1.2316	0.3006	0.001	0.0010909	5090	46038	26.319	1706	15225	8.7037	548	4898	2.8001	10.7662	10.639	32.1219	32.1708
DN	POU2F2_known14	cluster_3	DN	4111	3337.516	3229	3453	67.8282	1.2317	0.3006	0.001	0.0012632	1238	18993	84.41	326	4330	19.244	309	4111	18.27	24.9596	21.6448	94.7853	94.9423
DP	ETV4_2	cluster_6	Common_2	27310	22174.153	21829	22538	213.3044	1.2316	0.3005	0.001	0.0011429	5095	46084	26.755	9121	82630	47.972	3016	27310	15.855	59.1953	59.2613	33.0665	33.051

DP	HIF1A_2	cluster_2	DP	23876	19388.8	19015	19717	204.4407	1.2314	0.3003	0.001	0.001	13829	185302	69.991	2138	27848	10.519	1817	23876	9.0183	13.1391	12.8849	84.986	85.7369
DN	TCF4_2	cluster_3	DN	115765	94023.669	93560	94541	302.1775	1.2312	0.3001	0.001	0.001	45335	409696	76.215	13902	124083	23.083	12916	115765	21.536	28.4901	28.2563	92.9075	93.2964
DN	POU2F2_known11	cluster_5	Common_1_DN	13118	10658.667	10494	10864	112.3924	1.2307	0.2995	0.001	0.0010435	5502	55147	73.881	1531	14781	19.802	1346	13118	17.574	24.4638	23.7873	87.9164	88.7491
DN	SOX10_9	cluster_5	Common_1_DN	13096	10640.67	10373	10878	144.6057	1.2307	0.2995	0.001	0.0010435	4222	59134	70.343	1186	15642	18.607	974	13096	15.578	23.0696	22.1463	82.1248	83.7233
SP4	HIF1A_1	cluster_4	SP4	8839	7184.06	6985	7400	128.6912	1.2303	0.299	0.001	0.0010435	11555	130065	67.885	980	10689	5.5789	797	8839	4.6133	6.8974	6.7958	81.3265	82.6925
DN	RUNX_2	cluster_3	DN	120562	98006.896	97349	98635	383.6748	1.2301	0.2988	0.001	0.0010435	28180	397366	76.459	9259	129043	24.83	8615	120562	23.198	30.5713	30.3403	93.0446	93.4278
DN	ATF6_1	cluster_3	DN	5048	4103.862	3995	4207	62.6664	1.23	0.2987	0.001	0.0010435	4516	31617	73.316	810	5616	13.023	726	5048	11.706	16.0762	15.9661	89.6296	89.886
DN	MYBL2_4	cluster_3	DN	5017	4078.672	3958	4198	75.2879	1.23	0.2987	0.001	0.0010435	1996	27994	79.043	389	5253	14.832	369	5017	14.166	18.487	17.9217	94.8586	95.5073
SP4	ETS_known13	cluster_4	SP4	12878	10475.22	10184	10763	172.2648	1.2294	0.2979	0.001	0.001	10234	164235	67.178	1019	15787	6.4574	820	12878	5.2675	8.0125	7.8412	80.4711	81.5734
DN	ID4_1	cluster_3	DN	135445	110217.43	109640	110829	334.478	1.2289	0.2974	0.001	0.0010435	48845	442152	75.691	16281	146125	25.015	15027	135445	23.187	30.7647	30.6331	92.2978	92.6912
DN	KLF7_1	cluster_3	DN	166116	135176.049	134380	136021	503.4679	1.2289	0.2973	0.001	0.001	81419	1312000	74.111	12356	187429	10.587	10875	166116	9.3834	13.3568	12.6613	88.0139	88.6288
SP4	SP1_known2	cluster_4	SP4	90286	73505.197	72927	74109	388.4038	1.2283	0.2967	0.001	0.001	112736	1565680	69.085	8420	109754	4.8428	6843	90286	3.9838	6.0699	5.7666	81.2708	82.2621
DN	RUNX_1	cluster_3	DN	153443	124929.589	124273	125600	378.0052	1.2282	0.2966	0.001	0.0010435	34752	490327	76.602	11766	164110	25.638	10949	153443	23.972	31.5061	31.294	93.0563	93.5001
DN	HES5_1	cluster_3	DN	26155	21302.812	21030	21576	164.111	1.2278	0.296	0.001	0.0010435	12165	134258	77.001	2570	27859	15.978	2396	26155	15.001	19.6958	19.4811	93.2296	93.8835
DN	SOX_1	cluster_5	Common_1_DN	24062	19599.531	19258	19928	198.1788	1.2277	0.2959	0.001	0.0010435	8206	98530	71.552	2422	27958	20.303	2057	24062	17.474	25.067	24.421	84.9298	86.0648
DN	FOXO4_3	cluster_5	Common_1_DN	7871	6411.81	6234	6568	97.8183	1.2275	0.2958	0.001	0.0010909	2636	34308	73.038	731	9058	19.283	630	7871	16.756	23.8998	22.9422	86.1833	86.8956
DN	POU2F3_1	cluster_5	Common_1_DN	16322	13296.103	13013	13561	156.4306	1.2276	0.2958	0.001	0.0010435	4595	69456	74.624	1307	18511	19.888	1132	16322	17.537	24.6355	23.4998	86.6106	88.1746
DN	RBPJ_1	cluster_3	DN	29379	23933.685	23731	24123	127.031	1.2275	0.2957	0.001	0.0010435	16019	112173	78.007	4517	31035	21.582	4261	29379	20.431	26.5997	26.1908	94.3325	94.6641
SP4	HIF1A_2	cluster_4	SP4	11378	9270.447	9038	9522	149.5685	1.2273	0.2955	0.001	0.001	13692	183403	69.274	1053	13560	5.1218	876	11378	4.2976	6.3979	6.2038	83.1909	83.9086
SP8	KLF7_1	cluster_1	SP8	101738	82893.98	82054	83794	504.781	1.2273	0.2955	0.001	0.001	75221	1217400	68.768	8057	123464	6.9741	6563	101738	5.7469	8.725	8.357	81.4571	82.403
DP	EGR3_2	cluster_2	DP	91750	74794.737	74089	75450	414.5003	1.2267	0.2948	0.001	0.001	45781	699461	71.099	7154	106180	10.793	6096	91750	9.3262	13.3156	13.1172	85.2111	86.4099
DP	ETV1_1	cluster_6	Common_2	27833	22710.249	22349	23113	232.9696	1.2256	0.2934	0.001	0.0010909	5186	46934	26.831	9308	84323	48.205	3072	27833	15.911	59.2364	59.3024	33.0039	33.0076
DN	SOX21_3	cluster_5	Common_1_DN	15207	12411.97	12139	12664	153.3954	1.2252	0.293	0.001	0.0010909	4326	64953	73.757	1244	17494	19.865	1069	15207	17.268	24.711	23.4123	85.9325	86.9269
DN	HBP1_1	cluster_5	Common_1_DN	18994	15507.55	15211	15792	177.3965	1.2248	0.2926	0.001	0.0010435	5153	79302	73.662	1516	21821	20.269	1306	18994	17.643	25.3445	23.9515	86.1478	87.0446
SP4	SP4_1	cluster_4	SP4	86726	70809.848	69969	71546	473.1507	1.2248	0.2925	0.001	0.001	80215	1441564	69.533	6296	105261	5.0772	5094	86726	4.1832	6.3504	6.0161	80.9085	82.3914
DN	GATA_known16	cluster_5	Common_1_DN	31419	25653.901	25209	26075	262.2317	1.2247	0.2924	0.001	0.0010435	6076	128052	74.197	1823	35888	20.795	1564	31419	18.205	25.7406	24.5361	85.7926	87.5474
DN	MESP1_1	cluster_3	DN	112222	91643.823	91242	92080	258.517	1.2245	0.2922	0.001	0.001	44484	401632	76.45	13534	120595	22.955	12536	112222	21.361	28.1809	27.9415	92.626	93.0569
SP4	EGR4_1	cluster_4	SP4	28733	23476.774	23122	23883	221.8282	1.2239	0.2915	0.001	0.001	29753	337110	66.76	3257	35643	7.0586	2596	28733	5.6901	8.7252	8.5233	79.7053	80.6133
DN	SOX17_5	cluster_5	Common_1_DN	9358	7649.084	7458	7821	115.2982	1.2234	0.2909	0.001	0.0011429	2792	39191	71.088	850	11148	20.221	705	9358	16.974	25.2507	23.8779	82.9412	83.9433
DN	SOX9_4	cluster_5	Common_1_DN	23132	18909.38	18586	19208	184.8021	1.2233	0.2908	0.001	0.0010909	6382	95858	74.138	1850	26308	20.347	1614	23132	17.891	25.2899	24.1315	87.2432	87.9276
DP	EGR3_3	cluster_2	DP	60090	49124.699	48585	49704	336.8492	1.2232	0.2907	0.001	0.001	31417	463984	71.003	4864	69807	10.683	4090	60090	9.1955	13.1903	12.9509	85.1974	86.0802
DN	FOXP3_2	cluster_5	Common_1_DN	7378	6034.87	5930	6134	63.1563	1.2225	0.2899	0.001	0.0010909	5499	32994	72.569	1426	8406	18.489	1246	7378	16.228	22.6587	22.3616	87.3773	87.7706
DN	IKZF1_2	cluster_3	DN	44151	36115.352	35866	36376	157.7388	1.2225	0.2898	0.001	0.0010435	27213	217711	79.439	5902	46144	16.837	5633	44151	16.11	20.6997	20.2796	95.4422	95.6809
SP8	SP100_1	cluster_6	Common_2	6990	5721.025	5595	5839	73.5397	1.2218	0.289	0.001	0.0013333	956	12440	73.99	620	7821	46.518	546	6990	41.575	57.113	56.1897	88.0645	89.3748
SP4	ETV5_1	cluster_6	Common_2	71477	58511.593	58208	58835	184.0282	1.2216	0.2888	0.001	0.0010435	11580	105410	73.515	8751	79505	55.448	7836	71477	49.849	67.6684	67.8086	89.5441	89.9025
DP	ATF2_1	cluster_2	DP	784	641.682	598	692	28.7246	1.2214	0.2886	0.001	0.0010435	1296	9072	66.75	137	959	7.0561	112	784	5.7685	8.642	8.642	81.7518	81.7518
DN	SOX8_2	cluster_5	Common_1_DN	22475	18402.42	18095	18691	173.1491	1.2213	0.2884	0.001	0.0010909	6301	94659	74.032	1805	25656	20.065	1571	22475	17.577	24.9326	23.7431	87.036	87.6013
DN	SPZ1_1	cluster_3	DN	127182	104174.611	103559	104840	396.0638	1.2209	0.2879	0.001	0.001	58732	857887	77.786	9814	136683	12.393	9076	127182	11.532	15.4532	14.825	92.4801	93.0489
DN	ERG_4	cluster_3	DN	16199	13269.306	13020	13513	148.0284	1.2208	0.2878	0.001	0.001	8373	109062	76.033	1392	17593	12.265	1271	16199	11.293	15.1797	14.853	91.3075	92.0764
DN	KLF12_2	cluster_3	DN	94200	77178.437	76591	77723	347.9672	1.2205	0.2875	0.001	0.001	46999	672450	77.85	7345	100882	11.679	6818	94200	10.905	14.5067	14.0085	92.8251	93.3764
DN	CBX5_1	cluster_5	Common_1_DN	13411	10990.655	10803	11191	119.3042	1.2202	0.2871	0.001	0.0010435	5190	57165	75.199	1439	15113	19.881	1272	13411	17.642	24.5087	23.4602	88.3947	88.7382
DN	SP1_known3	cluster_3	DN	96007	78680.896	78150	79230	331.8559	1.2202	0.2871	0.001	0.001	47992	629173	78.933	8009	101310	12.71	7546	96007	12.045	15.7235	15.2592	94.219	94.7656
DP	SP4_1	cluster_2	DP	186313	152721.583	151573	153806	684.5286	1.22	0.2868	0.001	0.001	81673	1469721	70.891	12677	218773	10.552	10647	186313	8.9867	13.0361	12.6768	83.9867	85.1627
DP	EGR1_known9	cluster_2	DP	50814	41655.09	41164	42179	293.5091	1.2199	0.2867	0.001	0.001	29172	396004	73.325	4300	57195	10.59	3786	50814	9.4089	12.9782	12.8317	88.0465	88.8434
DN	MYBL2_3	cluster_3	DN	7726	6334.928	6173	6540	106.6722	1.2196	0.2864	0.001	0.0010435	2843	43039	79.915	562	8129	15.094	531	7726	14.346	18.6775	17.9512	94.484	95.0424
DN	PBX1_2	cluster_5	Common_1_DN	12085	9910.768	9708	10136	126.9147	1.2194	0.2861	0.001	0.0010435	3613	51272	73.03	1057	14017	19.965	891	12085	17.213	24.6609	23.5704	84.2952	86.2167
SP4	EGR4_2	cluster_4	SP4	53345	43751.237	43210	44312	333.5956	1.2193	0.286	0.001	0.001	48413	794683	69.102	4162	64752	5.6306	3368	53345	4.6387	6.9568	6.7127	80.9226	82.3836
DN	IKZF3_1	cluster_3	DN	22064	18096.772	17854	18344	146.8104	1.2192	0.2859	0.001	0.0010435	8816	106058	78.616	2031	23575	17.475	1890	22064	16.355	21.4383	20.8037	93.0576	93.5907
SP8	SP1_known2	cluster_1	SP8	105888	86858.333	86159	87608	437.8501	1.2191	0.2858	0.001	0.001	116483	1617881	71.388	9541	124977	5.5146	8025	105888	4.6723	6.8894	6.5449	84.1107	84.726
DN	EGR4_1	cluster_3	DN	48553	39835.649	39449	40211	227.4653	1.2188	0.2855	0.001	0.001	34373	389734	77.181	4776	52354	10.368	4409	48553	9.6152	12.8269	12.458	92.3157	92.7398
DN	NFATC2_1	cluster_3	DN	9372	7689.645	7578	7804	68.106	1.2188	0.2854	0.001	0.0010909	7464	44784	80.238	1665	9740	17.451	1600	9372	16.791	21.4362	20.9271	96.0961	96.2218
DP	SP1_known2	cluster_2	DP	207954	170780.257	169747	171813	617.1516	1.2177	0.2841	0.001	0.001	115319	1604370	70.792	17987	243799	10.758	15211	207954	9.1759	13.1904	12.9617	84.5666	85.2973
DN	MYCN_1	cluster_3	DN	21972	18047.518	17813	18303	152.4879	1.2174	0.2839	0.001	0.001	12503	137921	76.685	2200	23706	13.181	2023	21972	12.217	16.1801	15.9309	91.9545	92.6854
DN	E2F2_3	cluster_3	DN	6567	5395.022	5241	5553	99.6126	1.2172	0.2836	0.001	0.001	2541	43236	81.281	432	6875	12.925	410	6567	12.346	16.3854	15.1887	94.9074	95.52
DN	POU2F2_known12	cluster_5	Common_1_DN	12969	10654.697	10454	10809	108.8855	1.2172	0.2836	0.001	0.0010435	5446	54541	74.724	1513	14607	20.012	1332	12969	17.768	24.4583	23.7784	88.037	88.7862
DN	SOX10_4	cluster_5	Common_1_DN	24519	20146.43	19851	20449	184.2357	1.217	0.2834	0.001	0.0010909	6860	103161	73.858	1975	28093	20.113	1707	24519	17.554	24.8834	23.7677	86.4304	87.278
DP	ELK4_2	cluster_2	DP	10820	8890.645	8557	9170	182.1672	1.217	0.2833	0.001	0.0011429	7680	69469	25.728	3873	34667	12.839	1204	10820	4.0072	15.6771	15.5753	31.087	31.2112
DN	KLF13_1	cluster_3	DN	59419	48852.844	48383	49359	316.7889	1.2163	0.2825	0.001	0.001	24149	415074	75.178	4015	66134	11.978	3566	59419	10.762	14.7667	14.3153	88.8169	89.8464
DN	FOXP3_1	cluster_5	Common_1_DN	41268	33941.748	33561	34358	244.7502	1.2158	0.282	0.001	0.0010909	9966	159936	72.213	3180	48216	21.77	2666	41268	18.633	26.751	25.8028	83.8365	85.5898
DN	HMGA1_2	cluster_5	Common_1_DN	22173	18238.438	17949	18535	177.1959	1.2157	0.2818	0.001	0.0010435	6789	95209	77.457	1898	24720	20.111	1678	22173	18.039	24.7165	23.2888	88.4089	89.6966
SP8	EGR4_2	cluster_1	SP8	60561	49823.021	49223	50475	390.6171	1.2155	0.2816	0.001	0.001	50126	822981	71.563	4521	71084	6.1812	3792	60561	5.2661	7.5649	7.3587	83.8752	85.1964
DN	TCF21_1	cluster_3	DN	66439	54666.165	54220	55101	259.3469	1.2154	0.2814	0.001	0.001	16797	218881	77.73	5540	70941	25.193	5160	66439	23.594	30.7198	30.3539	93.1408	93.6539
DN	ARID5A_1	cluster_5	Common_1_DN	2942	2422.307	2330	2528	59.4805	1.2145	0.2803	0.001	0.0012632	992	13600	80.227	253	3206	18.912	227	2942	17.355	22.8831	21.6324	89.7233	91.7654
DN	TBX21_4	cluster_5	Common_1_DN	38438	31649.896	31243	32036	238.1051	1.2145	0.2803	0.001	0.0010909	9894	148765	73.27	3068	44178	21.759	2632	38438	18.932	26.602	25.8381	85.7888	87.0071
SP8	ETV5_1	cluster_6	Common_2	25689	21176.714	20829	21511	202.2506	1.2131	0.2787	0.001	0.0010435	4199	38058	26.542	8751	79505	55.448	2835	25689	17.916	67.5161	67.4996	32.3963	32.3112
DN	ETV6_1	cluster_3	DN	74046	61057.301	60554	61532	287.3667	1.2127	0.2783	0.001	0.001	32656	469723	77.86	5707	79282	13.142	5281	74046	12.274	16.1716	15.7638	92.5355	93.3957
DN	MTF1_3	cluster_3	DN	39129	32268.281	31917	32604	206.4536	1.2126	0.2781	0.001	0.001	16298	213937	79.022	3233	41341	15.27	3052	39129	14.453	18.7262	18.29	94.4015	94.6494
DN	TCF3_2	cluster_3	DN	185242	152879.99	152019	153607	511.0086	1.2117	0.277	0.001	0.0010435	39118	592154	77.209	13375	199427	26.003	12359	185242	24.153	31.5942	31.2827	92.4037	92.8871
DN	HES7_1	cluster_3	DN	25808	21301.839	21059	21566	148.5657	1.2115	0.2768	0.001	0.0010435	12542	138583	78.225	2533	27443	15.491	2363	25808	14.568	18.8407	18.6228	93.2886	94.0422
DN	POU2F2_known10	cluster_5	Common_1_DN	13068	10786.53	10615	10967	106.5549	1.2115	0.2768	0.001	0.0010435	6013	54229	74.503	1684	14748	20.262	1478	13068	17.954	24.5801	24.0978	87.7672	88.6086
DP	AHR::ARNT::HIF1A_1	cluster_2	DP	12663	10476.576	10272	10668	117.3206	1.2087	0.2734	0.001	0.0010435	12707	104472	71.635	1807	14673	10.061	1555	12663	8.6828	12.2373	12.121	86.0542	86.3014
DN	PBX1_4	cluster_5	Common_1_DN	9334	7723.581	7558	7880	98.1921	1.2085	0.2732	0.001	0.0010909	3459	38700	71.853	1046	11037	20.492	866	9334	17.33	25.0361	24.1189	82.7916	84.5701
DN	FLI1_4	cluster_3	DN	15821	13101.529	12878	13313	136.1998	1.2076	0.2721	0.001	0.001	8592	111995	76.37	1366	17315	11.807	1235	15821	10.788	14.3738	14.1265	90.41	91.3716
SP4	CUX2_1	cluster_6	Common_2	2942	2437.347	2324	2561	68.2305	1.207	0.2714	0.001	0.0012632	439	7949	74.631	241	3664	34.401	181	2942	27.622	41.2301	37.0109	75.1037	80.2948
SP8	SP4_1	cluster_1	SP8	97765	81052.835	80310	81895	491.7495	1.2062	0.2705	0.001	0.001	82983	1490454	71.892	6913	116187	5.6042	5732	97765	4.7157	6.9074	6.5594	82.9162	84.1445
SP4	GMEB2_3	cluster_4	SP4	2072	1718.269	1618	1815	61.375	1.2057	0.2699	0.001	0.0010435	2133	30178	72.468	180	2424	5.8209	149	2072	4.9756	6.9855	6.8659	82.7778	85.4785
DN	MYBL1_5	cluster_3	DN	10411	8635.645	8461	8817	109.1342	1.2056	0.2697	0.001	0.0010435	3552	57284	82.002	683	10712	15.334	660	10411	14.903	18.5811	18.1744	96.6325	97.1901
DN	GATA_known17	cluster_5	Common_1_DN	20191	16757.739	16475	17036	171.387	1.2049	0.2689	0.001	0.0010909	5009	80296	75.449	1511	22926	21.542	1312	20191	18.972	26.1929	25.1457	86.8299	88.0703
SP4	GMEB1_1	cluster_4	SP4	3897	3234.819	3091	3433	98.5617	1.2046	0.2686	0.001	0.0010435	5959	97141	70.771	294	4624	3.3688	245	3897	2.8391	4.1114	4.0117	83.3333	84.2777
DP	ETV3_1	cluster_2	DP	19108	15868.807	15618	16140	154.7063	1.2041	0.268	0.001	0.0010435	13947	126127	66.782	2666	23782	12.592	2129	19108	10.117	15.2649	15.1498	79.8575	80.3465
DN	HESX1_1	cluster_3	DN	3759	3123.653	3038	3199	46.8036	1.2034	0.267	0.001	0.0012632	1814	16345	82.401	454	3905	19.686	435	3759	18.95	23.9802	22.9979	95.815	96.2612
DN	GMEB2_2	cluster_3	DN	6755	5614.888	5478	5756	86.5139	1.203	0.2667	0.001	0.001	3215	42707	77.048	576	7389	13.331	525	6755	12.187	16.3297	15.8171	91.1458	91.4197
DN	ATF2_2	cluster_3	DN	10016	8327.656	8144	8499	105.5897	1.2027	0.2663	0.001	0.0010435	5805	63978	74.542	1035	11230	13.084	917	10016	11.67	15.7967	15.6554	88.599	89.1897
SP8	AHR::ARNT::HIF1A_1	cluster_1	SP8	5288	4397.932	4267	4526	80.5431	1.2023	0.2658	0.001	0.0010435	12611	103685	71.095	761	6233	4.2739	644	5288	3.6259	5.1067	5.1001	84.6255	84.8388
DN	ARID3A_2	cluster_5	Common_1_DN	6203	5166.034	5020	5313	88.7799	1.2007	0.2639	0.001	0.0013333	1580	25566	81.304	461	6775	21.546	414	6203	19.727	26.2025	24.2627	89.8048	91.5572
DN	POU2F2_known5	cluster_5	Common_1_DN	15051	12541.119	12298	12788	142.944	1.2001	0.2629	0.001	0.0010909	4660	60686	74.223	1410	17442	21.333	1204	15051	18.408	25.8369	24.8014	85.3901	86.2917
SP4	TCF3_2	cluster_1	SP8	17259	14381.765	13996	14785	240.7467	1.2	0.2631	0.001	0.0010435	23468	354325	46.199	2188	31655	4.1274	1157	17259	2.2503	4.9301	4.871	52.8793	54.5222
DN	EGR4_2	cluster_3	DN	104718	87332.308	86700	87986	395.103	1.1991	0.2619	0.001	0.001	55529	908524	79.001	7390	113656	9.883	6766	104718	9.1058	12.1846	11.5262	91.5562	92.1359
DN	SP4_2	cluster_3	DN	141888	118352.885	117597	119085	437.8529	1.1989	0.2617	0.001	0.001	66957	1120770	78.67	9762	154277	10.829	8928	141888	9.9595	13.3339	12.6599	91.4567	91.9696
SP8	EGR3_2	cluster_1	SP8	47826	39909.916	39442	40389	288.9824	1.1983	0.261	0.001	0.001	46634	711154	72.287	3895	56407	5.7337	3246	47826	4.8614	6.9606	6.7251	83.3376	84.7873
SP8	CUX2_1	cluster_6	Common_2	2924	2440.803	2315	2564	73.4992	1.1979	0.2605	0.001	0.0012632	434	7992	75.035	241	3664	34.401	182	2924	27.453	41.9355	36.5866	75.5187	79.8035
DN	POU2F2_known15	cluster_5	Common_1_DN	9709	8105.44	7922	8282	110.5206	1.1978	0.2604	0.001	0.0010435	3911	43212	75.283	1058	11078	19.286	916	9709	16.903	23.4211	22.4683	86.5784	87.6422
DP	ELK3_2	cluster_2	DP	8477	7076.889	6825	7328	164.2939	1.1978	0.2604	0.001	0.0011429	6179	55790	26.533	2993	26781	12.737	945	8477	4.0316	15.2937	15.1945	31.5737	31.653
DN	IRF6_1	cluster_3	DN	8961	7484.313	7324	7667	102.9815	1.1973	0.2598	0.001	0.0010909	3319	53207	81.206	609	9420	14.377	576	8961	13.677	17.3546	16.8418	94.5813	95.1274
DN	TBX2_2	cluster_5	Common_1_DN	30023	25077.51	24773	25363	173.682	1.1972	0.2597	0.001	0.0010909	11786	118013	74.082	3547	34386	21.586	3070	30023	18.847	26.0479	25.4404	86.552	87.3117
SP8	ELK4_1	cluster_6	Common_2	56327	47054.95	46813	47305	153.9286	1.197	0.2595	0.001	0.001	10505	84617	76.469	7657	61524	55.599	6994	56327	50.903	66.5778	66.567	91.3413	91.5529
DN	GMEB1_1	cluster_3	DN	3535	2953.381	2718	3178	145.8644	1.1969	0.2593	0.001	0.0010435	858	13450	9.7989	231	3535	2.5754	11.9627	11.4084	26.9231	26.2825			
DN	HES1_1	cluster_3	DN	71028	59347.722	58845	59871	302.277	1.1968	0.2592	0.001	0.001	30958	438366	78.548	5551	76425	13.694	5124	71028	12.727	16.5515	16.2029	92.3077	92.9382
SP4	EGR3_2	cluster_4	SP4	43342	36215.492	35739	36776	307.4112	1.1968	0.2592	0.001	0.001	45033	687127	69.845	3669	53073	5.3948	2941	43342	4.4056	6.5308	6.3077	80.1581	81.6649
SP4	ID4_1	cluster_1	SP8	14138	11814.081	11529	12090	172.1989	1.1967	0.2591	0.001	0.0010435	30226	273311	46.788	2880	25444	4.3557	1577	14138	2.4203	5.2174	5.1729	54.7569	55.5652
DN	E2F7_1	cluster_3	DN	13441	11233.521	11041	11430	114.2526	1.1965	0.2588	0.001	0.0010435	5640	73401	81.261	1136	14187	15.706	1071	13441	14.88	18.9894	18.3117	94.2782	94.7417
DP	ELK3_1	cluster_2	DP	8728	7294.683	7050	7571	156.8157	1.1965	0.2588	0.001	0.0011429	6415	57946	26.824	3057	27373	12.671	972	8728	4.0403	15.152	15.0623	31.7959	31.8854
DN	TBX5_5	cluster_5	Common_1_DN	37079	31010.896	30551	31473	260.9751	1.1957	0.2578	0.001	0.0010435	7574	144355	74.742	2353	42538	22.025	2008	37079	19.198	26.5118	25.686	85.3379	87.1668
DN	MGA_2	cluster_5	Common_1_DN	29117	24373.449	24025	24731	215.9546	1.1946	0.2565	0.001	0.0010909	6706	114146	75.028	2036	33173	21.805	1759	29117	19.139	26.2302	25.5086	86.3949	87.7732
DP	GMEB1_1	cluster_2	DP	11042	9243.374	8968	9513	163.1059	1.1946	0.2565	0.001	0.0010435	5680	92646	67.496	862	13770	10.032	683	11042	8.0445	12.0246	11.9185	79.2343	80.1888
DN	POU2F3_2	cluster_5	Common_1_DN	8027	6722.739	6590	6859	78.1232	1.194	0.2558	0.001	0.0010909	4271	34232	74.798	1185	9162	20.019	1030	8027	17.539	24.1161	23.4488	86.9198	87.6119
SP4	ELK4_1	cluster_6	Common_2	56198	47080.625	46838	47302	140.4245	1.1937	0.2554	0.001	0.001	10509	84669	76.516	7657	61524	55.599	6978	56198	50.786	66.4002	66.3738	91.1323	91.3432
SP4	AHR::ARNT::HIF1A_1	cluster_4	SP4	5592	4686.593	4557	4816	76.8405	1.1931	0.2548	0.001	0.0010435	12561	103445	70.93	821	6656	4.5639	686	5592	3.8343	5.4613	5.4058	83.5566	84.0144
DN	TBX20_5	cluster_5	Common_1_DN	63319	53078.725	52552	53544	294.8689	1.1929	0.2545	0.001	0.0010909	16570	253871	75.218	5012	72644	21.523	4316	63319	18.76	26.0471	24.9414	86.1133	87.1634
DN	SP1_known2	cluster_3	DN	208689	174947.82	174051	175813	508.1921	1.1929	0.2544	0.001	0.001	131181	1808239	79.788	17436	225537	9.9517	16144	208689	9.2083	12.3067	11.541	92.59	92.5298
DN	HMGA1_1	cluster_5	Common_1_DN	6362	5333.801	5230	5428	58.1378	1.1927	0.2543	0.001	0.0010435	4316	26040	76.796	1205	7071	20.853	1080	6362	18.763	25.0232	24.4316	89.6266	89.9731
DN	SP4_1	cluster_3	DN	184171	154496.827	153528	155480	571.3292	1.1921	0.2535	0.001	0.001	90889	1624561	78.36	12286	203749	9.8278	11038	184171	8.8834	12.1445	11.3367	89.8421	90.3911
DN	POU2F2_known1	cluster_5	Common_1_DN	17039	14302.525	14008	14567	178.5755	1.1913	0.2526	0.001	0.0010435	4025	72871	75.443	1186	19829	20.529	996	17039	17.64	24.7453	23.3824	83.9798	85.9297
SP4	SP100_1	cluster_6	Common_2	6834	5736.781	5620	5858	73.3928	1.1912	0.2524	0.001	0.0013333	962	12518	74.454	620	7821	46.518	534	6834	40.647	55.5094	54.5934	86.129	87.3801
SP4	KLF14_1	cluster_4	SP4	43496	36521.889	35947	37025	317.4409	1.191	0.2521	0.001	0.001	49055	664031	67.164	4312	55543	5.6179	3320	43496	4.3994	6.7679	6.5503	76.9944	78.3105
DN	IRF4_1	cluster_3	DN	9248	7771.474	7616	7923	94.2013	1.19	0.2509	0.001	0.0010909	3899	54808	80.813	712	9749	14.375	673	9248	13.636	17.2608	16.8734	94.5225	94.861
DN	SMAD3_3	cluster_5	Common_1_DN	11143	9365.002	9202	9520	99.2522	1.1898	0.2508	0.001	0.0010435	4812	43323	75.24	1443	12645	21.961	1265	11143	19.352	26.2884	25.7207	87.6646	88.1218
DN	NFE2L1::MAFG_1	cluster_5	Common_1_DN	63578	53446.177	52845	54016	352.9262	1.1896	0.2504	0.001	0.0010435	11526	243206	74.83	3722	73540	22.627	3149	63578	19.562	27.3208	26.1416	84.6051	86.4536
SP8	EGR3_3	cluster_1	SP8	32547	27364.974	26917	27794	260.1192	1.1894	0.2502	0.001	0.001	32104	473266	72.424	2726	38422	5.8797	2275	32547	4.9806	7.0863	6.8771	83.4556	84.7093
SP8	EGR1_known9	cluster_1	SP8	26751	22492.699	22176	22830	198.5551	1.1893	0.2501	0.001	0.001	29509	400130	74.089	2355	30796	5.7023	2015	26751	4.9533	6.8284	6.6856	85.5626	86.8652
DN	TBX19_1	cluster_5	Common_1_DN	22328	18775.622	18413	19088	201.6178	1.1892	0.25	0.001	0.0010435	4779	91001	71.181	1515	27072	21.176	1210	22328	17.465	25.3191	24.536	79.868	82.4764
DN	IKZF1_1	cluster_3	DN	29309	24648.645	24389	24914	158.0804	1.1891	0.2498	0.001	0.0010435	11933	143526	82.189	2665	30767	17.618	2524	29309	16.784	21.1514	20.4207	94.7092	95.2612
DN	TBX21_6	cluster_5	Common_1_DN	47936	40330.88	39843	40780	284.8973	1.1886	0.2492	0.001	0.0010909	10503	189588	74.498	3242	55459	21.793	2744	47936	18.836	26.1259	25.2843	84.6391	86.435
DN	POU2F2_known13	cluster_5	Common_1_DN	14181	11941.045	11653	12233	176.6935	1.1876	0.248	0.001	0.0010909	3197	67338	75.85	863	16530	18.619	719	14181	15.974	22.4898	21.0594	83.314	85.7895
SP4	EGR1_known9	cluster_4	SP4	22961	19335.28	18983	19656	197.9486	1.1875	0.2479	0.001	0.001	28464	386336	71.535	2102	27438	5.0805	1739	22961	4.2515	6.1095	5.9433	82.7307	83.6832

DN	EGR3_3	cluster_3	DN	55769	46966.804	46493	47412	279.5811	1.1874	0.2478	0.001	0.001	35070	516864	79.095	4306	60531	9.263	3957	55769	8.5343	11.2831	10.7899	91.895	92.133
SP8	KLF14_1	cluster_1	SP8	50962	42922.291	42471	43422	287.2942	1.1873	0.2477	0.001	0.001	51058	690823	69.873	4810	62544	6.326	3865	50962	5.1546	7.5698	7.377	80.3534	81.4818
DN	IRF_known21	cluster_5	Common_1_DN	30281	25519.473	25196	25861	198.0038	1.1866	0.2468	0.001	0.001	9344	135063	76.665	2468	34061	19.334	2162	30281	17.188	23.1378	22.4199	87.6013	88.9023
SP4	EGR3_3	cluster_4	SP4	28561	24073.238	23668	24432	235.0575	1.1864	0.2466	0.001	0.001	31018	457668	70.037	2490	34990	5.3545	2005	28561	4.3707	6.464	6.2405	80.5221	81.6262
DN	E2F8_1	cluster_3	DN	19379	16335.756	16127	16527	122.481	1.1863	0.2464	0.001	0.001	9501	104773	81.344	1924	20480	15.9	1812	19379	15.045	19.0717	18.4962	94.1788	94.624
DP	ETV5_1	cluster_6	Common_2	72237	60902.696	60638	61191	171.8058	1.1861	0.2462	0.001	0.0010435	12055	109704	76.51	8751	79505	55.448	7923	72237	50.379	65.7238	65.8472	90.5382	90.8584
DN	IKZF2_3	cluster_5	Common_1_DN	16710	14113.765	13902	14299	120.9859	1.1839	0.2436	0.001	0.0010909	7087	71094	80.484	1899	18181	20.582	1733	16710	18.917	24.4532	23.5041	91.2586	91.9091
DN	TBX1_2	cluster_5	Common_1_DN	29153	24639.579	24272	25046	229.0532	1.1832	0.2427	0.001	0.0010909	6383	115007	73.738	2030	34408	22.061	1690	29153	18.692	26.4766	25.3489	83.2512	84.7274
SP4	CUX2_2	cluster_6	Common_2	1429	1207.818	1152	1271	34.8385	1.183	0.2424	0.001	0.0013333	387	3483	71.8	207	1769	36.467	167	1429	29.458	43.1525	41.0278	80.6763	80.7801
DN	SOX1_3	cluster_5	Common_1_DN	12531	10594.897	10357	10835	144.3517	1.1827	0.2421	0.001	0.0010909	3794	53161	70.646	1186	15495	20.591	941	12531	16.652	24.8023	23.5718	79.3423	80.8712
DN	MAF_known10	cluster_5	Common_1_DN	31709	26814.703	26501	27111	172.5971	1.1825	0.2419	0.001	0.0010909	11107	122400	74.849	3417	36443	22.285	2946	31709	19.39	26.5238	25.906	86.216	87.0099
DN	CUX1_9	cluster_5	Common_1_DN	1895	1602.608	1498	1703	62.8777	1.1823	0.2416	0.001	0.0012632	631	10867	73.396	144	2263	15.284	118	1895	12.799	18.7005	17.4381	81.9444	83.7384
DN	BPTF_1	cluster_5	Common_1_DN	52119	44092.145	43726	44467	240.4908	1.182	0.2413	0.001	0.0010909	15844	211822	75.51	4683	60087	21.42	4028	52119	18.579	25.4229	24.6051	86.0132	86.7392
DN	RUNX2_5	cluster_3	DN	81080	68602.299	68072	69170	336.1558	1.1819	0.2411	0.001	0.001	23276	408017	80.689	5062	86023	17.012	4727	81080	16.034	20.3085	19.8717	93.3821	94.2539
DN	IRF4_2	cluster_5	Common_1_DN	25036	21184.149	20924	21503	176.1281	1.1818	0.241	0.001	0.001	7884	113080	77.321	2042	28028	19.165	1802	25036	17.119	22.8564	22.1401	88.2468	89.325
SP8	ETV5_1	cluster_1	SP8	1988	1682.698	1540	1813	85.2252	1.1813	0.2404	0.001	0.0010435	4199	38058	26.542	716	6355	4.4321	221	1988	1.3865	5.2632	5.2236	30.8659	31.2825
DN	GMEB2_3	cluster_3	DN	4177	3536.097	3421	3646	69.714	1.1812	0.2402	0.001	0.0010435	2360	33394	80.191	322	4464	10.72	299	4177	10.03	12.6695	12.5082	92.8571	93.5708
DN	MYCN_2	cluster_3	DN	29764	25209.486	24942	25464	153.1655	1.1807	0.2396	0.001	0.001	21157	191106	79.065	3625	32109	13.284	3347	29764	12.314	15.8198	15.5746	92.331	92.6968
DN	SOX14_3	cluster_5	Common_1_DN	13203	11186.371	10961	11449	143.4005	1.1803	0.2391	0.001	0.0010909	4092	57347	70.134	1254	16454	20.123	984	13203	16.147	24.0469	23.023	78.4689	80.2419
DN	STAT_known3	cluster_3	DN	27966	23742.366	23374	24084	219.1712	1.1779	0.2362	0.001	0.0010435	8877	177940	82.488	1560	29619	13.731	1463	27966	12.964	16.4808	15.7165	93.7821	94.4191
SP4	TCF4_1	cluster_1	SP8	13901	11805.037	11493	12125	190.1041	1.1775	0.2358	0.001	0.0010909	33174	299877	45.732	2953	26049	3.9725	1556	13901	2.1199	4.6904	4.6356	52.6922	53.3648
DN	ATF4_3	cluster_5	Common_1_DN	15127	12848.081	12628	13074	136.1796	1.1774	0.2356	0.001	0.0010909	4863	63288	72.723	1433	17948	20.624	1189	15127	17.382	24.4499	23.9018	82.9728	84.2824
DN	RUNX3_3	cluster_3	DN	94834	80545.696	79977	81093	336.0038	1.1774	0.2356	0.001	0.001	29706	527830	81.113	5889	100815	15.493	5487	94834	14.573	18.471	17.9668	93.1737	94.0674
DN	TBX15_1	cluster_5	Common_1_DN	35447	30110.212	29736	30554	243.7473	1.1772	0.2354	0.001	0.0010909	7884	142057	75.135	2443	41328	21.859	2057	35447	18.748	26.0908	24.9527	84.1998	85.7699
DN	TBX4_1	cluster_5	Common_1_DN	29622	25161.574	24924	25401	140.7921	1.1773	0.2354	0.001	0.0010435	16022	112186	74.769	4930	33931	22.614	4285	29622	19.742	26.7445	26.4044	86.9168	87.3007
DN	IRF_known14	cluster_5	Common_1_DN	78308	66561.618	65932	67189	367.5296	1.1765	0.2345	0.001	0.001	16394	342215	77.672	4593	89191	20.244	3956	78308	17.773	24.1308	22.8827	86.1311	87.7981
SP8	CTCF_known2	cluster_1	SP8	20320	17274.81	16706	17879	331.4189	1.1763	0.2342	0.001	0.0010435	15803	254255	30.429	3610	56958	6.8167	1271	20320	2.4319	8.0428	7.992	35.2078	35.6754
DN	ATF3_known4	cluster_3	DN	20122	17112.202	16847	17360	158.0448	1.1759	0.2337	0.001	0.001	10011	141182	80.298	1578	21515	12.237	1467	20122	11.445	14.6539	14.2525	92.9658	93.5254
DN	MGA_3	cluster_5	Common_1_DN	27401	23311.93	23006	23683	207.9899	1.1754	0.2332	0.001	0.0010909	6415	109267	77.12	1893	30937	21.835	1657	27401	19.34	25.8301	25.0771	87.533	88.5703
DN	TBX4_2	cluster_5	Common_1_DN	44314	37705.964	37299	38128	254.6268	1.1752	0.233	0.001	0.0010909	8920	170122	76.154	2786	50614	22.657	2390	44314	19.837	26.7937	26.0484	85.7861	87.5529
DN	ARID5B_1	cluster_5	Common_1_DN	18934	16128.113	15899	16340	137.7922	1.174	0.2314	0.001	0.0010909	5946	77508	76.405	1743	21717	21.408	1503	18934	18.664	25.2775	24.4284	86.2306	87.1852
DN	SPIB_1	cluster_5	Common_1_DN	35109	29927.183	29684	30181	148.3041	1.1731	0.2304	0.001	0.0010435	22113	132678	71.137	7127	42285	22.672	5898	35109	18.824	26.6721	26.4618	82.7557	83.0294
DN	HIF1A_1	cluster_3	DN	20610	17576.28	17352	17812	137.5842	1.1726	0.2297	0.001	0.0010435	13599	153110	79.913	2009	22190	11.582	1863	20610	10.757	13.6995	13.4609	92.7327	92.8797
DN	TBX5_3	cluster_5	Common_1_DN	48211	41127.06	40828	41485	204.9063	1.1722	0.2293	0.001	0.001	20740	186864	75.435	6304	55260	22.308	5461	48211	19.462	26.3308	25.8	86.6275	87.2439
SP4	ATF2_1	cluster_4	SP4	866	738.709	695	786	26.6274	1.1721	0.2291	0.001	0.0010435	1377	9639	70.922	151	1053	7.7478	124	866	6.3719	9.0051	8.9843	82.1192	82.2412
DN	FOXM1_1	cluster_5	Common_1_DN	13465	11491.829	11326	11665	106.2206	1.1717	0.2286	0.001	0.0010909	6570	52629	74.145	2009	15722	22.15	1704	13465	18.97	25.9361	25.5848	84.8183	85.6443
DN	IKZF2_2	cluster_5	Common_1_DN	12206	10423.865	10252	10589	103.0076	1.171	0.2277	0.001	0.0010435	4704	47046	78.526	1427	13658	22.797	1262	12206	20.374	26.8282	25.9448	88.4373	89.3689
DP	ELK4_1	cluster_6	Common_2	55911	47771.079	47512	48050	157.9697	1.1704	0.227	0.001	0.001	10661	85897	77.625	7657	61524	55.599	6942	55911	50.527	65.1158	65.0907	90.6621	90.8767
DN	RUNX3_1	cluster_3	DN	88198	75450.572	74939	75906	299.8735	1.1689	0.2252	0.001	0.0010435	25951	398073	82.383	6177	92639	19.172	5856	88198	18.253	22.5656	22.1562	94.8033	95.2061
DN	HIF1A::ARNT_1	cluster_3	DN	13136	11246.143	11123	11368	82.9753	1.168	0.2241	0.001	0.001	12592	90566	80.589	1969	14045	12.498	1835	13136	11.689	14.5727	14.5043	93.1945	93.5279
DN	MTF1_1	cluster_3	DN	101562	87017.358	86509	87524	314.246	1.1671	0.223	0.001	0.001	48686	649383	82.303	8195	106903	13.549	7760	101562	12.872	15.9389	15.6398	94.6919	95.0039
DN	E2F_known26	cluster_5	Common_1_DN	7800	6683.098	6510	6876	111.441	1.1671	0.2229	0.001	0.0010435	2046	34813	80.496	551	8698	20.112	485	7800	18.036	23.7048	22.4054	88.0218	89.6758
DN	ETS_known13	cluster_3	DN	27399	23484.912	23100	23838	206.7365	1.1667	0.2224	0.001	0.001	12306	197388	80.739	1895	29430	12.038	1750	27399	11.207	14.2207	13.8808	92.3483	93.0989
SP8	GMEB1_1	cluster_1	SP8	3915	3356.962	3208	3502	91.9812	1.1662	0.2218	0.001	0.0010435	6106	99511	72.498	293	4645	3.3841	244	3915	2.8522	3.9961	3.9342	83.2765	84.2842
DN	NFATC1_1	cluster_5	Common_1_DN	29201	25049.092	24723	25347	194.3282	1.1657	0.2213	0.001	0.0010909	6650	128410	79.943	1853	32944	20.51	1622	29201	18.179	24.391	22.7404	87.5337	88.6383
DN	TBX1_4	cluster_5	Common_1_DN	52245	44842.416	44305	45435	328.6655	1.1651	0.2204	0.001	0.0010909	11107	211898	72.371	3505	63358	21.639	2830	52245	17.844	25.4794	24.6557	80.7418	82.46
DN	SMAD_2	cluster_5	Common_1_DN	77143	66229.725	65782	66602	257.0275	1.1648	0.2201	0.001	0.0010435	28215	291809	73.323	9075	91574	23.01	7569	77143	19.384	26.8262	26.4361	83.405	84.2412
DN	EGR1_known9	cluster_3	DN	45758	39287.3	38894	39638	219.8804	1.1647	0.22	0.001	0.001	32718	444077	82.227	3721	48541	8.988	3498	45758	8.4727	10.6914	10.3041	94.007	94.2667
DN	TBX15_2	cluster_5	Common_1_DN	38031	32658.059	32385	32960	161.9239	1.1645	0.2197	0.001	0.0010909	20505	143570	74.719	6406	44116	22.96	5506	38031	19.793	26.852	26.4895	85.9507	86.2068
DN	RREB1_1	cluster_5	Common_1_DN	237353	204076.146	203208	205051	559.425	1.1631	0.2179	0.001	0.001	60217	1008607	79.968	17321	268951	21.324	15025	237353	18.819	24.9514	23.5328	86.7444	88.2514
DN	SMAD3_1	cluster_5	Common_1_DN	56264	48383.292	48021	48730	217.565	1.1629	0.2177	0.001	0.0010435	26078	216611	73.645	8111	66435	22.587	6846	56264	19.129	26.252	25.9747	84.4039	84.6903
DN	E2F2_2	cluster_5	Common_1_DN	14463	12445.7	12234	12671	139.4582	1.1621	0.2167	0.001	0.0010909	3788	64579	78.928	1050	16482	20.144	913	14463	17.677	24.1024	22.3958	86.9524	87.7503
SP8	TCF21_1	cluster_4	SP4	9630	8288.071	7992	8587	173.3829	1.1619	0.2165	0.001	0.001	10166	132387	47.014	1441	17927	6.3663	750	9630	3.4199	7.3775	7.2741	52.0472	53.7179
DN	RUNX2_4	cluster_3	DN	75517	65022.493	64591	65503	262.874	1.1614	0.2159	0.001	0.0010435	22375	342168	83.028	5278	79171	19.211	5017	75517	18.324	22.4223	22.0702	95.0549	95.3847
DN	TBX20_2	cluster_5	Common_1_DN	32009	27565.151	27201	27941	241.3449	1.1612	0.2156	0.001	0.0010435	7160	129186	74.304	2226	38036	21.877	1837	32009	18.411	25.6564	24.7775	82.5247	84.1545
DN	NFATC2_1	cluster_5	Common_1_DN	10679	9200.373	9082	9310	72.5341	1.1607	0.215	0.001	0.0010909	7464	44784	80.238	1994	11648	20.869	1823	10679	19.133	24.4239	23.8456	91.4243	91.681
DN	SP100_1	cluster_3	DN	2096	1807.393	1747	1868	37.6439	1.1596	0.2136	0.001	0.0013333	1107	14404	85.672	181	2174	12.93	172	2096	12.467	15.5375	14.5515	95.0276	96.4121
SP4	TCF21_1	cluster_1	SP8	8717	7519.113	7213	7798	174.4127	1.1593	0.2132	0.001	0.001	10517	136988	48.648	1237	15681	5.5687	675	8717	3.0956	6.4182	6.3633	54.5675	55.5896
SP8	TCF3_2	cluster_4	SP4	18844	16255.46	15760	16807	296.5846	1.1592	0.2132	0.001	0.0010435	23089	348566	45.448	2555	36581	4.7697	1272	18844	2.457	5.5091	5.4061	49.7847	51.5131
DN	SMAD_1	cluster_5	Common_1_DN	43894	37867.602	37606	38138	165.5573	1.1591	0.2131	0.001	0.0010435	20794	166413	76.255	6404	50170	22.989	5574	43894	20.113	26.8058	26.3765	87.0394	87.4905
DN	SPDEF_6	cluster_3	DN	21474	18525.452	18305	18762	139.8687	1.1592	0.2131	0.001	0.001	10392	145946	84.321	1652	22357	12.917	1579	21474	12.407	15.1944	14.7137	95.5811	96.0505
DN	NFKB_known11	cluster_5	Common_1_DN	40451	34920.307	34531	35261	226.9103	1.1584	0.2121	0.001	0.001	14339	173964	75.858	3973	46680	20.355	3411	40451	17.639	23.7883	23.2525	85.8545	86.656
DN	POU2F2_known14	cluster_5	Common_1_DN	4358	3764.186	3649	3875	70.0328	1.1577	0.2113	0.001	0.0012632	1238	18993	84.41	353	4849	21.55	311	4358	19.368	25.1212	22.9453	88.102	89.8742
DN	MAFG_1	cluster_5	Common_1_DN	42634	36840.381	36339	37366	309.1038	1.1573	0.2107	0.001	0.0010435	8246	165248	72.352	2762	52068	22.798	2186	42634	18.667	26.5098	25.8	79.1455	81.8814
DN	FOXP1_1	cluster_5	Common_1_DN	43124	37296.774	36652	38055	420.3581	1.1562	0.2094	0.001	0.0010435	10678	252594	65.084	3601	67880	17.49	2223	43124	11.111	20.8185	17.0725	61.7329	63.5298
DN	MAF_known12	cluster_5	Common_1_DN	41130	35592.379	35181	36046	255.5997	1.1556	0.2086	0.001	0.0010435	9989	170271	77.196	2875	47044	21.328	2475	41130	18.647	24.7773	24.1556	86.087	87.4288
DN	SPZ1_2	cluster_5	Common_1_DN	48087	41617.844	41291	41987	208.0201	1.1554	0.2084	0.001	0.0010435	18565	186152	75.582	5732	55792	22.653	4891	48087	19.524	26.3453	25.8321	85.328	86.1898
DN	SPIC_2	cluster_5	Common_1_DN	69500	60251.55	59774	60724	293.0821	1.1535	0.206	0.001	0.0010435	21125	275317	74.226	6525	82309	22.191	5433	69500	18.737	25.7183	25.2436	83.2644	84.4379
DN	SPIC_1	cluster_5	Common_1_DN	62626	54295.701	53735	54799	313.452	1.1534	0.2059	0.001	0.001	19310	251988	71.563	6133	77072	21.888	4906	62626	17.785	25.4065	24.8528	79.9935	81.2565
SP8	ELK4_1	cluster_1	SP8	4682	4059.856	3948	4167	64.6094	1.1532	0.2056	0.001	0.001	10505	84617	76.469	674	5337	4.8231	589	4682	4.2311	5.6069	5.5332	87.3887	87.7272
DN	HIF1A_2	cluster_3	DN	27508	23876.935	23591	24144	175.9042	1.1521	0.2042	0.001	0.001	15992	214291	80.94	2276	29792	11.253	2091	27508	10.39	13.0753	12.8368	91.8717	92.3335
SP4	TCF4_2	cluster_1	SP8	14682	12754.159	12467	13046	175.8893	1.1511	0.2031	0.001	0.001	28828	260376	48.438	3017	26562	4.9413	1643	14682	2.7313	5.6993	5.6388	54.4581	55.2745
DN	CUX1_8	cluster_6	Common_2	3115	2705.958	2603	2823	71.1831	1.1511	0.203	0.001	0.0011429	619	9964	75.019	266	3873	29.16	207	3115	23.453	33.441	31.2625	77.8195	80.4286
DN	TBX2_1	cluster_5	Common_1_DN	46872	40746.043	40332	41123	243.8866	1.1503	0.2021	0.001	0.0010435	11067	188839	77.429	3274	53684	22.012	2817	46872	19.219	25.4541	24.8211	86.0415	87.3109
SP4	ELK4_1	cluster_4	SP4	4875	4239.102	4128	4345	66.639	1.15	0.2016	0.001	0.001	10509	84669	76.516	703	5569	5.0327	610	4875	4.4055	5.8045	5.7577	86.771	87.5382
DN	NFKB_known10	cluster_5	Common_1_DN	37217	32389.305	32040	32708	205.0841	1.149	0.2004	0.001	0.001	12896	155666	77.254	3651	42572	21.128	3161	37217	18.47	24.5115	23.9082	86.579	87.4213
DN	SPI1_known4	cluster_5	Common_1_DN	63003	54834.712	54416	55250	254.0701	1.149	0.2003	0.001	0.0010435	19284	251359	76.991	5779	72542	22.219	4963	63003	19.298	25.7364	25.0649	85.8799	86.8504
SP4	PKNOX1_2	cluster_1	SP8	9929	8641.98	8390	8871	153.0809	1.1489	0.2003	0.001	0.0010435	9911	109901	52.023	1569	16823	7.9634	905	9929	4.7001	9.1313	9.0345	57.6801	59.0204
DN	STAT_known2	cluster_3	DN	31534	27447.486	27092	27778	213.0536	1.1489	0.2002	0.001	0.001	11028	221310	83.913	1770	33621	12.748	1643	31534	11.957	14.8984	14.2488	92.8249	93.7926
DN	ATF4_2	cluster_5	Common_1_DN	14254	12419.196	12183	12634	136.5565	1.1477	0.1988	0.001	0.0010909	4963	59634	73.418	1471	17153	21.118	1212	14254	17.549	24.4207	23.9025	82.3929	83.0992
DN	MYBL2_4	cluster_5	Common_1_DN	6288	5478.777	5334	5605	82.7753	1.1477	0.1987	0.001	0.0010435	1996	27994	79.043	524	7050	19.906	461	6288	17.755	23.0962	22.462	87.9771	89.1915
SP4	PKNOX2_2	cluster_1	SP8	10913	9516.461	9240	9823	181.4427	1.1467	0.1975	0.001	0.0010435	11364	126178	51.858	1726	18579	7.6357	991	10913	4.4851	8.7205	8.6489	57.416	58.7384
DN	PKNOX2_3	cluster_5	Common_1_DN	39584	34521.284	34124	34912	231.4681	1.1467	0.1974	0.001	0.0010435	13787	153514	71.649	4498	48655	22.709	3612	39584	18.475	26.1986	25.7853	80.3024	81.3565
DN	PKNOX1_1	cluster_5	Common_1_DN	58640	51152.219	50687	51686	291.1448	1.1464	0.1971	0.001	0.0010435	15318	230880	74.405	4839	69909	22.529	3986	58640	18.898	26.0217	25.3985	82.3724	83.8805
DN	E2F_known24	cluster_5	Common_1_DN	13861	12108.426	11885	12339	145.1559	1.1447	0.195	0.001	0.001	3603	61420	79.657	998	15843	20.547	862	13861	17.977	23.9245	22.5676	86.3727	87.4897
DN	ATF7_1	cluster_6	Common_2	21482	18790.136	18523	19086	168.9929	1.1433	0.1931	0.001	0.001	3904	50828	69.581	2132	27214	37.254	1658	21482	29.408	42.4693	42.2641	77.7674	78.9373
DN	PBX3_known1	cluster_5	Common_1_DN	24608	21526.068	21233	21792	173.0633	1.1432	0.193	0.001	0.0010435	8906	98882	73.528	2777	29759	22.128	2260	24608	18.298	25.3762	24.8862	81.3828	82.691
DN	SPIB_2	cluster_5	Common_1_DN	77117	67482.313	66999	67968	282.1733	1.1428	0.1925	0.001	0.0010435	23403	305650	75.803	7171	90414	22.423	6041	77117	19.126	25.8129	25.2305	84.2421	85.2932
DN	LMO2_2	cluster_5	Common_1_DN	9813	8589.277	8447	8731	86.1867	1.1425	0.1921	0.001	0.0010435	4777	38246	75.086	1473	11555	22.685	1247	9813	19.265	26.1042	25.6576	84.6572	84.9243
DN	CTCF_known1	cluster_5	Common_1_DN	129452	113420.357	111920	115083	883.2074	1.1413	0.1907	0.001	0.0010435	26314	479096	27.636	23232	415314	23.957	7218	129452	7.4674	27.4303	27.0201	31.0692	31.1697
DN	REL_1	cluster_5	Common_1_DN	30739	26940.716	26681	27216	164.8839	1.141	0.1903	0.001	0.001	15073	136291	76.106	4073	35821	20.003	3467	30739	17.165	23.0014	22.5539	85.1215	85.8128
DN	ATF2_1	cluster_3	DN	1373	1203.54	1149	1256	32.9067	1.1407	0.1899	0.001	0.0010435	1484	10388	76.433	228	1585	11.662	197	1373	10.102	13.2749	13.2172	86.4035	86.6246
DN	IRF6_1	cluster_5	Common_1_DN	12270	10757.715	10555	10958	125.7285	1.1406	0.1897	0.001	0.0010909	3319	53207	81.206	895	13587	20.737	797	12270	18.727	24.0133	23.0609	89.0503	90.3069
DN	REL_2	cluster_5	Common_1_DN	31324	27468.922	27212	27749	159.9191	1.1403	0.1895	0.001	0.0010435	15212	137460	76.313	4147	36451	20.236	3535	31324	17.39	23.2382	22.7877	85.2423	85.9345
SP8	RUNX_1	cluster_4	SP4	28039	24613.05	24144	25083	288.6663	1.1392	0.188	0.001	0.0010435	23767	334786	52.302	3468	47492	7.4195	2012	28039	4.3804	8.4655	8.3752	58.0161	59.0394
DN	ETV5_1	cluster_3	DN	3464	3043.4	2868	3245	116.7835	1.1382	0.1867	0.001	0.0010435	4257	38576	26.904	1288	11464	7.9952	389	3464	2.4159	9.1379	8.9797	30.2019	30.2163
DN	FOXO1_5	cluster_5	Common_1_DN	88975	78189.075	77667	78742	319.1541	1.1379	0.1864	0.001	0.0010435	32721	361528	75.797	9821	104850	21.983	8242	88975	18.654	25.1887	24.6108	83.9222	84.8593
DN	MYBL2_2	cluster_5	Common_1_DN	9608	8443.735	8267	8621	112.7289	1.1379	0.1863	0.001	0.0010435	2843	43039	79.915	738	10773	20.003	653	9608	17.84	22.9687	22.3239	88.4824	89.1859
DN	IKZF1_2	cluster_5	Common_1_DN	55613	48936.21	48609	49252	193.3123	1.1364	0.1845	0.001	0.0010435	27213	217711	79.439	8047	62565	22.829	7093	55613	20.292	26.0647	25.5444	88.1447	88.8884
DN	FOXO6_3	cluster_5	Common_1_DN	91159	80350.658	79835	80900	320.4523	1.1345	0.1821	0.001	0.0010435	28591	373838	76.349	8509	107163	21.886	7143	91159	18.617	24.9834	24.3846	83.9464	85.0657
DN	MTF1_3	cluster_5	Common_1_DN	51333	45249.763	44890	45638	224.6081	1.1344	0.182	0.001	0.001	16298	213937	79.022	4568	58056	21.444	4002	51333	18.961	24.5552	23.9944	87.6095	88.4198
DN	IRF4_1	cluster_5	Common_1_DN	12472	11009.73	10826	11196	113.5214	1.1328	0.1799	0.001	0.0010435	3899	54808	80.813	1029	13863	20.441	915	12472	18.39	23.4676	22.7558	88.9213	89.9661
DP	ELK4_1	cluster_2	DP	9951	8787.32	8637	8929	86.2534	1.1324	0.1794	0.001	0.001	10661	85897	77.625	1427	11332	10.241	1247	9951	8.9927	11.6968	11.5848	87.3861	87.8133
DN	PKNOX1_2	cluster_5	Common_1_DN	39735	35091.661	34716	35444	206.2395	1.1323	0.1793	0.001	0.0010435	13772	152898	72.377	4531	48957	23.175	3636	39735	18.809	26.4014	25.9879	80.2472	81.1631
DN	ATF2_2	cluster_6	Common_2	31758	28058.682	27839	28315	151.731	1.1318	0.1787	0.001	0.0010435	5805	63978	74.542	3437	37664	43.883	2883	31758	37.002	49.6641	49.6389	83.8813	84.3192
SP8	ATF2_1	cluster_1	SP8	735	649.331	608	692	26.2037	1.1317	0.1785	0.001	0.0010435	1370	9590	70.561	131	917	6.7471	105	735	5.408	7.6642	7.6642	80.1527	80.1527
DN	CUX2_2	cluster_6	Common_2	1302	1150.412	1082	1213	37.6345	1.1317	0.1784	0.001	0.0013333	368	3312	68.275	207	1769	36.467	151	1302	26.84	41.0326	39.3116	72.9469	73.6009
DN	E2F2_3	cluster_5	Common_1_DN	9910	8757.081	8572	8937	112.2403	1.1316	0.1784	0.001	0.001	2541	43236	81.281	696	11157	20.975	611	9910	18.63	24.0457	22.9207	87.7874	88.8232
DP	ETV5_1	cluster_2	DP	12364	10927.46	10738	11114	111.6943	1.1314	0.1782	0.001	0.0010435	12055	109704	76.51	1599	14322	9.9884	1375	12364	8.6229	11.4061	11.2703	85.9912	86.3287
SP4	RUNX_1	cluster_1	SP8	37141	32852.959	32304	33425	348.3856	1.1305	0.177	0.001	0.0010435	22871	322027	50.309	4759	65825	10.284	2651	37141	5.8024	11.5911	11.5335	55.705	56.4239
SP4	MESP1_1	cluster_1	SP8	13308	11774.545	11486	12080	175.7383	1.1302	0.1766	0.001	0.001	28293	255419	48.619	2782	24456	4.6552	1487	13308	2.5332	5.2557	5.2103	53.4508	54.4161
DN	PKNOX2_2	cluster_5	Common_1_DN	46506	41164.354	40784	41532	238.4318	1.1298	0.176	0.001	0.0010435	15871	176400	72.498	5291	57301	23.55	4248	46506	19.113	26.7658	26.3639	80.2873	81.1609
DN	CTCF_known1	cluster_6	Common_2	225746	199894.965	198248	201717	972.6558	1.1293	0.1755	0.001	0.0010435	26314	479096	27.636	39885	727160	41.946	12437	225746	13.022	47.2638	47.1192	31.1821	31.0449
DN	AHR::ARNT::HIF1A_1	cluster_3	DN	13495	11958.648	11812	12098	93.5169	1.1285	0.1744	0.001	0.0010435	14502	119460	81.912	1807	14714	10.089	1659	13495	9.2533	11.4398	11.2967	91.8096	91.7154
DN	MAF_known11	cluster_5	Common_1_DN	57102	50646.756	50139	51201	314.5982	1.1275	0.1731	0.001	0.0010435	13743	221582	72.337	4559	70796	23.112	3582	57102	18.641	26.0642	25.7701	78.5699	80.6571
SP8	TCF4_1	cluster_4	SP4	13593	12062.629	11743	12338	179.0565	1.1269	0.1723	0.001	0.0010909	32686	295493	45.063	3083	27113	4.1348	1520	13593	2.0729	4.6503	4.6001	49.3026	50.1346
DN	ATF2_1	cluster_6	Common_2	5909	5244.874	5162	5326	46.0328	1.1266	0.172	0.001	0.0010435	1484	10388	76.433	984	6855	50.438	846	5909	43.477	57.0081	56.8829	85.9756	86.1999
DN	ELK3_2	cluster_6	Common_2	26949	23921.26	23537	24312	244.7214	1.1266	0.1719	0.001	0.0011429	6130	55327	26.313	10075	90986	43.272	2984	26949	12.817	48.6786	48.7086	29.6179	29.6188
DN	HESX1_2	cluster_5	Common_1_DN	3647	3241.678	3125	3347	67.3982	1.125	0.1699	0.001	0.0014118	1143	16019	80.489	323	4233	21.269	275	3647	18.325	24.0595	22.7667	85.1393	86.1564
DN	ATF3_known5	cluster_5	Common_1_DN	49881	44348.337	43913	44845	275.237	1.1248	0.1696	0.001	0.001	14705	206515	71.894	4636	62555	21.777	3618	49881	17.365	24.6039	24.1537	78.0414	79.7394
DN	BATF_known1	cluster_5	Common_1_DN	14372	12785.525	12552	13016	144.2333	1.1241	0.1687	0.001	0.0010435	4780	62340	72.002	1417	17961	20.745	1116	14372	16.599	23.3473	23.0542	78.7579	80.0178
DN	ELK3_1	cluster_6	Common_2	28221	25142.192	24683	25541	253.2741	1.1225	0.1667	0.001	0.0011429	6350	57329	26.539	10482	94751	43.862	3124	28221	13.064	49.1969	49.2264	29.8035	29.7844
SP8	ID4_1	cluster_4	SP4	14422	12855.335	12511	13203	206.4099	1.1219	0.1659	0.001	0.0010435	29653	268120	45.899	3214	28330	4.8498	1608	14422	2.4689	5.4227	5.3789	50.0311	50.9072
DN	CTCF_known2	cluster_6	Common_2	150429	134098.49	133157	135129	649.9083	1.1218	0.1658	0.001	0.0010435	17849	287162	34.367	24385	390108	46.688	9351	150429	18.003	52.3895	52.3847	38.3473	38.5609
DN	XBP1_4	cluster_6	Common_2	36028	32123.12	31770	32474	208.1783	1.1216	0.1655	0.001	0.0010435	6101	79442	72.628	3479	44601	40.775	2782	36028	32.938	45.5991	45.3513	79.9655	80.7785

DN	ATF6_1	cluster_6	Common_2	14979	13383.714	13235	13528	86.7036	1.1192	0.1625	0.001	0.0010435	4516	31617	73.316	2634	18311	42.461	2145	14979	34.735	47.4978	47.3764	81.4351	81.8033
DN	HESX1_1	cluster_5	Common_1_DN	4278	3822.52	3725	3911	54.2599	1.1191	0.1624	0.001	0.0012632	1814	16345	82.401	556	4768	24.037	496	4278	21.567	27.3429	26.1731	89.2086	89.7232
DN	MYBL1_3	cluster_5	Common_1_DN	4782	4280.378	4162	4404	73.4057	1.1172	0.1598	0.001	0.0010909	1868	20588	77.661	534	5618	21.192	445	4782	18.038	23.8223	23.2271	83.3333	85.1193
DN	NFATC1_3	cluster_5	Common_1_DN	12913	11571.696	11380	11791	122.5357	1.1159	0.1582	0.001	0.0010909	4137	53815	79.338	1232	15103	22.266	1038	12913	19.037	25.0906	23.9952	84.2532	85.4996
DN	MAFF_1	cluster_5	Common_1_DN	44038	39506.82	39067	39963	265.7451	1.1147	0.1566	0.001	0.001	12562	176219	70.456	4204	56810	22.714	3187	44038	17.607	25.3702	24.9905	75.8088	77.518
DN	POU2F2_known12	cluster_6	Common_2	16585	14882.571	14685	15092	122.0183	1.1144	0.1562	0.001	0.0010435	5446	54541	74.724	2101	20388	27.933	1690	16585	22.722	31.032	30.4083	80.4379	81.3469
DN	ELK4_1	cluster_3	DN	8133	7300.138	7202	7415	65.04	1.1141	0.1558	0.001	0.001	11601	93425	84.428	1097	8678	7.8423	1023	8133	7.3498	8.8182	8.7054	93.2543	93.7198
DN	ETV3_1	cluster_6	Common_2	22815	20529.349	20158	20922	229.5811	1.1113	0.1523	0.001	0.0010435	5350	48305	25.577	8919	80297	42.516	2526	22815	12.08	47.215	47.2311	28.3216	28.4133
SP4	PKNOX2_3	cluster_1	SP8	10173	9156.366	8913	9391	149.6835	1.111	0.1519	0.001	0.0010435	10156	113004	52.742	1632	17576	8.2032	924	10173	4.748	9.0981	9.0023	56.6176	57.8801
SP4	RUNX_2	cluster_1	SP8	30372	27337.526	26800	27903	329.4396	1.111	0.1519	0.001	0.0010435	18786	264509	50.895	3931	54123	10.414	2165	30372	5.844	11.5245	11.4824	55.075	56.1166
SP8	RUNX_2	cluster_4	SP4	21823	19645.388	19222	20095	267.1049	1.1108	0.1517	0.001	0.0010435	19635	276608	53.223	2748	37394	7.1951	1569	21823	4.1991	7.9908	7.8895	57.0961	58.3596
DN	FLI1_2	cluster_6	Common_2	48114	43338.662	43014	43730	208.8218	1.1102	0.1508	0.001	0.001	8020	104443	74.293	4558	58681	41.741	3711	48114	34.225	46.2718	46.0672	81.4173	81.9925
DN	MYBL1_5	cluster_5	Common_1_DN	12776	11508.127	11313	11696	121.0433	1.1102	0.1508	0.001	0.0010435	3552	57284	82.002	933	14337	20.523	821	12776	18.289	23.1137	22.3029	87.9957	89.1121
DN	MAF_known1	cluster_5	Common_1_DN	129193	116421.184	115680	117234	458.0432	1.1097	0.1502	0.001	0.001	33530	506311	73.776	11086	160598	23.401	8741	129193	18.825	26.0692	25.5165	78.8472	80.445
DN	IRF_known21	cluster_6	Common_2	46383	41799.824	41429	42196	243.192	1.1096	0.1501	0.001	0.001	9344	135063	76.665	3962	55647	31.587	3255	46383	26.328	34.8352	34.3418	82.1555	83.3522
DN	GMEB2_2	cluster_5	Common_1_DN	9388	8465.149	8297	8644	105.9829	1.109	0.1493	0.001	0.001	3215	42707	77.048	866	11140	20.098	715	9388	16.937	22.2395	21.9823	82.5635	84.2729
DN	ETV2_1	cluster_6	Common_2	25337	22874.157	22437	23312	257.4939	1.1077	0.1475	0.001	0.0010435	7018	70432	23.896	9620	95999	32.57	2527	25337	8.5962	36.0074	35.9737	26.2682	26.393
DN	FLI1_4	cluster_6	Common_2	52383	47300.869	46933	47731	223.2658	1.1074	0.1472	0.001	0.001	8592	111995	76.37	4842	62328	42.502	4039	52383	35.72	47.0088	46.7726	83.4159	84.0441
DN	IRF4_2	cluster_6	Common_2	39871	36006.322	35640	36359	220.6387	1.1073	0.1471	0.001	0.001	7884	113080	77.321	3385	47396	32.408	2812	39871	27.263	35.6672	35.2591	83.0724	84.1231
DN	KLF13_1	cluster_6	Common_2	205085	185264.608	184412	186017	509.0192	1.107	0.1466	0.001	0.001	24149	415074	75.178	14628	248271	44.967	11959	205085	37.145	49.5217	49.4093	81.7542	82.6053
DP	ETV4_2	cluster_2	DP	6158	5563.677	5347	5791	140.1134	1.1068	0.1464	0.001	0.0011429	5095	46084	26.755	2328	20847	12.103	687	6158	3.5751	13.4838	13.3626	29.5103	29.539
DN	NFKB_known11	cluster_6	Common_2	70046	63302.143	62811	63783	272.3852	1.1065	0.146	0.001	0.001	14339	173964	75.858	7103	84596	36.889	5823	70046	30.544	40.6095	40.2647	81.9794	82.8006
DN	POU2F2_known1	cluster_6	Common_2	22112	19984.108	19723	20292	184.7106	1.1065	0.146	0.001	0.0010435	4025	72871	75.443	1610	27604	28.578	1257	22112	22.892	31.2298	30.344	78.0745	80.1043
DN	IKZF3_1	cluster_5	Common_1_DN	26081	23574.217	23281	23833	160.9966	1.1063	0.1458	0.001	0.0010435	8816	106058	78.616	2666	30716	22.768	2227	26081	19.333	25.2609	24.5913	83.5334	84.9101
DN	ETV7_1	cluster_5	Common_1_DN	69454	62786.526	62283	63229	292.9357	1.1062	0.1456	0.001	0.001	30877	278376	62.701	11316	100370	22.607	7730	69454	15.644	25.0348	24.9497	68.3104	69.198
DN	ELK4_2	cluster_6	Common_2	31103	28125.777	27688	28530	269.3021	1.1059	0.1452	0.001	0.0011429	7819	70678	26.176	11869	107416	39.782	3436	31103	11.519	43.9442	44.0066	28.9494	28.9556
DN	ERG_2	cluster_6	Common_2	48123	43552.424	43182	43946	233.7453	1.1049	0.144	0.001	0.001	8341	108611	75.068	4547	58454	40.401	3715	48123	33.261	44.539	44.3077	81.7022	82.3263
DN	ETS_known17	cluster_6	Common_2	114983	104074.209	103522	104760	388.235	1.1048	0.1438	0.001	0.001	15240	260380	73.574	8506	142533	40.275	6764	114983	32.49	44.3832	44.1597	79.5203	80.6711
DN	E2F7_1	cluster_5	Common_1_DN	16104	14576.812	14356	14780	128.5762	1.1048	0.1437	0.001	0.0010435	5640	73401	81.261	1499	18468	20.445	1293	16104	17.828	22.9255	21.9398	86.2575	87.1995
DN	ETV1_1	cluster_6	Common_2	24514	22203.545	21791	22569	229.0159	1.1041	0.1428	0.001	0.0010909	5090	46038	26.319	9308	84323	48.205	2708	24514	14.014	53.2024	53.2473	29.0933	29.0715
DN	GMEB2_2	cluster_6	Common_2	19869	17995.885	17792	18222	132.814	1.1041	0.1428	0.001	0.001	3215	42707	77.048	1808	23595	42.568	1511	19869	35.846	46.9984	46.524	83.573	84.2085
DN	E2F8_1	cluster_5	Common_1_DN	23278	21086.791	20871	21316	136.5612	1.1039	0.1426	0.001	0.001	9501	104773	81.344	2499	26482	20.56	2175	23278	18.073	22.8923	22.2176	87.0348	87.9012
DP	ETV1_1	cluster_2	DP	6208	5624.053	5401	5833	141.467	1.1038	0.1425	0.001	0.0010909	5186	46934	26.831	2353	21073	12.047	693	6208	3.5489	13.3629	13.2271	29.4518	29.4595
DN	ERG_4	cluster_6	Common_2	48679	44108.378	43733	44460	214.3319	1.1036	0.1422	0.001	0.001	8373	109062	76.033	4544	58432	40.736	3756	48679	33.937	44.8585	44.6342	82.6585	83.3088
DN	ETV4_2	cluster_6	Common_2	23994	21742.341	21415	22101	203.9253	1.1036	0.1422	0.001	0.0011429	5010	45275	26.285	9121	82630	47.972	2653	23994	13.93	52.9541	52.9961	29.0867	29.0379
DN	XBP1_2	cluster_6	Common_2	30118	27295.967	26923	27657	230.0394	1.1034	0.1419	0.001	0.0010435	3231	74429	77.172	1647	36240	37.575	1327	30118	31.228	41.0709	40.4654	80.5707	83.1071
DN	POU2F2_known13	cluster_6	Common_2	21554	19546.669	19205	19876	213.1205	1.1027	0.141	0.001	0.0010909	3197	67338	75.85	1401	27261	30.707	1071	21554	24.279	33.5002	32.0087	76.4454	79.0653
DN	POU2F2_known11	cluster_6	Common_2	16463	14937.419	14732	15150	123.3716	1.1021	0.1403	0.001	0.0010435	5502	55147	73.881	2129	20693	27.723	1677	16463	22.056	30.4798	29.8529	78.7694	79.5583
DN	KLF7_1	cluster_6	Common_2	682593	619400.907	617867	621103	945.3735	1.102	0.1402	0.001	0.001	81419	1312000	74.111	52413	841335	47.525	42322	682593	38.558	51.9805	52.0269	80.7471	81.1321
SP4	LMO2_1	cluster_1	SP8	19708	17889.43	17467	18281	248.595	1.1017	0.1397	0.001	0.001	35670	395160	47.238	3559	38297	4.5781	1789	19708	2.3559	5.0219	4.9873	50.2669	51.4609
DN	FOXP3_2	cluster_6	Common_2	10789	9810.267	9668	9932	81.7505	1.0998	0.1372	0.001	0.0010909	5499	32994	72.569	2303	13622	29.961	1814	10789	23.73	32.9878	32.6999	78.7668	79.2028
DN	KLF16_1	cluster_6	Common_2	483434	439633.532	438659	440692	607.7239	1.0996	0.137	0.001	0.001	87857	980098	74.479	54190	597132	45.377	43804	483434	36.737	49.8583	49.3251	80.8341	80.9593
DN	KLF4_1	cluster_6	Common_2	430638	391716.516	390916	392530	499.0541	1.0994	0.1367	0.001	0.001	90604	870001	74.9	54917	525754	45.263	44851	430638	37.075	49.5022	49.4986	81.6705	81.9086
DN	BATF_known1	cluster_6	Common_2	23292	21195.618	20915	21462	161.4653	1.0989	0.1361	0.001	0.0010435	4780	62340	72.002	2327	29701	34.304	1799	23292	26.902	37.636	37.3628	77.3098	78.4216
SP4	ETV5_1	cluster_4	SP4	6162	5610.792	5493	5737	77.3118	1.0982	0.1352	0.001	0.0010435	11580	105410	73.515	857	7666	5.3464	684	6162	4.2975	5.9067	5.8457	79.8133	80.3809
DN	KLF14_1	cluster_6	Common_2	120280	109590.601	108583	110573	590.5486	1.0975	0.1343	0.001	0.001	16591	224164	22.673	36453	489216	49.482	9006	120280	12.166	54.2824	53.6571	24.7058	24.5863
DN	POU2F3_2	cluster_6	Common_2	10294	9381.159	9242	9563	93.4353	1.0973	0.134	0.001	0.0010909	4271	34232	74.798	1627	12784	27.933	1302	10294	22.493	30.8447	30.0713	80.0246	80.5225
SP8	LMO2_1	cluster_4	SP4	19127	17440.149	17052	17884	234.4081	1.0967	0.1332	0.001	0.001	35542	394212	47.125	3507	37544	4.4881	1752	19127	2.2865	4.9294	4.8917	49.9572	50.9456
DN	PBX1_2	cluster_6	Common_2	15801	14417.35	14175	14684	151.189	1.096	0.1322	0.001	0.0010435	3613	54534	73.03	1495	20344	28.977	1137	15801	22.506	31.4697	30.818	76.0535	77.6691
DN	IRF6_1	cluster_6	Common_2	21296	19432.836	19207	19664	141.6087	1.0959	0.1321	0.001	0.0010909	3319	53207	81.206	1569	24456	37.325	1352	21296	32.503	40.7352	40.0248	86.1695	87.0788
DN	SP8_1	cluster_6	Common_2	123907	113095.083	111999	114097	634.2321	1.0956	0.1317	0.001	0.001	21179	262744	21.027	45886	551019	44.097	10255	123907	9.9161	48.4206	47.1588	22.3489	22.4869
DN	SOX12_1	cluster_6	Common_2	13125	11986.923	11774	12205	129.8951	1.0949	0.1308	0.001	0.0010435	3600	46915	74.344	1367	16802	26.625	1045	13125	20.799	29.0278	27.9761	76.4448	78.1157
DN	EGR4_1	cluster_6	Common_2	199409	182134.284	181531	182730	361.1797	1.0948	0.1307	0.001	0.001	34373	389734	77.181	21237	238147	47.161	17680	199409	39.49	51.4357	51.1654	83.2509	83.7336
DN	SP1_known3	cluster_5	Common_1_DN	140118	128004.297	127296	128645	415.2205	1.0946	0.1304	0.001	0.001	47992	629173	78.933	13072	164835	20.679	10959	140118	17.578	22.8351	22.2702	83.8357	85.005
DN	SOX21_3	cluster_6	Common_2	19334	17665.524	17371	17960	177.2284	1.0944	0.1302	0.001	0.0010909	4326	64953	73.757	1735	24817	28.181	1322	19334	21.954	30.5594	29.7661	76.196	77.9063
DN	REL_1	cluster_6	Common_2	53242	48651.095	48379	48971	180.47	1.0944	0.1301	0.001	0.001	15073	136291	76.106	7263	64662	36.108	5935	53242	29.731	39.375	39.0649	81.7155	82.3389
DN	RBPJ_2	cluster_5	Common_1_DN	34762	31767.747	31448	32070	184.3241	1.0943	0.1299	0.001	0.0010909	13859	138744	77.101	4334	41855	23.259	3543	34762	19.317	25.5646	25.0548	81.749	83.0534
DN	TCF7_1	cluster_6	Common_2	51287	46887.339	46357	47438	312.0205	1.0938	0.1294	0.001	0.0010435	13527	217263	71.046	4400	67875	22.195	3274	51287	16.771	24.2034	23.606	74.4091	75.561
DN	TCF7L1_1	cluster_6	Common_2	46531	42543.749	42026	43013	308.2938	1.0937	0.1292	0.001	0.0010435	12339	198117	70.718	4005	61781	22.053	2966	46531	16.609	24.0376	23.4866	74.0574	75.316
DN	REL_2	cluster_6	Common_2	53834	49237.204	48923	49533	186.3532	1.0934	0.1288	0.001	0.0010435	15212	137460	76.313	7335	65260	36.23	6007	53834	29.887	39.4886	39.1634	81.895	82.4916
DN	TCF7_2	cluster_6	Common_2	41979	38396.156	38037	38787	235.0545	1.0933	0.1287	0.001	0.0010435	15144	167157	70.642	5168	55411	23.417	3868	41979	17.741	25.5415	25.1135	74.8452	75.7593
SP4	RBPJ_1	cluster_1	SP8	6570	6009.159	5820	6176	111.4159	1.0933	0.1287	0.001	0.0010435	11294	79074	54.99	1618	11061	7.692	952	6570	4.5689	8.4293	8.3087	58.8381	59.3979
DN	IRF4_1	cluster_6	Common_2	21328	19514.872	19279	19718	138.6709	1.0929	0.1282	0.001	0.0010435	5839	54808	80.813	1798	24589	36.256	1536	21328	31.447	39.3947	38.914	85.4283	86.738
DN	MYCN_1	cluster_6	Common_2	63242	57863.268	57509	58200	203.0306	1.093	0.1282	0.001	0.001	12503	137921	76.685	6966	76024	42.27	5759	63242	35.163	46.0609	45.8538	82.673	83.1869
DN	POU2F2_known15	cluster_6	Common_2	13039	11935.365	11744	12141	122.3901	1.0925	0.1276	0.001	0.0010909	3911	43212	75.23	1524	16326	28.423	1212	13039	22.7	30.9895	30.1745	79.5276	79.8665
DN	XBP1_2	cluster_5	Common_1_DN	17198	15744.895	15419	16063	193.1538	1.0923	0.1273	0.001	0.0010435	3231	74429	77.172	971	20992	21.766	774	17198	17.832	23.9554	23.1066	79.7116	81.9264
SP4	HESX1_1	cluster_1	SP8	1097	1004.326	939	1084	43.9073	1.0922	0.1272	0.023	0.024	1328	11959	60.289	201	1706	8.6005	125	1097	5.5303	9.4127	9.173	62.1891	64.3025
SP8	TCF4_2	cluster_4	SP4	14901	13645.173	13247	13945	201.1069	1.092	0.127	0.001	0.001	28489	257334	47.872	3276	28814	5.3602	1668	14901	2.772	5.8549	5.7905	50.9158	51.7144
DN	FOXO3_1	cluster_6	Common_2	26458	24240.737	23939	24608	204.3849	1.0915	0.1263	0.001	0.0010435	6232	81801	74.85	2720	33781	30.911	2090	26458	24.21	33.5366	32.3443	76.8382	78.3221
DN	GMEB2_3	cluster_6	Common_2	19535	17897.159	17730	18056	107.2679	1.0915	0.1263	0.001	0.0010435	2360	33394	80.191	1593	22378	53.738	1382	19535	46.911	58.5593	58.4985	86.7546	87.2956
DN	FOXO4_2	cluster_6	Common_2	31958	29286.526	28959	29614	210.7873	1.0912	0.1259	0.001	0.0010435	7348	96298	75.255	3236	40377	31.554	2508	31958	24.974	34.1317	33.1866	77.5031	79.149
DN	KLF12_2	cluster_6	Common_2	326200	298959.471	298046	299864	517.9304	1.0911	0.1258	0.001	0.001	46999	672450	77.85	27197	386464	44.741	22801	326200	37.764	48.5138	48.5092	83.8365	84.4063
DN	TCF7L2_known7	cluster_6	Common_2	50437	46250.988	45798	46732	287.6711	1.0905	0.125	0.001	0.0010435	14358	201641	70.809	4944	66905	23.495	3667	50437	17.712	25.5398	25.0133	74.1707	75.386
DN	EGR1_known12	cluster_6	Common_2	478476	439032.775	437940	440175	679.5493	1.0898	0.1241	0.001	0.001	57885	958377	76.536	35995	584207	46.655	29281	478476	38.211	50.5848	49.9257	81.3474	81.9018
DN	POU2F2_known5	cluster_6	Common_2	17866	16395.722	16166	16671	158.1382	1.0897	0.1239	0.001	0.0010909	4660	60686	74.223	1820	22745	27.819	1409	17866	21.851	30.3261	29.4401	77.4176	78.5491
DN	MTF1_1	cluster_5	Common_1_DN	147500	135393.63	134692	136033	396.5806	1.0894	0.1236	0.001	0.001	48686	649383	82.303	12833	166540	21.107	11301	147500	18.694	23.212	22.7139	88.062	88.5673
DN	SOX11_1	cluster_6	Common_2	65339	59982.833	59453	60510	335.1456	1.0893	0.1234	0.001	0.0010435	13495	218540	74.839	5407	83403	28.561	4168	65339	22.375	30.8855	29.898	77.0853	78.3413
DN	ATF7_1	cluster_5	Common_1_DN	10479	9623.533	9399	9842	134.9087	1.0889	0.1229	0.001	0.001	3904	50828	69.581	1102	13946	19.091	817	10479	14.345	20.9273	20.6166	74.1379	75.1398
DN	EWSR1::FLI1_1	cluster_6	Common_2	391158	359246.188	357746	360782	978.2867	1.0888	0.1228	0.001	0.0010435	56590	1127364	69.149	28139	535783	32.863	20173	391158	23.992	35.6476	34.6967	71.6905	73.0068
DN	MYC_known20	cluster_6	Common_2	74298	68265.091	67717	68784	326.9979	1.0884	0.1222	0.001	0.001	11316	185626	73.632	5818	93509	37.092	4549	74298	29.472	40.1997	40.0256	78.1884	79.4555
DN	NFKB_known10	cluster_6	Common_2	61004	56050.481	55671	56425	238.9764	1.0884	0.1222	0.001	0.001	12896	155666	77.254	6222	73667	36.559	5103	61004	30.275	39.5704	39.189	82.0154	82.8105
DN	POU2F2_known10	cluster_6	Common_2	16479	15140.549	14936	15322	114.6051	1.0884	0.1222	0.001	0.0010435	6013	54229	74.503	2357	20734	28.485	1858	16479	22.64	30.8997	30.3878	78.829	79.4782
DN	HIF1A_1	cluster_6	Common_2	79968	73481.596	73136	73856	218.3724	1.0883	0.122	0.001	0.0010435	13599	153110	79.913	8327	92629	48.346	7145	79968	41.738	52.5406	52.2291	85.8052	86.3315
DN	GMEB1_1	cluster_6	Common_2	18438	16947.674	16546	17373	244.1402	1.0879	0.1216	0.001	0.0010435	1931	30986	22.575	4719	76123	55.459	1161	18438	13.433	60.1243	59.5043	24.6027	24.2213
DN	IKZF1_1	cluster_5	Common_1_DN	35029	32199.389	31907	32496	169.8863	1.0879	0.1215	0.001	0.0010435	11933	143526	82.189	3496	40165	23	3004	35029	20.059	25.1739	24.406	85.9268	87.2127
DN	IRF_known14	cluster_6	Common_2	116796	107388.891	106680	108175	471.2862	1.0876	0.1211	0.001	0.001	16394	342215	77.672	7220	143449	32.558	5745	116796	26.509	35.0433	34.1294	79.5706	81.4199
DN	EGR3_3	cluster_6	Common_2	286223	263376.079	262662	264149	452.7351	1.0867	0.12	0.001	0.001	35070	516864	79.095	23105	336738	51.531	19557	286223	43.8	55.7656	54.2184	84.644	84.9987
DN	TCF7L2_known5	cluster_6	Common_2	55750	51302.082	50791	51914	337.369	1.0867	0.12	0.001	0.0010435	13945	223921	71.487	4751	73608	23.499	3546	55750	17.798	25.4285	24.8972	74.6369	75.7391
DN	SPDEF_4	cluster_6	Common_2	40068	36873.192	36431	37240	238.5969	1.0866	0.1199	0.001	0.0010435	8815	132735	73.627	3617	51863	28.768	2732	40068	22.225	30.9926	30.1865	75.5322	77.2574
DN	ETV6_1	cluster_6	Common_2	205396	189040.26	188379	189748	424.1621	1.0865	0.1197	0.001	0.001	32656	469723	77.86	17137	244277	40.491	14275	205396	34.046	43.7133	43.727	83.2993	84.0832
DN	SMAD4_1	cluster_5	Common_1_DN	111353	102502.74	101849	103101	387.4224	1.0863	0.1195	0.001	0.001	32547	462220	75.022	10079	138396	22.463	7955	111353	18.074	24.4416	24.0909	78.9265	80.4597
DN	TCF7L1_2	cluster_6	Common_2	43723	40246.163	39864	40635	240.982	1.0864	0.1195	0.001	0.0010435	14657	161969	71.069	5380	57741	25.336	4019	43723	19.185	27.4203	26.9947	74.7026	75.7226
DN	SOX5_3	cluster_6	Common_2	12644	11642.082	11401	11871	138.6773	1.0861	0.1191	0.001	0.0011429	2922	43910	74.555	1177	16437	27.909	885	12644	21.468	30.2875	28.7953	75.1912	76.924
DN	SOX17_5	cluster_6	Common_2	10520	9686.962	9484	9896	128.1288	1.086	0.119	0.001	0.0011429	2792	39191	71.088	1054	14122	25.616	769	10520	19.082	27.543	26.8429	72.9602	74.4937
DN	SOX5_1	cluster_6	Common_2	7567	6968.029	6842	7102	78.9998	1.0859	0.119	0.001	0.0010909	2705	24345	72.35	1115	9815	29.169	856	7567	22.488	31.6451	31.0824	76.7713	77.0963
DN	POU2F3_1	cluster_6	Common_2	20940	19285.893	19008	19585	174.2886	1.0858	0.1187	0.001	0.0010435	4595	69456	74.624	1853	26777	28.77	1422	20940	22.498	30.9467	30.1486	76.7404	78.2014
DN	SOX13_1	cluster_6	Common_2	11182	10299.31	10089	10525	134.6248	1.0857	0.1186	0.001	0.0010909	2495	37512	75.346	1044	14517	29.159	783	11182	22.46	31.3828	29.8091	75	77.0269
DN	ETV7_1	cluster_6	Common_2	84387	77739.114	77234	78291	327.3504	1.0855	0.1184	0.001	0.001	30877	278376	62.701	13931	124176	27.969	9381	84387	19.007	30.3818	30.314	67.339	67.9576
DN	RREB1_2	cluster_6	Common_2	158441	145990.565	144278	147738	1059.3583	1.0853	0.1181	0.001	0.001	18681	474233	23.829	30076	678272	34.082	6886	158441	7.9613	36.861	33.4099	22.8953	23.3595
DN	SOX4_1	cluster_6	Common_2	70983	65408.665	64926	65926	309.701	1.0852	0.118	0.001	0.0010435	14546	235575	74.812	5901	90966	28.888	4520	70983	22.542	31.0738	30.1318	76.5972	78.0325
DN	FOXO3_4	cluster_6	Common_2	8637	7960.792	7798	8127	101.6815	1.0849	0.1176	0.001	0.0010909	2345	30568	73.157	913	11306	27.058	685	8637	20.671	29.2111	28.255	75.0274	76.3931
DN	SP4_2	cluster_6	Common_2	578065	533027.08	531845	534247	717.7934	1.0845	0.117	0.001	0.001	66957	1120770	78.67	40984	683173	47.954	34496	578065	40.576	51.5196	51.5775	84.1694	84.6147
DN	RBPJ_1	cluster_5	Common_1_DN	29369	27121.11	26917	27342	125.0273	1.0829	0.1149	0.001	0.0010435	16019	112173	78.007	5146	35199	24.478	4253	29369	20.424	26.5497	26.1819	82.6467	83.437
DN	CUX1_9	cluster_6	Common_2	3633	3356.481	3221	3499	85.0883	1.0824	0.1142	0.001	0.0012632	631	10867	73.396	296	4797	32.399	216	3633	24.537	34.2314	33.4335	72.973	75.7348
DN	HIF1A_2	cluster_6	Common_2	113764	105107.474	104649	105560	262.4276	1.0824	0.1142	0.001	0.001	15992	214291	80.94	9872	130767	49.392	8524	113764	42.97	53.3017	53.0886	86.3452	86.9975
DN	PBX1_4	cluster_6	Common_2	12235	11304.912	11132	11502	114.1038	1.0823	0.1141	0.001	0.0010909	3459	38700	71.853	1474	16039	29.779	1111	12235	22.716	32.1191	31.615	75.3731	76.2828
DN	EGR3_2	cluster_6	Common_2	111617	103141.505	101983	104262	676.8091	1.0822	0.1139	0.001	0.001	13370	205199	20.858	33684	506918	51.527	7420	111617	11.346	55.4974	54.3945	22.0283	22.0187
DN	SOX2_6	cluster_6	Common_2	10774	9958.256	9736	10173	141.7318	1.0819	0.1136	0.001	0.0010435	2362	37807	70.103	987	14899	27.626	699	10774	19.977	29.5936	28.4974	70.8207	72.3136
DN	HMGA1_1	cluster_6	Common_2	8644	7993.86	7894	8109	65.1381	1.0813	0.1128	0.001	0.0010435	4316	26040	76.796	1786	10593	31.24	1452	8644	25.493	33.6423	33.1951	81.299	81.6011
DN	MYC_known13	cluster_5	Common_1_DN	32229	29805.18	29485	30103	193.1147	1.0813	0.1128	0.001	0.0010909	12241	134783	75.676	3715	39745	22.315	2967	32229	18.095	24.2382	23.9118	79.8654	81.0894
DN	ATF3_known5	cluster_6	Common_2	75331	69683.663	69142	70356	343.9784	1.081	0.1124	0.001	0.001	14705	206515	71.894	7138	98043	34.132	5409	75331	26.225	36.7834	36.4773	75.7775	76.8347
DN	EGR4_2	cluster_6	Common_2	481443	445583.262	444538	446634	627.8143	1.0805	0.1117	0.001	0.001	55529	908524	79.001	35489	572963	49.822	29713	481443	41.864	53.509	52.9918	83.7245	84.0269
DN	SOX_1	cluster_6	Common_2	29756	27548.786	27218	27877	195.0474	1.0801	0.1112	0.001	0.0010435	8206	98530	71.552	3385	39308	28.545	2511	29756	21.609	30.5996	30.1999	74.1802	75.6996
DN	SOX11_2	cluster_6	Common_2	18675	17290.682	17000	17591	175.8617	1.0801	0.1111	0.001	0.0010909	4488	62863	74.087	1822	24297	28.635	1376	18675	22.009	30.6595	29.7075	75.5214	76.8613
DN	SP4_1	cluster_6	Common_2	877660	812811.731	811236	814302	910.0297	1.0798	0.1107	0.001	0.001	90889	1624561	78.36	58871	1048812	50.589	49166	877660	42.334	54.0946	54.0244	83.5148	83.6813
DN	SPI1_known1	cluster_5	Common_1_DN	26628	24666.973	24212	25077	250.1367	1.0795	0.1104	0.001	0.001	15535	109231	23.39	15325	106225	22.747	3826	26628	5.7021	24.6283	24.3777	24.9657	25.0675
DN	SOX1_3	cluster_6	Common_2	14602	13532.292	13265	13789	162.874	1.079	0.1098	0.001	0.0010909	3794	53161	70.646	1464	19722	26.209	1063	14602	19.405	28.0179	27.4675	72.6093	74.0391
DN	HMGA1_2	cluster_6	Common_2	29554	27391.457	27081	27693	187.4528	1.0789	0.1096	0.001	0.0010435	6789	95209	77.457	2777	36941	30.053	2183	29554	24.044	32.155	31.0412	78.61	80.0032
SP8	MESP1_1	cluster_4	SP4	12802	11867.706	11579	12200	168.542	1.0787	0.1093	0.001	0.001	28229	254858	48.512	2809	24683	4.6984	1430	12802	2.4368	5.0657	5.0232	50.9078	51.8657
DN	HIF1A::ARNT_1	cluster_6	Common_2	44470	41230.782	41012	41457	132.7718	1.0786	0.1091	0.001	0.001	12592	90566	80.589	7202	51412	45.748	6212	44470	39.571	49.3329	49.1023	86.2538	86.4973
DN	KLF12_1	cluster_6	Common_2	61251	56793.704	56505	57079	175.8431	1.0785	0.109	0.001	0.001	24570	147420	74.766	12826	76314	38.703	10243	61251	31.064	41.6891	41.5486	79.8612	80.2618
DN	FOXO4_3	cluster_6	Common_2	9951	9227.541	9059	9421	113.1499	1.0784	0.1089	0.001	0.0010909	2636	34308	73.038	1056	13081	27.848	789	9951	21.185	29.9317	29.0049	74.7159	76.0722
DN	ETS_known13	cluster_6	Common_2	96647	89636.313	89189	90110	277.7741	1.0782	0.1086	0.001	0.001	12306	197388	80.739	7079	111951	45.792	6053	96647	39.532	49.1874	48.963	85.5064	86.3297
DN	AHR::ARNT::HIF1A_1	cluster_6	Common_2	67387	62517.583	62240	62778	161.3843	1.0779	0.1082	0.001	0.0010435	14502	119460	81.912	9395	76813	52.669	8235	67387	46.206	56.7853	56.4097	87.653	87.7286
DN	SOX8_2	cluster_6	Common_2	27471	25489.81	25151	25841	208.9931	1.0777	0.108	0.001	0.0010909	6301	94659	74.032	2477	35522	27.781	1875	27471	21.485	29.7572	29.021	75.6964	77.3352

DN	SPIC_2	cluster_6	Common_2	82867	76897.085	76397	77456	326.841	1.0776	0.1079	0.001	0.0010435	21125	275317	74.226	8230	104958	28.297	6421	82867	22.341	30.3953	30.0988	78.0194	78.9525
DN	PATZ1_1	cluster_6	Common_2	522530	485109.865	483946	486345	712.2882	1.0771	0.1072	0.001	0.001	85759	1123464	76.273	49543	644744	43.772	40085	522530	35.475	46.7414	46.5106	80.9095	81.0446
DN	SOX10_4	cluster_6	Common_2	30242	28081.752	27708	28415	225.8079	1.0769	0.1069	0.001	0.0010909	6860	103161	73.858	2719	39147	28.027	2054	30242	21.652	29.9417	29.3153	75.5425	77.2524
DN	ETV6_1	cluster_5	Common_1_DN	103116	95755.758	95117	96381	370.9999	1.0769	0.1068	0.001	0.001	32656	469723	77.86	8947	124366	20.615	7297	103116	17.092	22.3451	21.9525	81.5581	82.9133
DN	SOX3_1	cluster_6	Common_2	11262	10458.66	10219	10695	149.4329	1.0768	0.1068	0.001	0.0010909	2538	40630	73.556	1001	15050	27.246	731	11262	20.389	28.8022	27.7184	73.027	74.8306
DN	SPIB_2	cluster_6	Common_2	94342	87610.019	87104	88152	317.222	1.0768	0.1068	0.001	0.0010435	23403	305650	75.803	9156	117165	29.058	7293	94342	23.398	31.1627	30.866	79.6527	80.5206
DN	ATF3_known4	cluster_6	Common_2	71386	66307.092	65941	66674	222.4489	1.0766	0.1065	0.001	0.001	10011	141182	80.298	5968	83194	47.317	5065	71386	40.601	50.5943	50.5631	84.8693	85.8067
DN	E2F7_1	cluster_6	Common_2	29479	27387.536	27138	27641	157.5887	1.0764	0.1062	0.001	0.0010435	5640	73401	81.261	2740	34586	38.289	2308	29479	32.636	40.922	40.1616	84.2336	85.2339
DN	E2F8_1	cluster_6	Common_2	41834	38898.519	38625	39155	168.609	1.0755	0.105	0.001	0.001	9501	104773	81.344	4519	48700	37.81	3838	41834	32.479	40.3957	39.9282	84.9303	85.9014
DN	SPIC_1	cluster_6	Common_2	75350	70060.737	69423	70559	330.7438	1.0755	0.105	0.001	0.001	19310	251988	71.563	7805	99240	28.184	5848	75350	21.399	30.2848	29.9022	74.9263	75.927
DN	ARID5A_1	cluster_6	Common_2	4107	3818.92	3707	3932	70.764	1.0754	0.1049	0.001	0.0012632	992	13600	80.227	410	5064	29.873	321	4107	24.227	32.3589	30.1985	78.2927	81.1019
DN	ETV6_2	cluster_6	Common_2	36810	34228.725	33733	34739	312.0934	1.0754	0.1049	0.001	0.0011429	10518	94973	25.655	14847	133594	36.088	4074	36810	9.9436	38.7336	38.7584	27.4399	27.5536
DN	SOX7_3	cluster_6	Common_2	19008	17679.503	17410	17965	176.6307	1.0751	0.1045	0.001	0.0010909	4157	66552	73.226	1672	25254	27.786	1228	19008	20.914	29.5405	28.5611	73.445	75.2673
DN	SOX10_9	cluster_6	Common_2	17297	16095.125	15838	16368	162.674	1.0747	0.1039	0.001	0.0010435	4222	59134	70.343	1761	23524	27.983	1267	17297	20.576	30.0095	29.2505	71.9478	73.5292
DN	FOXO6_1	cluster_6	Common_2	11589	10785.468	10569	11033	136.8089	1.0745	0.1037	0.001	0.0010909	3202	41765	72.845	1244	15377	26.82	922	11589	20.213	28.7945	27.7481	74.1158	75.3658
DN	ETV5_1	cluster_6	Common_2	22990	21397.037	21038	21697	200.2644	1.0744	0.1036	0.001	0.0010435	4257	38576	26.904	8751	79505	55.448	2535	22990	16.034	59.549	59.5966	28.9681	28.9164
SP8	PKNOX2_2	cluster_4	SP4	13099	12193.516	11848	12519	202.0986	1.0743	0.1033	0.001	0.0010435	10799	119886	49.272	2309	24959	10.258	1183	13099	5.3835	10.9547	10.9262	51.2343	52.4821
DN	CUX2_1	cluster_6	Common_2	2694	2508.702	2405	2631	69.044	1.0738	0.1028	0.001	0.0012632	444	7998	75.092	241	3664	34.401	163	2694	25.293	36.7117	33.6834	67.6349	73.5262
DN	HES7_1	cluster_6	Common_2	57959	53985.195	53686	54330	203.7303	1.0736	0.1025	0.001	0.0010435	12542	138583	78.225	6360	69516	39.239	5269	57959	32.716	42.0108	41.8226	82.8459	83.3751
DN	ERG_4	cluster_5	Common_1_DN	23767	22148.883	21848	22425	176.2723	1.0731	0.1017	0.001	0.001	8373	109062	76.033	2327	29418	20.509	1844	23767	16.569	22.0232	21.7922	79.2437	80.7907
DN	STAT_known3	cluster_6	Common_2	80462	74994.499	74518	75483	302.5419	1.0729	0.1015	0.001	0.0010435	8877	177940	82.488	4799	93340	43.27	4078	80462	37.3	45.9389	45.2186	84.976	86.2031
DN	HBP1_1	cluster_6	Common_2	24816	23132.181	22801	23461	205.2237	1.0728	0.1014	0.001	0.0010435	5153	79302	73.662	2217	32501	30.189	1651	24816	23.051	32.0396	31.293	74.47	76.3546
DN	SMAD4_1	cluster_6	Common_2	177020	165055.743	164292	165833	452.3312	1.0725	0.101	0.001	0.001	32547	462220	75.022	15994	222382	36.094	12567	177020	28.732	38.6119	38.2978	78.5732	79.6018
DN	STAT_known3	cluster_5	Common_1_DN	37070	34565.736	34162	34948	233.9292	1.0724	0.1009	0.001	0.0010435	8877	177940	82.488	2289	43138	19.998	1927	37070	17.185	21.7078	20.8329	84.1852	85.9335
DN	SPI1_known4	cluster_6	Common_2	76622	71501.522	71009	71956	292.6361	1.0716	0.0998	0.001	0.0010435	19284	251359	76.991	7415	94443	28.928	5948	76622	23.469	30.8442	30.4831	80.2158	81.1304
DN	SPZ1_1	cluster_6	Common_2	401563	374796.938	373829	375874	613.1807	1.0714	0.0995	0.001	0.001	58732	857887	77.786	33952	488441	44.288	27675	401563	36.41	47.1208	46.8084	81.5121	82.2132
SP4	HESX1_2	cluster_1	SP8	1499	1399.118	1306	1485	55.491	1.0713	0.0994	0.031	0.032348	906	12691	63.767	177	2307	11.592	110	1499	7.5319	12.1413	11.8115	62.1469	64.9762
DN	EWSR1::FLI1_1	cluster_5	Common_1_DN	260450	243120.733	241846	244471	812.4946	1.0713	0.0993	0.001	0.0010435	56590	1127364	69.149	19564	364049	22.329	13615	260450	15.975	24.059	23.1026	69.5921	71.5426
DN	SOX8_9	cluster_6	Common_2	15523	14490.095	14228	14773	164.7181	1.0713	0.0993	0.001	0.0010909	3303	52872	68.642	1457	22004	28.567	1000	15523	20.153	30.2755	29.3596	68.6342	70.5463
DN	ETV2_1	cluster_5	Common_1_DN	15311	14297.603	13917	14731	232.84	1.0709	0.0988	0.001	0.0010435	7018	70432	23.896	6080	60068	20.38	1543	15311	5.1946	21.9863	21.7387	25.3783	25.4894
DN	E2F_known26	cluster_6	Common_2	13972	13048.839	12853	13253	122.6907	1.0707	0.0986	0.001	0.0010435	2046	34813	80.496	1040	16856	38.975	841	13972	32.307	41.1046	40.1344	80.8654	82.8904
DN	E2F2_3	cluster_6	Common_2	18539	17316.491	17081	17541	138.8755	1.0706	0.0984	0.001	0.001	2541	43236	81.281	1330	21927	41.222	1105	18539	34.852	43.4868	42.8786	83.0827	84.5487
DN	TBX1_4	cluster_6	Common_2	65749	61412.169	60840	62094	367.9927	1.0706	0.0984	0.001	0.0010909	11107	211898	72.371	4722	86734	29.623	3503	65749	22.456	31.5387	31.0286	74.1847	75.8053
DN	SP1_known2	cluster_6	Common_2	973497	909393.102	908186	910915	857.4672	1.0705	0.0983	0.001	0.001	131181	1808239	79.788	82234	1146633	50.595	70265	973497	42.955	53.5634	53.8367	85.4452	84.9005
DN	ATF4_3	cluster_6	Common_2	19918	18607.345	18349	18841	162.0299	1.0704	0.0982	0.001	0.0010909	4863	63288	72.723	2058	25990	29.865	1552	19918	22.887	31.9145	31.472	75.413	76.6372
DN	LMO2_2	cluster_6	Common_2	12893	12044.164	11876	12215	101.6731	1.0705	0.0982	0.001	0.0010435	4777	38246	75.086	2056	16178	31.761	1622	12893	25.312	33.9544	33.7107	78.8911	79.6946
DN	ERG_2	cluster_5	Common_1_DN	23299	21769.241	21457	22066	191.9059	1.0703	0.098	0.001	0.001	8341	108611	75.068	2323	29323	20.267	1812	23299	16.103	21.724	21.4518	78.0026	79.4564
DN	TBX5_5	cluster_6	Common_2	43439	40586.325	40096	41065	293.5932	1.0703	0.098	0.001	0.0010435	7574	144355	74.742	3074	55701	28.84	2335	43439	22.491	30.8292	30.0918	75.9597	77.986
DN	RUNX1_2	cluster_6	Common_2	22967	21460.95	21165	21799	193.675	1.0702	0.0978	0.001	0.0010435	5376	83982	74.445	2063	30260	26.824	1537	22967	20.359	28.59	27.3475	74.5032	75.8989
DN	SPDEF_6	cluster_5	Common_1_DN	30968	28940.84	28702	29204	156.2201	1.07	0.0977	0.001	0.001	10392	145946	84.321	2581	34933	20.183	2266	30968	17.892	21.8052	21.2188	87.7954	88.6497
DN	EGR1_known9	cluster_6	Common_2	245600	229566.318	228931	230203	369.9161	1.0698	0.0974	0.001	0.001	32718	444077	82.227	21069	282472	52.303	18242	245600	45.476	55.7552	55.3057	86.5822	86.9467
DN	FOXP1_1	cluster_6	Common_2	72065	67358.116	66528	68331	554.0786	1.0699	0.0974	0.001	0.0010435	10678	252594	65.084	6055	120673	31.093	3444	72065	18.568	32.2532	28.53	56.8786	59.7192
DN	MYCN_2	cluster_6	Common_2	91114	85176.397	84844	85506	206.9441	1.0697	0.0972	0.001	0.001	21157	191106	79.065	12049	108167	44.751	10100	91114	37.696	47.7383	47.6772	83.8244	84.2346
DN	SOX9_4	cluster_6	Common_2	27510	25720.173	25307	26072	206.713	1.0696	0.0971	0.001	0.0010909	6382	95858	74.138	2495	35804	27.691	1876	27510	21.277	29.3952	28.6987	75.1904	76.835
DN	ELK4_1	cluster_6	Common_2	55453	51857.586	51664	52046	118.6943	1.0693	0.0967	0.001	0.001	11601	93425	84.428	7657	61524	55.599	6883	55453	50.113	59.3311	59.3556	89.8916	90.1323
DN	MGA_3	cluster_6	Common_2	35485	33183.481	32800	33535	224.064	1.0694	0.0967	0.001	0.0010909	6415	109267	77.12	2681	44055	31.094	2121	35485	25.045	33.0631	32.4755	79.1123	80.547
DN	HES1_1	cluster_6	Common_2	196628	183983.176	183194	184773	455.5556	1.0687	0.0959	0.001	0.001	30958	438366	78.548	16910	236209	42.325	13961	196628	35.233	45.0966	44.8548	82.5606	83.2432
DN	E2F2_2	cluster_6	Common_2	24932	23329.785	23045	23601	171.1291	1.0687	0.0958	0.001	0.0010909	3788	64579	78.928	1874	30764	37.6	1495	24932	30.472	39.4667	38.607	79.7759	81.0428
SP8	PKNOX1_2	cluster_4	SP4	11735	10980.918	10645	11285	194.0079	1.0687	0.0958	0.001	0.0010435	9433	104620	49.524	2075	22398	10.602	1062	11735	5.555	11.2583	11.2168	51.1807	52.3931
DN	NFATC2_1	cluster_6	Common_2	15433	14442.231	14290	14582	85.4812	1.0686	0.0957	0.001	0.0010909	7464	44784	80.238	3099	18285	32.761	2601	15433	27.651	34.8473	34.461	83.9303	84.4025
DN	ARID5B_1	cluster_6	Common_2	23824	22295.942	22053	22546	164.5038	1.0685	0.0956	0.001	0.0010909	5946	77508	76.405	2368	29918	29.492	1862	23824	23.485	31.3152	30.7375	78.6318	79.631
DN	EGR4_1	cluster_5	Common_1_DN	74965	70157.688	69621	70583	297.5653	1.0685	0.0956	0.001	0.001	34373	389734	77.181	8378	92071	18.233	6744	74965	14.846	19.6201	19.2349	80.4965	81.4209
DN	SOX7_4	cluster_6	Common_2	14799	13850.233	13588	14110	160.801	1.0685	0.0956	0.001	0.0010909	3341	53567	71.584	1326	20183	26.971	949	14799	19.777	28.4047	27.6271	71.5686	73.3241
DN	MAF_known12	cluster_6	Common_2	57529	53852.703	53376	54299	279.0399	1.0683	0.0953	0.001	0.0010435	9989	170271	77.196	4314	71119	32.243	3429	57529	26.082	34.3278	33.7867	79.4854	80.8912
SP4	NFATC1_3	cluster_1	SP8	4126	3863.119	3687	4053	105.2779	1.068	0.095	0.007	0.0073043	3149	40963	60.391	541	6612	9.7479	328	4126	6.0829	10.416	10.0725	60.6285	62.4017
DN	SP1_known3	cluster_6	Common_2	280350	262513.75	261779	263359	469.0207	1.0679	0.0948	0.001	0.001	47992	629173	78.933	26174	337202	42.304	21539	280350	35.171	44.8804	44.5585	82.2916	83.1401
DN	HES5_1	cluster_6	Common_2	54030	50604.759	50273	50938	201.2574	1.0677	0.0945	0.001	0.0010435	12165	134258	77.001	6066	66167	37.949	4918	54030	30.988	40.4275	40.2434	81.0748	81.657
SP4	SPI1_known4	cluster_1	SP8	19131	17919.746	17522	18285	226.4469	1.0676	0.0944	0.001	0.0010435	15528	202516	62.03	2317	29252	8.9598	1488	19131	5.8598	9.5827	9.4467	64.221	65.4007
DN	TBX19_1	cluster_6	Common_2	27625	25876.363	25419	26283	259.7267	1.0676	0.0943	0.001	0.0010435	4779	91001	71.181	2041	37143	29.053	1477	27625	21.608	30.906	30.3568	72.3665	74.3747
DN	SOX14_2	cluster_6	Common_2	13830	12957.336	12738	13180	137.0739	1.0673	0.094	0.001	0.0010909	4224	46656	72.886	1716	18299	28.587	1279	13830	21.605	30.2794	29.6425	74.5338	75.5779
DN	ATF4_2	cluster_6	Common_2	19417	18193.67	17935	18489	156.19	1.0672	0.0939	0.001	0.0010909	4963	59634	73.418	2144	25111	30.915	1632	19417	23.905	32.8833	32.5603	76.1194	77.3247
DN	MAFF_1	cluster_6	Common_2	54985	51520.105	51042	51974	294.0915	1.0673	0.0939	0.001	0.001	12562	176219	70.456	5394	73858	29.53	3946	54985	21.984	31.4122	31.2027	73.1554	74.4469
DN	FLI1_4	cluster_5	Common_1_DN	23618	22136.593	21837	22437	182.2342	1.0669	0.0934	0.001	0.001	8592	111995	76.37	2313	29272	19.961	1832	23618	16.105	21.3222	21.0884	79.2045	80.6846
DN	IKZF2_3	cluster_6	Common_2	22853	21430.138	21205	21659	142.5977	1.0664	0.0927	0.001	0.0010909	7087	71094	80.484	2881	27614	31.261	2351	22853	25.871	33.1734	32.1448	81.6036	82.7587
DN	EGR1_known9	cluster_5	Common_1_DN	80975	75941.885	75481	76430	284.4876	1.0663	0.0926	0.001	0.001	32718	444077	82.227	7106	93622	17.335	6091	80975	14.994	18.6167	18.2345	85.7163	86.4914
DN	E2F_known24	cluster_6	Common_2	25723	24126.237	23808	24490	194.04	1.0662	0.0925	0.001	0.001	3603	61420	79.657	1910	31448	40.785	1540	25723	33.361	42.7422	41.8805	80.6283	81.7953
DN	STAT_known2	cluster_6	Common_2	104848	98400.996	97840	98973	330.8707	1.0655	0.0916	0.001	0.001	11028	221310	83.913	6134	120009	45.503	5290	104848	39.755	47.9688	47.3761	86.2406	87.3668
DN	EGR1_known12	cluster_5	Common_1_DN	180737	169628.994	168731	170489	518.1065	1.0655	0.0915	0.001	0.001	57885	958377	76.536	14575	227849	18.196	11329	180737	14.434	19.5716	18.8587	77.729	79.3231
DN	FOXO1_4	cluster_6	Common_2	10141	9518.723	9324	9751	127.417	1.0654	0.0914	0.001	0.0010909	2797	36450	73.199	1097	13493	27.097	810	10141	20.365	28.9596	27.8217	73.8377	75.1575
DN	KLF12_2	cluster_5	Common_1_DN	138616	130186.786	129399	130836	430.1506	1.0647	0.0905	0.001	0.001	46999	672450	77.85	12334	169624	19.637	9914	138616	16.048	21.0941	20.6136	80.3794	81.7196
DN	SOX14_3	cluster_6	Common_2	15907	14942.129	14692	15207	152.2259	1.0646	0.0903	0.001	0.0010909	4092	57347	70.134	1626	21909	26.794	1160	15907	19.454	28.348	27.7382	71.3407	72.6049
DN	SMAD3_3	cluster_6	Common_2	14791	13894.177	13729	14074	112.7963	1.0645	0.0902	0.001	0.0010435	4812	43323	75.24	2115	18720	32.511	1664	14791	25.688	34.5802	34.1412	78.6761	79.0118
DN	SOX30_1	cluster_6	Common_2	19918	18711.34	18434	18996	173.3039	1.0645	0.0902	0.001	0.0010435	4394	65950	74.266	1811	26029	29.311	1364	19918	22.43	31.0423	30.2017	75.3175	76.5223
DN	MAF_known10	cluster_6	Common_2	35574	33420.543	33100	33767	201.6211	1.0644	0.0901	0.001	0.0010909	11107	122400	74.849	4229	45435	27.784	3276	35574	21.754	29.4949	29.0637	77.4651	78.2965
DN	NFATC1_1	cluster_6	Common_2	42331	39771.482	39366	40188	254.3859	1.0644	0.09	0.001	0.0010909	6650	128410	79.943	2869	52065	32.414	2276	42331	26.354	34.2256	32.9655	79.3308	81.3041
DN	TBX2_2	cluster_6	Common_2	35502	33356.753	33075	33653	185.154	1.0643	0.0899	0.001	0.0010909	11786	118013	74.082	4675	45779	28.737	3586	35502	22.286	30.4259	30.0831	76.7059	77.5508
DN	MTF1_3	cluster_6	Common_2	80933	76087.238	75613	76551	275.7794	1.0637	0.0891	0.001	0.001	16298	213937	79.022	7551	97546	36.03	6213	80933	29.894	38.1212	37.8303	82.2805	82.9691
DN	SMAD_1	cluster_6	Common_2	56394	53016.472	52697	53350	199.6055	1.0637	0.0891	0.001	0.0010435	20794	166413	76.255	8902	70228	32.18	7092	56394	25.841	34.106	33.888	79.6675	80.3013
DN	SOX9_6	cluster_6	Common_2	22316	20981.42	20645	21291	196.0496	1.0636	0.089	0.001	0.0010909	5272	79251	71.777	2124	30252	27.399	1540	22316	20.211	29.2109	28.1586	72.5047	73.767
DN	SOX21_2	cluster_6	Common_2	19123	17981.021	17728	18248	165.4887	1.0635	0.0888	0.001	0.0010909	4666	65357	73.59	1889	25323	28.513	1402	19123	21.532	30.0471	29.2593	74.2192	75.5163
DN	TBX4_2	cluster_6	Common_2	52470	49337.624	48801	49806	282.922	1.0635	0.0888	0.001	0.0010909	8920	170122	76.154	3636	66282	29.671	2809	52470	23.488	31.491	30.8426	77.2552	79.1618
DN	KLF12_1	cluster_5	Common_1_DN	34542	32493.722	32254	32715	138.5514	1.063	0.0882	0.001	0.001	24570	147420	74.766	7375	43670	22.148	5795	34542	17.518	23.5857	23.431	78.5763	79.0978
DN	SOX15_2	cluster_6	Common_2	14843	13964.314	13728	14196	148.1478	1.0629	0.088	0.001	0.0010909	3740	52487	73.258	1502	19922	27.806	1100	14843	20.717	29.4118	28.2794	73.2357	74.5056
DN	TBX2_1	cluster_6	Common_2	63521	59760.534	59264	60239	298.0215	1.0629	0.088	0.001	0.0010435	11067	188839	77.429	4773	78671	32.257	3782	63521	26.045	34.1737	33.6376	79.2374	80.7426
SP4	PKNOX1_1	cluster_1	SP8	14203	13368.535	13023	13781	219.367	1.0624	0.0873	0.001	0.0010435	11588	174615	56.273	1682	24195	7.7972	954	14203	4.5772	8.2327	8.1339	56.7182	58.7022
SP8	POU2F2_known14	cluster_4	SP4	1642	1545.547	1430	1664	69.592	1.0624	0.0873	0.074	0.077217	928	14244	63.304	195	2647	11.764	116	1642	7.2975	12.5	11.5277	59.4872	62.0325
DN	SPIB_1	cluster_6	Common_2	38110	35891.199	35628	36145	156.5586	1.0618	0.0865	0.001	0.0010435	22113	132678	71.137	8514	50762	27.217	6386	38110	20.433	28.8789	28.7237	75.0059	75.0758
DN	RUNX2_5	cluster_6	Common_2	170930	161033.712	160366	161711	402.0254	1.0615	0.086	0.001	0.001	23276	408017	80.689	11640	201282	39.805	9771	170930	33.803	41.9789	41.8929	83.9433	84.9207
DN	STAT_known2	cluster_5	Common_1_DN	47217	44483.666	44104	44909	251.2925	1.0614	0.086	0.001	0.001	11028	221310	83.913	2866	54457	20.648	2448	47217	17.903	22.198	21.3352	85.4152	86.7051
DN	MYBL1_3	cluster_6	Common_2	7400	6973.472	6837	7097	80.8928	1.0612	0.0856	0.001	0.0010909	1868	20588	77.661	844	9130	34.44	679	7400	27.914	36.349	35.9433	80.4502	81.0515
DN	SP100_1	cluster_5	Common_1_DN	2579	2431.397	2356	2507	48.5297	1.0607	0.085	0.004	0.0050526	1107	14404	85.672	242	2948	17.534	209	2579	15.339	18.8799	17.9047	86.3636	87.483
DN	RUNX3_3	cluster_6	Common_2	237865	224370.932	223596	225070	456.5822	1.0601	0.0843	0.001	0.001	29706	527830	81.113	15867	278975	42.871	13398	237865	36.554	45.102	45.0647	84.4394	85.2639
DN	SOX4_2	cluster_6	Common_2	24531	23146.141	22862	23442	190.5027	1.0598	0.0838	0.001	0.0010909	5733	86204	75.057	2240	32027	27.886	1683	24531	21.359	29.3564	28.4569	75.1339	76.5947
SP8	SOX18_4	cluster_4	SP4	4884	4608.951	4375	4823	125.5069	1.0597	0.0836	0.011	0.012	2159	30301	57.531	642	8375	15.901	362	4884	9.273	16.767	16.1183	56.3863	58.3164
SP8	PKNOX2_3	cluster_4	SP4	12634	11929.807	11617	12255	189.1017	1.059	0.0827	0.001	0.0010435	9661	107482	50.165	2218	23981	11.193	1141	12634	5.8967	11.8104	11.7545	51.4427	52.6834
DN	SOX18_2	cluster_6	Common_2	13400	12656.42	12402	12878	142.3512	1.0587	0.0824	0.001	0.0010909	3405	47699	72.637	1357	18124	27.599	988	13400	20.406	29.0162	28.0928	72.8077	73.9351
DN	SPZ1_1	cluster_5	Common_1_DN	177180	167391.335	166477	168167	513.1526	1.0585	0.082	0.001	0.001	58732	857887	77.786	15701	219487	19.901	12464	177180	16.065	21.2218	20.6531	79.3835	80.7246
SP4	SPIB_2	cluster_1	SP8	22725	21483.446	21092	21889	240.5676	1.0578	0.0811	0.001	0.0010435	18724	244697	60.687	2806	35711	8.8566	1753	22725	5.636	9.3623	9.287	62.4733	63.6359
DN	MAF_known11	cluster_6	Common_2	72934	68982.968	68421	69612	350.5293	1.0573	0.0804	0.001	0.0010435	13743	221582	72.337	6133	96460	31.49	4563	72934	23.81	33.2024	32.9151	74.4008	75.6106
DN	MYBL2_2	cluster_6	Common_2	16939	16021.365	15808	16257	137.0705	1.0573	0.0803	0.001	0.0010435	2843	43039	79.915	1404	20538	38.135	1140	16939	31.452	40.0985	39.3573	81.1966	82.4764
DN	IKZF1_1	cluster_6	Common_2	53676	50773.835	50473	51089	193.4655	1.0572	0.0802	0.001	0.0010435	11933	143526	82.189	5426	63254	36.222	4542	53676	30.737	38.0625	37.3981	83.7081	84.8579
DN	MAF_known1	cluster_6	Common_2	161128	152411.578	151571	153281	493.3713	1.0572	0.0802	0.001	0.001	33530	506311	73.776	14285	209608	30.543	10822	161128	23.478	32.2756	31.8239	75.7578	76.8711
DN	PBX3_known1	cluster_6	Common_2	33342	31545.086	31230	31867	190.8578	1.057	0.0799	0.001	0.0010435	8906	98882	73.528	4029	43510	32.354	3035	33342	24.793	34.0781	33.719	75.3289	76.6307
DN	IKZF3_1	cluster_6	Common_2	38413	36347.917	36032	36630	179.8714	1.0568	0.0797	0.001	0.0010435	8816	106058	78.616	4061	47307	35.067	3257	38413	28.474	36.9442	36.2189	80.2019	81.1994
DN	SPZ1_2	cluster_6	Common_2	62373	59021.6	58610	59427	252.8158	1.0568	0.0797	0.001	0.0010435	18565	186152	75.582	8044	79031	32.088	6284	62373	25.325	33.8486	33.5065	78.1203	78.9222
DN	MYBL2_4	cluster_6	Common_2	11248	10644.366	10474	10812	107.5835	1.0567	0.0796	0.001	0.0010435	1996	27994	79.043	990	13637	38.505	807	11248	31.76	40.4309	40.18	81.5152	82.4815
DN	MAFG_1	cluster_6	Common_2	49232	46599.395	46136	47158	323.3519	1.0565	0.0793	0.001	0.0010435	8246	165248	72.352	3395	65678	28.757	2492	49232	21.556	30.2207	29.7928	73.4021	74.9597
DN	FOXM1_1	cluster_6	Common_2	16963	16066.073	15908	16240	99.3255	1.0558	0.0784	0.001	0.0010909	6570	52629	74.145	2799	21955	30.931	2143	16963	23.898	32.618	32.2313	76.5631	77.2626
DN	MYC_known20	cluster_5	Common_1_DN	43017	40750.585	40285	41186	268.3456	1.0556	0.0781	0.001	0.001	11316	185626	73.632	3536	55966	22.2	2655	43017	17.063	23.4624	23.174	75.0848	76.8627
DN	MTF1_1	cluster_6	Common_2	287498	272464.627	271724	273233	438.288	1.0552	0.0775	0.001	0.001	48686	649383	82.303	25336	334415	42.384	21663	287498	36.438	44.4953	44.2725	85.5028	85.9704
DN	CBX5_1	cluster_6	Common_2	17135	16239.556	15994	16459	135.5642	1.0551	0.0774	0.001	0.0010435	5190	57165	75.199	2111	22302	29.338	1600	17135	22.541	30.8285	29.9746	75.7935	76.8317
DN	TCF21_1	cluster_5	Common_1_DN	57306	54312.988	53893	54772	272.2085	1.0551	0.0774	0.001	0.001	16797	218881	77.73	5599	70697	25.106	4466	57306	20.351	26.5881	26.1813	79.7642	81.0586
SP4	SPI1_known1	cluster_1	SP8	22759	21579.796	21248	21894	194.7248	1.0546	0.0768	0.001	0.001	38052	267768	57.339	5460	37816	8.0978	3256	22759	4.8736	8.5567	8.4995	59.6337	60.1835
DN	MYBL1_5	cluster_6	Common_2	23254	22050.259	21841	22276	129.4446	1.0546	0.0767	0.001	0.0010435	3552	57284	82.002	1754	27428	39.263	1462	23254	33.288	41.1599	40.5942	83.3523	84.782
DN	TBX21_6	cluster_6	Common_2	56067	53192.957	52672	53664	303.5318	1.054	0.0759	0.001	0.0010909	10503	189588	74.498	4211	73113	28.73	3165	56067	22.031	30.1342	29.5731	75.1603	76.6854
DN	FLI1_2	cluster_5	Common_1_DN	21977	20859.737	20587	21171	179.1192	1.0536	0.0753	0.001	0.001	8020	104443	74.293	2240	28311	20.138	1704	21977	15.633	21.2469	21.0421	76.0714	77.6271
DN	MYC_known13	cluster_6	Common_2	45945	43617.249	43231	43978	217.2582	1.0534	0.075	0.001	0.0010909	12241	134783	75.676	5376	58122	32.633	4204	45945	25.796	34.3436	34.0881	78.1994	79.0492
DN	RUNX3_1	cluster_6	Common_2	150730	143095.063	142497	143632	347.8763	1.0534	0.075	0.001	0.0010435	25951	398073	82.383	11621	175566	36.334	9882	150730	31.194	38.0795	37.8649	85.0357	85.8538
SP4	SPIC_1	cluster_1	SP8	19684	18686.671	18302	19049	230.015	1.0534	0.075	0.001	0.001	16203	211420	60.042	2474	31470	8.9373	1525	19684	5.5901	9.4118	9.3104	61.6411	62.5485
SP4	NFE2L1::MAFG_1	cluster_1	SP8	16781	15932.523	15470	16405	296.172	1.0533	0.0748	0.001	0.0010435	8781	185151	56.968	1453	28629	8.8086	815	16781	5.1632	9.2814	9.0634	56.0908	58.6154
DN	NFE2L1::MAFG_1	cluster_6	Common_2	67869	64446.397	63867	65064	371.9695	1.0531	0.0747	0.001	0.0010435	11526	243206	74.83	4408	88615	27.265	3288	67869	20.882	28.5268	27.906	74.5917	76.5886
DN	SMAD3_1	cluster_6	Common_2	67422	64028.473	63691	64394	222.7654	1.053	0.0745	0.001	0.0010435	26078	216611	73.645	10800	88038	29.932	8242	67422	22.923	31.6052	31.1258	76.3148	76.5828
DN	TCF4_2	cluster_5	Common_1_DN	107458	102047.099	101568	102560	312.6078	1.053	0.0745	0.001	0.001	45335	409696	76.215	15187	134709	25.06	11997	107458	19.99	26.463	26.2287	78.9952	79.7705
DN	BPTF_1	cluster_6	Common_2	62003	58899.903	58414	59369	292.8415	1.0527	0.0741	0.001	0.0010909	15844	211822	75.51	6263	80341	28.64	4768	62003	22.103	30.0934	29.2713	76.1297	77.1748
DN	FOXO6_3	cluster_6	Common_2	127540	121154.614	120553	121755	364.6038	1.0527	0.0741	0.001	0.0010435	28591	373838	76.349	12666	161340	32.951	9872	127540	26.048	34.5283	34.1164	77.9409	79.0505

SP4	ETV7_1	cluster_1	SP8	20634	19606.386	19306	19933	182.6942	1.0524	0.0737	0.001	0.001	27857	251236	56.588	3931	34860	7.8518	2298	20634	4.6475	8.2493	8.213	58.4584	59.191
SP4	TBX5_3	cluster_1	SP8	12147	11541.93	11302	11812	151.3098	1.0524	0.0737	0.001	0.001	15391	138670	55.98	2358	20762	8.3814	1363	12147	4.9036	8.8558	8.7596	57.8032	58.5059
SP4	SPIC_2	cluster_1	SP8	20979	19936.128	19569	20304	223.1985	1.0523	0.0736	0.001	0.0010435	17268	225108	60.689	2615	33138	8.934	1627	20979	5.656	9.4221	9.3195	62.218	63.308
DN	PATZ1_1	cluster_5	Common_1_DN	225904	214690.649	213808	215560	567.263	1.0522	0.0735	0.001	0.001	85759	1123464	76.273	23069	288137	19.562	17878	225904	15.337	20.8468	20.1078	77.4979	78.4016
DN	SOX1_2	cluster_6	Common_2	16294	15496.073	15259	15800	159.59	1.0515	0.0724	0.001	0.0010909	4151	58133	72.545	1650	22104	27.584	1196	16294	20.333	28.8123	28.0288	72.4848	73.7152
DN	FOXO1_5	cluster_6	Common_2	118896	113115.25	112509	113610	347.4308	1.0511	0.0719	0.001	0.0010435	32721	361528	75.797	13987	151415	31.745	10870	118896	24.927	33.2203	32.8871	77.715	78.5233
DN	TBX20_2	cluster_6	Common_2	39578	37657.559	37218	38199	287.3756	1.051	0.0718	0.001	0.0010435	7160	129186	74.304	2975	51847	29.821	2225	39578	22.764	31.0754	30.6364	74.7899	76.3361
DN	TBX20_5	cluster_6	Common_2	75334	71680.142	71129	72170	319.8559	1.051	0.0717	0.001	0.0010909	16570	253871	75.218	6610	97846	28.99	5013	75334	22.32	30.2535	29.6741	75.8396	76.9924
DN	EGR4_2	cluster_5	Common_1_DN	164953	156979.393	156280	157719	453.2512	1.0508	0.0715	0.001	0.001	55529	908524	79.001	13078	203425	17.689	10462	164953	14.344	18.8406	18.1562	79.9969	81.0879
DN	GATA_known16	cluster_6	Common_2	33094	31500.186	30997	31977	294.9418	1.0506	0.0712	0.001	0.0010435	6076	128052	74.197	2227	44036	25.516	1622	33094	19.176	26.6952	25.8442	72.8334	75.1521
DN	TBX5_3	cluster_6	Common_2	54128	51527.153	51164	51910	222.8892	1.0505	0.071	0.001	0.001	20740	186864	75.435	7844	69272	27.965	6066	54128	21.851	29.2478	28.9665	77.333	78.1384
DN	TBX4_1	cluster_6	Common_2	33067	31483.302	31207	31727	149.9421	1.0503	0.0708	0.001	0.0010435	16022	112186	74.769	6158	42515	28.335	4756	33067	22.038	29.6842	29.4752	77.2329	77.7773
DN	RUNX_2	cluster_5	Common_1_DN	105280	100258.77	99598	100846	381.2158	1.0501	0.0705	0.001	0.0010435	28180	397366	76.459	9707	132568	25.508	7583	105280	20.257	26.9092	26.4945	78.1189	79.4158
SP4	MAFF_1	cluster_1	SP8	11502	10963.456	10645	11267	183.7475	1.0491	0.0692	0.001	0.001	9927	139260	55.679	1463	19885	7.9504	827	11502	4.5987	8.3308	8.2594	56.5277	57.8426
DN	RUNX2_4	cluster_6	Common_2	131382	125241.657	124677	125761	334.1979	1.049	0.0691	0.001	0.0010435	22375	342168	83.028	10116	152450	36.993	8643	131382	31.88	38.6279	38.3969	85.4389	86.1804
SP4	TBX1_4	cluster_1	SP8	17000	16205.32	15772	16625	264.4627	1.049	0.0691	0.003	0.0031304	8929	170354	58.182	1543	28290	9.6621	903	17000	5.8061	10.1131	9.9792	58.5224	60.0919
DN	RBPJ_2	cluster_6	Common_2	44948	42855.794	42533	43149	186.803	1.0488	0.0688	0.001	0.0010909	13859	138744	77.101	5759	56386	31.334	4545	44948	24.978	32.7946	32.3964	78.92	79.7148
DN	IKZF1_2	cluster_6	Common_2	75194	71704.906	71312	72042	220.3915	1.0487	0.0685	0.001	0.0010435	27213	217711	79.439	11645	91533	33.399	9493	75194	27.437	34.8841	34.5384	81.52	82.1496
SP4	SPIB_1	cluster_1	SP8	9931	9471.102	9282	9659	117.0977	1.0486	0.0684	0.001	0.0010435	18137	108822	58.346	2742	16291	8.7346	1663	9931	5.3246	9.1691	9.1259	60.6492	60.96
DN	TBX15_2	cluster_6	Common_2	42464	40499.525	40236	40762	164.8571	1.0485	0.0683	0.001	0.0010909	20505	143570	74.719	7932	54774	28.506	6110	42464	22.1	29.7976	29.5772	77.0298	77.5258
DN	SOX15_4	cluster_6	Common_2	9810	9359.529	9129	9577	131.9926	1.0481	0.0678	0.001	0.0011429	2731	38267	69.358	1053	13999	25.373	720	9810	17.78	26.364	25.6357	68.3761	70.0764
DN	TBX21_4	cluster_6	Common_2	41352	39459.681	39014	39891	257.3335	1.048	0.0676	0.001	0.0010909	9894	148765	73.27	3809	55074	27.125	2807	41352	20.367	28.3707	27.7969	73.6939	75.0844
DN	HIF1A::ARNT_1	cluster_5	Common_1_DN	19234	18355.081	18201	18539	100.4244	1.0479	0.0675	0.001	0.001	12592	90566	80.589	3229	22887	20.366	2693	19234	17.115	21.3866	21.2376	83.4004	84.039
DN	HESX1_2	cluster_6	Common_2	4827	4606.497	4488	4734	76.2218	1.0479	0.0674	0.007	0.0093333	1143	16019	80.489	451	5980	30.047	356	4827	24.254	31.1461	30.133	78.9357	80.7191
DN	PKNOX1_1	cluster_6	Common_2	68262	65148.446	64608	65676	328.1873	1.0478	0.0674	0.001	0.0010435	15318	230880	74.405	6051	88894	28.648	4581	68262	21.999	29.906	29.566	75.7065	76.7903
DN	SMAD_2	cluster_6	Common_2	88389	84362.216	83847	84826	294.9888	1.0477	0.0673	0.001	0.0010435	28215	291809	73.323	11532	116795	29.347	8679	88389	22.209	30.7602	30.29	75.2601	75.6788
DN	RUNX2_4	cluster_5	Common_1_DN	76958	73460.065	72957	73886	270.8222	1.0476	0.0671	0.001	0.0010435	22375	342168	83.028	6052	89641	21.752	5126	76958	18.674	22.9095	22.4913	84.6993	85.8513
SP4	TBX4_1	cluster_1	SP8	8090	7726.409	7548	7909	112.2549	1.0471	0.0663	0.001	0.0010435	11905	83358	55.556	2026	13954	9.2999	1164	8090	5.3918	9.7774	9.7051	57.4531	57.9762
DN	POU2F2_known16	cluster_6	Common_2	11223	10722.271	10485	10962	145.5631	1.0467	0.0658	0.001	0.0010909	3024	39849	67.778	1305	16413	27.917	880	11223	19.089	29.1005	28.1638	67.433	68.3787
DN	FOXP3_1	cluster_6	Common_2	45339	43326.931	42871	43743	271.5942	1.0464	0.0655	0.001	0.0010909	9966	159936	72.213	3984	61409	27.727	2881	45339	20.471	28.9083	28.3482	72.3143	73.8312
DN	RUNX3_1	cluster_5	Common_1_DN	90358	86423.101	85923	86928	316.3774	1.0455	0.0642	0.001	0.0010435	25951	398073	82.383	7152	106249	21.989	6002	90358	18.7	23.1282	22.6989	83.9206	85.0436
DN	TCF12_known1	cluster_5	Common_1_DN	90154	86256.72	85891	86611	223.4792	1.0452	0.0638	0.001	0.0010909	48943	352576	75.728	16034	114471	24.587	12565	90154	19.364	25.6727	25.5701	78.3647	78.7571
SP8	ETV6_2	cluster_4	SP4	9463	9054.785	8732	9353	194.4224	1.0451	0.0636	0.015	0.016364	9151	82658	22.329	4569	40741	11.006	1053	9463	2.5563	11.5069	11.4484	23.0466	23.2272
DN	SP100_1	cluster_6	Common_2	6776	6485.178	6391	6585	58.181	1.0448	0.0633	0.001	0.0013333	1107	14404	85.672	620	7821	46.518	531	6776	40.302	47.9675	47.0425	85.6452	86.6385
DN	RREB1_1	cluster_6	Common_2	326327	312447.808	311432	313509	623.6571	1.0444	0.0627	0.001	0.001	60217	1008607	79.968	26028	411023	32.588	20497	326327	25.873	34.0386	32.3542	78.7498	79.3939
DN	TCF12_known1	cluster_6	Common_2	111808	107077.766	106659	107522	269.8082	1.0442	0.0624	0.001	0.0010909	48943	352576	75.728	19920	142178	30.538	15580	111808	24.015	31.8329	31.7117	78.2129	78.6395
SP4	RUNX1_2	cluster_1	SP8	7926	7590.917	7299	7835	158.447	1.0441	0.0623	0.007	0.007	4574	71425	63.314	859	12459	11.044	527	7926	7.0259	11.5216	11.097	61.3504	63.6167
DN	MYCN_1	cluster_5	Common_1_DN	29226	27993.006	27675	28290	185.5596	1.044	0.0622	0.001	0.001	12503	137921	76.685	3425	36786	20.453	2685	29226	16.25	21.4748	21.1904	78.3942	79.4487
DN	RUNX1_4	cluster_6	Common_2	9156	8775.296	8612	8915	94.6076	1.0434	0.0613	0.001	0.0010909	3683	37261	73.637	1246	12216	24.142	927	9156	18.095	25.1697	24.5726	74.3981	74.9509
DN	HES1_1	cluster_5	Common_1_DN	100037	95898.92	95343	96497	360.2915	1.0431	0.0609	0.001	0.001	30958	438366	78.548	9001	123465	22.123	7175	100037	17.925	23.1766	22.8204	79.7134	81.0246
DN	ETS_known13	cluster_5	Common_1_DN	41274	39574.221	39217	39896	207.944	1.043	0.0607	0.001	0.001	12306	197388	80.739	3191	49600	20.288	2612	41274	16.883	21.2254	20.9101	81.8552	83.2137
DN	GATA_known17	cluster_6	Common_2	20680	19828.252	19500	20126	183.9696	1.043	0.0607	0.001	0.0010909	5009	80296	75.449	1780	27104	25.468	1326	20680	19.432	26.4723	25.7547	74.4944	76.2987
SP8	TBX5_3	cluster_4	SP4	14288	13701.091	13375	13990	178.9281	1.0428	0.0605	0.001	0.001	14898	134209	54.179	2921	25603	10.336	1603	14288	5.7679	10.7598	10.6461	54.8785	55.806
DN	POU2F2_known14	cluster_6	Common_2	5293	5077.476	4949	5212	83.7672	1.0424	0.06	0.004	0.0048	1238	18993	84.41	474	6572	29.208	367	5293	23.523	29.6446	27.8682	77.4262	80.5386
DN	MGA_2	cluster_6	Common_2	34641	33238.567	32846	33620	232.7222	1.0422	0.0596	0.001	0.0010909	6706	114146	75.028	2767	45310	29.782	2074	34641	22.769	30.9275	30.348	74.9548	76.4533
DN	SPDEF_6	cluster_6	Common_2	70044	67221.55	66851	67555	206.9633	1.042	0.0593	0.001	0.001	10392	145946	84.321	5862	80942	46.765	5027	70044	40.468	48.3737	47.9931	85.7557	86.536
DN	RUNX_1	cluster_5	Common_1_DN	130886	125641.01	125038	126293	392.7883	1.0417	0.059	0.001	0.0010435	34752	490327	76.602	12058	165555	25.864	9388	130886	20.448	27.0143	26.6936	77.857	79.0589
DN	KLF4_1	cluster_5	Common_1_DN	171050	164230.352	163546	164940	417.4623	1.0415	0.0587	0.001	0.001	90604	870001	74.9	23920	222161	19.126	18219	171050	14.726	20.1084	19.6609	76.1664	76.9937
SP8	FOXO1_4	cluster_4	SP4	4608	4424.437	4248	4595	108.503	1.0415	0.0586	0.043	0.04487	2229	29043	58.324	628	7807	15.678	359	4608	9.2538	16.1059	15.8661	57.1656	59.024
DN	SOX3_3	cluster_6	Common_2	14442	13867.996	13594	14159	174.4916	1.0414	0.0585	0.001	0.0010909	3414	54700	70.442	1342	20452	26.338	930	14442	18.598	27.2408	26.4022	69.2996	70.6141
DN	SOX2_5	cluster_6	Common_2	17962	17257.863	16941	17513	175.4775	1.0408	0.0577	0.001	0.0010909	4046	64802	73.448	1609	24458	27.721	1152	17962	20.359	28.4726	27.7183	71.5973	73.4402
DN	ATF3_known4	cluster_5	Common_1_DN	26779	25732.075	25397	26029	186.9519	1.0407	0.0575	0.001	0.001	10011	141182	80.298	2360	32327	18.386	1930	26779	15.231	19.2788	18.9677	81.7797	82.8379
DN	HES7_1	cluster_5	Common_1_DN	31406	30187.665	29890	30487	180.4533	1.0404	0.0571	0.001	0.0010435	12542	138583	78.225	3600	38880	21.946	2875	31406	17.727	22.923	22.6622	79.8611	80.7767
SP4	POU2F2_known1	cluster_1	SP8	6347	6101.477	5848	6331	148.2073	1.0402	0.0569	0.031	0.031	3339	60392	62.523	595	10054	10.409	361	6347	6.571	10.8116	10.5097	60.6723	63.1291
DN	HES5_1	cluster_5	Common_1_DN	31054	29854.821	29562	30142	176.132	1.0402	0.0568	0.001	0.0010435	12165	134258	77.001	3606	39030	22.385	2850	31054	17.81	23.4279	23.1301	79.0349	79.5644
DN	MESP1_1	cluster_5	Common_1_DN	104366	100420.174	99971	100922	295.2246	1.0393	0.0556	0.001	0.001	44484	401632	76.45	14895	132166	25.158	11653	104366	19.866	26.1959	25.9855	78.2343	78.9658
SP4	TBX15_1	cluster_1	SP8	10704	10300.842	10016	10636	195.1561	1.0391	0.0554	0.024	0.025043	6066	109292	57.806	1054	18196	9.6241	602	10704	5.6615	9.9242	9.7939	57.1157	58.8261
DN	SP4_2	cluster_5	Common_1_DN	218220	210062.095	208981	211052	634.2969	1.0388	0.055	0.001	0.001	66957	1120770	78.67	17108	272423	19.122	13476	218220	15.318	20.1263	19.4705	78.7702	80.1034
DN	SOX17_4	cluster_6	Common_2	12819	12346.295	12124	12576	138.4962	1.0383	0.0542	0.001	0.0010909	3286	46015	72.668	1317	17642	27.861	943	12819	20.244	28.6975	27.8583	71.6021	72.6618
SP4	MAF_known10	cluster_1	SP8	9166	8836.764	8597	9078	146.6812	1.0373	0.0528	0.011	0.011478	8761	96521	59.023	1423	15177	9.2809	842	9166	5.6051	9.6108	9.4964	59.1708	60.394
SP4	CBX5_1	cluster_1	SP8	5732	5531.571	5346	5739	111.6635	1.0362	0.0513	0.057	0.057	4490	49467	65.073	838	8765	11.53	535	5732	7.5403	11.9154	11.5875	63.8425	65.3965
DN	RREB1_2	cluster_5	Common_1_DN	92369	89175.217	87753	90518	896.2467	1.0358	0.0508	0.001	0.001	18681	474233	23.829	18832	415372	20.871	4078	92369	4.6413	21.8297	19.4776	21.6546	22.2377
SP4	TBX2_2	cluster_1	SP8	9535	9205.848	8998	9457	139.9808	1.0358	0.0507	0.007	0.0073043	9302	93136	58.465	1622	15897	9.9792	961	9535	5.9855	10.3311	10.2377	59.2478	59.9799
SP8	SOX2_5	cluster_4	SP4	7519	7262.626	6994	7493	147.4586	1.0353	0.05	0.043	0.04487	3178	50901	57.693	874	13039	14.779	484	7519	8.5222	15.2297	14.7718	55.3776	57.6655
DN	EGR3_3	cluster_5	Common_1_DN	91041	87967.077	87366	88640	381.3745	1.0349	0.0496	0.001	0.001	35070	516864	79.095	7938	112982	17.29	6316	91041	13.932	18.0097	17.6141	79.5666	80.5801
SP8	SOX14_2	cluster_4	SP4	5347	5167.388	5007	5356	108.0865	1.0348	0.0493	0.057	0.059478	3330	36787	57.469	866	9223	14.408	489	5347	8.3531	14.6847	14.535	56.4665	57.9746
DN	TCF3_2	cluster_5	Common_1_DN	156574	151323.9	150582	152057	458.0418	1.0347	0.0492	0.001	0.0010435	39118	592154	77.209	13449	197852	25.797	10472	156574	20.415	26.7703	26.4414	77.8645	79.1369
SP4	POU2F2_known12	cluster_1	SP8	5141	4970.174	4806	5140	96.0392	1.0344	0.0487	0.04	0.04	4643	46514	63.727	834	7995	10.954	525	5141	7.0434	11.3073	11.0526	62.9496	64.3027
SP4	POU2F2_known15	cluster_1	SP8	3993	3861.049	3691	4003	94.2833	1.0342	0.0485	0.075	0.075	3260	36005	62.683	593	6304	10.975	370	3993	6.9516	11.3497	11.0901	62.3946	63.3407
DN	SOX18_4	cluster_6	Common_2	10255	9921.053	9688	10150	137.7398	1.0337	0.0478	0.003	0.0034286	2707	37982	72.115	1070	14264	27.082	755	10255	19.471	27.8907	26.9996	70.5607	71.8943
DN	ARID3A_2	cluster_6	Common_2	7443	7202.653	7028	7359	104.1768	1.0334	0.0473	0.007	0.0084	1580	25566	81.304	641	9493	30.189	488	7443	23.67	30.8861	29.1129	76.131	78.4051
DN	TBX15_1	cluster_6	Common_2	40971	39650.048	39237	40123	264.5503	1.0333	0.0473	0.001	0.0010909	7884	142057	75.135	3141	54275	28.707	2317	40971	21.67	29.3886	28.8412	73.7663	75.4878
SP8	TBX4_1	cluster_4	SP4	8483	8211.574	8027	8447	119.0967	1.0331	0.0469	0.019	0.019	11799	82621	55.065	2178	15032	10.018	1218	8483	5.6537	10.3229	10.2674	55.9229	56.4329
SP4	MAF_known1	cluster_1	SP8	34816	33709.286	33120	34274	346.4189	1.0328	0.0466	0.001	0.001	26418	399043	58.146	4027	58739	8.559	2329	34816	5.0731	8.816	8.7249	57.8346	59.2724
DN	HESX1_1	cluster_6	Common_2	5045	4885.732	4795	4977	55.4396	1.0326	0.0463	0.003	0.0036	1814	16345	82.401	700	6114	30.823	570	5045	25.434	31.4223	30.8657	81.4286	82.5155
SP4	ARID5B_1	cluster_1	SP8	6476	6271.594	6046	6498	138.435	1.0326	0.0463	0.078	0.078	4639	60416	59.556	872	10810	10.656	512	6476	6.3838	11.0369	10.719	58.7156	59.9075
DN	KLF16_1	cluster_5	Common_1_DN	189432	183494.279	182736	184286	462.7751	1.0324	0.0459	0.001	0.001	87857	980098	74.479	23523	251382	19.103	17452	189432	14.395	19.8641	19.3279	74.1912	75.3562
DN	IKZF2_2	cluster_6	Common_2	15581	15099.176	14928	15292	108.2544	1.0319	0.0453	0.001	0.0010435	4704	47046	78.526	2039	19720	32.915	1591	15581	26.007	33.8223	33.1186	78.0284	79.0112
DN	NFATC1_3	cluster_6	Common_2	15914	15424.547	15225	15652	133.783	1.0317	0.0451	0.001	0.0010909	4137	53815	79.338	1619	20119	29.661	1259	15914	23.462	30.4327	29.5717	77.7641	79.0994
SP4	RBPJ_2	cluster_1	SP8	8396	8139.626	7917	8399	136.2585	1.0315	0.0447	0.051	0.053217	10316	103270	57.388	1475	14370	7.9855	850	8396	4.6657	8.2396	8.1301	57.6271	58.4273
DN	ID4_1	cluster_5	Common_1_DN	116776	113229.481	112697	113740	321.2045	1.0313	0.0445	0.001	0.0010435	48845	442152	75.691	16901	150382	25.744	13005	116776	19.991	26.625	26.4108	76.9481	77.6529
SP8	HESX1_2	cluster_4	SP4	1568	1520.818	1415	1620	64.082	1.031	0.044	0.233	0.233	899	12600	63.31	192	2490	12.511	114	1568	7.8786	12.6808	12.4444	59.375	62.9719
DN	ETS_known17	cluster_5	Common_1_DN	56332	54645.889	54090	55171	327.6637	1.0309	0.0438	0.001	0.001	15240	260380	73.574	4576	75147	21.234	3340	56332	15.917	21.916	21.6345	72.9895	74.9624
DN	MYCN_2	cluster_5	Common_1_DN	41180	39956.721	39676	40231	172.4636	1.0306	0.0435	0.001	0.001	21157	191106	79.065	5735	50848	21.037	4598	41180	17.037	21.7328	21.5483	80.1744	80.9865
SP4	FOXP1_1	cluster_1	SP8	27164	26359.709	25717	26950	348.4423	1.0305	0.0434	0.007	0.007	11002	256998	66.218	2385	45631	11.757	1336	27164	6.9991	12.1432	10.5697	56.0168	59.5297
SP4	IKZF2_2	cluster_1	SP8	2879	2793.602	2686	2904	72.9148	1.0306	0.0434	0.129	0.129	3698	36980	61.725	483	4645	7.7532	293	2879	4.8055	7.9232	7.7853	60.6625	61.9806
DN	TBX1_2	cluster_6	Common_2	32420	31478.862	31037	31833	254.1531	1.0299	0.0425	0.001	0.0010909	6383	115007	73.738	2536	43862	28.122	1827	32420	20.786	28.6229	28.1896	72.0426	73.9136
SP8	ARID3A_2	cluster_4	SP4	2258	2193.115	2057	2352	86.4174	1.0296	0.042	0.213	0.22226	1242	20101	63.924	247	3631	11.547	148	2258	7.1808	11.9163	11.2333	59.919	62.1867
SP4	SOX11_1	cluster_1	SP8	20020	19456.162	19062	19836	239.8517	1.029	0.0412	0.009	0.009	11736	190304	65.17	2020	30899	10.581	1273	20020	6.8559	10.847	10.52	63.0198	64.7917
SP4	SOX5_1	cluster_1	SP8	2468	2398.76	2287	2512	66.4349	1.0289	0.041	0.148	0.15443	2386	21481	63.838	438	3829	11.379	277	2468	7.3345	11.6094	11.4892	63.242	64.4555
DN	RBPJ_1	cluster_6	Common_2	32996	32081.378	31831	32334	149.8123	1.0285	0.0406	0.001	0.0010435	16019	112173	78.007	6050	41625	28.947	4762	32996	22.946	29.7272	29.4153	78.7107	79.2697
SP4	MAF_known11	cluster_1	SP8	12957	12598.346	12251	12911	201.0186	1.0285	0.0405	0.032	0.032	10831	174705	57.034	1445	22364	7.3009	813	12957	4.2299	7.5062	7.4165	56.263	57.9369
SP4	SOX18_2	cluster_1	SP8	4889	4754.533	4567	4947	112.5282	1.0283	0.0402	0.108	0.1127	2930	41032	62.484	591	7867	11.98	358	4889	7.445	12.2184	11.9151	60.5753	62.1457
DN	ETV6_2	cluster_5	Common_1_DN	20435	19875.093	19450	20345	268.9462	1.0282	0.0401	0.019	0.019826	10518	94973	25.655	8770	77969	21.062	2291	20435	5.5202	21.7817	21.5166	26.1231	26.2091
SP4	TCF7L2_known7	cluster_1	SP8	20868	20309.196	19947	20739	230.6105	1.0275	0.0392	0.003	0.003	12562	176542	61.995	2430	33136	11.636	1502	20868	7.3281	11.9567	11.8204	61.8107	62.9768
DN	SPI1_known1	cluster_6	Common_2	31533	30689.75	30217	31147	289.7549	1.0275	0.0391	0.001	0.001	15535	109231	23.39	18984	132280	28.326	4522	31533	6.7524	29.1085	28.8682	23.8201	23.8381
DN	ELK4_1	cluster_5	Common_1_DN	15738	15317.999	15192	15464	86.3593	1.0274	0.039	0.001	0.001	11601	93425	84.428	2290	18216	16.462	1968	15738	14.222	16.9641	16.8456	85.9389	86.3966
DP	CUX2_1	cluster_5	Common_1_DN	842	819.714	735	906	50.8422	1.0272	0.0387	0.337	0.337	328	6022	56.539	100	1592	14.947	51	842	7.9054	15.5488	13.9821	51	52.8894
SP4	SOX4_2	cluster_1	SP8	8003	7791.634	7560	8059	146.024	1.0271	0.0386	0.072	0.07513	4816	72432	63.066	888	12727	11.081	549	8003	6.9682	11.3995	11.049	61.8243	62.8821
DN	RUNX2_5	cluster_5	Common_1_DN	89622	87309.217	86700	87943	365.2097	1.0265	0.0377	0.001	0.001	23276	408017	80.689	6528	109696	21.693	5209	89622	17.724	22.3793	21.9653	79.7947	81.7003
SP4	CUX2_1	cluster_1	SP8	1026	999.726	916	1082	51.7009	1.0263	0.0374	0.305	0.31826	439	7949	74.631	91	1512	14.196	61	1026	9.6329	13.8952	12.9073	67.033	67.8571
SP4	GATA_known17	cluster_1	SP8	6457	6292.948	6035	6534	152.6806	1.0261	0.0371	0.117	0.117	4062	65192	61.257	690	10533	9.8972	412	6457	6.0672	10.1428	9.9046	59.7101	61.3026
SP8	TBX2_2	cluster_4	SP4	10743	10471.604	10233	10695	146.2056	1.0259	0.0369	0.015	0.015	9067	90775	56.983	1911	18576	11.661	1083	10743	6.7438	11.9444	11.8348	56.6719	57.8327
DN	SP1_known2	cluster_5	Common_1_DN	332852	324504.995	323499	325515	631.3439	1.0257	0.0366	0.001	0.001	131181	1808239	79.788	31582	415613	18.339	25153	332852	14.687	19.1743	18.4075	79.6435	80.087
SP4	SOX2_5	cluster_1	SP8	6035	5885.533	5628	6126	144.5004	1.0254	0.0362	0.165	0.165	3385	54207	61.44	655	9906	11.228	386	6035	6.8402	11.4032	11.1332	58.9313	60.9227
SP4	TBX4_2	cluster_1	SP8	11605	11330.138	11004	11675	208.1241	1.0243	0.0346	0.091	0.091	6921	131760	58.982	1068	19559	8.7555	616	11605	5.1949	8.9004	8.8077	57.6779	59.3333
DN	MESP1_1	cluster_6	Common_2	107665	105142.454	104604	105656	315.684	1.024	0.0342	0.001	0.001	44484	401632	76.45	15538	138355	26.336	11979	107665	20.494	26.9288	26.8069	77.0949	77.8179
SP4	MAFG_1	cluster_1	SP8	10541	10295.816	9959	10657	217.69	1.0238	0.034	0.142	0.142	6447	129183	56.562	971	18472	8.0878	536	10541	4.6153	8.3139	8.1597	55.2008	57.0647
SP4	TBX15_2	cluster_1	SP8	9803	9575.599	9352	9790	132.0565	1.0237	0.0339	0.039	0.040696	14978	104871	54.579	2561	17651	9.1862	1411	9803	5.1018	9.4205	9.3477	55.0957	55.5379
DN	ELK4_2	cluster_5	Common_1_DN	14212	13884.072	13495	14272	247.5277	1.0236	0.0337	0.115	0.12	7819	70678	26.176	5965	53260	19.725	1588	14212	5.2634	20.3095	20.1081	26.622	26.6842
SP4	TBX5_5	cluster_1	SP8	10649	10404.602	10027	10782	224.9617	1.0235	0.0335	0.143	0.143	5992	114030	59.04	980	17964	9.3011	564	10649	5.5136	9.4126	9.3388	57.551	59.2797
SP4	MGA_2	cluster_1	SP8	7815	7636.827	7377	7896	168.4661	1.0233	0.0333	0.151	0.15757	5162	87892	57.771	839	13515	8.8834	467	7815	5.1368	9.0469	8.8916	55.6615	57.8246
SP4	BPTF_1	cluster_1	SP8	19232	18798.44	18458	19157	216.9969	1.0231	0.0329	0.023	0.024	12832	172114	61.355	2436	31404	11.195	1453	19232	6.8558	11.3233	11.174	59.647	61.2406
SP4	SOX21_2	cluster_1	SP8	6313	6171.908	5941	6396	135.0495	1.0229	0.0326	0.147	0.15339	3895	54547	61.419	767	10297	11.594	460	6313	7.1083	11.81	11.5735	59.9739	61.3091
SP4	HMGA1_2	cluster_1	SP8	9262	9056.816	8821	9311	161.9324	1.0227	0.0323	0.108	0.108	5885	82554	67.162	1076	14034	11.417	687	9262	7.5351	11.6737	11.2193	63.8476	65.9969
SP4	SOX10_4	cluster_1	SP8	9436	9230.902	8985	9506	156.916	1.0222	0.0317	0.107	0.11165	5739	86325	61.805	1057	15221	10.898	639	9436	6.7557	11.1343	10.9308	60.4541	61.9933
SP4	IKZF2_3	cluster_1	SP8	5650	5528.066	5383	5696	97.456	1.0221	0.0315	0.111	0.111	5825	58444	66.163	904	8622	9.7608	577	5650	6.3963	9.9056	9.6674	63.8274	65.53
SP4	SOX4_1	cluster_1	SP8	21037	20582.52	20137	21051	270.3364	1.0221	0.0315	0.053	0.053	12657	205161	65.153	2132	32642	10.366	1338	21037	6.6807	10.5712	10.2539	62.758	64.4476
SP4	POU2F3_1	cluster_1	SP8	6082	5958.774	5754	6197	130.5516	1.0207	0.0295	0.162	0.162	3852	58181	62.51	681	9801	10.53	414	6082	6.5346	10.7477	10.4536	60.793	62.0549
SP4	PBX1_2	cluster_1	SP8	5497	5388.465	5184	5582	123.461	1.0201	0.0288	0.201	0.201	3037	43176	61.498	666	8974	12.782	397	5497	7.8297	13.0721	12.7316	59.6096	61.2547
SP4	TCF12_known1	cluster_1	SP8	13981	13705.593	13453	13964	162.9291	1.0201	0.0287	0.038	0.038	34730	249719	53.636	3620	25716	5.5234	1963	13981	3.0029	5.6522	5.5987	54.2265	54.3669
SP4	NFATC1_1	cluster_1	SP8	9224	9049.033	8735	9345	191.5209	1.0193	0.0276	0.19	0.19	5359	103439	64.397	818	14633	9.1099	495	9224	5.7425	9.2368	8.9173	60.5134	63.0356
SP8	FOXM1_1	cluster_4	SP4	4333	4250.785	4087	4426	97.3133	1.0193	0.0276	0.182	0.18991	4986	39915	56.233	992	7674	10.811	548	4333	6.1045	10.9908	10.8556	55.2419	56.4634
DN	ETV1_1	cluster_5	Common_1_DN	8404	8251.171	7987	8520	164.2437	1.0185	0.0265	0.162	0.162	5090	46038	26.319	3527	31478	17.995	939	8404	4.8043	18.4479	18.2545	26.6232	26.698
DN	HIF1A_2	cluster_5	Common_1_DN	40888	40145.74	39818	40499	214.4515	1.0185	0.0264	0.001	0.001	15992	214291	80.94	3862	50133	18.936	3099	40888	15.444	19.3784	19.0806	80.2434	81.5591

SP4	TCF7L1_2	cluster_1	SP8	16019	15728.76	15410	16066	200.4045	1.0185	0.0264	0.069	0.069	12702	140496	61.647	2407	25849	11.342	1466	16019	7.0288	11.5415	11.4017	60.9057	61.9714
SP4	XBP1_2	cluster_1	SP8	4720	4635.076	4419	4877	139.2657	1.0183	0.0262	0.27	0.27	2736	63041	65.364	332	7249	7.5161	207	4720	4.8939	7.5658	7.4872	62.3494	65.1124
DN	KLF13_1	cluster_5	Common_1_DN	86005	84551.504	83846	85245	400.7072	1.0172	0.0246	0.001	0.001	24149	415074	75.178	6957	114292	20.701	5117	86005	15.577	21.1893	20.7204	73.5518	75.2502
DN	TCF21_1	cluster_6	Common_2	49871	49030.467	48565	49482	276.0501	1.0171	0.0245	0.001	0.001	16797	218881	77.73	5010	63749	22.639	3864	49871	17.71	23.0041	22.7845	77.1257	78.2302
SP4	HMGA1_1	cluster_1	SP8	2455	2414.079	2319	2493	51.8759	1.0169	0.0242	0.234	0.234	3745	22602	66.657	611	3646	10.753	408	2455	7.2402	10.8945	10.8619	66.7758	67.3341
SP8	TBX15_2	cluster_4	SP4	9953	9787.845	9594	10029	133.2958	1.0169	0.0241	0.104	0.104	14966	104782	54.532	2637	18153	9.4475	1430	9953	5.1799	9.555	9.4988	54.2283	54.8284
DN	RUNX3_3	cluster_5	Common_1_DN	111163	109351.634	108691	109995	370.112	1.0166	0.0237	0.001	0.001	29706	527830	81.113	8022	136756	21.016	6396	111163	17.083	21.531	21.0604	79.7307	81.2856
SP4	SOX8_2	cluster_1	SP8	8660	8520.481	8228	8777	172.3505	1.0164	0.0234	0.207	0.207	5294	79524	62.195	975	13966	10.923	586	8660	6.7729	11.0691	10.8898	60.1026	62.0077
DN	TCF4_2	cluster_6	Common_2	106196	104503.666	104047	105000	292.006	1.0162	0.0232	0.001	0.001	45335	409696	76.215	15507	138006	25.673	11815	106196	19.756	26.0615	25.9207	76.1914	76.9503
SP4	TBX1_2	cluster_1	SP8	9182	9037.398	8729	9306	174.8258	1.016	0.0229	0.198	0.20661	4990	89894	57.636	933	16006	10.262	521	9182	5.8871	10.4409	10.2143	55.8414	57.366
SP4	TCF7_2	cluster_1	SP8	17160	16893.683	16610	17196	183.9763	1.0158	0.0226	0.085	0.085	13256	146350	61.849	2583	27660	11.689	1577	17160	7.252	11.8965	11.7253	61.053	62.039
SP4	SOX12_1	cluster_1	SP8	4894	4818.095	4653	5002	109.6803	1.0158	0.0225	0.233	0.233	3166	41308	65.459	625	7670	12.154	388	4894	7.7553	12.2552	11.8476	62.08	63.807
SP8	SOX17_4	cluster_4	SP4	5500	5416.785	5216	5625	120.6714	1.0154	0.022	0.239	0.24939	2660	37246	58.82	725	9491	14.988	405	5500	8.6858	15.2256	14.7667	55.8621	57.9496
SP4	TBX19_1	cluster_1	SP8	7083	6978.19	6722	7266	164.0802	1.015	0.0215	0.26	0.26	3815	72708	56.872	692	12512	9.7869	379	7083	5.5403	9.9345	9.7417	54.7688	56.6097
DN	SP4_1	cluster_5	Common_1_DN	294249	290081.623	288888	291166	664.7831	1.0144	0.0206	0.001	0.001	90889	1624561	78.36	22652	380416	18.349	17214	294249	14.193	18.9396	18.1125	75.9933	77.3493
SP4	FOXP3_2	cluster_1	SP8	3313	3265.94	3164	3382	63.7012	1.0144	0.0206	0.243	0.243	4729	28374	62.407	895	5284	11.622	556	3313	7.2868	11.7572	11.6762	62.1229	62.6987
SP4	POU2F2_known5	cluster_1	SP8	5411	5335.039	5131	5564	122.7236	1.0142	0.0204	0.271	0.271	3860	50256	61.466	730	8982	10.986	431	5411	6.618	11.1658	10.7669	59.0411	60.2427
SP4	FOXP3_1	cluster_1	SP8	12528	12354.445	12005	12693	207.4968	1.014	0.0201	0.212	0.212	7981	128134	57.854	1425	21824	9.8538	792	12528	5.6566	9.9236	9.7773	55.5789	57.4047
SP4	ETV6_2	cluster_1	SP8	6348	6260.376	5995	6578	164.8989	1.014	0.02	0.263	0.263	10041	90698	24.501	2882	25678	6.9365	707	6348	1.7148	7.0411	6.9991	24.5316	24.7216
SP4	POU2F3_2	cluster_1	SP8	3221	3178.684	3070	3288	68.0749	1.0133	0.0191	0.261	0.261	3575	28645	62.59	656	5138	11.227	411	3221	7.038	11.4965	11.2445	62.6524	62.6898
SP4	FOXM1_1	cluster_1	SP8	3832	3781.912	3653	3913	82.546	1.0132	0.019	0.281	0.281	5181	41487	58.448	835	6541	9.2151	481	3832	5.3986	9.2839	9.2366	57.6048	58.5843
DN	PKNOX2_3	cluster_6	Common_2	38092	37597.864	37242	38014	230.1261	1.0131	0.0188	0.018	0.018	13787	153514	71.649	4884	53142	24.803	3465	38092	17.779	25.1324	24.8134	70.9459	71.6797
SP4	TCF7L1_1	cluster_1	SP8	21360	21083.928	20659	21527	257.8895	1.0131	0.0188	0.161	0.161	10758	172842	61.696	2221	34617	12.357	1343	21360	7.6244	12.4837	12.3581	60.4683	61.7038
SP4	SOX7_3	cluster_1	SP8	7042	6951.783	6704	7163	142.2253	1.013	0.0186	0.274	0.274	3572	57193	62.928	758	11442	12.589	453	7042	7.7482	12.682	12.3127	59.7625	61.5452
SP4	SOX21_3	cluster_1	SP8	5935	5859.448	5638	6071	137.6077	1.0129	0.0185	0.301	0.301	3663	54981	62.433	686	9659	10.968	404	5935	6.7394	11.0292	10.7946	58.8921	61.4453
SP4	TCF3_1	cluster_1	SP8	29992	29626.462	29053	30176	337.8365	1.0123	0.0177	0.129	0.13461	45052	639694	51.735	4255	58143	4.7023	2141	29992	2.4256	4.7523	4.6885	50.3173	51.5832
DN	XBP1_4	cluster_5	Common_1_DN	16532	16334.995	16066	16609	160.9087	1.0121	0.0173	0.111	0.111	6101	79442	72.628	1802	22724	20.775	1289	16532	15.114	21.1277	20.8102	71.5316	72.7513
DN	KLF7_1	cluster_5	Common_1_DN	241303	238556.731	237323	239791	730.7795	1.0115	0.0165	0.001	0.001	81419	1312000	74.111	21519	328882	18.578	15488	241303	13.631	19.0226	18.392	71.9736	73.3707
DN	PKNOX1_2	cluster_6	Common_2	36910	36505.642	36128	36865	232.833	1.0111	0.0159	0.039	0.039	13772	152898	72.377	4710	51072	24.176	3369	36910	17.472	24.4627	24.1403	71.5287	72.2705
SP4	SMAD_2	cluster_1	SP8	20195	19983.14	19651	20304	213.93	1.0106	0.0152	0.18	0.18	22080	227820	57.244	3462	35177	8.8389	1961	20195	5.0744	8.8813	8.8645	56.6436	57.4097
SP4	SOX11_2	cluster_1	SP8	6099	6035.174	5841	6253	120.8145	1.0106	0.0152	0.282	0.282	3896	54587	64.334	731	9670	11.397	447	6099	7.188	11.4733	11.173	61.1491	63.0714
DN	ETV3_1	cluster_5	Common_1_DN	9192	9097.75	8775	9408	190.7575	1.0104	0.0149	0.304	0.304	5350	48305	25.577	4036	35843	18.978	1028	9192	4.867	19.215	19.0291	25.4708	25.6452
DN	HIF1A_1	cluster_5	Common_1_DN	29326	29024.284	28753	29308	173.759	1.0104	0.0149	0.035	0.035	13599	153110	79.913	3336	36648	19.128	2630	29326	15.306	19.3397	19.1535	78.8369	80.0207
SP4	SOX1_2	cluster_1	SP8	5363	5310.375	5077	5524	128.8114	1.0099	0.0142	0.358	0.37357	3418	47871	59.739	686	9166	11.438	391	5363	6.6925	11.4394	11.203	56.9971	58.5097
DN	PKNOX2_2	cluster_6	Common_2	42760	42368.956	41983	42808	242.5446	1.0092	0.0133	0.063	0.063	15871	176400	72.498	5428	59074	24.279	3893	42760	17.574	24.529	24.2404	71.7207	72.3838
SP8	SOX15_4	cluster_4	SP4	4815	4770.812	4568	4962	119.6475	1.0093	0.0133	0.332	0.332	2193	30727	55.692	676	8954	16.229	357	4815	8.7271	16.2791	15.6703	52.8107	53.7748
DN	ETV5_1	cluster_5	Common_1_DN	6478	6418.895	6157	6697	164.7429	1.0092	0.0132	0.342	0.342	4257	38576	26.904	2699	24074	16.79	726	6478	4.5179	17.0543	16.7928	26.8989	26.9087
SP4	POU2F2_known14	cluster_1	SP8	1469	1455.951	1345	1557	65.8238	1.009	0.0129	0.436	0.436	922	14165	62.953	183	2502	11.12	105	1469	6.5286	11.3883	10.3706	57.377	58.713
SP4	SOX9_6	cluster_1	SP8	7550	7483.584	7277	7736	138.7762	1.0089	0.0127	0.305	0.305	4528	68048	61.63	871	12456	11.281	511	7550	6.838	11.2853	11.0951	58.6682	60.6134
SP4	MGA_3	cluster_1	SP8	7555	7495.589	7226	7804	167.7819	1.0079	0.0114	0.325	0.325	5104	86900	61.334	760	12400	8.7519	449	7555	5.3323	8.797	8.6939	59.0789	60.9274
DN	RUNX_2	cluster_6	Common_2	95864	95122.475	94512	95794	379.3819	1.0078	0.0112	0.017	0.017	28180	397366	76.459	9187	125839	24.213	6882	95864	18.446	24.4216	24.1249	74.9102	76.1799
SP8	FOXO3_4	cluster_4	SP4	3960	3929.762	3763	4106	100.8485	1.0077	0.0111	0.383	0.383	1903	24809	59.374	546	6810	16.298	308	3960	9.4773	16.185	15.9619	56.4103	58.1498
SP4	GATA_known16	cluster_1	SP8	9859	9792.639	9452	10128	210.7291	1.0068	0.0097	0.39	0.39	5122	107965	62.558	822	16180	9.3751	478	9859	5.7126	9.3323	9.1317	58.1509	60.9333
SP4	SOX15_2	cluster_1	SP8	5574	5537.042	5318	5749	131.5108	1.0067	0.0096	0.409	0.42678	3199	44866	62.621	679	9108	12.712	403	5574	7.7798	12.5977	12.4237	59.352	61.1989
SP4	POU2F2_known11	cluster_1	SP8	5068	5036.619	4888	5190	90.9155	1.0062	0.009	0.353	0.353	4718	47310	63.382	843	8123	10.882	516	5068	6.7897	10.9368	10.7123	61.21	62.3907
SP4	FOXO3_1	cluster_1	SP8	8818	8764.019	8547	8967	136.1035	1.0062	0.0089	0.345	0.345	5633	74003	67.715	1083	13447	12.304	691	8818	8.0687	12.267	11.9157	63.8042	65.576
DN	ETV4_2	cluster_5	Common_1_DN	8210	8160.915	7881	8421	175.3357	1.006	0.0087	0.391	0.391	5010	45275	26.285	3500	31215	18.122	919	8210	4.7664	18.3433	18.1336	26.2571	26.3015
SP8	TBX21_6	cluster_4	SP4	16916	16815.049	16383	17268	260.5033	1.006	0.0086	0.332	0.34643	8068	145632	57.226	1741	29870	11.737	952	16916	6.6471	11.7997	11.6156	54.6812	56.6321
DN	RUNX_1	cluster_6	Common_2	111917	111521.514	110822	112161	396.4417	1.0035	0.0051	0.152	0.152	34752	490327	76.602	10721	147133	22.986	8025	111917	17.484	23.0922	22.825	74.8531	76.0652
SP4	TBX21_4	cluster_1	SP8	11698	11662.447	11315	11948	192.9033	1.003	0.0044	0.425	0.425	7892	118588	58.407	1404	20314	10.005	787	11698	5.7615	9.9721	9.8644	56.0541	57.5859
SP4	SOX9_4	cluster_1	SP8	8324	8299.791	8031	8557	161.4034	1.0029	0.0042	0.422	0.422	5343	80252	62.068	957	13666	10.57	564	8324	6.4379	10.5559	10.3723	58.9342	60.9103
SP8	TBX20_5	cluster_4	SP4	23103	23045.405	22529	23513	285.5868	1.0025	0.0036	0.418	0.418	12908	197715	58.58	2754	40150	11.896	1538	23103	6.8451	11.9151	11.685	55.846	57.5417
SP4	MYBL2_2	cluster_1	SP8	2914	2907.112	2734	3063	96.391	1.0024	0.0034	0.47	0.47	2320	35130	65.23	315	4541	8.4317	196	2914	5.4107	8.4483	8.2949	62.2222	64.1709
DN	ELK3_1	cluster_5	Common_1_DN	10891	10867.947	10562	11203	200.8161	1.0021	0.0031	0.458	0.458	6350	57329	26.539	4619	41177	19.061	1220	10891	5.0416	19.2126	18.9974	26.4126	26.4492
SP8	SOX17_5	cluster_4	SP4	5152	5140.916	4920	5349	123.9627	1.0022	0.0031	0.489	0.489	2277	31990	58.026	691	9164	16.623	379	5152	9.3452	16.6447	16.105	54.848	56.22
DN	AHR::ARNT::HIF1A_1	cluster_5	Common_1_DN	21800	21755.002	21535	21946	130.2027	1.0021	0.003	0.365	0.365	14502	119460	81.912	3290	26751	18.343	2663	21800	14.948	18.363	18.2488	80.9422	81.4923
DN	ATF6_1	cluster_5	Common_1_DN	6315	6303.018	6189	6412	70.9	1.0019	0.0027	0.415	0.415	4516	31617	73.316	1255	8634	20.021	908	6315	14.644	20.1063	19.9734	72.3506	73.1411
DN	TCF4_1	cluster_5	Common_1_DN	127629	127409.049	126830	128016	353.7273	1.0017	0.0025	0.268	0.268	54166	490170	74.751	19242	171202	26.108	14191	127629	19.464	26.1991	26.0377	73.7501	74.5488
DN	ID4_1	cluster_6	Common_2	103412	103247.525	102759	103787	311.3731	1.0016	0.0023	0.289	0.289	48845	442152	75.691	15416	137254	23.496	11498	103412	17.703	23.5398	23.3883	74.5848	75.3435
SP8	SOX21_2	cluster_4	SP4	7098	7086.859	6847	7323	144.1869	1.0016	0.0023	0.491	0.491	3688	51655	58.162	940	12505	14.08	518	7098	7.9922	14.0456	13.7412	55.1064	56.7613
SP8	SOX15_2	cluster_4	SP4	6223	6216.566	6002	6435	133.989	1.001	0.0015	0.481	0.481	3083	43248	60.363	809	10650	14.865	457	6223	8.6856	14.8232	14.3891	56.4895	58.4319
DN	TCF3_1	cluster_6	Common_2	88604	88537.025	87429	89720	671.5458	1.0008	0.0011	0.437	0.437	20600	290144	23.465	27526	383634	31.026	6373	88604	7.1659	30.9369	30.5379	23.1527	23.096
SP4	SPZ1_2	cluster_1	SP8	11219	11220.484	10965	11488	159.4397	0.9999	-2.00E-04	0.515	0.515	14241	142725	57.95	1996	19554	7.9394	1129	11219	4.5552	7.9278	7.8606	56.5631	57.3745
SP4	TCF7L2_known5	cluster_1	SP8	21644	21646.411	21231	22062	258.9701	0.9999	-2.00E-04	0.506	0.506	11860	190547	60.832	2312	36014	11.497	1361	21644	6.9098	11.4755	11.3589	58.8668	60.0989
SP4	FOXO1_5	cluster_1	SP8	25372	25390.347	24954	25814	261.9069	0.9993	-0.001	0.43	0.43	25037	276688	58.01	4092	44229	9.2729	2311	25372	5.3194	9.2303	9.1699	56.4761	57.3651
DN	TCF3_2	cluster_6	Common_2	130250	130347.645	129680	131117	441.1095	0.9993	-0.0011	0.438	0.438	39118	592154	77.209	11592	170739	22.262	8705	130250	16.983	22.2532	21.996	75.0949	76.286
SP8	SOX1_2	cluster_4	SP4	6208	6214.51	5986	6433	132.9027	0.999	-0.0015	0.459	0.459	3221	45113	56.297	855	11323	14.13	453	6208	7.747	14.064	13.761	52.9825	54.8265
SP4	SMAD3_1	cluster_1	SP8	15310	15327.003	15045	15586	167.416	0.9989	-0.0016	0.47	0.47	20754	171916	58.449	3179	26335	8.9536	1838	15310	5.2052	8.8561	8.9055	57.8169	58.1356
SP8	FOXO6_1	cluster_4	SP4	4900	4905.291	4695	5092	116.6415	0.9989	-0.0016	0.466	0.466	2530	32988	57.537	700	8761	15.281	381	4900	8.5464	15.0593	14.8539	54.4286	55.9297
SP8	SOX10_4	cluster_4	SP4	11504	11520.514	11194	11799	189.8007	0.9986	-0.0021	0.432	0.432	5401	81262	58.18	1406	20226	14.481	775	11504	8.2363	14.3492	14.1567	55.1209	56.8773
SP4	SMAD_1	cluster_1	SP8	10605	10623.504	10402	10836	133.7582	0.9983	-0.0025	0.48	0.48	16152	129249	59.226	2290	18078	8.2838	1334	10605	4.8595	8.259	8.2051	58.2533	58.6625
SP4	SOX17_4	cluster_1	SP8	4565	4572.78	4380	4743	113.6787	0.9983	-0.0025	0.466	0.466	2822	39514	62.402	569	7564	11.945	335	4565	7.2092	11.871	11.5529	58.8752	60.3517
SP8	SOX4_2	cluster_4	SP4	9568	9584.847	9318	9913	172.7182	0.9982	-0.0025	0.508	0.508	4506	67750	58.989	1180	16678	14.521	656	9568	8.3308	14.5584	14.1225	55.5932	57.369
DN	TCF4_1	cluster_6	Common_2	111382	111623.628	111065	112186	344.6045	0.9978	-0.0031	0.243	0.25357	54166	490170	74.751	16835	150077	22.887	12378	111382	16.986	22.852	22.7231	73.5254	74.2166
SP4	TBX20_5	cluster_1	SP8	19647	19708.957	19306	20109	252.0337	0.9969	-0.0045	0.401	0.418	13251	203127	60.183	2275	33402	9.8965	1310	19647	5.8211	9.886	9.6723	57.5824	58.8198
SP4	HES5_1	cluster_1	SP8	5442	5459.328	5298	5681	118.1431	0.9968	-0.0046	0.458	0.458	9376	103478	59.348	867	9291	5.3287	499	5442	3.1211	5.3221	5.2591	57.5548	58.5728
SP4	HBP1_1	cluster_1	SP8	6910	6933.715	6693	7166	142.339	0.9966	-0.0049	0.418	0.418	4429	68253	63.399	791	11380	10.571	467	6910	6.4185	10.5441	10.1241	59.0392	60.7206
SP4	SOX14_2	cluster_1	SP8	4290	4310.176	4138	4494	105.2069	0.9953	-0.0068	0.446	0.446	3491	38572	60.257	690	7315	11.428	394	4290	6.7019	11.2862	11.1221	57.1014	58.6466
SP4	TBX21_6	cluster_1	SP8	14790	14875.011	14464	15285	251.9501	0.9943	-0.0083	0.409	0.409	8330	150313	59.065	1472	25611	10.064	829	14790	5.8117	9.952	9.8395	56.3179	57.7486
SP4	ARID3A_2	cluster_1	SP8	2343	2356.874	2226	2502	84.9482	0.9941	-0.0085	0.433	0.433	1263	20439	64.999	256	3819	12.145	150	2343	7.4511	11.8765	11.4634	58.5938	61.3511
SP8	SOX9_4	cluster_4	SP4	10875	10942.397	10638	11227	177.6227	0.9938	-0.0089	0.358	0.37357	5012	75280	58.223	1338	19227	14.871	736	10875	8.4109	14.6848	14.4461	55.0075	56.5611
SP4	SOX3_1	cluster_1	SP8	4376	4403.334	4223	4605	111.8	0.9938	-0.009	0.392	0.392	2214	35441	64.162	481	7166	12.973	282	4376	7.9222	12.7371	12.3473	58.6279	61.0661
DN	CTCF_known1	cluster_3	DN	57527	57904.531	56782	58914	648.3278	0.9935	-0.0094	0.284	0.284	26314	479096	27.636	11978	213108	12.293	3256	57527	3.3184	12.3736	12.0074	27.1832	26.9943
SP4	SOX18_4	cluster_1	SP8	3564	3587.741	3421	3753	98.4464	0.9934	-0.0096	0.405	0.405	2335	32760	62.2	451	5993	11.379	262	3564	6.7668	11.2206	10.8791	58.0931	59.4694
SP8	TBX1_2	cluster_4	SP4	9826	9893.703	9595	10234	196.2135	0.9932	-0.0099	0.376	0.376	4843	87270	55.954	1076	18201	11.67	560	9826	6.3	11.5631	11.2593	52.0446	53.986
SP4	TCF7_1	cluster_1	SP8	22468	22628.231	22196	23031	240.5105	0.9929	-0.0103	0.24	0.24	11748	188705	61.707	2397	37254	12.182	1415	22468	7.3471	12.0446	11.9064	59.0321	60.3103
SP4	EWSR1::FLI1_1	cluster_1	SP8	65924	66427.291	65448	67417	571.0727	0.9924	-0.011	0.17	0.17	47664	952228	58.406	6241	116963	7.1741	3376	65924	4.0435	7.0829	6.9231	54.0939	56.3631
DN	ELK3_2	cluster_5	Common_1_DN	10487	10582.908	10253	10897	200.6956	0.9909	-0.0131	0.296	0.296	6130	55327	26.313	4526	40342	19.186	1176	10487	4.9876	19.1843	18.9546	25.9832	25.9952
SP4	TBX20_2	cluster_1	SP8	9247	9332.152	9029	9669	199.5205	0.9909	-0.0132	0.364	0.364	5606	101105	58.153	942	16294	9.3719	519	9247	5.3186	9.2579	9.1459	55.0955	56.751
DP	CTCF_known2	cluster_5	Common_1_DN	57315	57858.053	56930	58828	602.7264	0.9906	-0.0136	0.228	0.228	14645	235553	28.191	13031	205911	24.643	3571	57315	6.8594	24.3837	24.3321	27.4039	27.8348
DN	ATF2_2	cluster_5	Common_1_DN	12329	12449.552	12244	12671	126.6522	0.9903	-0.014	0.175	0.175	5805	63978	74.542	1556	16788	19.56	1129	12329	14.365	19.4488	19.2707	72.5578	73.4394
SP8	SOX18_2	cluster_4	SP4	5638	5696.28	5463	5903	130.039	0.9898	-0.0148	0.299	0.299	2756	38602	58.784	766	10021	15.26	415	5638	8.5856	15.0581	14.6055	54.1775	56.2619
SP4	MAF_known12	cluster_1	SP8	10748	10873.409	10553	11189	195.6538	0.9885	-0.0167	0.254	0.254	7957	135675	61.511	1102	17974	8.1489	645	10748	4.8729	8.1061	7.9219	58.5299	59.7975
SP4	FOXO4_2	cluster_1	SP8	9152	9259.868	9017	9518	146.4478	0.9884	-0.0169	0.204	0.204	6478	84929	66.37	1152	14358	11.22	715	9152	7.1521	11.0374	10.7761	62.066	63.7415
SP8	SPIC_2	cluster_4	SP4	27851	28182.555	27695	28653	277.413	0.9882	-0.0171	0.105	0.105	15787	205822	55.49	4012	51184	13.799	2147	27851	7.5086	13.5998	13.5316	53.5145	54.4135
SP4	IKZF1_2	cluster_1	SP8	12780	12938.49	12700	13184	149.3425	0.9878	-0.0178	0.164	0.164	20507	164063	59.864	2799	21823	7.9629	1613	12780	4.6632	7.8656	7.7897	57.6277	58.5621
SP4	RUNX1_4	cluster_1	SP8	3040	3078.232	2930	3213	82.1019	0.9876	-0.018	0.323	0.323	3165	32119	63.475	512	4957	9.7962	312	3040	6.0078	9.8578	9.4648	60.9375	61.3274
SP8	SPI1_known4	cluster_4	SP4	24133	24445.905	24017	24892	263.8168	0.9872	-0.0186	0.113	0.113	14322	186719	57.192	3409	43240	13.244	1867	24133	7.3919	13.0359	12.9248	54.7668	55.8117
SP4	PBX1_4	cluster_1	SP8	3753	3801.862	3665	3957	87.8607	0.9872	-0.0187	0.307	0.307	2946	33026	61.318	591	6347	11.784	342	3753	6.9681	11.609	11.3638	57.868	59.1303
SP8	TBX15_1	cluster_4	SP4	11680	11839.801	11491	12226	208.1042	0.9865	-0.0196	0.224	0.224	5892	106171	56.155	1270	21552	11.399	664	11680	6.1777	11.2695	11.0011	52.2835	54.1945
SP4	SOX5_3	cluster_1	SP8	4453	4515.02	4334	4683	105.41	0.9863	-0.02	0.274	0.274	2611	39250	66.643	516	7140	12.123	312	4453	7.5608	11.9494	11.3452	60.4651	62.3669
SP8	POU2F2_known13	cluster_4	SP4	8093	8206.116	7877	8525	197.9896	0.9862	-0.02	0.258	0.258	2653	55941	63.012	704	13662	15.389	399	8093	9.116	15.0396	14.467	56.6761	59.2373
SP8	SOX2_6	cluster_4	SP4	5164	5236.785	4990	5446	135.3711	0.9861	-0.0202	0.3	0.3	1941	31108	57.681	627	9364	17.363	330	5164	9.5752	17.0015	16.6002	52.6316	55.1474
SP8	FOXP3_2	cluster_4	SP4	3710	3764.378	3658	3870	65.4019	0.9856	-0.021	0.208	0.21704	4518	27108	59.623	1078	6365	13.999	622	3710	8.1599	13.7672	13.686	57.6994	58.2875
SP4	SOX14_3	cluster_1	SP8	5229	5306.981	5087	5509	121.9392	0.9853	-0.0214	0.214	0.214	3418	47906	58.588	698	9269	11.336	385	5229	6.3949	11.2639	10.9151	55.1576	56.4139
SP8	SOX7_4	cluster_4	SP4	6561	6664.812	6446	6891	138.7913	0.9844	-0.0226	0.238	0.238	2647	42472	56.757	802	12131	16.211	422	6561	8.7678	15.9426	15.4478	52.6185	54.0846
SP8	BPTF_1	cluster_4	SP4	19667	19992.24	19622	20365	223.339	0.9837	-0.0237	0.06	0.06	12575	168522	60.074	2650	34033	12.132	1496	19667	7.0109	11.8966	11.6703	56.4528	57.788
DN	ATF2_1	cluster_5	Common_1_DN	1662	1689.662	1632	1745	33.9837	0.9836	-0.0238	0.212	0.212	1484	10388	76.433	323	2222	16.349	240	1662	12.229	16.1725	15.9992	74.3034	74.7975
SP4	ELK4_2	cluster_1	SP8	4469	4553.523	4321	4795	137.6392	0.9814	-0.027	0.285	0.285	7834	70855	26.241	1952	17453	6.4637	497	4469	1.6551	6.3441	6.3072	25.4611	25.6059
SP8	SOX21_3	cluster_4	SP4	7456	7596.693	7353	7828	151.0907	0.9815	-0.027	0.161	0.168	3510	52680	59.82	903	13016	14.78	505	7456	8.4666	14.3875	14.1534	55.9247	57.2833
SP8	MGA_2	cluster_4	SP4	9204	9381.457	9048	9700	195.7531	0.9811	-0.0275	0.2	0.2	5058	86090	56.587	1040	16927	11.126	549	9204	6.0498	10.8541	10.6911	52.7885	54.3747
SP8	SOX5_1	cluster_4	SP4	3120	3181.048	3059	3306	73.6035	0.9808	-0.0279	0.179	0.179	2249	20248	60.174	618	5390	16.018	351	3120	9.2722	15.6069	15.4089	56.7961	57.885
SP8	SOX8_2	cluster_4	SP4	10820	11043.378	10748	11336	182.3861	0.9798	-0.0295	0.104	0.10852	4985	74903	58.581	1344	19312	15.104	734	10820	8.4622	14.7242	14.4453	54.6131	56.0273
SP4	ETV2_1	cluster_1	SP8	5996	6125.067	5872	6421	158.2226	0.9789	-0.0307	0.201	0.201	7748	77780	26.389	2363	23363	7.9265	602	5996	2.0343	7.7697	7.7089	25.4761	25.6645
SP8	MAF_known10	cluster_4	SP4	10516	10742.516	10522	11003	151.4151	0.9789	-0.0307	0.048	0.048	8275	91208	55.774	1819	19475	11.909	965	10516	6.4306	11.6616	11.5297	53.0511	53.9974
SP4	MYC_known13	cluster_1	SP8	6593	6735.815	6518	6960	124.9059	0.9788	-0.0309	0.115	0.115	9038	99518	55.876	1134	12181	6.8392	603	6593	3.7017	6.6718	6.6249	53.1746	54.1253
SP8	RBPJ_1	cluster_4	SP4	6301	6439.828	6247	6606	104.1467	0.9784	-0.0314	0.092	0.092	10911	76393	53.125	1817	12285	8.5432	913	6301	4.3818	8.3677	8.2481	50.2477	51.2902
SP4	TBX2_1	cluster_1	SP8	12578	12856.518	12520	13193	204.7576	0.9783	-0.0316	0.073	0.073	8640	147190	60.352	1317	21623	8.866	745	12578	5.1573	8.6227	8.5454	56.568	58.1695
SP4	SOX30_1	cluster_1	SP8	5690	5816.365	5599	6020	129.1231	0.9783	-0.0317	0.164	0.164	3742	56229	63.32	664	9477	10.672	386	5690	6.4075	10.3153	10.1193	58.1325	60.0401
SP8	FOXP3_1	cluster_4	SP4	15284	15626.615	15232	15997	239.8379	0.9781	-0.032	0.098	0.10226	7647	122767	55.431	1889	28677	12.948	971	15284	6.9009	12.6978	12.4496	51.4029	53.2971
SP4	STAT_known3	cluster_1	SP8	9168	9390.108	9094	9695	183.0874	0.9763	-0.0345	0.123	0.123	7269	145730	67.556	756	14273	6.6166	468	9168	4.25	6.4383	6.2911	61.9048	64.2332
SP8	SOX9_6	cluster_4	SP4	9482	9714.994	9459	9989	162.2658	0.976	-0.035	0.078	0.081391	4230	63611	57.612	1220	17382	15.743	642	9482	8.5878	15.1773	14.9062	52.623	54.5507
SP8	SOX3_1	cluster_4	SP4	5082	5215.046	4992	5420	127.6424	0.9745	-0.0373	0.17	0.17739	2117	33889	61.352	600	8890	16.094	331	5082	9.2004	15.6353	14.996	55.1667	57.1654
SP8	MGA_3	cluster_4	SP4	8647	8874.518	8569	9141	186.5388	0.9744	-0.0375	0.103	0.10748	4956	84386	59.559	917	15111	10.665	514	8647	6.103	10.3713	10.247	56.0523	57.2232
SP4	SPDEF_4	cluster_1	SP8	10597	10878.662	10563	11184	182.4211	0.9741	-0.0378	0.076	0.076	7657	115318	63.966	1228	17521	9.7187	725	10597	5.878	9.4685	9.1894	59.0391	60.4817

SP4	ATF4_3	cluster_1	SP8	4959	5091.91	4895	5286	119.478	0.9739	-0.0382	0.143	0.143	3989	51908	59.647	688	8653	9.943	387	4959	5.6983	9.7017	9.5534	56.25	57.3096
SP8	FOXO4_3	cluster_4	SP4	4030	4138.503	3960	4320	114.3298	0.9738	-0.0383	0.173	0.173	2096	27285	58.087	581	7296	15.532	313	4030	8.5794	14.9332	14.77	53.8726	55.2357
SP8	NFATC1_1	cluster_4	SP4	11566	11878.447	11522	12243	222.9071	0.9737	-0.0385	0.089	0.09287	5066	97783	60.876	1121	20131	12.533	622	11566	7.2005	12.2779	11.8282	55.4862	57.4537
SP8	SOX11_2	cluster_4	SP4	7444	7648.591	7383	7892	145.2217	0.9733	-0.0391	0.078	0.081391	3666	51361	60.532	979	13014	15.338	544	7444	8.7731	14.8391	14.4935	55.5669	57.1999
SP8	RBPJ_2	cluster_4	SP4	8809	9051.421	8809	9270	144.7482	0.9732	-0.0392	0.054	0.054	10072	100834	56.034	1692	16343	9.0819	891	8809	4.8952	8.8463	8.7361	52.6596	53.9008
SP4	HES7_1	cluster_1	SP8	5328	5475.697	5275	5665	118.0669	0.973	-0.0394	0.121	0.121	9737	107540	60.702	845	9092	5.1321	489	5328	3.0075	5.0221	4.9544	57.8698	58.601
SP4	POU2F2_known10	cluster_1	SP8	4559	4688.935	4550	4829	83.8877	0.9723	-0.0405	0.06	0.06	5130	46258	63.552	854	7508	10.315	512	4559	6.2634	9.9805	9.8556	59.9532	60.7219
SP4	SMAD3_3	cluster_1	SP8	2953	3038.506	2897	3158	73.8586	0.9719	-0.0412	0.116	0.116	3934	35416	61.507	570	5013	8.7061	331	2953	5.1285	8.4138	8.338	58.0702	58.9068
SP8	SPIB_2	cluster_4	SP4	28433	29260.857	28760	29802	301.9257	0.9717	-0.0414	0.004	0.004	17242	225293	55.874	4131	52835	13.103	2183	28433	7.0516	12.6609	12.6205	52.8443	53.8147
SP4	FOXO6_3	cluster_1	SP8	24476	25194.403	24752	25658	268.1091	0.9715	-0.0417	0.004	0.004	21804	285200	58.247	3425	43712	8.9273	1886	24476	4.9988	8.6498	8.582	55.0657	55.9938
SP8	ATF4_2	cluster_4	SP4	5463	5625.403	5398	5825	120.7278	0.9711	-0.0423	0.108	0.108	3830	46012	56.648	871	10071	12.399	461	5463	6.7258	12.0366	11.873	52.9277	54.2449
SP8	RUNX2_4	cluster_2	DP	22914	23596.07	23166	24069	279.582	0.9711	-0.0423	0.008	0.008	17076	261045	63.344	2540	37614	9.1272	1516	22914	5.5602	8.878	8.7778	59.685	60.9188
SP8	TCF3_1	cluster_4	SP4	28116	28953.146	28463	29516	330.5532	0.9711	-0.0423	0.004	0.0043636	45409	645019	52.166	4178	56573	4.5754	2016	28116	2.2739	4.4396	4.3589	48.2528	49.6986
SP8	TCF7L2_known5	cluster_4	SP4	30122	31017.849	30462	31499	320.5908	0.9711	-0.0423	0.001	0.0010435	10823	173781	55.479	3628	56623	18.077	1890	30122	9.6164	17.4628	17.3333	52.0948	53.1975
SP8	SP100_1	cluster_4	SP4	982	1011.472	940	1084	42.7232	0.9709	-0.0426	0.276	0.276	956	12440	73.99	115	1406	8.3626	77	982	5.8407	8.0544	7.8939	66.9565	69.8435
DP	SOX13_1	cluster_5	Common_1_DN	4379	4510.449	4295	4724	131.0688	0.9709	-0.0427	0.156	0.156	1690	25424	51.067	667	9165	18.409	298	4379	8.7956	17.6331	17.2239	44.6777	47.7796
SP4	PBX3_known1	cluster_1	SP8	6718	6921.433	6708	7144	131.5471	0.9706	-0.043	0.058	0.058	7076	78591	58.439	1114	11983	8.9104	618	6718	4.9954	8.7337	8.5481	55.4758	56.0628
SP8	NFATC1_3	cluster_4	SP4	3940	4059.562	3891	4237	107.2013	0.9706	-0.0431	0.109	0.109	3018	39257	57.876	597	7250	10.688	311	3940	5.8086	10.3048	10.0364	52.0938	54.3448
SP8	MYC_known13	cluster_4	SP4	6992	7205.664	6921	7466	159.803	0.9704	-0.0434	0.104	0.10852	9016	99281	55.743	1221	13066	7.3361	642	6992	3.9258	7.1207	7.0426	52.5799	53.5129
SP8	TCF12_known1	cluster_4	SP4	14470	14916.459	14687	15186	165.0066	0.9701	-0.0438	0.004	0.0041739	34302	246920	53.035	4034	28399	6.0997	2039	14470	3.108	5.9443	5.8602	50.5454	50.9525
SP4	RREB1_2	cluster_1	SP8	115042	118612.317	117465	119822	738.2954	0.9699	-0.0441	0.001	0.001	51020	1245776	62.597	8721	197558	9.9268	4845	115042	5.7806	9.4963	9.2346	55.5556	58.232
SP4	IKZF1_1	cluster_1	SP8	6781	6996.254	6799	7195	120.9901	0.9692	-0.0451	0.046	0.046	8843	106411	60.935	1030	11778	6.7446	572	6781	3.8831	6.4684	6.3725	55.534	57.5734
SP4	RREB1_1	cluster_1	SP8	76076	78510.52	77684	79234	471.5963	0.969	-0.0454	0.001	0.001	47895	812464	64.416	7972	126523	10.031	4610	76076	6.0317	9.6252	9.3636	57.8274	60.1282
SP4	FOXO3_4	cluster_1	SP8	3096	3196.129	3064	3323	79.6048	0.9687	-0.0459	0.112	0.11687	2012	26245	62.811	419	5251	12.567	242	3096	7.4095	12.0278	11.7965	57.7566	58.9602
SP4	POU2F2_known16	cluster_1	SP8	3646	3767.443	3604	3949	107.4238	0.9678	-0.0473	0.136	0.136	2634	34687	58.999	529	6610	11.243	285	3646	6.2014	10.82	10.5111	53.8752	55.1589
SP8	CTCF_known2	cluster_5	Common_1_DN	60405	62417.724	61535	63341	576.0888	0.9678	-0.0473	0.001	0.0010435	15803	254255	30.429	13031	205911	24.643	3759	60405	7.2292	23.7866	23.7576	28.8466	29.3355
SP4	SOX1_3	cluster_1	SP8	4719	4878.475	4672	5093	123.9289	0.9673	-0.0479	0.106	0.106	3151	44155	58.678	648	8538	11.346	347	4719	6.2711	11.0124	10.6874	53.5494	55.2706
SP4	SOX17_5	cluster_1	SP8	3887	4019.561	3854	4179	101.7659	0.967	-0.0484	0.091	0.094957	2416	33940	61.564	514	6773	12.286	286	3887	7.0506	11.8377	11.4526	55.642	57.3896
SP8	SPIB_1	cluster_4	SP4	13466	13928.253	13691	14144	139.1479	0.9668	-0.0487	0.004	0.004	16443	98658	52.897	4466	26536	14.228	2251	13466	7.2199	13.6897	13.6492	50.403	50.7462
SP4	ETV6_1	cluster_1	SP8	22148	22915.091	22512	23358	254.4311	0.9665	-0.0491	0.001	0.001	27340	393690	65.257	2565	35529	5.8892	1564	22148	3.6712	5.7206	5.6257	60.9747	62.3378
SP4	IKZF3_1	cluster_1	SP8	5195	5374.936	5154	5580	126.073	0.9665	-0.0491	0.081	0.081	6633	79768	59.129	817	9334	6.9189	448	5195	3.8508	6.7541	6.5126	54.8348	55.6567
SP8	NFATC2_1	cluster_4	SP4	3739	3870.986	3736	3984	74.8253	0.9659	-0.05	0.051	0.051	5758	34548	61.898	1082	6319	11.322	633	3739	6.699	10.9934	10.8226	58.5028	59.1708
SP4	SOX13_1	cluster_1	SP8	3682	3812.297	3628	3976	106.7747	0.9658	-0.0502	0.094	0.098087	2170	32640	65.561	443	6166	12.385	257	3682	7.3957	11.8433	11.2806	58.0135	59.7146
SP8	PKNOX1_1	cluster_4	SP4	17163	17791.179	17369	18254	263.873	0.9647	-0.0519	0.012	0.012	10991	165550	53.351	2333	33838	10.905	1146	17163	5.5311	10.4267	10.3673	49.1213	50.7211
SP8	ETV7_1	cluster_4	SP4	30448	31572.468	31154	31973	256.3309	0.9644	-0.0523	0.001	0.001	25471	229674	51.731	6880	61259	13.798	3385	30448	6.858	13.2896	13.2571	49.2006	49.7037
SP4	CUX1_9	cluster_1	SP8	1021	1058.815	976	1144	51.7031	0.9643	-0.0524	0.252	0.252	597	10309	69.627	102	1619	10.935	63	1021	6.8959	10.5528	9.904	61.7647	63.0636
SP4	FOXO1_4	cluster_1	SP8	3503	3632.737	3462	3806	102.7815	0.9643	-0.0525	0.076	0.076	2354	30679	61.609	492	6089	12.228	277	3503	7.0347	11.7672	11.4182	56.3008	57.53
SP8	NFE2L1::MAFG_1	cluster_4	SP4	18768	19463.798	18939	19975	315.3905	0.9643	-0.0525	0.008	0.008	8430	177761	54.694	1843	36408	11.202	916	18768	5.7746	10.866	10.558	49.7016	51.5491
DN	SP8_1	cluster_5	Common_1_DN	47425	49192.254	48421	49950	474.0339	0.9641	-0.0528	0.001	0.001	21179	262744	21.027	20709	242789	19.43	3987	47425	3.7954	18.8253	18.0499	19.2525	19.5334
SP4	NFATC2_1	cluster_1	SP8	3119	3235.434	3136	3345	59.909	0.964	-0.0529	0.028	0.029217	5908	35448	63.511	877	5163	9.2504	528	3119	5.5882	8.937	8.7988	60.2052	60.4106
SP4	IRF_known14	cluster_1	SP8	28593	29670.032	29187	30180	302.0964	0.9637	-0.0533	0.001	0.001	13966	293131	66.532	2296	45729	10.379	1379	28593	6.4897	9.874	9.7543	60.061	62.5271
SP4	SOX8_9	cluster_1	SP8	5329	5531.267	5314	5794	145.9551	0.9634	-0.0537	0.074	0.074	2995	47951	62.253	615	9226	11.978	349	5329	6.9184	11.6528	11.1134	56.748	57.7607
SP8	SOX3_3	cluster_4	SP4	6148	6383.601	6152	6629	148.4507	0.9631	-0.0542	0.048	0.048	2699	43266	55.718	791	11919	15.349	396	6148	7.9174	14.6721	14.2098	50.0632	51.5815
SP8	SOX8_9	cluster_4	SP4	7059	7331.097	7072	7599	152.3768	0.9629	-0.0546	0.033	0.034435	2766	44281	57.488	884	13207	17.146	454	7059	9.1644	16.4136	15.9414	51.3575	53.4489
DN	GMEB2_3	cluster_5	Common_1_DN	5318	5523.918	5397	5668	83.7591	0.9627	-0.0548	0.012	0.012	2360	33394	80.191	509	6963	16.721	382	5318	12.77	16.1864	15.925	75.0491	76.3751
SP8	ARID5B_1	cluster_4	SP4	6376	6624.722	6411	6838	136.6695	0.9625	-0.0552	0.037	0.038609	4547	59233	58.39	937	11641	11.475	501	6376	6.2852	11.0183	10.7643	53.4685	54.7719
SP8	RUNX2_4	cluster_4	SP4	13093	13605.635	13240	13972	219.1587	0.9623	-0.0554	0.001	0.0010435	17076	261045	63.344	1481	21763	5.2809	873	13093	3.1771	5.1124	5.0156	58.9467	60.1617
SP4	MYBL2_4	cluster_1	SP8	1596	1658.801	1557	1763	62.8367	0.9622	-0.0556	0.184	0.184	1678	23553	66.504	186	2524	7.1267	114	1596	4.5064	6.7938	6.7762	61.2903	63.233
SP8	SPI1_known1	cluster_4	SP4	30698	31903.207	31523	32278	231.9891	0.9622	-0.0556	0.001	0.001	35122	247084	52.91	8708	60564	12.969	4379	30698	6.5736	12.468	12.4241	50.2871	50.6869
SP8	TCF7_1	cluster_4	SP4	31817	33071.182	32544	33630	320.7305	0.9621	-0.0558	0.001	0.0010435	10694	171757	56.165	3834	59721	19.529	2002	31817	10.404	18.7208	18.5244	52.217	53.2761
SP8	SOX1_3	cluster_4	SP4	5888	6122.494	5891	6327	132.8272	0.9617	-0.0563	0.044	0.045913	2972	41649	55.348	855	11384	15.128	432	5888	7.8246	14.5357	14.1372	50.5263	51.7217
DP	SOX5_3	cluster_5	Common_1_DN	5253	5465.245	5243	5694	147.0636	0.9612	-0.0571	0.075	0.078261	2038	30641	52.026	789	10931	18.56	358	5253	8.9191	17.5662	17.1437	45.3739	48.056
SP4	ATF4_2	cluster_1	SP8	4212	4382.42	4204	4572	111.4193	0.9611	-0.0572	0.071	0.074087	3978	47794	58.841	650	7549	9.2939	358	4212	5.1856	8.9995	8.8128	55.0769	55.7955
SP8	SOX7_3	cluster_4	SP4	7424	7727.212	7477	8015	159.1561	0.9608	-0.0577	0.016	0.016696	3353	53689	59.073	905	13558	14.918	478	7424	8.1685	14.2559	13.8278	52.8177	54.7573
SP8	SMAD_2	cluster_4	SP4	22525	23447.351	23054	23830	239.1797	0.9607	-0.0579	0.001	0.0010435	21409	221606	55.683	4228	42666	10.721	2204	22525	5.6598	10.2947	10.1644	52.1287	52.7938
SP8	TBX4_2	cluster_4	SP4	12328	12833.625	12429	13238	238.0694	0.9606	-0.058	0.016	0.016696	6718	127933	57.269	1263	22909	10.255	658	12328	5.5186	9.7946	9.6363	52.0982	53.8129
SP8	TCF7_2	cluster_4	SP4	23664	24656.917	24255	25036	249.0395	0.9597	-0.0593	0.001	0.0010435	11953	131925	55.753	4118	44691	18.887	2155	23664	10.001	18.0289	17.9375	52.3312	52.9503
SP8	TBX21_4	cluster_4	SP4	13180	13734.977	13356	14098	226.8487	0.9596	-0.0595	0.008	0.0083478	7628	114668	56.477	1733	24847	12.238	896	13180	6.4915	11.7462	11.4941	51.7023	53.0446
SP8	SPIC_1	cluster_4	SP4	25899	26997.432	26574	27463	276.3795	0.9593	-0.0599	0.001	0.001	14831	193520	54.959	3926	49711	14.118	1999	25899	7.3552	13.4785	13.3831	50.917	52.0991
SP4	FOXO4_3	cluster_1	SP8	3371	3514.754	3337	3667	95.7894	0.9591	-0.0602	0.074	0.077217	2238	29139	62.034	459	5801	12.35	262	3371	7.1765	11.7069	11.5687	57.0806	58.1107
SP8	SOX14_3	cluster_4	SP4	6245	6511.954	6286	6766	145.2541	0.959	-0.0604	0.028	0.029217	3228	45250	55.339	902	12080	14.774	455	6245	7.6375	14.0954	13.8011	50.4435	51.697
SP8	TBX1_4	cluster_4	SP4	17163	17900.006	17447	18374	296.3128	0.9588	-0.0607	0.012	0.012	8594	163899	55.977	1814	32620	11.141	918	17163	5.8618	10.6819	10.4717	50.6064	52.615
SP4	REL_2	cluster_1	SP8	10008	10440.165	10238	10653	131.0417	0.9586	-0.061	0.004	0.004	12661	114508	63.571	1862	16571	9.1996	1111	10008	5.5561	8.775	8.74	59.667	60.3947
SP8	HESX1_1	cluster_4	SP4	1107	1155.069	1065	1232	48.195	0.9584	-0.0613	0.135	0.135	1301	11720	59.084	235	1983	9.997	128	1107	5.5808	9.8386	9.4454	54.4681	55.8245
SP4	RUNX2_4	cluster_2	DP	21713	22669.91	22195	23117	281.4405	0.9578	-0.0622	0.001	0.0010435	16422	250904	60.883	2540	37614	9.1272	1437	21713	5.2687	8.7505	8.6539	56.5748	57.7258
SP4	LMO2_2	cluster_1	SP8	2385	2490.367	2391	2593	61.8312	0.9577	-0.0623	0.044	0.044	3781	30272	59.431	541	4238	8.3202	301	2385	4.6823	7.9609	7.8786	55.6377	56.2765
SP4	E2F2_2	cluster_1	SP8	4952	5170.854	4970	5378	128.3468	0.9577	-0.0624	0.041	0.041	3236	55175	67.435	488	7854	9.5991	298	4952	6.0523	9.2089	8.9751	61.0656	63.0507
SP4	MYBL1_3	cluster_1	SP8	1275	1332.077	1238	1416	53.0519	0.9572	-0.0631	0.146	0.146	1511	16654	62.822	205	2158	8.1403	117	1275	4.8095	7.7432	7.6558	57.0732	59.0825
SP4	SOX15_4	cluster_1	SP8	3796	3967.724	3785	4162	109.6407	0.9567	-0.0638	0.072	0.07513	2335	32728	59.319	529	6964	12.622	282	3796	6.8802	12.0771	11.5986	53.3081	54.5089
SP8	TCF7L1_1	cluster_4	SP4	29108	30425.326	29878	30896	310.029	0.9567	-0.0639	0.001	0.0010435	9778	157054	56.06	3526	55052	19.651	1832	29108	10.39	18.7359	18.5338	51.9569	52.8736
SP8	FOXP1_1	cluster_4	SP4	31152	32570.982	31971	33182	383.3103	0.9564	-0.0643	0.001	0.0010435	10498	244896	63.1	3085	58974	15.195	1523	31152	8.0267	14.5075	12.7205	49.3679	52.8233
SP8	ATF4_3	cluster_4	SP4	6167	6449.831	6239	6667	135.776	0.9562	-0.0647	0.012	0.012522	3790	49324	56.677	930	11616	13.348	483	6167	7.0864	12.7441	12.503	51.9355	53.0906
SP8	RUNX3_1	cluster_2	DP	26772	28000.419	27530	28490	286.53	0.9561	-0.0647	0.001	0.0010435	19753	302988	62.704	3043	45082	9.3299	1763	26772	5.5406	8.9252	8.836	57.9362	59.3851
SP8	ARID5A_1	cluster_4	SP4	1352	1414.506	1331	1508	56.8987	0.9558	-0.0652	0.127	0.127	834	11458	67.591	181	2239	13.208	106	1352	7.9755	12.7098	11.7996	58.5635	60.3841
SP8	RUNX3_1	cluster_4	SP4	14863	15553.754	15181	15968	242.2314	0.9556	-0.0655	0.004	0.004	19753	302988	62.704	1715	25185	5.2121	989	14863	3.076	5.0068	4.9055	57.6676	59.0153
SP8	TCF7L2_known7	cluster_4	SP4	28987	30333.092	29874	30851	304.5807	0.9556	-0.0655	0.001	0.0010435	11335	159258	55.926	4019	55081	19.343	2080	28987	10.179	18.3502	18.2013	51.7542	52.6261
SP8	TCF7L1_2	cluster_4	SP4	21120	22104.461	21728	22530	232.7209	0.9555	-0.0657	0.001	0.0010435	11685	129185	56.684	3643	39382	17.28	1923	21120	9.267	16.457	16.3486	52.7862	53.6286
SP8	IKZF1_2	cluster_4	SP4	12946	13556.549	13316	13804	151.5837	0.955	-0.0665	0.001	0.0010435	20377	163023	59.485	2975	23108	8.4318	1639	12946	4.7238	8.0434	7.9412	55.0924	56.0239
SP4	SOX10_9	cluster_1	SP8	5455	5713.383	5488	5936	132.329	0.9548	-0.0668	0.032	0.032	3684	51623	61.408	721	9597	11.416	403	5455	6.489	10.9392	10.567	55.8946	56.8407
SP4	REL_1	cluster_1	SP8	10165	10647.781	10428	10873	140.5181	0.9547	-0.0669	0.001	0.001	12552	113599	63.434	1898	16902	9.4382	1128	10165	5.6762	8.9866	8.9481	59.431	60.1408
SP4	SMAD4_1	cluster_1	SP8	24462	25623.563	25100	26072	287.9073	0.9547	-0.0669	0.001	0.001	25162	357474	58.021	3229	44540	7.2292	1734	24462	3.9704	6.8913	6.843	53.7008	54.9214
SP4	POU2F2_known13	cluster_1	SP8	5594	5863.188	5606	6130	159.1118	0.9541	-0.0678	0.04	0.041739	2728	57442	64.703	494	9555	10.763	274	5594	6.3011	10.044	9.7385	55.4656	58.5453
SP8	POU2F2_known5	cluster_4	SP4	6297	6602.023	6391	6813	132	0.9538	-0.0682	0.008	0.0083478	3679	47888	58.57	935	11592	14.178	493	6297	7.7016	13.4004	13.1494	52.7273	54.3219
SP4	MTF1_3	cluster_1	SP8	11018	11554.377	11290	11846	166.4386	0.9536	-0.0686	0.001	0.001	12953	170029	62.803	1462	18557	6.8544	849	11018	4.0697	6.5545	6.4801	58.0711	59.3738
SP4	SOX_1	cluster_1	SP8	8406	8826.434	8584	9078	155.2807	0.9524	-0.0704	0.008	0.008	7156	85938	62.408	1257	14434	10.482	718	8406	6.1044	10.0335	9.7815	57.1201	58.2375
SP4	RUNX3_1	cluster_2	DP	25511	26798.716	26325	27302	296.6749	0.952	-0.071	0.001	0.0010435	18902	289786	59.972	3043	45082	9.3299	1681	25511	5.2796	8.8932	8.8034	55.2415	56.588
SP8	FOXO4_2	cluster_4	SP4	10682	11220.919	10963	11486	162.4352	0.952	-0.071	0.001	0.0010435	6205	81377	63.594	1470	18241	14.255	843	10682	8.3477	13.5858	13.1266	57.3469	58.5604
SP8	IKZF2_3	cluster_4	SP4	6881	7230.508	7038	7398	112.1903	0.9517	-0.0715	0.004	0.0041739	5533	55534	62.869	1246	11893	13.464	707	6881	7.7898	12.7779	12.3906	56.7416	57.8576
DN	KLF14_1	cluster_5	Common_1_DN	38425	40405.211	39728	41277	454.0337	0.951	-0.0725	0.001	0.001	16591	224164	22.673	14140	183594	18.57	2952	38425	3.8865	17.7928	17.1415	20.8769	20.9293
SP8	GATA_known17	cluster_4	SP4	9356	9839.787	9526	10189	194.9289	0.9508	-0.0727	0.012	0.012522	3680	58991	55.43	1185	18187	17.089	596	9356	8.7913	16.1957	15.86	50.2954	51.4433
SP4	MYBL1_5	cluster_1	SP8	3044	3203.386	3046	3358	97.1853	0.9503	-0.0736	0.048	0.048	2888	46605	66.715	322	4890	7	193	3044	4.3575	6.6828	6.5315	59.9379	62.2495
SP4	RUNX_1	cluster_2	DP	23655	24893.046	24319	25427	333.239	0.9503	-0.0736	0.001	0.0010435	22871	322027	50.309	3658	49981	7.8084	1690	23655	3.6955	7.3893	7.3457	46.2001	47.328
SP8	CBX5_1	cluster_4	SP4	6393	6732.217	6548	6915	118.2624	0.9496	-0.0746	0.001	0.0010435	4252	46829	61.603	1068	11228	14.77	594	6393	8.4099	13.9699	13.6518	55.618	56.938
SP8	POU2F2_known16	cluster_4	SP4	4550	4792.822	4607	4979	110.0859	0.9493	-0.075	0.008	0.0083478	2431	32048	54.51	737	9083	15.449	352	4550	7.739	14.4796	14.1975	47.7612	50.0936
DN	EGR3_2	cluster_5	Common_1_DN	32150	33871.172	33091	34689	489.7603	0.9492	-0.0752	0.001	0.001	13370	205199	20.858	11617	169240	17.203	2228	32150	3.268	16.6642	15.6677	19.1788	18.9967
SP4	RUNX_2	cluster_2	DP	19455	20500.96	20057	20948	269.461	0.949	-0.0755	0.001	0.0010435	18786	264509	50.895	2986	40745	7.8399	1389	19455	3.7434	7.3938	7.3551	46.5171	47.7482
DN	SP100_1	cluster_2	DP	1001	1054.887	996	1103	32.2935	0.949	-0.0756	0.066	0.072	1107	14404	85.672	101	1262	7.5061	78	1001	5.9537	7.0461	6.9495	77.2277	79.3185
SP8	SOX5_3	cluster_4	SP4	5485	5780.392	5561	5985	129.5816	0.9489	-0.0757	0.024	0.026182	2486	37371	63.453	690	9567	16.244	378	5485	9.313	15.2051	14.6772	54.7826	57.3325
DP	SOX3_3	cluster_5	Common_1_DN	6532	6889.699	6625	7160	169.1059	0.9481	-0.0769	0.012	0.012522	2264	36279	46.72	1032	15271	19.666	424	6532	8.4119	18.7279	18.0049	41.0853	42.7739
SP8	TBX5_5	cluster_4	SP4	11501	12139.047	11780	12551	235.7028	0.9474	-0.0779	0.001	0.0010435	5748	109382	56.634	1206	21904	11.341	615	11501	5.9548	10.6994	10.5145	50.995	52.5064
SP4	IRF4_2	cluster_1	SP8	12267	12952.096	12658	13204	173.5681	0.9471	-0.0784	0.001	0.001	6862	99125	67.779	1355	19304	13.2	847	12267	8.3879	12.3433	12.3753	62.5092	63.5464
DN	PBX1_4	cluster_2	DP	2905	3068.707	2939	3200	74.2232	0.9467	-0.0791	0.008	0.0083478	3459	38700	71.853	409	4372	8.1173	266	2905	5.3936	7.6901	7.5065	65.0367	66.4456
SP4	ARID5A_1	cluster_5	Common_1_DN	1858	1962.812	1871	2079	64.7931	0.9466	-0.0791	0.032	0.034435	810	11102	65.491	253	3206	18.912	142	1858	10.96	17.5309	16.7357	56.1265	57.9538
SP4	E2F7_1	cluster_1	SP8	4510	4766.402	4582	4942	109.459	0.9462	-0.0798	0.008	0.008	4567	59426	65.789	603	7441	8.2378	353	4510	4.9929	7.7294	7.5893	58.5406	60.6101
DP	CUX1_8	cluster_5	Common_1_DN	1091	1153.35	1042	1264	65.1084	0.946	-0.0801	0.151	0.151	409	6571	49.473	164	2468	18.582	70	1091	8.2141	17.1149	16.6033	42.6829	44.2058
SP8	IRF6_1	cluster_4	SP4	4462	4718.495	4534	4923	118.2457	0.9457	-0.0806	0.02	0.02	2749	44071	67.262	466	7185	10.966	282	4462	6.81	10.2583	10.1246	60.515	62.1016
SP4	IRF4_1	cluster_2	DP	2897	3065.194	2911	3204	88.7204	0.9451	-0.0814	0.03	0.03	3307	46509	68.576	341	4540	6.6941	211	2897	4.2715	6.3804	6.2289	61.8768	63.8106
SP8	RUNX2_5	cluster_4	SP4	14159	14984.451	14574	15375	232.8571	0.9449	-0.0817	0.001	0.001	18306	320965	63.474	1441	24027	4.7516	823	14159	2.8001	4.4958	4.4114	57.1131	58.9295
SP8	ELK4_2	cluster_4	SP4	6448	6827.693	6572	7108	166.1158	0.9444	-0.0825	0.012	0.013091	7080	64048	23.72	3236	28879	10.695	717	6448	2.388	10.1271	10.0674	22.157	22.3276
SP4	CTCF_known2	cluster_5	Common_1_DN	56117	59429.68	58436	60437	579.8456	0.9443	-0.0827	0.001	0.0010435	15039	241923	28.953	13031	205911	24.643	3492	56117	6.7161	23.2196	23.1962	26.7976	27.253
SP8	TBX20_2	cluster_4	SP4	10059	10656.173	10316	11041	216.9964	0.944	-0.0832	0.001	0.0010435	5480	98825	56.841	1131	19160	11.02	572	10059	5.7857	10.438	10.1786	50.5747	52.5
SP8	ETV2_1	cluster_4	SP4	22399	23729.931	23389	24103	217.2557	0.9439	-0.0833	0.001	0.0010435	16594	166680	56.55	4238	42029	14.259	2235	22399	7.5994	13.4687	13.4383	52.7371	53.2942
SP8	RREB1_1	cluster_4	SP4	71327	75621.979	74799	76409	468.7944	0.9432	-0.0844	0.001	0.001	48842	818149	64.867	7266	119347	9.4625	4165	71327	5.6552	8.5275	8.7181	57.3218	59.7644
SP4	SOX7_4	cluster_1	SP8	4548	4823.106	4618	5025	128.1693	0.943	-0.0847	0.032	0.033391	2839	45561	60.885	551	8220	10.985	294	4548	6.0777	10.3558	9.9822	53.3575	55.3285
SP4	RUNX3_1	cluster_1	SP8	21534	22839.553	22353	23319	294.2171	0.9428	-0.0849	0.001	0.0010435	18902	289786	59.972	2579	38479	7.9634	1416	21534	4.4565	7.4913	7.431	54.905	55.963
SP8	MYBL1_3	cluster_4	SP4	1530	1623.614	1512	1728	65.9698	0.9424	-0.0856	0.087	0.090783	1462	16095	60.713	257	2708	10.215	142	1530	5.7714	9.7127	9.5061	55.2529	56.4993
SP4	SP100_1	cluster_2	DP	865	918.498	857	976	36.2938	0.9418	-0.0865	0.074	0.077217	962	12518	74.454	101	1262	7.5061	67	865	5.1448	6.9647	6.91	66.3366	68.542
SP4	ELK3_2	cluster_1	SP8	3032	3220.733	3007	3411	119.8296	0.9414	-0.0871	0.072	0.07513	6107	55145	26.227	1388	12358	5.8774	338	3032	1.442	5.5346	5.4982	24.3516	24.5347
SP8	POU2F3_2	cluster_4	SP4	3640	3868.532	3747	3986	74.7542	0.9409	-0.0878	0.008	0.0083478	3384	27131	59.282	848	6599	14.419	460	3640	7.9535	13.5934	13.4164	54.2453	55.1599
DP	RUNX1_4	cluster_5	Common_1_DN	4488	4770.207	4577	4951	119.3178	0.9409	-0.088	0.012	0.012522	2351	23810	47.054	1062	10306	20.367	449	4488	8.8694	19.0983	18.8492	42.2787	43.5474
SP8	FOXO1_5	cluster_4	SP4	22480	23897.642	23507	24325	251.1242	0.9407	-0.0882	0.001	0.0010435	25271	279379	58.574	3867	41421	8.6842	2059	22480	4.7131	8.1477	8.0464	53.2454	54.272
SP4	IRF_known21	cluster_1	SP8	14879	15822.058	15511	16172	209.4676	0.9404	-0.0887	0.001	0.001	8196	119181	67.65	1643	23596	13.394	1015	14879	8.4457	12.3841	12.4844	61.7772	63.0573
DN	LMO2_1	cluster_6	Common_2	58898	62666.279	61856	63526	480.9419	0.9399	-0.0895	0.001	0.001	22843	252436	30.177	19245	210148	25.122	5371	58898	7.0408	23.5127	23.3319	27.9085	28.0269
SP8	HMGA1_1	cluster_4	SP4	2859	3043.547	2941	3144	61.074	0.9394	-0.0902	0.001	0.0010435	3543	21381	63.056	821	4883	14.401	479	2859	8.4316	13.5196	13.3717	58.3435	58.5501
DP	CUX1_9	cluster_5	Common_1_DN	1053	1121.26	1013	1243	65.3567	0.9392	-0.0905	0.137	0.14296	440	7588	51.249	144	2263	15.284	64	1053	7.112	14.5455	13.8772	44.4444	46.5312

SP8	FOXO6_3	cluster_4	SP4	22335	23787.522	23363	24219	268.8822	0.9389	-0.0909	0.001	0.0010435	22108	289208	59.065	3237	40909	8.3549	1733	22335	4.5615	7.8388	7.7228	53.5372	54.5968
SP8	FOXO3_1	cluster_1	SP8	9518	10139.079	9916	10413	154.0583	0.9388	-0.0912	0.001	0.0010435	5381	70700	64.693	1311	16241	14.861	753	9518	8.7093	13.9937	13.4625	57.4371	58.6048
SP8	FOXO6_1	cluster_1	SP8	3770	4018.798	3855	4212	108.5657	0.9381	-0.0922	0.012	0.012522	2688	35069	61.166	544	6763	11.796	297	3770	6.5755	11.0491	10.7502	54.5956	55.7445
SP4	IRF4_1	cluster_1	SP8	4460	4754.704	4581	4924	99.3406	0.938	-0.0923	0.001	0.0010435	3307	46509	68.576	513	7035	10.373	319	4460	6.5761	9.6462	9.5895	62.1832	63.3973
SP4	STAT_known2	cluster_1	SP8	9200	9807.938	9496	10142	190.9056	0.938	-0.0923	0.001	0.001	9013	180903	68.592	782	14719	5.5809	471	9200	3.4883	5.2258	5.0856	60.2302	62.5042
SP8	SOX12_1	cluster_4	SP4	5896	6285.83	6057	6531	134.233	0.938	-0.0924	0.001	0.0010435	2969	38740	61.39	869	10644	16.867	466	5896	9.3432	15.6955	15.2194	53.6249	55.3927
SP4	MYCN_1	cluster_1	SP8	6314	6732.954	6514	6921	122.403	0.9378	-0.0927	0.001	0.001	10339	114073	63.425	997	10742	5.9726	574	6314	3.5106	5.5518	5.5351	57.5727	58.7786
SP4	TCF4_1	cluster_2	DP	47585	50741.838	50175	51378	363.0319	0.9378	-0.0927	0.001	0.0010909	33174	299877	45.732	12410	111339	16.979	5260	47585	7.2568	15.8558	15.8682	42.3852	42.7388
SP8	GATA_known16	cluster_4	SP4	15529	16566.058	16044	17018	290.1089	0.9374	-0.0933	0.001	0.0010435	4509	95037	55.067	1561	30959	17.939	755	15529	8.9979	16.7443	16.34	48.3664	50.1599
SP4	TCF3_2	cluster_2	DP	56204	60012.568	59113	60799	494.311	0.9365	-0.0946	0.001	0.0010435	23468	354325	46.199	8790	130696	17.041	3727	56204	7.3282	15.8812	15.8623	42.4005	43.0036
SP8	E2F2_2	cluster_4	SP4	5304	5668.373	5441	5891	137.3728	0.9357	-0.0958	0.004	0.0041739	3179	54200	66.243	551	8817	10.776	320	5304	6.4825	10.0661	9.786	58.0762	60.1565
SP8	GMEB1_1	cluster_2	DP	9287	9924.824	9665	10164	148.9685	0.9357	-0.0958	0.001	0.0010435	6106	99511	72.498	862	13770	10.032	572	9287	6.7659	9.3678	9.3326	66.3573	67.4437
SP4	E2F8_1	cluster_1	SP8	6469	6913.741	6706	7107	122.1052	0.9357	-0.0959	0.001	0.001	7471	82374	63.953	1040	11009	8.5472	596	6469	5.0224	7.9775	7.8532	57.3077	58.761
SP4	XBP1_4	cluster_1	SP8	4518	4829.536	4675	5042	110.4446	0.9355	-0.0962	0.001	0.0010435	5207	67800	61.985	616	7855	7.1813	348	4518	4.1305	6.6833	6.6637	56.4935	57.5175
SP8	SPZ1_2	cluster_4	SP4	11914	12735.539	12445	13025	183.529	0.9355	-0.0962	0.001	0.0010435	13891	139218	56.526	2339	22816	9.2638	1202	11914	4.8373	8.6531	8.5578	51.3895	52.2177
DN	LMO2_1	cluster_5	Common_1_DN	60186	64374.745	63665	65111	456.0311	0.9349	-0.0971	0.001	0.001	22843	252436	30.177	19801	215266	25.733	5528	60186	7.1948	24.2	23.8421	27.9178	27.9589
SP4	RUNX2_5	cluster_1	SP8	20978	22438.705	21978	22920	287.3042	0.9349	-0.0971	0.001	0.001	17450	305925	60.5	2260	37691	7.4537	1233	20978	4.1486	7.0659	6.8572	54.5575	55.6578
SP4	BATF_known1	cluster_1	SP8	4482	4794.703	4610	4980	116.4169	0.9348	-0.0973	0.008	0.008	3974	51803	59.832	638	8084	9.3369	347	4482	5.1767	8.7318	8.652	54.3887	55.4429
SP4	CUX2_2	cluster_1	SP8	429	459.339	418	499	24.1994	0.9341	-0.0984	0.117	0.12209	387	3483	71.8	79	675	13.915	50	429	8.8435	12.9199	12.317	63.2911	63.5556
SP8	POU2F3_1	cluster_4	SP4	7124	7627.916	7354	7884	158.2913	0.9339	-0.0986	0.001	0.0010435	3586	54118	58.145	940	13469	14.471	483	7124	7.6541	13.469	13.1638	51.383	52.8918
SP8	ELK3_2	cluster_4	SP4	4441	4755.901	4510	4988	143.3778	0.9338	-0.0988	0.012	0.013091	5710	51570	24.526	2198	19577	9.3107	494	4441	2.1121	8.6515	8.6116	22.475	22.6848
SP8	IRF4_1	cluster_4	SP4	4988	5342.95	5152	5520	111.1512	0.9336	-0.0992	0.001	0.0010435	3249	45692	67.371	591	8045	11.862	357	4988	7.3547	10.988	10.9166	60.4061	62.0012
SP8	SMAD3_1	cluster_4	SP4	16877	18086.011	17809	18403	178.6839	0.9332	-0.0998	0.001	0.0010435	19958	165967	56.427	3964	32413	11.02	2047	16877	5.738	10.2565	10.1689	51.6398	52.0686
SP8	IKZF1_1	cluster_4	SP4	7190	7705.453	7470	7945	142.3911	0.9331	-0.0999	0.001	0.0010435	8739	105142	60.209	1153	13125	7.5159	614	7190	4.1173	7.026	6.8384	53.2524	54.781
SP8	RREB1_2	cluster_4	SP4	102140	109511.292	108346	110609	702.6879	0.9327	-0.1005	0.001	0.001	51915	1257959	63.21	7941	179955	9.0423	4231	102140	5.1323	8.1499	8.1195	53.2804	56.7586
SP8	CUX2_1	cluster_4	SP4	1037	1112.337	1032	1202	52.2907	0.9323	-0.1011	0.07	0.076364	434	7992	75.035	110	1680	15.773	63	1037	9.7362	14.5161	14.1025	57.2727	61.7262
SP4	IRF6_1	cluster_1	SP8	3760	4033.276	3868	4216	103.1957	0.9323	-0.1012	0.001	0.0010909	2762	44279	67.58	391	6065	9.2566	237	3760	5.7386	8.5807	8.4916	60.6138	61.9951
DP	RUNX1_2	cluster_5	Common_1_DN	9910	10633.281	10314	10932	199.3907	0.932	-0.1016	0.001	0.0010435	3573	55648	49.329	1533	22215	19.692	657	9910	8.7846	18.3879	17.8084	42.8571	44.6095
DN	TCF3_1	cluster_5	Common_1_DN	65172	69955.331	68896	71097	632.8072	0.9316	-0.1022	0.001	0.0011429	20600	290144	23.465	21819	302495	24.464	4742	65172	5.2708	23.0194	22.4619	21.7334	21.5448
SP8	CUX1_9	cluster_4	SP4	1635	1755.399	1649	1881	68.3918	0.9315	-0.1024	0.028	0.032	577	9969	67.331	174	2765	18.675	100	1635	11.043	17.331	16.4008	57.4713	59.132
DN	IRF_known21	cluster_2	DP	7713	8289.774	8098	8481	126.1827	0.9304	-0.104	0.001	0.001	9344	135063	76.665	824	11147	6.3273	558	7713	4.3781	5.9717	5.7107	67.7184	69.1935
SP4	ETV3_1	cluster_1	SP8	7247	7789.473	7619	7975	109.1926	0.9304	-0.1041	0.001	0.0010435	14073	127252	67.378	1310	11627	6.1563	808	7247	3.8372	5.7415	5.695	61.6794	62.3291
SP8	HMGA1_2	cluster_4	SP4	10296	11069.284	10795	11325	161.4339	0.9301	-0.1045	0.001	0.0010435	5565	78050	63.498	1373	18136	14.755	755	10296	8.3763	13.5669	13.1915	54.9891	56.7711
SP8	MTF1_3	cluster_4	SP4	13221	14223.72	13857	14519	197.5033	0.9295	-0.1055	0.001	0.001	12742	167257	61.78	1830	23295	8.6045	1017	13221	4.8834	7.9815	7.9046	55.5738	56.7547
SP4	RUNX2_5	cluster_2	DP	26248	28241.142	27742	28700	297.9968	0.9294	-0.1056	0.001	0.001	17450	305925	60.5	2750	46946	9.284	1496	26248	5.1908	8.5731	8.5799	54.4	55.911
SP8	MAF_known1	cluster_4	SP4	36529	39308.272	38708	39965	383.4718	0.9293	-0.1058	0.001	0.001	25525	385561	56.181	4884	71057	10.354	2436	36529	5.3228	9.5436	9.4742	49.8771	51.408
DP	HMGA1_2	cluster_5	Common_1_DN	11980	12893.912	12575	13199	194.5155	0.9291	-0.1061	0.001	0.0010435	4747	66572	54.16	1898	24720	20.111	887	11980	9.7463	18.6855	17.9956	46.7334	48.4628
SP8	TBX2_1	cluster_4	SP4	12263	13199.933	12856	13557	214.2874	0.929	-0.1062	0.001	0.0010435	8536	145462	59.643	1365	22454	9.2067	729	12263	5.0281	8.5403	8.4304	53.4066	54.6139
DP	SOX18_4	cluster_5	Common_1_DN	4336	4673.203	4476	4890	131.4416	0.9279	-0.108	0.012	0.012522	1791	25128	47.709	773	10090	19.157	318	4336	8.2325	17.7554	17.2557	41.1384	42.9732
SP8	IRF4_2	cluster_4	SP4	12429	13396.285	13080	13704	181.4083	0.9278	-0.1081	0.001	0.001	6695	96589	66.045	1455	20483	14.006	860	12429	8.4986	12.8454	12.8679	59.1065	60.6796
SP8	POU2F2_known15	cluster_4	SP4	4562	4916.935	4744	5115	107.2026	0.9278	-0.1081	0.001	0.0010435	3075	33949	59.103	800	8497	14.793	418	4562	7.9422	13.5935	13.4378	52.25	53.6895
SP8	RUNX_1	cluster_2	DP	24035	25905.765	25355	26431	309.0044	0.9278	-0.1081	0.001	0.0010435	23767	334786	52.302	3658	49981	7.8084	1720	24035	3.7549	7.2369	7.1792	47.0202	48.0883
SP8	MTF1_1	cluster_1	SP8	23641	25490.529	25102	25881	255.1747	0.9274	-0.1087	0.001	0.001	38636	515202	65.297	3054	39477	5.0033	1797	23641	2.9963	4.6511	4.5867	58.8409	59.8855
SP8	SMAD_1	cluster_4	SP4	11360	12256.143	12001	12517	160.5902	0.9269	-0.1095	0.001	0.0010435	15801	126448	57.942	2718	21297	9.7589	1428	11360	5.2055	9.0374	8.9839	52.5386	53.3408
SP8	SOX10_9	cluster_4	SP4	7101	7664.971	7395	7943	160.1099	0.9264	-0.1102	0.001	0.0010435	3412	47797	56.857	1036	13825	16.446	515	7101	8.447	15.0938	14.8566	49.7104	51.3635
SP8	ELK3_1	cluster_4	SP4	4477	4832.635	4579	5060	141.8683	0.9264	-0.1103	0.016	0.016696	5938	53644	24.833	2201	19599	9.0727	498	4477	2.0725	8.3867	8.3458	22.6261	22.843
SP8	IKZF2_2	cluster_4	SP4	3443	3717.516	3579	3869	88.6152	0.9262	-0.1106	0.001	0.0010435	3469	34690	57.903	694	6615	11.041	353	3443	5.7469	10.1758	9.9251	50.8646	52.0484
DN	CUX1_9	cluster_2	DP	641	692.546	621	763	43.6677	0.9257	-0.1114	0.124	0.13527	631	10867	73.396	63	1004	6.781	38	641	4.3293	6.0222	5.8986	60.3175	63.8446
SP4	RUNX2_4	cluster_1	SP8	17556	18965.274	18590	19373	239.7288	0.9257	-0.1114	0.001	0.0010435	16422	250904	60.883	2118	31471	7.6366	1164	17556	4.26	7.0881	6.9147	54.9575	55.7847
SP8	E2F_known26	cluster_4	SP4	2470	2668.291	2510	2802	88.3923	0.9257	-0.1114	0.008	0.008	1668	28373	65.605	267	4221	9.76	152	2470	5.7112	9.1127	8.7055	56.9288	58.5169
SP8	SOX13_1	cluster_4	SP4	4224	4564.132	4389	4739	107.8891	0.9255	-0.1117	0.001	0.0010909	2089	31443	63.156	555	7626	15.318	294	4224	8.4843	14.0737	13.4338	52.973	55.3895
SP4	MYC_known20	cluster_1	SP8	8695	9397.388	9100	9672	187.5178	0.9253	-0.1121	0.001	0.001	8841	144867	57.464	1049	16496	6.5434	532	8695	3.449	6.0174	6.0021	50.715	52.7097
SP4	NFKB_known10	cluster_1	SP8	11069	11968.833	11669	12271	179.1865	0.9248	-0.1127	0.001	0.001	10445	126213	62.637	1617	19222	9.5395	918	11069	5.4933	8.7889	8.7701	56.7718	57.5851
SP4	CUX1_8	cluster_1	SP8	1073	1160.522	1066	1258	58.5069	0.9246	-0.113	0.067	0.069913	545	8762	65.969	122	1836	13.823	67	1073	8.0786	12.2936	12.2461	54.918	58.4423
DP	SOX17_5	cluster_5	Common_1_DN	4692	5078.98	4866	5290	126.3847	0.9238	-0.1143	0.004	0.0043636	1842	25857	46.902	850	11148	20.221	347	4692	8.5108	18.8382	18.146	40.8235	42.0883
SP4	ATF3_known5	cluster_1	SP8	13258	14353.658	13996	14712	216.893	0.9237	-0.1145	0.001	0.001	11915	167390	58.274	1826	24906	8.6706	955	13258	4.6155	8.0151	7.9204	52.3001	53.2322
SP8	RUNX3_3	cluster_2	DP	35827	38801.451	38197	39366	362.3869	0.9233	-0.1151	0.001	0.001	22811	405311	62.285	3611	62662	9.6295	2013	35827	5.5057	8.8247	8.8394	55.7463	57.175
SP4	ETS_known17	cluster_1	SP8	10961	11875.386	11538	12203	211.0793	0.923	-0.1156	0.001	0.001	12925	220877	62.412	1185	19310	5.4563	653	10961	3.0972	5.0522	4.9625	55.1055	56.7633
SP4	SOX3_3	cluster_1	SP8	4652	5040.652	4815	5268	136.7199	0.9229	-0.1157	0.001	0.0010909	2900	46500	59.883	591	8743	11.259	302	4652	5.9908	10.4138	10.0043	51.0998	53.2083
SP8	IRF4_1	cluster_2	DP	2781	3021.599	2877	3173	88.2176	0.9204	-0.1197	0.001	0.0010435	3249	45692	67.371	341	4540	6.6941	200	2781	4.1005	6.1557	6.0864	58.651	61.2555
SP8	SP100_1	cluster_2	DP	835	907.526	840	976	41.6475	0.9202	-0.12	0.036	0.041143	956	12440	73.99	101	1262	7.5061	65	835	4.9664	6.7992	6.7122	64.3564	66.1648
SP4	ERG_4	cluster_1	SP8	5435	5907.201	5684	6107	126.4891	0.9201	-0.1202	0.001	0.001	7152	93169	64.953	724	9199	6.4131	420	5435	3.789	5.8725	5.8335	58.011	59.0825
SP8	RUNX_2	cluster_2	DP	19731	21446.718	20957	21932	289.1716	0.92	-0.1203	0.001	0.0010435	19635	276608	53.223	2986	40745	7.8399	1409	19731	3.7965	7.176	7.1332	47.1869	48.4256
DP	SOX3_1	cluster_5	Common_1_DN	4078	4433.543	4205	4650	135.7764	0.9198	-0.1206	0.001	0.0010909	1698	27182	49.21	651	9351	16.929	270	4078	7.3827	15.9011	15.0026	41.4747	43.6103
SP4	NFKB_known11	cluster_1	SP8	13622	14818.489	14541	15079	173.6533	0.9193	-0.1215	0.001	0.001	11939	145036	63.244	1970	23567	10.277	1124	13622	5.94	9.4145	9.3922	57.0558	57.8012
SP8	SPDEF_4	cluster_4	SP4	15443	16800.893	16416	17241	241.347	0.9192	-0.1216	0.001	0.0010435	7014	105694	58.627	2070	29469	16.346	1056	15443	8.5661	15.0556	14.611	51.0145	52.4042
SP8	RUNX1_4	cluster_4	SP4	4843	5269.297	5097	5439	109.5599	0.9191	-0.1217	0.001	0.0010909	2766	28019	55.372	998	9723	19.215	484	4843	9.571	17.4982	17.2847	48.497	49.8097
DP	SOX4_2	cluster_5	Common_1_DN	10793	11745.594	11374	12137	213.9357	0.9189	-0.122	0.001	0.0010909	3926	59025	51.393	1660	23480	20.444	733	10793	9.3976	18.6704	18.2855	44.1566	45.9668
SP4	KLF12_1	cluster_1	SP8	7974	8681.902	8487	8864	112.5834	0.9185	-0.1227	0.001	0.001	19302	115812	58.735	2507	14856	7.5344	1336	7974	4.0441	6.9216	6.8853	53.2908	53.6753
SP4	LMO2_1	cluster_2	DP	56670	61715.541	60971	62426	433.6232	0.9182	-0.123	0.001	0.001	35624	395160	47.238	11969	131187	15.682	5109	56670	6.7744	14.3415	14.341	42.6853	43.1979
DP	ARID5A_1	cluster_5	Common_1_DN	1450	1579.82	1460	1698	71.0703	0.9179	-0.1236	0.033	0.034435	647	8899	52.495	253	3206	18.912	110	1450	8.5536	17.0015	16.294	43.4783	45.2277
SP4	SPDEF_6	cluster_1	SP8	4719	5142.442	4972	5317	107.4459	0.9177	-0.1239	0.001	0.001	8360	117397	67.827	572	7714	4.4568	342	4719	2.7264	4.0909	4.0197	59.7902	61.1745
SP8	RUNX3_3	cluster_2	DP	37170	40578.779	40047	41166	350.1001	0.916	-0.1266	0.001	0.001	23891	424439	65.225	3611	62662	9.6295	2098	37170	5.712	8.7815	8.7574	58.1002	59.3182
SP4	SP1_known3	cluster_1	SP8	29906	32671.225	32209	33090	276.4482	0.9154	-0.1276	0.001	0.001	39133	513297	64.396	4032	51385	6.4465	2307	29906	3.7519	5.8953	5.8263	57.2173	58.1999
SP8	SOX4_1	cluster_4	SP4	26392	28836.06	28272	29334	313.5301	0.9152	-0.1278	0.001	0.0010435	11786	191082	60.682	3207	48978	15.554	1681	26392	8.3813	14.2627	13.8119	52.4166	53.8854
SP8	RUNX2_5	cluster_2	DP	27076	29586.295	29041	30082	305.8695	0.9152	-0.1279	0.001	0.001	18306	320965	63.474	2750	46946	9.284	1548	27076	5.3545	8.4562	8.4358	56.2909	57.6748
DP	POU2F2_known13	cluster_5	Common_1_DN	7654	8365.532	8021	8747	208.0536	0.915	-0.1282	0.001	0.0010909	2228	46947	52.881	863	16530	18.619	379	7654	8.6215	17.0108	16.3035	43.9166	46.3037
SP8	CUX2_1	cluster_5	Common_1_DN	977	1068.261	985	1152	52.2779	0.9147	-0.1287	0.032	0.036571	434	7992	75.035	100	1592	14.947	59	977	9.1728	13.5945	12.2247	59	61.3693
SP8	SOX11_1	cluster_4	SP4	24751	27060.593	26583	27623	315.7809	0.9147	-0.1287	0.001	0.0010435	10921	177105	60.65	3012	45946	15.734	1580	24751	8.476	14.4675	13.9753	52.4568	53.8698
SP4	ELK3_1	cluster_1	SP8	2974	3252.102	3056	3433	113.1704	0.9145	-0.1289	0.008	0.0087273	6270	56640	26.22	1397	12448	5.7624	332	2974	1.3767	5.2951	5.2507	23.7652	23.8914
SP8	HBP1_1	cluster_4	SP4	7955	8699.07	8424	8956	164.8339	0.9145	-0.129	0.001	0.0010435	4130	63662	59.134	1056	15222	14.139	531	7955	7.3892	12.8571	12.4957	50.2841	52.2599
SP8	PBX1_4	cluster_4	SP4	4180	4573.398	4399	4754	103.6936	0.914	-0.1297	0.001	0.0010909	2771	31106	57.753	750	8086	15.013	378	4180	7.7609	13.6413	13.4379	50.4	51.6943
DP	HESX1_2	cluster_5	Common_1_DN	1922	2103.125	1970	2256	90.756	0.9139	-0.1299	0.012	0.013714	733	10262	51.563	323	4233	21.269	140	1922	9.6573	19.0996	18.7293	43.3437	45.4052
SP8	IRF_known14	cluster_4	SP4	35047	38357.43	37708	38997	398.8017	0.9137	-0.1302	0.001	0.001	13279	278624	63.239	3125	62308	14.142	1705	35047	7.9546	12.8398	12.5786	54.56	56.248
DN	HESX1_2	cluster_2	DP	915	1002.116	936	1074	40.2163	0.9132	-0.1311	0.016	0.017455	1143	16019	80.489	105	1325	6.6576	69	915	4.5975	6.0367	5.712	65.7143	69.0566
SP4	ID4_1	cluster_2	DP	41080	44990.952	44489	45489	303.8916	0.9131	-0.1312	0.001	0.0010435	30226	273311	46.788	10793	96618	16.54	4546	41080	7.0324	15.04	15.0305	42.1199	42.518
SP8	LMO2_2	cluster_4	SP4	3164	3466.269	3351	3594	77.216	0.9128	-0.1316	0.001	0.0010435	3634	29090	57.111	783	6146	12.066	398	3164	6.2117	10.9521	10.8766	50.8301	51.4806
SP4	CUX2_2	cluster_5	Common_1_DN	445	531.444	487	575	25.9981	0.9128	-0.1317	0.041	0.046857	387	3483	71.8	97	790	16.285	59	485	9.9979	15.2455	13.9248	60.8247	61.3924
SP4	MAFF_1	cluster_4	SP4	12468	13660.443	13317	14013	209.2834	0.9127	-0.1318	0.001	0.001	9420	132144	52.834	1924	26154	10.457	893	12468	4.9849	9.4798	9.4352	46.4137	47.6715
SP8	SOX_1	cluster_4	SP4	10630	11647.604	11396	11898	158.8206	0.9126	-0.1319	0.001	0.0010435	6625	79556	57.773	1772	20522	14.903	894	10630	7.7195	13.4943	13.3617	50.4515	51.7981
DP	SOX12_1	cluster_5	Common_1_DN	5234	5736.806	5526	5963	136.611	0.9124	-0.1323	0.001	0.0010435	2452	31970	50.662	975	11756	18.629	413	5234	8.2941	16.8434	16.3716	42.359	44.5219
SP8	POU2F2_known1	cluster_4	SP4	7326	8035.466	7752	8309	174.8395	0.9117	-0.1333	0.001	0.0010435	3160	57168	59.186	825	13977	14.47	417	7326	7.5846	13.1962	12.8149	50.5455	52.4147
SP8	SOX30_1	cluster_4	SP4	6594	7234.903	6978	7510	150.6862	0.9114	-0.1338	0.001	0.0010435	3553	53360	60.089	872	12336	13.892	452	6594	7.4255	12.7216	12.3576	51.8349	53.4533
SP4	ERG_2	cluster_1	SP8	5384	5908.607	5714	6085	119.2159	0.9112	-0.1341	0.001	0.001	7181	93517	64.636	729	9239	6.3857	417	5384	3.7212	5.807	5.7572	57.2016	58.2747
SP8	POU2F2_known10	cluster_4	SP4	5835	6403.616	6229	6572	102.7149	0.9112	-0.1341	0.001	0.0010435	4821	43499	59.761	1251	10899	14.974	656	5835	8.0164	13.6071	13.4141	52.438	53.537
SP4	ETV1_1	cluster_1	SP8	2210	2425.638	2256	2592	93.9609	0.9111	-0.1343	0.012	0.012522	5001	45274	25.882	1065	9493	5.4269	246	2210	1.2634	4.919	4.8814	23.0986	23.2803
SP4	MYCN_2	cluster_1	SP8	7149	7846.894	7646	8021	114.7879	0.9111	-0.1344	0.001	0.001	17249	155885	64.493	1383	12270	5.0764	796	7149	2.9577	4.6148	4.5861	57.556	58.2641
SP8	TCF3_2	cluster_2	DP	53744	59044.611	58245	59891	479.3678	0.9102	-0.1357	0.001	0.0010435	23089	348566	45.448	8790	130696	17.041	3567	53744	7.0075	15.4489	15.4186	40.5802	41.1214
SP8	IRF6_1	cluster_2	DP	2889	3176.339	3025	3353	98.7838	0.9096	-0.1367	0.008	0.0083478	2749	44071	67.262	317	4808	7.3381	183	2889	4.4093	6.657	6.5553	57.7287	60.0874
SP8	ETV4_2	cluster_4	SP4	2988	3285.613	3098	3479	114.3788	0.9094	-0.1369	0.004	0.0043636	4775	43196	25.078	1473	13170	7.646	332	2988	1.7347	6.9529	6.9173	22.539	22.6879
SP4	ATF6_1	cluster_1	SP8	1881	2069.102	1937	2170	55.2381	0.9091	-0.1374	0.001	0.0010435	3915	27415	63.572	470	3261	7.5619	268	1881	4.3618	6.8455	6.8612	57.0213	57.6817
SP4	MYBL2_4	cluster_2	DP	2092	2302.267	2178	2427	74.2524	0.9087	-0.1381	0.001	0.0010435	1678	23553	66.504	263	3526	9.956	150	2092	5.9069	8.9392	8.8821	57.0342	59.3307
SP4	SPZ1_1	cluster_1	SP8	39230	43174.799	42548	43747	364.7638	0.9086	-0.1382	0.001	0.001	48085	704127	63.844	4890	68733	6.2321	2731	39230	3.557	5.6795	5.5714	55.8487	57.0759
SP4	FLI1_2	cluster_1	SP8	4996	5499.544	5305	5720	127.975	0.9085	-0.1385	0.001	0.001	7024	91445	65.047	671	8532	6.069	385	4996	3.5538	5.4812	5.4637	57.377	58.556
SP4	RUNX3_3	cluster_1	SP8	24411	26878.275	26326	27441	334.2192	0.9082	-0.1389	0.001	0.001	22811	405311	62.285	2618	44080	6.7739	1424	24411	3.7513	6.2426	6.0228	54.3927	55.3789
SP8	ETV1_1	cluster_4	SP4	3006	3314.313	3128	3497	112.1862	0.907	-0.1408	0.001	0.0010909	4842	43840	25.062	1491	13333	7.6221	334	3006	1.7185	6.898	6.8568	22.4011	22.5456
SP4	FLI1_4	cluster_1	SP8	5219	5755.184	5558	5935	118.9548	0.9069	-0.1411	0.001	0.001	7443	97009	66.151	690	8773	5.9824	403	5219	3.5589	5.4145	5.3799	58.4058	59.4893
SP8	TCF4_1	cluster_1	SP8	21249	11625.24	11340	11948	176.9146	1.8278	0.8701	0.001	0.0010909	32686	295493	45.063	2953	26049	3.9725	2387	21249	3.2405	7.3028	7.191	80.8331	81.5732
SP8	GMEB1_1	cluster_2	DP	8792	9697.609	9453	9941	157.6026	0.9066	-0.1414	0.001	0.0010435	5959	97141	70.771	862	13770	10.032	541	8792	6.4053	9.0787	9.0508	62.761	63.8489
SP8	ARID5A_1	cluster_5	Common_1_DN	1829	2018.582	1903	2129	68.7533	0.9061	-0.1422	0.004	0.0048	834	11458	67.591	253	3206	18.912	138	1829	10.789	16.5468	15.9626	54.5455	57.0493
SP8	POU2F2_known11	cluster_4	SP4	6128	6763.247	6563	6956	122.6937	0.9061	-0.1423	0.001	0.0010435	4428	44372	59.446	1193	11573	15.504	622	6128	8.2097	14.047	13.8105	52.1375	52.9508
SP8	MAF_known12	cluster_4	SP4	12197	13463.23	13127	13828	219.222	0.906	-0.1425	0.001	0.001	7662	130608	59.214	1414	23154	10.497	730	12197	5.5298	9.5275	9.3386	51.6266	52.6777
DN	HESX1_1	cluster_2	DP	987	1089.787	1043	1135	28.7318	0.9058	-0.1428	0.001	0.0012632	1814	16345	82.401	158	1360	6.8562	111	987	4.9758	6.1191	6.0385	70.2532	72.5735
SP4	CUX2_1	cluster_5	Common_1_DN	965	1065.543	978	1152	52.941	0.9057	-0.1428	0.032	0.036571	439	7949	74.631	100	1592	14.947	59	965	9.0602	13.4396	12.1399	59	60.6156
SP4	ARID3A_2	cluster_2	DP	1265	1396.895	1278	1517	70.4197	0.9056	-0.143	0.04	0.043636	1263	20439	64.999	155	2265	7.2031	81	1265	4.0229	6.4133	6.1891	52.2581	55.8499
DN	IRF4_1	cluster_2	DP	3257	3597.207	3492	3707	66.4712	0.9055	-0.1433	0.001	0.0010435	3899	54808	80.813	341	4540	6.6941	237	3257	4.8023	6.0785	5.9426	69.5015	71.7401
DP	FOXP1_1	cluster_5	Common_1_DN	26594	29408.463	28668	30151	450.5302	0.9043	-0.1451	0.001	0.0010435	8153	191364	49.307	3601	67880	17.49	1298	26594	6.8522	15.9205	13.8971	36.0455	39.178
SP4	ATF7_1	cluster_1	SP8	3952	4374.652	4194	4546	111.5887	0.9034	-0.1466	0.001	0.001	3484	45358	62.093	561	7117	9.7428	307	3952	5.4101	8.8117	8.7129	54.7237	55.529
SP8	RUNX1_2	cluster_4	SP4	9588	10613.961	10319	10935	186.5013	0.9033	-0.1466	0.001	0.0010435	4117	64095	56.816	1297	19205	17.024	623	9588	8.4992	15.1324	14.959	48.0339	49.9245
DP	SOX11_2	cluster_5	Common_1_DN	7095	7855.117	7591	8146	166.1692	0.9032	-0.1468	0.001	0.0010909	3067	42939	50.606	1219	15941	18.787	521	7095	8.3618	16.9873	16.5234	42.74	44.5079
SP8	TBX19_1	cluster_4	SP4	7620	8437.846	8132	8755	187.6836	0.9031	-0.1471	0.001	0.0010435	3619	68942	53.926	888	16009	12.522	407	7620	5.9603	11.2462	11.0528	45.8333	47.5982
DP	CBX5_1	cluster_5	Common_1_DN	6880	7619.019	7362	7859	150.2528	0.903	-0.1472	0.001	0.0010435	3561	39214	51.585	1439	15113	19.881	638	6880	9.0505	17.9163	17.5448	44.3363	45.5237
DP	SOX10_4	cluster_5	Common_1_DN	12733	14110.062	13709	14458	229.2761	0.9024	-0.1481	0.001	0.0010909	4770	71762	51.378	1975	28093	20.113	863	12733	9.1162	18.0922	17.7434	43.6962	45.3245
DP	SOX15_4	cluster_5	Common_1_DN	4372	4846.77	4628	5088	136.9058	0.9021	-0.1487	0.004	0.0043636	1805	25291	45.839	840	10927	19.805	324	4372	7.9242	17.9501	17.2868	38.5714	40.011
DN	HMGA1_2	cluster_2	DP	6672	7397.964	7221	7597	113.74	0.9019	-0.149	0.001	0.0010435	6789	95209	77.457	772	10035	8.164	497	6672	5.428	7.3207	7.0077	64.3782	66.4873

SP4	ETV4_2	cluster_1	SP8	2150	2385.907	2214	2544	100.97	0.9012	-0.1501	0.008	0.0083478	4906	44382	25.767	1051	9352	5.4294	240	2150	1.2482	4.892	4.8443	22.8354	22.9897
DN	IRF4_2	cluster_2	DP	6530	7251.804	7068	7438	115.3202	0.9005	-0.1512	0.001	0.001	7884	113080	77.321	723	9646	6.5957	476	6530	4.465	6.0375	5.7747	65.8368	67.6965
DP	ARID3A_2	cluster_5	Common_1_DN	2936	3261.784	3042	3458	120.6028	0.9002	-0.1518	0.004	0.0050526	987	15963	50.765	461	6775	21.546	191	2936	9.3369	19.3516	18.3925	41.4317	43.3358
SP8	IKZF3_1	cluster_4	SP4	5875	6527.5	6317	6751	132.8967	0.9001	-0.1519	0.001	0.0010435	6473	77854	57.71	1001	11533	8.5489	497	5875	4.3549	7.678	7.5462	49.6503	50.9408
SP8	SMAD3_3	cluster_4	SP4	3394	3771.58	3635	3914	85.4013	0.8999	-0.1521	0.001	0.0010435	3767	33918	58.906	744	6513	11.311	381	3394	5.8944	10.1141	10.0065	51.2097	52.1112
DP	POU2F2_known11	cluster_5	Common_1_DN	6673	7415.714	7213	7637	134.8213	0.8999	-0.1522	0.001	0.0010435	3816	38242	51.233	1531	14781	19.802	685	6673	8.9399	17.9507	17.4494	44.742	45.1458
SP8	REL_1	cluster_4	SP4	10650	11842.953	11594	12074	143.4904	0.8993	-0.1532	0.001	0.001	12386	112079	62.586	2162	19122	10.678	1191	10650	5.947	9.6157	9.5022	55.0879	55.695
SP4	E2F_known24	cluster_1	SP8	4074	4531.654	4320	4703	113.9882	0.899	-0.1536	0.001	0.001	3037	51804	67.185	422	6854	8.8891	243	4074	5.2836	8.0013	7.8643	57.5829	59.4397
SP8	RUNX3_3	cluster_4	SP8	15796	17572.077	17157	17989	251.0435	0.8989	-0.1537	0.001	0.001	23891	424439	65.225	1632	27443	4.2173	914	15796	2.4274	3.8257	3.7216	56.0049	57.5593
SP8	IRF_known21	cluster_4	SP4	15285	17007.913	16666	17316	200.8021	0.8987	-0.1541	0.001	0.001	7912	114984	65.268	1864	26418	14.995	1050	15285	8.6761	13.271	13.2932	56.3305	57.8583
SP8	HESX1_2	cluster_5	Common_1_DN	2327	2590.117	2468	2717	78.1758	0.8985	-0.1545	0.001	0.0014118	899	12600	63.31	323	4233	21.269	172	2327	11.692	19.1324	18.4683	53.2508	54.9728
DP	SOX7_4	cluster_5	Common_1_DN	5914	6583.174	6254	6862	193.5269	0.8984	-0.1546	0.001	0.0010909	2231	35768	47.798	969	14283	19.087	385	5914	7.9031	17.2568	16.5343	39.7317	41.4059
SP8	E2F8_1	cluster_4	SP4	5958	6635.605	6458	6822	113.0623	0.8979	-0.1554	0.001	0.001	7551	83269	64.648	989	10443	8.1077	555	5958	4.6257	7.35	7.1551	56.1173	57.0526
DN	SOX12_1	cluster_2	DP	3939	4395.737	4252	4533	89.3431	0.8961	-0.1582	0.001	0.0010435	3600	46915	74.344	501	6174	9.7837	315	3939	6.242	8.75	8.396	62.8743	63.7998
DP	POU2F2_known12	cluster_5	Common_1_DN	6583	7346.569	7111	7568	132.2383	0.8961	-0.1583	0.001	0.0010435	3757	37632	51.558	1513	14607	20.012	674	6583	9.019	17.9398	17.4931	44.5473	45.0674
DP	SOX1_2	cluster_5	Common_1_DN	6639	7411.376	7124	7666	164.7416	0.8958	-0.1588	0.001	0.0010909	2774	38842	48.471	1185	15579	19.441	485	6639	8.2849	17.4838	17.0923	40.9283	42.6151
DN	POU2F2_known10	cluster_2	DP	4766	5322.5	5178	5428	77.5512	0.8955	-0.1593	0.001	0.0010435	6013	54229	74.503	838	7279	10	537	4766	6.5478	8.9307	8.7887	64.0811	65.476
SP8	MYBL1_5	cluster_2	DP	3947	4411.258	4218	4610	113.4211	0.8948	-0.1604	0.001	0.0010435	2849	45954	65.783	445	6853	9.81	247	3947	5.6501	8.6697	8.589	55.5056	57.5952
SP8	EGR1_known9	cluster_2	DP	37591	42016.57	41596	42439	277.6185	0.8947	-0.1606	0.001	0.001	29509	400130	74.089	4300	57195	10.59	2790	37591	6.9605	9.4547	9.3947	64.8837	65.7243
DP	POU2F2_known10	cluster_5	Common_1_DN	6794	7597.14	7385	7819	130.8361	0.8943	-0.1612	0.001	0.0010435	4216	38032	52.25	1684	14748	20.262	768	6794	9.334	18.2163	17.8639	45.6057	46.0673
SP8	MAF_known11	cluster_4	SP4	14165	15839.393	15446	16219	240.8734	0.8943	-0.1612	0.001	0.0010435	10315	166406	54.325	1919	29572	9.654	889	14165	4.6243	8.6185	8.5123	46.3262	47.9
SP4	MESP1_1	cluster_2	DP	36796	41157.585	40629	41651	305.6898	0.894	-0.1616	0.001	0.001	28293	255419	48.619	9529	85097	16.198	4085	36796	7.0041	14.4382	14.4061	42.8691	43.2401
DP	POU2F2_known5	cluster_5	Common_1_DN	7729	8646.498	8395	8907	160.8242	0.8939	-0.1618	0.001	0.0010909	3181	41440	50.684	1410	17442	21.333	605	7729	9.453	19.0192	18.6511	42.9078	44.3126
SP4	PATZ1_1	cluster_1	SP8	55396	62004.001	61340	62613	381.5196	0.8934	-0.1626	0.001	0.001	71621	944599	64.129	7774	98544	6.6902	4281	55396	3.7609	5.9773	5.8645	55.0682	56.2145
SP8	E2F2_3	cluster_4	SP4	2571	2878.012	2714	3023	94.9111	0.8934	-0.1627	0.001	0.001	2069	35210	66.193	283	4492	8.4447	155	2571	4.8333	7.4915	7.3019	54.7703	57.2351
DP	TCF7_1	cluster_5	Common_1_DN	26298	29440.843	28838	30037	355.374	0.8933	-0.1629	0.001	0.0010435	9326	149704	48.954	4000	61044	19.962	1667	26298	8.5995	17.8748	17.5667	41.675	43.0804
DP	CUX2_2	cluster_3	DN	264	295.694	246	338	27.7107	0.8932	-0.163	0.154	0.154	279	2511	51.763	69	591	12.183	30	264	5.4422	10.7527	10.5137	43.4783	44.6701
DN	FOXP1_1	cluster_2	DP	18475	20686.917	20206	21148	308.7899	0.8931	-0.1631	0.001	0.0010435	10678	252594	65.084	1880	37085	9.5554	873	18475	4.7603	8.1757	7.3141	46.4362	49.818
SP8	REL_2	cluster_4	SP4	10433	11692.414	11427	11945	151.242	0.8923	-0.1644	0.001	0.0010435	12473	112795	62.62	2133	18854	10.467	1167	10433	5.792	9.3562	9.2495	54.7117	55.3357
SP4	SP100_1	cluster_1	SP8	781	875.517	808	944	40.2023	0.8922	-0.1646	0.012	0.0144	962	12518	74.454	97	1202	7.1492	61	781	4.6452	6.341	6.239	62.8866	64.975
DP	TCF7_2	cluster_5	Common_1_DN	21128	23696.296	23290	24123	238.8912	0.8916	-0.1655	0.001	0.0010435	10707	118115	49.917	4487	48020	20.294	1942	21128	8.9289	18.1377	17.8877	43.2806	43.9983
DP	SOX15_2	cluster_5	Common_1_DN	5727	6423.819	6169	6671	149.5888	0.8915	-0.1656	0.001	0.0010909	2513	35262	49.216	1049	13569	18.939	427	5727	7.9934	16.9916	16.2413	40.7054	42.2065
SP8	NFKB_known10	cluster_4	SP4	10252	11500.874	11193	11762	175.7265	0.8914	-0.1658	0.001	0.001	10708	129395	64.216	1529	18124	8.9945	853	10252	5.0878	7.966	7.923	55.7881	56.5659
SP4	IRF_known21	cluster_2	DP	6549	7348.031	7116	7570	138.9663	0.8913	-0.1661	0.001	0.001	8196	119181	67.65	824	11147	6.3273	468	6549	3.7174	5.7101	5.495	56.7961	58.7512
SP4	HIF1A_2	cluster_2	DP	17107	19199.761	18857	19539	204.3776	0.891	-0.1665	0.001	0.001	13715	183722	69.394	2138	27848	10.519	1291	17107	6.4615	9.4131	9.3114	60.3835	61.4299
SP4	HES1_1	cluster_1	SP8	19284	21650.473	21230	22018	241.8791	0.8907	-0.167	0.001	0.001	24677	349612	62.645	2546	34903	6.2541	1373	19284	3.4554	5.5639	5.5158	53.9277	55.2503
SP8	LMO2_1	cluster_2	DP	54815	61567.039	60815	62255	455.4471	0.8903	-0.1676	0.001	0.001	35542	394212	47.125	11969	131187	15.682	4949	54815	6.5527	13.9244	13.905	41.3485	41.7839
SP4	TCF21_1	cluster_2	DP	18356	20622.973	20175	21120	273.7027	0.8901	-0.168	0.001	0.001	10517	136988	48.648	3322	42596	15.127	1415	18356	6.5187	13.4544	13.3997	42.5948	43.0932
SP4	NFATC1_3	cluster_2	DP	2897	3255.333	3088	3420	99.5223	0.89	-0.1682	0.001	0.0010909	3149	40963	60.391	457	5575	8.2191	229	2897	4.271	7.2721	7.0722	50.1094	51.9641
SP8	CUX2_2	cluster_4	SP4	435	489.053	449	533	26.3544	0.8897	-0.1686	0.012	0.015158	381	3429	70.686	84	716	14.76	49	435	8.9672	12.8609	12.6859	58.3333	60.7542
DP	TCF7L1_1	cluster_5	Common_1_DN	23681	26619.066	26085	27197	328.6542	0.8896	-0.1687	0.001	0.0010435	8494	136288	48.648	3640	55600	19.846	1497	23681	8.4529	17.6242	17.3757	41.1264	42.5917
SP8	AHR::ARNT::HIF1A_1	cluster_2	DP	9229	10375.648	10208	10581	110.9192	0.8895	-0.1689	0.001	0.0010435	12611	103685	71.095	1807	14673	10.061	1130	9229	6.3282	8.9604	8.901	62.5346	62.8978
SP8	TCF21_1	cluster_2	DP	17741	19946.608	19543	20338	247.4656	0.8894	-0.169	0.001	0.001	10166	132387	47.014	3322	42596	15.127	1367	17741	6.3003	13.4468	13.4009	41.1499	41.6495
SP4	E2F_known26	cluster_1	SP8	2203	2477.951	2312	2622	89.6755	0.8891	-0.1696	0.001	0.0010435	1689	28742	66.459	241	3860	8.9253	131	2203	5.0939	7.7561	7.6647	54.3568	57.0725
DN	NFATC2_1	cluster_2	DP	3259	3667.601	3584	3750	50.6086	0.8886	-0.1704	0.001	0.0010909	7464	44784	80.238	798	4659	8.3474	549	3259	5.839	7.3553	7.2772	68.797	69.9506
DP	POU2F3_2	cluster_5	Common_1_DN	4054	4562.691	4434	4693	81.5689	0.8885	-0.1705	0.001	0.0010909	2892	23182	50.653	1185	9162	20.019	515	4054	8.8581	17.8077	17.4877	43.4599	44.248
DP	SOX21_3	cluster_5	Common_1_DN	7709	8676.347	8413	8969	177.6694	0.8885	-0.1705	0.001	0.0010909	3002	45066	51.174	1244	17494	19.865	526	7709	8.7539	17.5217	17.106	42.283	44.0665
DP	SOX8_2	cluster_5	Common_1_DN	11324	12748.279	12382	13160	224.9556	0.8883	-0.1709	0.001	0.0010909	4342	65204	50.995	1805	25656	20.065	768	11324	8.8564	17.6877	17.367	42.5485	44.1378
DP	TCF7L2_known7	cluster_5	Common_1_DN	24315	27372.779	26840	27883	315.6078	0.8883	-0.1709	0.001	0.0010435	10010	140599	49.374	4202	56378	19.798	1761	24315	8.5386	17.5924	17.2939	41.9086	43.1285
SP8	EWSR1::FLI1_1	cluster_4	SP4	84340	94961.711	93914	95992	605.2133	0.8881	-0.1711	0.001	0.0010435	45443	904295	55.466	9280	176015	10.796	4330	84340	5.1731	9.5284	9.3266	46.6595	47.9164
DP	SOX14_2	cluster_5	Common_1_DN	5521	6216.62	6014	6424	125.3249	0.8881	-0.1712	0.001	0.0010909	2895	31972	49.947	1208	12699	19.838	510	5521	8.6249	17.6166	17.2682	42.2185	43.4759
SP8	MAFG_1	cluster_4	SP4	11674	13150.808	12672	13559	261.8278	0.8877	-0.1718	0.001	0.0010435	6025	120737	52.864	1341	25357	11.102	590	11674	5.1114	9.7925	9.6689	43.997	46.0386
SP8	E2F7_1	cluster_4	SP4	4669	5259.896	5065	5447	111.6691	0.8877	-0.1719	0.001	0.0010435	4510	58679	64.962	669	8269	9.1544	372	4669	5.1689	8.2483	7.9568	55.6054	56.4639
DP	SOX7_3	cluster_5	Common_1_DN	7295	8219.376	7963	8478	160.8964	0.8876	-0.1721	0.001	0.0010909	2800	44824	49.319	1166	17179	18.902	475	7295	8.0265	16.9643	16.2748	40.7376	42.4646
DP	SOX4_1	cluster_5	Common_1_DN	29123	32819.731	32233	33362	335.0471	0.8874	-0.1724	0.001	0.0010435	10641	172327	54.726	4095	61800	19.626	1848	29123	9.2486	17.3668	16.8998	45.1282	47.1246
DP	IKZF2_3	cluster_5	Common_1_DN	8670	9776.676	9545	10006	145.3012	0.8868	-0.1733	0.001	0.0010909	4868	48814	55.261	1899	18181	20.582	887	8670	9.8151	18.221	17.7613	46.7088	47.6871
SP8	HIF1A_1	cluster_2	DP	11837	13352.338	13127	13618	153.4051	0.8865	-0.1738	0.001	0.0010435	11568	130194	67.952	1804	19792	10.33	1059	11837	6.1781	9.1546	9.0918	58.7029	59.807
SP8	ID4_1	cluster_2	DP	39139	44155.963	43638	44693	325.124	0.8864	-0.174	0.001	0.0010435	29653	268120	45.899	10793	96618	16.54	4338	39139	6.7001	14.6292	14.5976	40.1927	40.509
DN	CUX1_8	cluster_2	DP	521	587.978	528	645	36.3234	0.8863	-0.1742	0.036	0.039273	619	9964	75.019	61	863	6.4975	34	521	3.9226	5.4927	5.2288	55.7377	60.3708
SP8	POU2F2_known12	cluster_4	SP4	5938	6704.969	6515	6896	113.6761	0.8856	-0.1752	0.001	0.0010435	4381	43877	60.114	1183	11402	15.621	604	5938	8.1354	13.7868	13.5333	51.0566	52.0786
SP8	ETV3_1	cluster_4	SP4	10164	11478.748	11259	11714	138.6352	0.8855	-0.1755	0.001	0.0010435	13610	123083	65.171	1986	17704	9.374	1129	10164	5.3817	8.2954	8.2578	56.8479	57.4108
DP	SPDEF_4	cluster_5	Common_1_DN	16446	18581.754	18179	19028	261.04	0.8851	-0.1761	0.001	0.0010435	6274	94414	52.37	2551	36276	20.122	1116	16446	9.1224	17.7877	17.419	43.7475	45.3358
DN	MYBL2_4	cluster_2	DP	2422	2737.304	2636	2833	59.0695	0.8849	-0.1765	0.001	0.0010435	1996	27994	79.043	263	3526	9.956	175	2422	6.8387	8.7675	8.6519	66.5399	68.6897
SP4	HESX1_2	cluster_2	DP	720	813.936	745	890	41.9378	0.8847	-0.1767	0.008	0.0096	906	12691	63.767	105	1325	6.6576	53	720	3.6177	5.8499	5.6733	50.4762	54.3396
DP	SOX11_1	cluster_5	Common_1_DN	26626	30096.868	29519	30583	320.6379	0.8847	-0.1768	0.001	0.0010435	9836	159338	54.566	3781	56921	19.493	1690	26626	9.1181	17.1818	16.7104	44.6972	46.7771
DN	EGR4_2	cluster_2	DP	84965	96087.425	95494	96801	387.4394	0.8842	-0.1775	0.001	0.001	55529	908524	79.001	7829	124130	10.794	5235	84965	7.3882	9.4275	9.352	66.8668	68.4484
DN	EGR4_1	cluster_2	DP	34472	39006.541	38647	39370	216.5733	0.8838	-0.1783	0.001	0.001	34373	389734	77.181	4596	51128	10.125	3050	34472	6.8267	8.8732	8.845	66.3621	67.4229
DN	LMO2_1	cluster_2	DP	34677	39239.017	38624	39903	376.7605	0.8837	-0.1783	0.001	0.001	22843	252436	30.177	11969	131187	15.682	3149	34677	4.1454	13.7854	13.7369	26.3096	26.4333
DP	NFATC2_1	cluster_5	Common_1_DN	5590	6326.575	6192	6468	86.3045	0.8836	-0.1785	0.001	0.0010909	5104	30624	54.868	1994	11648	20.869	944	5590	10.015	18.4953	18.2537	47.342	47.9911
SP8	PBX3_known1	cluster_4	SP4	6784	7677.525	7428	7924	146.0731	0.8836	-0.1785	0.001	0.0010435	6890	76602	56.96	1282	13739	10.216	618	6784	5.0445	8.9695	8.8562	48.2059	49.3777
DP	SOX9_4	cluster_5	Common_1_DN	11717	13261.075	12876	13613	229.5217	0.8836	-0.1786	0.001	0.0010909	4428	66520	51.448	1850	26308	20.347	795	11717	9.0622	17.9539	17.6143	42.973	44.5378
SP8	EGR3_3	cluster_2	DP	44187	50039.923	49559	50508	295.4334	0.883	-0.1795	0.001	0.001	32104	473266	72.424	4864	69807	10.683	3024	44187	6.7619	9.4194	9.3366	62.1711	63.2988
DP	TCF7L1_2	cluster_5	Common_1_DN	20579	23307.382	22966	23674	230.4269	0.8829	-0.1796	0.001	0.0010435	10535	116448	51.095	4326	46203	20.273	1887	20579	9.0296	17.9117	17.6723	43.62	44.5404
DN	NFATC1_3	cluster_2	DP	3753	4251.677	4111	4376	81.5179	0.8827	-0.1799	0.001	0.0010909	4137	53815	79.338	457	5575	8.2191	298	3753	5.533	7.2033	6.9739	65.2079	67.3184
SP8	XBP1_2	cluster_4	SP4	5627	6374.46	6108	6630	164.3288	0.8828	-0.1799	0.001	0.0010435	2655	61164	63.418	478	10402	10.785	249	5627	5.8344	9.3785	9.1999	52.0921	54.0954
DP	SOX5_1	cluster_5	Common_1_DN	2740	3104.983	2981	3235	77.311	0.8825	-0.1803	0.001	0.0010909	1887	16983	50.471	715	6233	18.524	309	2740	8.1429	16.3752	16.1338	43.2168	43.9596
DP	GATA_known17	cluster_5	Common_1_DN	9608	10890.002	10571	11273	208.432	0.8823	-0.1807	0.001	0.0010909	3215	51515	48.405	1511	22926	21.542	606	9608	9.028	18.8491	18.6509	40.1059	41.9087
SP4	SOX2_6	cluster_1	SP8	3355	3802.766	3606	4008	122.7678	0.8823	-0.1807	0.001	0.0010435	2099	33627	62.352	424	6325	11.728	217	3355	6.2209	10.3383	9.9771	51.1792	53.0435
DP	HBP1_1	cluster_5	Common_1_DN	9525	10799.233	10494	11138	195.2895	0.882	-0.1811	0.001	0.0010435	3549	54705	50.814	1516	21821	20.269	632	9525	8.8475	17.8078	17.4116	41.6887	43.6506
SP8	MYCN_2	cluster_2	DP	14800	16780.223	16530	17053	168.3827	0.882	-0.1812	0.001	0.001	17431	157501	65.162	2916	25887	10.71	1647	14800	6.1231	9.4487	9.3968	56.4815	57.1716
DN	TCF7_1	cluster_2	DP	19908	22574.32	22150	22978	248.7692	0.8819	-0.1813	0.001	0.0010435	13527	217263	71.046	2102	32593	10.658	1260	19908	6.51	9.3147	9.1631	59.9429	61.0806
DN	EGR1_known9	cluster_2	DP	40910	46390.09	45962	46785	249.1797	0.8819	-0.1814	0.001	0.001	32718	444077	82.227	4300	57195	10.59	3029	40910	7.575	9.2579	9.2124	70.4419	71.5272
DP	FOXO1_4	cluster_5	Common_1_DN	4173	4732.657	4517	4924	119.5891	0.8818	-0.1815	0.001	0.0010909	1950	25417	51.042	766	9486	19.05	327	4173	8.3802	16.7692	16.4181	42.6893	43.9911
SP4	TCF4_2	cluster_2	DP	36256	41122.085	40661	41667	314.6079	0.8817	-0.1817	0.001	0.001	28828	260376	48.438	9571	85376	15.882	4019	36256	6.7447	13.9413	13.9245	41.9914	42.4663
DP	SOX2_5	cluster_5	Common_1_DN	7277	8254.92	7964	8543	171.7727	0.8815	-0.1819	0.001	0.0010909	2721	43557	49.369	1164	17280	19.586	471	7277	8.2479	17.3098	16.7068	40.4639	42.1123
SP4	EGR1_known9	cluster_2	DP	35794	40622.327	40213	41070	274.4977	0.8811	-0.1826	0.001	0.001	28464	386336	71.535	4300	57195	10.59	2648	35794	6.6277	9.303	9.265	61.5814	62.5824
DN	SOX4_1	cluster_2	DP	21510	24414.788	24010	24812	252.1336	0.881	-0.1827	0.001	0.0010435	14546	235575	74.812	2224	34044	10.811	1359	21510	6.831	9.3428	9.1309	61.1061	63.1829
DN	GMEB1_1	cluster_2	DP	2705	3071.848	2808	3334	153.1978	0.8806	-0.1834	0.016	0.016	1931	30986	22.575	862	13770	10.032	170	2705	1.9707	8.8037	8.7297	19.7216	19.6442
DP	POU2F3_1	cluster_5	Common_1_DN	7983	9068.405	8745	9409	198.4098	0.8803	-0.1839	0.001	0.0010435	3087	46662	50.134	1307	18511	19.888	537	7983	8.577	17.3955	17.1081	41.0865	43.1257
SP4	HIF1A_2	cluster_2	DP	16837	19138.047	18792	19465	208.8659	0.8798	-0.1848	0.001	0.001	13692	183403	69.274	2138	27848	10.519	1270	16837	6.3595	9.2755	9.1803	59.4013	60.4604
SP4	TCF12_known1	cluster_2	DP	28912	32862.308	32457	33328	262.3484	0.8798	-0.1848	0.001	0.0010909	34730	249719	53.636	8606	61446	13.198	4037	28912	6.2099	11.624	11.5778	46.9091	47.0527
SP8	PBX1_2	cluster_4	SP4	5040	5730.118	5505	5961	135.276	0.8796	-0.1851	0.001	0.0010435	2880	41061	58.486	746	10083	14.362	361	5040	7.1788	12.5347	12.2744	48.3914	49.9851
DN	REL_2	cluster_2	DP	10818	12300.31	12098	12504	118.1809	0.8795	-0.1852	0.001	0.0010435	15212	137460	76.313	1867	16362	9.0836	1213	10818	6.0058	7.974	7.8699	64.9705	66.1166
DP	SOX1_3	cluster_5	Common_1_DN	6286	7148.526	6869	7399	165.3515	0.8794	-0.1855	0.001	0.0010909	2535	35523	47.207	1186	15495	20.591	463	6286	8.3535	18.2643	17.6956	39.0388	40.5679
DP	SOX17_4	cluster_5	Common_1_DN	5014	5702.603	5478	5928	142.6789	0.8793	-0.1856	0.001	0.0010909	2192	30688	48.463	919	12059	19.044	368	5014	7.9183	16.7883	16.3386	40.0435	41.5789
SP8	HESX1_1	cluster_2	DP	693	788.282	724	854	38.6431	0.8793	-0.1856	0.008	0.0087273	1301	11720	59.084	158	1360	6.8562	78	693	3.4936	5.9954	5.913	49.3671	50.9559
SP8	MYC_known20	cluster_4	SP4	9045	10286.805	9943	10628	207.319	0.8793	-0.1856	0.001	0.001	8781	143953	57.101	1173	18285	7.253	560	9045	3.5878	6.3774	6.2833	47.7408	49.4668
DN	REL_1	cluster_2	DP	10589	12045.993	11867	12217	107.7217	0.8791	-0.186	0.001	0.001	15073	136291	76.106	1831	16059	8.9675	1186	10589	5.913	7.8684	7.7694	64.7733	65.9381
SP4	ARID5A_1	cluster_1	SP8	1193	1357.838	1243	1452	60.7384	0.8787	-0.1866	0.008	0.0091429	810	11102	65.491	182	2224	13.119	95	1193	7.0375	11.7284	10.7458	52.1978	53.6421
DN	IRF_known14	cluster_2	DP	22303	25383.987	24935	25779	260.2505	0.8786	-0.1867	0.001	0.001	16394	342215	77.672	1777	34195	7.7612	1116	22303	5.0621	6.8074	6.5172	62.8025	65.223
DN	TCF7_2	cluster_2	DP	15475	17614.349	17333	17890	169.9748	0.8786	-0.1868	0.001	0.0010435	15144	167157	70.642	2353	25359	10.717	1416	15475	6.5399	9.3502	9.2578	60.1785	61.0237
DN	EGR3_3	cluster_2	DP	47800	54410.368	53948	54901	285.8065	0.8785	-0.1869	0.001	0.001	35070	516864	79.095	4864	69807	10.683	3264	47800	7.3148	9.3071	9.2481	67.1053	68.4745
DN	TCF7L1_1	cluster_2	DP	18301	20836.662	20455	21205	225.9812	0.8783	-0.1872	0.001	0.0010435	12339	198117	70.718	1956	30243	10.795	1159	18301	6.5325	9.393	9.2375	59.2536	60.5132
SP8	KLF12_1	cluster_4	SP4	6413	7301.708	7132	7452	97.3224	0.8783	-0.1872	0.001	0.001	19874	119244	60.476	2058	12154	6.164	1075	6413	3.2524	5.4091	5.378	52.2352	52.7645
SP8	SPDEF_6	cluster_2	DP	11141	12685.501	12410	12966	172.98	0.8783	-0.1873	0.001	0.001	8460	118816	68.647	1390	18717	10.814	800	11141	6.4368	9.4563	9.3767	57.554	59.5234
SP8	EGR3_2	cluster_2	DP	66636	75908.336	75260	76566	392.128	0.8778	-0.188	0.001	0.001	46634	711154	72.287	7154	106180	10.793	4394	66636	6.7734	9.4223	9.3701	61.4202	62.7576
SP8	MYBL1_5	cluster_4	SP4	3195	3639.953	3472	3813	104.0076	0.8778	-0.188	0.001	0.0010435	2849	45954	65.783	371	5637	8.0693	205	3195	4.5736	7.1955	6.9526	55.2561	56.6791
DN	HBP1_1	cluster_2	DP	6226	7094.07	6861	7286	125.0532	0.8777	-0.1883	0.001	0.0010435	5153	79302	73.662	703	10052	9.3371	417	6226	5.7832	8.0924	7.851	59.3172	61.9379
SP8	ETV5_1	cluster_4	SP4	1764	2010.698	1862	2162	94.2109	0.8774	-0.1887	0.001	0.0010435	4199	38058	26.542	857	7666	5.3464	196	1764	1.2302	4.6678	4.635	22.8705	23.0107
DN	PBX1_2	cluster_2	DP	3551	4050.485	3905	4188	90.2176	0.8767	-0.1898	0.001	0.0010435	3613	51272	73.03	430	5750	8.1901	259	3551	5.0579	7.1686	6.9258	60.2326	61.7565
DP	SOX_1	cluster_5	Common_1_DN	12281	14009.275	13694	14364	202.3429	0.8766	-0.1899	0.001	0.0010435	5830	70005	50.837	2422	27958	20.303	1038	12281	8.9184	17.8045	17.543	42.8571	43.9266
SP4	AHR::ARNT::HIF1A_1	cluster_2	DP	9079	10357.809	10177	10549	111.9588	0.8765	-0.1901	0.001	0.0010435	12561	103445	70.93	1807	14673	10.061	1111	9079	6.2253	8.8448	8.7766	61.4831	61.8756
DN	EGR1_known12	cluster_2	DP	89429	102052.509	101239	102840	482.1839	0.8763	-0.1905	0.001	0.001	57885	958377	76.536	8547	136439	10.896	5469	89429	7.1418	9.448	9.3313	63.9874	65.545
DN	ELK4_1	cluster_2	DP	8338	9515.4	9392	9624	72.9901	0.8763	-0.1905	0.001	0.001	11601	93425	84.428	1427	11332	10.241	1042	8338	7.5351	8.982	8.9248	73.0203	73.5792
DN	RREB1_2	cluster_2	DP	39726	45340.228	44348	46461	654.3797	0.8762	-0.1907	0.001	0.001	18681	474233	23.829	9551	211427	10.624	1702	39726	1.9961	9.1109	8.3769	17.8201	18.7895
SP8	E2F_known24	cluster_4	SP4	3862	4407.964	4229	4620	112.8087	0.8762	-0.1907	0.001	0.001	2992	51042	66.197	431	6870	8.9098	233	3862	5.0087	7.7874	7.5663	54.0603	56.2154
DN	POU2F2_known13	cluster_2	DP	5158	5888.929	5621	6126	136.7388	0.8759	-0.1912	0.001	0.0010909	3197	67338	75.85	436	8267	9.312	256	5158	5.81	8.0075	7.6599	58.7156	62.3926
DN	SP4_1	cluster_2	DP	147096	167965.153	167079	168838	538.5809	0.8758	-0.1914	0.001	0.001	90889	1624561	78.36	12677	218773	10.552	8293	147096	7.0951	9.1243	9.0545	65.4177	67.2368
DN	SOX11_1	cluster_2	DP	19824	22637.434	22262	23041	243.1102	0.8757	-0.1915	0.001	0.0010435	13495	218540	74.839	2057	31531	10.798	1254	19824	6.7888	9.2923	9.0711	60.9626	62.8715
SP4	HIF1A_1	cluster_1	SP8	5678	6484.868	6290	6684	116.0657	0.8756	-0.1917	0.001	0.0010435	11555	130065	67.885	879	9649	5.0361	511	5678	2.9635	4.4223	4.3655	58.1342	58.8455
SP8	NFKB_known11	cluster_4	SP4	12473	14248.967	13926	14540	190.6741	0.8754	-0.192	0.001	0.001	12335	149870	65.352	1852	22046	9.6133	1031	12473	5.439	8.3583	8.3225	55.6695	56.5772
DN	POU2F3_2	cluster_2	DP	2809	3209.423	3113	3296	53.7652	0.8753	-0.1922	0.001	0.0010909	4271	34232	74.798	567	4389	9.5901	357	2809	6.1377	8.3587	8.2058	62.963	64.0009
SP4	TCF3_1	cluster_2	DP	79685	91067.387	90087	91887	557.6346	0.875	-0.1926	0.001	0.0011429	45052	639694	51.735	12668	177031	14.317	5615	79685	6.4445	12.4634	12.4567	44.3243	45.0119
DN	KLF16_1	cluster_2	DP	90735	103735.24	103059	104339	371.635	0.8747	-0.1932	0.001	0.001	87857	980098	74.479	12962	141421	10.747	8151	90735	6.895	9.2776	9.2577	62.8838	64.1595
DN	POU2F2_known5	cluster_2	DP	4802	5492.713	5319	5649	99.2446	0.8743	-0.1938	0.001	0.0010909	4660	60686	74.223	612	7622	9.3222	379	4802	5.8731	8.133	7.9129	61.9281	63.0018

DN	CUX2_2	cluster_2	DP	179	204.953	176	234	17.7088	0.874	-0.1943	0.083	0.090545	368	3312	68.275	36	310	6.3904	20	179	3.69	5.4348	5.4046	55.5556	57.7419
DN	IRF6_1	cluster_2	DP	3326	3806.623	3681	3933	77.8249	0.8738	-0.1947	0.001	0.0010909	3319	53207	81.206	317	4808	7.3381	213	3326	5.0762	6.4176	6.2511	67.1924	69.1764
DP	SOX9_6	cluster_5	Common_1_DN	9332	10684.414	10378	11012	191.3442	0.8734	-0.1952	0.001	0.0010909	3625	54432	49.299	1562	22247	20.149	639	9332	8.4519	17.6276	17.1443	40.9091	41.9472
SP8	ATF3_known5	cluster_4	SP4	13465	15420.209	15013	15775	235.1878	0.8732	-0.1956	0.001	0.001	11933	167611	58.351	1972	26814	9.3348	969	13465	4.6876	8.1203	8.0335	49.1379	50.2163
DP	SOX30_1	cluster_5	Common_1_DN	7955	9111.121	8806	9454	197.1691	0.8731	-0.1957	0.001	0.0010435	3080	46247	52.079	1285	18059	20.336	541	7955	8.9581	17.5649	17.2011	42.1012	44.0501
DN	HMGA1_1	cluster_2	DP	1611	1845.379	1783	1903	37.4997	0.8731	-0.1958	0.001	0.0010435	4316	26040	76.796	417	2455	7.2402	272	1611	4.7511	6.3021	6.1866	65.2278	65.6212
SP4	HIF1A_2	cluster_1	SP8	7576	8677.789	8455	8906	142.7404	0.873	-0.1959	0.001	0.001	13692	183403	69.274	964	12652	4.7788	569	7576	2.8615	4.1557	4.1308	59.0249	59.8799
SP8	SP1_known2	cluster_2	DP	150158	172035.683	171012	173182	642.5745	0.8728	-0.1962	0.001	0.001	116483	1617881	71.388	17987	243799	10.758	10815	150158	6.6257	9.2846	9.2812	60.1268	61.5909
DN	SP1_known2	cluster_2	DP	167281	191676.171	190908	192544	470.9916	0.8727	-0.1964	0.001	0.001	131181	1808239	79.788	17987	243799	10.758	12131	167281	7.3812	9.2475	9.251	67.4432	68.6143
SP8	EGR4_2	cluster_2	DP	76363	87533.291	86857	88276	417.0322	0.8724	-0.197	0.001	0.001	50126	822981	71.563	7829	124130	10.794	4699	76363	6.6402	9.3744	9.2788	60.0204	61.5186
SP8	HES1_1	cluster_2	DP	32069	36760.154	36252	37225	305.6463	0.8724	-0.197	0.001	0.001	25160	356406	63.862	4230	58105	10.411	2280	32069	5.7462	9.062	8.9979	53.9007	55.1915
DN	POU2F2_known11	cluster_2	DP	4596	5270.695	5124	5422	93.4561	0.872	-0.1976	0.001	0.0010435	5502	55147	73.881	762	7325	9.8134	467	4596	6.1573	8.4878	8.3341	61.2861	62.744
SP4	HES1_1	cluster_2	DP	31455	36088.431	35592	36579	302.1297	0.8716	-0.1982	0.001	0.001	24677	349612	62.645	4230	58105	10.411	2238	31455	5.6362	9.0692	8.9971	52.9078	54.1348
DN	E2F7_1	cluster_2	DP	5050	5796.001	5655	5951	89.8336	0.8713	-0.1987	0.001	0.0010435	5640	73401	81.261	594	7377	8.1669	399	5050	5.5907	7.0745	6.88	67.1717	68.456
DN	RUNX2_4	cluster_2	DP	26816	30820.56	30505	31123	189.2484	0.8701	-0.2008	0.001	0.0010435	22375	342168	83.028	2540	37614	9.1272	1777	26816	6.507	7.9419	7.8371	69.9606	71.2926
DP	HMGA1_1	cluster_5	Common_1_DN	3256	3742.457	3631	3850	68.7168	0.8701	-0.2008	0.001	0.0010435	3004	18138	53.492	1205	7071	20.853	549	3256	9.6025	18.2756	17.9513	45.5602	46.0472
DP	RREB1_1	cluster_5	Common_1_DN	136095	156431.628	155388	157536	641.5028	0.87	-0.2009	0.001	0.001	45950	762192	60.431	17321	268951	21.324	8432	136095	10.79	18.3504	17.8557	48.6808	50.6022
DN	ARID3A_2	cluster_2	DP	1503	1729.091	1637	1817	57.7369	0.8693	-0.202	0.001	0.0013333	1580	25566	81.304	155	2265	7.2031	98	1503	4.7798	6.2025	5.8789	63.2258	66.3576
DN	SP8_1	cluster_2	DP	23237	26737.936	26153	27308	360.7389	0.8691	-0.2025	0.001	0.001	21179	262744	21.027	11105	131511	10.525	1915	23237	1.8596	9.042	8.844	17.2445	17.6692
SP8	SP4_1	cluster_2	DP	134468	154758.503	153690	155872	665.5255	0.8689	-0.2028	0.001	0.001	82983	1490454	71.892	12677	218773	10.552	7573	134468	6.486	9.126	9.0219	59.7381	61.4646
DP	SOX14_3	cluster_5	Common_1_DN	6592	7589.644	7313	7839	162.2028	0.8686	-0.2033	0.001	0.0010909	2759	38663	47.284	1254	16454	20.123	483	6592	8.0618	17.5063	17.0499	38.5167	40.0632
DN	KLF7_1	cluster_2	DP	115886	133452.761	132661	134382	532.8137	0.8684	-0.2036	0.001	0.001	81419	1312000	74.111	11820	183259	10.352	7274	115886	6.5461	8.934	8.8328	61.5398	63.2362
SP8	SOX13_1	cluster_5	Common_1_DN	4766	5488.376	5310	5692	117.8094	0.8684	-0.2036	0.001	0.0010909	2089	31443	63.156	667	9165	18.409	335	4766	9.573	16.0364	15.1576	50.2249	52.0022
DN	TCF7L2_known7	cluster_2	DP	18455	21256.14	20907	21608	211.561	0.8682	-0.2039	0.001	0.0010435	14358	201641	70.809	2272	30780	10.809	1337	18455	6.4808	9.3119	9.1524	58.8468	59.9578
SP8	HIF1A::ARNT_1	cluster_2	DP	6908	7956.62	7799	8123	100.8403	0.8682	-0.2039	0.001	0.001	10355	74439	66.239	1713	12082	10.751	968	6908	6.147	9.3481	9.2801	56.509	57.176
DN	POU2F2_known12	cluster_2	DP	4205	4843.88	4706	4968	83.7875	0.8681	-0.204	0.001	0.0010435	5446	54541	74.724	697	6675	9.1451	430	4205	5.7611	7.8957	7.7098	61.693	62.9963
DP	SOX21_2	cluster_5	Common_1_DN	7252	8354.341	8035	8623	170.9698	0.8681	-0.2041	0.001	0.0010909	3145	44041	49.589	1332	17389	19.58	534	7252	8.1656	16.9793	16.4665	40.0901	41.7045
DP	BPTF_1	cluster_5	Common_1_DN	26567	30613.866	30139	31064	289.7293	0.8678	-0.2045	0.001	0.0010909	10963	146228	52.127	4683	60087	21.42	2030	26567	9.4706	18.5168	18.1682	43.3483	44.2142
DN	PATZ1_1	cluster_2	DP	113630	130969.218	130249	131757	451.4352	0.8676	-0.2049	0.001	0.001	85759	1123464	76.273	13788	175131	11.89	8782	113630	7.7144	10.2403	10.1143	63.6931	64.8829
DP	CUX2_2	cluster_5	Common_1_DN	327	377.096	329	423	29.0222	0.8675	-0.2051	0.036	0.0432	279	2511	51.763	97	790	16.285	40	327	6.7409	14.3369	13.0227	41.2371	41.3924
SP8	HES5_1	cluster_2	DP	10732	12374.106	12098	12648	170.3633	0.8673	-0.2054	0.001	0.0010435	9348	103156	59.163	1941	21036	12.065	979	10732	6.1551	10.4728	10.4037	50.4379	51.0173
SP4	CTCF_known2	cluster_4	SP4	8878	10237.712	9843	10602	230.9727	0.8672	-0.2056	0.001	0.0010435	15039	241923	28.953	2287	35597	4.2602	556	8878	1.0625	3.6971	3.6698	24.3113	24.9403
DN	E2F8_1	cluster_2	DP	8048	9282.917	9120	9425	94.6923	0.867	-0.2059	0.001	0.001	9501	104773	81.344	1101	11689	9.0751	743	8048	6.2483	7.8202	7.6814	67.4841	68.8511
SP4	EGR3_3	cluster_2	DP	42031	48480.338	48000	48972	320.1087	0.867	-0.2059	0.001	0.001	31018	457668	70.037	4864	69807	10.683	2867	42031	6.432	9.243	9.1837	58.9433	60.2103
SP8	STAT_known2	cluster_4	SP4	9875	11390.793	11089	11713	192.5812	0.8669	-0.206	0.001	0.001	8911	178852	67.814	910	17211	6.5258	502	9875	3.7442	5.6335	5.5213	55.1648	57.3761
DN	E2F2_2	cluster_2	DP	4954	5714.996	5544	5882	100.5736	0.8669	-0.2061	0.001	0.0010909	3788	64579	78.928	465	7560	9.2398	298	4954	6.0548	7.8669	7.6712	64.086	65.5291
DN	SOX14_3	cluster_2	DP	4610	5318.543	5166	5511	101.8401	0.8668	-0.2062	0.001	0.0010909	4092	57347	70.134	592	7830	9.5759	336	4610	5.6379	8.2111	8.0388	56.7568	58.8761
SP4	POU2F2_known14	cluster_2	DP	809	933.443	828	1034	61.2926	0.8668	-0.2062	0.017	0.018545	922	14165	62.953	114	1601	7.1152	55	809	3.5954	5.9653	5.7113	48.2456	50.5309
SP4	ATF3_known4	cluster_1	SP8	6477	7472.865	7258	7703	137.8777	0.8668	-0.2063	0.001	0.001	8312	117305	66.718	825	11313	6.4343	462	6477	3.6838	5.5582	5.5215	56	57.2527
SP4	HIF1A::ARNT_1	cluster_1	SP8	3189	3679.569	3580	3777	65.1225	0.8667	-0.2064	0.001	0.001	10457	75241	66.952	775	5516	4.9083	447	3189	2.8377	4.2746	4.2384	57.6774	57.8136
SP8	ETV6_1	cluster_4	SP4	29616	34175.768	33610	34723	325.1325	0.8666	-0.2066	0.001	0.001	26153	376727	62.445	3975	55382	9.18	2082	29616	4.9091	7.9608	7.8614	52.3774	53.4759
SP8	CTCF_known1	cluster_2	DP	59594	68793.478	68024	69773	524.2271	0.8663	-0.2071	0.001	0.0010435	55221	1020372	58.86	6625	118066	6.8106	3260	59594	3.4376	5.9036	5.8404	49.2075	50.4752
DN	FOXO4_2	cluster_2	DP	8735	10089.209	9867	10292	132.3333	0.8658	-0.2079	0.001	0.0010435	7348	96298	75.255	1117	13917	10.876	685	8735	6.8262	9.3223	9.0708	61.325	62.765
DP	SOX10_9	cluster_5	Common_1_DN	6207	7169.694	6874	7414	161.7971	0.8657	-0.208	0.001	0.0010435	2814	39418	46.89	1186	15642	18.607	449	6207	7.3836	15.9559	15.7466	37.8583	39.6816
DP	SOX18_2	cluster_5	Common_1_DN	4915	5677.28	5430	5910	145.1593	0.8658	-0.208	0.001	0.0010909	2288	32032	48.779	919	11986	18.252	365	4915	7.4846	15.9528	15.344	39.7171	41.0062
DN	HESX1_1	cluster_2	DP	697	805.296	744	873	41.3766	0.8657	-0.2081	0.008	0.0087273	1328	11959	60.289	158	1360	6.8562	79	697	3.5138	5.9488	5.8282	50	51.25
SP4	SPDEF_6	cluster_2	DP	10848	12531.117	12255	12804	165.4274	0.8657	-0.2081	0.001	0.001	8360	117397	67.827	1390	18717	10.814	779	10848	6.2675	9.3182	9.2404	56.0432	57.958
SP8	TCF12_known1	cluster_2	DP	28112	32481.937	32102	32857	245.2083	0.8655	-0.2084	0.001	0.0010909	34302	246920	53.035	8606	61446	13.198	3912	28112	6.0381	11.4046	11.3851	45.4567	45.7507
SP8	SMAD4_1	cluster_4	SP4	22766	26308.081	25794	26855	316.1481	0.8654	-0.2086	0.001	0.001	25393	360702	58.545	3269	45282	7.3496	1614	22766	3.6951	6.3561	6.3116	49.3729	50.276
DN	AHR::ARNT::HIF1A_1	cluster_2	DP	10320	11926.856	11760	12075	96.3727	0.8653	-0.2088	0.001	0.0010435	14502	119460	81.912	1807	14673	10.061	1264	10320	7.0762	8.716	8.6389	69.9502	70.3333
DN	ATF6_1	cluster_2	DP	2340	2704.592	2631	2779	45.1883	0.8652	-0.2088	0.001	0.0010435	4516	31617	73.316	534	3697	8.573	335	2340	5.4262	7.4181	7.4011	62.7341	63.2946
DN	NFKB_known11	cluster_2	DP	15219	17590.508	17304	17855	157.9463	0.8652	-0.2089	0.001	0.001	14339	173964	75.858	1997	23563	10.275	1273	15219	6.6364	8.8779	8.7484	63.7456	64.5885
SP8	FLI1_2	cluster_4	SP4	6730	7778.556	7524	8031	147.7688	0.8652	-0.2089	0.001	0.001	6676	86936	61.84	1001	12687	9.0246	521	6730	4.7872	7.8041	7.7413	52.048	53.0464
DN	IKZF2_3	cluster_2	DP	4618	5341.289	5218	5448	70.1864	0.8646	-0.2099	0.001	0.0010909	7087	71094	80.484	728	6899	7.8102	478	4618	5.2279	6.7447	6.4956	65.6593	66.9372
SP8	RBPJ_2	cluster_2	DP	8269	9564.839	9299	9824	163.7484	0.8645	-0.21	0.001	0.0010909	10072	100834	56.034	1775	17292	9.6093	830	8269	4.5951	8.2407	8.2006	46.7606	47.8198
SP4	E2F_known24	cluster_2	DP	3984	4608.658	4422	4804	114.218	0.8645	-0.2101	0.001	0.001	3037	51804	67.185	441	7068	9.1666	238	3984	5.1669	7.8367	7.6905	53.9683	56.3667
DP	IRF4_2	cluster_5	Common_1_DN	12601	14583.06	14226	14985	235.9747	0.8641	-0.2107	0.001	0.001	5446	77752	53.165	2042	28028	19.165	894	12601	8.6162	16.4157	16.2067	43.7806	44.9586
DN	RUNX3_3	cluster_2	DP	43428	50261.777	49852	50723	266.9637	0.864	-0.2108	0.001	0.001	29706	527830	81.113	3611	62662	9.6295	2449	43428	6.6737	8.2441	8.2276	67.8205	69.3052
SP8	MTF1_1	cluster_2	DP	55177	63862.563	63211	64514	388.7679	0.864	-0.2109	0.001	0.001	38926	519131	65.795	7485	97795	12.395	4154	55177	6.9932	10.6715	10.6287	55.4977	56.4211
DP	POU2F2_known1	cluster_5	Common_1_DN	8514	9856.357	9534	10173	206.3065	0.8638	-0.2112	0.001	0.0010435	2722	49255	50.993	1186	19829	20.529	481	8514	8.8145	17.6708	17.2856	40.5565	42.9371
DP	IRF_known21	cluster_5	Common_1_DN	14771	17103.22	16713	17470	225.7161	0.8636	-0.2115	0.001	0.001	6273	90082	51.133	2468	34061	19.334	1045	14771	8.3844	16.6587	16.3973	42.342	43.3663
DN	RUNX3_1	cluster_2	DP	31628	36628.245	36257	36985	216.9234	0.8635	-0.2117	0.001	0.0010435	25951	398073	82.383	3043	45082	9.3299	2085	31628	6.5455	8.0344	7.9453	68.5179	70.1566
DN	SPDEF_6	cluster_2	DP	13377	15493.058	15271	15686	120.2893	0.8634	-0.2119	0.001	0.001	10392	145946	84.321	1390	18717	10.814	968	13377	7.7287	9.3149	9.1657	69.6403	71.4698
SP4	MYCN_2	cluster_2	DP	14358	16629.655	16365	16894	160.505	0.8634	-0.2119	0.001	0.001	17249	155885	64.493	2916	25887	10.71	1595	14358	5.9402	9.2469	9.2106	54.6982	55.4641
DN	SOX2_6	cluster_2	DP	3005	3480.8	3322	3642	91.5987	0.8633	-0.212	0.001	0.0010435	2362	37807	70.103	347	5211	9.6623	194	3005	5.5719	8.2134	7.9483	55.9078	57.6665
SP4	ELK4_1	cluster_1	SP8	3495	4048.393	3940	4148	64.5546	0.8633	-0.212	0.001	0.001	10509	84669	76.516	674	5337	4.8231	439	3495	3.1584	4.1774	4.1278	65.1335	65.4862
SP4	HIF1A_1	cluster_2	DP	11518	13341.804	13076	13608	156.7773	0.8633	-0.212	0.001	0.0010435	11555	130065	67.885	1804	19792	10.33	1030	11518	6.0116	8.9139	8.8556	57.0953	58.1952
DN	POU2F2_known15	cluster_2	DP	3494	4049.57	3915	4177	77.7517	0.8628	-0.2128	0.001	0.0010435	3911	43212	75.23	523	5548	9.6588	322	3494	6.0829	8.2332	8.0857	61.5679	62.9776
SP4	ATF2_2	cluster_1	SP8	3409	3951.576	3804	4095	90.9657	0.8627	-0.213	0.001	0.0010435	5130	56548	65.885	567	6053	7.0525	312	3409	3.9719	6.0819	6.0285	55.0265	56.3192
DN	TCF7L2_known5	cluster_2	DP	20910	24240.293	23846	24654	242.2775	0.8626	-0.2132	0.001	0.0010435	13945	223921	71.487	2240	34675	11.07	1327	20910	6.6755	9.516	9.3381	59.2411	60.3028
DP	E2F_known26	cluster_5	Common_1_DN	4367	5062.766	4857	5279	130.4955	0.8626	-0.2132	0.001	0.0010435	1515	25773	59.594	551	8698	20.112	261	4367	10.098	17.2277	16.9441	47.3684	50.2069
DN	KLF4_1	cluster_2	DP	77527	89899.94	89399	90469	335.2081	0.8624	-0.2136	0.001	0.001	90604	870001	74.9	12881	121193	10.442	8090	77527	6.6745	8.929	8.9111	62.8057	63.9171
SP4	MYC_known20	cluster_2	DP	14128	16384.077	16010	16825	243.9686	0.8623	-0.2137	0.001	0.001	8841	144867	57.464	1812	28806	11.426	867	14128	5.6041	9.8066	9.7524	47.8477	49.0453
SP8	ERG_2	cluster_4	SP4	7285	8448.058	8212	8693	142.387	0.8623	-0.2137	0.001	0.001	6800	88560	61.21	1107	13976	9.6597	564	7285	5.0351	8.2941	8.2261	50.9485	52.1251
DP	TBX21_4	cluster_5	Common_1_DN	19514	22649.667	22236	23079	271.3512	0.8616	-0.215	0.001	0.0010909	7045	105923	52.17	3068	44178	21.759	1319	19514	9.6111	18.7225	18.4228	42.9922	44.1713
DP	TCF7L2_known5	cluster_5	Common_1_DN	27249	31629.344	31065	32174	333.7083	0.8615	-0.2151	0.001	0.0010435	9748	156401	49.931	4181	64167	20.485	1718	27249	8.6992	17.6241	17.4225	41.0906	42.4658
SP4	IRF6_1	cluster_2	DP	2747	3191.718	3033	3343	92.6619	0.8607	-0.2164	0.001	0.0010909	2762	44279	67.58	317	4808	7.3381	175	2747	4.1925	6.336	6.2038	55.205	57.1339
SP4	MTF1_1	cluster_2	DP	54645	63496.026	62849	64156	385.1712	0.8606	-0.2166	0.001	0.001	38636	515202	65.297	7485	97795	12.395	4111	54645	6.9257	10.6403	10.6065	54.9232	55.8771
SP8	EGR4_1	cluster_2	DP	29910	34757.866	34359	35148	231.3201	0.8605	-0.2167	0.001	0.001	30552	346098	68.54	4596	51128	10.125	2669	29910	5.9232	8.7359	8.6421	58.0722	58.5002
DP	IRF_known14	cluster_5	Common_1_DN	39826	46287.856	45510	47067	494.2572	0.8604	-0.2169	0.001	0.001	11310	235509	53.453	4593	89191	20.244	1963	39826	9.0393	17.3563	16.9106	42.739	44.6525
DP	MAF_known10	cluster_5	Common_1_DN	16530	19215.667	18862	19553	203.5088	0.8602	-0.2172	0.001	0.0010909	7909	87167	53.303	3417	36443	22.285	1514	16530	10.108	19.1427	18.9636	44.3079	45.3585
SP8	TCF3_1	cluster_2	DP	78940	91774.8	90918	92658	552.5879	0.8602	-0.2173	0.001	0.0011429	45409	645019	52.166	12668	177031	14.317	5553	78940	6.3843	12.2289	12.1358	43.8349	44.5911
SP8	LMO2_2	cluster_2	DP	2268	2638.551	2521	2761	75.911	0.8596	-0.2182	0.001	0.0010435	3634	29090	57.111	598	4669	9.1664	286	2268	4.4526	7.8701	7.7965	47.8261	48.5757
SP4	SP8_1	cluster_1	SP8	51001	59334.971	58761	59972	367.4069	0.8595	-0.2184	0.001	0.001	64724	792047	63.386	8014	95072	7.6085	4195	51001	4.0815	6.4814	6.4391	52.3459	53.6446
SP4	ETS_known13	cluster_1	SP8	6532	7600.933	7342	7817	148.7467	0.8594	-0.2186	0.001	0.001	10234	164235	67.178	743	11441	4.6798	413	6532	2.6718	4.0356	3.9772	55.5855	57.0929
SP8	MESP1_1	cluster_2	DP	35307	41082.956	40482	41570	327.1181	0.8594	-0.2186	0.001	0.001	28229	254858	48.512	9529	85097	16.198	3921	35307	6.7206	13.89	13.8536	41.1481	41.4903
DN	E2F_known24	cluster_2	DP	4649	5410.303	5247	5560	99.0608	0.8593	-0.2187	0.001	0.001	4320	61420	79.657	441	7068	9.1666	283	4649	6.0294	7.8546	7.5692	64.1723	65.7753
DN	RREB1_1	cluster_2	DP	87139	101401.988	100735	102009	393.6476	0.8593	-0.2187	0.001	0.001	60217	1008607	79.968	8626	133890	10.616	5505	87139	6.9088	9.1419	8.6395	63.8187	65.0825
SP4	EGR3_2	cluster_2	DP	63149	73484.995	72876	74101	386.3801	0.8593	-0.2187	0.001	0.001	45033	687127	69.845	7154	106180	10.793	4153	63149	6.419	9.2221	9.1903	58.0514	59.4735
SP8	ERG_4	cluster_4	SP4	7169	8345.558	8113	8624	152.035	0.859	-0.2192	0.001	0.001	6801	88600	61.768	1082	13662	9.5245	555	7169	4.9979	8.1606	8.0914	51.2939	52.474
SP8	MYBL2_4	cluster_2	DP	1914	2228.274	2122	2362	69.7862	0.859	-0.2192	0.001	0.0010435	1616	22677	64.03	263	3526	9.956	137	1914	5.4043	8.4777	8.4403	52.0913	54.2825
DN	IKZF3_1	cluster_2	DP	8188	9532.716	9346	9706	109.0382	0.859	-0.2194	0.001	0.0010435	8816	106058	78.616	1082	12441	9.222	698	8188	6.0694	7.9174	7.7203	64.5102	65.8146
DN	SP4_2	cluster_2	DP	92815	108064.515	107329	108784	443.2716	0.8589	-0.2195	0.001	0.001	66957	1120770	78.67	8726	140136	9.8366	5635	92815	6.515	8.4158	8.2814	64.5771	66.2321
DN	GATA_known17	cluster_2	DP	5242	6104.879	5904	6289	117.3398	0.8587	-0.2198	0.001	0.0010909	5009	80296	75.449	554	8351	7.8469	335	5242	4.9256	6.688	6.5283	60.4693	62.7709
DP	IRF4_1	cluster_5	Common_1_DN	6747	7859.15	7649	8117	143.0192	0.8585	-0.2201	0.001	0.0010435	2787	39153	57.73	1029	13863	20.441	492	6747	9.9482	17.6534	17.2324	47.8134	48.6691
DP	NFATC1_1	cluster_5	Common_1_DN	14875	17330.016	16853	17794	298.1251	0.8583	-0.2204	0.001	0.0010909	4535	87510	54.48	1853	32944	20.51	802	14875	9.2606	17.6847	16.9981	43.2812	45.1524
SP4	MYBL1_5	cluster_2	DP	3834	4467.616	4292	4660	108.9962	0.8582	-0.2206	0.001	0.0010435	2888	46605	66.715	445	6853	9.81	239	3834	5.4884	8.2756	8.2266	53.7079	55.9463
DN	RUNX_1	cluster_2	DP	32518	37900.6	37523	38290	238.4153	0.858	-0.221	0.001	0.0010435	34752	490327	76.602	3658	49981	7.8084	2328	32518	5.0802	6.6989	6.6319	63.6413	65.0607
DP	FOXO6_1	cluster_5	Common_1_DN	4785	5577.775	5368	5795	134.2152	0.8579	-0.2211	0.001	0.0010909	2247	29326	51.149	901	11153	19.453	374	4785	8.3458	16.6444	16.3166	41.5094	42.9033
SP8	ATF3_known4	cluster_2	DP	10535	12282.301	12010	12554	170.4657	0.8577	-0.2214	0.001	0.001	8603	121435	69.067	1302	17867	10.162	755	10535	5.9919	8.776	8.6754	57.9877	58.9635
DP	SOX8_9	cluster_5	Common_1_DN	5222	6092.727	5840	6360	165.3394	0.8571	-0.2224	0.001	0.0010909	2184	34944	45.366	940	13886	18.028	332	5222	6.7795	15.2015	14.9439	35.3191	37.6062
SP8	ARID3A_2	cluster_2	DP	1179	1375.808	1268	1490	69.4637	0.8571	-0.2225	0.008	0.0091429	1242	20101	63.924	155	2265	7.2031	75	1179	3.7494	6.0386	5.8654	48.3871	52.053
DP	FOXO4_3	cluster_5	Common_1_DN	3851	4493.512	4308	4685	108.1238	0.857	-0.2226	0.001	0.0010909	1828	23799	50.665	731	9058	19.283	303	3851	8.1983	16.5755	16.1814	41.4501	42.5149
DN	SOX5_3	cluster_2	DP	3109	3628.377	3455	3793	98.6955	0.8569	-0.2228	0.001	0.0011429	2922	43910	74.555	366	5129	8.7086	216	3109	5.2788	7.3922	7.0804	59.0164	60.6161
SP4	MYC_known13	cluster_2	DP	10174	11872.812	11583	12171	176.1964	0.8569	-0.2228	0.001	0.0010909	9038	99518	55.876	1982	21373	12	930	10174	5.7123	10.2899	10.2233	46.9223	47.6021
SP8	HES5_1	cluster_4	SP4	5517	6441.159	6252	6645	120.2239	0.8565	-0.2234	0.001	0.0010435	9348	103156	59.163	1020	10976	6.2951	501	5517	3.1642	5.3594	5.3482	49.1176	50.2642
DP	FOXO3_1	cluster_5	Common_1_DN	8946	10449.154	10100	10752	187.2741	0.8562	-0.224	0.001	0.0010435	4685	61564	56.333	1584	19294	17.655	704	8946	8.1859	15.0267	14.5312	44.4444	46.3667
SP4	KLF16_1	cluster_1	SP8	52026	60771.915	60154	61336	354.8336	0.8561	-0.2242	0.001	0.001	76586	858580	65.244	8719	94633	7.1912	4657	52026	3.9535	6.0807	6.0595	53.4121	54.9766
DN	KLF14_1	cluster_2	DP	18545	21670.871	21095	22285	368.6399	0.8558	-0.2247	0.001	0.001	16591	224164	22.673	7473	98037	9.916	1390	18545	1.8757	8.378	8.273	18.6003	18.9163
SP4	HES1_1	cluster_2	DP	10628	12423.534	12156	12687	170.7741	0.8555	-0.2252	0.001	0.0010435	9376	103478	59.348	1941	21036	12.065	969	10628	6.0955	10.3349	10.2708	49.9227	50.5229
SP4	ETS_known13	cluster_2	DP	15002	17538.703	17187	17921	235.4324	0.8554	-0.2254	0.001	0.001	10234	164235	67.178	1676	26269	10.745	936	15002	6.1363	9.146	9.1345	55.8473	57.1091
SP8	BATF_known1	cluster_4	SP4	4752	5555.639	5331	5766	135.1874	0.8554	-0.2254	0.001	0.0010435	3843	50078	57.839	763	9682	11.183	368	4752	5.4885	9.5759	9.4892	48.2307	49.0808
DN	RUNX1_4	cluster_2	DP	2614	3056.552	2944	3179	66.806	0.8553	-0.2256	0.001	0.0010909	3683	37261	73.637	442	4280	8.4583	265	2614	5.1659	7.1952	7.0154	59.9548	61.0748
DN	MYCN_2	cluster_2	DP	17405	20352.316	20115	20588	140.4619	0.8552	-0.2257	0.001	0.001	21157	191106	79.065	2916	25887	10.71	1939	17405	7.2009	9.1648	9.1075	66.4952	67.2345
SP4	EGR4_2	cluster_2	DP	72402	84680.724	83955	85416	442.255	0.855	-0.226	0.001	0.001	48413	794683	69.102	7829	124130	10.794	4457	72402	6.2958	9.2062	9.1108	56.9294	58.3276
DN	NFATC1_1	cluster_2	DP	8720	10199.691	9960	10415	143.5439	0.8549	-0.2261	0.001	0.0010909	6650	128410	79.943	757	13426	8.3585	461	8720	5.4287	6.9323	6.7907	60.8983	64.9486
SP4	E2F2_2	cluster_2	DP	4230	4948.573	4760	5107	113.8271	0.8548	-0.2263	0.001	0.0010909	3236	55175	67.435	465	7560	9.2398	250	4230	5.1699	7.7256	7.6665	53.7634	55.9524
SP4	HIF1A::ARNT_1	cluster_2	DP	6866	8032.411	7882	8202	97.3071	0.8548	-0.2263	0.001	0.001	10457	75241	66.952	1713	12082	10.751	963	6866	6.1096	9.2091	9.1253	56.2172	56.8283
SP8	TCF4_2	cluster_2	DP	34760	40665.028	40110	41222	320.3393	0.8548	-0.2264	0.001	0.001	28489	257334	47.872	9571	85376	15.882	3854	34760	6.4664	13.528	13.5077	40.2675	40.714
SP8	KLF14_1	cluster_2	DP	57860	67696.416	67156	68358	363.3564	0.8547	-0.2265	0.001	0.001	51058	690823	69.873	7473	98037	9.916	4325	57860	5.8523	8.4708	8.3755	57.875	59.0185
DN	POU2F2_known14	cluster_2	DP	1049	1227.558	1148	1300	46.0845	0.8547	-0.2266	0.001	0.0012632	1238	18993	84.41	114	1601	7.1152	72	1049	4.662	5.8158	5.5231	63.1579	65.5215
SP4	IRF4_2	cluster_2	DP	5443	6370.622	6169	6581	126.8758	0.8544	-0.227	0.001	0.001	6862	99125	67.779	723	9646	6.5957	390	5443	3.7218	5.6835	5.491	53.9419	56.4275
SP4	ATF2_1	cluster_1	SP8	553	647.437	601	695	27.3651	0.8544	-0.2271	0.001	0.0010435	1377	9639	70.922	131	917	6.7471	79	553	4.0689	5.7371	5.7371	60.3053	60.3053
DN	NFKB_known10	cluster_2	DP	13760	16109.536	15859	16350	151.4828	0.8542	-0.2274	0.001	0.001	12896	155666	77.254	1818	21243	10.542	1159	13760	6.8288	8.9873	8.8394	63.7514	64.7743
SP8	SP1_known3	cluster_4	SP4	27921	32687.991	32213	33182	307.8082	0.8542	-0.2274	0.001	0.001	39576	519194	65.135	4045	50952	6.3922	2163	27921	3.5028	5.4654	5.3778	53.4734	54.7986

SP8	RBPJ_1	cluster_2	DP	5648	6613.368	6449	6809	109.0106	0.854	-0.2276	0.001	0.0010435	10911	76393	53.125	1836	12593	8.7574	809	5648	3.9277	7.4145	7.3933	44.0632	44.8503
DP	FOXO4_2	cluster_5	Common_1_DN	10984	12862.231	12556	13188	189.7844	0.854	-0.2277	0.001	0.0010435	5614	73565	57.489	1895	23131	18.076	869	10984	8.5837	15.4792	14.931	45.8575	47.4861
SP8	ATF2_1	cluster_2	DP	574	672.305	627	710	25.4299	0.854	-0.2277	0.001	0.0010435	1370	9590	70.561	137	959	7.0561	82	574	4.2234	5.9854	5.9854	59.854	59.854
SP8	STAT_known2	cluster_2	DP	13471	15774.252	15389	16157	233.7976	0.854	-0.2277	0.001	0.001	8911	178852	67.814	1258	23721	8.9942	684	13471	5.1077	7.6759	7.5319	54.372	56.7893
DP	POU2F2_known14	cluster_5	Common_1_DN	1861	2179.563	2041	2319	81.875	0.8539	-0.2278	0.001	0.0012632	685	10526	46.78	353	4849	21.55	125	1861	8.2707	18.2482	17.68	35.4108	38.379
DN	RUNX2_5	cluster_2	DP	32001	37476.813	37038	37933	272.7372	0.8539	-0.2279	0.001	0.001	23276	408017	80.689	2750	46946	9.284	1840	32001	6.3285	7.9051	7.8431	66.9091	68.1656
DP	FOXM1_1	cluster_5	Common_1_DN	7099	8314.057	8086	8522	133.2516	0.8539	-0.2279	0.001	0.0010909	4728	37858	53.335	2009	15722	22.15	893	7099	10.001	18.8875	18.7517	44.45	45.1533
DN	FOXP3_2	cluster_2	DP	3299	3864.382	3770	3955	58.578	0.8537	-0.2281	0.001	0.0010909	5499	32994	72.569	909	5388	11.851	554	3299	7.256	10.0746	9.9988	60.9461	61.2287
DP	TBX1_2	cluster_5	Common_1_DN	14575	17071.94	16659	17519	253.7532	0.8537	-0.2281	0.001	0.0010909	4390	79089	50.708	2030	34408	22.061	830	14575	9.3449	18.9066	18.4286	40.8867	42.3593
DN	ARID5A_1	cluster_2	DP	804	941.933	881	1005	37.2451	0.8537	-0.2282	0.001	0.0012632	992	13600	80.227	100	1257	7.4151	62	804	4.7428	6.25	5.9118	62	63.9618
DP	E2F2_2	cluster_5	Common_1_DN	8002	9378.298	9088	9674	177.7077	0.8533	-0.2289	0.001	0.0010909	2804	47831	58.459	1050	16482	20.144	486	8002	9.78	17.3324	16.7297	46.2857	48.5499
SP4	MYBL1_3	cluster_2	DP	1385	1623.973	1527	1730	60.7037	0.8529	-0.2295	0.001	0.0010909	1511	16654	62.822	247	2626	9.9057	127	1385	5.2244	8.405	8.3163	51.417	52.7418
DP	POU2F2_known15	cluster_5	Common_1_DN	4731	5547.173	5334	5729	119.5793	0.8529	-0.2296	0.001	0.0010435	2667	29451	51.273	1058	11078	19.286	438	4731	8.2364	16.4229	16.064	41.3989	42.7063
SP8	HES7_1	cluster_4	SP4	5500	6449.391	6243	6671	125.6124	0.8528	-0.2297	0.001	0.0010435	9675	106849	60.312	1003	10767	6.0776	500	5500	3.1045	5.168	5.1475	49.8504	51.082
SP4	KLF4_1	cluster_1	SP8	47151	55296.993	54730	55794	305.6838	0.8527	-0.2299	0.001	0.001	78036	752571	64.791	9288	86550	7.4513	4974	47151	4.0593	6.374	6.2653	53.553	54.4783
SP8	NFATC1_3	cluster_2	DP	2648	3105.561	2939	3258	96.6609	0.8527	-0.2299	0.001	0.0010909	3018	39257	57.876	457	5575	8.2191	208	2648	3.9039	6.892	6.7453	45.5142	47.4978
DN	IKZF2_2	cluster_2	DP	3639	4268.515	4149	4376	66.8876	0.8526	-0.2301	0.001	0.0010435	4704	47046	78.526	584	5590	9.3305	371	3639	6.074	7.8869	7.735	63.5274	65.0984
DN	SOX17_5	cluster_2	DP	2911	3414.464	3259	3544	84.2583	0.8526	-0.2301	0.001	0.0011429	2792	39191	71.088	381	5012	9.0912	212	2911	5.2802	7.5931	7.4277	55.643	58.0806
DN	HIF1A_2	cluster_2	DP	19015	22325.158	22030	22592	163.5243	0.8517	-0.2315	0.001	0.001	15992	214291	80.94	2138	27848	10.519	1436	19015	7.1822	8.9795	8.8734	67.1656	68.2814
SP8	SOX5_3	cluster_5	Common_1_DN	5628	6607.713	6393	6827	132.3977	0.8518	-0.2315	0.001	0.0011429	2486	37371	63.453	789	10931	18.56	391	5628	9.5558	15.7281	15.0598	49.5564	51.4866
DP	NFKB_known11	cluster_5	Common_1_DN	23417	27500.976	27073	27941	262.533	0.8515	-0.2319	0.001	0.001	11257	136572	59.553	3973	46680	20.355	1949	23417	10.211	17.3137	17.1463	49.0561	50.165
DP	POU2F2_known16	cluster_5	Common_1_DN	4666	5479.995	5241	5717	142.449	0.8515	-0.2319	0.001	0.0010909	2099	27573	46.898	965	11945	20.317	363	4666	7.9363	17.2939	16.9224	37.6166	39.0624
DN	SOX10_9	cluster_2	DP	4615	5420.639	5230	5623	110.834	0.8514	-0.2321	0.001	0.0010435	4222	59134	70.343	597	7939	9.4439	338	4615	5.4898	8.0057	7.8043	56.6164	58.1307
SP4	RBPJ_1	cluster_2	DP	5822	6838.716	6652	7025	116.314	0.8514	-0.2322	0.001	0.0010435	11294	79074	54.99	1836	12593	8.7574	836	5822	4.0487	7.4022	7.3627	45.5338	46.232
DP	PBX1_4	cluster_5	Common_1_DN	4449	5226.918	5046	5397	104.4699	0.8512	-0.2324	0.001	0.0010909	2319	25948	48.177	1046	11037	20.492	401	4449	8.2603	17.2919	17.1458	38.3365	40.3099
SP4	SOX13_1	cluster_5	Common_1_DN	4827	5673.332	5468	5857	117.5762	0.8508	-0.233	0.001	0.0010909	2170	32640	65.561	667	9165	18.409	337	4827	9.6955	15.53	14.7886	50.5247	52.6678
SP8	MYBL2_4	cluster_4	SP4	1843	2166.232	2047	2287	74.3286	0.8509	-0.233	0.001	0.0010435	1616	22677	64.03	254	3426	9.6736	133	1843	5.2039	8.2302	8.1272	52.3622	53.7945
SP8	MTF1_1	cluster_4	SP4	24194	28438.269	27996	28919	284.1489	0.8508	-0.2332	0.001	0.001	38926	519131	65.795	3421	43884	5.5619	1842	24194	3.0664	4.7321	4.6605	53.8439	55.1317
SP8	EGR1_known12	cluster_2	DP	78808	92641.665	91880	93474	474.4953	0.8507	-0.2333	0.001	0.001	52057	864606	69.047	8547	136439	10.896	4804	78808	6.2936	9.2283	9.1149	56.2069	57.7606
DN	HIF1A_1	cluster_2	DP	13330	15673.001	15452	15877	134.8274	0.8505	-0.2336	0.001	0.0010435	13599	153110	79.913	1804	19792	10.33	1194	13330	6.9573	8.7801	8.7062	66.1863	67.3504
DN	RUNX_2	cluster_2	DP	26187	30798.773	30408	31148	229.7721	0.8503	-0.234	0.001	0.0010435	28180	397366	76.459	2986	40745	7.8399	1876	26187	5.0388	6.6572	6.5901	62.8265	64.2705
DP	PBX3_known1	cluster_5	Common_1_DN	13142	15456.084	15143	15751	190.8843	0.8503	-0.234	0.001	0.0010435	6370	70693	52.566	2777	29759	22.128	1191	13142	9.7722	18.697	18.5902	42.888	44.1614
SP4	EGR4_1	cluster_1	SP8	19974	23493.749	23158	23891	228.0961	0.8502	-0.2341	0.001	0.001	29753	337110	66.76	3232	35618	7.0536	1779	19974	3.9556	5.9792	5.9251	55.0433	56.0784
SP8	MYBL2_2	cluster_4	SP4	2445	2875.851	2711	3025	97.4959	0.8502	-0.2341	0.001	0.0010435	2285	34653	64.344	325	4617	8.5729	166	2445	4.5399	7.2648	7.0557	51.0769	52.9565
DN	ATF3_known4	cluster_2	DP	12050	14181.518	13942	14402	137.8336	0.8497	-0.235	0.001	0.001	10011	141182	80.298	1302	17867	10.162	862	12050	6.8535	8.6105	8.5351	66.2058	67.4428
DN	TCF7L1_2	cluster_2	DP	14459	17021.116	16709	17255	170.3657	0.8495	-0.2353	0.001	0.0010435	14657	161969	71.069	2272	24364	10.69	1328	14459	6.3443	9.0605	8.927	58.4507	59.3458
DN	BPTF_1	cluster_2	DP	16486	19410.836	19116	19740	189.038	0.8493	-0.2356	0.001	0.0010909	15844	211822	75.51	2078	26531	9.4577	1263	16486	5.8769	7.9715	7.7829	60.7796	62.1386
DN	TCF3_1	cluster_2	DP	34757	40924.288	40104	41726	523.3146	0.8493	-0.2356	0.001	0.0011429	20600	290144	23.465	12668	177031	14.317	2481	34757	2.811	12.0437	11.9792	19.5848	19.6333
SP4	SP1_known2	cluster_1	SP8	71369	84038.483	83387	84712	413.781	0.8492	-0.2357	0.001	0.001	112736	1565680	69.085	9541	124977	5.5146	5338	71369	3.1491	4.735	4.5583	55.948	57.1057
DN	TBX21_4	cluster_2	DP	14350	16904.136	16575	17226	186.4758	0.8489	-0.2363	0.001	0.0010909	9894	148765	73.27	1649	23664	11.655	973	14350	7.0677	9.8342	9.6461	59.0055	60.6406
SP8	CUX1_8	cluster_4	SP4	1129	1330.076	1231	1426	60.1804	0.8489	-0.2363	0.001	0.0011429	544	8813	66.353	140	2170	16.338	73	1129	8.5002	13.4191	12.8106	52.1429	52.0276
DN	SOX13_1	cluster_2	DP	2546	2999.519	2873	3127	75.6731	0.8489	-0.2364	0.001	0.0010909	2495	37512	75.346	309	4228	8.4923	180	2546	5.1139	7.2144	6.7872	58.2524	60.2176
DN	GMEB1_1	cluster_5	Common_1_DN	4623	5447.409	5138	5754	186.4275	0.8487	-0.2367	0.001	0.0010435	1931	30986	22.575	1557	24649	17.958	296	4623	3.368	15.3288	14.9196	19.0109	18.7553
DP	MGA_2	cluster_5	Common_1_DN	15120	17816.285	17393	18246	266.8346	0.8487	-0.2367	0.001	0.0010909	4878	83018	54.568	2036	33173	21.805	900	15120	9.9383	18.4502	18.2129	44.2043	45.5792
SP8	KLF16_1	cluster_2	DP	79998	94293.421	93545	94942	420.1524	0.8484	-0.2372	0.001	0.001	79277	887559	67.446	12962	141421	10.747	7176	79998	6.0791	9.0518	9.0133	55.3618	56.5673
SP4	LMO2_2	cluster_2	DP	2339	2757.413	2629	2884	72.5491	0.8483	-0.2373	0.001	0.0010435	3781	30272	59.431	598	4669	9.1664	294	2339	4.592	7.7757	7.7266	49.1639	50.0964
SP8	GATA_known16	cluster_2	DP	6791	8007.058	7660	8338	204.1971	0.8481	-0.2376	0.001	0.0010435	4509	95037	55.067	772	15025	8.7059	331	6791	3.9349	7.3409	7.1456	42.8756	45.198
DN	FOXO3_1	cluster_2	DP	7165	8450.451	8234	8662	130.0911	0.8479	-0.238	0.001	0.0010435	6232	81801	74.85	965	11860	10.852	565	7165	6.5562	9.0661	8.7591	58.5492	60.4132
DN	HES1_1	cluster_2	DP	38232	45091.72	44657	45519	279.5105	0.8479	-0.2381	0.001	0.001	30958	438366	78.548	4230	58105	10.411	2722	38232	6.8506	8.7926	8.7215	64.3499	65.7981
DN	ATF2_2	cluster_2	DP	4695	5537.856	5389	5684	88.2681	0.8478	-0.2382	0.001	0.0010435	5805	63978	74.542	694	7476	8.7104	428	4695	5.4702	7.373	7.3385	61.6715	62.801
DP	SMAD3_3	cluster_5	Common_1_DN	5932	6998.525	6821	7195	108.2108	0.8476	-0.2385	0.001	0.0010435	3577	32203	55.927	1443	12645	21.961	663	5932	10.302	18.5351	18.4206	45.9459	46.9118
SP4	SP1_known2	cluster_2	DP	141267	166660.135	165714	167597	595.148	0.8476	-0.2385	0.001	0.001	112736	1565680	69.085	17987	243799	10.758	10176	141267	6.2333	9.0264	9.0227	56.5742	57.944
SP4	GMEB1_1	cluster_1	SP8	2767	3265.729	3113	3419	88.2322	0.8473	-0.239	0.001	0.0010435	5959	97141	70.771	293	4645	3.3841	171	2767	2.0159	2.8696	2.8484	58.3618	59.5694
DP	TBX2_2	cluster_5	Common_1_DN	15450	18236.176	17920	18532	197.1844	0.8472	-0.2392	0.001	0.0010909	8541	85538	53.696	3547	34386	21.586	1562	15450	9.6986	18.2883	18.0621	44.0372	44.9311
DP	IKZF2_2	cluster_5	Common_1_DN	6278	7413.635	7214	7612	127.1801	0.8468	-0.2398	0.001	0.0010435	3311	33116	55.275	1427	13658	22.797	638	6278	10.479	19.2691	18.9576	44.7092	45.9657
DP	SMAD_2	cluster_5	Common_1_DN	41122	48562.744	47976	49133	335.7873	0.8468	-0.2399	0.001	0.0010435	20655	212873	53.488	9075	91574	23.01	3983	41122	10.333	19.2835	19.3176	43.8898	44.9058
DN	SPZ1_1	cluster_2	DP	82094	97013.218	96384	97682	392.8231	0.8462	-0.2409	0.001	0.001	58732	857887	77.786	9016	127108	11.525	5688	82094	7.4436	9.6847	9.5693	63.0878	64.586
DN	KLF12_2	cluster_2	DP	53234	62918.12	62435	63421	291.6685	0.8461	-0.2411	0.001	0.001	46999	672450	77.85	5930	81999	9.4931	3754	53234	6.1629	7.9874	7.9164	63.3052	64.9203
DN	MYBL1_3	cluster_2	DP	1677	1983.054	1902	2067	50.1087	0.8457	-0.2417	0.001	0.0010909	1868	20588	77.661	247	2626	9.9057	156	1677	6.3259	8.3512	8.1455	63.1579	63.8614
SP4	KLF12_2	cluster_1	SP8	37468	44327.395	43733	44855	357.1936	0.8453	-0.2425	0.001	0.001	39236	562601	65.133	5003	69033	7.992	2649	37468	4.3377	6.7515	6.6598	52.9482	54.2755
SP8	RUNX1_4	cluster_5	Common_1_DN	4721	5585.46	5412	5781	112.3883	0.8453	-0.2425	0.001	0.0010909	2766	28019	55.372	1062	10306	20.367	475	4721	9.3299	17.1728	16.8493	44.7269	45.8083
DN	PKNOX1_2	cluster_2	DP	16954	20058.529	19779	20365	182.1944	0.8452	-0.2426	0.001	0.0010435	13772	152898	72.377	2578	28014	13.261	1536	16954	8.0254	11.1531	11.0884	59.5811	60.5197
SP8	PATZ1_1	cluster_2	DP	96527	114219.15	113415	115146	522.4041	0.8451	-0.2428	0.001	0.001	74077	975342	66.217	13788	175131	11.89	7435	96527	6.5533	10.0369	9.8967	53.9237	55.117
DN	EWSR1::FLI1_1	cluster_2	DP	103323	122276.094	121220	123260	614.4985	0.845	-0.243	0.001	0.0010435	56590	1127364	69.149	9825	183050	11.228	5342	103323	6.3375	9.4398	9.165	54.3715	56.4452
DP	TBX21_6	cluster_5	Common_1_DN	24641	29161.787	28559	29709	350.4862	0.845	-0.243	0.001	0.0010909	7546	136200	53.52	3242	55459	21.793	1383	24641	9.6827	18.3276	18.0918	42.6589	44.431
SP8	ETS_known13	cluster_2	DP	14595	17272.437	16911	17604	209.4318	0.845	-0.243	0.001	0.001	10079	161754	66.163	1676	26269	10.745	913	14595	5.9699	9.0584	9.023	54.4749	55.5598
SP4	KLF13_1	cluster_1	SP8	20018	23694.147	23236	24112	275.7929	0.8449	-0.2432	0.001	0.001	20223	347988	63.027	2316	38167	6.9128	1184	20018	3.6257	5.8547	5.7525	51.1226	52.4485
SP8	POU2F2_known14	cluster_2	DP	798	944.747	846	1038	59.8624	0.8448	-0.2433	0.012	0.013714	928	14244	63.304	114	1601	7.1152	53	798	3.5465	5.7112	5.6024	46.4912	49.8438
DN	PKNOX2_3	cluster_2	DP	16394	19415.289	19106	19695	175.6448	0.8444	-0.244	0.001	0.0010435	13787	153514	71.649	2518	27407	12.792	1481	16394	7.6516	10.742	10.6792	58.8165	59.8168
DN	POU2F2_known1	cluster_2	DP	5685	6733.14	6548	6924	121.0877	0.8444	-0.2441	0.001	0.0010435	4025	72871	75.443	558	9322	9.651	326	5685	5.8856	8.0994	7.8015	58.4229	60.9848
DN	SPDEF_4	cluster_2	DP	10746	12728.28	12429	13014	168.726	0.8443	-0.2442	0.001	0.0010435	8815	132735	73.627	1256	17937	9.9495	739	10746	5.9607	8.3834	8.0958	58.8376	59.9097
DN	TCF4_1	cluster_2	DP	69985	82895.984	82396	83381	302.0931	0.8443	-0.2443	0.001	0.0010909	54166	490170	74.751	12410	111339	16.979	7727	69985	10.673	14.2654	14.2777	62.2643	62.8576
DN	ETS_known13	cluster_2	DP	17743	21019.565	20720	21307	179.8101	0.8441	-0.2445	0.001	0.001	12306	197388	80.739	1676	26269	10.745	1113	17743	7.2575	9.0444	8.9889	66.4081	67.5435
SP8	HES7_1	cluster_2	DP	10857	12866.592	12592	13134	168.543	0.8438	-0.245	0.001	0.0010435	9675	106849	60.312	1979	21462	12.114	989	10857	6.1284	10.2222	10.1611	49.9747	50.5871
SP8	STAT_known3	cluster_4	SP4	8953	10611.237	10314	10957	188.6365	0.8437	-0.2451	0.001	0.0010435	7066	141634	65.658	869	16490	7.6443	457	8953	4.1504	6.4676	6.3212	52.5892	54.2935
SP8	ELK4_1	cluster_4	SP4	3576	4238.715	4131	4338	65.154	0.8437	-0.2452	0.001	0.001	10505	84617	76.469	703	5569	5.0327	447	3576	3.2316	4.2551	4.2261	63.5846	64.2126
DN	TCF12_known1	cluster_2	DP	39070	46312.587	46000	46611	187.5671	0.8436	-0.2453	0.001	0.0010909	48943	352576	75.728	8606	61446	13.198	5447	39070	8.3917	11.1293	11.0813	63.2931	63.5843
SP8	MYBL1_3	cluster_2	DP	1315	1559.189	1460	1661	58.4653	0.8435	-0.2456	0.001	0.0010909	1462	16095	60.713	247	2626	9.9057	121	1315	4.9604	8.2763	8.1702	48.9879	50.0762
DN	KLF13_1	cluster_2	DP	31861	37788.51	37289	38290	316.4629	0.8431	-0.2461	0.001	0.001	24149	415074	75.178	3116	51120	9.2588	1892	31861	5.7707	7.8347	7.676	60.7189	62.3259
SP8	E2F2_3	cluster_2	DP	2583	3063.863	2910	3217	99.0277	0.8431	-0.2462	0.001	0.001	2069	35210	66.193	304	4770	8.9673	157	2583	4.8559	7.5882	7.336	51.6447	54.1509
DN	MTF1_1	cluster_2	DP	67022	79499.157	79003	80001	301.8214	0.8431	-0.2463	0.001	0.001	48686	649383	82.303	7485	97795	12.395	5055	67022	8.4944	10.3829	10.3209	67.5351	68.5332
SP8	SP4_2	cluster_2	DP	80783	95824.144	94972	96629	496.8847	0.843	-0.2463	0.001	0.001	59003	991063	69.566	8726	140136	9.8366	4890	80783	5.6704	8.2877	8.1511	56.0394	57.6461
DP	SOX2_6	cluster_5	Common_1_DN	3750	4450.524	4226	4657	129.7343	0.8426	-0.247	0.001	0.0010435	1589	25439	47.17	667	9744	18.068	246	3750	6.9533	15.4814	14.7411	36.8816	38.4852
SP8	IRF_known21	cluster_2	DP	5978	7094.728	6875	7325	140.7487	0.8426	-0.247	0.001	0.001	7912	114984	65.268	824	11147	6.3273	426	5978	3.3933	5.3842	5.199	51.699	53.6288
SP8	MYC_known13	cluster_2	DP	9980	11844.256	11568	12129	176.0617	0.8426	-0.2471	0.001	0.0010909	9016	99281	55.743	1982	21373	12	908	9980	5.6034	10.071	10.0523	45.8123	46.6944
DN	IKZF1_1	cluster_2	DP	10487	12446.898	12250	12658	122.7485	0.8426	-0.2472	0.001	0.0010435	11933	143526	82.189	1347	15540	8.8989	887	10487	6.0053	7.4332	7.3067	65.85	67.4839
SP4	EGR1_known12	cluster_1	SP8	49158	58355.749	57631	59094	444.599	0.8424	-0.2474	0.001	0.001	50263	834726	66.661	5616	89364	7.1366	3001	49158	3.9258	5.9706	5.8891	53.4366	55.0087
SP8	SPDEF_6	cluster_4	SP4	4800	5698.198	5512	5882	108.8978	0.8424	-0.2474	0.001	0.001	8460	118816	68.647	625	8420	4.8647	347	4800	2.7732	4.1017	4.0399	55.52	57.0071
DN	CBX5_1	cluster_2	DP	4032	4787.407	4648	4938	90.4659	0.8422	-0.2477	0.001	0.0010435	5190	57165	75.199	629	6602	8.6848	376	4032	5.304	7.2447	7.0533	59.7774	61.0724
SP4	SP4_1	cluster_1	SP8	66051	78424.328	77589	79217	491.0328	0.8422	-0.2477	0.001	0.001	80215	1441564	69.533	6913	116187	5.6042	3802	66051	3.1859	4.7398	4.5819	54.9978	56.8489
SP8	FLI1_4	cluster_4	SP4	7040	8358.871	8121	8626	145.6417	0.8422	-0.2477	0.001	0.001	7101	92567	63.122	1063	13399	9.1369	544	7040	4.8006	7.6609	7.6053	51.1759	52.5412
DP	IKZF1_2	cluster_5	Common_1_DN	30641	36389.596	35971	36803	243.3539	0.842	-0.2481	0.001	0.0010435	20144	161159	58.804	8047	62565	22.829	3871	30641	11.18	19.2166	19.0129	48.1049	48.9747
DN	SOX9_4	cluster_2	DP	7842	9314.059	9088	9531	130.4025	0.842	-0.2482	0.001	0.0010909	6382	95858	74.138	910	13004	10.058	534	7842	6.0652	8.3673	8.1809	58.6813	60.3045
DN	SOX8_9	cluster_2	DP	3940	4681.482	4494	4872	113.9372	0.8416	-0.2487	0.001	0.0010909	3303	52872	68.642	485	7176	9.3163	255	3940	5.1152	7.7203	7.452	52.5773	54.9052
SP8	IKZF1_1	cluster_2	DP	7722	9175.069	8919	9454	170.3821	0.8416	-0.2487	0.001	0.0010435	8739	105142	60.209	1347	15540	8.8989	648	7722	4.4219	7.415	7.3444	48.1069	49.6911
SP8	HESX1_2	cluster_2	DP	669	795.122	714	882	50.2932	0.8416	-0.2488	0.008	0.0096	899	12600	63.31	105	1325	6.6576	50	669	3.3615	5.5617	5.3095	47.619	50.4906
DN	MTF1_3	cluster_2	DP	20913	24855.546	24545	25172	180.2269	0.8414	-0.2492	0.001	0.001	16298	213937	79.022	2484	31937	11.797	1601	20913	7.7246	9.8233	9.7753	64.4525	65.482
DN	MYBL1_5	cluster_2	DP	4622	5497.175	5348	5649	92.8135	0.8408	-0.2501	0.001	0.0010435	3552	57284	82.002	445	6853	9.81	291	4622	6.6164	8.1926	8.0686	65.3933	67.4449
DN	TBX1_2	cluster_2	DP	10021	11920.658	11628	12193	175.9637	0.8407	-0.2504	0.001	0.0010909	6383	115007	73.738	979	16664	10.684	569	10021	6.425	8.9143	8.7134	58.1205	60.1356
DP	SMAD3_1	cluster_5	Common_1_DN	29995	35680.57	35279	36058	239.8318	0.8407	-0.2504	0.001	0.0010435	19225	159039	54.071	8111	66435	22.587	3623	29995	10.198	18.8453	18.8602	44.6677	45.1494
DN	HES5_1	cluster_2	DP	13463	16022.503	15793	16240	133.0693	0.8403	-0.2511	0.001	0.0010435	12165	134258	77.001	1941	21036	12.065	1228	13463	7.7214	10.0945	10.0277	63.2664	63.9998
SP8	MYCN_1	cluster_2	DP	10964	13052.492	12800	13335	166.918	0.84	-0.2515	0.001	0.001	10432	115067	63.978	1894	20515	11.406	996	10964	6.0961	9.5475	9.5284	52.5871	53.4438
DN	HES7_1	cluster_2	DP	13999	16666.161	16454	16889	141.3193	0.84	-0.2516	0.001	0.0010435	12542	138583	78.225	1979	21462	12.114	1276	13999	7.9019	10.1738	10.1015	64.477	65.2269
SP8	KLF7_1	cluster_2	DP	104231	124087.987	123141	125011	542.6674	0.84	-0.2516	0.001	0.001	75221	1217400	68.768	11820	183259	10.352	6514	104231	5.8877	8.6598	8.5618	55.11	56.8763
DP	SPIB_1	cluster_5	Common_1_DN	17680	21052.708	20784	21299	163.2135	0.8398	-0.2519	0.001	0.0010435	15545	93270	50.008	7127	42285	22.672	2957	17680	9.4793	19.0222	18.9557	41.4901	41.8115
SP4	EGR4_1	cluster_2	DP	28446	33873.627	33454	34260	239.7406	0.8398	-0.2519	0.001	0.001	29753	337110	66.76	4596	51128	10.125	2535	28446	5.6333	8.5201	8.4382	55.1567	55.6368
DN	SOX_1	cluster_2	DP	7974	9499.036	9301	9704	126.4268	0.8395	-0.2524	0.001	0.0010435	8206	98530	71.552	1171	13573	9.8566	675	7974	5.7907	8.2257	8.093	57.643	58.749
DN	FOXO4_3	cluster_2	DP	2629	3133.148	3012	3251	73.843	0.8391	-0.253	0.001	0.0010909	2636	34308	73.038	359	4443	9.4586	208	2629	5.5968	7.8907	7.6629	57.9387	59.1717
DP	TBX15_1	cluster_5	Common_1_DN	17811	21229.755	20786	21711	294.6034	0.839	-0.2533	0.001	0.0010909	5512	99328	52.536	2443	41328	21.859	1010	17811	9.4204	18.3237	17.9315	41.3426	43.0967
SP4	GMEB2_3	cluster_2	DP	2200	2622.68	2510	2760	75.6538	0.8389	-0.2534	0.001	0.0010435	2133	30178	72.468	267	3659	8.7866	158	2200	5.283	7.4074	7.2901	59.176	60.1257
SP8	MTF1_3	cluster_2	DP	16409	19560.47	19154	19950	240.0589	0.8389	-0.2534	0.001	0.001	12742	167257	61.78	2484	31937	11.797	1255	16409	6.061	9.8493	9.8107	50.5233	51.3793
DP	PBX1_2	cluster_5	Common_1_DN	5569	6639.038	6399	6904	147.5627	0.8389	-0.2535	0.001	0.0010435	2395	33982	48.403	1057	14017	19.965	397	5569	7.9323	16.5762	16.3881	37.5591	39.7303
DN	RUNX1_2	cluster_2	DP	5946	7091.839	6878	7288	130.6775	0.8385	-0.2542	0.001	0.0010435	5376	83982	74.445	694	10049	8.9078	396	5946	5.2708	7.3661	7.0801	57.0605	59.1701
SP4	HES7_1	cluster_2	DP	10831	12918.75	12623	13176	169.3081	0.8384	-0.2543	0.001	0.0010435	9737	107540	60.702	1979	21462	12.114	986	10831	6.1137	10.1263	10.0716	49.8231	50.4659
DP	ARID5B_1	cluster_5	Common_1_DN	9368	11174.468	10848	11477	186.7123	0.8384	-0.2544	0.001	0.0010909	4087	53311	52.552	1743	21717	21.408	737	9368	9.2347	18.0328	17.5724	42.2834	43.1367
SP4	RREB1_1	cluster_2	DP	69152	82496.73	81692	83344	520.1051	0.8382	-0.2546	0.001	0.001	47895	812464	64.416	8626	133890	10.616	4253	69152	5.4827	8.8798	8.5114	49.3044	51.6484
SP4	CUX1_9	cluster_5	Common_1_DN	1257	1500.047	1416	1599	58.4543	0.8381	-0.2548	0.001	0.0012632	597	10309	69.627	144	2263	15.284	77	1257	8.4898	12.8978	12.1932	53.4722	55.5457
SP4	E2F2_3	cluster_1	SP8	2149	2564.645	2415	2712	87.0083	0.838	-0.255	0.001	0.001	2073	35280	66.325	247	3972	7.4671	128	2149	4.04	6.1746	6.0913	51.8219	54.1037
SP4	SP4_1	cluster_2	DP	125558	149828.357	148903	150795	564.3564	0.838	-0.255	0.001	0.001	80215	1441564	69.533	12677	218773	10.552	7049	125558	6.0562	8.7876	8.7098	55.6046	57.3919
SP4	ATF3_known4	cluster_2	DP	9914	11833.094	11542	12133	185.0453	0.8378	-0.2553	0.001	0.001	8312	117305	66.718	1302	17867	10.162	710	9914	5.6387	8.5419	8.4515	54.5315	55.4878
SP8	MYCN_1	cluster_4	SP4	6428	7673.297	7473	7889	131.83	0.8377	-0.2554	0.001	0.001	10432	115067	63.978	1121	12081	6.7171	586	6428	3.574	5.6173	5.5863	52.2748	53.2075
SP8	MYBL2_2	cluster_2	DP	2766	3303.52	3150	3454	93.185	0.8373	-0.2561	0.001	0.0010435	2285	34653	64.344	362	5258	9.7631	183	2766	5.1359	8.0088	7.982	50.5525	52.6056
DN	GMEB2_3	cluster_2	DP	2425	2896.541	2791	2989	59.9423	0.8373	-0.2562	0.001	0.0010435	2360	33394	80.191	267	3659	8.7866	175	2425	5.8233	7.4153	7.2618	65.5431	66.2749
DN	TCF3_2	cluster_2	DP	83706	99974.95	99328	100653	400.1405	0.8373	-0.2562	0.001	0.0010435	39118	592154	77.209	8790	130696	17.041	5541	83706	10.914	14.1648	14.1358	63.0375	64.0463
SP8	SOX3_3	cluster_5	Common_1_DN	6842	8171.603	7906	8421	167.1867	0.8373	-0.2562	0.001	0.0010909	2699	43266	55.718	1032	15271	19.666	445	6842	8.8111	16.4876	15.8138	43.1202	44.8039

DP	RREB1_2	cluster_5	Common_1_DN	196079	234231.151	232631	235720	986.6839	0.8371	-0.2565	0.001	0.001	48689	1172396	58.91	18832	415372	20.871	8422	196079	9.8525	17.2975	16.7246	44.7218	47.2056
DP	SPIC_1	cluster_5	Common_1_DN	32816	39207.074	38641	39752	342.885	0.837	-0.2567	0.001	0.001	13902	181322	51.494	6133	77072	21.888	2547	32816	9.3196	18.3211	18.0982	41.5294	42.5784
SP8	GMEB2_3	cluster_2	DP	2161	2582.673	2460	2704	72.1714	0.8368	-0.2571	0.001	0.0010435	2094	29627	71.145	267	3659	8.7866	156	2161	5.1893	7.4499	7.294	58.427	59.0599
SP8	KLF4_1	cluster_2	DP	67277	80402.006	79817	80981	346.8545	0.8368	-0.2571	0.001	0.001	80637	776764	66.873	12881	121293	10.442	7003	67277	5.792	8.6846	8.6612	54.3669	55.4665
DP	FOXO3_4	cluster_5	Common_1_DN	3343	3997.008	3809	4199	116.3298	0.8364	-0.2577	0.001	0.0010909	1629	21240	50.833	658	8084	19.347	263	3343	8.0007	16.1449	15.7392	39.9696	41.3533
DP	TBX5_5	cluster_5	Common_1_DN	18846	22533.307	21992	23072	316.4219	0.8364	-0.2578	0.001	0.0010435	5461	103978	53.836	2353	42538	22.025	1004	18846	9.7577	18.3849	18.125	42.6689	44.3039
DP	SMAD_1	cluster_5	Common_1_DN	23653	28282.131	27983	28638	209.8876	0.8363	-0.2579	0.001	0.0010435	15462	123736	56.699	6404	50170	22.989	2975	23653	10.838	19.2407	19.1157	46.4553	47.1457
DN	RBPJ_2	cluster_2	DP	10970	13119.474	12905	13335	125.3041	0.8362	-0.2581	0.001	0.0010909	13859	138744	77.101	1775	17292	9.6093	1105	10970	6.0961	7.9732	7.9066	62.2535	63.4397
DP	FOXP3_1	cluster_5	Common_1_DN	20156	24108.191	23575	24631	312.0521	0.8361	-0.2583	0.001	0.0010909	7025	112814	50.937	3180	48216	21.77	1283	20156	9.1007	18.2633	17.8666	40.3459	41.8036
SP8	SPZ1_1	cluster_2	DP	68805	82308.338	81614	83025	416.3191	0.8359	-0.2585	0.001	0.001	49464	724618	65.702	9016	127108	11.525	4743	68805	6.2387	9.5888	9.4953	52.6065	54.1311
DP	LMO2_2	cluster_5	Common_1_DN	5121	6127.227	5944	6303	107.161	0.8358	-0.2588	0.001	0.0010435	3407	27268	53.534	1473	11555	22.685	645	5121	10.054	18.9316	18.7803	43.7882	44.3185
SP4	HESX1_2	cluster_5	Common_1_DN	2182	2611.136	2488	2735	76.694	0.8357	-0.2589	0.001	0.0014118	906	12691	63.767	323	4233	21.269	160	2182	10.964	17.66	17.1933	49.5356	51.5474
DN	TBX2_1	cluster_2	DP	17570	21028.615	20706	21376	185.1027	0.8355	-0.2592	0.001	0.0010435	11067	188839	77.429	1691	27780	11.391	1046	17570	7.2042	9.4515	9.3042	61.8569	63.2469
SP4	SP4_2	cluster_1	SP8	50667	60641.268	60000	61435	447.15	0.8355	-0.2592	0.001	0.001	57022	957821	67.232	5789	92205	6.4721	3096	50667	3.5565	5.4295	5.2898	53.4807	54.9504
DN	MGA_3	cluster_2	DP	10444	12500.201	12243	12760	158.4591	0.8355	-0.2593	0.001	0.0010909	6415	109267	77.12	1019	16639	11.744	627	10444	7.3713	9.774	9.5582	61.5309	62.7682
DN	TCF21_1	cluster_2	DP	27349	32740.424	32379	33109	220.5752	0.8353	-0.2596	0.001	0.001	16797	218881	77.73	3322	42596	15.127	2108	27349	9.7123	12.5499	12.4949	63.4557	64.2056
DN	PKNOX2_2	cluster_2	DP	19479	23320.646	23045	23627	179.2233	0.8353	-0.2597	0.001	0.0010435	15871	176400	72.498	2996	32542	13.374	1763	19479	8.0056	11.1083	11.0425	58.8451	59.858
DN	MAF_known1	cluster_2	DP	43076	51576.042	51026	52042	304.5479	0.8352	-0.2598	0.001	0.001	33530	506311	73.776	4920	71240	10.381	2894	43076	6.2767	8.6311	8.5078	58.8211	60.466
DN	STAT_known2	cluster_2	DP	16172	19363.035	19058	19632	181.5692	0.8352	-0.2598	0.001	0.001	11028	221310	83.913	1258	23721	8.9942	832	16172	6.1318	7.5444	7.3074	66.1367	68.1759
SP4	EGR1_known12	cluster_2	DP	74795	89555.856	88625	90455	527.0437	0.8352	-0.2598	0.001	0.001	50263	834726	66.661	8547	136439	10.896	4560	74795	5.9731	9.0723	8.9604	53.3521	54.8194
DN	GATA_known16	cluster_2	DP	8918	10683.231	10397	10983	180.6113	0.8348	-0.2605	0.001	0.0010435	6076	128052	74.197	772	15025	8.7059	436	8918	5.1673	7.1758	6.9644	56.4767	59.3544
DN	SOX3_3	cluster_2	DP	4142	4963.073	4781	5157	113.5909	0.8346	-0.2608	0.001	0.0010909	3414	54700	70.442	486	7333	9.4434	268	4142	5.3341	7.85	7.5722	55.144	56.4844
DN	ETV6_1	cluster_2	DP	41478	49708.453	49230	50175	268.888	0.8344	-0.2611	0.001	0.001	32656	469723	77.86	4600	64454	10.684	2898	41478	6.8753	8.8743	8.8303	63	64.3529
SP8	REL_2	cluster_2	DP	8466	10151.639	9930	10377	130.7017	0.834	-0.2619	0.001	0.0010435	12473	112795	62.62	1867	16362	9.0836	944	8466	4.7	7.5683	7.5057	50.5624	51.7418
SP8	RREB1_1	cluster_2	DP	69594	83449.212	82566	84337	553.6064	0.834	-0.2619	0.001	0.001	48842	818149	64.867	8626	133890	10.616	4351	69594	5.5178	8.9083	8.5063	50.4405	51.9785
DN	HIF1A::ARNT_1	cluster_2	DP	8065	9672.383	9538	9804	82.1568	0.8338	-0.2622	0.001	0.001	12592	90566	80.589	1713	12082	10.751	1135	8065	7.1765	9.0137	8.9051	66.258	66.7522
DN	STAT_known3	cluster_2	DP	12561	15064.261	14785	15324	164.3997	0.8338	-0.2622	0.001	0.0010435	8877	177940	82.488	997	18856	8.7411	648	12561	5.8229	7.2998	7.0591	64.995	66.6154
SP8	CUX1_9	cluster_2	DP	534	640.804	556	708	45.7461	0.8336	-0.2626	0.02	0.024	577	9969	67.331	63	1004	6.781	32	534	3.6066	5.5459	5.3566	50.7937	53.1873
DN	SPIC_1	cluster_2	DP	21506	25809.419	25478	26131	205.6435	0.8333	-0.2631	0.001	0.001	19310	251988	71.563	2896	36649	10.408	1669	21506	6.1076	8.6432	8.5345	57.6312	58.681
SP8	KLF12_1	cluster_2	DP	10870	13047.459	12856	13241	126.7916	0.8331	-0.2634	0.001	0.001	19874	119244	60.476	3661	21678	10.994	1819	10870	5.5128	9.1527	9.1158	49.6859	50.143
SP4	STAT_known2	cluster_2	DP	13321	15991.757	15555	16423	252.8557	0.833	-0.2636	0.001	0.001	9013	180903	68.592	1258	23721	8.9942	679	13321	5.0508	7.5336	7.3636	53.9746	56.157
DP	TBX20_5	cluster_5	Common_1_DN	32060	38495.581	37862	39115	374.9665	0.8328	-0.2639	0.001	0.0010909	11935	182788	54.157	5012	72644	21.523	2138	32060	9.4989	17.9137	17.5394	42.6576	44.133
DN	ID4_1	cluster_2	DP	60583	72748.785	72265	73205	279.2441	0.8328	-0.264	0.001	0.0010435	48845	442152	75.691	10793	96618	16.54	6703	60583	10.371	13.723	13.7018	62.1051	62.7036
DP	NFKB_known10	cluster_5	Common_1_DN	21012	25232.732	24849	25617	237.6395	0.8327	-0.2641	0.001	0.001	10029	121099	60.099	3651	42572	21.128	1763	21012	10.428	17.579	17.3511	48.2881	49.3564
DP	MYBL1_5	cluster_5	Common_1_DN	7375	8862.573	8613	9115	148.7215	0.8322	-0.265	0.001	0.0010435	2741	44252	63.347	933	14337	20.523	466	7375	10.557	17.0011	16.6659	49.9464	51.4403
SP4	MTF1_3	cluster_2	DP	16581	19927.939	19582	20310	221.036	0.8321	-0.2652	0.001	0.001	12953	170029	62.803	2484	31937	11.797	1267	16581	6.1245	9.7815	9.7519	51.0064	51.9178
DP	TBX19_1	cluster_5	Common_1_DN	10891	13090.351	12715	13488	233.5533	0.832	-0.2653	0.001	0.0010435	3308	63063	49.328	1515	27072	21.176	580	10891	8.5189	17.5333	17.27	38.2838	40.2298
DN	SMAD3_3	cluster_2	DP	3539	4254.065	4144	4382	69.2133	0.8319	-0.2654	0.001	0.0010435	4812	43323	75.24	650	5760	10.003	395	3539	6.1462	8.2086	8.1689	60.7692	61.441
DP	TBX2_1	cluster_5	Common_1_DN	25371	30499.552	29883	31043	330.0003	0.8319	-0.2656	0.001	0.0010435	8226	140221	57.494	3274	53684	22.012	1505	25371	10.403	18.2956	18.0936	45.9682	47.2599
SP4	CUX1_5	cluster_2	DP	3482	4185.953	4042	4328	89.0231	0.8319	-0.2656	0.001	0.0010435	4490	49467	65.073	629	6602	8.6848	324	3482	4.5805	7.216	7.039	51.5103	52.7416
SP4	RREB1_2	cluster_2	DP	104824	126064.521	124677	127366	796.4778	0.8315	-0.2662	0.001	0.001	51020	1245776	62.597	9551	211427	10.624	4456	104824	5.2672	8.7338	8.4144	46.6548	49.5793
SP8	SP8_1	cluster_2	DP	71004	85398.922	84617	86200	435.7868	0.8314	-0.2663	0.001	0.001	67362	823083	65.87	11105	131511	10.525	5874	71004	5.6824	8.72	8.6266	52.8951	53.9909
DN	RBPJ_1	cluster_2	DP	8058	9692.649	9564	9824	81.8113	0.8314	-0.2664	0.001	0.0010435	16019	112173	78.007	1836	12593	8.7574	1162	8058	5.6037	7.2539	7.1835	63.2898	63.9879
SP8	KLF12_2	cluster_2	DP	45424	54665.041	54074	55282	360.3763	0.831	-0.2672	0.001	0.001	40686	583267	67.525	5930	81999	9.4931	3203	45424	5.2588	7.8725	7.7879	54.0135	55.3958
SP4	KLF7_1	cluster_1	SP8	66258	79750.249	78998	80480	465.085	0.8308	-0.2674	0.001	0.001	72331	1170811	66.136	8057	123464	6.9741	4214	66258	3.7427	5.826	5.6592	52.3023	53.6658
SP4	EGR4_2	cluster_1	SP8	39990	48150.98	47563	48770	375.8582	0.8305	-0.2679	0.001	0.001	48413	794683	69.102	4521	71084	6.1812	2468	39990	3.4774	5.0978	5.0322	54.5897	56.2574
DN	TBX15_1	cluster_2	DP	12521	15077.606	14781	15377	183.0815	0.8304	-0.268	0.001	0.0010909	7884	142057	75.135	1217	20690	10.943	714	12521	6.6225	9.0563	8.8141	58.6689	60.5172
DN	TBX1_4	cluster_2	DP	19967	24045.155	23672	24474	250.8444	0.8304	-0.2681	0.001	0.0010909	11107	211898	72.371	1881	34050	11.629	1062	19967	6.8194	9.5615	9.4229	56.4593	58.6402
DN	MAFG_1	cluster_2	DP	16487	19855.415	19505	20269	226.3036	0.8304	-0.2682	0.001	0.0010435	8246	165248	72.352	1475	28081	12.295	837	16487	7.2187	10.1504	9.9771	56.7458	58.7123
SP4	GATA_known17	cluster_2	DP	4134	4979.265	4767	5218	132.4826	0.8303	-0.2683	0.001	0.0010909	4062	65192	61.257	554	8351	7.8469	261	4134	3.8845	6.4254	6.3413	47.1119	49.5031
SP8	ATF2_2	cluster_4	SP4	3570	4301.698	4172	4463	89.8074	0.8299	-0.2689	0.001	0.0010435	5120	56438	65.757	616	6617	7.7096	326	3570	4.1595	6.3672	6.3255	52.9221	53.9519
SP8	NFATC2_1	cluster_2	DP	2348	2829.409	2720	2952	64.6377	0.8299	-0.269	0.001	0.0010909	5758	34548	61.898	798	4659	8.3474	396	2348	4.2068	6.8774	6.7963	49.6241	50.3971
DN	SP1_known3	cluster_2	DP	58913	71001.028	70496	71528	310.5911	0.8298	-0.2692	0.001	0.001	47992	629173	78.933	7198	91416	11.469	4550	58913	7.3909	9.4807	9.3636	63.212	64.445
DN	SOX1_3	cluster_2	DP	3993	4812.626	4633	4974	106.1461	0.8297	-0.2693	0.001	0.0010909	3794	53161	70.646	533	7047	9.3648	293	3993	5.3063	7.7227	7.5111	54.9719	56.6624
SP8	SOX12_1	cluster_2	DP	3033	3655.79	3499	3828	98.2084	0.8297	-0.2694	0.001	0.0010435	2969	38740	61.39	501	6174	9.7837	241	3033	4.8063	8.1172	7.8291	48.1038	49.1254
DN	MYC_known13	cluster_2	DP	13260	15984.185	15741	16224	147.471	0.8296	-0.2695	0.001	0.0010909	12241	134783	75.676	1982	21373	12	1208	13260	7.445	9.8685	9.838	60.9485	62.0409
SP8	ATF6_1	cluster_4	SP4	1899	2289.253	2204	2376	52.4017	0.8296	-0.2695	0.001	0.0010435	3930	27520	63.816	521	3605	8.3596	273	1899	4.4036	6.9466	6.9004	52.3992	52.6768
SP4	MAFG_1	cluster_2	DP	12954	15621.115	15161	16048	245.5543	0.8293	-0.2701	0.001	0.0010435	6447	129183	56.562	1475	28081	12.295	653	12954	5.6718	10.1287	10.0276	44.2712	46.1308
DN	SPI1_known4	cluster_2	DP	19870	23965.241	23663	24240	181.8283	0.8291	-0.2703	0.001	0.0010435	19284	251359	76.991	2515	31724	9.717	1547	19870	6.0861	8.0222	7.905	61.5109	62.634
SP8	IRF_known14	cluster_2	DP	17234	20785.361	20300	21288	285.8012	0.8291	-0.2703	0.001	0.001	13279	278624	63.239	1777	34195	7.7612	858	17234	3.9116	6.4613	6.1854	48.2836	50.3992
SP4	KLF12_1	cluster_2	DP	10510	12677.029	12467	12884	126.6387	0.8291	-0.2704	0.001	0.001	19302	115812	58.735	3661	21678	10.994	1758	10510	5.3303	9.1079	9.0751	48.0197	48.4823
DN	MESP1_1	cluster_2	DP	53578	64632.383	64238	65008	245.3023	0.829	-0.2706	0.001	0.001	44484	401632	76.45	9529	85097	16.198	5950	53578	10.198	13.3766	13.3401	62.441	62.9611
DN	LMO2_2	cluster_2	DP	2865	3457.007	3351	3555	64.3058	0.8288	-0.2709	0.001	0.0010435	4777	38246	75.086	598	4669	9.1664	362	2865	5.6247	7.578	7.491	60.5351	61.3622
SP8	E2F8_1	cluster_2	DP	6161	7433.788	7252	7659	119.9408	0.8288	-0.2709	0.001	0.001	7551	83269	64.648	1101	11689	9.0751	565	6161	4.7833	7.4825	7.3989	51.317	52.7077
DN	ETS_known17	cluster_2	DP	23959	28909.772	28495	29306	248.025	0.8288	-0.271	0.001	0.001	15240	260380	73.574	2413	39722	11.224	1414	23959	6.77	9.2782	9.2016	58.5993	60.3167
SP4	PATZ1_1	cluster_2	DP	91692	110639.701	109915	111387	466.5677	0.8287	-0.271	0.001	0.001	71621	944599	64.129	13788	175131	11.89	7059	91692	6.225	9.856	9.707	51.1967	52.3562
DN	SOX5_1	cluster_2	DP	1915	2311.141	2213	2403	56.5201	0.8287	-0.2711	0.001	0.0010909	2705	24345	72.35	373	3266	9.7061	215	1915	5.6911	7.9482	7.8661	57.6408	58.6344
SP8	GMEB2_2	cluster_4	SP4	2382	2874.658	2715	3033	93.2449	0.8287	-0.2711	0.001	0.001	2724	36104	65.136	360	4500	8.1185	183	2382	4.2974	6.7181	6.5976	50.8333	52.9333
SP8	MAF_known1	cluster_2	DP	33817	40813.118	40170	41419	366.8915	0.8286	-0.2713	0.001	0.001	26418	399043	58.146	4920	71240	10.381	2260	33817	4.9276	8.5548	8.4745	45.935	47.4691
DP	TBX4_2	cluster_5	Common_1_DN	22825	27550.355	27005	28018	327.4409	0.8285	-0.2714	0.001	0.0010909	6487	123601	55.329	2786	50614	22.657	1215	22825	10.218	18.7298	18.4667	43.6109	45.0962
DN	FOXO6_3	cluster_2	DP	33178	40048.619	39617	40416	245.1678	0.8284	-0.2715	0.001	0.0010435	28591	373838	76.349	4217	53359	10.898	2571	33178	6.776	8.9923	8.875	60.9675	62.1788
SP4	E2F8_1	cluster_2	DP	6082	7342.852	7113	7566	131.2992	0.8283	-0.2718	0.001	0.001	7471	82374	63.953	1101	11689	9.0751	558	6082	4.7219	7.4689	7.3834	50.6812	52.0318
SP8	MYCN_2	cluster_4	SP4	6636	8012.331	7819	8203	114.6399	0.8282	-0.2719	0.001	0.001	17431	157501	65.162	1415	12426	5.1409	743	6636	2.7455	4.2625	4.2133	52.5088	53.4042
DN	ATF4_3	cluster_2	DP	5073	6126.048	5954	6293	105.7539	0.8281	-0.2721	0.001	0.0010909	4863	63288	72.723	685	8615	9.8993	398	5073	5.8293	8.1842	8.0157	58.1022	58.8857
SP4	AHR::ARNT::HIF1A_1	cluster_1	SP8	3643	4399.465	4274	4523	75.5056	0.8281	-0.2721	0.001	0.0010435	12561	103445	70.93	761	6233	4.2739	444	3643	2.4979	3.5348	3.5217	58.3443	58.447
DP	RBPJ_1	cluster_5	Common_1_DN	14662	17708.22	17445	17991	168.6188	0.828	-0.2723	0.001	0.0010435	10434	73042	50.795	5146	35199	24.478	2113	14662	10.196	20.2511	20.0734	41.061	41.6546
SP8	ATF2_1	cluster_4	SP4	607	733.302	691	784	29.0941	0.828	-0.2723	0.001	0.0010435	1370	9590	70.561	151	1053	7.7478	87	607	4.4662	6.5504	6.3295	57.6159	57.6448
SP8	REL_1	cluster_2	DP	8243	9956.297	9744	10188	129.845	0.8279	-0.2724	0.001	0.001	12386	112079	60.739	1831	16059	8.6675	918	8243	4.4029	7.4116	7.3546	50.1365	51.3295
DP	REL_2	cluster_5	Common_1_DN	17113	20676.422	20356	21032	202.199	0.8277	-0.2729	0.001	0.0010435	11409	103099	57.237	4147	36451	20.236	1914	17113	9.5005	16.7762	16.5986	46.1538	46.948
DP	MYBL2_4	cluster_5	Common_1_DN	3540	4279.132	4102	4472	107.1313	0.8273	-0.2735	0.001	0.0010435	1563	21943	61.958	524	7050	19.906	256	3540	9.9955	16.3788	16.1327	48.855	50.2128
SP4	CTCF_known2	cluster_1	SP8	13569	16402.084	15884	16941	332.11	0.8273	-0.2735	0.001	0.0010435	15039	241293	28.953	3610	56958	6.8167	848	13569	1.6239	5.6387	5.6088	23.4903	23.8228
SP8	POU2F2_known15	cluster_2	DP	2660	3215.647	3054	3349	90.8396	0.8273	-0.2736	0.001	0.0010435	3075	33949	59.103	523	5548	9.6588	243	2660	4.6309	7.9024	7.8353	46.4627	47.9452
SP4	ETS_known17	cluster_2	DP	20360	24620.191	24160	25054	268.4344	0.827	-0.2741	0.001	0.001	12925	220877	62.412	2413	39722	11.224	1199	20360	5.753	9.2766	9.2178	49.6892	51.2562
DN	EGR3_2	cluster_2	DP	17738	21453.472	20765	22102	399.4272	0.8268	-0.2744	0.001	0.001	13370	205199	20.858	7154	106180	10.793	1175	17738	1.803	8.7883	8.6443	16.4244	16.7056
SP8	MGA_3	cluster_2	DP	8074	9767.511	9448	10058	184.8329	0.8266	-0.2747	0.001	0.0010909	4956	84386	59.559	1019	16639	11.744	481	8074	5.6986	9.7054	9.5679	47.2031	48.5246
SP8	ATF7_1	cluster_4	SP4	4110	4972.71	4775	5159	120.1397	0.8265	-0.2748	0.001	0.001	3427	44593	61.045	650	8215	11.246	318	4110	5.6264	9.2793	9.2167	48.9231	50.0304
DN	TCF4_2	cluster_2	DP	53426	64638.638	64219	65060	244.9128	0.8265	-0.2749	0.001	0.001	45335	409696	76.215	9571	85376	15.882	5920	53426	9.9388	13.0583	13.0404	61.8535	62.5773
DN	SOX30_1	cluster_2	DP	5228	6325.855	6149	6522	114.3677	0.8265	-0.275	0.001	0.0010435	4394	65950	74.266	626	8864	9.9818	358	5228	5.8873	8.1475	7.9272	57.1885	58.9801
DN	SOX9_6	cluster_2	DP	6194	7495.532	7263	7716	135.775	0.8264	-0.2751	0.001	0.0010909	5272	79251	71.777	759	10845	9.8222	421	6194	5.6098	7.9856	7.8157	55.4677	57.1139
SP4	KLF16_1	cluster_2	DP	75455	91304.229	90574	92010	409.2285	0.8264	-0.2751	0.001	0.001	76586	858580	65.244	12962	141421	10.747	6763	75455	5.7339	8.8306	8.7883	52.1756	53.3549
SP8	MYC_known20	cluster_2	DP	13438	16263.393	15842	16681	255.2435	0.8263	-0.2753	0.001	0.001	8781	143953	57.101	1812	28806	11.426	821	13438	5.3304	9.3497	9.335	45.3091	46.65
DP	IRF6_1	cluster_5	Common_1_DN	6428	7780.951	7519	8057	159.8328	0.8261	-0.2755	0.001	0.0010909	2388	38278	58.421	895	13587	20.737	413	6428	9.8106	17.2948	16.1453	47.3099	47.3099
SP8	ETS_known17	cluster_4	SP4	14165	17147.39	16731	17563	246.484	0.8261	-0.2756	0.001	0.001	12384	211645	59.804	1774	29054	8.2097	841	14165	4.0025	6.791	6.6928	47.407	48.754
DN	MAFF_1	cluster_2	DP	17592	21304.01	20973	21643	199.7528	0.8258	-0.2762	0.001	0.001	12562	176219	70.456	2253	30581	12.227	1268	17592	7.0336	10.0939	9.983	56.2805	57.5259
DP	REL_1	cluster_5	Common_1_DN	16656	20173.51	19840	20543	210.104	0.8256	-0.2764	0.001	0.001	11260	101830	56.863	4073	35821	20.003	1861	16656	9.3008	16.5275	16.3567	45.6911	46.4979
DN	KLF12_1	cluster_2	DP	13278	16085.474	15915	16230	104.5699	0.8255	-0.2767	0.001	0.001	24570	147420	74.766	3661	21678	10.994	2224	13278	9.0517	9.0069	9.0069	60.7484	61.251
DN	SPIB_1	cluster_2	DP	11063	13402.511	13202	13599	117.5451	0.8254	-0.2767	0.001	0.0010435	22113	132678	71.137	3185	18963	10.167	1856	11063	5.9316	8.3933	8.3382	58.2732	58.3399
DN	MYBL2_2	cluster_2	DP	3361	4072.039	3926	4227	88.998	0.8254	-0.2768	0.001	0.0010435	2843	43039	79.915	362	5258	9.7631	227	3361	6.2407	7.9845	7.8092	62.7072	63.9216
DN	SOX10_4	cluster_2	DP	8244	9988.558	9737	10261	150.8254	0.8254	-0.2769	0.001	0.0010909	6860	103161	73.858	973	13974	10.005	558	8244	5.9023	8.1341	7.9914	57.3484	58.9953
DN	TBX5_3	cluster_2	DP	19714	23885.454	23603	24156	171.2433	0.8254	-0.2769	0.001	0.001	20740	186864	75.435	3634	32120	12.967	2208	19714	7.9584	10.6461	10.5499	60.7595	61.3761
DN	MAF_known11	cluster_2	DP	22032	26701.766	26287	27086	236.3488	0.8251	-0.2773	0.001	0.0010435	13743	221582	72.337	2417	37391	12.207	1382	22032	7.1925	10.056	9.943	57.1783	58.9233
DP	GATA_known16	cluster_5	Common_1_DN	14104	17094.427	16485	17577	322.8898	0.8251	-0.2774	0.001	0.0010435	4004	84333	48.865	1823	35888	20.795	680	14104	8.1723	16.983	16.7242	37.3012	39.3
DN	MAF_known10	cluster_2	DP	11516	13959.007	13734	14187	133.4268	0.825	-0.2775	0.001	0.0010909	11107	122400	74.849	1769	18966	11.598	1056	11516	7.0421	9.5075	9.4085	59.6947	60.7192
DN	SOX18_4	cluster_2	DP	2889	3502.218	3361	3654	91.5987	0.825	-0.2776	0.001	0.0012	2707	37982	72.115	385	5045	9.5787	212	2889	5.4852	7.8315	7.6062	55.0649	57.2646
SP4	SP4_2	cluster_2	DP	76490	92731.24	91859	93630	521.6904	0.8249	-0.2778	0.001	0.001	57022	957821	67.232	8726	140136	9.8366	4610	76490	5.3691	8.0846	7.9858	52.8306	54.5827
DP	MGA_3	cluster_5	Common_1_DN	14142	17149.593	16763	17526	250.5397	0.8246	-0.2782	0.001	0.0010909	4692	79902	56.395	1893	30937	21.835	839	14142	9.9814	17.8815	17.6992	44.3212	45.7123
DN	MYCN_1	cluster_2	DP	12863	15603.757	15378	15826	137.4607	0.8244	-0.2786	0.001	0.001	12503	137921	76.685	1894	20515	11.406	1173	12863	7.1519	9.3817	9.3264	61.9324	62.7005
DN	POU2F3_1	cluster_2	DP	5347	6486.645	6287	6680	124.2023	0.8243	-0.2787	0.001	0.0010435	4595	69456	74.624	635	9011	9.6815	370	5347	5.7449	8.0522	7.6984	58.2677	59.3386
SP8	MAF_known1	cluster_2	DP	32465	39389.043	38841	40065	373.387	0.8242	-0.2789	0.001	0.001	25525	385561	56.181	4920	71240	10.381	2170	32465	4.7306	8.5015	8.4202	44.1057	45.5713
DN	XBP1_4	cluster_2	DP	5187	6293.68	6065	6492	134.6261	0.8242	-0.279	0.001	0.0010435	5207	67800	61.985	808	10192	9.3178	403	5187	4.7421	7.7396	7.6504	49.8762	50.8929
SP4	KLF7_1	cluster_2	DP	98479	119500.486	118537	120427	576.0719	0.8241	-0.2791	0.001	0.001	72331	1170811	66.136	11820	183259	10.352	6148	98479	5.5628	8.4998	8.4112	52.0135	53.7376
SP8	NFATC2_1	cluster_5	Common_1_DN	5850	7098.697	6949	7242	88.3913	0.8241	-0.2791	0.001	0.0010909	5758	34548	61.898	1994	11648	20.869	990	5850	10.481	17.1935	16.933	49.6489	50.2232
DN	SOX8_2	cluster_2	DP	7449	9039.938	8854	9264	124.3054	0.824	-0.2792	0.001	0.0010909	6301	94659	74.032	886	12634	9.8809	508	7449	5.8258	8.0622	7.8693	57.3363	58.9599
DN	SOX15_2	cluster_2	DP	3973	4821.986	4675	4983	94.0034	0.824	-0.2793	0.001	0.0010909	3740	52487	73.258	510	6849	9.5594	291	3973	5.5452	7.7807	7.5695	57.0588	58.0085
DN	SOX7_4	cluster_2	DP	4007	4863.263	4657	5066	115.6744	0.824	-0.2793	0.001	0.0010909	3341	53567	71.584	471	7079	9.46	259	4007	5.3547	7.7522	7.4804	54.9894	56.604
SP4	KLF14_1	cluster_2	DP	53638	65100.526	64371	65776	401.8617	0.8239	-0.2794	0.001	0.001	49055	664031	67.164	7473	98037	9.916	4004	53638	5.4252	8.1623	8.0776	53.5796	54.712
DN	FOXO1_5	cluster_2	DP	32449	39390.746	39060	39787	225.2216	0.8238	-0.2797	0.001	0.0010435	32721	361528	75.797	4914	52812	11.072	2974	32449	6.8032	9.089	8.9755	60.521	61.4425
SP8	RREB1_2	cluster_2	DP	105300	127858.148	126560	129162	751.1569	0.8236	-0.28	0.001	0.001	51915	1257959	63.21	9551	211427	10.624	4508	105300	5.2911	8.6834	8.3707	47.1992	49.8044
SP8	IRF4_2	cluster_2	DP	5113	6209.028	5994	6394	124.4896	0.8235	-0.2801	0.001	0.001	6695	96589	66.045	723	9646	6.5957	366	5113	3.4961	5.4668	5.2936	50.6224	53.0064
SP4	RBPJ_2	cluster_2	DP	8076	9807.507	9541	10076	156.2289	0.8235	-0.2802	0.001	0.0010909	10316	103270	57.388	1775	17292	9.6093	814	8076	4.4879	7.8907	7.8203	45.8592	46.7037
DN	TBX20_2	cluster_2	DP	11580	14067.645	13800	14376	181.8501	0.8232	-0.2807	0.001	0.0010435	7160	129186	74.304	1123	19395	11.155	651	11580	6.6605	9.0922	8.9638	57.9697	59.7061
SP4	ARID3A_2	cluster_5	Common_1_DN	3398	4128.401	3949	4300	106.1924	0.8231	-0.2808	0.001	0.0013333	1263	20439	64.999	461	6775	21.546	221	3398	10.806	17.498	16.6251	47.9393	50.155
DN	E2F2_3	cluster_2	DP	3067	3726.94	3611	3851	75.8468	0.823	-0.2811	0.001	0.001	2541	43236	81.281	304	4770	8.9673	192	3067	5.7658	7.5561	7.0936	63.1579	64.2977
SP8	POU2F2_known13	cluster_5	Common_1_DN	8169	9928.151	9603	10215	193.1537	0.8228	-0.2813	0.001	0.0010909	2653	55941	63.012	863	16530	18.619	408	8169	9.2016	15.3788	14.6029	47.2769	49.4192
SP4	MGA_3	cluster_2	DP	8282	10065.974	9725	10419	197.0284	0.8228	-0.2814	0.001	0.0010909	5104	86900	61.334	1019	16639	11.744	493	8282	5.8454	9.6591	9.5305	48.3808	49.7746

SP8	XBP1_4	cluster_4	SP4	4618	5612.706	5402	5829	132.8476	0.8228	-0.2814	0.001	0.0010435	5176	67397	61.616	739	9250	8.4566	359	4618	4.2219	6.9359	6.8519	48.5792	49.9243
DP	MAF_known12	cluster_5	Common_1_DN	21776	26469.331	26003	26969	295.3244	0.8227	-0.2816	0.001	0.0010435	7384	125901	57.08	2875	47044	21.328	1288	21776	9.8726	17.4431	17.2961	44.8	46.2886
DN	SOX17_4	cluster_2	DP	3545	4309.846	4149	4477	97.1515	0.8226	-0.2818	0.001	0.0010909	3286	46015	72.668	464	6163	9.7328	264	3545	5.5984	8.0341	7.704	56.8966	57.5207
DP	MYBL2_2	cluster_5	Common_1_DN	5300	6443.546	6196	6670	132.9239	0.8226	-0.2818	0.001	0.0010435	2149	32599	60.53	738	10773	20.003	352	5300	9.8411	16.3797	16.2582	47.6965	49.1971
SP4	KLF4_1	cluster_2	DP	64081	77939.348	77353	78498	358.1684	0.8222	-0.2825	0.001	0.001	78036	752571	64.791	12881	121293	10.442	6644	64081	5.5169	8.514	8.5149	51.5798	52.8316
DN	TBX19_1	cluster_2	DP	8071	9818.231	9514	10098	189.2836	0.8221	-0.2827	0.001	0.0010435	4779	91001	71.181	770	14099	11.028	432	8071	6.3131	9.0395	8.8691	56.1039	57.2452
DP	FOXP3_2	cluster_5	Common_1_DN	3709	4512.116	4389	4649	76.1757	0.822	-0.2827	0.001	0.0010909	4108	24648	54.212	1426	8406	18.489	623	3709	8.1577	15.1655	15.0479	43.6886	44.1232
DP	TBX20_2	cluster_5	Common_1_DN	16293	19820.394	19325	20327	296.7594	0.822	-0.2827	0.001	0.0010435	5116	92297	53.087	2226	38036	21.877	921	16293	9.3713	18.0023	17.6528	41.3747	42.8357
SP4	FOXP3_2	cluster_2	DP	2736	3328.573	3215	3446	67.9795	0.822	-0.2827	0.001	0.0010909	4729	28374	62.407	909	5388	11.851	459	2736	6.0177	9.7061	9.6426	50.495	50.7795
DN	PKNOX1_1	cluster_2	DP	22065	26844.226	26512	27185	212.4699	0.822	-0.2828	0.001	0.0010435	15318	230880	74.405	2530	36762	11.847	1483	22065	7.1108	9.6814	9.5569	58.6166	60.0212
SP8	ELK4_1	cluster_2	DP	7091	8630.166	8472	8771	90.5761	0.8217	-0.2834	0.001	0.001	10505	84617	76.469	1427	11332	10.241	885	7091	6.4081	8.4246	8.3801	62.0182	62.575
DP	MAFG_1	cluster_5	Common_1_DN	21985	26764.703	26272	27289	342.0806	0.8214	-0.2838	0.001	0.0010435	5947	119184	52.184	2762	52068	22.798	1113	21985	9.626	18.7153	18.4463	40.2969	42.2236
SP8	SP1_known3	cluster_2	DP	48418	58943.641	58320	59537	374.5485	0.8214	-0.2838	0.001	0.001	39576	519194	65.135	7198	91416	11.469	3735	48418	6.0743	9.4375	9.3256	51.8894	52.9645
SP8	STAT_known3	cluster_2	DP	9952	12115.646	11719	12490	237.0444	0.8214	-0.2838	0.001	0.0010435	7066	141634	65.658	997	18856	8.7411	505	9952	4.6135	7.1469	7.0266	50.652	52.779
DN	MGA_2	cluster_2	DP	10689	13014.167	12737	13287	173.6406	0.8213	-0.2839	0.001	0.0010909	6706	114146	75.028	1087	17784	11.689	640	10689	7.0259	9.5437	9.3643	58.8776	60.1046
SP4	MAFF_1	cluster_2	DP	13895	16922.846	16528	17326	239.1971	0.8211	-0.2844	0.001	0.001	9927	139260	55.679	2253	30581	12.227	995	13895	5.5555	10.0232	9.9777	44.1633	45.4367
DN	CUX2_1	cluster_2	DP	510	621.461	559	683	38.4994	0.8209	-0.2847	0.001	0.0012632	444	7998	75.092	51	888	8.3372	30	510	4.7883	6.7568	6.3766	58.8235	57.4324
SP8	NFKB_known10	cluster_2	DP	11086	13506.362	13219	13807	181.0696	0.8208	-0.2849	0.001	0.001	10708	129395	64.216	1818	21243	10.542	929	11086	5.5017	8.6758	8.5676	51.1001	52.1866
SP8	FOXO6_3	cluster_2	DP	25600	31193.557	30603	31708	321.2027	0.8207	-0.2851	0.001	0.0010435	22108	289208	59.065	4217	53359	10.898	1972	25600	5.2283	8.9198	8.8518	46.7631	47.9769
SP4	SMAD4_1	cluster_2	DP	33426	40733.017	40127	41264	339.0291	0.8206	-0.2852	0.001	0.001	25162	357474	58.021	5161	70716	11.478	2372	33426	5.4253	9.4269	9.3506	45.9601	47.2679
SP4	KLF12_2	cluster_2	DP	43352	52834.079	52226	53491	379.3304	0.8205	-0.2854	0.001	0.001	39236	562601	65.133	5930	81999	9.4931	3048	43352	5.0189	7.7684	7.7056	51.3997	52.8689
SP8	ETS_known17	cluster_2	DP	19344	23579.428	23138	24037	280.9168	0.8204	-0.2856	0.001	0.001	12384	211645	59.804	2413	39722	11.224	1139	19344	5.466	9.1974	9.1398	47.2027	48.6985
SP4	ETV5_1	cluster_1	SP8	3809	4643.497	4519	4778	75.1915	0.8203	-0.2857	0.001	0.0010435	11580	105410	73.515	716	6355	4.4321	424	3809	2.6565	3.6615	3.6135	59.2179	59.9371
SP4	IRF_known14	cluster_2	DP	17950	21881.324	21382	22300	269.2683	0.8203	-0.2857	0.001	0.001	13966	293131	66.532	1777	34195	7.7612	890	17950	4.0741	6.3726	6.1235	50.0844	52.4931
SP8	CUX2_2	cluster_5	Common_1_DN	425	518.41	474	565	26.7192	0.8202	-0.286	0.001	0.0013333	381	3429	70.686	97	790	16.285	52	425	8.7611	13.6483	12.3943	53.6082	53.7975
SP4	MAF_known11	cluster_2	DP	17302	21100.628	20645	21566	284.2524	0.82	-0.2863	0.001	0.0010435	10831	174705	57.034	2417	37391	12.207	1079	17302	5.6484	9.9621	9.9036	44.6421	46.2732
DN	SOX4_2	cluster_2	DP	6589	8037.785	7830	8267	130.0907	0.8198	-0.2867	0.001	0.0010909	5733	86204	75.057	791	11178	9.7326	454	6589	5.737	7.9191	7.6435	57.3957	58.9461
DP	NFE2L1::MAFG_1	cluster_5	Common_1_DN	31397	38300.933	37619	38979	401.1389	0.8197	-0.2867	0.001	0.0010435	8197	173116	53.265	3722	73540	22.627	1512	31397	9.6603	18.4458	18.1364	40.6233	42.6938
SP4	MYBL2_2	cluster_2	DP	2769	3377.861	3216	3540	100.4236	0.8198	-0.2867	0.001	0.0010435	2320	35130	65.23	362	5258	9.7631	184	2769	5.1415	7.931	7.8822	50.8287	52.6626
DN	SOX18_2	cluster_2	DP	3698	4511.496	4344	4683	104.5786	0.8197	-0.2868	0.001	0.0010909	3405	47699	72.637	483	6451	9.8237	273	3698	5.6314	8.0176	7.7528	56.5217	57.3244
SP4	E2F2_3	cluster_2	DP	2523	3079.248	2930	3239	95.0615	0.8194	-0.2873	0.001	0.001	2073	35280	66.325	304	4770	8.9673	154	2523	4.7431	7.4288	7.1514	50.6579	52.8931
DN	MYC_known20	cluster_2	DP	17180	20970.628	20662	21267	189.2163	0.8192	-0.2876	0.001	0.001	11316	185626	73.632	1812	28806	11.426	1059	17180	6.8147	9.3584	9.2552	58.4437	59.6404
DN	FOXO3_4	cluster_2	DP	2263	2762.688	2642	2881	72.0678	0.8192	-0.2877	0.001	0.0010909	2345	30568	73.157	319	3951	9.4558	178	2263	5.4159	7.5906	7.4032	55.7994	57.2766
SP8	FOXO1_5	cluster_2	DP	25060	30591.766	30132	31016	268.9772	0.8192	-0.2877	0.001	0.0010435	25271	279379	58.574	4914	52812	11.072	2291	25060	5.254	9.0657	8.9699	46.6219	47.4513
DN	SMAD4_1	cluster_2	DP	42847	52308.784	51734	52778	314.8896	0.8191	-0.2879	0.001	0.001	32547	462220	75.022	5161	70716	11.478	3045	42847	6.9544	9.3557	9.2698	59.0002	60.5902
DN	SOX7_3	cluster_2	DP	5011	6118.454	5900	6320	129.604	0.819	-0.288	0.001	0.0010909	4157	66552	73.226	583	8749	9.6263	323	5011	5.5135	7.77	7.5295	55.4031	57.2751
SP4	SPZ1_1	cluster_2	DP	65526	80024.956	79356	80795	444.2439	0.8188	-0.2884	0.001	0.001	48085	704127	63.844	9016	127108	11.525	4530	65526	5.9413	9.4208	9.306	50.244	51.5514
DN	XBP1_4	cluster_2	DP	5984	7309.212	7111	7489	115.2957	0.8187	-0.2886	0.001	0.0010435	6101	79442	72.628	808	10192	9.3178	464	5984	5.4707	7.6053	7.5325	57.4257	58.7127
DN	SMAD3_1	cluster_2	DP	19426	23730.358	23454	23994	158.2175	0.8186	-0.2887	0.001	0.0010435	26078	216611	73.645	4021	32716	11.123	2373	19426	6.6046	9.0996	8.9682	59.0152	59.3777
DN	ATF4_2	cluster_2	DP	4488	5482.695	5318	5641	94.8674	0.8186	-0.2888	0.001	0.0010909	4963	59634	73.418	650	7608	9.3666	378	4488	5.5254	7.6164	7.5259	58.1538	58.9905
DN	TBX4_2	cluster_2	DP	15730	19217.344	18818	19645	235.0224	0.8185	-0.2889	0.001	0.0010909	8920	170122	76.154	1423	25905	11.596	841	15730	7.0415	9.4283	9.2463	59.1005	60.7219
SP4	ETV6_1	cluster_2	DP	34221	41814.371	41310	42375	333.1775	0.8184	-0.2891	0.001	0.001	27340	393690	65.257	4600	64454	10.684	2390	34221	5.6724	8.7418	8.6924	51.9565	53.0937
SP4	MYCN_1	cluster_2	DP	10614	12969.42	12678	13248	160.1992	0.8184	-0.2891	0.001	0.001	10339	114073	63.425	1894	20515	11.406	962	10614	5.9015	9.3046	9.3046	50.792	51.7378
DP	TBX5_3	cluster_5	Common_1_DN	24269	29657.954	29299	30023	231.0691	0.8183	-0.2893	0.001	0.001	14895	134213	54.181	6304	55260	22.308	2717	24269	9.7972	18.241	18.0825	43.0996	43.9178
DN	SOX11_2	cluster_2	DP	4532	5540.442	5379	5699	98.5805	0.818	-0.2898	0.001	0.0010909	4488	62863	74.087	589	7774	9.1621	337	4532	5.3412	7.5089	7.2093	57.2156	58.2969
SP4	GATA_known16	cluster_2	DP	7447	9104.855	8758	9417	196.3474	0.8179	-0.2899	0.001	0.0010435	5122	107965	62.558	772	15025	8.7059	362	7447	4.315	7.0676	6.8976	46.8912	49.5641
DN	ERG_2	cluster_2	DP	9468	11575.906	11335	11810	139.3583	0.8179	-0.29	0.001	0.001	8341	108611	75.068	1235	15627	10.801	737	9468	6.544	8.8359	8.7173	59.6761	60.5874
SP4	GMEB2_3	cluster_1	SP8	1016	1242.499	1160	1336	52.4797	0.8179	-0.2901	0.001	0.0010435	2133	30178	72.468	130	1755	4.2144	73	1016	2.4398	3.4224	3.3667	56.1538	57.8917
DN	SPIB_2	cluster_2	DP	24598	30085.423	29718	30422	215.9103	0.8176	-0.2905	0.001	0.0010435	23403	305650	75.803	3187	40326	10.001	1905	24598	6.1005	8.14	8.0478	59.7741	60.9979
DP	SPI1_known4	cluster_5	Common_1_DN	31235	38221.186	37661	38759	327.3654	0.8172	-0.2912	0.001	0.0010435	13368	174316	53.393	5779	72542	22.219	2419	31235	9.5672	18.0955	17.9186	41.8585	43.0578
SP8	HMGA1_1	cluster_2	DP	1248	1527.973	1464	1597	40.4752	0.8169	-0.2918	0.001	0.0010435	3543	21381	63.056	417	2455	7.2402	209	1248	3.6805	5.899	5.837	50.1199	50.835
DN	ATF3_known5	cluster_2	DP	19133	23428.468	23025	23796	223.1271	0.8167	-0.2922	0.001	0.001	14705	206515	71.894	2441	33101	11.523	1379	19133	6.6608	9.3778	9.2647	56.4932	57.8019
DP	FOXO1_5	cluster_5	Common_1_DN	47025	57585.464	56995	58112	351.1375	0.8166	-0.2923	0.001	0.0010435	23967	264888	55.536	9821	104850	21.983	4300	47025	9.8591	17.9413	17.7528	43.7837	44.8498
DP	E2F_known24	cluster_5	Common_1_DN	7680	9408.536	9118	9699	172.2712	0.8163	-0.2928	0.001	0.001	2757	47016	60.976	998	15843	20.547	461	7680	9.9603	16.7211	16.3349	46.1924	48.4757
SP4	POU2F2_known1	cluster_2	DP	4616	5655.025	5434	5895	144.4681	0.8163	-0.2928	0.001	0.0010435	3339	60392	62.523	558	9322	9.651	261	4616	4.7789	7.8167	7.6434	46.7742	49.5173
SP8	EWSR1::FLI1_1	cluster_2	DP	80363	98470.088	97369	99558	689.0878	0.8161	-0.2932	0.001	0.0010435	45443	904295	55.466	9825	183050	11.228	4172	80363	4.9292	9.1807	8.8868	42.4631	43.9022
DN	SOX15_4	cluster_2	DP	2665	3266.024	3142	3407	82.4426	0.816	-0.2933	0.001	0.0011429	2731	38267	69.358	374	4898	8.8775	198	2665	4.8303	7.2501	6.9642	52.9412	54.41
SP8	ETV6_1	cluster_2	DP	32630	39985.98	39409	40490	336.546	0.816	-0.2933	0.001	0.001	26153	376727	62.445	4600	64454	10.684	2282	32630	5.4087	8.7256	8.6614	49.6087	50.6253
SP4	TBX2_1	cluster_2	DP	13445	16480.309	16113	16922	254.4919	0.8158	-0.2937	0.001	0.0010435	8640	147190	60.352	1691	27780	11.391	797	13445	5.5128	9.2245	9.1345	47.1319	48.3981
SP8	CUX1_9	cluster_5	Common_1_DN	1174	1439.3	1326	1560	72.4069	0.8158	-0.2937	0.001	0.0012632	577	9969	67.331	144	2263	15.284	73	1174	7.9292	12.6516	11.7765	50.6944	51.878
SP8	SOX9_4	cluster_5	Common_1_DN	12178	14928.195	14590	15302	213.9292	0.8158	-0.2937	0.001	0.0010909	5012	75280	58.223	1850	26308	20.347	830	12178	9.4187	16.5603	16.1769	44.8649	46.2901
SP4	ELK4_1	cluster_2	DP	7050	8642.602	8502	8774	87.5158	0.8157	-0.2938	0.001	0.001	10509	84669	76.516	1427	11332	10.241	879	7050	6.3711	8.3643	8.3265	61.5978	62.2132
DN	SPIC_2	cluster_2	DP	23097	28315.144	27995	28715	221.7449	0.8157	-0.2939	0.001	0.0010435	21125	275317	74.226	3056	38712	10.437	1789	23097	6.227	8.4686	8.3892	58.5406	59.6637
DN	SPZ1_2	cluster_2	DP	17593	21568.912	21283	21874	171.267	0.8157	-0.2939	0.001	0.0010435	18565	186152	75.582	2953	28884	11.728	1765	17593	7.1431	9.5071	9.4509	59.7697	60.9092
SP4	EGR3_3	cluster_1	SP8	21608	26489.793	26017	26888	260.1542	0.8157	-0.2939	0.001	0.001	31018	457668	70.037	2726	38422	5.8797	1490	21608	3.3067	4.8037	4.7213	54.6588	56.2386
SP4	EWSR1::FLI1_1	cluster_2	DP	84749	103919.47	102772	105066	673.285	0.8155	-0.2942	0.001	0.0010435	47664	952228	58.406	9825	183050	11.228	4369	84749	5.1982	9.1662	8.9001	44.4682	46.2983
SP4	IKZF1_1	cluster_2	DP	7577	9291.456	9035	9568	154.6316	0.8155	-0.2942	0.001	0.0010435	8843	106411	60.935	1347	15540	8.8989	636	7577	4.3389	7.1921	7.1205	47.216	48.758
SP4	STAT_known3	cluster_2	DP	10150	12446.646	12145	12798	202.5627	0.8155	-0.2943	0.001	0.0010435	7269	145730	67.556	997	18856	8.7411	516	10150	4.7053	7.0986	6.9649	51.7553	53.829
SP4	POU2F2_known15	cluster_2	DP	2774	3402.378	3268	3547	85.0988	0.8154	-0.2945	0.001	0.0010435	3260	36005	62.683	523	5548	9.6588	254	2774	4.8294	7.7914	7.7045	48.566	50
DP	TBX1_4	cluster_5	Common_1_DN	26462	32460.794	31813	33050	351.3565	0.8152	-0.2948	0.001	0.0010909	7984	152301	52.016	3505	63358	21.639	1405	26462	9.0377	17.5977	17.3748	40.0856	41.7658
SP8	SOX8_2	cluster_5	Common_1_DN	11890	14591.002	14246	14882	198.7926	0.8149	-0.2953	0.001	0.0010909	4985	74903	58.581	1805	25656	20.065	812	11890	9.299	16.2889	15.8739	44.9861	46.3439
DN	TBX15_2	cluster_2	DP	14181	17405.827	17203	17617	122.3067	0.8147	-0.2956	0.001	0.0010909	20505	143570	74.719	3419	23567	12.265	2039	14181	7.3803	9.9439	9.8774	59.6373	60.1731
DN	FLI1_2	cluster_2	DP	9415	11562.792	11324	11768	134.7786	0.8143	-0.2964	0.001	0.001	8020	104443	74.293	1240	15749	11.203	731	9415	6.6971	9.1147	9.0145	58.9516	59.7816
SP4	EGR3_2	cluster_1	SP8	31423	38592.584	38023	39170	346.3405	0.8142	-0.2965	0.001	0.001	45033	687127	69.845	3895	56407	5.7337	2098	31423	3.1941	4.6588	4.5731	53.8639	55.7076
DP	SPIC_2	cluster_5	Common_1_DN	35010	43008.806	42459	43539	325.7396	0.814	-0.2969	0.001	0.0010435	14989	195470	52.699	6525	82309	22.191	2703	35010	9.4387	18.0332	17.9107	41.4253	42.5348
SP8	SOX4_2	cluster_5	Common_1_DN	10933	13433.08	13087	13741	196.1886	0.8139	-0.2971	0.001	0.0010909	4506	67750	58.989	1660	23480	20.444	751	10933	9.5193	16.6667	16.1373	45.241	46.563
DN	ETV7_1	cluster_2	DP	28720	35302.518	34930	35702	226.2601	0.8135	-0.2977	0.001	0.001	30877	278376	62.701	6344	56465	12.718	3189	28720	6.4688	10.3281	10.317	50.268	50.8634
DN	TBX4_1	cluster_2	DP	11178	13740.682	13578	13898	100.2582	0.8135	-0.2978	0.001	0.0010435	16022	112186	74.769	2687	18553	12.365	1605	11178	7.4498	10.0175	9.9638	59.732	60.249
SP8	ATF2_2	cluster_2	DP	3972	4883.38	4722	5039	101.2384	0.8134	-0.2979	0.001	0.0010435	5120	56438	65.757	694	7476	8.7104	363	3972	4.6279	7.0898	7.0378	52.3055	53.13
DP	GMEB2_2	cluster_5	Common_1_DN	5580	6860.478	6636	7060	126.6776	0.8134	-0.298	0.001	0.001	2591	34443	62.139	866	11140	20.098	416	5580	10.067	16.0556	16.2007	48.037	50.0898
SP8	IKZF3_1	cluster_2	DP	5711	7021.662	6788	7249	143.0668	0.8134	-0.298	0.001	0.0010435	6473	77854	57.71	1082	12441	9.222	481	5711	4.2333	7.4309	7.3355	44.4547	45.9047
SP8	SOX11_2	cluster_5	Common_1_DN	7575	9313.243	9061	9575	155.5991	0.8134	-0.298	0.001	0.0010909	3666	51361	60.532	1219	15941	18.787	564	7575	8.9275	15.3846	14.7485	46.2674	47.519
DP	IKZF3_1	cluster_5	Common_1_DN	13639	16771.926	16436	17091	205.7953	0.8132	-0.2983	0.001	0.0010435	6241	75044	55.627	2666	30716	22.768	1148	13639	10.11	18.3945	18.1747	43.0608	44.4036
DP	MAF_known1	cluster_5	Common_1_DN	68923	84781.141	83856	85599	521.5706	0.813	-0.2988	0.001	0.001	24308	367004	53.477	11086	160598	23.401	4618	68923	10.043	18.9979	18.7799	41.6561	42.9165
SP8	SPIC_2	cluster_2	DP	17291	21284.083	20887	21691	239.2613	0.8124	-0.2997	0.001	0.0010435	15787	205822	55.49	3056	38712	10.437	1337	17291	4.6617	8.469	8.4009	43.75	44.6657
DP	SPI1_known1	cluster_5	Common_1_DN	44791	55153.991	54649	55628	303.5415	0.8121	-0.3003	0.001	0.001	34618	243610	52.166	15325	106225	22.747	6387	44791	9.5914	18.4499	18.3864	41.677	42.1662
SP8	PATZ1_1	cluster_4	SP4	47633	58657.245	58005	59255	373.1066	0.8121	-0.3003	0.001	0.001	74077	975342	66.217	7074	90275	6.1288	3630	47633	3.2338	4.9003	4.8837	51.3147	52.7643
SP8	RUNX1_2	cluster_5	Common_1_DN	9868	12152.094	11844	12482	196.7974	0.8121	-0.3003	0.001	0.0010435	4117	64095	56.816	1533	22215	19.692	656	9868	8.7474	15.9339	15.3959	42.7919	44.4204
SP4	SP8_1	cluster_2	DP	66763	82272.997	81565	83044	444.1144	0.8115	-0.3014	0.001	0.001	64724	792047	63.386	11105	131511	10.525	5504	66763	5.343	8.5038	8.4292	49.5633	50.7661
SP8	NFKB_known11	cluster_2	DP	12376	15251.917	14954	15541	177.6363	0.8115	-0.3014	0.001	0.001	12335	149870	65.352	1997	23563	10.275	1032	12376	5.3967	8.3664	8.2578	51.6775	52.523
DP	TBX15_2	cluster_5	Common_1_DN	18973	23386.286	23054	23699	204.9237	0.8113	-0.3017	0.001	0.0010909	14654	102598	53.396	6406	44116	22.96	2727	18973	9.8743	18.6093	18.4926	42.5695	43.0071
SP4	HMGA1_2	cluster_2	DP	5261	6485.083	6271	6705	126.0621	0.8113	-0.3017	0.001	0.0010435	5885	82554	67.162	772	10035	8.164	387	5261	4.2801	6.576	6.3728	50.1295	52.4265
DP	NFATC1_3	cluster_5	Common_1_DN	6297	7765.661	7511	8007	149.8414	0.8109	-0.3024	0.001	0.0010909	2771	36045	53.14	1232	15103	22.266	500	6297	9.2835	18.044	17.4698	40.5844	41.6937
DN	ERG_4	cluster_2	DP	9217	11368.046	11150	11570	127.1306	0.8108	-0.3026	0.001	0.001	8373	109062	76.033	1199	15137	10.553	716	9217	6.4256	8.5513	8.4512	59.7164	60.8905
SP8	GMEB2_2	cluster_2	DP	2623	3236.378	3086	3375	91.1469	0.8105	-0.3031	0.001	0.001	2724	36104	65.136	402	5050	9.1108	200	2623	4.7322	7.3421	7.2651	49.7512	51.9406
SP8	XBP1_4	cluster_2	DP	5068	6254.526	6047	6444	121.8782	0.8103	-0.3034	0.001	0.0010435	5176	67397	61.616	808	10192	9.3178	393	5068	4.6333	7.5927	7.5196	48.6386	49.7253
SP8	SMAD4_1	cluster_2	DP	33168	40936.65	40393	41532	360.0436	0.8102	-0.3036	0.001	0.001	25393	360702	58.545	5161	70716	11.478	2356	33168	5.3834	9.2781	9.1954	45.6501	46.9031
SP4	SP1_known3	cluster_2	DP	47183	58255.052	57607	59003	415.6879	0.8099	-0.3041	0.001	0.001	39133	513297	64.396	7198	91416	11.469	3631	47183	5.9193	9.2786	9.1921	50.4446	51.6135
DN	IKZF1_2	cluster_2	DP	18306	22607.1	22390	22834	130.6541	0.8098	-0.3044	0.001	0.0010435	27213	217711	79.439	3711	28886	10.54	2317	18306	6.6796	8.5143	8.4084	62.436	63.3733
DP	E2F2_3	cluster_5	Common_1_DN	5441	6720.023	6521	6927	127.1122	0.8097	-0.3045	0.001	0.001	1923	32730	61.531	696	11157	20.975	324	5441	10.229	16.8487	16.6239	46.5517	48.7676
DN	GMEB2_2	cluster_2	DP	3086	3812.196	3676	3918	74.1767	0.8096	-0.3048	0.001	0.001	3215	42707	77.048	402	5050	9.1108	235	3086	5.5675	7.3095	7.226	58.4577	61.1089
SP4	GMEB2_2	cluster_1	SP8	1994	2464.041	2321	2598	78.9236	0.8093	-0.3052	0.001	0.001	2773	36777	66.35	297	3755	6.7744	151	1994	3.5974	5.4454	5.4219	50.8418	53.1025
SP8	POU2F2_known10	cluster_5	Common_1_DN	7043	8702.548	8493	8921	131.6478	0.8093	-0.3052	0.001	0.0010435	4821	43499	59.761	1684	14748	20.262	797	7043	9.676	16.5318	16.1912	47.3278	47.7556
SP8	SPIC_1	cluster_2	DP	16110	19909.41	19553	20335	249.7091	0.8092	-0.3055	0.001	0.001	14831	193520	54.959	2896	36649	10.408	1250	16110	4.5751	8.4283	8.3247	43.163	43.9575
SP4	SPDEF_4	cluster_2	DP	9004	11128.409	10814	11420	174.3328	0.8091	-0.3056	0.001	0.0010435	7657	115318	63.966	1256	17937	9.9495	613	9004	4.9944	8.0057	7.808	48.8057	50.1979
SP4	NFKB_known10	cluster_2	DP	10669	13188.104	12867	13508	187.6441	0.809	-0.3058	0.001	0.001	10445	126213	62.637	1818	21243	10.542	893	10669	5.2948	8.5495	8.4532	49.1199	50.2236
DP	SPZ1_2	cluster_5	Common_1_DN	25079	31002.866	30566	31480	273.7364	0.8089	-0.3059	0.001	0.0010435	13790	138253	56.134	5732	55792	22.653	2527	25079	10.183	18.3249	18.1399	44.0858	44.9509
DP	PKNOX2_3	cluster_5	Common_1_DN	19442	24035.643	23638	24515	260.1855	0.8089	-0.306	0.001	0.0010435	9600	106895	49.891	4498	48655	22.709	1756	19442	9.0741	18.2917	18.1879	39.0396	39.9589
SP4	NFATC2_1	cluster_2	DP	2352	2907.954	2815	2995	56.3628	0.8089	-0.306	0.001	0.0010909	5908	35448	63.511	798	4659	8.3474	396	2352	4.214	6.7028	6.6351	49.6241	50.4829
DN	ETV5_1	cluster_2	DP	3078	3806.396	3601	4012	116.1106	0.8087	-0.3063	0.001	0.0010435	4257	38576	26.904	1599	14322	9.9884	342	3078	2.1467	8.0338	7.9791	21.3884	21.4914
SP4	SP100_1	cluster_5	Common_1_DN	1735	2145.959	2059	2237	51.758	0.8086	-0.3065	0.001	0.0013333	962	12518	74.454	242	2948	17.534	137	1735	10.319	14.2412	13.86	56.6116	58.8535
SP4	SPIC_2	cluster_2	DP	18845	23311.968	22924	23738	250.5261	0.8084	-0.3069	0.001	0.0010435	17268	225108	60.689	3056	38712	10.437	1454	18845	5.0806	8.4202	8.3715	47.5785	48.68
DN	XBP1_2	cluster_2	DP	5218	6456.091	6248	6653	124.2138	0.8083	-0.3071	0.001	0.0010435	3231	74429	77.172	396	8574	8.8899	232	5218	5.4103	7.1804	7.0107	58.5859	60.8584
SP8	KLF13_1	cluster_2	DP	26592	32904.35	32387	33412	307.619	0.8082	-0.3073	0.001	0.001	20972	360883	65.363	3116	51120	9.2588	1571	26592	4.8163	7.4909	7.3686	50.4172	52.0188
DP	ATF7_1	cluster_5	Common_1_DN	6122	7577.215	7283	7826	159.4385	0.808	-0.3076	0.001	0.001	3062	39860	54.566	1102	13946	19.091	474	6122	8.3807	15.4801	15.3588	43.0127	43.8979
SP8	IKZF2_3	cluster_5	Common_1_DN	8948	11077.901	10818	11324	149.2537	0.8078	-0.308	0.001	0.0010909	5533	55534	62.869	1899	18181	20.582	915	8948	10.13	16.5371	16.1127	48.1833	49.2162
DP	CTCF_known2	cluster_2	DP	15102	18697.388	18081	19375	390.9976	0.8077	-0.3081	0.001	0.0010435	14645	235553	28.191	4231	66340	7.9395	947	15102	1.8074	6.4664	6.4113	22.3824	22.7645
DP	MAF_known11	cluster_5	Common_1_DN	30842	38190.909	37544	38739	350.3267	0.8076	-0.3083	0.001	0.0010435	10294	166060	54.212	4559	70796	23.112	1905	30842	10.069	18.5059	18.5728	41.7855	43.5646
SP4	NFATC2_1	cluster_5	Common_1_DN	5886	7288.869	7144	7428	84.7345	0.8076	-0.3084	0.001	0.0010909	5908	35448	63.511	1994	11648	20.869	994	5886	10.546	16.8246	16.6046	49.8495	50.5323
SP8	SOX3_1	cluster_5	Common_1_DN	4400	5449.711	5211	5662	137.8336	0.8074	-0.3086	0.001	0.0010909	2117	33889	61.352	651	9351	16.929	294	4400	7.9657	13.8876	12.9836	45.1613	47.0538
SP8	SP8_1	cluster_4	SP4	44179	54727.005	54194	55333	339.5326	0.8073	-0.3089	0.001	0.001	67362	823083	65.87	7107	84496	6.7621	3600	44179	3.5356	5.3443	5.3675	50.6543	52.2853
SP4	KLF14_1	cluster_1	SP8	33287	41237.036	40776	41716	289.0451	0.8072	-0.309	0.001	0.001	49055	664031	67.164	4810	62544	6.326	2497	33287	3.3668	5.0902	5.0129	51.9127	53.2217
SP8	HIF1A::ARNT_1	cluster_4	SP4	3413	4228.388	4108	4345	73.7199	0.8072	-0.309	0.001	0.001	10355	74439	66.239	915	6438	5.7288	481	3413	3.037	4.6451	4.585	52.5683	53.0134
DN	FLI1_4	cluster_2	DP	9478	11743.952	11525	11955	131.5497	0.8071	-0.3092	0.001	0.001	8592	111995	76.37	1231	15560	10.611	737	9478	6.4631	8.5777	8.4629	59.87	60.9126
SP4	SOX3_1	cluster_5	Common_1_DN	4602	5702.209	5482	5958	140.1176	0.8071	-0.3092	0.001	0.0010909	2214	35441	64.162	651	9351	16.929	306	4602	8.3314	13.8211	12.985	47.0046	49.214

DP	ATF4_3	cluster_5	Common_1_DN	7314	9063.698	8780	9353	168.3373	0.807	-0.3094	0.001	0.0010909	3413	44407	51.027	1433	17948	20.624	568	7314	8.4044	16.6423	16.4704	39.6371	40.7511
DP	TBX4_1	cluster_5	Common_1_DN	14565	18049.728	17764	18333	170.1027	0.8069	-0.3095	0.001	0.0010435	11484	80419	53.597	4930	33931	22.614	2095	14565	9.7072	18.2428	18.1114	42.4949	42.9253
DN	SMAD_2	cluster_2	DP	26192	32464.528	32133	32805	211.0735	0.8068	-0.3097	0.001	0.0010435	28215	291809	73.323	4479	45031	11.315	2575	26192	6.5812	9.1264	8.9757	57.4905	58.1644
SP4	TBX20_2	cluster_2	DP	8896	11028.258	10669	11391	218.2	0.8067	-0.3099	0.001	0.0010435	5606	101105	58.153	1123	19395	11.155	496	8896	5.1167	8.8477	8.7988	44.1674	45.8675
SP8	CTCF_known1	cluster_2	DP	84323	104552.867	103517	105568	628.3069	0.8065	-0.3102	0.001	0.0010435	57414	1061210	61.215	9691	172310	9.9396	4600	84323	4.8641	8.012	7.9459	47.4667	48.9368
SP8	PKNOX2_3	cluster_2	DP	10948	13574.863	13229	13922	201.3196	0.8065	-0.3102	0.001	0.0010435	9661	107482	50.165	2518	27407	12.792	987	10948	5.1098	10.2163	10.1859	39.1978	39.946
SP8	PKNOX1_2	cluster_2	DP	11087	13747.366	13438	14095	197.7466	0.8065	-0.3103	0.001	0.0010435	9433	104620	49.524	2578	28014	13.261	1001	11087	5.2482	10.6117	10.5974	38.8285	39.5766
SP8	HMGA1_2	cluster_5	Common_1_DN	12169	15090.13	14810	15403	185.3411	0.8064	-0.3104	0.001	0.0010435	5565	78050	63.498	1898	24720	20.111	902	12169	9.9001	16.2084	15.5913	47.5237	49.2273
SP8	SOX21_3	cluster_5	Common_1_DN	8162	10123.03	9810	10391	170.1629	0.8063	-0.3106	0.001	0.0010909	3510	52680	59.82	1244	17494	19.865	559	8162	9.2683	15.9259	15.4935	44.9357	46.656
SP4	ETV5_1	cluster_2	DP	8459	10492.032	10293	10675	111.3643	0.8062	-0.3107	0.001	0.0010435	11580	105410	73.515	1599	14322	9.9884	936	8459	5.8995	8.0829	8.0249	58.5366	59.063
DN	SOX1_2	cluster_2	DP	4718	5853.054	5678	6026	106.5585	0.8061	-0.311	0.001	0.0010909	4151	58133	72.545	621	8368	10.443	345	4718	5.8876	8.3113	8.1159	55.5556	56.3815
SP4	FOXO1_5	cluster_2	DP	24448	30328.679	29834	30768	289.0748	0.8061	-0.311	0.001	0.0010435	25037	276688	58.01	4914	52812	11.072	2231	24448	5.1257	8.9108	8.8359	45.4009	46.2925
SP4	CBX5_1	cluster_5	Common_1_DN	7320	9080.888	8829	9306	142.1026	0.8061	-0.311	0.001	0.0010435	4252	46829	61.603	1439	15113	19.881	678	7320	9.6293	15.9454	15.6313	47.1161	48.4351
SP8	E2F7_1	cluster_2	DP	3776	4686.061	4507	4879	111.7129	0.8058	-0.3114	0.001	0.0010435	4510	58679	64.962	594	7377	8.1669	298	3776	4.1803	6.6075	6.435	50.1684	51.1861
DN	SOX21_3	cluster_2	DP	4891	6070.031	5897	6249	109.2113	0.8058	-0.3115	0.001	0.0010909	4326	64953	73.757	612	8556	9.7157	339	4891	5.5539	7.8363	7.5301	55.3922	57.1646
SP8	SPZ1_1	cluster_4	SP4	32380	40183.419	39636	40715	322.1366	0.8058	-0.3115	0.001	0.001	49464	724618	65.702	4489	62429	5.6605	2267	32380	2.9359	4.5831	4.4686	50.5012	51.8669
DP	MTF1_3	cluster_5	Common_1_DN	28978	35968.806	35528	36439	276.2025	0.8056	-0.3118	0.001	0.001	12915	169509	62.611	4568	58056	21.444	2226	28978	10.704	17.2358	17.0953	48.7303	49.9139
SP8	POU2F2_known1	cluster_2	DP	4338	5384.794	5122	5604	140.4085	0.8056	-0.3118	0.001	0.0010435	3160	57168	59.186	558	9322	9.651	242	4338	4.4911	7.6582	7.5882	43.3692	46.5351
SP4	FOXO6_3	cluster_2	DP	24776	30755.283	30316	31167	270.2612	0.8056	-0.3119	0.001	0.0010435	21804	285200	58.247	4217	53359	10.898	1904	24776	5.06	8.7323	8.6872	45.1506	46.4327
SP4	REL_2	cluster_2	DP	8304	10309.359	10102	10531	129.0897	0.8055	-0.312	0.001	0.0010435	12661	114508	63.571	1867	16362	9.0836	926	8304	4.6101	7.3138	7.2519	49.5983	50.7517
SP8	SPI1_known4	cluster_2	DP	14409	17887.998	17515	18267	227.9643	0.8055	-0.312	0.001	0.0010435	14322	186719	57.192	2515	31724	9.717	1120	14409	4.4134	7.8201	7.7169	44.5328	45.4199
DP	FOXO6_3	cluster_5	Common_1_DN	47920	59497.717	58778	60155	409.499	0.8054	-0.3122	0.001	0.0010435	21070	275513	56.268	8509	107163	21.886	3713	47920	9.7867	17.6222	17.393	43.6361	44.7169
SP8	SOX30_1	cluster_5	Common_1_DN	8488	10541.52	10259	10839	171.8407	0.8052	-0.3126	0.001	0.0010435	3553	53360	60.089	1285	18059	20.336	583	8488	9.5583	16.4087	15.907	45.3696	47.0015
DN	FOXO1_4	cluster_2	DP	2786	3461.148	3324	3598	81.1984	0.805	-0.313	0.001	0.0010909	2797	36450	73.199	405	4964	9.9687	221	2786	5.5948	7.9013	7.6433	54.5679	56.1241
DP	ATF4_2	cluster_5	Common_1_DN	6968	8658.794	8415	8880	141.6202	0.8048	-0.3134	0.001	0.0010909	3457	41526	51.125	1471	17153	21.118	587	6968	8.5786	16.98	16.7798	39.9048	40.6226
SP8	SPIB_2	cluster_2	DP	17926	22278.47	21858	22741	269.1703	0.8046	-0.3136	0.001	0.0010435	17242	225293	55.874	3187	40326	10.001	1385	17926	4.4458	8.0327	7.9567	43.4578	44.4527
SP8	E2F2_2	cluster_2	DP	3902	4850.456	4653	5045	121.9176	0.8045	-0.3138	0.001	0.0010909	3179	54200	66.243	465	7560	9.2398	232	3902	4.769	7.2979	7.1993	49.8925	51.6138
SP4	MAF_known10	cluster_2	DP	8892	11053.579	10760	11349	173.4449	0.8045	-0.3139	0.001	0.0010909	8761	96521	59.023	1769	18966	11.598	815	8892	5.4375	9.3026	9.2125	46.0712	46.8839
DP	XBP1_2	cluster_5	Common_1_DN	9543	11866.188	11451	12234	243.1912	0.8042	-0.3143	0.001	0.0010435	2416	55665	57.716	971	20992	21.766	421	9543	9.8947	17.4255	17.1436	43.3574	45.4602
DP	ELK4_2	cluster_1	SP8	3584	4456.788	4212	4685	141.6213	0.8042	-0.3144	0.001	0.0011429	7680	69469	25.728	1952	17453	6.4637	398	3584	1.3273	5.1823	5.1591	20.3893	20.5352
SP8	CTCF_known1	cluster_4	SP4	42532	52890.724	52164	53681	462.4681	0.8042	-0.3145	0.001	0.0010435	57414	1061210	61.215	4997	87610	5.0537	2348	42532	2.4534	4.0896	4.0079	46.9882	48.547
DN	TBX2_2	cluster_2	DP	10939	13604.494	13360	13856	146.1223	0.8041	-0.3146	0.001	0.0010909	11786	118013	74.082	1906	18653	11.709	1109	10939	6.8669	9.4095	9.2693	58.1847	58.6447
SP8	FOXO3_1	cluster_5	Common_1_DN	9639	11987.725	11712	12297	170.8053	0.8041	-0.3146	0.001	0.0010435	5381	70700	64.693	1584	19294	17.655	757	9639	8.82	14.068	13.6337	47.7904	49.9585
SP8	FOXP1_1	cluster_5	Common_1_DN	30103	37440.409	36755	38156	436.5876	0.804	-0.3147	0.001	0.0010435	10498	244896	63.1	3601	67880	17.49	1466	30103	7.7564	13.9646	12.2922	40.7109	44.3474
DN	TBX5_5	cluster_2	DP	12967	16129.482	15784	16437	202.4661	0.8039	-0.3148	0.001	0.0010435	7574	144355	74.742	1223	22231	11.51	695	12967	6.7138	9.1761	8.9827	56.8275	58.3285
DN	MAF_known12	cluster_2	DP	15117	18805.715	18470	19165	206.557	0.8039	-0.315	0.001	0.0010435	9989	170271	77.196	1524	24862	11.272	899	15117	6.8536	8.9999	8.8782	58.9895	60.8036
DN	TBX20_5	cluster_2	DP	22476	27960.193	27536	28358	246.3685	0.8039	-0.315	0.001	0.0010909	16570	253871	75.218	2625	38253	11.334	1490	22476	6.6593	8.9922	8.8533	56.7619	58.7562
SP8	ATF3_known4	cluster_4	SP4	5259	6544.56	6362	6748	117.9626	0.8036	-0.3155	0.001	0.001	8603	121435	69.067	717	9606	5.4635	380	5259	2.9911	4.4171	4.3307	52.9986	54.747
SP8	CUX1_8	cluster_5	Common_1_DN	1235	1537.636	1438	1637	61.6579	0.8033	-0.316	0.001	0.0011429	544	8813	66.353	164	2468	18.582	79	1235	9.2983	14.5221	14.0134	48.1707	50.0405
DP	RBPJ_2	cluster_5	Common_1_DN	17733	22079.155	21723	22409	206.1567	0.8032	-0.3162	0.001	0.0010909	9585	95962	53.327	4334	41855	23.259	1793	17733	9.8543	18.7063	18.4792	41.3706	42.3677
DN	PBX3_known1	cluster_2	DP	7577	9434.729	9223	9632	118.8522	0.8031	-0.3163	0.001	0.0010435	8906	98882	73.528	1236	13124	9.7589	695	7577	5.6342	7.8037	7.6627	56.2298	57.7339
SP8	POU2F2_known12	cluster_2	DP	3158	3933.24	3777	4091	98.1742	0.803	-0.3166	0.001	0.0010435	4381	43877	60.114	697	6675	9.1451	318	3158	4.3266	7.2586	7.1974	45.6241	47.3109
DP	RUNX_2	cluster_5	Common_1_DN	49889	62136.132	61383	62925	455.712	0.8029	-0.3167	0.001	0.0010435	17458	245690	47.274	9707	132568	25.508	3577	49889	9.5994	20.4892	20.3057	36.8497	37.6328
SP8	KLF12_2	cluster_4	SP4	29645	36923.598	36348	37425	300.6543	0.8029	-0.3167	0.001	0.001	40686	583267	67.525	4070	55774	6.457	2118	29645	3.432	5.2057	5.0826	52.0393	53.152
DN	POU2F2_known16	cluster_2	DP	3073	3827.953	3671	3964	91.928	0.8028	-0.3168	0.001	0.0010909	3024	39849	67.778	479	5889	10.016	244	3073	5.2268	8.0688	7.7116	50.9395	52.182
SP4	SPI1_known4	cluster_2	DP	15582	19408.479	19006	19787	233.3329	0.8029	-0.3168	0.001	0.0010435	15528	202516	62.03	2515	31724	9.717	1207	15582	4.7727	7.7731	7.6942	47.992	49.1174
DP	PKNOX1_2	cluster_5	Common_1_DN	19569	24379.302	23918	24857	275.5056	0.8027	-0.3171	0.001	0.0010435	9569	106189	50.266	4531	48957	23.175	1772	19569	9.2633	18.5181	18.4285	39.1084	39.9718
SP8	HMGA1_1	cluster_5	Common_1_DN	3533	4404.068	4294	4505	65.5571	0.8023	-0.3179	0.001	0.0010435	3543	21381	63.056	1205	7071	20.853	596	3533	10.419	16.8219	16.524	49.4606	49.9646
DP	E2F8_1	cluster_5	Common_1_DN	12797	15952.943	15640	16297	195.2206	0.8022	-0.318	0.001	0.001	7146	78795	61.175	2499	26482	20.56	1181	12797	9.9353	16.5267	16.2409	47.2589	48.3234
SP8	HESX1_1	cluster_5	Common_1_DN	2200	2742.712	2633	2845	64.5301	0.8022	-0.318	0.001	0.0012632	1301	11720	59.084	556	4768	24.037	249	2200	11.091	19.1391	18.7713	44.7842	46.1409
DP	RUNX_1	cluster_5	Common_1_DN	61296	76430.426	75453	77311	544.2328	0.802	-0.3183	0.001	0.0010435	21137	297645	46.5	12058	165555	25.864	4386	61296	9.5761	20.7503	20.5937	36.3742	37.0246
SP8	EGR1_known12	cluster_4	SP4	44463	55444.278	54798	56028	389.7111	0.8019	-0.3184	0.001	0.001	52057	864606	69.047	5218	82372	6.5782	2714	44463	3.5508	5.2135	5.1426	52.0123	53.9783
SP4	SPIC_1	cluster_2	DP	17411	21712.412	21322	22113	236.9741	0.8019	-0.3185	0.001	0.001	16203	211420	60.042	2896	36649	10.408	1345	17411	4.9446	8.3009	8.2353	46.4434	47.5074
SP8	PKNOX2_2	cluster_2	DP	12734	15882.111	15478	16277	231.6405	0.8018	-0.3187	0.001	0.0010435	10799	119886	49.272	2996	32542	13.374	1150	12734	5.2335	10.6491	10.6218	38.3845	39.131
SP4	SOX12_1	cluster_2	DP	3119	3891.796	3726	4047	95.7334	0.8015	-0.3193	0.001	0.0010435	3166	41308	65.459	501	6174	9.7837	245	3119	4.9426	7.7385	7.5506	48.9022	50.5183
SP4	POU2F2_known13	cluster_5	Common_1_DN	8121	10135.185	9812	10440	195.0908	0.8013	-0.3196	0.001	0.0010909	2728	57442	64.703	863	16530	18.619	410	8121	9.1475	15.0293	14.1377	47.5087	49.1289
SP8	RREB1_1	cluster_5	Common_1_DN	134191	167490.638	166399	168488	607.3915	0.8012	-0.3198	0.001	0.001	48842	818149	64.867	17321	268951	21.324	8206	134191	10.639	16.8011	16.4018	47.376	49.8942
SP8	SOX7_3	cluster_5	Common_1_DN	7821	9762.604	9478	10073	178.9237	0.8011	-0.3199	0.001	0.0010909	3353	53689	59.073	1166	17179	18.902	514	7821	8.6053	15.3296	14.5672	44.0823	45.5265
SP8	SOX11_1	cluster_5	Common_1_DN	26690	33327.85	32772	33832	302.9356	0.8008	-0.3204	0.001	0.0010435	10921	177105	60.65	3781	56921	19.493	1696	26690	9.14	15.5297	15.0702	44.8559	46.8895
SP4	E2F7_1	cluster_2	DP	3801	4747.084	4585	4919	103.2657	0.8007	-0.3206	0.001	0.0010435	4567	59426	65.789	594	7377	8.1669	302	3801	4.208	6.6127	6.3962	50.8418	51.525
SP8	MAF_known11	cluster_2	DP	16075	20076.964	19632	20510	267.8655	0.8007	-0.3207	0.001	0.0010435	10315	166406	54.325	2417	37391	12.207	1005	16075	5.2478	9.7431	9.6601	41.5805	42.9916
SP4	REL_1	cluster_2	DP	8064	10072.3	9854	10316	138.3627	0.8006	-0.3208	0.001	0.001	12552	113599	63.434	1831	16059	8.9675	898	8064	4.503	7.1542	7.0987	49.0442	50.2148
DP	SPIB_2	cluster_5	Common_1_DN	37907	47356.432	46772	47944	338.8652	0.8005	-0.3211	0.001	0.0010435	16381	214053	53.087	7171	90414	22.423	2932	37907	9.4012	17.8988	17.7092	40.8869	41.926
SP8	FLI1_4	cluster_2	DP	7802	9748	9488	10040	169.3134	0.8004	-0.3212	0.001	0.001	7101	92567	63.122	1231	15560	10.611	602	7802	5.3203	8.4777	8.4285	48.9033	50.1414
SP4	NFATC1_1	cluster_2	DP	6619	8270.255	7978	8576	181.808	0.8004	-0.3213	0.001	0.0010909	5359	103439	64.397	757	13426	8.3585	351	6619	4.1207	6.5497	6.3989	46.3672	49.2999
DN	TBX21_6	cluster_2	DP	15968	19952.471	19590	20310	218.7839	0.8003	-0.3214	0.001	0.0010909	10503	189588	74.498	1600	27516	10.812	902	15968	6.2746	8.588	8.4225	56.375	58.0317
SP8	SPZ1_2	cluster_2	DP	12944	16175.477	15859	16468	181.9966	0.8002	-0.3215	0.001	0.0010435	13891	139218	56.526	2953	28884	11.728	1297	12944	5.2556	9.337	9.2976	43.9214	44.8137
SP8	TBX2_1	cluster_2	DP	13058	16318.178	15882	16693	248.0293	0.8002	-0.3215	0.001	0.0010435	8536	145462	59.643	1691	27780	11.391	774	13058	5.3541	9.0675	8.9769	45.7717	47.005
SP4	SPIB_2	cluster_2	DP	19383	24223.201	23808	24632	255.4951	0.8002	-0.3216	0.001	0.0010435	18724	244697	60.687	3187	40326	10.001	1495	19383	4.8071	7.9844	7.9212	46.9093	48.0658
SP4	TBX1_2	cluster_2	DP	7468	9335.895	9011	9656	195.2026	0.7999	-0.322	0.001	0.0010909	4990	89894	57.636	979	16664	10.684	424	7468	4.7882	8.497	8.3076	43.3095	44.8152
SP4	FOXO4_3	cluster_2	DP	2144	2680.58	2532	2847	95.251	0.7999	-0.3221	0.001	0.0010909	2238	29139	62.034	359	4443	9.4586	168	2144	4.5643	7.5067	7.3578	46.7967	48.2557
SP8	SOX4_1	cluster_5	Common_1_DN	28975	36229.933	35716	36744	332.8518	0.7998	-0.3224	0.001	0.0010435	11786	191082	60.682	4095	61800	19.626	1842	28975	9.2016	15.6287	15.1636	44.9817	46.8851
DN	FOXM1_1	cluster_2	DP	4341	5429.09	5309	5543	72.8815	0.7996	-0.3226	0.001	0.0010909	6570	52629	74.145	953	7417	10.449	552	4341	6.1157	8.4018	8.2483	57.9224	58.5277
DP	ATF3_known5	cluster_5	Common_1_DN	27150	33954.646	33430	34565	344.1152	0.7996	-0.3226	0.001	0.001	11264	158203	55.075	4636	62555	21.777	1952	27150	9.4518	17.3295	17.1615	42.1053	43.4018
SP8	POU2F2_known5	cluster_2	DP	3494	4370.208	4189	4551	107.3522	0.7996	-0.3227	0.001	0.0010909	3679	47888	58.57	612	7622	9.3222	273	3494	4.2734	7.4205	7.2962	44.6078	45.841
SP4	POU2F2_known10	cluster_2	DP	3645	4559.326	4413	4720	91.5755	0.7995	-0.3228	0.001	0.0010435	5130	46258	63.552	838	7279	10	408	3645	5.0077	7.9532	7.8797	48.6874	50.0756
SP4	TBX21_4	cluster_2	DP	10821	13535.665	13193	13874	202.672	0.7995	-0.3229	0.001	0.0010909	7892	118588	58.407	1649	23664	11.655	734	10821	5.3296	9.3006	9.1249	44.5118	45.7277
SP8	POU2F2_known10	cluster_2	DP	3439	4301.883	4167	4443	87.4546	0.7995	-0.3229	0.001	0.0010435	4821	43499	59.761	838	7279	10	383	3439	4.7247	7.9444	7.9059	45.7041	47.2455
DN	CUX2_1	cluster_1	SP8	806	1008.673	924	1087	50.6589	0.7993	-0.3232	0.001	0.0012632	444	7998	75.092	91	1512	14.196	48	806	7.5674	10.8108	10.0775	52.7473	53.3069
DN	SPI1_known1	cluster_2	DP	10250	12826.583	12521	13161	195.2872	0.7991	-0.3235	0.001	0.001	15535	109231	23.39	7958	55408	11.865	1460	10250	2.1949	9.3981	9.3838	18.3463	18.4991
SP8	SOX17_5	cluster_5	Common_1_DN	4943	6185.803	5934	6402	132.9959	0.7991	-0.3235	0.001	0.0011429	2277	31990	58.026	850	11148	20.221	367	4943	8.9661	16.1177	15.4517	43.1765	44.3398
SP8	SPDEF_4	cluster_5	Common_1_DN	16519	20671.045	20267	21067	248.3708	0.7991	-0.3235	0.001	0.0010435	7014	105694	58.627	2551	36276	20.122	1120	16519	9.1629	15.9681	15.6291	43.9044	45.537
SP4	EGR1_known9	cluster_1	SP8	17364	21731.578	21402	22077	207.7046	0.799	-0.3237	0.001	0.001	28464	386336	71.535	2355	30796	5.7023	1294	17364	3.2152	4.5461	4.4945	54.9469	56.3839
SP8	GATA_known17	cluster_2	DP	3608	4516.339	4314	4744	132.0316	0.7989	-0.3239	0.001	0.0010909	3680	58991	55.43	554	8351	7.8469	229	3608	3.3902	6.2228	6.1162	41.3357	43.2044
SP4	POU2F3_2	cluster_2	DP	2167	2712.924	2610	2818	64.1065	0.7988	-0.324	0.001	0.0010909	3575	28645	62.59	567	4389	9.5901	273	2167	4.735	7.6364	7.565	48.1481	49.3734
SP4	SPZ1_2	cluster_2	DP	13224	16555.396	16179	16879	215.9257	0.7988	-0.3241	0.001	0.0010435	14241	142725	57.95	2953	28884	11.728	1325	13224	5.3692	9.3041	9.2654	44.8696	45.7831
SP4	SOX_1	cluster_2	DP	6643	8320.535	8093	8547	137.1701	0.7984	-0.3248	0.001	0.0010435	7156	85938	62.408	1171	13573	9.8566	559	6643	4.8241	7.8116	7.73	47.737	48.9428
SP8	TCF7_1	cluster_1	SP8	33104	20646.885	20243	21058	250.137	1.6033	0.6811	0.001	0.0010435	10694	171757	56.165	2397	37254	12.182	2112	33104	10.825	19.7494	19.2737	88.1101	88.8603
SP4	POU2F2_known5	cluster_2	DP	3654	4577.76	4400	4757	112.9531	0.7983	-0.3251	0.001	0.0010909	3860	50256	61.466	612	7622	9.3222	286	3654	4.4691	7.4093	7.2708	46.732	47.9402
SP8	ERG_2	cluster_2	DP	7566	9478.784	9255	9728	149.0926	0.7982	-0.3251	0.001	0.001	6800	88560	61.21	1235	15627	10.801	585	7566	5.2294	8.6029	8.5434	47.3684	48.4162
SP4	BPTF_1	cluster_2	DP	12613	15802.084	15473	16137	198.2637	0.7982	-0.3252	0.001	0.0010909	12832	172114	61.355	2078	26531	9.4577	965	12613	4.4963	7.5203	7.3283	46.4389	47.5406
SP8	ARID3A_2	cluster_5	Common_1_DN	3264	4090.208	3921	4244	100.4049	0.7981	-0.3254	0.001	0.0013333	1242	20101	63.924	461	6775	21.546	213	3264	10.38	17.1498	16.238	46.2039	48.1771
SP8	FOXM1_1	cluster_5	Common_1_DN	6976	8741.673	8528	8934	124.8844	0.798	-0.3255	0.001	0.0010909	4986	39915	56.233	2009	15722	22.15	878	6976	9.828	17.6093	17.4771	43.7033	44.3709
SP4	ATF3_known5	cluster_2	DP	15216	19073.922	18690	19479	244.0673	0.7977	-0.326	0.001	0.001	11915	167390	58.274	2441	33101	11.523	1092	15216	5.2972	9.1649	9.0901	44.7358	45.9684
SP4	SOX4_1	cluster_2	DP	17108	21445.683	21007	21863	266.185	0.7977	-0.326	0.001	0.0010435	12657	205161	65.153	2224	34044	10.811	1076	17108	5.433	8.5012	8.3388	48.3813	50.2526
SP8	SOX12_1	cluster_5	Common_1_DN	5515	6913.434	6684	7136	137.0285	0.7978	-0.326	0.001	0.0010435	2969	38740	61.39	975	11756	18.629	444	5515	8.7394	14.9545	14.2359	45.5385	46.9122
DP	IKZF1_1	cluster_5	Common_1_DN	18527	23226.508	22860	23611	222.7899	0.7977	-0.3261	0.001	0.0010435	8537	102703	58.812	3496	40165	23	1565	18527	10.609	18.332	18.0394	44.7654	46.1272
SP4	SOX5_3	cluster_5	Common_1_DN	5523	6924.67	6708	7120	133.7248	0.7976	-0.3262	0.001	0.0011429	2611	39250	66.643	789	10931	18.56	384	5523	9.3775	14.707	14.0713	48.6692	50.526
SP8	HBP1_1	cluster_5	Common_1_DN	9961	12489.38	12100	12832	211.3091	0.7976	-0.3263	0.001	0.0010435	4130	63662	59.134	1516	21821	20.269	666	9961	9.2525	16.1259	15.6467	43.9314	45.6487
SP8	GATA_known17	cluster_5	Common_1_DN	9892	12403.661	12082	12765	204.2092	0.7975	-0.3264	0.001	0.0010909	3680	58991	55.43	1511	22926	21.542	629	9892	9.2949	17.0924	16.7687	41.6281	43.1475
SP8	POU2F2_known11	cluster_5	Common_1_DN	6861	8603.151	8404	8826	134.7805	0.7975	-0.3264	0.001	0.0010435	4428	44372	59.446	1531	14781	19.802	703	6861	9.1918	15.8762	15.4625	45.9177	46.4177
SP8	TBX20_2	cluster_2	DP	8632	10823.692	10493	11162	203.884	0.7975	-0.3264	0.001	0.0010435	5480	98825	56.841	1123	19395	11.155	482	8632	4.9649	8.7956	8.7346	42.9207	44.5063
SP4	SOX3_3	cluster_5	Common_1_DN	6983	8759.43	8471	9034	165.2267	0.7972	-0.3269	0.001	0.0010909	2900	46500	59.883	1032	15271	19.666	454	6983	8.9927	15.6552	15.0172	43.9922	45.7272
DP	PKNOX2_2	cluster_5	Common_1_DN	22839	28648.742	28207	29125	282.8633	0.7972	-0.327	0.001	0.0010435	11043	122680	50.42	5291	57301	23.55	2066	22839	9.3866	18.7087	18.6167	39.0474	39.8579
SP8	SOX15_2	cluster_5	Common_1_DN	6258	7851.171	7600	8076	146.8445	0.7971	-0.3272	0.001	0.0010909	3083	43248	60.363	1049	13569	18.939	474	6258	8.7345	15.3746	14.47	45.1859	46.1198
SP8	SPI1_known1	cluster_2	DP	23246	29172.639	28823	29548	221.3128	0.7968	-0.3276	0.001	0.001	35122	247084	52.91	7958	55408	11.865	3304	23246	4.9778	9.4072	9.4081	41.518	41.9542
SP8	GMEB2_3	cluster_4	SP4	1337	1678.469	1581	1771	59.5372	0.7967	-0.3279	0.001	0.0010435	2094	29627	71.145	180	2424	5.8209	97	1337	3.2106	4.6323	4.5128	53.8889	55.1568
DP	MAFF_1	cluster_5	Common_1_DN	23329	29284.019	28749	29762	298.7586	0.7967	-0.328	0.001	0.001	9294	130389	52.132	4204	56810	22.714	1677	23329	9.3274	18.0439	17.8918	39.8906	41.065
SP8	KLF4_1	cluster_4	SP4	39359	49409.881	48882	49867	303.8428	0.7966	-0.3281	0.001	0.001	80637	776764	66.873	8010	74996	6.4566	4128	39359	3.3885	5.1192	5.067	51.5356	52.4815
DN	E2F_known26	cluster_2	DP	2262	2840.549	2713	2962	75.1549	0.7964	-0.3284	0.001	0.0010435	2046	34813	80.496	237	3703	8.5622	140	2262	5.2303	6.8426	6.4976	59.0717	61.0856
DN	SOX21_2	cluster_2	DP	5027	6312.792	6150	6506	108.1599	0.7964	-0.3285	0.001	0.0010909	4666	65357	73.59	668	8902	10.023	367	5027	5.6603	7.8654	7.6916	54.9401	56.4705
SP8	ATF3_known5	cluster_2	DP	15201	19089.217	18684	19519	249.1797	0.7963	-0.3286	0.001	0.001	11933	167611	58.351	2441	33101	11.523	1090	15201	5.2919	9.1343	9.0692	44.6538	45.9231
DP	PKNOX1_1	cluster_5	Common_1_DN	29127	36580.996	36001	37229	365.7206	0.7962	-0.3287	0.001	0.0010435	10906	164350	52.965	4839	69909	22.529	1963	29127	9.3867	17.9993	17.7225	40.5662	41.6642
SP8	SMAD_2	cluster_5	Common_1_DN	40147	50426.993	49813	50993	344.9424	0.7961	-0.3289	0.001	0.0010435	21409	221606	55.683	9075	91574	23.01	3887	40147	10.088	18.1559	18.1164	42.832	43.841
SP8	TCF7L1_1	cluster_2	DP	13262	16665.19	16253	17110	260.0082	0.7958	-0.3295	0.001	0.0010435	9778	157054	56.06	1956	30243	10.795	833	13262	4.7339	8.5191	8.4442	42.5869	43.8515
SP4	SOX4_2	cluster_5	Common_1_DN	11402	14330.432	14074	14658	185.4724	0.7957	-0.3298	0.001	0.0010909	4816	72432	63.066	1660	23480	20.444	784	11402	9.9276	16.2791	15.7417	47.2289	48.5605
SP8	FOXO4_3	cluster_2	DP	2013	2531.16	2383	2663	79.928	0.7954	-0.3303	0.001	0.0010909	2096	27285	58.087	359	4443	9.4586	157	2013	4.2854	7.4905	7.3777	43.7326	45.3072
SP8	MAFF_1	cluster_2	DP	12766	16050.528	15663	16458	237.3789	0.7954	-0.3303	0.001	0.001	9420	132144	52.834	2253	30581	12.227	916	12766	5.1041	9.724	9.6607	40.6569	41.7449
SP4	ETV7_1	cluster_2	DP	25403	31943.576	31533	32346	252.264	0.7953	-0.3305	0.001	0.001	27857	251236	56.588	6344	56465	12.718	2816	25403	5.7217	10.1088	10.1112	44.3884	44.9889
DN	ETV2_1	cluster_2	DP	7148	8989.662	8671	9305	190.1388	0.7952	-0.3307	0.001	0.0010435	7018	70432	23.896	3829	37988	12.888	715	7148	2.4251	10.1881	10.1488	18.6733	18.8165
SP4	FOXO4_2	cluster_2	DP	7124	8963.091	8722	9183	146.8724	0.7948	-0.3313	0.001	0.0010435	6478	84929	66.37	1117	13917	10.876	557	7124	5.5672	8.5983	8.3882	49.8657	51.1892
SP4	SOX30_1	cluster_2	DP	4315	5429.447	5219	5664	136.0627	0.7948	-0.3314	0.001	0.0010435	3742	56229	63.32	626	8864	9.9818	292	4315	4.8591	7.8033	7.674	46.6454	48.6801
SP4	CTCF_known1	cluster_2	DP	79966	100624.342	99463	101729	658.5065	0.7947	-0.3315	0.001	0.0010435	55221	1020372	58.86	9691	172310	9.9396	4357	79966	4.6128	7.8901	7.8369	44.9592	46.4082
DN	BATF_known1	cluster_2	DP	4252	5351.206	5166	5526	103.1822	0.7946	-0.3317	0.001	0.0010435	4780	62340	72.002	592	7502	8.6647	330	4252	4.911	6.9038	6.8207	55.7432	56.6782
SP8	POU2F3_2	cluster_5	Common_1_DN	4249	5348.377	5207	5501	88.9674	0.7945	-0.3319	0.001	0.0010909	3384	27131	59.282	1185	9162	20.019	538	4249	9.2842	15.8983	15.6611	45.4008	46.3763

SP8	IKZF2_3	cluster_2	DP	3333	4195.73	4037	4349	91.4974	0.7944	-0.332	0.001	0.0010909	5533	55534	62.869	728	6899	7.8102	342	3333	3.7732	6.1811	6.0017	46.978	48.3113
SP8	TCF4_1	cluster_5	Common_1_DN	60987	76785.355	76160	77352	383.4195	0.7943	-0.3323	0.001	0.0010909	32686	295493	45.063	19242	171202	26.108	6774	60987	9.3006	20.7245	20.6391	35.2042	35.6228
DP	EWSR1::FLI1_1	cluster_5	Common_1_DN	149726	188537.291	187006	190119	945.1849	0.7941	-0.3325	0.001	0.0010435	43660	868688	53.282	19564	364049	22.329	7722	149726	9.1837	17.6867	17.2359	39.4705	41.128
SP4	HMGA1_1	cluster_2	DP	1279	1611.105	1549	1682	40.5264	0.794	-0.3328	0.001	0.0010435	3745	22602	66.657	417	2455	7.2402	214	1279	3.772	5.7143	5.6588	51.3189	52.0978
SP4	PKNOX2_2	cluster_2	DP	13282	16733.841	16387	17065	197.1207	0.7937	-0.3333	0.001	0.0010435	11364	126178	51.858	2996	32542	13.374	1199	13282	5.4587	10.5509	10.5264	40.02	40.8149
SP8	IKZF2_2	cluster_2	DP	2520	3175.097	3055	3338	86.9893	0.7937	-0.3333	0.001	0.0010435	3469	34690	57.903	584	5590	9.3305	256	2520	4.2062	7.3796	7.2643	43.8356	45.0805
SP4	PKNOX1_2	cluster_2	DP	11476	14460.809	14123	14750	185.0895	0.7936	-0.3335	0.001	0.0010435	9911	109901	52.023	2578	28014	13.261	1036	11476	5.4323	10.453	10.4421	40.1862	40.9652
SP8	CBX5_1	cluster_2	DP	3137	3953.059	3785	4116	97.8266	0.7936	-0.3335	0.001	0.0010435	4252	46829	61.603	629	6602	8.6848	292	3137	4.1267	6.8674	6.6988	46.4229	47.5159
DP	E2F7_1	cluster_5	Common_1_DN	8569	10800.231	10525	11063	162.4784	0.7934	-0.3338	0.001	0.0010435	4150	54006	59.789	1499	18468	20.445	676	8569	9.4865	16.2892	15.8668	45.0967	46.3992
SP8	ETS_known13	cluster_4	SP4	8168	10296.278	10006	10546	175.8214	0.7933	-0.334	0.001	0.001	10079	161754	66.163	1019	15787	6.4574	516	8168	3.341	5.1196	5.0496	50.6379	51.7388
SP8	SOX10_4	cluster_5	Common_1_DN	12631	15923.446	15585	16295	216.7158	0.7932	-0.3342	0.001	0.0010909	5401	81262	58.18	1975	28093	20.113	860	12631	9.0432	15.923	15.5436	43.5443	44.9614
SP4	TBX15_1	cluster_2	DP	9263	11678.371	11357	12000	201.5446	0.7932	-0.3343	0.001	0.0010909	6066	109292	57.806	1217	20690	10.943	527	9263	4.8993	8.6878	8.4755	43.3032	44.7704
SP8	LMO2_1	cluster_5	Common_1_DN	79951	100833.222	99959	101627	495.5513	0.7929	-0.3348	0.001	0.001	35542	394212	47.125	19801	215266	25.733	7242	79951	9.5575	20.3759	20.2812	36.5739	37.1406
SP8	EGR4_2	cluster_4	SP4	35830	45204.355	44642	45816	345.1097	0.7926	-0.3353	0.001	0.001	50126	822981	71.563	4162	64752	5.6306	2227	35830	3.1156	4.4428	4.3537	53.5079	55.3342
SP4	ATF4_2	cluster_2	DP	3503	4421.086	4219	4598	110.4203	0.7924	-0.3357	0.001	0.0010909	3978	47794	58.841	650	7608	9.3666	293	3503	4.3127	7.3655	7.3294	45.0769	46.0436
SP4	PKNOX2_3	cluster_2	DP	11348	14324.52	14006	14661	190.4942	0.7922	-0.336	0.001	0.0010435	10156	113004	52.742	2518	27407	12.792	1021	11348	5.2964	10.0532	10.0421	40.5481	41.4055
SP8	ETV7_1	cluster_2	DP	23093	29157.317	28775	29561	227.9104	0.792	-0.3364	0.001	0.001	25471	229674	51.731	6344	56465	12.718	2562	23093	5.2014	10.0585	10.0547	40.3846	40.8979
DN	RUNX1_4	cluster_1	SP8	2814	3554.05	3436	3674	73.7125	0.7918	-0.3367	0.001	0.0010909	3683	37261	73.637	512	4957	9.7962	286	2814	5.5612	7.7654	7.5521	55.8594	56.7682
SP4	HMGA1_2	cluster_5	Common_1_DN	12630	15951.046	15656	16259	182.5379	0.7918	-0.3368	0.001	0.0010435	5885	82554	67.162	1898	24720	20.111	936	12630	10.275	15.9048	15.2991	49.3151	51.0922
SP4	POU2F2_known10	cluster_5	Common_1_DN	7291	9208.39	9011	9391	119.1035	0.7918	-0.3368	0.001	0.0010435	5130	46258	63.552	1684	14748	20.262	823	7291	10.017	16.0429	15.7616	48.8717	49.4372
SP8	SOX4_1	cluster_2	DP	15861	20032.188	19617	20431	253.1346	0.7918	-0.3368	0.001	0.0010435	11786	191082	60.682	2224	34044	10.811	994	15861	5.037	8.4337	8.3006	44.6942	46.5897
SP4	SOX21_3	cluster_5	Common_1_DN	8356	10554.064	10235	10828	169.1065	0.7918	-0.3369	0.001	0.0010909	3663	54981	62.433	1244	17494	19.865	577	8356	9.4886	15.7521	15.198	46.3826	47.7649
SP8	E2F_known24	cluster_2	DP	3592	4537.47	4346	4720	122.3122	0.7917	-0.337	0.001	0.001	2992	51042	66.197	441	7068	9.1666	214	3592	4.6585	7.1524	7.0373	48.5261	50.8206
DP	CTCF_known1	cluster_5	Common_1_DN	193687	244686.564	243230	246117	944.7875	0.7916	-0.3372	0.001	0.0010435	55720	1030489	59.443	23232	415314	23.957	10521	193687	11.173	18.8819	18.7956	45.2867	46.6363
SP4	TCF7_1	cluster_2	DP	15671	19799.573	19355	20221	254.2812	0.7915	-0.3374	0.001	0.0010435	11748	188705	61.707	2102	32593	10.658	987	15671	5.1245	8.4014	8.3045	46.9553	48.0809
DN	SOX3_1	cluster_2	DP	3086	3900.019	3736	4055	92.4305	0.7913	-0.3376	0.001	0.0010909	2538	40630	73.556	376	5627	10.187	202	3086	5.5868	7.959	7.5954	53.7234	54.8427
DP	ETV7_1	cluster_5	Common_1_DN	40794	51554.089	50957	52086	351.2295	0.7913	-0.3377	0.001	0.001	25386	228951	51.568	11316	100370	22.607	4531	40794	9.1883	17.8484	17.8178	40.0407	40.6436
SP4	SOX9_4	cluster_5	Common_1_DN	12571	15886.373	15526	16233	204.9482	0.7913	-0.3377	0.001	0.0010909	5343	80252	62.068	1850	26308	20.347	860	12571	9.7227	16.0958	15.6644	46.4865	47.7839
DN	SMAD_1	cluster_2	DP	14008	17704.026	17492	17896	123.5814	0.7912	-0.3378	0.001	0.0010435	20794	166413	76.255	3011	23523	10.779	1771	14008	6.4189	8.5169	8.4176	58.8177	59.5502
SP4	SPI1_known1	cluster_2	DP	25022	31624.072	31295	31928	200.9872	0.7912	-0.3378	0.001	0.001	38052	267768	57.339	7958	55408	11.865	3555	25022	5.3582	9.3425	9.3447	44.672	45.1595
SP8	SOX10_9	cluster_5	Common_1_DN	6845	8653.143	8386	8903	164.3127	0.7911	-0.3381	0.001	0.0010435	3412	47797	56.857	1186	15642	18.607	498	6845	8.1425	14.5955	14.321	41.9899	43.7604
SP8	MGA_2	cluster_2	DP	7798	9858.343	9549	10186	190.7256	0.791	-0.3382	0.001	0.0010909	5058	86090	56.587	1087	17784	11.689	466	7798	5.1256	9.2131	9.058	42.8703	43.8484
SP8	HBP1_1	cluster_2	DP	4525	5720.917	5511	5953	141.4661	0.791	-0.3383	0.001	0.0010435	4130	63662	59.134	703	10052	9.3371	300	4525	4.2032	7.2639	7.1079	42.6743	45.0159
SP8	ID4_1	cluster_5	Common_1_DN	54293	68643.338	68045	69326	398.1619	0.7909	-0.3383	0.001	0.0010435	29653	268120	45.899	16901	150382	25.744	6032	54293	9.2943	20.342	20.2495	35.6902	36.1034
SP8	TBX21_4	cluster_2	DP	10359	13097.13	12786	13454	198.6129	0.791	-0.3383	0.001	0.0010909	7628	114668	56.477	1649	23664	11.655	702	10359	5.1021	9.2029	9.0339	42.5713	43.7754
SP8	SOX1_3	cluster_5	Common_1_DN	6575	8313.177	8047	8544	152.9199	0.7909	-0.3384	0.001	0.0010909	2972	41649	55.348	1186	15495	20.591	485	6575	8.7375	16.319	15.7867	40.8938	42.433
SP8	MAFG_1	cluster_2	DP	11567	14627.519	14140	15107	271.1731	0.7908	-0.3386	0.001	0.0010435	6025	120737	52.864	1475	28081	12.295	584	11567	5.0645	9.6929	9.5803	39.5932	41.1916
SP8	SOX18_4	cluster_5	Common_1_DN	4391	5552.728	5341	5780	132.1799	0.7908	-0.3386	0.001	0.0012	2159	30301	57.531	773	10090	19.157	327	4391	8.337	15.1459	14.4913	42.3027	43.5183
SP8	SOX8_2	cluster_5	Common_1_DN	12256	15499.707	15117	15804	209.6513	0.7907	-0.3387	0.001	0.0010909	5294	79524	62.195	1805	25656	20.065	840	12256	9.5853	15.867	15.4117	46.5374	47.7705
SP4	TBX4_2	cluster_2	DP	11860	15001.01	14635	15394	228.4102	0.7906	-0.3389	0.001	0.0010909	6921	131760	58.982	1423	25905	11.596	630	11860	5.3091	9.1027	9.0012	44.2727	45.7827
SP4	KLF16_1	cluster_4	SP4	44753	56623.67	56087	57155	320.5644	0.7904	-0.3394	0.001	0.001	79277	887559	67.446	7768	85275	6.4801	3954	44753	3.4008	4.9876	5.0423	50.9011	52.4808
SP8	TBX20_2	cluster_5	Common_1_DN	16733	21175.077	20724	21611	264.5722	0.7902	-0.3397	0.001	0.0010435	5480	98825	56.841	2226	38036	21.877	945	16733	9.6244	17.2445	16.932	42.4528	43.9925
SP4	GMEB2_2	cluster_2	DP	2606	3298.754	3148	3437	88.8772	0.7901	-0.34	0.001	0.001	2773	36777	66.35	402	5050	9.1108	200	2606	4.7015	7.2124	7.086	49.7512	51.604
SP8	KLF7_1	cluster_4	SP4	55903	70759.466	70066	71539	447.8146	0.79	-0.34	0.001	0.001	75221	1217400	68.768	6936	105944	5.9845	3557	55903	3.1578	4.7287	4.592	51.2832	52.7666
SP4	SOX18_4	cluster_2	DP	2386	3020.563	2878	3171	90.2088	0.79	-0.3401	0.001	0.0012	385	5045	62.2	385	5045	9.5787	178	2386	4.5302	7.6231	7.2833	46.2338	47.2944
SP8	SOX_1	cluster_2	DP	6123	7751.176	7496	7985	150.3185	0.79	-0.3401	0.001	0.0010435	6625	79556	57.773	1171	13573	9.8566	515	6123	4.4465	7.7736	7.6965	43.9795	45.1116
DN	NFE2L1::MAFG_1	cluster_2	DP	23171	29337.995	28895	29801	271.0613	0.7898	-0.3404	0.001	0.0010435	11526	243206	74.83	2037	40418	12.436	1115	23171	7.1293	9.6738	9.5273	54.7374	57.3284
SP8	TCF3_2	cluster_5	Common_1_DN	70432	89183.508	88272	90137	570.494	0.7897	-0.3405	0.001	0.0010435	23089	348566	45.448	13449	197852	25.797	4695	70432	9.1834	20.3344	20.2062	34.9097	35.5983
SP8	EGR3_2	cluster_4	SP4	29542	37408.103	36893	37946	295.5053	0.7897	-0.3406	0.001	0.001	46634	711154	72.287	3669	53073	5.3948	1985	29542	3.0029	4.2566	4.1541	54.1019	55.663
DN	FOXP3_1	cluster_2	DP	13335	16889.717	16531	17227	201.1777	0.7895	-0.3409	0.001	0.0010909	9966	159936	72.213	1558	23954	10.816	828	13335	6.0209	8.3082	8.3377	53.1451	55.6692
SP4	POU2F3_1	cluster_2	DP	4315	5465.52	5264	5659	128.202	0.7895	-0.3409	0.001	0.0010435	3852	58181	62.51	635	9011	9.6815	290	4315	4.6361	7.5286	7.4165	45.6693	47.8859
SP4	SMAD_2	cluster_2	DP	20131	25500.84	25060	25888	252.0878	0.7894	-0.3411	0.001	0.0010435	22080	227820	57.244	4479	45031	11.315	1981	20131	5.0583	8.9719	8.8364	44.2286	44.7048
SP8	SOX7_4	cluster_5	Common_1_DN	6113	7744.957	7490	8025	170.7347	0.7893	-0.3413	0.001	0.0010909	2647	42472	56.757	969	14283	19.087	400	6113	8.1691	15.1114	14.393	41.2797	42.7991
DN	SOX14_2	cluster_2	DP	3557	4508.234	4368	4662	85.6916	0.789	-0.3418	0.001	0.0010909	4224	46656	72.886	603	6367	9.9466	332	3557	5.5568	7.8598	7.6239	55.058	55.8662
SP8	SMAD3_3	cluster_5	Common_1_DN	5823	7380.373	7212	7571	110.6413	0.789	-0.3419	0.001	0.0010435	3767	33918	58.906	1443	12645	21.961	653	5823	10.113	17.3347	17.1679	45.2529	46.0498
SP4	PBX1_4	cluster_2	DP	2069	2623.803	2499	2740	76.7992	0.7886	-0.3426	0.001	0.0010909	2946	33026	61.318	409	4372	8.1173	189	2069	3.8414	6.4155	6.2648	46.2103	47.3239
SP8	TBX1_2	cluster_2	DP	7143	9059.876	8717	9391	196.567	0.7884	-0.3429	0.001	0.0010909	4843	87270	55.954	979	16664	10.684	403	7143	4.5798	8.3213	8.1849	41.1645	42.8649
SP8	EGR4_1	cluster_4	SP4	18973	24071.978	23724	24434	214.3365	0.7882	-0.3434	0.001	0.001	30552	346098	68.54	3257	35643	7.0586	1698	18973	3.7573	5.5577	5.482	52.1339	53.2306
SP4	SOX11_1	cluster_2	DP	15656	19877.621	19428	20307	264.4201	0.7876	-0.3444	0.001	0.0010435	11736	190304	65.17	2057	31531	10.798	982	15656	5.3614	8.3674	8.2268	47.7394	49.6527
DN	ETV2_1	cluster_1	SP8	4341	5512.497	5250	5795	160.5816	0.7875	-0.3446	0.001	0.0010435	7018	70432	23.896	2363	23363	7.9265	435	4341	1.4728	6.1983	6.1634	18.4088	18.5807
DN	SOX2_5	cluster_2	DP	4730	6007.724	5828	6200	112.0849	0.7874	-0.3449	0.001	0.0010909	6046	64802	73.448	561	8492	9.6251	305	4730	5.3611	7.5383	7.2992	54.3672	55.6995
SP8	TBX20_5	cluster_5	Common_1_DN	32765	41618.872	41041	42172	341.8757	0.7873	-0.3451	0.001	0.0010909	12908	197715	58.58	5012	72644	21.523	2174	32765	9.7078	16.8423	16.5718	43.3759	45.1035
SP4	ERG_2	cluster_2	DP	7879	10009.16	9763	10255	145.8674	0.7872	-0.3452	0.001	0.001	7181	93517	64.636	1235	15627	10.801	609	7879	5.4457	8.4807	8.4252	49.3117	50.4191
SP4	IKZF3_1	cluster_2	DP	5667	7200.614	6950	7448	143.7217	0.787	-0.3455	0.001	0.0010435	6633	79768	59.129	1082	12441	9.222	480	5667	4.2007	7.2365	7.1044	44.3623	45.551
SP4	FLI1_4	cluster_2	DP	8034	10210.761	9990	10446	143.4705	0.7868	-0.3459	0.001	0.001	7443	97009	66.151	1231	15560	10.611	620	8034	5.4785	8.33	8.2817	50.3656	51.6324
SP8	ATF6_1	cluster_2	DP	1851	2353.027	2262	2451	57.9713	0.7867	-0.346	0.001	0.0010435	3930	27520	63.816	534	3697	8.573	265	1851	4.2923	6.743	6.726	49.6255	50.0676
SP8	SOX30_1	cluster_2	DP	4069	5176.519	4959	5380	127.9827	0.7861	-0.3472	0.001	0.0010435	3553	53360	60.089	626	8864	9.9818	274	4069	4.5821	7.7118	7.6256	43.77	45.9048
SP8	TCF21_1	cluster_5	Common_1_DN	25891	32936.144	32401	33467	324.8764	0.7861	-0.3472	0.001	0.001	10166	132387	47.014	5599	70697	25.106	2004	25891	9.1945	19.7128	19.5571	35.7921	36.6225
SP4	IKZF2_3	cluster_2	DP	3472	4417.76	4259	4559	93.9804	0.786	-0.3475	0.001	0.0010909	5825	58444	66.163	728	6899	7.8102	356	3472	3.9306	6.1116	5.9407	48.9011	50.3261
DP	SP100_1	cluster_5	Common_1_DN	1511	1922.888	1822	2019	61.2329	0.7859	-0.3476	0.001	0.0013333	864	11233	66.811	242	2948	17.534	120	1511	8.9871	13.8889	13.4514	49.5868	51.2551
SP4	KLF13_1	cluster_2	DP	24940	31740.453	31155	32250	348.952	0.7858	-0.3478	0.001	0.001	20223	347988	63.027	3116	51120	9.2588	1471	24940	4.5171	7.2739	7.1669	47.208	48.7872
SP4	POU2F2_known14	cluster_5	Common_1_DN	2248	2862.065	2727	3023	92.6879	0.7855	-0.3483	0.001	0.0012632	922	14165	62.953	353	4849	21.55	157	2248	9.9907	17.0282	15.8701	44.4759	46.3601
SP4	XBP1_2	cluster_2	DP	4347	5534.636	5307	5768	137.03	0.7855	-0.3484	0.001	0.0010435	2736	63041	65.364	396	8574	8.8899	192	4347	4.5072	7.0175	6.8955	48.4848	50.6998
DP	CUX1_9	cluster_3	DN	904	1151.397	1049	1269	69.7849	0.7853	-0.3486	0.001	0.0012632	440	7588	51.249	151	2358	15.926	55	904	6.1056	12.5	11.9135	36.4238	38.3376
SP4	FLI1_2	cluster_2	DP	7986	10168.824	9907	10407	152.5302	0.7854	-0.3486	0.001	0.001	7024	91445	65.047	1240	15749	11.203	618	7986	5.6806	8.7984	8.7331	49.8387	50.708
SP4	SOX14_3	cluster_2	DP	3507	4465.858	4268	4650	116.4952	0.7853	-0.3486	0.001	0.0010909	3418	47906	58.588	592	7830	9.5759	255	3507	4.289	7.4605	7.3206	43.0743	44.7893
SP4	MGA_2	cluster_2	DP	7922	10087.946	9781	10409	203.9381	0.7853	-0.3487	0.001	0.0010909	5162	87892	57.771	1087	17784	11.689	472	7922	5.2071	9.1437	9.0133	43.4223	44.5457
SP8	ERG_4	cluster_2	DP	7267	9254.143	8984	9512	158.7532	0.7853	-0.3487	0.001	0.001	6801	88600	61.768	1199	15137	10.553	561	7267	5.0662	8.2488	8.202	46.789	48.0082
SP8	SP4_1	cluster_4	SP4	57395	73087.781	72335	73787	445.8305	0.7853	-0.3487	0.001	0.001	82983	1490454	71.892	6296	105261	5.0772	3307	57395	2.7684	3.9852	3.8508	52.5254	54.5264
DN	FOXO6_1	cluster_2	DP	3426	4364.243	4227	4508	91.664	0.7851	-0.3491	0.001	0.0010909	3202	41765	72.845	496	6186	10.789	267	3426	5.9755	8.3385	8.203	53.8306	55.3831
SP4	ERG_4	cluster_2	DP	7646	9739.91	9476	9962	148.3996	0.785	-0.3492	0.001	0.001	7152	93169	64.953	1199	15137	10.553	590	7646	5.3304	8.2494	8.2066	49.2077	50.512
SP8	MYBL2_4	cluster_5	Common_1_DN	3475	4427.031	4267	4604	105.3726	0.785	-0.3492	0.001	0.0010435	1616	22677	64.03	524	7050	19.906	253	3475	9.8119	15.6559	15.3239	48.2824	49.2908
DN	ELK4_2	cluster_1	SP8	3554	4528.63	4288	4754	141.4633	0.7848	-0.3495	0.001	0.0011429	7819	70678	26.176	1952	17453	6.4637	395	3554	1.3162	5.0518	5.0284	20.2357	20.3633
SP4	SOX10_4	cluster_5	Common_1_DN	13252	16884.643	16548	17262	216.108	0.7849	-0.3495	0.001	0.0010909	5739	86325	61.805	1975	28093	20.113	906	13252	9.4878	15.7867	15.3513	45.8734	47.1719
DP	MYBL1_3	cluster_5	Common_1_DN	2414	3076.888	2944	3208	84.709	0.7846	-0.3499	0.001	0.0010909	1344	14803	55.839	534	5618	21.192	220	2414	9.106	16.369	16.3075	41.1985	42.969
SP8	SP4_2	cluster_4	SP4	43649	55630.902	54916	56213	402.5105	0.7846	-0.3499	0.001	0.001	59003	991063	69.566	5244	82431	5.7861	2689	43649	3.0639	4.5574	4.4043	51.2777	52.9522
SP4	IKZF2_2	cluster_2	DP	2644	3370.188	3224	3497	79.5819	0.7846	-0.35	0.001	0.0010435	3698	36980	61.725	584	5590	9.3305	269	2644	4.4132	7.2742	7.1498	46.0616	47.2987
SP8	POU2F2_known12	cluster_5	Common_1_DN	6737	8587.394	8359	8804	135.1965	0.7845	-0.3501	0.001	0.0010435	4381	43877	60.114	1513	14607	20.012	692	6737	9.23	15.7955	15.3543	45.7369	46.1217
SP4	ATF2_2	cluster_2	DP	3827	4879.358	4705	5057	106.0527	0.7844	-0.3504	0.001	0.0010435	5130	56548	65.885	694	7476	8.7104	350	3827	4.4589	6.8226	6.7677	50.4323	51.1905
SP4	TCF7L1_1	cluster_2	DP	14377	18332.521	17992	18750	239.8503	0.7842	-0.3506	0.001	0.0010435	10758	172842	61.696	1956	30243	10.795	904	14377	5.1319	8.403	8.318	46.2168	47.5383
SP4	SOX11_2	cluster_5	Common_1_DN	7778	9919.602	9686	10170	148.55	0.7841	-0.3508	0.001	0.0010909	3896	54587	64.334	1219	15941	18.787	577	7778	9.1668	14.8101	14.2488	47.3339	48.7924
SP8	PKNOX1_1	cluster_2	DP	15166	19340.576	18931	19754	258.8186	0.7842	-0.3508	0.001	0.0010435	10991	165550	53.351	2530	36762	11.847	1015	15166	4.8875	9.2348	9.1611	40.1186	41.2546
SP8	ETV6_2	cluster_2	DP	8500	10844.875	10510	11156	195.6291	0.7838	-0.3514	0.001	0.0011429	10041	90698	24.501	4960	44381	11.989	944	8500	2.2961	9.4015	9.3718	19.0323	19.1523
SP4	NFKB_known11	cluster_2	DP	11575	14767.523	14457	15077	189.61	0.7838	-0.3514	0.001	0.001	11939	145036	63.244	1997	23563	10.275	961	11575	5.0474	8.0493	7.9808	48.1222	49.1236
SP4	SOX15_2	cluster_5	Common_1_DN	6398	8163.719	7920	8417	152.4507	0.7837	-0.3516	0.001	0.0010909	3199	44866	62.621	1049	13569	18.939	479	6398	8.9299	14.9734	14.2602	45.6625	47.1516
SP8	TCF7L2_known5	cluster_2	DP	14842	18938.653	18534	19359	260.9781	0.7837	-0.3516	0.001	0.0010435	10823	173781	55.479	2240	34675	11.07	931	14842	5.4406	8.6021	8.5406	41.5625	42.8032
SP4	IKZF1_2	cluster_5	Common_1_DN	28811	36767.794	36388	37234	253.8037	0.7836	-0.3518	0.001	0.0010435	20377	163023	59.485	8047	62565	22.829	3641	28811	10.513	17.8682	17.673	45.2467	46.0497
SP8	ETV5_1	cluster_2	DP	2966	3785.65	3569	3984	125.7685	0.7835	-0.3519	0.001	0.0010435	4199	38058	26.542	1599	14322	9.9884	328	2966	2.0685	7.8114	7.7934	20.5128	20.7094
DN	ETV6_2	cluster_1	SP8	5108	6520.875	6261	6775	161.5207	0.7834	-0.3522	0.001	0.0011429	10518	94973	25.655	2882	25678	6.9365	570	5108	1.3798	5.4193	5.3784	19.7779	19.8925
SP8	EGR1_known9	cluster_4	SP4	15647	19978.344	19665	20333	206.8914	0.7832	-0.3525	0.001	0.001	29509	400130	74.089	2102	27438	5.0805	1175	15647	2.8972	3.9818	3.9105	55.8991	57.0268
SP4	HBP1_1	cluster_2	DP	4782	6106.124	5866	6311	129.578	0.7832	-0.3526	0.001	0.0010435	4429	68253	63.399	703	10052	9.3371	317	4782	4.4419	7.1574	7.0063	45.0925	47.5726
SP8	TBX21_4	cluster_5	Common_1_DN	19180	24491.599	24080	24916	253.1941	0.7831	-0.3527	0.001	0.0010909	7628	114668	56.477	3068	44178	21.759	1301	19180	9.4466	17.0556	16.7265	42.4055	43.4153
DN	ELK4_2	cluster_2	DP	7048	9001.641	8663	9308	191.6975	0.783	-0.3529	0.001	0.0011429	7819	70678	26.176	3873	34667	12.839	784	7048	2.6102	10.0269	9.972	20.2427	20.3306
SP8	MAF_known10	cluster_5	Common_1_DN	15708	20061.304	19685	20372	200.6031	0.783	-0.3529	0.001	0.0010909	8275	91208	55.774	3417	36443	22.285	1439	15708	9.6056	17.3897	17.2222	42.113	43.1029
DN	ATF2_1	cluster_2	DP	567	724.547	684	763	25.204	0.7829	-0.3532	0.001	0.0010435	1484	10388	76.433	137	959	7.0561	81	567	4.1719	5.4582	5.4582	59.1241	59.1241
SP8	POU2F3_1	cluster_2	DP	4022	5138.218	4923	5369	132.4292	0.7828	-0.3533	0.001	0.0010435	3586	54118	58.145	635	9011	9.6815	269	4022	4.3213	7.5014	7.4319	42.3622	44.6343
SP8	TBX1_2	cluster_5	Common_1_DN	14726	18814.253	18392	19258	272.8926	0.7827	-0.3534	0.001	0.0010909	4843	87270	55.954	2030	34408	22.061	842	14726	9.4417	17.3859	16.8741	41.4778	42.7982
SP4	RREB1_1	cluster_5	Common_1_DN	129699	165716.554	164600	166801	667.8267	0.7827	-0.3535	0.001	0.001	47895	812464	64.416	17321	268951	21.324	7891	129699	10.283	16.4756	15.9637	45.5574	48.224
SP4	SMAD3_1	cluster_2	DP	14807	18920.216	18621	19220	179.0805	0.7826	-0.3536	0.001	0.0010435	20754	171916	58.449	4021	32716	11.123	1810	14807	5.0342	8.7212	8.6129	45.0137	45.2592
SP8	SOX11_1	cluster_2	DP	14483	18508.702	18081	18947	268.5399	0.7825	-0.3538	0.001	0.0010435	10921	177105	60.65	2057	31531	10.798	906	14483	4.9597	8.2959	8.1776	44.0447	45.9326
SP8	TCF7_2	cluster_2	DP	12127	15507.161	15212	15797	184.3015	0.782	-0.3547	0.001	0.0010435	13256	146350	61.849	2353	25359	10.717	1104	12127	5.125	8.3283	8.2863	46.9188	47.8213
SP4	TCF7L2_known5	cluster_2	DP	16284	20827.214	20444	21237	244.1213	0.7819	-0.355	0.001	0.0010435	11860	190547	60.832	2240	34675	11.07	1024	16284	5.1987	8.6341	8.5459	45.7143	46.9618
SP8	ETV2_1	cluster_2	DP	16795	21482.701	21161	21833	203.7228	0.7818	-0.3551	0.001	0.0010435	16594	166680	56.55	3829	37988	12.888	1677	16795	5.6981	10.1061	10.0762	43.7973	44.2113
SP4	RUNX1_4	cluster_2	DP	2077	2657.167	2533	2779	74.3026	0.7817	-0.3552	0.001	0.0010909	3165	32119	63.475	442	4280	8.4583	208	2077	4.1047	6.5719	6.4666	47.0588	48.528
SP8	SOX18_2	cluster_5	Common_1_DN	5336	6825.704	6627	7054	132.2874	0.7818	-0.3552	0.001	0.0010909	2756	38602	58.784	919	11986	18.252	399	5336	8.1257	14.4775	13.8231	43.4168	44.5186
SP4	IKZF1_2	cluster_2	DP	13369	17108.738	16817	17379	175.7107	0.7814	-0.3558	0.001	0.0010435	20507	164063	59.864	3711	28886	10.54	1686	13369	4.8781	8.2216	8.1487	45.4325	46.2819
SP4	SOX7_4	cluster_2	DP	3232	4137.482	3981	4344	117.4532	0.7812	-0.3562	0.001	0.0010909	2839	45561	60.885	471	7079	9.46	206	3232	4.3191	7.2561	7.0938	43.7367	45.6562
SP4	TCF21_1	cluster_5	Common_1_DN	26563	34002.79	33532	34502	294.5018	0.7812	-0.3562	0.001	0.001	10517	136988	48.648	5599	70697	25.106	2060	26563	9.4332	19.5873	19.3907	36.7923	37.573
SP8	IKZF2_2	cluster_5	Common_1_DN	6032	7721.75	7515	7934	127.4114	0.7812	-0.3562	0.001	0.0010435	3469	34690	57.903	1427	13658	22.797	618	6032	10.068	17.8149	17.3883	43.3076	44.1646
SP8	PBX3_known1	cluster_5	Common_1_DN	13053	16710.149	16365	17056	208.5611	0.7812	-0.3563	0.001	0.0010435	6890	76602	56.96	2777	29759	22.128	1185	13053	9.7061	17.1988	17.04	42.6719	43.8624
SP4	TBX15_2	cluster_2	DP	9977	12775.485	12523	13048	153.8759	0.781	-0.3567	0.001	0.0010435	14978	104871	54.579	3419	23567	12.265	1434	9977	5.1924	9.574	9.5136	41.9421	42.3346
SP4	LMO2_1	cluster_5	Common_1_DN	78888	101025.411	100267	101870	503.2579	0.7809	-0.3568	0.001	0.001	35624	395160	47.238	19801	215266	25.733	7150	78888	9.4304	20.0707	19.9636	36.1093	36.6468
SP8	TBX15_1	cluster_5	Common_1_DN	17692	22658.724	22167	23135	311.571	0.7808	-0.357	0.001	0.0010909	5892	106171	56.155	2443	41328	21.859	1006	17692	9.3575	17.074	16.6637	41.1789	42.8087
SP8	SOX17_4	cluster_5	Common_1_DN	5366	6873.443	6638	7118	138.9611	0.7807	-0.3571	0.001	0.0010909	2660	37246	58.82	919	12059	19.044	400	5366	8.4741	15.0376	14.4069	43.5256	44.4979
SP4	ATF4_3	cluster_2	DP	3940	5047.428	4855	5254	123.0132	0.7806	-0.3573	0.001	0.0010909	3989	51908	59.647	685	8615	9.8993	307	3940	4.5274	7.6962	7.5904	44.8175	45.7342

SP8	IKZF1_2	cluster_2	DP	13266	16994.994	16711	17284	177.1219	0.7806	-0.3574	0.001	0.0010435	20377	163023	59.485	3711	28886	10.54	1672	13266	4.8406	8.2053	8.1375	45.0552	45.9254
SP4	E2F_known26	cluster_5	Common_1_DN	4358	5584.917	5393	5786	121.0137	0.7804	-0.3578	0.001	0.0010435	1689	28742	66.459	551	8698	20.112	264	4358	10.077	15.6306	15.1625	47.9129	50.1035
SP8	SPIB_1	cluster_2	DP	7802	10000.325	9808	10178	115.0375	0.7802	-0.3581	0.001	0.0010435	16443	98658	52.897	3185	18963	10.167	1304	7802	4.1831	7.9304	7.9081	40.9419	41.1433
SP8	MAF_known12	cluster_5	Common_1_DN	21392	27422.121	26932	27937	299.6093	0.7801	-0.3583	0.001	0.0010435	7662	130608	59.214	2875	47044	21.328	1269	21392	9.6986	16.5623	16.3788	44.1391	45.4723
SP8	FOXO4_2	cluster_5	Common_1_DN	11090	14218.73	13907	14532	183.1801	0.78	-0.3585	0.001	0.0010435	6205	81377	63.594	1895	23131	18.076	874	11090	8.6666	14.0854	13.6279	46.1214	47.9443
SP8	SP1_known2	cluster_4	SP4	59274	75994.042	75327	76690	404.742	0.78	-0.3585	0.001	0.001	116483	1617881	71.388	8420	109754	4.8428	4438	59274	2.6154	3.81	3.6637	52.7078	54.0062
SP8	SMAD_1	cluster_5	Common_1_DN	22534	28896.903	28547	29286	222.6583	0.7798	-0.3588	0.001	0.0010435	15801	126448	57.942	6404	50170	22.989	2829	22534	10.326	17.9039	17.8208	44.1755	44.9153
DP	HESX1_1	cluster_5	Common_1_DN	1874	2403.79	2292	2518	69.9443	0.7797	-0.359	0.001	0.0012632	1124	10127	51.054	556	4768	24.037	210	1874	9.4475	18.6833	18.505	37.7698	39.3037
SP4	FOXO3_1	cluster_2	DP	6014	7713.295	7509	7931	132.9962	0.7797	-0.359	0.001	0.0010435	5633	74003	67.715	965	11860	10.852	474	6014	5.503	8.4147	8.1267	49.1192	50.7083
SP4	TCF4_1	cluster_5	Common_1_DN	60670	77814.777	77211	78426	400.4488	0.7797	-0.3591	0.001	0.0010909	33174	299877	45.732	19242	171202	26.108	6740	60670	9.2522	20.3171	20.2316	35.0275	35.4377
SP8	MYBL1_5	cluster_5	Common_1_DN	7176	9206.284	8909	9464	168.0555	0.7795	-0.3594	0.001	0.0010435	2849	45954	65.783	933	14337	20.523	452	7176	10.272	15.8652	15.6156	48.4459	50.0523
SP8	SOX21_2	cluster_5	Common_1_DN	7639	9802.032	9522	10062	161.7594	0.7794	-0.3597	0.001	0.0010909	3688	51655	58.162	1332	17389	19.58	566	7639	8.6013	15.3471	14.7885	42.4925	43.9301
SP8	POU2F3_1	cluster_5	Common_1_DN	8166	10480.649	10219	10755	167.0002	0.7792	-0.36	0.001	0.0010435	3586	54118	58.145	1307	18511	19.888	554	8166	8.7737	15.449	15.0892	42.3871	44.1143
SP8	LMO2_2	cluster_5	Common_1_DN	5083	6524.298	6382	6692	96.6299	0.7791	-0.3601	0.001	0.0010435	3634	29090	57.111	1473	11555	22.685	641	5083	9.9792	17.639	17.4734	43.5166	43.9896
SP4	IKZF2_2	cluster_5	Common_1_DN	6407	8226.138	8048	8414	109.806	0.7789	-0.3605	0.001	0.0010435	3698	36980	61.725	1427	13658	22.797	655	6407	10.694	17.7123	17.3256	45.9005	46.9102
SP4	PKNOX1_1	cluster_2	DP	15880	20389.92	19924	20804	270.5884	0.7788	-0.3606	0.001	0.0010435	11588	174615	56.273	2530	36762	11.847	1058	15880	5.1176	9.1301	9.0943	41.8182	43.1968
SP8	FOXP3_2	cluster_2	DP	2484	3189.661	3088	3301	63.1682	0.7788	-0.3606	0.001	0.0010909	4518	27108	59.623	909	5388	11.851	417	2484	5.4634	9.2297	9.1633	45.8746	46.1024
SP8	PBX1_4	cluster_2	DP	1931	2480.052	2357	2604	74.1975	0.7787	-0.3609	0.001	0.0010909	2771	31106	57.753	409	4372	8.1173	177	1931	3.5852	6.3876	6.2078	43.2763	44.1674
DN	ARID5B_1	cluster_2	DP	5629	7231.309	7036	7419	111.5651	0.7785	-0.3613	0.001	0.0010909	5946	77508	76.405	781	9708	9.5698	443	5629	5.5489	7.4504	7.2625	56.7222	57.9831
SP8	FLI1_2	cluster_2	DP	7530	9674.296	9366	9942	163.1877	0.7784	-0.3615	0.001	0.001	6676	86936	61.84	1240	15749	11.203	582	7530	5.3563	8.7178	8.6615	46.9355	47.8126
SP4	ELK3_1	cluster_2	DP	5565	7151.383	6857	7407	171.6495	0.7782	-0.3618	0.001	0.0011429	6270	56640	26.22	3057	27373	12.671	618	5565	2.5761	9.8565	9.8252	20.2159	20.3303
SP4	TBX1_4	cluster_2	DP	15147	19465.111	18980	19937	281.3121	0.7782	-0.3618	0.001	0.0010909	8929	170354	58.182	1881	34050	11.629	804	15147	5.1732	9.0044	8.8915	42.7432	44.4846
DN	ETV3_1	cluster_1	SP8	2298	2953.555	2771	3140	109.4742	0.7781	-0.3619	0.001	0.0010435	5350	48305	25.577	1310	11627	6.1563	256	2298	1.2168	4.785	4.7573	19.542	19.7643
DP	KLF12_1	cluster_5	Common_1_DN	19955	25655.661	25352	25959	179.7191	0.7778	-0.3625	0.001	0.001	19413	116478	59.073	7375	43670	22.148	3338	19955	10.12	17.1947	17.132	45.261	45.695
SP8	SOX14_3	cluster_5	Common_1_DN	6867	8828.949	8568	9101	154.7099	0.7778	-0.3625	0.001	0.0010909	3228	45250	55.339	1254	16454	20.123	507	6867	8.3982	15.7063	15.1757	40.4306	41.7345
SP8	E2F2_2	cluster_5	Common_1_DN	8173	10511.191	10234	10772	169.1367	0.7776	-0.3629	0.001	0.0010909	3179	54200	66.243	1050	16482	20.144	499	8173	9.989	15.6968	15.0793	47.5238	49.5874
SP8	TBX5_5	cluster_5	Common_1_DN	18386	23644.023	23112	24180	311.5828	0.7776	-0.3629	0.001	0.0010435	5748	109382	56.634	2353	42538	22.025	978	18386	9.5196	17.0146	16.809	41.564	43.2225
SP4	ARID5B_1	cluster_2	DP	4405	5666.459	5472	5875	123.907	0.7774	-0.3632	0.001	0.0010909	4639	60416	59.556	781	9708	9.5698	347	4405	4.3423	7.4801	7.2911	44.4302	45.3749
SP8	BATF_known1	cluster_2	DP	3339	4296.59	4116	4479	112.9717	0.7772	-0.3637	0.001	0.0010435	3843	50078	57.839	592	7502	8.6647	258	3339	3.8565	6.7135	6.6676	43.5811	44.5081
SP8	TBX19_1	cluster_5	Common_1_DN	11020	14179.945	13773	14610	244.6984	0.7772	-0.3637	0.001	0.0010435	3619	68942	53.926	1515	27072	21.176	590	11020	8.6198	16.3028	15.9845	38.9439	40.7063
SP8	RBPJ_1	cluster_5	Common_1_DN	14378	18504.812	18244	18755	162.3647	0.777	-0.364	0.001	0.0010435	10911	76393	53.125	5146	35199	24.478	2072	14378	9.9987	18.99	18.8211	40.2643	40.8478
SP8	SMAD3_1	cluster_2	DP	14172	18238.875	17917	18579	197.1355	0.777	-0.364	0.001	0.0010435	19958	165967	56.427	4021	32716	11.123	1729	14172	4.8183	8.6632	8.539	42.9993	43.3183
DP	SMAD4_1	cluster_5	Common_1_DN	61542	79209.061	78537	79983	460.1964	0.777	-0.3641	0.001	0.001	25085	356038	57.788	10079	138396	22.463	4372	61542	9.9888	17.4287	17.2852	43.3773	44.468
SP4	ELK3_2	cluster_2	DP	5419	6974.752	6664	7270	172.6974	0.777	-0.3641	0.001	0.0011429	6107	55145	26.227	2993	26781	12.737	603	5419	2.5772	9.8739	9.8268	20.147	20.2345
SP8	RREB1_2	cluster_5	Common_1_DN	194883	250834.388	248946	252492	1083.5243	0.7769	-0.3641	0.001	0.001	51915	1257959	63.21	18832	415372	20.871	8324	194883	9.7924	16.0339	15.492	44.2014	46.9177
SP8	HES1_1	cluster_4	SP4	14194	18271.387	17898	18633	218.9854	0.7769	-0.3643	0.001	0.001	25160	356406	63.862	2115	28979	5.1926	1011	14194	2.5433	4.0183	3.9825	47.8014	48.9803
SP8	MAF_known10	cluster_2	DP	8103	10431.42	10170	10677	155.7077	0.7768	-0.3644	0.001	0.0010909	8275	91208	55.774	1769	18966	11.598	739	8103	4.9551	8.9305	8.8841	41.775	42.7238
DN	ETV6_2	cluster_2	DP	8767	11287.452	10955	11599	213.8494	0.7767	-0.3645	0.001	0.0011429	10518	94973	25.655	4960	44381	11.989	976	8767	2.3683	9.2793	9.231	19.6774	19.7539
SP4	SOX15_4	cluster_5	Common_1_DN	4794	6172.208	5938	6392	130.1839	0.7767	-0.3645	0.001	0.0011429	2335	32728	59.319	840	10927	19.805	360	4794	8.689	15.4176	14.648	42.8571	43.873
SP8	ARID5B_1	cluster_5	Common_1_DN	9612	12375.288	12126	12649	165.7022	0.7767	-0.3645	0.001	0.0010909	4547	59233	58.39	1743	21717	21.408	753	9612	9.4752	16.5604	16.2274	43.2014	44.2603
SP8	SOX_1	cluster_5	Common_1_DN	12317	15862.449	15535	16218	203.5052	0.7765	-0.3649	0.001	0.0010435	6625	79556	57.773	2422	27958	20.303	1037	12317	8.9445	15.6528	15.4822	42.8159	44.0554
SP4	TBX5_3	cluster_2	DP	13797	17770.524	17443	18082	198.0308	0.7764	-0.3651	0.001	0.001	15391	138670	55.98	3634	32120	12.967	1540	13797	5.5697	10.0058	9.9495	42.3775	42.9545
DP	TCF7L2_known7	cluster_3	DN	15969	20574.773	20080	21033	291.7788	0.7762	-0.3656	0.001	0.0010435	10010	140599	49.374	3148	42486	14.92	1154	15969	5.6078	11.5285	11.3578	36.6582	37.5865
SP4	TBX21_6	cluster_2	DP	12424	16008.269	15602	16451	255.8673	0.7761	-0.3657	0.001	0.0010909	8330	150313	59.065	1600	27516	10.812	699	12424	4.882	8.3914	8.2654	43.6875	45.1519
SP8	POU2F2_known1	cluster_5	Common_1_DN	8835	11389.354	11072	11742	201.7975	0.7757	-0.3663	0.001	0.0010435	3160	57168	59.186	1186	19829	20.529	506	8835	9.1468	16.0127	15.4545	42.6644	44.556
SP4	CBX5_1	cluster_5	Common_1_DN	7408	9551.024	9334	9780	136.2342	0.7756	-0.3665	0.001	0.0010435	4490	49467	65.073	1439	15113	19.881	686	7408	9.7451	15.2784	14.9756	47.672	49.0174
SP8	SOX1_2	cluster_5	Common_1_DN	6627	8544.581	8263	8799	160.0619	0.7756	-0.3666	0.001	0.0010909	3221	45113	56.297	1185	15579	19.441	489	6627	8.2699	15.1816	14.6898	41.2658	42.538
SP4	SPIB_1	cluster_2	DP	8556	11032.442	10840	11228	113.5085	0.7756	-0.3667	0.001	0.0010435	18137	108822	58.346	3185	18963	10.167	1431	8556	4.5874	7.8899	7.8624	44.9294	45.1194
SP8	BPTF_1	cluster_2	DP	12006	15480.323	15111	15854	213.0531	0.7756	-0.3667	0.001	0.0010909	12575	168522	60.074	2078	26531	9.4577	919	12006	4.2799	7.3082	7.1243	44.2252	45.2527
SP4	SOX17_5	cluster_2	DP	2311	2980.738	2825	3123	94.0168	0.7754	-0.367	0.001	0.0011429	2416	33940	61.564	381	5012	9.0912	172	2311	4.1919	7.1192	6.8091	45.1444	46.1093
SP8	POU2F3_2	cluster_2	DP	2003	2583.536	2474	2695	67.1336	0.7754	-0.367	0.001	0.0010909	3384	27131	59.282	567	4389	9.5901	252	2003	4.3766	7.4468	7.3827	44.4444	45.6368
SP8	SOX8_9	cluster_5	Common_1_DN	5970	7699.454	7447	7935	158.5179	0.7754	-0.367	0.001	0.0010909	2766	44281	57.488	940	13886	18.028	381	5970	7.7506	13.7744	13.4821	40.5319	42.9929
SP8	TCF7L2_known7	cluster_5	Common_1_DN	24014	30969.696	30461	31542	323.7233	0.7754	-0.367	0.001	0.0010435	11335	159258	55.926	4202	56378	19.798	1745	24014	8.4329	15.3948	15.0787	41.5278	42.5946
SP8	GATA_known16	cluster_5	Common_1_DN	14847	19153.54	18668	19608	282.5707	0.7752	-0.3674	0.001	0.0010435	4509	95037	55.067	1823	35888	20.795	721	14847	8.6028	15.9902	15.6223	39.5502	41.3704
SP4	SOX5_1	cluster_5	Common_1_DN	3033	3913.258	3782	4049	82.501	0.7751	-0.3675	0.001	0.0010909	2386	21481	63.838	715	6233	18.524	339	3033	9.0136	14.2079	14.1195	47.4126	48.6604
DP	SP1_known3	cluster_5	Common_1_DN	82001	105813.625	105046	106551	456.6339	0.775	-0.3678	0.001	0.001	39534	518521	65.051	13072	164835	20.679	6326	82001	10.287	16.0014	15.8144	48.3935	49.7473
DP	TCF7_1	cluster_3	DN	17622	22741.386	22230	23273	309.0171	0.7749	-0.3679	0.001	0.0010435	9326	149704	48.954	3079	47321	15.474	1110	17622	5.7624	11.9022	11.7712	36.0507	37.2393
SP4	MYBL2_4	cluster_5	Common_1_DN	3564	4600.248	4445	4755	96.5635	0.7748	-0.3681	0.001	0.0010435	1678	23553	66.504	524	7050	19.906	258	3564	10.063	15.3754	15.1318	49.2366	50.5532
SP4	RUNX1_2	cluster_2	DP	4757	6139.878	5898	6376	145.7675	0.7748	-0.3681	0.001	0.0010435	4574	71425	63.314	694	10049	8.9078	314	4757	4.2168	6.8649	6.6601	45.245	47.338
SP4	SOX30_1	cluster_5	Common_1_DN	8551	11037.201	10766	11303	160.0367	0.7748	-0.3682	0.001	0.0010435	3742	56229	63.32	1285	18059	20.336	586	8551	9.6293	15.6601	15.2075	45.6031	47.3504
SP8	IRF4_2	cluster_5	Common_1_DN	14080	18179.9	17857	18507	200.6475	0.7745	-0.3687	0.001	0.001	6695	96589	66.045	2042	28028	19.165	996	14080	9.6275	14.8768	14.5772	48.7757	50.2355
SP8	TCF4_2	cluster_5	Common_1_DN	49656	64133.267	63454	64755	380.4355	0.7743	-0.3691	0.001	0.001	28489	257334	47.872	15187	134709	25.06	5523	49656	9.2375	19.3864	19.2963	36.3666	36.8617
SP8	TCF7_2	cluster_2	DP	10807	13960.359	13668	14279	188.2548	0.7741	-0.3693	0.001	0.0010435	11953	131925	55.753	2353	25359	10.717	983	10807	4.5671	8.2239	8.1918	41.7765	42.616
SP4	SMAD3_3	cluster_2	DP	2703	3492.082	3350	3630	80.3977	0.7741	-0.3694	0.001	0.0010435	3934	35416	61.507	650	5760	10.003	301	2703	4.6943	7.6512	7.6321	46.3077	46.9271
SP8	TBX15_1	cluster_2	DP	8772	11332.034	10927	11702	227.9247	0.7741	-0.3694	0.001	0.0010909	5892	106171	56.155	1217	20690	10.943	495	8772	4.6396	8.4012	8.2621	40.6738	42.3973
SP8	MAFG_1	cluster_5	Common_1_DN	20940	27061.033	26474	27608	340.6542	0.7738	-0.3699	0.001	0.0010435	6025	120737	52.864	2762	52068	22.798	1062	20940	9.1684	17.6266	17.3435	38.4504	40.2166
SP4	TBX20_5	cluster_2	DP	17440	22542.742	22129	22995	274.7196	0.7737	-0.3702	0.001	0.0010909	13251	203127	60.183	2625	38253	11.334	1153	17440	5.1672	8.7012	8.5858	43.9238	45.5912
SP8	SOX15_4	cluster_5	Common_1_DN	4529	5857.535	5649	6100	136.387	0.7732	-0.371	0.001	0.0011429	2193	30727	55.692	840	10927	19.805	336	4529	8.2087	15.3215	14.7395	40	41.4478
SP4	PBX1_2	cluster_2	DP	2655	3434.242	3268	3617	104.357	0.7732	-0.3712	0.001	0.0010435	3037	43176	61.498	430	5750	8.1901	191	2655	3.7817	6.2891	6.1492	44.4186	46.1739
SP4	SOX1_2	cluster_5	Common_1_DN	6987	9037.746	8765	9276	152.2605	0.7731	-0.3712	0.001	0.0010909	3418	47871	59.739	1185	15579	19.441	514	6987	8.7191	15.038	14.5955	43.3755	44.8488
DN	ETV4_2	cluster_1	SP8	1869	2418.882	2259	2590	100.5884	0.7728	-0.3719	0.001	0.0011429	5010	45275	26.285	1051	9352	5.4294	208	1869	1.0851	4.1517	4.1281	19.7907	19.985
SP4	SOX7_3	cluster_5	Common_1_DN	7982	10329.863	10042	10614	175.0168	0.7727	-0.372	0.001	0.0010909	3572	57193	62.928	1166	17179	18.902	528	7982	8.7824	14.7816	13.9563	45.283	46.4637
DP	ETV6_2	cluster_1	SP8	4653	6024.809	5736	6290	171.3658	0.7723	-0.3727	0.001	0.0011429	9680	87477	23.63	2882	25678	6.9365	517	4653	1.2569	5.3409	5.3191	17.9389	18.1206
DN	ELK3_2	cluster_2	DP	5387	6975.548	6714	7250	159.012	0.7723	-0.3728	0.001	0.0011429	6130	55327	26.313	2993	26781	12.737	600	5387	2.562	9.7879	9.7367	20.0468	20.115
SP8	TCF7_1	cluster_5	Common_1_DN	25906	33545.866	33041	34032	300.4087	0.7723	-0.3728	0.001	0.0010435	10694	171757	56.165	4000	61044	19.962	1639	25906	8.4713	15.3264	15.0829	40.975	42.4382
SP8	ATF4_3	cluster_5	Common_1_DN	7732	10013.727	9772	10286	154.7793	0.7722	-0.373	0.001	0.0010909	3790	49324	56.677	1433	17948	20.624	601	7732	8.8847	15.8575	15.6759	41.94	43.08
SP4	SOX5_3	cluster_2	DP	2513	3255.565	3102	3410	95.8178	0.772	-0.3734	0.001	0.0011429	2611	39250	66.643	366	5129	8.7086	173	2513	4.2668	6.6258	6.4025	47.2678	48.9959
SP4	ID4_1	cluster_5	Common_1_DN	53995	69952.671	69343	70602	381.3727	0.7719	-0.3735	0.001	0.0010435	30226	273311	46.788	16901	150382	25.744	6002	53995	9.2433	19.8571	19.7559	35.5127	35.9052
DP	BATF_known1	cluster_5	Common_1_DN	7247	9389.762	9134	9677	171.9806	0.7718	-0.3737	0.001	0.0010435	3507	45701	52.784	1417	17961	20.745	561	7247	8.3702	15.9966	15.8574	39.5907	40.3485
SP4	SOX1_3	cluster_2	DP	3092	4006.824	3794	4205	123.1014	0.7717	-0.3738	0.001	0.0010909	3151	44155	58.678	533	7047	9.3648	227	3092	4.109	7.2041	7.0026	42.5891	43.8768
SP8	IKZF3_1	cluster_5	Common_1_DN	13374	17331.244	16988	17637	198.9185	0.7717	-0.3739	0.001	0.0010435	6473	77854	57.71	2666	30716	22.768	1131	13374	9.9136	17.4726	17.1783	42.4231	43.5408
DP	TCF21_1	cluster_5	Common_1_DN	28634	37111.558	36630	37640	311.5025	0.7716	-0.3741	0.001	0.001	11430	148923	52.886	5599	70697	25.106	2217	28634	10.169	19.3963	19.2274	39.5964	40.5024
SP8	SPDEF_4	cluster_2	DP	7885	10219.435	9881	10533	191.1152	0.7716	-0.3741	0.001	0.0010435	7014	105694	58.627	1256	17937	9.9495	541	7885	4.3737	7.7131	7.4602	43.0732	43.9594
SP8	TBX15_2	cluster_2	DP	9848	12765.087	12517	13024	151.2698	0.7715	-0.3743	0.001	0.0010909	14966	104782	54.532	3419	23567	12.265	1412	9848	5.1253	9.4347	9.3986	41.2986	41.7872
SP8	POU2F2_known11	cluster_2	DP	3302	4280.791	4124	4432	94.6224	0.7714	-0.3744	0.001	0.0010435	4428	44372	59.446	762	7325	9.8134	331	3302	4.4237	7.4752	7.4416	43.4383	45.0785
SP4	TBX4_1	cluster_2	DP	7899	10240.465	10033	10475	133.7955	0.7714	-0.3745	0.001	0.0010435	11905	83358	55.556	2687	18553	12.365	1134	7899	5.2645	9.5254	9.476	42.2032	42.5753
SP4	TBX1_2	cluster_5	Common_1_DN	14888	19303.384	18849	19736	270.9417	0.7713	-0.3747	0.001	0.0010909	4990	89894	57.636	2030	34408	22.061	848	14888	9.5455	16.994	16.5617	41.7734	43.269
SP8	GMEB1_1	cluster_4	SP4	2538	3290.927	3154	3428	84.7301	0.7713	-0.3747	0.001	0.0010435	6106	99511	72.498	294	4624	3.3688	158	2538	1.849	2.5876	2.5505	53.7415	54.8875
SP8	MYBL1_3	cluster_5	Common_1_DN	2582	3347.931	3222	3485	78.5843	0.7713	-0.3747	0.001	0.0010909	1462	16095	60.713	534	5618	21.192	238	2582	9.7397	16.2791	16.0422	44.5693	45.9594
SP4	SOX3_3	cluster_2	DP	3267	4236.625	4047	4432	116.7597	0.7712	-0.3748	0.001	0.0010909	2900	46500	59.883	486	7333	9.4434	210	3267	4.2072	7.2414	7.0258	43.2099	44.552
SP4	FOXO3_1	cluster_5	Common_1_DN	9646	12508.778	12267	12778	153.369	0.7712	-0.3749	0.001	0.0010435	5633	74003	67.715	1584	19294	17.655	757	9646	8.8264	13.4387	13.0346	47.7904	49.9948
DP	TCF7L1_1	cluster_3	DN	15815	20511.824	20055	21011	285.6807	0.771	-0.3751	0.001	0.0010435	8494	136288	48.648	2782	42859	15.298	998	15815	5.6451	11.7495	11.6041	35.8735	36.9001
SP8	BPTF_1	cluster_5	Common_1_DN	27097	35151.258	34662	35676	305.5408	0.7709	-0.3754	0.001	0.0010909	12575	168522	60.074	4683	60087	21.42	2057	27097	9.6595	16.3579	16.0792	43.9248	45.0963
SP8	SMAD3_1	cluster_5	Common_1_DN	28684	37208.28	36782	37627	244.2681	0.7709	-0.3754	0.001	0.0010435	19958	165967	56.427	8111	66435	22.587	3453	28684	9.7522	17.3013	17.283	42.5718	43.176
DP	TCF7_2	cluster_3	DN	13444	17442.835	17080	17815	217.083	0.7708	-0.3756	0.001	0.0010435	10707	118115	49.917	3302	35484	14.996	1228	13444	5.6816	11.4691	11.3821	37.1896	37.8875
SP8	CUX1_8	cluster_2	DP	410	532.229	465	603	39.877	0.7708	-0.3756	0.001	0.0011429	544	8813	66.353	61	863	6.4975	27	410	3.0869	4.9632	4.6522	44.2623	47.5087
SP8	TCF7_2	cluster_5	Common_1_DN	20343	26392.092	25988	26832	239.8318	0.7708	-0.3756	0.001	0.0010435	11953	131925	55.753	4487	48020	20.294	1866	20343	8.5971	15.6111	15.4201	41.5868	42.3636
SP8	SOX9_6	cluster_5	Common_1_DN	9582	12434.619	12111	12737	195.6169	0.7706	-0.3759	0.001	0.0010909	4230	63611	57.612	1562	22247	20.149	653	9582	8.6783	15.4374	15.0634	41.8054	43.071
DP	TCF12_known1	cluster_5	Common_1_DN	49380	64107.556	63644	64628	302.0032	0.7703	-0.3766	0.001	0.0010909	36358	261819	56.235	16034	114471	24.587	6860	49380	10.606	18.8679	18.8604	42.7841	43.1376
DP	TCF7L2_known5	cluster_3	DN	18228	23667.159	23169	24228	322.1761	0.7702	-0.3767	0.001	0.0010435	9748	156401	49.931	3117	48148	15.371	1147	18228	5.8193	11.7665	11.6547	36.7982	37.8583
DP	STAT_known3	cluster_5	Common_1_DN	20779	26980.271	26509	27430	288.8092	0.7702	-0.3768	0.001	0.0010435	6852	137400	63.695	2289	43138	19.998	1056	20779	9.6326	15.4116	15.123	46.1337	48.1687
SP8	TCF7L1_1	cluster_5	Common_1_DN	23475	30495.192	29939	31053	330.4408	0.7698	-0.3774	0.001	0.0010435	9778	157054	56.06	3640	55600	19.846	1487	23475	8.3794	15.2076	14.9471	40.8516	42.2212
SP4	SOX4_2	cluster_2	DP	5236	6802.276	6575	7028	142.8713	0.7698	-0.3775	0.001	0.0010909	4816	72432	63.066	791	11178	9.7326	353	5236	4.559	7.3297	7.2288	44.6271	46.842
SP8	MAF_known11	cluster_5	Common_1_DN	29416	38214.892	37641	38746	341.3116	0.7698	-0.3775	0.001	0.0010435	10315	166406	54.325	4559	70796	23.112	1812	29416	9.6031	17.5667	17.6772	39.7456	41.5504
SP4	HBP1_1	cluster_5	Common_1_DN	10307	13393.726	13051	13706	184.4633	0.7696	-0.3779	0.001	0.0010435	4429	68253	63.399	1516	21821	20.269	689	10307	9.5739	15.5566	15.1012	45.4485	47.2343
SP8	POU2F2_known16	cluster_5	Common_1_DN	4867	6324.82	6106	6530	133.2915	0.7695	-0.3779	0.001	0.0010909	2431	32048	54.51	965	11945	20.317	376	4867	8.2782	15.4669	15.1866	38.9637	40.7451
SP4	IKZF1_2	cluster_5	Common_1_DN	28460	36984.778	36547	37393	237.8826	0.7695	-0.378	0.001	0.0010435	20507	164063	59.864	8047	62565	22.829	3592	28460	10.385	17.516	17.347	44.6378	45.4887
SP4	FOXO1_4	cluster_5	Common_1_DN	4346	5648.915	5446	5848	121.7371	0.7694	-0.3782	0.001	0.0010909	2354	30679	61.609	766	9486	19.05	342	4346	8.7276	14.5285	14.166	44.6475	45.8149
SP4	TBX20_5	cluster_5	Common_1_DN	32844	42689.482	42112	43291	358.5131	0.7694	-0.3782	0.001	0.0010909	13251	203127	60.183	5012	72644	21.523	2178	32844	9.7312	16.4365	16.1692	43.4557	45.2123
DN	SPI1_known1	cluster_1	SP8	6735	8754.66	8488	9054	165.4795	0.7693	-0.3783	0.001	0.001	15535	109231	23.39	5460	37816	8.0978	964	6735	1.4422	6.2053	6.1658	17.6557	17.8099
SP8	SP100_1	cluster_5	Common_1_DN	1631	2120.331	2025	2232	60.955	0.7693	-0.3783	0.001	0.0013333	956	12440	73.99	242	2948	17.534	128	1631	9.7008	13.3891	13.1109	52.8926	55.3256
SP8	HIF1A_1	cluster_4	SP4	5528	7188.764	6989	7388	119.9008	0.769	-0.3789	0.001	0.0010435	11568	130194	67.952	980	10689	5.5789	499	5528	2.8852	4.3136	4.246	50.9184	51.7167
SP8	SOX14_3	cluster_2	DP	3236	4209.059	4003	4395	124.1218	0.7689	-0.3792	0.001	0.0010909	3228	45250	55.339	592	7830	9.5759	235	3236	3.9575	7.28	7.1514	39.6959	41.3282
SP8	MESP1_1	cluster_5	Common_1_DN	49012	63749.372	63124	64352	379.1151	0.7688	-0.3793	0.001	0.001	28229	254858	48.512	14895	132166	25.158	5451	49012	9.3294	19.3099	19.2311	36.5962	37.0837
DN	ETV3_1	cluster_2	DP	4639	6035.623	5778	6289	149.4398	0.7686	-0.3796	0.001	0.0010435	5350	48305	25.577	2666	23782	12.592	516	4639	2.4563	9.6449	9.6036	19.3548	19.5063
DP	NFE2L1::MAFG_1	cluster_3	DN	23042	29979.614	29418	30601	389.1722	0.7686	-0.3797	0.001	0.0010435	8197	173116	53.265	2840	57401	17.661	1101	23042	7.0896	13.4317	13.3102	38.7676	40.1422
SP8	SPIB_1	cluster_5	Common_1_DN	17102	22258.12	21944	22572	186.9634	0.7684	-0.3801	0.001	0.0010435	16443	98658	52.897	7127	42285	22.672	2863	17102	9.1694	17.4117	17.3346	40.1712	40.4446
DP	MTF1_1	cluster_5	Common_1_DN	86944	113178.58	112402	113965	469.4147	0.7682	-0.3804	0.001	0.001	40601	541813	68.67	12833	166540	21.107	6574	86944	11.019	16.1917	16.0469	51.2273	52.2061
SP4	ATF6_1	cluster_2	DP	1799	2342.335	2246	2455	63.0259	0.7681	-0.3806	0.001	0.0010435	3915	27415	63.572	534	3697	8.573	257	1799	4.1717	6.5645	6.5621	48.1273	48.6611
SP8	SMAD_1	cluster_2	DP	10376	13508.468	13245	13747	144.983	0.7681	-0.3806	0.001	0.0010435	15801	126448	57.942	3011	23523	10.779	1304	10376	4.7546	8.2526	8.2057	43.3079	44.11
SP8	FOXP3_1	cluster_5	Common_1_DN	20087	26156.637	25705	26719	302.799	0.768	-0.3809	0.001	0.0010909	7647	122767	55.431	3180	48216	21.77	1278	20087	9.0696	16.7124	16.3619	40.1887	41.6604
SP8	NFATC1_1	cluster_5	Common_1_DN	14790	19263.233	18840	19738	277.3621	0.7678	-0.3812	0.001	0.0010909	5066	97783	60.876	1853	32944	20.51	799	14790	9.2077	15.7718	15.1253	43.1193	44.8944
SP4	E2F_known26	cluster_2	DP	1831	2385.512	2257	2525	83.7987	0.7676	-0.3815	0.001	0.0010435	1689	28742	66.459	237	3703	8.5622	110	1831	4.2337	6.5127	6.3705	46.4135	49.4464
SP8	POU2F2_known5	cluster_5	Common_1_DN	7642	9955.485	9637	10239	168.1376	0.7676	-0.3815	0.001	0.0010909	3679	47888	58.57	1410	17442	21.333	601	7642	9.3466	16.336	15.9581	42.6241	43.8138
SP8	NFATC1_1	cluster_2	DP	6013	7834.45	7571	8123	172.7805	0.7675	-0.3817	0.001	0.0010909	5066	97783	60.876	757	13426	8.3585	319	6013	3.7435	6.2969	6.1493	42.14	44.7862

SP8	KLF13_1	cluster_4	SP4	16733	21803.194	21401	22240	241.6833	0.7675	-0.3818	0.001	0.001	20972	360883	65.363	2092	34137	6.1829	990	16733	3.0307	4.7206	4.6367	47.3231	49.0172
SP4	SOX_1	cluster_5	Common_1_DN	13114	17088.082	16804	17416	181.3877	0.7674	-0.3819	0.001	0.0010435	7156	85938	62.408	2422	27958	20.303	1111	13114	9.5233	15.5254	15.2598	45.8712	46.9061
SP4	SOX1_3	cluster_5	Common_1_DN	6755	8802.599	8534	9085	160.3324	0.7674	-0.3819	0.001	0.0010909	3151	44155	58.678	1186	15495	20.591	501	6755	8.9767	15.8997	15.2984	42.2428	43.5947
SP4	SOX11_1	cluster_5	Common_1_DN	27410	35716.94	35153	36287	347.4583	0.7674	-0.3819	0.001	0.0010435	11736	190304	65.17	3781	56921	19.493	1749	27410	9.3866	14.9029	14.4033	46.2576	48.1545
SP4	FOXM1_1	cluster_5	Common_1_DN	6965	9076.642	8899	9270	117.5573	0.7674	-0.382	0.001	0.0010909	5181	41487	58.448	2009	15722	22.15	877	6965	9.8125	16.9272	16.7884	43.6536	44.301
DP	MYC_known13	cluster_5	Common_1_DN	17209	22427.016	22065	22806	234.2173	0.7673	-0.3821	0.001	0.0010909	9190	101216	56.829	3715	39745	22.315	1576	17209	9.6622	17.1491	17.0023	42.4226	43.2985
SP8	TBX21_6	cluster_5	Common_1_DN	23903	31152.132	30567	31727	339.4013	0.7673	-0.3821	0.001	0.0010909	8068	145632	57.226	3242	55459	21.793	1340	23903	9.3927	16.6088	16.4133	41.3325	43.1003
SP4	SOX17_4	cluster_5	Common_1_DN	5583	7278.254	7063	7500	132.9016	0.7671	-0.3825	0.001	0.0010909	2822	39514	62.402	919	12059	19.044	413	5583	8.8168	14.635	14.1292	44.9402	46.2974
DN	ELK3_1	cluster_2	DP	5526	7204.828	6975	7452	151.125	0.767	-0.3827	0.001	0.0011429	6350	57329	26.539	3057	27373	12.671	615	5526	2.5581	9.685	9.6391	20.1178	20.1878
SP8	NFE2L1::MAFG_1	cluster_5	Common_1_DN	30052	39190.093	38425	39860	419.3529	0.7668	-0.383	0.001	0.0010435	8430	177761	54.694	3722	73540	22.627	1451	30052	9.2465	17.2123	16.9058	38.9844	40.8648
SP8	PBX3_known1	cluster_2	DP	5618	7327.253	7120	7547	127.1563	0.7668	-0.3832	0.001	0.0010435	6890	76602	56.96	1236	13124	9.7589	514	5618	4.1775	7.4601	7.334	41.5858	42.8071
SP8	POU2F2_known14	cluster_5	Common_1_DN	2198	2868.908	2746	3009	85.6672	0.7662	-0.3842	0.001	0.0012632	928	14244	63.304	353	4849	21.55	154	2198	9.7685	16.5948	15.4311	43.6261	45.3289
SP8	XBP1_2	cluster_5	Common_1_DN	9948	12985.358	12584	13373	241.9522	0.7661	-0.3844	0.001	0.0010435	2655	61164	63.418	971	20992	21.766	441	9948	10.315	16.6102	16.2645	45.4171	47.3895
SP4	IRF4_2	cluster_5	Common_1_DN	14226	18572.614	18282	18922	191.0601	0.766	-0.3846	0.001	0.001	6862	99125	67.779	2042	28028	19.165	1001	14226	9.7274	14.5876	14.3516	49.0206	50.7564
SP4	TBX5_5	cluster_2	DP	9856	12869.004	12521	13226	217.3105	0.7659	-0.3848	0.001	0.0010435	5992	114030	59.04	1223	22231	11.51	524	9856	5.1031	8.745	8.6433	42.8455	44.3345
SP8	TBX4_2	cluster_2	DP	11137	14543.467	14073	14974	262.3246	0.7658	-0.385	0.001	0.0010909	6718	127933	57.269	1423	25905	11.596	595	11137	4.9854	8.8568	8.7053	41.8131	42.9917
SP4	TCF3_2	cluster_5	Common_1_DN	69310	90536.052	89669	91479	582.8769	0.7656	-0.3854	0.001	0.0010435	23468	354325	46.199	13449	197852	25.797	4627	69310	9.0371	19.7162	19.5611	34.404	35.0312
SP8	IRF_known14	cluster_5	Common_1_DN	41692	54479.802	53709	55238	457.844	0.7653	-0.3859	0.001	0.001	13279	278624	63.239	4593	89191	20.244	2047	41692	9.4628	15.4153	14.9635	44.5678	46.7446
SP4	FOXO1_4	cluster_2	DP	2256	2948.931	2796	3096	90.7804	0.7651	-0.3863	0.001	0.0010909	2354	30679	61.609	405	4964	9.9687	177	2256	4.5305	7.5191	7.3536	43.7037	45.4472
DP	SPDEF_6	cluster_5	Common_1_DN	18537	24232.438	23908	24526	193.7466	0.765	-0.3865	0.001	0.001	8667	121736	70.334	2581	34933	20.183	1335	18537	10.71	15.4033	15.2272	51.7241	53.0644
SP4	RBPJ_1	cluster_5	Common_1_DN	14633	19128.325	18863	19409	168.7394	0.765	-0.3865	0.001	0.0010435	11294	79074	54.99	5146	35199	24.478	2110	14633	10.176	18.6825	18.5055	41.0027	41.5722
SP8	HMGA1_2	cluster_2	DP	4676	6114.791	5879	6348	135.9246	0.7647	-0.387	0.001	0.0010435	5565	78050	63.498	772	10035	8.164	343	4676	3.8042	6.1635	5.991	44.4301	46.5969
SP4	POU2F2_known11	cluster_5	Common_1_DN	7009	9167.297	8948	9391	140.7101	0.7646	-0.3872	0.001	0.0010435	4718	47310	63.382	1531	14781	19.802	712	7009	9.39	15.0911	14.815	46.5056	47.419
SP8	SOX2_5	cluster_5	Common_1_DN	7369	9637.897	9327	9951	176.2384	0.7646	-0.3872	0.001	0.0010909	3178	50901	57.693	1164	17280	19.586	481	7369	8.3522	15.1353	14.4771	41.323	42.6447
SP8	TCF7L1_2	cluster_5	Common_1_DN	19718	25790.587	25408	26152	233.6629	0.7646	-0.3873	0.001	0.0010435	11685	129185	56.684	4326	46203	20.273	1804	19718	8.6519	15.4386	15.2634	41.7013	42.6769
SP4	PBX3_known1	cluster_5	Common_1_DN	13085	17115.685	16808	17424	189.0704	0.7645	-0.3874	0.001	0.0010435	7076	78591	58.439	2777	29759	22.128	1187	13085	9.7299	16.775	16.6495	42.744	43.9699
SP4	TBX19_1	cluster_5	Common_1_DN	11465	14999.548	14625	15404	235.603	0.7644	-0.3877	0.001	0.0010435	3815	72708	56.872	1515	27072	21.176	611	11465	8.9679	16.0157	15.7686	40.33	42.35
SP8	SMAD_2	cluster_2	DP	18935	24773.312	24386	25168	232.6707	0.7643	-0.3877	0.001	0.0010435	21409	221606	55.683	4479	45031	11.315	1858	18935	4.7578	8.6786	8.5444	41.4825	42.0488
SP8	TBX2_1	cluster_5	Common_1_DN	24160	31610.776	31043	32132	322.5597	0.7643	-0.3878	0.001	0.0010435	8536	145462	59.643	3274	53684	22.012	1431	24160	9.9062	16.7643	16.6091	43.708	45.0041
SP8	TBX2_2	cluster_5	Common_1_DN	14765	19318.485	18978	19657	201.8757	0.7643	-0.3878	0.001	0.0010909	9067	90775	56.983	3547	34386	21.586	1494	14765	9.2686	16.4773	16.2655	42.1201	42.939
SP4	SOX18_2	cluster_5	Common_1_DN	5512	7212.722	7009	7441	133.9004	0.7642	-0.3879	0.001	0.0010909	2930	41032	62.484	919	11986	18.252	412	5512	8.3937	14.0614	13.4334	44.8313	45.987
SP4	SOX11_2	cluster_2	DP	3695	4835.741	4631	5017	114.8848	0.7642	-0.3881	0.001	0.0010909	3896	54587	64.334	589	7774	9.1621	269	3695	4.3547	6.9045	6.769	45.6706	47.5302
DP	LMO2_1	cluster_5	Common_1_DN	88463	115777.827	114971	116649	484.0696	0.7641	-0.3882	0.001	0.001	40696	452099	54.045	19801	215266	25.733	8003	88463	10.575	19.6653	19.5672	40.4172	41.0947
SP4	FOXP1_1	cluster_2	DP	16491	21582.315	21067	22097	317.0729	0.7641	-0.3882	0.001	0.0010435	11002	256998	66.218	1880	37085	9.5554	773	16491	4.2491	7.026	6.4168	41.117	44.4681
SP8	TBX4_2	cluster_5	Common_1_DN	21771	28496.775	27967	29050	330.3631	0.764	-0.3884	0.001	0.0010909	6718	127933	57.269	2786	50614	22.657	1158	21771	9.7457	17.2373	17.0175	41.565	43.0138
DP	EGR1_known12	cluster_5	Common_1_DN	113043	147977.112	146945	148989	616.2003	0.7639	-0.3885	0.001	0.001	50191	833460	66.56	14575	227849	18.196	6953	113043	9.0276	13.8531	13.5631	47.705	49.6131
SP4	POU2F2_known12	cluster_5	Common_1_DN	6944	9090.818	8896	9289	121.0123	0.7639	-0.3886	0.001	0.0010435	4643	46514	63.727	1513	14607	20.012	708	6944	9.5136	15.2488	14.9288	46.7944	47.5389
SP4	SOX17_5	cluster_5	Common_1_DN	5014	6564.449	6349	6792	129.0034	0.7638	-0.3886	0.001	0.0011429	2416	33940	61.564	850	11148	20.221	372	5014	9.0949	15.3974	14.7731	43.7647	44.9767
SP4	SOX4_1	cluster_5	Common_1_DN	29671	38850.248	38301	39371	324.1392	0.7637	-0.3889	0.001	0.0010435	12657	205161	65.153	4095	61800	19.626	1890	29671	9.4227	14.9324	14.4623	46.1538	48.0113
SP4	TBX2_2	cluster_5	Common_1_DN	15115	19793.035	19471	20126	193.5634	0.7637	-0.389	0.001	0.0010909	9302	93136	58.465	3547	34386	21.586	1523	15115	9.4883	16.3728	16.229	42.9377	43.9568
SP8	E2F_known26	cluster_2	DP	1791	2345.786	2191	2483	86.9419	0.7636	-0.3891	0.001	0.0010435	1668	28373	65.605	237	3703	8.5622	108	1791	4.1412	6.4748	6.3123	45.5696	48.3662
SP8	PKNOX2_2	cluster_5	Common_1_DN	21340	27957.762	27483	28442	279.8441	0.7633	-0.3897	0.001	0.0010435	10799	119886	49.272	5291	57301	23.55	1939	21340	8.7705	17.9554	17.8002	36.6471	37.2419
SP4	MAF_known12	cluster_2	DP	11501	15069.133	14719	15448	228.8429	0.7632	-0.3898	0.001	0.0010435	7957	135675	61.511	1524	24862	11.272	684	11501	5.2142	8.5962	8.4769	44.8819	46.2594
SP8	CTCF_known1	cluster_5	Common_1_DN	192290	251965.207	250464	253305	852.3863	0.7632	-0.3899	0.001	0.0010435	57414	1061210	61.215	23232	415314	23.957	10446	192290	11.092	18.1942	18.1199	44.9638	46.2999
DP	ETV2_1	cluster_5	Common_1_DN	23941	31371.838	30919	31839	265.0346	0.7631	-0.39	0.001	0.0010435	15356	154289	52.346	6080	60068	20.38	2390	23941	8.1226	15.5639	15.517	39.3092	39.8565
SP8	EGR3_3	cluster_4	SP4	18971	24862.743	24485	25257	234.5357	0.763	-0.3902	0.001	0.001	32104	473266	72.424	2490	34990	5.3545	1322	18971	2.9031	4.1179	4.0085	53.0924	54.2183
SP8	PKNOX1_2	cluster_5	Common_1_DN	18301	23987.474	23623	24401	230.8618	0.7629	-0.3903	0.001	0.0010435	9433	104620	49.524	4531	48957	23.175	1666	18301	8.6631	17.6614	17.4928	36.7689	37.3818
SP4	FOXP1_1	cluster_5	Common_1_DN	29739	38984.818	38324	39799	422.5157	0.7628	-0.3905	0.001	0.0010435	11002	256998	66.218	3601	67880	17.49	1460	29739	7.6626	13.2703	11.5717	40.5443	43.8111
DP	PATZ1_1	cluster_5	Common_1_DN	139971	183492.23	182611	184471	581.8686	0.7628	-0.3906	0.001	0.001	72910	959184	65.12	23069	288137	19.562	10909	139971	9.5027	14.9623	14.5927	47.2886	48.5779
SP8	FOXO3_1	cluster_2	DP	5637	7390.691	7175	7611	133.76	0.7627	-0.3907	0.001	0.0010435	5381	70700	64.693	965	11860	10.852	440	5637	5.158	8.1769	7.9731	45.5959	47.5295
SP4	SMAD_1	cluster_2	DP	10512	13783.888	13565	14011	150.3534	0.7626	-0.3909	0.001	0.0010435	16152	129249	59.226	3011	23523	10.779	1324	10512	4.8169	8.1971	8.1331	43.9721	44.6882
SP8	TCF12_known1	cluster_5	Common_1_DN	46087	60429.305	59949	60938	293.4865	0.7627	-0.3909	0.001	0.0010909	34302	246920	53.035	16034	114471	24.587	6402	46087	9.8988	18.6636	18.6647	39.9277	40.2609
SP8	GMEB2_2	cluster_5	Common_1_DN	5456	7155.984	6950	7365	130.0607	0.7625	-0.3912	0.001	0.001	2724	36104	65.136	866	11140	20.098	411	5456	9.8432	15.0881	15.1119	47.4596	48.9767
SP8	FOXO6_1	cluster_2	DP	2659	3488.803	3303	3649	104.6354	0.7622	-0.3917	0.001	0.0010909	2530	32988	57.537	496	6186	10.789	207	2659	4.6377	8.1818	8.0605	41.7339	42.9842
SP4	ETV3_1	cluster_2	DP	12191	15996.741	15738	16259	153.4353	0.7621	-0.3919	0.001	0.0010435	14073	127252	67.378	2666	23782	12.592	1354	12191	6.4549	9.6213	9.5802	50.7877	51.2615
SP4	TBX19_1	cluster_2	DP	6006	7881.661	7584	8171	187.8838	0.7621	-0.392	0.001	0.0010435	3815	72708	56.872	770	14099	11.028	317	6006	4.6979	8.3093	8.2604	41.1688	42.5988
SP8	ETV6_2	cluster_2	DP	7509	9853.666	9532	10154	197.968	0.7621	-0.392	0.001	0.0011429	9151	82658	22.329	4960	44381	11.989	833	7509	2.0284	9.1028	9.0844	16.7944	16.9194
SP8	TBX5_3	cluster_5	Common_1_DN	22568	29616.298	29221	29979	227.9532	0.762	-0.3921	0.001	0.001	14898	134209	54.179	6304	55260	22.308	2528	22568	9.1105	16.9687	16.8156	40.1015	40.8397
SP8	SPIC_1	cluster_5	Common_1_DN	31811	41755.462	41191	42234	318.6943	0.7618	-0.3924	0.001	0.001	14831	193520	54.959	6133	77072	21.888	2468	31811	9.0341	16.6408	16.4381	40.2413	41.2744
SP8	TBX20_5	cluster_2	DP	16746	21981.691	21586	22395	250.3162	0.7618	-0.3925	0.001	0.0010909	12908	197715	58.58	2625	38253	11.334	1106	16746	4.9616	8.5683	8.4698	42.1333	43.777
SP4	SOX2_5	cluster_5	Common_1_DN	7759	10186.899	9928	10423	160.6702	0.7617	-0.3927	0.001	0.0010909	3385	54207	61.44	1164	17280	19.586	506	7759	8.7943	14.9483	14.3136	43.4708	44.9016
SP8	SMAD3_3	cluster_2	DP	2556	3356.048	3228	3493	81.0513	0.7617	-0.3927	0.001	0.0010435	3767	33918	58.906	650	5760	10.003	285	2556	4.439	7.5657	7.5358	43.8462	44.375
SP4	TBX20_2	cluster_5	Common_1_DN	16477	21639.765	21167	22113	281.3164	0.7614	-0.3932	0.001	0.0010435	5606	101105	58.153	2226	38036	21.877	930	16477	9.4771	16.5894	16.2969	41.779	43.3195
SP4	FOXO6_1	cluster_5	Common_1_DN	5027	6603.098	6382	6820	133.6283	0.7613	-0.3934	0.001	0.0010909	2688	35069	61.166	901	11153	19.453	393	5027	8.7679	14.6205	14.3346	43.6182	45.0731
SP8	TBX1_4	cluster_2	DP	14255	18728.015	18251	19239	287.7738	0.7612	-0.3937	0.001	0.0010909	8594	163899	55.977	1881	34050	11.629	758	14255	4.8686	8.8201	8.6974	40.2977	41.8649
SP8	MGA_3	cluster_5	Common_1_DN	13789	18117.265	17723	18495	245.6604	0.7611	-0.3938	0.001	0.0010909	4956	84386	59.559	1893	30937	21.835	820	13789	9.7322	16.5456	16.3404	43.3175	44.5712
SP8	PKNOX2_3	cluster_5	Common_1_DN	18402	24177.132	23771	24561	243.2013	0.7611	-0.3938	0.001	0.0010435	9661	107482	50.165	4498	48655	22.709	1670	18402	8.5888	17.286	17.121	37.1276	37.8214
DP	ETV1_1	cluster_5	Common_1_DN	6393	8402.961	8148	8689	179.4702	0.7608	-0.3944	0.001	0.0010909	5186	46934	26.831	3527	31478	17.995	709	6393	3.6547	13.6714	13.6213	20.1021	20.3094
SP8	PBX1_4	cluster_5	Common_1_DN	4730	6220.604	6029	6436	124.2925	0.7604	-0.3951	0.001	0.0010909	2771	31106	57.753	1046	11037	20.492	428	4730	8.782	15.4457	15.2061	40.9178	42.8558
SP4	BPTF_1	cluster_5	Common_1_DN	27258	35848.497	35380	36313	301.366	0.7604	-0.3952	0.001	0.0010909	12832	172114	61.355	4683	60087	21.42	2073	27258	9.7169	16.1549	15.8372	44.2665	45.3642
SP4	E2F2_2	cluster_5	Common_1_DN	8143	10709.708	10447	10986	164.1705	0.7604	-0.3952	0.001	0.0010909	3236	55175	67.435	1050	16482	20.144	491	8143	9.9523	15.1731	14.7585	46.7619	49.4054
SP4	SOX12_1	cluster_5	Common_1_DN	5627	7400.718	7206	7633	128.683	0.7604	-0.3952	0.001	0.0010435	3166	41308	65.459	975	11756	18.629	446	5627	8.9169	14.0872	13.6221	45.7436	47.8649
DN	ELK3_2	cluster_1	SP8	2450	3222.736	3022	3404	112.4456	0.7603	-0.3954	0.001	0.0011429	6130	55327	26.313	1388	12358	5.8774	273	2450	1.1652	4.4535	4.4282	19.6686	19.8252
SP8	MGA_2	cluster_5	Common_1_DN	14000	18415.805	17981	18838	261.3075	0.7602	-0.3955	0.001	0.0010909	5058	86090	56.587	2036	33173	21.805	839	14000	9.2022	16.5876	16.2621	41.2083	42.203
SP4	ETV1_1	cluster_2	DP	4131	5435.2	5208	5678	140.8711	0.7601	-0.3958	0.001	0.0010909	5001	45274	25.882	2353	21073	12.047	459	4131	2.3616	9.1782	9.1244	19.507	19.6033
SP8	TCF7L2_known7	cluster_2	DP	12839	16895.928	16536	17261	225.0001	0.7599	-0.3961	0.001	0.0010435	11335	159258	55.926	2272	30780	10.809	925	12839	4.5086	8.1606	8.0618	40.713	41.7122
DP	RUNX2_4	cluster_5	Common_1_DN	41105	54094.754	53414	54756	404.0335	0.7599	-0.3962	0.001	0.0010435	16438	251335	60.987	6052	89641	21.752	2713	41105	9.9743	16.5044	16.3547	44.8282	45.8551
SP8	IRF_known21	cluster_5	Common_1_DN	16568	21809.626	21425	22184	229.0018	0.7597	-0.3965	0.001	0.001	7912	114984	65.268	2468	34061	19.334	1166	16568	9.4044	14.7371	14.409	47.2447	48.6421
SP8	SOX2_6	cluster_5	Common_1_DN	4100	5398.414	5180	5590	127.8112	0.7595	-0.3968	0.001	0.0010435	1941	31108	57.681	667	9744	18.068	267	4100	7.6023	13.7558	13.1799	40.03	42.0772
SP8	TCF7L1_2	cluster_2	DP	10336	13609.744	13292	13876	185.4896	0.7595	-0.3969	0.001	0.0010435	11685	129185	56.684	2272	24364	10.69	942	10336	4.5352	8.0616	8.0009	41.4613	42.4232
SP4	SPDEF_4	cluster_5	Common_1_DN	17104	22522.604	22186	22882	219.1478	0.7594	-0.397	0.001	0.0010435	7657	115318	63.966	2551	36276	20.122	1157	17104	9.4874	15.1104	14.832	45.3548	47.1496
SP8	ETV3_1	cluster_2	DP	11749	15473.822	15244	15697	144.5069	0.7593	-0.3973	0.001	0.0010435	13610	123083	65.171	2666	23782	12.592	1304	11749	6.2209	9.5812	9.5456	48.9122	49.4029
DP	RUNX3_1	cluster_5	Common_1_DN	47896	63115.879	62372	63803	431.9075	0.7589	-0.3981	0.001	0.0010435	18870	289545	59.922	7152	106249	21.989	3147	47896	9.9123	16.6773	16.5418	44.0017	45.079
DP	KLF16_1	cluster_5	Common_1_DN	120943	159382.431	158501	160253	525.5938	0.7588	-0.3982	0.001	0.001	76014	850789	64.652	23523	251382	19.103	11015	120943	9.1906	14.4908	14.2154	46.8265	48.1112
SP4	MYBL1_5	cluster_5	Common_1_DN	7072	9321.181	9037	9566	158.5084	0.7587	-0.3983	0.001	0.0010435	2888	46605	66.715	933	14337	20.523	448	7072	10.124	15.5125	15.1743	48.0171	49.3269
SP4	HMGA1_1	cluster_5	Common_1_DN	3526	4649.718	4541	4752	64.9844	0.7584	-0.399	0.001	0.0010435	3745	22602	66.657	1205	7071	20.853	593	3526	10.399	15.8344	15.6004	49.2116	49.8656
DN	ETV1_1	cluster_1	SP8	1866	2461.212	2298	2630	96.6144	0.7583	-0.3992	0.001	0.0010909	5090	46038	26.319	1065	9493	5.4269	207	1866	1.0667	4.0668	4.0532	19.4366	19.6566
SP8	NFATC1_3	cluster_5	Common_1_DN	6420	8469.287	8251	8736	145.2558	0.7581	-0.3996	0.001	0.0010909	3018	39257	57.876	1232	15103	22.266	507	6420	9.4648	16.7992	16.3538	41.1526	42.5081
DP	EGR1_known9	cluster_5	Common_1_DN	51364	67760.994	67176	68355	353.746	0.758	-0.3997	0.001	0.001	29172	396004	73.325	7106	93622	17.335	3837	51364	9.5107	13.153	12.9706	53.9966	54.8632
SP4	FOXP3_1	cluster_2	DP	10333	13632.717	13255	13975	216.0117	0.758	-0.3998	0.001	0.0010909	7981	128134	57.854	1558	23954	10.816	640	10333	4.6655	8.019	8.0642	41.0783	43.1368
SP8	FOXO1_5	cluster_5	Common_1_DN	45950	60623.545	60030	61198	374.2106	0.758	-0.3998	0.001	0.0010435	25271	279379	58.574	9821	104850	21.983	4203	45950	9.6337	16.6317	16.4472	42.796	43.8245
SP8	POU2F2_known15	cluster_5	Common_1_DN	4845	6393.168	6211	6593	120.6272	0.7579	-0.4	0.001	0.0010435	3075	33949	59.103	1058	11078	19.286	452	4845	8.4349	14.6992	14.2714	42.7221	43.7353
SP8	MAF_known12	cluster_2	DP	10978	14489.668	14148	14844	220.7116	0.7577	-0.4004	0.001	0.0010435	7662	130608	59.214	1524	24862	11.272	653	10978	4.9771	8.5226	8.4053	42.8478	44.1557
SP4	SOX18_4	cluster_5	Common_1_DN	4550	6007.562	5783	6240	129.8198	0.7574	-0.4008	0.001	0.0012	2335	32760	62.2	773	10090	19.157	337	4550	8.6389	14.4325	13.8889	43.5964	45.0942
DP	ELK4_1	cluster_5	Common_1_DN	10656	14070.854	13880	14267	116.8148	0.7573	-0.401	0.001	0.001	10661	85897	77.625	2290	18216	16.462	1325	10656	9.6298	12.4285	12.4056	57.8603	58.498
DP	KLF13_1	cluster_5	Common_1_DN	52863	69818.076	69034	70617	461.5093	0.7572	-0.4013	0.001	0.001	19895	342302	61.998	6957	114292	20.701	3113	52863	9.5745	15.6471	15.4434	44.7463	46.2526
SP8	ATF3_known5	cluster_5	Common_1_DN	27234	35978.883	35450	36482	311.5409	0.757	-0.4017	0.001	0.001	11933	167611	58.351	4636	62555	21.777	1961	27234	9.481	16.4334	16.2483	42.2994	43.5361
SP4	IRF6_1	cluster_5	Common_1_DN	6827	9020.333	8790	9260	144.3084	0.7569	-0.4019	0.001	0.0010909	2762	44279	67.58	895	13587	20.737	433	6827	10.42	15.677	15.4181	48.3799	50.2466
SP8	NFKB_known11	cluster_5	Common_1_DN	22806	30137.309	29775	30517	225.7373	0.7567	-0.4021	0.001	0.001	12335	149870	65.352	3973	46680	20.355	1897	22806	9.9448	15.379	15.2172	47.7473	48.856
DP	SP4_2	cluster_5	Common_1_DN	135925	179643.014	178593	180615	664.7421	0.7566	-0.4023	0.001	0.001	56997	957954	67.242	17108	272423	19.122	8258	135925	9.541	14.4885	14.1891	48.2698	49.8948
SP4	E2F_known24	cluster_5	Common_1_DN	7815	10329.308	10028	10581	167.5555	0.7566	-0.4024	0.001	0.001	3037	51804	67.185	998	15843	20.547	470	7815	10.135	15.4758	15.0857	47.0942	49.3278
SP4	SOX10_9	cluster_5	Common_1_DN	7045	9314.414	9074	9557	162.3317	0.7564	-0.4028	0.001	0.0010435	3684	51623	61.408	1186	15642	18.607	512	7045	8.3804	13.8979	13.647	43.1703	45.039
SP4	POU2F2_known11	cluster_2	DP	3452	4564.469	4405	4718	93.3844	0.7563	-0.4029	0.001	0.0010435	4718	47310	63.382	762	7325	9.8134	348	3452	4.6247	7.376	7.2966	45.6693	47.1263
SP8	ATF7_1	cluster_2	DP	2931	3875.928	3702	4028	100.2318	0.7563	-0.403	0.001	0.001	3427	44593	61.045	503	6389	8.7462	227	2931	4.0124	6.6239	6.5728	45.1292	45.8757
SP4	SOX14_3	cluster_5	Common_1_DN	7059	9335.462	9082	9586	158.742	0.7562	-0.4032	0.001	0.0010909	3418	47906	58.588	1254	16454	20.123	523	7059	8.633	15.3013	14.7351	41.7065	42.9014
SP4	ELK4_2	cluster_2	DP	6852	9062.432	8771	9359	178.5005	0.7561	-0.4033	0.001	0.0011429	7834	70855	26.241	3873	34667	12.839	761	6852	2.5376	9.7141	9.6705	19.6489	19.7652
SP4	SOX17_4	cluster_2	DP	2809	3715.232	3526	3897	103.0663	0.7561	-0.4033	0.001	0.0010909	2822	39514	62.402	464	6163	9.7328	204	2809	4.4361	7.2289	7.1089	43.9655	45.5785
DN	SPI1_known4	cluster_1	SP8	16682	22065.044	21727	22433	205.8117	0.756	-0.4034	0.001	0.0010435	19284	251359	76.991	2317	29252	8.9598	1298	16682	5.1097	6.731	6.6367	56.0207	57.0286
SP8	MAF_known1	cluster_5	Common_1_DN	67172	88843.672	87952	89786	560.4249	0.7561	-0.4034	0.001	0.001	25525	385561	56.181	11086	160598	23.401	4507	67172	9.7878	17.6572	17.4219	40.6549	41.8262
SP8	SPZ1_2	cluster_5	Common_1_DN	23601	31215.931	30805	31636	249.6595	0.7561	-0.4034	0.001	0.0010435	13891	139218	56.526	5732	55792	22.653	2374	23601	9.5825	17.0902	16.9525	41.4166	42.3018
SP8	TBX5_3	cluster_2	DP	13035	17242.337	16914	17569	196.0975	0.756	-0.4035	0.001	0.001	14898	134209	54.179	3634	32120	12.967	1450	13035	5.2621	9.7329	9.7125	39.9009	40.5822
SP4	TBX2_2	cluster_2	DP	8151	10790.719	10517	11056	155.6539	0.7554	-0.4047	0.001	0.0010909	9302	93136	58.465	1906	18653	11.709	820	8151	5.1167	8.8153	8.7517	43.022	43.6981
DP	ID4_1	cluster_5	Common_1_DN	61457	81365.847	80723	81992	380.7477	0.7553	-0.4048	0.001	0.0010435	35081	317583	54.366	16901	150382	25.744	6815	61457	10.521	19.4265	19.3515	40.3231	40.8673
SP8	TBX15_2	cluster_5	Common_1_DN	18040	23884.306	23590	24195	176.9934	0.7553	-0.4048	0.001	0.0010909	14966	104782	54.532	6406	44116	22.96	2595	18040	9.3887	17.3393	17.2167	40.5089	40.8922
SP8	SOX14_2	cluster_5	Common_1_DN	5389	7135.466	6938	7330	119.4579	0.7553	-0.4049	0.001	0.0010909	3330	36787	57.469	1208	12699	19.838	498	5389	8.4187	14.955	14.6492	41.2252	42.4364
SP4	POU2F3_2	cluster_5	Common_1_DN	4256	5635.501	5499	5756	82.5505	0.7553	-0.405	0.001	0.0010909	3575	28645	62.59	1185	9162	20.019	540	4256	9.2995	15.1049	14.8577	45.5696	46.4527
DP	SP8_1	cluster_5	Common_1_DN	112253	148656.847	147767	149582	549.3951	0.7551	-0.4052	0.001	0.001	63765	778558	62.307	20709	242789	19.43	9328	112253	8.9835	14.6287	14.4181	45.0432	46.2348
SP8	NFKB_known10	cluster_5	Common_1_DN	20390	27004.542	26643	27367	227.2275	0.7551	-0.4053	0.001	0.001	10708	129395	64.216	3651	42572	21.128	1706	20390	10.119	15.932	15.758	46.7269	47.8953
SP8	KLF14_1	cluster_4	SP4	28664	37967.883	37502	38386	268.6712	0.755	-0.4055	0.001	0.001	51058	690823	69.873	4312	55543	5.6179	2164	28664	2.8992	4.2383	4.1493	50.1855	51.6069
SP4	MYBL2_2	cluster_5	Common_1_DN	5197	6886.095	6676	7081	127.7647	0.7547	-0.4059	0.001	0.0010435	2320	35130	65.23	738	10773	20.003	348	5197	9.6498	15	14.7936	47.1545	48.241
SP8	EWSR1::FLI1_1	cluster_5	Common_1_DN	147733	195737.526	194115	197339	935.6357	0.7548	-0.4059	0.001	0.0010435	45443	904295	55.466	19564	364049	22.329	7636	147733	9.0614	16.8035	16.3368	39.0309	40.5805
SP8	TCF7L2_known5	cluster_5	Common_1_DN	26420	35004.659	34460	35639	353.3454	0.7548	-0.4059	0.001	0.0010435	10823	173781	55.479	4181	64167	20.485	1669	26420	8.4346	15.4209	15.203	39.9187	41.1738
DP	ETV3_1	cluster_5	Common_1_DN	17971	23816.691	23518	24142	194.0058	0.7546	-0.4063	0.001	0.0010435	13947	126127	66.782	4036	35843	18.978	1997	17971	9.5154	14.3185	14.2483	49.4797	50.1381
SP4	IRF4_1	cluster_5	Common_1_DN	7049	9343.354	9133	9555	132.6736	0.7545	-0.4065	0.001	0.0010435	3307	46509	68.576	1029	13863	20.441	511	7049	10.394	15.4521	15.1562	49.6599	50.8476
SP4	POU2F3_1	cluster_5	Common_1_DN	8470	11231.623	10963	11558	186.0735	0.7541	-0.4071	0.001	0.0010435	3852	58181	62.51	1307	18511	19.888	571	8470	9.1003	14.8235	14.558	43.6878	45.7566

SP4	TBX5_3	cluster_5	Common_1_DN	23040	30551.192	30151	30937	232.6092	0.7542	-0.4071	0.001	0.001	15391	138670	55.98	6304	55260	22.308	2578	23040	9.301	16.75	16.615	40.8947	41.6938
SP8	FOXO6_1	cluster_5	Common_1_DN	4708	6243.418	6050	6448	126.8209	0.7541	-0.4071	0.001	0.0010909	2530	32988	57.537	901	11153	19.453	367	4708	8.2115	14.5059	14.2719	40.7325	42.2129
SP4	RUNX1_4	cluster_5	Common_1_DN	4820	6392.556	6199	6559	106.2647	0.754	-0.4073	0.001	0.0010909	3165	32119	63.475	1062	10306	20.367	484	4820	9.5255	15.2923	15.0067	45.5744	46.7689
SP4	SMAD_2	cluster_5	Common_1_DN	39098	51853.22	51306	52348	313.6478	0.754	-0.4073	0.001	0.0010435	22080	227820	57.244	9075	91574	23.01	3794	39098	9.8241	17.183	17.1618	41.8072	42.6955
SP8	SPI1_known4	cluster_5	Common_1_DN	30828	40884.462	40315	41411	325.7366	0.754	-0.4073	0.001	0.0010435	14322	186719	57.192	5779	72542	22.219	2389	30828	9.4425	16.6806	16.5104	41.3393	42.4968
SP4	RUNX1_2	cluster_5	Common_1_DN	10194	13522.085	13224	13812	183.9336	0.7539	-0.4076	0.001	0.0010435	4574	71425	63.314	1533	22215	19.692	673	10194	9.0364	14.7136	14.2723	43.9008	45.8879
SP4	TCF12_known1	cluster_5	Common_1_DN	46075	61120.238	60669	61610	295.0492	0.7538	-0.4077	0.001	0.0010909	34730	249719	53.636	16034	114471	24.587	6410	46075	9.8963	18.4567	18.4507	39.9775	40.2504
SP4	TCF4_2	cluster_5	Common_1_DN	48868	64834.503	64195	65452	364.3335	0.7537	-0.4079	0.001	0.001	28828	260376	48.438	15187	134709	25.06	5445	48868	9.0909	18.8879	18.7682	35.853	36.2767
SP8	FOXO4_2	cluster_2	DP	6485	8606.103	8350	8884	158.215	0.7536	-0.4082	0.001	0.0010435	6205	81377	63.594	1117	13917	10.876	502	6485	5.0679	8.0902	7.9691	44.9418	46.5977
SP4	SMAD_1	cluster_5	Common_1_DN	22220	29489.35	29164	29835	207.6938	0.7535	-0.4083	0.001	0.0010435	16152	129249	59.226	6404	50170	22.989	2789	22220	10.182	17.2672	17.1916	43.5509	44.2894
SP8	E2F7_1	cluster_5	Common_1_DN	8809	11691.027	11415	11927	156.4141	0.7535	-0.4083	0.001	0.0010435	4510	58679	64.962	1499	18468	20.445	696	8809	9.7522	15.4324	15.0122	46.431	47.6987
DP	KLF12_2	cluster_5	Common_1_DN	80226	106481.959	105656	107384	510.5169	0.7534	-0.4085	0.001	0.001	38298	549311	63.594	12334	169624	19.637	5669	80226	9.2878	14.8023	14.6048	45.9624	47.2964
SP4	SOX2_6	cluster_5	Common_1_DN	4381	5815.211	5617	6009	128.1352	0.7534	-0.4085	0.001	0.0010435	2099	33627	62.352	667	9744	18.068	284	4381	8.1233	13.5303	13.0282	42.5787	44.961
DN	ATF7_1	cluster_2	DP	3314	4399.588	4244	4577	96.4117	0.7533	-0.4087	0.001	0.001	3904	50828	69.581	503	6389	8.7462	257	3314	4.5367	6.583	6.52	51.0934	51.8704
SP8	SOX5_1	cluster_5	Common_1_DN	2780	3691.252	3566	3818	77.9992	0.7532	-0.4089	0.001	0.0010909	2249	20248	60.174	715	6233	18.524	313	2780	8.2618	13.9173	13.7298	43.7762	44.6013
SP4	MAF_known10	cluster_5	Common_1_DN	15972	21206.949	20868	21558	199.1939	0.7532	-0.409	0.001	0.0010909	8761	96521	59.023	3417	36443	22.285	1468	15972	9.767	16.7561	16.5477	42.9617	43.8273
DP	GMEB2_3	cluster_5	Common_1_DN	3650	4847.015	4680	4995	100.5526	0.7531	-0.4091	0.001	0.0010435	2081	29462	70.749	509	6963	16.721	260	3650	8.765	12.494	12.3888	51.0806	52.4199
SP4	ETV4_2	cluster_2	DP	4023	5342.333	5095	5558	138.7887	0.7531	-0.4091	0.001	0.0011429	4906	44382	25.767	2328	20847	12.103	447	4023	2.3356	9.1113	9.0645	19.201	19.2977
SP8	TBX4_1	cluster_2	DP	7643	10149.377	9925	10384	138.1683	0.7531	-0.4091	0.001	0.0010435	11799	82621	55.065	2687	18553	12.365	1096	7643	5.0938	9.2889	9.2507	40.789	41.1955
DN	ELK3_1	cluster_1	SP8	2468	3277.606	3089	3471	113.526	0.7531	-0.4092	0.001	0.0011429	6350	57329	26.539	1397	12448	5.7624	275	2468	1.1425	4.3307	4.305	19.685	19.8265
SP8	FOXO1_4	cluster_5	Common_1_DN	4030	5352.091	5137	5566	121.7975	0.753	-0.4092	0.001	0.0010909	2229	29043	58.324	766	9486	19.05	316	4030	8.093	14.1768	13.876	41.2533	42.4837
SP8	TBX1_4	cluster_5	Common_1_DN	26201	34800.946	34236	35383	358.2462	0.7529	-0.4095	0.001	0.0010909	8594	163899	55.977	3505	63358	21.639	1396	26201	8.9486	16.2439	15.9861	39.8288	41.3539
SP4	RREB1_2	cluster_5	Common_1_DN	186133	247268.223	245565	249026	1009.9198	0.7528	-0.4097	0.001	0.001	51020	1245776	62.597	18832	415372	20.871	7904	186133	9.3528	15.492	14.9411	41.9711	44.8112
SP8	TBX19_1	cluster_2	DP	5612	7455.885	7135	7775	186.5716	0.7527	-0.4098	0.001	0.0010435	3619	68942	53.926	770	14099	11.028	298	5612	4.3897	8.2343	8.1402	38.7013	39.8042
SP8	TBX5_5	cluster_2	DP	9279	12329.245	11977	12711	242.4976	0.7526	-0.41	0.001	0.0010435	5748	109382	56.634	1223	22231	11.51	495	9279	4.8043	8.6117	8.4831	40.4742	41.739
SP8	TCF3_1	cluster_5	Common_1_DN	117848	156587.992	155457	157698	685.3707	0.7526	-0.41	0.001	0.0011429	45409	645019	52.166	21819	302495	24.464	8335	117848	9.531	18.3554	18.2705	38.2007	38.9587
DP	SOX2_6	cluster_3	DN	2889	3839.04	3617	4034	127.9891	0.7526	-0.4101	0.001	0.0010435	1589	25439	47.17	562	8388	15.553	184	2889	5.3568	11.5796	11.3566	32.7402	34.4421
DP	TCF4_2	cluster_5	Common_1_DN	55510	73771.205	73084	74382	398.1167	0.7525	-0.4103	0.001	0.001	32705	295539	54.979	15187	134709	25.06	6168	55510	10.326	18.8595	18.7826	40.6137	41.2073
SP4	MGA_3	cluster_5	Common_1_DN	14059	18691.925	18307	19100	239.1921	0.7522	-0.4109	0.001	0.0010909	5104	86900	61.334	1893	30937	21.835	836	14059	9.9228	16.3793	16.1784	44.1627	45.444
SP4	IRF_known21	cluster_5	Common_1_DN	16945	22529.967	22160	22877	216.307	0.7521	-0.411	0.001	0.001	8196	119181	67.65	2468	34061	19.334	1183	16945	9.6184	14.4339	14.2179	47.9335	49.749
SP8	FOXO1_4	cluster_2	DP	2113	2809.757	2659	2964	95.7382	0.7521	-0.411	0.001	0.0010909	2229	29043	58.324	405	4964	9.9687	168	2113	4.2433	7.537	7.2754	41.4815	42.5665
SP8	TBX4_1	cluster_5	Common_1_DN	13917	18510.288	18218	18780	164.5759	0.7519	-0.4115	0.001	0.0010435	11799	82621	55.065	4930	33931	22.614	2003	13917	9.2753	16.976	16.8444	40.6288	41.0156
SP4	FOXO4_2	cluster_5	Common_1_DN	11130	14805.737	14480	15117	188.103	0.7518	-0.4117	0.001	0.0010435	6478	84929	66.37	1895	23131	18.076	875	11130	8.6978	13.5073	13.1051	46.1741	48.1172
SP4	MAF_known12	cluster_5	Common_1_DN	21361	28418.69	27910	28923	287.835	0.7517	-0.4118	0.001	0.0010435	7957	135675	61.511	2875	47044	21.328	1264	21361	9.6845	15.8854	15.7442	43.9652	45.4064
SP8	SPI1_known1	cluster_5	Common_1_DN	42012	55911.095	55464	56411	293.3003	0.7514	-0.4123	0.001	0.001	35122	247084	52.91	15325	106225	22.747	5997	42012	8.9964	17.0748	17.0031	39.1321	39.55
DP	KLF4_1	cluster_5	Common_1_DN	105073	139857.674	139128	140654	446.1574	0.7513	-0.4126	0.001	0.001	76804	740702	63.769	23920	222161	19.126	11074	105073	9.046	14.4185	14.1856	46.296	47.2959
SP4	POU2F2_known12	cluster_2	DP	3122	4155.887	4001	4303	89.4045	0.7513	-0.4126	0.001	0.0010435	4643	46514	63.727	697	6675	9.1451	317	3122	4.2773	6.8275	6.712	45.4806	46.7715
DP	ELK3_1	cluster_5	Common_1_DN	8262	10998.546	10666	11338	201.0815	0.7512	-0.4127	0.001	0.0011429	6415	57946	26.824	4619	41177	19.061	919	8262	3.8246	14.3258	14.2581	19.8961	20.0646
SP8	RUNX_1	cluster_5	Common_1_DN	64458	85805.885	84956	86631	491.642	0.7512	-0.4127	0.001	0.0010435	23767	334786	52.302	12058	165555	25.864	4607	64458	10.07	19.384	19.2535	38.207	38.9345
DP	HIF1A::ARNT_1	cluster_5	Common_1_DN	11484	15288.687	15091	15484	126.8268	0.7512	-0.4128	0.001	0.001	10494	75476	67.161	3229	22887	20.366	1590	11484	10.219	15.1515	15.2154	49.2413	50.177
SP4	SOX9_6	cluster_5	Common_1_DN	10007	13322.968	12986	13636	196.9957	0.7511	-0.4129	0.001	0.0010909	4528	68048	61.63	1562	22247	20.149	683	10007	9.0632	15.0839	14.7058	43.726	44.9813
SP8	ATF4_2	cluster_2	DP	3193	4251.894	4083	4463	113.8271	0.751	-0.4131	0.001	0.0010909	3830	46012	56.648	650	7608	9.3666	269	3193	3.9311	7.0235	6.9395	41.3846	41.969
SP4	IKZF2_3	cluster_5	Common_1_DN	8760	11666.065	11439	11895	140.7642	0.7509	-0.4133	0.001	0.0010909	5825	58444	66.163	1899	18181	20.582	894	8760	9.917	15.3476	14.9887	47.0774	48.1822
DP	SP4_1	cluster_5	Common_1_DN	197041	262426.973	260985	263568	813.5378	0.7508	-0.4134	0.001	0.001	81673	1469721	70.891	22652	380416	18.349	11338	197041	9.5042	13.8822	13.4067	50.053	51.7962
DP	GATA_known16	cluster_3	DN	10881	14492.836	13988	14946	303.2067	0.7508	-0.4135	0.001	0.0010435	4004	84333	48.865	1533	30496	17.67	524	10881	6.3048	13.0869	12.9024	34.1813	35.6801
SP4	MESP1_1	cluster_5	Common_1_DN	47943	63880.849	63303	64479	368.1883	0.7505	-0.4141	0.001	0.001	28293	255419	48.619	14895	132166	25.158	5339	47943	9.1259	18.8704	18.7703	35.8442	36.2748
SP8	IRF4_1	cluster_5	Common_1_DN	6889	9182.271	8968	9432	141.3786	0.7503	-0.4145	0.001	0.0010435	3249	45692	67.371	1029	13863	20.441	496	6889	10.158	15.2662	15.077	48.2021	49.6934
SP4	TCF7L2_known7	cluster_2	DP	14079	18770.396	18371	19170	242.8984	0.7501	-0.4149	0.001	0.0010435	12562	176542	61.995	2272	30780	10.809	1013	14079	4.9441	8.064	7.9749	44.5863	45.7407
SP8	E2F8_1	cluster_5	Common_1_DN	12600	16799.208	16530	17116	186.5325	0.7501	-0.4149	0.001	0.001	7551	83269	64.648	2499	26482	20.56	1160	12600	9.7824	15.3622	15.1317	46.4186	47.5795
DP	PKNOX2_2	cluster_3	DN	19133	25510.614	25078	25927	262.6542	0.75	-0.415	0.001	0.0010435	11043	122680	50.42	4632	50861	20.903	1721	19133	7.8634	15.5845	15.5959	37.1546	37.6182
DP	EGR4_1	cluster_5	Common_1_DN	45237	60318.765	59806	60918	330.9931	0.75	-0.4151	0.001	0.001	29511	334771	66.296	8378	92071	18.233	4057	45237	8.9585	13.7474	13.5128	48.4244	49.1327
DP	ELK3_2	cluster_5	Common_1_DN	7968	10624.466	10327	10942	185.5713	0.75	-0.4151	0.001	0.0011429	6179	55790	26.533	4526	40342	19.186	887	7968	3.7895	14.3551	14.2821	19.5979	19.7511
SP8	MAFF_1	cluster_5	Common_1_DN	22273	29702.652	29243	30151	286.2301	0.7499	-0.4153	0.001	0.001	9420	132144	52.834	4204	56810	22.714	1602	22273	8.9052	17.0064	16.8551	38.1066	39.2061
SP4	SOX5_1	cluster_2	DP	1543	2058.271	1964	2167	60.5833	0.7498	-0.4155	0.001	0.0010909	2386	21481	63.838	373	3266	9.7061	174	1543	4.5856	7.2925	7.1831	46.6488	47.2443
SP4	SOX15_4	cluster_2	DP	2092	2790.999	2634	2931	87.9434	0.7496	-0.4157	0.001	0.0011429	2335	32728	59.319	374	4898	8.8775	155	2092	3.7917	6.6381	6.3921	41.4439	42.7113
SP8	RBPJ_2	cluster_5	Common_1_DN	17364	23171.049	22832	23507	230.9098	0.7494	-0.4162	0.001	0.0010909	10072	100834	56.034	4334	41855	23.259	1757	17364	9.6493	17.4444	17.2204	40.5399	41.4861
DP	SPZ1_1	cluster_5	Common_1_DN	105518	140818.734	139873	141769	561.2573	0.7493	-0.4163	0.001	0.001	49189	720565	65.335	15701	219487	19.901	7332	105518	9.5675	14.9058	14.6438	46.6977	48.0748
SP8	AHR::ARNT::HIF1A_1	cluster_4	SP4	3521	4699.432	4574	4826	79.9916	0.7493	-0.4164	0.001	0.0010435	12611	103685	71.095	821	6656	4.5639	431	3521	2.4143	3.4177	3.3959	52.497	52.8996
SP8	TBX21_6	cluster_2	DP	11609	15500.741	15011	15893	248.8972	0.7489	-0.4171	0.001	0.0010909	8068	145632	57.226	1600	27516	10.812	648	11609	4.5617	8.0317	7.9715	40.5	42.19
SP8	E2F_known24	cluster_5	Common_1_DN	7608	10159.206	9881	10468	171.0409	0.7489	-0.4172	0.001	0.001	2992	51042	66.197	998	15843	20.547	461	7608	9.8669	15.4078	14.9054	46.1924	48.0212
SP8	IKZF1_1	cluster_5	Common_1_DN	17739	23690.92	23356	24078	220.824	0.7488	-0.4174	0.001	0.0010435	8739	105142	60.209	3496	40165	23	1499	17739	10.158	17.153	16.8715	42.8776	44.1653
SP4	SOX14_2	cluster_5	Common_1_DN	5590	7467.354	7271	7662	120.4031	0.7486	-0.4177	0.001	0.0010909	3491	38572	60.257	1208	12699	19.838	516	5590	8.7327	14.7809	14.4924	42.7152	44.0192
DP	TCF3_2	cluster_5	Common_1_DN	80067	106975.312	106074	107932	568.0131	0.7485	-0.418	0.001	0.0010435	27591	417427	54.427	13449	197852	25.797	5332	80067	10.44	19.3251	19.1811	39.6461	40.4681
SP8	FOXO6_3	cluster_5	Common_1_DN	46741	62450.26	61779	63163	400.948	0.7485	-0.418	0.001	0.0010435	22108	289208	59.065	8509	107163	21.886	3619	46741	9.546	16.3696	16.1617	42.5314	43.6167
DP	EGR4_2	cluster_5	Common_1_DN	104218	139265.403	138181	140177	602.5517	0.7483	-0.4182	0.001	0.001	49002	804691	69.973	13078	203425	17.689	6502	104218	9.0624	13.2688	12.9513	49.7171	51.2317
DP	PKNOX2_3	cluster_3	DN	16167	21602.936	21200	22086	257.6268	0.7484	-0.4182	0.001	0.0010435	9600	106895	49.891	3958	43496	20.301	1452	16167	7.5456	15.125	15.1242	36.6852	37.1689
DP	ETS_known17	cluster_5	Common_1_DN	33599	44903.558	44239	45604	400.5784	0.7483	-0.4184	0.001	0.001	12494	213553	60.343	4576	75147	21.234	1980	33599	9.4939	15.8476	15.7333	43.2692	44.711
SP8	E2F_known26	cluster_5	Common_1_DN	4143	5537.696	5355	5730	119.7663	0.7482	-0.4185	0.001	0.0010435	1668	28373	65.605	551	8698	20.112	250	4143	9.5796	14.988	14.6019	45.3721	47.6316
SP8	ETV1_1	cluster_2	DP	3923	5244.277	5012	5478	136.6382	0.7481	-0.4187	0.001	0.0010909	4842	43840	25.062	2353	21073	12.047	435	3923	2.2427	8.9839	8.9484	18.487	18.6162
SP4	SPIB_1	cluster_5	Common_1_DN	18323	24500.314	24230	24799	169.0843	0.7479	-0.4191	0.001	0.0010435	18137	108822	58.346	7127	42285	22.672	3068	18323	9.8241	16.9157	16.8376	43.0476	43.3322
SP4	E2F2_3	cluster_5	Common_1_DN	5392	7210.655	6965	7444	138.4667	0.7478	-0.4192	0.001	0.001	2073	35280	66.325	696	11157	20.975	325	5392	10.137	15.6778	15.2834	46.6954	48.3284
SP8	IRF6_1	cluster_5	Common_1_DN	6720	8987.539	8726	9241	153.1236	0.7477	-0.4194	0.001	0.0010909	2749	44071	67.262	895	13587	20.737	425	6720	10.256	15.4602	15.2481	47.486	49.459
DN	SPIB_2	cluster_1	SP8	19900	26615.832	26247	27073	230.4812	0.7477	-0.4195	0.001	0.0010435	23403	305650	75.803	2806	35711	8.8566	1534	19900	4.9353	6.5547	6.5107	54.6686	55.7251
SP4	SOX8_9	cluster_5	Common_1_DN	6198	8290.286	8037	8567	156.5522	0.7477	-0.4196	0.001	0.0010909	2995	47951	62.253	940	13886	18.028	401	6198	8.0466	13.389	12.9257	42.6596	44.6349
SP4	FOXO1_5	cluster_5	Common_1_DN	44821	59957.465	59369	60520	350.0427	0.7476	-0.4198	0.001	0.0010435	25037	276688	58.01	9821	104850	21.983	4103	44821	9.397	16.3877	16.1991	41.7778	42.7477
SP4	CTCF_known1	cluster_5	Common_1_DN	181252	242487.82	240949	243981	869.5157	0.7475	-0.4199	0.001	0.0010435	55221	1020372	58.86	23232	415314	23.957	9843	181252	10.455	17.8247	17.7633	42.3683	43.6422
SP8	POU2F2_known13	cluster_2	DP	3687	4932.917	4677	5163	145.9605	0.7475	-0.4199	0.001	0.0010909	2653	55941	63.012	436	8267	9.312	180	3687	4.1531	6.7848	6.5909	41.2844	44.599
SP4	ETV2_1	cluster_2	DP	7467	9991.083	9609	10320	207.8685	0.7474	-0.4201	0.001	0.0010435	7748	77780	26.389	3829	37988	12.888	746	7467	2.5334	9.6283	9.6002	19.4829	19.6562
DP	KLF7_1	cluster_5	Common_1_DN	158521	212133.464	210980	213286	730.8756	0.7473	-0.4203	0.001	0.001	72055	1166887	65.914	21519	328882	18.578	10044	158521	8.9544	13.9394	13.5849	46.675	48.2
DP	MESP1_1	cluster_5	Common_1_DN	55106	73741.862	73169	74376	358.2122	0.7473	-0.4203	0.001	0.001	32605	294479	56.054	14895	132166	25.158	6127	55106	10.489	18.7916	18.713	41.1346	41.6945
SP8	HIF1A_2	cluster_4	SP4	6926	9268.613	9052	9493	131.0588	0.7473	-0.4203	0.001	0.001	13715	183722	69.394	1053	13560	5.1218	525	6926	2.616	3.8279	3.7698	49.8575	51.0767
SP4	LMO2_2	cluster_5	Common_1_DN	5071	6787.197	6635	6936	95.5702	0.7472	-0.4205	0.001	0.0010435	3781	30272	59.431	1473	11555	22.685	639	5071	9.9556	16.9003	16.7515	43.3809	43.8858
SP4	FOXO3_4	cluster_2	DP	1799	2408.249	2257	2539	82.3484	0.7471	-0.4206	0.001	0.0010909	2012	26245	62.811	319	3951	9.4558	140	1799	4.3055	6.9583	6.8546	43.8871	45.5328
SP8	XBP1_2	cluster_2	DP	3975	5321.841	5056	5590	159.5352	0.747	-0.4209	0.001	0.0010435	2655	61164	63.418	396	8574	8.8899	176	3975	4.1215	6.629	6.4989	44.4444	46.3611
DP	TCF3_1	cluster_5	Common_1_DN	128672	172271.776	171236	173277	643.7229	0.7469	-0.421	0.001	0.0011429	49855	709136	57.352	21819	302495	24.464	9101	128672	10.406	18.2549	18.1449	41.7114	42.5369
SP8	SMAD4_1	cluster_5	Common_1_DN	59833	80146.735	79346	80881	484.1559	0.7465	-0.4217	0.001	0.001	25393	360702	58.545	10079	138396	22.463	4247	59833	9.7114	16.7251	16.5879	42.1371	43.2332
DP	TCF4_1	cluster_5	Common_1_DN	69154	92645.484	91987	93267	395.3343	0.7464	-0.4219	0.001	0.0010909	39369	356288	54.334	19242	171202	26.108	7671	69154	10.546	19.4849	19.4096	39.8659	40.3932
SP8	FOXM1_1	cluster_2	DP	3065	4106.757	3946	4253	91.5089	0.7464	-0.422	0.001	0.0010909	4986	39915	56.233	953	7417	10.449	388	3065	4.3181	7.7818	7.6788	40.7135	41.324
SP8	SOX9_4	cluster_2	DP	5512	7385.11	7113	7652	150.3822	0.7464	-0.422	0.001	0.0010909	5012	75280	58.223	910	13004	10.058	374	5512	4.2631	7.4621	7.322	41.0989	42.387
SP4	NFE2L1::MAFG_1	cluster_2	DP	16798	22513.391	21937	23101	355.0997	0.7461	-0.4225	0.001	0.0010435	8781	185151	56.968	2037	40418	12.436	814	16798	5.1684	9.27	9.0726	39.9607	41.5607
SP8	SOX10_4	cluster_2	DP	5922	7937.148	7686	8243	169.1814	0.7461	-0.4225	0.001	0.0010909	5401	81262	58.18	973	13974	10.005	401	5922	4.2399	7.4246	7.2875	41.2127	42.3787
DP	ETV6_1	cluster_5	Common_1_DN	57624	77247.818	76493	78062	450.4909	0.746	-0.4228	0.001	0.001	26268	378682	62.769	8947	124366	20.615	4045	57624	9.5516	15.399	15.217	45.2107	46.3342
DP	STAT_known2	cluster_5	Common_1_DN	26704	35798.988	35232	36339	320.4527	0.7459	-0.4228	0.001	0.001	8789	176424	66.894	2866	54457	20.648	1361	26704	10.125	15.4853	15.1363	47.4878	49.0369
DP	KLF14_1	cluster_5	Common_1_DN	90648	121529.964	120686	122413	507.2506	0.7459	-0.423	0.001	0.001	49087	664659	67.227	14140	183594	18.57	6782	90648	9.1686	13.8163	13.6383	47.9632	49.3742
SP4	TBX15_1	cluster_5	Common_1_DN	17385	23312.771	22781	23835	321.1992	0.7457	-0.4233	0.001	0.0010909	6066	109292	57.806	2443	41328	21.859	985	17385	9.1951	16.238	15.9069	40.3193	42.0659
SP4	SMAD3_3	cluster_5	Common_1_DN	5718	7668.552	7492	7884	117.9587	0.7457	-0.4234	0.001	0.0010435	3934	35416	61.507	1443	12645	21.961	641	5718	9.9305	16.2938	16.1452	44.4213	45.2195
SP4	BATF_known1	cluster_2	DP	3309	4438.154	4248	4617	115.6848	0.7456	-0.4235	0.001	0.0010435	3974	51803	59.832	592	7502	8.6647	255	3309	3.8219	6.4167	6.3877	43.0743	44.1082
DN	CUX1_9	cluster_1	SP8	832	1116.485	1029	1223	56.8757	0.7454	-0.4239	0.001	0.0012632	631	10867	73.396	102	1619	10.935	52	832	5.6193	8.2409	7.6562	50.9804	51.3897
SP4	FOXM1_1	cluster_2	DP	3187	4276	4138	4418	87.5705	0.7454	-0.4239	0.001	0.0010909	5181	41487	58.448	953	7417	10.449	401	3187	4.4899	7.7398	7.6819	42.0776	42.9689
SP8	PKNOX1_1	cluster_5	Common_1_DN	27414	36787.173	36207	37439	370.3804	0.7452	-0.4243	0.001	0.0010435	10991	165550	53.351	4839	69909	22.529	1847	27414	8.8346	16.8047	16.5593	38.169	39.2138
SP4	SOX10_9	cluster_2	DP	3551	4765.975	4581	4947	111.2226	0.7451	-0.4244	0.001	0.0010435	3684	51623	61.408	597	7939	9.4439	258	3551	4.2241	7.0033	6.8787	43.2161	44.7286
SP4	RUNX_1	cluster_5	Common_1_DN	61465	82496.955	81757	83281	479.8212	0.7451	-0.4246	0.001	0.0010435	22871	322027	50.309	12058	165555	25.864	4397	61465	9.6025	19.2252	19.0869	36.4654	37.1266
SP4	FOXO3_4	cluster_5	Common_1_DN	3665	4919.82	4728	5081	109.9039	0.745	-0.4247	0.001	0.0010909	2012	26245	62.811	658	8084	19.347	286	3665	8.7713	14.2147	13.9646	43.465	45.3365
DP	TBX4_1	cluster_3	DN	10327	13863.559	13639	14110	148.6002	0.7449	-0.4248	0.001	0.0010435	11484	80419	53.597	3769	26059	17.368	1482	10327	6.8826	12.9049	12.8415	39.3208	39.6293
SP4	SOX21_2	cluster_5	Common_1_DN	7701	10339.406	10082	10613	163.4686	0.7448	-0.425	0.001	0.0010909	3895	54547	61.419	1332	17389	19.58	571	7701	8.6711	14.6598	14.1181	42.8679	44.2866
SP4	FOXO6_1	cluster_2	DP	2737	3676.078	3522	3847	102.2554	0.7446	-0.4254	0.001	0.0010909	2688	35069	61.166	496	6186	10.789	212	2737	4.7738	7.8869	7.8046	42.7419	44.2451
SP4	TBX5_5	cluster_5	Common_1_DN	18294	24572.469	24037	25073	309.4891	0.7445	-0.4257	0.001	0.0010435	5992	114030	59.04	2353	42538	22.025	976	18294	9.4719	16.2884	16.0431	41.479	43.0063
SP4	IKZF1_1	cluster_5	Common_1_DN	17838	23965.748	23608	24293	219.1338	0.7443	-0.426	0.001	0.0010435	8843	106411	60.935	3496	40165	23	1511	17838	10.215	17.087	16.7633	43.2208	44.4118
SP4	TBX4_2	cluster_5	Common_1_DN	21785	29275.207	28780	29806	320.0351	0.7442	-0.4263	0.001	0.0010909	6921	131760	58.982	2786	50614	22.657	1161	21785	9.752	16.775	16.5338	41.6726	43.0415
SP4	HESX1_1	cluster_5	Common_1_DN	2087	2805.301	2688	2936	74.2044	0.744	-0.4265	0.001	0.0012632	1328	11959	60.289	556	4768	24.037	235	2087	10.521	17.6958	17.4513	42.2662	43.771
DN	ETV4_2	cluster_2	DP	4032	5419.659	5169	5679	141.911	0.744	-0.4266	0.001	0.0011429	5010	45275	26.285	2328	20847	12.103	449	4032	2.3408	8.9621	8.9056	19.2869	19.3409
SP8	NFE2L1::MAFG_1	cluster_2	DP	16077	21612.01	21114	22199	329.1417	0.7439	-0.4268	0.001	0.0010435	8430	177761	54.694	2037	40418	12.436	772	16077	4.9466	9.1578	9.0442	37.8989	39.7768
SP4	TCF7L1_2	cluster_2	DP	11013	14809.168	14495	15118	184.5294	0.7437	-0.4272	0.001	0.0010435	12702	140496	61.647	2272	24364	10.69	1004	11013	4.8323	7.9043	7.8387	44.1901	45.2019
DP	ETV5_1	cluster_5	Common_1_DN	13633	18333.454	18109	18559	136.6854	0.7436	-0.4273	0.001	0.0010435	12055	109704	76.51	2699	24074	16.79	1509	13633	9.5079	12.5176	12.4271	55.9096	56.6296
SP8	SPIC_2	cluster_5	Common_1_DN	33545	45113.601	44515	45727	368.8013	0.7436	-0.4275	0.001	0.0010435	15787	205822	55.49	6525	82309	22.191	2592	33545	9.0438	16.4186	16.2981	39.7241	40.755
SP8	STAT_known3	cluster_5	Common_1_DN	20677	27810.804	27326	28257	288.2319	0.7435	-0.4276	0.001	0.0010435	7066	141634	65.658	2289	43138	19.998	1053	20677	9.5853	14.9023	14.5989	46.0026	47.9322
SP4	IRF_known14	cluster_5	Common_1_DN	42612	57324.821	56677	58059	415.7971	0.7433	-0.4279	0.001	0.001	13966	293131	66.532	4593	89191	20.244	2089	42612	9.6716	14.9578	14.5368	45.4823	47.7761
SP4	TCF3_1	cluster_5	Common_1_DN	115390	155290.919	154162	156295	665.717	0.7431	-0.4285	0.001	0.0011429	45052	639694	51.735	21819	302495	24.464	8172	115390	9.3322	18.139	18.0383	37.4536	38.1461
SP4	POU2F2_known13	cluster_2	DP	3751	5051.682	4801	5302	142.4554	0.7426	-0.4294	0.001	0.0010909	2728	57442	64.703	436	8267	9.312	187	3751	4.2251	6.8548	6.5301	42.8899	45.3732
SP4	TBX21_6	cluster_5	Common_1_DN	23810	32072.566	31575	32541	304.9447	0.7424	-0.4298	0.001	0.0010909	8330	150313	59.065	3242	55459	21.793	1336	23810	9.3561	16.0384	15.8403	41.2091	42.9326
SP8	MYBL2_2	cluster_5	Common_1_DN	5074	6835.939	6624	7034	123.8105	0.7423	-0.4299	0.001	0.0010435	2285	34653	64.344	738	10773	20.003	339	5074	9.4214	14.8359	14.6423	45.935	47.0992
SP4	MAF_known1	cluster_5	Common_1_DN	68300	92017.845	91184	92762	508.4631	0.7423	-0.43	0.001	0.001	26418	399043	58.146	11086	160598	23.401	4567	68300	9.9522	17.2875	17.1159	41.1961	42.5285
SP4	TBX15_2	cluster_5	Common_1_DN	17748	23917.783	23633	24210	183.3309	0.7421	-0.4304	0.001	0.0010909	14978	104871	54.579	6406	44116	22.96	2550	17748	9.2367	17.025	16.9236	39.8064	40.2303
SP8	REL_2	cluster_5	Common_1_DN	16760	22587.699	22240	22922	202.266	0.742	-0.4305	0.001	0.0010435	12473	112795	62.62	4147	36451	20.236	1874	16760	9.3045	15.0245	14.8588	45.1893	45.9795
DN	ETV1_1	cluster_2	DP	4068	5483.549	5255	5759	155.5201	0.7419	-0.4307	0.001	0.0010909	5090	46038	26.319	2353	21073	12.047	453	4068	2.3256	8.8998	8.8362	19.252	19.3043

SP4	TBX21_4	cluster_5	Common_1_DN	18773	25304.697	24857	25749	284.7069	0.7419	-0.4307	0.001	0.0010909	7892	118588	58.407	3068	44178	21.759	1271	18773	9.2461	16.1049	15.8304	41.4276	42.494
DP	HES1_1	cluster_5	Common_1_DN	57147	77035.7	76366	77785	434.5555	0.7418	-0.4308	0.001	0.001	24843	352037	63.079	9001	123465	22.123	4076	57147	10.24	16.407	16.2332	45.2839	46.286
SP8	ATF7_1	cluster_5	Common_1_DN	6251	8427.559	8198	8654	147.635	0.7418	-0.431	0.001	0.001	3427	44593	61.045	1102	13946	19.091	485	6251	8.5573	14.1523	14.0179	44.0109	44.8229
DP	TCF7L1_2	cluster_3	DN	12835	17309.448	16891	17694	236.8432	0.7415	-0.4314	0.001	0.0010435	10535	116448	51.095	3198	34366	15.079	1172	12835	5.6317	11.1248	11.0221	36.6479	37.348
SP4	SMAD3_1	cluster_5	Common_1_DN	28573	38533.328	38115	38934	261.7491	0.7415	-0.4314	0.001	0.0010435	20754	171916	58.449	8111	66435	22.587	3450	28573	9.7144	16.6233	16.6203	42.5348	43.009
DP	EGR3_3	cluster_5	Common_1_DN	58582	79006.513	78331	79628	379.975	0.7415	-0.4315	0.001	0.001	31417	463984	71.003	7938	112982	17.29	4036	58582	8.9648	12.8465	12.6259	50.844	51.8507
DP	ERG_4	cluster_5	Common_1_DN	13220	17831.115	17486	18158	209.2662	0.7414	-0.4316	0.001	0.001	6744	87870	61.259	2327	29418	20.509	1019	13220	9.2163	15.1097	15.045	43.7903	44.9385
DN	RUNX1_2	cluster_1	SP8	6499	8768.564	8538	8980	134.4276	0.7412	-0.4321	0.001	0.0010435	5376	83982	74.445	859	12459	11.044	431	6499	5.761	8.0171	7.7386	50.1746	52.1631
SP4	IKZF3_1	cluster_5	Common_1_DN	13156	17750.778	17411	18098	202.9406	0.7412	-0.4321	0.001	0.0010435	6633	79768	59.129	2666	30716	22.768	1115	13156	9.752	16.8099	16.4928	41.823	42.8311
SP4	REL_2	cluster_5	Common_1_DN	16986	22929.136	22624	23257	197.0176	0.7408	-0.4328	0.001	0.0010435	12661	114508	63.571	4147	36451	20.236	1895	16986	9.43	14.9672	14.8339	45.6957	46.5995
DP	ETV4_2	cluster_5	Common_1_DN	6168	8326.63	8046	8609	166.241	0.7408	-0.4329	0.001	0.0011429	5095	46084	26.755	3500	31215	18.122	685	6168	3.5809	13.4446	13.3843	19.5714	19.7597
SP8	SOX10_9	cluster_2	DP	3253	4392.208	4182	4612	126.4268	0.7407	-0.4331	0.001	0.0010435	3412	47797	56.857	597	7939	9.4439	235	3253	3.8696	6.8875	6.8059	39.3635	40.9749
SP4	SPIC_1	cluster_5	Common_1_DN	33763	45588.781	45019	46106	346.0524	0.7406	-0.4332	0.001	0.001	16203	211420	60.042	6133	77072	21.888	2615	33763	9.5885	16.139	15.9696	42.6382	43.8071
SP4	TBX2_1	cluster_5	Common_1_DN	23686	31982.286	31452	32529	316.5422	0.7406	-0.4332	0.001	0.0010435	8640	147190	60.352	3274	53684	22.012	1406	23686	9.7119	16.2731	16.0921	42.9444	44.1212
SP8	RUNX_2	cluster_5	Common_1_DN	51730	69845.683	68997	70528	468.4782	0.7406	-0.4332	0.001	0.0010435	19635	276608	53.223	9707	132568	25.508	3702	51730	9.9536	18.8541	18.7016	38.1374	39.0215
SP4	PKNOX2_2	cluster_5	Common_1_DN	21772	29411.074	28897	29912	287.6696	0.7403	-0.4339	0.001	0.0010435	11364	126178	51.858	5291	57301	23.55	1975	21772	8.948	17.3794	17.255	37.3275	37.9958
DP	TCF4_1	cluster_3	DN	68068	91990.402	91385	92704	418.4046	0.7399	-0.4345	0.001	0.0010909	39369	356288	54.334	18932	169953	25.918	7519	68068	10.38	19.0988	19.1048	39.7158	40.0511
SP8	SOX5_3	cluster_2	DP	2308	3119.551	2943	3288	105.2031	0.7399	-0.4345	0.001	0.0011429	2486	37371	63.453	366	5129	8.7086	159	2308	3.9188	6.3958	6.1759	43.4426	44.999
SP4	SOX7_4	cluster_5	Common_1_DN	6160	8328.978	8032	8592	162.4314	0.7396	-0.4351	0.001	0.0010909	2839	45561	60.885	969	14283	19.087	403	6160	8.2319	14.1951	13.5203	41.5893	43.1282
SP8	FOXO3_4	cluster_5	Common_1_DN	3448	4663.966	4507	4846	104.3791	0.7393	-0.4357	0.001	0.0010909	1903	24809	59.374	658	8084	19.347	271	3448	8.252	14.2407	13.8982	41.1854	42.6522
SP8	FOXO3_4	cluster_2	DP	1693	2290.197	2154	2416	81.8948	0.7394	-0.4357	0.001	0.0010909	1903	24809	59.374	319	3951	9.4558	133	1693	4.0518	6.989	6.8241	41.6928	42.8499
SP8	SOX4_2	cluster_2	DP	4722	6387.027	6146	6617	138.8769	0.7394	-0.4357	0.001	0.0010909	4506	67750	58.989	791	11178	9.7326	319	4722	4.1114	7.0794	6.9697	40.3287	42.2437
SP8	ATF4_2	cluster_5	Common_1_DN	7104	9612.001	9336	9882	154.589	0.7391	-0.4362	0.001	0.0010909	3830	46012	56.648	1471	17153	21.118	597	7104	8.7461	15.5875	15.4395	40.5846	41.4155
DP	TCF3_2	cluster_3	DN	79792	107977.04	107043	108940	580.7484	0.739	-0.4364	0.001	0.0010435	27591	417427	54.427	13375	199427	26.003	5275	79792	10.404	19.1186	19.1152	39.4393	40.0106
SP8	REL_1	cluster_5	Common_1_DN	16404	22199.972	21942	22493	175.2767	0.7389	-0.4365	0.001	0.001	12386	112079	62.586	4073	35821	20.003	1833	16404	9.1601	14.799	14.6361	45.0037	45.7944
SP4	SOX10_4	cluster_2	DP	6214	8412.227	8147	8697	163.4404	0.7387	-0.4369	0.001	0.0010909	5739	86325	61.805	973	13974	10.005	419	6214	4.4489	7.3009	7.1984	43.0627	44.4683
SP8	TBX2_2	cluster_2	DP	7737	10474.444	10195	10750	157.242	0.7387	-0.437	0.001	0.0010909	9067	90775	56.983	1906	18653	11.709	778	7737	4.8568	8.5806	8.5233	40.8185	41.4786
SP4	REL_1	cluster_5	Common_1_DN	16601	22478.079	22204	22818	189.5617	0.7386	-0.4372	0.001	0.001	12552	113599	63.434	4073	35821	20.003	1851	16601	9.2701	14.7467	14.6137	45.4456	46.3443
DP	EGR3_2	cluster_5	Common_1_DN	87193	118068.654	117371	118891	478.6214	0.7385	-0.4373	0.001	0.001	45781	699461	71.099	11617	169240	17.203	5836	87193	8.863	12.7476	12.4657	50.2367	51.5203
SP4	PBX3_known1	cluster_2	DP	5538	7502.182	7234	7729	138.4464	0.7382	-0.4379	0.001	0.0010435	7076	78591	58.439	1236	13124	9.7589	506	5538	4.118	7.1509	7.0466	40.9385	42.1975
SP8	SOX11_2	cluster_2	DP	3364	4557.414	4388	4741	109.4931	0.7382	-0.4379	0.001	0.0010909	3666	51361	60.532	589	7774	9.1621	243	3364	3.9646	6.6285	6.5497	41.2564	43.2724
SP8	PBX1_2	cluster_5	Common_1_DN	5877	7964.786	7728	8209	144.2258	0.7379	-0.4385	0.001	0.0010435	2880	41061	58.486	1057	14017	19.965	422	5877	8.371	14.6528	14.3129	39.9243	41.9277
SP4	EWSR1::FLI1_1	cluster_5	Common_1_DN	152012	206013.794	204668	207408	862.5423	0.7379	-0.4386	0.001	0.0010435	47664	952228	58.406	19564	364049	22.329	7820	152012	9.3239	16.4065	15.9638	39.9714	41.7559
SP8	SOX5_1	cluster_2	DP	1426	1933.598	1839	2030	57.8517	0.7376	-0.439	0.001	0.0010909	2249	20248	60.174	373	3266	9.7061	161	1426	4.2379	7.1587	7.0427	43.1635	43.662
SP4	RBPJ_2	cluster_5	Common_1_DN	17468	23685.993	23325	24051	217.2549	0.7375	-0.4393	0.001	0.0010909	10316	103270	57.388	4334	41855	23.259	1764	17468	9.7071	17.0997	16.9149	40.7014	41.7346
SP8	ELK3_1	cluster_2	DP	4994	6772.012	6523	7036	158.0905	0.7375	-0.4393	0.001	0.0011429	5938	53644	24.833	3057	27373	12.671	554	4994	2.3118	9.3297	9.3095	18.1223	18.2443
SP8	SOX1_3	cluster_2	DP	2786	3778.159	3601	3976	109.5395	0.7375	-0.4394	0.001	0.0010909	2972	41649	55.348	533	7047	9.3648	203	2786	3.7023	6.8304	6.6892	38.0863	39.5346
DP	ERG_2	cluster_5	Common_1_DN	12917	17519.694	17179	17880	213.9704	0.7373	-0.4397	0.001	0.001	6712	87427	60.427	2323	29323	20.267	995	12917	8.9278	14.8242	14.7746	42.8325	44.0507
DP	ETV6_2	cluster_5	Common_1_DN	13520	18338.292	17921	18739	247.621	0.7373	-0.4397	0.001	0.0011429	9680	87477	23.63	8770	77969	21.062	1502	13520	3.6522	15.5165	15.4555	17.1266	17.3402
SP8	E2F3_3	cluster_5	Common_1_DN	5305	7195.524	6925	7423	150.8535	0.7373	-0.4397	0.001	0.001	2069	35210	66.193	696	11157	20.975	321	5305	9.9731	15.5147	15.0667	46.1207	47.5486
SP8	ELK4_2	cluster_2	DP	6038	8190.194	7912	8466	173.442	0.7373	-0.4398	0.001	0.0011429	7080	64048	23.72	3873	34667	12.839	670	6038	2.2362	9.4633	9.4273	17.2993	17.4171
DP	ID4_1	cluster_3	DN	58318	79111.065	78518	79751	383.785	0.7372	-0.4399	0.001	0.0010435	35081	317583	54.366	16281	146125	25.015	6432	58318	9.9833	18.3347	18.3631	39.5062	39.9097
SP4	RUNX_2	cluster_5	Common_1_DN	49261	66837.248	66081	67559	450.6621	0.737	-0.4402	0.001	0.0010435	18786	264509	50.895	9707	132568	25.508	3529	49261	9.4785	18.7853	18.6236	36.3552	37.159
SP8	ETV4_2	cluster_2	DP	3833	5201.138	4951	5423	134.5704	0.737	-0.4403	0.001	0.0011429	4775	43196	25.078	2328	20847	12.103	425	3833	2.2253	8.9005	8.8735	18.256	18.3863
DP	PKNOX1_2	cluster_3	DN	16181	21967.11	21576	22412	260.8148	0.7366	-0.441	0.001	0.0010435	9569	106189	50.266	4017	43989	20.823	1459	16181	7.6595	15.2472	15.2379	36.3206	36.7842
SP4	POU2F2_known16	cluster_5	Common_1_DN	5007	6797.355	6571	6996	129.9818	0.7366	-0.441	0.001	0.0010909	2634	34687	58.999	965	11945	20.317	387	5007	8.5163	14.6925	14.4348	40.1036	41.9171
SP4	TCF7L1_2	cluster_5	Common_1_DN	20655	28040.262	27638	28441	235.1123	0.7366	-0.441	0.001	0.0010435	12702	140496	61.647	4326	46203	20.273	1894	20655	9.063	14.911	14.7015	43.7818	44.7049
SP4	MAFG_1	cluster_5	Common_1_DN	21271	28885.426	28345	29446	337.5229	0.7364	-0.4414	0.001	0.0010435	6447	129183	56.562	2762	52068	22.798	1077	21271	9.3133	16.7054	16.4658	38.9935	40.8523
DP	ELK4_2	cluster_5	Common_1_DN	10040	13638.221	13242	13986	228.2706	0.7362	-0.4419	0.001	0.0011429	7680	69469	25.728	5965	53260	19.725	1116	10040	3.7183	14.5312	14.4525	18.7091	18.8509
SP8	ARID5B_1	cluster_2	DP	4071	5530.432	5345	5738	122.1282	0.7362	-0.4419	0.001	0.0010909	4547	59233	58.39	781	9708	9.5698	320	4071	4.0131	7.0376	6.8729	40.9731	41.9345
SP8	MTF1_3	cluster_5	Common_1_DN	26134	35502.736	35072	35953	271.4302	0.7361	-0.442	0.001	0.001	12742	167257	61.78	4568	58056	21.444	2006	26134	9.6531	15.7432	15.6251	43.9142	45.0152
SP4	FOXO6_3	cluster_5	Common_1_DN	45262	61501.344	60912	62136	365.5192	0.736	-0.4423	0.001	0.0010435	21804	285200	58.247	8509	107163	21.886	3506	45262	9.2439	16.0796	15.8703	41.2034	42.2366
DP	FLI1_4	cluster_5	Common_1_DN	13434	18255.487	17920	18609	215.8252	0.7359	-0.4424	0.001	0.001	7089	92416	63.019	2313	29272	19.961	1036	13434	9.1608	14.6142	14.5364	44.7903	45.8937
SP4	SPI1_known1	cluster_5	Common_1_DN	44536	60524.006	59992	60985	295.801	0.7358	-0.4425	0.001	0.001	38052	267768	57.339	15325	106225	22.747	6357	44536	9.5368	16.7061	16.6323	41.4812	41.9261
SP8	MYC_known13	cluster_5	Common_1_DN	16147	21957.596	21584	22383	228.5807	0.7354	-0.4434	0.001	0.0010909	9016	99281	55.743	3715	39745	22.315	1481	16147	9.0659	16.4264	16.2639	39.8654	40.6265
SP8	SP1_known3	cluster_5	Common_1_DN	77890	105919.308	105136	106722	480.0036	0.7354	-0.4435	0.001	0.001	39576	519194	65.135	13072	164835	20.679	6014	77890	9.7717	15.1961	15.0021	46.0067	47.2533
SP4	SOX1_2	cluster_2	DP	3568	4852.829	4625	5076	131.3069	0.7353	-0.4436	0.001	0.0010909	3418	47871	59.739	621	8368	10.443	260	3568	4.4525	7.6068	7.4534	41.868	42.6386
SP8	SOX2_6	cluster_2	DP	2114	2876.056	2682	3065	109.6932	0.7351	-0.4439	0.001	0.0010435	1941	31108	57.681	347	5211	9.6623	134	2114	3.9198	6.9037	6.7957	38.6167	40.568
DP	FOXP3_1	cluster_3	DN	13772	18737.174	18268	19194	275.5489	0.735	-0.4441	0.001	0.0010909	7025	112814	50.937	2429	37397	16.885	869	13772	6.2183	12.3701	12.2077	35.776	36.8265
DP	SOX15_4	cluster_3	DN	3095	4211.362	4035	4396	112.368	0.735	-0.4442	0.001	0.0011429	1805	25291	45.839	715	9431	17.094	223	3095	5.6096	12.3546	12.2376	31.1888	32.8173
SP8	SOX15_4	cluster_2	DP	1943	2643.992	2492	2790	94.8617	0.735	-0.4442	0.001	0.0011429	2193	30727	55.692	374	4898	8.8775	142	1943	3.5217	6.4751	6.3234	37.9679	39.6693
DN	GATA_known17	cluster_1	SP8	5665	7709.646	7480	7917	138.1015	0.7348	-0.4445	0.001	0.0010909	5009	80296	75.449	690	10533	9.8972	363	5665	5.323	7.247	7.0551	52.6087	53.7833
DP	SP1_known2	cluster_5	Common_1_DN	211407	287709.35	286427	288893	776.0175	0.7348	-0.4446	0.001	0.001	115319	1604370	70.792	31582	415613	18.339	15731	211407	9.3282	13.6413	13.1769	49.81	50.8663
SP4	NFKB_known11	cluster_5	Common_1_DN	21450	29199.025	28782	29567	241.9483	0.7346	-0.4449	0.001	0.001	11939	145036	63.244	3973	46680	20.355	1781	21450	9.3535	14.9175	14.7894	44.8276	45.9512
SP8	SPDEF_6	cluster_5	Common_1_DN	17371	23646.006	23291	24001	220.3982	0.7346	-0.4449	0.001	0.001	8460	118816	68.647	2581	34933	20.183	1251	17371	10.036	14.7872	14.6201	48.4696	49.7266
DP	ETV6_2	cluster_4	SP4	7017	9552.775	9229	9860	189.1317	0.7346	-0.445	0.001	0.0011429	9680	87477	23.63	4569	40741	11.006	779	7017	1.8955	8.0475	8.0215	17.0497	17.2234
SP4	ATF3_known5	cluster_5	Common_1_DN	26421	35969.304	35455	36548	333.844	0.7346	-0.4451	0.001	0.001	11915	167390	58.274	4636	62555	21.777	1899	26421	9.198	15.9379	15.7841	40.962	42.2364
DP	RUNX_1	cluster_4	SP4	15999	21785.151	21293	22304	298.2752	0.7344	-0.4453	0.001	0.0010435	21137	297645	46.5	3468	47492	7.4195	1144	15999	2.4995	5.4123	5.3752	32.9873	33.6878
DP	TBX21_4	cluster_3	DN	13170	17932.66	17499	18402	271.3069	0.7344	-0.4453	0.001	0.0010909	7045	105923	52.17	2406	34959	17.218	882	13170	6.4865	12.5195	12.4336	36.6584	37.6727
DN	SPIC_2	cluster_1	SP8	17755	24176.789	23873	24524	194.9167	0.7344	-0.4454	0.001	0.0010435	21125	275317	74.226	2615	33138	8.934	1376	17755	4.7868	6.5136	6.4489	52.6195	53.579
DP	MYCN_2	cluster_5	Common_1_DN	24360	33180.572	32805	33569	228.636	0.7342	-0.4458	0.001	0.001	17531	158428	65.545	5735	50848	21.037	2711	24360	10.078	15.464	15.3761	47.2711	47.9075
SP8	RUNX1_2	cluster_2	DP	4048	5514.337	5303	5718	131.2143	0.7341	-0.4459	0.001	0.0010435	4117	64095	56.816	694	10049	8.9078	269	4048	3.5883	6.5339	6.3156	38.7608	40.2826
SP8	ATF4_3	cluster_2	DP	3520	4796.657	4591	4984	125.9006	0.7339	-0.4463	0.001	0.0010909	3790	49324	56.677	685	8615	9.8993	276	3520	4.0448	7.2823	7.1365	40.292	40.859
SP8	SPIB_2	cluster_5	Common_1_DN	36569	49850.689	49287	50493	354.5366	0.7336	-0.447	0.001	0.0010435	17242	225293	55.874	7171	90414	22.423	2830	36569	9.0694	16.4134	16.2318	39.4645	40.4462
SP4	GATA_known17	cluster_5	Common_1_DN	10062	13717.874	13415	14061	203.5093	0.7335	-0.4471	0.001	0.0010909	4062	65192	61.257	1511	22926	21.542	635	10062	9.4546	15.6327	15.4344	42.0251	43.889
SP4	NFATC1_1	cluster_5	Common_1_DN	14950	20384.008	19947	20781	266.2714	0.7334	-0.4473	0.001	0.0010909	5359	103439	64.397	1853	32944	20.51	806	14950	9.3073	15.0401	14.453	43.497	45.38
SP8	STAT_known2	cluster_5	Common_1_DN	26593	36263.371	35771	36835	313.3552	0.7333	-0.4475	0.001	0.001	8911	178852	67.814	2866	54457	20.648	1352	26593	10.083	15.1723	14.8687	47.1738	48.833
SP4	TBX4_1	cluster_5	Common_1_DN	13711	18701.771	18448	18959	153.7091	0.7332	-0.4478	0.001	0.0010435	11905	83358	55.556	4930	33931	22.614	1970	13711	9.138	16.5477	16.4483	39.9594	40.4085
DN	TCF7L1_1	cluster_1	SP8	17458	23816.836	23407	24247	242.7896	0.733	-0.4481	0.001	0.0010435	12339	198117	70.718	2221	34617	12.357	1098	17458	6.2316	8.8986	8.812	49.4372	50.4319
SP4	ATF7_1	cluster_2	DP	2885	3936.436	3760	4115	99.7988	0.733	-0.4482	0.001	0.001	3484	45358	62.093	503	6389	8.7462	224	2885	3.9494	6.4294	6.3605	44.5328	45.1557
DP	ELK3_1	cluster_1	SP8	2426	3311.202	3108	3506	117.5991	0.7327	-0.4486	0.001	0.0011429	6415	57946	26.824	1397	12448	5.7624	270	2426	1.123	4.2089	4.1867	19.3271	19.4891
SP4	SPI1_known4	cluster_5	Common_1_DN	32411	44231.019	43644	44723	316.006	0.7328	-0.4486	0.001	0.0010435	15528	202516	62.03	5779	72542	22.219	2516	32411	9.9274	16.203	16.0042	43.5369	44.6789
SP4	SPZ1_2	cluster_5	Common_1_DN	23420	31965.961	31528	32402	259.7676	0.7327	-0.4488	0.001	0.0010435	14241	142725	57.95	5732	55792	22.653	2357	23420	9.509	16.5508	16.4092	41.12	41.9773
SP8	ELK3_2	cluster_2	DP	4788	6535.353	6217	6796	175.5981	0.7327	-0.4488	0.001	0.0011429	5710	51570	24.526	2993	26781	12.737	532	4788	2.2771	9.317	9.2845	17.7748	17.8783
SP4	NFKB_known10	cluster_5	Common_1_DN	19311	26360.075	25964	26734	238.4585	0.7326	-0.4489	0.001	0.001	10445	126213	62.637	3651	42572	21.128	1613	19311	9.5836	15.4428	15.3003	44.1797	45.3608
SP4	NFATC1_3	cluster_5	Common_1_DN	6456	8815.714	8555	9088	158.6142	0.7324	-0.4494	0.001	0.0010909	3149	40963	60.391	1232	15103	22.266	509	6456	9.5179	16.1639	15.7606	41.3149	42.7465
SP4	SOX9_4	cluster_2	DP	5758	7866.465	7591	8145	161.7949	0.732	-0.4501	0.001	0.0010909	5343	80252	62.068	910	13004	10.058	390	5758	4.4533	7.2993	7.1749	42.8571	44.2787
SP4	SOX21_2	cluster_2	DP	3874	5293.574	5111	5505	124.8528	0.7319	-0.4503	0.001	0.0010909	3895	54547	61.419	668	8902	10.023	281	3874	4.362	7.2144	7.1021	42.0659	43.5183
SP4	ARID5B_1	cluster_5	Common_1_DN	9229	12611.025	12323	12900	172.5121	0.7318	-0.4504	0.001	0.0010909	4639	60416	59.556	1743	21717	21.408	719	9229	9.0976	15.499	15.2758	41.2507	42.4967
DP	SMAD_2	cluster_3	DN	25816	35279.587	34777	35825	317.727	0.7318	-0.4506	0.001	0.0010435	20655	212873	53.488	6601	66737	16.769	2511	25816	6.4868	12.1569	12.1274	38.0397	38.6832
DP	ETV7_1	cluster_1	SP8	13051	17839.845	17487	18199	212.9312	0.7316	-0.4509	0.001	0.001	25386	228951	51.568	3931	34860	7.8518	1449	13051	2.9396	5.7079	5.7003	36.8608	37.4383
SP4	GMEB2_2	cluster_5	Common_1_DN	5339	7300.254	7084	7536	131.0599	0.7314	-0.4513	0.001	0.001	2773	36777	66.35	866	11140	20.098	402	5339	9.6321	14.4969	14.5172	46.4203	47.9264
SP8	PATZ1_1	cluster_5	Common_1_DN	136568	186747.805	185765	187765	625.2055	0.7313	-0.4515	0.001	0.001	74077	975342	66.217	23069	288137	19.562	10650	136568	9.2717	14.3769	14.0021	46.1659	47.3969
DP	TBX5_5	cluster_3	DN	12699	17375.885	16928	17811	266.3035	0.7309	-0.4523	0.001	0.0010435	5461	103978	53.836	1794	32801	16.983	672	12699	6.5751	12.3054	12.2132	37.4582	38.7153
SP4	NFE2L1::MAFG_1	cluster_5	Common_1_DN	29809	40789.831	40115	41449	440.2306	0.7308	-0.4524	0.001	0.0010435	8781	185151	56.968	3722	73540	22.627	1439	29809	9.1717	16.3877	16.0998	38.662	40.5344
SP4	TCF7_1	cluster_5	Common_1_DN	26979	36916.46	36408	37495	320.9146	0.7308	-0.4524	0.001	0.0010435	11748	188705	61.707	4000	61044	19.962	1701	26979	8.8222	14.4791	14.2969	42.525	44.196
DP	MYCN_1	cluster_5	Common_1_DN	16854	23067.973	22712	23424	217.3784	0.7306	-0.4528	0.001	0.001	10303	113712	63.225	3425	36786	20.453	1537	16854	9.3709	14.918	14.8217	44.8759	45.8163
SP4	MAF_known11	cluster_5	Common_1_DN	29292	40098.177	39480	40610	352.7264	0.7305	-0.453	0.001	0.0010435	10831	174705	57.034	4559	70796	23.112	1803	29292	9.5626	16.6467	16.7665	39.5481	41.3752
SP4	SOX14_2	cluster_2	DP	2764	3784.58	3622	3955	98.583	0.7304	-0.4532	0.001	0.0010909	3491	38572	60.257	603	6367	9.9466	254	2764	4.3179	7.2759	7.1658	42.1227	43.4113
SP8	CUX2_2	cluster_2	DP	153	209.925	179	238	17.4235	0.7301	-0.4538	0.001	0.0013333	381	3429	70.686	36	310	6.3904	17	153	3.154	4.4619	4.4619	47.2222	49.3548
DP	ATF2_1	cluster_5	Common_1_DN	1075	1473.467	1405	1540	41.4822	0.7298	-0.4545	0.001	0.0010435	1296	9072	66.75	323	2222	16.349	155	1075	7.9096	11.9599	11.8496	47.9876	48.3798
SP4	PKNOX2_3	cluster_5	Common_1_DN	18540	25408.014	25018	25877	266.1168	0.7297	-0.4546	0.001	0.0010435	10156	113004	52.742	4498	48655	22.709	1677	18540	8.6532	16.5124	16.4065	37.2832	38.105
SP4	E2F8_1	cluster_5	Common_1_DN	12122	16615.422	16346	16866	164.2489	0.7296	-0.4549	0.001	0.001	7471	82374	63.953	2499	26482	20.56	1124	12122	9.4113	15.0448	14.7158	44.978	45.7745
DN	SOX18_4	cluster_1	SP8	3019	4138.903	3991	4298	94.1976	0.7295	-0.455	0.001	0.0012	2707	37982	72.115	451	5993	11.379	218	3019	5.732	8.0532	7.9485	48.337	50.3754
SP8	KLF13_1	cluster_5	Common_1_DN	53664	73563.693	72774	74377	466.8074	0.7295	-0.455	0.001	0.001	20972	360883	65.363	6957	114292	20.701	3162	53664	9.7196	15.0772	14.8702	45.4506	46.9534
SP4	POU2F2_known1	cluster_5	Common_1_DN	8718	11951.372	11650	12255	187.9474	0.7295	-0.4551	0.001	0.0010435	3339	60392	62.523	1186	19829	20.529	498	8718	9.0257	14.9146	14.4357	41.9899	43.9659
SP4	ATF2_1	cluster_2	DP	490	672.37	627	714	25.7262	0.7292	-0.4557	0.001	0.0010435	1377	9639	70.922	137	959	7.0561	70	490	3.6053	5.0835	5.0835	51.0949	51.0949
SP4	TCF7_2	cluster_5	Common_1_DN	21359	29298.896	28958	29648	214.6306	0.729	-0.456	0.001	0.0010435	13256	146350	61.849	4487	48020	20.294	1957	21359	9.0265	14.7631	14.5945	43.6149	44.4794
DP	ELK3_2	cluster_1	SP8	2372	3254.488	3061	3423	108.1082	0.7289	-0.4562	0.001	0.0011429	6179	55790	26.533	1388	12358	5.8774	264	2372	1.1281	4.2725	4.2517	19.0202	19.194
DP	ETS_known13	cluster_5	Common_1_DN	24223	33237.484	32765	33718	291.6695	0.7288	-0.4564	0.001	0.001	10314	165505	67.697	3191	49600	20.288	1520	24223	9.908	14.7373	14.6358	47.634	48.8367
SP4	PKNOX1_2	cluster_5	Common_1_DN	18353	25185	24749	25602	249.688	0.7287	-0.4565	0.001	0.0010435	9911	109901	52.023	4531	48957	23.175	1666	18353	8.6877	16.8096	16.6996	36.7689	37.488
SP8	ETV7_1	cluster_5	Common_1_DN	37666	51686.572	51143	52220	338.419	0.7287	-0.4565	0.001	0.001	25471	229674	51.731	11316	100370	22.607	4191	37666	8.4838	16.454	16.3998	37.0361	37.5271
DP	TCF21_1	cluster_3	DN	27095	37194.152	36637	37727	330.5579	0.7285	-0.457	0.001	0.001	11430	148923	52.886	5540	70941	25.193	2082	27095	9.6221	18.2152	18.194	37.5812	38.1937
DP	CUX1_8	cluster_3	DN	694	953.678	847	1051	65.7541	0.728	-0.458	0.001	0.0011429	409	6571	49.473	143	2072	15.6	46	694	5.2251	11.2469	10.5616	32.1678	33.4942
DN	GATA_known16	cluster_1	SP8	8410	11553.519	11215	11852	199.0593	0.7279	-0.4581	0.001	0.0010435	6076	128052	74.197	822	16180	9.3751	410	8410	4.873	6.7479	6.5676	49.8783	51.9778
DP	ATF6_1	cluster_5	Common_1_DN	3734	5130.685	4990	5278	86.484	0.7278	-0.4583	0.001	0.0010435	3671	25707	59.612	1255	8634	20.021	535	3734	8.6588	14.5737	14.5252	42.6295	43.2476
SP4	POU2F2_known5	cluster_5	Common_1_DN	7597	10438.484	10154	10713	158.093	0.7278	-0.4584	0.001	0.0010909	3860	50256	61.466	1410	17442	21.333	598	7597	9.2916	15.4922	15.1166	42.4113	43.5558
DP	TBX5_3	cluster_3	DN	17450	23995.435	23591	24375	227.6464	0.7272	-0.4595	0.001	0.001	14895	134213	54.181	5059	44697	18.044	1948	17450	7.0444	13.0782	13.0017	38.5056	39.0407
SP4	FOXO4_3	cluster_5	Common_1_DN	3956	5440.178	5249	5628	109.5523	0.7272	-0.4595	0.001	0.0010909	2238	29139	62.034	731	9058	19.283	310	3956	8.4219	13.8517	13.5763	42.4077	43.6741
DP	SOX9_6	cluster_3	DN	5997	8255.611	7953	8573	190.3262	0.7264	-0.4611	0.001	0.0010909	3625	54432	49.299	1215	17231	15.606	406	5997	5.4314	11.2	11.0174	33.4156	34.8036
DP	SOX8_2	cluster_3	DN	7519	10352.323	10027	10677	196.7318	0.7263	-0.4613	0.001	0.0010909	4342	65204	50.995	1449	20773	16.246	506	7519	5.8805	11.6536	11.5315	34.9206	36.196
DP	ATF2_2	cluster_5	Common_1_DN	7616	10488.198	10269	10735	132.3629	0.7262	-0.4616	0.001	0.0010435	4893	53941	62.848	1556	16788	19.56	695	7616	8.8736	14.204	14.1191	44.6658	45.3657
SP8	SOX18_4	cluster_2	DP	2025	2789.403	2641	2958	96.2641	0.7261	-0.4618	0.001	0.0012	2159	30301	57.531	385	5045	9.5787	149	2025	3.8448	6.9013	6.6829	38.7013	40.1388
SP4	TBX1_4	cluster_5	Common_1_DN	26223	36122.25	35493	36708	379.291	0.726	-0.462	0.001	0.0010909	8929	170354	58.182	3505	63358	21.639	1392	26223	8.9561	15.5897	15.3932	39.7147	41.3886
SP4	POU2F2_known15	cluster_5	Common_1_DN	4898	6749.348	6560	6949	113.6253	0.7257	-0.4625	0.001	0.0010435	3260	36005	62.683	1058	11078	19.286	455	4898	8.5272	13.9571	13.6037	43.0057	44.2138

DP	SOX10_9	cluster_3	DN	4504	6207.254	5955	6438	147.0385	0.7256	-0.4627	0.001	0.0010435	2814	39418	46.89	1017	13538	16.104	327	4504	5.3578	11.6205	11.4263	32.1534	33.2693
DP	SOX21_3	cluster_3	DN	5261	7252.349	6967	7565	183.1186	0.7255	-0.463	0.001	0.0010909	3002	45066	51.174	1020	14522	16.49	355	5261	5.9741	11.8254	11.674	34.8039	36.2278
DN	FOXO1_4	cluster_1	SP8	3110	4287.657	4140	4436	88.2345	0.7254	-0.4631	0.001	0.0010909	2797	36450	73.199	492	6089	12.228	243	3110	6.2455	8.6879	8.5322	49.3902	51.0757
SP8	FOXP3_1	cluster_2	DP	9495	13089.034	12774	13520	219.0084	0.7254	-0.4631	0.001	0.0010909	7647	122767	55.431	1558	23954	10.816	587	9495	4.2871	7.6762	7.7342	37.6765	39.6385
SP4	ATF7_1	cluster_5	Common_1_DN	6210	8562.64	8356	8805	133.9222	0.7253	-0.4634	0.001	0.001	3484	45358	62.093	1102	13946	19.091	480	6210	8.5011	13.7773	13.6911	43.5572	44.5289
DP	MAFG_1	cluster_3	DN	14505	20004.442	19516	20543	313.768	0.7251	-0.4637	0.001	0.0010435	5947	119184	52.184	2020	38737	16.961	729	14505	6.3509	12.2583	12.1703	36.0891	37.4448
SP8	BATF_known1	cluster_5	Common_1_DN	7455	10281.1	10055	10519	146.3725	0.7251	-0.4637	0.001	0.0010435	3843	50078	57.839	1417	17961	20.745	575	7455	8.6104	14.9623	14.8868	40.5787	41.5066
DN	ETV7_1	cluster_1	SP8	15758	21733.361	21393	22072	205.637	0.7251	-0.4638	0.001	0.001	30877	278376	62.701	3931	34860	7.8518	1753	15758	3.5493	5.6774	5.6607	44.5943	45.2037
SP4	SPIC_2	cluster_5	Common_1_DN	35783	49363.308	48854	49934	351.8651	0.7249	-0.4642	0.001	0.0010435	17268	225108	60.689	6525	82309	22.191	2767	35783	9.6471	16.0239	15.8959	42.4061	43.474
DP	FLI1_2	cluster_5	Common_1_DN	12578	17361.047	17030	17716	201.3084	0.7245	-0.4649	0.001	0.001	6679	86994	61.881	2240	28311	20.138	970	12578	8.947	14.5231	14.4585	43.3036	44.428
DP	FOXP1_1	cluster_3	DN	18066	24949.151	24234	25688	416.6914	0.7241	-0.4657	0.001	0.0010435	8153	191364	49.307	3115	57864	14.909	882	18066	4.6549	10.8181	9.4406	28.3146	31.2215
SP4	TCF7L2_known7	cluster_5	Common_1_DN	24837	34303.872	33858	34822	309.6768	0.724	-0.4659	0.001	0.0010435	12562	176542	61.995	4202	56378	19.798	1797	24837	8.7219	14.305	14.0686	42.7653	44.0544
DN	SOX15_2	cluster_1	SP8	4615	6375.586	6205	6568	107.7785	0.7239	-0.4661	0.001	0.0010909	3740	52487	73.258	679	9108	12.712	335	4615	6.4413	8.9572	8.7927	49.3373	50.6697
DN	SOX17_4	cluster_1	SP8	3813	5268.036	5097	5437	101.7697	0.7239	-0.4662	0.001	0.0010909	3286	46015	72.668	569	7564	11.945	278	3813	6.0216	8.4601	8.2864	48.8576	50.4098
SP4	MAFF_1	cluster_5	Common_1_DN	22654	31313.894	30783	31794	307.5811	0.7235	-0.467	0.001	0.001	9927	139260	55.679	4204	56810	22.714	1627	22654	9.0575	16.3896	16.2674	38.7012	39.8768
DP	HES5_1	cluster_5	Common_1_DN	17122	23667.912	23290	24027	226.5929	0.7234	-0.4671	0.001	0.0010435	9609	106096	60.849	3606	39030	22.385	1557	17122	9.82	16.2036	16.1382	43.178	43.8688
SP4	FOXP3_1	cluster_5	Common_1_DN	19724	27267.864	26792	27782	297.7742	0.7234	-0.4672	0.001	0.0010909	7981	128134	57.854	3180	48216	21.77	1256	19724	8.9057	15.7374	15.3933	39.4969	40.9076
SP4	SOX8_9	cluster_2	DP	3111	4302.201	4110	4505	117.4365	0.7232	-0.4676	0.001	0.0010909	2995	47951	62.253	485	7176	9.3163	199	3111	4.0389	6.6444	6.4879	41.0309	43.3528
DN	CBX5_1	cluster_1	SP8	4591	6349.342	6184	6517	99.1537	0.7231	-0.4677	0.001	0.0010435	5190	57165	75.199	838	8765	11.53	428	4591	6.0394	8.2466	8.0311	51.074	52.3788
DN	SPIC_1	cluster_1	SP8	16018	22153.362	21804	22495	217.0344	0.7231	-0.4678	0.001	0.001	19310	251988	71.563	2474	31470	8.9373	1236	16018	4.549	6.4008	6.3567	49.9596	50.8993
SP4	STAT_known2	cluster_5	Common_1_DN	26451	36589.189	36057	37095	319.9594	0.7229	-0.4681	0.001	0.001	9013	180903	68.592	2866	54457	20.648	1349	26451	10.029	14.9673	14.6216	47.0691	48.5723
DN	TCF7_1	cluster_1	SP8	18560	25682.081	25241	26059	238.4823	0.7227	-0.4685	0.001	0.0010435	13527	217263	71.046	2397	37254	12.182	1170	18560	6.0692	8.6494	8.5426	48.811	49.8202
DP	SOX17_5	cluster_3	DN	2942	4071.199	3872	4266	120.7462	0.7227	-0.4685	0.001	0.0011429	1842	25857	46.902	680	8911	16.164	213	2942	5.3365	11.5635	11.378	31.3235	33.0154
DP	HIF1A_2	cluster_5	Common_1_DN	25084	34720.289	34312	35125	248.8089	0.7225	-0.469	0.001	0.001	13829	185302	69.991	3862	50133	18.936	1882	25084	9.4745	13.6091	13.5368	48.7312	50.0349
SP4	E2F7_1	cluster_5	Common_1_DN	8535	11822.881	11565	12084	153.2558	0.7219	-0.4701	0.001	0.0010435	4567	59426	65.789	1499	18468	20.445	678	8535	9.4489	14.8456	14.3624	45.2302	46.2151
SP8	MTF1_1	cluster_5	Common_1_DN	78388	108604.265	107889	109336	457.0905	0.7218	-0.4704	0.001	0.001	38926	519131	65.795	12833	166540	21.107	5931	78388	9.9349	15.2366	15.0998	46.2168	47.0686
SP8	FOXP3_2	cluster_5	Common_1_DN	3583	4964.986	4841	5090	75.1906	0.7217	-0.4705	0.001	0.0010909	4518	27108	59.623	1426	8406	18.489	604	3583	7.8806	13.3687	13.2175	42.3562	42.6243
DP	TBX15_2	cluster_3	DN	12942	17937.129	17666	18200	171.3997	0.7215	-0.4709	0.001	0.0010909	14654	102598	53.396	4913	33885	17.635	1860	12942	6.7355	12.6928	12.6143	37.8587	38.1939
SP4	MGA_2	cluster_5	Common_1_DN	13593	18844.13	18436	19232	248.9337	0.7214	-0.4712	0.001	0.0010909	5162	87892	57.771	2036	33173	21.805	812	13593	8.9347	15.7303	15.4656	39.8821	40.9761
SP4	TCF7L1_1	cluster_5	Common_1_DN	24237	33600.518	33049	34169	335.636	0.7213	-0.4713	0.001	0.0010435	10758	172842	61.696	3640	55600	19.846	1530	24237	8.6514	14.222	14.0226	42.033	43.5917
DP	SPI1_known1	cluster_1	SP8	14148	19617.396	19338	19923	184.3814	0.7212	-0.4715	0.001	0.001	34618	243610	52.166	5460	37816	8.0978	2018	14148	3.0296	5.8293	5.8076	36.9597	37.4127
SP8	KLF12_1	cluster_5	Common_1_DN	18953	26284.188	26018	26579	164.6519	0.7211	-0.4717	0.001	0.001	19874	119244	60.476	7375	43670	22.148	3171	18953	9.6122	15.9555	15.8943	42.9966	43.4005
DP	ATF3_known4	cluster_5	Common_1_DN	15605	21642.385	21299	22034	224.0378	0.7211	-0.4718	0.001	0.001	8408	118694	67.508	2360	32327	18.386	1117	15605	8.8755	13.285	13.1473	47.3305	48.2723
SP4	SOX15_2	cluster_2	DP	3001	4162.171	3999	4332	103.8446	0.7211	-0.4718	0.001	0.0010909	3199	44866	62.621	510	6849	9.5594	218	3001	4.1886	6.8146	6.6888	42.7451	43.8166
DP	SOX30_1	cluster_3	DN	5130	7115.472	6826	7404	170.0273	0.721	-0.4719	0.001	0.0010435	3080	46247	52.079	987	14037	15.807	350	5130	5.7769	11.3636	11.0926	35.461	36.5463
DP	SOX5_3	cluster_3	DN	3530	4896.466	4697	5098	130.5735	0.721	-0.472	0.001	0.0011429	2038	30641	52.026	681	9692	16.456	239	3530	5.9936	11.7272	11.5205	35.0954	36.4218
SP8	KLF16_1	cluster_5	Common_1_DN	120000	166488.007	165643	167310	555.2716	0.7208	-0.4724	0.001	0.001	79277	887559	67.446	23523	251382	19.103	10916	120000	9.1189	13.7694	13.5202	46.4056	47.7361
DP	MYC_known20	cluster_5	Common_1_DN	22684	31474.975	30910	32024	316.7971	0.7207	-0.4725	0.001	0.001	8735	143000	56.723	3536	55966	22.2	1409	22684	8.998	16.1305	15.8629	39.8473	40.5318
SP8	SOX17_4	cluster_2	DP	2542	3527.561	3359	3714	110.1193	0.7207	-0.4725	0.001	0.0010909	2660	37246	58.82	464	6163	9.7328	184	2542	4.0144	6.9173	6.8249	39.6552	41.2461
SP8	SP8_1	cluster_5	Common_1_DN	113130	157014.085	156032	157976	585.1612	0.7205	-0.4729	0.001	0.001	67362	823083	65.87	20709	242789	19.43	9412	113130	9.0536	13.9723	13.7447	45.4488	46.596
DP	LMO2_1	cluster_3	DN	79156	109872.73	109146	110738	502.4135	0.7204	-0.4731	0.001	0.001	40696	452099	54.045	18571	204084	24.397	7121	79156	9.4625	17.498	17.5086	38.3447	38.786
DP	SOX7_4	cluster_3	DN	4328	6009.729	5708	6315	175.993	0.7202	-0.4735	0.001	0.0010909	2231	35768	47.798	863	12935	17.286	273	4328	5.7837	12.2367	12.1002	31.6338	33.4596
DP	HES7_1	cluster_5	Common_1_DN	17356	24102.847	23812	24447	196.5593	0.7201	-0.4737	0.001	0.0010435	9992	110416	62.326	3600	38880	21.946	1576	17356	9.7968	15.7726	15.7187	43.7778	44.6399
DP	SPIB_2	cluster_1	SP8	13508	18757.981	18331	19168	248.6581	0.7201	-0.4737	0.001	0.0010435	16381	214053	53.087	2806	35711	8.8566	1038	13508	3.3501	6.3366	6.3106	36.9922	37.8259
SP4	GATA_known16	cluster_5	Common_1_DN	15673	21765.106	21239	22241	284.2779	0.7201	-0.4737	0.001	0.0010435	5122	107965	62.558	1823	35888	20.795	760	15673	9.0814	14.838	14.5167	41.6895	43.672
DN	SOX3_1	cluster_1	SP8	3586	4980.884	4810	5155	104.8223	0.72	-0.4739	0.001	0.0010909	2538	40630	73.556	481	7166	12.973	234	3586	6.492	9.2199	8.826	48.6486	50.0419
DP	TCF4_2	cluster_3	DN	48868	67876.506	67273	68551	389.875	0.72	-0.474	0.001	0.001	32705	295539	54.979	13902	124083	23.083	5413	48868	9.0909	16.551	16.5352	38.9368	39.3833
SP8	PBX1_2	cluster_2	DP	2346	3259.23	3083	3429	104.1085	0.7199	-0.4742	0.001	0.0010435	2880	41061	58.486	430	5750	8.1901	171	2346	3.3415	5.9375	5.7135	39.7674	40.8
SP4	MTF1_3	cluster_5	Common_1_DN	25910	36022.328	35513	36447	292.672	0.7193	-0.4754	0.001	0.001	12953	170029	62.803	4568	58056	21.444	1988	25910	9.5704	15.3478	15.2386	43.5201	44.6293
DP	XBP1_4	cluster_5	Common_1_DN	9322	12963.319	12678	13291	193.8867	0.7191	-0.4757	0.001	0.0010435	4834	62945	57.546	1802	22724	20.775	720	9322	8.5224	14.8945	14.8098	39.9556	41.0227
SP8	SOX8_2	cluster_2	DP	5185	7211.939	6948	7480	161.502	0.719	-0.476	0.001	0.0010909	4985	74903	58.581	886	12634	9.8809	353	5185	4.0551	7.0812	6.9223	39.842	41.0401
DP	ETV4_2	cluster_1	SP8	1772	2465.739	2313	2622	97.7415	0.7188	-0.4764	0.001	0.0011429	5095	46084	26.755	1051	9352	5.4294	197	1772	1.0288	3.8665	3.8452	18.7441	18.9478
SP4	SMAD4_1	cluster_5	Common_1_DN	57174	79543.042	78822	80357	472.6863	0.7188	-0.4764	0.001	0.001	25162	357474	58.021	10079	138396	22.463	4052	57174	9.2798	16.1036	15.9939	40.2024	41.3119
SP4	SPIB_2	cluster_5	Common_1_DN	38846	54048.301	53412	54626	353.647	0.7187	-0.4765	0.001	0.0010435	18724	244697	60.687	7171	90414	22.423	3008	38846	9.6341	16.0649	15.8751	41.9467	42.9646
DN	TCF7_2	cluster_1	SP8	13773	19167.71	18867	19461	179.8084	0.7186	-0.4768	0.001	0.0010435	15144	167157	70.642	2583	27660	11.689	1265	13773	5.8206	8.3531	8.2396	48.9741	49.7939
SP4	SP1_known3	cluster_5	Common_1_DN	75169	104700.812	103892	105470	471.6688	0.7179	-0.4781	0.001	0.001	39133	513297	64.396	13072	164835	20.679	5799	75169	9.4303	14.8187	14.6443	44.362	45.6026
DP	PKNOX1_1	cluster_3	DN	21317	29703.924	29202	30250	309.8039	0.7177	-0.4786	0.001	0.0010435	10906	164350	52.965	3874	56704	18.274	1419	21317	6.8698	13.0112	12.9705	36.6288	37.5935
DP	SPIC_2	cluster_1	SP8	12393	17270.287	16825	17655	246.8698	0.7176	-0.4787	0.001	0.0010435	14989	195470	52.699	2615	33138	8.934	956	12393	3.3412	6.378	6.3401	36.5583	37.3982
DN	FOXO6_1	cluster_1	SP8	3400	4740.654	4605	4885	88.5529	0.7173	-0.4794	0.001	0.0010909	3202	41765	72.845	544	6763	11.796	264	3400	5.9302	8.2448	8.1408	48.5294	50.2735
SP8	SP4_2	cluster_5	Common_1_DN	133320	185871.662	184799	186958	640.5427	0.7173	-0.4794	0.001	0.001	59003	991063	69.566	17108	272423	19.122	8102	133320	9.3581	13.7315	13.4522	47.358	48.9386
DP	ETV1_1	cluster_1	SP8	1805	2517.185	2348	2680	100.6919	0.7172	-0.4796	0.001	0.0010909	5186	46934	26.831	1065	9493	5.4269	200	1805	1.0319	3.8565	3.8458	18.7793	19.014
DP	SPI1_known4	cluster_1	SP8	11061	15423.898	15046	15812	227.4693	0.7172	-0.4796	0.001	0.0010435	13368	174316	53.393	2317	29252	8.9598	853	11061	3.388	6.3809	6.3454	36.8148	37.8128
SP8	EGR1_known12	cluster_5	Common_1_DN	110023	153432.355	152419	154482	611.2183	0.7171	-0.4798	0.001	0.001	52057	864606	69.047	14575	227849	18.196	6788	110023	8.7864	13.0396	12.7252	46.5729	48.2877
SP4	SOX18_2	cluster_2	DP	2783	3881.769	3690	4064	107.4	0.717	-0.4799	0.001	0.0010909	2930	41032	62.484	483	6451	9.8237	200	2783	4.238	6.8259	6.7825	41.4079	43.1406
DP	TBX2_2	cluster_3	DN	9901	13813.089	13545	14092	173.97	0.7168	-0.4803	0.001	0.0010909	8541	85538	53.696	2659	26010	16.328	994	9901	6.2153	11.638	11.575	37.3825	38.0661
SP4	PKNOX1_1	cluster_5	Common_1_DN	27768	38742.102	38171	39332	360.7903	0.7167	-0.4805	0.001	0.0010435	11588	174615	56.273	4839	69909	22.529	1874	27768	8.9487	16.1719	15.9024	38.727	39.7202
SP8	FOXP1_1	cluster_2	DP	14838	20702.563	20134	21212	334.8741	0.7167	-0.4805	0.001	0.0010435	10498	244896	63.1	1880	37085	9.5554	689	14838	3.8232	6.5632	6.0589	36.6489	40.0108
SP8	SOX17_5	cluster_2	DP	2008	2801.921	2639	2980	99.6087	0.7168	-0.4805	0.001	0.0011429	2277	31990	58.026	381	5012	9.0912	146	2008	3.6423	6.4119	6.277	38.3202	40.0638
DP	HIF1A_1	cluster_5	Common_1_DN	17744	24761.012	24417	25030	194.545	0.7166	-0.4807	0.001	0.0010435	11588	130466	68.094	3336	36648	19.128	1575	17744	9.2611	13.5916	13.6005	47.2122	48.4174
SP4	PBX1_4	cluster_5	Common_1_DN	4723	6591.007	6405	6789	116.266	0.7166	-0.4807	0.001	0.0010909	2946	33026	61.318	1046	11037	20.492	428	4723	8.769	14.5282	14.3009	40.9178	42.7924
DP	PBX1_2	cluster_3	DN	3733	5211.593	4986	5439	135.8089	0.7163	-0.4813	0.001	0.0010435	2395	33982	48.403	821	11039	15.724	270	3733	5.3171	11.2735	10.9852	32.8867	33.8165
DP	SOX_1	cluster_3	DN	7828	10930.378	10640	11196	172.6056	0.7162	-0.4816	0.001	0.0010435	5830	70005	50.837	1911	21909	15.91	658	7828	5.6847	11.2864	11.1821	34.4322	35.7296
SP8	SP4_1	cluster_5	Common_1_DN	190798	266439.032	265080	267801	792.74	0.7161	-0.4818	0.001	0.001	82983	1490454	71.892	22652	380416	18.349	10999	190798	9.2031	13.2545	12.8013	48.5564	50.1551
DP	ETV2_1	cluster_1	SP8	8692	12139.701	11860	12431	171.7217	0.716	-0.4819	0.001	0.0010435	15356	154289	52.346	2363	23363	7.9265	867	8692	2.949	5.646	5.6336	36.6906	37.2041
DP	SMAD3_1	cluster_3	DN	18454	25773.29	25412	26106	208.2248	0.716	-0.4819	0.001	0.0010435	19225	159039	54.071	5906	48192	16.385	2242	18454	6.2741	11.6619	11.6034	37.9614	38.2927
SP8	FOXO4_3	cluster_5	Common_1_DN	3673	5130.429	4935	5317	115.8804	0.716	-0.482	0.001	0.0010909	2096	27285	58.087	731	9058	19.283	288	3673	7.8194	13.7405	13.4616	39.3981	40.5498
SP4	SOX2_5	cluster_2	DP	3631	5073.423	4858	5317	138.4093	0.7157	-0.4825	0.001	0.0010909	3385	54207	61.44	561	8492	9.6251	230	3631	4.1155	6.7947	6.6984	40.9982	42.7579
SP4	MYBL1_3	cluster_5	Common_1_DN	2476	3460.495	3330	3585	76.068	0.7156	-0.4828	0.001	0.0010909	1511	16654	62.822	534	5618	21.192	226	2476	9.3399	14.957	14.8673	42.3221	44.0726
SP8	KLF7_1	cluster_5	Common_1_DN	158374	221349.044	220116	222627	727.1947	0.7155	-0.483	0.001	0.001	75221	1217400	68.768	21519	328882	18.578	10032	158374	8.9461	13.3367	13.0092	46.6193	48.1553
DP	MAFF_1	cluster_3	DN	15854	22160.373	21673	22601	295.5179	0.7154	-0.4831	0.001	0.001	9294	130389	52.132	3131	42825	17.122	1134	15854	6.3387	12.2014	12.159	36.2185	37.0204
SP8	HES1_1	cluster_5	Common_1_DN	55822	78050.88	77259	78818	453.8682	0.7152	-0.4836	0.001	0.001	25160	356406	63.862	9001	123465	22.123	3971	55822	10.002	15.783	15.6625	44.1173	45.2128
DN	HESX1_2	cluster_1	SP8	1247	1744.215	1655	1827	52.1953	0.7151	-0.4838	0.001	0.0014118	1143	16019	80.489	177	2307	11.592	91	1247	6.2657	7.9615	7.7845	51.4124	54.0529
SP4	SOX2_6	cluster_2	DP	2229	3118.333	2929	3295	105.9377	0.7149	-0.4842	0.001	0.0010435	2099	33627	62.352	347	5211	9.6623	144	2229	4.1331	6.8604	6.6286	41.4986	42.7749
SP4	STAT_known3	cluster_5	Common_1_DN	20410	28553.286	28042	29078	301.7229	0.7148	-0.4844	0.001	0.0010435	7269	145730	67.556	2289	43138	19.998	1039	20410	9.4615	14.2936	14.0054	45.391	47.3133
DP	TBX15_1	cluster_3	DN	12166	17023.769	16578	17510	284.1622	0.7147	-0.4847	0.001	0.0010909	5512	99328	52.536	1916	33027	17.468	684	12166	6.4347	12.4093	12.2483	35.6994	36.8365
SP4	TCF7L2_known5	cluster_5	Common_1_DN	27419	38383.851	37848	38969	349.8059	0.7143	-0.4853	0.001	0.0010435	11860	190547	60.832	4181	64167	20.485	1724	27419	8.7535	14.5363	14.3896	41.2342	42.7307
SP8	KLF4_1	cluster_5	Common_1_DN	104758	146672.116	146025	147455	445.2295	0.7142	-0.4855	0.001	0.001	80637	776764	66.873	23920	222161	19.126	11051	104758	9.0189	13.7046	13.4865	46.1998	47.1541
DN	NFATC1_3	cluster_1	SP8	3595	5034.756	4875	5172	92.0214	0.7141	-0.4858	0.001	0.0010909	4137	53815	79.338	541	6612	9.7479	285	3595	5.3	6.8891	6.6803	52.6802	54.3708
SP8	RUNX3_1	cluster_5	Common_1_DN	47127	65998.621	65279	66796	453.8173	0.7141	-0.4859	0.001	0.0010435	19753	302988	62.704	7152	106249	21.989	3095	47127	9.7531	15.6685	15.5541	43.2746	44.3552
SP8	SOX21_3	cluster_2	DP	3546	4967.386	4765	5174	123.8897	0.7139	-0.4862	0.001	0.0010909	3510	52680	59.82	612	8556	9.7157	242	3546	4.0266	6.8946	6.7312	39.5425	41.4446
SP4	PATZ1_1	cluster_5	Common_1_DN	128946	180631.819	179514	181780	648.3285	0.7139	-0.4863	0.001	0.001	71621	944599	64.129	23069	288137	19.562	10015	128946	8.7542	13.9833	13.6509	43.4132	44.7516
SP8	KLF14_1	cluster_5	Common_1_DN	90140	126278.082	125480	127093	491.8186	0.7138	-0.4864	0.001	0.001	51058	690823	69.873	14140	183594	18.57	6763	90140	9.1172	13.2457	13.0482	47.8289	49.0975
DN	SOX8_2	cluster_1	SP8	7134	9995.885	9779	10229	139.5251	0.7137	-0.4866	0.001	0.0010909	6301	94659	74.032	975	13966	10.923	486	7134	5.5794	7.7131	7.5365	49.8462	51.0812
SP4	ARID5A_1	cluster_2	DP	550	771.564	696	848	48.1625	0.7132	-0.4876	0.001	0.0012632	810	11102	65.491	100	1257	7.4151	42	550	3.2445	5.1852	4.9541	42	43.755
SP4	CUX1_8	cluster_5	Common_1_DN	1106	1551.183	1442	1651	63.4814	0.7132	-0.4876	0.001	0.0011429	545	8762	65.969	164	2468	18.582	72	1106	8.3271	13.211	12.6227	43.9024	44.8136
DN	TCF7L2_known7	cluster_1	SP8	16307	22878.416	22476	23225	227.3268	0.7128	-0.4885	0.001	0.0010435	14358	201641	70.809	2430	33136	11.636	1175	16307	5.7265	8.1836	8.0871	48.3539	49.2123
SP4	SOX3_1	cluster_2	DP	2449	3436.255	3253	3629	102.9093	0.7128	-0.4885	0.001	0.0010909	2214	35441	64.162	376	5627	10.187	158	2449	4.4336	7.1364	6.9101	42.0213	43.5223
DN	SOX21_2	cluster_1	SP8	5216	7319.576	7122	7508	115.564	0.7126	-0.4887	0.001	0.0010909	4666	65357	73.59	767	10297	11.594	378	5216	5.8731	8.1012	7.9808	49.2829	50.6555
DP	RUNX_2	cluster_4	SP4	12391	17391.831	16961	17862	270.9379	0.7125	-0.4891	0.001	0.0010435	17458	245690	47.274	2748	37394	7.1951	886	12391	2.3842	5.075	5.0433	32.2416	33.1363
SP4	KLF12_1	cluster_5	Common_1_DN	18189	25533.952	25245	25790	172.6935	0.7124	-0.4893	0.001	0.001	19302	115812	58.735	7375	43670	22.148	3041	18189	9.2248	15.7548	15.7056	41.2339	41.651
DN	POU2F2_known14	cluster_1	SP8	1371	1925.292	1835	2016	56.5151	0.7122	-0.4895	0.001	0.0012632	1238	18993	84.41	183	2502	11.12	96	1371	6.0931	7.7544	7.2184	52.459	54.7962
DP	POU2F2_known12	cluster_3	DN	4266	5991.185	5768	6180	122.9958	0.7121	-0.4899	0.001	0.0010435	3757	37632	51.558	1240	11923	16.335	433	4266	5.8446	11.5252	11.3361	34.9194	35.7796
DN	FOXO3_1	cluster_1	SP8	6829	9594.71	9392	9788	119.6784	0.7118	-0.4905	0.001	0.0010435	6232	81801	74.85	1083	13447	12.304	536	6829	6.2487	8.6008	8.3483	49.4922	50.7846
DN	SOX7_3	cluster_1	SP8	5720	8037.291	7819	8256	134.3867	0.7117	-0.4906	0.001	0.0010909	4157	66552	73.226	758	11442	12.589	368	5720	6.2936	8.8525	8.5948	48.5488	49.9913
SP4	ATF4_3	cluster_5	Common_1_DN	7506	10546.511	10284	10811	156.9053	0.7117	-0.4906	0.001	0.0010909	3989	51908	59.647	1433	17948	20.624	584	7506	8.625	14.6403	14.4602	40.7537	41.8208
DN	FOXO4_3	cluster_1	SP8	2922	4106.618	3968	4252	83.9272	0.7116	-0.4909	0.001	0.0010909	2636	34308	73.038	459	5801	12.35	226	2922	6.2206	8.5736	8.517	49.2375	50.3706
SP4	SOX7_3	cluster_2	DP	3752	5273.824	5037	5509	139.0165	0.7115	-0.4911	0.001	0.0010909	3572	57193	62.928	583	8749	9.6263	238	3752	4.1282	6.6629	6.5602	40.8233	42.8849
DP	RUNX2_5	cluster_5	Common_1_DN	47285	66477.111	65784	67227	451.4232	0.7113	-0.4915	0.001	0.001	17640	310092	61.324	6528	109696	21.693	2729	47285	9.3511	15.4705	15.2487	41.8045	43.1055
DP	TBX20_2	cluster_3	DN	10818	15211.807	14771	15628	271.5661	0.7112	-0.4917	0.001	0.0010435	5116	92297	53.087	1683	29129	16.754	605	10818	6.2222	11.8256	11.7209	35.9477	37.1382
SP8	KLF12_2	cluster_5	Common_1_DN	80356	113009.869	112197	113898	519.0516	0.7111	-0.492	0.001	0.001	40686	583267	67.525	12334	169624	19.637	5677	80356	9.3029	13.9532	13.7769	46.0272	47.373
SP8	SOX9_6	cluster_2	DP	4324	6083.195	5854	6338	145.7541	0.7109	-0.4924	0.001	0.0010909	4230	63611	57.612	759	10845	9.8222	292	4324	3.9162	6.9031	6.7976	38.4717	39.8709
DN	ETV5_1	cluster_1	SP8	1205	1695.846	1557	1834	81.7846	0.7107	-0.4926	0.001	0.0010435	4257	38576	26.904	716	6355	4.4321	134	1205	0.84039	3.1478	3.1237	18.7151	18.9614
DP	TBX20_5	cluster_3	DN	20808	29277.462	28756	29756	307.0674	0.7107	-0.4926	0.001	0.0010909	11935	182788	54.157	3760	55218	16.36	1381	20808	6.1651	11.571	11.3837	36.7287	37.6834
DP	SOX18_4	cluster_3	DN	2952	4154.035	3960	4348	120.1823	0.7107	-0.4927	0.001	0.0012	1791	25128	47.709	673	8902	16.902	212	2952	5.6048	11.837	11.7479	31.5007	33.1611
DP	PBX1_4	cluster_3	DN	2663	3749.913	3563	3917	105.436	0.7102	-0.4936	0.001	0.0010909	2319	25948	48.177	745	7979	14.814	245	2663	4.9443	10.5649	10.2628	32.8859	33.3751
SP8	MYCN_1	cluster_5	Common_1_DN	16567	23332.267	22952	23703	222.6085	0.7101	-0.494	0.001	0.001	10432	115067	63.978	3425	36786	20.453	1513	16567	9.2114	14.5035	14.3977	44.1752	45.0362
DN	TCF7L2_known5	cluster_1	SP8	17782	25047.153	24635	25468	250.616	0.71	-0.4942	0.001	0.0010435	13945	223921	71.487	2312	36014	11.497	1119	17782	5.6769	8.0244	7.9412	48.3997	49.3752
SP4	XBP1_2	cluster_5	Common_1_DN	9524	13416.795	13076	13768	207.6542	0.7099	-0.4944	0.001	0.0010435	2736	63041	65.364	971	20992	21.766	420	9524	9.875	15.3509	15.1076	43.2544	45.3697
SP8	RUNX2_4	cluster_5	Common_1_DN	39904	56228.108	55630	56858	374.8964	0.7097	-0.4947	0.001	0.0010435	17076	261045	63.344	6052	89641	21.752	2627	39904	9.6829	15.3842	15.2863	43.4071	44.5153
DN	SOX10_9	cluster_1	SP8	4618	6509.06	6322	6693	118.347	0.7095	-0.4951	0.001	0.0010435	4222	59134	70.343	721	9597	11.416	337	4618	5.4934	7.982	7.8094	46.7406	48.1192
SP8	SOX13_1	cluster_2	DP	1805	2544.75	2408	2716	95.2536	0.7094	-0.4953	0.001	0.0010909	2089	31443	63.156	309	4228	8.4923	127	1805	3.6255	6.0795	5.7405	41.1003	42.6916
SP4	ETV7_1	cluster_5	Common_1_DN	40093	56530.712	56007	57018	312.5599	0.7092	-0.4957	0.001	0.001	27857	251236	56.588	11316	100370	22.607	4458	40093	9.0304	16.0032	15.9583	39.3955	39.9452
DP	TBX4_2	cluster_3	DN	14735	20787.464	20301	21259	293.0138	0.7089	-0.4964	0.001	0.0010909	6487	123601	55.329	2070	38122	17.065	775	14735	6.5961	11.947	11.9214	37.4396	38.6522
SP4	SPDEF_6	cluster_5	Common_1_DN	16572	23379.416	23040	23715	218.7972	0.7088	-0.4965	0.001	0.001	8360	117397	67.827	2581	34933	20.183	1195	16572	9.5746	14.2943	14.1162	46.2999	47.4394
DP	FOXO6_1	cluster_3	DN	3201	4518.086	4307	4731	123.9678	0.7086	-0.4971	0.001	0.0010909	2247	29326	51.149	738	9094	15.861	251	3201	5.5831	11.1704	10.9152	34.0108	35.199

SP8	SOX15_2	cluster_2	DP	2838	4006.277	3819	4186	114.2989	0.7085	-0.4972	0.001	0.0010909	3083	43248	60.363	510	6849	9.5594	207	2838	3.9611	6.7142	6.5622	40.5882	41.4367
SP4	SOX9_6	cluster_2	DP	4614	6515.257	6268	6728	146.5561	0.7082	-0.4977	0.001	0.0010909	4528	68048	61.63	759	10845	9.8222	309	4614	4.1789	6.8242	6.7805	40.7115	42.545
SP8	ETV2_1	cluster_5	Common_1_DN	23937	33801.165	33359	34195	258.6202	0.7082	-0.4978	0.001	0.0010435	16594	166680	56.55	6080	60068	20.38	2391	23937	8.1212	14.4088	14.3611	39.3257	39.8498
DP	RUNX_1	cluster_3	DN	53814	76025.268	75122	76910	514.9489	0.7078	-0.4985	0.001	0.0010435	21137	297645	46.5	11766	164110	25.638	3811	53814	8.4072	18.03	18.0799	32.3899	32.7914
DN	ARID5B_1	cluster_1	SP8	5660	7997.146	7830	8171	101.6351	0.7078	-0.4986	0.001	0.0010909	5946	77508	76.405	872	10810	10.656	446	5660	5.5794	7.5008	7.3025	51.1468	52.3589
SP4	BATF_known1	cluster_5	Common_1_DN	7535	10647.983	10392	10923	152.36	0.7077	-0.4988	0.001	0.0010435	3974	51803	59.832	1417	17961	20.745	581	7535	8.7028	14.62	14.5455	41.0021	41.952
DN	SOX18_2	cluster_1	SP8	3869	5470.155	5286	5625	100.8776	0.7073	-0.4995	0.001	0.0010909	3405	47699	72.637	591	7867	11.98	282	3869	5.8918	8.2819	8.1113	47.7157	49.1801
SP8	SPZ1_1	cluster_5	Common_1_DN	100164	141639.692	140736	142644	551.7432	0.7072	-0.4999	0.001	0.001	49464	724618	65.702	15701	219487	19.901	6961	100164	9.082	14.0729	13.823	44.3348	45.6355
DN	FOXP1_1	cluster_1	SP8	17761	25125.886	24548	25725	352.7499	0.7069	-0.5004	0.001	0.0010435	10678	252594	65.084	2385	45631	11.757	872	17761	4.5763	8.1663	7.0314	36.5618	38.9231
DN	NFATC1_1	cluster_1	SP8	7853	11112.154	10861	11352	147.1852	0.7067	-0.5008	0.001	0.0010909	6650	128410	79.943	818	14633	9.1099	418	7853	4.889	6.2857	6.1156	51.1002	53.6664
SP8	HIF1A::ARNT_1	cluster_5	Common_1_DN	10678	15113.708	14902	15327	127.5418	0.7065	-0.5012	0.001	0.001	10355	74439	66.239	3229	22887	20.366	1474	10678	9.5017	14.2347	14.3446	45.6488	46.6553
SP4	FOXP3_2	cluster_5	Common_1_DN	3665	5188.186	5066	5305	74.6727	0.7065	-0.5013	0.001	0.0010909	4729	28374	62.407	1426	8406	18.489	617	3665	8.061	13.0472	12.9168	43.2679	43.5998
DP	HBP1_1	cluster_3	DN	5829	8252.838	7936	8527	178.0041	0.7063	-0.5016	0.001	0.0010435	3549	54705	50.814	1145	16681	15.495	390	5829	5.4144	10.989	10.6553	34.0611	34.9439
DN	SPIB_1	cluster_1	SP8	8118	11495.638	11337	11656	97.363	0.7062	-0.5018	0.001	0.0010435	22113	132678	71.137	2742	16291	8.7346	1358	8118	4.3526	6.1412	6.1186	49.5259	49.8312
DP	MAF_known11	cluster_3	DN	18832	26670.971	26117	27263	332.6291	0.7061	-0.5021	0.001	0.0010435	10294	166060	54.212	3193	49735	16.236	1176	18832	6.1479	11.4241	11.3405	36.8306	37.8647
DP	MESP1_1	cluster_3	DN	47465	67234.651	66714	67788	341.5739	0.706	-0.5023	0.001	0.001	32605	294479	56.054	13534	120595	22.955	5265	47465	9.0349	16.1478	16.1183	38.902	39.359
DP	SOX11_1	cluster_3	DN	16139	22870.422	22446	23369	294.9538	0.7057	-0.5029	0.001	0.0010435	9836	159338	54.566	2854	43312	14.832	1019	16139	5.5268	10.3599	10.1288	35.7043	37.2622
DP	SOX9_4	cluster_3	DN	7554	10705.105	10378	11017	203.9852	0.7057	-0.5029	0.001	0.0010909	4428	66520	51.448	1492	21287	16.464	508	7554	5.8424	11.4724	11.356	34.0483	35.4864
DP	SOX10_4	cluster_3	DN	8178	11591.034	11257	11896	202.2303	0.7056	-0.5031	0.001	0.0010909	4770	71762	51.378	1603	23013	16.476	551	8178	5.8551	11.5514	11.396	34.3731	35.5364
SP8	SOX3_3	cluster_2	DP	2781	3941.807	3746	4140	121.4221	0.7056	-0.5031	0.001	0.0010909	2699	43266	55.718	486	7333	9.4434	178	2781	3.5814	6.595	6.4277	36.6255	37.9245
DN	IKZF2_3	cluster_1	SP8	4703	6666.257	6526	6801	85.3467	0.7055	-0.5032	0.001	0.0010909	7087	71094	80.484	904	8622	9.7608	481	4703	5.3242	6.7871	6.6152	53.208	54.5465
DP	ELK4_2	cluster_4	SP4	5202	7373.586	7080	7669	176.7692	0.7055	-0.5032	0.001	0.0011429	7680	69469	25.728	3236	28879	10.695	578	5202	1.9266	7.526	7.4882	17.8616	18.0131
SP4	MTF1_1	cluster_5	Common_1_DN	75919	107711.863	107026	108435	449.3221	0.7048	-0.5046	0.001	0.001	38636	515202	65.297	12833	166540	21.107	5744	75919	9.622	14.867	14.7358	44.7596	45.586
SP8	ETS_known17	cluster_5	Common_1_DN	31291	44427.931	43823	45052	379.9026	0.7043	-0.5057	0.001	0.001	12384	211645	59.804	4576	75147	21.234	1844	31291	8.8418	14.8902	14.7847	40.2972	41.6397
SP4	ATF4_2	cluster_5	Common_1_DN	7009	9953.767	9708	10180	143.4914	0.7042	-0.506	0.001	0.0010909	3978	47794	58.841	1471	17153	21.118	590	7009	8.6291	14.8316	14.665	40.1088	40.8617
SP4	MYC_known13	cluster_5	Common_1_DN	15482	21993.706	21606	22415	235.5515	0.7039	-0.5065	0.001	0.0010909	9038	99518	55.876	3715	39745	22.315	1419	15482	8.6926	15.7004	15.557	38.1965	38.9533
DP	ARID5B_1	cluster_3	DN	6394	9086.427	8837	9361	159.8305	0.7037	-0.5069	0.001	0.0010909	4087	53311	52.552	1409	17650	17.399	497	6394	6.303	12.1605	11.9938	35.2732	36.2266
SP4	RUNX3_1	cluster_5	Common_1_DN	44415	63116.777	62424	63791	397.137	0.7037	-0.507	0.001	0.0010435	18902	289786	59.972	7152	106249	21.989	2909	44415	9.1918	15.3899	15.3268	40.6739	41.8027
DP	NFATC1_3	cluster_3	DN	4789	6806.486	6579	7049	139.0546	0.7036	-0.5071	0.001	0.0010909	2771	36045	53.14	1065	13171	19.418	377	4789	7.0603	13.6052	13.2862	35.3991	36.3602
SP8	ELK4_1	cluster_5	Common_1_DN	9755	13867.916	13691	14043	107.9542	0.7034	-0.5075	0.001	0.001	10505	84617	76.469	2290	18216	16.462	1215	9755	8.8156	11.5659	11.5284	53.0568	53.5518
SP4	RUNX2_4	cluster_5	Common_1_DN	37995	54022.293	53437	54650	374.3218	0.7033	-0.5077	0.001	0.0010435	16422	250904	60.883	6052	89641	21.752	2497	37995	9.2196	15.2052	15.1432	41.2591	42.3857
DN	SP100_1	cluster_1	SP8	699	994.36	940	1043	32.268	0.7033	-0.5079	0.001	0.0013333	1107	14404	85.672	97	1202	7.1492	56	699	4.1575	5.0587	4.8528	57.732	58.1531
DP	ARID5A_1	cluster_3	DN	1036	1473.713	1366	1589	68.8451	0.7032	-0.508	0.001	0.0012632	647	8899	52.495	232	2962	17.473	77	1036	6.1114	11.9011	11.6418	33.1897	34.9764
DP	TBX1_2	cluster_3	DN	9371	13332.376	12890	13759	243.9737	0.7029	-0.5086	0.001	0.0010909	4390	79089	50.708	1562	26827	17.2	527	9371	6.0083	12.0046	11.8487	33.7388	34.9312
SP8	SOX3_1	cluster_2	DP	2302	3276.842	3098	3477	114.6071	0.7026	-0.5092	0.001	0.0010909	2117	33889	61.352	376	5627	10.187	147	2302	4.1675	6.9438	6.7928	39.0957	40.9099
DN	RUNX_1	cluster_1	SP8	35107	49969.494	49547	50486	296.2506	0.7026	-0.5093	0.001	0.0010435	34752	490327	76.602	4759	65825	10.284	2489	35107	5.4846	7.1622	7.1599	52.3009	53.3338
SP8	EGR4_2	cluster_5	Common_1_DN	100015	142357.241	141379	143249	576.6517	0.7026	-0.5093	0.001	0.001	50126	822981	71.563	13078	203425	17.689	6233	100015	8.6969	12.4347	12.1528	47.6602	49.1655
DP	POU2F2_known11	cluster_3	DN	4256	6059.098	5819	6244	126.677	0.7025	-0.5095	0.001	0.0010435	3816	38242	51.233	1256	12148	16.275	430	4256	5.7018	11.2683	11.1291	34.2357	35.0346
DN	TCF7L1_2	cluster_1	SP8	12648	18010.261	17738	18277	168.3685	0.7023	-0.5099	0.001	0.0010435	14657	161969	71.069	2407	25849	11.342	1159	12648	5.5497	7.9075	7.8089	48.1512	48.9303
SP8	XBP1_4	cluster_5	Common_1_DN	9744	13879.778	13531	14180	189.9247	0.702	-0.5104	0.001	0.0010435	5176	67397	61.616	1802	22724	20.775	751	9744	8.9082	14.5093	14.4576	41.6759	42.8798
SP4	POU2F2_known16	cluster_2	DP	2343	3338.18	3184	3511	98.513	0.702	-0.5105	0.001	0.0010909	2634	34687	58.999	479	5889	10.016	182	2343	3.9852	6.9096	6.7547	37.9958	39.786
DP	TBX21_6	cluster_3	DN	15833	22556.603	22088	23057	296.775	0.7019	-0.5106	0.001	0.0010909	7546	136200	53.52	2481	42917	16.864	889	15833	6.2216	11.7811	11.6248	35.8323	36.8921
DP	SOX3_3	cluster_3	DN	4451	6344.815	6071	6627	166.3443	0.7016	-0.5114	0.001	0.0010909	2264	36279	46.72	920	13934	17.944	280	4451	5.732	12.3675	12.2688	30.4348	31.9434
DN	SOX4_1	cluster_1	SP8	16439	23434.528	23080	23803	230.4571	0.7015	-0.5115	0.001	0.0010435	14546	235575	74.812	2132	32642	10.366	1042	16439	5.2206	7.1635	6.9782	48.8743	50.3615
DP	FOXO1_4	cluster_3	DN	2759	3934.547	3732	4095	104.4537	0.7013	-0.5119	0.001	0.0010909	1950	25417	51.042	647	7957	15.979	216	2759	5.5406	11.0769	10.8549	33.3849	34.6739
DN	HMGA1_2	cluster_1	SP8	7253	10343.312	10135	10556	130.4297	0.7013	-0.512	0.001	0.0010435	6789	95209	77.457	1076	14034	11.417	540	7253	5.9007	7.954	7.618	50.1859	51.6816
DP	FOXO3_4	cluster_4	SP4	2350	3352.827	3170	3520	112.1117	0.701	-0.5125	0.001	0.0010909	1629	21240	50.833	546	6810	16.298	182	2350	5.6242	11.1725	11.064	33.3333	34.5081
DP	HMGA1_1	cluster_3	DN	1932	2756.437	2649	2866	65.3702	0.701	-0.5125	0.001	0.0010435	3004	18138	53.492	893	5260	15.513	324	1932	5.6978	10.7856	10.6517	36.2822	36.73
SP8	SOX18_2	cluster_2	DP	2581	3682.243	3502	3841	104.6207	0.701	-0.5125	0.001	0.0010909	2756	38602	58.784	483	6451	9.8237	186	2581	3.9304	6.7489	6.6862	38.5093	40.0093
SP4	PBX1_2	cluster_5	Common_1_DN	5873	8380.953	8125	8629	147.9663	0.7008	-0.5129	0.001	0.0010435	3037	43176	61.498	1057	14017	19.965	422	5873	8.3653	13.8953	13.6025	39.9243	41.8991
DN	MAF_known1	cluster_1	SP8	29810	42552.285	42056	43095	312.1326	0.7006	-0.5134	0.001	0.001	33530	506311	73.776	4027	58739	8.559	1990	29810	4.3437	5.935	5.8877	49.4164	50.7499
DP	HESX1_1	cluster_3	DN	1372	1958.965	1855	2064	63.4917	0.7005	-0.5135	0.001	0.0012632	1124	10127	51.054	454	3905	19.686	154	1372	6.9167	13.7011	13.5479	33.9207	35.1344
DN	RBPJ_1	cluster_1	SP8	5957	8504.608	8361	8637	82.1764	0.7005	-0.5136	0.001	0.0010435	16019	112173	78.007	1618	11061	7.692	859	5957	4.1426	5.3624	5.3105	53.0902	53.8559
SP8	RUNX1_4	cluster_2	DP	1616	2307.837	2182	2422	77.7013	0.7004	-0.5138	0.001	0.0010909	2766	28019	55.372	442	4280	8.4583	161	1616	3.1936	5.8207	5.7675	36.4253	37.757
SP4	KLF16_1	cluster_5	Common_1_DN	112637	160881.922	159954	161877	547.1403	0.7001	-0.5143	0.001	0.001	76586	858580	65.244	23523	251382	19.103	10223	112637	8.5594	13.3484	13.119	43.4596	44.8071
DP	RUNX3_3	cluster_5	Common_1_DN	60367	86276.202	85486	87090	495.5673	0.6997	-0.5152	0.001	0.001	23341	416133	63.949	8022	136756	21.016	3449	60367	9.2768	14.7766	14.5067	42.9943	44.1421
DP	AHR::ARNT::HIF1A_1	cluster_5	Common_1_DN	13331	19055.198	18821	19270	138.5235	0.6996	-0.5154	0.001	0.0010435	12707	104472	71.635	3290	26751	18.343	1618	13331	9.1408	12.7331	12.7604	49.1793	49.8337
SP8	HES5_1	cluster_5	Common_1_DN	16066	22968.067	22621	23324	208.1031	0.6995	-0.5156	0.001	0.0010435	9348	103156	59.163	3606	39030	22.385	1463	16066	9.2143	15.6504	15.5745	40.5713	41.1632
SP4	EGR1_known12	cluster_5	Common_1_DN	103641	148170.512	147148	149109	610.5958	0.6995	-0.5157	0.001	0.001	50263	834726	66.661	14575	227849	18.196	6367	103641	8.2768	12.6674	12.4162	43.6844	45.4867
SP8	ATF6_1	cluster_5	Common_1_DN	3830	5480.236	5363	5615	82.2688	0.6989	-0.5168	0.001	0.0010435	3930	27520	63.816	1255	8634	20.021	548	3830	8.8814	13.944	13.9172	43.6653	44.3595
DP	SOX12_1	cluster_3	DN	3448	4933.963	4701	5135	128.9447	0.6989	-0.5169	0.001	0.0010435	2452	31970	50.662	813	10059	15.94	272	3448	5.4639	11.093	10.7851	33.4563	34.2778
SP8	SOX2_5	cluster_2	DP	3325	4758.199	4541	4985	131.5839	0.6989	-0.5169	0.001	0.0010909	3178	50901	57.693	561	8492	9.6251	210	3325	3.7686	6.6079	6.5323	37.4332	39.1545
SP8	ATF2_2	cluster_5	Common_1_DN	7661	10967.831	10774	11176	125.8507	0.6985	-0.5176	0.001	0.0010435	5120	56438	65.757	1556	16788	19.56	701	7661	8.926	13.6914	13.5742	45.0514	45.6338
DN	SOX10_4	cluster_1	SP8	7598	10880.002	10622	11143	157.6328	0.6984	-0.5179	0.001	0.0010909	6860	103161	73.858	1057	15221	10.898	517	7598	5.4398	7.5364	7.3652	48.912	49.9179
DP	SPIC_1	cluster_1	SP8	11184	16017.786	15684	16395	210.8728	0.6982	-0.5182	0.001	0.001	13902	181322	51.494	2474	31470	8.9373	863	11184	3.1762	6.2077	6.168	34.8828	35.5386
DP	RUNX_2	cluster_3	DN	42445	60813.682	60058	61566	440.3438	0.698	-0.5188	0.001	0.0010435	17458	245690	47.274	9259	129043	24.83	3008	42445	8.167	17.2299	17.2758	32.4873	32.8921
SP8	ETV6_1	cluster_5	Common_1_DN	53588	76805.117	76146	77503	405.417	0.6977	-0.5193	0.001	0.001	26153	376727	62.445	8947	124366	20.615	3769	53588	8.8826	14.4113	14.2246	42.1259	43.0889
SP4	KLF13_1	cluster_5	Common_1_DN	49491	70966.298	70180	71659	456.3119	0.6974	-0.52	0.001	0.001	20223	347988	63.027	6957	114292	20.701	2913	49491	8.9638	14.4044	14.222	41.8715	43.3022
DN	SOX2_5	cluster_1	SP8	4856	6964.161	6746	7192	132.521	0.6973	-0.5201	0.001	0.0010909	4046	64802	73.448	655	9906	11.228	311	4856	5.5039	7.6866	7.4936	47.4809	49.0208
DN	SOX8_9	cluster_1	SP8	4212	6042.022	5832	6254	128.2561	0.6972	-0.5204	0.001	0.0010909	3303	52872	68.642	615	9226	11.978	273	4212	5.4683	8.2652	7.9664	44.3902	45.6536
DN	MYBL2_4	cluster_1	SP8	1358	1948.377	1856	2045	56.2296	0.6971	-0.5205	0.001	0.0010435	1996	27994	79.043	186	2524	7.1267	97	1358	3.8344	4.8597	4.851	52.1505	53.8035
DN	SOX11_1	cluster_1	SP8	15451	22168.637	21835	22523	215.4429	0.697	-0.5208	0.001	0.0010435	13495	218540	74.839	2020	30899	10.581	978	15451	5.2912	7.2471	7.0701	48.4158	50.0049
SP8	EGR1_known9	cluster_5	Common_1_DN	47733	68488.548	67956	69007	315.5448	0.697	-0.5209	0.001	0.001	29509	400130	74.089	7106	93622	17.335	3561	47733	8.8384	12.0675	11.9294	50.1126	50.9848
SP4	ETV6_1	cluster_5	Common_1_DN	55933	80290.585	79493	81042	458.3753	0.6966	-0.5215	0.001	0.001	27340	393690	65.257	8947	124366	20.615	3926	55933	9.2713	14.3599	14.2074	43.8806	44.9745
SP4	SP8_1	cluster_5	Common_1_DN	105217	151088.372	150141	152059	560.1229	0.6964	-0.522	0.001	0.001	64724	792047	63.386	20709	242789	19.43	8713	105217	8.4204	13.4618	13.2842	42.0735	43.3368
SP4	HES1_1	cluster_5	Common_1_DN	53335	76598.52	75881	77304	413.6013	0.6963	-0.5222	0.001	0.001	24677	349612	62.645	9001	123465	22.123	3796	53335	9.5568	15.3827	15.2555	42.1731	43.1985
SP4	SOX8_2	cluster_2	DP	5340	7670.765	7408	7912	154.144	0.6962	-0.5224	0.001	0.0010909	5294	79524	62.195	886	12634	9.8809	361	5340	4.1763	6.819	6.715	40.7449	42.2669
SP4	SP4_1	cluster_5	Common_1_DN	179461	257773.004	256413	259053	835.6872	0.6962	-0.5224	0.001	0.001	80215	1441564	69.533	22652	380416	18.349	10308	179461	8.6562	12.8505	12.449	45.5059	47.1749
SP8	SOX14_2	cluster_2	DP	2493	3582.003	3420	3752	102.906	0.6961	-0.5227	0.001	0.0010909	3330	36787	57.469	603	6367	9.9466	229	2493	3.8946	6.8769	6.7769	37.9768	39.155
DN	ETV6_1	cluster_1	SP8	18979	27273.578	26936	27636	227.7306	0.6959	-0.5231	0.001	0.001	32656	469723	77.86	2565	35529	5.8892	1338	18979	3.1459	4.0973	4.0405	52.1637	53.4183
DP	SOX4_2	cluster_3	DN	6526	9379.086	9103	9664	180.3326	0.6958	-0.5232	0.001	0.0010909	3926	59025	51.393	1328	18761	16.335	441	6526	5.6821	11.2328	11.0563	33.2078	34.7849
DN	SOX4_2	cluster_1	SP8	6379	9170.113	8960	9385	134.9813	0.6957	-0.5235	0.001	0.0010909	5733	86204	75.057	888	12727	11.081	436	6379	5.5542	7.6051	7.3999	49.0991	50.1218
SP4	ELK4_1	cluster_5	Common_1_DN	9638	13864.545	13698	14038	104.9244	0.6952	-0.5245	0.001	0.001	10509	84669	76.516	2290	18216	16.462	1201	9638	8.7099	11.4283	11.3832	52.4454	52.9095
SP4	CUX1_9	cluster_2	DP	456	656.395	583	723	44.5417	0.6952	-0.5246	0.001	0.0012632	597	10309	69.627	63	1004	6.781	28	456	3.0798	4.6901	4.4233	44.4444	45.4183
DN	SOX12_1	cluster_1	SP8	3772	5427.006	5252	5586	98.4349	0.6951	-0.5247	0.001	0.0010435	3600	46915	74.344	625	7670	12.154	296	3772	5.9773	8.2222	8.0401	47.36	49.1786
DN	MGA_2	cluster_1	SP8	6863	9874.449	9655	10085	135.6571	0.6951	-0.5248	0.001	0.0010909	6706	114146	75.028	839	13515	8.8834	407	6863	4.511	6.0692	6.0125	48.5101	50.7806
DP	SOX4_1	cluster_3	DN	17092	24591.172	24082	25121	320.2226	0.6951	-0.5248	0.001	0.0010435	10641	172327	54.726	3071	46460	14.754	1083	17092	5.4279	10.1776	9.9184	35.2654	36.7886
DP	MAF_known12	cluster_3	DN	14247	20502.714	20025	20972	275.6251	0.6949	-0.5251	0.001	0.0010435	7384	125901	57.08	2214	36416	16.51	841	14247	6.4592	11.3895	11.316	37.9855	39.1229
DP	SOX7_3	cluster_3	DN	4892	7040.558	6776	7299	162.3772	0.6949	-0.5252	0.001	0.0010909	2800	44824	49.319	976	14704	16.179	312	4892	5.3826	11.1429	10.9138	31.9672	33.2699
SP8	SP1_known2	cluster_5	Common_1_DN	201627	290200.212	289022	291429	732.5998	0.6948	-0.5254	0.001	0.001	116483	1617881	71.388	31582	415613	18.339	15033	201627	8.8967	12.9057	12.4624	47.5999	48.5132
SP8	SOX1_2	cluster_2	DP	3186	4586.674	4399	4821	129.1222	0.6947	-0.5256	0.001	0.0010909	3221	45113	56.297	621	8368	10.443	231	3186	3.9758	7.1717	7.0623	37.1981	38.0736
SP8	ETV5_1	cluster_5	Common_1_DN	4390	6323.658	6091	6562	146.8382	0.6943	-0.5264	0.001	0.0010435	4199	38058	26.542	2699	24074	16.79	489	4390	3.0617	11.6456	11.535	18.1178	18.2354
SP8	MYCN_2	cluster_5	Common_1_DN	22849	32916.555	32612	33233	207.6058	0.6942	-0.5267	0.001	0.001	17431	157501	65.162	5735	50848	21.037	2543	22849	9.4532	14.589	14.5072	44.3418	44.9359
DP	FOXO4_2	cluster_3	DN	6938	9998.856	9716	10267	169.7857	0.6939	-0.5272	0.001	0.0010435	5614	73565	57.489	1450	17939	14.019	544	6938	5.4219	9.6901	9.4311	37.5172	38.6755
SP8	HES7_1	cluster_5	Common_1_DN	16179	23316.369	22976	23633	200.6103	0.6939	-0.5272	0.001	0.0010435	9675	106849	60.312	3600	38880	21.946	1470	16179	9.1324	15.1938	15.1419	40.8333	41.6127
DP	POU2F2_known10	cluster_3	DN	4137	5967.172	5758	6175	126.3874	0.6933	-0.5284	0.001	0.0010435	4216	38032	52.25	1333	11620	15.964	462	4137	5.6836	10.9583	10.8777	34.6587	35.6024
DN	ATF2_1	cluster_1	SP8	483	697.126	654	741	25.0446	0.6933	-0.5285	0.001	0.0010435	1484	10388	76.433	131	917	6.7471	69	483	3.5538	4.6496	4.6496	52.6718	52.6718
DN	RUNX_2	cluster_1	SP8	28303	40831.445	40382	41215	256.7607	0.6932	-0.5287	0.001	0.0010435	28180	397366	76.459	3931	54123	10.414	2007	28303	5.4459	7.1221	7.1227	51.0557	52.2938
SP4	ETS_known17	cluster_5	Common_1_DN	32153	46387.891	45794	47075	374.9465	0.6931	-0.5288	0.001	0.001	12925	220877	62.412	4576	75147	21.234	1892	32153	9.0853	14.6383	14.557	41.3462	42.7868
DP	TBX19_1	cluster_3	DN	7092	10235.836	9881	10635	229.6777	0.6929	-0.5293	0.001	0.0010435	3308	63063	49.328	1156	21010	16.434	376	7092	5.5473	11.3664	11.2459	32.526	33.7554
SP8	ATF3_known4	cluster_5	Common_1_DN	15334	22135.765	21797	22507	204.625	0.6927	-0.5296	0.001	0.001	8603	121435	69.067	2360	32327	18.386	1095	15334	8.7213	12.7281	12.6273	46.3983	47.434
SP8	SOX8_9	cluster_2	DP	2766	3993.344	3800	4205	123.347	0.6927	-0.5296	0.001	0.0010909	2766	44281	57.488	485	7176	9.3163	176	2766	3.591	6.363	6.2465	36.2887	38.5452
SP8	GMEB2_3	cluster_5	Common_1_DN	3383	4884.552	4723	5047	100.6151	0.6927	-0.5298	0.001	0.0010435	2094	29627	71.145	509	6963	16.721	242	3383	8.1238	11.5568	11.4186	47.5442	48.5854
SP4	KLF4_1	cluster_5	Common_1_DN	98320	142036.227	141328	142875	467.2933	0.6922	-0.5307	0.001	0.001	78036	752571	64.791	23920	222161	19.126	10366	98320	8.4646	13.2836	13.0645	43.3361	44.2562
SP4	SOX13_1	cluster_2	DP	1814	2622.889	2480	2768	83.9664	0.6917	-0.5317	0.001	0.0010909	2170	32640	65.561	309	4228	8.4923	128	1814	3.6436	5.8986	5.5576	41.4239	42.9044
DN	IRF4_1	cluster_1	SP8	3859	5579.95	5441	5721	83.1954	0.6916	-0.5319	0.001	0.0010435	3899	54808	80.813	513	7035	10.373	276	3859	5.69	7.0787	7.0409	53.8012	54.8543
SP8	EGR4_1	cluster_5	Common_1_DN	43141	62375.806	61899	62904	314.7211	0.6916	-0.5319	0.001	0.001	30552	346098	68.54	8378	92071	18.233	3865	43141	8.5434	12.6506	12.465	46.1327	46.8562
DN	MYBL2_2	cluster_1	SP8	2433	3518.72	3396	3641	74.7674	0.6915	-0.5321	0.001	0.0010435	2843	43039	79.915	315	4541	8.4317	165	2433	4.5176	5.8037	5.653	52.381	53.5785
SP8	SOX21_2	cluster_2	DP	3494	5055.004	4865	5238	118.5515	0.6913	-0.5327	0.001	0.0010909	3688	51655	58.162	668	8902	10.023	253	3494	3.9342	6.8601	6.7641	37.8743	39.2496
SP4	KLF7_1	cluster_5	Common_1_DN	147171	212976.736	211740	214133	756.3059	0.691	-0.5332	0.001	0.001	72331	1170811	66.136	21519	328882	18.578	9310	147171	8.3133	12.8714	12.57	43.2641	44.7489
SP4	HIF1A::ARNT_1	cluster_5	Common_1_DN	10550	15269.359	15052	15454	125.5813	0.6909	-0.5334	0.001	0.001	10457	75241	66.952	3229	22887	20.366	1447	10550	9.3878	13.8376	14.0216	44.8126	46.096
SP8	HIF1A_2	cluster_5	Common_1_DN	23821	34480.045	34065	34862	245.0404	0.6909	-0.5335	0.001	0.001	13715	183722	69.394	3862	50133	18.936	1788	23821	8.9975	13.0368	12.9658	46.2973	47.5156
DP	ARID5A_1	cluster_4	SP4	771	1116.706	1007	1224	66.0506	0.6907	-0.5339	0.001	0.0012632	647	8899	52.495	181	2239	13.208	58	771	4.5481	8.9645	8.6639	32.0442	34.435
DN	TCF21_1	cluster_1	SP8	8318	12044.513	11813	12277	147.5065	0.6906	-0.534	0.001	0.001	16797	218881	77.73	1237	15681	5.5687	641	8318	2.9539	3.8162	3.8002	51.8189	53.0451
DN	STAT_known2	cluster_1	SP8	8282	11993.113	11763	12233	142.2448	0.6906	-0.5341	0.001	0.001	11028	221310	83.913	782	14719	5.5809	427	8282	3.1402	3.872	3.7423	54.6036	56.2674
SP4	SPZ1_1	cluster_5	Common_1_DN	94992	137583.347	136559	138677	631.7661	0.6904	-0.5344	0.001	0.001	48085	704127	63.844	15701	219487	19.901	6598	94992	8.6131	13.7215	13.4907	42.0228	43.2791
DN	IRF4_2	cluster_1	SP8	10133	14679.076	14429	14899	149.6382	0.6903	-0.5347	0.001	0.001	7884	113080	77.321	1355	19304	13.2	707	10133	6.9287	8.9675	8.9609	52.1771	52.4917
DP	SMAD_1	cluster_3	DN	13550	19631.194	19343	19937	186.0057	0.6902	-0.5348	0.001	0.0010435	15462	123736	56.699	4442	34936	16.009	1701	13550	6.209	11.0012	10.9507	38.2936	38.7852
SP8	ATF2_1	cluster_5	Common_1_DN	1078	1562.236	1499	1618	38.5413	0.6902	-0.5348	0.001	0.0010435	1370	9590	70.561	323	2222	16.349	155	1078	7.9317	11.3139	11.2409	47.9876	48.5149
DP	ARID3A_2	cluster_4	SP4	1212	1756.58	1604	1899	86.886	0.6902	-0.535	0.001	0.0013333	987	15963	50.765	247	3631	11.547	79	1212	3.8543	8.0041	7.5926	31.9838	33.3792
DN	FOXO4_2	cluster_1	SP8	7175	10397.717	10162	10613	136.4637	0.6901	-0.5352	0.001	0.0010435	7348	96298	75.255	1152	14358	11.22	564	7175	5.6071	7.6756	7.4508	48.9583	49.9721
SP8	ERG_2	cluster_5	Common_1_DN	12260	17768.99	17434	18114	209.1563	0.69	-0.5354	0.001	0.001	6800	88560	61.21	2323	29323	20.267	948	12260	8.4737	13.9412	13.8437	40.8093	41.8102
SP8	ERG_4	cluster_5	Common_1_DN	12413	17995.317	17659	18308	199.6386	0.6898	-0.5357	0.001	0.001	6801	88600	61.768	2327	29418	20.509	960	12413	8.6537	14.1156	14.0102	41.2548	42.1953
DP	GATA_known17	cluster_3	DN	6307	9145.034	8805	9431	200.8316	0.6897	-0.536	0.001	0.0010909	3215	51515	48.405	1265	19323	18.157	403	6307	5.9263	12.535	12.243	31.8577	32.6399
SP8	ETV1_1	cluster_5	Common_1_DN	5411	7848.423	7551	8129	169.6167	0.6895	-0.5364	0.001	0.0010909	4842	43840	25.062	3527	31478	17.995	602	5411	3.0933	12.4329	12.3426	17.0683	17.1898

DN	POU2F2_known15	cluster_1	SP8		3168	4596.534	4466	4731	82.3971	0.6893	-0.5368	0.001	0.0010435	3911	43212	75.23	593	6304	10.975	292	3168	5.5153	7.4661	7.3313	49.2411	50.2538
DP	RUNX1_4	cluster_3	DN		2911	4225.47	4073	4402	99.2566	0.689	-0.5374	0.001	0.0010909	2351	23810	47.054	931	9119	18.021	294	2911	5.7529	12.5053	12.226	31.5789	31.9224
DP	TBX1_4	cluster_3	DN		16846	24449.476	23859	24995	337.7828	0.689	-0.5374	0.001	0.0010909	7984	152301	52.016	2605	47743	16.306	892	16846	5.7535	11.1723	11.061	34.2418	35.2848
DN	SP100_1	cluster_4	SP4		793	1151.742	1097	1210	34.0051	0.6888	-0.5379	0.001	0.0013333	1107	14404	85.672	115	1406	8.3626	61	793	4.7166	5.5104	5.5054	53.0435	56.4011
DN	TBX1_2	cluster_1	SP8		7883	11447.898	11178	11710	156.4333	0.6886	-0.5382	0.001	0.0010909	6383	115007	73.738	933	16006	10.262	443	7883	5.0542	6.9403	6.8544	47.4812	49.2503
DP	SPZ1_2	cluster_3	DN		15400	22364.104	21998	22696	205.8312	0.6886	-0.5382	0.001	0.0010435	13790	138253	56.134	4088	40215	16.328	1543	15400	6.2527	11.1893	11.139	37.7446	38.2942
SP8	MYC_known20	cluster_5	Common_1_DN		21787	31639.756	31162	32156	308.8329	0.6886	-0.5383	0.001	0.001	8781	143953	57.101	3536	55966	22.2	1354	21787	8.6422	15.4197	15.1348	38.2919	38.929
SP8	ELK4_2	cluster_5	Common_1_DN		8651	12565.055	12237	12883	201.1461	0.6885	-0.5384	0.001	0.0011429	7080	64048	23.72	5965	53260	19.725	962	8651	3.2039	13.5876	13.5071	16.1274	16.243
DN	IRF_known21	cluster_1	SP8		12235	17771.805	17481	18040	171.2345	0.6885	-0.5385	0.001	0.001	9344	135063	76.665	1643	23596	13.394	841	12235	6.9449	9.0004	9.0587	51.1869	51.852
DP	HESX1_2	cluster_1	SP8		777	1129.607	1032	1235	62.6291	0.6881	-0.5393	0.001	0.0014118	733	10262	51.563	177	2307	11.592	57	777	3.9041	7.7763	7.5716	32.2034	33.6801
DN	FOXP3_2	cluster_1	SP8		2606	3787.924	3705	3872	52.9433	0.6881	-0.5394	0.001	0.0010909	5499	32994	72.569	895	5284	11.622	436	2606	5.7318	7.9287	7.8984	48.7151	49.3187
SP8	FLI1_4	cluster_5	Common_1_DN		12559	18256.377	17918	18573	196.9841	0.6879	-0.5396	0.001	0.001	7101	92567	63.122	2313	29272	19.961	971	12559	8.5641	13.6741	13.5675	41.9801	42.9045
SP8	ELK3_1	cluster_5	Common_1_DN		6994	10167.793	9828	10489	198.5031	0.6879	-0.5398	0.001	0.0011429	5938	53644	24.833	4619	41177	19.061	779	6994	3.2376	13.1189	13.0378	16.8651	16.9852
DP	BPTF_1	cluster_3	DN		16806	24434.748	23990	24906	276.2385	0.6878	-0.5399	0.001	0.0010909	10963	146228	52.127	3764	48126	17.156	1286	16806	5.991	11.7304	11.493	34.1658	34.9208
DN	TBX15_1	cluster_1	SP8		9116	13254.823	12923	13525	184.2622	0.6878	-0.54	0.001	0.0010909	7884	142057	75.135	1054	18196	9.6241	513	9116	4.8215	6.5068	6.4171	48.6717	50.0989
SP8	EGR3_3	cluster_5	Common_1_DN		55449	80629.079	79886	81314	403.6852	0.6877	-0.5401	0.001	0.001	32104	473266	72.424	7938	112982	17.29	3818	55449	8.4853	11.8926	11.7162	48.0978	49.0777
DP	ETV7_1	cluster_3	DN		23608	34342.03	33847	34851	289.8734	0.6874	-0.5407	0.001	0.001	25386	228951	51.568	7508	66847	15.056	2622	23608	5.3174	10.3285	10.3114	34.9227	35.3165
SP8	EGR3_2	cluster_5	Common_1_DN		82446	120039.916	119251	120871	485.1864	0.6868	-0.542	0.001	0.001	46634	711154	72.287	11617	169240	17.203	5522	82446	8.3805	11.8411	11.5933	47.5338	48.7154
DN	TBX20_2	cluster_1	SP8		8071	11753.597	11459	12051	172.2743	0.6867	-0.5422	0.001	0.0010435	7160	129186	74.304	942	16294	9.3719	455	8071	4.6422	6.3547	6.2476	48.3015	49.5336
DN	SPDEF_4	cluster_1	SP8		8529	12421.238	12186	12672	153.9788	0.6867	-0.5423	0.001	0.0010435	8815	132735	73.627	1228	17521	9.7187	576	8529	4.7309	6.5343	6.4256	46.9055	48.6787
SP4	KLF12_2	cluster_5	Common_1_DN		74928	109135.098	108462	110014	478.9009	0.6866	-0.5425	0.001	0.001	39236	562601	65.133	12334	169624	19.637	5298	74928	8.6745	13.5029	13.3181	42.9544	44.173
SP4	SP4_2	cluster_5	Common_1_DN		123317	179624.88	178461	180689	695.4322	0.6865	-0.5426	0.001	0.001	57022	957821	67.232	17108	272423	19.122	7483	123317	8.656	13.123	12.8747	43.7398	45.2667
DP	FOXO3_1	cluster_3	DN		5453	7945.616	7680	8186	152.195	0.6863	-0.543	0.001	0.0010435	4685	61564	56.333	1195	14663	13.417	426	5453	4.9897	9.0928	8.8574	35.6485	37.1888
DP	FOXO3_4	cluster_3	DN		2147	3128.659	2964	3294	99.9779	0.6863	-0.543	0.001	0.0010909	1629	21240	50.833	521	6382	15.274	169	2147	5.1383	10.3745	10.1083	32.4376	33.6415
DP	MAF_known10	cluster_3	DN		10139	14776.614	14470	15078	185.9096	0.6862	-0.5434	0.001	0.0010909	7909	87167	53.303	2609	28034	17.143	926	10139	6.2001	11.7082	11.6317	35.4925	36.1668
DN	MAF_known12	cluster_1	SP8		9287	13536.508	13225	13830	175.5659	0.6861	-0.5435	0.001	0.0010435	9989	170271	77.196	1102	17974	8.1489	552	9287	4.2105	5.5261	5.4542	50.0907	51.6691
DN	IKZF1_2	cluster_1	SP8		11693	17048.865	16857	17239	119.8394	0.6859	-0.544	0.001	0.0010435	27213	217711	79.439	2799	21823	7.9629	1479	11693	4.2666	5.4349	5.3709	52.8403	53.5811
DN	SOX_1	cluster_1	SP8		6915	10086.614	9874	10358	139.6748	0.6856	-0.5446	0.001	0.0010435	8206	98530	71.552	1257	14434	10.482	583	6915	5.0216	7.1046	7.0182	46.3803	47.9077
DN	IKZF2_2	cluster_1	SP8		2414	3522.68	3424	3628	61.671	0.6854	-0.5451	0.001	0.0010435	4704	47046	78.526	483	4645	7.7532	245	2414	4.0293	5.2083	5.1311	50.7246	51.9699
DN	SOX9_4	cluster_1	SP8		6694	9770.489	9537	9999	137.8641	0.6852	-0.5455	0.001	0.0010909	6382	95858	74.138	957	13666	10.57	455	6694	5.1773	7.1294	6.9832	47.5444	48.9829
DP	SOX18_2	cluster_3	DN		3660	5345.054	5108	5590	147.084	0.6848	-0.5462	0.001	0.0010909	2288	32082	48.779	847	11219	17.084	264	3660	5.5735	11.5385	11.4261	31.1688	32.6232
SP8	ETS_known13	cluster_5	Common_1_DN		22251	32493.064	32008	33024	307.9035	0.6848	-0.5462	0.001	0.001	10079	161754	66.163	3191	49600	20.288	1395	22251	9.1014	13.8407	13.7561	43.7167	44.8609
SP4	MYCN_1	cluster_5	Common_1_DN		15816	23107.316	22787	23460	204.5137	0.6845	-0.5469	0.001	0.001	10339	114073	63.425	3425	36786	20.453	1443	15816	8.7938	13.9569	13.8648	42.1314	42.9946
SP8	SOX7_3	cluster_2	DP		3410	4982.676	4736	5211	142.6836	0.6844	-0.547	0.001	0.0010909	3353	53689	59.073	583	8749	9.6263	215	3410	3.752	6.4122	6.3514	36.8782	38.9759
SP4	ERG_2	cluster_5	Common_1_DN		12824	18737.956	18420	19064	200.016	0.6844	-0.5471	0.001	0.001	7181	93517	64.636	2323	29323	20.267	990	12824	8.8635	13.7864	13.713	42.6173	43.7336
DP	SOX3_1	cluster_3	DN		2949	4310.045	4095	4513	123.2874	0.6843	-0.5473	0.001	0.0010909	1698	27182	49.21	615	9153	16.57	189	2949	5.3388	11.1307	10.8491	30.7317	32.2189
SP4	ERG_4	cluster_5	Common_1_DN		12900	18877.031	18574	19213	198.8012	0.6834	-0.5492	0.001	0.001	7152	93169	64.953	2327	29418	20.509	996	12900	8.9932	13.9262	13.8458	42.8019	43.8507
DP	SMAD3_3	cluster_3	DN		3385	4954.058	4802	5122	100.4817	0.6833	-0.5493	0.001	0.0010435	3577	32203	55.927	1019	8929	15.507	378	3385	5.8788	10.5675	10.5114	37.0952	37.9102
DN	SOX14_3	cluster_1	SP8		4283	6270.005	6068	6475	119.4756	0.6831	-0.5497	0.001	0.0010909	4092	57347	70.134	698	9269	11.336	311	4283	5.238	7.6002	7.4686	44.5559	46.2078
DN	HESX1_1	cluster_1	SP8		924	1353.112	1293	1408	34.7723	0.6831	-0.5498	0.001	0.0012632	1814	16345	82.401	201	1706	8.6005	104	924	4.6582	5.7332	5.6531	51.7413	54.1618
DN	FOXO1_4	cluster_1	SP8		2058	3013.17	2858	3177	97.0381	0.6831	-0.5498	0.001	0.0010909	1950	25417	51.042	492	6089	12.228	160	2058	4.1329	8.2051	8.0969	32.5203	33.7987
DP	SPIB_1	cluster_1	SP8		5528	8092.907	7912	8293	121.2393	0.6831	-0.5498	0.001	0.0010435	15545	93270	50.008	2742	16291	8.7346	923	5528	2.9639	5.9376	5.9269	33.6616	33.9328
DP	NFATC1_1	cluster_3	DN		9859	14441.903	14052	14892	251.4613	0.6827	-0.5507	0.001	0.0010909	4535	87510	54.48	1524	27428	17.076	521	9859	6.1378	11.4884	11.2661	34.1864	35.945
DP	SOX2_5	cluster_3	DN		4923	7212.881	6976	7479	161.1346	0.6826	-0.5509	0.001	0.0010909	2721	43557	49.369	1000	15053	17.061	315	4923	5.5799	11.5766	11.3024	31.5	32.7044
SP4	FLI1_4	cluster_5	Common_1_DN		13051	19123.529	18806	19445	189.1518	0.6825	-0.5512	0.001	0.001	7443	97009	66.151	2313	29272	19.961	1007	13051	8.8996	13.5295	13.4534	43.5365	44.5853
DP	SOX21_3	cluster_2	DP		3524	5164.331	4945	5360	128.5206	0.6824	-0.5512	0.001	0.0010909	3663	54981	62.433	612	8556	9.7157	242	3524	4.0016	6.6066	6.4095	39.5425	41.1875
DN	CTCF_known2	cluster_3	DN		18809	27563.324	26912	28290	391.0945	0.6824	-0.5513	0.001	0.0010435	17849	287162	34.367	5148	80650	9.6522	1177	18809	2.2511	6.5942	6.55	22.8632	23.3218
SP4	ATF6_1	cluster_5	Common_1_DN		3722	5456.096	5325	5591	82.9313	0.6822	-0.5517	0.001	0.0010435	3915	27415	63.572	1255	8634	20.021	534	3722	8.6309	13.6398	13.5765	42.5498	43.1086
DP	SOX1_3	cluster_4	SP4		3575	5241.842	5010	5478	136.7639	0.6821	-0.552	0.001	0.0010909	2535	35523	47.207	855	11384	15.128	260	3575	4.7508	10.2564	10.0639	30.4094	31.4037
DP	RUNX_1	cluster_3	DN		20714	30373.059	29761	30953	371.88	0.682	-0.5522	0.001	0.0010435	21137	297645	46.5	4759	65825	10.284	1473	20714	3.2361	6.9688	6.9593	30.9519	31.4683
DN	HMGA1_1	cluster_1	SP8		1877	2753.509	2680	2819	42.6792	0.6818	-0.5526	0.001	0.0010435	4316	26040	76.796	611	3646	10.753	314	1877	5.5356	7.2753	7.2081	51.3912	51.4811
SP8	FLI1_2	cluster_5	Common_1_DN		11823	17344.074	17008	17682	201.3979	0.6817	-0.5528	0.001	0.001	6676	86936	61.84	2240	28311	20.138	912	11823	8.41	13.6609	13.5997	40.7143	41.7612
DP	TBX2_1	cluster_3	DN		15373	22552.486	22129	22958	258.5702	0.6817	-0.5529	0.001	0.0010435	8226	140221	57.494	2405	39675	16.268	905	15373	6.3033	11.0017	10.9634	37.6299	38.7473
DP	XBP1_2	cluster_2	SP8		2798	4106.472	3853	4367	149.0572	0.6814	-0.5533	0.001	0.0010435	2416	55665	57.716	332	7249	7.5161	122	2798	2.9011	5.0497	5.0265	36.747	38.5984
DP	POU2F2_known5	cluster_3	DN		4510	6619.59	6383	6870	147.379	0.6814	-0.5535	0.001	0.0010909	3181	41440	50.684	1066	13379	16.363	351	4510	5.516	11.0343	10.8832	32.9268	33.7095
SP8	ETV3_1	cluster_5	Common_1_DN		15819	23228.322	22913	23535	194.7784	0.681	-0.5542	0.001	0.0010435	13610	123083	65.171	4036	35843	18.978	1757	15819	8.3759	12.9096	12.8523	43.5332	44.1341
SP4	MYCN_2	cluster_5	Common_1_DN		22187	32587.471	32218	32958	227.7169	0.6809	-0.5546	0.001	0.001	17249	155885	64.493	5735	50848	21.037	2468	22187	9.1793	14.3081	14.2329	43.034	43.634
SP4	KLF14_1	cluster_5	Common_1_DN		82658	121415.252	120669	122146	460.0069	0.6808	-0.5547	0.001	0.001	49055	664031	67.164	14140	183594	18.57	6181	82658	8.3605	12.6001	12.4479	43.7129	45.0222
SP4	ETV2_1	cluster_5	Common_1_DN		10739	15776.828	15402	16205	222.2112	0.6807	-0.5549	0.001	0.0010435	7748	77780	26.389	6080	60068	20.38	1073	10739	3.6435	13.8487	13.8069	17.648	17.8781
SP4	FLI1_2	cluster_5	Common_1_DN		12429	18259.222	17934	18583	195.6603	0.6807	-0.5549	0.001	0.001	7024	91445	65.047	2240	28311	20.138	959	12429	8.841	13.6532	13.5918	42.8125	43.9017
SP8	ELK3_2	cluster_5	Common_1_DN		6703	9851.219	9547	10118	174.9058	0.6805	-0.5554	0.001	0.0011429	5845	51570	24.526	4526	40342	19.186	747	6703	3.1879	13.0823	12.9979	16.5046	16.6154
DP	RREB1_1	cluster_3	DN		79428	116743.547	115778	117743	607.8576	0.6804	-0.5556	0.001	0.001	45950	762192	60.431	13009	201534	15.979	4996	79428	6.2975	10.8727	10.421	38.4042	39.4117
DP	SPI1_known1	cluster_3	DN		26393	38802.096	38350	39238	261.6734	0.6802	-0.556	0.001	0.001	16728	243610	52.166	5135	74696	15.995	1756	26393	5.6517	10.8498	10.8341	35.0112	35.3339
SP4	ATF2_1	cluster_5	Common_1_DN		1064	1565.088	1497	1630	39.9616	0.68	-0.5563	0.001	0.0010435	1377	9639	70.922	323	2222	16.349	153	1064	7.8287	11.1111	11.0385	47.3684	47.8848
DN	RUNX2_4	cluster_1	SP8		17504	25749.243	25428	26073	192.4841	0.6798	-0.5568	0.001	0.0010435	22375	342168	83.028	2118	31471	7.6366	1154	17504	4.2474	5.1575	5.1156	54.4854	55.6195
DN	POU2F2_known10	cluster_1	SP8		3734	5496.578	5347	5632	81.5688	0.6794	-0.5577	0.001	0.0010435	6013	54229	74.503	854	7508	10.315	418	3734	5.13	6.9516	6.8856	48.9461	49.7336
DN	TBX20_5	cluster_1	SP8		16549	24359.34	23972	24702	216.2933	0.6794	-0.5577	0.001	0.0010909	16570	253871	75.218	2275	33402	9.8965	1096	16549	4.9032	6.6144	6.5187	48.1758	49.5449
DN	SOX5_1	cluster_1	SP8		1849	2722.464	2624	2820	60.6456	0.6793	-0.5579	0.001	0.0010909	2705	24345	72.39	438	3829	11.379	208	1849	5.495	7.6895	7.595	47.4886	48.2894
DP	MGA_2	cluster_3	DN		9274	13656.152	13308	14020	222.2637	0.6791	-0.5582	0.001	0.0010909	4878	83018	54.568	1556	25429	16.714	551	9274	6.0958	11.2956	11.1711	35.4113	36.4702
DN	STAT_known3	cluster_1	SP8		7742	11400.911	11164	11672	149.1	0.6791	-0.5583	0.001	0.0010435	8877	177940	82.488	756	14273	6.6166	400	7742	3.589	4.506	4.3509	52.9101	54.2423
DN	TBX1_4	cluster_1	SP8		13571	19987.56	19624	20356	226.8992	0.679	-0.5585	0.001	0.0010909	11107	211898	72.371	1543	28290	9.6621	719	13571	4.635	6.4734	6.4045	46.5975	47.971
DN	POU2F3_1	cluster_1	SP8		4784	7054.151	6861	7259	119.8819	0.6782	-0.5602	0.001	0.0010435	4595	69456	74.624	681	9801	10.53	321	4784	5.14	6.9859	6.8878	47.1366	48.8113
DN	BPTF_1	cluster_1	SP8		15583	22981.382	22631	23294	200.0743	0.6781	-0.5605	0.001	0.0010909	15844	211822	75.51	2436	31404	11.195	1187	15583	7.5566	7.4918	7.3566	48.7274	49.6211
SP4	ETV3_1	cluster_5	Common_1_DN		16287	24026.642	23696	24301	186.7236	0.6779	-0.5609	0.001	0.0010435	14073	127252	67.378	4036	35843	18.978	1811	16287	8.6237	12.8686	12.799	44.8712	45.4398
SP4	EGR4_2	cluster_5	Common_1_DN		93222	137547.204	136546	138549	569.9008	0.6777	-0.5612	0.001	0.001	48413	794683	69.102	13078	203425	17.689	5798	93222	8.1062	11.9761	11.7307	44.334	45.8262
DP	IKZF2_3	cluster_3	DN		5495	8113.781	7878	8341	138.9872	0.6773	-0.5622	0.001	0.0010909	4868	48814	55.261	1594	15124	17.122	564	5495	6.2208	11.5859	11.257	35.3827	36.333
SP4	ELK4_2	cluster_5	Common_1_DN		9424	13916.527	13596	14267	211.7236	0.6772	-0.5623	0.001	0.0011429	7834	70855	26.241	5965	53260	19.725	1048	9424	3.4902	13.3776	13.3004	17.5692	17.6943
SP8	HIF1A_1	cluster_5	Common_1_DN		16712	24685.384	24332	25019	199.7113	0.677	-0.5627	0.001	0.0010435	11568	130194	67.952	3336	36648	19.128	1482	16712	8.7225	12.8112	12.8362	44.4245	45.6014
DN	POU2F3_2	cluster_1	SP8		2547	3763.997	3660	3860	63.133	0.6768	-0.5633	0.001	0.0010909	4271	34232	74.798	656	5138	11.227	323	2547	5.5653	7.5626	7.4404	49.2378	49.5718
SP4	XBP1_4	cluster_5	Common_1_DN		9453	13973.882	13653	14274	182.7954	0.6765	-0.5638	0.001	0.0010435	5207	67800	61.985	1802	22724	20.775	729	9453	8.6422	14.0004	13.9425	40.455	41.5992
DN	RREB1_1	cluster_1	SP8		65073	96263.646	95572	96918	391.5855	0.6761	-0.5648	0.001	0.001	60217	1008607	79.968	7972	126523	10.031	3957	65073	5.1598	6.5712	6.4524	49.6362	51.4365
DN	PKNOX2_2	cluster_1	SP8		8976	13280.704	13028	13548	158.2435	0.6759	-0.5651	0.001	0.0010435	15871	176400	72.498	1726	18579	7.6357	818	8976	3.689	5.1541	5.0884	47.3928	48.3126
SP8	ETV6_2	cluster_5	Common_1_DN		11678	17290.254	16904	17703	245.2487	0.6754	-0.5661	0.001	0.0011429	9151	82658	22.329	8770	77969	21.062	1299	11678	3.1546	14.1952	14.1281	14.8119	14.9777
SP8	ETV4_2	cluster_5	Common_1_DN		5255	7783.194	7505	8079	164.9594	0.6752	-0.5666	0.001	0.0011429	4775	43196	25.078	3500	31215	18.122	585	5255	3.0509	12.2513	12.1655	16.7143	16.8349
DP	FOXP3_2	cluster_3	DN		2318	3434.035	3333	3554	69.2442	0.6751	-0.5668	0.001	0.0010909	4108	24648	54.212	1087	6401	14.079	389	2318	5.0983	9.4693	9.4044	35.7866	36.2131
DN	SOX1_2	cluster_1	SP8		4330	6414.769	6238	6607	112.7889	0.6751	-0.5669	0.001	0.0010909	4151	58133	72.545	686	9166	11.438	313	4330	5.4034	7.5404	7.4484	45.6268	47.2398
DP	SOX17_4	cluster_3	DN		3292	4876.907	4632	5109	134.5918	0.6751	-0.5669	0.001	0.0010909	2192	30688	48.463	786	10403	16.429	238	3292	5.1988	10.8577	10.7273	30.2799	31.6447
DN	HES5_1	cluster_1	SP8		4762	7055.969	6893	7218	101.4117	0.6749	-0.5672	0.001	0.0010435	12165	134258	77.001	867	9291	5.3287	437	4762	2.7311	3.5923	3.5469	50.4037	51.2539
DP	SOX21_2	cluster_3	DN		4680	6937.369	6676	7225	155.8843	0.6747	-0.5678	0.001	0.0010909	3145	44041	49.589	1089	14396	16.21	341	4680	5.2696	10.8426	10.6265	31.3131	32.509
SP4	SP1_known2	cluster_5	Common_1_DN		189469	280836.698	279634	282166	775.9966	0.6747	-0.5678	0.001	0.001	112736	1565680	69.085	31582	415613	18.339	14104	189469	8.3602	12.5106	12.1014	44.6583	45.5878
DN	RUNX3_1	cluster_1	SP8		21072	31245.178	30922	31611	200.7178	0.6744	-0.5683	0.001	0.0010435	25951	398870	82.383	2579	38479	7.9634	1379	21072	4.3609	5.3139	5.2935	53.4703	54.7623
DN	SOX21_3	cluster_1	SP8		4618	6847.798	6616	7040	127.6185	0.6744	-0.5683	0.001	0.0010909	4326	64953	73.757	696	9659	10.968	314	4618	5.2439	7.2584	7.1098	45.1149	47.8103
DP	POU2F2_known16	cluster_3	DN		2721	4036.267	3838	4237	123.4349	0.6742	-0.5687	0.001	0.0010909	2099	27573	46.898	719	8853	15.058	212	2721	4.6281	10.1	9.8683	29.4854	30.7353
DP	POU2F3_1	cluster_3	DN		5106	7577.392	7290	7843	175.3009	0.6739	-0.5694	0.001	0.0010435	3087	46662	50.134	1089	15505	16.659	343	5106	5.486	11.1111	10.9425	31.4968	32.9313
DP	SOX14_2	cluster_3	DN		3337	4952.948	4766	5136	116.9675	0.6738	-0.5696	0.001	0.0010909	2895	31972	49.947	952	10109	15.792	307	3337	5.2131	10.6045	10.4373	32.2479	33.0102
DP	ETV3_1	cluster_1	SP8		5193	7712.816	7544	7896	107.9159	0.6733	-0.5706	0.001	0.0010435	13947	126127	66.782	1310	11627	6.1563	577	5193	2.7496	4.1371	4.1173	44.0458	44.6633
DP	CUX1_8	cluster_2	DP		366	544.225	486	609	38.823	0.6731	-0.5711	0.001	0.0011429	545	8762	65.969	61	863	6.4975	24	366	2.7556	4.4037	4.1771	39.3443	42.4102
DP	TCF12_known1	cluster_3	DN		35192	52290.117	51853	52785	299.8708	0.673	-0.5713	0.001	0.0010909	36358	261819	56.235	13030	93370	20.055	4885	35192	7.5587	13.4358	13.4413	37.4904	37.6909
SP4	ETS_known13	cluster_5	Common_1_DN		22208	33003.037	32503	33469	295.7887	0.6729	-0.5715	0.001	0.001	10234	164235	67.178	3191	49600	20.288	1394	22208	9.0838	13.6213	13.5221	43.6854	44.7742
DN	SOX11_2	cluster_1	SP8		4619	6866.371	6680	7053	115.8239	0.6727	-0.5719	0.001	0.0010909	4488	62863	74.087	731	9670	11.397	338	4619	5.4437	7.5312	7.3477	46.238	47.7663
DP	CBX5_1	cluster_3	DN		4057	6032.515	5789	6249	135.8196	0.6726	-0.5722	0.001	0.0010435	3561	39214	51.585	1143	12008	15.796	374	4057	5.3369	10.5027	10.3458	32.7209	33.7858
SP4	ETV6_2	cluster_5	Common_1_DN		12775	18993.732	18568	19438	252.6809	0.6726	-0.5722	0.001	0.0011429	10041	90698	24.501	8770	77969	21.062	1421	12775	3.451	14.152	14.0852	16.203	16.3847
DN	POU2F2_known1	cluster_1	SP8		4879	7255.083	7024	7486	139.7156	0.6725	-0.5723	0.001	0.0010435	4025	72871	75.443	595	10054	10.409	277	4879	5.0512	6.882	6.6954	46.5546	48.5279
DN	TCF3_2	cluster_1	SP8		16189	24076.645	23715	24444	215.6787	0.6724	-0.5726	0.001	0.0010435	39118	592154	77.209	2188	31655	4.1274	1080	16189	2.1108	2.7609	2.7339	49.3601	51.142
SP8	POU2F2_known16	cluster_2	DP		2085	3101.614	2928	3254	101.9034	0.6723	-0.5727	0.001	0.0010909	2431	32048	54.51	479	5889	10.016	163	2085	3.5463	6.7051	6.5059	34.0292	35.405
SP4	HES7_1	cluster_5	Common_1_DN		15763	23445.74	23101	23746	188.5798	0.6723	-0.5728	0.001	0.0010435	9737	107540	60.702	3600	38880	21.946	1432	15763	8.8976	14.7068	14.6578	39.7778	40.5427
DN	ERG_4	cluster_1	SP8		4636	6898.917	6718	7096	109.2791	0.672	-0.5734	0.001	0.001	8373	109062	76.033	724	9199	6.4131	358	4636	3.232	4.2756	4.2508	49.4475	50.3968
DN	SOX17_5	cluster_1	SP8		3098	4613.187	4469	4778	90.5753	0.6716	-0.5743	0.001	0.0011429	2792	39191	71.088	514	6773	12.286	225	3098	5.6194	8.0587	7.9049	43.7743	45.7404
DN	RBPJ_2	cluster_1	SP8		7300	10871.409	10695	11045	106.3974	0.6715	-0.5745	0.001	0.0010909	13859	138744	77.101	1475	14370	7.9855	737	7300	4.0567	5.3178	5.2615	49.9661	50.8003
DN	TBX2_2	cluster_1	SP8		7765	11570.723	11368	11789	132.4758	0.6711	-0.5754	0.001	0.0010909	11786	118013	74.082	1622	15897	9.9792	778	7765	4.8744	6.6011	6.5798	47.9655	48.8457
SP8	RUNX2_5	cluster_5	Common_1_DN		46135	68794.72	68051	69462	444.418	0.6706	-0.5764	0.001	0.001	18306	320965	63.474	6528	109696	21.693	2672	46135	9.1236	14.5963	14.3738	40.9314	42.0571
SP8	HIF1A_2	cluster_5	Common_1_DN		23058	34397.011	33956	34815	275.3538	0.6704	-0.577	0.001	0.001	13692	183403	69.274	3862	50133	18.936	1724	23058	8.7093	12.5913	12.5723	44.6401	45.9937
DN	IRF_known14	cluster_1	SP8		22925	34206.942	33714	34663	297.0978	0.6702	-0.5773	0.001	0.001	16394	342215	77.672	2296	45729	10.379	1108	22925	5.2033	6.7586	6.699	48.2578	50.1323
DP	POU2F3_2	cluster_3	DN		2560	3820.757	3668	3970	87.3764	0.6701	-0.5775	0.001	0.0010909	2892	23182	50.653	988	7694	16.812	319	2560	5.5937	11.0304	11.0431	32.2874	33.2727
DN	ATF6_1	cluster_1	SP8		1596	2382.243	2305	2460	48.0948	0.6701	-0.5776	0.001	0.0010435	4516	31617	73.316	470	3261	7.5619	228	1596	3.701	5.0487	5.0479	48.5106	48.942
DN	SMAD_1	cluster_1	SP8		9127	13621.66	13444	13807	113.7702	0.6701	-0.5776	0.001	0.0010435	20794	166413	76.255	2290	18078	8.2838	1146	9127	4.1822	5.5112	5.4845	50.0437	50.4868
DN	BATF_known1	cluster_1	SP8		3829	5717.051	5529	5895	108.4354	0.6698	-0.5782	0.001	0.0010435	4780	62340	72.002	638	8084	9.3369	297	3829	4.4224	6.2134	6.1421	46.5517	47.3652
DN	PKNOX1_2	cluster_1	SP8		8030	11991.346	11753	12219	139.4187	0.6697	-0.5785	0.001	0.0010435	13772	152898	72.377	1569	16823	7.9634	734	8030	3.8011	5.3297	5.2519	46.7814	47.7323
DP	FOXO4_3	cluster_3	DN		2405	3592.08	3438	3788	105.1793	0.6696	-0.5786	0.001	0.0010909	1828	23799	50.665	592	7294	15.528	188	2405	5.12	10.2845	10.1055	31.7568	32.9723
DP	SPIC_2	cluster_2	SP4		17877	26704.862	26213	27219	308.4558	0.6694	-0.579	0.001	0.0010435	14989	195470	52.699	4012	51184	13.799	1374	17877	4.8197	9.1667	9.1456	34.2473	34.9269
SP4	EGR4_1	cluster_5	Common_1_DN		40678	60770.466	60256	61362	323.79	0.6694	-0.5791	0.001	0.001	29753	337110	66.76	8378	92071	18.233	3641	40678	8.0557	12.2374	12.0667	43.4591	44.1811
DN	MYBL1_3	cluster_1	SP8		1090	1629.39	1557	1702	46.1408	0.6692	-0.5796	0.001	0.0010909	1868	20588	77.661	205	2158	8.1403	101	1090	4.1117	5.4069	5.2943	49.2683	50.5097
SP4	HES5_1	cluster_5	Common_1_DN		15408	23032.676	22657	23422	230.7902	0.669	-0.58	0.001	0.0010435	9376	103478	59.348	3606	39030	22.385	1401	15408	8.8369	14.9242	14.8901	38.8519	39.4773
DN	PKNOX1_1	cluster_1	SP8		11799	17641.462	17324	17932	185.1023	0.6688	-0.5803	0.001	0.0010435	15318	230880	74.405	1682	24195	7.7972	792	11799	3.8024	5.1704	5.1104	47.0868	48.7663
DP	SOX15_2	cluster_3	DN		3686	5517.285	5288	5789	148.9197	0.6681	-0.5818	0.001	0.0010909	2513	35262	49.216	869	11549	16.119	268	3686	5.1447	10.6645	10.4532	30.84	31.9162

SP4	GMEB2_3	cluster_5	Common_1_DN	3323	4976.249	4833	5141	94.3839	0.6678	-0.5824	0.001	0.0010435	2133	30178	72.468	509	6963	16.721	238	3323	7.9797	11.158	11.0113	46.7583	47.7237
DN	SOX13_1	cluster_1	SP8	2924	4379.738	4219	4537	93.5315	0.6677	-0.5827	0.001	0.0010909	2495	37512	75.346	443	6166	12.385	202	2924	5.8731	8.0962	7.7948	45.5982	47.4213
DP	FOXO1_4	cluster_4	SP4	2575	3856.842	3676	4038	112.4989	0.6677	-0.5827	0.001	0.0010909	1950	25417	51.042	628	7807	15.678	200	2575	5.1711	10.2564	10.131	31.8471	32.9832
DP	SOX5_1	cluster_4	SP4	1783	2670.738	2550	2802	76.6013	0.6677	-0.5827	0.001	0.0010909	1887	16983	50.471	618	5390	16.018	200	1783	5.2988	10.5988	10.4987	32.3625	33.0798
DP	FOXO4_3	cluster_1	SP8	1929	2892.317	2721	3052	101.3148	0.6671	-0.5841	0.001	0.0010909	1828	23799	50.665	459	5801	12.35	149	1929	4.1066	8.151	8.1054	32.4619	33.2529
SP4	ETV1_1	cluster_5	Common_1_DN	5407	8106.61	7817	8375	172.3973	0.667	-0.5842	0.001	0.0010909	5001	45274	25.882	3527	31478	17.995	601	5407	3.091	12.0176	11.9428	17.04	17.1771
SP4	ELK3_1	cluster_5	Common_1_DN	7159	10734.663	10398	11049	185.3545	0.6669	-0.5844	0.001	0.0011429	6270	56640	26.22	4619	41177	19.061	798	7159	3.314	12.7273	12.6395	17.2765	17.3859
SP4	EGR1_known9	cluster_5	Common_1_DN	44157	66212.874	65674	66764	336.7208	0.6669	-0.5845	0.001	0.001	28464	386336	71.535	7106	93622	17.335	3291	44157	8.1763	11.562	11.4297	46.313	47.1652
DP	HMGA1_2	cluster_3	DN	6625	9936.742	9694	10213	168.0065	0.6668	-0.5848	0.001	0.0010435	4747	66572	54.16	1457	19052	15.5	485	6625	5.3898	10.217	9.9516	33.2876	34.7733
SP8	SOX7_4	cluster_2	DP	2570	3856.03	3648	4057	124.4424	0.6666	-0.5852	0.001	0.0010909	2647	42472	56.757	471	7079	9.46	162	2570	3.4344	6.1201	6.051	34.3949	36.3046
DN	TBX4_1	cluster_1	SP8	6881	10324.199	10145	10470	100.8992	0.6665	-0.5853	0.001	0.0010435	16022	112186	74.769	2026	13954	9.2999	988	6881	4.586	6.1665	6.1336	48.766	49.312
DN	FOXO3_4	cluster_1	SP8	2472	3713.523	3586	3853	81.1074	0.6658	-0.5869	0.001	0.0010909	2345	30568	73.157	419	5251	12.567	193	2472	5.9161	8.2303	8.0869	46.0621	47.0767
DP	SPIB_1	cluster_3	DN	10488	15753.554	15487	15979	150.4711	0.6658	-0.5869	0.001	0.0010435	15545	93270	50.008	5324	31674	16.982	1752	10488	5.6233	11.2705	11.2448	32.9076	33.1123
SP4	ELK3_2	cluster_5	Common_1_DN	6996	10515.064	10184	10858	198.0479	0.6654	-0.5878	0.001	0.0011429	6107	55145	26.227	4526	40342	19.186	780	6996	3.3273	12.7722	12.6866	17.2338	17.3417
DN	FLI1_4	cluster_1	SP8	4406	6624.213	6438	6793	113.5136	0.6652	-0.5882	0.001	0.001	8592	111995	76.37	690	8773	5.9824	340	4406	3.0045	3.9572	3.9341	49.2754	50.2223
DN	PKNOX2_3	cluster_1	SP8	8249	12402.093	12164	12626	139.7357	0.6652	-0.5882	0.001	0.0010435	13787	153514	71.649	1632	17576	8.2032	751	8249	3.85	5.4472	5.3735	46.0172	46.9333
DN	MYBL1_5	cluster_1	SP8	2596	3903.55	3765	4035	80.6783	0.6651	-0.5883	0.001	0.0010435	3552	57284	82.002	322	4890	7	165	2596	3.7162	4.6453	4.5318	51.2422	53.0879
SP4	ATF2_2	cluster_5	Common_1_DN	7284	10952.837	10736	11183	130.2253	0.6651	-0.5884	0.001	0.0010435	5130	56548	65.885	1556	16788	19.56	664	7284	8.4867	12.9435	12.8811	42.6735	43.3881
DP	SOX15_4	cluster_4	SP4	2627	3951.403	3736	4160	130.1863	0.6649	-0.5888	0.001	0.0011429	1805	25291	45.839	676	8954	16.229	194	2627	4.7614	10.7479	10.3871	28.6982	29.3388
DP	MAF_known1	cluster_1	SP8	20583	30958.364	30348	31565	341.6075	0.6649	-0.5889	0.001	0.001	24308	367004	53.477	4027	58739	8.559	1372	20583	2.9992	5.6442	5.6084	34.07	35.0415
DP	SPI1_known4	cluster_4	SP4	15185	22841.549	22403	23251	260.1941	0.6648	-0.589	0.001	0.0010435	13368	174316	53.393	3409	43240	13.244	1171	15185	4.6511	8.7597	8.7112	34.3502	35.1179
SP4	ETV4_2	cluster_5	Common_1_DN	5308	7987.605	7711	8256	164.3963	0.6646	-0.5895	0.001	0.0011429	4906	44382	25.767	3500	31215	18.122	591	5308	3.0816	12.0465	11.9598	16.8857	17.0046
DN	MAF_known10	cluster_1	SP8	7406	11149.417	10914	11385	143.3555	0.6643	-0.5901	0.001	0.0010909	11107	122400	74.849	1423	15177	9.2809	680	7406	4.5288	6.1223	6.0507	47.7864	48.7975
DN	SOX15_4	cluster_1	SP8	3067	4617.382	4458	4795	98.1393	0.6643	-0.5901	0.001	0.0011429	2731	38267	69.358	529	6964	12.622	226	3067	5.5589	8.2754	8.0147	42.7221	44.0408
DN	HES7_1	cluster_1	SP8	4669	7030.333	6859	7179	94.353	0.6642	-0.5904	0.001	0.0010435	12542	138583	78.225	845	9092	5.1321	429	4669	2.6355	3.4205	3.3691	50.7692	51.3528
DP	MGA_3	cluster_3	DN	8297	12499.139	12139	12868	221.0881	0.6638	-0.5911	0.001	0.0010909	4692	79902	56.395	1383	22542	15.91	489	8297	5.856	10.422	10.384	35.3579	36.8068
DP	MYC_known13	cluster_3	DN	12562	18930.65	18542	19266	221.0989	0.6636	-0.5916	0.001	0.0010909	9190	101216	56.829	3113	33619	18.876	1148	12562	7.0531	12.4918	12.4111	36.8776	37.3658
DN	TBX15_2	cluster_1	SP8	8641	13027.2	12839	13211	112.7843	0.6633	-0.5922	0.001	0.0010909	20505	143570	74.719	2561	17651	9.1862	1240	8641	4.4971	6.0473	6.0187	48.4186	48.9547
DN	ERG_2	cluster_1	SP8	4532	6833.377	6653	7001	108.9741	0.6633	-0.5923	0.001	0.001	8341	108611	75.068	729	9239	6.3857	350	4532	3.1324	4.1961	4.1727	48.011	49.0529
DN	FOXO1_5	cluster_1	SP8	21880	32993.463	32625	33373	214.4087	0.6632	-0.5925	0.001	0.0010435	32721	361528	75.797	4092	44229	9.2729	1992	21880	4.5873	6.0878	6.0521	48.6804	49.4698
DP	TCF7L1_1	cluster_1	SP8	10992	16579.156	16105	17000	275.0921	0.663	-0.5929	0.001	0.0010435	8494	136288	48.648	2221	34617	12.357	689	10992	3.9236	8.1116	8.0653	31.0221	31.7532
DN	TBX5_3	cluster_1	SP8	10222	15419.803	15213	15643	136.6566	0.6629	-0.5931	0.001	0.001	20740	186864	75.435	2358	20762	8.3814	1143	10222	4.1265	5.5111	5.4703	48.4733	49.2342
DP	POU2F2_known1	cluster_3	DN	5185	7824.491	7513	8124	192.2374	0.6627	-0.5936	0.001	0.0010435	2722	49255	50.993	935	15805	16.363	292	5185	5.368	10.7274	10.5269	31.2299	32.8061
DN	SOX14_2	cluster_1	SP8	3429	5178.7	5015	5316	92.6542	0.6622	-0.5947	0.001	0.0010909	4224	46656	72.886	690	7315	11.428	314	3429	5.3568	7.4337	7.3495	45.5072	46.8763
DN	SOX9_6	cluster_1	SP8	5694	8600.497	8396	8832	140.5255	0.6621	-0.5949	0.001	0.0010909	5272	79251	71.777	871	12456	11.281	386	5694	5.157	7.3217	7.1848	44.3169	45.7129
SP4	EGR3_2	cluster_5	Common_1_DN	76856	116127.497	115387	117013	468.1718	0.6618	-0.5955	0.001	0.001	45033	687127	69.845	11617	169240	17.203	5148	76856	7.8123	11.4316	11.1851	44.3144	45.4124
DP	TCF7_1	cluster_1	SP8	11837	17894.429	17410	18309	280.2445	0.6615	-0.5962	0.001	0.0010435	9326	149704	48.954	2397	37254	12.182	741	11837	3.8707	7.9455	7.9069	30.9136	31.7738
DN	CTCF_known2	cluster_1	SP8	12886	19489.472	18894	20113	352.1271	0.6612	-0.5969	0.001	0.0010435	17849	287162	34.367	3610	56958	6.8167	803	12886	1.5422	4.4989	4.4874	22.2438	22.6237
DN	ELK4_1	cluster_1	SP8	2956	4472.693	4386	4555	52.5417	0.661	-0.5973	0.001	0.001	11601	93425	84.428	674	5337	4.8231	369	2956	2.6713	3.1808	3.164	54.7478	55.3869
SP4	HIF1A_1	cluster_5	Common_1_DN	16293	24657.207	24305	24996	206.5982	0.6608	-0.5977	0.001	0.0010435	11555	130065	67.885	3336	36648	19.128	1443	16293	8.5038	12.4881	12.5268	43.2554	44.4581
DN	POU2F2_known13	cluster_1	SP8	4540	6871.537	6652	7092	135.271	0.6607	-0.5978	0.001	0.0010909	3197	67338	75.85	494	9555	10.763	221	4540	5.1139	6.9127	6.7421	44.7368	47.5144
DN	E2F8_1	cluster_1	SP8	5788	8760.877	8595	8927	102.2418	0.6607	-0.5979	0.001	0.001	9501	104773	81.344	1040	11009	8.5472	533	5788	4.4937	5.6099	5.5243	51.25	52.5752
DN	SMAD3_1	cluster_1	SP8	12661	19167.97	18940	19378	141.5203	0.6605	-0.5983	0.001	0.0010435	26078	216611	73.645	3179	26335	8.9536	1515	12661	4.3046	5.8095	5.845	47.6565	48.0767
DN	IKZF1_1	cluster_1	SP8	6210	9404.699	9237	9580	106.193	0.6603	-0.5987	0.001	0.0010435	11933	143526	82.189	1030	11778	6.7446	529	6210	3.5561	4.4331	4.3267	51.3592	52.7254
SP8	RUNX3_3	cluster_5	Common_1_DN	58170	88088.134	87228	88846	522.5941	0.6604	-0.5987	0.001	0.001	23891	424439	65.225	8022	136756	21.016	3341	58170	8.9392	13.9843	13.7051	41.648	42.5356
DN	SMAD3_3	cluster_1	SP8	2451	3712.483	3620	3814	62.3657	0.6603	-0.5988	0.001	0.0010435	4812	43323	75.24	570	5013	8.7061	275	2451	4.2567	5.7149	5.6575	48.2456	48.8929
DP	SOX1_3	cluster_3	DN	4004	6064.746	5818	6309	157.5731	0.6603	-0.5989	0.001	0.0010909	2535	35523	47.207	979	13064	17.361	290	4004	5.3209	11.4398	11.2716	29.6221	30.6491
DN	ATF4_3	cluster_1	SP8	4072	6170.825	6010	6347	105.6764	0.6599	-0.5996	0.001	0.0010909	4863	63288	72.723	688	8653	9.943	316	4072	4.6791	6.498	6.4341	45.9302	47.0588
SP4	ETV5_1	cluster_5	Common_1_DN	11620	17608.299	17385	17845	136.6182	0.6599	-0.5996	0.001	0.0010435	11580	105410	73.515	2699	24074	16.79	1288	11620	8.104	11.1226	11.0236	47.7214	48.2678
DN	NFE2L1::MAFG_1	cluster_1	SP8	13689	20750.472	20312	21127	247.4604	0.6597	-0.6001	0.001	0.0010435	11526	243206	74.83	1453	28629	8.8086	664	13689	4.2119	5.7609	5.6286	45.6986	47.8152
SP4	ATF3_known4	cluster_5	Common_1_DN	14092	21370.43	21030	21708	207.542	0.6594	-0.6007	0.001	0.001	8312	117305	66.718	2360	32327	18.386	1006	14092	8.0149	12.103	12.0131	42.6271	43.592
DN	SOX1_3	cluster_1	SP8	3832	5811.843	5638	6004	120.391	0.6594	-0.6008	0.001	0.0010909	3794	53161	70.646	648	8538	11.346	280	3832	5.0924	7.3801	7.2083	43.2099	44.8817
DN	ID4_1	cluster_1	SP8	12589	19097.764	18867	19354	160.2049	0.6592	-0.6012	0.001	0.0010435	48845	442152	75.691	2880	25444	4.3557	1399	12589	2.1551	2.8642	2.8472	48.5764	49.4773
DN	SOX5_3	cluster_1	SP8	3335	5060.683	4890	5203	96.3173	0.6591	-0.6015	0.001	0.0011429	2922	43910	74.555	516	7140	12.123	230	3335	5.6625	7.8713	7.5951	44.5736	46.7087
DP	ETV7_1	cluster_4	SP4	20748	31482.274	31026	31921	265.2887	0.659	-0.6015	0.001	0.001	25386	228951	51.568	6880	61259	13.798	2301	20748	4.6732	9.0641	9.0622	33.4448	33.8693
DN	SOX30_1	cluster_1	SP8	4464	6776.35	6576	6965	118.9411	0.6588	-0.6021	0.001	0.0010435	4394	65950	74.266	664	9477	10.672	303	4464	5.0269	6.8958	6.7688	45.6325	47.1035
DP	MYBL2_2	cluster_3	DN	3179	4827.238	4619	5009	116.9272	0.6586	-0.6025	0.001	0.0010435	2149	32599	60.53	562	8129	15.094	213	3179	5.9028	9.9116	9.7518	37.9004	39.1069
SP8	ARID5A_1	cluster_2	DP	514	781.068	700	850	46.185	0.6585	-0.6027	0.001	0.0012632	834	11458	67.591	100	1257	7.4151	39	514	3.0321	4.6763	4.4859	39	40.891
DN	FLI1_2	cluster_1	SP8	4121	6262.94	6097	6421	100.8659	0.6581	-0.6037	0.001	0.001	8020	104443	74.293	671	8532	6.069	318	4121	2.9314	3.9651	3.9457	47.392	48.3005
DP	RUNX_2	cluster_1	SP8	16652	25306.549	24753	25819	323.3717	0.658	-0.6038	0.001	0.0010435	17458	245690	47.274	3931	54123	10.414	1182	16652	3.2041	6.7705	6.7776	30.0687	30.767
DP	SPDEF_4	cluster_3	DN	9131	13879.302	13509	14319	225.4033	0.6579	-0.604	0.001	0.0010435	6274	94414	52.37	1919	27215	15.096	624	9131	5.0649	9.9458	9.6712	32.5169	33.5514
SP4	MYC_known20	cluster_5	Common_1_DN	20939	31829.134	31303	32341	330.5474	0.6579	-0.6041	0.001	0.001	8841	144867	57.464	3536	55966	22.2	1304	20939	8.3058	14.7495	14.4539	36.8778	37.4138
DP	SOX11_2	cluster_3	DN	4574	6955.589	6694	7224	161.2619	0.6576	-0.6046	0.001	0.0010909	3067	42939	50.606	1065	14154	16.681	331	4574	5.3907	10.7923	10.6523	31.0798	32.316
DN	FOXO6_3	cluster_1	SP8	21563	32794.118	32443	33192	231.4841	0.6575	-0.6049	0.001	0.0010435	28591	373838	76.349	3425	43712	8.9273	1662	21563	4.4038	5.813	5.768	48.5255	49.3297
DN	ATF4_2	cluster_1	SP8	3571	5431.919	5282	5604	95.184	0.6575	-0.605	0.001	0.0010909	4963	59634	73.418	650	7549	9.2939	302	3571	4.3964	6.085	5.9882	46.4615	47.3043
DP	POU2F2_known15	cluster_3	DN	3205	4875.252	4676	5084	120.2301	0.6575	-0.605	0.001	0.0010435	2667	29451	51.273	905	9687	16.865	295	3205	5.5797	11.0611	10.8825	32.5967	33.0856
SP4	CUX2_2	cluster_2	DP	141	214.974	190	241	16.2344	0.6575	-0.605	0.001	0.0013333	387	3483	71.8	36	310	6.3904	16	141	2.9066	4.1344	4.0482	44.4444	45.4839
DP	GATA_known17	cluster_4	SP4	5680	8641.684	8330	8969	195.0879	0.6573	-0.6053	0.001	0.0010909	3215	51515	48.405	1185	18187	17.089	356	5680	5.3371	11.0731	11.0259	30.0422	31.2311
DP	ELK4_2	cluster_3	DN	4766	7252.995	6949	7542	171.9166	0.6572	-0.6057	0.001	0.0011429	7680	69469	25.728	3169	28339	10.495	529	4766	1.7651	6.888	6.8606	16.693	16.8178
DP	MAF_known1	cluster_3	DN	39970	60834.133	59947	61546	461.567	0.657	-0.606	0.001	0.001	24308	367004	53.477	7841	115038	16.763	2661	39970	5.8242	10.947	10.8909	33.937	34.745
DP	FOXO4_3	cluster_4	SP4	2359	3591.268	3386	3783	116.6517	0.657	-0.6061	0.001	0.0010909	1828	23799	50.665	581	7296	15.532	183	2359	5.022	10.0109	9.9122	31.4974	32.3328
DN	XBP1_2	cluster_1	SP8	3558	5418.601	5241	5604	114.0999	0.6567	-0.6067	0.001	0.0010435	3231	74429	77.172	332	7249	7.5161	157	3558	3.6891	4.8592	4.7804	47.2892	49.0826
DN	TBX21_4	cluster_1	SP8	9547	14544.863	14263	14862	175.7251	0.6564	-0.6073	0.001	0.0010909	9894	148765	73.27	1404	20314	10.005	640	9547	4.7021	6.4686	6.4175	45.584	46.9971
DP	TCF3_1	cluster_3	DN	96816	147508.163	146467	148456	614.7628	0.6563	-0.6075	0.001	0.0011429	49855	709136	57.352	18450	258597	20.914	6806	96816	7.83	13.6516	13.6527	36.8889	37.4389
DP	SOX5_1	cluster_3	DN	1670	2545.47	2438	2667	71.6404	0.6562	-0.6078	0.001	0.0010909	1887	16983	50.471	590	5116	15.204	187	1670	4.963	9.9099	9.8334	31.6949	32.6427
DN	EWSR1::FLI1_1	cluster_1	SP8	51247	78110.069	77148	78903	563.2569	0.6561	-0.608	0.001	0.0010435	56590	1127364	69.149	6241	116963	7.1741	2638	51247	3.1433	4.6616	4.5457	42.2689	43.8147
DP	FOXO1_5	cluster_3	DN	29618	45147.538	44625	45702	328.6878	0.656	-0.6082	0.001	0.0010435	23967	264888	55.536	7636	82243	17.243	2695	29618	6.2096	11.2446	11.1813	35.2933	36.0128
DP	IKZF2_2	cluster_3	DN	3443	5249.148	5063	5411	102.179	0.656	-0.6083	0.001	0.0010435	3311	33116	55.275	1013	9683	16.162	348	3443	5.7469	10.5104	10.3968	34.3534	35.5572
DP	SPIB_2	cluster_4	SP4	18231	27794.057	27307	28254	284.8581	0.6559	-0.6084	0.001	0.0010435	16381	214053	53.087	4131	52835	13.103	1398	18231	4.5214	8.5343	8.517	33.8417	34.5055
DN	ATF7_1	cluster_1	SP8	3213	4900.101	4736	5052	99.3042	0.6558	-0.6087	0.001	0.001	3904	50828	69.581	561	7117	9.7428	249	3213	4.3984	6.3781	6.3213	44.385	45.1454
DN	TBX21_6	cluster_1	SP8	12191	18597.179	18225	18901	215.0523	0.6555	-0.6092	0.001	0.0010909	10503	189588	74.498	1472	25611	10.064	681	12191	4.7904	6.4839	6.4303	46.2636	47.6006
DN	TBX19_1	cluster_1	SP8	5673	8657.669	8393	8947	159.7699	0.6553	-0.6098	0.001	0.0010435	4779	91001	71.181	692	12512	9.7869	300	5673	4.4374	6.2775	6.234	43.3526	45.3405
DP	IRF_known21	cluster_3	DN	8312	12686.663	12340	13055	226.4865	0.6552	-0.61	0.001	0.001	6273	90082	51.133	1832	25304	14.363	587	8312	4.7181	9.3576	9.2271	32.0415	32.8486
SP4	EGR3_3	cluster_5	Common_1_DN	51155	78074.939	77482	78707	374.0127	0.6552	-0.61	0.001	0.001	31018	457668	70.037	7938	112982	17.29	3518	51155	7.8282	11.3418	11.1773	44.3185	45.2771
DN	MTF1_3	cluster_1	SP8	9454	14433.277	14226	14651	132.3392	0.655	-0.6103	0.001	0.001	16298	213937	79.022	1462	18557	6.8544	725	9454	3.492	4.4484	4.4191	49.5896	50.9457
DN	SMAD_2	cluster_1	SP8	16644	25418.361	25115	25709	183.3478	0.6548	-0.6108	0.001	0.0010435	28215	291809	73.323	3462	35177	8.8389	1611	16644	4.1821	5.7097	5.7037	46.5338	47.315
DP	RUNX1_2	cluster_3	DN	5865	8956.857	8656	9253	180.1561	0.6548	-0.6108	0.001	0.0010435	3573	55648	49.329	1268	18623	16.508	388	5865	5.199	10.8592	10.5395	30.5994	31.4933
DP	TCF3_2	cluster_4	SP4	12802	19550.215	19104	20021	279.4422	0.6548	-0.6108	0.001	0.0010435	27591	417427	54.427	2555	36581	4.7697	856	12802	1.6692	3.1025	3.0669	33.5029	34.9963
DN	FOXP3_1	cluster_1	SP8	10029	15317.068	15003	15608	187.213	0.6548	-0.6109	0.001	0.0010909	9966	159936	72.213	1425	21824	9.8538	633	10029	4.5282	6.3516	6.2706	44.4211	45.954
DP	FOXO6_1	cluster_4	SP4	2862	4372.616	4182	4563	115.1509	0.6546	-0.6113	0.001	0.0010909	2247	29326	51.149	700	8761	15.281	221	2862	4.9918	9.8353	9.7593	31.5714	32.6675
DP	FOXM1_1	cluster_3	DN	4024	6155.575	5993	6320	106.0712	0.6538	-0.6131	0.001	0.0010909	4728	37858	53.335	1490	11672	16.444	505	4024	5.6691	10.681	10.6292	33.8926	34.4757
DP	SOX13_1	cluster_3	DN	2595	3972.219	3773	4182	124.4442	0.6534	-0.614	0.001	0.0010909	1690	25424	51.067	581	8084	16.237	178	2595	5.2123	10.5325	10.2069	30.6368	32.1004
DP	HESX1_2	cluster_3	DN	1165	1783.622	1649	1919	79.5139	0.6534	-0.6141	0.001	0.0014118	733	10262	51.563	281	3567	17.923	87	1165	5.8537	11.869	11.3526	30.9609	32.6605
DP	GATA_known17	cluster_1	SP8	3249	4975.288	4732	5239	144.2349	0.6531	-0.6146	0.001	0.0010909	3215	51515	48.405	690	10533	9.8972	207	3249	3.0529	6.4386	6.3069	30	30.8459
DP	MTF1_3	cluster_3	DN	16774	25687.282	25256	26169	270.3226	0.653	-0.6148	0.001	0.001	12915	169509	62.611	3233	41341	15.27	1289	16774	6.1958	9.9806	9.8956	39.8701	40.5747
DP	PBX3_known1	cluster_3	DN	7534	11541.27	11214	11851	181.0077	0.6528	-0.6152	0.001	0.0010435	6370	70693	52.566	2087	22368	16.633	693	7534	5.6022	10.8791	10.6573	33.2056	33.682
SP4	RUNX2_5	cluster_5	Common_1_DN	42819	65609.273	64943	66384	467.586	0.6526	-0.6156	0.001	0.001	17450	305925	60.5	6528	109696	21.693	2480	42819	8.4679	14.212	13.9966	37.9902	39.0342
DN	RUNX3_3	cluster_1	SP8	22903	35097.575	34675	35473	237.4364	0.6526	-0.6158	0.001	0.001	29706	527830	81.113	2618	44080	6.7739	1328	22903	3.5196	4.4705	4.3391	50.7257	51.9578
DN	E2F2_2	cluster_1	SP8	3842	5891.447	5707	6065	107.3134	0.6522	-0.6166	0.001	0.0010909	3788	64579	78.928	488	7854	9.5991	232	3842	4.6957	6.1246	5.9493	47.541	48.9177
DN	POU2F2_known12	cluster_1	SP8	3774	5788.922	5625	5924	89.0441	0.652	-0.6171	0.001	0.0010435	5446	54541	74.724	834	7995	10.954	388	3774	5.1706	7.1245	6.9196	46.5228	47.2045
DP	TCF7L2_known5	cluster_1	SP8	11515	17673.361	17278	18123	260.1666	0.6516	-0.618	0.001	0.0010435	9748	156401	49.931	2312	36014	11.497	722	11515	3.6762	7.4066	7.3625	31.2284	31.9737
DP	SOX17_4	cluster_4	SP4	2907	4463.901	4271	4666	129.2869	0.6513	-0.6186	0.001	0.0010909	2192	30688	48.463	725	9491	14.988	214	2907	4.5908	9.7628	9.4728	29.5172	30.629
DN	RUNX2_5	cluster_1	SP8	19434	29844.532	29450	30222	229.6907	0.6512	-0.6189	0.001	0.001	23276	408017	80.689	2260	37691	7.4537	1137	19434	3.8433	4.8849	4.763	50.3097	51.5614
DN	IRF6_1	cluster_1	SP8	3123	4797.771	4642	4933	88.6303	0.651	-0.6193	0.001	0.0010909	3319	53207	81.206	391	6065	9.2566	196	3123	4.7664	5.9054	5.8695	50.1279	51.4922
DP	ATF2_1	cluster_1	SP8	392	602.794	561	648	27.7594	0.6509	-0.6195	0.001	0.0010435	1296	9072	66.75	131	917	6.7471	56	392	2.8843	4.321	4.321	42.7481	42.7481
DN	E2F7_1	cluster_1	SP8	3814	5861.456	5698	5996	89.0058	0.6508	-0.6198	0.001	0.0010435	5640	73401	81.261	603	7441	8.2378	296	3814	4.2224	5.2482	5.1961	49.0879	51.2566
DP	RUNX1_2	cluster_1	SP8	3859	5933.579	5694	6204	159.8082	0.6504	-0.6205	0.001	0.0010435	3573	55648	49.329	859	12459	11.044	255	3859	3.4208	7.1369	6.9347	29.6857	30.9736
DP	SOX8_9	cluster_3	DN	3309	5088.058	4823	5354	157.8985	0.6504	-0.6206	0.001	0.0010909	2184	34944	45.366	784	11527	14.965	213	3309	4.296	9.7527	9.4694	27.1684	28.7065
DP	RREB1_2	cluster_3	DN	112124	172435.079	170815	173884	941.1949	0.6502	-0.621	0.001	0.001	48689	1172396	58.91	14003	307557	15.454	4887	112124	5.634	10.0372	9.5637	34.8997	36.4563
DN	PBX3_known1	cluster_1	SP8	5621	8650.001	8453	8848	120.4799	0.6499	-0.6218	0.001	0.0010435	8906	98882	73.528	1114	11983	8.9104	511	5621	4.1797	5.7377	5.6846	45.8707	46.9081
DN	MAFF_1	cluster_1	SP8	8996	13846.719	13572	14113	170.487	0.6497	-0.6221	0.001	0.001	12562	176219	70.456	1463	19885	7.9504	643	8996	3.5968	5.1186	5.105	43.9508	45.2401
DP	SOX9_4	cluster_4	SP4	6290	9681.36	9352	9985	194.9669	0.6497	-0.6221	0.001	0.0010909	4428	66520	51.448	1338	19227	14.871	429	6290	4.8648	9.6883	9.4558	32.0628	32.7144
DP	SOX17_5	cluster_4	SP4	2705	4164.087	3958	4388	137.4195	0.6497	-0.6222	0.001	0.0011429	1842	25857	46.902	691	9164	16.623	198	2705	4.9066	10.7492	10.4614	28.6541	29.5177
DN	ARID5A_1	cluster_1	SP8	1080	1663.534	1583	1747	52.1952	0.6494	-0.6228	0.001	0.0012632	992	13600	80.227	182	2224	13.119	84	1080	6.3709	8.4677	7.9412	46.1538	48.5612
DN	MGA_3	cluster_1	SP8	6064	9338.415	9110	9553	140.7543	0.6494	-0.6228	0.001	0.0010909	6415	109267	77.12	760	12400	8.7519	361	6064	4.2799	5.6274	5.5497	47.5	48.9032
DP	SOX2_6	cluster_4	SP4	2763	4255.692	4039	4467	130.9316	0.6493	-0.623	0.001	0.0010435	1589	25439	47.17	627	9364	17.363	176	2763	5.1232	11.0761	10.8613	28.0702	29.5066
DN	TCF4_2	cluster_1	SP8	13017	20053.785	19807	20285	142.3748	0.6491	-0.6234	0.001	0.001	45335	409696	76.215	3017	26562	4.9413	1448	13017	2.4215	3.194	3.1772	47.9947	49.0061
DN	ETS_known13	cluster_1	SP8	5907	9100.762	8901	9319	127.6362	0.6491	-0.6235	0.001	0.001	12306	197388	80.739	743	11441	4.6798	372	5907	2.4162	3.0229	2.9926	50.0673	51.6301
DP	SOX4_2	cluster_4	SP4	5399	8319.441	8013	8623	185.9474	0.649	-0.6237	0.001	0.0010909	3926	59025	51.393	1180	16678	14.521	367	5399	4.7009	9.3479	9.147	31.1017	32.372
DN	MTF1_1	cluster_1	SP8	20757	31998.894	31705	32305	195.8361	0.6487	-0.6244	0.001	0.001	48686	649383	82.303	3054	39477	5.0033	1577	20757	2.6308	3.2391	3.1964	51.6372	52.58
DN	PBX1_4	cluster_1	SP8	2881	4442.87	4283	4583	87.3747	0.6485	-0.6247	0.001	0.0010909	3459	38700	71.853	591	6347	11.784	261	2881	5.3491	7.5455	7.4444	44.1624	45.3915
DN	SPDEF_6	cluster_1	SP8	4129	6367.578	6214	6521	92.3989	0.6485	-0.6248	0.001	0.001	10392	145946	84.321	572	7714	4.4568	297	4129	2.3856	2.858	2.8291	51.9231	53.5261
DP	ATF4_2	cluster_4	SP4	3274	5050.484	4869	5230	118.0318	0.6483	-0.6252	0.001	0.0010909	3457	41526	51.125	871	10071	12.399	274	3274	4.0308	7.9259	7.8842	31.4581	32.5092
DN	NFATC2_1	cluster_1	SP8	2635	4065.018	3992	4145	46.5075	0.6483	-0.6253	0.001	0.0010909	7464	44784	80.238	877	5163	9.2504	444	2635	4.721	5.9486	5.8838	50.6271	51.0362
DP	SPIC_1	cluster_3	DN	19145	29538.264	29029	30041	290.9573	0.6482	-0.6256	0.001	0.001	13902	181322	51.494	4562	57978	16.465	1474	19145	5.4371	10.6028	10.5586	32.3104	33.0211

DP	TCF7_2	cluster_1	SP8	8838	13636.151	13338	13971	191.5283	0.6482	-0.6256	0.001	0.0010435	10707	118115	49.917	2583	27660	11.689	808	8838	3.735	7.5465	7.4825	31.2815	31.9523
DN	TBX5_5	cluster_1	SP8	8495	13108.332	12811	13428	181.7856	0.6481	-0.6257	0.001	0.0010435	7574	144355	74.742	980	17964	9.3011	450	8495	4.3984	5.9414	5.8848	45.9184	47.289
DN	TCF4_1	cluster_1	SP8	12518	19318.432	19039	19573	162.7415	0.648	-0.6259	0.001	0.0010909	54166	490170	74.751	2953	26049	3.9725	1389	12518	1.909	2.5643	2.5538	47.0369	48.0556
DP	ETV2_1	cluster_4	SP4	14183	21893.994	21467	22287	241.9131	0.6478	-0.6263	0.001	0.0010435	15356	154289	52.346	4238	42029	14.259	1415	14183	4.8119	9.2146	9.1925	33.3884	33.7457
DN	XBP1_4	cluster_1	SP8	3662	5653.542	5494	5823	102.5789	0.6478	-0.6264	0.001	0.0010435	6101	79442	72.628	616	7855	7.1813	282	3662	3.3479	4.6222	4.6097	45.7792	46.62
DP	IKZF1_2	cluster_3	DN	17355	26800.192	26449	27153	219.1404	0.6476	-0.6269	0.001	0.0010435	20144	161159	58.804	5902	46144	16.837	2186	17355	6.3326	10.8519	10.7689	37.0383	37.6105
DP	PKNOX1_2	cluster_1	SP8	5430	8392.324	8111	8648	166.8821	0.6471	-0.628	0.001	0.0010435	9569	106189	50.266	1569	16823	7.9634	494	5430	2.5704	5.1625	5.1135	31.485	32.2772
SP8	AHR::ARNT::HIF1A_1	cluster_5	Common_1_DN	12231	18909.3	18661	19125	142.0686	0.6468	-0.6285	0.001	0.0010435	12611	103685	71.095	3290	26751	18.343	1484	12231	8.3866	11.7675	11.7963	45.1064	45.7217
DN	POU2F2_known16	cluster_1	SP8	2755	4260.068	4096	4399	92.2465	0.6468	-0.6286	0.001	0.0010909	3024	39849	67.778	529	6610	11.243	215	2755	4.6859	7.1098	6.9136	40.6427	41.6793
DP	GATA_known16	cluster_4	SP4	9501	14691.134	14184	15168	299.7355	0.6467	-0.6287	0.001	0.0010435	4004	84333	48.865	1561	30959	17.939	458	9501	5.5051	11.4386	11.2661	29.3402	30.689
DP	ELK3_2	cluster_4	SP4	3339	5163.696	4947	5416	144.4248	0.6467	-0.6288	0.001	0.0011429	6179	55790	26.533	2198	19577	9.3107	371	3339	1.588	6.0042	5.9849	16.879	17.0557
DP	SPIC_1	cluster_4	SP4	16337	25261.379	24829	25721	281.0431	0.6467	-0.6288	0.001	0.001	13902	181322	51.494	3926	49711	14.118	1256	16337	4.6396	9.0347	9.0099	31.9918	32.864
DP	CUX2_1	cluster_1	SP8	494	764.755	669	865	60.4225	0.6464	-0.6295	0.001	0.0012632	328	6022	56.539	91	1512	14.196	30	494	4.6381	9.1463	8.2033	32.967	32.672
DP	FOXP3_2	cluster_4	SP4	2208	3418.118	3302	3527	68.7916	0.6461	-0.6302	0.001	0.0010909	4108	24648	54.212	1078	6365	13.999	368	2208	4.8564	8.9581	8.9581	34.1373	34.6897
DP	TCF7_1	cluster_4	SP4	18595	28785.157	28257	29364	341.5373	0.646	-0.6304	0.001	0.0010435	9326	149704	48.954	3834	59721	19.529	1165	18595	6.0806	12.492	12.4212	30.386	31.1365
DN	TBX2_1	cluster_1	SP8	10570	16366.676	16052	16646	179.1099	0.6458	-0.6307	0.001	0.0010435	11067	188839	77.429	1317	21623	8.866	627	10570	4.334	5.6655	5.5974	47.6082	48.8831
DP	SPI1_known4	cluster_3	DN	18848	29205.677	28746	29768	291.1554	0.6454	-0.6318	0.001	0.0010435	13368	174316	53.393	4360	55279	16.932	1457	18848	5.7731	10.8992	10.8125	33.4174	34.0961
DN	SOX2_6	cluster_1	SP8	2715	4208.506	4050	4364	96.7034	0.6452	-0.6322	0.001	0.0010435	2362	37807	70.103	424	6325	11.728	172	2715	5.0342	7.282	7.1812	40.566	42.9249
DP	SOX9_6	cluster_4	SP4	5367	8319.664	8026	8620	177.0932	0.6451	-0.6323	0.001	0.0010909	3625	54432	49.299	1220	17382	15.743	361	5367	4.8608	9.9586	9.86	29.5902	30.8768
DP	SPIC_2	cluster_3	DN	20393	31626.208	31057	32149	323.8888	0.6448	-0.633	0.001	0.0010435	14989	195470	52.699	4773	60618	16.343	1572	20393	5.498	10.4877	10.4328	32.9353	33.6418
DP	HESX1_2	cluster_4	SP4	798	1238.522	1118	1351	71.8033	0.6446	-0.6335	0.001	0.0014118	733	10262	51.563	192	2490	12.511	57	798	4.0096	7.7763	7.7763	29.6875	32.0482
DP	TCF7L1_1	cluster_4	SP4	17002	26376.64	25854	26887	311.4073	0.6446	-0.6335	0.001	0.0010435	8494	136288	48.648	3526	55052	19.651	1068	17002	6.0688	12.5736	12.4751	30.2893	30.8835
DP	TCF7L1_2	cluster_1	SP8	8384	13011.504	12675	13313	192.1531	0.6444	-0.634	0.001	0.0010435	10535	116448	51.095	2407	25849	11.342	766	8384	3.6787	7.271	7.1998	31.8238	32.4345
DN	POU2F2_known5	cluster_1	SP8	4155	6450.717	6299	6608	100.0406	0.6442	-0.6345	0.001	0.0010909	4660	60686	74.223	730	8982	10.986	329	4155	5.0818	7.0601	6.8467	45.0685	46.2592
DP	SOX14_3	cluster_3	DN	4248	6596.317	6355	6845	149.9002	0.644	-0.6348	0.001	0.0010909	2759	38663	47.284	1064	14226	17.398	308	4248	5.1952	11.1635	10.9872	28.9474	29.8608
DN	E2F_known24	cluster_1	SP8	3364	5224.621	5057	5383	101.9858	0.6439	-0.635	0.001	0.001	3603	61420	79.657	422	6854	8.8891	201	3364	4.3628	5.5787	5.477	47.6303	49.0808
DP	SOX18_2	cluster_4	SP4	3043	4727.434	4542	4955	127.5175	0.6438	-0.6354	0.001	0.0010909	2288	32032	48.779	766	10021	15.26	223	3043	4.6339	9.7465	9.4999	29.1123	30.3662
DP	PKNOX2_3	cluster_1	SP8	5569	8655.391	8405	8887	148.5395	0.6435	-0.6361	0.001	0.0010435	9600	106895	49.891	1632	17576	8.2032	503	5569	2.5992	5.2396	5.2098	30.8211	31.6853
DP	IRF4_2	cluster_3	DN	7154	11122.204	10774	11484	206.9523	0.6432	-0.6365	0.001	0.001	5446	77752	53.165	1556	21390	14.626	509	7154	4.8917	9.3463	9.201	32.7121	33.4455
DN	MESP1_1	cluster_1	SP8	11916	18530.205	18316	18757	139.4411	0.6431	-0.6369	0.001	0.001	44484	401632	76.45	2782	24456	4.6552	1328	11916	2.2682	2.9853	2.9669	47.7354	48.7242
DP	SPIB_1	cluster_4	SP4	8469	13173.45	12946	13395	134.5718	0.6429	-0.6373	0.001	0.0010435	15545	93270	50.008	4466	26536	14.228	1415	8469	4.5408	9.1026	9.0801	31.6838	31.9151
DP	ELK3_1	cluster_4	SP4	3357	5223.476	5001	5461	136.106	0.6427	-0.6377	0.001	0.0011429	6415	57946	26.824	2201	19599	9.0727	373	3357	1.554	5.8145	5.7933	16.9468	17.1284
DP	SPIB_2	cluster_3	DN	22559	35098.025	34562	35626	337.577	0.6428	-0.6377	0.001	0.0010435	16381	214053	53.087	5238	66763	16.558	1732	22559	5.5948	10.5732	10.539	33.0661	33.7897
DP	FOXO6_3	cluster_3	DN	29713	46239.852	45647	46885	370.4015	0.6426	-0.638	0.001	0.0010435	21070	275513	56.268	6537	83159	16.984	2293	29713	6.0683	10.8828	10.7846	35.0773	35.7303
DP	TCF21_1	cluster_4	SP4	6001	9341.132	9060	9646	176.3193	0.6425	-0.6383	0.001	0.001	11430	148923	52.886	1441	17927	6.3663	466	6001	2.1311	4.077	4.0296	32.3387	33.4746
DN	REL_1	cluster_1	SP8	8157	12697.73	12503	12896	121.4742	0.6424	-0.6384	0.001	0.001	15073	136291	76.106	1898	16902	9.4382	905	8157	4.5549	6.0041	5.985	47.6818	48.2606
DP	SOX12_1	cluster_4	SP4	3343	5205.302	4986	5414	127.2156	0.6423	-0.6387	0.001	0.0010435	2452	31970	50.662	869	10644	16.867	264	3343	5.2975	10.7667	10.4567	30.3797	31.4074
DN	LMO2_2	cluster_1	SP8	2010	3130.992	3030	3231	60.8598	0.6421	-0.6392	0.001	0.0010435	4777	38246	75.086	541	4238	8.3202	252	2010	3.9461	5.2753	5.2555	46.5804	47.428
DP	MAF_known10	cluster_1	SP8	5116	7973.784	7751	8179	140.3705	0.6416	-0.6401	0.001	0.0010909	7909	87167	53.303	1423	15177	9.2809	469	5116	3.1285	5.93	5.8692	32.9585	33.7089
DP	TCF7_2	cluster_4	SP4	14129	22025.443	21572	22417	247.6098	0.6415	-0.6405	0.001	0.0010435	10707	118115	49.917	4118	44691	18.887	1282	14129	5.9711	11.9735	11.9621	31.1316	31.6149
DP	TCF7L2_known5	cluster_4	SP4	17890	27888.592	27370	28480	335.2429	0.6415	-0.6405	0.001	0.0010435	9748	156401	49.931	3628	56623	18.077	1121	17890	5.7114	11.4998	11.4385	30.8986	31.5949
SP4	RUNX3_3	cluster_5	Common_1_DN	54010	84217.976	83344	84943	498.8421	0.6413	-0.6409	0.001	0.001	22811	405311	62.285	8022	136756	21.016	3108	54010	8.2999	13.625	13.3256	38.7435	39.4937
DN	CTCF_known1	cluster_1	SP8	20592	32115.099	31319	32970	514.6297	0.6412	-0.6411	0.001	0.0010435	26314	479096	27.636	6625	118066	6.8106	1145	20592	1.1878	4.3513	4.2981	17.283	17.4411
DP	IKZF2_2	cluster_1	SP8	1602	2500.265	2370	2634	81.2839	0.6409	-0.6419	0.001	0.0010435	3311	33116	55.275	483	4645	7.7532	161	1602	2.674	4.8626	4.8375	33.3333	34.4887
DP	ETV2_1	cluster_3	DN	11782	18387.303	18049	18737	209.3955	0.6408	-0.6421	0.001	0.0010435	15356	154289	52.346	3573	35299	11.976	1178	11782	3.9973	7.6713	7.6363	32.9695	33.3777
SP4	AHR::ARNT::HIF1A_1	cluster_5	Common_1_DN	12094	18878.732	18635	19129	155.8836	0.6406	-0.6424	0.001	0.0010435	12561	103445	70.93	3290	26751	18.343	1457	12094	8.2926	11.5994	11.6912	44.2857	45.2095
DP	SOX14_3	cluster_4	SP4	3573	5578.236	5346	5821	146.428	0.6406	-0.6425	0.001	0.0010909	2759	38663	47.284	902	12080	14.774	259	3573	4.3697	9.3875	9.2414	28.714	29.5778
DP	SOX1_2	cluster_3	DN	4132	6451.556	6218	6711	150.9321	0.6405	-0.6427	0.001	0.0010909	2774	38842	48.471	1031	13594	16.964	298	4132	5.1564	10.7426	10.638	28.904	30.3958
DP	SOX14_2	cluster_4	SP4	2877	4493.92	4324	4671	108.8162	0.6403	-0.6432	0.001	0.0010909	2895	31972	49.947	866	9223	14.408	263	2877	4.4945	9.0846	8.9985	30.3695	31.1938
DP	ETV3_1	cluster_3	DN	8349	13040.76	12831	13285	139.4588	0.6403	-0.6433	0.001	0.0010435	13947	126127	66.782	2201	19610	10.383	928	8349	4.4207	6.6538	6.6195	42.1627	42.5752
DP	TCF7L2_known7	cluster_4	SP4	17095	26721.651	26316	27158	280.1177	0.6398	-0.6444	0.001	0.0010435	10010	140599	49.374	4019	55081	19.343	1222	17095	6.0032	12.2078	12.1587	30.4056	31.0361
DN	POU2F2_known11	cluster_1	SP8	3743	5852.449	5700	5981	90.0797	0.6396	-0.6447	0.001	0.0010435	5502	55147	73.881	843	8123	10.882	383	3743	5.0145	6.9611	6.7873	45.433	46.079
DN	REL_2	cluster_1	SP8	7975	12470.937	12289	12663	110.2497	0.6395	-0.6449	0.001	0.0010435	15212	137460	76.313	1862	16571	9.1996	885	7975	4.4274	5.8178	5.8017	47.5295	48.1262
DN	ATF2_2	cluster_1	SP8	2852	4461.136	4313	4614	82.5439	0.6394	-0.6453	0.001	0.0010435	5805	63978	74.542	567	6053	7.0525	260	2852	3.3229	4.4789	4.4578	45.8554	47.1171
SP8	GMEB1_1	cluster_5	Common_1_DN	11297	17671.257	17355	17961	185.1641	0.6393	-0.6454	0.001	0.0010435	6106	99511	72.498	1557	24649	17.958	701	11297	8.2303	11.4805	11.3525	45.0225	45.8315
DN	TBX4_2	cluster_1	SP8	9285	14542.418	14217	14832	195.1257	0.6385	-0.6472	0.001	0.0010909	8920	170122	76.154	1068	19559	8.7555	493	9285	4.1564	5.5269	5.4578	46.161	47.4718
DN	HES1_1	cluster_1	SP8	17253	27025.428	26664	27385	210.1286	0.6384	-0.6474	0.001	0.001	30958	438366	78.548	2546	34903	6.2541	1226	17253	3.0915	3.9602	3.9358	48.154	49.4313
DP	ATF4_3	cluster_3	DN	4568	7162.375	6928	7429	147.1091	0.6378	-0.6488	0.001	0.0010909	3413	44407	51.027	1124	14204	16.322	355	4568	5.249	10.4014	10.2867	31.5836	32.16
DP	PKNOX2_2	cluster_1	SP8	5911	9274.734	9006	9570	176.3822	0.6374	-0.6498	0.001	0.0010435	11043	122680	50.42	1726	18579	7.6357	535	5911	2.4294	4.8447	4.8182	30.9965	31.8155
DP	SOX2_5	cluster_4	SP4	3953	6204.421	5945	6469	154.012	0.6372	-0.6502	0.001	0.0010909	2721	43557	49.369	874	13039	14.779	252	3953	4.4804	9.2613	9.0755	28.833	30.3167
DP	SOX11_2	cluster_4	SP4	4089	6418.848	6176	6666	151.0516	0.6371	-0.6504	0.001	0.0010909	3067	42939	50.606	979	13014	15.338	297	4089	4.8191	9.6837	9.5228	30.3371	31.42
DN	FOXM1_1	cluster_1	SP8	3043	4779.69	4660	4898	74.5716	0.6367	-0.6513	0.001	0.0010909	6570	52629	74.145	835	6541	9.2151	380	3043	4.2871	5.7839	5.782	45.509	46.5219
DP	TCF7L2_known7	cluster_1	SP8	10256	16112.602	15691	16523	253.9095	0.6365	-0.6517	0.001	0.0010435	10010	140599	49.374	2430	33136	11.636	735	10256	3.6016	7.3427	7.2945	30.2469	30.9512
DN	SPZ1_2	cluster_1	SP8	9248	14535.032	14278	14749	143.1995	0.6363	-0.6523	0.001	0.0010435	18565	186152	75.582	1996	19554	7.9394	930	9248	3.7549	5.0094	4.968	46.5932	47.2947
DP	REL_1	cluster_3	DN	9474	14897.485	14614	15168	169.2723	0.636	-0.653	0.001	0.001	11260	101830	56.863	3006	26515	14.806	1055	9474	5.2903	9.3694	9.3037	35.0965	35.7307
DP	RBPJ_1	cluster_3	DN	9944	15638.823	15367	15860	151.8831	0.6359	-0.6532	0.001	0.0010435	10434	73042	50.795	4517	31035	21.582	1430	9944	6.9153	13.7052	13.6141	31.6582	32.0412
DP	ETV4_2	cluster_4	SP4	2205	3472.253	3280	3665	113.1592	0.6351	-0.6549	0.001	0.0011429	5095	46084	26.755	1473	13170	7.646	245	2205	1.2801	4.8086	4.7847	16.6327	16.7426
DP	EWSR1::FLI1_1	cluster_3	DN	83851	132072.254	130589	133363	831.0677	0.6349	-0.6554	0.001	0.0010435	43660	868688	53.282	13531	254490	15.61	4273	83851	5.1431	9.787	9.6526	31.5793	32.9486
DP	NFATC1_3	cluster_1	SP8	2157	3397.989	3223	3569	112.7582	0.6349	-0.6554	0.001	0.0010909	2771	36045	53.14	541	6612	9.7479	169	2157	3.18	6.0989	5.9842	31.2384	32.6225
DN	SOX7_4	cluster_1	SP8	3556	5603.207	5408	5799	114.6332	0.6347	-0.6558	0.001	0.0010909	3341	53567	71.584	551	8220	10.985	229	3556	4.752	6.8542	6.6384	41.5608	43.2603
DN	TCF3_1	cluster_1	SP8	8407	13248.932	12757	13766	301.2813	0.6346	-0.6562	0.001	0.0011429	20600	290144	23.465	4255	58143	4.7023	607	8407	0.67992	2.9466	2.8975	14.2656	14.4592
DN	MAF_known11	cluster_1	SP8	10075	15885.592	15543	16206	199.8242	0.6342	-0.6569	0.001	0.0010435	13743	221582	72.337	1445	22364	7.3009	628	10075	3.2891	4.5696	4.5468	43.4602	45.0501
DP	HMGA1_1	cluster_1	SP8	1220	1925.788	1842	2014	52.1787	0.6337	-0.6581	0.001	0.0010435	3004	18138	53.492	611	3646	10.753	203	1220	3.598	6.7577	6.7262	33.2242	33.4613
DP	SPI1_known1	cluster_4	SP4	19916	31428.76	31035	31873	250.4833	0.6337	-0.6581	0.001	0.001	34618	243610	52.166	8708	60564	12.969	2838	19916	4.2648	8.198	8.1754	32.5907	32.8842
DP	FOXP3_2	cluster_1	SP8	1799	2840.194	2737	2943	62.3795	0.6335	-0.6585	0.001	0.0010909	4108	24648	54.212	895	5284	11.622	300	1799	3.9568	7.3028	7.2988	33.5196	34.0462
DP	GATA_known16	cluster_1	SP8	4853	7661.78	7348	8053	225.2232	0.6335	-0.6587	0.001	0.0010435	4004	84333	48.865	822	16180	9.3751	233	4853	2.812	5.8192	5.7546	28.3455	29.9938
DP	SOX21_2	cluster_4	SP4	3793	5989.645	5743	6255	152.1137	0.6333	-0.659	0.001	0.0010909	3145	44041	49.589	940	12505	14.08	277	3793	4.2708	8.8076	8.6124	29.4681	30.3319
DP	REL_2	cluster_3	DN	9532	15052.986	14781	15348	175.9959	0.6333	-0.6591	0.001	0.0010435	11409	103099	57.237	3018	26629	14.783	1062	9532	5.2918	9.3084	9.2455	35.1889	35.7956
DN	TCF12_known1	cluster_1	SP8	12251	19347.751	19145	19558	127.152	0.6332	-0.6592	0.001	0.0010909	48943	352576	75.728	3620	25716	5.5234	1712	12251	2.6313	3.4979	3.4747	47.2928	47.6396
DP	SOX10_4	cluster_4	SP4	6449	10186.509	9896	10519	198.6133	0.6331	-0.6594	0.001	0.0010909	4770	71762	51.378	1406	20226	14.481	436	6449	4.6172	9.1405	8.9867	31.01	31.8847
DN	SMAD4_1	cluster_1	SP8	20850	32934.871	32504	33373	253.2079	0.6331	-0.6595	0.001	0.001	32547	462220	75.022	3229	44540	7.2292	1480	20850	3.3841	4.5473	4.5108	45.8346	46.8119
DN	MAFG_1	cluster_1	SP8	8265	13056.203	12718	13338	195.1785	0.6331	-0.6596	0.001	0.0010435	8246	165248	72.352	971	18472	8.0878	416	8265	3.6188	5.0449	5.0016	42.8424	44.7434
DP	POU2F2_known16	cluster_4	SP4	2594	4098.578	3931	4317	118.1372	0.633	-0.6597	0.001	0.0010909	2099	27573	46.898	737	9083	15.449	202	2594	4.4121	9.6236	9.4078	27.4084	28.5588
DP	ELK4_1	cluster_3	DN	4229	6682.351	6555	6828	85.8407	0.6329	-0.6599	0.001	0.001	10661	85897	77.625	1097	8678	7.8423	529	4229	3.8218	4.962	4.9233	48.2224	48.7324
DN	PATZ1_1	cluster_1	SP8	46459	73408.93	72881	73918	326.4199	0.6329	-0.66	0.001	0.001	85759	1123464	76.273	7774	98544	6.6902	3599	46459	3.1541	4.1966	4.1353	46.2953	47.1454
DN	IKZF3_1	cluster_1	SP8	4509	7129.705	6980	7287	94.6367	0.6325	-0.6609	0.001	0.0010435	8816	106058	78.616	817	9334	6.9189	387	4509	3.3423	4.3897	4.2514	47.3684	48.3073
DP	NFKB_known11	cluster_3	DN	10685	16903.929	16606	17243	196.3792	0.6321	-0.6617	0.001	0.001	11257	136572	59.553	2446	28875	12.591	889	10685	4.6593	7.8973	7.8237	36.3451	37.0043
DP	MYBL2_4	cluster_3	DN	2027	3207.916	3040	3361	97.9476	0.632	-0.662	0.001	0.0010435	1563	21943	61.958	389	5253	14.832	145	2027	5.7234	9.277	9.2376	37.2751	38.5875
DP	SOX1_2	cluster_4	SP4	3382	5353.176	5131	5591	139.9298	0.6318	-0.6624	0.001	0.0010909	2774	38842	48.471	855	11323	14.13	244	3382	4.2204	8.796	8.7071	28.538	29.8684
DP	IRF_known14	cluster_3	DN	21447	33971.913	33305	34714	435.2367	0.6313	-0.6635	0.001	0.001	11310	235509	53.453	3403	65717	14.916	1061	21447	4.8678	9.3811	9.1067	31.1784	32.6354
DP	SOX18_4	cluster_4	SP4	2419	3831.958	3639	4037	118.7469	0.6314	-0.6635	0.001	0.0012	1791	25128	47.709	642	8375	15.901	176	2419	4.5928	9.8269	9.6267	27.4143	28.8836
DP	MGA_2	cluster_1	SP8	4554	7226.503	6912	7516	177.8561	0.6302	-0.666	0.001	0.0010909	4878	83018	54.568	839	13515	8.8834	268	4554	2.9933	5.4941	5.4856	31.9428	33.6959
DN	HIF1A_2	cluster_1	SP8	6390	10141.121	9966	10322	106.1453	0.6301	-0.6662	0.001	0.001	15992	214291	80.94	964	12652	4.7788	478	6390	2.4136	2.989	2.9819	49.5851	50.5058
DP	ETV6_1	cluster_1	SP8	13864	22003.906	21601	22406	243.274	0.6301	-0.6664	0.001	0.001	26268	378682	62.769	2565	35529	5.8892	975	13864	2.2981	3.7117	3.6611	38.0117	39.0216
DN	HBP1_1	cluster_1	SP8	5039	8000.248	7761	8238	136.7184	0.6299	-0.6668	0.001	0.0010435	5153	79302	73.662	791	11380	10.571	341	5039	4.6806	6.6175	6.3542	43.11	44.2794
DN	ARID3A_2	cluster_1	SP8	1820	2890.65	2772	3001	72.5602	0.6297	-0.6672	0.001	0.0013333	1580	25566	81.304	256	3819	12.145	116	1820	5.7879	7.3418	7.1188	45.3125	47.6565
DP	TBX20_5	cluster_4	SP4	13378	21244.379	20779	21664	274.7098	0.6297	-0.6672	0.001	0.0010909	11935	182788	54.157	2754	40150	11.896	880	13378	3.9637	7.3733	7.3189	31.9535	33.32
DN	HESX1_2	cluster_4	SP4	1190	1890.467	1817	1974	49.9718	0.6297	-0.6673	0.001	0.0014118	1143	16019	80.489	192	2490	12.511	87	1190	5.9793	7.6115	7.4287	45.3125	47.7912
DN	NFKB_known10	cluster_1	SP8	9198	14609.315	14355	14829	144.6033	0.6296	-0.6674	0.001	0.001	12896	155666	77.254	1617	19222	9.5395	766	9198	4.5648	5.9398	5.9088	47.3717	47.8514
DP	NFKB_known10	cluster_3	DN	9935	15785.104	15496	16085	177.6157	0.6294	-0.6679	0.001	0.001	10029	121099	60.099	2275	26672	13.237	829	9935	4.9305	8.266	8.204	36.4396	37.2488
DP	POU2F2_known13	cluster_3	DN	4307	6844.268	6496	7147	191.8377	0.6293	-0.6681	0.001	0.0010909	2228	46947	52.881	702	13503	15.21	210	4307	4.8514	9.4255	9.1742	29.9145	31.8966
DN	ATF3_known4	cluster_1	SP8	5648	8975.865	8797	9154	107.7816	0.6293	-0.6682	0.001	0.001	10011	141182	80.298	825	11313	6.4343	403	5648	3.2123	4.0256	4.0005	48.8485	49.9249
DN	E2F_known26	cluster_1	SP8	1858	2956.941	2839	3090	78.8542	0.6285	-0.6701	0.001	0.0010435	2046	34813	80.496	241	3860	8.9253	111	1858	4.2962	5.4252	5.3371	46.0581	48.1347
DN	PBX1_2	cluster_1	SP8	3975	6325.727	6145	6501	107.705	0.6284	-0.6701	0.001	0.0010435	3613	51272	73.03	666	8974	12.782	285	3975	5.6618	7.8882	7.7528	42.7928	44.2946
SP8	CTCF_known2	cluster_4	SP4	6742	10728.022	10251	11184	279.1674	0.6285	-0.6701	0.001	0.0010435	15803	254255	30.429	2287	35597	4.2602	422	6742	0.80688	2.6704	2.6517	18.4521	18.9398
DP	SOX8_2	cluster_4	SP4	6034	9602.388	9331	9922	174.1376	0.6284	-0.6702	0.001	0.0010909	4342	65204	50.995	1344	19312	15.104	413	6034	4.7191	9.5117	9.254	30.7292	31.2448
DP	TBX15_1	cluster_1	SP8	5896	9384.13	9035	9734	213.1594	0.6283	-0.6704	0.001	0.0010909	5512	99328	52.536	1054	18196	9.6241	328	5896	3.1185	5.9507	5.9359	31.1195	32.4027
DP	FOXP3_1	cluster_1	SP8	6847	10902.952	10582	11260	207.1892	0.628	-0.6711	0.001	0.0010909	7025	112814	50.937	1425	21824	9.8538	431	6847	3.0915	6.1352	6.0693	30.2456	31.3737
DN	HIF1A_1	cluster_1	SP8	4795	7638.054	7490	7786	93.2125	0.6278	-0.6716	0.001	0.0010435	13599	153110	79.913	879	9649	5.0361	429	4795	2.5026	3.1546	3.1317	48.8055	49.6943
DP	RBPJ_2	cluster_1	SP8	4742	7554.005	7314	7792	144.0055	0.6278	-0.6716	0.001	0.0010909	9585	95962	53.327	1475	14370	7.9855	478	4742	2.6352	4.987	4.9415	32.4068	32.9993
DP	SOX15_2	cluster_4	SP4	3169	5052.104	4813	5272	142.0998	0.6273	-0.6727	0.001	0.0010909	2513	35262	49.216	809	10650	14.865	230	3169	4.4231	9.1524	8.987	28.4302	29.7559
DP	POU2F2_known14	cluster_4	SP4	731	1165.973	1057	1280	66.4306	0.6273	-0.6729	0.001	0.0012632	685	10526	46.78	195	2647	11.764	50	731	3.2487	7.2993	6.9447	25.641	27.6162
DP	SOX7_4	cluster_4	SP4	3514	5604.193	5340	5895	166.9637	0.6271	-0.6732	0.001	0.0010909	2231	35768	47.798	802	12131	16.211	224	3514	4.6959	10.0403	9.8244	27.9302	28.9671
DP	E2F7_1	cluster_3	DN	5205	8301.445	8096	8527	132.6049	0.627	-0.6734	0.001	0.0010435	4150	54006	59.789	1136	14187	15.706	402	5205	5.7623	9.6867	9.6378	35.3873	36.6885
DP	NFATC2_1	cluster_3	DN	3308	5277.741	5136	5428	84.6656	0.6269	-0.6738	0.001	0.0010909	5104	30624	54.868	1665	9740	17.451	555	3308	5.9268	10.8738	10.802	33.3333	33.963
DN	CTCF_known1	cluster_2	DP	29410	46940.925	45908	47790	570.1444	0.6265	-0.6745	0.001	0.0010435	26314	479096	27.636	9691	172310	9.9396	1631	29410	1.6965	6.1982	6.1386	16.83	17.0681
DN	SOX3_3	cluster_1	SP8	3679	5873.388	5648	6077	128.7712	0.6264	-0.6747	0.001	0.0010909	3414	54700	70.442	591	8743	11.259	235	3679	4.7378	6.8834	6.7258	39.7631	42.0794
DP	FOXM1_1	cluster_4	SP4	2511	4011.437	3875	4146	80.6987	0.6261	-0.6756	0.001	0.0010909	4728	37858	53.335	992	7674	10.811	316	2511	3.5376	6.6836	6.6327	31.8548	32.7209
DN	ETV6_2	cluster_4	SP4	6469	10338.585	9972	10695	211.6496	0.6258	-0.6763	0.001	0.0011429	10518	94973	25.655	4569	40741	11.006	721	6469	1.7475	6.8549	6.8114	15.7803	15.8784
DP	SMAD_1	cluster_1	SP8	6342	10141.278	9937	10384	133.7841	0.6254	-0.6771	0.001	0.0010435	15462	123736	56.699	2290	18078	8.2838	794	6342	2.9061	5.1352	5.1254	34.6725	35.0813
DP	FOXO6_1	cluster_1	SP8	2115	3382.568	3210	3558	108.6011	0.6254	-0.6772	0.001	0.0010909	2247	29326	51.149	544	6763	11.796	165	2115	3.6889	7.3431	7.212	30.3309	31.2731
DP	MYBL2_4	cluster_1	SP8	952	1523.133	1417	1636	68.6214	0.6253	-0.6774	0.001	0.0010435	1563	21943	61.958	186	2524	7.1267	68	952	2.6881	4.3506	4.3385	36.5591	37.7179
DP	RBPJ_1	cluster_1	SP8	3470	5549.922	5376	5711	106.1136	0.6253	-0.6774	0.001	0.0010435	10434	73042	50.795	1618	11061	7.692	500	3470	2.4131	4.792	4.7507	30.9023	31.3715
DP	BATF_known1	cluster_1	SP8	2636	4216.824	4041	4415	117.0838	0.6252	-0.6776	0.001	0.0010435	3507	45701	52.784	638	8084	9.3369	205	2636	3.0445	5.8455	5.7679	32.1317	32.6076
DP	ETV1_1	cluster_4	SP4	2214	3542.345	3335	3739	122.2263	0.6251	-0.6778	0.001	0.0010909	5186	46934	26.831	1491	13333	7.6221	246	2214	1.2657	4.7435	4.7173	16.499	16.6054

DN	ELK4_2	cluster_4	SP4	4673	7477.813	7200	7802	182.1984	0.625	-0.6782	0.001	0.0011429	7819	70678	26.176	3236	28879	10.695	522	4673	1.7307	6.676	6.6117	16.131	16.1813
DN	ETS_known17	cluster_1	SP8	8718	13959.129	13649	14306	187.4391	0.6246	-0.6791	0.001	0.001	15240	260380	73.574	1185	19310	5.4563	518	8718	2.4634	3.399	3.3482	43.7131	45.1476
DN	MYCN_2	cluster_1	SP8	5997	9603.329	9456	9768	97.3633	0.6245	-0.6792	0.001	0.001	21157	191106	79.065	1383	12270	5.0764	668	5997	2.4811	3.1573	3.138	48.3008	48.8753
DP	BATF_known1	cluster_3	DN	4480	7174.844	6930	7439	153.6835	0.6245	-0.6793	0.001	0.0010435	3507	45701	52.784	1070	13651	15.767	346	4480	5.1743	9.866	9.8028	32.3364	32.8181
DP	RUNX1_4	cluster_4	SP4	2792	4472.466	4303	4629	103.6516	0.6243	-0.6796	0.001	0.0010909	2351	23810	47.054	998	9723	19.215	276	2792	5.5177	11.7397	11.7262	27.6553	28.7154
DP	TBX20_2	cluster_1	SP8	5307	8501.336	8139	8837	205.8416	0.6243	-0.6797	0.001	0.0010435	5116	92297	53.087	942	16294	9.3719	297	5307	3.0524	5.8053	5.7499	31.5287	32.5703
DN	SPZ1_1	cluster_1	SP8	32684	52359.33	51893	52850	297.9355	0.6242	-0.6798	0.001	0.001	58732	857887	77.786	4890	68733	6.2321	2274	32684	2.9635	3.8718	3.8098	46.5031	47.5521
DN	HIF1A::ARNT_1	cluster_1	SP8	2749	4405.045	4318	4487	50.8637	0.6241	-0.6801	0.001	0.001	12592	90566	80.589	775	5516	4.9083	385	2749	2.4462	3.0575	3.0354	49.6774	49.8368
DN	ETV3_1	cluster_4	SP4	2805	4496.263	4295	4711	128.8748	0.6239	-0.6805	0.001	0.0010435	5350	48305	25.577	1986	17704	9.374	312	2805	1.4852	5.8318	5.8069	15.71	15.8439
DN	ATF3_known5	cluster_1	SP8	10999	17642.523	17331	17929	178.8692	0.6235	-0.6816	0.001	0.001	14705	206515	71.894	1826	24906	8.6706	787	10999	3.8291	5.3519	5.326	43.0997	44.162
DP	SOX5_3	cluster_4	SP4	2983	4785.509	4571	4988	129.7026	0.6234	-0.6817	0.001	0.0011429	2038	30641	52.026	690	9567	16.244	205	2983	5.0649	10.0589	9.7353	29.7101	31.1801
DP	SMAD4_1	cluster_3	DN	33755	54158.356	53476	54824	421.8332	0.6233	-0.6821	0.001	0.001	25085	356038	57.788	6894	94795	15.386	2400	33755	5.4787	9.5675	9.4807	34.8129	35.6084
DP	TCF7L1_2	cluster_4	SP4	12336	19802.445	19387	20185	242.2054	0.623	-0.6828	0.001	0.0010435	10535	116448	51.095	3643	39382	17.28	1117	12336	5.4128	10.6028	10.5936	30.6615	31.324
DN	KLF12_1	cluster_1	SP8	6859	11026.428	10883	11181	87.321	0.6221	-0.6848	0.001	0.001	24570	147420	74.766	2507	14856	7.5344	1148	6859	3.4786	4.6724	4.6527	45.7918	46.1699
DN	SP1_known3	cluster_1	SP8	24749	39828.401	39461	40219	233.9419	0.6214	-0.6864	0.001	0.001	47992	629173	78.933	4032	51385	6.4465	1908	24749	3.1049	3.9757	3.9336	47.3214	48.1639
DP	ATF4_3	cluster_1	SP8	2716	4372.583	4167	4581	126.1745	0.6212	-0.6868	0.001	0.0010909	3413	44407	51.027	688	8653	9.943	209	2716	3.1209	6.1236	6.1162	30.3779	31.388
DP	ELK3_2	cluster_3	DN	3310	5331.461	5073	5584	152.8206	0.6209	-0.6875	0.001	0.0011429	6179	55790	26.533	2266	20219	9.6161	368	3310	1.5742	5.9557	5.933	16.2401	16.3707
DP	TBX21_6	cluster_4	SP4	9730	15673.681	15235	16107	272.945	0.6208	-0.6878	0.001	0.0010909	7546	136200	53.52	1741	29870	11.737	541	9730	3.8234	7.1694	7.1439	31.0741	32.5745
DP	ELK3_1	cluster_3	DN	3409	5492.825	5280	5724	139.5943	0.6207	-0.688	0.001	0.0011429	6415	57946	26.824	2319	20674	9.5703	379	3409	1.5781	5.908	5.8831	16.3433	16.4893
DP	ATF3_known5	cluster_3	DN	14111	22746.776	22300	23266	288.9192	0.6204	-0.6888	0.001	0.001	11264	158203	55.075	3073	41829	14.562	1013	14111	4.9125	8.9933	8.9196	32.9645	33.735
DP	SOX7_3	cluster_4	SP4	4013	6469.788	6193	6734	168.0387	0.6203	-0.6889	0.001	0.0010909	2800	44824	49.319	905	13558	14.918	256	4013	4.4154	9.1429	8.9528	28.2873	29.5988
DP	TCF21_1	cluster_1	SP8	5067	8168.89	7925	8437	158.1508	0.6203	-0.6889	0.001	0.001	11430	148923	52.886	1237	15681	5.5687	390	5067	1.7994	3.4121	3.4024	31.5279	32.313
DP	RUNX1_4	cluster_1	SP8	1408	2270.491	2134	2410	83.0298	0.6203	-0.689	0.001	0.0010909	2351	23810	47.054	512	4957	9.7962	142	1408	2.7826	6.04	5.9135	27.7344	28.4043
DP	SOX3_1	cluster_4	SP4	2599	4191.469	3975	4438	141.3633	0.6202	-0.6893	0.001	0.0010909	1698	27182	49.21	600	8890	16.094	168	2599	4.7052	9.894	9.5615	28	29.2351
DP	ELK4_1	cluster_1	SP8	2542	4105.761	3986	4212	66.6597	0.6192	-0.6915	0.001	0.001	10661	85897	77.625	674	5337	4.8231	317	2542	2.2972	2.9735	2.9594	47.0326	47.6298
DP	RBPJ_2	cluster_3	DN	11007	17794.89	17474	18146	196.2481	0.6186	-0.693	0.001	0.0010909	9585	95962	53.327	3446	33705	18.73	1109	11007	6.1167	11.5702	11.4702	32.1822	32.6569
DP	HES7_1	cluster_3	DN	10513	16998.287	16726	17266	175.3523	0.6185	-0.6932	0.001	0.0010435	9992	110416	62.326	2533	27443	15.491	957	10513	5.9342	9.5777	9.5213	37.7813	38.3085
DP	FOXO4_2	cluster_1	SP8	4947	8000.563	7754	8227	141.067	0.6184	-0.6934	0.001	0.0010435	5614	73565	57.489	1152	14358	11.22	386	4947	3.866	6.8757	6.7247	33.5069	34.4547
DP	FOXO3_1	cluster_4	SP4	5442	8801.438	8531	9048	160.1914	0.6184	-0.6935	0.001	0.0010435	4685	61564	56.333	1311	16241	14.861	427	5442	4.9796	9.1142	8.8396	32.5706	33.5078
DP	ETV6_2	cluster_3	DN	6942	11238.782	10921	11592	211.1361	0.6177	-0.695	0.001	0.0011429	9680	87477	23.63	5363	47824	12.919	771	6942	1.8753	7.9649	7.9358	14.3763	14.5157
DP	HES5_1	cluster_3	DN	10395	16838.278	16480	17144	199.584	0.6174	-0.6958	0.001	0.0010435	9609	106096	60.849	2570	27859	15.978	946	10395	5.9618	9.8449	9.7977	36.8093	37.3129
DP	IKZF2_3	cluster_1	SP8	2868	4647.314	4478	4834	110.3116	0.6172	-0.6962	0.001	0.0010909	4868	48814	55.261	904	8622	9.7608	290	2868	3.2468	5.9573	5.8754	32.0796	33.2637
DP	IKZF3_1	cluster_3	DN	7926	12848.821	12568	13145	176.3137	0.6169	-0.6969	0.001	0.0010435	6241	75044	55.627	2031	23575	17.475	666	7926	5.8752	10.6714	10.5618	32.7917	33.6204
DN	AHR::ARNT::HIF1A_1	cluster_1	SP8	3123	5065.601	4973	5167	58.5168	0.6166	-0.6976	0.001	0.0010435	14502	119460	81.912	761	6233	4.2739	378	3123	2.1414	2.6065	2.6143	49.6715	50.1043
DP	CBX5_1	cluster_4	SP4	3468	5626.071	5410	5840	129.2569	0.6165	-0.6979	0.001	0.0010435	3561	39214	51.585	1068	11228	14.77	323	3468	4.5621	9.0705	8.8438	30.2434	30.8871
DP	LMO2_2	cluster_1	SP8	1384	2246.373	2132	2359	70.6089	0.6163	-0.6984	0.001	0.0010435	3407	27268	53.534	541	4238	8.3202	174	1384	2.7171	5.1071	5.0755	32.1627	32.6569
DP	TBX1_2	cluster_1	SP8	4900	7964.754	7664	8253	186.4885	0.6153	-0.7007	0.001	0.0010909	4390	79089	50.708	933	16006	10.262	273	4900	3.1417	6.2187	6.1956	29.2605	30.6135
DN	SP1_known2	cluster_1	SP8	59653	96970.789	96431	97514	339.5166	0.6152	-0.7009	0.001	0.001	131181	1808239	79.788	9541	124977	5.5146	4461	59653	2.6322	3.4006	3.299	46.7561	47.7312
DP	SPDEF_4	cluster_1	SP8	5476	8909.56	8576	9232	191.6648	0.6147	-0.7021	0.001	0.0010435	6274	94414	52.37	1228	17521	9.7187	370	5476	3.0375	5.8974	5.8	30.1303	31.2539
DP	ID4_1	cluster_1	SP8	8431	13721.146	13421	14053	191.6444	0.6145	-0.7026	0.001	0.0010435	35081	317583	54.366	2880	25444	4.3557	937	8431	1.4433	2.671	2.6547	32.5347	33.1355
DP	ETV5_1	cluster_1	SP8	2956	4813	4701	4940	72.8308	0.6143	-0.7031	0.001	0.0010435	12055	109704	76.51	716	6355	4.4321	328	2956	2.0616	2.7209	2.6945	45.8101	46.5146
DP	FOXP1_1	cluster_4	SP4	15773	25692.869	24984	26359	409.6924	0.6139	-0.7039	0.001	0.0010435	8153	191364	49.307	3085	58974	15.195	745	15773	4.0641	9.1377	8.2424	24.1491	26.7457
DP	TCF4_1	cluster_4	SP4	8955	14587.626	14270	14905	189.9499	0.6139	-0.7039	0.001	0.0010909	39369	356288	54.334	3083	27113	4.1348	997	8955	1.3656	2.5324	2.5134	32.3386	33.0284
DP	SOX21_3	cluster_4	SP4	3968	6467.205	6229	6760	159.2467	0.6136	-0.7046	0.001	0.0010909	3002	45066	51.174	903	13016	14.78	267	3968	4.5058	8.8941	8.8049	29.5681	30.4856
DN	SP8_1	cluster_1	SP8	11818	19267.949	18641	19833	357.872	0.6134	-0.7052	0.001	0.001	21179	262744	21.027	8014	95072	7.6085	966	11818	0.94578	4.5611	4.4979	12.0539	12.4306
DP	MAF_known10	cluster_4	SP4	6267	10225.677	9955	10496	161.0037	0.6129	-0.7063	0.001	0.0010909	7909	87167	53.303	1819	19475	11.909	572	6267	3.8323	7.2323	7.1896	31.4458	32.1797
DP	PKNOX1_1	cluster_1	SP8	7719	12601.27	12236	12979	232.1322	0.6126	-0.707	0.001	0.0010435	10906	164350	52.965	1682	24195	7.7972	515	7719	2.4876	4.7222	4.6967	30.6183	31.9033
DP	TBX20_5	cluster_1	SP8	10850	17711.802	17284	18159	271.6397	0.6126	-0.707	0.001	0.0010909	11935	182788	54.157	2275	33402	9.8965	717	10850	3.2147	6.0075	5.9358	31.5165	32.4831
DN	RUNX_1	cluster_4	SP4	21984	35890.429	35494	36269	244.7515	0.6125	-0.7071	0.001	0.0010435	34752	490327	76.602	3468	47492	7.4195	1573	21984	3.4345	4.5264	4.4835	45.3576	46.2899
DN	KLF12_2	cluster_1	SP8	32322	52769.772	52311	53343	292.6254	0.6125	-0.7072	0.001	0.001	46999	672450	77.85	5003	69033	7.992	2291	32322	3.7419	4.8746	4.8066	45.7925	46.8211
DP	HMGA1_1	cluster_4	SP4	1578	2576.861	2475	2687	63.6182	0.6125	-0.7072	0.001	0.0010435	3004	18138	53.492	821	4883	14.401	263	1578	4.6538	8.755	8.7	32.0341	32.3162
DP	SOX3_3	cluster_4	SP4	3309	5405.448	5175	5640	153.7574	0.6122	-0.7078	0.001	0.0010909	2264	36279	46.72	791	11919	15.349	211	3309	4.2613	9.3198	9.121	26.6751	27.7624
DP	POU2F2_known15	cluster_4	SP4	2606	4258.918	4062	4460	116.7628	0.612	-0.7084	0.001	0.0010435	2667	29451	51.273	800	8497	14.793	240	2606	4.5369	8.9989	8.8486	30	30.6696
DP	ID4_1	cluster_4	SP4	9347	15276.688	14987	15599	188.0931	0.6119	-0.7087	0.001	0.0010435	35081	317583	54.366	3214	28330	4.8498	1038	9347	1.6001	2.9589	2.9432	32.2962	32.9933
DN	E2F2_3	cluster_1	SP8	1895	3099.004	2972	3198	73.5883	0.6116	-0.7093	0.001	0.001	2541	43236	81.281	247	3972	7.4671	113	1895	3.5625	4.4471	4.3829	45.749	47.709
DN	MYCN_1	cluster_1	SP8	4980	8145.242	7972	8311	101.4093	0.6114	-0.7097	0.001	0.001	12503	137921	76.685	997	10742	5.9726	453	4980	2.7689	3.6231	3.6108	45.4363	46.3601
DP	FOXO3_4	cluster_1	SP8	1588	2598.445	2457	2749	88.2961	0.6113	-0.7101	0.001	0.0010909	1629	21240	50.833	419	5251	12.567	123	1588	3.8005	7.5506	7.4765	29.3556	30.2419
DP	SP100_1	cluster_4	SP4	546	893.905	815	973	46.9	0.6112	-0.7102	0.001	0.0013333	864	11233	66.811	115	1406	8.3626	42	546	3.2475	4.8611	4.8607	36.5217	38.8336
DN	RREB1_2	cluster_1	SP8	26095	42701.035	41411	43981	753.9518	0.6111	-0.7105	0.001	0.001	18681	474233	23.829	8721	197558	9.9268	1105	26095	1.3112	5.9151	5.5026	12.6706	13.2088
DP	ETV3_1	cluster_4	SP4	7185	11759.082	11509	11973	144.5024	0.6111	-0.7106	0.001	0.0010435	13947	126127	66.782	1986	17704	9.374	798	7185	3.8043	5.7217	5.6966	40.1813	40.584
DP	FOXM1_1	cluster_1	SP8	2106	3448.083	3324	3591	80.2926	0.6109	-0.711	0.001	0.0010909	4728	37858	53.335	835	6541	9.2151	262	2106	2.967	5.5415	5.5629	31.3772	32.1969
DP	TBX4_1	cluster_1	SP8	4524	7411.447	7213	7588	107.4682	0.6105	-0.712	0.001	0.0010435	11484	80419	53.597	2026	13954	9.2999	649	4524	3.0151	5.6513	5.6255	32.0336	32.4208
DP	ATF4_2	cluster_1	SP8	2320	3803.321	3627	3988	110.1212	0.6101	-0.7129	0.001	0.0010909	3457	41526	51.125	650	7549	9.2939	195	2320	2.8563	5.6407	5.5869	30	30.7325
DP	RUNX1_2	cluster_4	SP4	5600	9180.464	8882	9475	190.9779	0.61	-0.713	0.001	0.0010435	3573	55648	49.329	1297	19205	17.024	353	5600	4.9641	9.8797	10.0633	27.2167	29.1591
DP	TCF4_1	cluster_1	SP8	8559	14036.225	13757	14312	180.1923	0.6098	-0.7136	0.001	0.0010909	39369	356288	54.334	2953	26049	3.9725	951	8559	1.3053	2.4156	2.4023	32.2045	32.8573
DP	SOX21_2	cluster_1	SP8	3026	4964.94	4739	5197	136.355	0.6096	-0.7142	0.001	0.0010909	3145	44041	49.589	767	10297	11.594	219	3026	3.4072	6.9634	6.8709	28.5528	29.3872
DP	CBX5_1	cluster_1	SP8	2659	4369.285	4189	4558	116.0705	0.6087	-0.7163	0.001	0.0010435	3561	39214	51.585	838	8765	11.53	247	2659	3.4979	6.9363	6.7807	29.4749	30.3366
DP	FOXO1_5	cluster_1	SP8	14803	24322.172	23897	24726	242.3085	0.6086	-0.7163	0.001	0.0010435	23967	264888	55.536	4092	44229	9.2729	1345	14803	3.1035	5.6119	5.5884	32.869	33.469
DP	MTF1_1	cluster_3	DN	44236	72692.741	72038	73317	403.0091	0.6085	-0.7166	0.001	0.001	40601	541813	68.67	8195	106903	13.549	3337	44236	5.6065	8.219	8.1644	40.72	41.3796
DP	ATF4_2	cluster_3	DN	4225	6945.18	6697	7193	149.8557	0.6084	-0.7169	0.001	0.0010909	3457	41526	51.125	1173	13733	16.907	355	4225	5.2016	10.269	10.1743	30.2643	30.7653
DP	E2F_known24	cluster_3	DN	3228	5310.755	5078	5517	132.8786	0.6079	-0.7181	0.001	0.001	2757	47016	60.976	582	9023	11.702	194	3228	4.1864	7.0366	6.8657	33.3333	35.7752
DP	IKZF1_2	cluster_1	SP8	7690	12662.081	12425	12923	142.5207	0.6074	-0.7194	0.001	0.0010435	20144	161159	58.804	2799	21823	7.9629	967	7690	2.806	4.8004	4.7717	34.5481	35.2381
DP	MYBL1_5	cluster_3	DN	4046	6664.68	6424	6877	136.8255	0.6071	-0.7199	0.001	0.0010435	2741	44252	63.347	683	10712	15.334	254	4046	5.7918	9.2667	9.1431	37.1889	37.7707
DP	LMO2_2	cluster_3	DN	2613	4306.241	4148	4466	92.0874	0.6069	-0.7205	0.001	0.0010435	3407	27268	53.534	1039	8150	16	328	2613	5.13	9.6272	9.5827	31.5688	32.0613
DP	SOX8_2	cluster_1	SP8	4231	6972.133	6683	7250	170.4335	0.6069	-0.7205	0.001	0.0010909	4342	65204	50.995	975	13966	10.923	285	4231	3.309	6.5638	6.4889	29.2308	30.295
DN	NFKB_known11	cluster_1	SP8	10683	17607.366	17318	17878	167.0258	0.6068	-0.7208	0.001	0.001	14339	173964	75.858	1970	23567	10.277	885	10683	4.6584	6.172	6.1409	44.9239	45.3303
DP	FOXP3_1	cluster_4	SP4	8689	14325.2	13915	14715	241.0792	0.6066	-0.7212	0.001	0.0010909	7025	112814	50.937	1889	28677	12.948	548	8689	3.9232	7.8007	7.7021	29.0101	30.2995
SP8	CUX2_1	cluster_2	DP	368	607.407	535	665	38.5504	0.6065	-0.7214	0.001	0.0012632	434	7992	75.035	51	888	8.3372	22	368	3.4551	5.0691	4.6046	43.1373	41.4414
DP	RBPJ_1	cluster_4	SP4	3724	6141.329	5960	6348	114.3122	0.6064	-0.7215	0.001	0.0010435	10434	73042	50.795	1817	12285	8.5432	538	3724	2.5897	5.1562	5.0984	29.6092	30.3134
DN	CUX1_8	cluster_1	SP8	794	1310.027	1226	1392	49.1938	0.6064	-0.7217	0.001	0.0011429	619	9964	75.019	122	1836	13.823	52	794	5.978	8.4006	7.9687	42.623	43.2462
DP	TBX2_2	cluster_1	SP8	5106	8422.218	8183	8636	137.2473	0.6063	-0.7219	0.001	0.0010909	8541	85538	53.696	1622	15897	9.9792	511	5106	3.2053	5.9829	5.9693	31.5043	32.1193
DN	ETV1_1	cluster_4	SP4	2114	3487.877	3293	3699	126.5655	0.6062	-0.7221	0.001	0.0010909	5090	46038	26.319	1491	13333	7.6221	235	2114	1.2085	4.6169	4.5919	15.7612	15.8554
DP	FOXO6_3	cluster_1	SP8	14733	24304.927	23876	24802	274.7771	0.6062	-0.7222	0.001	0.0010435	21070	275513	56.268	3425	43712	8.9273	1131	14733	3.0089	5.3678	5.3475	33.0219	33.7047
DP	FOXO3_1	cluster_1	SP8	4417	7288.993	7055	7536	153.1725	0.606	-0.7225	0.001	0.0010435	4685	61564	56.333	1083	13447	12.304	344	4417	4.0417	7.3426	7.1746	31.7636	32.8475
DN	SP4_2	cluster_1	SP8	42852	70767.937	70202	71347	346.7815	0.6055	-0.7237	0.001	0.001	66957	1120770	78.67	5789	92205	6.4721	2623	42852	3.0079	3.9174	3.8234	45.3101	46.4747
DP	ATF4_3	cluster_4	SP4	3512	5803.043	5574	6041	138.5593	0.6053	-0.7244	0.001	0.0010909	3413	44407	51.027	930	11616	13.348	271	3512	4.0356	7.9402	7.9087	29.1398	30.2342
DP	PKNOX2_3	cluster_4	SP4	7167	11856.286	11546	12187	191.5205	0.6045	-0.7261	0.001	0.0010435	9600	106895	49.891	2218	23981	11.193	643	7167	3.345	6.6979	6.7047	28.9901	29.8862
DN	MYC_known13	cluster_1	SP8	5501	9104.564	8919	9275	108.1691	0.6042	-0.7268	0.001	0.0010909	12241	134783	75.676	1134	12181	6.8392	504	5501	3.0886	4.1173	4.0814	44.4444	45.1605
DP	ATF7_1	cluster_1	SP8	2320	3840.24	3669	4015	109.6187	0.6042	-0.7268	0.001	0.001	3062	39860	54.566	561	7117	9.7428	180	2320	3.176	5.8785	5.8204	32.0856	32.598
DP	SOX10_4	cluster_1	SP8	4632	7666.685	7379	7947	173.7069	0.6042	-0.7268	0.001	0.0010909	4770	71762	51.378	1057	15221	10.898	312	4632	3.3163	6.5409	6.4547	29.5175	30.4316
DP	MAF_known12	cluster_1	SP8	6085	10076.571	9698	10425	219.6182	0.6039	-0.7276	0.001	0.0010435	7384	125901	57.08	1102	17974	8.1489	360	6085	2.7588	4.8754	4.8332	32.6679	33.8545
DP	MESP1_1	cluster_4	SP4	8289	13726.641	13430	14044	179.3514	0.6039	-0.7276	0.001	0.001	32605	294479	56.054	2809	24683	4.6984	922	8289	1.5778	2.8278	2.8148	32.8231	33.5818
DN	KLF14_1	cluster_1	SP8	8263	13684.425	13164	14183	300.9371	0.6039	-0.7277	0.001	0.001	16591	224164	22.673	4810	62544	6.326	628	8263	0.83576	3.7852	3.6861	13.0561	13.2115
DP	TCF4_2	cluster_4	SP4	9472	15690.093	15347	16008	198.914	0.6037	-0.7281	0.001	0.001	32705	295539	54.979	3276	28814	5.3602	1056	9472	1.7621	3.2289	3.205	32.2344	32.8729
DP	PKNOX2_2	cluster_4	SP4	7540	12492.879	12191	12791	179.359	0.6036	-0.7284	0.001	0.0010435	11043	122680	50.42	2309	24959	10.258	678	7540	3.0989	6.1396	6.1461	29.3634	30.2095
DN	EGR3_2	cluster_1	SP8	6741	11171.085	10703	11673	278.0023	0.6035	-0.7286	0.001	0.001	13370	205199	20.858	3895	56407	5.7337	445	6741	0.68521	3.3283	3.2851	11.4249	11.9506
DN	RUNX_2	cluster_4	SP4	17009	28184.732	27813	28562	221.7693	0.6035	-0.7286	0.001	0.0010435	28180	397366	76.459	2748	37394	7.1951	1217	17009	3.2728	4.3187	4.2804	44.2868	45.4859
DP	IKZF2_3	cluster_4	SP4	3829	6355.296	6153	6553	122.3727	0.6026	-0.7308	0.001	0.0010909	4868	48814	55.261	1246	11893	13.464	388	3829	4.3347	7.9704	7.8441	31.1396	32.1954
DP	POU2F2_known15	cluster_1	SP8	1901	3159.013	3004	3305	90.6025	0.6019	-0.7324	0.001	0.0010435	2667	29451	51.273	593	6304	10.975	175	1901	3.3095	6.5617	6.4548	29.511	30.1555
DP	TBX1_4	cluster_1	SP8	8704	14464.68	14006	14915	264.9983	0.6018	-0.7327	0.001	0.0010909	7984	152301	52.016	1543	28290	9.6621	461	8704	2.9727	5.774	5.715	29.8769	30.7671
DP	TCF3_2	cluster_1	SP8	10214	16981.87	16505	17415	269.7202	0.6015	-0.7334	0.001	0.0010435	27591	417427	54.427	2188	31655	4.1274	680	10214	1.3318	2.4646	2.4469	31.0786	32.2666
DP	TCF4_2	cluster_1	SP8	8721	14500.059	14184	14818	190.3966	0.6015	-0.7334	0.001	0.001	32705	295539	54.979	3017	26562	4.9413	971	8721	1.6224	2.969	2.9509	32.1843	32.8326
SP4	GMEB1_1	cluster_5	Common_1_DN	10396	17286.905	16958	17594	192.5862	0.6014	-0.7336	0.001	0.0010435	5959	97141	70.771	1557	24649	17.958	644	10396	7.5739	10.8072	10.702	41.3616	42.1762
DP	NFATC2_1	cluster_4	SP4	2057	3421.654	3310	3538	70.3302	0.6013	-0.7339	0.001	0.0010909	5104	30624	54.868	1082	6319	11.322	346	2057	3.6855	6.779	6.717	31.9778	32.5526
DN	EGR1_known9	cluster_1	SP8	14942	24862.504	24548	25139	175.7061	0.601	-0.7346	0.001	0.001	32718	444077	82.227	2355	30796	5.7023	1111	14942	2.7667	3.3957	3.3647	47.1762	48.5193
DP	SP100_1	cluster_1	SP8	467	777.852	713	849	42.394	0.6009	-0.7348	0.001	0.0013333	864	11233	66.811	97	1202	7.1492	36	467	2.7776	4.1667	4.1574	37.1134	38.8519
DP	IKZF1_1	cluster_3	DN	10669	17760.688	17397	18118	214.7804	0.6007	-0.7352	0.001	0.0010435	8537	102703	58.812	2665	30767	17.618	898	10669	6.1095	10.5189	10.3882	33.6961	34.6768
DP	TBX1_2	cluster_4	SP4	5395	8985.628	8638	9319	207.4594	0.6004	-0.7359	0.001	0.0010909	4390	79089	50.708	1076	18201	11.67	305	5395	3.459	6.9476	6.8214	28.3457	29.6412
DP	TBX15_2	cluster_1	SP8	5606	9337.163	9117	9538	123.9283	0.6004	-0.7359	0.001	0.0010909	14654	102598	53.396	2561	17651	9.1862	803	5606	2.9176	5.4797	5.464	31.3549	31.7602
DP	SOX11_1	cluster_1	SP8	9823	16364.834	15973	16762	234.2736	0.6003	-0.7363	0.001	0.0010435	9836	159338	54.566	2020	30899	10.581	620	9823	3.3639	6.3034	6.1649	30.6931	31.7907
DP	ETS_known17	cluster_1	SP8	6884	11470.947	11112	11816	210.195	0.6002	-0.7366	0.001	0.001	12494	213553	60.343	1185	19310	5.4563	409	6884	1.9452	3.2736	3.2236	34.5148	35.6499
DP	MAFF_1	cluster_1	SP8	6137	10230.76	9890	10590	198.9216	0.5999	-0.7372	0.001	0.001	9294	130389	52.132	1463	19885	7.9504	438	6137	2.4537	4.7127	4.7067	29.9385	30.8625
DP	SMAD3_1	cluster_1	SP8	8479	14137.523	13833	14403	172.3013	0.5998	-0.7375	0.001	0.0010435	19225	159039	54.071	3179	26335	8.9536	1023	8479	2.8827	5.3212	5.3314	32.1799	32.1967
DP	POU2F2_known5	cluster_4	SP4	3439	5737.064	5493	5979	147.8169	0.5995	-0.7382	0.001	0.0010909	3181	41440	50.684	935	11592	14.178	271	3439	4.2061	8.5193	8.2987	28.984	29.667
DP	TBX21_6	cluster_1	SP8	8073	13468.627	13043	13850	243.318	0.5994	-0.7384	0.001	0.0010909	7546	136200	53.52	1472	25611	10.064	450	8073	3.1723	5.9634	5.9273	30.5707	31.5216
DP	RREB1_1	cluster_1	SP8	44351	74002.511	73129	74728	494.9242	0.5993	-0.7386	0.001	0.001	45950	762192	60.431	7972	126523	10.031	2695	44351	3.5164	5.8651	5.8189	33.8058	35.0537
DP	SPDEF_4	cluster_4	SP4	8998	15018.43	14630	15424	230.3856	0.5992	-0.739	0.001	0.0010435	6274	94414	52.37	2070	29469	16.346	606	8998	4.9911	9.6589	9.5304	29.2754	30.5338
DP	IRF4_1	cluster_4	SP4	2729	4556.129	4361	4740	112.1567	0.5991	-0.7392	0.001	0.0010435	2787	39153	57.73	591	8045	11.862	196	2729	4.0238	7.0327	6.9701	33.1641	33.9217
DP	IRF4_2	cluster_1	SP8	6094	10174.862	9852	10499	196.1273	0.599	-0.7395	0.001	0.001	5446	77752	53.165	1355	19304	13.2	424	6094	4.1669	7.7855	7.8377	31.2915	31.5686
DP	E2F2_2	cluster_3	DN	3493	5835.996	5584	6073	147.3653	0.5986	-0.7403	0.001	0.0010909	2804	47831	58.459	657	10343	12.641	210	3493	4.2691	7.4893	7.3028	31.9635	33.7716
DP	SMAD_2	cluster_1	SP8	11159	18646.085	18310	19027	211.6584	0.5985	-0.7406	0.001	0.0010435	20655	212873	53.488	3462	35177	8.8389	1082	11159	2.8039	5.2384	5.2421	31.2536	31.7224
DP	TBX2_2	cluster_4	SP4	5866	9802.421	9560	10082	157.4222	0.5985	-0.7407	0.001	0.0010909	8541	85538	53.696	1911	18576	11.661	592	5866	3.6823	6.9313	6.8578	30.9785	31.5784
DP	MAF_known1	cluster_4	SP4	22396	37449.68	36830	38130	384.0752	0.598	-0.7417	0.001	0.001	24308	367004	53.477	4884	71057	10.354	1487	22396	3.2634	6.1173	6.1024	30.4464	31.5184
DP	POU2F2_known10	cluster_1	SP8	2310	3863.413	3706	4018	99.1889	0.598	-0.7417	0.001	0.0010435	4216	38032	52.25	854	7508	10.315	260	2310	3.1736	6.167	6.0738	30.445	30.7672
DP	HES5_1	cluster_1	SP8	3341	5588.738	5390	5787	118.0705	0.5979	-0.7421	0.001	0.0010435	9609	106096	60.849	867	9291	5.3287	306	3341	1.9162	3.1845	3.149	35.2941	35.9595

DN	ETV4_2	cluster_4	SP4	2051	3433.911	3232	3615	118.2809	0.5974	-0.7432	0.001	0.0011429	5010	45275	26.285	1473	13170	7.646	228	2051	1.1907	4.5509	4.5301	15.4786	15.5733
DP	MYC_known20	cluster_3	DN	13067	21873.338	21412	22315	274.9474	0.5974	-0.7432	0.001	0.001	8735	143000	56.723	2458	39039	15.485	805	13067	5.1832	9.2158	9.1378	32.7502	33.4717
DP	IRF4_1	cluster_3	DN	3323	5564.377	5356	5813	136.6497	0.5973	-0.7436	0.001	0.0010435	2787	39153	57.73	712	9749	14.375	239	3323	4.8997	8.5755	8.4872	33.5674	34.0855
DP	SOX10_9	cluster_1	SP8	2609	4371.563	4152	4604	133.3796	0.5969	-0.7444	0.001	0.0010435	2814	39418	46.89	721	9597	11.416	189	2609	3.1036	6.7164	6.6188	26.2136	27.1856
DP	ETV4_2	cluster_3	DN	2383	3993.548	3800	4188	121.9023	0.5968	-0.7446	0.001	0.0011429	5095	46084	26.755	1685	15032	8.7271	265	2383	1.3835	5.2012	5.171	15.727	15.8528
DP	SOX13_1	cluster_4	SP4	2198	3683.483	3482	3861	115.2137	0.5968	-0.7446	0.001	0.0010909	1690	25424	51.067	555	7626	15.318	151	2198	4.4149	8.9349	8.6454	27.2072	28.8224
DP	MGA_3	cluster_1	SP8	4097	6867.769	6549	7162	180.2818	0.5966	-0.7451	0.001	0.0010909	4692	79902	56.395	760	12400	8.7519	242	4097	2.8916	5.1577	5.1275	31.8421	33.0403
DP	EWSR1::FLI1_1	cluster_1	SP8	36214	60703.787	59730	61622	578.0727	0.5966	-0.7452	0.001	0.0010435	43660	868688	53.282	6241	116963	7.1741	1838	36214	2.2212	4.2098	4.1688	29.4504	30.9619
DP	PKNOX1_2	cluster_4	SP4	6672	11193.667	10868	11531	191.932	0.5961	-0.7464	0.001	0.0010435	9569	106189	50.266	2075	22398	10.602	600	6672	3.1583	6.2702	6.2831	28.9157	29.7884
DP	SOX8_9	cluster_4	SP4	3446	5781.944	5493	6045	163.4777	0.5961	-0.7465	0.001	0.0010909	2184	34944	45.366	884	13207	17.146	219	3446	4.4738	10.0275	9.8615	24.7738	26.0922
DP	MYBL2_2	cluster_4	SP4	1628	2737.381	2578	2894	96.5252	0.5949	-0.7493	0.001	0.0010435	2149	32599	60.53	325	4617	8.5729	109	1628	3.0229	5.0721	4.994	33.5385	35.261
DN	KLF16_1	cluster_1	SP8	41098	69110.371	68548	69598	323.5073	0.5947	-0.7498	0.001	0.001	87857	980098	74.479	8719	94633	7.1912	3697	41098	3.1231	4.208	4.1933	42.4017	43.4288
DN	SP4_1	cluster_1	SP8	52377	88120.831	87380	88771	428.1731	0.5944	-0.7505	0.001	0.001	90889	1624561	78.36	6913	116187	5.6042	3018	52377	2.5264	3.3205	3.2241	43.6569	45.0799
DP	RREB1_1	cluster_4	SP4	41895	70491.062	69691	71317	479.1455	0.5943	-0.7506	0.001	0.001	45950	762192	60.431	7266	119347	9.4625	2422	41895	3.3217	5.2709	5.4966	33.3333	35.1035
DP	E2F8_1	cluster_3	DN	7321	12322.204	12021	12567	168.2703	0.5942	-0.7511	0.001	0.001	7146	78795	61.175	1924	20480	15.9	672	7321	5.6839	9.4039	9.2912	34.9272	35.7471
DN	KLF4_1	cluster_1	SP8	37891	63783.208	63305	64250	280.2514	0.5941	-0.7513	0.001	0.001	90604	870001	74.9	9288	86550	7.4513	4002	37891	3.2621	4.417	4.3553	43.0879	43.7793
DP	SOX4_2	cluster_1	SP8	3779	6362.013	6113	6621	149.8405	0.5941	-0.7513	0.001	0.0010909	3926	59025	51.393	888	12727	11.081	257	3779	3.2904	6.5461	6.4024	28.9414	29.6928
DP	TBX21_4	cluster_4	SP4	7546	12703.899	12299	13155	251.3911	0.594	-0.7514	0.001	0.0010909	7045	105923	52.17	1733	24847	12.238	508	7546	3.7166	7.2108	7.124	29.3133	30.3699
DP	SOX8_9	cluster_1	SP8	2407	4054.15	3833	4287	144.6747	0.5938	-0.7519	0.001	0.0010909	2184	34944	45.366	615	9226	11.978	154	2407	3.1249	7.0513	6.8882	25.0407	26.0893
DP	IRF_known14	cluster_4	SP4	19250	32435.488	31789	33062	382.5934	0.5935	-0.7527	0.001	0.001	11310	235509	53.453	3125	62308	14.142	940	19250	4.3692	8.3112	8.1738	30.08	30.8949
DP	SOX4_1	cluster_1	SP8	10293	17367.918	16963	17754	247.122	0.5927	-0.7547	0.001	0.0010435	10641	172327	54.726	2132	32642	10.366	649	10293	3.2688	6.0991	5.9729	30.4409	31.533
DP	MAFG_1	cluster_1	SP8	5610	9473.329	9133	9816	195.447	0.5922	-0.7558	0.001	0.0010435	5947	119184	52.184	971	18472	8.0878	283	5610	2.4563	4.7587	4.707	29.1452	30.3703
DP	MTF1_3	cluster_1	SP8	6813	11505.71	11176	11789	188.1086	0.5922	-0.7559	0.001	0.001	12915	169509	62.611	1462	18557	6.8544	522	6813	2.5165	4.0418	4.0193	35.7045	36.7139
DP	HMGA1_2	cluster_4	SP4	5624	9499.025	9242	9774	165.2194	0.5921	-0.7561	0.001	0.0010435	4747	66572	54.16	1373	18136	14.755	404	5624	4.5754	8.5106	8.448	29.4246	31.0101
DP	TBX5_3	cluster_1	SP8	6597	11142.191	10876	11440	161.2242	0.5921	-0.7561	0.001	0.001	14895	134213	54.181	2358	20762	8.3814	736	6597	2.6632	4.9413	4.9153	31.2129	31.7744
DP	SOX_1	cluster_1	SP8	4273	7221.919	6993	7488	153.5387	0.5917	-0.757	0.001	0.0010435	5830	70005	50.837	1257	14434	10.482	361	4273	3.103	6.1921	6.1038	28.7192	29.6037
DP	GMEB1_1	cluster_5	Common_1_DN	9736	16456.554	16133	16803	197.6598	0.5916	-0.7572	0.001	0.0010435	5680	92646	67.496	1557	24649	17.958	600	9736	7.0931	10.5634	10.5088	38.5356	39.4986
DP	TBX15_1	cluster_4	SP4	6551	11074.405	10661	11457	227.4853	0.5916	-0.7574	0.001	0.0010909	5512	99328	52.536	1270	21552	11.399	370	6551	3.4649	6.7126	6.5953	29.1339	30.3963
DN	ETV2_1	cluster_4	SP4	5877	9937.866	9603	10273	199.9912	0.5914	-0.7578	0.001	0.0010435	7018	70432	23.896	4238	42029	14.259	589	5877	1.9939	8.3927	8.3442	13.8981	13.9832
DP	TBX19_1	cluster_1	SP8	3596	6081.633	5794	6367	172.3565	0.5914	-0.7579	0.001	0.0010435	3308	63063	49.328	692	12512	9.7869	190	3596	2.8128	5.7437	5.7022	27.4566	28.7404
DP	BPTF_1	cluster_1	SP8	9461	16000.559	15624	16382	225.9593	0.5913	-0.758	0.001	0.0010909	10963	146228	52.127	2436	31404	11.195	723	9461	3.3726	6.5949	6.47	29.6798	30.1267
DP	SOX10_9	cluster_4	SP4	3733	6313.74	6015	6563	166.7181	0.5913	-0.758	0.001	0.0010435	2814	39418	46.89	1036	13825	16.446	270	3733	4.4406	9.5949	9.4703	26.0618	27.0018
DP	ETV1_1	cluster_3	DN	2392	4048.207	3819	4277	132.0807	0.591	-0.7588	0.001	0.0010909	5186	46934	26.831	1706	15225	8.7037	266	2392	1.3674	5.1292	5.0965	15.592	15.711
DP	TBX5_3	cluster_4	SP4	8089	13691.188	13419	13958	164.1927	0.5908	-0.7591	0.001	0.001	14895	134213	54.181	2921	25603	10.336	904	8089	3.2655	6.0692	6.027	30.9483	31.594
DP	ARID5B_1	cluster_1	SP8	3278	5551.665	5316	5806	148.1785	0.5905	-0.7599	0.001	0.0010909	4087	53311	52.552	872	10810	10.656	255	3278	3.2313	6.2393	6.1488	29.2431	30.3238
DN	EGR4_1	cluster_1	SP8	15992	27083.949	26785	27396	187.1191	0.5905	-0.7601	0.001	0.001	34373	389734	77.181	3232	35618	7.0536	1425	15992	3.167	4.1457	4.1033	44.0903	44.8986
DP	HMGA1_2	cluster_1	SP8	4339	7349.182	7102	7598	150.842	0.5905	-0.7601	0.001	0.0010435	4747	66572	54.16	1076	14034	11.417	319	4339	3.53	6.72	6.5178	29.6468	30.9178
DP	POU2F2_known12	cluster_1	SP8	2367	4011.374	3849	4187	99.4913	0.5902	-0.7608	0.001	0.0010435	3757	37632	51.558	834	7995	10.954	243	2367	3.2429	6.4679	6.2899	29.1367	29.606
DP	TCF12_known1	cluster_1	SP8	8468	14351.036	14106	14596	151.3894	0.5901	-0.761	0.001	0.0010909	36358	261819	56.235	3620	25716	5.5234	1188	8468	1.8188	3.2675	3.2343	32.8177	32.9289
DP	BPTF_1	cluster_4	SP4	10230	17343.561	16917	17705	244.8592	0.5899	-0.7615	0.001	0.0010909	10963	146228	52.127	2650	34033	12.132	784	10230	3.6468	7.1513	6.9959	29.5849	30.0591
DP	E2F2_2	cluster_1	SP8	2638	4479.678	4275	4692	124.6572	0.589	-0.7637	0.001	0.0010909	2804	47831	58.459	488	7854	9.5991	158	2638	3.2242	5.6348	5.5153	32.377	33.588
DP	RBPJ_2	cluster_4	SP4	5047	8569.719	8318	8818	147.59	0.589	-0.7637	0.001	0.0010909	9585	95962	53.327	1692	16343	9.0819	507	5047	2.8047	5.2895	5.2594	29.9645	30.8817
DP	MYBL1_3	cluster_1	SP8	693	1177.369	1083	1262	57.2132	0.5889	-0.7638	0.001	0.0010909	1344	14803	55.839	205	2158	8.1403	63	693	2.6141	4.6875	4.6815	30.7317	32.1131
DP	IRF_known21	cluster_4	SP4	7819	13278.292	12932	13612	215.2985	0.5889	-0.7639	0.001	0.001	6273	90082	51.133	1864	26418	14.995	550	7819	4.4383	8.7677	8.6799	29.5064	29.5972
DP	MESP1_1	cluster_1	SP8	8014	13610.116	13314	13875	174.4774	0.5889	-0.764	0.001	0.001	32605	294479	56.054	2782	24456	4.6552	891	8014	1.5255	2.7327	2.7214	32.0273	32.7691
DP	POU2F2_known1	cluster_1	SP8	2933	4981.642	4740	5248	150.2811	0.5888	-0.764	0.001	0.0010435	2722	49255	50.993	595	10054	10.409	164	2933	3.0365	6.025	5.9547	27.563	29.1725
DP	SOX5_1	cluster_1	SP8	1120	1902.618	1793	2015	68.0346	0.5889	-0.764	0.001	0.0010909	1887	16983	50.471	438	3829	11.379	125	1120	3.3285	6.6243	6.5948	28.5388	29.2505
DN	LMO2_1	cluster_1	SP8	6699	11378.699	11042	11728	211.2969	0.5888	-0.7642	0.001	0.001	22843	252436	30.177	3559	38297	4.5781	613	6699	0.80081	2.6835	2.6537	17.2239	17.4922
DP	SP1_known3	cluster_3	DN	38272	65001.936	64319	65658	372.6123	0.5888	-0.7642	0.001	0.001	39534	518521	65.051	8009	101310	12.71	2960	38272	4.8014	7.4872	7.381	36.9584	37.7771
DP	POU2F2_known1	cluster_4	SP4	4069	6912.955	6593	7186	175.4343	0.5887	-0.7645	0.001	0.0010435	2722	49255	50.993	825	13977	14.47	228	4069	4.2126	8.3762	8.2611	27.6364	29.1121
DP	POU2F3_1	cluster_1	SP8	2823	4796.996	4553	5013	136.3283	0.5886	-0.7647	0.001	0.0010435	3087	46662	50.134	681	9801	10.53	188	2823	3.0331	6.0901	6.0499	27.6065	28.8032
DP	NFATC1_1	cluster_4	SP4	6255	10637.497	10302	10976	215.7946	0.5881	-0.766	0.001	0.0010909	4535	87510	54.48	1121	20131	12.533	331	6255	3.8941	7.2988	7.1478	29.5272	31.0715
DN	KLF13_1	cluster_1	SP8	16540	28136.342	27683	28532	251.7812	0.5879	-0.7664	0.001	0.001	24149	415074	75.178	2316	38167	6.9128	976	16540	2.9957	4.0416	3.9848	42.1416	43.3359
DP	SOX11_1	cluster_4	SP4	14324	24367.791	23865	24857	306.7077	0.5878	-0.7665	0.001	0.0010435	9836	159338	54.566	3012	45946	15.734	904	14324	4.9053	9.1907	8.9897	30.0133	31.1757
DP	CUX2_1	cluster_3	DN	386	657.539	576	747	51.9756	0.5877	-0.7669	0.001	0.0012632	328	6022	56.539	86	1315	12.346	24	386	3.6241	7.3171	6.4098	27.907	29.3536
DP	HESX1_1	cluster_1	SP8	498	848.116	768	932	49.8635	0.5877	-0.7669	0.001	0.0012632	1124	10127	51.054	201	1706	8.6005	57	498	2.5106	5.0712	4.9175	28.3582	29.1911
DN	FOXO3_4	cluster_4	SP4	2804	4773.177	4640	4915	83.7864	0.5875	-0.7673	0.001	0.0010909	2345	30568	73.157	546	6810	16.298	220	2804	6.7107	9.3817	9.173	40.293	41.1747
DN	SPI1_known1	cluster_4	SP4	8219	13990.731	13637	14311	209.1855	0.5875	-0.7674	0.001	0.001	15535	109231	23.39	8708	60564	12.969	1177	8219	1.76	7.5764	7.5244	13.5163	13.5708
DP	FOXO4_2	cluster_4	SP4	5966	10162.612	9889	10457	169.2147	0.5871	-0.7683	0.001	0.0010435	5614	73565	57.489	1470	18241	14.255	463	5966	4.6623	8.2472	8.1098	31.4966	32.7065
DP	STAT_known2	cluster_1	SP8	5641	9611.156	9311	9920	188.1971	0.587	-0.7687	0.001	0.001	8789	176424	66.894	782	14719	5.5809	287	5641	2.1389	3.2654	3.1974	36.7008	38.3246
DP	SMAD_2	cluster_4	SP4	13244	22566.727	22103	22955	243.549	0.5869	-0.7688	0.001	0.0010435	20655	212873	53.488	4228	42666	10.721	1302	13244	3.3278	6.3036	6.2215	30.7947	31.0411
DP	ERG_2	cluster_3	DN	6350	10821.554	10539	11124	173.0605	0.5868	-0.769	0.001	0.001	6712	87427	60.427	1430	18064	12.485	490	6350	4.3889	7.3004	7.2632	34.2657	35.1528
DN	E2F2_2	cluster_4	SP4	3876	6609.503	6416	6810	119.5442	0.5865	-0.7698	0.001	0.0010909	3788	64579	78.928	551	8817	10.776	233	3876	4.7372	6.151	6.002	42.2868	43.9605
DP	LMO2_1	cluster_4	SP4	11721	19993.658	19508	20437	270.9299	0.5863	-0.7704	0.001	0.001	40696	452099	54.045	3507	37544	4.4881	1064	11721	1.4012	2.6145	2.5926	30.3393	31.2194
DP	NFATC1_3	cluster_4	SP4	2167	3699.492	3527	3882	110.5265	0.5859	-0.7714	0.001	0.0010909	2771	36045	53.14	597	7250	10.688	170	2167	3.1948	6.135	6.0119	28.4757	29.8897
DN	MYC_known20	cluster_1	SP8	7015	11977.578	11705	12278	172.3255	0.5857	-0.7717	0.001	0.001	11316	185626	73.632	1049	16496	6.5434	430	7015	2.7826	3.7999	3.7791	40.9914	42.5255
DN	GMEB2_3	cluster_1	SP8	806	1377.019	1305	1464	48.1231	0.5856	-0.772	0.001	0.0010435	2360	33394	80.191	130	1755	4.2144	58	806	1.9355	2.4576	2.4136	44.6154	45.9259
DN	EGR1_known12	cluster_1	SP8	38965	66580.495	66011	67210	384.3811	0.5852	-0.7729	0.001	0.001	57885	958377	76.536	5616	89364	7.1366	2382	38965	3.1117	4.1151	4.0657	42.4145	43.6026
DP	ATF2_2	cluster_1	SP8	2198	3758.203	3616	3898	90.5837	0.585	-0.7736	0.001	0.0010435	4893	53941	62.848	567	6053	7.0525	200	2198	2.5609	4.0875	4.0748	35.2734	36.3126
DN	ELK3_1	cluster_4	SP4	2998	5127.78	4892	5347	141.2585	0.5847	-0.7741	0.001	0.0011429	6350	57329	26.539	2201	19599	9.0727	335	2998	1.3878	5.2756	5.2295	15.2204	15.2967
DN	ELK3_2	cluster_4	SP4	2971	5081.824	4876	5313	137.9032	0.5847	-0.7742	0.001	0.0011429	6130	55327	26.313	2198	19577	9.3107	332	2971	1.413	5.416	5.3699	15.1046	15.176
DP	ATF7_1	cluster_3	DN	3201	5476.034	5257	5692	138.5131	0.5846	-0.7744	0.001	0.001	3062	39860	54.566	801	10168	13.919	246	3201	4.382	8.034	8.0306	30.7116	31.4811
DP	MYC_known13	cluster_4	SP4	4261	7297.082	7053	7540	142.8988	0.584	-0.776	0.001	0.0010909	9190	101216	56.829	1221	13066	7.3361	390	4261	2.3924	4.2437	4.2098	31.941	32.6114
DP	POU2F2_known13	cluster_1	SP8	2820	4830.73	4587	5088	150.571	0.5838	-0.7763	0.001	0.0010909	2228	46947	52.881	494	9555	10.763	136	2820	3.1765	6.1041	6.0068	27.5304	29.5133
DP	FLI1_2	cluster_4	SP4	4547	7790.641	7546	8040	155.1674	0.5837	-0.7767	0.001	0.001	6679	86994	61.881	1001	12687	9.0246	351	4547	3.2344	5.2553	5.2268	35.0649	35.8398
DP	ERG_4	cluster_4	SP4	4830	8276.246	8030	8529	152.8053	0.5836	-0.7768	0.001	0.001	6744	87870	61.259	1082	13662	9.5245	372	4830	3.3672	5.516	5.4968	34.3808	35.3535
DP	NFE2L1::MAFG_1	cluster_1	SP8	8702	14910.338	14409	15378	288.0805	0.5836	-0.7768	0.001	0.0010435	8197	173116	53.265	1453	28629	8.8086	417	8702	2.6774	5.0872	5.0267	28.6992	30.3958
DP	MTF1_3	cluster_4	SP4	8421	14431.056	14141	14722	181.0093	0.5836	-0.777	0.001	0.001	12915	169509	62.611	1830	23295	8.6045	644	8421	3.1105	4.9864	4.9679	35.1913	36.1494
DP	E2F7_1	cluster_4	SP4	2804	4809.807	4605	5031	121.0635	0.5831	-0.7783	0.001	0.0010435	4150	54006	59.789	669	8269	9.1544	220	2804	3.1042	5.3012	5.192	32.8849	33.9098
DP	IKZF1_2	cluster_4	SP4	7801	13380.917	13144	13623	151.3363	0.583	-0.7784	0.001	0.0010435	20144	161159	58.804	2975	23108	8.4318	984	7801	2.8465	4.8848	4.8406	33.0756	33.7589
DP	POU2F3_2	cluster_4	SP4	1911	3280.539	3152	3400	78.2856	0.5827	-0.7793	0.001	0.0010909	2892	23182	50.653	848	6599	14.419	244	1911	4.1756	8.4371	8.2435	28.7736	28.9589
DN	KLF7_1	cluster_1	SP8	51901	89086.761	88427	89905	436.6032	0.5826	-0.7794	0.001	0.001	81419	1312000	74.111	8057	123464	6.9741	3308	51901	2.9317	4.0629	3.9559	41.0575	42.0374
DP	ETV5_1	cluster_3	DN	5072	8708.274	8544	8870	94.5779	0.5825	-0.7797	0.001	0.0010435	12055	109704	76.51	1288	11464	7.9952	562	5072	3.5373	4.662	4.6234	43.6335	44.2428
DP	STAT_known3	cluster_1	SP8	5163	8870.715	8566	9197	193.7364	0.5821	-0.7807	0.001	0.0010435	6852	137400	63.695	756	14273	6.6166	262	5163	2.3934	3.8237	3.7576	34.6561	36.1732
DP	ERG_2	cluster_4	SP4	4862	8353.917	8108	8608	161.1086	0.5821	-0.7808	0.001	0.001	6712	87427	60.427	1107	13976	9.6597	375	4862	3.3605	5.587	5.5612	33.8753	34.7882
DP	MGA_2	cluster_4	SP4	5282	9075.919	8793	9394	178.071	0.582	-0.7808	0.001	0.0010909	4878	83018	54.568	1040	16927	11.126	312	5282	3.4718	6.3961	6.3625	30	31.2046
DN	POU2F2_known14	cluster_4	SP4	1174	2018.088	1931	2106	54.7393	0.5819	-0.781	0.001	0.0012632	1238	18993	84.41	195	2647	11.764	83	1174	5.2175	6.7044	6.1812	42.5641	44.3521
DP	ATF6_1	cluster_3	DN	1925	3310.137	3200	3421	67.0257	0.5817	-0.7817	0.001	0.0010435	3671	25707	59.612	810	5616	13.023	275	1925	4.4639	7.4911	7.4882	33.9506	34.2771
DN	EGR4_2	cluster_1	SP8	31821	54736.999	54181	55230	324.5729	0.5814	-0.7825	0.001	0.001	55529	908524	79.001	4521	71084	6.1812	1968	31821	2.767	3.5441	3.5025	43.5302	44.7653
DP	ATF6_1	cluster_1	SP8	1125	1939.102	1850	2017	52.9754	0.5804	-0.7849	0.001	0.0010435	3671	25707	59.612	470	3261	7.5619	160	1125	2.6088	4.3585	4.3762	34.0426	34.4986
DN	ETV5_1	cluster_4	SP4	1179	2032.426	1877	2211	96.2444	0.5803	-0.7851	0.001	0.0010435	4257	38576	26.904	857	7666	5.3464	131	1179	0.82226	3.0773	3.0563	15.2859	15.3796
DP	LMO2_2	cluster_4	SP4	1887	3254.831	3096	3383	84.7885	0.5799	-0.7862	0.001	0.0010435	3407	27268	53.534	783	6146	12.066	237	1887	3.7046	6.9563	6.9202	30.2682	30.7029
DP	RUNX2_4	cluster_3	DN	27707	47795.534	47125	48368	370.352	0.5797	-0.7866	0.001	0.0010435	16438	251335	60.987	5278	79171	19.211	1815	27707	6.7232	11.0415	11.0239	34.388	34.9964
DP	SOX15_4	cluster_1	SP8	1799	3104.064	2916	3270	111.937	0.5797	-0.7866	0.001	0.0011429	1805	25291	45.839	529	6964	12.622	130	1799	3.2607	7.2022	7.1132	24.5747	25.8329
DP	ETV6_1	cluster_3	DN	28523	49207.336	48574	49798	346.2733	0.5797	-0.7867	0.001	0.001	26268	378682	62.769	5707	79282	13.142	2005	28523	4.7279	7.6329	7.5322	35.1323	35.9766
DN	ARID3A_2	cluster_4	SP4	1590	2743.898	2633	2860	68.703	0.5796	-0.7868	0.001	0.0013333	1580	25566	81.304	247	3631	11.547	101	1590	5.0564	6.3924	6.2192	40.8907	43.7896
DP	FLI1_4	cluster_1	SP8	3174	5477.424	5275	5685	122.8972	0.5795	-0.787	0.001	0.001	7089	92416	63.019	690	8773	5.9824	245	3174	2.1644	3.4561	3.4345	35.5072	36.1792
DP	POU2F2_known5	cluster_1	SP8	2569	4435.248	4191	4672	138.712	0.5793	-0.7876	0.001	0.0010909	3181	41440	50.684	730	8982	10.986	202	2569	3.142	6.3502	6.1993	27.6712	28.6016
DP	ATF3_known4	cluster_1	SP8	4371	7546.318	7347	7764	124.9939	0.5793	-0.7877	0.001	0.001	8408	118694	67.508	825	11313	6.4343	310	4371	2.486	3.687	3.6826	37.5758	38.637
DP	SOX4_1	cluster_4	SP4	15084	26042.939	25511	26580	311.7847	0.5792	-0.7878	0.001	0.0010435	10641	172327	54.726	3207	48978	15.554	951	15084	4.7902	8.9371	8.7531	29.6539	30.7975
DP	TBX21_4	cluster_1	SP8	6029	10409.739	10063	10753	207.1139	0.5792	-0.7878	0.001	0.0010909	7045	105923	52.17	1404	20314	10.005	405	6029	2.9694	5.7488	5.6919	28.8462	29.679
DP	SOX14_3	cluster_1	SP8	2464	4255.985	4031	4466	134.1777	0.579	-0.7882	0.001	0.0010909	2759	38663	47.284	698	9269	11.336	177	2464	3.0134	6.4154	6.373	25.3582	26.5832
DP	PBX1_4	cluster_4	SP4	2202	3804.171	3615	3996	110.6611	0.5789	-0.7885	0.001	0.0010909	2319	25948	48.177	750	8086	15.013	198	2202	4.0884	8.5382	8.4862	26.4	27.2323
DP	ERG_4	cluster_3	DN	6190	10697.442	10431	10943	157.5371	0.5787	-0.7892	0.001	0.001	6744	87870	61.259	1392	17593	12.265	478	6190	4.3154	7.0878	7.0445	34.3391	35.1844
DP	SPZ1_2	cluster_1	SP8	6265	10834.502	10565	11114	167.2301	0.5783	-0.7901	0.001	0.0010435	13790	138253	56.134	1996	19554	7.9394	629	6265	2.5437	4.5613	4.5315	31.513	32.0395
DP	XBP1_4	cluster_3	DN	4860	8406.973	8180	8640	152.9877	0.5781	-0.7905	0.001	0.0010435	4834	62945	57.546	1167	14760	13.494	374	4860	4.4431	7.7369	7.721	32.048	32.9268
DN	EGR3_3	cluster_1	SP8	17199	29759.426	29404	30115	215.3661	0.5779	-0.791	0.001	0.001	35070	516864	79.095	2726	38422	5.8797	1182	17199	2.6319	3.3704	3.3276	43.3602	44.7634
DP	SOX21_3	cluster_1	SP8	2778	4809.776	4610	5053	137.3417	0.5777	-0.7917	0.001	0.0010909	3002	45066	51.174	686	9659	10.968	188	2778	3.1545	6.2625	6.1643	27.4052	28.7607
DP	SOX9_4	cluster_1	SP8	3961	6858.866	6555	7117	168.4288	0.5776	-0.792	0.001	0.0010909	4428	66520	51.448	957	13666	10.57	266	3961	3.0635	6.0072	5.9546	27.7952	28.9843
DP	RREB1_2	cluster_1	SP8	64632	111923.658	110816	113302	764.2505	0.5775	-0.7922	0.001	0.001	48689	1172396	58.91	8721	197558	9.9268	2715	64632	3.2476	5.5762	5.5128	31.1318	32.7155
DN	IRF4_1	cluster_4	SP4	3661	6341.769	6204	6491	87.3271	0.5774	-0.7925	0.001	0.0010435	3899	54808	80.813	591	8045	11.862	262	3661	5.398	6.7197	6.6797	44.3316	45.5065
DP	MYC_known13	cluster_1	SP8	3952	6848.942	6618	7076	141.0779	0.5771	-0.7931	0.001	0.0010909	9190	101216	56.829	1134	12181	6.8392	360	3952	2.2189	3.9173	3.9045	31.746	32.444
DP	NFE2L1::MAFG_1	cluster_4	SP4	10938	18957.465	18448	19506	310.6021	0.577	-0.7934	0.001	0.0010435	8197	173116	53.265	1843	36408	11.202	525	10938	3.3654	6.4048	6.3183	28.4862	30.0428
DP	SMAD3_1	cluster_4	SP4	10012	17353.49	17061	17661	188.5227	0.577	-0.7934	0.001	0.0010435	19225	159039	54.071	3964	32413	11.02	1222	10012	3.4039	6.3563	6.2953	30.8274	30.8888
DP	ARID5B_1	cluster_4	SP4	3448	5977.373	5757	6197	133.469	0.5769	-0.7936	0.001	0.0010909	4087	53311	52.552	937	11641	11.475	268	3448	3.3989	6.5574	6.4677	28.6019	29.6194
DP	SOX7_3	cluster_1	SP8	3157	5473.932	5223	5726	152.8254	0.5768	-0.7938	0.001	0.0010909	2800	44824	49.319	758	11442	12.589	201	3157	3.4736	7.1786	7.0431	26.5172	27.5913
DP	SOX11_2	cluster_1	SP8	2757	4780.817	4554	5035	140.0264	0.5768	-0.7939	0.001	0.0010909	3067	42939	50.606	731	9670	11.397	200	2757	3.2493	6.521	6.4207	27.3598	28.5109
DP	PKNOX1_1	cluster_4	SP4	10188	17683.791	17197	18088	260.2658	0.5761	-0.7955	0.001	0.0010435	10906	164350	52.965	2333	33838	10.905	677	10188	3.2833	6.2076	6.199	29.0184	30.1082
DP	KLF12_1	cluster_3	DN	9622	16705.995	16479	16918	140.5956	0.576	-0.7959	0.001	0.001	19413	116478	59.073	4821	28504	14.456	1610	9622	4.8799	8.2934	8.2608	33.3956	33.7567
DN	CUX2_2	cluster_4	SP4	265	461.043	418	504	26.7632	0.5757	-0.7966	0.001	0.0013333	368	3312	68.275	84	716	14.76	30	265	5.4628	8.1522	8.0012	35.7143	37.0112
DP	POU2F2_known11	cluster_1	SP8	2337	4060.172	3876	4216	106.0175	0.5757	-0.7966	0.001	0.0010435	3816	38242	51.233	843	8123	10.882	241	2337	3.1309	6.3155	6.1111	28.5884	28.7702
DP	POU2F2_known14	cluster_3	DN	1105	1920.918	1775	2070	88.7329	0.5755	-0.7972	0.001	0.0012632	685	10526	46.78	326	4330	19.244	77	1105	4.9109	11.2409	10.4978	23.6196	25.5196
DN	IRF_known21	cluster_4	SP4	11361	19769.034	19437	20097	188.6199	0.5747	-0.7991	0.001	0.001	9344	135063	76.665	1864	26418	14.995	791	11361	6.4488	8.4653	8.4116	42.4356	43.0048
DP	PBX3_known1	cluster_1	SP8	3560	6195.547	5996	6412	130.1894	0.5747	-0.7992	0.001	0.0010435	6370	70693	52.566	1114	11983	8.9104	324	3560	2.6472	5.0863	5.0359	29.0844	29.7088
DN	GMEB1_1	cluster_1	SP8	589	1026.248	886	1189	95.1024	0.5744	-0.8	0.001	0.0010435	1931	30986	22.575	293	4645	3.3841	37	589	0.42911	1.9161	1.9009	12.628	12.6803

DP	IRF_known21	cluster_1	SP8	6865	11954.524	11619	12317	205.9312	0.5743	-0.8001	0.001	0.001	6273	90082	51.133	1643	23596	13.394	475	6865	3.8967	7.5721	7.6208	28.9105	29.0939
DP	IRF4_1	cluster_1	SP8	2306	4015.994	3823	4225	113.5174	0.5743	-0.8001	0.001	0.0010435	2787	39153	57.73	513	7035	10.373	164	2306	3.4001	5.8845	5.8897	31.9688	32.779
DP	RREB1_2	cluster_4	SP4	58636	102169.56	100843	103390	737.5789	0.5739	-0.8011	0.001	0.001	48689	1172396	58.91	7941	179955	9.0423	2411	58636	2.9463	4.9518	5.0014	30.3614	32.5837
DP	SPDEF_6	cluster_1	SP8	3069	5349.999	5177	5531	106.3386	0.5737	-0.8016	0.001	0.001	8667	121736	70.334	572	7714	4.4568	220	3069	1.7731	2.5384	2.521	38.4615	39.7848
DP	RUNX3_1	cluster_3	DN	31524	54965.607	54418	55587	365.3976	0.5735	-0.8021	0.001	0.0010435	18870	289545	59.922	6177	92639	19.172	2059	31524	6.524	10.9115	10.8874	33.3333	34.0289
DN	ARID5A_1	cluster_4	SP4	970	1692.56	1611	1772	50.9265	0.5733	-0.8025	0.001	0.0012632	992	13600	80.227	181	2239	13.208	74	970	5.722	7.4597	7.1324	40.884	43.3229
DP	IKZF2_2	cluster_4	SP4	2029	3539.607	3398	3701	89.0824	0.5733	-0.8025	0.001	0.0010435	3311	33116	55.275	694	6615	11.041	206	2029	3.3867	6.2217	6.1269	29.683	30.6727
DP	CTCF_known1	cluster_3	DN	71662	125036.248	123811	126221	747.4104	0.5731	-0.8031	0.001	0.0010435	55720	1030489	59.443	11978	213108	12.293	3925	71662	4.1338	7.0441	6.9542	32.7684	33.6271
DP	SMAD3_3	cluster_1	SP8	1584	2766.862	2623	2900	78.2635	0.5726	-0.8043	0.001	0.0010435	3577	32203	55.927	570	5013	8.7061	176	1584	2.751	4.9203	4.9188	30.8772	31.5978
DP	E2F8_1	cluster_1	SP8	3781	6605.384	6389	6811	130.1948	0.5725	-0.8047	0.001	0.001	7146	78795	61.175	1040	11009	8.5472	347	3781	2.9355	4.8559	4.7985	33.3654	34.3446
DP	ETV6_1	cluster_4	SP4	19676	34370.651	33888	34842	312.7442	0.5725	-0.8047	0.001	0.001	26268	378682	62.769	3975	55382	9.18	1376	19676	3.2614	5.2383	5.1959	34.6164	35.5278
DP	RUNX2_4	cluster_4	SP4	7452	13022.966	12673	13398	217.021	0.5723	-0.8053	0.001	0.0010435	16438	251335	60.987	1481	21763	5.2809	494	7452	1.8083	3.0052	2.965	33.3558	34.2416
DP	ARID3A_2	cluster_3	DN	1490	2607.007	2440	2794	104.3916	0.5717	-0.8067	0.001	0.0013333	987	15963	50.765	379	5462	17.37	96	1490	4.7384	9.7264	9.3341	25.3298	27.2794
DP	TBX15_2	cluster_4	SP4	5477	9582.648	9370	9797	136.0917	0.5716	-0.8069	0.001	0.0010909	14654	102598	53.396	2637	18153	9.4475	786	5477	2.8504	5.3637	5.3383	29.8066	30.1713
DP	SOX3_1	cluster_1	SP8	1940	3395.225	3181	3599	125.732	0.5715	-0.8071	0.001	0.0010909	1698	27182	49.21	481	7166	12.973	124	1940	3.5121	7.3027	7.1371	25.7796	27.0723
DP	PBX1_2	cluster_4	SP4	2720	4760.529	4520	4969	135.4619	0.5715	-0.8073	0.001	0.0010435	2395	33982	48.403	746	10083	14.362	194	2720	3.8743	8.1002	8.0042	26.0054	26.9761
DP	NFATC1_1	cluster_1	SP8	4398	7702.717	7375	8030	198.2248	0.571	-0.8084	0.001	0.0010909	4535	87510	54.48	818	14633	9.1099	234	4398	2.738	5.1599	5.0257	28.6064	30.0554
DP	SOX15_2	cluster_1	SP8	2479	4342.661	4149	4603	132.5672	0.5709	-0.8086	0.001	0.0010909	2513	35262	49.216	679	9108	12.712	179	2479	3.46	7.123	7.0302	26.3623	27.2178
SP4	CUX2_1	cluster_2	DP	340	596.279	528	657	39.8937	0.5709	-0.8086	0.001	0.0012632	439	7949	74.631	51	888	8.3372	20	340	3.1922	4.5558	4.2773	39.2157	38.2883
DP	LMO2_1	cluster_1	SP8	11677	20456.955	19997	20862	265.7051	0.5708	-0.8089	0.001	0.001	40696	452099	54.045	3559	38297	4.5781	1056	11677	1.3959	2.5948	2.5828	29.6713	30.4906
DP	HBP1_1	cluster_4	SP4	4259	7465.4	7236	7721	153.3493	0.5706	-0.8096	0.001	0.0010435	3549	54705	50.814	1056	15222	14.139	283	4259	3.9561	7.9741	7.7854	26.7992	27.9792
DP	KLF12_2	cluster_3	DN	35918	63007.735	62383	63720	401.5114	0.5701	-0.8108	0.001	0.001	38298	549311	63.594	7345	100882	11.679	2555	35918	4.1583	6.6714	6.5387	34.7856	35.604
DN	CUX1_9	cluster_4	SP4	1070	1878.24	1766	1980	66.5498	0.5699	-0.8112	0.001	0.0012632	631	10867	73.396	174	2765	18.675	64	1070	7.2268	10.1426	9.8463	36.7816	38.698
DP	MYBL1_5	cluster_1	SP8	1718	3015.823	2843	3181	104.1656	0.5698	-0.8115	0.001	0.0010435	2741	44252	63.347	322	4890	7	109	1718	2.4593	3.9767	3.8823	33.8509	35.1329
DP	IRF4_2	cluster_4	SP4	6124	10755.999	10438	11083	199.2937	0.5694	-0.8125	0.001	0.001	5446	77752	53.165	1455	20483	14.006	434	6124	4.1874	7.9692	7.8763	29.8282	29.898
DP	PATZ1_1	cluster_3	DN	63682	111838.699	111066	112769	501.1781	0.5694	-0.8125	0.001	0.001	72910	959184	65.12	14139	176126	11.957	4980	63682	4.3234	6.8303	6.6392	35.2217	36.1571
DP	MYBL1_5	cluster_4	SP4	1989	3495.564	3316	3668	104.7054	0.5691	-0.8132	0.001	0.0010435	2741	44252	63.347	371	5637	8.0693	126	1989	2.8472	4.5969	4.4947	33.9623	35.2847
DP	SOX2_5	cluster_1	SP8	2689	4725.731	4507	4985	152.1692	0.5691	-0.8132	0.001	0.0010909	2721	43557	49.369	655	9906	11.228	172	2689	3.0478	6.3212	6.1735	26.2595	27.1452
DP	IKZF1_1	cluster_1	SP8	3842	6752.934	6520	6980	141.4349	0.569	-0.8135	0.001	0.0010435	8537	102703	58.812	1030	11778	6.7446	328	3842	2.2001	3.8421	3.7409	31.8447	32.6201
DP	MGA_3	cluster_4	SP4	4781	8403.273	8117	8696	167.1701	0.569	-0.8135	0.001	0.0010909	4692	79902	56.395	917	15111	10.665	282	4781	3.3744	6.0102	5.9836	30.7525	31.6392
DP	SMAD3_3	cluster_4	SP4	2044	3593.973	3446	3741	86.1893	0.5688	-0.8139	0.001	0.0010435	3577	32203	55.927	744	6513	11.311	228	2044	3.5498	6.3741	6.3472	30.6452	31.3834
DP	FLI1_4	cluster_4	SP4	4744	8342.71	8098	8590	148.8772	0.5687	-0.8143	0.001	0.001	7089	92416	63.019	1063	13399	9.1369	365	4744	3.235	5.1488	5.1333	34.3368	35.4056
DP	SOX1_2	cluster_1	SP8	2449	4307.74	4095	4535	135.2418	0.5686	-0.8145	0.001	0.0010909	2774	38842	48.471	686	9166	11.438	178	2449	3.0561	6.4167	6.305	25.9475	26.7183
DP	POU2F3_1	cluster_4	SP4	3746	6597.512	6330	6871	161.7616	0.5679	-0.8164	0.001	0.0010435	3087	46662	50.134	940	13469	14.471	252	3746	4.0248	8.1633	8.0279	26.8085	27.812
DN	GATA_known17	cluster_4	SP4	7542	13282.965	13010	13558	166.612	0.5678	-0.8165	0.001	0.0010909	5009	80296	75.449	1185	18187	17.089	472	7542	7.0867	9.423	9.3927	39.8312	41.4692
DN	SPIC_2	cluster_4	SP4	21223	37377.169	36984	37774	250.5448	0.5678	-0.8165	0.001	0.0010435	21125	275317	74.226	4012	51184	13.799	1638	21223	5.7217	7.7538	7.7086	40.8275	41.4641
DP	POU2F2_known13	cluster_4	SP4	3928	6920.466	6667	7230	184.902	0.5677	-0.8169	0.001	0.0010909	2228	46947	52.881	704	13662	15.389	194	3928	4.4245	8.7074	8.3669	27.5568	28.7513
DP	FLI1_2	cluster_3	DN	5786	10195.942	9912	10438	167.5394	0.5675	-0.8172	0.001	0.001	6679	86994	61.881	1315	16623	11.824	446	5786	4.1157	6.6776	6.651	33.9163	34.8072
DP	MAF_known11	cluster_1	SP8	6776	11940.77	11590	12294	216.1926	0.5675	-0.8173	0.001	0.0010435	10294	166060	54.212	1445	22364	7.3009	421	6776	2.2121	4.0898	4.0805	29.1349	30.2987
DP	CTCF_known2	cluster_1	SP8	9038	15931.141	15318	16524	346.8447	0.5673	-0.8177	0.001	0.0010435	14645	235553	28.191	3610	56958	6.8167	565	9038	1.0817	3.858	3.8369	15.651	15.8678
DP	ETS_known13	cluster_1	SP8	4306	7602.059	7367	7830	142.3504	0.5665	-0.8199	0.001	0.001	10314	165505	67.697	743	11441	4.6798	272	4306	1.7613	2.6372	2.6017	36.6083	37.6366
DP	SMAD4_1	cluster_1	SP8	14404	25432.064	24972	25980	294.3055	0.5664	-0.8201	0.001	0.001	25085	356038	57.788	3229	44540	7.2292	1015	14404	2.3379	4.0462	4.0456	31.4339	32.3395
DP	RUNX2_5	cluster_4	SP4	8177	14444.363	14068	14867	228.9882	0.5661	-0.8208	0.001	0.001	17640	310092	61.324	1441	24027	4.7516	471	8177	1.6171	2.6701	2.637	32.6856	34.0325
DP	TBX5_5	cluster_1	SP8	5381	9514.159	9144	9839	209.2413	0.5656	-0.8221	0.001	0.0010435	5461	103978	53.836	980	17964	9.3011	285	5381	2.7861	5.2188	5.1751	29.0816	29.9544
DP	TBX19_1	cluster_4	SP4	4366	7724.058	7379	8090	211.1061	0.5653	-0.8229	0.001	0.0010435	3308	63063	49.328	888	16009	12.522	230	4366	3.4151	6.9528	6.9232	25.9009	27.2722
DP	SOX9_6	cluster_1	SP8	3378	5977.219	5709	6258	165.7465	0.5652	-0.8231	0.001	0.0010909	3625	54432	49.299	871	12456	11.281	226	3378	3.0594	6.2345	6.2059	25.9472	27.1195
DP	GMEB2_2	cluster_3	DN	2551	4518.126	4320	4701	111.4234	0.5647	-0.8244	0.001	0.001	2591	34443	62.139	576	7389	13.331	194	2551	4.6023	7.4875	7.4064	33.6806	34.5243
DP	POU2F3_2	cluster_1	SP8	1447	2563.628	2436	2684	72.6793	0.5646	-0.8247	0.001	0.0010909	2892	23182	50.653	656	5138	11.227	183	1447	3.1617	6.3278	6.2419	27.8963	28.1627
DP	SOX18_4	cluster_1	SP8	1575	2793.46	2630	2989	102.837	0.564	-0.8263	0.001	0.0012	1791	25128	47.709	451	5993	11.379	114	1575	2.9904	6.3652	6.2679	25.2772	26.2807
DP	TBX4_2	cluster_1	SP8	6019	10672.967	10281	11054	236.6287	0.564	-0.8263	0.001	0.0010909	6487	123601	55.329	1068	19559	8.7555	320	6019	2.6944	4.9329	4.8697	29.9625	30.7736
DP	ELK4_1	cluster_4	SP4	2413	4281.212	4186	4398	62.7005	0.5637	-0.8269	0.001	0.001	10661	85897	77.625	703	5569	5.0327	301	2413	2.1806	2.8234	2.8092	42.8165	43.3291
DP	SPDEF_6	cluster_3	DN	8699	15439.495	15155	15731	178.956	0.5635	-0.8276	0.001	0.001	8667	121736	70.334	1652	22357	12.917	625	8699	5.0259	7.2113	7.1458	37.8329	38.9095
DP	TBX4_1	cluster_4	SP4	4491	7975.974	7794	8154	110.7396	0.5631	-0.8285	0.001	0.0010435	11484	80419	53.597	2178	15032	10.018	644	4491	2.9931	5.6078	5.5845	29.5684	29.8763
DP	SOX13_1	cluster_1	SP8	1689	3004.167	2813	3200	113.6905	0.5624	-0.8304	0.001	0.0010909	1690	25424	51.067	443	6166	12.385	116	1689	3.3925	6.8639	6.6433	26.1851	27.3922
DN	GMEB1_1	cluster_4	SP4	568	1011.44	864	1169	90.3202	0.562	-0.8313	0.001	0.0010435	1931	30986	22.575	294	4624	3.3688	36	568	0.41381	1.8643	1.8331	12.2449	12.2837
DP	PBX1_2	cluster_1	SP8	2390	4253.232	4043	4427	115.5547	0.562	-0.8313	0.001	0.0010435	2395	33982	48.403	666	8974	12.782	173	2390	3.4042	7.2234	7.0331	25.976	26.6325
DN	IRF4_2	cluster_4	SP4	8712	15531.204	15283	15788	148.8366	0.561	-0.834	0.001	0.001	7884	113080	77.321	1455	20483	14.006	602	8712	5.957	7.6357	7.7043	41.3746	42.5328
DP	FLI1_2	cluster_1	SP8	2925	5217.079	5015	5409	119.3742	0.5607	-0.8346	0.001	0.001	6679	86994	61.881	671	8532	6.069	225	2925	2.0806	3.3688	3.3623	33.532	34.2827
DP	SOX2_6	cluster_1	SP8	1613	2879.64	2698	3061	112.3155	0.5603	-0.8357	0.001	0.0010435	1589	25439	47.17	424	6325	11.728	102	1613	2.9909	6.4191	6.3407	24.0566	25.502
DP	MYCN_1	cluster_1	SP8	3755	6703.082	6470	6903	132.7881	0.5603	-0.8358	0.001	0.001	10303	113712	63.225	997	10742	5.9726	340	3755	2.0878	3.3	3.3022	34.1023	34.9562
DP	XBP1_4	cluster_1	SP8	2509	4479.879	4288	4690	122.185	0.5602	-0.8361	0.001	0.0010435	4834	62945	57.546	616	7855	7.1813	193	2509	2.2938	3.9926	3.986	31.3312	31.9414
DP	MTF1_1	cluster_1	SP8	14969	26726.638	26374	27120	244.7054	0.5601	-0.8363	0.001	0.001	40601	541813	68.67	3054	39477	5.0033	1137	14969	1.8972	2.8004	2.7628	37.2299	37.9183
DN	FOXO1_4	cluster_4	SP4	3086	5511.776	5352	5673	98.0965	0.56	-0.8366	0.001	0.0010909	2797	36450	73.199	628	7807	15.678	240	3086	6.1973	8.5806	8.4664	38.2166	39.5286
DP	ERG_4	cluster_1	SP8	3122	5577.601	5403	5768	115.9062	0.5598	-0.837	0.001	0.001	6744	87870	61.259	724	9199	6.4131	241	3122	2.1765	3.5735	3.553	33.2873	33.9385
DP	RUNX3_1	cluster_4	SP4	8289	14808.322	14421	15181	242.2419	0.5598	-0.8371	0.001	0.0010435	18870	289545	59.922	1715	25185	5.2121	550	8289	1.7154	2.9147	2.8628	32.07	32.9124
DP	SPZ1_1	cluster_3	DN	48945	87438.007	86764	88258	460.3174	0.5598	-0.8371	0.001	0.001	49189	720565	65.335	9814	136683	12.393	3422	48945	4.4379	6.9568	6.7926	34.8686	35.8091
DP	ERG_2	cluster_1	SP8	3091	5524.527	5308	5714	129.2282	0.5596	-0.8376	0.001	0.001	6712	87427	60.427	729	9239	6.3857	239	3091	2.1364	3.5608	3.5355	32.7846	33.456
DN	RREB1_2	cluster_4	SP4	21759	38890.413	37813	39969	685.5905	0.5595	-0.8378	0.001	0.001	18681	474233	23.829	7941	179955	9.0423	886	21759	1.0933	4.7428	4.5883	11.1573	12.0914
DP	E2F7_1	cluster_1	SP8	2433	4350.927	4175	4547	107.3407	0.5593	-0.8383	0.001	0.0010435	4150	54006	59.789	603	7441	8.2378	190	2433	2.6935	4.5783	4.5051	31.5091	32.6972
DP	TBX2_1	cluster_1	SP8	6845	12247.865	11868	12625	219.4819	0.5589	-0.8393	0.001	0.0010435	8226	140221	57.494	1317	21623	8.866	404	6845	2.8066	4.9113	4.8816	30.6758	31.6561
DN	IRF_known14	cluster_4	SP4	26015	46549.557	45888	47128	365.439	0.5589	-0.8394	0.001	0.001	16394	342215	77.672	3125	62308	14.142	1263	26015	5.9046	7.704	7.6019	40.416	41.7523
DN	SPIB_2	cluster_4	SP4	22027	39425.955	39051	39849	249.4259	0.5587	-0.8398	0.001	0.0010435	23403	305650	75.803	4131	52835	13.103	1695	22027	5.4629	7.2427	7.2066	41.0312	41.6902
DP	FLI1_4	cluster_3	DN	6031	10800.016	10540	11069	159.6448	0.5585	-0.8405	0.001	0.001	7089	92416	63.019	1366	17315	11.807	464	6031	4.1126	6.5454	6.5259	33.9678	34.8311
DP	SOX12_1	cluster_1	SP8	2082	3731.218	3547	3922	116.2873	0.5581	-0.8414	0.001	0.0010435	2452	31970	50.662	625	7670	12.154	163	2082	3.2993	6.6476	6.5124	26.08	27.1447
DP	IKZF3_1	cluster_1	SP8	2826	5065.786	4863	5277	121.1501	0.5579	-0.8418	0.001	0.0010435	6241	75044	55.627	817	9334	6.9189	241	2826	2.0948	3.8616	3.7658	29.4982	30.2764
DP	MAF_known12	cluster_4	SP4	7224	12950.695	12590	13307	228.1212	0.5578	-0.8421	0.001	0.0010435	7384	125901	57.08	1414	23154	10.497	429	7224	3.2752	5.8099	5.7378	30.3395	31.1998
DN	TCF7L2_known5	cluster_4	SP4	22021	39481.442	39016	39943	273.5512	0.5578	-0.8423	0.001	0.0010435	13945	223921	71.487	3628	56623	18.077	1381	22021	7.0302	9.9032	9.8343	38.065	38.8906
DN	RUNX2_4	cluster_4	SP4	9911	17775.15	17521	18004	151.4686	0.5576	-0.8427	0.001	0.0010435	22375	342168	83.028	1481	21763	5.2809	658	9911	2.4049	2.9408	2.8965	44.4294	45.5406
DN	SPI1_known4	cluster_4	SP4	18193	32633.664	32252	32967	219.6151	0.5575	-0.8429	0.001	0.0010435	19284	251359	76.991	3409	43240	13.244	1406	18193	5.5725	7.291	7.2379	41.2438	42.0745
DP	HES7_1	cluster_1	SP8	3119	5595.174	5409	5787	113.5172	0.5575	-0.8429	0.001	0.0010435	9992	110416	62.326	845	9092	5.1321	285	3119	1.7606	2.8523	2.8248	33.7278	34.3049
DP	SOX18_2	cluster_1	SP8	2075	3722.936	3541	3892	105.8855	0.5575	-0.843	0.001	0.0010909	2288	32032	48.779	591	7867	11.98	151	2075	3.1598	6.5997	6.4779	25.5499	26.376
DP	IRF6_1	cluster_3	DN	3021	5425.077	5183	5655	138.7653	0.5569	-0.8444	0.001	0.0010909	2388	38278	58.421	609	9420	14.377	190	3021	4.6107	7.9564	7.8923	31.1987	32.0701
DP	E2F8_1	cluster_4	SP4	3495	6277.43	6094	6497	125.1921	0.5568	-0.8447	0.001	0.001	7146	78795	61.175	989	10443	8.1077	325	3495	2.7134	4.548	4.4356	32.8615	33.4674
DN	GATA_known16	cluster_4	SP4	12287	22070.497	21581	22463	251.2931	0.5567	-0.8449	0.001	0.0010435	6076	128052	74.197	1561	30959	17.939	591	12287	7.1194	9.7268	9.5953	37.8603	39.688
DP	ETV5_1	cluster_4	SP4	3232	5806.664	5684	5944	76.8278	0.5567	-0.8451	0.001	0.0010435	12055	109704	76.51	857	7666	5.3464	358	3232	2.2541	2.9697	2.9461	41.7736	42.1602
DP	TBX4_2	cluster_4	SP4	6907	12425.849	12084	12827	232.3407	0.5559	-0.8471	0.001	0.0010909	6487	123601	55.329	1263	22909	10.255	364	6907	3.0919	5.6112	5.5881	28.8203	30.1497
DP	RUNX2_4	cluster_1	SP8	10519	18927.484	18479	19385	264.6345	0.5558	-0.8474	0.001	0.0010435	16438	251335	60.987	2118	31471	7.6366	693	10519	2.5525	4.2158	4.1853	32.7195	33.4244
DP	REL_1	cluster_1	SP8	5286	9517.958	9282	9761	145.8532	0.5554	-0.8484	0.001	0.001	11260	101830	56.863	1898	16902	9.4382	584	5286	2.9517	5.1865	5.191	30.7692	31.2744
DP	POU2F2_known12	cluster_4	SP4	3173	5714.329	5509	5903	120.6284	0.5553	-0.8485	0.001	0.0010435	3757	37632	51.558	1183	11402	15.621	326	3173	4.3472	8.6771	8.4317	27.5571	27.8285
DN	TCF7L2_known7	cluster_4	SP4	21095	37990.636	37498	38441	270.1603	0.5553	-0.8487	0.001	0.0010435	14358	201641	70.809	4019	55081	19.343	1511	21095	7.4078	10.5237	10.4617	37.5964	38.2981
DP	MYBL1_3	cluster_4	SP4	824	1485.226	1384	1589	61.4952	0.5551	-0.8492	0.001	0.0010909	1344	14803	55.839	257	2708	10.215	76	824	3.1083	5.6548	5.5664	29.572	30.4284
DP	RUNX3_1	cluster_1	SP8	12620	22750.395	22292	23181	266.1799	0.5547	-0.8501	0.001	0.0010435	18870	289545	59.922	2579	38479	7.9634	825	12620	2.6118	4.372	4.3586	31.9891	32.7971
DP	SOX1_3	cluster_1	SP8	2170	3912.914	3700	4126	125.5611	0.5547	-0.8503	0.001	0.0010909	2535	35523	47.207	648	8538	11.346	156	2170	2.8837	6.1538	6.1087	24.0741	25.4158
DP	ATF3_known5	cluster_1	SP8	7503	13528.57	13142	13884	225.9747	0.5546	-0.8504	0.001	0.001	11264	158203	55.075	1826	24906	8.6706	538	7503	2.612	4.7763	4.7426	29.4633	30.1253
DP	REL_2	cluster_1	SP8	5204	9383.852	9162	9600	131.818	0.5546	-0.8504	0.001	0.0010435	11409	103099	57.237	1862	16571	9.1996	575	5204	2.8891	5.0399	5.0476	30.8808	31.4043
DP	FOXO1_5	cluster_4	SP4	12547	22661.364	22280	23071	232.4677	0.5537	-0.8528	0.001	0.0010435	23967	264888	55.536	3867	41421	8.6842	1142	12547	2.6306	4.7649	4.7367	29.5319	30.2914
DP	TBX5_5	cluster_4	SP4	6400	11561.472	11185	11946	227.9539	0.5536	-0.8531	0.001	0.0010435	5461	103978	53.836	1206	21904	11.341	338	6400	3.3137	6.1893	6.1551	28.0265	29.2184
DP	KLF4_1	cluster_3	DN	45376	81991.024	81417	82610	362.0065	0.5534	-0.8535	0.001	0.001	76804	740702	63.769	14112	130789	11.26	4811	45376	3.9065	6.264	6.1261	34.0916	34.694
DP	STAT_known3	cluster_3	DN	10227	18485.47	18084	18912	261.5746	0.5533	-0.8539	0.001	0.0010435	6852	137400	63.695	1560	29619	13.731	517	10227	4.741	7.5452	7.4432	33.141	34.5285
DP	TBX2_1	cluster_4	SP4	7012	12675.736	12338	13068	229.8388	0.5532	-0.8541	0.001	0.0010435	8226	140221	57.494	1365	22454	9.2067	414	7012	2.8751	5.0328	5.0007	30.3297	31.2283
DP	IRF6_1	cluster_4	SP4	2241	4053.414	3853	4256	121.8279	0.553	-0.8547	0.001	0.0010909	2388	38278	58.421	466	7185	10.966	141	2241	3.4203	5.9045	5.8545	30.2575	31.19
DN	ATF4_2	cluster_4	SP4	3991	7222.672	7044	7415	110.228	0.5526	-0.8556	0.001	0.0010909	4963	59634	73.418	871	10071	12.399	334	3991	4.9135	6.7298	6.6925	38.3467	39.6286
DP	SOX30_1	cluster_1	SP8	2653	4801.467	4582	5027	136.6054	0.5526	-0.8556	0.001	0.0010435	3080	46247	52.079	664	9477	10.672	177	2653	2.9875	5.7468	5.7366	26.6566	27.9941
DP	ETS_known17	cluster_3	DN	15882	28741.372	28202	29293	316.8341	0.5526	-0.8557	0.001	0.001	12494	213553	60.343	2924	48134	13.601	938	15882	4.4877	7.5076	7.437	32.0793	32.9954
DP	HIF1A::ARNT_1	cluster_3	DN	5173	9363.231	9194	9549	107.9201	0.5525	-0.8559	0.001	0.001	10494	75476	67.161	1969	14045	12.498	723	5173	4.6031	6.8897	6.8538	36.7191	36.8316
DP	TCF12_known1	cluster_4	SP4	8721	15786.494	15549	16066	153.5257	0.5525	-0.8561	0.001	0.0010909	36358	261819	56.235	4034	28399	6.0997	1228	8721	1.8731	3.3775	3.3309	30.4412	30.7088
DP	IRF_known14	cluster_1	SP8	13175	23851.52	23244	24521	366.8972	0.5524	-0.8562	0.001	0.001	11310	235509	53.453	2296	45729	10.379	639	13175	2.9903	5.6499	5.5943	27.831	28.811
DP	FOXP1_1	cluster_1	SP8	11004	19923.657	19328	20565	365.3364	0.5523	-0.8564	0.001	0.0010435	8153	191364	49.307	2385	45631	11.757	531	11004	2.8353	6.5129	5.7503	22.2642	24.1152
DN	RUNX3_1	cluster_4	SP4	11256	20383.761	20078	20655	172.9185	0.5522	-0.8567	0.001	0.0010435	25951	398073	82.383	1715	25185	5.2121	747	11256	2.3295	2.8785	2.8276	43.5569	44.6933
DP	SOX30_1	cluster_4	SP4	3455	6258.672	6019	6529	153.6073	0.5521	-0.857	0.001	0.0010435	3080	46247	52.079	872	12336	13.892	235	3455	3.8907	7.6299	7.4708	26.9495	28.0075
DP	ATF2_2	cluster_3	DN	3864	7000.243	6802	7205	122.271	0.552	-0.8571	0.001	0.0010435	4893	53941	62.848	1035	11230	13.084	352	3864	4.502	7.194	7.1634	34.0097	34.4078
DP	E2F_known24	cluster_1	SP8	2245	4067.67	3863	4280	123.7249	0.552	-0.8572	0.001	0.001	2757	47016	60.976	422	6854	8.8891	133	2245	2.9116	4.8241	4.775	31.5166	32.7546
DP	MAFF_1	cluster_4	SP4	7447	13491	13119	13856	225.2087	0.552	-0.8572	0.001	0.001	9294	130389	52.132	1924	26154	10.457	532	7447	2.9775	5.7241	5.7114	27.6507	28.4737
DN	TBX21_6	cluster_4	SP4	11928	21611.362	21216	21981	235.5252	0.552	-0.8574	0.001	0.0010909	10503	189588	74.498	1741	29870	11.737	669	11928	4.6871	6.3696	6.2915	38.4262	39.933
SP4	NFE2L1::MAFG_1	cluster_3	DN	17659	31999.506	31275	32634	401.5774	0.5519	-0.8576	0.001	0.0010435	8781	185151	56.968	2840	57401	17.661	844	17659	5.4334	9.6117	9.5376	29.7183	30.7643
DP	E2F2_3	cluster_3	DN	2270	4114.663	3924	4295	114.4341	0.5518	-0.8578	0.001	0.001	1923	32730	61.531	432	6875	12.925	139	2270	4.2675	7.2283	6.9355	32.1759	33.0182
DN	NFATC1_3	cluster_4	SP4	3037	5506.446	5354	5662	91.6986	0.5516	-0.8583	0.001	0.0010909	4137	53815	79.338	597	7250	10.688	240	3037	4.4774	5.8013	5.6434	40.201	41.8897
DN	CUX2_2	cluster_1	SP8	239	434.348	391	477	25.6788	0.5513	-0.8591	0.001	0.0013333	368	3312	68.275	79	675	13.915	28	239	4.9268	7.6087	7.2162	35.443	35.4074
DP	KLF16_1	cluster_3	DN	50939	92430.816	91760	93111	420.3691	0.5511	-0.8596	0.001	0.001	76014	850789	64.652	13662	146104	11.103	4647	50939	3.8709	6.1133	5.9873	34.0141	34.8649
DP	TBX1_4	cluster_4	SP4	9165	16638.864	16129	17164	311.8295	0.5508	-0.8603	0.001	0.0010909	7984	152301	52.016	1814	32620	11.141	485	9165	3.1302	6.0746	6.0177	26.7365	28.0963
DN	E2F_known26	cluster_4	SP4	1781	3236.87	3095	3371	82.6461	0.5504	-0.8615	0.001	0.0010435	2046	34813	80.496	267	4221	9.76	108	1781	4.1181	5.2786	5.1159	40.4494	42.1938
DN	HMGA1_1	cluster_4	SP4	2027	3684.33	3592	3763	48.0315	0.5503	-0.8617	0.001	0.0010435	4316	26040	76.796	821	4883	14.401	340	2027	5.9779	7.8777	7.7842	41.4129	41.5114
DP	PBX3_known1	cluster_4	SP4	3895	7083.915	6843	7295	138.2738	0.5499	-0.8628	0.001	0.0010435	6370	70693	52.566	1282	13739	10.216	355	3895	2.8963	5.573	5.5097	27.6911	28.35
DP	SP8_1	cluster_3	DN	48441	88092.172	87425	88779	430.5749	0.5499	-0.8628	0.001	0.001	63765	778558	62.307	12378	144666	11.577	4056	48441	3.8767	6.3609	6.2219	32.7678	33.4847
DN	HESX1_1	cluster_4	SP4	866	1575.886	1505	1637	38.4726	0.5498	-0.863	0.001	0.0012632	1814	16345	82.401	235	1983	9.997	98	866	4.3658	5.4024	5.2983	41.7021	43.6712

DN	TCF7_1	cluster_4	SP4	22653	41235.41	40728	41760	314.4715	0.5494	-0.8642	0.001	0.0010435	13527	217263	71.046	3834	59721	19.529	1420	22653	7.4076	10.4975	10.4265	37.037	37.9314
DP	SOX14_2	cluster_1	SP8	1954	3558.974	3360	3731	104.4615	0.5492	-0.8647	0.001	0.0010909	2895	31972	49.947	690	7315	11.428	178	1954	3.0526	6.1485	6.1116	25.7971	26.7122
DP	MYCN_1	cluster_3	DN	8154	14852.936	14570	15147	171.5822	0.549	-0.8651	0.001	0.001	10303	113712	63.225	2200	23706	13.181	742	8154	4.5337	7.2018	7.1707	33.7273	34.3964
DP	FOXP3_2	cluster_4	SP4	2497	4551.503	4457	4640	59.4087	0.5487	-0.8659	0.001	0.0010909	5499	32994	72.569	1078	6365	13.999	418	2497	5.492	7.6014	7.568	38.7755	39.2302
DP	SPZ1_2	cluster_4	SP4	6917	12607.397	12327	12890	169.1762	0.5487	-0.866	0.001	0.0010435	13790	138253	56.134	2339	22816	9.2638	696	6917	2.8085	5.0471	5.0031	29.7563	30.3164
DP	SP4_2	cluster_3	DN	55374	100982.299	100063	101970	558.8334	0.5484	-0.8668	0.001	0.001	56997	957954	67.242	9762	154277	10.829	3408	55374	3.8869	5.9793	5.7804	34.9109	35.8926
SP8	NFE2L1::MAFG_1	cluster_3	DN	16847	30724.645	30124	31304	361.8118	0.5483	-0.8669	0.001	0.0010435	8430	177761	54.694	2840	57401	17.661	807	16847	5.1835	9.573	9.4773	28.4155	29.3497
DP	FOXO6_3	cluster_4	SP4	12420	22653.391	22231	23064	253.2735	0.5483	-0.867	0.001	0.0010435	21070	275513	56.268	3237	40909	8.3549	955	12420	2.5365	4.5325	4.508	29.5026	30.3601
DN	CBX5_1	cluster_4	SP4	4463	8141.731	7989	8291	93.5159	0.5482	-0.8672	0.001	0.0010435	5190	57165	75.199	1068	11228	14.77	414	4463	5.871	7.9769	7.8072	38.764	39.7488
DP	STAT_known2	cluster_4	SP4	6176	11268.936	10966	11575	186.2608	0.5481	-0.8675	0.001	0.001	8789	176424	66.894	910	17211	6.5258	312	6176	2.3417	3.5499	3.5007	34.2857	35.884
DN	TBX20_5	cluster_4	SP4	16001	29208.201	28827	29611	235.2568	0.5478	-0.8682	0.001	0.0010909	16570	253871	75.218	2754	40150	11.896	1058	16001	4.7409	6.385	6.3028	38.4168	39.8531
DN	TCF3_1	cluster_1	SP8	17984	32832.812	32292	33416	318.8648	0.5478	-0.8684	0.001	0.0011429	49855	709136	57.352	4255	58143	4.7023	1277	17984	1.4545	2.5614	2.536	30.0118	30.9306
DN	TCF7_2	cluster_4	SP4	16954	30976.97	30645	31340	220.7566	0.5473	-0.8695	0.001	0.0010435	15144	167157	70.642	4118	44691	18.887	1538	16954	7.1649	10.1558	10.1426	37.3482	37.936
DP	KLF12_1	cluster_1	SP8	4766	8708.862	8528	8881	111.848	0.5473	-0.8696	0.001	0.001	19413	116478	59.073	2507	14856	7.5344	798	4766	2.4171	4.1106	4.0918	31.8309	32.0813
DN	TCF7L1_1	cluster_4	SP4	20725	37888.853	37369	38330	294.0445	0.547	-0.8704	0.001	0.0010435	12339	198117	70.718	3526	55052	19.651	1301	20725	7.3978	10.5438	10.461	36.8973	37.6462
DP	HESX1_1	cluster_4	SP4	544	995.449	907	1089	52.9479	0.5469	-0.8705	0.001	0.0012632	1124	10127	51.054	235	1983	9.997	61	544	2.7425	5.427	5.3718	25.9574	27.4332
DP	MAFG_1	cluster_4	SP4	7111	13002.917	12609	13439	250.2237	0.5469	-0.8706	0.001	0.0010435	5947	119184	52.184	1341	25357	11.102	356	7111	3.1135	5.9862	5.9664	26.5474	28.0435
DN	STAT_known2	cluster_4	SP4	7643	13986.898	13693	14240	165.9208	0.5465	-0.8718	0.001	0.001	11028	221310	83.913	910	17211	6.5258	388	7643	2.898	3.5183	3.4535	42.6374	44.4076
DP	POU2F2_known11	cluster_4	SP4	3173	5808.385	5639	6001	112.1241	0.5464	-0.8721	0.001	0.0010435	3816	38242	51.233	1193	11573	15.504	324	3173	4.2509	8.4906	8.2972	27.1584	27.4173
DP	PATZ1_1	cluster_1	SP8	34327	62843.183	62129	63531	407.0481	0.5462	-0.8724	0.001	0.001	72910	959184	65.12	7774	98544	6.6902	2650	34327	2.3305	3.6346	3.5788	34.088	34.8342
DP	EGR1_known12	cluster_3	DN	46545	85233.951	84369	86018	519.5773	0.5461	-0.8728	0.001	0.001	50191	833460	66.56	8501	131962	10.538	2885	46545	3.7171	5.748	5.5846	33.9372	35.2715
DN	POU2F2_known13	cluster_4	SP4	5338	9778.754	9494	10056	163.6126	0.5459	-0.8732	0.001	0.0010909	3197	67338	75.85	704	13662	15.389	264	5338	6.0128	8.2577	7.9272	37.5	39.0719
DN	BPTF_1	cluster_4	SP4	13582	24881.696	24558	25196	187.8404	0.5459	-0.8733	0.001	0.0010909	15844	211822	75.51	2650	34033	12.132	1033	13582	4.8417	6.5198	6.412	38.9811	39.9083
DP	STAT_known3	cluster_4	SP4	5618	10292.11	9977	10590	186.2476	0.5459	-0.8733	0.001	0.0010435	6852	137400	63.695	869	16490	7.6443	284	5618	2.6044	4.1448	4.0888	32.6812	34.0691
DN	SOX14_2	cluster_4	SP4	3568	6537.2	6364	6716	101.1678	0.5459	-0.8734	0.001	0.0010909	4224	46656	72.886	866	9223	14.408	327	3568	5.574	7.7415	7.6475	37.7598	38.6859
DP	HES1_1	cluster_3	DN	25990	47669.032	47029	48234	375.1645	0.5452	-0.8751	0.001	0.001	24843	352037	63.079	5551	76425	13.694	1850	25990	4.657	7.4468	7.3827	33.3273	34.0072
DP	E2F_known26	cluster_4	SP4	1314	2411.115	2257	2580	98.0616	0.5452	-0.8752	0.001	0.0010435	1515	25773	59.594	267	4221	9.76	78	1314	3.0383	5.1485	5.0984	29.2135	31.1301
DN	ATF4_3	cluster_4	SP4	4480	8222.872	8032	8417	117.5852	0.5449	-0.876	0.001	0.0010909	4863	63288	72.723	930	11616	13.348	346	4480	5.1479	7.1149	7.0788	37.2043	38.5675
DP	SMAD_1	cluster_4	SP4	6499	11933.988	11696	12185	156.8824	0.5446	-0.8767	0.001	0.0010435	15462	123736	56.699	2718	21297	9.7589	817	6499	2.978	5.2839	5.2523	30.0589	30.516
DP	SOX_1	cluster_4	SP4	5582	10260.39	9968	10536	173.8399	0.5441	-0.8781	0.001	0.0010435	5830	70005	50.837	1772	20522	14.903	467	5582	4.0536	8.0103	7.9737	26.3544	27.2001
DP	MYBL2_2	cluster_1	SP8	1460	2684.406	2543	2828	89.033	0.5441	-0.8782	0.001	0.0010435	2149	32599	60.53	315	4541	8.4317	98	1460	2.7109	4.5603	4.4787	31.1111	32.1515
DN	RREB1_1	cluster_4	SP4	49654	91320.218	90696	91981	404.28	0.5437	-0.879	0.001	0.001	60217	1008607	79.968	7266	119347	9.4625	2872	49654	3.9368	4.7694	4.923	39.5266	41.6047
DP	E2F2_2	cluster_4	SP4	2706	4980.362	4757	5188	138.7913	0.5434	-0.8798	0.001	0.0010909	2804	47831	58.459	551	8817	10.776	160	2706	3.3073	5.7061	5.6574	29.0381	30.6907
DN	FOXO3_1	cluster_4	SP4	6277	11556.954	11313	11811	144.382	0.5432	-0.8805	0.001	0.0010435	6232	81801	74.85	1311	16241	14.861	494	6277	5.7436	7.9268	7.6735	37.6812	38.6491
DP	XBP1_2	cluster_3	DN	3982	7332.828	7047	7649	188.3802	0.5431	-0.8807	0.001	0.0010435	2416	55665	57.716	600	12989	13.468	174	3982	4.1287	7.202	7.1535	29	30.6567
DN	ELK4_1	cluster_4	SP4	2531	4663.751	4564	4750	56.7383	0.5428	-0.8815	0.001	0.001	11601	93425	84.428	703	5569	5.0327	316	2531	2.2873	2.7239	2.7091	44.9502	45.448
DN	SP8_1	cluster_4	SP4	9275	17095.101	16593	17590	316.3473	0.5426	-0.8821	0.001	0.001	21179	262744	21.027	7107	84496	6.7621	739	9275	0.74227	3.4893	3.5301	10.3982	10.9769
DN	MYBL1_5	cluster_4	SP4	2432	4483.57	4353	4613	84.7385	0.5425	-0.8822	0.001	0.0010435	3552	57284	82.002	371	5637	8.0693	153	2432	3.4814	4.3074	4.2455	41.2399	43.1435
DP	TCF3_1	cluster_4	SP4	17235	31772.3	31243	32305	313.8313	0.5425	-0.8824	0.001	0.0011429	49855	709136	57.352	4178	56573	4.5754	1226	17235	1.3939	2.4591	2.4304	29.3442	30.4651
DN	STAT_known3	cluster_4	SP4	7123	13142.509	12890	13381	154.5607	0.542	-0.8836	0.001	0.0010435	8877	177940	82.488	869	16490	7.6443	362	7123	3.302	4.078	4.003	41.6571	43.1959
DN	MYBL1_3	cluster_4	SP4	1114	2056.586	1982	2131	47.0942	0.5419	-0.8839	0.001	0.0010909	1868	20588	77.661	257	2708	10.215	102	1114	4.2022	5.4604	5.4109	39.6887	41.1374
DP	TBX20_2	cluster_4	SP4	5379	9926.802	9621	10236	193.7981	0.5419	-0.8839	0.001	0.0010435	5116	92297	53.087	1131	19160	11.02	302	5379	3.0939	5.903	5.8279	26.702	28.0741
DP	SOX17_4	cluster_1	SP8	1936	3574.267	3396	3761	108.5022	0.5418	-0.8842	0.001	0.0010909	2192	30688	48.463	569	7564	11.945	141	1936	3.0574	6.4325	6.3087	24.7803	25.5949
DN	CUX2_1	cluster_4	SP4	609	1125.139	1027	1226	57.9139	0.5417	-0.8845	0.001	0.0012632	444	7998	75.092	110	1680	15.773	37	609	5.7178	8.3333	7.6144	33.6364	36.25
DN	SOX2_5	cluster_4	SP4	4957	9165.901	8939	9396	136.2497	0.5409	-0.8867	0.001	0.0010909	4046	64802	73.448	874	13039	14.779	316	4957	5.6184	7.8102	7.6495	36.1556	38.0167
DP	HIF1A_1	cluster_1	SP8	3508	6487.16	6300	6691	117.3987	0.5408	-0.8867	0.001	0.0010435	11588	130466	68.094	879	9649	5.0361	316	3508	1.8309	2.727	2.6888	35.9499	36.3561
DN	LMO2_1	cluster_4	SP4	5992	11084.338	10717	11434	231.3509	0.5406	-0.8873	0.001	0.001	22843	252436	30.177	3507	37544	4.4881	549	5992	0.7163	2.4034	2.3737	15.6544	15.9599
DN	ERG_4	cluster_4	SP4	5533	10235.879	10025	10422	119.0919	0.5406	-0.8874	0.001	0.001	8373	109062	76.033	1082	13662	9.5245	428	5533	3.8573	5.1117	5.0733	39.5564	40.4992
DN	IKZF2_3	cluster_4	SP4	4965	9187.262	9018	9357	101.0092	0.5405	-0.8877	0.001	0.0010909	7087	71094	80.484	1246	11893	13.464	507	4965	5.6208	7.1539	6.9837	40.6902	41.7472
DN	RBPJ_2	cluster_4	SP4	6674	12349.085	12160	12556	115.2192	0.5405	-0.8877	0.001	0.0010909	13859	138744	77.101	1692	16343	9.0819	677	6674	3.7088	4.8849	4.8103	40.0118	40.8371
DN	SOX18_4	cluster_4	SP4	3104	5743.77	5563	5929	107.7598	0.5405	-0.8877	0.001	0.0012	2707	37982	72.115	642	8375	15.901	227	3104	5.8934	8.3857	8.1723	35.3583	37.0627
DN	ETV7_1	cluster_4	SP4	20615	38150.602	37658	38568	267.7631	0.5404	-0.888	0.001	0.001	30877	278376	62.701	6880	61259	13.798	2288	20615	4.6433	7.41	7.4055	33.2558	33.6522
DN	LMO2_2	cluster_4	SP4	2453	4543.42	4444	4652	63.259	0.54	-0.889	0.001	0.0010435	4777	38246	75.086	783	6146	12.066	306	2453	4.8158	6.4057	6.4137	39.0805	39.9121
SP8	TCF7L1_1	cluster_3	DN	12674	23509.116	23042	23943	283.872	0.5391	-0.8913	0.001	0.0010435	9778	157054	56.06	2782	42859	15.298	799	12674	4.524	8.1714	8.0698	28.7203	29.5714
DN	TCF3_1	cluster_4	SP4	6946	12887.788	12391	13369	285.5868	0.539	-0.8917	0.001	0.0011429	20600	290144	23.465	4178	56573	4.5754	502	6946	0.56176	2.4369	2.394	12.0153	12.2779
DN	RUNX1_4	cluster_4	SP4	3750	6959.979	6794	7103	94.4941	0.5389	-0.892	0.001	0.0010909	3683	37261	73.637	998	9723	19.215	377	3750	7.4109	10.2362	10.0641	37.7756	38.5683
DN	TCF7L1_2	cluster_4	SP4	14775	27434.016	27116	27838	213.2719	0.5386	-0.8928	0.001	0.0010435	14657	161969	71.069	3643	39382	17.28	1342	14775	6.483	9.156	9.1221	36.8378	37.5171
DP	EWSR1::FLI1_1	cluster_4	SP4	49214	91464.526	90234	92528	690.2724	0.5381	-0.8941	0.001	0.0010435	43660	868688	53.282	9280	176015	10.796	2516	49214	3.0186	5.7627	5.6653	27.1121	27.9601
DN	FOXO4_3	cluster_4	SP4	2765	5139.868	4962	5288	96.6527	0.538	-0.8942	0.001	0.0010909	2636	34308	73.038	581	7296	15.532	215	2765	5.8864	8.1563	8.0593	37.0052	37.8975
DN	RUNX2_5	cluster_4	SP4	10230	19020	18707	19319	188.4115	0.5379	-0.8946	0.001	0.001	23276	408017	80.689	1441	24027	4.7516	593	10230	2.0231	2.5477	2.5072	41.152	42.5771
DP	POU2F2_known10	cluster_4	SP4	3001	5581.013	5375	5767	121.4828	0.5378	-0.8949	0.001	0.0010435	4216	38032	52.25	1251	10899	14.974	339	3001	4.1229	8.0408	7.8907	27.0983	27.5346
DP	CUX2_2	cluster_4	SP4	192	358.272	306	406	29.643	0.5372	-0.8965	0.001	0.0013333	279	2511	51.763	84	716	14.76	22	192	3.9579	7.8853	7.6464	26.1905	26.8156
DN	FOXM1_4	cluster_4	SP4	2993	5572.834	5439	5698	78.0594	0.5372	-0.8966	0.001	0.0010909	5282	52629	74.145	992	7674	10.811	380	2993	4.2166	5.7839	5.687	38.3065	39.0018
DP	CTCF_known1	cluster_1	SP8	37162	69204.34	68291	70134	560.9373	0.537	-0.897	0.001	0.0010435	55720	1030489	59.443	6625	118066	6.8106	2022	37162	2.1437	3.6289	3.6062	30.5208	31.4756
DP	IRF6_1	cluster_1	SP8	1870	3483.83	3292	3662	110.4954	0.5369	-0.8973	0.001	0.0010909	2388	38278	58.421	391	6065	9.2566	116	1870	2.854	4.8576	4.8853	29.6675	30.8326
DP	MYCN_2	cluster_1	SP8	4273	7962.533	7786	8146	111.9908	0.5367	-0.8978	0.001	0.001	17531	158428	65.545	1383	12270	5.0764	475	4273	1.7678	2.7095	2.6971	34.3456	34.8248
DN	SOX4_2	cluster_4	SP4	6411	11952.391	11721	12187	144.3065	0.5364	-0.8986	0.001	0.0010909	5733	86204	75.057	1180	16678	14.521	435	6411	5.582	7.5877	7.437	36.8644	38.4399
SP8	RUNX_2	cluster_3	DN	36577	68228.148	67520	68973	438.8459	0.5361	-0.8994	0.001	0.0010435	19635	276608	53.223	9259	129043	24.83	2580	36577	7.0379	13.1398	13.2234	27.8648	28.3448
DN	POU2F2_known15	cluster_4	SP4	3304	6164.871	6004	6317	93.989	0.536	-0.8997	0.001	0.0010435	3911	43212	75.23	800	8497	14.793	304	3304	5.7521	7.7729	7.646	38	38.8843
DP	KLF7_1	cluster_3	DN	64379	120120.82	119130	121059	614.1577	0.536	-0.8998	0.001	0.001	72055	1166887	65.914	12356	187429	10.587	4108	64379	3.6366	5.7012	5.5172	33.247	34.3485
DP	MYBL1_3	cluster_3	DN	1243	2321.498	2193	2455	79.6867	0.5356	-0.9007	0.001	0.0010909	1344	14803	55.839	400	4270	16.107	113	1243	4.6888	8.4077	8.3969	28.25	29.1101
DN	ARID5B_1	cluster_4	SP4	4620	8626.974	8454	8810	114.3427	0.5356	-0.9008	0.001	0.0010909	5946	77508	76.405	937	11641	11.475	360	4620	4.5542	6.0545	5.9607	38.4205	39.6873
DN	NFATC2_1	cluster_4	SP4	2658	4966.516	4864	5057	56.1106	0.5353	-0.9016	0.001	0.0010909	7464	44784	80.238	1082	6319	11.322	447	2658	4.7622	5.9887	5.9352	41.3124	42.0636
DN	SOX12_1	cluster_4	SP4	4026	7522.259	7323	7702	114.6912	0.5353	-0.9017	0.001	0.0010435	3600	46915	74.344	869	10644	16.867	316	4026	6.3798	8.7778	8.5815	36.3636	37.8241
DP	HIF1A_2	cluster_1	SP8	4675	8736.763	8488	8966	148.6352	0.5351	-0.902	0.001	0.001	13829	185302	69.991	964	12652	4.7788	353	4675	1.7658	2.5526	2.5229	36.6183	36.9507
SP8	RUNX_1	cluster_3	DN	45607	85224.353	84349	86061	533.115	0.5351	-0.902	0.001	0.0010435	23767	334786	52.302	11766	164110	25.638	3226	45607	7.125	13.5734	13.6227	27.418	27.7905
DN	SOX5_1	cluster_4	SP4	2033	3801.298	3672	3900	69.9884	0.5349	-0.9026	0.001	0.0010909	2705	24345	72.35	618	5390	16.018	228	2033	6.0418	8.4288	8.3508	36.8932	37.718
DP	SPZ1_1	cluster_1	SP8	23575	44072.454	43466	44676	372.0218	0.5349	-0.9026	0.001	0.001	49189	720565	65.335	4890	68733	6.2321	1634	23575	2.1376	3.3219	3.2717	33.4151	34.2994
SP4	TCF7L2_known5	cluster_3	DN	15386	28764.687	28274	29345	319.6882	0.5349	-0.9026	0.001	0.0010435	11860	190547	60.832	3117	48148	15.371	963	15386	4.912	8.1197	8.0746	30.8951	31.9556
DP	PBX1_4	cluster_1	SP8	1605	3002.067	2843	3166	102.0522	0.5348	-0.903	0.001	0.0010909	2319	25948	48.177	591	6347	11.784	147	1605	2.9799	6.3389	6.1854	24.8731	25.2875
DN	SOX1_3	cluster_4	SP4	4137	7738.246	7511	7962	128.3594	0.5347	-0.9033	0.001	0.0010909	3794	53161	70.646	855	11384	15.128	298	4137	5.4977	7.8545	7.782	34.8538	36.3405
DP	ETS_known13	cluster_3	DN	10537	19722.434	19386	20069	205.8566	0.5343	-0.9043	0.001	0.001	10314	165505	67.697	1895	29430	12.038	660	10537	4.31	6.3991	6.3666	34.8285	35.8036
DP	ATF3_known5	cluster_4	SP4	7770	14547.259	14205	14889	216.2993	0.5342	-0.9047	0.001	0.001	11264	158203	55.075	1972	26814	9.3348	557	7770	2.705	4.945	4.9114	28.2454	28.9774
DN	E2F7_1	cluster_4	SP4	3464	6489.457	6335	6641	94.9292	0.5339	-0.9055	0.001	0.0010435	5640	73401	81.261	669	8269	9.1544	273	3464	3.8349	4.8404	4.7193	40.8072	41.8914
DP	SP100_1	cluster_3	DN	756	1417.499	1328	1511	55.9307	0.5337	-0.906	0.001	0.0013333	864	11233	66.811	181	2174	12.93	61	756	4.4965	7.0602	6.7302	33.7017	34.7746
DN	FOXP3_1	cluster_4	SP4	10764	20172.222	19795	20515	211.5265	0.5336	-0.9061	0.001	0.0010909	9966	159936	72.213	1889	28677	12.948	682	10764	4.8601	6.8433	6.7302	36.1038	37.5353
DN	SOX2_6	cluster_4	SP4	3318	6220.202	6026	6421	119.6321	0.5335	-0.9064	0.001	0.0010435	2362	37807	70.103	627	9364	17.363	211	3318	6.1523	8.9331	8.7762	33.6523	35.4336
SP4	GATA_known16	cluster_3	DN	9856	18476.775	18097	18902	255.289	0.5335	-0.9066	0.001	0.0010435	5122	107965	62.558	1533	30496	17.67	471	9856	5.7108	9.1956	9.1289	30.7241	32.319
DP	HIF1A::ARNT_1	cluster_1	SP8	1961	3677.468	3561	3791	69.9558	0.5334	-0.9068	0.001	0.001	10494	75476	67.161	775	5516	4.9083	279	1961	1.745	2.6587	2.5982	36	35.5511
SP4	SOX30_1	cluster_3	DN	4548	8529.674	8280	8800	164.5031	0.5333	-0.9071	0.001	0.0010435	3742	56229	63.32	987	14037	15.807	310	4548	5.1215	8.2843	8.0884	31.4083	32.4001
DP	HES1_1	cluster_1	SP8	11564	21703.701	21283	22166	270.0065	0.5331	-0.9076	0.001	0.001	24843	352037	63.079	2546	34903	6.2541	819	11564	2.073	3.2967	3.2863	32.1681	33.1461
DN	EGR3_2	cluster_4	SP4	5588	10485.321	10050	10888	255.3511	0.533	-0.9078	0.001	0.001	13370	205199	20.858	3669	53073	5.3948	368	5588	0.56801	2.7524	2.7232	10.03	10.5289
DP	RUNX2_5	cluster_3	DN	27735	52042.625	51443	52705	383.3419	0.5329	-0.908	0.001	0.001	17640	310092	61.324	5062	86023	17.012	1597	27735	5.4849	9.0533	8.9441	31.5488	32.2414
DN	FOXO4_2	cluster_4	SP4	7014	13163.676	12902	13396	157.4603	0.5329	-0.9082	0.001	0.0010435	7348	96298	75.255	1470	18241	14.255	547	7014	5.4813	7.4442	7.2836	37.2109	38.4518
DP	KLF13_1	cluster_3	DN	21436	40250.546	39628	40845	380.1712	0.5326	-0.9089	0.001	0.001	19895	342302	61.998	4015	66134	11.978	1270	21436	3.8825	6.3835	6.2623	31.6314	32.413
SP8	TCF7_1	cluster_3	DN	13803	25927.31	25516	26356	258.3163	0.5324	-0.9094	0.001	0.0010435	10694	171757	56.165	3079	47321	15.474	869	13803	4.5136	8.1261	8.0364	28.2234	29.1689
DP	NFKB_known10	cluster_1	SP8	6089	11439.367	11135	11719	167.4332	0.5323	-0.9096	0.001	0.001	10029	121099	60.099	1617	19222	9.5395	501	6089	3.0218	4.9955	5.0281	30.9833	31.6772
DP	HIF1A_1	cluster_3	DN	7950	14938.148	14679	15211	172.3069	0.5322	-0.9099	0.001	0.0010435	11588	130466	68.094	2009	22190	11.582	711	7950	4.1493	6.1357	6.0935	35.3907	35.8269
DP	MYCN_2	cluster_3	DN	11114	20894.332	20620	21183	175.8346	0.5319	-0.9107	0.001	0.001	17531	158428	65.545	3625	32109	13.284	1236	11114	4.5981	7.0504	7.0164	34.0966	34.6133
DN	FOXO6_1	cluster_4	SP4	3271	6152.846	5960	6333	111.0894	0.5317	-0.9113	0.001	0.0010909	3202	41765	72.845	700	8761	15.281	255	3271	5.7052	7.9638	7.8319	36.4286	37.3359
SP4	RUNX_1	cluster_3	DN	43603	82028.435	81180	83001	543.4482	0.5316	-0.9117	0.001	0.0010435	22871	322027	50.309	11766	164110	25.638	3082	43603	6.8119	13.4756	13.5402	26.1941	26.5694
DN	MYBL2_2	cluster_4	SP4	1893	3562.83	3449	3658	66.2491	0.5315	-0.912	0.001	0.0010435	2843	43039	79.915	325	4617	8.5729	127	1893	3.5149	4.4671	4.3983	39.0769	41.0006
DP	SP1_known3	cluster_1	SP8	17510	32953.608	32455	33417	303.1608	0.5314	-0.9122	0.001	0.001	39534	518521	65.051	4032	51385	6.4465	1349	17510	2.1967	3.4123	3.3769	33.4573	34.0761
DN	SPIC_1	cluster_4	SP4	18568	34948.426	34536	35323	247.6284	0.5313	-0.9124	0.001	0.001	19310	251988	71.563	3926	49711	14.118	1428	18568	5.2732	7.3951	7.3686	36.3729	37.3519
DN	SOX9_6	cluster_4	SP4	6368	11995.153	11713	12302	171.5205	0.5309	-0.9134	0.001	0.0010909	5272	79251	71.777	1220	17382	15.743	429	6368	5.7674	8.1373	8.0352	35.1639	36.6356
DP	MAF_known11	cluster_4	SP4	8357	15759.978	15377	16193	245.6788	0.5303	-0.9151	0.001	0.0010435	10294	160060	54.212	1919	29572	9.654	523	8357	2.7282	5.0806	5.0325	27.2538	28.2598
DN	RBPJ_1	cluster_4	SP4	4992	9415.186	9264	9559	89.4707	0.5303	-0.9152	0.001	0.0010435	16019	112173	78.007	1817	12285	8.5432	722	4992	3.4715	4.5071	4.4503	39.7358	40.6349
DN	SOX15_4	cluster_4	SP4	3126	5897.212	5722	6081	109.0194	0.5302	-0.9155	0.001	0.0011429	2731	38267	69.358	676	8954	16.229	229	3126	5.6658	8.3852	8.1689	33.8757	34.9118
DN	SOX17_5	cluster_4	SP4	3307	6238.627	6058	6435	116.0863	0.5302	-0.9155	0.001	0.0011429	2792	39191	71.088	691	9164	16.623	240	3307	5.9985	8.596	8.4382	34.7323	36.0869
DN	SOX7_4	cluster_4	SP4	4399	8298.817	8083	8529	131.7449	0.5301	-0.9156	0.001	0.0010909	3341	53567	71.584	802	12131	16.211	279	4399	5.8786	8.3508	8.2121	34.788	36.2625
DN	SOX21_2	cluster_4	SP4	4685	8839.706	8637	9055	134.671	0.53	-0.9158	0.001	0.0010909	4666	65357	73.59	940	12505	14.08	340	4685	5.2752	7.2868	7.1683	36.1702	37.465
SP4	RUNX1_4	cluster_3	DN	3001	5667.664	5487	5827	107.1048	0.5296	-0.9171	0.001	0.0010435	3165	32119	63.475	931	9119	18.021	295	3001	5.9307	9.3207	9.3434	31.6864	32.9093
DP	IKZF3_1	cluster_4	SP4	3306	6244.557	6038	6443	129.193	0.5295	-0.9173	0.001	0.0010435	6241	75044	55.627	1001	11533	8.5489	276	3306	2.4506	4.4224	4.4054	27.5724	28.6656
SP8	TCF7L2_known5	cluster_3	DN	13886	26241.033	25767	26763	312.318	0.5292	-0.9181	0.001	0.0010435	10823	173781	55.479	3117	48148	15.371	877	13886	4.4331	8.1031	7.9905	28.136	28.8402
DN	SOX10_4	cluster_4	SP4	7631	14423.488	14119	14717	185.8593	0.5291	-0.9184	0.001	0.0010909	6860	103161	73.858	1406	20226	14.481	513	7631	5.4634	7.4781	7.3972	36.4865	37.7287
DN	TBX15_1	cluster_4	SP4	8252	15602.079	15269	15917	198.6297	0.5289	-0.9188	0.001	0.0010909	7884	142057	75.135	1270	21552	11.399	466	8252	4.3646	5.9107	5.8089	36.6929	38.2888
SP8	TCF3_2	cluster_3	DN	47543	89905.978	88949	90846	551.6618	0.5288	-0.9192	0.001	0.0010435	23089	348566	45.448	13375	199427	26.003	3157	47543	6.199	13.6732	13.6396	23.6037	23.8398
DP	CUX1_9	cluster_4	SP4	703	1330.521	1223	1437	69.3855	0.5287	-0.9194	0.001	0.0012632	440	7588	51.249	174	2765	18.675	43	703	4.7481	9.7727	9.2646	24.7126	25.425
DN	SOX5_3	cluster_4	SP4	3569	6754.577	6562	6956	118.9546	0.5285	-0.9202	0.001	0.0011429	2922	43910	74.555	690	9567	16.244	244	3569	6.0598	8.3504	8.128	35.3623	37.3053
DN	MYC_known13	cluster_4	SP4	5134	9719.071	9538	9906	115.4268	0.5283	-0.9206	0.001	0.0010909	12241	134783	75.676	1221	13066	7.3361	467	5134	2.8826	3.815	3.8091	38.2473	39.2928
DP	IKZF1_1	cluster_4	SP4	3972	7521.313	7307	7723	136.1943	0.5282	-0.9209	0.001	0.0010435	8537	102703	58.812	1153	13125	7.5159	338	3972	2.2745	3.9592	3.8675	29.3148	30.2629
DN	SOX9_4	cluster_4	SP4	7260	13746.782	13466	14001	158.9893	0.5282	-0.921	0.001	0.0010909	6382	95858	74.138	1338	19227	14.871	488	7260	5.615	7.6465	7.5737	36.4723	37.7594
DN	SPDEF_4	cluster_4	SP4	10990	20811.48	20438	21165	221.2335	0.5281	-0.9211	0.001	0.0010435	8815	132735	73.627	2070	29469	16.346	742	10990	6.096	8.4175	8.2797	35.8454	37.2934
SP8	TCF7_2	cluster_3	DN	10274	19460.201	19105	19826	220.0525	0.528	-0.9215	0.001	0.0010435	11953	131925	55.759	3302	35484	14.996	938	10274	4.3419	7.8474	7.7878	28.407	28.9539
DP	SP1_known2	cluster_3	DN	81523	154580.311	153575	155417	585.0499	0.5274	-0.9231	0.001	0.001	115319	1604370	70.792	17436	225537	9.9517	6189	81523	3.5972	5.3669	5.0813	35.4955	36.1462

SP4	TCF7_1	cluster_3	DN	15073	28601.661	28126	29065	286.8887	0.527	-0.9241	0.001	0.0010435	11748	188705	61.707	3079	47321	15.474	941	15073	4.9289	8.0099	7.9876	30.5619	31.8527
DN	IKZF1_2	cluster_4	SP4	9484	18024.469	17824	18220	118.5378	0.5262	-0.9263	0.001	0.0010435	27213	217711	79.439	2975	23108	8.4318	1198	9484	3.4606	4.4023	4.3562	40.2689	41.0421
SP4	TCF7L1_1	cluster_3	DN	13649	25941.488	25490	26387	269.8264	0.5262	-0.9264	0.001	0.0010435	10758	172842	61.696	2782	42859	15.298	854	13649	4.872	7.9383	7.8968	30.6973	31.8463
DN	TBX1_2	cluster_4	SP4	6806	12939.977	12627	13260	187.7933	0.526	-0.9269	0.001	0.0010909	6383	115007	73.738	1076	18201	11.67	385	6806	4.3637	6.0316	5.9179	35.7807	37.3935
DN	TCF21_1	cluster_4	SP4	7182	13666.82	13417	13885	132.4953	0.5255	-0.9281	0.001	0.001	16797	218881	77.73	1441	17927	6.3663	558	7182	2.5505	3.322	3.2812	38.7231	40.0625
DN	ETV6_1	cluster_4	SP4	22321	42496.409	42027	42904	272.4265	0.5253	-0.9289	0.001	0.001	32656	469723	77.86	3975	55382	9.18	1567	22321	3.6999	4.7985	4.7519	39.4214	40.3037
DN	FLI1_4	cluster_4	SP4	5306	10102.251	9857	10317	127.4306	0.5253	-0.9289	0.001	0.001	8592	111995	76.37	1063	13399	9.1369	409	5306	3.6182	4.7602	4.7377	38.476	39.6
DN	NFATC1_1	cluster_4	SP4	8036	15299.846	15010	15584	175.4014	0.5253	-0.9289	0.001	0.0010909	6650	128410	79.943	1121	20131	12.533	429	8036	5.0029	6.4511	6.2581	38.2694	39.9185
SP4	TCF3_2	cluster_3	DN	47952	91333.512	90298	92226	603.7022	0.525	-0.9295	0.001	0.0010435	23468	354325	46.199	13375	199427	26.003	3178	47952	6.2523	13.5418	13.5333	23.7607	24.0449
DP	MTF1_1	cluster_4	SP4	15565	29657.357	29224	30119	260.948	0.5248	-0.93	0.001	0.001	40601	541813	68.67	3421	43884	5.5619	1179	15565	1.9727	2.9039	2.8728	34.4636	35.4685
DP	SP4_1	cluster_3	DN	73172	139456.295	138447	140488	622.1341	0.5247	-0.9304	0.001	0.001	81673	1469721	70.891	12286	203749	9.8278	4276	73172	3.5294	5.2355	4.9786	34.8038	35.9128
DP	EGR4_1	cluster_3	DN	17919	34152.555	33713	34562	253.3164	0.5247	-0.9305	0.001	0.001	29511	334771	66.296	4776	52354	10.368	1598	17919	3.5486	5.4149	5.3526	33.459	34.2266
DN	POU2F2_known16	cluster_4	SP4	3074	5862.274	5682	6062	115.5167	0.5245	-0.9311	0.001	0.0010909	3024	39849	67.778	737	9083	15.449	238	3074	5.2285	7.8704	7.7141	32.2931	33.8434
DN	ERG_2	cluster_4	SP4	5409	10318.246	10088	10553	137.3831	0.5243	-0.9316	0.001	0.001	8341	108611	75.068	1107	13976	9.6597	419	5409	3.7385	5.0234	4.9802	37.85	38.7021
SP8	TCF7L1_2	cluster_3	DN	10057	19184.529	18812	19570	231.5153	0.5242	-0.9317	0.001	0.0010435	11685	129185	56.684	3198	34366	15.079	917	10057	4.4128	7.8477	7.785	28.6742	29.2644
DN	E2F_known24	cluster_4	SP4	2733	5217.85	5043	5366	98.7976	0.5239	-0.9327	0.001	0.001	3603	61420	79.657	431	6870	8.9098	164	2733	3.5445	4.5518	4.4497	38.051	39.7817
DN	MGA_3	cluster_4	SP4	5947	11356.636	11111	11589	141.8976	0.5237	-0.9332	0.001	0.0010909	6415	109267	77.12	917	15111	10.665	352	5947	4.1974	5.4871	5.4426	38.386	39.3554
DN	FOXP1_1	cluster_4	SP4	16987	32439.208	31763	33163	419.0148	0.5237	-0.9333	0.001	0.0010435	10678	252594	65.084	3085	58974	15.195	803	16987	4.3769	7.5201	6.725	26.0292	28.8042
DP	ATF3_known4	cluster_3	DN	7515	14352.417	14065	14648	190.734	0.5236	-0.9334	0.001	0.001	8408	118694	67.508	1578	21515	12.237	539	7515	4.2742	6.4106	6.3314	34.1572	34.9291
SP8	FOXP3_1	cluster_3	DN	10646	20337.05	19869	20782	275.7993	0.5235	-0.9337	0.001	0.0010909	7647	122767	55.431	2429	37397	16.885	666	10646	4.8068	8.7093	8.6717	27.4187	28.4675
DN	TBX2_2	cluster_4	SP4	7048	13467.294	13237	13702	142.1471	0.5234	-0.9341	0.001	0.0010909	11786	118013	74.082	1911	18576	11.661	708	7048	4.4243	6.0071	5.9722	37.0487	37.9414
DP	MYCN_1	cluster_4	SP4	3937	7527.916	7327	7753	124.0852	0.5231	-0.935	0.001	0.001	10303	113712	63.225	1121	12081	6.7171	358	3937	2.189	3.4747	3.4623	31.9358	32.5884
DN	IRF6_1	cluster_4	SP4	2942	5626.61	5465	5780	98.6688	0.523	-0.9352	0.001	0.0010909	3319	53207	81.206	466	7185	10.966	185	2942	4.4902	5.574	5.5293	39.6996	40.9464
DN	E2F8_1	cluster_4	SP4	4333	8287.081	8134	8436	92.1604	0.5229	-0.9353	0.001	0.001	9501	104773	81.344	989	10443	8.1077	400	4333	3.3641	4.2101	4.1356	40.4449	41.4919
SP4	MAFG_1	cluster_3	DN	11291	21603.732	21084	22098	292.9616	0.5227	-0.936	0.001	0.0010435	6447	129183	56.562	2020	38737	16.961	568	11291	4.9437	8.8103	8.7403	28.1188	29.1478
SP4	TCF7_2	cluster_3	DN	11293	21614.96	21235	21915	211.5013	0.5225	-0.9365	0.001	0.0010435	13256	146350	61.849	3302	35484	14.996	1030	11293	4.7725	7.7701	7.7164	31.1932	31.8256
DN	SPIB_1	cluster_4	SP4	9755	18687.886	18477	18933	133.0822	0.522	-0.9378	0.001	0.0010435	22113	132678	71.137	4466	26536	14.228	1631	9755	5.2303	7.3758	7.3524	36.5204	36.7614
DN	E2F2_3	cluster_4	SP4	1827	3501.133	3369	3626	77.1503	0.522	-0.938	0.001	0.001	2541	43236	81.281	283	4492	8.4447	110	1827	3.4347	4.329	4.2256	38.8693	40.6723
SP8	RUNX1_4	cluster_3	DN	2592	4968.643	4778	5142	112.4477	0.5218	-0.9385	0.001	0.0010909	2766	28019	55.372	931	9119	18.021	261	2592	5.1224	9.436	9.2509	28.0344	28.4242
DP	HIF1A_2	cluster_3	DN	10731	20572.849	20238	20897	203.8812	0.5216	-0.9389	0.001	0.001	13829	185302	69.991	2276	29792	11.253	807	10731	4.0532	5.8356	5.7911	35.4569	36.0197
DN	SOX8_2	cluster_4	SP4	7174	13757.31	13474	14029	162.0538	0.5215	-0.9393	0.001	0.0010909	6301	94659	74.032	1344	19312	15.104	482	7174	5.6107	7.6496	7.5788	35.8631	37.1479
DP	RUNX3_3	cluster_3	DN	33083	63480.442	62832	64241	436.7745	0.5212	-0.9402	0.001	0.001	23341	416133	63.949	5889	100815	15.493	1890	33083	5.084	8.0973	7.9501	32.0937	32.8156
SP8	TCF7L2_known7	cluster_3	DN	12144	23313.624	22934	23746	266.7575	0.5209	-0.9409	0.001	0.0010435	11335	159258	55.926	3148	42486	14.92	874	12144	4.2646	7.7106	7.6254	27.7637	28.5835
DP	SP1_known2	cluster_1	SP8	44784	85981.561	85285	86594	409.1204	0.5209	-0.941	0.001	0.001	115319	1604370	70.792	9541	124977	5.5146	3327	44784	1.9761	2.885	2.7914	34.8706	35.8338
DP	E2F2_3	cluster_4	SP4	1385	2660.861	2495	2824	98.6765	0.5207	-0.9415	0.001	0.001	1923	32730	61.531	283	4492	8.4447	83	1385	2.6037	4.3162	4.2316	29.3286	30.8326
DN	MAF_known10	cluster_4	SP4	7422	14258.643	14020	14484	143.5695	0.5206	-0.9419	0.001	0.0010909	11107	122400	74.849	1819	19475	11.909	679	7422	4.5386	6.1133	6.0637	37.3282	38.1104
DN	HMGA1_2	cluster_4	SP4	6961	13387.857	13115	13626	148.0347	0.52	-0.9435	0.001	0.0010435	6789	95209	77.457	1373	18136	14.755	507	6961	5.6631	7.468	7.3113	36.9264	38.3822
SP8	MAFF_1	cluster_3	DN	11655	22426.052	21986	22865	274.6341	0.5197	-0.9442	0.001	0.001	9420	132144	52.834	3131	42825	17.122	835	11655	4.6599	8.8641	8.8199	26.6688	27.2154
SP4	ETV3_1	cluster_3	DN	6813	13119.811	12906	13349	132.5034	0.5193	-0.9453	0.001	0.0010435	14073	127252	67.378	2201	19610	10.383	757	6813	3.6074	5.3791	5.3539	34.3935	34.7425
DN	SOX14_3	cluster_4	SP4	4258	8202.533	7945	8434	140.2072	0.5192	-0.9457	0.001	0.0010909	4092	57347	70.134	902	12080	14.774	307	4258	5.2074	7.5024	7.425	34.0355	35.2483
DP	RUNX3_3	cluster_4	SP4	8929	17199.7	16788	17651	254.6782	0.5192	-0.9457	0.001	0.001	23341	416133	63.949	1632	27443	4.2173	512	8929	1.3721	2.1936	2.1457	31.3725	32.5365
DP	RUNX2_5	cluster_1	SP8	11746	22647.886	22222	23084	274.6634	0.5187	-0.9471	0.001	0.001	17640	310092	61.324	2260	37691	7.4537	685	11746	2.3229	3.8832	3.7879	30.3097	31.1639
SP4	RUNX_2	cluster_3	DN	33811	65190.11	64446	65904	438.6212	0.5187	-0.9471	0.001	0.0010435	18786	264509	50.895	9259	129043	24.83	2388	33811	6.5057	12.7116	12.7826	25.7911	26.2013
DN	MAF_known12	cluster_4	SP4	9041	17436.132	17144	17765	177.9577	0.5185	-0.9474	0.001	0.0010435	9989	170271	77.196	1414	23154	10.497	536	9041	4.0989	5.3659	5.3098	37.9066	39.0472
DN	PKNOX1_2	cluster_4	SP4	8267	15943.145	15678	16182	158.6928	0.5186	-0.9474	0.001	0.0010435	13772	152898	72.377	2075	22398	10.602	740	8267	3.9133	5.3732	5.4069	35.6627	36.9095
DN	TCF3_2	cluster_4	SP4	14385	27740.324	27397	28057	209.8252	0.5186	-0.9474	0.001	0.0010435	39118	592154	77.209	2555	36581	4.7697	961	14385	1.8756	2.4567	2.4293	37.6125	39.3237
DP	NFKB_known11	cluster_1	SP8	7217	13917.952	13593	14220	194.136	0.5186	-0.9474	0.001	0.001	11257	136572	59.553	1970	23567	10.277	594	7217	3.147	5.2767	5.2844	30.1523	30.6233
SP4	TCF7L2_known7	cluster_3	DN	13398	25840.636	25377	26273	268.4329	0.5185	-0.9476	0.001	0.0010435	12562	176542	61.995	3148	42486	14.92	962	13398	4.7049	7.658	7.5891	30.5591	31.5351
DP	SOX5_3	cluster_1	SP8	1853	3576.031	3383	3765	113.7818	0.5183	-0.9481	0.001	0.0011429	2038	30641	52.026	516	7140	12.123	127	1853	3.1462	6.2316	6.0475	24.6124	25.9524
DN	SOX1_2	cluster_4	SP4	4099	7911.38	7727	8107	118.0034	0.5182	-0.9485	0.001	0.0010909	4151	58133	72.545	855	11323	14.13	297	4099	5.1152	7.1549	7.0511	34.7368	36.2007
DP	E2F_known26	cluster_3	DN	1754	3387.38	3216	3554	111.0886	0.5179	-0.9491	0.001	0.0010435	1515	25773	59.594	379	5910	13.665	109	1754	4.0557	7.1947	6.8056	28.7599	29.6785
DN	PKNOX2_2	cluster_4	SP4	9226	17813.966	17528	18064	162.7028	0.5179	-0.9492	0.001	0.0010435	15871	176400	72.498	2309	24959	10.258	827	9226	3.7918	5.2108	5.2302	35.8164	36.9646
DN	XBP1_2	cluster_4	SP4	4011	7746.525	7490	8003	153.6036	0.5178	-0.9494	0.001	0.0010435	3231	74429	77.172	478	10402	10.785	176	4011	4.1588	5.4472	5.389	36.8201	38.5599
SP8	PKNOX2_2	cluster_3	DN	12865	24843.851	24452	25291	249.8111	0.5179	-0.9494	0.001	0.0010435	10799	119886	49.272	4632	50861	20.903	1161	12865	5.2874	10.751	10.731	25.0648	25.2944
DN	MAF_known1	cluster_4	SP4	26560	51307.069	50755	51769	314.3638	0.5177	-0.9499	0.001	0.001	33530	506311	73.776	4884	71057	10.354	1768	26560	3.8701	5.2729	5.2458	36.1998	37.3784
DP	SP1_known3	cluster_4	SP4	16875	32611.671	32141	33086	288.1817	0.5175	-0.9505	0.001	0.001	39534	518521	65.051	4045	50952	6.3922	1301	16875	2.117	3.2908	3.2544	32.1632	33.1194
DP	ATF2_1	cluster_3	DN	539	1042.737	985	1099	35.5409	0.5174	-0.9507	0.001	0.0010435	1296	9072	66.75	228	1585	11.662	77	539	3.9659	5.9414	5.9414	33.7719	34.0063
SP8	TBX21_4	cluster_3	DN	10014	19356.948	18921	19781	245.1492	0.5174	-0.9508	0.001	0.0010909	7628	114668	56.477	2406	34959	17.218	671	10014	4.9321	8.7965	8.733	27.8886	28.645
DP	MYC_known20	cluster_4	SP4	5273	10199.682	9859	10525	190.0128	0.517	-0.9517	0.001	0.001	8735	143000	56.723	1173	18285	7.253	328	5273	2.0916	3.755	3.6874	27.9625	28.8378
SP4	TCF4_1	cluster_3	DN	39961	77291.816	76580	77998	435.1988	0.517	-0.9517	0.001	0.0010909	33174	299877	45.732	18932	169953	25.918	4423	39961	6.0941	13.3327	13.3258	23.3626	23.513
DN	RUNX3_3	cluster_4	SP4	11275	21828.467	21509	22171	199.2569	0.5165	-0.953	0.001	0.001	29706	527830	81.113	1632	27443	4.2173	649	11275	1.7327	2.1847	2.1361	39.7672	41.0852
SP8	SOX21_3	cluster_3	DN	4352	8426.907	8167	8659	155.4774	0.5165	-0.9532	0.001	0.0010909	3510	52680	59.82	1020	14522	16.49	294	4352	4.9419	8.3761	8.2612	28.8235	29.9683
SP8	MAFG_1	cluster_3	DN	10416	20169.974	19671	20637	307.3233	0.5164	-0.9533	0.001	0.0010435	6025	120737	52.864	2020	38737	16.961	526	10416	4.5606	8.7303	8.627	26.0396	26.889
DP	NFATC2_1	cluster_1	SP8	1434	2778.042	2673	2882	65.2231	0.5164	-0.9535	0.001	0.0010909	5104	30624	54.868	877	5163	9.2504	241	1434	2.5692	4.7218	4.6826	27.48	27.7745
DN	FLI1_2	cluster_4	SP4	4800	9299.427	9082	9511	131.1957	0.5162	-0.954	0.001	0.001	8020	104443	74.293	1001	12687	9.0246	371	4800	3.4144	4.6259	4.5958	37.0629	37.834
SP4	SMAD3_1	cluster_3	DN	14370	27846.839	27496	28170	206.333	0.5161	-0.9544	0.001	0.0010435	20754	171916	58.449	5906	48192	16.385	1738	14370	4.8856	8.3743	8.3587	29.4277	29.8182
DN	TBX2_1	cluster_4	SP4	8757	16974.67	16704	17282	177.3871	0.5159	-0.9548	0.001	0.0010435	11067	188839	77.429	1365	22454	9.2067	518	8757	3.5906	4.6806	4.6373	37.9487	38.9997
DN	ID4_1	cluster_4	SP4	10929	21191.388	20936	21425	148.2211	0.5158	-0.9553	0.001	0.0010435	48845	442152	75.691	3214	28330	4.8498	1213	10929	1.8709	2.4834	2.4718	37.7411	38.5775
DN	SOX3_1	cluster_4	SP4	3170	6147.661	5940	6322	117.3771	0.5157	-0.9553	0.001	0.0010909	2538	40630	73.556	600	8890	16.094	207	3170	5.7389	8.156	7.8021	34.5	35.658
DN	ATF3_known4	cluster_4	SP4	3903	7569.143	7381	7765	112.2316	0.5157	-0.9554	0.001	0.001	10011	141182	80.298	717	9606	5.4635	284	3903	2.2199	2.8369	2.7645	39.6095	40.6309
DP	SOX7_4	cluster_1	SP8	1960	3802.941	3577	4007	132.2949	0.5155	-0.9559	0.001	0.0010909	2231	35768	47.798	551	8220	10.985	124	1960	2.6192	5.558	5.4798	22.5045	23.8443
SP8	TBX20_5	cluster_3	DN	16300	31629.134	31170	32107	296.8704	0.5154	-0.9563	0.001	0.0010909	12908	197715	58.58	3760	55218	16.36	1078	16300	4.8294	8.3514	8.2442	28.6702	29.5194
DP	ARID5A_1	cluster_1	SP8	569	1105.229	1007	1203	59.8817	0.5153	-0.9566	0.001	0.0012632	647	8899	52.495	182	2224	13.119	44	569	3.3565	6.8006	6.394	24.1758	25.5845
SP4	MAFF_1	cluster_3	DN	12181	23651.124	23215	24032	278.4078	0.515	-0.9572	0.001	0.001	9927	139260	55.679	3131	42825	17.122	869	12181	4.8702	8.7539	8.7469	27.7547	28.4437
DP	SP4_2	cluster_1	SP8	31143	60489.871	59765	61210	414.624	0.5149	-0.9578	0.001	0.001	56997	957954	67.242	5789	92205	6.4721	1892	31143	2.186	3.3195	3.251	32.6827	33.7758
DN	MGA_2	cluster_4	SP4	6351	12341.859	12095	12616	160.4379	0.5146	-0.9584	0.001	0.0010909	6706	114146	75.028	1040	16927	11.126	376	6351	4.1745	5.6069	5.5639	36.1538	37.5199
DN	SOX11_1	cluster_4	SP4	16912	32864.391	32398	33317	278.5604	0.5146	-0.9584	0.001	0.0010435	13495	218540	74.839	3012	45946	15.734	1068	16912	5.7915	7.914	7.7386	35.4582	36.8084
DN	SOX10_9	cluster_4	SP4	4828	9387.326	9170	9606	132.576	0.5144	-0.9591	0.001	0.0010435	4222	59134	70.343	1036	13825	16.446	348	4828	5.7432	8.2425	8.1645	33.5907	34.9222
SP4	MAF_known11	cluster_3	DN	14397	28001.936	27477	28441	285.0589	0.5142	-0.9597	0.001	0.0010435	10831	174705	57.034	3193	49735	16.236	899	14397	4.7	8.3002	8.2407	28.1553	28.9474
SP4	TCF7L1_2	cluster_3	DN	10718	20847.543	20506	21184	200.9642	0.5141	-0.9598	0.001	0.0010435	12702	140496	61.647	3198	34366	15.079	975	10718	4.7028	7.676	7.6287	30.4878	31.1878
DN	TBX19_1	cluster_4	SP4	5699	11087.079	10778	11391	181.7452	0.5141	-0.96	0.001	0.0010435	4779	91001	71.181	888	16009	12.522	302	5699	4.4577	6.3193	6.2626	34.009	35.5987
DP	CUX1_8	cluster_1	SP8	444	864.984	749	973	64.5312	0.5139	-0.9605	0.001	0.0011429	409	6571	49.473	122	1836	13.823	30	444	3.3429	7.335	6.757	24.5902	24.183
DP	SOX17_5	cluster_1	SP8	1583	3081.682	2892	3267	109.9344	0.5138	-0.9606	0.001	0.0011429	1842	25857	46.902	514	6773	12.286	114	1583	2.8714	6.1889	6.1221	22.179	23.3722
DN	SOX17_4	cluster_4	SP4	3387	6592.731	6399	6773	116.8047	0.5138	-0.9607	0.001	0.0010909	3286	46015	72.668	725	9491	14.988	251	3387	5.3489	7.6385	7.3606	34.6207	35.6864
SP8	TCF4_1	cluster_3	DN	39121	76153.367	75515	76761	393.3736	0.5137	-0.9609	0.001	0.0010909	32686	295493	45.063	18932	169953	25.918	4332	39121	5.966	13.2534	13.2392	22.8819	23.0187
DP	SPDEF_6	cluster_4	SP4	2988	5821.4	5617	5996	116.5366	0.5134	-0.962	0.001	0.001	8667	121736	70.334	625	8420	4.8647	218	2988	1.7263	2.5153	2.4545	34.88	35.4869
DP	KLF14_1	cluster_3	DN	33702	65658.321	65008	66267	386.983	0.5133	-0.9621	0.001	0.001	49087	664659	67.227	7718	99743	10.089	2550	33702	3.4088	5.1949	5.0706	33.0396	33.7888
DN	SOX13_1	cluster_4	SP4	2748	5355.589	5191	5510	98.6283	0.5132	-0.9624	0.001	0.0010909	2495	37512	75.346	555	7626	15.318	190	2748	5.5196	7.6152	7.3257	34.2342	36.0346
DN	POU2F3_2	cluster_4	SP4	2465	4807.268	4693	4930	68.7686	0.5129	-0.9633	0.001	0.0010909	4271	34232	74.798	848	6599	14.419	312	2465	5.3861	7.3051	7.2009	36.7925	37.3541
DP	SMAD4_1	cluster_4	SP4	13301	25940.603	25468	26438	288.0137	0.5128	-0.9636	0.001	0.001	25085	356038	57.788	3269	45282	7.3496	939	13301	2.1589	3.7433	3.7358	28.7244	29.3737
SP8	SMAD3_1	cluster_3	DN	13762	26838.267	26505	27193	234.5547	0.5128	-0.9636	0.001	0.0010435	19958	165967	56.427	5906	48192	16.385	1665	13762	4.6789	8.3425	8.292	28.1917	28.5566
DN	SOX11_2	cluster_4	SP4	4732	9229.955	9000	9442	133.588	0.5127	-0.9637	0.001	0.0010909	4488	62863	74.087	979	13014	15.338	347	4732	5.5769	7.7317	7.5275	35.4443	36.3608
DP	AHR::ARNT::HIF1A_1	cluster_1	SP8	2274	4435.913	4306	4561	76.0291	0.5127	-0.9637	0.001	0.0010435	12707	104472	71.635	761	6233	4.2739	284	2274	1.5592	2.235	2.1767	37.3193	36.4832
DN	PKNOX2_3	cluster_4	SP4	8680	16931.56	16681	17197	159.626	0.5127	-0.9639	0.001	0.0010435	13787	153514	71.649	2218	23981	11.193	777	8680	4.0512	5.6357	5.6542	35.0316	36.1953
DP	HBP1_1	cluster_1	SP8	2859	5580.093	5304	5826	161.4652	0.5124	-0.9645	0.001	0.0010435	3549	54705	50.814	791	11380	10.571	190	2859	2.6557	5.3536	5.2262	24.0202	25.123
SP4	ETV7_1	cluster_3	DN	19287	37643.46	37187	38104	265.146	0.5124	-0.9647	0.001	0.001	27857	251236	56.588	7508	66847	15.056	2140	19287	4.3441	7.6821	7.6768	28.5029	28.8525
DN	IKZF2_2	cluster_4	SP4	2566	5010.679	4881	5140	74.3328	0.5122	-0.9652	0.001	0.0010435	4704	47046	78.526	694	6615	11.041	263	2566	4.283	5.591	5.4542	37.8963	38.7906
DP	KLF13_1	cluster_1	SP8	11885	23212.554	22738	23712	285.1936	0.512	-0.9657	0.001	0.001	19895	342302	61.998	2316	38167	6.9128	698	11885	2.1526	3.5084	3.4721	30.1382	31.1395
DN	GMEB2_2	cluster_1	SP8	1451	2835.507	2720	2957	69.2972	0.5119	-0.9661	0.001	0.001	3215	42707	77.048	297	3755	6.7744	111	1451	2.6178	3.4526	3.3976	37.3737	38.6418
DN	TCF4_2	cluster_4	SP4	11109	21702.77	21412	21919	156.6214	0.5119	-0.9661	0.001	0.001	45335	409696	76.215	3276	28814	5.3602	1239	11109	2.0666	2.733	2.7115	37.8205	38.5542
DN	ETS_known13	cluster_4	SP4	6439	12582.732	12363	12809	142.6439	0.5118	-0.9664	0.001	0.001	12306	197388	80.739	1019	15787	6.4574	406	6439	2.6338	3.2992	3.2621	39.843	40.7867
SP8	PKNOX2_3	cluster_3	DN	11075	21640.305	21246	22001	227.6701	0.5118	-0.9664	0.001	0.0010435	9661	107482	50.165	3958	43496	20.301	997	11075	5.169	10.3198	10.3041	25.1895	25.4621
DN	TBX4_1	cluster_4	SP4	5672	11085.671	10918	11277	104.8601	0.5117	-0.9666	0.001	0.0010435	16022	112186	74.769	2178	15032	10.018	811	5672	3.7802	5.0618	5.0559	37.236	37.7328
DP	ETS_known13	cluster_4	SP4	5405	10564.018	10301	10857	166.4922	0.5117	-0.9667	0.001	0.001	10314	165505	67.697	1019	15787	6.4574	340	5405	2.2108	3.2965	3.2658	33.366	34.237
DP	ETS_known17	cluster_4	SP4	8812	17233.668	16808	17648	256.6371	0.5114	-0.9676	0.001	0.001	12494	213553	60.343	1774	29054	8.2097	519	8812	2.49	4.154	4.1264	29.2559	30.3297
DP	KLF4_1	cluster_1	SP8	27761	54299.338	53793	54801	303.3446	0.5113	-0.9678	0.001	0.001	76804	740702	63.769	9288	86550	7.4513	2923	27761	2.39	3.8058	3.7479	31.4707	32.0751
DP	EGR4_2	cluster_3	DN	39491	77250.6	76405	77997	465.2547	0.5112	-0.968	0.001	0.001	49002	804691	69.973	7390	113656	9.883	2486	39491	3.434	5.0733	4.9076	33.6401	34.7461
SP8	SOX30_1	cluster_3	DN	4159	8139.964	7870	8386	159.1702	0.511	-0.9686	0.001	0.0010435	3553	53360	60.089	987	14037	15.807	281	4159	4.6835	7.9088	7.7942	28.4701	29.6288
DP	RUNX3_3	cluster_1	SP8	14088	27599.116	27107	28090	307.5413	0.5105	-0.9701	0.001	0.001	23341	416133	63.949	2618	44080	6.7739	816	14088	2.1649	3.496	3.3855	31.1688	31.9601
DN	SMAD_2	cluster_4	SP4	15674	30713.351	30379	31036	207.2169	0.5103	-0.9704	0.001	0.0010435	28215	291809	73.323	4228	42666	10.721	1530	15674	3.9384	5.4226	5.3713	36.1873	36.7365
DP	STAT_known2	cluster_3	DN	11231	22011.96	21584	22496	269.8449	0.5102	-0.9707	0.001	0.001	8789	176424	66.894	1770	33621	12.748	568	11231	4.2584	6.4626	6.3659	32.0904	33.4047
SP4	TBX21_4	cluster_3	DN	10207	20007.072	19559	20484	275.8088	0.5102	-0.9709	0.001	0.0010909	7892	118588	58.407	2406	34959	17.218	684	10207	5.0272	8.667	8.6071	28.4289	29.1971
DP	E2F_known24	cluster_4	SP4	2055	4029.705	3795	4232	128.1525	0.5101	-0.9712	0.001	0.001	2757	47016	60.976	431	6870	8.9098	122	2055	2.6652	4.4251	4.3709	28.3063	29.9127
DN	MTF1_3	cluster_4	SP4	9221	18084.268	17810	18362	160.3012	0.5099	-0.9717	0.001	0.001	16298	213937	79.022	1830	23295	8.6045	705	9221	3.406	4.3257	4.3101	38.5246	39.5836
DN	SOX4_1	cluster_4	SP4	17888	35084.729	34651	35601	279.5947	0.5099	-0.9718	0.001	0.0010435	14546	235575	74.812	3207	48978	15.554	1129	17888	5.6807	7.7616	7.5933	35.2042	36.5225
SP4	SOX_1	cluster_3	DN	6818	13374.464	13083	13653	183.4348	0.5098	-0.972	0.001	0.0010435	7156	85938	62.408	1911	21909	15.91	574	6818	4.9512	8.0212	7.9336	30.0366	31.1196
DP	E2F_known26	cluster_1	SP8	1121	2199.985	2041	2351	91.241	0.5098	-0.9721	0.001	0.0010435	1515	25773	59.594	241	3860	8.9253	66	1121	2.592	4.3564	4.3495	27.3859	29.0415
DN	TCF4_1	cluster_4	SP4	10227	20064.434	19809	20315	158.8967	0.5097	-0.9722	0.001	0.0010909	54166	490170	74.751	3083	27113	4.1348	1138	10227	1.5596	2.1009	2.0864	36.9121	37.7199
DN	SOX18_2	cluster_4	SP4	3539	6944.562	6738	7121	120.0909	0.5097	-0.9723	0.001	0.0010909	3405	47699	72.637	766	10021	15.26	261	3539	5.3892	7.6652	7.4194	34.0731	35.3158
DN	SMAD3_1	cluster_4	SP4	11964	23478.789	23191	23760	166.3098	0.5096	-0.9726	0.001	0.0010435	26078	216611	73.645	3964	32413	11.02	1451	11964	4.0676	5.5641	5.5233	36.6044	36.9111
DN	PKNOX1_1	cluster_4	SP4	12544	24620.226	24239	24999	227.4969	0.5095	-0.9728	0.001	0.0010435	15318	230880	74.405	2333	33838	10.905	840	12544	4.0425	5.4837	5.4331	36.0051	37.0707
DN	SOX3_3	cluster_4	SP4	4090	8033.776	7805	8254	146.6753	0.5092	-0.9738	0.001	0.0010909	3414	54700	70.442	791	11919	15.349	260	4090	5.2671	7.6157	7.4771	32.8698	34.315
DN	SOX30_1	cluster_4	SP4	4473	8788.559	8591	9026	124.0344	0.509	-0.9742	0.001	0.0010435	4394	65950	74.266	872	12336	13.892	305	4473	5.037	6.9413	6.7824	34.9771	36.2597
DN	FOXO1_5	cluster_4	SP4	15633	30721.214	30365	31057	203.5628	0.5089	-0.9746	0.001	0.0010435	32721	361528	75.797	3867	41421	8.6842	1427	15633	3.2776	4.3611	4.3241	36.902	37.7417
SP4	SMAD_2	cluster_3	DN	19169	37669.614	37176	38176	291.3647	0.5089	-0.9746	0.001	0.0010435	22080	227820	57.244	6601	66737	16.769	1859	19169	4.8166	8.4194	8.4141	28.1624	28.7232

SP4	PKNOX2_2	cluster_3	DN	13293	26145.03	25709	26569	267.1809	0.5085	-0.9758	0.001	0.0010435	11364	126178	51.858	4632	50861	20.903	1198	13293	5.4633	10.5421	10.5351	25.8636	26.1359
SP4	TCF21_1	cluster_3	DN	17384	34196.495	33709	34719	307.2196	0.5084	-0.976	0.001	0.001	10517	136988	48.648	5540	70941	25.193	1335	17384	6.1735	12.6937	12.6902	24.0975	24.5049
DN	NFE2L1::MAFG_1	cluster_4	SP4	13403	26366.127	25881	26750	262.3905	0.5084	-0.9761	0.001	0.0010435	11526	243206	74.83	1843	36408	11.202	648	13403	4.1239	5.6221	5.511	35.1601	36.8133
SP4	ID4_1	cluster_3	DN	34522	67939.016	67308	68601	388.7336	0.5081	-0.9767	0.001	0.0010435	30226	273311	46.788	16281	146125	25.015	3811	34522	5.9098	12.6084	12.631	23.4077	23.625
SP8	TCF21_1	cluster_3	DN	16797	33062.794	32509	33688	347.6136	0.508	-0.977	0.001	0.001	10166	132387	47.014	5540	70941	25.193	1291	16797	5.965	12.6992	12.6878	23.3032	23.6774
SP4	MAF_known12	cluster_3	DN	11192	22037.682	21581	22471	263.1728	0.5079	-0.9774	0.001	0.0010435	7957	135675	61.511	2214	36416	16.51	659	11192	5.0741	8.282	8.2491	29.7651	30.7337
SP8	GATA_known16	cluster_3	DN	8292	16333.239	15942	16764	249.6562	0.5077	-0.9779	0.001	0.0010435	4509	95037	55.067	1533	30496	17.67	398	8292	4.8046	8.8268	8.725	25.9622	27.1905
DN	SPDEF_6	cluster_4	SP4	3526	6946.122	6796	7094	90.9411	0.5077	-0.978	0.001	0.001	10392	145946	84.321	625	8420	4.8647	255	3526	2.0372	2.4538	2.416	40.8	41.8765
DP	SP4_1	cluster_1	SP8	40469	79729.11	78968	80539	469.8344	0.5076	-0.9783	0.001	0.001	81673	1469721	70.891	6913	116187	5.6042	2322	40469	1.952	2.843	2.7535	33.5889	34.8309
SP8	SOX9_6	cluster_3	DN	4895	9645.621	9383	9944	172.2246	0.5075	-0.9784	0.001	0.0010909	4230	63611	57.612	1215	17231	15.606	332	4895	4.4334	7.8487	7.6952	27.3251	28.4081
SP4	PBX1_2	cluster_3	DN	3329	6563.524	6345	6781	128.673	0.5073	-0.9792	0.001	0.0010435	3037	43176	61.498	821	11039	15.724	241	3329	4.7417	7.9355	7.7103	29.3544	30.1567
DN	TBX20_2	cluster_4	SP4	6980	13762.939	13497	14047	168.7244	0.5072	-0.9794	0.001	0.0010435	7160	129186	74.304	1131	19160	11.02	394	6980	4.0147	5.5028	5.4031	34.8364	36.4301
DP	KLF12_2	cluster_1	SP8	21877	43154.485	42515	43734	353.9335	0.507	-0.9801	0.001	0.001	38298	549311	63.594	5003	69033	7.992	1545	21877	2.5327	4.0342	3.9826	30.8815	31.6906
SP4	TBX5_5	cluster_3	DN	9607	18953.535	18545	19441	267.8821	0.5069	-0.9802	0.001	0.0010435	5992	114030	59.04	1794	32801	16.983	510	9607	4.9741	8.5113	8.425	28.4281	29.2887
SP8	ETV7_1	cluster_3	DN	17419	34363.501	33863	34859	304.0253	0.5069	-0.9802	0.001	0.001	25471	229674	51.731	7508	66847	15.056	1934	17419	3.9234	7.5929	7.5842	25.7592	26.058
SP8	ETV3_1	cluster_3	DN	6435	12697.677	12476	12933	147.1282	0.5068	-0.9804	0.001	0.0010435	13610	123083	65.171	2201	19610	10.383	715	6435	3.4072	5.2535	5.2282	32.4852	32.8149
SP8	MAF_known10	cluster_3	DN	7818	15426.612	15094	15747	194.9344	0.5068	-0.9805	0.001	0.0010909	8275	91208	55.774	2609	28034	17.143	713	7818	4.7808	8.6163	8.5716	27.3285	27.8876
SP4	TBX20_5	cluster_3	DN	16410	32383.015	31835	32892	322.5401	0.5068	-0.9806	0.001	0.0010909	13251	203127	60.183	3760	55218	16.36	1085	16410	4.862	8.1881	8.0787	28.8564	29.7186
DN	SOX7_3	cluster_4	SP4	4804	9485.169	9259	9703	138.2562	0.5065	-0.9813	0.001	0.0010909	4157	66552	73.226	905	13558	14.918	306	4804	5.2857	7.3611	7.2184	33.8122	35.433
DP	MYC_known20	cluster_1	SP8	4683	9247.686	8882	9576	195.3784	0.5065	-0.9815	0.001	0.001	8735	143000	56.723	1049	16496	6.5434	287	4683	1.8576	3.2856	3.2748	27.3594	28.3887
SP8	ID4_1	cluster_3	DN	33752	66658.843	65949	67347	413.8096	0.5063	-0.9818	0.001	0.0010435	29653	268120	45.899	16281	146125	25.015	3729	33752	5.7779	12.5755	12.5884	22.904	23.098
SP4	TBX4_2	cluster_3	DN	11180	22093.015	21608	22574	294.8691	0.5061	-0.9826	0.001	0.0010909	6921	131760	58.982	2070	38122	17.065	589	11180	5.0047	8.5103	8.4851	28.4541	29.3269
SP4	PBX3_known1	cluster_3	DN	6486	12823.899	12530	13118	167.4602	0.5058	-0.9833	0.001	0.0010435	7076	78591	58.439	2087	22368	16.633	594	6486	4.8229	8.3946	8.2529	28.4619	28.9968
DP	BATF_known1	cluster_4	SP4	2560	5065.416	4834	5275	133.2541	0.5055	-0.9843	0.001	0.0010435	3507	45701	52.784	763	9682	11.183	196	2560	2.9568	5.5888	5.6016	25.6881	26.4408
DP	MYBL2_4	cluster_4	SP4	1053	2084.345	1941	2220	81.8719	0.5054	-0.9844	0.001	0.0010435	1563	21943	61.958	254	3426	9.6736	75	1053	2.9732	4.7985	4.7988	29.5276	30.7356
DN	POU2F2_known11	cluster_4	SP4	4195	8301.739	8131	8479	102.1352	0.5054	-0.9846	0.001	0.0010435	5502	55147	73.881	1193	11573	15.504	426	4195	5.6201	7.7426	7.6069	35.7083	36.2482
SP8	MAF_known11	cluster_3	DN	13489	26708.103	26221	27254	314.2993	0.5051	-0.9854	0.001	0.0010435	10315	166406	54.325	3193	49735	16.236	844	13489	4.4036	8.1823	8.1061	26.4328	27.1217
SP4	SPI1_known1	cluster_3	DN	21492	42638.16	42213	43010	254.2879	0.5041	-0.9883	0.001	0.001	38052	267768	57.339	10728	74696	15.995	3052	21492	4.6022	8.0206	8.0264	28.4489	28.7726
DN	TBX15_2	cluster_4	SP4	6741	13374.98	13160	13547	116.2414	0.504	-0.9884	0.001	0.0010909	20505	143570	74.719	2637	18153	9.4475	967	6741	3.5083	4.7159	4.6953	36.6705	37.1344
SP4	MAF_known10	cluster_3	DN	8229	16330.059	15991	16643	201.4094	0.5039	-0.9887	0.001	0.0010909	8761	96521	59.023	2609	28034	17.143	748	8229	5.0321	8.5378	8.5256	28.67	29.3536
DN	FOXO6_3	cluster_4	SP4	15409	30582.407	30219	30927	209.2606	0.5039	-0.9889	0.001	0.0010435	28591	373838	76.349	3237	40909	8.3549	1189	15409	3.147	4.1587	4.1218	36.7315	37.6665
SP8	POU2F2_known11	cluster_3	DN	3554	7056.75	6864	7272	122.9423	0.5037	-0.9894	0.001	0.0010435	4428	44372	59.446	1256	12148	16.275	356	3554	4.7613	8.0397	8.0096	28.3439	29.2558
SP8	TBX1_4	cluster_3	DN	13240	26304.026	25839	26812	308.8276	0.5034	-0.9903	0.001	0.0010909	8594	163899	55.977	2605	47743	16.306	703	13240	4.5219	8.1801	8.0781	26.9866	27.7318
SP8	PKNOX1_2	cluster_3	DN	10863	21591.628	21203	21986	237.5765	0.5031	-0.991	0.001	0.0010435	9433	104620	49.524	4017	43989	20.823	982	10863	5.1422	10.4103	10.3833	24.4461	24.6948
DP	KLF7_1	cluster_1	SP8	39861	79233.222	78444	79966	458.4282	0.5031	-0.9911	0.001	0.001	72055	1166887	65.914	8057	123464	6.9741	2518	39861	2.2516	3.4946	3.416	31.2523	32.2855
SP8	POU2F2_known10	cluster_3	DN	3436	6834.317	6650	7017	108.1968	0.5028	-0.9919	0.001	0.0010435	4821	43499	59.761	1333	11620	15.964	384	3436	4.7206	7.9652	7.899	28.8072	29.5697
SP4	SPI1_known4	cluster_3	DN	16986	33788.375	33326	34322	300.2146	0.5027	-0.9921	0.001	0.0010435	15528	202516	62.03	4360	55279	16.932	1311	16986	5.2028	8.4428	8.3875	30.0688	30.7278
SP4	MGA_2	cluster_3	DN	7245	14423.738	14060	14798	216.6442	0.5023	-0.9933	0.001	0.0010909	5162	87892	57.771	1556	25429	16.714	429	7245	4.7621	8.3107	8.2431	27.5707	28.4911
DN	SOX15_2	cluster_4	SP4	3738	7443.256	7246	7618	113.2185	0.5023	-0.9935	0.001	0.0010909	3740	52487	73.258	809	10650	14.865	275	3738	5.2172	7.3529	7.1218	33.9926	35.0986
SP4	SPIC_2	cluster_3	DN	18237	36329.142	35813	36827	294.9983	0.502	-0.9942	0.001	0.0010435	17268	225108	60.689	4773	60618	16.343	1406	18237	4.9167	8.1422	8.1014	29.4574	30.0851
SP8	SOX2_6	cluster_3	DN	2337	4656.68	4430	4831	120.5379	0.502	-0.9943	0.001	0.0010435	1941	31108	57.681	562	8388	15.553	147	2337	4.3333	7.5734	7.5125	26.1566	27.8612
DN	SPZ1_2	cluster_4	SP4	8507	16962.508	16713	17212	147.2267	0.5015	-0.9955	0.001	0.0010435	18565	186152	75.582	2339	22816	9.2638	852	8507	3.454	4.5893	4.5699	36.4258	37.2852
DN	IKZF1_1	cluster_4	SP4	5246	10473.871	10290	10650	103.2792	0.5009	-0.9974	0.001	0.0010435	11933	143526	82.189	1153	13125	7.5159	447	5246	3.0041	3.7459	3.6551	38.7684	39.9695
DP	XBP1_2	cluster_4	SP4	2915	5827.268	5557	6101	168.2862	0.5003	-0.9991	0.001	0.0010435	2416	55665	57.716	478	10402	10.785	127	2915	3.0224	5.2566	5.2367	26.569	28.0235
SP8	SMAD_2	cluster_3	DN	18361	36703.35	36272	37185	276.8795	0.5003	-0.9992	0.001	0.0010435	21409	221606	55.683	6601	66737	16.769	1785	18361	4.6135	8.3376	8.2854	27.0414	27.5125
SP4	SPIB_2	cluster_3	DN	20011	40013.283	39460	40527	317.5528	0.5001	-0.9996	0.001	0.0010435	18724	244697	60.687	5238	66763	16.558	1539	20011	4.9629	8.2194	8.1779	29.3814	29.9732
DN	RUNX1_2	cluster_4	SP4	6802	13601.7	13349	13862	159.4217	0.5001	-0.9997	0.001	0.0010435	5376	83982	74.445	1297	19205	17.024	447	6802	6.0296	8.3147	8.0994	34.4641	35.4179
SP8	MAF_known12	cluster_3	DN	10612	21235.538	20774	21656	258.8596	0.4998	-1.0007	0.001	0.0010435	7662	130608	59.214	2214	36416	16.51	625	10612	4.8112	8.1571	8.1251	28.2294	29.141
DN	TBX5_3	cluster_4	SP4	9476	18964.185	18745	19186	136.1282	0.4997	-1.0009	0.001	0.001	20740	186864	75.435	2921	25603	10.336	1063	9476	3.8254	5.1254	5.0711	36.3916	37.0113
DN	PBX1_4	cluster_4	SP4	2812	5630.07	5476	5777	90.9689	0.4995	-1.0013	0.001	0.0010909	3459	38700	71.853	750	8086	15.013	249	2812	5.2209	7.1986	7.2661	33.2	34.7762
DP	CUX2_1	cluster_4	SP4	409	819.783	720	918	59.2941	0.4995	-1.0014	0.001	0.0012632	328	6022	56.539	110	1680	15.773	26	409	3.84	7.9268	6.7918	23.6364	24.3452
SP4	PKNOX2_3	cluster_3	DN	11346	22734.202	22337	23161	233.4054	0.4991	-1.0026	0.001	0.0010435	10156	113004	52.742	3958	43496	20.301	1019	11346	5.2955	10.0335	10.0404	25.7453	26.0852
DP	POU2F2_known14	cluster_1	SP8	550	1103.172	996	1208	62.9825	0.499	-1.0028	0.001	0.0012632	685	10526	46.78	183	2502	11.12	38	550	2.4443	5.5474	5.2252	20.765	21.9824
DP	CTCF_known1	cluster_4	SP4	25538	51218.534	50406	51983	481.8137	0.4986	-1.004	0.001	0.0010435	55720	1030489	59.443	4997	87610	5.0537	1397	25538	1.4731	2.5072	2.4782	27.9568	29.1496
SP8	SMAD3_3	cluster_3	DN	2589	5195.672	5038	5346	97.005	0.4984	-1.0046	0.001	0.0010435	3767	33918	58.906	1019	8929	15.507	290	2589	4.4964	7.6984	7.6331	28.4593	28.9954
SP4	SOX21_3	cluster_3	DN	4380	8789.974	8554	9038	146.5256	0.4984	-1.0048	0.001	0.0010909	3663	54981	62.433	1020	14522	16.49	299	4380	4.9737	8.1627	7.9664	29.3137	30.1611
SP4	SOX8_2	cluster_3	DN	6243	12528.922	12226	12885	207.5732	0.4983	-1.0048	0.001	0.0010909	5294	79524	62.195	1449	20773	16.246	422	6243	4.8826	7.9713	7.8505	29.1235	30.0534
DN	MESP1_1	cluster_4	SP4	9304	18680.145	18416	18921	143.1018	0.4981	-1.0055	0.001	0.001	44484	401632	76.45	2809	24683	4.6984	1038	9304	1.771	2.3334	2.3165	36.9527	37.694
DP	SP8_1	cluster_1	SP8	28993	58229.991	57587	58904	400.1162	0.4979	-1.006	0.001	0.001	63765	778558	62.307	8014	95072	7.6085	2385	28993	2.3203	3.7403	3.7239	29.7604	30.4958
DN	PBX1_2	cluster_4	SP4	3520	7079.421	6891	7269	117.7413	0.4973	-1.0078	0.001	0.0010435	3613	51272	73.03	746	10083	14.362	251	3520	5.0137	6.9471	6.8653	33.6461	34.9102
SP4	PKNOX1_2	cluster_3	DN	11274	22673.388	22316	23038	221.6724	0.4973	-1.0079	0.001	0.0010435	9911	109901	52.023	4017	43989	20.823	1017	11274	5.3367	10.2613	10.2583	25.3174	25.6291
SP4	TBX2_1	cluster_3	DN	11723	23578.883	23131	24072	272.037	0.4972	-1.0081	0.001	0.0010435	8640	147190	60.352	2405	39675	16.268	692	11723	4.8067	8.0093	7.9645	28.7734	29.5476
SP8	MGA_2	cluster_3	DN	7015	14128.375	13729	14484	242.5282	0.4966	-1.01	0.001	0.0010909	5058	86090	56.587	1556	25429	16.714	416	7015	4.6109	8.2246	8.1484	26.7352	27.5866
DP	ATF7_1	cluster_4	SP4	2197	4426.424	4245	4627	116.5275	0.4965	-1.0103	0.001	0.001	3062	39860	54.566	650	8215	11.246	169	2197	3.0076	5.5193	5.5118	26	26.7438
DN	POU2F2_known1	cluster_4	SP4	4979	10032.934	9782	10256	143.509	0.4963	-1.0107	0.001	0.0010435	4025	72871	75.443	825	13977	14.47	285	4979	5.1547	7.0807	6.8326	34.5455	35.6228
DP	KLF16_1	cluster_1	SP8	29805	60069.012	59464	60624	366.9434	0.4962	-1.011	0.001	0.001	76014	850789	64.652	8719	94633	7.1912	2667	29805	2.2649	3.5086	3.5032	30.5884	31.4954
SP8	SOX7_4	cluster_3	DN	3503	7067.407	6815	7306	149.9246	0.4957	-1.0124	0.001	0.0010909	2647	42472	56.757	863	12935	17.286	222	3503	4.6812	8.3869	8.2478	25.7242	27.0816
DN	POU2F2_known10	cluster_4	SP4	3925	7919.728	7762	8096	99.2141	0.4957	-1.0126	0.001	0.0010435	6013	54229	74.503	1251	10899	14.974	443	3925	5.3924	7.3674	7.2378	35.4117	36.0125
DN	SOX8_9	cluster_4	SP4	4282	8644.398	8377	8919	160.6248	0.4954	-1.0133	0.001	0.0010909	3303	52872	68.642	884	13207	17.146	272	4282	5.5592	8.2349	8.0988	30.7692	32.4222
SP4	SOX9_6	cluster_3	DN	5094	10283.246	10002	10579	172.5006	0.4954	-1.0133	0.001	0.0010909	4528	68048	61.63	1215	17231	15.606	344	5094	4.6136	7.5972	7.4859	28.3128	29.563
DN	HBP1_1	cluster_4	SP4	5301	10705.153	10441	10937	150.183	0.4952	-1.0138	0.001	0.0010435	5153	79302	73.662	1056	15222	14.139	350	5301	4.924	6.7922	6.6846	33.1439	34.8246
DP	EGR1_known9	cluster_3	DN	17307	34959.818	34533	35386	264.6181	0.4951	-1.0143	0.001	0.001	29172	396004	73.325	3721	48541	8.988	1300	17307	3.2046	4.4563	4.3704	34.9368	35.6544
DN	SOX21_3	cluster_4	SP4	4566	9225.249	8970	9487	152.3127	0.495	-1.0145	0.001	0.0010909	4326	64953	73.757	903	13016	14.78	305	4566	5.1849	7.0504	7.0297	33.7763	35.0799
SP8	RREB1_1	cluster_3	DN	61845	124952.074	123908	125897	590.0185	0.495	-1.0146	0.001	0.001	48842	818149	64.867	13009	201534	15.979	3852	61845	4.9034	7.8867	7.5591	29.6103	30.6871
DN	POU2F2_known12	cluster_4	SP4	4079	8242.435	8066	8429	111.5285	0.4949	-1.0147	0.001	0.0010435	5446	54541	74.724	1183	11402	15.621	415	4079	5.5884	7.6203	7.4788	35.0803	35.7744
SP8	BPTF_1	cluster_3	DN	13867	28037.642	27575	28490	262.6587	0.4946	-1.0157	0.001	0.0010909	12575	168522	60.074	3764	48126	17.156	1051	13867	4.9433	8.3579	8.2286	27.9224	28.8139
SP8	SOX8_2	cluster_3	DN	5857	11845.64	11501	12125	187.9357	0.4945	-1.016	0.001	0.0010909	4985	74903	58.581	1449	20773	16.246	397	5857	4.5807	7.9639	7.8194	27.3982	28.1953
DN	MTF1_1	cluster_4	SP4	17551	35502.336	35142	35869	216.6933	0.4944	-1.0163	0.001	0.001	48686	649383	82.303	3421	43884	5.5619	1331	17551	2.2244	2.7338	2.7027	38.9068	39.9941
DN	TCF12_known1	cluster_4	SP4	10522	21295.559	21054	21529	143.9367	0.4941	-1.0171	0.001	0.0010909	48943	352576	75.728	4034	28399	6.0997	1482	10522	2.26	3.028	2.9843	36.7377	37.0506
DP	HES5_1	cluster_4	SP4	3267	6615.177	6419	6822	120.783	0.4939	-1.0176	0.001	0.0010435	9609	106096	60.849	1020	10976	6.2951	296	3267	1.8737	3.0804	3.0793	29.0196	29.7649
DN	PBX3_known1	cluster_4	SP4	4877	9879.226	9671	10092	129.972	0.4937	-1.0183	0.001	0.0010435	8906	98882	73.528	1282	13739	10.216	441	4877	3.6265	4.9517	4.9321	34.3994	35.4975
SP4	SMAD_1	cluster_3	DN	10119	20506.978	20193	20767	173.8394	0.4935	-1.019	0.001	0.0010435	16152	129249	59.226	4442	34936	16.009	1271	10119	4.6368	7.869	7.8291	28.6132	28.9644
SP4	SPIB_1	cluster_3	DN	9050	18342.974	18110	18567	146.2368	0.4934	-1.0192	0.001	0.0010435	18137	108822	58.346	5324	31674	16.982	1511	9050	4.8523	8.331	8.3163	28.3809	28.5723
DN	POU2F2_known5	cluster_4	SP4	4100	8312.071	8099	8518	122.4749	0.4933	-1.0194	0.001	0.0010909	4660	60686	74.223	935	11592	14.178	320	4100	5.0146	6.867	6.7561	34.2246	35.3692
DN	TBX21_4	cluster_4	SP4	8751	17743.397	17411	18097	198.0648	0.4932	-1.0197	0.001	0.0010909	9894	148765	73.27	1733	24847	12.238	589	8751	4.3101	5.9531	5.8824	33.9873	35.2195
DN	SMAD_1	cluster_4	SP4	7891	16002.6	15797	16218	127.8309	0.4931	-1.0199	0.001	0.0010435	20794	166413	76.255	2718	21297	9.7589	994	7891	3.6159	4.7802	4.7418	36.571	37.0522
DN	MYCN_1	cluster_4	SP4	4501	9128.705	8938	9315	118.3012	0.4931	-1.02	0.001	0.001	12503	137921	76.685	1121	12081	6.7171	410	4501	2.5026	3.2792	3.2635	36.5745	37.2568
SP8	SOX_1	cluster_3	DN	6104	12386.615	12110	12665	171.0989	0.4928	-1.0208	0.001	0.0010435	6625	79556	57.773	1911	21909	15.91	514	6104	4.4327	7.7585	7.6726	26.8969	27.8607
SP4	SPIC_1	cluster_3	DN	16917	34340.42	33906	34804	276.6453	0.4926	-1.0214	0.001	0.001	16203	211420	60.042	4562	57978	16.465	1305	16917	4.8043	8.0541	8.0016	28.6059	29.1783
SP8	TBX4_1	cluster_3	DN	7008	14230.27	13981	14467	140.8514	0.4925	-1.0218	0.001	0.0010435	11799	82621	55.065	3769	26059	17.368	1004	7008	4.6706	8.5092	8.4821	26.6384	26.8928
SP4	SOX5_3	cluster_3	DN	3026	6145.817	5946	6351	118.8953	0.4924	-1.022	0.001	0.0011429	2611	39250	66.643	681	9692	16.456	205	3026	5.1379	7.8514	7.7096	30.1028	31.2216
DN	ATF3_known5	cluster_4	SP4	9321	18936.977	18609	19248	189.0152	0.4922	-1.0226	0.001	0.001	14705	206515	71.894	1972	26814	9.3348	668	9321	3.2449	4.5427	4.5135	33.8742	34.7617
DN	TBX5_5	cluster_4	SP4	7809	15869.655	15535	16223	202.1719	0.4921	-1.023	0.001	0.0010435	7574	144355	74.742	1206	21904	11.341	413	7809	4.0432	5.4529	5.4096	34.2454	35.651
SP4	POU2F2_known16	cluster_3	DN	2473	5027.121	4830	5232	120.6059	0.492	-1.0232	0.001	0.0010909	2634	34687	58.999	719	8853	15.058	195	2473	4.2063	7.4032	7.1295	27.121	27.934
DN	SOX_1	cluster_4	SP4	7046	14335.679	14064	14626	164.7535	0.4915	-1.0246	0.001	0.0010435	8206	98530	71.552	1772	20522	14.903	591	7046	5.1168	7.202	7.1511	33.3521	34.3339
SP4	SOX12_1	cluster_3	DN	3107	6326.32	6140	6542	122.8172	0.4912	-1.0256	0.001	0.0010435	3166	41308	65.459	813	10059	15.94	243	3107	4.9235	7.6753	7.5215	29.8893	30.8878
SP8	PKNOX1_1	cluster_3	DN	14665	29864.047	29318	30383	324.3551	0.4911	-1.026	0.001	0.0010435	10991	165550	53.351	3874	56704	18.274	979	14665	4.726	8.9073	8.8584	25.271	25.8624
SP8	SOX10_4	cluster_3	DN	6416	13073.923	12728	13381	197.1202	0.4908	-1.0268	0.001	0.0010909	5401	81262	58.18	1603	23013	16.476	435	6416	4.5936	8.0541	7.8954	27.1366	27.8799
SP8	TBX4_2	cluster_3	DN	10546	21488.587	20944	21996	314.339	0.4908	-1.0268	0.001	0.0010909	6718	127933	57.269	2070	38122	17.065	554	10546	4.7209	8.2465	8.2434	26.7633	27.6638
SP8	TBX15_1	cluster_3	DN	8910	18156.122	17773	18628	261.0101	0.4908	-1.0269	0.001	0.0010909	5892	106171	56.155	1916	33027	17.468	501	8910	4.7126	8.5031	8.3921	26.1482	26.9779
DP	GMEB2_3	cluster_1	SP8	596	1215.778	1143	1290	47.1262	0.4906	-1.0273	0.001	0.0010435	2081	29462	70.749	130	1755	4.2144	43	596	1.4312	2.0663	2.0229	33.0769	33.9601
DN	KLF14_1	cluster_4	SP4	5917	12062.364	11646	12525	258.9645	0.4906	-1.0275	0.001	0.001	16591	224164	22.673	4312	55543	5.6179	450	5917	0.59848	2.7123	2.6396	10.436	10.653
SP8	ETV2_1	cluster_3	DN	9737	19850.661	19513	20168	202.7184	0.4905	-1.0276	0.001	0.0010435	16594	166680	56.55	3573	35299	11.976	973	9737	3.3035	5.8636	5.8417	27.232	27.5844
DP	POU2F2_known16	cluster_1	SP8	1467	2992.798	2822	3164	102.8201	0.4903	-1.0281	0.001	0.0010909	2099	27573	46.898	529	6610	11.243	116	1467	2.4952	5.5264	5.3204	21.9282	22.1936
DN	TBX4_2	cluster_4	SP4	8310	16951.387	16625	17283	204.0274	0.4903	-1.0284	0.001	0.0010909	8920	170122	76.154	1263	22909	10.255	440	8310	3.7199	4.9327	4.8847	34.8377	36.274
SP4	SOX2_6	cluster_3	DN	2466	5032.126	4816	5255	130.8972	0.4902	-1.0287	0.001	0.0010435	2099	33627	62.352	562	8388	15.553	156	2466	4.5725	7.4321	7.3334	27.758	29.3991
DP	ATF3_known4	cluster_4	SP4	3130	6387.366	6202	6584	117.22	0.4901	-1.0288	0.001	0.001	8408	118694	67.508	717	9606	5.4635	225	3130	1.7802	2.676	2.637	31.3808	32.5838
DP	SOX3_3	cluster_1	SP8	1931	3940.975	3736	4143	127.7816	0.4901	-1.0288	0.001	0.0010909	2264	36279	46.72	591	8743	11.259	122	1931	2.4867	5.3887	5.3226	20.643	22.0862
SP8	SPI1_known1	cluster_3	DN	19272	39324.77	38902	39722	253.6429	0.4901	-1.0289	0.001	0.001	35122	247084	52.91	10728	74696	15.995	2740	19272	4.1269	7.8014	7.7998	25.5406	25.8006
SP8	FOXO1_4	cluster_3	DN	2192	4474.508	4285	4642	106.5968	0.49	-1.0291	0.001	0.0010909	2229	29043	58.324	647	7957	15.979	170	2192	4.402	7.6267	7.5474	26.2751	27.5481
DN	SP1_known3	cluster_4	SP4	19309	39412.946	39055	39773	232.4733	0.4899	-1.0294	0.001	0.001	47992	629173	78.933	4045	50952	6.3922	1492	19309	2.4224	3.1089	3.0689	36.885	37.8965
SP8	SOX7_3	cluster_3	DN	4107	8388.302	8088	8631	163.4972	0.4897	-1.0301	0.001	0.0010909	3353	53689	59.073	976	14704	16.179	260	4107	4.5188	7.7542	7.6496	26.6393	27.9312
DN	SMAD3_3	cluster_4	SP4	2340	4782.996	4665	4906	73.2466	0.4893	-1.0311	0.001	0.0010435	4812	43323	75.24	744	6513	11.311	261	2340	4.0639	5.4239	5.4013	35.0806	35.9281
SP8	POU2F2_known5	cluster_3	DN	3719	7602.197	7380	7817	143.0371	0.4893	-1.0313	0.001	0.0010909	3679	47888	58.57	1066	13379	16.363	288	3719	4.5486	7.8282	7.766	27.0169	27.7973
SP4	SMAD3_3	cluster_3	DN	2648	5414.796	5250	5572	97.4461	0.4891	-1.0317	0.001	0.0010435	3934	35416	61.507	1019	8929	15.507	295	2648	4.5988	7.4987	7.4768	28.95	29.6562
SP4	SOX9_4	cluster_3	DN	6280	12848.862	12539	13136	183.2483	0.4888	-1.0327	0.001	0.0010909	5343	80252	62.068	1492	21287	16.464	425	6280	4.8571	7.9543	7.8254	28.4853	29.5016
SP4	TCF4_2	cluster_3	DN	29166	59669.844	59073	60256	354.4075	0.4888	-1.0327	0.001	0.001	28828	260376	48.438	13902	124083	23.083	3231	29166	5.4257	11.2079	11.2015	23.2413	23.5052
SP8	TBX2_2	cluster_3	DN	7168	14666.843	14390	14938	167.9663	0.4888	-1.0328	0.001	0.0010909	9067	90775	56.983	2659	26010	16.328	720	7168	4.4997	7.9409	7.8964	27.0778	27.5586
SP8	HMGA1_1	cluster_3	DN	1596	3268.237	3162	3374	58.987	0.4885	-1.0336	0.001	0.0010435	3543	21381	63.056	893	5260	15.513	267	1596	4.7069	7.536	7.4646	29.8992	30.3422
SP4	MAF_known1	cluster_3	DN	32226	65984.804	65169	66700	451.3366	0.4884	-1.0339	0.001	0.001	26418	399043	58.146	7841	115038	16.763	2139	32226	4.6958	8.0968	8.0758	27.2797	28.0134
SP8	TBX5_3	cluster_3	DN	11714	23986.583	23595	24373	223.0442	0.4884	-1.0339	0.001	0.001	14898	134209	54.179	5059	44697	18.044	1306	11714	4.7288	8.7663	8.7282	25.8154	26.2076
SP4	FOXP3_1	cluster_3	DN	10322	21139.084	20721	21629	266.1471	0.4883	-1.0341	0.001	0.0010909	7981	128134	57.854	2429	37397	16.885	647	10322	4.6605	8.1068	8.0556	26.6365	27.6011
SP8	SOX15_4	cluster_3	DN	2476	5071.648	4888	5260	122.3789	0.4883	-1.0341	0.001	0.0011429	2193	30727	55.692	715	9431	17.094	178	2476	4.4877	8.1167	8.0581	24.8951	26.2538
SP8	POU2F2_known12	cluster_3	DN	3408	6980.357	6764	7177	122.1251	0.4883	-1.0342	0.001	0.0010435	4381	43877	60.114	1240	11923	16.335	343	3408	4.6691	7.8293	7.7672	27.6613	28.5834
SP4	LMO2_1	cluster_3	DN	46773	95870.518	94997	96671	505.2537	0.4879	-1.0354	0.001	0.001	35624	395160	47.238	18571	204084	24.397	4216	46773	5.5913	11.8347	11.8365	22.7021	22.9185

DP	EGR1_known12	cluster_1	SP8	28342	58106.143	57476	58879	436.6923	0.4878	-1.0357	0.001	0.001	50191	833460	66.56	5616	89364	7.1366	1729	28342	2.2634	3.4448	3.4005	30.787	31.7152
DP	PATZ1_1	cluster_4	SP4	28093	57607.21	56903	58245	385.9498	0.4877	-1.036	0.001	0.001	72910	959184	65.12	7074	90275	6.1288	2132	28093	1.9073	2.9242	2.9288	30.1385	31.1194
SP4	ELK4_1	cluster_3	DN	3215	6593.781	6445	6726	84.7257	0.4877	-1.0361	0.001	0.001	10509	84669	76.516	1097	8678	7.8423	402	3215	2.9054	3.8253	3.7971	36.6454	37.0477
SP4	RREB1_1	cluster_3	DN	60293	123651.981	122675	124635	618.4243	0.4876	-1.0362	0.001	0.001	47895	812464	64.416	13009	201534	15.979	3687	60293	4.7803	7.6981	7.421	28.3419	29.917
SP8	TCF4_2	cluster_3	DN	28736	58951.69	58331	59602	355.0463	0.4875	-1.0366	0.001	0.001	28489	257334	47.872	13902	124083	23.083	3187	28736	5.3457	11.1868	11.1668	22.9248	23.1587
SP4	MGA_3	cluster_3	DN	6611	13565.692	13228	13918	202.4136	0.4874	-1.0369	0.001	0.0010909	5104	86900	61.334	1383	22542	15.91	391	6611	4.666	7.6607	7.6076	28.2719	29.3275
DN	CTCF_known2	cluster_2	DP	11047	22677.607	22092	23274	371.0839	0.4872	-1.0375	0.001	0.0010435	17849	287162	34.367	4231	66340	7.9395	688	11047	1.3221	3.8546	3.847	16.2609	16.6521
SP8	SOX4_2	cluster_3	DN	5212	10700.948	10374	10991	185.7063	0.4871	-1.0377	0.001	0.0010909	4506	67750	58.989	1328	18761	16.335	352	5212	4.5381	7.8118	7.693	26.506	27.781
SP8	POU2F3_1	cluster_3	DN	4273	8781.597	8478	9067	175.5454	0.4866	-1.0391	0.001	0.0010435	3586	54118	58.145	1089	15505	16.659	288	4273	4.591	8.0312	7.8957	26.4463	27.5589
SP8	LMO2_1	cluster_3	DN	46600	95766.644	94965	96651	502.5562	0.4866	-1.0392	0.001	0.001	35542	394212	47.125	18571	204084	24.397	4199	46600	5.5707	11.8142	11.8211	22.6105	22.8337
SP8	SOX9_4	cluster_3	DN	5880	12086.832	11758	12396	195.9248	0.4865	-1.0394	0.001	0.0010909	5012	75280	58.223	1492	21287	16.464	398	5880	4.5477	7.9409	7.8108	26.6756	27.6225
SP8	TBX19_1	cluster_3	DN	5381	11068.41	10663	11474	238.932	0.4862	-1.0404	0.001	0.0010435	3619	68942	53.926	1156	21010	16.434	289	5381	4.209	7.9856	7.8051	25	25.6116
SP4	HBP1_1	cluster_3	DN	4950	10182.675	9896	10485	176.6234	0.4862	-1.0405	0.001	0.0010435	4429	68253	63.399	1145	16681	15.495	331	4950	4.5979	7.4735	7.2524	28.9083	29.6745
SP4	SOX10_4	cluster_3	DN	6725	13836.696	13520	14184	200.5802	0.4861	-1.0408	0.001	0.0010909	5739	86325	61.805	1603	23013	16.476	455	6725	4.8148	7.9282	7.7903	28.3843	29.2226
SP8	TBX5_5	cluster_3	DN	8856	18230.469	17786	18679	272.9374	0.4858	-1.0415	0.001	0.0010435	5748	109382	56.634	1794	32801	16.983	468	8856	4.5853	8.142	8.0964	26.087	26.9992
DP	GMEB2_3	cluster_3	DN	1502	3093.046	2959	3236	82.5016	0.4858	-1.0416	0.001	0.0010435	2081	29462	70.749	322	4464	10.72	106	1502	3.6068	5.0937	5.0981	32.9193	33.647
SP8	FOXO3_4	cluster_3	DN	1778	3664.18	3512	3807	93.5458	0.4854	-1.0428	0.001	0.0010909	1903	24809	59.374	521	6382	15.274	138	1778	4.2552	7.2517	7.1668	26.4875	27.8596
SP4	TBX1_4	cluster_3	DN	13272	27366.708	26882	27897	319.0572	0.485	-1.044	0.001	0.0010909	8929	170354	58.182	2605	47743	16.306	702	13272	4.5329	7.862	7.7908	26.9482	27.7988
DN	POU2F3_1	cluster_4	SP4	4666	9622.704	9382	9896	145.577	0.4849	-1.0441	0.001	0.0010435	4595	69456	74.624	940	13469	14.471	314	4666	5.0132	6.8335	6.7179	33.4043	34.6425
SP4	TBX21_6	cluster_3	DN	12037	24839.996	24354	25367	298.541	0.4846	-1.0451	0.001	0.0010909	8330	150313	59.065	2481	42917	16.864	671	12037	4.7299	8.0552	8.008	27.0455	28.0472
SP8	TBX15_2	cluster_3	DN	8877	18319.444	18002	18609	176.5366	0.4846	-1.0451	0.001	0.0010909	14966	104782	54.532	4913	33885	17.635	1274	8877	4.6199	8.5126	8.4719	25.9312	26.1974
SP8	IKZF2_3	cluster_3	DN	4453	9192.461	8953	9423	134.092	0.4845	-1.0455	0.001	0.0010909	5533	55534	62.869	1594	15124	17.122	454	4453	5.0412	8.2053	8.0185	28.4818	29.4433
DP	EGR4_1	cluster_1	SP8	11260	23251.094	22903	23544	213.1004	0.4843	-1.046	0.001	0.001	29511	334771	66.296	3232	35618	7.0536	1004	11260	2.2299	3.4021	3.3635	31.0644	31.6132
SP8	SPDEF_4	cluster_3	DN	7477	15458.683	15087	15856	232.0845	0.4837	-1.0478	0.001	0.0010435	7014	105694	58.627	1919	27215	15.096	507	7477	4.1474	7.2284	7.0742	26.42	27.4738
SP4	CUX1_8	cluster_3	DN	620	1282.97	1189	1380	58.3541	0.4837	-1.0479	0.001	0.0011429	545	8762	65.969	143	2072	15.6	41	620	4.668	7.5229	7.076	28.6713	29.9228
SP4	MESP1_1	cluster_3	DN	28149	58209.196	57644	58772	350.3553	0.4836	-1.0481	0.001	0.001	28293	255419	48.619	13534	120595	22.955	3125	28149	5.3581	11.0451	11.0207	23.09	23.3418
SP8	FOXO6_1	cluster_3	DN	2447	5061.724	4879	5256	120.7359	0.4835	-1.0483	0.001	0.0010909	2530	32988	57.537	738	9094	15.861	191	2447	4.268	7.5494	7.4178	25.8808	26.9079
SP4	SOX7_3	cluster_3	DN	4286	8872.528	8593	9122	169.7299	0.4831	-1.0495	0.001	0.0010909	3572	57193	62.928	976	14704	16.179	274	4286	4.7158	7.6708	7.4939	28.0738	29.1485
SP4	GATA_known17	cluster_3	DN	5545	11479.54	11148	11829	197.7605	0.4831	-1.0497	0.001	0.0010909	4062	65192	61.257	1265	19323	18.157	350	5545	5.2103	8.6164	8.5056	27.668	28.6964
SP8	TBX2_1	cluster_3	DN	11301	23401.489	22958	23891	280.3686	0.4829	-1.0501	0.001	0.0010435	8536	145462	59.643	2405	39675	16.268	667	11301	4.6337	7.814	7.769	27.7339	28.4839
SP4	IKZF2_3	cluster_3	DN	4671	9674.112	9471	9901	134.1567	0.4829	-1.0502	0.001	0.0010909	5825	58444	66.163	1594	15124	17.122	476	4671	5.2879	8.1717	7.9923	29.862	30.8847
SP4	POU2F2_known5	cluster_3	DN	3850	7978.749	7727	8202	144.9716	0.4826	-1.0511	0.001	0.0010909	3860	50256	61.466	1066	13379	16.363	298	3850	4.7088	7.7202	7.6608	27.955	28.7764
SP8	SMAD_1	cluster_3	DN	9684	20069.73	19797	20370	183.3584	0.4825	-1.0513	0.001	0.0010435	15801	126448	57.942	4442	34936	16.009	1215	9684	4.4375	7.6894	7.6585	27.3525	27.7193
DP	MYCN_2	cluster_4	SP4	3878	8040.955	7837	8231	114.8392	0.4823	-1.0519	0.001	0.001	17531	158428	65.545	1415	12426	5.1409	433	3878	1.6044	2.4699	2.4478	30.6007	31.2088
SP8	SOX12_1	cluster_3	DN	2864	5941.4	5742	6139	122.023	0.4821	-1.0525	0.001	0.0010435	2969	38740	61.39	813	10059	15.94	225	2864	4.5385	7.5783	7.3929	27.6753	28.472
DP	EGR3_2	cluster_3	DN	30720	63734.952	63097	64407	397.6081	0.482	-1.0529	0.001	0.001	45781	699461	71.099	6402	91970	9.3486	2069	30720	3.1226	4.5193	4.392	32.318	33.4022
SP8	SOX17_4	cluster_3	DN	2846	5907.562	5673	6134	140.4049	0.4818	-1.0534	0.001	0.0010909	2660	37246	58.82	786	10403	16.429	204	2846	4.4945	7.6692	7.6411	25.9542	27.3575
SP4	PKNOX1_1	cluster_3	DN	15159	31474.928	30951	31990	310.4804	0.4816	-1.054	0.001	0.0010435	11588	174615	56.273	3874	56704	18.274	1010	15159	4.8852	8.7159	8.6814	26.0712	26.7336
SP8	FOXO4_3	cluster_3	DN	1982	4118.076	3956	4305	111.6025	0.4814	-1.0546	0.001	0.0010909	2096	27285	58.087	592	7294	15.528	154	1982	4.2194	7.3473	7.2641	26.0135	27.173
DN	IKZF3_1	cluster_4	SP4	4234	8803.841	8633	8979	107.9919	0.481	-1.0559	0.001	0.0010435	8816	106058	78.616	1001	11533	8.5489	357	4234	3.1385	4.0495	3.9922	35.6643	36.712
SP8	TBX21_6	cluster_3	DN	11589	24094.693	23622	24537	287.1169	0.481	-1.0559	0.001	0.0010909	8068	145632	57.226	2481	42917	16.864	651	11589	4.5539	8.0689	7.9577	26.2394	27.0033
SP4	SPZ1_2	cluster_3	DN	11094	23069.262	22768	23390	193.4904	0.4809	-1.0561	0.001	0.0010435	14241	142725	57.95	4088	40215	16.328	1114	11094	4.5044	7.8225	7.773	27.2505	27.5867
DN	ETS_known17	cluster_4	SP4	10103	21010.546	20629	21419	231.9601	0.4809	-1.0563	0.001	0.001	15240	260380	73.574	1774	29054	8.2097	596	10103	2.8548	3.9108	3.8801	33.5964	34.7732
DP	GMEB2_2	cluster_4	SP4	1307	2720.321	2588	2852	85.904	0.4806	-1.0569	0.001	0.001	2591	34443	62.139	360	4500	8.1185	100	1307	2.358	3.8595	3.7947	27.7778	29.0444
DN	TBX1_4	cluster_4	SP4	11049	23006.947	22575	23397	251.8195	0.4803	-1.0581	0.001	0.0010909	11107	211898	72.371	1814	32620	11.141	583	11049	3.7736	5.2489	5.2143	32.1389	33.8719
DN	MAFF_1	cluster_4	SP4	8728	18175.088	17858	18504	202.0089	0.4802	-1.0582	0.001	0.001	12562	176219	70.456	1924	26154	10.457	625	8728	3.4896	4.9753	4.9529	32.4844	33.3716
SP8	MESP1_1	cluster_3	DN	27863	58069.374	57451	58664	362.3456	0.4798	-1.0594	0.001	0.001	28229	254858	48.512	13534	120595	22.955	3093	27863	5.3037	10.9568	10.9328	22.8536	23.1046
SP4	PBX1_4	cluster_3	DN	2283	4759.202	4585	4906	97.1367	0.4798	-1.0595	0.001	0.0010909	2946	33026	61.318	745	7979	14.814	207	2283	4.2388	7.0265	6.9127	27.7852	28.6126
DN	REL_1	cluster_4	SP4	6866	14313.952	14108	14500	120.5413	0.4797	-1.0598	0.001	0.001	15073	136291	76.106	2162	19122	10.678	764	6866	3.834	5.0687	5.0378	35.3377	35.9063
SP4	ELK4_2	cluster_3	DN	3546	7393.336	7143	7687	164.7844	0.4797	-1.0598	0.001	0.0011429	7834	70855	26.241	3169	28339	10.495	394	3546	1.3133	5.0294	5.0046	12.4329	12.5128
DP	HES1_1	cluster_4	SP4	8620	17977.501	17566	18383	227.8577	0.4795	-1.0603	0.001	0.001	24843	352037	63.079	2115	28979	5.1926	613	8620	1.5446	2.4675	2.4486	28.9835	29.7457
SP4	SOX10_9	cluster_3	DN	3864	8058.975	7850	8310	140.5627	0.4795	-1.0603	0.001	0.0010435	3684	51623	61.408	1017	13538	16.104	283	3864	4.5964	7.6819	7.485	27.8269	28.5419
DN	EWSR1::FLI1_1	cluster_4	SP4	56371	117580.899	116393	118709	690.663	0.4794	-1.0606	0.001	0.0010435	56590	1127364	69.149	9280	176015	10.796	2891	56371	3.4576	5.1087	5.0002	31.153	32.0262
SP4	SOX15_4	cluster_3	DN	2587	5400.329	5177	5608	126.8951	0.4791	-1.0615	0.001	0.0011429	2335	32728	59.319	715	9431	17.094	187	2587	4.6889	8.0086	7.9045	26.1538	27.4308
SP8	ARID5B_1	cluster_3	DN	4818	10057.996	9742	10328	169.7688	0.4791	-1.0617	0.001	0.0010909	4547	59233	58.39	1409	17650	17.399	375	4818	4.7494	8.2472	8.134	26.6146	27.2975
SP4	SOX7_4	cluster_3	DN	3607	7532.028	7269	7820	158.9246	0.479	-1.062	0.001	0.0010909	2839	45561	60.885	863	12935	17.286	228	3607	4.8202	8.031	7.9169	26.4195	27.8856
SP8	ATF4_3	cluster_3	DN	3797	7931.313	7695	8191	143.1379	0.4788	-1.0625	0.001	0.0010909	3790	49324	56.677	1124	14204	16.322	294	3797	4.3631	7.7573	7.6981	26.1566	26.7319
DP	KLF12_1	cluster_4	SP4	3409	7123.936	6951	7287	97.4656	0.4786	-1.0631	0.001	0.001	19413	116478	59.073	2058	12154	6.164	569	3409	1.7289	2.931	2.9267	27.6482	28.0484
SP4	TBX4_1	cluster_3	DN	6870	14355.678	14081	14609	159.5804	0.4786	-1.0631	0.001	0.0010435	11905	83358	55.556	3769	26059	17.368	983	6870	4.5787	8.257	8.2416	26.0812	26.3633
DN	NFKB_known10	cluster_4	SP4	6568	13727.129	13475	13988	152.4319	0.4785	-1.0634	0.001	0.001	12896	155666	77.254	1529	18124	8.9945	548	6568	3.2596	4.2494	4.2193	35.8404	36.2392
DP	EGR1_known9	cluster_1	SP8	10615	22189.011	21817	22518	213.1012	0.4784	-1.0637	0.001	0.001	29172	396004	73.325	2355	30796	5.7023	789	10615	1.9655	2.7046	2.6805	33.5032	34.4688
DP	ATF6_1	cluster_4	SP4	1016	2125.846	2036	2219	57.8055	0.4782	-1.0644	0.001	0.0010435	3671	25707	59.612	521	3605	8.3596	146	1016	2.356	3.9771	3.9522	28.023	28.1831
DP	SPZ1_1	cluster_4	SP4	19083	39908.127	39359	40469	322.3154	0.4782	-1.0644	0.001	0.001	49189	720565	65.335	4489	62429	5.6605	1319	19083	1.7303	2.6815	2.6483	29.3829	30.5675
SP4	SPDEF_4	cluster_3	DN	8038	16830.481	16479	17188	233.0673	0.4776	-1.0661	0.001	0.0010435	7657	115318	63.966	1919	27215	15.096	544	8038	4.4586	7.1046	6.9703	28.3481	29.5352
DP	SP8_1	cluster_4	SP4	24650	51651.013	51086	52223	359.4791	0.4773	-1.0672	0.001	0.001	63765	778558	62.307	7107	84496	6.7621	1988	24650	1.9727	3.1177	3.1661	27.9724	29.173
SP8	SOX18_2	cluster_3	DN	3037	6365.484	6170	6557	114.9182	0.4772	-1.0674	0.001	0.0010909	2756	38602	58.784	847	11219	17.084	218	3037	4.6248	7.91	7.8675	25.7379	27.0701
SP4	SOX4_2	cluster_3	DN	5423	11373.893	11085	11674	177.554	0.4768	-1.0684	0.001	0.0010909	4816	72432	63.066	1328	18761	16.335	369	5423	4.7218	7.662	7.487	27.7861	28.9057
DP	EGR4_2	cluster_1	SP8	23117	48497.39	47907	49116	362.844	0.4767	-1.0689	0.001	0.001	49002	804691	69.973	4521	71084	6.1812	1421	23117	2.0102	2.8999	2.8728	31.4311	32.5207
DN	SP1_known2	cluster_4	SP4	40454	84927.689	84450	85468	311.6073	0.4763	-1.0699	0.001	0.001	131181	1808239	79.788	8420	109754	4.8428	3027	40454	1.785	2.3075	2.2372	35.9501	36.8588
DP	KLF14_1	cluster_1	SP8	19632	41213.316	40730	41797	309.2288	0.4764	-1.0699	0.001	0.001	49087	664659	67.227	4810	62544	6.326	1464	19632	1.9857	2.9825	2.9537	30.4366	31.3891
SP4	TBX15_2	cluster_3	DN	8730	18332.107	18067	18602	168.1307	0.4762	-1.0702	0.001	0.0010909	14978	104871	54.579	4913	33885	17.635	1251	8730	4.5434	8.3522	8.3245	25.4631	25.7636
SP4	ATF4_3	cluster_3	DN	3973	8344.885	8146	8585	131.4581	0.4762	-1.0705	0.001	0.0010909	3989	51908	59.647	1124	14204	16.322	306	3973	4.5653	7.6711	7.6539	27.2242	27.971
SP8	SOX3_3	cluster_3	DN	3565	7488.793	7210	7753	168.607	0.4761	-1.0706	0.001	0.0010909	2699	43266	55.718	920	13934	17.944	226	3565	4.591	8.3735	8.2397	24.5652	25.5849
SP4	BPTF_1	cluster_3	DN	13616	28606.551	28146	29055	267.08	0.476	-1.071	0.001	0.0010909	12832	172114	61.355	3764	48126	17.156	1036	13616	4.8538	8.0736	7.911	27.5239	28.2924
DP	E2F2_3	cluster_1	SP8	1124	2362.685	2194	2503	95.4077	0.476	-1.0711	0.001	0.001	1923	32730	61.531	247	3972	7.4671	67	1124	2.1131	3.4841	3.4342	27.1255	28.2981
SP4	RBPJ_1	cluster_3	DN	8023	16863.223	16630	17121	155.9239	0.4758	-1.0716	0.001	0.0010435	11294	79074	54.99	4517	31035	21.582	1156	8023	5.5794	10.2355	10.1462	25.5922	25.8515
SP4	POU2F2_known11	cluster_3	DN	3568	7503.157	7302	7685	123.08	0.4756	-1.0722	0.001	0.0010435	4718	47310	63.382	1256	12148	16.275	359	3568	4.7801	7.6092	7.5417	28.5828	29.3711
DN	PATZ1_1	cluster_4	SP4	31980	67256.529	66616	67842	364.3148	0.4755	-1.0725	0.001	0.001	85759	1123464	76.273	7074	90275	6.1288	2448	31980	2.1711	2.8545	2.8466	34.6056	35.4251
SP8	SOX11_2	cluster_3	DN	3931	8268.363	8008	8522	152.9279	0.4755	-1.0725	0.001	0.0010909	3666	51361	60.532	1065	14154	16.681	285	3931	4.6329	7.7741	7.6537	26.7606	27.7731
SP4	HMGA1_1	cluster_3	DN	1649	3470.944	3386	3574	58.815	0.4752	-1.0733	0.001	0.0010435	3745	22602	66.657	893	5260	15.513	275	1649	4.8632	7.3431	7.2958	30.7951	31.3498
DP	EGR3_3	cluster_1	SP8	12709	26755.657	26338	27136	239.1629	0.475	-1.0739	0.001	0.001	31417	463984	71.003	2726	38422	5.8797	874	12709	1.9448	2.7819	2.7391	32.0616	33.0774
SP4	TBX15_1	cluster_3	DN	8862	18665.244	18245	19110	273.8876	0.4748	-1.0746	0.001	0.0010909	6066	109292	57.806	1916	33027	17.468	495	8862	4.6872	8.1602	8.1086	25.8351	26.8326
SP8	SPI1_known4	cluster_3	DN	14827	31230.518	30738	31690	292.8963	0.4748	-1.0747	0.001	0.0010435	14322	186719	57.192	4360	55279	16.932	1143	14827	4.5415	7.9807	7.9408	26.2156	26.8221
DN	CTCF_known1	cluster_4	SP4	11248	23699.123	22919	24325	418.824	0.4746	-1.0751	0.001	0.0010435	26314	479096	27.636	4997	87610	5.0537	627	11248	0.64884	2.3828	2.3478	12.5475	12.8387
SP8	MAF_known1	cluster_3	DN	30246	63745.623	63025	64523	448.1159	0.4745	-1.0756	0.001	0.001	25525	385561	56.181	7841	115038	16.763	2010	30246	4.4072	7.8746	7.8447	25.6345	26.2922
SP4	MTF1_3	cluster_3	DN	12193	25701.383	25241	26133	254.0325	0.4744	-1.0757	0.001	0.001	12953	170029	62.803	3233	41341	15.27	929	12193	4.5037	7.1721	7.1711	28.7349	29.4937
SP8	IRF_known21	cluster_3	DN	7681	16192.264	15871	16521	202.273	0.4744	-1.0758	0.001	0.001	7912	114984	65.268	1832	25304	14.363	536	7681	4.3599	6.7745	6.6801	29.2576	30.3549
DP	CUX1_8	cluster_4	SP4	471	994.244	892	1094	61.3754	0.4743	-1.0763	0.001	0.0011429	409	6571	49.473	140	2170	16.338	30	471	3.5462	7.335	7.1679	21.4286	21.7051
SP8	SOX10_9	cluster_3	DN	3562	7511.884	7263	7781	160.8414	0.4743	-1.0763	0.001	0.0010435	3412	47797	56.857	1017	13538	16.104	259	3562	4.2372	7.5909	7.4524	25.4671	26.3111
SP4	NFATC1_1	cluster_3	DN	8024	16924.269	16541	17350	233.6592	0.4741	-1.0766	0.001	0.0010909	5359	103439	64.397	1524	27428	17.076	419	8024	4.9954	7.8186	7.7572	27.4934	29.2548
DP	HES7_1	cluster_4	SP4	3140	6624.144	6435	6834	126.637	0.4741	-1.0767	0.001	0.0010435	9992	110416	62.326	1003	10767	6.0776	285	3140	1.7724	2.8523	2.8438	28.4148	29.1632
SP8	SPIC_2	cluster_3	DN	15746	33229.804	32740	33755	298.8751	0.4739	-1.0774	0.001	0.0010435	15787	205822	55.49	4773	60618	16.343	1213	15746	4.2451	7.6835	7.6503	25.4138	25.9758
SP8	POU2F2_known16	cluster_3	DN	2207	4658.98	4493	4866	106.5017	0.4738	-1.0776	0.001	0.0010909	2431	32048	54.51	719	8853	15.058	173	2207	3.7538	7.1164	6.8865	24.0612	24.9294
DN	MAFG_1	cluster_4	SP4	8454	17850.553	17479	18208	232.3019	0.4736	-1.0782	0.001	0.0010435	8246	165248	72.352	1341	25357	11.102	427	8454	3.7015	5.1783	5.1159	31.8419	33.3399
DN	MYC_known20	cluster_4	SP4	6263	13226.605	12904	13525	180.4791	0.4736	-1.0784	0.001	0.001	11316	185626	73.632	1173	18285	7.253	386	6263	2.4843	3.4111	3.374	32.9071	34.2521
SP4	SOX3_3	cluster_3	DN	3813	8053.774	7757	8346	172.0464	0.4735	-1.0785	0.001	0.0010909	2900	46500	59.883	920	13934	17.944	240	3813	4.9104	8.2759	8.2	26.087	27.3647
SP4	TBX5_3	cluster_3	DN	11712	24742.647	24366	25074	220.2794	0.4734	-1.0789	0.001	0.001	15391	138670	55.98	5059	44697	18.044	1304	11712	4.728	8.4725	8.446	25.7758	26.2031
SP8	SPZ1_2	cluster_3	DN	10661	22530.354	22163	22908	220.9505	0.4732	-1.0795	0.001	0.0010435	13891	139218	56.526	4088	40215	16.328	1072	10661	4.3286	7.7172	7.6578	26.2231	26.51
DN	MAF_known11	cluster_4	SP4	9926	20980.886	20575	21328	227.1422	0.4731	-1.0797	0.001	0.0010435	13743	221582	72.337	1919	29572	9.654	623	9926	3.2404	4.5332	4.4796	32.4648	33.5655
SP8	SOX5_3	cluster_3	DN	2774	5865.236	5663	6053	116.4073	0.473	-1.0799	0.001	0.0011429	2486	37371	63.453	681	9692	16.456	189	2774	4.71	7.6026	7.4229	27.7533	28.6215
SP8	SPIC_1	cluster_3	DN	14894	31486.354	31042	32028	296.4605	0.473	-1.0799	0.001	0.001	14831	193520	54.959	4562	57978	16.465	1148	14894	4.2298	7.7405	7.6964	25.1644	25.6891
DN	SPZ1_1	cluster_4	SP4	22398	47362.411	46882	47822	296.0043	0.4729	-1.0803	0.001	0.001	58732	857887	77.786	4489	62429	5.6605	1555	22398	2.0309	2.6476	2.6108	34.6402	35.8776
SP8	HMGA1_2	cluster_3	DN	5468	11562.963	11270	11863	177.7766	0.4729	-1.0803	0.001	0.0010435	5565	78050	63.498	1457	19052	15.5	399	5468	4.4485	7.1698	7.0058	27.385	28.7004
SP4	MYBL2_2	cluster_3	DN	2447	5181.182	4999	5357	119.8032	0.4724	-1.082	0.001	0.0010435	2320	35130	65.23	562	8129	15.094	162	2447	4.5436	6.9828	6.9656	28.8256	30.1021
DN	HES1_1	cluster_4	SP4	10578	22404.415	22070	22720	194.0433	0.4722	-1.0826	0.001	0.001	30958	438366	78.548	2115	28979	5.1926	751	10578	1.8954	2.4259	2.4131	35.5083	36.5023
DN	SMAD4_1	cluster_4	SP4	15804	33475.365	33064	33875	254.3471	0.4721	-1.0828	0.001	0.001	32547	462220	75.022	3269	45282	7.3496	1115	15804	2.5651	3.4258	3.4192	34.1083	34.9013
DN	REL_2	cluster_4	SP4	6684	14160.932	13964	14384	124.6746	0.472	-1.083	0.001	0.0010435	15212	137460	76.313	2133	18854	10.467	743	6684	3.7107	4.8843	4.8625	34.8336	35.4514
SP4	MYBL1_5	cluster_3	DN	3299	6989.621	6779	7213	130.8584	0.4721	-1.083	0.001	0.0010435	2888	46605	66.715	683	10712	15.334	205	3299	4.7225	7.0983	7.0786	30.0146	30.7972
SP8	SPIB_2	cluster_3	DN	17422	36909.705	36370	37458	336.0771	0.472	-1.083	0.001	0.0010435	17242	225293	55.874	5238	66763	16.558	1339	17422	4.3208	7.7659	7.733	25.5632	26.0953
SP8	GATA_known17	cluster_3	DN	4925	10443.447	10116	10760	193.4448	0.4716	-1.0842	0.001	0.0010909	3680	58991	55.43	1265	19323	18.157	309	4925	4.6277	8.3967	8.3487	24.4269	25.4878
DP	EGR3_3	cluster_3	DN	19827	42054.634	41548	42539	300.0945	0.4715	-1.0848	0.001	0.001	31417	463984	71.003	4306	60531	9.263	1373	19827	3.0341	4.3702	4.2732	31.8857	32.7551
SP8	TBX20_2	cluster_3	DN	7667	16265.099	15844	16727	262.7586	0.4714	-1.0849	0.001	0.0010435	5480	98825	56.841	1683	29129	16.754	430	7667	4.4098	7.8467	7.7582	25.5496	26.3208
SP4	POU2F2_known12	cluster_3	DN	3486	7396.477	7208	7617	116.4374	0.4714	-1.085	0.001	0.0010435	4643	46514	63.727	1240	11923	16.335	352	3486	4.776	7.5813	7.4945	28.3871	29.2376
DN	SP4_1	cluster_4	SP4	37512	79585.303	78940	80209	406.648	0.4713	-1.0851	0.001	0.001	90889	1624561	78.36	6296	105261	5.0772	2160	37512	1.8094	2.3765	2.3091	34.3075	35.6371
SP8	SOX11_1	cluster_3	DN	11927	25304.297	24762	25750	288.7878	0.4714	-1.0851	0.001	0.0010435	10921	177105	60.65	2854	43312	14.832	754	11927	4.0844	6.9041	6.7344	26.4191	27.5374
SP4	IKZF3_1	cluster_3	DN	6412	13604.845	13287	13876	180.5494	0.4713	-1.0852	0.001	0.0010435	6633	79768	59.129	2031	23575	17.475	542	6412	4.7529	8.1713	8.0383	26.6864	27.1983
DP	EGR3_2	cluster_1	SP8	18454	39157.143	38681	39668	305.6373	0.4713	-1.0853	0.001	0.001	45781	699461	71.099	3895	56407	5.7337	1231	18454	1.8758	2.6889	2.6383	31.6046	32.7158
SP8	PBX1_2	cluster_3	DN	2928	6213.602	5983	6452	141.9288	0.4713	-1.0853	0.001	0.0010435	2880	41061	58.486	821	11039	15.724	212	2928	4.1705	7.3611	7.1309	25.8222	26.5241
SP8	MYBL1_5	cluster_3	DN	3267	6934.28	6702	7153	134.3891	0.4712	-1.0855	0.001	0.0010435	2849	45954	65.783	683	10712	15.334	201	3267	4.6767	7.0551	7.1093	29.429	30.4985
DP	XBP1_4	cluster_4	SP4	2464	5235.944	5025	5454	134.4292	0.4707	-1.0871	0.001	0.0010435	4834	62945	57.546	739	9250	8.4566	190	2464	2.2527	3.9305	3.9145	25.7104	26.6378
DP	ATF2_1	cluster_4	SP4	322	686.353	639	741	30.8173	0.4699	-1.0895	0.001	0.0010435	1296	9072	66.75	151	1053	7.7478	46	322	2.3692	3.5494	3.5494	30.4636	30.5793
SP8	FOXO4_2	cluster_3	DN	5177	11019.262	10753	11296	175.6211	0.4699	-1.0897	0.001	0.0010435	6205	81377	63.594	1450	17939	14.019	404	5177	4.0457	6.5109	6.3617	27.8621	28.8589
SP8	TBX1_2	cluster_3	DN	6899	14686.2	14290	15065	228.5201	0.4698	-1.0899	0.001	0.0010909	4843	87270	55.954	1562	26827	17.2	389	6899	4.4233	8.0322	7.9054	24.904	25.7166
DN	KLF12_2	cluster_4	SP4	19970	42525.99	42033	42966	276.3666	0.4696	-1.0905	0.001	0.001	46999	672450	77.85	4070	55774	6.457	1421	19970	2.3119	3.0235	2.9697	34.914	35.8052
DP	HIF1A::ARNT_1	cluster_4	SP4	1998	4257.352	4132	4367	72.3232	0.4694	-1.091	0.001	0.001	10494	75476	67.161	915	6438	5.7288	284	1998	1.7779	2.7063	2.6472	31.0383	31.0345

SP4	TBX1_2	cluster_3	DN	7086	15095.43	14694	15508	247.0269	0.4694	-1.091	0.001	0.0010909	4990	89894	57.636	1562	26827	17.2	395	7086	4.5432	7.9158	7.8826	25.2881	26.4137
DP	NFKB_known10	cluster_4	SP4	5033	10740.13	10448	11045	170.7216	0.4687	-1.0934	0.001	0.001	10029	121099	60.099	1529	18124	8.9945	418	5033	2.4978	4.1679	4.1561	27.3381	27.7698
DN	MYCN_2	cluster_4	SP4	4545	9701.623	9538	9875	101.4533	0.4685	-1.0938	0.001	0.001	21157	191106	79.065	1415	12426	5.1409	505	4545	1.8804	2.3869	2.3783	35.689	36.5765
DP	SP4_1	cluster_4	SP4	33727	71986.382	71236	72750	458.9261	0.4685	-1.0938	0.001	0.001	81673	1469721	70.891	6296	105261	5.0772	1921	33727	1.6268	2.3521	2.2948	30.5114	32.0413
DP	AHR::ARNT::HIF1A_1	cluster_3	DN	4889	10447.883	10285	10604	101.4324	0.468	-1.0954	0.001	0.0010435	12707	104472	71.635	1807	14714	10.089	592	4889	3.3523	4.6588	4.6797	32.7615	33.2269
SP8	FOXO1_5	cluster_3	DN	22244	47546.978	47018	48129	336.9661	0.4678	-1.0959	0.001	0.0010435	25271	279379	58.574	7636	82243	17.243	2025	22244	4.6636	8.0131	7.9619	26.5191	27.0467
SP8	RREB1_2	cluster_3	DN	86294	184529.048	183077	186105	916.5694	0.4676	-1.0965	0.001	0.001	51915	1257959	63.21	14003	307557	15.454	3721	86294	4.3361	7.1675	6.8598	26.5729	28.0579
SP8	IRF4_1	cluster_3	DN	3024	6467.983	6267	6672	119.5455	0.4676	-1.0966	0.001	0.0010435	3249	45692	67.371	712	9749	14.375	218	3024	4.4588	6.7098	6.6182	30.618	31.0186
SP8	SPIB_1	cluster_3	DN	7788	16655.747	16388	16889	153.6493	0.4676	-1.0966	0.001	0.0010435	16443	98658	52.897	5324	31674	16.982	1300	7788	4.1756	7.9061	7.8939	24.4177	24.588
DN	GMEB2_3	cluster_4	SP4	887	1898.363	1815	1987	52.2111	0.4675	-1.0969	0.001	0.0010435	2360	33394	80.191	180	2424	5.8209	64	887	2.13	2.7119	2.6562	35.5556	36.5924
SP8	MYC_known13	cluster_3	DN	8675	18556.902	18261	18916	205.2846	0.4675	-1.0969	0.001	0.0010909	9016	99281	55.743	3113	33619	18.876	792	8675	4.8707	8.7844	8.7378	25.4417	25.8039
SP4	SOX17_4	cluster_3	DN	2912	6235.21	6025	6472	130.0108	0.4671	-1.0982	0.001	0.0010909	2822	39514	62.402	786	10403	16.429	211	2912	4.5987	7.477	7.3695	26.8448	27.9919
DP	SP1_known2	cluster_4	SP4	35105	75182.75	74498	75808	396.9806	0.4669	-1.0987	0.001	0.001	115319	1604370	70.792	8420	109754	4.8428	2606	35105	1.549	2.2598	2.1881	30.9501	31.9852
DP	KLF16_1	cluster_4	SP4	25311	54210.628	53640	54697	330.8817	0.4669	-1.0988	0.001	0.001	76014	850789	64.652	7768	85275	6.4801	2212	25311	1.9234	2.91	2.975	28.4758	29.6816
DP	KLF12_2	cluster_4	SP4	16214	34738.327	34206	35307	332.7685	0.4668	-1.0992	0.001	0.001	38298	549311	63.594	4070	55774	6.457	1158	16214	1.8771	3.0237	2.9517	28.4521	29.0709
DN	CTCF_known2	cluster_4	SP4	5650	12107.657	11661	12560	263.1051	0.4667	-1.0995	0.001	0.0010435	17849	287162	34.367	2287	35597	4.2602	353	5650	0.67619	1.9777	1.9675	15.4351	15.8721
SP4	FOXO1_5	cluster_3	DN	21978	47099.38	46582	47681	325.5349	0.4666	-1.0996	0.001	0.0010435	25037	276688	58.01	7636	82243	17.243	2001	21978	4.6078	7.9922	7.9432	26.2048	26.7232
DN	SP4_2	cluster_4	SP4	29353	62914.267	62351	63538	367.3806	0.4666	-1.0999	0.001	0.001	66957	1120770	78.67	5244	82431	5.7861	1799	29353	2.0604	2.6868	2.619	34.3059	35.6092
SP8	SOX17_5	cluster_3	DN	2318	4969.923	4773	5187	119.9308	0.4665	-1.1	0.001	0.0011429	2277	31990	58.026	680	8911	16.164	170	2318	4.2046	7.466	7.246	25	26.0128
SP4	IRF_known14	cluster_3	DN	19657	42144.678	41518	42724	370.775	0.4664	-1.1003	0.001	0.001	13966	293131	66.532	3403	65717	14.916	967	19657	4.4615	6.924	6.7059	28.4161	29.9116
DP	KLF4_1	cluster_4	SP4	21938	47039.294	46558	47548	297.5064	0.4664	-1.1004	0.001	0.001	76804	740702	63.769	8010	74996	6.4566	2275	21938	1.8887	2.9621	2.9618	28.402	29.2522
SP8	NFATC1_3	cluster_3	DN	3441	7380.847	7131	7615	151.7789	0.4663	-1.1007	0.001	0.0010909	3018	39257	57.876	1065	13171	19.418	267	3441	5.073	8.8469	8.7653	25.0704	26.1256
DN	NFKB_known11	cluster_4	SP4	7659	16437.344	16175	16704	159.134	0.466	-1.1016	0.001	0.001	14339	173964	75.858	1852	22046	9.6133	634	7659	3.3398	4.4215	4.4026	34.2333	34.741
SP8	SOX4_1	cluster_3	DN	12618	27093.66	26657	27575	281.8792	0.4657	-1.1024	0.001	0.0010435	11786	191082	60.682	3071	46460	14.754	797	12618	4.0071	6.7623	6.6034	25.9525	27.1588
SP4	MYC_known13	cluster_3	DN	8653	18583.084	18270	18923	196.295	0.4657	-1.1026	0.001	0.0010909	9038	99518	55.876	3113	33619	18.876	789	8653	4.8583	8.7298	8.6949	25.3453	25.7384
DN	HIF1A::ARNT_1	cluster_4	SP4	2385	5123.124	5023	5232	59.7322	0.4656	-1.1027	0.001	0.001	12592	90566	80.589	915	6438	5.7288	332	2385	2.1223	2.6366	2.6334	36.2842	37.0457
SP4	SOX15_2	cluster_3	DN	3245	6976.294	6749	7219	141.2791	0.4652	-1.104	0.001	0.0010909	3199	44866	62.621	869	11549	16.119	233	3245	4.5291	7.2835	7.2326	26.8124	28.0977
SP4	TBX2_2	cluster_3	DN	6992	15032.171	14760	15318	175.0293	0.4652	-1.1042	0.001	0.0010909	9302	93136	58.465	2659	26010	16.328	701	6992	4.3892	7.536	7.5073	26.3633	26.882
DN	ATF6_1	cluster_4	SP4	1215	2614.699	2533	2708	52.7488	0.4649	-1.1051	0.001	0.0010435	4516	31617	73.316	521	3605	8.3596	174	1215	2.8175	3.853	3.8429	33.3973	33.7032
DN	KLF12_1	cluster_4	SP4	4180	8992.99	8868	9115	78.6328	0.4649	-1.1051	0.001	0.001	24570	147420	74.766	2058	12154	6.164	698	4180	2.1199	2.8409	2.8354	33.9164	34.392
SP8	ARID5A_1	cluster_3	DN	862	1855.631	1749	1957	63.4875	0.4648	-1.1053	0.001	0.0012632	834	11458	67.591	232	2962	17.473	66	862	5.0849	7.9137	7.5231	28.4483	29.102
SP4	TBX20_2	cluster_3	DN	7727	16636.963	16220	17074	256.4456	0.4645	-1.1063	0.001	0.0010435	5606	101105	58.153	1683	29129	16.754	431	7727	4.4444	7.6882	7.6425	25.609	26.5268
SP8	POU2F2_known15	cluster_3	DN	2598	5594.917	5419	5775	105.654	0.4644	-1.1064	0.001	0.0010435	3075	33949	59.103	905	9687	16.865	237	2598	4.523	7.7073	7.6527	26.1878	26.8194
SP8	CUX1_8	cluster_3	DN	572	1232.893	1142	1324	56.4562	0.4644	-1.1066	0.001	0.0011429	544	8813	66.353	143	2072	15.6	39	572	4.3066	7.1691	6.4904	27.2727	27.6062
SP4	SOX11_2	cluster_3	DN	4060	8749.808	8503	8996	152.0568	0.4641	-1.1076	0.001	0.0010909	3896	54587	64.334	1065	14154	16.681	296	4060	4.7849	7.5975	7.4377	27.7934	28.6845
SP8	PBX3_known1	cluster_3	DN	5794	12486.919	12198	12769	183.9446	0.464	-1.1077	0.001	0.0010435	6890	76602	56.96	2087	22368	16.633	529	5794	4.3084	7.6778	7.5638	25.3474	25.9031
SP4	IRF_known21	cluster_3	DN	7753	16712.446	16385	17032	203.3482	0.4639	-1.108	0.001	0.001	8196	119181	67.65	1832	25304	14.363	543	7753	4.4008	6.6252	6.5052	29.6397	30.6394
DN	AHR::ARNT::HIF1A_1	cluster_4	SP4	2500	5392.712	5284	5499	66.641	0.4637	-1.1088	0.001	0.0010435	14502	119460	81.912	821	6656	4.5639	303	2500	1.7142	2.0894	2.0928	36.9062	37.5601
SP8	RBPJ_1	cluster_3	DN	7561	16312.304	16068	16577	156.4244	0.4635	-1.1092	0.001	0.0010435	10911	76393	53.125	4517	31035	21.582	1090	7561	5.2581	9.9899	9.8975	24.1311	24.3628
DP	GMEB1_1	cluster_1	SP8	1443	3114.475	2965	3280	95.2343	0.4635	-1.1094	0.001	0.0010435	5680	92646	67.496	293	4645	3.3841	88	1443	1.0513	1.5493	1.5575	30.0341	31.0657
SP4	POU2F3_1	cluster_3	DN	4356	9402.711	9131	9667	166.1718	0.4633	-1.1099	0.001	0.0010435	3852	58181	62.51	1089	15505	16.659	297	4356	4.6801	7.7103	7.487	27.2727	28.0942
SP4	FOXO6_3	cluster_3	DN	22097	47727.674	47185	48280	347.2349	0.463	-1.1109	0.001	0.0010435	21804	285200	58.247	6537	83159	16.984	1701	22097	4.5129	7.8013	7.7479	26.0211	26.572
SP8	MYBL2_2	cluster_3	DN	2375	5131.339	4933	5350	117.7698	0.4629	-1.1111	0.001	0.0010435	2285	34653	64.344	562	8129	15.094	156	2375	4.4099	6.8271	6.8537	27.758	29.2164
DN	BATF_known1	cluster_4	SP4	3173	6857.605	6677	7050	111.722	0.4628	-1.1116	0.001	0.0010435	4780	62340	72.002	763	9682	11.183	244	3173	3.6648	5.1046	5.0898	31.979	32.7722
DP	AHR::ARNT::HIF1A_1	cluster_4	SP4	2185	4723.977	4604	4850	76.3805	0.4626	-1.112	0.001	0.0010435	12707	104472	71.635	821	6656	4.5639	268	2185	1.4982	2.1091	2.0915	32.6431	32.8275
SP8	HBP1_1	cluster_3	DN	4420	9561.161	9259	9828	172.5828	0.4623	-1.113	0.001	0.0010435	4130	63662	59.134	1145	16681	15.495	296	4420	4.1056	7.1671	6.9429	25.8515	26.4972
SP8	SOX13_1	cluster_3	DN	2233	4832.122	4624	5019	126.1809	0.4622	-1.1133	0.001	0.0010909	2089	31443	63.156	581	8084	16.237	152	2233	4.4852	7.2762	7.1017	26.1618	27.6225
SP4	SOX17_5	cluster_3	DN	2425	5248.625	5077	5438	113.8317	0.4621	-1.1136	0.001	0.0011429	2416	33940	61.564	680	8911	16.164	178	2425	4.3987	7.3675	7.145	26.1765	27.2136
SP4	SOX11_1	cluster_3	DN	12529	27115.381	26654	27557	269.0122	0.4621	-1.1138	0.001	0.0010435	11736	190304	65.17	2854	43312	14.832	792	12529	4.2906	6.7485	6.5837	27.7505	28.9273
SP8	CBX5_1	cluster_3	DN	3319	7183.996	6985	7366	119.4177	0.4621	-1.1138	0.001	0.0010435	4252	46829	61.603	1143	12008	15.796	305	3319	4.3661	7.1731	7.0875	26.6842	27.6399
DP	ARID3A_2	cluster_1	SP8	858	1858.521	1706	1997	88.3292	0.4619	-1.1142	0.001	0.0013333	987	15963	50.765	256	3819	12.145	54	858	2.7286	5.4711	5.3749	21.0938	22.4666
SP4	RBPJ_2	cluster_3	DN	8811	19082.725	18716	19432	212.2314	0.4618	-1.1148	0.001	0.0010909	10316	103270	57.388	3446	33705	18.73	886	8811	4.8963	8.5886	8.532	25.711	26.1415
SP8	MYBL1_3	cluster_3	DN	1174	2545.579	2423	2672	74.8426	0.4614	-1.1159	0.001	0.0010909	1462	16095	60.713	400	4270	16.107	107	1174	4.4285	7.3187	7.2942	26.75	27.4941
SP8	ATF4_2	cluster_3	DN	3547	7692.45	7460	7921	135.0128	0.4612	-1.1166	0.001	0.0010909	3830	46012	56.648	1173	13733	16.907	297	3547	4.3669	7.7546	7.7089	25.3197	25.8283
SP8	SOX5_1	cluster_3	DN	1387	3008.898	2892	3132	74.372	0.4611	-1.1167	0.001	0.0010909	2249	20248	60.174	590	5116	15.204	157	1387	4.122	6.9809	6.8501	26.6102	27.111
SP4	ERG_2	cluster_3	DN	5336	11574.731	11286	11816	153.4197	0.4611	-1.117	0.001	0.001	7181	93517	64.636	1430	18064	12.485	412	5336	3.6881	5.7374	5.7059	28.8112	29.5394
SP8	ATF3_known5	cluster_3	DN	11099	24084.051	23671	24554	259.9676	0.4609	-1.1176	0.001	0.001	11933	167611	58.351	3073	41829	14.562	797	11099	3.8639	6.679	6.6219	25.9356	26.5342
DP	CUX1_9	cluster_1	SP8	360	782.601	682	874	60.7374	0.4607	-1.1181	0.001	0.0012632	440	7588	51.249	102	1619	10.935	23	360	2.4314	5.2273	4.7443	22.549	22.2359
SP4	IKZF1_2	cluster_3	DN	12568	27287.2	26940	27622	202.6388	0.4606	-1.1184	0.001	0.0010435	20507	164063	59.864	5902	46144	16.837	1577	12568	4.5859	7.6901	7.6605	26.7198	27.2365
SP8	FOXO3_1	cluster_3	DN	4194	9106.404	8856	9377	151.7514	0.4606	-1.1184	0.001	0.0010435	5381	70700	64.693	1195	14663	13.417	329	4194	3.8376	6.1141	5.9321	27.5314	28.6026
SP8	POU2F3_2	cluster_3	DN	2064	4482.27	4365	4609	74.5294	0.4606	-1.1184	0.001	0.0010909	3384	27131	59.282	988	7694	16.812	257	2064	4.5099	7.5946	7.6075	26.0121	26.8261
SP8	MTF1_3	cluster_3	DN	11651	25299.654	24851	25712	258.791	0.4605	-1.1186	0.001	0.001	12742	167257	61.78	3233	41341	15.27	891	11651	4.3035	6.9926	6.9659	27.5595	28.1827
SP4	ATF4_2	cluster_3	DN	3666	7964.115	7737	8174	136.3931	0.4604	-1.1191	0.001	0.0010909	3978	47794	58.841	1173	13733	16.907	306	3666	4.5134	7.6923	7.6704	26.087	26.6948
SP4	SOX18_2	cluster_3	DN	3101	6739.122	6530	6972	127.6022	0.4602	-1.1196	0.001	0.0010909	2930	41032	62.484	847	11219	17.084	225	3101	4.7222	7.6792	7.5575	26.5643	27.6406
SP8	IRF4_2	cluster_3	DN	6368	13839.637	13556	14135	176.7303	0.4602	-1.1198	0.001	0.001	6695	96589	66.045	1556	21390	14.626	448	6368	4.3543	6.6916	6.5929	28.7918	29.7709
DN	GMEB2_2	cluster_4	SP4	1551	3373.222	3256	3502	70.1394	0.46	-1.1204	0.001	0.001	3215	42707	77.048	360	4500	8.1185	118	1551	2.7982	3.6703	3.6317	32.7778	34.4667
DP	HIF1A_1	cluster_4	SP4	3297	7169.332	6961	7352	120.3715	0.46	-1.1204	0.001	0.0010435	11588	130466	68.094	980	10689	5.5789	297	3297	1.7208	2.563	2.5271	30.3061	30.8448
DP	REL_1	cluster_4	SP4	4928	10718.957	10458	10980	153.3704	0.4598	-1.1209	0.001	0.001	11260	101830	56.863	2162	19122	10.678	548	4928	2.7518	4.8668	4.8394	25.3469	25.7714
SP4	RREB1_2	cluster_3	DN	83707	182055.294	180581	183720	993.5942	0.4598	-1.1209	0.001	0.001	51020	1245776	62.597	14003	307557	15.454	3583	83707	4.2061	7.0227	6.7193	25.5874	27.2167
SP8	SOX8_9	cluster_3	DN	2938	6393.56	6137	6648	150.2163	0.4596	-1.1215	0.001	0.0010909	2766	44281	57.488	784	11527	14.965	187	2938	3.8143	6.7607	6.6349	23.852	25.488
SP4	TCF12_known1	cluster_3	DN	22882	49809.322	49362	50257	261.8585	0.4594	-1.1222	0.001	0.0010909	34730	249719	53.636	13030	93370	20.055	3187	22882	4.9147	9.1765	9.1631	24.4589	24.5068
SP4	ARID5B_1	cluster_3	DN	4704	10241.864	9967	10545	180.1626	0.4593	-1.1224	0.001	0.0010909	4639	60416	59.556	1409	17650	17.399	366	4704	4.637	7.8896	7.786	25.9759	26.6516
DN	ATF7_1	cluster_4	SP4	2583	5626.303	5475	5802	99.7456	0.4592	-1.1228	0.001	0.001	3904	50828	69.581	650	8215	11.246	199	2583	3.536	5.0973	5.0818	30.6154	31.4425
SP8	MGA_3	cluster_3	DN	6046	13167.829	12835	13502	214.1517	0.4592	-1.1228	0.001	0.0010909	4956	84386	59.559	1383	22542	15.91	358	6046	4.2672	7.2236	7.1647	25.8858	26.821
SP4	SOX1_2	cluster_3	DN	3614	7874.677	7602	8150	155.4546	0.459	-1.1234	0.001	0.0010909	3418	47871	59.739	1031	13594	16.964	260	3614	4.5099	7.6068	7.5495	25.2182	26.5853
SP8	SOX3_1	cluster_3	DN	2455	5350.824	5121	5565	137.8413	0.4589	-1.1237	0.001	0.0010909	2117	33889	61.352	615	9153	16.57	156	2455	4.4445	7.3689	7.2442	25.3659	26.8218
DN	KLF4_1	cluster_4	SP4	25337	55235.838	54782	55642	258.0577	0.4587	-1.1243	0.001	0.001	90604	870001	74.9	8010	74996	6.4566	2652	25337	2.1813	2.927	2.9123	33.1086	33.7845
SP4	SOX18_4	cluster_3	DN	2441	5323.525	5125	5502	120.8905	0.4586	-1.1246	0.001	0.0012	2335	32760	62.2	673	8902	16.902	176	2441	4.6346	7.5375	7.4512	26.1516	27.4208
DP	EGR1_known12	cluster_4	SP4	24442	53325.377	52587	53911	394.9099	0.4584	-1.1254	0.001	0.001	50191	833460	66.56	5218	82372	6.5782	1481	24442	1.9519	2.9507	2.9326	28.3825	29.6727
DN	EGR1_known12	cluster_4	SP4	28023	61151.004	60586	61784	345.4954	0.4583	-1.1257	0.001	0.001	57885	958377	76.536	5218	82372	6.5782	1707	28023	2.2379	2.949	2.924	32.7137	34.0201
SP4	LMO2_2	cluster_3	DN	2184	4773.035	4644	4922	85.2505	0.4577	-1.1276	0.001	0.0010435	3781	30272	59.431	1039	8150	16	274	2184	4.2877	7.2468	7.2146	26.3715	26.7975
SP8	BATF_known1	cluster_3	DN	3573	7813.064	7582	8034	140.6216	0.4574	-1.1285	0.001	0.0010435	3843	50078	57.839	1070	13651	15.767	276	3573	4.1268	7.1819	7.1349	25.7944	26.1739
DP	KLF7_1	cluster_4	SP4	30980	67742.71	66999	68476	450.1274	0.4573	-1.1287	0.001	0.001	72055	1166887	65.914	6936	105944	5.9845	1945	30980	1.75	2.6993	2.6549	28.0421	29.2419
DN	MYBL2_4	cluster_4	SP4	1211	2650.579	2538	2744	63.0953	0.4571	-1.1295	0.001	0.0010435	1996	27994	79.043	254	3426	9.6736	87	1211	3.4194	4.3587	4.3259	34.252	35.3473
DP	SP4_2	cluster_4	SP4	24566	53760.396	53063	54410	423.3665	0.457	-1.1298	0.001	0.001	56997	957954	67.242	5244	82431	5.7861	1501	24566	1.7244	2.6335	2.5644	28.6232	29.8019
SP8	RUNX2_4	cluster_3	DN	22674	49637.124	49002	50168	360.5442	0.4568	-1.1303	0.001	0.0010435	17076	261045	63.344	5278	79171	19.211	1480	22674	5.5019	8.6671	8.6859	28.0409	28.6393
SP8	FOXO6_3	cluster_3	DN	22107	48410.242	47876	48990	338.8922	0.4567	-1.1308	0.001	0.0010435	22108	289208	59.065	6537	83159	16.984	1701	22107	4.5149	7.694	7.644	26.0211	26.584
SP8	ELK4_1	cluster_3	DN	3005	6582.046	6438	6716	83.1617	0.4566	-1.1309	0.001	0.001	10505	84617	76.469	1097	8678	7.8423	375	3005	2.7156	3.5697	3.5513	34.1841	34.6278
SP8	SOX15_2	cluster_3	DN	3065	6713.181	6499	6956	136.5764	0.4566	-1.1309	0.001	0.0010909	3083	43248	60.363	869	11549	16.119	220	3065	4.2779	7.1359	7.087	25.3165	26.5391
SP4	SOX4_1	cluster_3	DN	13285	29097.011	28622	29565	288.6124	0.4566	-1.131	0.001	0.0010435	12657	205161	65.153	3071	46460	14.754	841	13285	4.2189	6.6445	6.4754	27.3852	28.5945
SP4	IRF4_2	cluster_3	DN	6463	14156.624	13864	14467	182.8928	0.4566	-1.1311	0.001	0.001	6862	99125	67.779	1556	21390	14.626	456	6463	4.4192	6.6453	6.5201	29.3059	30.2151
SP8	ATF7_1	cluster_3	DN	2808	6155.393	5933	6376	134.9117	0.4563	-1.132	0.001	0.001	3427	44593	61.045	801	10168	13.919	216	2808	3.844	6.3029	6.297	26.9663	27.6161
SP4	EWSR1::FLI1_1	cluster_3	DN	65737	144115.881	142897	145617	783.853	0.4561	-1.1324	0.001	0.0010435	47664	952228	58.406	13531	254490	15.61	3346	65737	4.0321	7.02	6.9035	24.7284	25.8309
SP4	SOX2_5	cluster_3	DN	4052	8889.312	8562	9171	175.4961	0.4559	-1.1332	0.001	0.0010909	3385	54207	61.44	1000	15053	17.061	258	4052	4.5926	7.6219	7.475	25.8	26.9182
DN	XBP1_4	cluster_4	SP4	3011	6613.281	6409	6803	118.4919	0.4554	-1.1349	0.001	0.0010435	6101	79442	72.628	739	9250	8.4566	233	3011	2.7527	3.819	3.7902	31.5291	32.5514
SP8	RUNX3_1	cluster_3	DN	26193	57520.225	56869	58228	398.3793	0.4554	-1.1349	0.001	0.0010435	19753	302988	62.704	6177	92639	19.172	1704	26193	5.4207	8.6265	8.6449	27.5862	28.2743
SP4	ATF7_1	cluster_3	DN	2839	6237.107	6040	6427	119.3058	0.4553	-1.1352	0.001	0.001	3484	45358	62.093	801	10168	13.919	218	2839	3.8864	6.2572	6.2591	27.216	27.9209
SP4	NFATC1_3	cluster_3	DN	3500	7691.127	7455	7936	143.438	0.4551	-1.1356	0.001	0.0010909	3149	40963	60.391	1065	13171	19.418	273	3500	5.16	8.6694	8.5443	25.6338	26.5735
SP4	POU2F2_known1	cluster_3	DN	4328	9512.649	9204	9775	179.5819	0.455	-1.136	0.001	0.0010435	3339	60392	62.523	935	15805	16.363	244	4328	4.4807	7.3076	7.1665	26.0963	27.3837
DN	KLF16_1	cluster_4	SP4	28327	62272.875	61744	62724	301.4027	0.4549	-1.1364	0.001	0.001	87857	980098	74.479	7768	85275	6.4801	2499	28327	2.1526	2.8444	2.8902	32.1704	33.2184
SP4	REL_2	cluster_3	DN	7599	16731.21	16438	17015	168.7447	0.4542	-1.1386	0.001	0.0010435	12661	114508	63.571	3018	26629	14.783	845	7599	4.2187	6.674	6.6362	27.9987	28.5366
SP4	SOX14_2	cluster_3	DN	2708	5965.598	5776	6176	113.7922	0.454	-1.1391	0.001	0.0010909	3491	38572	60.257	952	10109	15.792	249	2708	4.2305	7.1326	7.0206	26.1555	26.788
DN	ATF2_1	cluster_4	SP4	357	787.54	739	833	28.4116	0.454	-1.1392	0.001	0.0010435	1484	10388	76.433	151	1053	7.7478	51	357	2.6267	3.4367	3.4367	33.7748	33.9031
SP4	CUX2_2	cluster_3	DN	180	397.669	358	437	25.1135	0.454	-1.1392	0.001	0.0013333	387	3483	71.8	69	591	12.183	20	180	3.7106	5.168	5.168	28.9855	30.4569
SP8	CUX1_9	cluster_3	DN	675	1488.496	1370	1593	68.9935	0.4538	-1.1397	0.001	0.0012632	577	9969	67.331	151	2358	15.926	40	675	4.559	6.9324	6.771	26.4901	28.626
SP4	REL_1	cluster_3	DN	7533	16600.506	16352	16864	159.6534	0.4538	-1.1398	0.001	0.001	12552	113599	63.434	3006	26515	14.806	838	7533	4.2065	6.6762	6.6312	27.8776	28.4103
SP8	E2F7_1	cluster_3	DN	4073	8979.515	8710	9228	150.7324	0.4536	-1.1404	0.001	0.0010435	4510	58679	64.962	1136	14187	15.706	317	4073	4.5091	7.0288	6.9412	27.9049	28.7094
SP4	SP100_1	cluster_3	DN	706	1557.826	1468	1646	52.5134	0.4535	-1.1407	0.001	0.0013333	962	12518	74.454	181	2174	12.93	56	706	4.1991	5.8212	5.6399	30.9392	32.4747
SP8	IKZF1_2	cluster_3	DN	12282	27084.547	26684	27439	225.8354	0.4535	-1.1409	0.001	0.0010435	20377	163023	59.485	5902	46144	16.837	1544	12282	4.4815	7.5772	7.5339	26.1606	26.6167
DP	REL_2	cluster_4	SP4	4831	10658.421	10396	10888	150.2516	0.4533	-1.1414	0.001	0.0010435	11409	103099	57.237	2133	18854	10.467	538	4831	2.682	4.7156	4.6858	25.2227	25.6232
SP8	REL_1	cluster_3	DN	7427	16391.392	16106	16689	170.8006	0.4531	-1.142	0.001	0.001	12386	112079	62.586	3006	26515	14.806	823	7427	4.1473	6.6446	6.6266	27.3786	28.0106
DN	EGR4_2	cluster_4	SP4	22506	49682.312	49216	50157	289.441	0.453	-1.1424	0.001	0.001	55529	908524	79.001	4162	64752	5.6306	1385	22506	1.957	2.4942	2.4772	33.2773	34.7572
SP8	POU2F2_known1	cluster_3	DN	4096	9044.491	8695	9337	193.6411	0.4529	-1.1426	0.001	0.0010435	3160	57168	59.186	935	15805	16.363	228	4096	4.2406	7.2152	7.1648	24.385	25.9158
DP	NFKB_known11	cluster_4	SP4	5867	12960.654	12673	13235	174.398	0.4527	-1.1433	0.001	0.001	11257	136572	59.553	1852	22046	9.6133	485	5867	2.5584	4.3084	4.2959	26.1879	26.6125
DN	CUX1_8	cluster_4	SP4	685	1515.455	1427	1607	56.2853	0.4524	-1.1444	0.001	0.0011429	619	9964	75.019	140	2170	16.338	43	685	5.1574	6.9467	6.8747	30.7143	31.5668
SP4	SOX3_1	cluster_3	DN	2530	5594.755	5373	5819	133.2782	0.4523	-1.1446	0.001	0.0010909	2214	35441	64.162	615	9153	16.57	162	2530	4.5803	7.3171	7.1386	26.3415	27.6412
SP8	FOXP1_1	cluster_3	DN	14312	31641.83	31111	32260	378.7264	0.4523	-1.1446	0.001	0.0010435	10498	244896	63.1	3115	57864	14.909	695	14312	3.6876	6.6203	5.8441	22.3114	24.7339
SP4	FOXO3_1	cluster_3	DN	4304	9517.124	9291	9774	146.97	0.4523	-1.1447	0.001	0.0010435	5633	74003	67.715	1195	14663	13.417	336	4304	3.9383	5.9648	5.816	28.1172	29.3528
SP4	IKZF2_2	cluster_3	DN	2636	5831.913	5663	5996	101.8178	0.4521	-1.1453	0.001	0.0010435	3698	36980	61.725	1013	9683	16.162	268	2636	4.3999	7.2472	7.1282	26.4561	27.223
SP8	FOXP3_2	cluster_3	DN	1703	3769.061	3658	3876	66.4863	0.452	-1.1457	0.001	0.0010909	4518	27108	59.623	1087	6401	14.079	285	1703	3.7457	6.3081	6.2823	26.219	26.6052
DN	EGR1_known9	cluster_4	SP4	9997	22120.89	21838	22408	165.2229	0.452	-1.1458	0.001	0.001	32718	444077	82.227	2102	27438	5.0805	747	9997	1.8511	2.2831	2.2512	35.5376	36.4349
SP4	E2F7_1	cluster_3	DN	4101	9089.328	8853	9322	148.0696	0.4512	-1.148	0.001	0.0010435	4567	59426	65.789	1136	14187	15.706	317	4101	4.5401	6.9411	6.901	27.9049	28.9067
SP4	SOX21_2	cluster_3	DN	3847	8538.052	8324	8773	136.5362	0.4506	-1.15	0.001	0.0010909	3895	54547	61.419	1089	14396	16.21	282	3847	4.3316	7.2401	7.0526	25.8953	26.7227
SP4	ETV2_1	cluster_3	DN	4167	9251.674	8920	9563	199.2437	0.4505	-1.1505	0.001	0.0010435	7748	77780	26.389	3573	35299	11.976	416	4167	1.4138	5.3691	5.3574	11.6429	11.8049
DN	HIF1A_1	cluster_4	SP4	3792	8421.071	8268	8581	99.7241	0.4504	-1.1508	0.001	0.0010435	13599	153110	79.913	980	10689	5.5789	338	3792	1.9792	2.4855	2.4767	34.4898	35.4757

SP4	POU2F2_known15	cluster_3	DN	2666	5921.221	5720	6100	109.1418	0.4503	-1.1509	0.001	0.0010435	3260	36005	62.683	905	9687	16.865	243	2666	4.6414	7.454	7.4045	26.8508	27.5214
SP4	POU2F3_2	cluster_3	DN	2125	4723.203	4580	4856	84.21	0.45	-1.1519	0.001	0.0010909	3575	28645	62.59	988	7694	16.812	265	2125	4.6432	7.4126	7.4184	26.8219	27.6189
SP4	ERG_4	cluster_3	DN	5082	11306.572	11025	11550	163.852	0.4495	-1.1535	0.001	0.001	7152	93169	64.953	1392	17593	12.265	392	5082	3.5429	5.481	5.4546	28.1609	28.8865
SP8	RBPJ_2	cluster_3	DN	8385	18658.167	18332	18989	193.1907	0.4494	-1.1538	0.001	0.0010909	10072	100834	56.034	3446	33705	18.73	844	8385	4.6596	8.3797	8.3156	24.4922	24.8776
SP8	LMO2_2	cluster_3	DN	2062	4592.453	4446	4736	83.9651	0.4491	-1.1548	0.001	0.0010435	3634	29090	57.111	1039	8150	16	258	2062	4.0482	7.0996	7.0883	24.8316	25.3006
SP8	REL_2	cluster_3	DN	7379	16454.185	16175	16709	162.7777	0.4485	-1.1568	0.001	0.0010435	12473	112795	62.62	3018	26629	14.783	818	7379	4.0966	6.5582	6.542	27.104	27.7104
SP8	FOXM1_1	cluster_3	DN	2891	6447.462	6281	6620	105.2523	0.4485	-1.1569	0.001	0.0010909	4986	39915	56.233	1490	11672	16.444	363	2891	4.0729	7.2804	7.2429	24.3624	24.7687
DP	EGR4_2	cluster_4	SP4	19761	44069.319	43505	44633	351.5005	0.4484	-1.1571	0.001	0.001	49002	804691	69.973	4162	64752	5.6306	1216	19761	1.7183	2.4815	2.4557	29.2167	30.518
SP4	NFKB_known11	cluster_3	DN	8078	18019.57	17684	18384	202.6527	0.4483	-1.1574	0.001	0.001	11939	145036	63.244	2446	28875	12.591	670	8078	3.5225	5.6119	5.5697	27.3917	27.9758
SP8	ELK3_2	cluster_3	DN	2204	4917.935	4688	5158	142.279	0.4483	-1.1576	0.001	0.0011429	5710	51570	24.526	2266	20219	9.6161	245	2204	1.0482	4.2907	4.2738	10.812	10.9006
SP8	ETV6_2	cluster_3	DN	4759	10618.753	10303	10922	192.4212	0.4482	-1.1577	0.001	0.0011429	9151	82658	22.329	5363	47824	12.919	528	4759	1.2856	5.7699	5.7575	9.8452	9.9511
DN	HIF1A_2	cluster_4	SP4	4839	10797.676	10588	10997	127.1301	0.4482	-1.1578	0.001	0.001	15992	214291	80.94	1053	13560	5.1218	364	4839	1.8277	2.2761	2.2581	34.5679	35.6858
SP8	SOX21_2	cluster_3	DN	3642	8128.108	7873	8359	151.2001	0.4481	-1.158	0.001	0.0010909	3688	51655	58.162	1089	14396	16.21	264	3642	4.1008	7.1584	7.0506	24.2424	25.2987
SP8	SOX18_4	cluster_3	DN	2210	4935.499	4715	5154	130.0348	0.4479	-1.1588	0.001	0.0012	2159	30301	57.531	673	8902	16.902	162	2210	4.196	7.5035	7.2935	24.0713	24.8259
DP	GMEB2_2	cluster_1	SP8	1032	2305.538	2166	2443	84.9328	0.4479	-1.1589	0.001	0.001	2591	34443	62.139	297	3755	6.7744	80	1032	1.8618	3.0876	2.9963	26.936	27.4834
SP4	ETV6_2	cluster_3	DN	5223	11664.546	11311	12047	216.2954	0.4478	-1.159	0.001	0.0011429	10041	90698	24.501	5363	47824	12.919	579	5223	1.4109	5.7664	5.7587	10.7962	10.9213
SP4	ATF2_1	cluster_3	DN	497	1111.393	1060	1165	33.5126	0.4477	-1.1594	0.001	0.0010435	1377	9639	70.922	228	1585	11.662	71	497	3.6568	5.1561	5.1561	31.1404	31.3565
SP4	SOX5_1	cluster_3	DN	1430	3197.112	3065	3319	76.0279	0.4475	-1.1602	0.001	0.0010909	2386	21481	63.838	590	5116	15.204	162	1430	4.2498	6.7896	6.657	27.4576	27.9515
SP8	SOX14_2	cluster_3	DN	2549	5698.666	5489	5892	117.2226	0.4474	-1.1604	0.001	0.0010909	3330	36787	57.469	952	10109	15.792	236	2549	3.9821	7.0871	6.9291	24.7899	25.2152
SP4	TBX19_1	cluster_3	DN	5233	11704.719	11321	12014	200.9974	0.4471	-1.1612	0.001	0.0010435	3815	72708	56.872	1156	21010	16.434	279	5233	4.0932	7.3132	7.1973	24.1349	24.9072
SP4	ARID5A_1	cluster_3	DN	801	1793.959	1679	1903	68.6697	0.4468	-1.1623	0.001	0.0012632	810	11102	65.491	232	2962	17.473	60	801	4.7251	7.4074	7.2149	25.8621	27.0425
SP4	FOXO4_2	cluster_3	DN	5126	11479.5	11232	11738	156.8537	0.4466	-1.163	0.001	0.0010435	6478	84929	66.37	1450	17939	14.019	396	5126	4.0058	6.113	6.0356	27.3103	28.5746
DN	KLF7_1	cluster_4	SP4	33974	76098.705	75328	76784	446.6789	0.4465	-1.1634	0.001	0.001	81419	1312000	74.111	6936	105944	5.9845	2152	33974	1.9191	2.6431	2.5895	31.0265	32.0679
SP8	CUX2_1	cluster_3	DN	386	866.055	785	938	45.7444	0.4463	-1.1638	0.001	0.0012632	434	7992	75.035	86	1315	12.346	24	386	3.6241	5.53	4.8298	27.907	29.3536
SP4	BATF_known1	cluster_3	DN	3600	8067.761	7806	8326	150.296	0.4463	-1.1639	0.001	0.0010435	3974	51803	59.832	1070	13651	15.767	278	3600	4.158	6.9955	6.9494	25.9813	26.3717
SP4	POU2F2_known10	cluster_3	DN	3237	7254.471	7060	7425	118.4227	0.4463	-1.164	0.001	0.0010435	5130	46258	63.552	1333	11620	15.964	361	3237	4.4472	7.037	6.9977	27.0818	27.8571
SP4	FOXP1_1	cluster_3	DN	14763	33104.426	32437	33742	379.5294	0.446	-1.165	0.001	0.0010435	11002	256998	66.218	3115	57864	14.909	717	14763	3.8038	6.517	5.7444	23.0177	25.5133
SP4	FLI1_2	cluster_3	DN	4788	10738.197	10463	11008	169.5651	0.4459	-1.1651	0.001	0.001	7024	91445	65.047	1315	16623	11.824	369	4788	3.4058	5.2534	5.2359	28.0608	28.8035
DP	ATF2_2	cluster_4	SP4	1836	4119.002	3971	4297	99.6211	0.4459	-1.1653	0.001	0.0010435	4893	53941	62.848	616	6617	7.7096	167	1836	2.1392	3.413	3.4037	27.1104	27.7467
SP4	ATF3_known5	cluster_3	DN	10713	24029.718	23619	24520	264.1625	0.4458	-1.1654	0.001	0.001	11915	167390	58.274	3073	41829	14.562	768	10713	3.7295	6.4457	6.4	24.9919	25.6114
SP4	NFKB_known10	cluster_3	DN	7343	16482.217	16161	16824	194.8934	0.4455	-1.1664	0.001	0.001	10445	126213	62.637	2275	26672	13.237	611	7343	3.6442	5.8497	5.8179	26.8571	27.5307
SP4	HMGA1_2	cluster_3	DN	5465	12268.898	11999	12549	165.6123	0.4455	-1.1666	0.001	0.0010435	5885	82554	67.162	1457	19052	15.5	398	5465	4.4461	6.763	6.6199	27.3164	28.6847
SP4	SOX8_9	cluster_3	DN	3067	6892.023	6646	7139	149.2096	0.4451	-1.1678	0.001	0.0010909	2995	47951	62.253	784	11527	14.965	197	3067	3.9818	6.5776	6.3961	25.1276	26.6071
DN	HES5_1	cluster_4	SP4	3704	8324.884	8143	8491	112.4691	0.445	-1.1681	0.001	0.0010435	12165	134258	77.001	1020	10976	6.2951	337	3704	2.1244	2.7702	2.7589	33.0392	33.7464
SP4	SOX13_1	cluster_3	DN	2230	5012.684	4816	5212	116.6141	0.445	-1.1682	0.001	0.0010909	2170	32640	65.561	581	8084	16.237	151	2230	4.4792	6.9585	6.8321	25.9897	27.5854
SP8	SOX1_2	cluster_3	DN	3309	7441.946	7177	7687	155.6484	0.4447	-1.169	0.001	0.0010909	3221	45113	56.297	1031	13594	16.964	239	3309	4.1293	7.4201	7.3349	23.1814	24.3416
SP8	TCF12_known1	cluster_3	DN	21919	49330.746	48901	49855	286.5468	0.4443	-1.1703	0.001	0.0010909	34302	246920	53.035	13030	93370	20.055	3047	21919	4.7079	8.8829	8.877	23.3845	23.4754
SP4	CBX5_1	cluster_3	DN	3382	7616.726	7377	7843	132.0359	0.4441	-1.1711	0.001	0.0010435	4490	49467	65.073	1143	12008	15.796	310	3382	4.4489	6.9042	6.8369	27.1216	28.1646
SP8	ELK3_1	cluster_3	DN	2258	5087.073	4837	5333	148.7506	0.444	-1.1714	0.001	0.0011429	5938	53644	24.833	2319	20674	9.5703	251	2258	1.0453	4.227	4.2092	10.8236	10.9219
SP4	ETV5_1	cluster_3	DN	3709	8369.035	8207	8534	103.2617	0.4432	-1.1738	0.001	0.0010435	11580	105410	73.515	1288	11464	7.9952	412	3709	2.5867	3.5579	3.5186	31.9876	32.3535
SP4	MYBL1_3	cluster_3	DN	1159	2617.539	2503	2746	75.052	0.443	-1.1746	0.001	0.0010909	1511	16654	62.822	400	4270	16.107	105	1159	4.3719	6.949	6.9593	26.25	27.1429
SP8	NFATC1_1	cluster_3	DN	7084	15993.508	15579	16363	231.9192	0.443	-1.1747	0.001	0.0010909	5066	97783	60.876	1524	27428	17.076	372	7084	4.4102	7.3431	7.2446	24.4094	25.8276
SP8	SOX1_3	cluster_3	DN	3099	6998.144	6764	7220	140.07	0.4429	-1.1749	0.001	0.0010909	2972	41649	55.348	979	13064	17.361	224	3099	4.1183	7.537	7.4408	22.8805	23.7217
DP	KLF14_1	cluster_4	SP4	16110	36449.433	35919	36954	312.014	0.442	-1.1779	0.001	0.001	49087	664659	67.227	4312	55543	5.6179	1209	16110	1.6295	2.463	2.4238	28.038	29.0046
SP4	RUNX1_2	cluster_3	DN	5018	11355.842	11069	11679	182.3129	0.4419	-1.1781	0.001	0.0010435	4574	71425	63.314	1268	18623	16.508	325	5018	4.4481	7.1054	7.0256	25.6309	26.9452
SP4	TCF3_1	cluster_3	DN	58751	132982.619	132015	134225	670.8421	0.4418	-1.1785	0.001	0.0011429	45052	639694	51.735	18450	258597	20.914	4138	58751	4.7515	9.1849	9.1842	22.4282	22.7191
SP4	IKZF1_1	cluster_3	DN	8097	18328.931	17944	18681	213.1601	0.4418	-1.1786	0.001	0.0010435	8843	106411	60.935	2665	30767	17.618	685	8097	4.6367	7.7462	7.6092	25.7036	26.3172
SP8	IRF_known14	cluster_3	DN	17703	40075.493	39306	40778	437.0624	0.4418	-1.1787	0.001	0.001	13279	278624	63.239	3403	65717	14.916	873	17703	4.018	6.5743	6.3537	25.6538	26.9382
SP8	NFKB_known11	cluster_3	DN	8210	18586.588	18254	18907	198.8269	0.4417	-1.1787	0.001	0.001	12335	149870	65.352	2446	28875	12.591	678	8210	3.58	5.4966	5.4781	27.7187	28.4329
SP4	FOXM1_1	cluster_3	DN	2967	6721.805	6557	6893	105.0177	0.4415	-1.1796	0.001	0.0010909	5181	41487	58.448	1490	11672	16.444	373	2967	4.18	7.1994	7.1516	25.0336	25.4198
SP8	ETV4_2	cluster_3	DN	1646	3729.894	3522	3937	127.3811	0.4414	-1.1797	0.001	0.0011429	4775	43196	25.078	1685	15032	8.7271	183	1646	0.95561	3.8325	3.8105	10.8605	10.95
SP4	ETV4_2	cluster_3	DN	1692	3834.42	3630	4017	120.962	0.4414	-1.1798	0.001	0.0011429	4906	44382	25.767	1685	15032	8.7271	188	1692	0.98232	3.832	3.8124	11.1573	11.256
SP4	SOX1_3	cluster_3	DN	3270	7413.115	7171	7664	154.4248	0.4412	-1.1805	0.001	0.0010909	3151	44155	58.678	979	13064	17.361	238	3270	4.3455	7.5532	7.4057	24.3105	25.0306
SP4	RUNX2_4	cluster_3	DN	21044	47714.851	47111	48341	337.7008	0.441	-1.181	0.001	0.0010435	16422	250904	60.883	5278	79171	19.211	1381	21044	5.1064	8.4095	8.3873	26.1652	26.5804
SP8	ELK4_2	cluster_3	DN	2946	6680.859	6382	6909	160.0424	0.441	-1.181	0.001	0.0011429	7080	64048	23.72	3169	28339	10.495	326	2946	1.0911	4.6045	4.5997	10.2872	10.3956
SP4	NFATC2_1	cluster_3	DN	2679	6084.651	5949	6230	83.7622	0.4404	-1.1832	0.001	0.0010909	5908	35448	63.511	1665	9740	17.451	449	2679	4.7999	7.5999	7.5575	26.967	27.5051
DP	KLF13_1	cluster_4	SP4	9071	20625.609	20152	21095	284.7884	0.4398	-1.185	0.001	0.001	19895	342302	61.998	2092	34137	6.1829	538	9071	1.6429	2.7042	2.65	25.717	26.5723
SP4	ETV6_1	cluster_3	DN	22474	51101.137	50523	51667	345.595	0.4398	-1.1851	0.001	0.001	27340	393690	65.257	5707	79282	13.142	1581	22474	3.7252	5.7827	5.7086	27.7028	28.3469
SP8	CTCF_known2	cluster_2	DP	8850	20127.136	19500	20734	372.6668	0.4397	-1.1853	0.001	0.0010435	15803	254255	30.429	4231	66340	7.9395	552	8850	1.0592	3.493	3.4808	13.0466	13.3404
DN	EGR4_1	cluster_4	SP4	11894	27064.662	26772	27394	193.6966	0.4395	-1.1861	0.001	0.001	34373	389734	77.181	3257	35643	7.0586	1065	11894	2.3554	3.0984	3.0518	32.6988	33.3698
DP	EGR3_2	cluster_4	SP4	16154	36758.453	36255	37242	305.2668	0.4395	-1.1861	0.001	0.001	45781	699461	71.099	3669	53073	5.3948	1069	16154	1.642	2.335	2.3095	29.136	30.4373
SP4	FOXO6_1	cluster_3	DN	2351	5353.625	5144	5535	124.5475	0.4392	-1.1869	0.001	0.0010909	2688	35069	61.166	738	9094	15.861	185	2351	4.1005	6.8824	6.7039	25.0678	25.8522
SP4	IRF4_1	cluster_3	DN	2884	6568.81	6379	6763	120.5293	0.4391	-1.1873	0.001	0.0010435	3307	46509	68.576	712	9749	14.375	206	2884	4.2524	6.2292	6.201	28.9326	29.5825
DN	HES7_1	cluster_4	SP4	3651	8317.143	8145	8475	100.3156	0.439	-1.1876	0.001	0.0010435	12542	138583	78.225	1003	10767	6.0776	331	3651	2.0608	2.6391	2.6345	33.001	33.9092
SP8	RUNX1_2	cluster_3	DN	4492	10238.51	9878	10629	202.5239	0.4388	-1.1884	0.001	0.0010435	4117	64095	56.816	1268	18623	16.508	295	4492	3.9819	7.1654	7.0083	23.265	24.1207
SP8	POU2F2_known13	cluster_3	DN	3556	8108.9	7842	8401	174.5844	0.4386	-1.189	0.001	0.0010909	2653	55941	63.012	702	13503	15.21	170	3556	4.0055	6.4078	6.3567	24.2165	26.3349
SP4	SOX14_3	cluster_3	DN	3554	8105.654	7821	8349	148.0487	0.4385	-1.1893	0.001	0.0010909	3418	47906	58.588	1064	14226	17.398	257	3554	4.3464	7.519	7.4187	24.1541	24.9824
SP8	EWSR1::FLI1_1	cluster_3	DN	59926	136865.758	135672	138185	791.7043	0.4378	-1.1915	0.001	0.0010435	45443	904295	55.466	13531	254490	15.61	3077	59926	3.6757	6.7711	6.6268	22.7404	23.5475
SP4	HESX1_1	cluster_3	DN	1004	2295.749	2200	2401	62.6457	0.4376	-1.1924	0.001	0.0012632	1328	11959	60.289	454	3905	19.686	114	1004	5.0615	8.5843	8.3954	25.1101	25.7106
SP4	ELK3_1	cluster_3	DN	2349	5373.663	5138	5614	146.635	0.4372	-1.1935	0.001	0.0011429	6270	56640	26.22	2319	20674	9.5703	261	2349	1.0874	4.1627	4.1472	11.2549	11.3621
SP8	IKZF3_1	cluster_3	DN	5807	13299.121	13011	13582	168.8843	0.4367	-1.1953	0.001	0.0010435	6473	77854	57.71	2031	23575	17.475	490	5807	4.3045	7.5699	7.4588	24.126	24.632
DP	HIF1A_2	cluster_4	SP4	4064	9308.211	9086	9573	145.5476	0.4367	-1.1954	0.001	0.001	13829	185302	69.991	1053	13560	5.1218	309	4064	1.535	2.2344	2.1932	29.3447	29.9705
SP8	SOX2_5	cluster_3	DN	3657	8393.101	8104	8683	178.0528	0.4358	-1.1983	0.001	0.0010909	3178	50901	57.693	1000	15053	17.061	231	3657	4.1449	7.2687	7.1845	23.1	24.2942
SP4	FLI1_4	cluster_3	DN	4933	11328.206	11076	11574	146.3707	0.4355	-1.1992	0.001	0.001	7443	97009	66.151	1366	17315	11.807	380	4933	3.3639	5.1055	5.0851	27.8184	28.4897
SP8	IRF6_1	cluster_3	DN	2712	6229.181	6018	6455	132.0005	0.4355	-1.1994	0.001	0.0010909	2749	44071	67.262	609	9420	14.377	171	2712	4.1391	6.2204	6.1537	28.0788	28.7898
SP8	PBX1_4	cluster_3	DN	1950	4480.117	4338	4648	96.8336	0.4354	-1.1996	0.001	0.0010909	2771	31106	57.753	745	7979	14.814	178	1950	3.6205	6.4237	6.2689	23.8926	24.4392
SP4	E2F8_1	cluster_3	DN	5592	12854.258	12579	13139	163.3356	0.4351	-1.2007	0.001	0.001	7471	82374	63.953	1924	20480	15.9	510	5592	4.3415	6.8264	6.7885	26.5073	27.3047
SP4	ELK3_2	cluster_3	DN	2286	5264.482	5005	5513	149.5247	0.4343	-1.2031	0.001	0.0011429	6107	55145	26.227	2266	20219	9.6161	254	2286	1.0872	4.1592	4.1454	11.2092	11.3062
SP4	RUNX3_1	cluster_3	DN	23877	54979.248	54325	55670	402.8492	0.4343	-1.2032	0.001	0.0010435	18902	289786	59.972	6177	92639	19.172	1561	23877	4.9414	8.2584	8.2395	25.2712	25.7742
SP4	XBP1_2	cluster_3	DN	3593	8288.157	7997	8598	185.4105	0.4336	-1.2056	0.001	0.0010435	2736	63041	65.364	600	12989	13.468	157	3593	3.7254	5.7383	5.6995	26.1667	27.6619
SP8	SOX14_3	cluster_3	DN	3296	7604.571	7354	7862	156.6275	0.4335	-1.2059	0.001	0.0010909	3228	45250	55.339	1064	14226	17.398	238	3296	4.0309	7.373	7.284	22.3684	23.1688
SP4	MYBL2_4	cluster_3	DN	1484	3432.806	3292	3554	80.8357	0.4325	-1.2093	0.001	0.0010435	1678	23553	66.504	389	5253	14.832	106	1484	4.1902	6.317	6.3007	27.2494	28.2505
SP8	TCF3_1	cluster_3	DN	57949	134090.225	132932	135208	687.5881	0.4322	-1.2103	0.001	0.0011429	45409	645019	52.166	18450	258597	20.914	4082	57949	4.6866	8.9894	8.9841	22.1247	22.409
SP8	MYBL2_4	cluster_3	DN	1428	3308.931	3167	3455	88.7642	0.4317	-1.2118	0.001	0.0010435	1616	22677	64.03	389	5253	14.832	102	1428	4.0321	6.3119	6.2971	26.2211	27.1845
SP4	CUX1_9	cluster_3	DN	674	1564.484	1466	1691	68.0564	0.4312	-1.2136	0.001	0.0012632	597	10309	69.627	151	2358	15.926	41	674	4.5522	6.8677	6.538	27.1523	28.5835
SP4	ATF2_2	cluster_3	DN	3149	7311.876	7121	7523	125.0525	0.4307	-1.2151	0.001	0.0010435	5130	56548	65.885	1035	11230	13.084	287	3149	3.669	5.5945	5.5687	27.7295	28.041
SP4	FOXO1_4	cluster_3	DN	2032	4720.053	4551	4889	110.4384	0.4306	-1.2155	0.001	0.0010909	2354	30679	61.609	647	7957	15.979	159	2032	4.0806	6.7545	6.6234	24.575	25.5373
SP4	HES5_1	cluster_3	DN	7035	16347.36	16075	16647	171.7779	0.4304	-1.2163	0.001	0.0010435	9376	103478	59.348	2570	27859	15.978	638	7035	4.0348	6.8046	6.7985	24.8249	25.2522
SP4	SMAD4_1	cluster_3	DN	23393	54376.09	53676	55109	433.2049	0.4302	-1.2169	0.001	0.001	25162	357474	58.021	6894	94795	15.386	1655	23393	3.7969	6.5774	6.544	24.0064	24.6775
SP4	HES7_1	cluster_3	DN	7093	16516.331	16219	16839	189.1175	0.4295	-1.2193	0.001	0.0010435	9737	107540	60.702	2533	27443	15.491	644	7093	4.0037	6.6139	6.5957	25.4244	25.8463
SP8	NFATC2_1	cluster_3	DN	2549	5937.708	5810	6069	77.016	0.4294	-1.2197	0.001	0.0010909	5758	34548	61.898	1665	9740	17.451	430	2549	4.567	7.4679	7.3781	25.8258	26.1704
DN	KLF13_1	cluster_4	SP4	10766	25080.354	24665	25458	240.5267	0.4293	-1.22	0.001	0.001	24149	415074	75.178	2092	34137	6.1829	639	10766	1.9499	2.6461	2.5938	30.5449	31.5376
SP4	FOXO3_4	cluster_3	DN	1650	3855.205	3698	4010	96.6783	0.4281	-1.2238	0.001	0.0010909	2012	26245	62.811	521	6382	15.274	130	1650	3.9489	6.4612	6.2869	24.952	25.854
SP8	IKZF1_1	cluster_3	DN	7742	18095.766	17729	18393	205.2858	0.4279	-1.2248	0.001	0.0010435	8739	105142	60.209	2665	30767	17.618	657	7742	4.4334	7.518	7.3634	24.6529	25.1633
SP4	ETV1_1	cluster_3	DN	1665	3902.761	3703	4086	115.4083	0.4268	-1.2285	0.001	0.0010909	5001	45274	25.882	1706	15225	8.7037	185	1665	0.95184	3.6993	3.6776	10.8441	10.936
SP4	ARID3A_2	cluster_3	DN	1413	3314.146	3154	3482	96.76	0.4265	-1.2293	0.001	0.0013333	1263	20439	64.999	379	5462	17.37	90	1413	4.4936	7.1259	6.9133	23.7467	25.8696
SP4	IRF6_1	cluster_3	DN	2672	6268.828	6043	6479	131.1112	0.4263	-1.23	0.001	0.0010909	2762	44279	67.58	609	9420	14.377	167	2672	4.0781	6.0463	6.0345	27.422	28.3652
SP4	STAT_known3	cluster_3	DN	8345	19577.599	19179	19942	251.2251	0.4263	-1.2301	0.001	0.0010435	7269	145730	67.556	1560	29619	13.731	420	8345	3.8685	5.778	5.7263	26.9231	28.1745
SP8	SMAD4_1	cluster_3	DN	23384	54856.989	54282	55504	386.6149	0.4263	-1.2301	0.001	0.001	25393	360702	58.545	6894	94795	15.386	1656	23384	3.7954	6.5215	6.4829	24.0209	24.668
DP	EGR1_known9	cluster_4	SP4	8388	19691.186	19390	19984	182.9722	0.426	-1.2311	0.001	0.001	29172	396004	73.325	2102	27438	5.0805	626	8388	1.5531	2.1459	2.1182	29.7812	30.5707
DP	EGR4_1	cluster_4	SP4	9876	23225.098	22886	23585	215.216	0.4253	-1.2336	0.001	0.001	29511	334771	66.296	3257	35643	7.0586	884	9876	1.9558	2.9955	2.9501	27.1415	27.7081
DN	EGR3_3	cluster_4	SP4	11481	27028.276	26683	27354	208.801	0.4248	-1.2351	0.001	0.001	35070	516864	79.095	2490	34990	5.3545	794	11481	1.7569	2.264	2.2213	31.8876	32.8122
SP4	FOXO4_3	cluster_3	DN	1863	4392.915	4219	4564	103.144	0.4242	-1.2371	0.001	0.0010909	2238	29139	62.034	592	7294	15.528	146	1863	3.9661	6.5237	6.3935	24.6622	25.5415
SP8	RUNX2_5	cluster_3	DN	22838	53884.517	53136	54491	428.0806	0.4238	-1.2384	0.001	0.001	18306	320965	63.474	5062	86023	17.012	1313	22838	4.5164	7.1725	7.1154	25.9384	26.5487
DN	ATF2_2	cluster_4	SP4	2067	4880.838	4739	4998	81.0016	0.4236	-1.2392	0.001	0.0010435	5805	63978	74.542	616	6617	7.7096	188	2067	2.4083	3.2386	3.2308	30.5195	31.2377
SP8	IKZF2_2	cluster_3	DN	2315	5470.721	5308	5648	108.369	0.4233	-1.2404	0.001	0.0010435	3469	34690	57.903	1013	9683	16.162	237	2315	3.8641	6.8319	6.6734	23.3959	23.9079
SP8	ERG_2	cluster_3	DN	4634	10953.511	10709	11236	163.2673	0.4231	-1.2409	0.001	0.001	6800	88560	61.21	1430	18064	12.485	357	4634	3.2029	5.25	5.2326	24.965	25.6532
SP8	NFKB_known10	cluster_3	DN	7139	16875.828	16565	17213	204.0105	0.4231	-1.241	0.001	0.001	10708	129395	64.216	2275	26672	13.237	593	7139	3.5429	5.5379	5.5172	26.0659	26.7659
SP4	CTCF_known2	cluster_2	DP	8059	19122.799	18554	19738	362.3349	0.4215	-1.2465	0.001	0.0010435	15039	241923	28.953	4231	66340	7.9395	502	8059	0.9645	3.338	3.3312	11.8648	12.148
SP8	ETV1_1	cluster_3	DN	1589	3774.246	3589	3976	120.1507	0.4212	-1.2475	0.001	0.0010909	4842	43840	25.062	1706	15225	8.7037	176	1589	0.90839	3.6349	3.6245	10.3165	10.4368
SP4	MTF1_1	cluster_3	DN	29119	69141.629	68464	69792	406.0102	0.4212	-1.2476	0.001	0.001	38636	515202	65.297	8195	106903	13.549	2194	29119	3.6906	5.6786	5.652	26.7724	27.2387
SP4	ETS_known13	cluster_3	DN	8223	19533.106	19166	19895	229.0248	0.421	-1.2481	0.001	0.001	10234	164235	67.178	1895	29430	12.038	516	8223	3.3635	5.042	5.0068	27.2296	27.9409
DP	CTCF_known2	cluster_4	SP4	4153	9904.233	9430	10312	266.5161	0.4194	-1.2537	0.001	0.0010435	14645	235553	28.191	2287	35597	4.2602	260	4153	0.49703	1.7753	1.7631	11.3686	11.6667
SP8	HES5_1	cluster_3	DN	6837	16338.224	16003	16666	194.6419	0.4185	-1.2567	0.001	0.0010435	9348	103156	59.163	2570	27859	15.978	620	6837	3.9212	6.6324	6.6278	24.1245	24.5414
SP4	GMEB2_2	cluster_3	DN	2024	4840.466	4685	4998	97.3421	0.4183	-1.2575	0.001	0.001	2773	36777	66.35	576	7389	13.331	154	2024	3.6515	5.5536	5.5034	26.7361	27.3921
SP8	ERG_4	cluster_3	DN	4485	10748.826	10508	10997	151.8344	0.4173	-1.2608	0.001	0.001	6801	88600	61.768	1392	17593	12.265	346	4485	3.1267	5.0875	5.0621	24.8563	25.4931
SP8	HES7_1	cluster_3	DN	6841	16440.43	16110	16763	203.8518	0.4161	-1.2648	0.001	0.0010435	9675	106849	60.312	2533	27443	15.491	621	6841	3.8615	6.4186	6.4025	24.5164	24.928
SP8	ETV6_1	cluster_3	DN	20338	48932.233	48283	49498	374.7706	0.4156	-1.2666	0.001	0.001	26153	376727	62.445	5707	79282	13.142	1434	20338	3.3712	5.4831	5.3986	25.127	25.6527
SP4	MYC_known20	cluster_3	DN	9214	22174.9	21754	22602	283.3275	0.4155	-1.2669	0.001	0.001	8841	144867	57.464	2458	39039	15.485	568	9214	3.6549	6.4246	6.3603	23.1082	23.602
SP8	E2F8_1	cluster_3	DN	5394	12982.795	12723	13220	145.0837	0.4155	-1.267	0.001	0.001	7551	83269	64.648	1924	20480	15.9	493	5394	4.1878	6.5289	6.4778	25.6237	26.3379
SP4	ATF6_1	cluster_3	DN	1470	3553.635	3433	3658	69.5132	0.4138	-1.2729	0.001	0.0010435	3915	27415	63.572	810	5616	13.023	210	1470	3.4088	5.364	5.362	25.9259	26.1752
SP8	MTF1_1	cluster_3	DN	28816	69681.139	69028	70359	413.5935	0.4135	-1.2739	0.001	0.001	38926	519131	65.795	8195	106903	13.549	2182	28816	3.6522	5.6055	5.5508	26.626	26.9553
SP4	FOXP3_2	cluster_3	DN	1633	3950.611	3838	4070	68.7444	0.4135	-1.274	0.001	0.0010909	4729	28374	62.407	1087	6401	14.079	273	1633	3.5917	5.7729	5.7553	25.115	25.5116
SP4	CUX2_1	cluster_3	DN	355	863.758	786	940	48.01	0.4117	-1.2804	0.001	0.0012632	439	7949	74.631	86	1315	12.346	23	355	3.333	5.2392	4.466	26.7442	26.9962
SP4	SP1_known3	cluster_3	DN	26428	64206.364	63626	64868	381.2848	0.4116	-1.2806	0.001	0.001	39133	513297	64.396	8009	101310	12.71	2037	26428	3.3155	5.2053	5.1487	25.4339	26.0863

SP8	MYC_known20	cluster_3	DN	9060	22021.804	21617	22390	237.0254	0.4114	-1.2813	0.001	0.001	8781	143953	57.101	2458	39039	15.485	558	9060	3.5938	6.3546	6.2937	22.7014	23.2076
SP8	ATF2_1	cluster_3	DN	455	1107.908	1054	1165	36.4256	0.4112	-1.282	0.001	0.0010435	1370	9590	70.561	228	1585	11.662	65	455	3.3478	4.7445	4.7445	28.5088	28.7066
SP4	POU2F2_known14	cluster_3	DN	1015	2471.711	2347	2601	79.1223	0.4109	-1.2832	0.001	0.0012632	922	14165	62.953	326	4330	19.244	71	1015	4.5109	7.7007	7.1655	21.7791	23.4411
SP8	E2F2_2	cluster_3	DN	2702	6602.701	6353	6858	149.345	0.4093	-1.2887	0.001	0.0010909	3179	54200	66.243	657	10343	12.641	163	2702	3.3024	5.1274	4.9852	24.8097	26.1239
DP	GMEB2_3	cluster_4	SP4	679	1661.98	1558	1764	62.7418	0.4089	-1.2902	0.001	0.0010435	2081	29462	70.749	180	2424	5.8209	50	679	1.6305	2.4027	2.3047	27.7778	28.0116
SP4	XBP1_4	cluster_3	DN	3688	9026.468	8765	9297	161.426	0.4086	-1.2911	0.001	0.0010435	5207	67800	61.985	1167	14760	13.494	283	3688	3.3717	5.435	5.4395	24.2502	24.9864
DP	EGR3_3	cluster_4	SP4	9911	24259.636	23847	24639	241.702	0.4086	-1.2914	0.001	0.001	31417	463984	71.003	2490	34990	5.3545	684	9911	1.5167	2.1772	2.1361	27.4699	28.3252
SP4	E2F2_2	cluster_3	DN	2753	6757.673	6545	6999	128.6017	0.4075	-1.2952	0.001	0.0010909	3236	55175	67.435	657	10343	12.641	163	2753	3.3647	5.0371	4.9896	24.8097	26.617
SP8	KLF12_2	cluster_3	DN	27229	66829.574	66102	67534	403.6046	0.4074	-1.2953	0.001	0.001	40686	583267	67.525	7345	100882	11.679	1936	27229	3.1523	4.7584	4.6684	26.3581	26.9909
SP8	ATF6_1	cluster_3	DN	1447	3558.871	3444	3675	70.6032	0.4068	-1.2978	0.001	0.0010435	3930	27520	63.816	810	5616	13.023	207	1447	3.3554	5.2672	5.258	25.5556	25.7657
SP4	MYCN_1	cluster_3	DN	6051	14887.945	14627	15178	165.7901	0.4065	-1.2988	0.001	0.001	10339	114073	63.425	2200	23706	13.181	551	6051	3.3644	5.3293	5.3045	25.0455	25.5252
SP4	HES1_1	cluster_3	DN	19196	47356.648	46833	47884	320.8445	0.4054	-1.3027	0.001	0.001	24677	349612	62.645	5551	76425	13.694	1363	19196	3.4396	5.5234	5.4907	24.5541	25.1174
SP8	RUNX3_3	cluster_3	DN	26258	64791.92	64088	65555	442.3858	0.4053	-1.303	0.001	0.001	23891	424439	65.225	5889	100815	15.493	1501	26258	4.0352	6.2827	6.1865	25.4882	26.0457
SP4	RUNX2_5	cluster_3	DN	20809	51380.031	50653	52098	434.4399	0.405	-1.304	0.001	0.001	17450	305925	60.5	5062	86023	17.012	1202	20809	4.1152	6.8883	6.802	23.7456	24.19
SP4	KLF12_1	cluster_3	DN	6736	16639.717	16404	16867	145.4007	0.4049	-1.3045	0.001	0.001	19302	115812	58.735	4821	28504	14.456	1125	6736	3.4162	5.8284	5.8163	23.3354	23.6318
SP8	GMEB2_2	cluster_3	DN	1911	4727.331	4543	4897	107.1061	0.4044	-1.3062	0.001	0.001	2724	36104	65.136	576	7389	13.331	148	1911	3.4477	5.4332	5.293	25.6944	25.8628
SP4	HESX1_2	cluster_3	DN	878	2173.45	2058	2306	75.4603	0.4042	-1.3067	0.001	0.0014118	906	12691	63.767	281	3567	17.923	66	878	4.4116	7.2848	6.9183	23.4875	24.6145
SP8	SP100_1	cluster_3	DN	626	1552.974	1461	1637	52.7165	0.4035	-1.3094	0.001	0.0013333	956	12440	73.99	181	2174	12.93	50	626	3.7233	5.2301	5.0322	27.6243	28.7948
SP8	CUX2_2	cluster_3	DN	157	390.814	356	427	23.0693	0.4033	-1.3102	0.001	0.0013333	381	3429	70.686	69	591	12.183	18	157	3.2364	4.7244	4.5786	26.087	26.5651
SP4	HIF1A::ARNT_1	cluster_3	DN	3766	9346.261	9183	9534	103.9317	0.403	-1.3111	0.001	0.001	10457	75241	66.952	1969	14045	12.498	525	3766	3.3511	5.0206	5.0052	26.6633	26.8138
SP4	SPDEF_6	cluster_3	DN	6008	14909.138	14612	15209	175.8669	0.403	-1.3111	0.001	0.001	8360	117397	67.827	1652	22357	12.917	432	6008	3.4712	5.1675	5.1177	26.1501	26.873
SP8	XBP1_4	cluster_3	DN	3629	9010.531	8782	9265	147.2965	0.4028	-1.3118	0.001	0.0010435	5176	67397	61.616	1167	14760	13.494	279	3629	3.3177	5.3903	5.3845	23.9075	24.5867
SP8	ARID3A_2	cluster_3	DN	1317	3273.258	3095	3444	104.124	0.4025	-1.3128	0.001	0.0013333	1242	20101	63.924	379	5462	17.37	85	1317	4.1883	6.8438	6.5519	22.4274	24.112
SP8	FLI1_2	cluster_3	DN	4101	10205.501	9916	10459	170.6479	0.4019	-1.3151	0.001	0.001	6676	86936	61.84	1315	16623	11.824	316	4101	2.9171	4.7334	4.7173	24.0304	24.6706
SP8	KLF12_1	cluster_3	DN	6866	17094.587	16850	17362	149.5867	0.4017	-1.3159	0.001	0.001	19874	119244	60.476	4821	28504	14.456	1148	6866	3.4822	5.7764	5.7579	23.8125	24.0878
SP4	KLF12_2	cluster_3	DN	25734	64470.596	63707	65184	425.8246	0.3992	-1.3249	0.001	0.001	39236	562601	65.133	7345	100882	11.679	1827	25734	2.9792	4.6564	4.5741	24.8741	25.509
SP8	E2F_known24	cluster_3	DN	2282	5723.799	5507	5910	124.4982	0.3988	-1.3263	0.001	0.001	2992	51042	66.197	582	9023	11.702	138	2282	2.9596	4.6123	4.4708	23.7113	25.2909
SP4	ETS_known17	cluster_3	DN	11838	29698.393	29200	30261	314.9071	0.3986	-1.3269	0.001	0.001	12925	220877	62.412	2924	48134	13.601	696	11838	3.345	5.3849	5.3595	23.803	24.5938
SP8	MYCN_1	cluster_3	DN	5960	14992.487	14745	15258	159.9334	0.3976	-1.3307	0.001	0.001	10432	115067	63.978	2200	23706	13.181	546	5960	3.3138	5.2339	5.1796	24.8182	25.1413
SP8	E2F2_3	cluster_3	DN	1752	4413.095	4214	4611	117.8465	0.3971	-1.3323	0.001	0.001	2069	35210	66.193	432	6875	12.925	107	1752	3.2937	5.1716	4.9759	24.7685	25.4836
SP4	POU2F2_known13	cluster_3	DN	3284	8297.876	7968	8591	187.2956	0.3958	-1.337	0.001	0.0010909	2728	57442	64.703	702	13503	15.21	159	3284	3.6991	5.8284	5.7171	22.6496	24.3205
SP8	FLI1_4	cluster_3	DN	4284	10830.23	10573	11104	160.9319	0.3956	-1.3378	0.001	0.001	7101	92567	63.122	1366	17315	11.807	330	4284	2.9213	4.6472	4.628	24.1581	24.7416
SP4	SPZ1_1	cluster_3	DN	33752	85411.832	84589	86340	527.9806	0.3952	-1.3394	0.001	0.001	48085	704127	63.844	9814	136683	12.393	2354	33752	3.0603	4.8955	4.7935	23.9861	24.6936
SP4	CTCF_known1	cluster_3	DN	48942	123906.78	122787	125215	719.5018	0.395	-1.3401	0.001	0.0010435	55221	1020372	58.86	11978	213108	12.293	2678	48942	2.8232	4.8496	4.7965	22.3577	22.9658
SP8	E2F_known26	cluster_3	DN	1468	3723.69	3556	3885	106.0076	0.3944	-1.3423	0.001	0.0010435	1668	28373	65.605	379	5910	13.665	89	1468	3.3944	5.3357	5.1739	23.4828	24.8393
SP8	SPDEF_6	cluster_3	DN	5929	15064.562	14731	15365	192.4424	0.3936	-1.3452	0.001	0.001	8460	118816	68.647	1652	22357	12.917	427	5929	3.4255	5.0473	4.9901	25.8475	26.5197
SP8	ATF2_2	cluster_3	DN	2867	7303.812	7108	7490	112.4168	0.3926	-1.3488	0.001	0.0010435	5120	56438	65.757	1035	11230	13.084	262	2867	3.3404	5.1172	5.0799	25.314	25.5298
SP8	XBP1_2	cluster_3	DN	3139	8009.545	7666	8329	192.6835	0.392	-1.3511	0.001	0.0010435	2655	61164	63.418	600	12989	13.468	138	3139	3.2547	5.1977	5.1321	23	24.1666
SP8	ETV5_1	cluster_3	DN	1170	2987.582	2812	3152	103.4153	0.3918	-1.3517	0.001	0.0010435	4199	38058	26.542	1288	11464	7.9952	130	1170	0.81598	3.096	3.0743	10.0932	10.2059
SP8	HESX1_1	cluster_3	DN	876	2245.1	2134	2355	63.1276	0.3905	-1.3568	0.001	0.0012632	1301	11720	59.084	454	3905	19.686	99	876	4.4162	7.6095	7.4744	21.8062	22.4328
SP4	PATZ1_1	cluster_3	DN	42881	109993.628	109128	110802	499.2828	0.3899	-1.359	0.001	0.001	71621	944599	64.129	14139	176126	11.957	3326	42881	2.9112	4.6439	4.5396	23.5236	24.3468
SP8	SP1_known3	cluster_3	DN	25334	65039.737	64387	65685	407.0306	0.3895	-1.3602	0.001	0.001	39576	519194	65.135	8009	101310	12.71	1953	25334	3.1783	4.9348	4.8795	24.3851	25.0064
SP8	ETS_known13	cluster_3	DN	7504	19274.819	18915	19638	229.1111	0.3893	-1.3609	0.001	0.001	10079	161754	66.163	1895	29430	12.038	469	7504	3.0694	4.6532	4.6391	24.7493	25.4978
SP8	HES1_1	cluster_3	DN	18795	48275.076	47712	48861	342.6498	0.3893	-1.3609	0.001	0.001	25160	356406	63.862	5551	76425	13.694	1334	18795	3.3678	5.3021	5.2735	24.0317	24.5927
DP	GMEB1_1	cluster_4	SP4	1195	3079.597	2912	3233	93.3323	0.3882	-1.365	0.001	0.0010435	5680	92646	67.496	294	4624	3.3688	74	1195	0.8706	1.3028	1.2899	25.1701	25.8434
SP8	PATZ1_1	cluster_3	DN	44136	113689.822	112850	114469	500.6972	0.3882	-1.3651	0.001	0.001	74077	975342	66.217	14139	176126	11.957	3421	44136	2.9964	4.6182	4.5252	24.1955	25.0593
SP8	SP8_1	cluster_3	DN	36125	93081.39	92342	93733	427.7876	0.3881	-1.3655	0.001	0.001	67362	823083	65.87	12378	144666	11.577	3027	36125	2.891	4.4936	4.389	24.4547	24.9713
SP4	RUNX3_3	cluster_3	DN	23954	61871.67	61096	62689	467.7041	0.3872	-1.369	0.001	0.001	22811	405311	62.285	5889	100815	15.493	1373	23954	3.6811	6.019	5.91	23.3147	23.7604
SP8	KLF4_1	cluster_3	DN	33129	85981.636	85388	86533	341.8966	0.3853	-1.3759	0.001	0.001	80637	776764	66.873	14112	130789	11.26	3520	33129	2.8522	4.3652	4.265	24.9433	25.3301
SP4	E2F_known24	cluster_3	DN	2242	5830.537	5610	6050	134.1428	0.3846	-1.3784	0.001	0.001	3037	51804	67.185	582	9023	11.702	133	2242	2.9077	4.3793	4.3279	22.8522	24.8476
SP8	KLF16_1	cluster_3	DN	37028	96390.873	95744	97148	424.3236	0.3842	-1.3803	0.001	0.001	79277	887559	67.446	13662	146104	11.103	3378	37028	2.8138	4.261	4.1719	24.7255	25.3436
SP4	STAT_known2	cluster_3	DN	8664	22576.887	22025	23026	288.5929	0.3838	-1.3816	0.001	0.001	9013	180903	68.592	1770	33621	12.748	436	8664	3.2851	4.8375	4.7893	24.6328	25.7696
SP4	SP4_2	cluster_3	DN	38701	100882.66	100100	101686	488.148	0.3836	-1.3822	0.001	0.001	57022	957821	67.232	9762	154277	10.829	2382	38701	2.7165	4.1773	4.0405	24.4007	25.0854
SP4	SP8_1	cluster_3	DN	34260	89583.198	88878	90310	449.6017	0.3824	-1.3867	0.001	0.001	64724	792047	63.386	12378	144666	11.577	2859	34260	2.7418	4.4172	4.3255	23.0974	23.6821
SP8	SP4_2	cluster_3	DN	39910	104421.011	103578	105312	528.0711	0.3822	-1.3876	0.001	0.001	59003	991063	69.566	9762	154277	10.829	2456	39910	2.8014	4.1625	4.027	25.1588	25.8691
SP4	E2F_known26	cluster_3	DN	1430	3752.689	3601	3926	99.6923	0.3812	-1.3913	0.001	0.0010435	1689	28742	66.459	379	5910	13.665	85	1430	3.3065	5.0326	4.9753	22.4274	24.1963
SP4	HIF1A_1	cluster_3	DN	5681	14908.772	14621	15188	172.5937	0.3811	-1.3918	0.001	0.0010435	11555	130065	67.885	2009	22190	11.582	509	5681	2.9651	4.405	4.3678	25.336	25.6016
SP8	STAT_known3	cluster_3	DN	7257	19056.108	18608	19481	257.7753	0.3809	-1.3927	0.001	0.0010435	7066	141634	65.658	1560	29619	13.731	366	7257	3.3641	5.1797	5.1238	23.4615	24.5012
SP4	KLF16_1	cluster_3	DN	35522	93286.026	92513	94147	481.3225	0.3808	-1.3929	0.001	0.001	76586	858580	65.244	13662	146104	11.103	3235	35522	2.6993	4.224	4.1373	23.6788	24.3128
SP8	CTCF_known1	cluster_3	DN	49043	128813.8	127606	130036	708.8126	0.3807	-1.3931	0.001	0.0010435	57414	1061210	61.215	11978	213108	12.293	2688	49043	2.829	4.6818	4.6214	22.4411	23.0132
SP4	HIF1A_2	cluster_3	DN	7766	20404.38	20106	20704	181.7508	0.3806	-1.3935	0.001	0.001	13692	183403	69.274	2276	29792	11.253	586	7766	2.9333	4.2799	4.2344	25.7469	26.0674
SP8	HIF1A::ARNT_1	cluster_3	DN	3519	9247.159	9086	9413	103.1656	0.3806	-1.3936	0.001	0.001	10355	74439	66.239	1969	14045	12.498	489	3519	3.1313	4.7224	4.7274	24.8349	25.0552
SP4	KLF4_1	cluster_3	DN	31710	83320.488	82677	83983	401.5004	0.3806	-1.3937	0.001	0.001	78036	752571	64.791	14112	130789	11.26	3363	31710	2.73	4.3095	4.2136	23.8308	24.2452
SP8	SPZ1_1	cluster_3	DN	33454	87958.849	87152	88643	473.2735	0.3803	-1.3946	0.001	0.001	49464	724618	65.702	9814	136683	12.393	2342	33454	3.0333	4.7348	4.6168	23.8639	24.4756
SP8	KLF13_1	cluster_3	DN	16083	42383.335	41770	43069	392.221	0.3795	-1.3979	0.001	0.001	20972	360883	65.363	4015	66134	11.978	946	16083	2.9129	4.5108	4.4566	23.5616	24.3188
SP8	ETS_known17	cluster_3	DN	10801	28488.217	27983	29069	341.9037	0.3792	-1.3991	0.001	0.001	12384	211645	59.804	2924	48134	13.601	635	10801	3.052	5.1276	5.1034	21.7168	22.4394
SP4	MYCN_2	cluster_3	DN	7784	20564.871	20267	20886	185.4984	0.3785	-1.4015	0.001	0.001	17249	155885	64.493	3625	32109	13.284	867	7784	3.2204	5.0264	4.9934	23.9172	24.2424
SP8	EGR1_known12	cluster_3	DN	33436	88447.182	87679	89211	476.8849	0.378	-1.4034	0.001	0.001	52057	864606	69.047	8501	131962	10.538	2066	33436	2.6702	3.9687	3.8672	24.303	25.3376
SP4	E2F2_3	cluster_3	DN	1656	4418.552	4216	4596	119.9301	0.3749	-1.4153	0.001	0.001	2073	35280	66.325	432	6875	12.925	98	1656	3.1132	4.7274	4.6939	22.6852	24.0873
SP8	ATF3_known4	cluster_3	DN	5497	14669.332	14374	14966	175.2708	0.3748	-1.4159	0.001	0.001	8603	121435	69.067	1578	21515	12.237	393	5497	3.1265	4.5682	4.5267	24.9049	25.5496
SP4	EGR1_known12	cluster_3	DN	31896	85318.333	84468	86160	486.5932	0.3739	-1.4195	0.001	0.001	50263	834726	66.661	8501	131962	10.538	1958	31896	2.5472	3.8955	3.8211	23.0326	24.1706
SP8	KLF7_1	cluster_3	DN	46647	125223.362	124229	126222	580.4051	0.3725	-1.4246	0.001	0.001	75221	1217400	68.768	12356	187429	10.587	2978	46647	2.635	3.959	3.8317	24.1017	24.8878
SP8	MYCN_2	cluster_3	DN	7710	20765.111	20469	21059	190.5397	0.3713	-1.4292	0.001	0.001	17431	157501	65.162	3625	32109	13.284	860	7710	3.1898	4.9337	4.8952	23.7241	24.012
SP4	KLF13_1	cluster_3	DN	15166	40898.3	40215	41519	384.2747	0.3708	-1.4311	0.001	0.001	20223	347988	63.027	4015	66134	11.978	894	15166	2.7469	4.4207	4.3582	22.2665	22.9322
SP4	EGR4_1	cluster_3	DN	12646	34446.671	33990	34890	264.7325	0.3671	-1.4456	0.001	0.001	29753	337110	66.76	4776	52354	10.368	1121	12646	2.5044	3.7677	3.7513	23.4715	24.1548
SP4	SP4_1	cluster_3	DN	50264	136904.72	135899	138010	631.5575	0.3672	-1.4456	0.001	0.001	80215	1441564	69.533	12286	203749	9.8278	2931	50264	2.4245	3.6539	3.4868	23.8564	24.6696
SP4	SP1_known2	cluster_3	DN	55181	150989.406	150138	151906	550.2317	0.3655	-1.4522	0.001	0.001	112736	1565680	69.085	17436	225537	9.9517	4196	55181	2.4348	3.722	3.5244	24.0652	24.4665
SP4	KLF7_1	cluster_3	DN	43744	120483.949	119419	121481	637.9763	0.3631	-1.4617	0.001	0.001	72331	1170811	66.136	12356	187429	10.587	2785	43744	2.471	3.8504	3.7362	22.5397	23.339
SP8	SP1_known2	cluster_3	DN	56326	155959.707	155088	156910	548.7044	0.3612	-1.4693	0.001	0.001	116483	1617881	71.388	17436	225537	9.9517	4295	56326	2.4854	3.6872	3.4815	24.6329	24.9742
SP8	HESX1_2	cluster_3	DN	775	2149.069	2025	2275	76.1107	0.3609	-1.4703	0.001	0.0014118	899	12600	63.31	281	3567	17.923	58	775	3.8941	6.4516	6.1508	20.6406	21.7269
SP8	EGR4_1	cluster_3	DN	12737	35355.722	34972	35801	244.3443	0.3603	-1.4728	0.001	0.001	30552	346098	68.54	4776	52354	10.368	1138	12737	2.5224	3.7248	3.6802	23.8275	24.3286
SP8	POU2F2_known14	cluster_3	DN	904	2512.029	2362	2642	86.2382	0.3601	-1.4734	0.001	0.0012632	928	14244	63.304	326	4330	19.244	61	904	4.0176	6.5733	6.3465	18.7117	20.8776
SP4	ATF3_known4	cluster_3	DN	5094	14161.878	13831	14460	189.6725	0.3597	-1.475	0.001	0.001	8312	117305	66.718	1578	21515	12.237	365	5094	2.8972	4.3912	4.3425	23.1305	23.6765
SP4	EGR4_2	cluster_3	DN	27451	76351.401	75574	77107	441.6258	0.3595	-1.4758	0.001	0.001	48413	794683	69.102	7390	113656	9.883	1717	27451	2.387	3.5466	3.4543	23.2341	24.1527
SP8	STAT_known2	cluster_3	DN	7940	22291.114	21840	22800	293.6912	0.3562	-1.4891	0.001	0.001	8911	178852	67.814	1770	33621	12.748	401	7940	3.0106	4.5001	4.4394	22.6554	23.6162
DP	CTCF_known2	cluster_3	DN	8037	22605.843	21896	23296	423.8342	0.3556	-1.4919	0.001	0.0010435	14645	235553	28.191	5148	80650	9.6522	501	8037	0.96187	3.421	3.412	9.7319	9.9653
SP8	SP4_1	cluster_3	DN	50308	141652.47	140576	142634	613.7312	0.3552	-1.4935	0.001	0.001	82983	1490454	71.892	12286	203749	9.8278	2943	50308	2.4266	3.5465	3.3753	23.9541	24.6912
SP8	EGR4_2	cluster_3	DN	28039	79081.748	78468	79735	423.2975	0.3546	-1.4959	0.001	0.001	50126	822981	71.563	7390	113656	9.883	1766	28039	2.4382	3.5231	3.407	23.8972	24.6701
SP8	KLF14_1	cluster_3	DN	24094	68153.886	67407	68872	400.0977	0.3535	-1.5001	0.001	0.001	51058	690823	69.873	7718	99743	10.089	1822	24094	2.437	3.5685	3.4877	23.6072	24.1561
DP	GMEB1_1	cluster_3	DN	3150	8937.872	8701	9224	157.5727	0.3525	-1.5043	0.001	0.0010435	5680	92646	67.496	858	13450	9.7989	194	3150	2.2949	3.4155	3.4	22.6107	23.4201
SP4	KLF14_1	cluster_3	DN	23079	65549.189	64892	66220	409.6886	0.3521	-1.506	0.001	0.001	49055	664031	67.164	7718	99743	10.089	1745	23079	2.3343	3.5572	3.4756	22.6095	23.1385
SP8	HIF1A_1	cluster_3	DN	5201	14918.127	14609	15198	175.408	0.3487	-1.52	0.001	0.0010435	11568	130194	67.952	2009	22190	11.582	463	5201	2.7146	4.0024	3.9948	23.0463	23.4385
SP4	EGR1_known9	cluster_3	DN	11875	34121.937	33726	34567	256.7316	0.348	-1.5227	0.001	0.001	28464	386336	71.535	3721	48541	8.988	886	11875	2.1988	3.1127	3.0737	23.8108	24.4639
SP8	HIF1A_2	cluster_3	DN	7011	20410.573	20100	20710	199.4922	0.3435	-1.5415	0.001	0.001	13715	183722	69.394	2276	29792	11.253	526	7011	2.6481	3.8352	3.8161	23.1107	23.5332
DP	CUX2_2	cluster_1	SP8	113	331.566	289	373	25.9296	0.3428	-1.5446	0.001	0.0013333	279	2511	51.763	79	675	13.915	14	113	2.3294	5.0179	4.5002	17.7215	16.7407
SP4	AHR::ARNT::HIF1A_1	cluster_3	DN	3541	10357.441	10174	10530	110.9289	0.3419	-1.5482	0.001	0.0010435	12561	103445	70.93	1807	14714	10.089	427	3541	2.428	3.3994	3.4231	23.6303	24.0655
SP4	EGR3_2	cluster_3	DN	21043	62672.514	62006	63397	435.0604	0.3358	-1.5744	0.001	0.001	45033	687127	69.845	6402	91970	9.3486	1418	21043	2.139	3.1488	3.0625	22.1493	22.8803
SP8	EGR1_known9	cluster_3	DN	11833	35321.883	34936	35714	246.2497	0.335	-1.5777	0.001	0.001	29509	400130	74.089	3721	48541	8.988	894	11833	2.191	3.0296	2.9573	24.0258	24.3773
SP4	EGR3_3	cluster_3	DN	13879	41586.029	41092	42110	297.0255	0.3338	-1.5831	0.001	0.001	31018	457668	70.037	4306	60531	9.263	954	13879	2.1239	3.0756	3.0325	22.1551	22.9287
SP4	GMEB2_3	cluster_3	DN	1056	3183.038	3060	3297	75.51	0.332	-1.5909	0.001	0.0010435	2133	30178	72.468	322	4464	10.72	76	1056	2.5358	3.5631	3.4992	23.6025	23.6559
SP8	EGR3_2	cluster_3	DN	21057	64784.631	64178	65406	378.3498	0.325	-1.6213	0.001	0.001	46634	711154	72.287	6402	91970	9.3486	1426	21057	2.1404	3.0579	2.961	22.2743	22.8955
SP8	EGR3_3	cluster_3	DN	13838	42918.486	42407	43437	315.8707	0.3224	-1.6329	0.001	0.001	32104	473266	72.424	4306	60531	9.263	963	13838	2.1176	2.9996	2.9239	22.3641	22.861
SP8	GMEB2_3	cluster_3	DN	974	3111.088	2971	3236	83.0127	0.3133	-1.6744	0.001	0.0010435	2094	29627	71.145	322	4464	10.72	70	974	2.3389	3.3429	3.2875	21.7391	21.819
SP8	AHR::ARNT::HIF1A_1	cluster_3	DN	3195	10368.546	10174	10539	111.4642	0.3082	-1.698	0.001	0.0010435	12611	103685	71.095	1807	14714	10.089	390	3195	2.1908	3.0925	3.0814	21.5827	21.714
SP4	GMEB1_1	cluster_3	DN	2867	9368.995	9121	9621	151.6603	0.3061	-1.708	0.001	0.0010435	5959	97141	70.771	858	13450	9.7989	178	2867	2.0887	2.9871	2.9514	20.7459	21.316
SP4	CTCF_known2	cluster_3	DN	6770	23179.86	22602	23868	376.7453	0.2921	-1.7755	0.001	0.0010435	15039	241923	28.953	5148	80650	9.6522	423	6770	0.81023	2.8127	2.7984	8.2168	8.3943
SP8	GMEB1_1	cluster_3	DN	2737	9596.987	9333	9824	151.2022	0.2853	-1.8096	0.001	0.0010435	6106	99511	72.498	858	13450	9.7989	169	2737	1.994	2.7678	2.7504	19.697	20.3494
SP8	CTCF_known2	cluster_3	DN	6643	24338.298	23763	24891	358.4212	0.273	-1.8732	0.001	0.0010435	15803	254255	30.429	5148	80650	9.6522	415	6643	0.79503	2.6261	2.6127	8.0614	8.2368
